# Prevalence and patterns of higher-order drug interactions in *Escherichia coli*

**DOI:** 10.1038/s41540-018-0069-9

**Published:** 2018-09-03

**Authors:** Elif Tekin, Cynthia White, Tina Manzhu Kang, Nina Singh, Mauricio Cruz-Loya, Robert Damoiseaux, Van M. Savage, Pamela J. Yeh

**Affiliations:** 1 Department of Ecology and Evolutionary Biology, University of California, Los Angeles, CA 90095 USA; 2 Department of Biomathematics, University of California, David Geffen School of Medicine, Los Angeles, CA 90095 USA; 3 California NanoSystems Institute, University of California, Los Angeles, 570 Westwood Plaza, Los Angeles, CA 90095 USA; 4 Santa Fe Institute, Santa Fe, NM 87501 USA

## Abstract

Interactions and emergent processes are essential for research on complex systems involving many components. Most studies focus solely on pairwise interactions and ignore higher-order interactions among three or more components. To gain deeper insights into higher-order interactions and complex environments, we study antibiotic combinations applied to pathogenic *Escherichia coli* and obtain unprecedented amounts of detailed data (251 two-drug combinations, 1512 three-drug combinations, 5670 four-drug combinations, and 13608 five-drug combinations). Directly opposite to previous assumptions and reports, we find higher-order interactions increase in frequency with the number of drugs in the bacteria’s environment. Specifically, as more drugs are added, we observe an elevated frequency of net synergy (effect greater than expected based on independent individual effects) and also increased instances of emergent antagonism (effect less than expected based on lower-order interaction effects). These findings have implications for the potential efficacy of drug combinations and are crucial for better navigating problems associated with the combinatorial complexity of multi-component systems.

## Complex systems: emergent interactions are prevalent in drug combinations

Interactions play an important role in determining the dynamics of complex systems yet higher-order interactions that involve more than two components are poorly understood. A research team from University of California, Los Angeles led by Pamela Yeh, use a bacteria system to show that higher-order interactions among antibiotics are prevalent and also that there are systematic patterns in how they occur: the frequency of higher-order interactions increases with the number of components, net interactions tend to be more synergistic, and emergent interactions—arising at specific higher-order levels—tend toward antagonism. By detecting patterns in interactions as the number of drugs increases, they provide a method to handle the combinatorial complexity that results from higher-order interactions, yielding a solid foundation for exploring the patterns and consequences of emergent phenomena in other research areas.

## Introduction

Interactions are the key to unlocking emergent and unintuitive properties across many fields: reactions in biochemistry, food webs and flocking among birds in ecology, environmental stressors and effects on species diversity in conservation biology, genetic interactions in evolution and bioinformatics, bound states and many-body interactions in physics, social interactions in economics and political science, and drug interactions in pharmacology.^1–6^ Understanding whether components interact in a manner that enhances (synergy) or weakens (antagonism) the individual effects of the mixed components is important because the type of interaction governs the dynamics of complex systems. For example, in conservation biology, understanding multiple-stressor effects informs the development of strategies to prevent loss of biodiversity.^6^ In pharmacology, understanding drug interactions enables the effective design of treatment strategies to combat complex diseases such as cancer^7^ and HIV,^8^ which increasingly rely on multidrug treatments. Despite the structural and terminological differences between natural and social systems, multi-component interactions constitute a unifying theme in studying and predicting patterns in large complex systems.

The formation of complex structures and dynamics often results from emergent properties that cannot be explained based on the effects of individual components or even interactions between pairwise parts or other lower-order interactions—fewer numbers of components than the whole combination. It is crucial to distinguish between two types of higher-order interactions, namely net and emergent interactions. Net higher-order interactions are most commonly measured and defined relative to the null expectation that would arise from a non-interacting combination of independent single components and their effects.^9^ Thus, for a four-drug combination, a net higher-order effect could arise from pairwise interactions, three-way interactions, and/or four-way interactions. For this reason, we also define and measure emergent higher-order effects that require all components to be present for an interaction to exist. For instance, when the addition of a small amount of a third drug alters the interaction between two drugs—as opposed to the third drug interacting with either of the individual drugs already present—this is an emergent interaction. Moreover, some interactions only exist when many components are present, even though there is no interaction between any of the isolated pairs or triples,^9^ such as a protein or molecule that requires all parts to be joined before it can properly function and any activity or response can be measured. Therefore, an approach that explicitly distinguishes emergent interactions—interactions that are not due to the presence of lower-order interactions—from the existence of any (net) interaction is indispensable to fully represent complex system dynamics.^10,11^

Despite the possibility of higher-order interactions, studies have often focused on pairwise interactions,^12–15^ whereas presuming or concluding that higher-order effects—owing to three or more components—are either extremely rare or negligible.^16–18^ There are both conceptual and practical reasons that have led to this view. On the conceptual side, it has been commonly argued that lower-order effects are likely to counteract each other in higher-order combinations such that they essentially cancel out and result in zero or negligible net effect.^19,20^ Alternatively, it has been argued that higher-order interactions prevent large systems from exhibiting stability, and thus concluded that these effects either do not exist or are insignificant.^21^ For these reasons, many studies either implicitly assume higher-order interactions do not exist or argue against the importance of measuring and considering higher-order interactions.^13,16,22,23^

A practical reason why higher-order interactions have received much less attention relative to two-way (pairwise) interactions is owing to combinatorial complexity, i.e., difficulty in collecting data for all subsets of combinations of components.^15,16^ Another practical challenge to examining and classifying higher-order interactions is that it entails calculating the contribution from all lower levels—subsets of fewer components—to the emergent behavior of the whole combination. These calculations are surprisingly subtle and correspond to having well defined and understood emergent interaction metrics with straightforward generalization to higher-orders and quantification of uncertainties. The lack of such methods in many fields has stood as a theoretical limitation for studying interacting systems.^10,14^

Recent progress has made it possible to overcome these latter two practical limitations and thus enables us to directly test the above conceptual presumption by measuring how frequent higher-order interactions are and what types of interactions are present. In this regard, our recent studies of combinations of three stressors^9,24^ showed that many more interactions arise with three-way interactions compared with two-way interactions. Moreover, these three-way interactions exhibit an important feature: higher degrees of emergent antagonism, compared with pairwise interactions. This greater amount of emergent antagonism—reduced effect relative to the expected effect based on no-interaction—suggests a need to further explore interactions among three, four, and five drugs. Correspondingly, an insightful study by Mayfield et al.^13^ found that incorporating higher-order interactions as opposed to a constrained approach of two-way interactions leads to better expectations of variations in natural plant communities, and a very recent and intriguing review by Levine et al.^14^ has provided a theoretical approach that shows how higher-order interactions can promote species richness. In addition, another compelling study demonstrated the existence of higher-order gene interactions and revealed how they can explain complex alterations in gene expression.^25^

These studies have shown the presence of higher-order effects and the importance of exploring them. However, what is lacking is a comprehensive framework and data set that can be used to reveal the patterns of higher-order interactions. In particular, a feasible empirical study design (which provides easy manipulation of the environmental disturbances and the number of components in the system) and a comprehensive theoretical approach are necessary for gaining crucial insights into biological systems (or more generally, complex systems) comprised of many components. Indeed, the theoretical framework in this setting should be conveniently generalizable to distinguish different types of higher-order effects (i.e., net versus emergent) in the presence of any number of components. Here, net interactions refer to an overall interaction compared relative to non-interacting single component effects, whereas emergent interactions correspond to higher-order effects that only exist when all the components are present.

Using large numbers of drug combinations, we ask: (1) Are there more (or fewer) interactions when more stressors are added? (2) Are there any trends in the frequency of interaction types that amplify or attenuate the complexity of a system when the number of components is increased? (3) Do these trends in interaction types differ between net versus emergent interactions?

In contrast to previous work, we show that emergent higher-order interactions are much more pervasive than commonly assumed and that there are characteristic patterns of net versus emergent higher-order interactions as the number of drugs increases. Addressing these issues will be extremely useful if we are to find a perspicuous path forward in searching for higher-order interactions and dealing with combinatorial complexities.

**Fig. 1:**
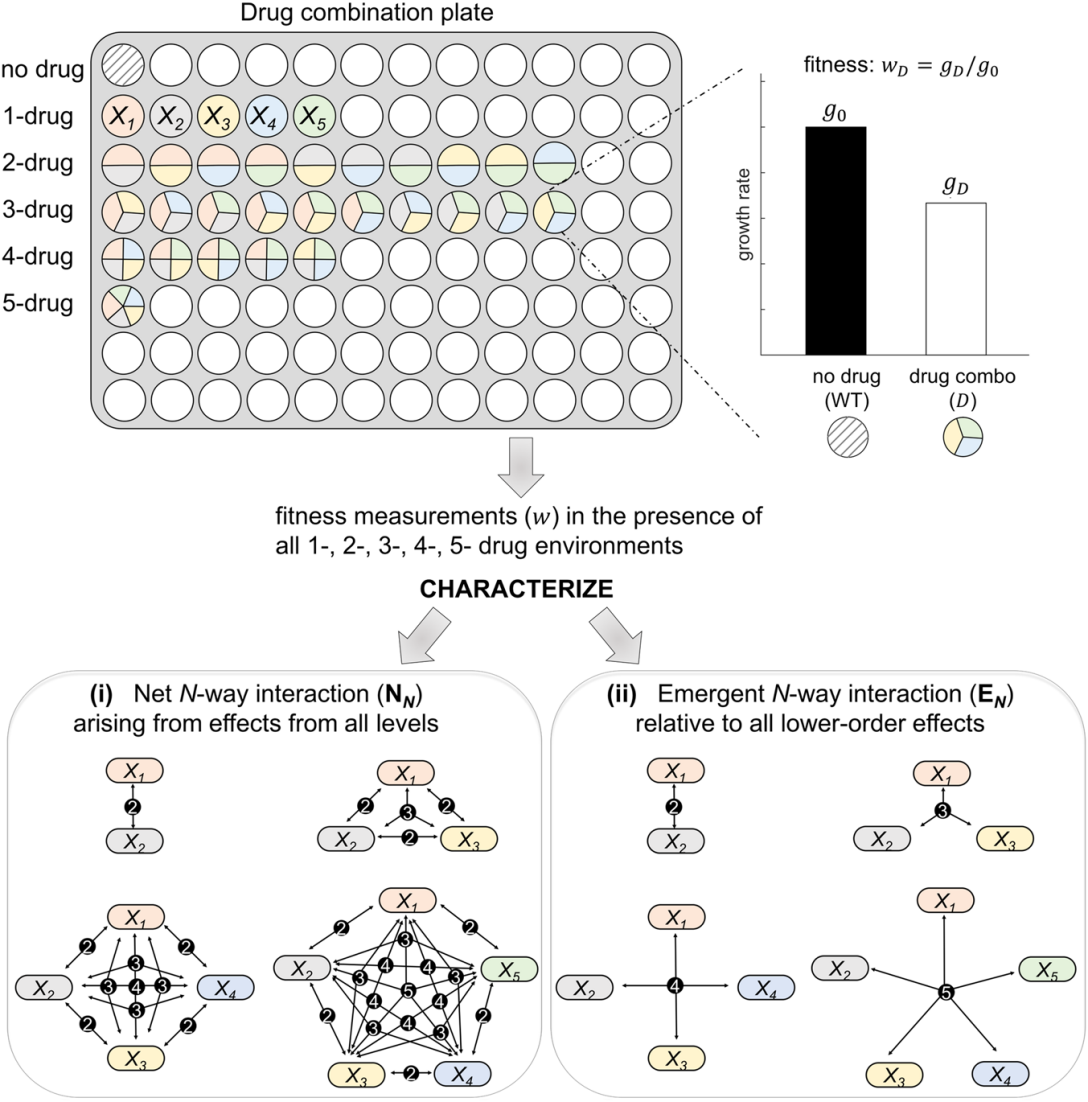
Experimental and theoretical setup for the characterization of higher-order interactions. Schematic representation of a drug combination plate, where the shaded well in the first row represents the control strain with no drug added and colored wells correspond to single or *N*-way (up to five-way) combinations from a set of drugs denoted by *X*_*i*_. The *N*-way combinations of drugs are represented by wells divided into *N* identical slices with the colors signifying the drugs in the combination (see 1-drug row for each color). The concentration of each drug is kept the same across single, two-, three-, four-, and five-drug combination experiments. Here, the experimental setup is simplified for illustration, but in actuality, (1) we filled all wells with bacteria and drug combinations to obtain replicate measurements (see “Experimental details”), and (2) we used multiple 96-well plates for each five-drug combination. From these experiments, fitness of bacteria (*w*) in the presence of drug combination (*D*) is assessed by the relative growth rate with respect to the no-drug control (WT). In the figure, schematics for the net *N*-way interaction include all possible lower-order connections, whereas an emergent interaction schematic connects all *N* drugs (such as dyad and triad for two- and three-drug combinations, respectively)

In this paper, we ground these questions in a highly controlled antibiotic study system that examines bacterial growth responses to an environment that consists of different drug combinations. We obtain data for how eight single drugs with three concentrations each, 251 two-drug combinations, 1,512 three-drug combinations, 5,670 four-drug combinations, and 13,608 five-drug combinations affect pathogenic *E. coli* growth rates (Fig. 1). This full-factorial design of drug combinations allows characterization of net and emergent interactions for all five-way and lower-order interactions (two-, three-, four-way), and thus represents a staggering amount of data compared with previous studies, allowing us to shed new light on how interactions change as more and more drugs (components) are added.

### Theoretical framework for the characterization of higher-order interactions

To measure interactions, we must first carefully define what an interaction is and what response measurements we use to assess the interactions. Based on standard definitions within the field, we can then extend and generalize this interactions framework to higher orders for both net and emergent interactions.

For drug studies the key response measurement is growth rate of a bacteria population in the presence of a drug relative to growth of bacteria in a no-drug environment. This growth rate is interpreted as the relative fitness in the presence of a drug treatment *X* and is typically denoted by *w*_*X*_ (Fig. 1), where no-growth (*w*_*X*_ = 0) represents complete lethality and maximum-growth (*w*_*X*_ = 1) represents the case that the drug treatment is not effective at all. Because we are using relative fitness, the effect of each individual drug can be interpreted as a percent reduction in growth rate, so the null expectation for the combined effects of two non-interacting drugs would be the product of two percentages, corresponding to a multiplicative definition of no interaction. Furthermore, the effect of drug treatment *X* on the fitness can also be represented in terms of the selection coefficient $s_X:w_X = e^{ - s_X}$. The case when no drug is added would generate no effect on the bacterial fitness, i.e., *s*_*X*_ = 0 and the product of the effect of two single drugs, *X*_*1*_ and *X*_*2*_, would be $w_{X_1}w_{X_2} = e^{ - (s_{X1} + s_{X2})}$, which means the null expectation of no interaction is additive in terms of the selection coefficients. Consistent with the drug literature, we will use the term additivity to refer to no interaction throughout the rest of this paper. Interaction classifications are then defined based on whether a drug combination yields more than (antagonistic) or less than (synergistic) the expected fitness measures when there are no interactions.^10,26^

Here, we review net and emergent measures for the quantification of *N*-way interaction effects (see Fig. 1 and S1 Fig). Throughout the paper, we denote single drugs by *X*_*i*_, where *i* > *0* and use a list of *X*_*i*_ to represent drug combinations (such as *X*_1_*X*_2_ for combining two drugs *X*_1_ and *X*_2_). Moreover, for notational tractability, *N*-way interaction measures are defined for *X*_1_*X*_2_… *X*_*N*_, which stands for any *N*-drug combination.

### Net *N*-way (N_*N*_) interactions

We follow the Bliss Independence model^27^ to characterize a net *N*-way interaction (N_*N*_) for the total interaction in comparison with that expected from all the independent and individual effects of each drug. Based on Bliss Independence, drugs *X*_1_ and *X*_2_ are not interacting when the addition of a second drug (*X*_2_) does not influence the percent decrease in the pathogen fitness due to another single drug (*X*_1_), i.e., $w_{X_1X_2} = w_{X_1}w_{X_2}$. Accordingly, the interaction between *X*_1_ and *X*_2_ is measured by the deviation from the non-interacting (termed additive) case as

1 $${\mathrm{N}}_2 = w_{X_1X_2} - w_{X_1}w_{X_2}$$

A correct rescaling method^28^ is needed to properly interpret the magnitude of these measures. After rescaling, a sufficiently large negative value of N_2_ suggests a synergistic interaction, as drugs together produce a superior inhibition effect relative to the case that drugs are not interacting, whereas a large positive value of N_2_ indicates an antagonistic interaction.

As a test of this model and baseline definition of no interaction, Beppler et al.^9^ performed experiments in which a single drug was treated as three different drugs to see if there was any interaction. Confirming the expectation of our interaction model and validating its correctness, no interaction of a drug with itself was observed for the vast majority of experiments (i.e., doubling and tripling the dosages of the same drug).

Extending Eq. (1) to systems with more than two drugs enables measurements and calculations to determine the presence of any kind of interaction relative to the single-drug effects. Thus, the generalized form of the net interaction measure for an *N*-drug combination is.^10,29,30^

2 $${\mathrm{N}}_N = w_{X_1X_2 \ldots X_N} - w_{X_1}w_{X_2} \ldots w_{X_N}$$

Observe that the subscript *X*_1_*X*_2_… *X*_*N*_ means the bacteria’s environment contains drug *X*_1_ plus drug *X*_2_ plus all drugs up to *X*_*N*_. Therefore, if only *k* of the *N* drugs remain in the environment, the subscript becomes *X*_1_*X*_2_… *X*_*k*_0…0, which is equivalent to *X*_1_*X*_2_… *X*_*k*_ because adding 0 or no drug is equivalent to just having the *k*-drug subset. Moreover, the relative fitness of the drugs at 0 concentration will be 1, so the net *N*-way interaction will reduce to just a *k*-way interaction, as expected.

### Emergent *N*-way (E_*N*_) interactions

To assess interactions that require all *N* drugs to be present, or equivalently, interactions beyond what is expected from the effects of all lower-order combinations, we use and extend our emergent interaction framework presented in Beppler et al.^9^ When two drugs are combined, the definitions of net and emergent interactions converge to become identical because single drugs constitute the one and only lower-order component of a two-drug environment, so there is nothing from which to emerge except the single-order effects. Thus, the emergent two-way (E_2_) interaction is identical to the net two-way interaction, N_2_ (Eq. (1)). However, when there are more than two drugs in the environment, interactions among different lower-order subsets (two-way versus three-way versus four-way or the combination of two-ways, etc.) of drugs can change the dynamics of *N*-way interactions, and those effects need to be accounted for characterizing the emergent interactions.^11^ Here, the contribution to the effect that comes solely from a lower-order combination corresponds to the total interaction when only that specific lower-order interaction is present. Based on this, we systematically determine all the lower-order interactions in an *N*-way combination, calculate the total sum of all of these lower-order interactions, and subtract this sum from the net interaction to capture any emergent interaction (see details in the Materials and Methods). When *N* = 3, this corresponds to the total (net) three-drug interaction effect that is not due to the contributions from all the two-drug combinations. As described in Materials and Methods, E_3_ yields an expression that includes fitness measurements in the presence of every possible drug combination in the three-drug environment.

3 $${\mathrm{E}}_3 = {\mathrm\it{w}}_{{\mathrm\it{X}}_1{\mathrm\it{X}}_2{\mathrm\it{X}}_3} - {\mathrm\it{w}}_{{\mathrm\it{X}}_1}{\mathrm\it{w}}_{{\mathrm\it{X}}_2{\mathrm\it{X}}_3} - {\mathrm\it{w}}_{{\mathrm\it{X}}_2}{\mathrm\it{w}}_{{\mathrm\it{X}}_1{\mathrm\it{X}}_3} - {\mathrm\it{w}}_{{\mathrm\it{X}}_3}{\mathrm\it{w}}_{{\mathrm\it{X}}_1{\mathrm\it{X}}_2} + 2{\mathrm\it{w}}_{{\mathrm\it{X}}_1}w_{X_2}w_{X_3}$$

The emergent four-way and five-way interaction formulas are derived in the Materials and Methods with explanation given for how to correctly count the combinatorics of all drug subsets while avoiding any issues of double- or overcounting of contributions.

Notably, recent papers^22,31^ have proposed new formulae to predict three-way interactions based on equations with weighted mixtures of pairwise interactions. This valuable work is useful for trying to mechanistically understand how three-way interactions might arise, with the recipe for the weights of the mixture perhaps corresponding to specific ways in which a third drug might affect the interaction of another pair. However, there is no sense in which this explains away the existence of emergent higher-order drug interactions. Our goal in this paper is merely to measure the existence of emergent interactions and characterize how prevalent they are and any patterns for their frequency. Therefore, the data produced and patterns identified in the present paper are complementary to these other recent papers^22,31^ in trying to identify the patterns and mechanisms that underlie emergent interactions.

## Results

In this study, we explored the consequences of increased complexity in multi-component systems by employing an experimental design of higher-order drug combinations and by using and extending our recently developed mathematical framework^9,24^ to characterize higher-order interactions. We categorized net interactions based on the deviation of the whole combination effect from the expected effect of no-interaction among individual drugs. To measure emergent interactions, we calculated the deviation of net interaction from the expected effect from all interactions that result from lower-order subsets/combinations of drugs (Fig. 1, S1 Fig, “Theoretical Framework for the Characterization of Higher-order Interactions” and “Materials and Methods”). As with pairwise studies for drugs, epistasis, and other biological systems, interactions are defined as synergistic or antagonistic when the effect of two components is sufficiently (see Materials and Methods for precise values) stronger or weaker than expected effects of no-interaction (net) or all lower-order interactions (emergent), respectively.

**Fig. 2:**
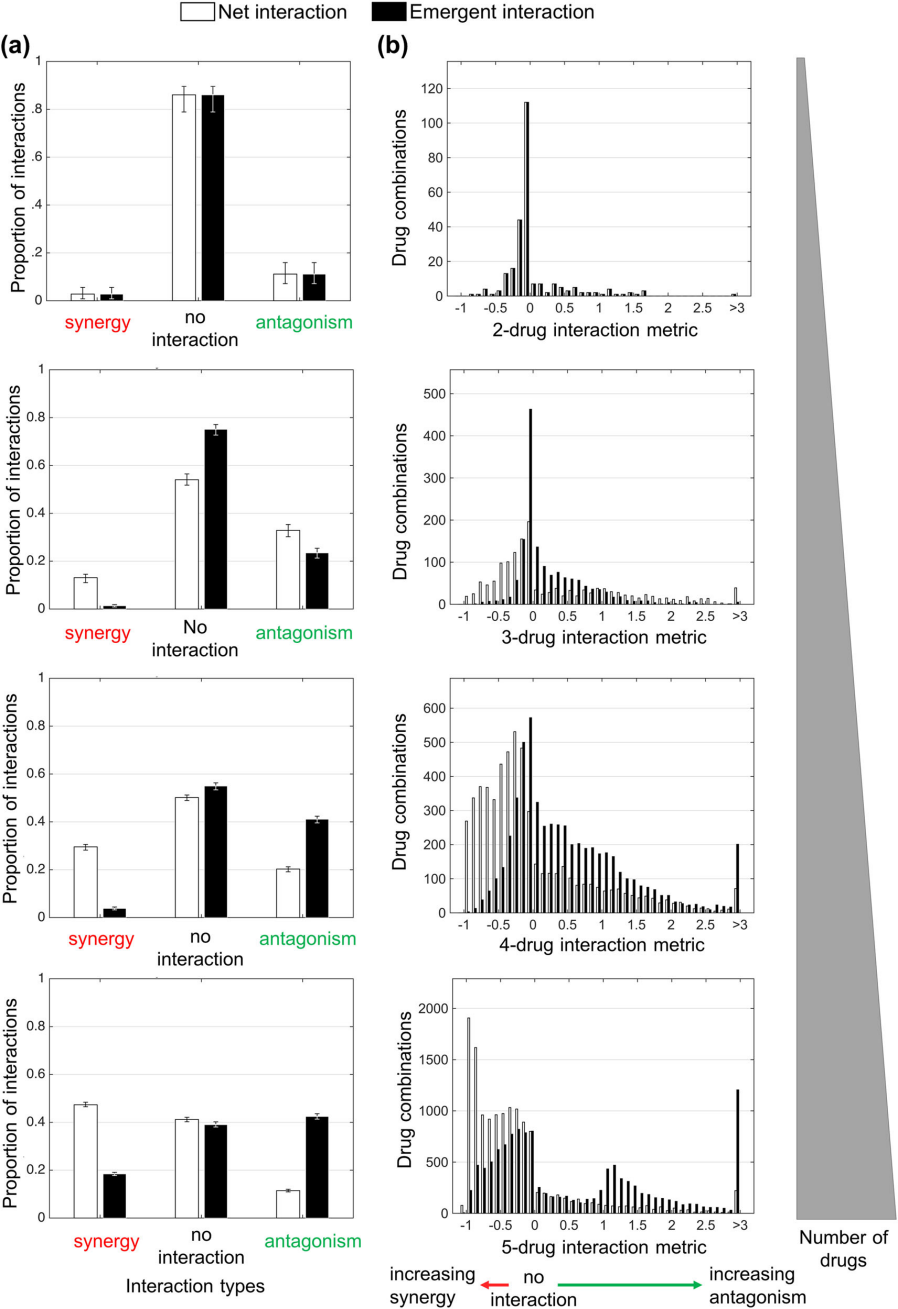
Overall behavior of interaction metric results and categorization of interactions. For each *N*-way interaction, **a** bar plots for interaction classifications (synergy, no-interaction, antagonism) of net (white) and emergent (black) interactions with 95% confidence intervals resulting from bootstrapping experiments via sampling with replacement over all measured drug combinations and **b** histograms of net (white) and emergent (black) interaction metric results with a bin size of 0.1 are plotted. A diagram is shown that displays the direction in which the strength of specific interaction class enlarges. Note here that the definitions of net two-way and emergent two-way interactions are identical. Hence, plots corresponding to the distribution of interaction metrics and the proportion of interactions are indistinguishable for two-drug combinations

As shown in Fig. 2a, (No-interaction bar) and S2 Fig, interactions become significantly more frequent as the number of drugs in *E. coli*’s environment increases (sum of Synergy and Antagonism bars in Fig. 2a). This startling finding suggests not only that higher-order interactions are not negligible and should not be ignored, but that they may be even more important than pairwise interactions in determining the structure and dynamics of systems because they are substantially more prevalent than two-component interactions. Understanding this phenomenon is thus fundamental to understanding interactions in biological and complex systems in general. Evaluating whether any patterns exist in the types of interactions as the number of drugs increases, we found that the net interactions among drugs tend toward more synergy, whereas emergent interactions exhibit a shift toward more antagonism (Fig. 2).

**Fig. 3:**
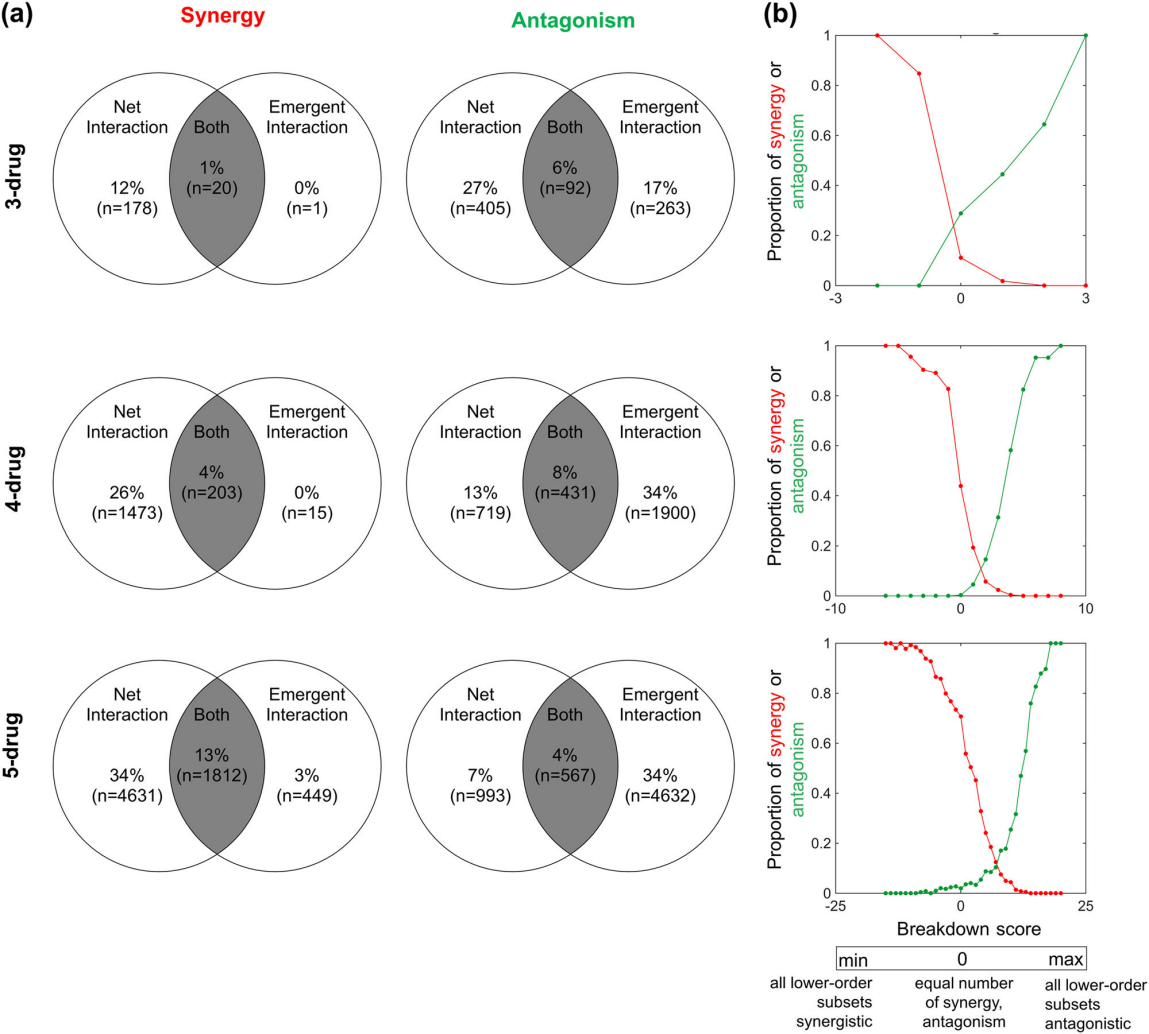
Comparison of net and emergent interactions by synergy and antagonism. **a** Venn diagrams comparing an overlap for different interaction categorizations (synergy: left column, antagonism: right column) according to net and emergent interaction measures of three-, four-, and five-drug combinations. For each *N*-drug Venn diagram, the percentages are calculated relative to the number of *N*-drug combinations. **b** The proportion of net synergies and net antagonisms are plotted versus the total breakdown score. Breakdown scores represent the dominant form of interaction types at the lower-order combinations calculated by the summation of −1 and 1 over each lower-order synergy and lower-order antagonism, respectively (see S1 Table). The minimum and maximum values of breakdown score differ across each plot as the total number of lower-order combinations of an *N*-drug combination depends on the value of *N*

Further dissecting the nature of these interactions and comparing net with emergent interactions, we found that net synergy seldom implies emergent synergy (Fig. 3a, Synergy column). This makes sense because any interactions will create a net effect, while emergence is only an interaction among all drugs in the combination, so will almost certainly be a subset of net interactions. Consequently, a net three-way synergy is usually due to pairwise synergies between two drugs in which a third drug may not be increasing efficacy but still increases toxicity to patients.

In addition, we found that emergent antagonism is less likely to imply net antagonism as the number of drugs increases (Fig. 3a, Antagonism column). Explicitly, this fraction is given by 26%, 18%, and 11% at the three-, four-, and five-drug level, respectively. As another method for summarizing the data, we construct a breakdown score that is calculated as the sum over all lower-order interactions with a 1 added for each lower-order antagonism and a −1 added for each lower-order synergy. With this breakdown score, we can then assess how the summarized category of lower-order interactions affects the net (overall) interaction. For example, when there are three drugs in the bacterial environment (as denoted by *X*_1_*X*_2_*X*_3_in the Theoretical Framework above) and when all three pairwise parts (i.e., *X*_1_*X*_2_, *X*_1_*X*_3_, and *X*_2_*X*_3_) are synergistic, the breakdown score equates to −3, whereas when all are antagonistic, the breakdown score is equal to 3. These two cases represent the minimum and maximum values attained with *N* = 3, respectively. Intriguingly, we showed that lower-order net synergistic effects tend to overcome lower-order net antagonistic effects (Fig. 3b). This demonstrates the fallacy of the prominent presumption that higher-order interactions cancel out and are negligible. Establishing this result was only possible due to our full-factorial experiments and large-scale data set as well as our development and comparison of emergent versus net interaction measures. Overall, our comparison analysis of net and emergent interactions indicates that emergent synergy mostly suggests net synergy, whereas emergent antagonism does not imply net antagonism.

## Discussion

We have explored the consequences of increasing the number of components in the context of net and emergent higher-order interactions through a systematic analysis of bacterial responses in the presence of drug combinations. For both net and emergent higher-order interactions we found the number of interactions substantially increased as the number of drugs increased. Although this may not be surprising for net interactions, it is extremely surprising for emergent interactions because these have been largely ignored in the literature. Yet our new analysis reveals emergent interactions are highly prevalent in our drug systems. Although we have only shown this result for drug combinations, it contradicts the prevailing views and previous limited results in the field.

We also observed an increasing trend toward synergy in net interaction effects and toward antagonism in emergent interaction effects. These trends use tremendous amounts of data to extend and elaborate on recent patterns found in the comparison of two- with three-drug combinations.^24^ These general trends suggest that increasing the number of drugs continually adds new layers of complexity and leads to the natural question of whether this layering of complexity also applies to interactions among multiple components across a myriad of other systems. Indeed, if this finding continues to hold as the number of drugs increases and also applies to other systems and fields, this bypass approach could revolutionize studies of both net and emergent higher-order interactions by making the intractability of the combinatorics suddenly become tractable via systematic patterns that enable predictability.

From a clinical standpoint, synergies offer higher treatment efficacies with low toxicity. Hence, they are valued and used clinically, whereas antagonistic combinations have been traditionally avoided.^11^ Our study reveals that for most higher-order drug combinations, net synergy does not imply emergent synergy. The abundance of such cases suggests the criteria for determining clinically advantageous (or disadvantageous) drug combinations should now consider both net and emergent effects as it is also critical to identify whether addition of drugs yields a real (emergent) benefit that justifies the inclusion of each additional drug in the combination.

Studies on pairwise-drug combinations have shown that antagonistic interactions can lead to a selective advantage of the wild-type pathogen population and reduce the rate of adaptation to drugs.^32,33^ Extending these ideas to more rugged fitness landscapes that correspond to higher-order interactions among drugs and determining the consequences for drug-resistance has not yet been pursued. To our knowledge, this is one of the only and by far the largest set of empirical data obtained to examine the role of net and emergent higher-order interactions. Our observations suggest that the fitness landscapes for multidrug combinations should be extremely rugged due to the pervasiveness of higher-order interactions.

Although our study focuses on a drug-bacteria system, the conceptualization of interaction types (net versus emergent, and synergy versus antagonism) and the systematic analysis of higher-order interactions can be extrapolated into other fields. A relatively straightforward application of our framework includes multiple-stressor effects as drugs in our study are essentially stressors to the bacterial population. Moreover, the interaction model used in multiple predator effect studies is equivalent to the net interaction measure,^34^ suggesting a strong correspondence of our mathematical framework for characterizing emergent interactions.^9^ In microeconomics, relationships between individuals (creating demand) and market firms (supplying demand) affect the allocation of resources.^2^ In the context of social groups, the idea of emergence can be well represented by one individual’s role in controlling a conflict between others in the group. Indeed, studies in primates^35^ and in people^36^ have predicted that cohesion dynamics of groups differ significantly between dyads and triads—groups of two and three people, respectively—with triads being more stable in conserving the association of a group.

Here, we also note several caveats in our framework in its application to clinical practice as well as to other interaction-network settings. From a clinical standpoint, drug combination experiments in this study are carried out in vitro, in highly controllable systems. Hence, in vivo studies of higher-order drug combinations are needed for guiding studies with clinical applications. In addition, as opposed to the drug–bacteria system, natural systems are comprised of many interacting species and functional groups that span trophic levels and that are impacted by diverse but sometimes correlated environmental drivers such as temperature, precipitation, stoichiometry, fires, etc. Consequently, new theory needs to be developed that incorporates additional information to study higher-order interactions and their outcomes in natural systems that involve many more complexities.

In conclusion, we introduce an approach to studying higher-order interactions in biological systems. We provide an enormous amount of data that we analyze with a recently developed theoretical framework to characterize net and emergent interactions among all possible combinations of two, three, four, and five drugs out of a set of eight antibiotics. Notably, we find many interactions that only emerge when multiple drugs are present, and even more surprisingly, we find that the frequency of interactions increases as the number of components in the system increases, contradicting the assumptions and limited findings of many previous studies. Beyond this, we find that emergent interactions tend toward antagonism, whereas net interactions tend toward synergy. These findings suggest that higher-order interactions may be of fundamental importance in understanding and predicting the structure and dynamics of complex biological systems with many interacting parts, such as drugs, genes, food webs, environmental stressors, and more.

Intriguingly, the general questions explored here for drug interactions are highly relevant for other fields, and we anticipate that the research program presented here will be useful for revealing higher-order emergent properties and patterns in ecological, medical, evolutionary, and social systems. It is possible that the prevalence and patterns of higher-order interactions described here will be generic to many other biological and complex systems. Alternatively, these findings may be specific to the type of system being studied. The answer can only be elucidated through future work in other systems. For our drug–bacteria system, we have combined a large-scale experimental system with a new conceptual framework to establish the strong prevalence and importance of higher-order interactions and to identify patterns of these interactions that should help to circumvent combinatorial complexity as well as to inform effective design of multidrug treatments.

## Materials and Methods

### Experimental details

#### Bacterial storage and preparation

Pathogenic *E. Coli* strain CFT073 (ATCC^®^ 700928™) isolated from human clinical specimens was used for all experiments. Bacterial aliquots were made using a single colony collected using streak purification. The aliquots were stored in 25% glycerol at − 80 °C. A culture was prepared each day using a thawed aliquot diluted 10^−2^ in Luria Broth (10 g/l tryptone, 5 g/l yeast extract, and 10 g/l NaCl). The culture was grown at 37 °C for about 4 h.

#### Antibiotics

We used eight antibiotics in this study that were chosen specifically to cover a broad range of antibiotic mechanisms of action.^37^ Additionally, drugs needed to be soluble in dimethyl sulfoxide (DMSO) and therefore were chosen based on solubility properties. These antibiotics were Ampicillin (Sigma A9518), Cefoxitin Sodium Salt (Sigma C4786), Ciprofloxacin Hydrochloride (MP Biomedicals 199020), Doxycycline hyclate (Sigma D9891), Erythromycin (Sigma Aldrich E6376), Fusidic Acid Sodium Salt (Sigma F0881), Streptomycin (Sigma Aldrich S6501), and Trimethoprim (Sigma T7883). A list of drugs, abbreviations, mechanisms of action, and concentrations used in this assay is in Table 1.

**Table 1:** Summary of antibiotics

Antibiotic	Abbreviation	Mechanism of action	Inhibitory concentration (IC)	Concentration (µM)
Ampicillin	AMP	Cell wall	IC_10_	2.89
			IC_5_	2.52
			IC_1_	1.87
Cefoxitin sodium salt	FOX	Cell wall	IC_10_	1.78
			IC_5_	1.37
			IC_1_	0.78
Trimethoprim	TMP	Folic acid biosynthesis	IC_10_	0.22
			IC_5_	0.15
			IC_1_	0.07
Ciprofloxacin hydrochloride	CPR	DNA gyrase	IC_10_	0.03
			IC_5_	0.02
			IC_1_	0.01
Streptomycin	STR	Aminoglycoside, protein synthesis, 30S	IC_10_	19.04
			IC_5_	16.6
			IC_1_	12.25
Doxycycline hyclate	DOX	Protein synthesis, 50S	IC_10_	0.35
			IC_5_	0.27
			IC_1_	0.15
Erythromycin	ERY	Protein synthesis, 50S	IC_10_	16.62
			IC_5_	8.29
			IC_1_	1.78
Fusidic acid sodium salt	FUS	Protein synthesis, 30S	IC_10_	94.42
			IC_5_	71.01
			IC_1_	37.85

The antibiotics used are listed with their mechanism of action and concentrations corresponding to 10, 5, and 1% inhibitory concentration levels (IC10, IC5, and IC1, respectively)

#### Antibiotic concentration determination and preparation

A dose curve was generated to determine antibiotic drug concentrations for this assay. Dose curves were generated using 20 drug concentrations with a dilution factor of two and a starting concentration of 0.1 mM. For Fusidic Acid, the highest concentration was 1 mM, as the lower concentrations were found to be ineffective in generating lethality needed to determine the IC50 (50% inhibition concentration). Graphpad Prism 7 was used to graph the dose curve and determine the IC50. In addition, Graphpad (http://www.graphpad.com/quickcalcs/Ecanything1/) was used to estimate the IC10, IC5, and IC1. Minimally effective concentrations were chosen in order to maintain bacterial growth even in multidrug combinations. Each antibiotic was weighed and solubilized in 100% DMSO (Sigma), except for Streptomycin, which was solubilized in 50% DMSO, to a final concentration of × 400 the determined IC10, IC5, and IC1 concentrations. Antibiotics were then pipetted into a source plate (Thermo Scientific) as either a single antibiotic with additional DMSO or in combination with another antibiotic. The final concentration in the source plate was × 200 the target concentration.

#### Experimental setup

In total, we tested all two-, three-, four-, and five-drug combinations from a set of eight antibiotics (Table 1), meaning that 28, 56, 70, and 56 distinct drug combinations were tested at given concentrations, respectively. For each experiment, 25 µL of Luria Broth was added to each well of a 384-well plate (Greiner BioOne) using a Multidrop 384 (Thermo Scientific). An additional 25 µL of media was added to the media-only control. Using a Biomek FX (Beckman Coulter) with a 250 nL pin tool (V&P Scientific), we pinned 250 nL from every well of each of the three premade source plates (one plate with one antibiotic and DMSO and two plates with two antibiotics in combination) into the experimental plate. A 25 µL of a 10^-4^ dilution of the overday culture was then added to each well (except for the negative control). Plates were incubated at 37°C and read using an OD_590_ measurement every 4 h for 16 h. Each two-, three-, four- and five-drug experiment was tested at least three times (S1 Data).

#### Growth measurements

For each plate, the Z’-factor^38^ was calculated to determine the quality of the assay. If a Z’ value was below 0.5, the plate was not used for final analysis. For a few plates, there was one well that was more than two standard deviations from the average of the negative control. For these plates, that well was removed from the final Z’-factor calculation. The exponential rate of growth was determined for each experimental well and compared with the average exponential rate of growth for the no drug control to give a growth percentage. Growth percentages were used to determine interaction types based on a framework developed in,^9,24^ as summarized below.

### Mathematical framework

Here, we derive the formula for emergent three-way interactions and also generalize the emergent interaction measure to higher orders and any *N*-way combination. As described briefly in “Theoretical Framework for the Characterization of Higher-order Interactions”, we accomplish this by starting from the definition of Bliss Independence (i.e., N_*N*_) and by subtracting all lower-order contribution effects from N_*N*_. For any *N*, we can denote these lower-order contributions by $\left[ {{\mathrm{N}}_N} \right]_{D_1 \bot D_2 \bot \ldots \bot D_n}$, where ⊥ represents no-interaction, and *D*_*i*_ represents one or more drugs that is a subset of interacting components (i.e., combinations of drugs). These subsets are composed of *n* disjoint sets of drugs with the union of all such sets equaling the total set of all drugs (i.e., *D*_1_U *D*_2_ U … U *D*_*n*_ = *X*_1_*X*_2_… *X*_*N*_) because all subsets must combine to produce the original *N*-drug combination. For this reason, no drug is allowed to occur in more than one subset of drugs. In other words, for any lower-order contribution term, each drug can be part of only one subset. For example, $\left[ {{\mathrm{N}}_3} \right]_{X_1X_2 \bot X_3}$, where *D*_*1*_ = *X*_1_*X*_2_ and *D*_*2*_ = *X*_3_, represents the pairwise combination effect of *X*_1_*X*_2_ on the three-way interaction, and it measures the expected three-way effect when the remaining drug *X*_3_ does not interact with the pair. This effect is calculated as

$$\left[ {{\mathrm{N}}_3} \right]_{X_1X_2 \bot X_3} = w_{X_1X_2}w_{X_3} - {\mathrm{w}}_{{\mathrm{X}}_1}w_{X_2}w_{X_3} = w_{X_3}\left[ {{\mathrm{N}}_2} \right]_{X_1X_2}$$

For measuring emergent interactions, all different factorizations of drugs are subtracted in a combinatorial fashion. When *N* = 3, all the pairwise combination effects are subtracted from the net three-way interaction

$${\mathrm{E}}_3 = {\mathrm{N}}_3 - \left[ {{\mathrm{N}}_3} \right]_{X_1X_2 \bot X_3} - \left[ {{\mathrm{N}}_3} \right]_{X_1X_3 \bot X_2} - \left[ {{\mathrm{N}}_3} \right]_{X_2X_3 \bot X_1}$$

Analogously calculating the effects due to *X*_1_*X*_3_ and *X*_2_*X*_3_ (i.e., $\left[ {{\mathrm{N}}_3} \right]_{{{X}}_1{{X}}_3 \bot {{X}}_2}$ and $\left[ {{\mathrm{N}}_3} \right]_{{{X}}_2{{X}}_3 \bot {{X}}_1}$), the definition of *E*_3_ yields Eq. (3) in the “Theoretical Framework for the Characterization of Higher-order Interactions”.

The most challenging issue in deriving higher-order equations for more than three drugs is to carefully correct for the possible double counting of interactions when subtracting lower-order effects that may be subsumed in multiple higher-order terms. For instance, the lower-order interaction terms of $\left[ {{\mathrm{N}}_4} \right]_{X_1 \bot X_2X_3X_4}$ and $\left[ {{\mathrm{N}}_4} \right]_{X_2 \bot X_1X_3X_4}$both subsume the effect of $\left[ {{\mathrm{N}}_4} \right]_{X_3X_4 \bot X_1 \bot X_2}$ as all three are identical when *X*_1_, *X*_2_, and the combined drug pair *X*_3_*X*_4_ are all non-interacting with respect to each other. Such cases should be dealt with systematically to make sure that each lower-order effect is subtracted off exactly once (see S1 Text). Moreover, when *N* > 3, it is also necessary to exclude the effect that results from interactions of different subsets of drug combinations. For example, the two-way interactions of *X*_1_*X*_2_ and *X*_3_*X*_4_ on the four-way combination of *X*_1_*X*_2_*X*_3_*X*_4_, as denoted by $\left[ {{\mathrm{N}}_4} \right]_{X_1X_2 \bot X_3X_4}$ and calculated as $w_{X_1X_2}w_{X_3X_4} - {\mathrm{w}}_{{\mathrm{X}}_1}w_{X_2}w_{X_3}w_{X_4}$, must be accounted for when considering all lower-order effects.

Following the same logic as in the calculation of pairwise interactions, the lower-order contribution of drugs, i.e., $\left[ {{\mathrm{N}}_N} \right]_{D_1 \bot D_2 \bot \ldots \bot D_n}$, is given by the expected *N*-way effect when only interactions within the non-single-drug subsets (i.e., *D*_*i*_ containing more than one drug) are present. Thus, lower-order effects from the mixture of lower-order combinations and single drugs complementing the total combination will be calculated similarly by replacing the *N*-way drug response, $w_{X_1X_2 \ldots X_N},$ with the product of drug combination responses that are assumed to be only interacting pieces within the *N*-drug combination as

$$\left[ {{\mathrm{N}}_N} \right]_{D_1 \bot D_2 \bot \ldots \bot D_n} = w_{D_1}w_{D_2} \ldots w_{D_n} - w_{D_1}w_{D_2} \ldots w_{D_N}$$

For example, the *k*-drug effect of *X*_1_*X*_2_…*X*_*k*_ on the *N*-way combination for any value of *N* and *k* with *k* < *N* is given by the expected *N*-way effect when only *k* drugs interact, i.e. $\left[ {{\mathrm{N}}_N} \right]_{X_1X_2 \ldots X_k \bot X_{k + 1} \bot \ldots \bot X_N} = w_{X_1X_2 \ldots X_k}w_{X_{k + 1}} \ldots w_{X_N} - w_{X_1}w_{X_2} \ldots w_{X_N}$. Indeed, this is equivalent to the net interaction arising from the *k*-drug combination multiplied by the single-drug fitnesses of the remaining *N*−*k* drugs: $w_{X_{k + 1}} \ldots w_{X_N}\left[ {{\mathrm{N}}_k} \right]_{X_1X_2 \ldots X_k}$.

Subtracting all possible lower-order interaction contributions (via factorizations of *D*_1_⊥ *D*_2_⊥…⊥ *D*_*n*_) from the net *N*-way interaction defines newly emergent interactions among all *N* drugs. Accordingly, we now define E_4_ and E_5_ purely in terms of fitness measurements based on the conceptual framework just described and using the above formulas and notation for the lower-order component effects. Guaranteeing that overcounting is eliminated when distinguishing every possible lower-order effect from the net interaction (see S1 Text), the emergent four-way interaction is given by

$$\begin{array}{*{20}{l}} {{\mathrm{E}}_4} \hfill & = \hfill & {w_{X_1X_2X_3X_4} - w_{X_1}w_{X_2X_3X_4} - w_{X_2}w_{X_1X_3X_4} - w_{X_3}w_{X_1X_2X_4} - w_{X_4}w_{X_1X_2X_3}} \hfill \cr {} \hfill & {} \hfill & { - w_{X_1X_2}w_{X_3X_4} - w_{X_1X_3}w_{X_2X_4} - w_{X_1X_4}w_{X_2X_3} + 2w_{X_1}w_{X_2}w_{X_3X_4}} \hfill \cr {} \hfill & {} \hfill & { + 2w_{X_1}w_{X_3}w_{X_2X_4} + 2w_{X_1}w_{X_4}w_{X_2X_3} + 2w_{X_2}w_{X_3}w_{X_1X_4} + 2w_{X_2}w_{X_4}w_{X_1X_3}} \hfill \cr {} \hfill & {} \hfill & { + 2w_{X_3}w_{X_4}w_{X_1X_2} - 6w_{X_1}w_{X_2}w_{X_3}w_{X_4}} \hfill \end{array}$$

The emergent five-way measure in terms of relative fitnesses is given as

$$\it \begin{array}{l}{\mathrm{E}}_5 = w_{X_1X_2X_3X_4X_5} - w_{X_1}w_{X_2X_3X_4X_5} - w_{X_2}w_{X_1X_3X_4X_5} - w_{X_3}w_{X_1X_2X_4X_5} - w_{X_4}w_{X_1X_2X_3X_5} - \cr w_{X5}w_{X_1X_2X_3X_4} - w_{X_1X_2}w_{X_3X_4X_5} - w_{X_1X_3}w_{X_2X_4X_5} - w_{X_1X_4}w_{X_2X_3X_5} - w_{X_1X_5}w_{X_2X_3X_4} - \cr w_{X_2X_3}w_{X_1X_4X_5} - w_{X_2X_4}w_{X_1X_3X_5} - w_{X_2X_5}w_{X_1X_3X_4} - w_{X_3X_4}w_{X_1X_2X_5} - w_{X_3X_5}w_{X_1X_2X_4} - w_{X_4X_5} - \cr w_{X_4X_5}w_{X_1X_2X_3} + 2w_{X_1}w_{X_2}w_{X_3X_4X_5} + 2w_{X_1}w_{X_3}w_{X_2X_4X_5} + 2w_{X_1}w_{X_4}w_{X_2X_3X_5} + \cr 2w_{X_1}w_{X_5}w_{X_2X_3X_4} + 2w_{X_2}w_{X_3}w_{X_1X_4X_5} + 2w_{X_2}w_{X_4}w_{X_1X_3X_5} + 2w_{X_2}w_{X_5}w_{X_1X_3X_4} + \cr 2w_{X_3}w_{X_4}w_{X_1X_2X_5} + 2w_{X_3}w_{X_5}w_{X_1X_2X_4} + 2w_{X_4}w_{X_5}w_{X_1X_2X_3} + 2w_{X_1X_2}w_{X_3X_4}w_{X_5} + \cr 2w_{X_1X_2}w_{X_3X_5}w_{X_4} + 2w_{X_1X_2}w_{X_4X_5}w_{X_3} + 2w_{X_1X_3}w_{X_2X_4}w_{X_5} + 2w_{X_1X_3}w_{X_2X_5}w_{X_4} + \cr 2w_{X_1X_3}w_{X_4X_5}w_{X_2} + 2w_{X_1X_4}w_{X_2X_3}w_{X_5} + 2w_{X_1X_4}w_{X_2X_5}w_{X_3} + 2w_{X_1X_4}w_{X_3X_5}w_{X_2} + \cr 2w_{X_1X_5}w_{X_2X_3}w_{X_4} + 2w_{X_1X_5}w_{X_2X_4}w_{X_3} + 2w_{X_1X_5}w_{X_3X_4}w_{X_2} + 2w_{X_2X_3}w_{X_4X_5}w_{X_1} + \cr 2w_{X_2X_4}w_{X_3X_5}w_{X_1} + 2w_{X_2X_5}w_{X_3X_4}w_{X_1} - 6w_{X_1X_2}w_{X_3}w_{X_4}w_{X_5} - 6w_{X_1X_3}w_{X_2}w_{X_4}w_{X_5} - \cr 6w_{X_1X_4}w_{X_2}w_{X_3}w_{X_5} - 6w_{X_1X_5}w_{X_2}w_{X_3}w_{X_4} - 6w_{X_2X_3}w_{X_1}w_{X_4}w_{X_5} - 6w_{X_2X_4}w_{X_1}w_{X_3}w_{X_5} - \cr 6w_{X_2X_5}w_{X_1}w_{X_3}w_{X_4} - 6w_{X_3X_4}w_{X_1}w_{X_2}w_{X_5} - 6w_{X_3X_5}w_{X_1}w_{X_2}w_{X_4} - 6w_{X_4X_5}w_{X_1}w_{X_2}w_{X_3} + \cr 24w_{X_1}w_{X_2}w_{X_3}w_{X_4}w_{X_5}\end{array}$$

Notably, analyzing deviations from appropriate baselines of these interaction measures allows us to assign information and meaning to the magnitude of the interaction and thus form a correspondence with the type and strength of interaction. This is accomplished via rescaling methods we developed for these interaction measures as defined in ref.^24,28,29^ (see S2 Text) and that we use to analyze the experimental data of drug combinations.

#### Details of data analysis and cutoff values for the categorization of interactions

Median growth measurements for each experiment across replicates were used to determine net and emergent interaction types according to the rescaled interaction metric definitions. Owing to the full-factorial design of our experiments, bacterial growth in the presence of each *k*-drug combination is measured within each drug combination experiment that contains that specific *k*-way drug combination (the pairwise combination *X*_1_*X*_*2*_ is repeated within the experiments of *X*_1_*X*_*2*_*X*_3_, *X*_1_*X*_*2*_*X*_4_, and so on). For such cases (i.e., for two-, three-, and four-drug combinations) the median interaction metric calculation across these experiments was used to determine the interaction type of each drug combination. Given the interaction metric calculation, we categorize synergy, additivity, and antagonism regions according to cutoff values established by previous work.^24,28,39^ The interaction among drugs is identified as synergistic when the interaction metric is < −0.5, antagonistic if it is larger than 0.5, and additive if it ranges between −0.5 and 0.5. Note that the total range of the rescaled metric is from −1 to 1 when the combination of drugs reduces the growth relative to at least one of the lower-order combination growth rates (i.e., fitness), hence leading to rare instances of values above 1.

Finally, we excluded several specific cases in our analysis. First, for two-drug combinations *X*_1_*X*_2_, the effect of the second drug on the bacterial growth is indistinguishable when both the maximum of single and pairwise growth measurements (i.e., $\max \left( {w_{X_1},w_{X_2}} \right)$ and $w_{X_2}$) are > 90% growth.^40^ Next, using the same reasoning, in the extreme case that *k* drugs by themselves kill off the bacteria populations (lethality: measurements below 4.7% as determined by Tekin et al.^24^) and the addition of another drug into the environment also leads to lethality, then it is meaningless to look for emergent *k* + 1 drug interactions. We identify these cases as inconclusive and excluded them in our data analysis of the frequency of interaction types.

### Data availability

The data sets for this article have been uploaded as part of the Supplementary Information.

### Code availability

Data analysis is performed in MATLAB version R2015a. The scripts are available upon request.

## Electronic supplementary material

Supplementary Information

Dataset

## Electronic supplementary material

**Supplementary information** accompanies the paper on the *npj Systems Biology and Applications* website (10.1038/s41540-018-0069-9).
