# Supplementary material for: Prevalence and patterns of higher-order drug interactions in Escherichia coli
Source: NPJ Syst Biol Appl. 2018 Sep 3;4:31. doi: 10.1038/s41540-018-0069-9 (PMC6119685; doi:10.1038/s41540-018-0069-9)
Supplement: Supplementary file 2 — Dataset [file 41540_2018_69_MOESM2_ESM.pdf]

AMP1CPR1ERY1DOX1TMP1	AMP	CPR	ERY	DOX	TMP	AMP+CPR	AMP+ERY	AMP+DOX	AMP+TMP	CPR+ERY	CPR+DOX	CPR+TMP	ERY+DOX	ERY+TMP	DOX+TMP	AMP+CPR+ERY	AMP+CPR+DOX	AMP+CPR+TMP	AMP+ERY+DOX	AMP+ERY+TMP	AMP+DOX+TMP	CPR+ERY+DOX	CPR+ERY+TMP	CPR+DOX+TMP	ERY+DOX+TMP	AMP+CPR+ERY+DOX	AMP+CPR+ERY+TMP	AMP+CPR+DOX+TMP	AMP+ERY+DOX+TMP	CPR+ERY+DOX+TMP	AMP+CPR+ERY+DOX+TMP
	86.21081004	91.65839126	90.29649596	98.24088523	91.88537381	81.44417648	81.21719393	88.253653	87.7996879	100.9646758	101.4186409	88.93460065	83.71400199	90.29649596	92.79330401	80.08228117	80.76322883	89.84253086	77.13150802	93.70123422	99.14881543	97.55993758	90.97744361	95.51709462	80.99021138	71.45694425	85.75684494	91.88537381	98.24088523	85.07589729	85.30287984
	86.39503736	101.5085295	96.99703933	105.1177217	100.1550825	73.98843931	77.37205696	83.46256873	96.99703933	99.02720993	103.3131256	103.5387001	95.19244325	100.1550825	99.25278444	74.43958833	77.59763147	88.87635697	80.75567461	96.09474129	97.22261384	90.45537854	99.47835895	101.0573805	90.45537854	80.30452559	89.77865501	94.51571972	88.87635697	87.97405893	81.20682363
	74.51039019	92.45029152	94.12468231	98.66945732	106.8022126	72.11840335	72.59680072	85.99192704	96.03827179	95.79907311	100.8222455	106.5630139	94.363881	102.0182389	107.28061	72.35760203	90.77590073	96.51666916	72.11840335	94.363881	95.79907311	94.363881	96.51666916	106.0846165	96.03827179	94.363881	97.95186126	103.453431	82.88234415	89.57990731	106.0846165
AMP1CPR1ERY1DOX1TMP2	AMP	CPR	ERY	DOX	TMP	AMP+CPR	AMP+ERY	AMP+DOX	AMP+TMP	CPR+ERY	CPR+DOX	CPR+TMP	ERY+DOX	ERY+TMP	DOX+TMP	AMP+CPR+ERY	AMP+CPR+DOX	AMP+CPR+TMP	AMP+ERY+DOX	AMP+ERY+TMP	AMP+DOX+TMP	CPR+ERY+DOX	CPR+ERY+TMP	CPR+DOX+TMP	ERY+DOX+TMP	AMP+CPR+ERY+DOX	AMP+CPR+ERY+TMP	AMP+CPR+DOX+TMP	AMP+ERY+DOX+TMP	CPR+ERY+DOX+TMP	AMP+CPR+ERY+DOX+TMP
	86.21081004	91.65839126	90.29649596	98.24088523	95.97105972	81.44417648	81.21719393	88.253653	102.7805362	100.9646758	101.4186409	96.87898993	83.71400199	88.48063555	95.51709462	80.08228117	80.76322883	89.1615832	77.13150802	94.15519932	101.872606	97.55993758	94.83614697	92.11235636	95.06312952	71.45694425	85.52986239	89.1615832	96.87898993	96.65200738	92.11235636
	86.39503736	101.5085295	96.99703933	105.1177217	102.1852531	73.98843931	77.37205696	83.46256873	98.3504864	99.02720993	103.3131256	108.5013393	95.19244325	100.6062315	102.8619766	74.43958833	77.59763147	90.00422952	80.75567461	94.96686874	98.80163542	90.45537854	101.9596786	100.6062315	99.02720993	80.30452559	90.22980403	89.32750599	90.45537854	97.89933737	84.81601579
	74.51039019	92.45029152	94.12468231	98.66945732	110.1509942	72.11840335	72.59680072	85.99192704	101.7790402	95.79907311	100.8222455	110.6293915	94.363881	103.453431	106.3238152	72.35760203	90.77590073	95.32067574	72.11840335	93.88548363	100.1046494	94.363881	98.43025863	110.1509942	106.8022126	94.363881	96.27747047	94.363881	88.38391389	97.95186126	102.9750336
AMP1CPR1ERY1DOX1TMP3	AMP	CPR	ERY	DOX	TMP	AMP+CPR	AMP+ERY	AMP+DOX	AMP+TMP	CPR+ERY	CPR+DOX	CPR+TMP	ERY+DOX	ERY+TMP	DOX+TMP	AMP+CPR+ERY	AMP+CPR+DOX	AMP+CPR+TMP	AMP+ERY+DOX	AMP+ERY+TMP	AMP+DOX+TMP	CPR+ERY+DOX	CPR+ERY+TMP	CPR+DOX+TMP	ERY+DOX+TMP	AMP+CPR+ERY+DOX	AMP+CPR+ERY+TMP	AMP+CPR+DOX+TMP	AMP+ERY+DOX+TMP	CPR+ERY+DOX+TMP	AMP+CPR+ERY+DOX+TMP
	86.21081004	91.65839126	90.29649596	98.24088523	98.69485033	81.44417648	81.21719393	88.253653	94.15519932	100.9646758	101.4186409	101.4186409	83.71400199	92.11235636	100.9646758	80.08228117	80.76322883	90.97744361	77.13150802	93.70123422	100.9646758	97.55993758	103.4614839	105.7313094	96.65200738	71.45694425	78.26642077	85.75684494	95.97105972	100.9646758	78.03943822
	86.39503736	101.5085295	96.99703933	105.1177217	98.80163542	73.98843931	77.37205696	83.46256873	90.90652756	99.02720993	103.3131256	105.5688707	95.19244325	99.02720993	105.3432962	74.43958833	77.59763147	85.94388834	80.75567461	90.22980403	98.3504864	90.45537854	103.0875511	104.8921472	99.02720993	80.30452559	80.98124912	83.23699422	85.49273932	100.380657	83.68814324
	74.51039019	92.45029152	94.12468231	98.66945732	109.1941994	72.11840335	72.59680072	85.99192704	79.77276125	95.79907311	100.8222455	109.6725968	94.363881	105.6062192	109.4333981	72.35760203	90.77590073	95.55987442	72.11840335	86.94872178	99.62625206	94.363881	101.7790402	112.7821797	108.7158021	94.363881	89.10150994	96.51666916	85.75272836	106.0846165	80.72955599
AMP1CPR1ERY1DOX2TMP1	AMP	CPR	ERY	DOX	TMP	AMP+CPR	AMP+ERY	AMP+DOX	AMP+TMP	CPR+ERY	CPR+DOX	CPR+TMP	ERY+DOX	ERY+TMP	DOX+TMP	AMP+CPR+ERY	AMP+CPR+DOX	AMP+CPR+TMP	AMP+ERY+DOX	AMP+ERY+TMP	AMP+DOX+TMP	CPR+ERY+DOX	CPR+ERY+TMP	CPR+DOX+TMP	ERY+DOX+TMP	AMP+CPR+ERY+DOX	AMP+CPR+ERY+TMP	AMP+CPR+DOX+TMP	AMP+ERY+DOX+TMP	CPR+ERY+DOX+TMP	AMP+CPR+ERY+DOX+TMP
	86.21081004	91.65839126	90.29649596	99.82976309	91.88537381	81.44417648	81.21719393	90.52347851	87.7996879	100.9646758	98.46786778	88.93460065	93.47425167	90.29649596	97.33295503	80.08228117	80.53624628	89.84253086	83.48701944	93.70123422	98.92183288	101.6456235	90.97744361	98.46786778	95.97105972	72.36487445	85.75684494	86.66477515	95.06312952	98.01390268	83.71400199
	86.39503736	101.5085295	96.99703933	103.9898491	100.1550825	73.98843931	77.37205696	93.83899619	96.99703933	99.02720993	103.9898491	103.5387001	96.99703933	100.1550825	98.12491188	74.43958833	82.56027069	88.87635697	82.7858452	96.09474129	99.47835895	92.71112364	99.47835895	94.96686874	96.99703933	78.95107853	89.77865501	93.83899619	78.72550402	96.54589031	93.38784717
	74.51039019	92.45029152	94.12468231	105.1278218	106.8022126	72.11840335	72.59680072	91.49349679	96.03827179	95.79907311	100.3438481	106.5630139	96.27747047	102.0182389	99.86545074	72.35760203	92.45029152	96.51666916	79.77276125	94.363881	103.2142323	101.5398415	96.51666916	107.28061	100.3438481	92.21109284	97.95186126	101.0614442	95.55987442	105.3670205	87.90551652
AMP1CPR1ERY1DOX2TMP2	AMP	CPR	ERY	DOX	TMP	AMP+CPR	AMP+ERY	AMP+DOX	AMP+TMP	CPR+ERY	CPR+DOX	CPR+TMP	ERY+DOX	ERY+TMP	DOX+TMP	AMP+CPR+ERY	AMP+CPR+DOX	AMP+CPR+TMP	AMP+ERY+DOX	AMP+ERY+TMP	AMP+DOX+TMP	CPR+ERY+DOX	CPR+ERY+TMP	CPR+DOX+TMP	ERY+DOX+TMP	AMP+CPR+ERY+DOX	AMP+CPR+ERY+TMP	AMP+CPR+DOX+TMP	AMP+ERY+DOX+TMP	CPR+ERY+DOX+TMP	AMP+CPR+ERY+DOX+TMP
	86.21081004	91.65839126	90.29649596	99.82976309	95.97105972	81.44417648	81.21719393	90.52347851	102.7805362	100.9646758	98.46786778	96.87898993	93.47425167	88.48063555	100.9646758	80.08228117	80.53624628	89.1615832	83.48701944	94.15519932	100.9646758	101.6456235	94.83614697	103.915449	97.55993758	72.36487445	85.52986239	96.87898993	74.63469996	98.01390268	85.07589729
	86.39503736	101.5085295	96.99703933	103.9898491	102.1852531	73.98843931	77.37205696	93.83899619	98.3504864	99.02720993	103.9898491	108.5013393	96.99703933	100.6062315	98.80163542	74.43958833	82.56027069	90.00422952	82.7858452	94.96686874	102.1852531	92.71112364	101.9596786	100.1550825	91.8088256	78.95107853	90.22980403	97.44818835	93.16227266	96.3203158	87.74848442
	74.51039019	92.45029152	94.12468231	105.1278218	110.1509942	72.11840335	72.59680072	91.49349679	101.7790402	95.79907311	100.3438481	110.6293915	96.27747047	103.453431	99.14785469	72.35760203	92.45029152	95.32067574	79.77276125	93.88548363	104.8886231	101.5398415	98.43025863	108.2374047	93.88548363	92.21109284	96.27747047	102.2574376	96.03827179	104.1710271	82.88234415
AMP1CPR1ERY1DOX2TMP3	AMP	CPR	ERY	DOX	TMP	AMP+CPR	AMP+ERY	AMP+DOX	AMP+TMP	CPR+ERY	CPR+DOX	CPR+TMP	ERY+DOX	ERY+TMP	DOX+TMP	AMP+CPR+ERY	AMP+CPR+DOX	AMP+CPR+TMP	AMP+ERY+DOX	AMP+ERY+TMP	AMP+DOX+TMP	CPR+ERY+DOX	CPR+ERY+TMP	CPR+DOX+TMP	ERY+DOX+TMP	AMP+CPR+ERY+DOX	AMP+CPR+ERY+TMP	AMP+CPR+DOX+TMP	AMP+ERY+DOX+TMP	CPR+ERY+DOX+TMP	AMP+CPR+ERY+DOX+TMP
	86.21081004	91.65839126	90.29649596	99.82976309	98.69485033	81.44417648	81.21719393	90.52347851	94.15519932	100.9646758	98.46786778	101.4186409	93.47425167	92.11235636	100.7376933	80.08228117	80.53624628	90.97744361	83.48701944	93.70123422	97.55993758	101.6456235	103.4614839	102.5535537	97.55993758	72.36487445	78.26642077	87.7996879	89.1615832	103.0075188	89.38856575
	86.39503736	101.5085295	96.99703933	103.9898491	98.80163542	73.98843931	77.37205696	93.83899619	90.90652756	99.02720993	103.9898491	105.5688707	96.99703933	99.02720993	98.80163542	74.43958833	82.56027069	85.94388834	82.7858452	90.22980403	93.16227266	92.71112364	103.0875511	95.41801776	90.45537854	78.95107853	80.98124912	84.81601579	90.45537854	91.8088256	82.7858452
	74.51039019	92.45029152	94.12468231	105.1278218	109.1941994	72.11840335	72.59680072	91.49349679	79.77276125	95.79907311	100.3438481	109.6725968	96.27747047	105.6062192	98.19105995	72.35760203	92.45029152	95.55987442	79.77276125	86.94872178	98.19105995	101.5398415	101.7790402	103.6926297	91.73269547	92.21109284	89.10150994	78.33756914	90.77590073	105.8454179	83.5999402
AMP1CPR1ERY1DOX3TMP1	AMP	CPR	ERY	DOX	TMP	AMP+CPR	AMP+ERY	AMP+DOX	AMP+TMP	CPR+ERY	CPR+DOX	CPR+TMP	ERY+DOX	ERY+TMP	DOX+TMP	AMP+CPR+ERY	AMP+CPR+DOX	AMP+CPR+TMP	AMP+ERY+DOX	AMP+ERY+TMP	AMP+DOX+TMP	CPR+ERY+DOX	CPR+ERY+TMP	CPR+DOX+TMP	ERY+DOX+TMP	AMP+CPR+ERY+DOX	AMP+CPR+ERY+TMP	AMP+CPR+DOX+TMP	AMP+ERY+DOX+TMP	CPR+ERY+DOX+TMP	AMP+CPR+ERY+DOX+TMP
	86.21081004	91.65839126	90.29649596	104.1424316	91.88537381	81.44417648	81.21719393	90.52347851	87.7996879	100.9646758	102.0995886	88.93460065	90.75046106	90.29649596	104.1424316	80.08228117	87.11874025	89.84253086	87.11874025	93.70123422	102.3265711	97.33295503	90.97744361	101.1916584	99.60278054	74.18073486	85.75684494	100.0567456	87.7996879	97.33295503	95.74407717
	86.39503736	101.5085295	96.99703933	105.5688707	100.1550825	73.98843931	77.37205696	96.09474129	96.99703933	99.02720993	108.2757648	103.5387001	97.22261384	100.1550825	92.25997462	74.43958833	83.68814324	88.87635697	85.49273932	96.09474129	92.03440011	95.19244325	99.47835895	91.58325109	82.56027069	79.62780206	89.77865501	96.54589031	95.86916678	82.7858452	89.77865501
	74.51039019	92.45029152	94.12468231	103.6926297	106.8022126	72.11840335	72.59680072	95.79907311	96.03827179	95.79907311	102.0182389	106.5630139	100.8222455	102.0182389	93.16788758	72.35760203	93.16788758	96.51666916	80.4903573	94.363881	99.86545074	99.86545074	96.51666916	102.0182389	85.51352968	86.94872178	97.95186126	92.68949021	89.34070863	97.23426521	93.88548363
AMP1CPR1ERY1DOX3TMP2	AMP	CPR	ERY	DOX	TMP	AMP+CPR	AMP+ERY	AMP+DOX	AMP+TMP	CPR+ERY	CPR+DOX	CPR+TMP	ERY+DOX	ERY+TMP	DOX+TMP	AMP+CPR+ERY	AMP+CPR+DOX	AMP+CPR+TMP	AMP+ERY+DOX	AMP+ERY+TMP	AMP+DOX+TMP	CPR+ERY+DOX	CPR+ERY+TMP	CPR+DOX+TMP	ERY+DOX+TMP	AMP+CPR+ERY+DOX	AMP+CPR+ERY+TMP	AMP+CPR+DOX+TMP	AMP+ERY+DOX+TMP	CPR+ERY+DOX+TMP	AMP+CPR+ERY+DOX+TMP
	86.21081004	91.65839126	90.29649596	104.1424316	95.97105972	81.44417648	81.21719393	90.52347851	102.7805362	100.9646758	102.0995886	96.87898993	90.75046106	88.48063555	99.37579799	80.08228117	87.11874025	89.1615832	87.11874025	94.15519932	103.0075188	97.33295503	94.83614697	89.38856575	99.37579799	74.18073486	85.52986239	94.60916442	85.30287984	101.4186409	95.51709462
	86.39503736	101.5085295	96.99703933	105.5688707	102.1852531	73.98843931	77.37205696	96.09474129	98.3504864	99.02720993	108.2757648	108.5013393	97.22261384	100.6062315	88.42520795	74.43958833	83.68814324	90.00422952	85.49273932	94.96686874	88.42520795	95.19244325	101.9596786	86.16946285	83.23699422	79.62780206	90.22980403	89.10193148	95.41801776	84.59044128	87.97405893
	74.51039019	92.45029152	94.12468231	103.6926297	110.1509942	72.11840335	72.59680072	95.79907311	101.7790402	95.79907311	102.0182389	110.6293915	100.8222455	103.453431	96.99506653	72.35760203	93.16788758	95.32067574	80.4903573	93.88548363	96.03827179	99.86545074	98.43025863	93.64628495	88.1447152	86.94872178	96.27747047	91.2542981	92.45029152	98.19105995	94.60307968
AMP1CPR1ERY1DOX3TMP3	AMP	CPR	ERY	DOX	TMP	AMP+CPR	AMP+ERY	AMP+DOX	AMP+TMP	CPR+ERY	CPR+DOX	CPR+TMP	ERY+DOX	ERY+TMP	DOX+TMP	AMP+CPR+ERY	AMP+CPR+DOX	AMP+CPR+TMP	AMP+ERY+DOX	AMP+ERY+TMP	AMP+DOX+TMP	CPR+ERY+DOX	CPR+ERY+TMP	CPR+DOX+TMP	ERY+DOX+TMP	AMP+CPR+ERY+DOX	AMP+CPR+ERY+TMP	AMP+CPR+DOX+TMP	AMP+ERY+DOX+TMP	CPR+ERY+DOX+TMP	AMP+CPR+ERY+DOX+TMP
	86.21081004	91.65839126	90.29649596	104.1424316	98.69485033	81.44417648	81.21719393	90.52347851	94.15519932	100.9646758	102.0995886	101.4186409	90.75046106	92.11235636	97.55993758	80.08228117	87.11874025	90.97744361	87.11874025	93.70123422	96.65200738	97.33295503	103.4614839	98.69485033	88.93460065	74.18073486	78.26642077	65.10143283	84.62193219	99.60278054	93.24726912
	86.39503736	101.5085295	96.99703933	105.5688707	98.80163542	73.98843931	77.37205696	96.09474129	90.90652756	99.02720993	108.2757648	105.5688707	97.22261384	99.02720993	94.29014521	74.43958833	83.68814324	85.94388834	85.49273932	90.22980403	92.93669815	95.19244325	103.0875511	94.29014521	82.7858452	79.62780206	80.98124912	91.58325109	98.3504864	92.25997462	92.25997462
	74.51039019	92.45029152	94.12468231	103.6926297	109.1941994	72.11840335	72.59680072	95.79907311	79.77276125	95.79907311	102.0182389	109.6725968	100.8222455	105.6062192	97.71266258	72.35760203	93.16788758	95.55987442	80.4903573	86.94872178	101.0614442	99.86545074	101.7790402	96.03827179	88.86231126	86.94872178	89.10150994	87.42711915	92.21109284	105.1278218	91.2542981
AMP1CPR1ERY2DOX1TMP1	AMP	CPR	ERY	DOX	TMP	AMP+CPR	AMP+ERY	AMP+DOX	AMP+TMP	CPR+ERY	CPR+DOX	CPR+TMP	ERY+DOX	ERY+TMP	DOX+TMP	AMP+CPR+ERY	AMP+CPR+DOX	AMP+CPR+TMP	AMP+ERY+DOX	AMP+ERY+TMP	AMP+DOX+TMP	CPR+ERY+DOX	CPR+ERY+TMP	CPR+DOX+TMP	ERY+DOX+TMP	AMP+CPR+ERY+DOX	AMP+CPR+ERY+TMP	AMP+CPR+DOX+TMP	AMP+ERY+DOX+TMP	CPR+ERY+DOX+TMP	AMP+CPR+ERY+DOX+TMP
	86.21081004	91.65839126	95.55789791	98.24088523	91.88537381	81.44417648	86.8917577	88.253653	87.7996879	99.60278054	101.4186409	88.93460065	96.52160819	97.00346333	92.79330401	75.76961271	80.76322883	89.84253086	86.21081004	85.07589729	99.14881543	100.5107107	93.92821677	95.51709462	92.42583948	68.96013619	92.11235636	91.88537381	95.97105972	92.33933891	89.1615832
	86.39503736	101.5085295	99.10541226	105.1177217	100.1550825	73.98843931	84.36486677	83.46256873	96.99703933	97.44818835	103.3131256	103.5387001	97.1969584	99.82108245	99.25278444	69.92809812	77.59763147	88.87635697	74.43958833	95.86916678	97.22261384	99.70393346	99.92950797	101.0573805	92.6643805	77.37205696	88.87635697	94.51571972	94.96686874	99.92950797	89.32750599
	74.51039019	92.45029152	96.19192688	98.66945732	106.8022126	72.11840335	73.07519809	85.99192704	96.03827179	92.92868889	100.8222455	106.5630139	98.385377	107.4028941	107.28061	78.57676783	90.77590073	96.51666916	82.40394678	96.27747047	95.79907311	101.5398415	97.95186126	106.0846165	102.0411272	84.55673494	101.5398415	103.453431	91.2542981	101.7790402	111.3469876
AMP1CPR1ERY2DOX1TMP2	AMP	CPR	ERY	DOX	TMP	AMP+CPR	AMP+ERY	AMP+DOX	AMP+TMP	CPR+ERY	CPR+DOX	CPR+TMP	ERY+DOX	ERY+TMP	DOX+TMP	AMP+CPR+ERY	AMP+CPR+DOX	AMP+CPR+TMP	AMP+ERY+DOX	AMP+ERY+TMP	AMP+DOX+TMP	CPR+ERY+DOX	CPR+ERY+TMP	CPR+DOX+TMP	ERY+DOX+TMP	AMP+CPR+ERY+DOX	AMP+CPR+ERY+TMP	AMP+CPR+DOX+TMP	AMP+ERY+DOX+TMP	CPR+ERY+DOX+TMP	AMP+CPR+ERY+DOX+TMP
	86.21081004	91.65839126	95.55789791	98.24088523	95.97105972	81.44417648	86.8917577	88.253653	102.7805362	99.60278054	101.4186409	96.87898993	96.52160819	96.03975305	95.51709462	75.76961271	80.76322883	89.1615832	86.21081004	99.14881543	101.872606	100.5107107	100.9646758	92.11235636	97.00346333	68.96013619	91.20442616	89.1615832	97.78692013	92.79330401	86.66477515
	86.39503736	101.5085295	99.10541226	105.1177217	102.1852531	73.98843931	84.36486677	83.46256873	98.3504864	97.44818835	103.3131256	108.5013393	97.1969584	98.62829879	102.8619766	69.92809812	77.59763147	90.00422952	74.43958833	99.92950797	98.80163542	99.70393346	107.1478923	100.6062315	93.14149396	77.37205696	87.52290991	89.32750599	94.74129423	97.67376286	81.43239814
	74.51039019	92.45029152	96.19192688	98.66945732	110.1509942	72.11840335	73.07519809	85.99192704	101.7790402	92.92868889	100.8222455	110.6293915	98.385377	103.5034273	106.3238152	78.57676783	90.77590073	95.32067574	82.40394678	98.43025863	100.1046494	101.5398415	103.9318284	110.1509942	104.478294	84.55673494	94.60307968	94.363881	94.363881	101.0614442	103.6926297
AMP1CPR1ERY2DOX1TMP3	AMP	CPR	ERY	DOX	TMP	AMP+CPR	AMP+ERY	AMP+DOX	AMP+TMP	CPR+ERY	CPR+DOX	CPR+TMP	ERY+DOX	ERY+TMP	DOX+TMP	AMP+CPR+ERY	AMP+CPR+DOX	AMP+CPR+TMP	AMP+ERY+DOX	AMP+ERY+TMP	AMP+DOX+TMP	CPR+ERY+DOX	CPR+ERY+TMP	CPR+DOX+TMP	ERY+DOX+TMP	AMP+CPR+ERY+DOX	AMP+CPR+ERY+TMP	AMP+CPR+DOX+TMP	AMP+ERY+DOX+TMP	CPR+ERY+DOX+TMP	AMP+CPR+ERY+DOX+TMP
	86.21081004	91.65839126	95.55789791	98.24088523	98.69485033	81.44417648	86.8917577	88.253653	94.15519932	99.60278054	101.4186409	101.4186409	96.52160819	98.44902876	100.9646758	75.76961271	80.76322883	90.97744361	86.21081004	96.42502483	100.9646758	100.5107107	102.5535537	105.7313094	96.76253576	68.96013619	85.52986239	85.75684494	99.14881543	104.5963967	81.21719393
	86.39503736	101.5085295	99.10541226	105.1177217	98.80163542	73.98843931	84.36486677	83.46256873	90.90652756	97.44818835	103.3131256	105.5688707	97.1969584	99.34396899	105.3432962	69.92809812	77.59763147	85.94388834	74.43958833	91.35767658	98.3504864	99.70393346	101.5085295	104.8921472	70.24004771	77.37205696	83.01141971	83.23699422	93.16227266	104.2154237	84.59044128
	74.51039019	92.45029152	96.19192688	98.66945732	109.1941994	72.11840335	73.07519809	85.99192704	79.77276125	92.92868889	100.8222455	109.6725968	98.385377	97.1667936	109.4333981	78.57676783	90.77590073	95.55987442	82.40394678	88.1447152	99.62625206	101.5398415	102.9750336	112.7821797	101.3099772	84.55673494	93.64628495	96.51666916	91.49349679	109.6725968	91.49349679
AMP1CPR1ERY2DOX2TMP1	AMP	CPR	ERY	DOX	TMP	AMP+CPR	AMP+ERY	AMP+DOX	AMP+TMP	CPR+ERY	CPR+DOX	CPR+TMP	ERY+DOX	ERY+TMP	DOX+TMP	AMP+CPR+ERY	AMP+CPR+DOX	AMP+CPR+TMP	AMP+ERY+DOX	AMP+ERY+TMP	AMP+DOX+TMP	CPR+ERY+DOX	CPR+ERY+TMP	CPR+DOX+TMP	ERY+DOX+TMP	AMP+CPR+ERY+DOX	AMP+CPR+ERY+TMP	AMP+CPR+DOX+TMP	AMP+ERY+DOX+TMP	CPR+ERY+DOX+TMP	AMP+CPR+ERY+DOX+TMP
	86.21081004	91.65839126	95.55789791	99.82976309	91.88537381	81.44417648	86.8917577	90.52347851	87.7996879	99.60278054	98.46786778	88.93460065	96.52160819	97.00346333	97.33295503	75.76961271	80.53624628	89.84253086	87.3457228	85.07589729	98.92183288	100.0567456	93.92821677	98.46786778	99.65366662	71.45694425	92.11235636	86.66477515	93.02028657	97.33295503	79.40133352
	86.39503736	101.5085295	99.10541226	103.9898491	100.1550825	73.98843931	84.36486677	93.83899619	96.99703933	97.44818835	103.9898491	103.5387001	96.24273147	99.82108245	98.12491188	69.92809812	82.56027069	88.87635697	83.23699422	95.86916678	99.47835895	98.80163542	99.92950797	94.96686874	103.6379902	76.92090794	88.87635697	93.83899619	94.29014521	92.93669815	86.16946285
	74.51039019	92.45029152	96.19192688	105.1278218	106.8022126	72.11840335	73.07519809	91.49349679	96.03827179	92.92868889	100.3438481	106.5630139	99.6039604	107.4028941	99.86545074	78.57676783	92.45029152	96.51666916	87.42711915	96.27747047	103.2142323	102.2574376	97.95186126	107.28061	106.4280274	91.2542981	101.5398415	101.0614442	94.84227837	105.3670205	84.31753625
AMP1CPR1ERY2DOX2TMP2	AMP	CPR	ERY	DOX	TMP	AMP+CPR	AMP+ERY	AMP+DOX	AMP+TMP	CPR+ERY	CPR+DOX	CPR+TMP	ERY+DOX	ERY+TMP	DOX+TMP	AMP+CPR+ERY	AMP+CPR+DOX	AMP+CPR+TMP	AMP+ERY+DOX	AMP+ERY+TMP	AMP+DOX+TMP	CPR+ERY+DOX	CPR+ERY+TMP	CPR+DOX+TMP	ERY+DOX+TMP	AMP+CPR+ERY+DOX	AMP+CPR+ERY+TMP	AMP+CPR+DOX+TMP	AMP+ERY+DOX+TMP	CPR+ERY+DOX+TMP	AMP+CPR+ERY+DOX+TMP
	86.21081004	91.65839126	95.55789791	99.82976309	95.97105972	81.44417648	86.8917577	90.52347851	102.7805362	99.60278054	98.46786778	96.87898993	96.52160819	96.03975305	100.9646758	75.76961271	80.53624628	89.1615832	87.3457228	99.14881543	100.9646758	100.0567456	100.9646758	103.915449	100.1355218	71.45694425	91.20442616	96.87898993	98.46786778	104.1424316	87.7996879
	86.39503736	101.5085295	99.10541226	103.9898491	102.1852531	73.98843931	84.36486677	93.83899619	98.3504864	97.44818835	103.9898491	108.5013393	96.24273147	98.62829879	98.80163542	69.92809812	82.56027069	90.00422952	83.23699422	99.92950797	102.1852531	98.80163542	107.1478923	100.1550825	97.1969584	76.92090794	87.52290991	97.44818835	99.25278444	95.19244325	86.84618638
	74.51039019	92.45029152	96.19192688	105.1278218	110.1509942	72.11840335	73.07519809	91.49349679	101.7790402	92.92868889	100.3438481	110.6293915	99.6039604	103.5034273	99.14785469	78.57676783	92.45029152	95.32067574	87.42711915	98.43025863	104.8886231	102.2574376	103.9318284	108.2374047	103.2597106	91.2542981	94.60307968	102.2574376	101.0614442	108.7158021	81.92554941
AMP1CPR1ERY2DOX2TMP3	AMP	CPR	ERY	DOX	TMP	AMP+CPR	AMP+ERY	AMP+DOX	AMP+TMP	CPR+ERY	CPR+DOX	CPR+TMP	ERY+DOX	ERY+TMP	DOX+TMP	AMP+CPR+ERY	AMP+CPR+DOX	AMP+CPR+TMP	AMP+ERY+DOX	AMP+ERY+TMP	AMP+DOX+TMP	CPR+ERY+DOX	CPR+ERY+TMP	CPR+DOX+TMP	ERY+DOX+TMP	AMP+CPR+ERY+DOX	AMP+CPR+ERY+TMP	AMP+CPR+DOX+TMP	AMP+ERY+DOX+TMP	CPR+ERY+DOX+TMP	AMP+CPR+ERY+DOX+TMP
	86.21081004	91.65839126	95.55789791	99.82976309	98.69485033	81.44417648	86.8917577	90.52347851	94.15519932	99.60278054	98.46786778	101.4186409	96.52160819	98.44902876	100.7376933	75.76961271	80.53624628	90.97744361	87.3457228	96.42502483	97.55993758	100.0567456	102.5535537	102.5535537	101.099232	71.45694425	85.52986239	87.7996879	76.90452546	90.06951341	84.62193219
	86.39503736	101.5085295	99.10541226	103.9898491	98.80163542	73.98843931	84.36486677	93.83899619	90.90652756	97.44818835	103.9898491	105.5688707	96.24273147	99.34396899	98.80163542	69.92809812	82.56027069	85.94388834	83.23699422	91.35767658	93.16227266	98.80163542	101.5085295	95.41801776	92.42582377	76.92090794	83.01141971	84.81601579	94.29014521	88.42520795	78.274355
	74.51039019	92.45029152	96.19192688	105.1278218	109.1941994	72.11840335	73.07519809	91.49349679	79.77276125	92.92868889	100.3438481	109.6725968	99.6039604	97.1667936	98.19105995	78.57676783	92.45029152	95.55987442	87.42711915	88.1447152	98.19105995	102.2574376	102.9750336	103.6926297	99.6039604	91.2542981	93.64628495	78.33756914	93.40708626	104.4102257	78.81596651
AMP1CPR1ERY2DOX3TMP1	AMP	CPR	ERY	DOX	TMP	AMP+CPR	AMP+ERY	AMP+DOX	AMP+TMP	CPR+ERY	CPR+DOX	CPR+TMP	ERY+DOX	ERY+TMP	DOX+TMP	AMP+CPR+ERY	AMP+CPR+DOX	AMP+CPR+TMP	AMP+ERY+DOX	AMP+ERY+TMP	AMP+DOX+TMP	CPR+ERY+DOX	CPR+ERY+TMP	CPR+DOX+TMP	ERY+DOX+TMP	AMP+CPR+ERY+DOX	AMP+CPR+ERY+TMP	AMP+CPR+DOX+TMP	AMP+ERY+DOX+TMP	CPR+ERY+DOX+TMP	AMP+CPR+ERY+DOX+TMP
	86.21081004	91.65839126	95.55789791	104.1424316	91.88537381	81.44417648	86.8917577	90.52347851	87.7996879	99.60278054	102.0995886	88.93460065	94.83511519	97.00346333	104.1424316	75.76961271	87.11874025	89.84253086	86.66477515	85.07589729	102.3265711	101.872606	93.92821677	101.1916584	103.7494353	79.17435097	92.11235636	100.0567456	94.38218187	103.2345013	100.0567456
	86.39503736	101.5085295	99.10541226	105.5688707	100.1550825	73.98843931	84.36486677	96.09474129	96.99703933	97.44818835	108.2757648	103.5387001	98.62829879	99.82108245	92.25997462	69.92809812	83.68814324	88.87635697	86.62061187	95.86916678	92.03440011	105.5688707	99.92950797	91.58325109	93.85716416	79.85337657	88.87635697	96.54589031	103.3131256	86.16946285	95.64359227
	74.51039019	92.45029152	96.19192688	103.6926297	106.8022126	72.11840335	73.07519809	95.79907311	96.03827179	92.92868889	102.0182389	106.5630139	101.7974105	107.4028941	93.16788758	78.57676783	93.16788758	96.51666916	87.66631784	96.27747047	99.86545074	104.1710271	97.95186126	102.0182389	99.6039604	86.47032441	101.5398415	92.68949021	98.66945732	104.8886231	98.19105995
AMP1CPR1ERY2DOX3TMP2	AMP	CPR	ERY	DOX	TMP	AMP+CPR	AMP+ERY	AMP+DOX	AMP+TMP	CPR+ERY	CPR+DOX	CPR+TMP	ERY+DOX	ERY+TMP	DOX+TMP	AMP+CPR+ERY	AMP+CPR+DOX	AMP+CPR+TMP	AMP+ERY+DOX	AMP+ERY+TMP	AMP+DOX+TMP	CPR+ERY+DOX	CPR+ERY+TMP	CPR+DOX+TMP	ERY+DOX+TMP	AMP+CPR+ERY+DOX	AMP+CPR+ERY+TMP	AMP+CPR+DOX+TMP	AMP+ERY+DOX+TMP	CPR+ERY+DOX+TMP	AMP+CPR+ERY+DOX+TMP
	86.21081004	91.65839126	95.55789791	104.1424316	95.97105972	81.44417648	86.8917577	90.52347851	102.7805362	99.60278054	102.0995886	96.87898993	94.83511519	96.03975305	99.37579799	75.76961271	87.11874025	89.1615832	86.66477515	99.14881543	103.0075188	101.872606	100.9646758	89.38856575	104.2312905	79.17435097	91.20442616	94.60916442	94.38218187	100.9646758	92.79330401
	86.39503736	101.5085295	99.10541226	105.5688707	102.1852531	73.98843931	84.36486677	96.09474129	98.3504864	97.44818835	108.2757648	108.5013393	98.62829879	98.62829879	88.42520795	69.92809812	83.68814324	90.00422952	86.62061187	99.92950797	88.42520795	105.5688707	107.1478923	86.16946285	97.1969584	79.85337657	87.52290991	89.10193148	98.3504864	84.59044128	86.62061187
	74.51039019	92.45029152	96.19192688	103.6926297	110.1509942	72.11840335	73.07519809	95.79907311	101.7790402	92.92868889	102.0182389	110.6293915	101.7974105	103.5034273	96.99506653	78.57676783	93.16788758	95.32067574	87.66631784	98.43025863	96.03827179	104.1710271	103.9318284	93.64628495	88.8804265	86.47032441	94.60307968	91.2542981	89.81910599	100.5830468	92.92868889
AMP1CPR1ERY2DOX3TMP3	AMP	CPR	ERY	DOX	TMP	AMP+CPR	AMP+ERY	AMP+DOX	AMP+TMP	CPR+ERY	CPR+DOX	CPR+TMP	ERY+DOX	ERY+TMP	DOX+TMP	AMP+CPR+ERY	AMP+CPR+DOX	AMP+CPR+TMP	AMP+ERY+DOX	AMP+ERY+TMP	AMP+DOX+TMP	CPR+ERY+DOX	CPR+ERY+TMP	CPR+DOX+TMP	ERY+DOX+TMP	AMP+CPR+ERY+DOX	AMP+CPR+ERY+TMP	AMP+CPR+DOX+TMP	AMP+ERY+DOX+TMP	CPR+ERY+DOX+TMP	AMP+CPR+ERY+DOX+TMP
	86.21081004	91.65839126	95.55789791	104.1424316	98.69485033	81.44417648	86.8917577	90.52347851	94.15519932	99.60278054	102.0995886	101.4186409	94.83511519	98.44902876	97.55993758	75.76961271	87.11874025	90.97744361	86.66477515	96.42502483	96.65200738	101.872606	102.5535537	98.69485033	103.9903629	79.17435097	85.52986239	65.10143283	93.24726912	100.9646758	94.60916442
	86.39503736	101.5085295	99.10541226	105.5688707	98.80163542	73.98843931	84.36486677	96.09474129	90.90652756	97.44818835	108.2757648	105.5688707	98.62829879	99.34396899	94.29014521	69.92809812	83.68814324	85.94388834	86.62061187	91.35767658	92.93669815	105.5688707	101.5085295	94.29014521	93.85716416	79.85337657	83.01141971	91.58325109	94.74129423	91.13210207	90.00422952
	74.51039019	92.45029152	96.19192688	103.6926297	109.1941994	72.11840335	73.07519809	95.79907311	79.77276125	92.92868889	102.0182389	109.6725968	101.7974105	97.1667936	97.71266258	78.57676783	93.16788758	95.55987442	87.66631784	88.1447152	101.0614442	104.1710271	102.9750336	96.03827179	92.04874334	86.47032441	93.64628495	87.42711915	91.73269547	100.5830468	85.27433099
AMP1CPR1ERY3DOX1TMP1	AMP	CPR	ERY	DOX	TMP	AMP+CPR	AMP+ERY	AMP+DOX	AMP+TMP	CPR+ERY	CPR+DOX	CPR+TMP	ERY+DOX	ERY+TMP	DOX+TMP	AMP+CPR+ERY	AMP+CPR+DOX	AMP+CPR+TMP	AMP+ERY+DOX	AMP+ERY+TMP	AMP+DOX+TMP	CPR+ERY+DOX	CPR+ERY+TMP	CPR+DOX+TMP	ERY+DOX+TMP	AMP+CPR+ERY+DOX	AMP+CPR+ERY+TMP	AMP+CPR+DOX+TMP	AMP+ERY+DOX+TMP	CPR+ERY+DOX+TMP	AMP+CPR+ERY+DOX+TMP
	86.21081004	91.65839126	95.82539683	98.24088523	91.88537381	81.44417648	89.6155483	88.253653	87.7996879	98.69485033	101.4186409	88.93460065	89.98412698	92.26984127	92.79330401	81.21719393	80.76322883	89.84253086	91.43140871	90.52347851	99.14881543	92.33933891	97.78692013	95.51709462	94.04761905	77.58547312	88.253653	91.88537381	97.78692013	90.06951341	88.02667045
	86.39503736	101.5085295	97.32768454	105.1177217	100.1550825	73.98843931	90.22980403	83.46256873	96.99703933	101.9596786	103.3131256	103.5387001	89.24555972	104.8883819	99.25278444	71.95826872	77.59763147	88.87635697	84.13929226	98.80163542	97.22261384	100.6062315	106.4711688	101.0573805	105.4098094	76.69533343	83.91371775	94.51571972	96.77146482	96.77146482	88.19963344
	74.51039019	92.45029152	99.21451583	98.66945732	106.8022126	72.11840335	81.68635073	85.99192704	96.03827179	98.19105995	100.8222455	106.5630139	96.45616266	113.282117	107.28061	83.36074152	90.77590073	96.51666916	81.44715204	102.4966363	95.79907311	97.95186126	104.6494244	106.0846165	115.4887996	98.19105995	94.12468231	103.453431	91.97189415	98.66945732	100.5830468
AMP1CPR1ERY3DOX1TMP2	AMP	CPR	ERY	DOX	TMP	AMP+CPR	AMP+ERY	AMP+DOX	AMP+TMP	CPR+ERY	CPR+DOX	CPR+TMP	ERY+DOX	ERY+TMP	DOX+TMP	AMP+CPR+ERY	AMP+CPR+DOX	AMP+CPR+TMP	AMP+ERY+DOX	AMP+ERY+TMP	AMP+DOX+TMP	CPR+ERY+DOX	CPR+ERY+TMP	CPR+DOX+TMP	ERY+DOX+TMP	AMP+CPR+ERY+DOX	AMP+CPR+ERY+TMP	AMP+CPR+DOX+TMP	AMP+ERY+DOX+TMP	CPR+ERY+DOX+TMP	AMP+CPR+ERY+DOX+TMP
	86.21081004	91.65839126	95.82539683	98.24088523	95.97105972	81.44417648	89.6155483	88.253653	102.7805362	98.69485033	101.4186409	96.87898993	89.98412698	89.73015873	95.51709462	81.21719393	80.76322883	89.1615832	91.43140871	98.69485033	101.872606	92.33933891	98.24088523	92.11235636	96.58730159	77.58547312	90.29649596	89.1615832	103.6884664	99.37579799	89.6155483
	86.39503736	101.5085295	97.32768454	105.1177217	102.1852531	73.98843931	90.22980403	83.46256873	98.3504864	101.9596786	103.3131256	108.5013393	89.24555972	101.7598175	102.8619766	71.95826872	77.59763147	90.00422952	84.13929226	99.70393346	98.80163542	100.6062315	103.5387001	100.6062315	106.1919505	76.69533343	91.58325109	89.32750599	99.70393346	101.734104	90.00422952
	74.51039019	92.45029152	99.21451583	98.66945732	110.1509942	72.11840335	81.68635073	85.99192704	101.7790402	98.19105995	100.8222455	110.6293915	96.45616266	110.2479285	106.3238152	83.36074152	90.77590073	95.32067574	81.44715204	100.1046494	100.1046494	97.95186126	108.9550007	110.1509942	115.2129643	98.19105995	101.5398415	94.363881	98.908656	109.9117955	96.75586784
AMP1CPR1ERY3DOX1TMP3	AMP	CPR	ERY	DOX	TMP	AMP+CPR	AMP+ERY	AMP+DOX	AMP+TMP	CPR+ERY	CPR+DOX	CPR+TMP	ERY+DOX	ERY+TMP	DOX+TMP	AMP+CPR+ERY	AMP+CPR+DOX	AMP+CPR+TMP	AMP+ERY+DOX	AMP+ERY+TMP	AMP+DOX+TMP	CPR+ERY+DOX	CPR+ERY+TMP	CPR+DOX+TMP	ERY+DOX+TMP	AMP+CPR+ERY+DOX	AMP+CPR+ERY+TMP	AMP+CPR+DOX+TMP	AMP+ERY+DOX+TMP	CPR+ERY+DOX+TMP	AMP+CPR+ERY+DOX+TMP
	86.21081004	91.65839126	95.82539683	98.24088523	98.69485033	81.44417648	89.6155483	88.253653	94.15519932	98.69485033	101.4186409	101.4186409	89.98412698	95.06349206	100.9646758	81.21719393	80.76322883	90.97744361	91.43140871	97.55993758	100.9646758	92.33933891	109.1360477	105.7313094	106.7460317	77.58547312	86.8917577	85.75684494	98.01390268	103.915449	86.8917577
	86.39503736	101.5085295	97.32768454	105.1177217	98.80163542	73.98843931	90.22980403	83.46256873	90.90652756	101.9596786	103.3131256	105.5688707	89.24555972	102.2812449	105.3432962	71.95826872	77.59763147	85.94388834	84.13929226	93.38784717	98.3504864	100.6062315	105.3432962	104.8921472	109.5812286	76.69533343	82.10912167	83.23699422	93.38784717	102.6364021	84.36486677
	74.51039019	92.45029152	99.21451583	98.66945732	109.1941994	72.11840335	81.68635073	85.99192704	79.77276125	98.19105995	100.8222455	109.6725968	96.45616266	109.6962579	109.4333981	83.36074152	90.77590073	95.55987442	81.44715204	94.12468231	99.62625206	97.95186126	109.9117955	112.7821797	114.3854583	98.19105995	95.55987442	96.51666916	94.84227837	109.4333981	94.60307968
AMP1CPR1ERY3DOX2TMP1	AMP	CPR	ERY	DOX	TMP	AMP+CPR	AMP+ERY	AMP+DOX	AMP+TMP	CPR+ERY	CPR+DOX	CPR+TMP	ERY+DOX	ERY+TMP	DOX+TMP	AMP+CPR+ERY	AMP+CPR+DOX	AMP+CPR+TMP	AMP+ERY+DOX	AMP+ERY+TMP	AMP+DOX+TMP	CPR+ERY+DOX	CPR+ERY+TMP	CPR+DOX+TMP	ERY+DOX+TMP	AMP+CPR+ERY+DOX	AMP+CPR+ERY+TMP	AMP+CPR+DOX+TMP	AMP+ERY+DOX+TMP	CPR+ERY+DOX+TMP	AMP+CPR+ERY+DOX+TMP
	86.21081004	91.65839126	95.82539683	99.82976309	91.88537381	81.44417648	89.6155483	90.52347851	87.7996879	98.69485033	98.46786778	88.93460065	86.17460317	92.26984127	97.33295503	81.21719393	80.53624628	89.84253086	97.10597248	90.52347851	98.92183288	100.9646758	97.78692013	98.46786778	106.2380952	79.85529862	88.253653	86.66477515	99.82976309	101.1916584	88.93460065
	86.39503736	101.5085295	97.32768454	103.9898491	100.1550825	73.98843931	90.22980403	93.83899619	96.99703933	101.9596786	103.9898491	103.5387001	102.8026723	104.8883819	98.12491188	71.95826872	82.56027069	88.87635697	93.16227266	98.80163542	99.47835895	108.2757648	106.4711688	94.96686874	110.6240834	73.53729029	83.91371775	93.83899619	100.1550825	95.19244325	90.90652756
	74.51039019	92.45029152	99.21451583	105.1278218	106.8022126	72.11840335	81.68635073	91.49349679	96.03827179	98.19105995	100.3438481	106.5630139	99.21451583	113.282117	99.86545074	83.36074152	92.45029152	96.51666916	81.68635073	102.4966363	103.2142323	104.4102257	104.6494244	107.28061	112.1787758	84.79593362	94.12468231	101.0614442	102.735835	110.6293915	90.05830468
AMP1CPR1ERY3DOX2TMP2	AMP	CPR	ERY	DOX	TMP	AMP+CPR	AMP+ERY	AMP+DOX	AMP+TMP	CPR+ERY	CPR+DOX	CPR+TMP	ERY+DOX	ERY+TMP	DOX+TMP	AMP+CPR+ERY	AMP+CPR+DOX	AMP+CPR+TMP	AMP+ERY+DOX	AMP+ERY+TMP	AMP+DOX+TMP	CPR+ERY+DOX	CPR+ERY+TMP	CPR+DOX+TMP	ERY+DOX+TMP	AMP+CPR+ERY+DOX	AMP+CPR+ERY+TMP	AMP+CPR+DOX+TMP	AMP+ERY+DOX+TMP	CPR+ERY+DOX+TMP	AMP+CPR+ERY+DOX+TMP
	86.21081004	91.65839126	95.82539683	99.82976309	95.97105972	81.44417648	89.6155483	90.52347851	102.7805362	98.69485033	98.46786778	96.87898993	86.17460317	89.73015873	100.9646758	81.21719393	80.53624628	89.1615832	97.10597248	98.69485033	100.9646758	100.9646758	98.24088523	103.915449	108.5238095	79.85529862	90.29649596	96.87898993	100.2837282	100.5107107	85.07589729
	86.39503736	101.5085295	97.32768454	103.9898491	102.1852531	73.98843931	90.22980403	93.83899619	98.3504864	101.9596786	103.9898491	108.5013393	102.8026723	101.7598175	98.80163542	71.95826872	82.56027069	90.00422952	93.16227266	99.70393346	102.1852531	108.2757648	103.5387001	100.1550825	105.9312368	73.53729029	91.58325109	97.44818835	97.89933737	92.71112364	87.2973354
	74.51039019	92.45029152	99.21451583	105.1278218	110.1509942	72.11840335	81.68635073	91.49349679	101.7790402	98.19105995	100.3438481	110.6293915	99.21451583	110.2479285	99.14785469	83.36074152	92.45029152	95.32067574	81.68635073	100.1046494	104.8886231	104.4102257	108.9550007	108.2374047	107.7654107	84.79593362	101.5398415	102.2574376	101.0614442	107.998206	83.12154283
AMP1CPR1ERY3DOX2TMP3	AMP	CPR	ERY	DOX	TMP	AMP+CPR	AMP+ERY	AMP+DOX	AMP+TMP	CPR+ERY	CPR+DOX	CPR+TMP	ERY+DOX	ERY+TMP	DOX+TMP	AMP+CPR+ERY	AMP+CPR+DOX	AMP+CPR+TMP	AMP+ERY+DOX	AMP+ERY+TMP	AMP+DOX+TMP	CPR+ERY+DOX	CPR+ERY+TMP	CPR+DOX+TMP	ERY+DOX+TMP	AMP+CPR+ERY+DOX	AMP+CPR+ERY+TMP	AMP+CPR+DOX+TMP	AMP+ERY+DOX+TMP	CPR+ERY+DOX+TMP	AMP+CPR+ERY+DOX+TMP
	86.21081004	91.65839126	95.82539683	99.82976309	98.69485033	81.44417648	89.6155483	90.52347851	94.15519932	98.69485033	98.46786778	101.4186409	86.17460317	95.06349206	100.7376933	81.21719393	80.53624628	90.97744361	97.10597248	97.55993758	97.55993758	100.9646758	109.1360477	102.5535537	105.984127	79.85529862	86.8917577	87.7996879	97.78692013	105.5043269	88.48063555
	86.39503736	101.5085295	97.32768454	103.9898491	98.80163542	73.98843931	90.22980403	93.83899619	90.90652756	101.9596786	103.9898491	105.5688707	102.8026723	102.2812449	98.80163542	71.95826872	82.56027069	85.94388834	93.16227266	93.38784717	93.16227266	108.2757648	105.3432962	95.41801776	92.89555157	73.53729029	82.10912167	84.81601579	95.19244325	90.90652756	83.91371775
	74.51039019	92.45029152	99.21451583	105.1278218	109.1941994	72.11840335	81.68635073	91.49349679	79.77276125	98.19105995	100.3438481	109.6725968	99.21451583	109.6962579	98.19105995	83.36074152	92.45029152	95.55987442	81.68635073	94.12468231	98.19105995	104.4102257	109.9117955	103.6926297	101.4211984	84.79593362	95.55987442	78.33756914	99.62625206	107.998206	82.16474809
AMP1CPR1ERY3DOX3TMP1	AMP	CPR	ERY	DOX	TMP	AMP+CPR	AMP+ERY	AMP+DOX	AMP+TMP	CPR+ERY	CPR+DOX	CPR+TMP	ERY+DOX	ERY+TMP	DOX+TMP	AMP+CPR+ERY	AMP+CPR+DOX	AMP+CPR+TMP	AMP+ERY+DOX	AMP+ERY+TMP	AMP+DOX+TMP	CPR+ERY+DOX	CPR+ERY+TMP	CPR+DOX+TMP	ERY+DOX+TMP	AMP+CPR+ERY+DOX	AMP+CPR+ERY+TMP	AMP+CPR+DOX+TMP	AMP+ERY+DOX+TMP	CPR+ERY+DOX+TMP	AMP+CPR+ERY+DOX+TMP
	86.21081004	91.65839126	95.82539683	104.1424316	91.88537381	81.44417648	89.6155483	90.52347851	87.7996879	98.69485033	102.0995886	88.93460065	90.49206349	92.26984127	104.1424316	81.21719393	87.11874025	89.84253086	94.83614697	90.52347851	102.3265711	100.9646758	97.78692013	101.1916584	108.2698413	80.08228117	88.253653	100.0567456	92.79330401	97.33295503	96.65200738
	86.39503736	101.5085295	97.32768454	105.5688707	100.1550825	73.98843931	90.22980403	96.09474129	96.99703933	101.9596786	108.2757648	103.5387001	102.0205312	104.8883819	92.25997462	71.95826872	83.68814324	88.87635697	90.45537854	98.80163542	92.03440011	106.9223178	106.4711688	91.58325109	89.50627342	81.65797265	83.91371775	96.54589031	97.22261384	81.88354716	89.5530805
	74.51039019	92.45029152	99.21451583	103.6926297	106.8022126	72.11840335	81.68635073	95.79907311	96.03827179	98.19105995	102.0182389	106.5630139	103.6278809	113.282117	93.16788758	83.36074152	93.16788758	96.51666916	95.55987442	102.4966363	99.86545074	100.8222455	104.6494244	102.0182389	106.9379047	88.1447152	94.12468231	92.68949021	96.75586784	99.38705337	90.29750336
AMP1CPR1ERY3DOX3TMP2	AMP	CPR	ERY	DOX	TMP	AMP+CPR	AMP+ERY	AMP+DOX	AMP+TMP	CPR+ERY	CPR+DOX	CPR+TMP	ERY+DOX	ERY+TMP	DOX+TMP	AMP+CPR+ERY	AMP+CPR+DOX	AMP+CPR+TMP	AMP+ERY+DOX	AMP+ERY+TMP	AMP+DOX+TMP	CPR+ERY+DOX	CPR+ERY+TMP	CPR+DOX+TMP	ERY+DOX+TMP	AMP+CPR+ERY+DOX	AMP+CPR+ERY+TMP	AMP+CPR+DOX+TMP	AMP+ERY+DOX+TMP	CPR+ERY+DOX+TMP	AMP+CPR+ERY+DOX+TMP
	86.21081004	91.65839126	95.82539683	104.1424316	95.97105972	81.44417648	89.6155483	90.52347851	102.7805362	98.69485033	102.0995886	96.87898993	90.49206349	89.73015873	99.37579799	81.21719393	87.11874025	89.1615832	94.83614697	98.69485033	103.0075188	100.9646758	98.24088523	89.38856575	103.4444444	80.08228117	90.29649596	94.60916442	99.14881543	103.2345013	97.10597248
	86.39503736	101.5085295	97.32768454	105.5688707	102.1852531	73.98843931	90.22980403	96.09474129	98.3504864	101.9596786	108.2757648	108.5013393	102.0205312	101.7598175	88.42520795	71.95826872	83.68814324	90.00422952	90.45537854	99.70393346	88.42520795	106.9223178	103.5387001	86.16946285	85.33485416	81.65797265	91.58325109	89.10193148	96.77146482	88.65078246	90.45537854
	74.51039019	92.45029152	99.21451583	103.6926297	110.1509942	72.11840335	81.68635073	95.79907311	101.7790402	98.19105995	102.0182389	110.6293915	103.6278809	110.2479285	96.99506653	83.36074152	93.16788758	95.32067574	95.55987442	100.1046494	96.03827179	100.8222455	108.9550007	93.64628495	108.3170813	88.1447152	101.5398415	91.2542981	94.84227837	102.2574376	93.16788758
AMP1CPR1ERY3DOX3TMP3	AMP	CPR	ERY	DOX	TMP	AMP+CPR	AMP+ERY	AMP+DOX	AMP+TMP	CPR+ERY	CPR+DOX	CPR+TMP	ERY+DOX	ERY+TMP	DOX+TMP	AMP+CPR+ERY	AMP+CPR+DOX	AMP+CPR+TMP	AMP+ERY+DOX	AMP+ERY+TMP	AMP+DOX+TMP	CPR+ERY+DOX	CPR+ERY+TMP	CPR+DOX+TMP	ERY+DOX+TMP	AMP+CPR+ERY+DOX	AMP+CPR+ERY+TMP	AMP+CPR+DOX+TMP	AMP+ERY+DOX+TMP	CPR+ERY+DOX+TMP	AMP+CPR+ERY+DOX+TMP
	86.21081004	91.65839126	95.82539683	104.1424316	98.69485033	81.44417648	89.6155483	90.52347851	94.15519932	98.69485033	102.0995886	101.4186409	90.49206349	95.06349206	97.55993758	81.21719393	87.11874025	90.97744361	94.83614697	97.55993758	96.65200738	100.9646758	109.1360477	98.69485033	100.9047619	80.08228117	86.8917577	65.10143283	100.9646758	100.0567456	92.33933891
	86.39503736	101.5085295	97.32768454	105.5688707	98.80163542	73.98843931	90.22980403	96.09474129	90.90652756	101.9596786	108.2757648	105.5688707	102.0205312	102.2812449	94.29014521	71.95826872	83.68814324	85.94388834	90.45537854	93.38784717	92.93669815	106.9223178	105.3432962	94.29014521	94.19912009	81.65797265	82.10912167	91.58325109	92.03440011	93.38784717	93.16227266
	74.51039019	92.45029152	99.21451583	103.6926297	109.1941994	72.11840335	81.68635073	95.79907311	79.77276125	98.19105995	102.0182389	109.6725968	103.6278809	109.6962579	97.71266258	83.36074152	93.16788758	95.55987442	95.55987442	94.12468231	101.0614442	100.8222455	109.9117955	96.03827179	106.3862341	88.1447152	95.55987442	87.42711915	100.8222455	96.27747047	85.27433099
AMP1CPR2ERY1DOX1TMP1	AMP	CPR	ERY	DOX	TMP	AMP+CPR	AMP+ERY	AMP+DOX	AMP+TMP	CPR+ERY	CPR+DOX	CPR+TMP	ERY+DOX	ERY+TMP	DOX+TMP	AMP+CPR+ERY	AMP+CPR+DOX	AMP+CPR+TMP	AMP+ERY+DOX	AMP+ERY+TMP	AMP+DOX+TMP	CPR+ERY+DOX	CPR+ERY+TMP	CPR+DOX+TMP	ERY+DOX+TMP	AMP+CPR+ERY+DOX	AMP+CPR+ERY+TMP	AMP+CPR+DOX+TMP	AMP+ERY+DOX+TMP	CPR+ERY+DOX+TMP	AMP+CPR+ERY+DOX+TMP
	86.21081004	101.5810872	90.29649596	98.24088523	91.88537381	61.92367712	81.21719393	88.253653	87.7996879	96.03975305	102.5447975	100.3764493	83.71400199	90.29649596	92.79330401	82.80607178	85.52986239	90.29649596	77.13150802	93.70123422	99.14881543	97.2443909	101.5810872	102.3038699	80.99021138	80.76322883	88.93460065	88.7076181	98.24088523	93.1486222	69.86806639
	86.39503736	97.67407187	96.99703933	105.1177217	100.1550825	79.85337657	77.37205696	83.46256873	96.99703933	91.71015357	102.2066498	105.7850007	95.19244325	100.1550825	99.25278444	78.95107853	85.0415903	86.16946285	80.75567461	96.09474129	97.22261384	93.14149396	106.0235575	95.76561801	90.45537854	72.18384323	91.13210207	88.65078246	88.87635697	95.28850455	75.34188637
	74.51039019	97.65422696	94.12468231	98.66945732	106.8022126	70.20481387	72.59680072	85.99192704	96.03827179	66.458492	105.6968774	110.0837776	94.363881	102.0182389	107.28061	76.42397967	85.51352968	97.23426521	72.11840335	94.363881	95.79907311	94.24219345	104.7220107	111.5460777	96.03827179	81.68635073	99.14785469	101.0614442	82.88234415	97.41051028	101.3006428
AMP1CPR2ERY1DOX1TMP2	AMP	CPR	ERY	DOX	TMP	AMP+CPR	AMP+ERY	AMP+DOX	AMP+TMP	CPR+ERY	CPR+DOX	CPR+TMP	ERY+DOX	ERY+TMP	DOX+TMP	AMP+CPR+ERY	AMP+CPR+DOX	AMP+CPR+TMP	AMP+ERY+DOX	AMP+ERY+TMP	AMP+DOX+TMP	CPR+ERY+DOX	CPR+ERY+TMP	CPR+DOX+TMP	ERY+DOX+TMP	AMP+CPR+ERY+DOX	AMP+CPR+ERY+TMP	AMP+CPR+DOX+TMP	AMP+ERY+DOX+TMP	CPR+ERY+DOX+TMP	AMP+CPR+ERY+DOX+TMP
	86.21081004	101.5810872	90.29649596	98.24088523	95.97105972	61.92367712	81.21719393	88.253653	102.7805362	96.03975305	102.5447975	104.472218	83.71400199	88.48063555	95.51709462	82.80607178	85.52986239	88.93460065	77.13150802	94.15519932	101.872606	97.2443909	98.68995633	101.8220148	95.06312952	80.76322883	84.39494964	87.3457228	96.87898993	97.72624605	85.30287984
	86.39503736	97.67407187	96.99703933	105.1177217	102.1852531	79.85337657	77.37205696	83.46256873	98.3504864	91.71015357	102.2066498	106.7392277	95.19244325	100.6062315	102.8619766	78.95107853	85.0415903	85.49273932	80.75567461	94.96686874	98.80163542	93.14149396	100.0596392	92.90293723	99.02720993	72.18384323	85.0415903	89.32750599	90.45537854	95.52706128	80.5301001
	74.51039019	97.65422696	94.12468231	98.66945732	110.1509942	70.20481387	72.59680072	85.99192704	101.7790402	66.458492	105.6968774	105.9405941	94.363881	103.453431	106.3238152	76.42397967	85.51352968	95.55987442	72.11840335	93.88548363	100.1046494	94.24219345	103.0159939	111.302361	106.8022126	81.68635073	96.03827179	97.71266258	88.38391389	103.5034273	104.1710271
AMP1CPR2ERY1DOX1TMP3	AMP	CPR	ERY	DOX	TMP	AMP+CPR	AMP+ERY	AMP+DOX	AMP+TMP	CPR+ERY	CPR+DOX	CPR+TMP	ERY+DOX	ERY+TMP	DOX+TMP	AMP+CPR+ERY	AMP+CPR+DOX	AMP+CPR+TMP	AMP+ERY+DOX	AMP+ERY+TMP	AMP+DOX+TMP	CPR+ERY+DOX	CPR+ERY+TMP	CPR+DOX+TMP	ERY+DOX+TMP	AMP+CPR+ERY+DOX	AMP+CPR+ERY+TMP	AMP+CPR+DOX+TMP	AMP+ERY+DOX+TMP	CPR+ERY+DOX+TMP	AMP+CPR+ERY+DOX+TMP
	86.21081004	101.5810872	90.29649596	98.24088523	98.69485033	61.92367712	81.21719393	88.253653	94.15519932	96.03975305	102.5447975	103.7494353	83.71400199	92.11235636	100.9646758	82.80607178	85.52986239	86.8917577	77.13150802	93.70123422	100.9646758	97.2443909	98.9308839	101.3401596	96.65200738	80.76322883	82.35210668	90.29649596	95.97105972	99.41273905	80.08228117
	86.39503736	97.67407187	96.99703933	105.1177217	98.80163542	79.85337657	77.37205696	83.46256873	90.90652756	91.71015357	102.2066498	101.7295363	95.19244325	99.02720993	105.3432962	78.95107853	85.0415903	83.01141971	80.75567461	90.22980403	98.3504864	93.14149396	99.82108245	100.7753094	99.02720993	72.18384323	86.84618638	88.87635697	85.49273932	86.46190547	74.89073735
	74.51039019	97.65422696	94.12468231	98.66945732	109.1941994	70.20481387	72.59680072	85.99192704	79.77276125	66.458492	105.6968774	106.6717441	94.363881	105.6062192	109.4333981	76.42397967	85.51352968	86.7095231	72.11840335	86.94872178	99.62625206	94.24219345	102.7722772	107.6466108	108.7158021	81.68635073	95.55987442	95.79907311	85.75272836	108.6214775	96.99506653
AMP1CPR2ERY1DOX2TMP1	AMP	CPR	ERY	DOX	TMP	AMP+CPR	AMP+ERY	AMP+DOX	AMP+TMP	CPR+ERY	CPR+DOX	CPR+TMP	ERY+DOX	ERY+TMP	DOX+TMP	AMP+CPR+ERY	AMP+CPR+DOX	AMP+CPR+TMP	AMP+ERY+DOX	AMP+ERY+TMP	AMP+DOX+TMP	CPR+ERY+DOX	CPR+ERY+TMP	CPR+DOX+TMP	ERY+DOX+TMP	AMP+CPR+ERY+DOX	AMP+CPR+ERY+TMP	AMP+CPR+DOX+TMP	AMP+ERY+DOX+TMP	CPR+ERY+DOX+TMP	AMP+CPR+ERY+DOX+TMP
	86.21081004	101.5810872	90.29649596	99.82976309	91.88537381	61.92367712	81.21719393	90.52347851	87.7996879	96.03975305	100.6173769	100.3764493	93.47425167	90.29649596	97.33295503	82.80607178	90.52347851	90.29649596	83.48701944	93.70123422	98.92183288	97.48531848	101.5810872	102.785725	95.97105972	83.26003688	88.93460065	85.30287984	95.06312952	95.79882548	90.97744361
	86.39503736	97.67407187	96.99703933	103.9898491	100.1550825	79.85337657	77.37205696	93.83899619	96.99703933	91.71015357	102.2066498	105.7850007	96.99703933	100.1550825	98.12491188	78.95107853	86.39503736	86.16946285	82.7858452	96.09474129	99.47835895	96.95840167	106.0235575	100.7753094	96.99703933	76.0186099	91.13210207	90.90652756	78.72550402	86.93901894	87.74848442
	74.51039019	97.65422696	94.12468231	105.1278218	106.8022126	70.20481387	72.59680072	91.49349679	96.03827179	66.458492	94.24219345	110.0837776	96.27747047	102.0182389	99.86545074	76.42397967	87.90551652	97.23426521	79.77276125	94.363881	103.2142323	97.1667936	104.7220107	106.4280274	100.3438481	81.20795336	99.14785469	98.908656	95.55987442	108.8651942	105.8454179
AMP1CPR2ERY1DOX2TMP2	AMP	CPR	ERY	DOX	TMP	AMP+CPR	AMP+ERY	AMP+DOX	AMP+TMP	CPR+ERY	CPR+DOX	CPR+TMP	ERY+DOX	ERY+TMP	DOX+TMP	AMP+CPR+ERY	AMP+CPR+DOX	AMP+CPR+TMP	AMP+ERY+DOX	AMP+ERY+TMP	AMP+DOX+TMP	CPR+ERY+DOX	CPR+ERY+TMP	CPR+DOX+TMP	ERY+DOX+TMP	AMP+CPR+ERY+DOX	AMP+CPR+ERY+TMP	AMP+CPR+DOX+TMP	AMP+ERY+DOX+TMP	CPR+ERY+DOX+TMP	AMP+CPR+ERY+DOX+TMP
	86.21081004	101.5810872	90.29649596	99.82976309	95.97105972	61.92367712	81.21719393	90.52347851	102.7805362	96.03975305	100.6173769	104.472218	93.47425167	88.48063555	100.9646758	82.80607178	90.52347851	88.93460065	83.48701944	94.15519932	100.9646758	97.48531848	98.68995633	98.20810119	97.55993758	83.26003688	84.39494964	91.43140871	74.63469996	96.03975305	86.8917577
	86.39503736	97.67407187	96.99703933	103.9898491	102.1852531	79.85337657	77.37205696	93.83899619	98.3504864	91.71015357	102.2066498	106.7392277	96.99703933	100.6062315	98.80163542	78.95107853	86.39503736	85.49273932	82.7858452	94.96686874	102.1852531	96.95840167	100.0596392	100.7753094	91.8088256	76.0186099	85.0415903	92.25997462	93.16227266	86.46190547	90.45537854
	74.51039019	97.65422696	94.12468231	105.1278218	110.1509942	70.20481387	72.59680072	91.49349679	101.7790402	66.458492	94.24219345	105.9405941	96.27747047	103.453431	99.14785469	76.42397967	87.90551652	95.55987442	79.77276125	93.88548363	104.8886231	97.1667936	103.0159939	111.5460777	93.88548363	81.20795336	96.03827179	94.84227837	96.03827179	108.3777609	100.1046494
AMP1CPR2ERY1DOX2TMP3	AMP	CPR	ERY	DOX	TMP	AMP+CPR	AMP+ERY	AMP+DOX	AMP+TMP	CPR+ERY	CPR+DOX	CPR+TMP	ERY+DOX	ERY+TMP	DOX+TMP	AMP+CPR+ERY	AMP+CPR+DOX	AMP+CPR+TMP	AMP+ERY+DOX	AMP+ERY+TMP	AMP+DOX+TMP	CPR+ERY+DOX	CPR+ERY+TMP	CPR+DOX+TMP	ERY+DOX+TMP	AMP+CPR+ERY+DOX	AMP+CPR+ERY+TMP	AMP+CPR+DOX+TMP	AMP+ERY+DOX+TMP	CPR+ERY+DOX+TMP	AMP+CPR+ERY+DOX+TMP
	86.21081004	101.5810872	90.29649596	99.82976309	98.69485033	61.92367712	81.21719393	90.52347851	94.15519932	96.03975305	100.6173769	103.7494353	93.47425167	92.11235636	100.7376933	82.80607178	90.52347851	86.8917577	83.48701944	93.70123422	97.55993758	97.48531848	98.9308839	101.3401596	97.55993758	83.26003688	82.35210668	93.92821677	89.1615832	99.17181147	86.21081004
	86.39503736	97.67407187	96.99703933	103.9898491	98.80163542	79.85337657	77.37205696	93.83899619	90.90652756	91.71015357	102.2066498	101.7295363	96.99703933	99.02720993	98.80163542	78.95107853	86.39503736	83.01141971	82.7858452	90.22980403	93.16227266	96.95840167	99.82108245	96.71984494	90.45537854	76.0186099	86.84618638	87.74848442	90.45537854	90.75592664	91.58325109
	74.51039019	97.65422696	94.12468231	105.1278218	109.1941994	70.20481387	72.59680072	91.49349679	79.77276125	66.458492	94.24219345	106.6717441	96.27747047	105.6062192	98.19105995	76.42397967	87.90551652	86.7095231	79.77276125	86.94872178	98.19105995	97.1667936	102.7722772	104.9657273	91.73269547	81.20795336	95.55987442	92.45029152	90.77590073	87.41812643	90.29750336
AMP1CPR2ERY1DOX3TMP1	AMP	CPR	ERY	DOX	TMP	AMP+CPR	AMP+ERY	AMP+DOX	AMP+TMP	CPR+ERY	CPR+DOX	CPR+TMP	ERY+DOX	ERY+TMP	DOX+TMP	AMP+CPR+ERY	AMP+CPR+DOX	AMP+CPR+TMP	AMP+ERY+DOX	AMP+ERY+TMP	AMP+DOX+TMP	CPR+ERY+DOX	CPR+ERY+TMP	CPR+DOX+TMP	ERY+DOX+TMP	AMP+CPR+ERY+DOX	AMP+CPR+ERY+TMP	AMP+CPR+DOX+TMP	AMP+ERY+DOX+TMP	CPR+ERY+DOX+TMP	AMP+CPR+ERY+DOX+TMP
	86.21081004	101.5810872	90.29649596	104.1424316	91.88537381	61.92367712	81.21719393	90.52347851	87.7996879	96.03975305	98.44902876	100.3764493	90.75046106	90.29649596	104.1424316	82.80607178	90.52347851	90.29649596	87.11874025	93.70123422	102.3265711	98.68995633	101.5810872	103.0266526	99.60278054	76.45056036	88.93460065	100.2837282	87.7996879	94.11233248	92.79330401
	86.39503736	97.67407187	96.99703933	105.5688707	100.1550825	79.85337657	77.37205696	96.09474129	96.99703933	91.71015357	103.3994334	105.7850007	97.22261384	100.1550825	92.25997462	78.95107853	84.59044128	86.16946285	85.49273932	96.09474129	92.03440011	77.87386313	106.0235575	103.6379902	82.56027069	74.43958833	91.13210207	95.41801776	95.86916678	102.4452065	97.67376286
	74.51039019	97.65422696	94.12468231	103.6926297	106.8022126	70.20481387	72.59680072	95.79907311	96.03827179	66.458492	105.9405941	110.0837776	100.8222455	102.0182389	93.16788758	76.42397967	91.73269547	97.23426521	80.4903573	94.363881	99.86545074	98.14166032	104.7220107	110.0837776	85.51352968	80.4903573	99.14785469	107.998206	89.34070863	103.7471439	88.86231126
AMP1CPR2ERY1DOX3TMP2	AMP	CPR	ERY	DOX	TMP	AMP+CPR	AMP+ERY	AMP+DOX	AMP+TMP	CPR+ERY	CPR+DOX	CPR+TMP	ERY+DOX	ERY+TMP	DOX+TMP	AMP+CPR+ERY	AMP+CPR+DOX	AMP+CPR+TMP	AMP+ERY+DOX	AMP+ERY+TMP	AMP+DOX+TMP	CPR+ERY+DOX	CPR+ERY+TMP	CPR+DOX+TMP	ERY+DOX+TMP	AMP+CPR+ERY+DOX	AMP+CPR+ERY+TMP	AMP+CPR+DOX+TMP	AMP+ERY+DOX+TMP	CPR+ERY+DOX+TMP	AMP+CPR+ERY+DOX+TMP
	86.21081004	101.5810872	90.29649596	104.1424316	95.97105972	61.92367712	81.21719393	90.52347851	102.7805362	96.03975305	98.44902876	104.472218	90.75046106	88.48063555	99.37579799	82.80607178	90.52347851	88.93460065	87.11874025	94.15519932	103.0075188	98.68995633	98.68995633	81.10224364	99.37579799	76.45056036	84.39494964	99.60278054	85.30287984	97.48531848	101.6456235
	86.39503736	97.67407187	96.99703933	105.5688707	102.1852531	79.85337657	77.37205696	96.09474129	98.3504864	91.71015357	103.3994334	106.7392277	97.22261384	100.6062315	88.42520795	78.95107853	84.59044128	85.49273932	85.49273932	94.96686874	88.42520795	77.87386313	100.0596392	96.00417474	83.23699422	74.43958833	85.0415903	96.09474129	95.41801776	100.7753094	99.47835895
	74.51039019	97.65422696	94.12468231	103.6926297	110.1509942	70.20481387	72.59680072	95.79907311	101.7790402	66.458492	105.9405941	105.9405941	100.8222455	103.453431	96.99506653	76.42397967	91.73269547	95.55987442	80.4903573	93.88548363	96.03827179	98.14166032	103.0159939	102.2848439	88.1447152	80.4903573	96.03827179	100.3438481	92.45029152	97.41051028	92.21109284
AMP1CPR2ERY1DOX3TMP3	AMP	CPR	ERY	DOX	TMP	AMP+CPR	AMP+ERY	AMP+DOX	AMP+TMP	CPR+ERY	CPR+DOX	CPR+TMP	ERY+DOX	ERY+TMP	DOX+TMP	AMP+CPR+ERY	AMP+CPR+DOX	AMP+CPR+TMP	AMP+ERY+DOX	AMP+ERY+TMP	AMP+DOX+TMP	CPR+ERY+DOX	CPR+ERY+TMP	CPR+DOX+TMP	ERY+DOX+TMP	AMP+CPR+ERY+DOX	AMP+CPR+ERY+TMP	AMP+CPR+DOX+TMP	AMP+ERY+DOX+TMP	CPR+ERY+DOX+TMP	AMP+CPR+ERY+DOX+TMP
	86.21081004	101.5810872	90.29649596	104.1424316	98.69485033	61.92367712	81.21719393	90.52347851	94.15519932	96.03975305	98.44902876	103.7494353	90.75046106	92.11235636	97.55993758	82.80607178	90.52347851	86.8917577	87.11874025	93.70123422	96.65200738	98.68995633	98.9308839	106.6405662	88.93460065	76.45056036	82.35210668	101.6456235	84.62193219	98.68995633	88.253653
	86.39503736	97.67407187	96.99703933	105.5688707	98.80163542	79.85337657	77.37205696	96.09474129	90.90652756	91.71015357	103.3994334	101.7295363	97.22261384	99.02720993	94.29014521	78.95107853	84.59044128	83.01141971	85.49273932	90.22980403	92.93669815	77.87386313	99.82108245	101.7295363	82.7858452	74.43958833	86.84618638	94.96686874	98.3504864	101.2524228	93.16227266
	74.51039019	97.65422696	94.12468231	103.6926297	109.1941994	70.20481387	72.59680072	95.79907311	79.77276125	66.458492	105.9405941	106.6717441	100.8222455	105.6062192	97.71266258	76.42397967	91.73269547	86.7095231	80.4903573	86.94872178	101.0614442	98.14166032	102.7722772	96.67936024	88.86231126	80.4903573	95.55987442	88.38391389	92.21109284	81.08149276	88.62311257
AMP1CPR2ERY2DOX1TMP1	AMP	CPR	ERY	DOX	TMP	AMP+CPR	AMP+ERY	AMP+DOX	AMP+TMP	CPR+ERY	CPR+DOX	CPR+TMP	ERY+DOX	ERY+TMP	DOX+TMP	AMP+CPR+ERY	AMP+CPR+DOX	AMP+CPR+TMP	AMP+ERY+DOX	AMP+ERY+TMP	AMP+DOX+TMP	CPR+ERY+DOX	CPR+ERY+TMP	CPR+DOX+TMP	ERY+DOX+TMP	AMP+CPR+ERY+DOX	AMP+CPR+ERY+TMP	AMP+CPR+DOX+TMP	AMP+ERY+DOX+TMP	CPR+ERY+DOX+TMP	AMP+CPR+ERY+DOX+TMP
	86.21081004	101.5810872	95.55789791	98.24088523	91.88537381	61.92367712	86.8917577	88.253653	87.7996879	100.3764493	102.5447975	100.3764493	96.52160819	97.00346333	92.79330401	78.03943822	85.52986239	90.29649596	86.21081004	85.07589729	99.14881543	100.8583045	100.3764493	102.3038699	92.42583948	82.80607178	84.84891474	88.7076181	95.97105972	98.68995633	83.71400199
	86.39503736	97.67407187	99.10541226	105.1177217	100.1550825	79.85337657	84.36486677	83.46256873	96.99703933	96.24273147	102.2066498	105.7850007	97.1969584	99.82108245	99.25278444	72.63499225	85.0415903	86.16946285	74.43958833	95.86916678	97.22261384	99.58252572	104.3536604	95.76561801	92.6643805	78.72550402	86.84618638	88.65078246	94.96686874	100.2981959	78.72550402
	74.51039019	97.65422696	96.19192688	98.66945732	106.8022126	70.20481387	73.07519809	85.99192704	96.03827179	94.72962681	105.6968774	110.0837776	98.385377	107.4028941	107.28061	68.2912244	85.51352968	97.23426521	82.40394678	96.27747047	95.79907311	104.478294	70.84539223	111.5460777	102.0411272	79.0551652	95.32067574	101.0614442	91.2542981	108.6214775	105.6062192
AMP1CPR2ERY2DOX1TMP2	AMP	CPR	ERY	DOX	TMP	AMP+CPR	AMP+ERY	AMP+DOX	AMP+TMP	CPR+ERY	CPR+DOX	CPR+TMP	ERY+DOX	ERY+TMP	DOX+TMP	AMP+CPR+ERY	AMP+CPR+DOX	AMP+CPR+TMP	AMP+ERY+DOX	AMP+ERY+TMP	AMP+DOX+TMP	CPR+ERY+DOX	CPR+ERY+TMP	CPR+DOX+TMP	ERY+DOX+TMP	AMP+CPR+ERY+DOX	AMP+CPR+ERY+TMP	AMP+CPR+DOX+TMP	AMP+ERY+DOX+TMP	CPR+ERY+DOX+TMP	AMP+CPR+ERY+DOX+TMP
	86.21081004	101.5810872	95.55789791	98.24088523	95.97105972	61.92367712	86.8917577	88.253653	102.7805362	100.3764493	102.5447975	104.472218	96.52160819	96.03975305	95.51709462	78.03943822	85.52986239	88.93460065	86.21081004	99.14881543	101.872606	100.8583045	102.5447975	101.8220148	97.00346333	82.80607178	86.8917577	87.3457228	97.78692013	98.20810119	82.35210668
	86.39503736	97.67407187	99.10541226	105.1177217	102.1852531	79.85337657	84.36486677	83.46256873	98.3504864	96.24273147	102.2066498	106.7392277	97.1969584	98.62829879	102.8619766	72.63499225	85.0415903	85.49273932	74.43958833	99.92950797	98.80163542	99.58252572	106.2621142	92.90293723	93.14149396	78.72550402	84.36486677	89.32750599	94.74129423	93.61860743	74.66516284
	74.51039019	97.65422696	96.19192688	98.66945732	110.1509942	70.20481387	73.07519809	85.99192704	101.7790402	94.72962681	105.6968774	105.9405941	98.385377	103.5034273	106.3238152	68.2912244	85.51352968	95.55987442	82.40394678	98.43025863	100.1046494	104.478294	110.0837776	111.302361	104.478294	79.0551652	99.14785469	97.71266258	94.363881	100.8225438	97.95186126
AMP1CPR2ERY2DOX1TMP3	AMP	CPR	ERY	DOX	TMP	AMP+CPR	AMP+ERY	AMP+DOX	AMP+TMP	CPR+ERY	CPR+DOX	CPR+TMP	ERY+DOX	ERY+TMP	DOX+TMP	AMP+CPR+ERY	AMP+CPR+DOX	AMP+CPR+TMP	AMP+ERY+DOX	AMP+ERY+TMP	AMP+DOX+TMP	CPR+ERY+DOX	CPR+ERY+TMP	CPR+DOX+TMP	ERY+DOX+TMP	AMP+CPR+ERY+DOX	AMP+CPR+ERY+TMP	AMP+CPR+DOX+TMP	AMP+ERY+DOX+TMP	CPR+ERY+DOX+TMP	AMP+CPR+ERY+DOX+TMP
	86.21081004	101.5810872	95.55789791	98.24088523	98.69485033	61.92367712	86.8917577	88.253653	94.15519932	100.3764493	102.5447975	103.7494353	96.52160819	98.44902876	100.9646758	78.03943822	85.52986239	86.8917577	86.21081004	96.42502483	100.9646758	100.8583045	99.17181147	101.3401596	96.76253576	82.80607178	82.80607178	90.29649596	99.14881543	102.785725	86.8917577
	86.39503736	97.67407187	99.10541226	105.1177217	98.80163542	79.85337657	84.36486677	83.46256873	90.90652756	96.24273147	102.2066498	101.7295363	97.1969584	99.34396899	105.3432962	72.63499225	85.0415903	83.01141971	74.43958833	91.35767658	98.3504864	99.58252572	103.6379902	100.7753094	70.24004771	78.72550402	75.34188637	88.87635697	93.16227266	95.52706128	80.30452559
	74.51039019	97.65422696	96.19192688	98.66945732	109.1941994	70.20481387	73.07519809	85.99192704	79.77276125	94.72962681	105.6968774	106.6717441	98.385377	97.1667936	109.4333981	68.2912244	85.51352968	86.7095231	82.40394678	88.1447152	99.62625206	104.478294	105.9405941	107.6466108	101.3099772	79.0551652	92.68949021	95.79907311	91.49349679	112.7646611	97.95186126
AMP1CPR2ERY2DOX2TMP1	AMP	CPR	ERY	DOX	TMP	AMP+CPR	AMP+ERY	AMP+DOX	AMP+TMP	CPR+ERY	CPR+DOX	CPR+TMP	ERY+DOX	ERY+TMP	DOX+TMP	AMP+CPR+ERY	AMP+CPR+DOX	AMP+CPR+TMP	AMP+ERY+DOX	AMP+ERY+TMP	AMP+DOX+TMP	CPR+ERY+DOX	CPR+ERY+TMP	CPR+DOX+TMP	ERY+DOX+TMP	AMP+CPR+ERY+DOX	AMP+CPR+ERY+TMP	AMP+CPR+DOX+TMP	AMP+ERY+DOX+TMP	CPR+ERY+DOX+TMP	AMP+CPR+ERY+DOX+TMP
	86.21081004	101.5810872	95.55789791	99.82976309	91.88537381	61.92367712	86.8917577	90.52347851	87.7996879	100.3764493	100.6173769	100.3764493	96.52160819	97.00346333	97.33295503	78.03943822	90.52347851	90.29649596	87.3457228	85.07589729	98.92183288	95.79882548	100.3764493	102.785725	99.65366662	83.03305433	84.84891474	85.30287984	93.02028657	95.79882548	89.1615832
	86.39503736	97.67407187	99.10541226	103.9898491	100.1550825	79.85337657	84.36486677	93.83899619	96.99703933	96.24273147	102.2066498	105.7850007	96.24273147	99.82108245	98.12491188	72.63499225	86.39503736	86.16946285	83.23699422	95.86916678	99.47835895	98.86685552	104.3536604	100.7753094	103.6379902	78.04878049	86.84618638	90.90652756	94.29014521	91.9487103	84.81601579
	74.51039019	97.65422696	96.19192688	105.1278218	106.8022126	70.20481387	73.07519809	91.49349679	96.03827179	94.72962681	94.24219345	110.0837776	99.6039604	107.4028941	99.86545074	68.2912244	87.90551652	97.23426521	87.42711915	96.27747047	103.2142323	101.5536938	70.84539223	106.4280274	106.4280274	80.72955599	95.32067574	98.908656	94.84227837	110.0837776	100.3438481
AMP1CPR2ERY2DOX2TMP2	AMP	CPR	ERY	DOX	TMP	AMP+CPR	AMP+ERY	AMP+DOX	AMP+TMP	CPR+ERY	CPR+DOX	CPR+TMP	ERY+DOX	ERY+TMP	DOX+TMP	AMP+CPR+ERY	AMP+CPR+DOX	AMP+CPR+TMP	AMP+ERY+DOX	AMP+ERY+TMP	AMP+DOX+TMP	CPR+ERY+DOX	CPR+ERY+TMP	CPR+DOX+TMP	ERY+DOX+TMP	AMP+CPR+ERY+DOX	AMP+CPR+ERY+TMP	AMP+CPR+DOX+TMP	AMP+ERY+DOX+TMP	CPR+ERY+DOX+TMP	AMP+CPR+ERY+DOX+TMP
	86.21081004	101.5810872	95.55789791	99.82976309	95.97105972	61.92367712	86.8917577	90.52347851	102.7805362	100.3764493	100.6173769	104.472218	96.52160819	96.03975305	100.9646758	78.03943822	90.52347851	88.93460065	87.3457228	99.14881543	100.9646758	95.79882548	102.5447975	98.20810119	100.1355218	83.03305433	86.8917577	91.43140871	98.46786778	103.5085078	93.70123422
	86.39503736	97.67407187	99.10541226	103.9898491	102.1852531	79.85337657	84.36486677	93.83899619	98.3504864	96.24273147	102.2066498	106.7392277	96.24273147	98.62829879	98.80163542	72.63499225	86.39503736	85.49273932	83.23699422	99.92950797	102.1852531	98.86685552	106.2621142	100.7753094	97.1969584	78.04878049	84.36486677	92.25997462	99.25278444	93.61860743	93.38784717
	74.51039019	97.65422696	96.19192688	105.1278218	110.1509942	70.20481387	73.07519809	91.49349679	101.7790402	94.72962681	94.24219345	105.9405941	99.6039604	103.5034273	99.14785469	68.2912244	87.90551652	95.55987442	87.42711915	98.43025863	104.8886231	101.5536938	110.0837776	111.5460777	103.2597106	80.72955599	99.14785469	94.84227837	101.0614442	111.0586443	103.9318284
AMP1CPR2ERY2DOX2TMP3	AMP	CPR	ERY	DOX	TMP	AMP+CPR	AMP+ERY	AMP+DOX	AMP+TMP	CPR+ERY	CPR+DOX	CPR+TMP	ERY+DOX	ERY+TMP	DOX+TMP	AMP+CPR+ERY	AMP+CPR+DOX	AMP+CPR+TMP	AMP+ERY+DOX	AMP+ERY+TMP	AMP+DOX+TMP	CPR+ERY+DOX	CPR+ERY+TMP	CPR+DOX+TMP	ERY+DOX+TMP	AMP+CPR+ERY+DOX	AMP+CPR+ERY+TMP	AMP+CPR+DOX+TMP	AMP+ERY+DOX+TMP	CPR+ERY+DOX+TMP	AMP+CPR+ERY+DOX+TMP
	86.21081004	101.5810872	95.55789791	99.82976309	98.69485033	61.92367712	86.8917577	90.52347851	94.15519932	100.3764493	100.6173769	103.7494353	96.52160819	98.44902876	100.7376933	78.03943822	90.52347851	86.8917577	87.3457228	96.42502483	97.55993758	95.79882548	99.17181147	101.3401596	101.099232	83.03305433	82.80607178	93.92821677	76.90452546	100.1355218	88.93460065
	86.39503736	97.67407187	99.10541226	103.9898491	98.80163542	79.85337657	84.36486677	93.83899619	90.90652756	96.24273147	102.2066498	101.7295363	96.24273147	99.34396899	98.80163542	72.63499225	86.39503736	83.01141971	83.23699422	91.35767658	93.16227266	98.86685552	103.6379902	96.71984494	92.42582377	78.04878049	75.34188637	87.74848442	94.29014521	91.9487103	85.49273932
	74.51039019	97.65422696	96.19192688	105.1278218	109.1941994	70.20481387	73.07519809	91.49349679	79.77276125	94.72962681	94.24219345	106.6717441	99.6039604	97.1667936	98.19105995	68.2912244	87.90551652	86.7095231	87.42711915	88.1447152	98.19105995	101.5536938	105.9405941	104.9657273	99.6039604	80.72955599	92.68949021	92.45029152	93.40708626	91.80502666	94.363881
AMP1CPR2ERY2DOX3TMP1	AMP	CPR	ERY	DOX	TMP	AMP+CPR	AMP+ERY	AMP+DOX	AMP+TMP	CPR+ERY	CPR+DOX	CPR+TMP	ERY+DOX	ERY+TMP	DOX+TMP	AMP+CPR+ERY	AMP+CPR+DOX	AMP+CPR+TMP	AMP+ERY+DOX	AMP+ERY+TMP	AMP+DOX+TMP	CPR+ERY+DOX	CPR+ERY+TMP	CPR+DOX+TMP	ERY+DOX+TMP	AMP+CPR+ERY+DOX	AMP+CPR+ERY+TMP	AMP+CPR+DOX+TMP	AMP+ERY+DOX+TMP	CPR+ERY+DOX+TMP	AMP+CPR+ERY+DOX+TMP
	86.21081004	101.5810872	95.55789791	104.1424316	91.88537381	61.92367712	86.8917577	90.52347851	87.7996879	100.3764493	98.44902876	100.3764493	94.83511519	97.00346333	104.1424316	78.03943822	90.52347851	90.29649596	86.66477515	85.07589729	102.3265711	97.96717362	100.3764493	103.0266526	103.7494353	82.57908923	84.84891474	100.2837282	94.38218187	105.1950008	95.29011207
	86.39503736	97.67407187	99.10541226	105.5688707	100.1550825	79.85337657	84.36486677	96.09474129	96.99703933	96.24273147	103.3994334	105.7850007	98.62829879	99.82108245	92.25997462	72.63499225	84.59044128	86.16946285	86.62061187	95.86916678	92.03440011	99.58252572	104.3536604	103.6379902	93.85716416	83.23699422	86.84618638	95.41801776	103.3131256	101.2524228	100.6062315
	74.51039019	97.65422696	96.19192688	103.6926297	106.8022126	70.20481387	73.07519809	95.79907311	96.03827179	94.72962681	105.9405941	110.0837776	101.7974105	107.4028941	93.16788758	68.2912244	91.73269547	97.23426521	87.66631784	96.27747047	99.86545074	101.0662605	70.84539223	110.0837776	99.6039604	87.18792047	95.32067574	107.998206	98.66945732	108.8651942	101.0614442
AMP1CPR2ERY2DOX3TMP2	AMP	CPR	ERY	DOX	TMP	AMP+CPR	AMP+ERY	AMP+DOX	AMP+TMP	CPR+ERY	CPR+DOX	CPR+TMP	ERY+DOX	ERY+TMP	DOX+TMP	AMP+CPR+ERY	AMP+CPR+DOX	AMP+CPR+TMP	AMP+ERY+DOX	AMP+ERY+TMP	AMP+DOX+TMP	CPR+ERY+DOX	CPR+ERY+TMP	CPR+DOX+TMP	ERY+DOX+TMP	AMP+CPR+ERY+DOX	AMP+CPR+ERY+TMP	AMP+CPR+DOX+TMP	AMP+ERY+DOX+TMP	CPR+ERY+DOX+TMP	AMP+CPR+ERY+DOX+TMP
	86.21081004	101.5810872	95.55789791	104.1424316	95.97105972	61.92367712	86.8917577	90.52347851	102.7805362	100.3764493	98.44902876	104.472218	94.83511519	96.03975305	99.37579799	78.03943822	90.52347851	88.93460065	86.66477515	99.14881543	103.0075188	97.96717362	102.5447975	81.10224364	104.2312905	82.57908923	86.8917577	99.60278054	94.38218187	99.41273905	101.4186409
	86.39503736	97.67407187	99.10541226	105.5688707	102.1852531	79.85337657	84.36486677	96.09474129	98.3504864	96.24273147	103.3994334	106.7392277	98.62829879	98.62829879	88.42520795	72.63499225	84.59044128	85.49273932	86.62061187	99.92950797	88.42520795	99.58252572	106.2621142	96.00417474	97.1969584	83.23699422	84.36486677	96.09474129	98.3504864	102.92232	100.1550825
	74.51039019	97.65422696	96.19192688	103.6926297	110.1509942	70.20481387	73.07519809	95.79907311	101.7790402	94.72962681	105.9405941	105.9405941	101.7974105	103.5034273	96.99506653	68.2912244	91.73269547	95.55987442	87.66631784	98.43025863	96.03827179	101.0662605	110.0837776	102.2848439	88.8804265	87.18792047	99.14785469	100.3438481	89.81910599	102.5285605	97.71266258
AMP1CPR2ERY2DOX3TMP3	AMP	CPR	ERY	DOX	TMP	AMP+CPR	AMP+ERY	AMP+DOX	AMP+TMP	CPR+ERY	CPR+DOX	CPR+TMP	ERY+DOX	ERY+TMP	DOX+TMP	AMP+CPR+ERY	AMP+CPR+DOX	AMP+CPR+TMP	AMP+ERY+DOX	AMP+ERY+TMP	AMP+DOX+TMP	CPR+ERY+DOX	CPR+ERY+TMP	CPR+DOX+TMP	ERY+DOX+TMP	AMP+CPR+ERY+DOX	AMP+CPR+ERY+TMP	AMP+CPR+DOX+TMP	AMP+ERY+DOX+TMP	CPR+ERY+DOX+TMP	AMP+CPR+ERY+DOX+TMP
	86.21081004	101.5810872	95.55789791	104.1424316	98.69485033	61.92367712	86.8917577	90.52347851	94.15519932	100.3764493	98.44902876	103.7494353	94.83511519	98.44902876	97.55993758	78.03943822	90.52347851	86.8917577	86.66477515	96.42502483	96.65200738	97.96717362	99.17181147	106.6405662	103.9903629	82.57908923	82.80607178	101.6456235	93.24726912	100.1355218	98.69485033
	86.39503736	97.67407187	99.10541226	105.5688707	98.80163542	79.85337657	84.36486677	96.09474129	90.90652756	96.24273147	103.3994334	101.7295363	98.62829879	99.34396899	94.29014521	72.63499225	84.59044128	83.01141971	86.62061187	91.35767658	92.93669815	99.58252572	103.6379902	101.7295363	93.85716416	83.23699422	75.34188637	94.96686874	94.74129423	98.62829879	90.22980403
	74.51039019	97.65422696	96.19192688	103.6926297	109.1941994	70.20481387	73.07519809	95.79907311	79.77276125	94.72962681	105.9405941	106.6717441	101.7974105	97.1667936	97.71266258	68.2912244	91.73269547	86.7095231	87.66631784	88.1447152	101.0614442	101.0662605	105.9405941	96.67936024	92.04874334	87.18792047	92.68949021	88.38391389	91.73269547	101.0662605	88.38391389
AMP1CPR2ERY3DOX1TMP1	AMP	CPR	ERY	DOX	TMP	AMP+CPR	AMP+ERY	AMP+DOX	AMP+TMP	CPR+ERY	CPR+DOX	CPR+TMP	ERY+DOX	ERY+TMP	DOX+TMP	AMP+CPR+ERY	AMP+CPR+DOX	AMP+CPR+TMP	AMP+ERY+DOX	AMP+ERY+TMP	AMP+DOX+TMP	CPR+ERY+DOX	CPR+ERY+TMP	CPR+DOX+TMP	ERY+DOX+TMP	AMP+CPR+ERY+DOX	AMP+CPR+ERY+TMP	AMP+CPR+DOX+TMP	AMP+ERY+DOX+TMP	CPR+ERY+DOX+TMP	AMP+CPR+ERY+DOX+TMP
	86.21081004	101.5810872	95.82539683	98.24088523	91.88537381	61.92367712	89.6155483	88.253653	87.7996879	100.1355218	102.5447975	100.3764493	89.98412698	92.26984127	92.79330401	83.03305433	85.52986239	90.29649596	91.43140871	90.52347851	99.14881543	102.785725	103.2675802	102.3038699	94.04761905	85.75684494	88.48063555	88.7076181	97.78692013	97.2443909	79.85529862
	86.39503736	97.67407187	97.32768454	105.1177217	100.1550825	79.85337657	90.22980403	83.46256873	96.99703933	98.62829879	102.2066498	105.7850007	89.24555972	104.8883819	99.25278444	80.75567461	85.0415903	86.16946285	84.13929226	98.80163542	97.22261384	102.4452065	108.1705681	95.76561801	105.4098094	79.85337657	85.0415903	88.65078246	96.77146482	93.85716416	80.07895108
	74.51039019	97.65422696	99.21451583	98.66945732	106.8022126	70.20481387	81.68635073	85.99192704	96.03827179	98.385377	105.6968774	110.0837776	96.45616266	113.282117	107.28061	70.92240993	85.51352968	97.23426521	81.44715204	102.4966363	95.79907311	102.2848439	112.7646611	111.5460777	115.4887996	78.57676783	99.86545074	101.0614442	91.97189415	107.8903275	103.453431
AMP1CPR2ERY3DOX1TMP2	AMP	CPR	ERY	DOX	TMP	AMP+CPR	AMP+ERY	AMP+DOX	AMP+TMP	CPR+ERY	CPR+DOX	CPR+TMP	ERY+DOX	ERY+TMP	DOX+TMP	AMP+CPR+ERY	AMP+CPR+DOX	AMP+CPR+TMP	AMP+ERY+DOX	AMP+ERY+TMP	AMP+DOX+TMP	CPR+ERY+DOX	CPR+ERY+TMP	CPR+DOX+TMP	ERY+DOX+TMP	AMP+CPR+ERY+DOX	AMP+CPR+ERY+TMP	AMP+CPR+DOX+TMP	AMP+ERY+DOX+TMP	CPR+ERY+DOX+TMP	AMP+CPR+ERY+DOX+TMP
	86.21081004	101.5810872	95.82539683	98.24088523	95.97105972	61.92367712	89.6155483	88.253653	102.7805362	100.1355218	102.5447975	104.472218	89.98412698	89.73015873	95.51709462	83.03305433	85.52986239	88.93460065	91.43140871	98.69485033	101.872606	102.785725	106.3996386	101.8220148	96.58730159	85.75684494	92.11235636	87.3457228	103.6884664	103.7494353	87.7996879
	86.39503736	97.67407187	97.32768454	105.1177217	102.1852531	79.85337657	90.22980403	83.46256873	98.3504864	98.62829879	102.2066498	106.7392277	89.24555972	101.7598175	102.8619766	80.75567461	85.0415903	85.49273932	84.13929226	99.70393346	98.80163542	102.4452065	105.3078873	92.90293723	106.1919505	79.85337657	82.56027069	89.32750599	99.70393346	93.85716416	85.49273932
	74.51039019	97.65422696	99.21451583	98.66945732	110.1509942	70.20481387	81.68635073	85.99192704	101.7790402	98.385377	105.6968774	105.9405941	96.45616266	110.2479285	106.3238152	70.92240993	85.51352968	95.55987442	81.44715204	100.1046494	100.1046494	102.2848439	109.3526276	111.302361	115.2129643	78.57676783	94.363881	97.71266258	98.908656	110.3274943	101.7790402
AMP1CPR2ERY3DOX1TMP3	AMP	CPR	ERY	DOX	TMP	AMP+CPR	AMP+ERY	AMP+DOX	AMP+TMP	CPR+ERY	CPR+DOX	CPR+TMP	ERY+DOX	ERY+TMP	DOX+TMP	AMP+CPR+ERY	AMP+CPR+DOX	AMP+CPR+TMP	AMP+ERY+DOX	AMP+ERY+TMP	AMP+DOX+TMP	CPR+ERY+DOX	CPR+ERY+TMP	CPR+DOX+TMP	ERY+DOX+TMP	AMP+CPR+ERY+DOX	AMP+CPR+ERY+TMP	AMP+CPR+DOX+TMP	AMP+ERY+DOX+TMP	CPR+ERY+DOX+TMP	AMP+CPR+ERY+DOX+TMP
	86.21081004	101.5810872	95.82539683	98.24088523	98.69485033	61.92367712	89.6155483	88.253653	94.15519932	100.1355218	102.5447975	103.7494353	89.98412698	95.06349206	100.9646758	83.03305433	85.52986239	86.8917577	91.43140871	97.55993758	100.9646758	102.785725	107.1224213	101.3401596	106.7460317	85.75684494	79.40133352	90.29649596	98.01390268	101.3401596	89.38856575

	AMP	CPR	ERY	DOX	TMP	AMP+CPR	AMP+ERY	AMP+DOX	AMP+TMP	CPR+ERY	CPR+DOX	CPR+TMP	ERY+DOX	ERY+TMP	DOX+TMP	AMP+CPR+ERY	AMP+CPR+DOX	AMP+CPR+TMP	AMP+ERY+DOX	AMP+ERY+TMP	AMP+DOX+TMP	CPR+ERY+DOX	CPR+ERY+TMP	CPR+DOX+TMP	ERY+DOX+TMP	AMP+CPR+ERY+DOX	AMP+CPR+ERY+TMP	AMP+CPR+DOX+TMP	AMP+ERY+DOX+TMP	CPR+ERY+DOX+TMP	AMP+CPR+ERY+DOX+TMP
	86.39503736	97.67407187	97.32768454	105.1177217	98.80163542	79.85337657	90.22980403	83.46256873	90.90652756	98.62829879	102.2066498	101.7295363	89.24555972	102.2812449	105.3432962	80.75567461	85.0415903	83.01141971	84.13929226	93.38784717	98.3504864	102.4452065	102.6837632	100.7753094	109.5812286	79.85337657	83.23699422	88.87635697	93.38784717	97.1969584	83.01141971
	74.51039019	97.65422696	99.21451583	98.66945732	109.1941994	70.20481387	81.68635073	85.99192704	79.77276125	98.385377	105.6968774	106.6717441	96.45616266	109.6962579	109.4333981	70.92240993	85.51352968	86.7095231	81.44715204	94.12468231	99.62625206	102.2848439	106.4280274	107.6466108	114.3854583	78.57676783	84.31753625	95.79907311	94.84227837	111.5460777	96.03827179
AMP1CPR2ERY3DOX2TMP1	AMP	CPR	ERY	DOX	TMP	AMP+CPR	AMP+ERY	AMP+DOX	AMP+TMP	CPR+ERY	CPR+DOX	CPR+TMP	ERY+DOX	ERY+TMP	DOX+TMP	AMP+CPR+ERY	AMP+CPR+DOX	AMP+CPR+TMP	AMP+ERY+DOX	AMP+ERY+TMP	AMP+DOX+TMP	CPR+ERY+DOX	CPR+ERY+TMP	CPR+DOX+TMP	ERY+DOX+TMP	AMP+CPR+ERY+DOX	AMP+CPR+ERY+TMP	AMP+CPR+DOX+TMP	AMP+ERY+DOX+TMP	CPR+ERY+DOX+TMP	AMP+CPR+ERY+DOX+TMP
	86.21081004	101.5810872	95.82539683	99.82976309	91.88537381	61.92367712	89.6155483	90.52347851	87.7996879	100.1355218	100.6173769	100.3764493	86.17460317	92.26984127	97.33295503	83.03305433	90.52347851	90.29649596	97.10597248	90.52347851	98.92183288	101.5810872	103.2675802	102.785725	106.2380952	92.11235636	88.48063555	85.30287984	99.82976309	103.9903629	91.65839126
	86.39503736	97.67407187	97.32768454	103.9898491	100.1550825	79.85337657	90.22980403	93.83899619	96.99703933	98.62829879	102.2066498	105.7850007	102.8026723	104.8883819	98.12491188	80.75567461	86.39503736	86.16946285	93.16227266	98.80163542	99.47835895	103.8765469	108.1705681	100.7753094	110.6240834	79.17665304	85.0415903	90.90652756	100.1550825	103.1608767	91.8088256
	74.51039019	97.65422696	99.21451583	105.1278218	106.8022126	70.20481387	81.68635073	91.49349679	96.03827179	98.385377	94.24219345	110.0837776	99.21451583	113.282117	99.86545074	70.92240993	87.90551652	97.23426521	81.68635073	102.4966363	103.2142323	104.478294	112.7646611	106.4280274	112.1787758	84.31753625	99.86545074	98.908656	102.735835	110.3274943	102.9750336
AMP1CPR2ERY3DOX2TMP2	AMP	CPR	ERY	DOX	TMP	AMP+CPR	AMP+ERY	AMP+DOX	AMP+TMP	CPR+ERY	CPR+DOX	CPR+TMP	ERY+DOX	ERY+TMP	DOX+TMP	AMP+CPR+ERY	AMP+CPR+DOX	AMP+CPR+TMP	AMP+ERY+DOX	AMP+ERY+TMP	AMP+DOX+TMP	CPR+ERY+DOX	CPR+ERY+TMP	CPR+DOX+TMP	ERY+DOX+TMP	AMP+CPR+ERY+DOX	AMP+CPR+ERY+TMP	AMP+CPR+DOX+TMP	AMP+ERY+DOX+TMP	CPR+ERY+DOX+TMP	AMP+CPR+ERY+DOX+TMP
	86.21081004	101.5810872	95.82539683	99.82976309	95.97105972	61.92367712	89.6155483	90.52347851	102.7805362	100.1355218	100.6173769	104.472218	86.17460317	89.73015873	100.9646758	83.03305433	90.52347851	88.93460065	97.10597248	98.69485033	100.9646758	101.5810872	106.3996386	98.20810119	108.5238095	92.11235636	92.11235636	91.43140871	100.2837282	102.3038699	91.43140871
	86.39503736	97.67407187	97.32768454	103.9898491	102.1852531	79.85337657	90.22980403	93.83899619	98.3504864	98.62829879	102.2066498	106.7392277	102.8026723	101.7598175	98.80163542	80.75567461	86.39503736	85.49273932	93.16227266	99.70393346	102.1852531	103.8765469	105.3078873	100.7753094	105.9312368	79.17665304	82.56027069	92.25997462	97.89933737	79.54376025	87.74848442
	74.51039019	97.65422696	99.21451583	105.1278218	110.1509942	70.20481387	81.68635073	91.49349679	101.7790402	98.385377	94.24219345	105.9405941	99.21451583	110.2479285	99.14785469	70.92240993	87.90551652	95.55987442	81.68635073	100.1046494	104.8886231	104.478294	109.3526276	111.5460777	107.7654107	84.31753625	94.363881	94.84227837	101.0614442	110.3274943	94.363881
AMP1CPR2ERY3DOX2TMP3	AMP	CPR	ERY	DOX	TMP	AMP+CPR	AMP+ERY	AMP+DOX	AMP+TMP	CPR+ERY	CPR+DOX	CPR+TMP	ERY+DOX	ERY+TMP	DOX+TMP	AMP+CPR+ERY	AMP+CPR+DOX	AMP+CPR+TMP	AMP+ERY+DOX	AMP+ERY+TMP	AMP+DOX+TMP	CPR+ERY+DOX	CPR+ERY+TMP	CPR+DOX+TMP	ERY+DOX+TMP	AMP+CPR+ERY+DOX	AMP+CPR+ERY+TMP	AMP+CPR+DOX+TMP	AMP+ERY+DOX+TMP	CPR+ERY+DOX+TMP	AMP+CPR+ERY+DOX+TMP
	86.21081004	101.5810872	95.82539683	99.82976309	98.69485033	61.92367712	89.6155483	90.52347851	94.15519932	100.1355218	100.6173769	103.7494353	86.17460317	95.06349206	100.7376933	83.03305433	90.52347851	86.8917577	97.10597248	97.55993758	97.55993758	101.5810872	107.1224213	101.3401596	105.984127	92.11235636	79.40133352	93.92821677	97.78692013	105.4359283	89.84253086
	86.39503736	97.67407187	97.32768454	103.9898491	98.80163542	79.85337657	90.22980403	93.83899619	90.90652756	98.62829879	102.2066498	101.7295363	102.8026723	102.2812449	98.80163542	80.75567461	86.39503736	83.01141971	93.16227266	93.38784717	93.16227266	103.8765469	102.6837632	96.71984494	92.89555157	79.17665304	83.23699422	87.74848442	95.19244325	94.09572089	89.5530805
	74.51039019	97.65422696	99.21451583	105.1278218	109.1941994	70.20481387	81.68635073	91.49349679	79.77276125	98.385377	94.24219345	106.6717441	99.21451583	109.6962579	98.19105995	70.92240993	87.90551652	86.7095231	81.68635073	94.12468231	98.19105995	104.478294	106.4280274	104.9657273	101.4211984	84.31753625	84.31753625	92.45029152	99.62625206	109.5963442	97.4734639
AMP1CPR2ERY3DOX3TMP1	AMP	CPR	ERY	DOX	TMP	AMP+CPR	AMP+ERY	AMP+DOX	AMP+TMP	CPR+ERY	CPR+DOX	CPR+TMP	ERY+DOX	ERY+TMP	DOX+TMP	AMP+CPR+ERY	AMP+CPR+DOX	AMP+CPR+TMP	AMP+ERY+DOX	AMP+ERY+TMP	AMP+DOX+TMP	CPR+ERY+DOX	CPR+ERY+TMP	CPR+DOX+TMP	ERY+DOX+TMP	AMP+CPR+ERY+DOX	AMP+CPR+ERY+TMP	AMP+CPR+DOX+TMP	AMP+ERY+DOX+TMP	CPR+ERY+DOX+TMP	AMP+CPR+ERY+DOX+TMP
	86.21081004	101.5810872	95.82539683	104.1424316	91.88537381	61.92367712	89.6155483	90.52347851	87.7996879	100.1355218	98.44902876	100.3764493	90.49206349	92.26984127	104.1424316	83.03305433	90.52347851	90.29649596	94.83614697	90.52347851	102.3265711	98.9308839	103.2675802	103.0266526	108.2698413	84.39494964	88.48063555	100.2837282	92.79330401	99.17181147	96.65200738
	86.39503736	97.67407187	97.32768454	105.5688707	100.1550825	[illegible]	[illegible]	[illegible]	[illegible]	[illegible]	[illegible]	[illegible]	[illegible]	[illegible]	[illegible]	[illegible]	[illegible]	[illegible]	[illegible]	[illegible]	[illegible]	[illegible]	[illegible]	[illegible]	[illegible]	[illegible]	[illegible]	[illegible]	[illegible]	[illegible]	[illegible]
	74.51039019	97.65422696	99.21451583	103.6926297	106.8022126	[illegible]	[illegible]	[illegible]	[illegible]	[illegible]	[illegible]	[illegible]	[illegible]	[illegible]	[illegible]	[illegible]	[illegible]	[illegible]	[illegible]	[illegible]	[illegible]	[illegible]	[illegible]	[illegible]	[illegible]	[illegible]	[illegible]	[illegible]	[illegible]	[illegible]	[illegible]
AMP1CPR2ERY3DOX3TMP2	AMP	CPR	ERY	DOX	TMP	[illegible]	[illegible]	[illegible]	[illegible]	[illegible]	[illegible]	[illegible]	[illegible]	[illegible]	[illegible]	[illegible]	[illegible]	[illegible]	[illegible]	[illegible]	[illegible]	[illegible]	[illegible]	[illegible]	[illegible]	[illegible]	[illegible]	[illegible]	[illegible]	[illegible]	[illegible]
	86.21081004	101.5810872	95.82539683	104.1424316	95.97105972	[illegible]	[illegible]	[illegible]	[illegible]	[illegible]	[illegible]	[illegible]	[illegible]	[illegible]	[illegible]	[illegible]	[illegible]	[illegible]	[illegible]	[illegible]	[illegible]	[illegible]	[illegible]	[illegible]	[illegible]	[illegible]	[illegible]	[illegible]	[illegible]	[illegible]	[illegible]
	86.39503736	97.67407187	97.32768454	105.5688707	102.1852531	[illegible]	[illegible]	[illegible]	[illegible]	[illegible]	[illegible]	[illegible]	[illegible]	[illegible]	[illegible]	[illegible]	[illegible]	[illegible]	[illegible]	[illegible]	[illegible]	[illegible]	[illegible]	[illegible]	[illegible]	[illegible]	[illegible]	[illegible]	[illegible]	[illegible]	[illegible]
	74.51039019	97.65422696	99.21451583	103.6926297	110.1509942	[illegible]	[illegible]	[illegible]	[illegible]	[illegible]	[illegible]	[illegible]	[illegible]	[illegible]	[illegible]	[illegible]	[illegible]	[illegible]	[illegible]	[illegible]	[illegible]	[illegible]	[illegible]	[illegible]	[illegible]	[illegible]	[illegible]	[illegible]	[illegible]	[illegible]	[illegible]

[illegible]

AMP2CPR1ERY2DOX2TMP3	AMP	CPR	ERY	DOX	TMP	AMP+CPR	AMP+ERY	AMP+DOX	AMP+TMP	CPR+ERY	CPR+DOX	CPR+TMP	ERY+DOX	ERY+TMP	DOX+TMP	AMP+CPR+ERY	AMP+CPR+DOX	AMP+CPR+TMP	AMP+ERY+DOX	AMP+ERY+TMP	AMP+DOX+TMP	CPR+ERY+DOX	CPR+ERY+TMP	CPR+DOX+TMP	ERY+DOX+TMP	AMP+CPR+ERY+DOX	AMP+CPR+ERY+TMP	AMP+CPR+DOX+TMP	AMP+ERY+DOX+TMP	CPR+ERY+DOX+TMP	AMP+CPR+ERY+DOX+TMP
	85.67986749	91.65839126	95.55789791	99.82976309	98.69485033	79.41575064	87.84821563	95.07604276	95.31697034	99.60278054	98.46786778	101.4186409	96.52160819	98.44902876	100.7376933	73.39256136	90.73934648	98.44902876	93.63047734	95.31697034	93.87140491	100.0567456	102.5535537	102.5535537	101.099232	85.43893992	90.01656377	89.53470863	91.70305677	90.06951341	93.38954977
	86.46190547	101.5085295	99.10541226	103.9898491	98.80163542	88.60891606	86.46190547	93.85716416	97.67407187	97.44818835	103.9898491	105.5688707	96.24273147	99.34396899	98.80163542	82.40644103	93.61860743	100.0596392	88.60891606	94.57283435	87.89324586	98.80163542	101.5085295	95.41801776	92.42582377	83.36066796	90.99448338	88.37035933	88.13180259	88.42520795	73.81839869
	93.02361005	92.45029152	96.19192688	105.1278218	109.1941994	82.05635948	96.19192688	80.59405941	96.67936024	92.92868889	100.3438481	109.6725968	99.6039604	97.1667936	98.19105995	81.32520944	92.53617669	92.29246002	91.3175933	90.34272658	98.385377	102.2574376	102.9750336	103.6926297	99.6039604	85.71210967	82.30007616	88.39299315	93.02361005	104.4102257	40.62452399

AMP2CPR1ERY2DOX3TMP1	AMP	CPR	ERY	DOX	TMP	AMP+CPR	AMP+ERY	AMP+DOX	AMP+TMP	CPR+ERY	CPR+DOX	CPR+TMP	ERY+DOX	ERY+TMP	DOX+TMP	AMP+CPR+ERY	AMP+CPR+DOX	AMP+CPR+TMP	AMP+ERY+DOX	AMP+ERY+TMP	AMP+DOX+TMP	CPR+ERY+DOX	CPR+ERY+TMP	CPR+DOX+TMP	ERY+DOX+TMP	AMP+CPR+ERY+DOX	AMP+CPR+ERY+TMP	AMP+CPR+DOX+TMP	AMP+ERY+DOX+TMP	CPR+ERY+DOX+TMP	AMP+CPR+ERY+DOX+TMP
	85.67986749	91.65839126	95.55789791	104.1424316	91.88537381	79.41575064	87.84821563	93.38954977	99.17181147	99.60278054	102.0995886	88.93460065	94.83511519	97.00346333	104.1424316	73.39256136	96.76253576	91.22120163	96.28068062	100.8583045	99.17181147	101.872606	93.92821677	101.1916584	103.7494353	90.25749134	100.8583045	100.1355218	93.63047734	103.2345013	100.8583045
	86.46190547	101.5085295	99.10541226	105.5688707	100.1550825	88.60891606	86.46190547	95.04994782	101.4909796	97.44818835	108.2757648	103.5387001	98.62829879	99.82108245	92.25997462	82.40644103	90.51736991	94.81139108	93.85716416	100.0596392	100.2981959	105.5688707	99.92950797	91.58325109	93.85716416	86.22334874	91.71015357	97.43551513	94.81139108	86.16946285	93.38005069
	93.02361005	92.45029152	96.19192688	103.6926297	106.8022126	82.05635948	96.19192688	96.19192688	100.3351104	92.92868889	102.0182389	106.5630139	101.7974105	107.4028941	93.16788758	81.32520944	94.24219345	99.11652704	78.15689261	98.14166032	102.7722772	104.1710271	97.95186126	102.0182389	99.6039604	88.14927647	92.77989337	99.11652704	101.3099772	104.8886231	97.89794364

AMP2CPR1ERY2DOX3TMP2	AMP	CPR	ERY	DOX	TMP	AMP+CPR	AMP+ERY	AMP+DOX	AMP+TMP	CPR+ERY	CPR+DOX	CPR+TMP	ERY+DOX	ERY+TMP	DOX+TMP	AMP+CPR+ERY	AMP+CPR+DOX	AMP+CPR+TMP	AMP+ERY+DOX	AMP+ERY+TMP	AMP+DOX+TMP	CPR+ERY+DOX	CPR+ERY+TMP	CPR+DOX+TMP	ERY+DOX+TMP	AMP+CPR+ERY+DOX	AMP+CPR+ERY+TMP	AMP+CPR+DOX+TMP	AMP+ERY+DOX+TMP	CPR+ERY+DOX+TMP	AMP+CPR+ERY+DOX+TMP
	85.67986749	91.65839126	95.55789791	104.1424316	95.97105972	79.41575064	87.84821563	93.38954977	103.0266526	99.60278054	102.0995886	96.87898993	94.83511519	96.03975305	99.37579799	73.39256136	96.76253576	99.41273905	96.28068062	104.7131456	93.38954977	101.872606	100.9646758	89.38856575	104.2312905	90.25749134	95.31697034	98.68995633	93.87140491	100.9646758	90.01656377
	86.46190547	101.5085295	99.10541226	105.5688707	102.1852531	88.60891606	86.46190547	95.04994782	101.0138661	97.44818835	108.2757648	108.5013393	98.62829879	98.62829879	88.42520795	82.40644103	90.51736991	99.34396899	93.85716416	101.0138661	95.28850455	105.5688707	107.1478923	86.16946285	97.1969584	86.22334874	93.14149396	99.58252572	94.33427762	84.59044128	86.22334874
	93.02361005	92.45029152	96.19192688	103.6926297	110.1509942	82.05635948	96.19192688	96.19192688	101.3099772	92.92868889	102.0182389	110.6293915	101.7974105	103.5034273	96.99506653	81.32520944	94.24219345	99.11652704	78.15689261	98.62909368	97.89794364	104.1710271	103.9318284	93.64628495	88.8804265	88.14927647	91.80502666	87.17440975	95.21706017	100.5830468	90.58644326

AMP2CPR1ERY2DOX3TMP3	AMP	CPR	ERY	DOX	TMP	AMP+CPR	AMP+ERY	AMP+DOX	AMP+TMP	CPR+ERY	CPR+DOX	CPR+TMP	ERY+DOX	ERY+TMP	DOX+TMP	AMP+CPR+ERY	AMP+CPR+DOX	AMP+CPR+TMP	AMP+ERY+DOX	AMP+ERY+TMP	AMP+DOX+TMP	CPR+ERY+DOX	CPR+ERY+TMP	CPR+DOX+TMP	ERY+DOX+TMP	AMP+CPR+ERY+DOX	AMP+CPR+ERY+TMP	AMP+CPR+DOX+TMP	AMP+ERY+DOX+TMP	CPR+ERY+DOX+TMP	AMP+CPR+ERY+DOX+TMP
	85.67986749	91.65839126	95.55789791	104.1424316	98.69485033	79.41575064	87.84821563	93.38954977	95.31697034	99.60278054	102.0995886	101.4186409	94.83511519	98.44902876	97.55993758	73.39256136	96.76253576	98.44902876	96.28068062	95.31697034	93.38954977	101.872606	102.5535537	98.69485033	103.9903629	90.25749134	90.01656377	97.00346333	92.18491191	100.9646758	90.49841891
	86.46190547	101.5085295	99.10541226	105.5688707	98.80163542	88.60891606	86.46190547	95.04994782	97.67407187	97.44818835	108.2757648	105.5688707	98.62829879	99.34396899	94.29014521	82.40644103	90.51736991	100.0596392	93.85716416	94.57283435	94.33427762	105.5688707	101.5085295	94.29014521	93.85716416	86.22334874	90.99448338	92.90293723	90.99448338	91.13210207	84.79200835
	93.02361005	92.45029152	96.19192688	103.6926297	109.1941994	82.05635948	96.19192688	96.19192688	96.67936024	92.92868889	102.0182389	109.6725968	101.7974105	97.1667936	97.71266258	81.32520944	94.24219345	92.29246002	78.15689261	90.34272658	90.34272658	104.1710271	102.9750336	96.03827179	92.04874334	88.14927647	82.30007616	86.93069307	91.3175933	100.5830468	85.71210967

AMP2CPR1ERY3DOX1TMP1	AMP	CPR	ERY	DOX	TMP	AMP+CPR	AMP+ERY	AMP+DOX	AMP+TMP	CPR+ERY	CPR+DOX	CPR+TMP	ERY+DOX	ERY+TMP	DOX+TMP	AMP+CPR+ERY	AMP+CPR+DOX	AMP+CPR+TMP	AMP+ERY+DOX	AMP+ERY+TMP	AMP+DOX+TMP	CPR+ERY+DOX	CPR+ERY+TMP	CPR+DOX+TMP	ERY+DOX+TMP	AMP+CPR+ERY+DOX	AMP+CPR+ERY+TMP	AMP+CPR+DOX+TMP	AMP+ERY+DOX+TMP	CPR+ERY+DOX+TMP	AMP+CPR+ERY+DOX+TMP
	85.67986749	91.65839126	95.82539683	98.24088523	91.88537381	79.41575064	91.70305677	91.70305677	99.17181147	98.69485033	101.4186409	88.93460065	89.98412698	92.26984127	92.79330401	80.37946092	91.4621292	91.22120163	95.79882548	102.3038699	95.55789791	92.33933891	97.78692013	95.51709462	94.04761905	83.02966421	102.5447975	101.099232	94.11233248	90.06951341	101.8220148
	86.46190547	101.5085295	97.32768454	105.1177217	100.1550825	88.60891606	87.89324586	92.6643805	101.4909796	101.9596786	103.3131256	103.5387001	89.24555972	104.8883819	99.25278444	85.98479201	91.9487103	94.81139108	89.32458625	100.7753094	96.71984494	100.6062315	106.4711688	101.0573805	105.4098094	88.13180259	92.6643805	98.15118533	95.28850455	96.77146482	92.18726703
	93.02361005	92.45029152	99.21451583	98.66945732	106.8022126	82.05635948	95.21706017	90.0990099	100.3351104	98.19105995	100.8222455	106.5630139	96.45616266	113.282117	107.28061	85.46839299	95.94821021	99.11652704	88.8804265	102.5285605	103.2597106	97.95186126	104.6494244	106.0846165	115.4887996	89.61157654	98.87281036	99.11652704	98.14166032	98.66945732	93.26732673

AMP2CPR1ERY3DOX1TMP2	AMP	CPR	ERY	DOX	TMP	AMP+CPR	AMP+ERY	AMP+DOX	AMP+TMP	CPR+ERY	CPR+DOX	CPR+TMP	ERY+DOX	ERY+TMP	DOX+TMP	AMP+CPR+ERY	AMP+CPR+DOX	AMP+CPR+TMP	AMP+ERY+DOX	AMP+ERY+TMP	AMP+DOX+TMP	CPR+ERY+DOX	CPR+ERY+TMP	CPR+DOX+TMP	ERY+DOX+TMP	AMP+CPR+ERY+DOX	AMP+CPR+ERY+TMP	AMP+CPR+DOX+TMP	AMP+ERY+DOX+TMP	CPR+ERY+DOX+TMP	AMP+CPR+ERY+DOX+TMP
	85.67986749	91.65839126	95.82539683	98.24088523	95.97105972	79.41575064	91.70305677	91.70305677	103.0266526	98.69485033	101.4186409	96.87898993	89.98412698	89.73015873	95.51709462	80.37946092	91.4621292	99.41273905	95.79882548	100.6173769	96.03975305	92.33933891	98.24088523	92.11235636	96.58730159	83.02966421	99.41273905	99.89459419	97.72624605	99.37579799	100.8583045
	86.46190547	101.5085295	97.32768454	105.1177217	102.1852531	88.60891606	87.89324586	92.6643805	101.0138661	101.9596786	103.3131256	108.5013393	89.24555972	101.7598175	102.8619766	85.98479201	91.9487103	99.34396899	89.32458625	99.58252572	96.95840167	100.6062315	103.5387001	100.6062315	106.1919505	88.13180259	93.85716416	97.43551513	97.9126286	101.734104	94.09572089
	93.02361005	92.45029152	99.21451583	98.66945732	110.1509942	82.05635948	95.21706017	90.0990099	101.3099772	98.19105995	100.8222455	110.6293915	96.45616266	110.2479285	106.3238152	85.46839299	95.94821021	99.11652704	88.8804265	99.84767708	99.84767708	97.95186126	108.9550007	110.1509942	115.2129643	89.61157654	96.43564356	93.51104341	99.84767708	109.9117955	91.56130998

AMP2CPR1ERY3DOX1TMP3	AMP	CPR	ERY	DOX	TMP	AMP+CPR	AMP+ERY	AMP+DOX	AMP+TMP	CPR+ERY	CPR+DOX	CPR+TMP	ERY+DOX	ERY+TMP	DOX+TMP	AMP+CPR+ERY	AMP+CPR+DOX	AMP+CPR+TMP	AMP+ERY+DOX	AMP+ERY+TMP	AMP+DOX+TMP	CPR+ERY+DOX	CPR+ERY+TMP	CPR+DOX+TMP	ERY+DOX+TMP	AMP+CPR+ERY+DOX	AMP+CPR+ERY+TMP	AMP+CPR+DOX+TMP	AMP+ERY+DOX+TMP	CPR+ERY+DOX+TMP	AMP+CPR+ERY+DOX+TMP
	85.67986749	91.65839126	95.82539683	98.24088523	98.69485033	79.41575064	91.70305677	91.70305677	95.31697034	98.69485033	101.4186409	101.4186409	89.98412698	95.06349206	100.9646758	80.37946092	91.4621292	98.44902876	95.79882548	99.17181147	95.79882548	92.33933891	109.1360477	105.7313094	106.7460317	83.02966421	97.48531848	99.17181147	95.07604276	103.915449	99.89459419
	86.46190547	101.5085295	97.32768454	105.1177217	98.80163542	88.60891606	87.89324586	92.6643805	97.67407187	101.9596786	103.3131256	105.5688707	89.24555972	102.2812449	105.3432962	85.98479201	91.9487103	100.0596392	89.32458625	97.43551513	90.27881318	100.6062315	105.3432962	104.8921472	109.5812286	88.13180259	89.80169972	96.00417474	89.08602952	102.6364021	90.27881318
	93.02361005	92.45029152	99.21451583	98.66945732	109.1941994	82.05635948	95.21706017	90.0990099	96.67936024	98.19105995	100.8222455	109.6725968	96.45616266	109.6962579	109.4333981	85.46839299	95.94821021	92.29246002	88.8804265	89.36785986	96.43564356	97.95186126	109.9117955	112.7821797	114.3854583	89.61157654	85.71210967	97.1667936	95.46077685	109.4333981	96.67936024

AMP2CPR1ERY3DOX2TMP1	AMP	CPR	ERY	DOX	TMP	AMP+CPR	AMP+ERY	AMP+DOX	AMP+TMP	CPR+ERY	CPR+DOX	CPR+TMP	ERY+DOX	ERY+TMP	DOX+TMP	AMP+CPR+ERY	AMP+CPR+DOX	AMP+CPR+TMP	AMP+ERY+DOX	AMP+ERY+TMP	AMP+DOX+TMP	CPR+ERY+DOX	CPR+ERY+TMP	CPR+DOX+TMP	ERY+DOX+TMP	AMP+CPR+ERY+DOX	AMP+CPR+ERY+TMP	AMP+CPR+DOX+TMP	AMP+ERY+DOX+TMP	CPR+ERY+DOX+TMP	AMP+CPR+ERY+DOX+TMP
	85.67986749	91.65839126	95.82539683	99.82976309	91.88537381	79.41575064	91.70305677	95.07604276	99.17181147	98.69485033	98.46786778	88.93460065	86.17460317	92.26984127	97.33295503	80.37946092	90.73934648	91.22120163	94.35326005	102.3038699	94.59418762	100.9646758	97.78692013	98.46786778	106.2380952	83.51151935	102.5447975	101.099232	95.55789791	101.1916584	107.845204
	86.46190547	101.5085295	97.32768454	103.9898491	100.1550825	88.60891606	87.89324586	93.85716416	101.4909796	101.9596786	103.9898491	103.5387001	102.8026723	104.8883819	98.12491188	85.98479201	93.61860743	94.81139108	92.6643805	100.7753094	85.26912181	108.2757648	106.4711688	94.96686874	110.6240834	87.4161324	92.6643805	100.5367526	91.23304011	95.19244325	96.48128821
	93.02361005	92.45029152	99.21451583	105.1278218	106.8022126	82.05635948	95.21706017	80.59405941	100.3351104	98.19105995	100.3438481	106.5630139	99.21451583	113.282117	99.86545074	85.46839299	92.53617669	99.11652704	91.56130998	102.5285605	102.0411272	104.4102257	104.6494244	107.28061	112.1787758	90.58644326	98.87281036	105.209444	100.8225438	110.6293915	103.5034273

AMP2CPR1ERY3DOX2TMP2	AMP	CPR	ERY	DOX	TMP	AMP+CPR	AMP+ERY	AMP+DOX	AMP+TMP	CPR+ERY	CPR+DOX	CPR+TMP	ERY+DOX	ERY+TMP	DOX+TMP	AMP+CPR+ERY	AMP+CPR+DOX	AMP+CPR+TMP	AMP+ERY+DOX	AMP+ERY+TMP	AMP+DOX+TMP	CPR+ERY+DOX	CPR+ERY+TMP	CPR+DOX+TMP	ERY+DOX+TMP	AMP+CPR+ERY+DOX	AMP+CPR+ERY+TMP	AMP+CPR+DOX+TMP	AMP+ERY+DOX+TMP	CPR+ERY+DOX+TMP	AMP+CPR+ERY+DOX+TMP
	85.67986749	91.65839126	95.82539683	99.82976309	95.97105972	79.41575064	91.70305677	95.07604276	103.0266526	98.69485033	98.46786778	96.87898993	86.17460317	89.73015873	100.9646758	80.37946092	90.73934648	99.41273905	94.35326005	100.6173769	98.68995633	100.9646758	98.24088523	103.915449	108.5238095	83.51151935	99.41273905	103.0266526	93.87140491	100.5107107	96.76253576
	86.46190547	101.5085295	97.32768454	103.9898491	102.1852531	88.60891606	87.89324586	93.85716416	101.0138661	101.9596786	103.9898491	108.5013393	102.8026723	101.7598175	98.80163542	85.98479201	93.61860743	99.34396899	92.6643805	99.58252572	70.71716117	108.2757648	103.5387001	100.1550825	105.9312368	87.4161324	93.85716416	95.52706128	85.26912181	92.71112364	91.47159684
	93.02361005	92.45029152	99.21451583	105.1278218	110.1509942	82.05635948	95.21706017	80.59405941	101.3099772	98.19105995	100.3438481	110.6293915	99.21451583	110.2479285	99.14785469	85.46839299	92.53617669	99.11652704	91.56130998	99.84767708	105.209444	104.4102257	108.9550007	108.2374047	107.7654107	90.58644326	96.43564356	101.5536938	99.84767708	107.998206	95.94821021

AMP2CPR1ERY3DOX2TMP3	AMP	CPR	ERY	DOX	TMP	AMP+CPR	AMP+ERY	AMP+DOX	AMP+TMP	CPR+ERY	CPR+DOX	CPR+TMP	ERY+DOX	ERY+TMP	DOX+TMP	AMP+CPR+ERY	AMP+CPR+DOX	AMP+CPR+TMP	AMP+ERY+DOX	AMP+ERY+TMP	AMP+DOX+TMP	CPR+ERY+DOX	CPR+ERY+TMP	CPR+DOX+TMP	ERY+DOX+TMP	AMP+CPR+ERY+DOX	AMP+CPR+ERY+TMP	AMP+CPR+DOX+TMP	AMP+ERY+DOX+TMP	CPR+ERY+DOX+TMP	AMP+CPR+ERY+DOX+TMP
	85.67986749	91.65839126	95.82539683	99.82976309	98.69485033	79.41575064	91.70305677	95.07604276	95.31697034	98.69485033	98.46786778	101.4186409	86.17460317	95.06349206	100.7376933	80.37946092	90.73934648	98.44902876	94.35326005	99.17181147	93.87140491	100.9646758	109.1360477	102.5535537	105.984127	83.51151935	97.48531848	89.53470863	92.90769462	105.5043269	97.48531848
	86.46190547	101.5085295	97.32768454	103.9898491	98.80163542	88.60891606	87.89324586	93.85716416	97.67407187	101.9596786	103.9898491	105.5688707	102.8026723	102.2812449	98.80163542	85.98479201	93.61860743	100.0596392	92.6643805	97.43551513	87.89324586	108.2757648	105.3432962	95.41801776	92.89555157	87.4161324	89.80169972	88.37035933	85.26912181	90.90652756	83.12211123
	93.02361005	92.45029152	99.21451583	105.1278218	109.1941994	82.05635948	95.21706017	80.59405941	96.67936024	98.19105995	100.3438481	109.6725968	99.21451583	109.6962579	98.19105995	85.46839299	92.53617669	92.29246002	91.56130998	89.36785986	98.385377	104.4102257	109.9117955	103.6926297	101.4211984	90.58644326	85.71210967	88.39299315	97.41051028	107.998206	90.83015994

AMP2CPR1ERY3DOX3TMP1	AMP	CPR	ERY	DOX	TMP	AMP+CPR	AMP+ERY	AMP+DOX	AMP+TMP	CPR+ERY	CPR+DOX	CPR+TMP	ERY+DOX	ERY+TMP	DOX+TMP	AMP+CPR+ERY	AMP+CPR+DOX	AMP+CPR+TMP	AMP+ERY+DOX	AMP+ERY+TMP	AMP+DOX+TMP	CPR+ERY+DOX	CPR+ERY+TMP	CPR+DOX+TMP	ERY+DOX+TMP	AMP+CPR+ERY+DOX	AMP+CPR+ERY+TMP	AMP+CPR+DOX+TMP	AMP+ERY+DOX+TMP	CPR+ERY+DOX+TMP	AMP+CPR+ERY+DOX+TMP
	85.67986749	91.65839126	95.82539683	104.1424316	91.88537381	79.41575064	91.70305677	93.38954977	99.17181147	98.69485033	102.0995886	88.93460065	90.49206349	92.26984127	104.1424316	80.37946092	96.76253576	91.22120163	83.02966421	102.3038699	99.17181147	100.9646758	97.78692013	101.1916584	108.2698413	89.7756362	102.5447975	100.1355218	91.4621292	97.33295503	94.83511519
	86.46190547	101.5085295	97.32768454	105.5688707	100.1550825	88.60891606	87.89324586	95.04994782	101.4909796	101.9596786	108.2757648	103.5387001	102.0205312	104.8883819	92.25997462	85.98479201	90.51736991	94.81139108	97.9126286	100.7753094	100.2981959	106.9223178	106.4711688	91.58325109	89.50627342	88.84747279	92.6643805	97.43551513	94.57283435	81.88354716	95.28850455
	93.02361005	92.45029152	99.21451583	103.6926297	106.8022126	82.05635948	95.21706017	96.19192688	100.3351104	98.19105995	102.0182389	106.5630139	103.6278809	113.282117	93.16788758	85.46839299	94.24219345	99.11652704	90.58644326	102.5285605	102.7722772	100.8222455	104.6494244	102.0182389	106.9379047	81.8126428	98.87281036	99.11652704	59.63442498	99.38705337	95.94821021

AMP2CPR1ERY3DOX3TMP2	AMP	CPR	ERY	DOX	TMP	AMP+CPR	AMP+ERY	AMP+DOX	AMP+TMP	CPR+ERY	CPR+DOX	CPR+TMP	ERY+DOX	ERY+TMP	DOX+TMP	AMP+CPR+ERY	AMP+CPR+DOX	AMP+CPR+TMP	AMP+ERY+DOX	AMP+ERY+TMP	AMP+DOX+TMP	CPR+ERY+DOX	CPR+ERY+TMP	CPR+DOX+TMP	ERY+DOX+TMP	AMP+CPR+ERY+DOX	AMP+CPR+ERY+TMP	AMP+CPR+DOX+TMP	AMP+ERY+DOX+TMP	CPR+ERY+DOX+TMP	AMP+CPR+ERY+DOX+TMP
	85.67986749	91.65839126	95.82539683	104.1424316	95.97105972	79.41575064	91.70305677	93.38954977	103.0266526	98.69485033	102.0995886	96.87898993	90.49206349	89.73015873	99.37579799	80.37946092	96.76253576	99.41273905	83.02966421	100.6173769	93.38954977	100.9646758	98.24088523	89.38856575	103.4444444	89.7756362	99.41273905	98.68995633	93.87140491	103.2345013	95.79882548
	86.46190547	101.5085295	97.32768454	105.5688707	102.1852531	88.60891606	87.89324586	95.04994782	101.0138661	101.9596786	108.2757648	108.5013393	102.0205312	101.7598175	88.42520795	85.98479201	90.51736991	99.34396899	97.9126286	99.58252572	95.28850455	106.9223178	103.5387001	86.16946285	85.33485416	88.84747279	93.85716416	99.58252572	98.15118533	88.65078246	92.18726703
	93.02361005	92.45029152	99.21451583	103.6926297	110.1509942	82.05635948	95.21706017	96.19192688	101.3099772	98.19105995	102.0182389	110.6293915	103.6278809	110.2479285	96.99506653	85.46839299	94.24219345	99.11652704	90.58644326	99.84767708	97.89794364	100.8222455	108.9550007	93.64628495	108.3170813	81.8126428	96.43564356	87.17440975	98.385377	102.2574376	92.77989337

AMP2CPR1ERY3DOX3TMP3	AMP	CPR	ERY	DOX	TMP	AMP+CPR	AMP+ERY	AMP+DOX	AMP+TMP	CPR+ERY	CPR+DOX	CPR+TMP	ERY+DOX	ERY+TMP	DOX+TMP	AMP+CPR+ERY	AMP+CPR+DOX	AMP+CPR+TMP	AMP+ERY+DOX	AMP+ERY+TMP	AMP+DOX+TMP	CPR+ERY+DOX	CPR+ERY+TMP	CPR+DOX+TMP	ERY+DOX+TMP	AMP+CPR+ERY+DOX	AMP+CPR+ERY+TMP	AMP+CPR+DOX+TMP	AMP+ERY+DOX+TMP	CPR+ERY+DOX+TMP	AMP+CPR+ERY+DOX+TMP
	85.67986749	91.65839126	95.82539683	104.1424316	98.69485033	79.41575064	91.70305677	93.38954977	95.31697034	98.69485033	102.0995886	101.4186409	90.49206349	95.06349206	97.55993758	80.37946092	96.76253576	98.44902876	83.02966421	99.17181147	93.38954977	100.9646758	109.1360477	98.69485033	100.9047619	89.7756362	97.48531848	97.00346333	94.35326005	100.0567456	94.83511519
	86.46190547	101.5085295	97.32768454	105.5688707	98.80163542	88.60891606	87.89324586	95.04994782	97.67407187	101.9596786	108.2757648	105.5688707	102.0205312	102.2812449	94.29014521	85.98479201	90.51736991	100.0596392	97.9126286	97.43551513	94.33427762	106.9223178	105.3432962	94.29014521	94.19912009	88.84747279	89.80169972	92.90293723	96.24273147	93.38784717	92.90293723
	93.02361005	92.45029152	99.21451583	103.6926297	109.1941994	82.05635948	95.21706017	96.19192688	96.67936024	98.19105995	102.0182389	109.6725968	103.6278809	109.6962579	97.71266258	85.46839299	94.24219345	92.29246002	90.58644326	89.36785986	90.34272658	100.8222455	109.9117955	96.03827179	106.3862341	81.8126428	85.71210967	86.93069307	90.58644326	96.27747047	87.66184311

AMP2CPR2ERY1DOX1TMP1	AMP	CPR	ERY	DOX	TMP	AMP+CPR	AMP+ERY	AMP+DOX	AMP+TMP	CPR+ERY	CPR+DOX	CPR+TMP	ERY+DOX	ERY+TMP	DOX+TMP	AMP+CPR+ERY	AMP+CPR+DOX	AMP+CPR+TMP	AMP+ERY+DOX	AMP+ERY+TMP	AMP+DOX+TMP	CPR+ERY+DOX	CPR+ERY+TMP	CPR+DOX+TMP	ERY+DOX+TMP	AMP+CPR+ERY+DOX	AMP+CPR+ERY+TMP	AMP+CPR+DOX+TMP	AMP+ERY+DOX+TMP	CPR+ERY+DOX+TMP	AMP+CPR+ERY+DOX+TMP
	85.67986749	101.5810872	90.29649596	98.24088523	91.88537381	86.88450535	80.13853335	91.70305677	99.17181147	96.03975305	102.5447975	100.3764493	83.71400199	90.29649596	92.79330401	75.07905436	97.96717362	95.31697034	85.67986749	100.8583045	95.55789791	97.2443909	101.5810872	102.3038699	80.99021138	80.13853335	104.472218	95.31697034	93.63047734	93.1486222	92.66676705
	86.46190547	97.67407187	96.99703933	105.1177217	100.1550825	86.7004622	76.44252274	92.6643805	101.4909796	91.71015357	102.2066498	105.7850007	95.19244325	100.1550825	99.25278444	83.83778142	92.90293723	96.48128821	79.78231698	96.48128821	96.71984494	93.14149396	106.0235575	95.76561801	90.45537854	80.73654391	96.71984494	103.6379902	86.93901894	95.28850455	95.28850455
	93.02361005	97.65422696	94.12468231	98.66945732	106.8022126	84.73724296	70.60167555	90.0990099	100.3351104	66.458492	105.6968774	110.0837776	94.363881	102.0182389	107.28061	81.56892612	89.85529322	101.7974105	88.8804265	97.89794364	103.2597106	94.24219345	104.7220107	111.5460777	96.03827179	87.90555979	103.9908606	97.65422696	91.56130998	97.41051028	77.18202589

AMP2CPR2ERY1DOX1TMP2	AMP	CPR	ERY	DOX	TMP	AMP+CPR	AMP+ERY	AMP+DOX	AMP+TMP	CPR+ERY	CPR+DOX	CPR+TMP	ERY+DOX	ERY+TMP	DOX+TMP	AMP+CPR+ERY	AMP+CPR+DOX	AMP+CPR+TMP	AMP+ERY+DOX	AMP+ERY+TMP	AMP+DOX+TMP	CPR+ERY+DOX	CPR+ERY+TMP	CPR+DOX+TMP	ERY+DOX+TMP	AMP+CPR+ERY+DOX	AMP+CPR+ERY+TMP	AMP+CPR+DOX+TMP	AMP+ERY+DOX+TMP	CPR+ERY+DOX+TMP	AMP+CPR+ERY+DOX+TMP
	85.67986749	101.5810872	90.29649596	98.24088523	95.97105972	86.88450535	80.13853335	91.70305677	103.0266526	96.03975305	102.5447975	104.472218	83.71400199	88.48063555	95.51709462	75.07905436	97.96717362	95.07604276	85.67986749	98.20810119	96.03975305	97.2443909	98.68995633	101.8220148	95.06312952	80.13853335	98.9308839	94.35326005	95.79882548	97.72624605	99.89459419
	86.46190547	97.67407187	96.99703933	105.1177217	102.1852531	86.7004622	76.44252274	92.6643805	101.0138661	91.71015357	102.2066498	106.7392277	95.19244325	100.6062315	102.8619766	83.83778142	92.90293723	99.58252572	79.78231698	93.61860743	96.95840167	93.14149396	100.0596392	92.90293723	99.02720993	80.73654391	94.09572089	102.6837632	90.27881318	95.52706128	95.76561801
	93.02361005	97.65422696	94.12468231	98.66945732	110.1509942	84.73724296	70.60167555	90.0990099	101.3099772	66.458492	105.6968774	105.9405941	94.363881	103.453431	106.3238152	81.56892612	89.85529322	101.5536938	88.8804265	94.97334349	99.84767708	94.24219345	103.0159939	111.302361	106.8022126	87.90555979	98.87281036	93.51104341	92.04874334	103.5034273	56.95354151

AMP2CPR2ERY1DOX1TMP3	AMP	CPR	ERY	DOX	TMP	AMP+CPR	AMP+ERY	AMP+DOX	AMP+TMP	CPR+ERY	CPR+DOX	CPR+TMP	ERY+DOX	ERY+TMP	DOX+TMP	AMP+CPR+ERY	AMP+CPR+DOX	AMP+CPR+TMP	AMP+ERY+DOX	AMP+ERY+TMP	AMP+DOX+TMP	CPR+ERY+DOX	CPR+ERY+TMP	CPR+DOX+TMP	ERY+DOX+TMP	AMP+CPR+ERY+DOX	AMP+CPR+ERY+TMP	AMP+CPR+DOX+TMP	AMP+ERY+DOX+TMP	CPR+ERY+DOX+TMP	AMP+CPR+ERY+DOX+TMP
	85.67986749	101.5810872	90.29649596	98.24088523	98.69485033	86.88450535	80.13853335	91.70305677	95.31697034	96.03975305	102.5447975	103.7494353	83.71400199	92.11235636	100.9646758	75.07905436	97.96717362	88.81192591	85.67986749	96.03975305	95.79882548	97.2443909	98.9308839	101.3401596	96.65200738	80.13853335	94.83511519	96.28068062	90.01656377	99.41273905	91.4621292
	86.46190547	97.67407187	96.99703933	105.1177217	98.80163542	86.7004622	76.44252274	92.6643805	97.67407187	91.71015357	102.2066498	101.7295363	95.19244325	99.02720993	105.3432962	83.83778142	92.90293723	95.52706128	79.78231698	93.14149396	90.27881318	93.14149396	99.82108245	100.7753094	99.02720993	80.73654391	96.24273147	100.5367526	79.06664679	86.46190547	86.7004622
	93.02361005	97.65422696	94.12468231	98.66945732	109.1941994	84.73724296	70.60167555	90.0990099	96.67936024	66.458492	105.6968774	106.6717441	94.363881	105.6062192	109.4333981	81.56892612	89.85529322	92.29246002	88.8804265	90.58644326	96.43564356	94.24219345	102.7722772	107.6466108	108.7158021	87.90555979	84.2498096	73.5262757	82.78750952	108.6214775	88.39299315

AMP2CPR2ERY1DOX2TMP1	AMP	CPR	ERY	DOX	TMP	AMP+CPR	AMP+ERY	AMP+DOX	AMP+TMP	CPR+ERY	CPR+DOX	CPR+TMP	ERY+DOX	ERY+TMP	DOX+TMP	AMP+CPR+ERY	AMP+CPR+DOX	AMP+CPR+TMP	AMP+ERY+DOX	AMP+ERY+TMP	AMP+DOX+TMP	CPR+ERY+DOX	CPR+ERY+TMP	CPR+DOX+TMP	ERY+DOX+TMP	AMP+CPR+ERY+DOX	AMP+CPR+ERY+TMP	AMP+CPR+DOX+TMP	AMP+ERY+DOX+TMP	CPR+ERY+DOX+TMP	AMP+CPR+ERY+DOX+TMP
	85.67986749	101.5810872	90.29649596	99.82976309	91.88537381	86.88450535	80.13853335	95.07604276	99.17181147	96.03975305	100.6173769	100.3764493	93.47425167	90.29649596	97.33295503	75.07905436	97.2443909	95.31697034	92.66676705	100.8583045	94.59418762	97.48531848	101.5810872	102.785725	95.97105972	90.49841891	104.472218	98.68995633	94.59418762	95.79882548	96.76253576
	86.46190547	97.67407187	96.99703933	103.9898491	100.1550825	86.7004622	76.44252274	93.85716416	101.4909796	91.71015357	102.2066498	105.7850007	96.99703933	100.1550825	98.12491188	83.83778142	96.24273147	96.48128821	90.27881318	96.48128821	85.26912181	96.95840167	106.0235575	100.7753094	96.99703933	87.4161324	96.71984494	101.7295363	95.76561801	86.93901894	94.81139108
	93.02361005	97.65422696	94.12468231	105.1278218	106.8022126	84.73724296	70.60167555	80.59405941	100.3351104	66.458492	94.24219345	110.0837776	96.27747047	102.0182389	99.86545074	81.56892612	92.77989337	101.7974105	82.78750952	97.89794364	102.0411272	97.1667936	104.7220107	106.4280274	100.3438481	88.8804265	103.9908606	96.19192688	93.99847677	108.8651942	99.36024372

AMP2CPR2ERY1DOX2TMP2	AMP	CPR	ERY	DOX	TMP	AMP+CPR	AMP+ERY	AMP+DOX	AMP+TMP	CPR+ERY	CPR+DOX	CPR+TMP	ERY+DOX	ERY+TMP	DOX+TMP	AMP+CPR+ERY	AMP+CPR+DOX	AMP+CPR+TMP	AMP+ERY+DOX	AMP+ERY+TMP	AMP+DOX+TMP	CPR+ERY+DOX	CPR+ERY+TMP	CPR+DOX+TMP	ERY+DOX+TMP	AMP+CPR+ERY+DOX	AMP+CPR+ERY+TMP	AMP+CPR+DOX+TMP	AMP+ERY+DOX+TMP	CPR+ERY+DOX+TMP	AMP+CPR+ERY+DOX+TMP
	85.67986749	101.5810872	90.29649596	99.82976309	95.97105972	86.88450535	80.13853335	95.07604276	103.0266526	96.03975305	100.6173769	104.472218	93.47425167	88.48063555	100.9646758	75.07905436	97.2443909	95.07604276	92.66676705	98.20810119	98.68995633	97.48531848	98.68995633	98.20810119	97.55993758	90.49841891	98.9308839	103.7494353	90.98027406	96.03975305	99.41273905
	86.46190547	97.67407187	96.99703933	103.9898491	102.1852531	86.7004622	76.44252274	93.85716416	101.0138661	91.71015357	102.2066498	106.7392277	96.99703933	100.6062315	98.80163542	83.83778142	96.24273147	99.58252572	90.27881318	93.61860743	70.71716117	96.95840167	100.0596392	100.7753094	91.8088256	87.4161324	94.09572089	101.7295363	93.61860743	86.46190547	89.80169972
	93.02361005	97.65422696	94.12468231	105.1278218	110.1509942	84.73724296	70.60167555	80.59405941	101.3099772	66.458492	94.24219345	105.9405941	96.27747047	103.453431	99.14785469	81.56892612	92.77989337	101.5536938	82.78750952	94.97334349	105.209444	97.1667936	103.0159939	111.5460777	93.88548363	88.8804265	98.87281036	97.89794364	93.02361005	108.3777609	58.1721249

AMP2CPR2ERY1DOX2TMP3	AMP	CPR	ERY	DOX	TMP	AMP+CPR	AMP+ERY	AMP+DOX	AMP+TMP	CPR+ERY	CPR+DOX	CPR+TMP	ERY+DOX	ERY+TMP	DOX+TMP	AMP+CPR+ERY	AMP+CPR+DOX	AMP+CPR+TMP	AMP+ERY+DOX	AMP+ERY+TMP	AMP+DOX+TMP	CPR+ERY+DOX	CPR+ERY+TMP	CPR+DOX+TMP	ERY+DOX+TMP	AMP+CPR+ERY+DOX	AMP+CPR+ERY+TMP	AMP+CPR+DOX+TMP	AMP+ERY+DOX+TMP	CPR+ERY+DOX+TMP	AMP+CPR+ERY+DOX+TMP
	85.67986749	101.5810872	90.29649596	99.82976309	98.69485033	86.88450535	80.13853335	95.07604276	95.31697034	96.03975305	100.6173769	103.7494353	93.47425167	92.11235636	100.7376933	75.07905436	97.2443909	88.81192591	92.66676705	96.03975305	93.87140491	97.48531848	98.9308839	101.3401596	97.55993758	90.49841891	94.83511519	93.38954977	88.81192591	99.17181147	95.31697034
	86.46190547	97.67407187	96.99703933	103.9898491	98.80163542	86.7004622	76.44252274	93.85716416	97.67407187	91.71015357	102.2066498	101.7295363	96.99703933	99.02720993	98.80163542	83.83778142	96.24273147	95.52706128	90.27881318	93.14149396	87.89324586	96.95840167	99.82108245	96.71984494	90.45537854	87.4161324	96.24273147	97.67407187	89.32458625	90.75592664	83.59922469
	93.02361005	97.65422696	94.12468231	105.1278218	109.1941994	84.73724296	70.60167555	80.59405941	96.67936024	66.458492	94.24219345	106.6717441	96.27747047	105.6062192	98.19105995	81.56892612	92.77989337	92.29246002	82.78750952	90.58644326	98.385377	97.1667936	102.7722772	104.9657273	91.73269547	88.8804265	84.2498096	88.39299315	87.66184311	87.41812643	80.59405941

AMP2CPR2ERY1DOX3TMP1	AMP	CPR	ERY	DOX	TMP	AMP+CPR	AMP+ERY	AMP+DOX	AMP+TMP	CPR+ERY	CPR+DOX	CPR+TMP	ERY+DOX	ERY+TMP	DOX+TMP	AMP+CPR+ERY	AMP+CPR+DOX	AMP+CPR+TMP	AMP+ERY+DOX	AMP+ERY+TMP	AMP+DOX+TMP	CPR+ERY+DOX	CPR+ERY+TMP	CPR+DOX+TMP	ERY+DOX+TMP	AMP+CPR+ERY+DOX	AMP+CPR+ERY+TMP	AMP+CPR+DOX+TMP	AMP+ERY+DOX+TMP	CPR+ERY+DOX+TMP	AMP+CPR+ERY+DOX+TMP
	85.67986749	101.5810872	90.29649596	104.1424316	91.88537381	86.88450535	80.13853335	93.38954977	99.17181147	96.03975305	98.44902876	100.3764493	90.75046106	90.29649596	104.1424316	75.07905436	89.7756362	95.31697034	93.1486222	100.8583045	99.17181147	98.68995633	101.5810872	103.0266526	99.60278054	91.4621292	104.472218	93.63047734	90.98027406	94.11233248	102.5447975
	86.46190547	97.67407187	96.99703933	105.5688707	100.1550825	86.7004622	76.44252274	95.04994782	101.4909796	91.71015357	103.3994334	105.7850007	97.22261384	100.1550825	92.25997462	83.83778142	90.75592664	96.48128821	89.08602952	96.48128821	100.2981959	77.87386313	106.0235575	103.6379902	82.56027069	84.79200835	96.71984494	106.9777844	90.27881318	102.4452065	91.71015357
	93.02361005	97.65422696	94.12468231	103.6926297	106.8022126	84.73724296	70.60167555	96.19192688	100.3351104	66.458492	105.9405941	110.0837776	100.8222455	102.0182389	93.16788758	81.56892612	95.70449353	101.7974105	88.14927647	97.89794364	102.7722772	98.14166032	104.7220107	110.0837776	85.51352968	88.63670982	103.9908606	102.2848439	93.99847677	103.7471439	92.53617669

AMP2CPR2ERY1DOX3TMP2	AMP	CPR	ERY	DOX	TMP	AMP+CPR	AMP+ERY	AMP+DOX	AMP+TMP	CPR+ERY	CPR+DOX	CPR+TMP	ERY+DOX	ERY+TMP	DOX+TMP	AMP+CPR+ERY	AMP+CPR+DOX	AMP+CPR+TMP	AMP+ERY+DOX	AMP+ERY+TMP	AMP+DOX+TMP	CPR+ERY+DOX	CPR+ERY+TMP	CPR+DOX+TMP	ERY+DOX+TMP	AMP+CPR+ERY+DOX	AMP+CPR+ERY+TMP	AMP+CPR+DOX+TMP	AMP+ERY+DOX+TMP	CPR+ERY+DOX+TMP	AMP+CPR+ERY+DOX+TMP
	85.67986749	101.5810872	90.29649596	104.1424316	95.97105972	86.88450535	80.13853335	93.38954977	103.0266526	96.03975305	98.44902876	104.472218	90.75046106	88.48063555	99.37579799	75.07905436	89.7756362	95.07604276	93.1486222	98.20810119	93.38954977	98.68995633	98.68995633	81.10224364	95.37579799	91.4621292	98.9308839	95.55789791	93.87140491	97.48531848	97.96717362
	86.46190547	97.67407187	96.99703933	105.5688707	102.1852531	86.7004622	76.44252274	95.04994782	101.0138661	91.71015357	103.3994334	106.7392277	97.22261384	100.6062315	88.42520795	83.83778142	90.75592664	99.58252572	89.08602952	93.61860743	95.28850455	77.87386313	100.0596392	96.00417474	83.23699422	84.79200835	94.09572089	105.546444	88.84747279	100.7753094	97.1969584
	93.02361005	97.65422696	94.12468231	103.6926297	110.1509942	84.73724296	70.60167555	96.19192688	101.3099772	66.458492	105.9405941	105.9405941	100.8222455	103.453431	96.99506653	81.56892612	95.70449353	101.5536938	88.14927647	94.97334349	97.89794364	98.14166032	103.0159939	102.2848439	88.1447152	88.63670982	98.87281036	100.0913938	91.80502666	97.41051028	91.80502666

AMP2CPR2ERY1DOX3TMP3	AMP	CPR	ERY	DOX	TMP	AMP+CPR	AMP+ERY	AMP+DOX	AMP+TMP	CPR+ERY	CPR+DOX	CPR+TMP	ERY+DOX	ERY+TMP	DOX+TMP	AMP+CPR+ERY	AMP+CPR+DOX	AMP+CPR+TMP	AMP+ERY+DOX	AMP+ERY+TMP	AMP+DOX+TMP	CPR+ERY+DOX	CPR+ERY+TMP	CPR+DOX+TMP	ERY+DOX+TMP	AMP+CPR+ERY+DOX	AMP+CPR+ERY+TMP	AMP+CPR+DOX+TMP	AMP+ERY+DOX+TMP	CPR+ERY+DOX+TMP	AMP+CPR+ERY+DOX+TMP
	85.67986749	101.5810872	90.29649596	104.1424316	98.69485033	86.88450535	80.13853335	93.38954977	95.31697034	96.03975305	98.44902876	103.7494353	90.75046106	92.11235636	97.55993758	75.07905436	89.7756362	88.81192591	93.1486222	96.03975305	93.38954977	98.68995633	98.9308839	106.6405662	88.93460065	91.4621292	94.83511519	95.07604276	93.63047734	98.68995633	99.65366662
	86.46190547	97.67407187	96.99703933	105.5688707	98.80163542	86.7004622	76.44252274	95.04994782	97.67407187	91.71015357	103.3994334	101.7295363	97.22261384	99.02720993	94.29014521	83.83778142	90.75592664	95.52706128	89.08602952	93.14149396	94.33427762	77.87386313	99.82108245	101.7295363	82.7858452	84.79200835	96.24273147	100.5367526	84.55345162	101.2524228	49.39615327
	93.02361005	97.65422696	94.12468231	103.6926297	109.1941994	84.73724296	70.60167555	96.19192688	96.67936024	66.458492	105.9405941	106.6717441	100.8222455	105.6062192	97.71266258	81.56892612	95.70449353	92.29246002	88.14927647	90.58644326	90.34272658	98.14166032	102.7722772	96.67936024	88.86231126	88.63670982	84.2498096	91.07387662	92.77989337	81.08149276	80.35034273

AMP2CPR2ERY2DOX1TMP1	AMP	CPR	ERY	DOX	TMP	AMP+CPR	AMP+ERY	AMP+DOX	AMP+TMP	CPR+ERY	CPR+DOX	CPR+TMP	ERY+DOX	ERY+TMP	DOX+TMP	AMP+CPR+ERY	AMP+CPR+DOX	AMP+CPR+TMP	AMP+ERY+DOX	AMP+ERY+TMP	AMP+DOX+TMP	CPR+ERY+DOX	CPR+ERY+TMP	CPR+DOX+TMP	ERY+DOX+TMP	AMP+CPR+ERY+DOX	AMP+CPR+ERY+TMP	AMP+CPR+DOX+TMP	AMP+ERY+DOX+TMP	CPR+ERY+DOX+TMP	AMP+CPR+ERY+DOX+TMP
	85.67986749	101.5810872	95.55789791	98.24088523	91.88537381	86.88450535	87.84821563	91.70305677	99.17181147	100.3764493	102.5447975	100.3764493	96.52160819	97.00346333	92.79330401	78.21111278	97.96717362	95.31697034	97.00346333	100.8583045	95.55789791	100.8583045	100.3764493	102.3038699	92.42583948	93.1486222	103.5085078	95.31697034	97.96717362	98.68995633	98.44902876
	86.46190547	97.67407187	99.10541226	105.1177217	100.1550825	86.7004622	86.46190547	92.6643805	101.4909796	96.24273147	102.2066498	105.7850007	97.1969584	99.82108245	99.25278444	81.21365737	92.90293723	96.48128821	85.50767854	100.0596392	96.71984494	99.58252572	104.3536604	95.76561801	92.6643805	84.31489489	97.9126286	103.6379902	92.42582377	100.2981959	101.0138661
	93.02361005	97.65422696	96.19192688	98.66945732	106.8022126	84.73724296	96.19192688	90.0990099	100.3351104	94.72962681	105.6968774	110.0837776	98.385377	107.4028941	107.28061	80.59405941	89.85529322	101.7974105	93.02361005	98.14166032	103.2597106	104.478294	70.84539223	111.5460777	102.0411272	86.44325971	103.2597106	97.65422696	95.94821021	108.6214775	103.0159939

AMP2CPR2ERY2DOX1TMP2	AMP	CPR	ERY	DOX	TMP	AMP+CPR	AMP+ERY	AMP+DOX	AMP+TMP	CPR+ERY	CPR+DOX	CPR+TMP	ERY+DOX	ERY+TMP	DOX+TMP	AMP+CPR+ERY	AMP+CPR+DOX	AMP+CPR+TMP	AMP+ERY+DOX	AMP+ERY+TMP	AMP+DOX+TMP	CPR+ERY+DOX	CPR+ERY+TMP	CPR+DOX+TMP	ERY+DOX+TMP	AMP+CPR+ERY+DOX	AMP+CPR+ERY+TMP	AMP+CPR+DOX+TMP	AMP+ERY+DOX+TMP	CPR+ERY+DOX+TMP	AMP+CPR+ERY+DOX+TMP
	85.67986749	101.5810872	95.55789791	98.24088523	95.97105972	86.88450535	87.84821563	91.70305677	103.0266526	100.3764493	102.5447975	104.472218	96.52160819	96.03975305	95.51709462	78.21111278	97.96717362	95.07604276	97.00346333	104.7131456	96.03975305	100.8583045	102.5447975	101.8220148	97.00346333	93.1486222	100.8583045	94.35326005	95.31697034	98.20810119	90.49841891
	86.46190547	97.67407187	99.10541226	105.1177217	102.1852531	86.7004622	86.46190547	92.6643805	101.0138661	96.24273147	102.2066498	106.7392277	97.1969584	98.62829879	102.8619766	81.21365737	92.90293723	99.58252572	85.50767854	101.0138661	96.95840167	99.58252572	106.2621142	92.90293723	93.14149396	84.31489489	93.14149396	102.6837632	91.47159684	93.61860743	96.71984494
	93.02361005	97.65422696	96.19192688	98.66945732	110.1509942	84.73724296	96.19192688	90.0990099	101.3099772	94.72962681	105.6968774	105.9405941	98.385377	103.5034273	106.3238152	80.59405941	89.85529322	101.5536938	93.02361005	98.62909368	99.84767708	104.478294	110.0837776	111.302361	104.478294	86.44325971	101.5536938	93.51104341	96.19192688	100.8225438	97.41051028

AMP2CPR2ERY2DOX1TMP3	AMP	CPR	ERY	DOX	TMP	AMP+CPR	AMP+ERY	AMP+DOX	AMP+TMP	CPR+ERY	CPR+DOX	CPR+TMP	ERY+DOX	ERY+TMP	DOX+TMP	AMP+CPR+ERY	AMP+CPR+DOX	AMP+CPR+TMP	AMP+ERY+DOX	AMP+ERY+TMP	AMP+DOX+TMP	CPR+ERY+DOX	CPR+ERY+TMP	CPR+DOX+TMP	ERY+DOX+TMP	AMP+CPR+ERY+DOX	AMP+CPR+ERY+TMP	AMP+CPR+DOX+TMP	AMP+ERY+DOX+TMP	CPR+ERY+DOX+TMP	AMP+CPR+ERY+DOX+TMP
	85.67986749	101.5810872	95.55789791	98.24088523	98.69485033	86.88450535	87.84821563	91.70305677	95.31697034	100.3764493	102.5447975	103.7494353	96.52160819	98.44902876	100.9646758	78.21111278	97.96717362	88.81192591	97.00346333	95.31697034	95.79882548	100.8583045	99.17181147	101.3401596	96.76253576	93.1486222	90.73934648	96.28068062	92.66676705	102.785725	87.60728806
	86.46190547	97.67407187	99.10541226	105.1177217	98.80163542	86.7004622	86.46190547	92.6643805	97.67407187	96.24273147	102.2066498	101.7295363	97.1969584	99.34396899	105.3432962	81.21365737	92.90293723	95.52706128	85.50767854	94.57283435	90.27881318	99.58252572	103.6379902	100.7753094	70.24004771	84.31489489	92.18726703	100.5367526	89.32458625	95.52706128	88.37035933
	93.02361005	97.65422696	96.19192688	98.66945732	109.1941994	84.73724296	96.19192688	90.0990099	96.67936024	94.72962681	105.6968774	106.6717441	98.385377	97.1667936	109.4333981	80.59405941	89.85529322	92.29246002	93.02361005	90.34272658	96.43564356	104.478294	105.9405941	107.6466108	101.3099772	86.44325971	95.70449353	73.5262757	91.3175933	112.7646611	90.58644326

AMP2CPR2ERY2DOX2TMP1	AMP	CPR	ERY	DOX	TMP	AMP+CPR	AMP+ERY	AMP+DOX	AMP+TMP	CPR+ERY	CPR+DOX	CPR+TMP	ERY+DOX	ERY+TMP	DOX+TMP	AMP+CPR+ERY	AMP+CPR+DOX	AMP+CPR+TMP	AMP+ERY+DOX	AMP+ERY+TMP	AMP+DOX+TMP	CPR+ERY+DOX	CPR+ERY+TMP	CPR+DOX+TMP	ERY+DOX+TMP	AMP+CPR+ERY+DOX	AMP+CPR+ERY+TMP	AMP+CPR+DOX+TMP	AMP+ERY+DOX+TMP	CPR+ERY+DOX+TMP	AMP+CPR+ERY+DOX+TMP
	85.67986749	101.5810872	95.55789791	99.82976309	91.88537381	86.88450535	87.84821563	95.07604276	99.17181147	100.3764493	100.6173769	100.3764493	96.52160819	97.00346333	97.33295503	78.21111278	97.2443909	95.31697034	93.63047734	100.8583045	94.59418762	95.79882548	100.3764493	102.785725	99.65366662	95.07604276	103.5085078	98.68995633	93.38954977	95.79882548	96.28068062
	86.46190547	97.67407187	99.10541226	103.9898491	100.1550825	86.7004622	86.46190547	93.85716416	101.4909796	96.24273147	102.2066498	105.7850007	96.24273147	99.82108245	98.12491188	81.21365737	96.24273147	96.48128821	88.60891606	100.0596392	85.26912181	98.86685552	104.3536604	100.7753094	103.6379902	88.13180259	97.9126286	101.7295363	92.90293723	91.9487103	95.28850455
	93.02361005	97.65422696	96.19192688	105.1278218	106.8022126	84.73724296	96.19192688	80.59405941	100.3351104	94.72962681	94.24219345	110.0837776	99.6039604	107.4028941	99.86545074	80.59405941	92.77989337	101.7974105	91.3175933	98.14166032	102.0411272	101.5536938	70.84539223	106.4280274	106.4280274	88.39299315	103.2597106	96.19192688	65.23990861	110.0837776	96.67936024

AMP2CPR2ERY2DOX2TMP2	AMP	CPR	ERY	DOX	TMP	AMP+CPR	AMP+ERY	AMP+DOX	AMP+TMP	CPR+ERY	CPR+DOX	CPR+TMP	ERY+DOX	ERY+TMP	DOX+TMP	AMP+CPR+ERY	AMP+CPR+DOX	AMP+CPR+TMP	AMP+ERY+DOX	AMP+ERY+TMP	AMP+DOX+TMP	CPR+ERY+DOX	CPR+ERY+TMP	CPR+DOX+TMP	ERY+DOX+TMP	AMP+CPR+ERY+DOX	AMP+CPR+ERY+TMP	AMP+CPR+DOX+TMP	AMP+ERY+DOX+TMP	CPR+ERY+DOX+TMP	AMP+CPR+ERY+DOX+TMP
	85.67986749	101.5810872	95.55789791	99.82976309	95.97105972	86.88450535	87.84821563	95.07604276	103.0266526	100.3764493	100.6173769	104.472218	96.52160819	96.03975305	100.9646758	78.21111278	97.2443909	95.07604276	93.63047734	104.7131456	98.68995633	95.79882548	102.5447975	98.20810119	100.1355218	95.07604276	100.8583045	103.7494353	94.59418762	103.5085078	103.9903629
	86.46190547	97.67407187	99.10541226	103.9898491	102.1852531	86.7004622	86.46190547	93.85716416	101.0138661	96.24273147	102.2066498	106.7392277	96.24273147	98.62829879	98.80163542	81.21365737	96.24273147	99.58252572	88.60891606	101.0138661	70.71716117	98.86685552	106.2621142	100.7753094	97.1969584	88.13180259	93.14149396	101.7295363	92.42582377	93.61860743	95.76561801
	93.02361005	97.65422696	96.19192688	105.1278218	110.1509942	84.73724296	96.19192688	80.59405941	101.3099772	94.72962681	94.24219345	105.9405941	99.6039604	103.5034273	99.14785469	80.59405941	92.77989337	101.5536938	91.3175933	98.62909368	105.209444	101.5536938	110.0837776	111.5460777	103.2597106	88.39299315	101.5536938	97.89794364	99.36024372	111.0586443	96.19192688

AMP2CPR2ERY2DOX2TMP3	AMP	CPR	ERY	DOX	TMP	AMP+CPR	AMP+ERY	AMP+DOX	AMP+TMP	CPR+ERY	CPR+DOX	CPR+TMP	ERY+DOX	ERY+TMP	DOX+TMP	AMP+CPR+ERY	AMP+CPR+DOX	AMP+CPR+TMP	AMP+ERY+DOX	AMP+ERY+TMP	AMP+DOX+TMP	CPR+ERY+DOX	CPR+ERY+TMP	CPR+DOX+TMP	ERY+DOX+TMP	AMP+CPR+ERY+DOX	AMP+CPR+ERY+TMP	AMP+CPR+DOX+TMP	AMP+ERY+DOX+TMP	CPR+ERY+DOX+TMP	AMP+CPR+ERY+DOX+TMP
	85.67986749	101.5810872	95.55789791	99.82976309	98.69485033	86.88450535	87.84821563	95.07604276	95.31697034	100.3764493	100.6173769	103.7494353	96.52160819	98.44902876	100.7376933	78.21111278	97.2443909	88.81192591	93.63047734	95.31697034	93.87140491	95.79882548	99.17181147	101.3401596	101.099232	95.07604276	90.73934648	93.38954977	91.70305677	100.1355218	92.90769462
	86.46190547	97.67407187	99.10541226	103.9898491	98.80163542	86.7004622	86.46190547	93.85716416	97.67407187	96.24273147	102.2066498	101.7295363	96.24273147	99.34396899	98.80163542	81.21365737	96.24273147	95.52706128	88.60891606	94.57283435	87.89324586	98.86685552	103.6379902	96.71984494	92.42582377	88.13180259	92.18726703	97.67407187	88.13180259	91.9487103	88.37035933
	93.02361005	97.65422696	96.19192688	105.1278218	109.1941994	84.73724296	96.19192688	80.59405941	96.67936024	94.72962681	94.24219345	106.6717441	99.6039604	97.1667936	98.19105995	80.59405941	92.77989337	92.29246002	91.3175933	90.34272658	98.385377	101.5536938	105.9405941	104.9657273	99.6039604	88.39299315	95.70449353	88.39299315	93.02361005	91.80502666	87.66184311

AMP2CPR2ERY2DOX3TMP1	AMP	CPR	ERY	DOX	TMP	AMP+CPR	AMP+ERY	AMP+DOX	AMP+TMP	CPR+ERY	CPR+DOX	CPR+TMP	ERY+DOX	ERY+TMP	DOX+TMP	AMP+CPR+ERY	AMP+CPR+DOX	AMP+CPR+TMP	AMP+ERY+DOX	AMP+ERY+TMP	AMP+DOX+TMP	CPR+ERY+DOX	CPR+ERY+TMP	CPR+DOX+TMP	ERY+DOX+TMP	AMP+CPR+ERY+DOX	AMP+CPR+ERY+TMP	AMP+CPR+DOX+TMP	AMP+ERY+DOX+TMP	CPR+ERY+DOX+TMP	AMP+CPR+ERY+DOX+TMP
	85.67986749	101.5810872	95.55789791	104.1424316	91.88537381	86.88450535	87.84821563	93.38954977	99.17181147	100.3764493	98.44902876	100.3764493	94.83511519	97.00346333	104.1424316	78.21111278	89.7756362	95.31697034	96.28068062	100.8583045	99.17181147	97.96717362	100.3764493	103.0266526	103.7494353	90.98027406	103.5085078	93.63047734	93.63047734	105.1950008	97.96717362
	86.46190547	97.67407187	99.10541226	105.5688707	100.1550825	86.7004622	86.46190547	95.04994782	101.4909796	96.24273147	103.3994334	105.7850007	98.62829879	99.82108245	92.25997462	81.21365737	90.75592664	96.48128821	93.85716416	100.0596392	100.2981959	99.58252572	104.3536604	103.6379902	93.85716416	90.27881318	97.9126286	106.9777844	94.81139108	101.2524228	99.82108245
	93.02361005	97.65422696	96.19192688	103.6926297	106.8022126	84.73724296	96.19192688	96.19192688	100.3351104	94.72962681	105.9405941	110.0837776	101.7974105	107.4028941	93.16788758	80.59405941	95.70449353	101.7974105	78.15689261	98.14166032	102.7722772	101.0662605	70.84539223	110.0837776	99.6039604	93.02361005	103.2597106	102.2848439	101.3099772	108.8651942	98.14166032

AMP2CPR2ERY2DOX3TMP2	AMP	CPR	ERY	DOX	TMP	AMP+CPR	AMP+ERY	AMP+DOX	AMP+TMP	CPR+ERY	CPR+DOX	CPR+TMP	ERY+DOX	ERY+TMP	DOX+TMP	AMP+CPR+ERY	AMP+CPR+DOX	AMP+CPR+TMP	AMP+ERY+DOX	AMP+ERY+TMP	AMP+DOX+TMP	CPR+ERY+DOX	CPR+ERY+TMP	CPR+DOX+TMP	ERY+DOX+TMP	AMP+CPR+ERY+DOX	AMP+CPR+ERY+TMP	AMP+CPR+DOX+TMP	AMP+ERY+DOX+TMP	CPR+ERY+DOX+TMP	AMP+CPR+ERY+DOX+TMP
	85.67986749	101.5810872	95.55789791	104.1424316	95.97105972	86.88450535	87.84821563	93.38954977	103.0266526	100.3764493	98.44902876	104.472218	94.83511519	96.03975305	99.37579799	78.21111278	89.7756362	95.07604276	96.28068062	104.7131456	93.38954977	97.96717362	102.5447975	81.10224364	104.2312905	90.98027406	100.8583045	95.55789791	93.87140491	99.41273905	96.03975305
	86.46190547	97.67407187	99.10541226	105.5688707	102.1852531	86.7004622	86.46190547	95.04994782	101.0138661	96.24273147	103.3994334	106.7392277	98.62829879	98.62829879	88.42520795	81.21365737	90.75592664	99.58252572	93.85716416	101.0138661	95.28850455	99.58252572	106.2621142	96.00417474	97.1969584	90.27881318	93.14149396	105.546444	94.33427762	102.92232	98.15118533
	93.02361005	97.65422696	96.19192688	103.6926297	110.1509942	84.73724296	96.19192688	96.19192688	101.3099772	94.72962681	105.9405941	105.9405941	101.7974105	103.5034273	96.99506653	80.59405941	95.70449353	101.5536938	78.15689261	98.62909368	97.89794364	101.0662605	110.0837776	102.2848439	88.8804265	93.02361005	101.5536938	100.0913938	95.21706017	102.5285605	62.55902513

AMP2CPR2ERY2DOX3TMP3	AMP	CPR	ERY	DOX	TMP	AMP+CPR	AMP+ERY	AMP+DOX	AMP+TMP	CPR+ERY	CPR+DOX	CPR+TMP	ERY+DOX	ERY+TMP	DOX+TMP	AMP+CPR+ERY	AMP+CPR+DOX	AMP+CPR+TMP	AMP+ERY+DOX	AMP+ERY+TMP	AMP+DOX+TMP	CPR+ERY+DOX	CPR+ERY+TMP	CPR+DOX+TMP	ERY+DOX+TMP	AMP+CPR+ERY+DOX	AMP+CPR+ERY+TMP	AMP+CPR+DOX+TMP	AMP+ERY+DOX+TMP	CPR+ERY+DOX+TMP	AMP+CPR+ERY+DOX+TMP
	85.67986749	101.5810872	95.55789791	104.1424316	98.69485033	86.88450535	87.84821563	93.38954977	95.31697034	100.3764493	98.44902876	103.7494353	94.83511519	98.44902876	97.55993758	78.21111278	89.7756362	88.81192591	96.28068062	95.31697034	93.38954977	97.96717362	99.17181147	106.6405662	103.9903629	90.98027406	90.73934648	95.07604276	92.18491191	100.1355218	96.52160819
	86.46190547	97.67407187	99.10541226	105.5688707	98.80163542	86.7004622	86.46190547	95.04994782	97.67407187	96.24273147	103.3994334	101.7295363	98.62829879	99.34396899	94.29014521	81.21365737	90.75592664	95.52706128	93.85716416	94.57283435	94.33427762	99.58252572	103.6379902	101.7295363	93.85716416	90.27881318	92.18726703	100.5367526	90.99448338	98.62829879	93.38005069
	93.02361005	97.65422696	96.19192688	103.6926297	109.1941994	84.73724296	96.19192688	96.19192688	96.67936024	94.72962681	105.9405941	106.6717441	101.7974105	97.1667936	97.71266258	80.59405941	95.70449353	92.29246002	78.15689261	90.34272658	90.34272658	101.0662605	105.9405941	96.67936024	92.04874334	93.02361005	95.70449353	91.07387662	91.3175933	101.0662605	86.93069307

AMP2CPR2ERY3DOX1TMP1	AMP	CPR	ERY	DOX	TMP	AMP+CPR	AMP+ERY	AMP+DOX	AMP+TMP	CPR+ERY	CPR+DOX	CPR+TMP	ERY+DOX	ERY+TMP	DOX+TMP	AMP+CPR+ERY	AMP+CPR+DOX	AMP+CPR+TMP	AMP+ERY+DOX	AMP+ERY+TMP	AMP+DOX+TMP	CPR+ERY+DOX	CPR+ERY+TMP	CPR+DOX+TMP	ERY+DOX+TMP	AMP+CPR+ERY+DOX	AMP+CPR+ERY+TMP	AMP+CPR+DOX+TMP	AMP+ERY+DOX+TMP	CPR+ERY+DOX+TMP	AMP+CPR+ERY+DOX+TMP
	85.67986749	101.5810872	95.82539683	98.24088523	91.88537381	86.88450535	91.70305677	91.70305677	99.17181147	100.1355218	102.5447975	100.3764493	89.98412698	92.26984127	92.79330401	85.67986749	97.96717362	95.31697034	95.79882548	102.3038699	95.55789791	102.785725	103.2675802	102.3038699	94.04761905	97.96717362	104.2312905	95.31697034	94.11233248	97.2443909	89.7756362
	86.46190547	97.67407187	97.32768454	105.1177217	100.1550825	86.7004622	87.89324586	92.6643805	101.4909796	98.62829879	102.2066498	105.7850007	89.24555972	104.8883819	99.25278444	82.8835545	92.90293723	96.48128821	89.32458625	100.7753094	96.71984494	102.4452065	108.1705681	95.76561801	105.4098094	93.38005069	96.48128821	103.6379902	95.28850455	93.85716416	97.9126286
	93.02361005	97.65422696	99.21451583	98.66945732	106.8022126	84.73724296	95.21706017	90.0990099	100.3351104	98.385377	105.6968774	110.0837776	96.45616266	113.282117	107.28061	83.76237624	89.85529322	101.7974105	88.8804265	102.5285605	103.2597106	102.2848439	112.7646611	111.5460777	115.4887996	91.56130998	105.9405941	97.65422696	98.14166032	107.8903275	100.0913938

AMP2CPR2ERY3DOX1TMP2	AMP	CPR	ERY	DOX	TMP	AMP+CPR	AMP+ERY	AMP+DOX	AMP+TMP	CPR+ERY	CPR+DOX	CPR+TMP	ERY+DOX	ERY+TMP	DOX+TMP	AMP+CPR+ERY	AMP+CPR+DOX	AMP+CPR+TMP	AMP+ERY+DOX	AMP+ERY+TMP	AMP+DOX+TMP	CPR+ERY+DOX	CPR+ERY+TMP	CPR+DOX+TMP	ERY+DOX+TMP	AMP+CPR+ERY+DOX	AMP+CPR+ERY+TMP	AMP+CPR+DOX+TMP	AMP+ERY+DOX+TMP	CPR+ERY+DOX+TMP	AMP+CPR+ERY+DOX+TMP
	85.67986749	101.5810872	95.82539683	98.24088523	95.97105972	86.88450535	91.70305677	91.70305677	103.0266526	100.1355218	102.5447975	104.472218	89.98412698	89.73015873	95.51709462	85.67986749	97.96717362	95.07604276	95.79882548	100.6173769	96.03975305	102.785725	106.3996386	101.8220148	96.58730159	97.96717362	98.20810119	94.35326005	97.72624605	103.7494353	91.4621292
	86.46190547	97.67407187	97.32768454	105.1177217	102.1852531	86.7004622	87.89324586	92.6643805	101.0138661	98.62829879	102.2066498	106.7392277	89.24555972	101.7598175	102.8619766	82.8835545	92.90293723	99.58252572	89.32458625	99.58252572	96.95840167	102.4452065	105.3078873	92.90293723	106.1919505	93.38005069	96.00417474	102.6837632	97.9126286	93.85716416	102.6837632
	93.02361005	97.65422696	99.21451583	98.66945732	110.1509942	84.73724296	95.21706017	90.0990099	101.3099772	98.385377	105.6968774	105.9405941	96.45616266	110.2479285	106.3238152	83.76237624	89.85529322	101.5536938	88.8804265	99.84767708	99.84767708	102.2848439	109.3526276	111.302361	115.2129643	91.56130998	78.15689261	93.51104341	99.84767708	110.3274943	99.36024372

AMP2CPR2ERY3DOX1TMP3	AMP	CPR	ERY	DOX	TMP	AMP+CPR	AMP+ERY	AMP+DOX	AMP+TMP	CPR+ERY	CPR+DOX	CPR+TMP	ERY+DOX	ERY+TMP	DOX+TMP	AMP+CPR+ERY	AMP+CPR+DOX	AMP+CPR+TMP	AMP+ERY+DOX	AMP+ERY+TMP	AMP+DOX+TMP	CPR+ERY+DOX	CPR+ERY+TMP	CPR+DOX+TMP	ERY+DOX+TMP	AMP+CPR+ERY+DOX	AMP+CPR+ERY+TMP	AMP+CPR+DOX+TMP	AMP+ERY+DOX+TMP	CPR+ERY+DOX+TMP	AMP+CPR+ERY+DOX+TMP
	85.67986749	101.5810872	95.82539683	98.24088523	98.69485033	86.88450535	91.70305677	91.70305677	95.31697034	100.1355218	102.5447975	103.7494353	89.98412698	95.06349206	100.9646758	85.67986749	97.96717362	88.81192591	95.79882548	99.17181147	95.79882548	102.785725	107.1224213	101.3401596	106.7460317	97.96717362	87.12543292	96.28068062	95.07604276	101.3401596	89.53470863
	86.46190547	97.67407187	97.32768454	105.1177217	98.80163542	86.7004622	87.89324586	92.6643805	97.67407187	98.62829879	102.2066498	101.7295363	89.24555972	102.2812449	105.3432962	82.8835545	92.90293723	95.52706128	89.32458625	97.43551513	90.27881318	102.4452065	102.6837632	100.7753094	109.5812286	93.38005069	93.14149396	100.5367526	89.08602952	97.1969584	95.28850455
	93.02361005	97.65422696	99.21451583	98.66945732	109.1941994	84.73724296	95.21706017	90.0990099	96.67936024	98.385377	105.6968774	106.6717441	96.45616266	109.6962579	109.4333981	83.76237624	89.85529322	92.29246002	88.8804265	89.36785986	96.43564356	102.2848439	106.4280274	107.6466108	114.3854583	91.56130998	91.3175933	73.5262757	95.46077685	111.5460777	94.48591013

AMP2CPR2ERY3DOX2TMP1	AMP	CPR	ERY	DOX	TMP	AMP+CPR	AMP+ERY	AMP+DOX	AMP+TMP	CPR+ERY	CPR+DOX	CPR+TMP	ERY+DOX	ERY+TMP	DOX+TMP	AMP+CPR+ERY	AMP+CPR+DOX	AMP+CPR+TMP	AMP+ERY+DOX	AMP+ERY+TMP	AMP+DOX+TMP	CPR+ERY+DOX	CPR+ERY+TMP	CPR+DOX+TMP	ERY+DOX+TMP	AMP+CPR+ERY+DOX	AMP+CPR+ERY+TMP	AMP+CPR+DOX+TMP	AMP+ERY+DOX+TMP	CPR+ERY+DOX+TMP	AMP+CPR+ERY+DOX+TMP
	85.67986749	101.5810872	95.82539683	99.82976309	91.88537381	86.88450535	91.70305677	95.07604276	99.17181147	100.1355218	100.6173769	100.3764493	86.17460317	92.26984127	97.33295503	85.67986749	97.2443909	95.31697034	94.35326005	102.3038699	94.59418762	101.5810872	103.2675802	102.785725	106.2380952	98.9308839	104.2312905	98.68995633	95.55789791	103.9903629	97.48531848
	86.46190547	97.67407187	97.32768454	103.9898491	100.1550825	86.7004622	87.89324586	93.85716416	101.4909796	98.62829879	102.2066498	105.7850007	102.8026723	104.8883819	98.12491188	82.8835545	96.24273147	96.48128821	92.6643805	100.7753094	85.26912181	103.8765469	108.1705681	100.7753094	110.6240834	96.95840167	96.48128821	101.7295363	91.23304011	103.1608767	100.0596392
	93.02361005	97.65422696	99.21451583	105.1278218	106.8022126	84.73724296	95.21706017	80.59405941	100.3351104	98.385377	94.24219345	110.0837776	99.21451583	113.282117	99.86545074	83.76237624	92.77989337	101.7974105	91.56130998	102.5285605	102.0411272	104.478294	112.7646611	106.4280274	112.1787758	93.26732673	105.9405941	96.19192688	100.8225438	110.3274943	100.8225438

AMP2CPR2ERY3DOX2TMP2	AMP	CPR	ERY	DOX	TMP	AMP+CPR	AMP+ERY	AMP+DOX	AMP+TMP	CPR+ERY	CPR+DOX	CPR+TMP	ERY+DOX	ERY+TMP	DOX+TMP	AMP+CPR+ERY	AMP+CPR+DOX	AMP+CPR+TMP	AMP+ERY+DOX	AMP+ERY+TMP	AMP+DOX+TMP	CPR+ERY+DOX	CPR+ERY+TMP	CPR+DOX+TMP	ERY+DOX+TMP	AMP+CPR+ERY+DOX	AMP+CPR+ERY+TMP	AMP+CPR+DOX+TMP	AMP+ERY+DOX+TMP	CPR+ERY+DOX+TMP	AMP+CPR+ERY+DOX+TMP
	85.67986749	101.5810872	95.82539683	99.82976309	95.97105972	86.88450535	91.70305677	95.07604276	103.0266526	100.1355218	100.6173769	104.472218	86.17460317	89.73015873	100.9646758	85.67986749	97.2443909	95.07604276	94.35326005	100.6173769	98.68995633	101.5810872	106.3996386	98.20810119	108.5238095	98.9308839	98.20810119	103.7494353	93.87140491	102.3038699	98.68995633
	86.46190547	97.67407187	97.32768454	103.9898491	102.1852531	86.7004622	87.89324586	93.85716416	101.0138661	98.62829879	102.2066498	106.7392277	102.8026723	101.7598175	98.80163542	82.8835545	96.24273147	99.58252572	92.6643805	99.58252572	70.71716117	103.8765469	105.3078873	100.7753094	105.9312368	96.95840167	96.00417474	101.7295363	85.26912181	79.54376025	97.1969584
	93.02361005	97.65422696	99.21451583	105.1278218	110.1509942	84.73724296	95.21706017	80.59405941	101.3099772	98.385377	94.24219345	105.9405941	99.21451583	110.2479285	99.14785469	83.76237624	92.77989337	101.5536938	91.56130998	99.84767708	105.209444	104.478294	109.3526276	111.5460777	107.7654107	93.26732673	78.15689261	97.89794364	99.84767708	110.3274943	95.46077685

AMP2CPR2ERY3DOX2TMP3	AMP	CPR	ERY	DOX	TMP	AMP+CPR	AMP+ERY	AMP+DOX	AMP+TMP	CPR+ERY	CPR+DOX	CPR+TMP	ERY+DOX	ERY+TMP	DOX+TMP	AMP+CPR+ERY	AMP+CPR+DOX	AMP+CPR+TMP	AMP+ERY+DOX	AMP+ERY+TMP	AMP+DOX+TMP	CPR+ERY+DOX	CPR+ERY+TMP	CPR+DOX+TMP	ERY+DOX+TMP	AMP+CPR+ERY+DOX	AMP+CPR+ERY+TMP	AMP+CPR+DOX+TMP	AMP+ERY+DOX+TMP	CPR+ERY+DOX+TMP	AMP+CPR+ERY+DOX+TMP
	85.67986749	101.5810872	95.82539683	99.82976309	98.69485033	86.88450535	91.70305677	95.07604276	95.31697034	100.1355218	100.6173769	103.7494353	86.17460317	95.06349206	100.7376933	85.67986749	97.2443909	88.81192591	94.35326005	99.17181147	93.87140491	101.5810872	107.1224213	101.3401596	105.984127	98.9308839	87.12543292	93.38954977	92.90769462	105.4359283	97.2443909
	86.46190547	97.67407187	97.32768454	103.9898491	98.80163542	86.7004622	87.89324586	93.85716416	97.67407187	98.62829879	102.2066498	101.7295363	102.8026723	102.2812449	98.80163542	82.8835545	96.24273147	95.52706128	92.6643805	97.43551513	87.89324586	103.8765469	102.6837632	96.71984494	92.89555157	96.95840167	93.14149396	97.67407187	85.26912181	94.09572089	95.28850455
	93.02361005	97.65422696	99.21451583	105.1278218	109.1941994	84.73724296	95.21706017	80.59405941	96.67936024	98.385377	94.24219345	106.6717441	99.21451583	109.6962579	98.19105995	83.76237624	92.77989337	92.29246002	91.56130998	89.36785986	98.385377	104.478294	106.4280274	104.9657273	101.4211984	93.26732673	91.3175933	88.39299315	97.41051028	109.5963442	92.77989337

AMP2CPR2ERY3DOX3TMP1	AMP	CPR	ERY	DOX	TMP	AMP+CPR	AMP+ERY	AMP+DOX	AMP+TMP	CPR+ERY	CPR+DOX	CPR+TMP	ERY+DOX	ERY+TMP	DOX+TMP	AMP+CPR+ERY	AMP+CPR+DOX	AMP+CPR+TMP	AMP+ERY+DOX	AMP+ERY+TMP	AMP+DOX+TMP	CPR+ERY+DOX	CPR+ERY+TMP	CPR+DOX+TMP	ERY+DOX+TMP	AMP+CPR+ERY+DOX	AMP+CPR+ERY+TMP	AMP+CPR+DOX+TMP	AMP+ERY+DOX+TMP	CPR+ERY+DOX+TMP	AMP+CPR+ERY+DOX+TMP
	85.67986749	101.5810872	95.82539683	104.1424316	91.88537381	86.88450535	91.70305677	93.38954977	99.17181147	100.1355218	98.44902876	100.3764493	90.49206349	92.26984127	104.1424316	85.67986749	89.7756362	95.31697034	83.02966421	102.3038699	99.17181147	98.9308839	103.2675802	103.0266526	108.2698413	97.00346333	104.2312905	93.63047734	91.4621292	99.17181147	100.8583045
	86.46190547	97.67407187	97.32768454	105.5688707	100.1550825	86.7004622	87.89324586	95.04994782	101.4909796	98.62829879	103.3994334	105.7850007	102.0205312	104.8883819	92.25997462	82.8835545	90.75592664	96.48128821	97.9126286	100.7753094	100.2981959	102.4452065	108.1705681	103.6379902	89.50627342	88.60891606	96.48128821	106.9777844	94.57283435	93.85716416	103.1608767
	93.02361005	97.65422696	99.21451583	103.6926297	106.8022126	84.73724296	95.21706017	96.19192688	100.3351104	98.385377	105.9405941	110.0837776	103.6278809	113.282117	93.16788758	83.76237624	95.70449353	101.7974105	90.58644326	102.5285605	102.7722772	104.7220107	112.7646611	110.0837776	106.9379047	95.70449353	105.9405941	102.2848439	59.63442498	108.6214775	74.7448591

AMP2CPR2ERY3DOX3TMP2	AMP	CPR	ERY	DOX	TMP	AMP+CPR	AMP+ERY	AMP+DOX	AMP+TMP	CPR+ERY	CPR+DOX	CPR+TMP	ERY+DOX	ERY+TMP	DOX+TMP	AMP+CPR+ERY	AMP+CPR+DOX	AMP+CPR+TMP	AMP+ERY+DOX	AMP+ERY+TMP	AMP+DOX+TMP	CPR+ERY+DOX	CPR+ERY+TMP	CPR+DOX+TMP	ERY+DOX+TMP	AMP+CPR+ERY+DOX	AMP+CPR+ERY+TMP	AMP+CPR+DOX+TMP	AMP+ERY+DOX+TMP	CPR+ERY+DOX+TMP	AMP+CPR+ERY+DOX+TMP
	85.67986749	101.5810872	95.82539683	104.1424316	95.97105972	86.88450535	91.70305677	93.38954977	103.0266526	100.1355218	98.44902876	104.472218	90.49206349	89.73015873	99.37579799	85.67986749	89.7756362	95.07604276	83.02966421	100.6173769	93.38954977	98.9308839	106.3996386	81.10224364	103.4444444	97.00346333	98.20810119	95.55789791	93.87140491	103.2675802	91.70305677
	86.46190547	97.67407187	97.32768454	105.5688707	102.1852531	86.7004622	87.89324586	95.04994782	101.0138661	98.62829879	103.3994334	106.7392277	102.0205312	101.7598175	88.42520795	82.8835545	90.75592664	99.58252572	97.9126286	99.58252572	95.28850455	102.4452065	105.3078873	96.00417474	85.33485416	88.60891606	96.00417474	105.546444	98.15118533	98.62829879	99.58252572
	93.02361005	97.65422696	99.21451583	103.6926297	110.1509942	84.73724296	95.21706017	96.19192688	101.3099772	98.385377	105.9405941	105.9405941	103.6278809	110.2479285	96.99506653	83.76237624	95.70449353	101.5536938	90.58644326	99.84767708	97.89794364	104.7220107	109.3526276	102.2848439	108.3170813	95.70449353	78.15689261	100.0913938	98.385377	104.7220107	98.14166032

AMP2CPR2ERY3DOX3TMP3	AMP	CPR	ERY	DOX	TMP	AMP+CPR	AMP+ERY	AMP+DOX	AMP+TMP	CPR+ERY	CPR+DOX	CPR+TMP	ERY+DOX	ERY+TMP	DOX+TMP	AMP+CPR+ERY	AMP+CPR+DOX	AMP+CPR+TMP	AMP+ERY+DOX	AMP+ERY+TMP	AMP+DOX+TMP	CPR+ERY+DOX	CPR+ERY+TMP	CPR+DOX+TMP	ERY+DOX+TMP	AMP+CPR+ERY+DOX	AMP+CPR+ERY+TMP	AMP+CPR+DOX+TMP	AMP+ERY+DOX+TMP	CPR+ERY+DOX+TMP	AMP+CPR+ERY+DOX+TMP
	85.67986749	101.5810872	95.82539683	104.1424316	98.69485033	86.88450535	91.70305677	93.38954977	95.31697034	100.1355218	98.44902876	103.7494353	90.49206349	95.06349206	97.55993758	85.67986749	89.7756362	88.81192591	83.02966421	99.17181147	93.38954977	98.9308839	107.1224213	106.6405662	100.9047619	97.00346333	87.12543292	95.07604276	94.35326005	103.0266526	96.28068062
	86.46190547	97.67407187	97.32768454	105.5688707	98.80163542	86.7004622	87.89324586	95.04994782	97.67407187	98.62829879	103.3994334	101.7295363	102.0205312	102.2812449	94.29014521	82.8835545	90.75592664	95.52706128	97.9126286	97.43551513	94.33427762	102.4452065	102.6837632	101.7295363	94.19912009	88.60891606	93.14149396	100.5367526	96.24273147	93.85716416	96.24273147
	93.02361005	97.65422696	99.21451583	103.6926297	109.1941994	84.73724296	95.21706017	96.19192688	96.67936024	98.385377	105.9405941	106.6717441	103.6278809	109.6962579	97.71266258	83.76237624	95.70449353	92.29246002	90.58644326	89.36785986	90.34272658	104.7220107	106.4280274	96.67936024	106.3862341	95.70449353	91.3175933	91.07387662	90.58644326	100.3351104	93.02361005

AMP2CPR3ERY1DOX1TMP1	AMP	CPR	ERY	DOX	TMP	AMP+CPR	AMP+ERY	AMP+DOX	AMP+TMP	CPR+ERY	CPR+DOX	CPR+TMP	ERY+DOX	ERY+TMP	DOX+TMP	AMP+CPR+ERY	AMP+CPR+DOX	AMP+CPR+TMP	AMP+ERY+DOX	AMP+ERY+TMP	AMP+DOX+TMP	CPR+ERY+DOX	CPR+ERY+TMP	CPR+DOX+TMP	ERY+DOX+TMP	AMP+CPR+ERY+DOX	AMP+CPR+ERY+TMP	AMP+CPR+DOX+TMP	AMP+ERY+DOX+TMP	CPR+ERY+DOX+TMP	AMP+CPR+ERY+DOX+TMP
	85.67986749	97.34920635	90.29649596	98.24088523	91.88537381	83.75244692	80.13853335	91.70305677	99.17181147	92.77777778	92.52380952	99.12698413	83.71400199	90.29649596	92.79330401	78.69296793	90.01656377	104.472218	85.67986749	100.8583045	95.55789791	96.84126984	98.11111111	96.33333333	80.99021138	85.92079506	98.20810119	101.8220148	93.63047734	100.3968254	91.70305677
	86.46190547	102.8026723	96.99703933	105.1177217	100.1550825	68.09303713	76.44252274	92.6643805	101.4909796	92.89555157	104.1062408	109.8419423	95.19244325	100.1550825	99.25278444	83.59922469	88.60891606	101.968093	79.78231698	96.48128821	96.71984494	90.02770083	108.2776601	110.8847971	90.45537854	77.15819293	94.57283435	96.00417474	86.93901894	96.54554343	96.95840167
	93.02361005	101.972869	94.12468231	98.66945732	106.8022126	82.05635948	70.60167555	90.0990099	100.3351104	104.4553869	103.0762103	117.4196468	94.363881	102.0182389	107.28061	75.47600914	91.56130998	100.8225438	88.8804265	97.89794364	103.2597106	104.4553869	117.6954821	120.178	96.03827179	75.9634425	93.51104341	98.14166032	91.56130998	116.3163055	87.90555979

AMP2CPR3ERY1DOX1TMP2	AMP	CPR	ERY	DOX	TMP	AMP+CPR	AMP+ERY	AMP+DOX	AMP+TMP	CPR+ERY	CPR+DOX	CPR+TMP	ERY+DOX	ERY+TMP	DOX+TMP	AMP+CPR+ERY	AMP+CPR+DOX	AMP+CPR+TMP	AMP+ERY+DOX	AMP+ERY+TMP	AMP+DOX+TMP	CPR+ERY+DOX	CPR+ERY+TMP	CPR+DOX+TMP	ERY+DOX+TMP	AMP+CPR+ERY+DOX	AMP+CPR+ERY+TMP	AMP+CPR+DOX+TMP	AMP+ERY+DOX+TMP	CPR+ERY+DOX+TMP	AMP+CPR+ERY+DOX+TMP
	85.67986749	97.34920635	90.29649596	98.24088523	95.97105972	83.75244692	80.13853335	91.70305677	103.0266526	92.77777778	92.52380952	96.33333333	83.71400199	88.48063555	95.51709462	78.69296793	90.01656377	106.8814938	85.67986749	98.20810119	96.03975305	96.84126984	90.74603175	99.88888889	95.06312952	85.92079506	93.63047734	82.06595392	95.79882548	107.2539683	81.34317121
	86.46190547	102.8026723	96.99703933	105.1177217	102.1852531	68.09303713	76.44252274	92.6643805	101.0138661	92.89555157	104.1062408	105.9312368	95.19244325	100.6062315	102.8619766	83.59922469	88.60891606	101.4909796	79.78231698	93.61860743	96.95840167	90.02770083	102.2812449	108.5383738	99.02720993	77.15819293	94.09572089	96.71984494	90.27881318	103.8455271	100.0596392
	93.02361005	101.972869	94.12468231	98.66945732	110.1509942	82.05635948	70.60167555	90.0990099	101.3099772	104.4553869	103.0762103	113.8337877	94.363881	103.453431	106.3238152	75.47600914	91.56130998	104.478294	88.8804265	94.97334349	99.84767708	104.4553869	109.4204226	117.6954821	106.8022126	75.9634425	91.80502666	90.0990099	92.04874334	116.5921408	93.26732673

AMP2CPR3ERY1DOX1TMP3	AMP	CPR	ERY	DOX	TMP	AMP+CPR	AMP+ERY	AMP+DOX	AMP+TMP	CPR+ERY	CPR+DOX	CPR+TMP	ERY+DOX	ERY+TMP	DOX+TMP	AMP+CPR+ERY	AMP+CPR+DOX	AMP+CPR+TMP	AMP+ERY+DOX	AMP+ERY+TMP	AMP+DOX+TMP	CPR+ERY+DOX	CPR+ERY+TMP	CPR+DOX+TMP	ERY+DOX+TMP	AMP+CPR+ERY+DOX	AMP+CPR+ERY+TMP	AMP+CPR+DOX+TMP	AMP+ERY+DOX+TMP	CPR+ERY+DOX+TMP	AMP+CPR+ERY+DOX+TMP
	85.67986749	97.34920635	90.29649596	98.24088523	98.69485033	83.75244692	80.13853335	91.70305677	95.31697034	92.77777778	92.52380952	93.03174603	83.71400199	92.11235636	100.9646758	78.69296793	90.01656377	96.28068062	85.67986749	96.03975305	95.79882548	96.84126984	94.3015873	106.7460317	96.65200738	85.92079506	95.79882548	102.5447975	90.01656377	99.63492063	89.53470863
	86.46190547	102.8026723	96.99703933	105.1177217	98.80163542	68.09303713	76.44252274	92.6643805	97.67407187	92.89555157	104.1062408	104.8883819	95.19244325	99.02720993	105.3432962	83.59922469	88.60891606	97.9126286	79.78231698	93.14149396	90.27881318	90.02770083	101.4991038	113.4919342	99.02720993	77.15819293	92.90293723	96.95840167	79.06664679	103.063386	92.42582377
	93.02361005	101.972869	94.12468231	98.66945732	109.1941994	82.05635948	70.60167555	90.0990099	96.67936024	104.4553869	103.0762103	111.9029404	94.363881	105.6062192	109.4333981	75.47600914	91.56130998	92.29246002	88.8804265	90.58644326	96.43564356	104.4553869	106.1103988	116.5921408	108.7158021	75.9634425	87.66184311	90.0990099	82.78750952	113.8337877	85.71210967

AMP2CPR3ERY1DOX2TMP1	AMP	CPR	ERY	DOX	TMP	AMP+CPR	AMP+ERY	AMP+DOX	AMP+TMP	CPR+ERY	CPR+DOX	CPR+TMP	ERY+DOX	ERY+TMP	DOX+TMP	AMP+CPR+ERY	AMP+CPR+DOX	AMP+CPR+TMP	AMP+ERY+DOX	AMP+ERY+TMP	AMP+DOX+TMP	CPR+ERY+DOX	CPR+ERY+TMP	CPR+DOX+TMP	ERY+DOX+TMP	AMP+CPR+ERY+DOX	AMP+CPR+ERY+TMP	AMP+CPR+DOX+TMP	AMP+ERY+DOX+TMP	CPR+ERY+DOX+TMP	AMP+CPR+ERY+DOX+TMP
	85.67986749	97.34920635	90.29649596	99.82976309	91.88537381	83.75244692	80.13853335	95.07604276	99.17181147	92.77777778	91	99.12698413	93.47425167	90.29649596	97.33295503	78.69296793	95.55789791	104.472218	92.66676705	100.8583045	94.59418762	98.11111111	98.11111111	105.2222222	95.97105972	85.92079506	98.20810119	100.8583045	94.59418762	110.047619	96.03975305
	86.46190547	102.8026723	96.99703933	103.9898491	100.1550825	68.09303713	76.44252274	93.85716416	101.4909796	92.89555157	101.7598175	109.8419423	96.99703933	100.1550825	98.12491188	83.59922469	94.09572089	101.968093	90.27881318	96.48128821	85.26912181	95.24197491	108.2776601	115.5776438	96.99703933	82.64499776	94.57283435	103.3994334	95.76561801	102.0205312	98.86685552
	93.02361005	101.972869	94.12468231	105.1278218	106.8022126	82.05635948	70.60167555	80.59405941	100.3351104	104.4553869	101.4211984	117.4196468	96.27747047	102.0182389	99.86545074	75.47600914	95.46077685	100.8225438	82.78750952	97.89794364	102.0411272	108.3170813	117.6954821	117.4196468	100.3438481	72.30769231	93.51104341	93.99847677	93.99847677	116.0404702	96.43564356

AMP2CPR3ERY1DOX2TMP2	AMP	CPR	ERY	DOX	TMP	AMP+CPR	AMP+ERY	AMP+DOX	AMP+TMP	CPR+ERY	CPR+DOX	CPR+TMP	ERY+DOX	ERY+TMP	DOX+TMP	AMP+CPR+ERY	AMP+CPR+DOX	AMP+CPR+TMP	AMP+ERY+DOX	AMP+ERY+TMP	AMP+DOX+TMP	CPR+ERY+DOX	CPR+ERY+TMP	CPR+DOX+TMP	ERY+DOX+TMP	AMP+CPR+ERY+DOX	AMP+CPR+ERY+TMP	AMP+CPR+DOX+TMP	AMP+ERY+DOX+TMP	CPR+ERY+DOX+TMP	AMP+CPR+ERY+DOX+TMP
	85.67986749	97.34920635	90.29649596	99.82976309	95.97105972	83.75244692	80.13853335	95.07604276	103.0266526	92.77777778	91	96.33333333	93.47425167	88.48063555	100.9646758	78.69296793	95.55789791	106.8814938	92.66676705	98.20810119	98.68995633	98.11111111	90.74603175	108.015873	97.55993758	85.92079506	93.63047734	101.8220148	90.98027406	92.52380952	90.73934648
	86.46190547	102.8026723	96.99703933	103.9898491	102.1852531	68.09303713	76.44252274	93.85716416	101.0138661	92.89555157	101.7598175	105.9312368	96.99703933	100.6062315	98.80163542	83.59922469	94.09572089	101.4909796	90.27881318	93.61860743	70.71716117	95.24197491	102.2812449	112.1883657	91.8088256	82.64499776	94.09572089	104.5922171	93.61860743	106.1919505	91.9487103
	93.02361005	101.972869	94.12468231	105.1278218	110.1509942	82.05635948	70.60167555	80.59405941	101.3099772	104.4553869	101.4211984	113.8337877	96.27747047	103.453431	99.14785469	75.47600914	95.46077685	104.478294	82.78750952	94.97334349	105.209444	108.3170813	109.4204226	114.3854583	93.88548363	72.30769231	91.80502666	102.2848439	93.02361005	116.5921408	91.07387662

AMP2CPR3ERY1DOX2TMP3	AMP	CPR	ERY	DOX	TMP	AMP+CPR	AMP+ERY	AMP+DOX	AMP+TMP	CPR+ERY	CPR+DOX	CPR+TMP	ERY+DOX	ERY+TMP	DOX+TMP	AMP+CPR+ERY	AMP+CPR+DOX	AMP+CPR+TMP	AMP+ERY+DOX	AMP+ERY+TMP	AMP+DOX+TMP	CPR+ERY+DOX	CPR+ERY+TMP	CPR+DOX+TMP	ERY+DOX+TMP	AMP+CPR+ERY+DOX	AMP+CPR+ERY+TMP	AMP+CPR+DOX+TMP	AMP+ERY+DOX+TMP	CPR+ERY+DOX+TMP	AMP+CPR+ERY+DOX+TMP
	85.67986749	97.34920635	90.29649596	99.82976309	98.69485033	83.75244692	80.13853335	95.07604276	95.31697034	92.77777778	91	93.03174603	93.47425167	92.11235636	100.7376933	78.69296793	95.55789791	96.28068062	92.66676705	96.03975305	93.87140491	98.11111111	94.3015873	104.2063492	97.55993758	85.92079506	95.79882548	95.55789791	88.81192591	101.9206349	87.12543292
	86.46190547	102.8026723	96.99703933	103.9898491	98.80163542	68.09303713	76.44252274	93.85716416	97.67407187	92.89555157	101.7598175	104.8883819	96.99703933	99.02720993	98.80163542	83.59922469	94.09572089	97.9126286	90.27881318	93.14149396	87.89324586	95.24197491	101.4991038	102.8026723	90.45537854	82.64499776	92.90293723	96.95840167	89.32458625	98.10982565	93.61860743
	93.02361005	101.972869	94.12468231	105.1278218	109.1941994	82.05635948	70.60167555	80.59405941	96.67936024	104.4553869	101.4211984	111.9029404	96.27747047	105.6062192	98.19105995	75.47600914	95.46077685	92.29246002	82.78750952	90.58644326	98.385377	108.3170813	106.1103988	112.1787758	91.73269547	72.30769231	87.66184311	72.30769231	87.66184311	102.2487043	87.66184311

AMP2CPR3ERY1DOX3TMP1	AMP	CPR	ERY	DOX	TMP	AMP+CPR	AMP+ERY	AMP+DOX	AMP+TMP	CPR+ERY	CPR+DOX	CPR+TMP	ERY+DOX	ERY+TMP	DOX+TMP	AMP+CPR+ERY	AMP+CPR+DOX	AMP+CPR+TMP	AMP+ERY+DOX	AMP+ERY+TMP	AMP+DOX+TMP	CPR+ERY+DOX	CPR+ERY+TMP	CPR+DOX+TMP	ERY+DOX+TMP	AMP+CPR+ERY+DOX	AMP+CPR+ERY+TMP	AMP+CPR+DOX+TMP	AMP+ERY+DOX+TMP	CPR+ERY+DOX+TMP	AMP+CPR+ERY+DOX+TMP
	85.67986749	97.34920635	90.29649596	104.1424316	91.88537381	83.75244692	80.13853335	93.38954977	99.17181147	92.77777778	90.23809524	99.12698413	90.75046106	90.29649596	104.1424316	78.69296793	101.099232	104.472218	93.1486222	100.8583045	99.17181147	97.34920635	98.11111111	108.7777778	99.60278054	81.10224364	98.20810119	104.9540732	90.98027406	100.9047619	92.90769462
	86.46190547	102.8026723	96.99703933	105.5688707	100.1550825	68.09303713	76.44252274	95.04994782	101.4909796	92.89555157	98.89196676	109.8419423	97.22261384	100.1550825	92.25997462	83.59922469	99.82108245	101.968093	89.08602952	96.48128821	100.2981959	93.67769268	108.2776601	107.2348053	82.56027069	79.78231698	94.57283435	99.58252572	90.27881318	88.72413231	102.4452065
	93.02361005	101.972869	94.12468231	103.6926297	106.8022126	82.05635948	70.60167555	96.19192688	100.3351104	104.4553869	104.7312222	117.4196468	100.8222455	102.0182389	93.16788758	75.47600914	95.21706017	100.8225438	88.14927647	97.89794364	102.7722772	104.1795516	117.6954821	106.6620694	85.51352968	77.42574257	93.51104341	104.478294	93.99847677	115.7646349	96.43564356

AMP2CPR3ERY1DOX3TMP2	AMP	CPR	ERY	DOX	TMP	AMP+CPR	AMP+ERY	AMP+DOX	AMP+TMP	CPR+ERY	CPR+DOX	CPR+TMP	ERY+DOX	ERY+TMP	DOX+TMP	AMP+CPR+ERY	AMP+CPR+DOX	AMP+CPR+TMP	AMP+ERY+DOX	AMP+ERY+TMP	AMP+DOX+TMP	CPR+ERY+DOX	CPR+ERY+TMP	CPR+DOX+TMP	ERY+DOX+TMP	AMP+CPR+ERY+DOX	AMP+CPR+ERY+TMP	AMP+CPR+DOX+TMP	AMP+ERY+DOX+TMP	CPR+ERY+DOX+TMP	AMP+CPR+ERY+DOX+TMP

	85.67986749	97.34920635	90.29649596	104.1424316	95.97105972	83.75244692	80.13853335	93.38954977	103.0266526	92.77777778	90.23809524	96.33333333	90.75046106	88.48063555	99.37579799	78.69296793	101.099232	106.8814938	93.1486222	98.20810119	93.38954977	97.34920635	90.74603175	101.4126984	99.37579799	81.10224364	93.63047734	94.83511519	93.87140491	93.28571429	92.66676705
	86.46190547	102.8026723	96.99703933	105.5688707	102.1852531	68.09303713	76.44252274	95.04994782	101.0138661	92.89555157	98.89196676	105.9312368	97.22261384	100.6062315	88.42520795	83.59922469	99.82108245	101.4909796	89.08602952	93.61860743	95.28850455	93.67769268	102.2812449	100.1955353	83.23699422	79.78231698	94.09572089	98.15118533	88.84747279	100.1955353	101.968093
	93.02361005	101.972869	94.12468231	103.6926297	110.1509942	82.05635948	70.60167555	96.19192688	101.3099772	104.4553869	104.7312222	113.8337877	100.8222455	103.453431	96.99506653	75.47600914	95.21706017	104.478294	88.14927647	94.97334349	97.89794364	104.1795516	109.4204226	107.21374	88.1447152	77.42574257	91.80502666	103.0159939	91.80502666	112.1787758	90.34272658
AMP2CPR3ERY1DOX3TMP3	AMP	CPR	ERY	DOX	TMP	AMP+CPR	AMP+ERY	AMP+DOX	AMP+TMP	CPR+ERY	CPR+DOX	CPR+TMP	ERY+DOX	ERY+TMP	DOX+TMP	AMP+CPR+ERY	AMP+CPR+DOX	AMP+CPR+TMP	AMP+ERY+DOX	AMP+ERY+TMP	AMP+DOX+TMP	CPR+ERY+DOX	CPR+ERY+TMP	CPR+DOX+TMP	ERY+DOX+TMP	AMP+CPR+ERY+DOX	AMP+CPR+ERY+TMP	AMP+CPR+DOX+TMP	AMP+ERY+DOX+TMP	CPR+ERY+DOX+TMP	AMP+CPR+ERY+DOX+TMP
	85.67986749	97.34920635	90.29649596	104.1424316	98.69485033	83.75244692	80.13853335	93.38954977	95.31697034	92.77777778	90.23809524	93.03174603	90.75046106	92.11235636	97.55993758	78.69296793	101.099232	96.28068062	93.1486222	96.03975305	93.38954977	97.34920635	94.3015873	99.38095238	88.93460065	81.10224364	95.79882548	100.6173769	93.63047734	96.58730159	90.98027406
	86.46190547	102.8026723	96.99703933	105.5688707	98.80163542	68.09303713	76.44252274	95.04994782	97.67407187	92.89555157	98.89196676	104.8883819	97.22261384	99.02720993	94.29014521	83.59922469	99.82108245	97.9126286	89.08602952	93.14149396	94.33427762	93.67769268	101.4991038	97.06697083	82.7858452	79.78231698	92.90293723	91.71015357	84.55345162	101.2383901	84.31489489
	93.02361005	101.972869	94.12468231	103.6926297	109.1941994	82.05635948	70.60167555	96.19192688	96.67936024	104.4553869	104.7312222	111.9029404	100.8222455	105.6062192	97.71266258	75.47600914	95.21706017	92.29246002	88.14927647	90.58644326	90.34272658	104.1795516	106.1103988	103.3520456	88.86231126	77.42574257	87.66184311	94.48591013	92.77989337	105.8345635	69.62680883
AMP2CPR3ERY2DOX1TMP1	AMP	CPR	ERY	DOX	TMP	AMP+CPR	AMP+ERY	AMP+DOX	AMP+TMP	CPR+ERY	CPR+DOX	CPR+TMP	ERY+DOX	ERY+TMP	DOX+TMP	AMP+CPR+ERY	AMP+CPR+DOX	AMP+CPR+TMP	AMP+ERY+DOX	AMP+ERY+TMP	AMP+DOX+TMP	CPR+ERY+DOX	CPR+ERY+TMP	CPR+DOX+TMP	ERY+DOX+TMP	AMP+CPR+ERY+DOX	AMP+CPR+ERY+TMP	AMP+CPR+DOX+TMP	AMP+ERY+DOX+TMP	CPR+ERY+DOX+TMP	AMP+CPR+ERY+DOX+TMP
	85.67986749	97.34920635	95.55789791	98.24088523	91.88537381	83.75244692	87.84821563	91.70305677	99.17181147	94.04761905	92.52380952	99.12698413	96.52160819	97.00346333	92.79330401	80.37946092	90.01656377	104.472218	97.00346333	100.8583045	95.55789791	95.82539683	93.79365079	96.33333333	92.42583948	85.19801235	101.5810872	101.8220148	97.96717362	101.1587302	98.44902876
	86.46190547	102.8026723	99.10541226	105.1177217	100.1550825	68.09303713	86.46190547	92.6643805	101.4909796	95.50268861	104.1062408	109.8419423	97.1969584	99.82108245	99.25278444	84.07633815	88.60891606	101.968093	85.50767854	100.0596392	96.71984494	95.24197491	107.495519	110.8847971	92.6643805	80.97510064	95.04994782	96.00417474	92.42582377	103.5848134	99.58252572
	93.02361005	101.972869	96.19192688	98.66945732	106.8022126	82.05635948	96.19192688	90.0990099	100.3351104	103.3520456	103.0762103	117.4196468	98.385377	107.4028941	107.28061	81.8126428	91.56130998	100.8225438	93.02361005	98.14166032	103.2597106	108.5929166	117.4196468	120.178	102.0411272	84.98095963	101.7974105	98.14166032	95.94821021	118.2471528	97.1667936
AMP2CPR3ERY2DOX1TMP2	AMP	CPR	ERY	DOX	TMP	AMP+CPR	AMP+ERY	AMP+DOX	AMP+TMP	CPR+ERY	CPR+DOX	CPR+TMP	ERY+DOX	ERY+TMP	DOX+TMP	AMP+CPR+ERY	AMP+CPR+DOX	AMP+CPR+TMP	AMP+ERY+DOX	AMP+ERY+TMP	AMP+DOX+TMP	CPR+ERY+DOX	CPR+ERY+TMP	CPR+DOX+TMP	ERY+DOX+TMP	AMP+CPR+ERY+DOX	AMP+CPR+ERY+TMP	AMP+CPR+DOX+TMP	AMP+ERY+DOX+TMP	CPR+ERY+DOX+TMP	AMP+CPR+ERY+DOX+TMP
	85.67986749	97.34920635	95.55789791	98.24088523	95.97105972	83.75244692	87.84821563	91.70305677	103.0266526	94.04761905	92.52380952	96.33333333	96.52160819	96.03975305	95.51709462	80.37946092	90.01656377	106.8814938	97.00346333	104.7131456	96.03975305	95.82539683	94.04761905	99.88888889	97.00346333	85.19801235	103.5085078	82.06595392	95.31697034	98.11111111	89.29378106
	86.46190547	102.8026723	99.10541226	105.1177217	102.1852531	68.09303713	86.46190547	92.6643805	101.0138661	95.50268861	104.1062408	105.9312368	97.1969584	98.62829879	102.8619766	84.07633815	88.60891606	101.4909796	85.50767854	101.0138661	96.95840167	95.24197491	104.1062408	108.5383738	93.14149396	80.97510064	96.71984494	96.71984494	91.47159684	102.0205312	99.34396899
	93.02361005	101.972869	96.19192688	98.66945732	110.1509942	82.05635948	96.19192688	90.0990099	101.3099772	103.3520456	103.0762103	113.8337877	98.385377	103.5034273	106.3238152	81.8126428	91.56130998	104.478294	93.02361005	98.62909368	99.84767708	108.5929166	109.9720932	117.6954821	104.478294	84.98095963	98.62909368	90.0990099	96.19192688	115.4887996	92.04874334
AMP2CPR3ERY2DOX1TMP3	AMP	CPR	ERY	DOX	TMP	AMP+CPR	AMP+ERY	AMP+DOX	AMP+TMP	CPR+ERY	CPR+DOX	CPR+TMP	ERY+DOX	ERY+TMP	DOX+TMP	AMP+CPR+ERY	AMP+CPR+DOX	AMP+CPR+TMP	AMP+ERY+DOX	AMP+ERY+TMP	AMP+DOX+TMP	CPR+ERY+DOX	CPR+ERY+TMP	CPR+DOX+TMP	ERY+DOX+TMP	AMP+CPR+ERY+DOX	AMP+CPR+ERY+TMP	AMP+CPR+DOX+TMP	AMP+ERY+DOX+TMP	CPR+ERY+DOX+TMP	AMP+CPR+ERY+DOX+TMP
	85.67986749	97.34920635	95.55789791	98.24088523	98.69485033	83.75244692	87.84821563	91.70305677	95.31697034	94.04761905	92.52380952	93.03174603	96.52160819	98.44902876	100.9646758	80.37946092	90.01656377	96.28068062	97.00346333	95.31697034	95.79882548	95.82539683	93.53968254	106.7460317	96.76253576	85.19801235	94.83511519	102.5447975	92.66676705	105.984127	95.31697034
	86.46190547	102.8026723	99.10541226	105.1177217	98.80163542	68.09303713	86.46190547	92.6643805	97.67407187	95.50268861	104.1062408	104.8883819	97.1969584	99.34396899	105.3432962	84.07633815	88.60891606	97.9126286	85.50767854	94.57283435	90.27881318	95.24197491	98.89196676	113.4919342	70.24004771	80.97510064	93.61860743	96.95840167	89.32458625	109.3205149	92.18726703
	93.02361005	101.972869	96.19192688	98.66945732	109.1941994	82.05635948	96.19192688	90.0990099	96.67936024	103.3520456	103.0762103	111.9029404	98.385377	97.1667936	109.4333981	81.8126428	91.56130998	92.29246002	93.02361005	90.34272658	96.43564356	108.5929166	106.6620694	116.5921408	101.3099772	84.98095963	93.51104341	90.0990099	91.3175933	111.0754345	86.93069307
AMP2CPR3ERY2DOX2TMP1	AMP	CPR	ERY	DOX	TMP	AMP+CPR	AMP+ERY	AMP+DOX	AMP+TMP	CPR+ERY	CPR+DOX	CPR+TMP	ERY+DOX	ERY+TMP	DOX+TMP	AMP+CPR+ERY	AMP+CPR+DOX	AMP+CPR+TMP	AMP+ERY+DOX	AMP+ERY+TMP	AMP+DOX+TMP	CPR+ERY+DOX	CPR+ERY+TMP	CPR+DOX+TMP	ERY+DOX+TMP	AMP+CPR+ERY+DOX	AMP+CPR+ERY+TMP	AMP+CPR+DOX+TMP	AMP+ERY+DOX+TMP	CPR+ERY+DOX+TMP	AMP+CPR+ERY+DOX+TMP
	85.67986749	97.34920635	95.55789791	99.82976309	91.88537381	83.75244692	87.84821563	95.07604276	99.17181147	94.04761905	91	99.12698413	96.52160819	97.00346333	97.33295503	80.37946092	95.55789791	104.472218	93.63047734	100.8583045	94.59418762	96.84126984	93.79365079	105.2222222	99.65366662	83.02966421	101.5810872	100.8583045	93.38954977	111.5714286	98.68995633
	86.46190547	102.8026723	99.10541226	103.9898491	100.1550825	68.09303713	86.46190547	93.85716416	101.4909796	95.50268861	101.7598175	109.8419423	96.24273147	99.82108245	98.12491188	84.07633815	94.09572089	101.968093	88.60891606	100.0596392	85.26912181	95.76340231	107.495519	115.5776438	103.6379902	82.1678843	95.04994782	103.3994334	92.90293723	111.6669382	96.24273147
	93.02361005	101.972869	96.19192688	105.1278218	106.8022126	82.05635948	96.19192688	80.59405941	100.3351104	103.3520456	101.4211984	117.4196468	99.6039604	107.4028941	99.86545074	81.8126428	95.46077685	100.8225438	91.3175933	98.14166032	102.0411272	109.6962579	117.4196468	117.4196468	106.4280274	82.30007616	101.7974105	93.99847677	65.23990861	119.350494	93.75476009
AMP2CPR3ERY2DOX2TMP2	AMP	CPR	ERY	DOX	TMP	AMP+CPR	AMP+ERY	AMP+DOX	AMP+TMP	CPR+ERY	CPR+DOX	CPR+TMP	ERY+DOX	ERY+TMP	DOX+TMP	AMP+CPR+ERY	AMP+CPR+DOX	AMP+CPR+TMP	AMP+ERY+DOX	AMP+ERY+TMP	AMP+DOX+TMP	CPR+ERY+DOX	CPR+ERY+TMP	CPR+DOX+TMP	ERY+DOX+TMP	AMP+CPR+ERY+DOX	AMP+CPR+ERY+TMP	AMP+CPR+DOX+TMP	AMP+ERY+DOX+TMP	CPR+ERY+DOX+TMP	AMP+CPR+ERY+DOX+TMP
	85.67986749	97.34920635	95.55789791	99.82976309	95.97105972	83.75244692	87.84821563	95.07604276	103.0266526	94.04761905	91	96.33333333	96.52160819	96.03975305	100.9646758	80.37946092	95.55789791	106.8814938	93.63047734	104.7131456	98.68995633	96.84126984	94.04761905	108.015873	100.1355218	83.02966421	103.5085078	101.8220148	94.59418762	114.3650794	99.41273905
	86.46190547	102.8026723	99.10541226	103.9898491	102.1852531	68.09303713	86.46190547	93.85716416	101.0138661	95.50268861	101.7598175	105.9312368	96.24273147	98.62829879	98.80163542	84.07633815	94.09572089	101.4909796	88.60891606	101.0138661	70.71716117	95.76340231	104.1062408	112.1883657	97.1969584	82.1678843	96.71984494	104.5922171	92.42582377	113.2312205	98.86685552
	93.02361005	101.972869	96.19192688	105.1278218	110.1509942	82.05635948	96.19192688	80.59405941	101.3099772	103.3520456	101.4211984	113.8337877	99.6039604	103.5034273	99.14785469	81.8126428	95.46077685	104.478294	91.3175933	98.62909368	105.209444	109.6962579	109.9720932	114.3854583	103.2597106	82.30007616	98.62909368	102.2848439	99.36024372	117.1438115	96.43564356
AMP2CPR3ERY2DOX2TMP3	AMP	CPR	ERY	DOX	TMP	AMP+CPR	AMP+ERY	AMP+DOX	AMP+TMP	CPR+ERY	CPR+DOX	CPR+TMP	ERY+DOX	ERY+TMP	DOX+TMP	AMP+CPR+ERY	AMP+CPR+DOX	AMP+CPR+TMP	AMP+ERY+DOX	AMP+ERY+TMP	AMP+DOX+TMP	CPR+ERY+DOX	CPR+ERY+TMP	CPR+DOX+TMP	ERY+DOX+TMP	AMP+CPR+ERY+DOX	AMP+CPR+ERY+TMP	AMP+CPR+DOX+TMP	AMP+ERY+DOX+TMP	CPR+ERY+DOX+TMP	AMP+CPR+ERY+DOX+TMP
	85.67986749	97.34920635	95.55789791	99.82976309	98.69485033	83.75244692	87.84821563	95.07604276	95.31697034	94.04761905	91	93.03174603	96.52160819	98.44902876	100.7376933	80.37946092	95.55789791	96.28068062	93.63047734	95.31697034	93.87140491	96.84126984	93.53968254	104.2063492	101.099232	83.02966421	94.83511519	95.55789791	91.70305677	109.031746	91.70305677
	86.46190547	102.8026723	99.10541226	103.9898491	98.80163542	68.09303713	86.46190547	93.85716416	97.67407187	95.50268861	101.7598175	104.8883819	96.24273147	99.34396899	98.80163542	84.07633815	94.09572089	97.9126286	88.60891606	94.57283435	87.89324586	95.76340231	98.89196676	102.8026723	92.42582377	82.1678843	93.61860743	96.95840167	88.13180259	98.37053935	87.89324586
	93.02361005	101.972869	96.19192688	105.1278218	109.1941994	82.05635948	96.19192688	80.59405941	96.67936024	103.3520456	101.4211984	111.9029404	99.6039604	97.1667936	98.19105995	81.8126428	95.46077685	92.29246002	91.3175933	90.34272658	98.385377	109.6962579	106.6620694	112.1787758	99.6039604	82.30007616	93.51104341	72.30769231	93.02361005	110.2479285	91.07387662
AMP2CPR3ERY2DOX3TMP1	AMP	CPR	ERY	DOX	TMP	AMP+CPR	AMP+ERY	AMP+DOX	AMP+TMP	CPR+ERY	CPR+DOX	CPR+TMP	ERY+DOX	ERY+TMP	DOX+TMP	AMP+CPR+ERY	AMP+CPR+DOX	AMP+CPR+TMP	AMP+ERY+DOX	AMP+ERY+TMP	AMP+DOX+TMP	CPR+ERY+DOX	CPR+ERY+TMP	CPR+DOX+TMP	ERY+DOX+TMP	AMP+CPR+ERY+DOX	AMP+CPR+ERY+TMP	AMP+CPR+DOX+TMP	AMP+ERY+DOX+TMP	CPR+ERY+DOX+TMP	AMP+CPR+ERY+DOX+TMP
	85.67986749	97.34920635	95.55789791	104.1424316	91.88537381	83.75244692	87.84821563	93.38954977	99.17181147	94.04761905	90.23809524	99.12698413	94.83511519	97.00346333	104.1424316	80.37946092	101.099232	104.472218	96.28068062	100.8583045	99.17181147	91.50793651	93.79365079	108.7777778	103.7494353	92.90769462	101.5810872	104.9540732	93.63047734	104.7142857	100.8583045
	86.46190547	102.8026723	99.10541226	105.5688707	100.1550825	68.09303713	86.46190547	95.04994782	101.4909796	95.50268861	98.89196676	109.8419423	98.62829879	99.82108245	92.25997462	84.07633815	99.82108245	101.968093	93.85716416	100.0596392	100.2981959	97.06697083	107.495519	107.2348053	93.85716416	91.71015357	95.04994782	99.58252572	94.81139108	107.2348053	97.9126286
	93.02361005	101.972869	96.19192688	103.6926297	106.8022126	82.05635948	96.19192688	96.19192688	100.3351104	103.3520456	104.7312222	117.4196468	101.7974105	107.4028941	93.16788758	81.8126428	95.21706017	100.8225438	78.15689261	98.14166032	102.7722772	102.2487043	117.4196468	106.6620694	99.6039604	89.12414318	101.7974105	104.478294	101.3099772	124.5913651	99.6039604
AMP2CPR3ERY2DOX3TMP2	AMP	CPR	ERY	DOX	TMP	AMP+CPR	AMP+ERY	AMP+DOX	AMP+TMP	CPR+ERY	CPR+DOX	CPR+TMP	ERY+DOX	ERY+TMP	DOX+TMP	AMP+CPR+ERY	AMP+CPR+DOX	AMP+CPR+TMP	AMP+ERY+DOX	AMP+ERY+TMP	AMP+DOX+TMP	CPR+ERY+DOX	CPR+ERY+TMP	CPR+DOX+TMP	ERY+DOX+TMP	AMP+CPR+ERY+DOX	AMP+CPR+ERY+TMP	AMP+CPR+DOX+TMP	AMP+ERY+DOX+TMP	CPR+ERY+DOX+TMP	AMP+CPR+ERY+DOX+TMP
	85.67986749	97.34920635	95.55789791	104.1424316	95.97105972	83.75244692	87.84821563	93.38954977	103.0266526	94.04761905	90.23809524	96.33333333	94.83511519	96.03975305	99.37579799	80.37946092	101.099232	106.8814938	96.28068062	104.7131456	93.38954977	91.50793651	94.04761905	101.4126984	104.2312905	92.90769462	103.5085078	94.83511519	93.87140491	96.33333333	96.76253576
	86.46190547	102.8026723	99.10541226	105.5688707	102.1852531	68.09303713	86.46190547	95.04994782	101.0138661	95.50268861	98.89196676	105.9312368	98.62829879	98.62829879	88.42520795	84.07633815	99.82108245	101.4909796	93.85716416	101.0138661	95.28850455	97.06697083	104.1062408	100.1955353	97.1969584	91.71015357	96.71984494	98.15118533	94.33427762	101.2383901	103.1608767
	93.02361005	101.972869	96.19192688	103.6926297	110.1509942	82.05635948	96.19192688	96.19192688	101.3099772	103.3520456	104.7312222	113.8337877	101.7974105	103.5034273	96.99506653	81.8126428	95.21706017	104.478294	78.15689261	98.62909368	97.89794364	102.2487043	109.9720932	107.21374	88.8804265	89.12414318	98.62909368	103.0159939	95.21706017	113.282117	91.56130998
AMP2CPR3ERY2DOX3TMP3	AMP	CPR	ERY	DOX	TMP	AMP+CPR	AMP+ERY	AMP+DOX	AMP+TMP	CPR+ERY	CPR+DOX	CPR+TMP	ERY+DOX	ERY+TMP	DOX+TMP	AMP+CPR+ERY	AMP+CPR+DOX	AMP+CPR+TMP	AMP+ERY+DOX	AMP+ERY+TMP	AMP+DOX+TMP	CPR+ERY+DOX	CPR+ERY+TMP	CPR+DOX+TMP	ERY+DOX+TMP	AMP+CPR+ERY+DOX	AMP+CPR+ERY+TMP	AMP+CPR+DOX+TMP	AMP+ERY+DOX+TMP	CPR+ERY+DOX+TMP	AMP+CPR+ERY+DOX+TMP
	85.67986749	97.34920635	95.55789791	104.1424316	98.69485033	83.75244692	87.84821563	93.38954977	95.31697034	94.04761905	90.23809524	93.03174603	94.83511519	98.44902876	97.55993758	80.37946092	101.099232	96.28068062	96.28068062	95.31697034	93.38954977	91.50793651	93.53968254	99.38095238	103.9903629	92.90769462	94.83511519	100.6173769	92.18491191	93.79365079	94.35326005
	86.46190547	102.8026723	99.10541226	105.5688707	98.80163542	68.09303713	86.46190547	95.04994782	97.67407187	95.50268861	98.89196676	104.8883819	98.62829879	99.34396899	94.29014521	84.07633815	99.82108245	97.9126286	93.85716416	94.57283435	94.33427762	97.06697083	98.89196676	97.06697083	93.85716416	91.71015357	93.61860743	91.71015357	90.99448338	97.58839824	101.4909796
	93.02361005	101.972869	96.19192688	103.6926297	109.1941994	82.05635948	96.19192688	96.19192688	96.67936024	103.3520456	104.7312222	111.9029404	101.7974105	97.1667936	97.71266258	81.8126428	95.21706017	92.29246002	78.15689261	90.34272658	90.34272658	102.2487043	106.6620694	103.3520456	92.04874334	89.12414318	93.51104341	94.48591013	91.3175933	106.6620694	82.54379284
AMP2CPR3ERY3DOX1TMP1	AMP	CPR	ERY	DOX	TMP	AMP+CPR	AMP+ERY	AMP+DOX	AMP+TMP	CPR+ERY	CPR+DOX	CPR+TMP	ERY+DOX	ERY+TMP	DOX+TMP	AMP+CPR+ERY	AMP+CPR+DOX	AMP+CPR+TMP	AMP+ERY+DOX	AMP+ERY+TMP	AMP+DOX+TMP	CPR+ERY+DOX	CPR+ERY+TMP	CPR+DOX+TMP	ERY+DOX+TMP	AMP+CPR+ERY+DOX	AMP+CPR+ERY+TMP	AMP+CPR+DOX+TMP	AMP+ERY+DOX+TMP	CPR+ERY+DOX+TMP	AMP+CPR+ERY+DOX+TMP
	85.67986749	97.34920635	95.82539683	98.24088523	91.88537381	83.75244692	91.70305677	91.70305677	99.17181147	96.84126984	92.52380952	99.12698413	89.98412698	92.26984127	92.79330401	82.06595392	90.01656377	104.472218	95.79882548	102.3038699	95.55789791	88.20634921	94.55555556	96.33333333	94.04761905	85.19801235	105.4359283	101.8220148	94.11233248	95.06349206	96.52160819
	86.46190547	102.8026723	97.32768454	105.1177217	100.1550825	68.09303713	87.89324586	92.6643805	101.4909796	98.63125306	104.1062408	109.8419423	89.24555972	104.8883819	99.25278444	84.55345162	88.60891606	101.968093	89.32458625	100.7753094	96.71984494	94.7205475	110.102656	110.8847971	105.4098094	85.26912181	99.82108245	96.00417474	95.28850455	105.4098094	79.06664679
	93.02361005	101.972869	99.21451583	98.66945732	106.8022126	82.05635948	95.21706017	90.0990099	100.3351104	106.9379047	103.0762103	117.4196468	96.45616266	113.282117	107.28061	89.61157654	91.56130998	100.8225438	88.8804265	102.5285605	103.2597106	107.21374	116.3163055	120.178	115.4887996	85.71210967	103.2597106	98.14166032	98.14166032	118.7988234	97.65422696
AMP2CPR3ERY3DOX1TMP2	AMP	CPR	ERY	DOX	TMP	AMP+CPR	AMP+ERY	AMP+DOX	AMP+TMP	CPR+ERY	CPR+DOX	CPR+TMP	ERY+DOX	ERY+TMP	DOX+TMP	AMP+CPR+ERY	AMP+CPR+DOX	AMP+CPR+TMP	AMP+ERY+DOX	AMP+ERY+TMP	AMP+DOX+TMP	CPR+ERY+DOX	CPR+ERY+TMP	CPR+DOX+TMP	ERY+DOX+TMP	AMP+CPR+ERY+DOX	AMP+CPR+ERY+TMP	AMP+CPR+DOX+TMP	AMP+ERY+DOX+TMP	CPR+ERY+DOX+TMP	AMP+CPR+ERY+DOX+TMP
	85.67986749	97.34920635	95.82539683	98.24088523	95.97105972	83.75244692	91.70305677	91.70305677	103.0266526	96.84126984	92.52380952	96.33333333	89.98412698	89.73015873	95.51709462	82.06595392	90.01656377	106.8814938	95.79882548	100.6173769	96.03975305	88.20634921	96.58730159	99.88888889	96.58730159	85.19801235	101.8220148	82.06595392	97.72624605	103.952381	99.41273905
	86.46190547	102.8026723	97.32768454	105.1177217	102.1852531	68.09303713	87.89324586	92.6643805	101.0138661	98.63125306	104.1062408	105.9312368	89.24555972	101.7598175	102.8619766	84.55345162	88.60891606	101.4909796	89.32458625	99.58252572	96.95840167	94.7205475	94.4598338	108.5383738	106.1919505	85.26912181	97.43551513	96.71984494	97.9126286	109.0598012	97.9126286
	93.02361005	101.972869	99.21451583	98.66945732	110.1509942	82.05635948	95.21706017	90.0990099	101.3099772	106.9379047	103.0762103	113.8337877	96.45616266	110.2479285	106.3238152	89.61157654	91.56130998	104.478294	88.8804265	99.84767708	99.84767708	107.21374	112.4546111	117.6954821	115.2129643	85.71210967	101.5536938	90.0990099	99.84767708	119.0746587	74.50114242
AMP2CPR3ERY3DOX1TMP3	AMP	CPR	ERY	DOX	TMP	AMP+CPR	AMP+ERY	AMP+DOX	AMP+TMP	CPR+ERY	CPR+DOX	CPR+TMP	ERY+DOX	ERY+TMP	DOX+TMP	AMP+CPR+ERY	AMP+CPR+DOX	AMP+CPR+TMP	AMP+ERY+DOX	AMP+ERY+TMP	AMP+DOX+TMP	CPR+ERY+DOX	CPR+ERY+TMP	CPR+DOX+TMP	ERY+DOX+TMP	AMP+CPR+ERY+DOX	AMP+CPR+ERY+TMP	AMP+CPR+DOX+TMP	AMP+ERY+DOX+TMP	CPR+ERY+DOX+TMP	AMP+CPR+ERY+DOX+TMP
	85.67986749	97.34920635	95.82539683	98.24088523	98.69485033	83.75244692	91.70305677	91.70305677	95.31697034	96.84126984	92.52380952	93.03174603	89.98412698	95.06349206	100.9646758	82.06595392	90.01656377	96.28068062	95.79882548	99.17181147	95.79882548	88.20634921	93.28571429	106.7460317	106.7460317	85.19801235	98.20810119	102.5447975	95.07604276	105.4761905	95.55789791
	86.46190547	102.8026723	97.32768454	105.1177217	98.80163542	68.09303713	87.89324586	92.6643805	97.67407187	98.63125306	104.1062408	104.8883819	89.24555972	102.2812449	105.3432962	84.55345162	88.60891606	97.9126286	89.32458625	97.43551513	90.27881318	94.7205475	101.2383901	113.4919342	109.5812286	85.26912181	94.57283435	96.95840167	89.08602952	108.0169464	79.06664679
	93.02361005	101.972869	99.21451583	98.66945732	109.1941994	82.05635948	95.21706017	90.0990099	96.67936024	106.9379047	103.0762103	111.9029404	96.45616266	109.6962579	109.4333981	89.61157654	91.56130998	92.29246002	88.8804265	89.36785986	96.43564356	107.21374	107.7654107	116.5921408	114.3854583	85.71210967	52.07920792	90.0990099	95.46077685	112.1787758	88.14927647
AMP2CPR3ERY3DOX2TMP1	AMP	CPR	ERY	DOX	TMP	AMP+CPR	AMP+ERY	AMP+DOX	AMP+TMP	CPR+ERY	CPR+DOX	CPR+TMP	ERY+DOX	ERY+TMP	DOX+TMP	AMP+CPR+ERY	AMP+CPR+DOX	AMP+CPR+TMP	AMP+ERY+DOX	AMP+ERY+TMP	AMP+DOX+TMP	CPR+ERY+DOX	CPR+ERY+TMP	CPR+DOX+TMP	ERY+DOX+TMP	AMP+CPR+ERY+DOX	AMP+CPR+ERY+TMP	AMP+CPR+DOX+TMP	AMP+ERY+DOX+TMP	CPR+ERY+DOX+TMP	AMP+CPR+ERY+DOX+TMP
	85.67986749	97.34920635	95.82539683	99.82976309	91.88537381	83.75244692	91.70305677	95.07604276	99.17181147	96.84126984	91	99.12698413	86.17460317	92.26984127	97.33295503	82.06595392	95.55789791	104.472218	94.35326005	102.3038699	94.59418762	90.74603175	94.55555556	105.2222222	106.2380952	95.07604276	105.4359283	100.8583045	95.55789791	115.3809524	99.65366662
	86.46190547	102.8026723	97.32768454	103.9898491	100.1550825	68.09303713	87.89324586	93.85716416	101.4909796	98.63125306	101.7598175	109.8419423	102.8026723	104.8883819	98.12491188	84.55345162	94.09572089	101.968093	92.6643805	100.7753094	85.26912181	100.1955353	110.102656	115.5776438	110.6240834	93.85716416	99.82108245	103.3994334	91.23304011	117.4026397	67.85448039
	93.02361005	101.972869	99.21451583	105.1278218	106.8022126	82.05635948	95.21706017	80.59405941	100.3351104	106.9379047	101.4211984	117.4196468	99.21451583	113.282117	99.86545074	89.61157654	95.46077685	100.8225438	91.56130998	102.5285605	102.0411272	104.7312222	116.3163055	117.4196468	112.1787758	92.04874334	103.2597106	93.99847677	100.8225438	121.2813412	81.08149276
AMP2CPR3ERY3DOX2TMP2	AMP	CPR	ERY	DOX	TMP	AMP+CPR	AMP+ERY	AMP+DOX	AMP+TMP	CPR+ERY	CPR+DOX	CPR+TMP	ERY+DOX	ERY+TMP	DOX+TMP	AMP+CPR+ERY	AMP+CPR+DOX	AMP+CPR+TMP	AMP+ERY+DOX	AMP+ERY+TMP	AMP+DOX+TMP	CPR+ERY+DOX	CPR+ERY+TMP	CPR+DOX+TMP	ERY+DOX+TMP	AMP+CPR+ERY+DOX	AMP+CPR+ERY+TMP	AMP+CPR+DOX+TMP	AMP+ERY+DOX+TMP	CPR+ERY+DOX+TMP	AMP+CPR+ERY+DOX+TMP
	85.67986749	97.34920635	95.82539683	99.82976309	95.97105972	83.75244692	91.70305677	95.07604276	103.0266526	96.84126984	91	96.33333333	86.17460317	89.73015873	100.9646758	82.06595392	95.55789791	106.8814938	94.35326005	100.6173769	98.68995633	90.74603175	96.58730159	108.015873	108.5238095	95.07604276	101.8220148	101.8220148	93.87140491	111.5714286	96.52160819
	86.46190547	102.8026723	97.32768454	103.9898491	102.1852531	68.09303713	87.89324586	93.85716416	101.0138661	98.63125306	101.7598175	105.9312368	102.8026723	101.7598175	98.80163542	84.55345162	94.09572089	101.4909796	92.6643805	99.58252572	70.71716117	100.1955353	94.4598338	112.1883657	105.9312368	93.85716416	97.43551513	104.5922171	85.26912181	102.2812449	104.5922171
	93.02361005	101.972869	99.21451583	105.1278218	110.1509942	82.05635948	95.21706017	80.59405941	101.3099772	106.9379047	101.4211984	113.8337877	99.21451583	110.2479285	99.14785469	89.61157654	95.46077685	104.478294	91.56130998	99.84767708	105.209444	104.7312222	112.4546111	114.3854583	107.7654107	92.04874334	101.5536938	102.2848439	99.84767708	114.109623	98.385377
AMP2CPR3ERY3DOX2TMP3	AMP	CPR	ERY	DOX	TMP	AMP+CPR	AMP+ERY	AMP+DOX	AMP+TMP	CPR+ERY	CPR+DOX	CPR+TMP	ERY+DOX	ERY+TMP	DOX+TMP	AMP+CPR+ERY	AMP+CPR+DOX	AMP+CPR+TMP	AMP+ERY+DOX	AMP+ERY+TMP	AMP+DOX+TMP	CPR+ERY+DOX	CPR+ERY+TMP	CPR+DOX+TMP	ERY+DOX+TMP	AMP+CPR+ERY+DOX	AMP+CPR+ERY+TMP	AMP+CPR+DOX+TMP	AMP+ERY+DOX+TMP	CPR+ERY+DOX+TMP	AMP+CPR+ERY+DOX+TMP
	85.67986749	97.34920635	95.82539683	99.82976309	98.69485033	83.75244692	91.70305677	95.07604276	95.31697034	96.84126984	91	93.03174603	86.17460317	95.06349206	100.7376933	82.06595392	95.55789791	96.28068062	94.35326005	99.17181147	93.87140491	90.74603175	93.28571429	104.2063492	105.984127	95.07604276	98.20810119	95.55789791	92.90769462	107.5079365	97.00346333
	86.46190547	102.8026723	97.32768454	103.9898491	98.80163542	68.09303713	87.89324586	93.85716416	97.67407187	98.63125306	101.7598175	104.8883819	102.8026723	102.2812449	98.80163542	84.55345162	94.09572089	97.9126286	92.6643805	97.43551513	87.89324586	100.1955353	101.2383901	102.8026723	92.89555157	93.85716416	94.57283435	96.95840167	85.26912181	105.4098094	95.04994782
	93.02361005	101.972869	99.21451583	105.1278218	109.1941994	82.05635948	95.21706017	80.59405941	96.67936024	106.9379047	101.4211984	111.9029404	99.21451583	109.6962579	98.19105995	89.61157654	95.46077685	92.29246002	91.56130998	89.36785986	98.385377	104.7312222	107.7654107	112.1787758	101.4211984	92.04874334	52.07920792	72.30769231	97.41051028	116.0404702	94.72962681
AMP2CPR3ERY3DOX3TMP1	AMP	CPR	ERY	DOX	TMP	AMP+CPR	AMP+ERY	AMP+DOX	AMP+TMP	CPR+ERY	CPR+DOX	CPR+TMP	ERY+DOX	ERY+TMP	DOX+TMP	AMP+CPR+ERY	AMP+CPR+DOX	AMP+CPR+TMP	AMP+ERY+DOX	AMP+ERY+TMP	AMP+DOX+TMP	CPR+ERY+DOX	CPR+ERY+TMP	CPR+DOX+TMP	ERY+DOX+TMP	AMP+CPR+ERY+DOX	AMP+CPR+ERY+TMP	AMP+CPR+DOX+TMP	AMP+ERY+DOX+TMP	CPR+ERY+DOX+TMP	AMP+CPR+ERY+DOX+TMP
	85.67986749	97.34920635	95.82539683	104.1424316	91.88537381	83.75244692	91.70305677	93.38954977	99.17181147	96.84126984	90.23809524	99.12698413	90.49206349	92.26984127	104.1424316	82.06595392	101.099232	104.472218	83.02966421	102.3038699	99.17181147	92.52380952	94.55555556	108.7777778	108.2698413	98.9308839	105.4359283	104.9540732	91.4621292	103.952381	103.0266526
	86.46190547	102.8026723	97.32768454	105.5688707	100.1550825	68.09303713	87.89324586	95.04994782	101.4909796	98.63125306	98.89196676	109.8419423	102.0205312	104.8883819	92.25997462	84.55345162	99.82108245	101.968093	97.9126286	100.7753094	100.2981959	101.4991038	110.102656	107.2348053	89.50627342	91.71015357	99.82108245	99.58252572	94.57283435	105.1490956	97.43551513
	93.02361005	101.972869	99.21451583	103.6926297	106.8022126	82.05635948	95.21706017	96.19192688	100.3351104	106.9379047	104.7312222	117.4196468	103.6278809	113.282117	93.16788758	89.61157654	95.21706017	100.8225438	90.58644326	102.5285605	102.7722772	105.0070575	116.3163055	106.6620694	106.9379047	91.3175933	103.2597106	104.478294	59.63442498	119.9021647	99.11652704
AMP2CPR3ERY3DOX3TMP2	AMP	CPR	ERY	DOX	TMP	AMP+CPR	AMP+ERY	AMP+DOX	AMP+TMP	CPR+ERY	CPR+DOX	CPR+TMP	ERY+DOX	ERY+TMP	DOX+TMP	AMP+CPR+ERY	AMP+CPR+DOX	AMP+CPR+TMP	AMP+ERY+DOX	AMP+ERY+TMP	AMP+DOX+TMP	CPR+ERY+DOX	CPR+ERY+TMP	CPR+DOX+TMP	ERY+DOX+TMP	AMP+CPR+ERY+DOX	AMP+CPR+ERY+TMP	AMP+CPR+DOX+TMP	AMP+ERY+DOX+TMP	CPR+ERY+DOX+TMP	AMP+CPR+ERY+DOX+TMP
	85.67986749	97.34920635	95.82539683	104.1424316	95.97105972	83.75244692	91.70305677	93.38954977	103.0266526	96.84126984	90.23809524	96.33333333	90.49206349	89.73015873	99.37579799	82.06595392	101.099232	106.8814938	83.02966421	100.6173769	93.38954977	92.52380952	96.58730159	101.4126984	103.4444444	98.9308839	101.8220148	94.83511519	93.87140491	103.4444444	104.2312905
	86.46190547	102.8026723	97.32768454	105.5688707	102.1852531	68.09303713	87.89324586	95.04994782	101.0138661	98.63125306	98.89196676	105.9312368	102.0205312	101.7598175	88.42520795	84.55345162	99.82108245	101.4909796	97.9126286	99.58252572	95.28850455	101.4991038	94.4598338	100.1955353	85.33485416	91.71015357	97.43551513	98.15118533	98.15118533	102.5419586	99.58252572
	93.02361005	101.972869	99.21451583	103.6926297	110.1509942	82.05635948	95.21706017	96.19192688	101.3099772	106.9379047	104.7312222	113.8337877	103.6278809	110.2479285	96.99506653	89.61157654	95.21706017	104.478294	90.58644326	99.84767708	97.89794364	105.0070575	112.4546111	107.21374	108.3170813	91.3175933	101.5536938	103.0159939	98.385377	108.868752	95.94821021
AMP2CPR3ERY3DOX3TMP3	AMP	CPR	ERY	DOX	TMP	AMP+CPR	AMP+ERY	AMP+DOX	AMP+TMP	CPR+ERY	CPR+DOX	CPR+TMP	ERY+DOX	ERY+TMP	DOX+TMP	AMP+CPR+ERY	AMP+CPR+DOX	AMP+CPR+TMP	AMP+ERY+DOX	AMP+ERY+TMP	AMP+DOX+TMP	CPR+ERY+DOX	CPR+ERY+TMP	CPR+DOX+TMP	ERY+DOX+TMP	AMP+CPR+ERY+DOX	AMP+CPR+ERY+TMP	AMP+CPR+DOX+TMP	AMP+ERY+DOX+TMP	CPR+ERY+DOX+TMP	AMP+CPR+ERY+DOX+TMP
	85.67986749	97.34920635	95.82539683	104.1424316	98.69485033	83.75244692	91.70305677	93.38954977	95.31697034	96.84126984	90.23809524	93.03174603	90.49206349	95.06349206	97.55993758	82.06595392	101.099232	96.28068062	83.02966421	99.17181147	93.38954977	92.52380952	93.28571429	99.38095238	100.9047619	98.9308839	98.20810119	100.6173769	94.35326005	101.4126984	97.48531848
	86.46190547	102.8026723	97.32768454	105.5688707	98.80163542	68.09303713	87.89324586	95.04994782	97.67407187	98.63125306	98.89196676	104.8883819	102.0205312	102.2812449	94.29014521	84.55345162	99.82108245	97.9126286	97.9126286	97.43551513	94.33427762	101.4991038	101.2383901	97.06697083	94.19912009	91.71015357	94.57283435	91.71015357	96.24273147	101.7598175	97.1969584
	93.02361005	101.972869	99.21451583	103.6926297	109.1941994	82.05635948	95.21706017	96.19192688	96.67936024	106.9379047	104.7312222	111.9029404	103.6278809	109.6962579	97.71266258	89.61157654	95.21706017	92.29246002	90.58644326	89.36785986	90.34272658	105.0070575	107.7654107	103.3520456	106.3862341	91.3175933	52.07920792	94.48591013	90.58644326	108.5929166	90.83015994
AMP3CPR1ERY1DOX1TMP1	AMP	CPR	ERY	DOX	TMP	AMP+CPR	AMP+ERY	AMP+DOX	AMP+TMP	CPR+ERY	CPR+DOX	CPR+TMP	ERY+DOX	ERY+TMP	DOX+TMP	AMP+CPR+ERY	AMP+CPR+DOX	AMP+CPR+TMP	AMP+ERY+DOX	AMP+ERY+TMP	AMP+DOX+TMP	CPR+ERY+DOX	CPR+ERY+TMP	CPR+DOX+TMP	ERY+DOX+TMP	AMP+CPR+ERY+DOX	AMP+CPR+ERY+TMP	AMP+CPR+DOX+TMP	AMP+ERY+DOX+TMP	CPR+ERY+DOX+TMP	AMP+CPR+ERY+DOX+TMP
	96.07936508	91.65839126	90.29649596	98.24088523	91.88537381	91.25396825	91.25396825	94.04761905	102.4285714	100.9646758	101.4186409	88.93460065	83.71400199	90.29649596	92.79330401	84.14285714	93.28571429	108.2698413	86.42857143	102.9365079	104.2063492	97.55993758	90.97744361	95.51709462	80.99021138	77.28571429	105.4761905	105.984127	105.7301587	85.07589729	93.79365079
	94.7205475	101.5085295	96.99703933	105.1177217	100.1550825	90.80984194	91.07055565	93.93840639	102.2812449	99.02720993	103.3131256	103.5387001	95.19244325	100.1550825	99.25278444	81.1634349	92.37412416	99.15268046	83.24914453	97.58839824	102.5419586	90.45537854	99.47835895	101.0573805	90.45537854	79.07772527	92.89555157	100.456249	94.9812612	87.97405893	86.89913639
	100.0420218	92.45029152	94.12468231	98.66945732	106.8022126	91.49112694	95.07698607	107.21374	112.1787758	95.79907311	100.8222455	106.5630139	94.363881	102.0182389	107.28061	90.66362099	100.5936924	106.3862341	90.9394563	108.3170813	113.8337877	94.363881	96.51666916	106.0846165	96.03827179	92.04279757	104.1795516	109.1445873	108.5929166	89.57990731	99.21451583
AMP3CPR1ERY1DOX1TMP2	AMP	CPR	ERY	DOX	TMP	AMP+CPR	AMP+ERY	AMP+DOX	AMP+TMP	CPR+ERY	CPR+DOX	CPR+TMP	ERY+DOX	ERY+TMP	DOX+TMP	AMP+CPR+ERY	AMP+CPR+DOX	AMP+CPR+TMP	AMP+ERY+DOX	AMP+ERY+TMP	AMP+DOX+TMP	CPR+ERY+DOX	CPR+ERY+TMP	CPR+DOX+TMP	ERY+DOX+TMP	AMP+CPR+ERY+DOX	AMP+CPR+ERY+TMP	AMP+CPR+DOX+TMP	AMP+ERY+DOX+TMP	CPR+ERY+DOX+TMP	AMP+CPR+ERY+DOX+TMP
	96.07936508	91.65839126	90.29649596	98.24088523	95.97105972	91.25396825	91.25396825	94.04761905	97.34920635	100.9646758	101.4186409	96.87898993	83.71400199	88.48063555	95.51709462	84.14285714	93.28571429	101.6666667	86.42857143	103.4444444	101.4126984	97.55993758	94.83614697	92.11235636	95.06312952	77.28571429	98.61904762	103.4444444	103.6984127	96.65200738	91.25396825
	94.7205475	101.5085295	96.99703933	105.1177217	102.1852531	90.80984194	91.07055565	93.93840639	104.3669545	99.02720993	103.3131256	108.5013393	95.19244325	100.6062315	102.8619766	81.1634349	92.37412416	100.7169627	83.24914453	94.4598338	103.3240997	90.45537854	101.9596786	100.6062315	99.02720993	79.07772527	90.02770083	98.89196676	92.63483787	97.89933737	91.85269676
	100.0420218	92.45029152	94.12468231	98.66945732	110.1509942	91.49112694	95.07698607	107.21374	110.2479285	95.79907311	100.8222455	110.6293915	94.363881	103.453431	106.3238152	90.66362099	100.5936924	103.6278809	90.9394563	106.3862341	107.7654107	94.363881	98.43025863	110.1509942	106.8022126	92.04279757	95.90449202	103.0762103	109.9720932	97.95186126	100.8695277
AMP3CPR1ERY1DOX1TMP3	AMP	CPR	ERY	DOX	TMP	AMP+CPR	AMP+ERY	AMP+DOX	AMP+TMP	CPR+ERY	CPR+DOX	CPR+TMP	ERY+DOX	ERY+TMP	DOX+TMP	AMP+CPR+ERY	AMP+CPR+DOX	AMP+CPR+TMP	AMP+ERY+DOX	AMP+ERY+TMP	AMP+DOX+TMP	CPR+ERY+DOX	CPR+ERY+TMP	CPR+DOX+TMP	ERY+DOX+TMP	AMP+CPR+ERY+DOX	AMP+CPR+ERY+TMP	AMP+CPR+DOX+TMP	AMP+ERY+DOX+TMP	CPR+ERY+DOX+TMP	AMP+CPR+ERY+DOX+TMP
	96.07936508	91.65839126	90.29649596	98.24088523	98.69485033	91.25396825	91.25396825	94.04761905	95.06349206	100.9646758	101.4186409	101.4186409	83.71400199	92.11235636	100.9646758	84.14285714	93.28571429	100.6507937	86.42857143	101.4126984	99.12698413	97.55993758	103.4614839	105.7313094	96.65200738	77.28571429	87.95238095	107.2539683	94.55555556	100.9646758	96.07936508
	94.7205475	101.5085295	96.99703933	105.1177217	98.80163542	90.80984194	91.07055565	93.93840639	99.41339417	99.02720993	103.3131256	105.5688707	95.19244325	99.02720993	105.3432962	81.1634349	92.37412416	94.9812612	83.24914453	95.50268861	101.2383901	90.45537854	103.0875511	104.8921472	99.02720993	79.07772527	77.51344305	96.80625713	91.33126935	100.380657	92.89555157
	100.0420218	92.45029152	94.12468231	98.66945732	109.1941994	91.49112694	95.07698607	107.21374	107.21374	95.79907311	100.8222455	109.6725968	94.363881	105.6062192	109.4333981	90.66362099	100.5936924	104.4553869	90.9394563	106.1103988	106.9379047	94.363881	101.7790402	112.7821797	108.7158021	92.04279757	89.56027971	105.5587281	101.4211984	106.0846165	95.07698607
AMP3CPR1ERY1DOX2TMP1	AMP	CPR	ERY	DOX	TMP	AMP+CPR	AMP+ERY	AMP+DOX	AMP+TMP	CPR+ERY	CPR+DOX	CPR+TMP	ERY+DOX	ERY+TMP	DOX+TMP	AMP+CPR+ERY	AMP+CPR+DOX	AMP+CPR+TMP	AMP+ERY+DOX	AMP+ERY+TMP	AMP+DOX+TMP	CPR+ERY+DOX	CPR+ERY+TMP	CPR+DOX+TMP	ERY+DOX+TMP	AMP+CPR+ERY+DOX	AMP+CPR+ERY+TMP	AMP+CPR+DOX+TMP	AMP+ERY+DOX+TMP	CPR+ERY+DOX+TMP	AMP+CPR+ERY+DOX+TMP
	96.07936508	91.65839126	90.29649596	99.82976309	91.88537381	91.25396825	91.25396825	95.82539683	102.4285714	100.9646758	98.46786778	88.93460065	93.47425167	90.29649596	97.33295503	84.14285714	100.6507937	108.2698413	93.79365079	102.9365079	96.84126984	101.6456235	90.97744361	98.46786778	95.97105972	88.20634921	105.4761905	115.1269841	97.6031746	98.01390268	96.58730159
	94.7205475	101.5085295	96.99703933	103.9898491	100.1550825	90.80984194	91.07055565	96.28482972	102.2812449	99.02720993	103.9898491	103.5387001	96.99703933	100.1550825	98.12491188	81.1634349	89.24555972	99.15268046	90.02770083	97.58839824	100.456249	92.71112364	99.47835895	94.96686874	96.99703933	82.72771713	92.89555157	106.7133779	100.7169627	96.54589031	100.456249
	100.0420218	92.45029152	94.12468231	105.1278218	106.8022126	91.49112694	95.07698607	108.5929166	112.1787758	95.79907311	100.3438481	106.5630139	96.27747047	102.0182389	99.86545074	90.66362099	100.5936924	106.3862341	97.55950393	108.3170813	108.3170813	101.5398415	96.51666916	107.28061	100.3438481	94.52531543	104.1795516	109.6962579	109.4204226	105.3670205	105.5587281
AMP3CPR1ERY1DOX2TMP2	AMP	CPR	ERY	DOX	TMP	AMP+CPR	AMP+ERY	AMP+DOX	AMP+TMP	CPR+ERY	CPR+DOX	CPR+TMP	ERY+DOX	ERY+TMP	DOX+TMP	AMP+CPR+ERY	AMP+CPR+DOX	AMP+CPR+TMP	AMP+ERY+DOX	AMP+ERY+TMP	AMP+DOX+TMP	CPR+ERY+DOX	CPR+ERY+TMP	CPR+DOX+TMP	ERY+DOX+TMP	AMP+CPR+ERY+DOX	AMP+CPR+ERY+TMP	AMP+CPR+DOX+TMP	AMP+ERY+DOX+TMP	CPR+ERY+DOX+TMP	AMP+CPR+ERY+DOX+TMP
	96.07936508	91.65839126	90.29649596	99.82976309	95.97105972	91.25396825	91.25396825	95.82539683	97.34920635	100.9646758	98.46786778	96.87898993	93.47425167	88.48063555	100.9646758	84.14285714	100.6507937	101.6666667	93.79365079	103.4444444	105.2222222	101.6456235	94.83614697	103.915449	97.55993758	88.20634921	98.61904762	101.9206349	98.11111111	98.01390268	94.04761905
	94.7205475	101.5085295	96.99703933	103.9898491	102.1852531	90.80984194	91.07055565	96.28482972	104.3669545	99.02720993	103.9898491	108.5013393	96.99703933	100.6062315	98.80163542	81.1634349	89.24555972	100.7169627	90.02770083	94.4598338	105.6705231	92.71112364	101.9596786	100.1550825	91.8088256	82.72771713	90.02770083	100.456249	98.89196676	96.3203158	98.89196676
	100.0420218	92.45029152	94.12468231	105.1278218	110.1509942	91.49112694	95.07698607	108.5929166	110.2479285	95.79907311	100.3438481	110.6293915	96.27747047	103.453431	99.14785469	90.66362099	100.5936924	103.6278809	97.55950393	106.3862341	108.3170813	101.5398415	98.43025863	108.2374047	93.88548363	94.52531543	95.90449202	107.4895754	108.041246	104.1710271	101.972869
AMP3CPR1ERY1DOX2TMP3	AMP	CPR	ERY	DOX	TMP	AMP+CPR	AMP+ERY	AMP+DOX	AMP+TMP	CPR+ERY	CPR+DOX	CPR+TMP	ERY+DOX	ERY+TMP	DOX+TMP	AMP+CPR+ERY	AMP+CPR+DOX	AMP+CPR+TMP	AMP+ERY+DOX	AMP+ERY+TMP	AMP+DOX+TMP	CPR+ERY+DOX	CPR+ERY+TMP	CPR+DOX+TMP	ERY+DOX+TMP	AMP+CPR+ERY+DOX	AMP+CPR+ERY+TMP	AMP+CPR+DOX+TMP	AMP+ERY+DOX+TMP	CPR+ERY+DOX+TMP	AMP+CPR+ERY+DOX+TMP
	96.07936508	91.65839126	90.29649596	99.82976309	98.69485033	91.25396825	91.25396825	95.82539683	95.06349206	100.9646758	98.46786778	101.4186409	93.47425167	92.11235636	100.7376933	84.14285714	100.6507937	100.6507937	93.79365079	101.4126984	100.9047619	101.6456235	103.4614839	102.5535537	97.55993758	88.20634921	87.95238095	104.7142857	96.84126984	103.0075188	90.49206349
	94.7205475	101.5085295	96.99703933	103.9898491	98.80163542	90.80984194	91.07055565	96.28482972	99.41339417	99.02720993	103.9898491	105.5688707	96.99703933	99.02720993	98.80163542	81.1634349	89.24555972	94.9812612	90.02770083	95.50268861	100.1955353	92.71112364	103.0875511	95.41801776	90.45537854	82.72771713	77.51344305	96.02411602	91.33126935	91.8088256	95.50268861
	100.0420218	92.45029152	94.12468231	105.1278218	109.1941994	91.49112694	95.07698607	108.5929166	107.21374	95.79907311	100.3438481	109.6725968	96.27747047	105.6062192	98.19105995	90.66362099	100.5936924	104.4553869	97.55950393	106.1103988	91.21529162	101.5398415	101.7790402	103.6926297	91.73269547	94.52531543	89.56027971	110.2479285	101.6970337	105.8454179	89.00860908
AMP3CPR1ERY1DOX3TMP1	AMP	CPR	ERY	DOX	TMP	AMP+CPR	AMP+ERY	AMP+DOX	AMP+TMP	CPR+ERY	CPR+DOX	CPR+TMP	ERY+DOX	ERY+TMP	DOX+TMP	AMP+CPR+ERY	AMP+CPR+DOX	AMP+CPR+TMP	AMP+ERY+DOX	AMP+ERY+TMP	AMP+DOX+TMP	CPR+ERY+DOX	CPR+ERY+TMP	CPR+DOX+TMP	ERY+DOX+TMP	AMP+CPR+ERY+DOX	AMP+CPR+ERY+TMP	AMP+CPR+DOX+TMP	AMP+ERY+DOX+TMP	CPR+ERY+DOX+TMP	AMP+CPR+ERY+DOX+TMP
	96.07936508	91.65839126	90.29649596	104.1424316	91.88537381	91.25396825	91.25396825	97.34920635	102.4285714	100.9646758	102.0995886	88.93460065	90.75046106	90.29649596	104.1424316	84.14285714	96.84126984	108.2698413	91.76190476	102.9365079	102.1746032	97.33295503	90.97744361	101.1916584	99.60278054	86.68253968	105.4761905	109.2857143	104.2063492	97.33295503	99.63492063
	94.7205475	101.5085295	96.99703933	105.5688707	100.1550825	90.80984194	91.07055565	96.02411602	102.2812449	99.02720993	108.2757648	103.5387001	97.22261384	100.1550825	92.25997462	81.1634349	89.50627342	99.15268046	89.24555972	97.58839824	96.02411602	95.19244325	99.47835895	91.58325109	82.56027069	79.85986638	92.89555157	103.8455271	94.4598338	82.7858452	105.4098094
	100.0420218	92.45029152	94.12468231	103.6926297	106.8022126	91.49112694	95.07698607	106.3862341	112.1787758	95.79907311	102.0182389	106.5630139	100.8222455	102.0182389	93.16788758	90.66362099	96.73199797	106.3862341	99.76618647	108.3170813	109.6962579	99.86545074	96.51666916	102.0182389	85.51352968	88.18110313	104.1795516	114.9371289	112.4546111	97.23426521	95.6286567
AMP3CPR1ERY1DOX3TMP2	AMP	CPR	ERY	DOX	TMP	AMP+CPR	AMP+ERY	AMP+DOX	AMP+TMP	CPR+ERY	CPR+DOX	CPR+TMP	ERY+DOX	ERY+TMP	DOX+TMP	AMP+CPR+ERY	AMP+CPR+DOX	AMP+CPR+TMP	AMP+ERY+DOX	AMP+ERY+TMP	AMP+DOX+TMP	CPR+ERY+DOX	CPR+ERY+TMP	CPR+DOX+TMP	ERY+DOX+TMP	AMP+CPR+ERY+DOX	AMP+CPR+ERY+TMP	AMP+CPR+DOX+TMP	AMP+ERY+DOX+TMP	CPR+ERY+DOX+TMP	AMP+CPR+ERY+DOX+TMP
	96.07936508	91.65839126	90.29649596	104.1424316	95.97105972	91.25396825	91.25396825	97.34920635	97.34920635	100.9646758	102.0995886	96.87898993	90.75046106	88.48063555	99.37579799	84.14285714	96.84126984	101.6666667	91.76190476	103.4444444	100.1428571	97.33295503	94.83614697	89.38856575	99.37579799	86.68253968	98.61904762	96.07936508	103.952381	101.4186409	97.85714286
	94.7205475	101.5085295	96.99703933	105.5688707	102.1852531	90.80984194	91.07055565	96.02411602	104.3669545	99.02720993	108.2757648	108.5013393	97.22261384	100.6062315	88.42520795	81.1634349	89.50627342	100.7169627	89.24555972	94.4598338	98.10982565	95.19244325	101.9596786	86.16946285	83.23699422	79.85986638	90.02770083	107.2348053	91.59198305	84.59044128	103.5848134
	100.0420218	92.45029152	94.12468231	103.6926297	110.1509942	91.49112694	95.07698607	106.3862341	110.2479285	95.79907311	102.0182389	110.6293915	100.8222455	103.453431	96.99506653	90.66362099	96.73199797	103.6278809	99.76618647	106.3862341	110.2479285	99.86545074	98.43025863	93.64628495	88.1447152	88.18110313	95.90449202	104.1795516	101.972869	98.19105995	95.90449202
AMP3CPR1ERY1DOX3TMP3	AMP	CPR	ERY	DOX	TMP	AMP+CPR	AMP+ERY	AMP+DOX	AMP+TMP	CPR+ERY	CPR+DOX	CPR+TMP	ERY+DOX	ERY+TMP	DOX+TMP	AMP+CPR+ERY	AMP+CPR+DOX	AMP+CPR+TMP	AMP+ERY+DOX	AMP+ERY+TMP	AMP+DOX+TMP	CPR+ERY+DOX	CPR+ERY+TMP	CPR+DOX+TMP	ERY+DOX+TMP	AMP+CPR+ERY+DOX	AMP+CPR+ERY+TMP	AMP+CPR+DOX+TMP	AMP+ERY+DOX+TMP	CPR+ERY+DOX+TMP	AMP+CPR+ERY+DOX+TMP
	96.07936508	91.65839126	90.29649596	104.1424316	98.69485033	91.25396825	91.25396825	97.34920635	95.06349206	100.9646758	102.0995886	101.4186409	90.75046106	92.11235636	97.55993758	84.14285714	96.84126984	100.6507937	91.76190476	101.4126984	100.9047619	97.33295503	103.4614839	98.69485033	88.93460065	86.68253968	87.95238095	93.03174603	102.4285714	99.60278054	93.79365079
	94.7205475	101.5085295	96.99703933	105.5688707	98.80163542	90.80984194	91.07055565	96.02411602	99.41339417	99.02720993	108.2757648	105.5688707	97.22261384	99.02720993	94.29014521	81.1634349	89.50627342	94.9812612	89.24555972	95.50268861	91.85269676	95.19244325	103.0875511	94.29014521	82.7858452	79.85986638	77.51344305	102.5419586	103.8455271	92.25997462	93.67769268
	100.0420218	92.45029152	94.12468231	103.6926297	109.1941994	91.49112694	95.07698607	106.3862341	107.21374	95.79907311	102.0182389	109.6725968	100.8222455	105.6062192	97.71266258	90.66362099	96.73199797	104.4553869	99.76618647	106.1103988	104.1795516	99.86545074	101.7790402	96.03827179	88.86231126	88.18110313	89.56027971	95.35282139	99.21451583	105.1278218	92.87030353
AMP3CPR1ERY2DOX1TMP1	AMP	CPR	ERY	DOX	TMP	AMP+CPR	AMP+ERY	AMP+DOX	AMP+TMP	CPR+ERY	CPR+DOX	CPR+TMP	ERY+DOX	ERY+TMP	DOX+TMP	AMP+CPR+ERY	AMP+CPR+DOX	AMP+CPR+TMP	AMP+ERY+DOX	AMP+ERY+TMP	AMP+DOX+TMP	CPR+ERY+DOX	CPR+ERY+TMP	CPR+DOX+TMP	ERY+DOX+TMP	AMP+CPR+ERY+DOX	AMP+CPR+ERY+TMP	AMP+CPR+DOX+TMP	AMP+ERY+DOX+TMP	CPR+ERY+DOX+TMP	AMP+CPR+ERY+DOX+TMP
	96.07936508	91.65839126	95.55789791	98.24088523	91.88537381	91.25396825	92.01587302	94.04761905	102.4285714	99.60278054	101.4186409	88.93460065	96.52160819	97.00346333	92.79330401	87.19047619	93.28571429	108.2698413	89.98412698	101.1587302	104.2063492	100.5107107	93.92821677	95.51709462	92.42583948	84.9047619	104.7142857	105.984127	103.1904762	92.33933891	102.6825397
	94.7205475	101.5085295	99.10541226	105.1177217	100.1550825	90.80984194	91.33126935	93.93840639	102.2812449	97.44818835	103.3131256	103.5387001	97.1969584	99.82108245	99.25278444	83.24914453	92.37412416	99.15268046	88.46341861	101.7598175	102.5419586	99.70393346	99.92950797	101.0573805	92.6643805	85.33485416	99.41339417	100.456249	98.89196676	99.92950797	92.63483787
	100.0420218	92.45029152	96.19192688	98.66945732	106.8022126	91.49112694	99.49035115	107.21374	112.1787758	92.92868889	100.8222455	106.5630139	98.385377	107.4028941	107.28061	90.9394563	100.5936924	106.3862341	100.8695277	109.9720932	113.8337877	101.5398415	97.95186126	106.0846165	102.0411272	98.93868052	101.4211984	109.1445873	114.3854583	101.7790402	98.38700988
AMP3CPR1ERY2DOX1TMP2	AMP	CPR	ERY	DOX	TMP	AMP+CPR	AMP+ERY	AMP+DOX	AMP+TMP	CPR+ERY	CPR+DOX	CPR+TMP	ERY+DOX	ERY+TMP	DOX+TMP	AMP+CPR+ERY	AMP+CPR+DOX	AMP+CPR+TMP	AMP+ERY+DOX	AMP+ERY+TMP	AMP+DOX+TMP	CPR+ERY+DOX	CPR+ERY+TMP	CPR+DOX+TMP	ERY+DOX+TMP	AMP+CPR+ERY+DOX	AMP+CPR+ERY+TMP	AMP+CPR+DOX+TMP	AMP+ERY+DOX+TMP	CPR+ERY+DOX+TMP	AMP+CPR+ERY+DOX+TMP
	96.07936508	91.65839126	95.55789791	98.24088523	95.97105972	91.25396825	92.01587302	94.04761905	97.34920635	99.60278054	101.4186409	96.87898993	96.52160819	96.03975305	95.51709462	87.19047619	93.28571429	101.6666667	89.98412698	103.1904762	101.4126984	100.5107107	100.9646758	92.11235636	97.00346333	84.9047619	103.6984127	103.4444444	95.82539683	92.79330401	96.58730159
	94.7205475	101.5085295	99.10541226	105.1177217	102.1852531	90.80984194	91.33126935	93.93840639	104.3669545	97.44818835	103.3131256	108.5013393	97.1969584	98.62829879	102.8619766	83.24914453	92.37412416	100.7169627	88.46341861	101.4991038	103.3240997	99.70393346	107.1478923	100.6062315	93.14149396	85.33485416	95.76340231	98.89196676	93.93840639	97.67376286	90.80984194
	100.0420218	92.45029152	96.19192688	98.66945732	110.1509942	91.49112694	99.49035115	107.21374	110.2479285	92.92868889	100.8222455	110.6293915	98.385377	103.5034273	106.3238152	90.9394563	100.5936924	103.6278809	100.8695277	109.1445873	107.7654107	101.5398415	103.9318284	110.1509942	104.478294	98.93868052	97.00783329	103.0762103	108.5929166	101.0614442	97.28366861
AMP3CPR1ERY2DOX1TMP3	AMP	CPR	ERY	DOX	TMP	AMP+CPR	AMP+ERY	AMP+DOX	AMP+TMP	CPR+ERY	CPR+DOX	CPR+TMP	ERY+DOX	ERY+TMP	DOX+TMP	AMP+CPR+ERY	AMP+CPR+DOX	AMP+CPR+TMP	AMP+ERY+DOX	AMP+ERY+TMP	AMP+DOX+TMP	CPR+ERY+DOX	CPR+ERY+TMP	CPR+DOX+TMP	ERY+DOX+TMP	AMP+CPR+ERY+DOX	AMP+CPR+ERY+TMP	AMP+CPR+DOX+TMP	AMP+ERY+DOX+TMP	CPR+ERY+DOX+TMP	AMP+CPR+ERY+DOX+TMP
	96.07936508	91.65839126	95.55789791	98.24088523	98.69485033	91.25396825	92.01587302	94.04761905	95.06349206	99.60278054	101.4186409	101.4186409	96.52160819	98.44902876	100.9646758	87.19047619	93.28571429	100.6507937	89.98412698	100.9047619	99.12698413	100.5107107	102.5535537	105.7313094	96.76253576	84.9047619	100.3968254	107.2539683	92.52380952	104.5963967	106.7460317
	94.7205475	101.5085295	99.10541226	105.1177217	98.80163542	90.80984194	91.33126935	93.93840639	99.41339417	97.44818835	103.3131256	105.5688707	97.1969584	99.34396899	105.3432962	83.24914453	92.37412416	94.9812612	88.46341861	96.02411602	101.2383901	99.70393346	101.5085295	104.8921472	70.24004771	85.33485416	89.24555972	96.80625713	94.19912009	104.2154237	98.10982565
	100.0420218	92.45029152	96.19192688	98.66945732	109.1941994	91.49112694	99.49035115	107.21374	107.21374	92.92868889	100.8222455	109.6725968	98.385377	97.1667936	109.4333981	90.9394563	100.5936924	104.4553869	100.8695277	108.5929166	106.9379047	101.5398415	102.9750336	112.7821797	101.3099772	98.93868052	94.80115075	105.5587281	103.6278809	109.6725968	96.45616266
AMP3CPR1ERY2DOX2TMP1	AMP	CPR	ERY	DOX	TMP	AMP+CPR	AMP+ERY	AMP+DOX	AMP+TMP	CPR+ERY	CPR+DOX	CPR+TMP	ERY+DOX	ERY+TMP	DOX+TMP	AMP+CPR+ERY	AMP+CPR+DOX	AMP+CPR+TMP	AMP+ERY+DOX	AMP+ERY+TMP	AMP+DOX+TMP	CPR+ERY+DOX	CPR+ERY+TMP	CPR+DOX+TMP	ERY+DOX+TMP	AMP+CPR+ERY+DOX	AMP+CPR+ERY+TMP	AMP+CPR+DOX+TMP	AMP+ERY+DOX+TMP	CPR+ERY+DOX+TMP	AMP+CPR+ERY+DOX+TMP
	96.07936508	91.65839126	95.55789791	99.82976309	91.88537381	91.25396825	92.01587302	95.82539683	102.4285714	99.60278054	98.46786778	88.93460065	96.52160819	97.00346333	97.33295503	87.19047619	100.6507937	108.2698413	91.25396825	101.1587302	96.84126984	100.0567456	93.92821677	98.46786778	99.65366662	93.79365079	104.7142857	115.1269841	97.34920635	97.33295503	99.88888889
	94.7205475	101.5085295	99.10541226	103.9898491	100.1550825	90.80984194	91.33126935	96.28482972	102.2812449	97.44818835	103.9898491	103.5387001	96.24273147	99.82108245	98.12491188	83.24914453	89.24555972	99.15268046	87.6812775	101.7598175	100.456249	98.80163542	99.92950797	94.96686874	103.6379902	85.85628157	99.41339417	106.7133779	97.58839824	92.93669815	100.7169627
	100.0420218	92.45029152	96.19192688	105.1278218	106.8022126	91.49112694	99.49035115	108.5929166	112.1787758	92.92868889	100.3438481	106.5630139	99.6039604	107.4028941	99.86545074	90.9394563	100.5936924	106.3862341	101.4211984	109.9720932	108.3170813	102.2574376	97.95186126	107.28061	106.4280274	97.00783329	101.4211984	109.6962579	110.5237639	105.3670205	103.0762103
AMP3CPR1ERY2DOX2TMP2	AMP	CPR	ERY	DOX	TMP	AMP+CPR	AMP+ERY	AMP+DOX	AMP+TMP	CPR+ERY	CPR+DOX	CPR+TMP	ERY+DOX	ERY+TMP	DOX+TMP	AMP+CPR+ERY	AMP+CPR+DOX	AMP+CPR+TMP	AMP+ERY+DOX	AMP+ERY+TMP	AMP+DOX+TMP	CPR+ERY+DOX	CPR+ERY+TMP	CPR+DOX+TMP	ERY+DOX+TMP	AMP+CPR+ERY+DOX	AMP+CPR+ERY+TMP	AMP+CPR+DOX+TMP	AMP+ERY+DOX+TMP	CPR+ERY+DOX+TMP	AMP+CPR+ERY+DOX+TMP
	96.07936508	91.65839126	95.55789791	99.82976309	95.97105972	91.25396825	92.01587302	95.82539683	97.34920635	99.60278054	98.46786778	96.87898993	96.52160819	96.03975305	100.9646758	87.19047619	100.6507937	101.6666667	91.25396825	103.1904762	105.2222222	100.0567456	100.9646758	103.915449	100.1355218	93.79365079	103.6984127	101.9206349	98.61904762	104.1424316	103.1904762
	94.7205475	101.5085295	99.10541226	103.9898491	102.1852531	90.80984194	91.33126935	96.28482972	104.3669545	97.44818835	103.9898491	108.5013393	96.24273147	98.62829879	98.80163542	83.24914453	89.24555972	100.7169627	87.6812775	101.4991038	105.6705231	98.80163542	107.1478923	100.1550825	97.1969584	85.85628157	95.76340231	100.456249	102.8026723	95.19244325	98.10982565
	100.0420218	92.45029152	96.19192688	105.1278218	110.1509942	91.49112694	99.49035115	108.5929166	110.2479285	92.92868889	100.3438481	110.6293915	99.6039604	103.5034273	99.14785469	90.9394563	100.5936924	103.6278809	101.4211984	109.1445873	108.3170813	102.2574376	103.9318284	108.2374047	103.2597106	97.00783329	97.00783329	107.4895754	108.041246	108.7158021	98.6628452
AMP3CPR1ERY2DOX2TMP3	AMP	CPR	ERY	DOX	TMP	AMP+CPR	AMP+ERY	AMP+DOX	AMP+TMP	CPR+ERY	CPR+DOX	CPR+TMP	ERY+DOX	ERY+TMP	DOX+TMP	AMP+CPR+ERY	AMP+CPR+DOX	AMP+CPR+TMP	AMP+ERY+DOX	AMP+ERY+TMP	AMP+DOX+TMP	CPR+ERY+DOX	CPR+ERY+TMP	CPR+DOX+TMP	ERY+DOX+TMP	AMP+CPR+ERY+DOX	AMP+CPR+ERY+TMP	AMP+CPR+DOX+TMP	AMP+ERY+DOX+TMP	CPR+ERY+DOX+TMP	AMP+CPR+ERY+DOX+TMP
	96.07936508	91.65839126	95.55789791	99.82976309	98.69485033	91.25396825	92.01587302	95.82539683	95.06349206	99.60278054	98.46786778	101.4186409	96.52160819	98.44902876	100.7376933	87.19047619	100.6507937	100.6507937	91.25396825	100.9047619	100.9047619	100.0567456	102.5535537	102.5535537	101.099232	93.79365079	100.3968254	104.7142857	87.44444444	90.06951341	92.52380952
	94.7205475	101.5085295	99.10541226	103.9898491	98.80163542	90.80984194	91.33126935	96.28482972	99.41339417	97.44818835	103.9898491	105.5688707	96.24273147	99.34396899	98.80163542	83.24914453	89.24555972	94.9812612	87.6812775	96.02411602	100.1955353	98.80163542	101.5085295	95.41801776	92.42582377	85.85628157	89.24555972	96.02411602	94.19912009	88.42520795	96.02411602
	100.0420218	92.45029152	96.19192688	105.1278218	109.1941994	91.49112694	99.49035115	108.5929166	107.21374	92.92868889	100.3438481	109.6725968	99.6039604	97.1667936	98.19105995	90.9394563	100.5936924	104.4553869	101.4211984	108.5929166	91.21529162	102.2574376	102.9750336	103.6926297	99.6039604	97.00783329	94.80115075	110.2479285	101.972869	104.4102257	96.18032734
AMP3CPR1ERY2DOX3TMP1	AMP	CPR	ERY	DOX	TMP	AMP+CPR	AMP+ERY	AMP+DOX	AMP+TMP	CPR+ERY	CPR+DOX	CPR+TMP	ERY+DOX	ERY+TMP	DOX+TMP	AMP+CPR+ERY	AMP+CPR+DOX	AMP+CPR+TMP	AMP+ERY+DOX	AMP+ERY+TMP	AMP+DOX+TMP	CPR+ERY+DOX	CPR+ERY+TMP	CPR+DOX+TMP	ERY+DOX+TMP	AMP+CPR+ERY+DOX	AMP+CPR+ERY+TMP	AMP+CPR+DOX+TMP	AMP+ERY+DOX+TMP	CPR+ERY+DOX+TMP	AMP+CPR+ERY+DOX+TMP
	96.07936508	91.65839126	95.55789791	104.1424316	91.88537381	91.25396825	92.01587302	97.34920635	102.4285714	99.60278054	102.0995886	88.93460065	94.83511519	97.00346333	104.1424316	87.19047619	96.84126984	108.2698413	93.03174603	101.1587302	102.1746032	101.872606	93.92821677	101.1916584	103.7494353	93.79365079	104.7142857	109.2857143	107.2539683	103.2345013	108.7777778
	94.7205475	101.5085295	99.10541226	105.5688707	100.1550825	90.80984194	91.33126935	96.02411602	102.2812449	97.44818835	108.2757648	103.5387001	98.62829879	99.82108245	92.25997462	83.24914453	89.50627342	99.15268046	92.63483787	101.7598175	96.02411602	105.5688707	99.92950797	91.58325109	93.85716416	86.11699527	99.41339417	103.8455271	96.80625713	86.16946285	105.4098094
	100.0420218	92.45029152	96.19192688	103.6926297	106.8022126	91.49112694	99.49035115	106.3862341	112.1787758	92.92868889	102.0182389	106.5630139	101.7974105	107.4028941	93.16788758	90.9394563	96.73199797	106.3862341	104.4553869	109.9720932	109.6962579	104.1710271	97.95186126	102.0182389	99.6039604	91.76696226	101.4211984	114.9371289	116.5921408	104.8886231	98.93868052
AMP3CPR1ERY2DOX3TMP2	AMP	CPR	ERY	DOX	TMP	AMP+CPR	AMP+ERY	AMP+DOX	AMP+TMP	CPR+ERY	CPR+DOX	CPR+TMP	ERY+DOX	ERY+TMP	DOX+TMP	AMP+CPR+ERY	AMP+CPR+DOX	AMP+CPR+TMP	AMP+ERY+DOX	AMP+ERY+TMP	AMP+DOX+TMP	CPR+ERY+DOX	CPR+ERY+TMP	CPR+DOX+TMP	ERY+DOX+TMP	AMP+CPR+ERY+DOX	AMP+CPR+ERY+TMP	AMP+CPR+DOX+TMP	AMP+ERY+DOX+TMP	CPR+ERY+DOX+TMP	AMP+CPR+ERY+DOX+TMP
	96.07936508	91.65839126	95.55789791	104.1424316	95.97105972	91.25396825	92.01587302	97.34920635	97.34920635	99.60278054	102.0995886	96.87898993	94.83511519	96.03975305	99.37579799	87.19047619	96.84126984	101.6666667	93.03174603	103.1904762	100.1428571	101.872606	100.9646758	89.38856575	104.2312905	93.79365079	103.6984127	96.07936508	108.2698413	100.9646758	98.87301587
	94.7205475	101.5085295	99.10541226	105.5688707	102.1852531	90.80984194	91.33126935	96.02411602	104.3669545	97.44818835	108.2757648	108.5013393	98.62829879	98.62829879	88.42520795	83.24914453	89.50627342	100.7169627	92.63483787	101.4991038	98.10982565	105.5688707	107.1478923	86.16946285	97.1969584	86.11699527	95.76340231	107.2348053	99.15268046	84.59044128	105.6705231
	100.0420218	92.45029152	96.19192688	103.6926297	110.1509942	91.49112694	99.49035115	106.3862341	110.2479285	92.92868889	102.0182389	110.6293915	101.7974105	103.5034273	96.99506653	90.9394563	96.73199797	103.6278809	104.4553869	109.1445873	110.2479285	104.1710271	103.9318284	93.64628495	88.8804265	91.76696226	97.00783329	104.1795516	108.868752	100.5830468	94.24948012
AMP3CPR1ERY2DOX3TMP3	AMP	CPR	ERY	DOX	TMP	AMP+CPR	AMP+ERY	AMP+DOX	AMP+TMP	CPR+ERY	CPR+DOX	CPR+TMP	ERY+DOX	ERY+TMP	DOX+TMP	AMP+CPR+ERY	AMP+CPR+DOX	AMP+CPR+TMP	AMP+ERY+DOX	AMP+ERY+TMP	AMP+DOX+TMP	CPR+ERY+DOX	CPR+ERY+TMP	CPR+DOX+TMP	ERY+DOX+TMP	AMP+CPR+ERY+DOX	AMP+CPR+ERY+TMP	AMP+CPR+DOX+TMP	AMP+ERY+DOX+TMP	CPR+ERY+DOX+TMP	AMP+CPR+ERY+DOX+TMP
	96.07936508	91.65839126	95.55789791	104.1424316	98.69485033	91.25396825	92.01587302	97.34920635	95.06349206	99.60278054	102.0995886	101.4186409	94.83511519	98.44902876	97.55993758	87.19047619	96.84126984	100.6507937	93.03174603	100.9047619	100.9047619	101.872606	102.5535537	98.69485033	103.9903629	93.79365079	100.3968254	93.03174603	101.4126984	100.9646758	86.93650794
	94.7205475	101.5085295	99.10541226	105.5688707	98.80163542	90.80984194	91.33126935	96.02411602	99.41339417	97.44818835	108.2757648	105.5688707	98.62829879	99.34396899	94.29014521	83.24914453	89.50627342	94.9812612	92.63483787	96.02411602	91.85269676	105.5688707	101.5085295	94.29014521	93.85716416	86.11699527	89.24555972	102.5419586	102.0205312	91.13210207	81.94557601
	100.0420218	92.45029152	96.19192688	103.6926297	109.1941994	91.49112694	99.49035115	106.3862341	107.21374	92.92868889	102.0182389	109.6725968	101.7974105	97.1667936	97.71266258	90.9394563	96.73199797	104.4553869	104.4553869	108.5929166	104.1795516	104.1710271	102.9750336	96.03827179	92.04874334	91.76696226	94.80115075	95.35282139	100.5936924	100.5830468	95.35282139
AMP3CPR1ERY3DOX1TMP1	AMP	CPR	ERY	DOX	TMP	AMP+CPR	AMP+ERY	AMP+DOX	AMP+TMP	CPR+ERY	CPR+DOX	CPR+TMP	ERY+DOX	ERY+TMP	DOX+TMP	AMP+CPR+ERY	AMP+CPR+DOX	AMP+CPR+TMP	AMP+ERY+DOX	AMP+ERY+TMP	AMP+DOX+TMP	CPR+ERY+DOX	CPR+ERY+TMP	CPR+DOX+TMP	ERY+DOX+TMP	AMP+CPR+ERY+DOX	AMP+CPR+ERY+TMP	AMP+CPR+DOX+TMP	AMP+ERY+DOX+TMP	CPR+ERY+DOX+TMP	AMP+CPR+ERY+DOX+TMP
	96.07936508	91.65839126	95.82539683	98.24088523	91.88537381	91.25396825	95.06349206	94.04761905	102.4285714	98.69485033	101.4186409	88.93460065	89.98412698	92.26984127	92.79330401	90.74603175	93.28571429	108.2698413	93.79365079	104.2063492	104.2063492	92.33933891	97.78692013	95.51709462	94.04761905	88.20634921	107.2539683	105.984127	97.0952381	90.06951341	107.5079365
	94.7205475	101.5085295	97.32768454	105.1177217	100.1550825	90.80984194	96.28482972	93.93840639	102.2812449	101.9596786	103.3131256	103.5387001	89.24555972	104.8883819	99.25278444	90.54912824	92.37412416	99.15268046	90.80984194	104.1062408	102.5419586	100.6062315	106.4711688	101.0573805	105.4098094	87.15985009	103.3240997	100.456249	100.1955353	96.77146482	92.11341046
	100.0420218	92.45029152	99.21451583	98.66945732	106.8022126	91.49112694	102.800375	107.21374	112.1787758	98.19105995	100.8222455	106.5630139	96.45616266	113.282117	107.28061	91.76696226	100.5936924	106.3862341	107.4895754	116.5921408	113.8337877	97.95186126	104.6494244	106.0846165	115.4887996	69.85529421	103.9037162	109.1445873	110.2479285	98.66945732	108.041246
AMP3CPR1ERY3DOX1TMP2	AMP	CPR	ERY	DOX	TMP	AMP+CPR	AMP+ERY	AMP+DOX	AMP+TMP	CPR+ERY	CPR+DOX	CPR+TMP	ERY+DOX	ERY+TMP	DOX+TMP	AMP+CPR+ERY	AMP+CPR+DOX	AMP+CPR+TMP	AMP+ERY+DOX	AMP+ERY+TMP	AMP+DOX+TMP	CPR+ERY+DOX	CPR+ERY+TMP	CPR+DOX+TMP	ERY+DOX+TMP	AMP+CPR+ERY+DOX	AMP+CPR+ERY+TMP	AMP+CPR+DOX+TMP	AMP+ERY+DOX+TMP	CPR+ERY+DOX+TMP	AMP+CPR+ERY+DOX+TMP
	96.07936508	91.65839126	95.82539683	98.24088523	95.97105972	91.25396825	95.06349206	94.04761905	97.34920635	98.69485033	101.4186409	96.87898993	89.98412698	89.73015873	95.51709462	90.74603175	93.28571429	101.6666667	93.79365079	102.4285714	101.4126984	92.33933891	98.24088523	92.11235636	96.58730159	88.20634921	105.4761905	103.4444444	99.12698413	99.37579799	102.6825397
	94.7205475	101.5085295	97.32768454	105.1177217	102.1852531	90.80984194	96.28482972	93.93840639	104.3669545	101.9596786	103.3131256	108.5013393	89.24555972	101.7598175	102.8619766	90.54912824	92.37412416	100.7169627	90.80984194	99.67410787	103.3240997	100.6062315	103.5387001	100.6062315	106.1919505	87.15985009	99.93482157	98.89196676	103.3240997	101.734104	99.67410787
	100.0420218	92.45029152	99.21451583	98.66945732	110.1509942	91.49112694	102.800375	107.21374	110.2479285	98.19105995	100.8222455	110.6293915	96.45616266	110.2479285	106.3238152	91.76696226	100.5936924	103.6278809	107.4895754	112.1787758	107.7654107	97.95186126	108.9550007	110.1509942	115.2129643	69.85529421	101.972869	103.0762103	111.0754345	109.9117955	105.0070575
AMP3CPR1ERY3DOX1TMP3	AMP	CPR	ERY	DOX	TMP	AMP+CPR	AMP+ERY	AMP+DOX	AMP+TMP	CPR+ERY	CPR+DOX	CPR+TMP	ERY+DOX	ERY+TMP	DOX+TMP	AMP+CPR+ERY	AMP+CPR+DOX	AMP+CPR+TMP	AMP+ERY+DOX	AMP+ERY+TMP	AMP+DOX+TMP	CPR+ERY+DOX	CPR+ERY+TMP	CPR+DOX+TMP	ERY+DOX+TMP	AMP+CPR+ERY+DOX	AMP+CPR+ERY+TMP	AMP+CPR+DOX+TMP	AMP+ERY+DOX+TMP	CPR+ERY+DOX+TMP	AMP+CPR+ERY+DOX+TMP
	96.07936508	91.65839126	95.82539683	98.24088523	98.69485033	91.25396825	95.06349206	94.04761905	95.06349206	98.69485033	101.4186409	101.4186409	89.98412698	95.06349206	100.9646758	90.74603175	93.28571429	100.6507937	93.79365079	95.57142857	99.12698413	92.33933891	109.1360477	105.7313094	106.7460317	88.20634921	97.6031746	107.2539683	91.76190476	103.915449	110.3015873
	94.7205475	101.5085295	97.32768454	105.1177217	98.80163542	90.80984194	96.28482972	93.93840639	99.41339417	101.9596786	103.3131256	105.5688707	89.24555972	102.2812449	105.3432962	90.54912824	92.37412416	94.9812612	90.80984194	97.84911194	101.2383901	100.6062315	105.3432962	104.8921472	109.5812286	87.15985009	89.24555972	96.80625713	98.37053935	102.6364021	92.63483787
	100.0420218	92.45029152	99.21451583	98.66945732	109.1941994	91.49112694	102.800375	107.21374	107.21374	98.19105995	100.8222455	109.6725968	96.45616266	109.6962579	109.4333981	91.76696226	100.5936924	104.4553869	107.4895754	105.5587281	106.9379047	97.95186126	109.9117955	112.7821797	114.3854583	69.85529421	94.24948012	105.5587281	105.8345635	109.4333981	104.4553869
AMP3CPR1ERY3DOX2TMP1	AMP	CPR	ERY	DOX	TMP	AMP+CPR	AMP+ERY	AMP+DOX	AMP+TMP	CPR+ERY	CPR+DOX	CPR+TMP	ERY+DOX	ERY+TMP	DOX+TMP	AMP+CPR+ERY	AMP+CPR+DOX	AMP+CPR+TMP	AMP+ERY+DOX	AMP+ERY+TMP	AMP+DOX+TMP	CPR+ERY+DOX	CPR+ERY+TMP	CPR+DOX+TMP	ERY+DOX+TMP	AMP+CPR+ERY+DOX	AMP+CPR+ERY+TMP	AMP+CPR+DOX+TMP	AMP+ERY+DOX+TMP	CPR+ERY+DOX+TMP	AMP+CPR+ERY+DOX+TMP
	96.07936508	91.65839126	95.82539683	99.82976309	91.88537381	91.25396825	95.06349206	95.82539683	102.4285714	98.69485033	98.46786778	88.93460065	86.17460317	92.26984127	97.33295503	90.74603175	100.6507937	108.2698413	96.07936508	104.2063492	96.84126984	100.9646758	97.78692013	98.46786778	106.2380952	86.42857143	107.2539683	115.1269841	97.85714286	101.1916584	109.2857143
	94.7205475	101.5085295	97.32768454	103.9898491	100.1550825	90.80984194	96.28482972	96.28482972	102.2812449	101.9596786	103.9898491	103.5387001	102.8026723	104.8883819	98.12491188	90.54912824	89.24555972	99.15268046	95.76340231	104.1062408	100.456249	108.2757648	106.4711688	94.96686874	110.6240834	86.89913639	103.3240997	106.7133779	102.2812449	95.19244325	103.8455271
	100.0420218	92.45029152	99.21451583	105.1278218	106.8022126	91.49112694	102.800375	108.5929166	112.1787758	98.19105995	100.3438481	106.5630139	99.21451583	113.282117	99.86545074	91.76696226	100.5936924	106.3862341	106.1103988	116.5921408	108.3170813	104.4102257	104.6494244	107.28061	112.1787758	96.45616266	103.9037162	109.6962579	111.0754345	110.6293915	108.5929166
AMP3CPR1ERY3DOX2TMP2	AMP	CPR	ERY	DOX	TMP	AMP+CPR	AMP+ERY	AMP+DOX	AMP+TMP	CPR+ERY	CPR+DOX	CPR+TMP	ERY+DOX	ERY+TMP	DOX+TMP	AMP+CPR+ERY	AMP+CPR+DOX	AMP+CPR+TMP	AMP+ERY+DOX	AMP+ERY+TMP	AMP+DOX+TMP	CPR+ERY+DOX	CPR+ERY+TMP	CPR+DOX+TMP	ERY+DOX+TMP	AMP+CPR+ERY+DOX	AMP+CPR+ERY+TMP	AMP+CPR+DOX+TMP	AMP+ERY+DOX+TMP	CPR+ERY+DOX+TMP	AMP+CPR+ERY+DOX+TMP
	96.07936508	91.65839126	95.82539683	99.82976309	95.97105972	91.25396825	95.06349206	95.82539683	97.34920635	98.69485033	98.46786778	96.87898993	86.17460317	89.73015873	100.9646758	90.74603175	100.6507937	101.6666667	96.07936508	102.4285714	105.2222222	100.9646758	98.24088523	103.915449	108.5238095	86.42857143	105.4761905	101.9206349	94.80952381	100.5107107	101.1587302
	94.7205475	101.5085295	97.32768454	103.9898491	102.1852531	90.80984194	96.28482972	96.28482972	104.3669545	101.9596786	103.9898491	108.5013393	102.8026723	101.7598175	98.80163542	90.54912824	89.24555972	100.7169627	95.76340231	99.67410787	105.6705231	108.2757648	103.5387001	100.1550825	105.9312368	86.89913639	99.93482157	100.456249	87.15985009	92.71112364	99.93482157
	100.0420218	92.45029152	99.21451583	105.1278218	110.1509942	91.49112694	102.800375	108.5929166	110.2479285	98.19105995	100.3438481	110.6293915	99.21451583	110.2479285	99.14785469	91.76696226	100.5936924	103.6278809	106.1103988	112.1787758	108.3170813	104.4102257	108.9550007	108.2374047	107.7654107	96.45616266	101.972869	107.4895754	109.1445873	107.998206	100.3178571
AMP3CPR1ERY3DOX2TMP3	AMP	CPR	ERY	DOX	TMP	AMP+CPR	AMP+ERY	AMP+DOX	AMP+TMP	CPR+ERY	CPR+DOX	CPR+TMP	ERY+DOX	ERY+TMP	DOX+TMP	AMP+CPR+ERY	AMP+CPR+DOX	AMP+CPR+TMP	AMP+ERY+DOX	AMP+ERY+TMP	AMP+DOX+TMP	CPR+ERY+DOX	CPR+ERY+TMP	CPR+DOX+TMP	ERY+DOX+TMP	AMP+CPR+ERY+DOX	AMP+CPR+ERY+TMP	AMP+CPR+DOX+TMP	AMP+ERY+DOX+TMP	CPR+ERY+DOX+TMP	AMP+CPR+ERY+DOX+TMP
	96.07936508	91.65839126	95.82539683	99.82976309	98.69485033	91.25396825	95.06349206	95.82539683	95.06349206	98.69485033	98.46786778	101.4186409	86.17460317	95.06349206	100.7376933	90.74603175	100.6507937	100.6507937	96.07936508	95.57142857	100.9047619	100.9646758	109.1360477	102.5535537	105.984127	86.42857143	97.6031746	104.7142857	95.31746032	105.5043269	101.1587302
	94.7205475	101.5085295	97.32768454	103.9898491	98.80163542	90.80984194	96.28482972	96.28482972	99.41339417	101.9596786	103.9898491	105.5688707	102.8026723	102.2812449	98.80163542	90.54912824	89.24555972	94.9812612	95.76340231	97.84911194	100.1955353	108.2757648	105.3432962	95.41801776	92.89555157	86.89913639	89.24555972	96.02411602	95.76340231	90.90652756	91.07055565
	100.0420218	92.45029152	99.21451583	105.1278218	109.1941994	91.49112694	102.800375	108.5929166	107.21374	98.19105995	100.3438481	109.6725968	99.21451583	109.6962579	98.19105995	91.76696226	100.5936924	104.4553869	106.1103988	105.5587281	91.21529162	104.4102257	109.9117955	103.6926297	101.4211984	96.45616266	94.24948012	110.2479285	102.2487043	107.998206	104.1795516
AMP3CPR1ERY3DOX3TMP1	AMP	CPR	ERY	DOX	TMP	AMP+CPR	AMP+ERY	AMP+DOX	AMP+TMP	CPR+ERY	CPR+DOX	CPR+TMP	ERY+DOX	ERY+TMP	DOX+TMP	AMP+CPR+ERY	AMP+CPR+DOX	AMP+CPR+TMP	AMP+ERY+DOX	AMP+ERY+TMP	AMP+DOX+TMP	CPR+ERY+DOX	CPR+ERY+TMP	CPR+DOX+TMP	ERY+DOX+TMP	AMP+CPR+ERY+DOX	AMP+CPR+ERY+TMP	AMP+CPR+DOX+TMP	AMP+ERY+DOX+TMP	CPR+ERY+DOX+TMP	AMP+CPR+ERY+DOX+TMP
	96.07936508	91.65839126	95.82539683	104.1424316	91.88537381	91.25396825	95.06349206	97.34920635	102.4285714	98.69485033	102.0995886	88.93460065	90.49206349	92.26984127	104.1424316	90.74603175	96.84126984	108.2698413	95.82539683	104.2063492	102.1746032	100.9646758	97.78692013	101.1916584	108.2698413	96.84126984	107.2539683	109.2857143	104.4603175	97.33295503	108.2698413
	94.7205475	101.5085295	97.32768454	105.5688707	100.1550825	90.80984194	96.28482972	96.02411602	102.2812449	101.9596786	108.2757648	103.5387001	102.0205312	104.8883819	92.25997462	90.54912824	89.50627342	99.15268046	95.24197491	104.1062408	96.02411602	106.9223178	106.4711688	91.58325109	89.50627342	84.55271305	103.3240997	103.8455271	98.37053935	81.88354716	99.67410787
	100.0420218	92.45029152	99.21451583	103.6926297	106.8022126	91.49112694	102.800375	106.3862341	112.1787758	98.19105995	102.0182389	106.5630139	103.6278809	113.282117	93.16788758	91.76696226	96.73199797	106.3862341	105.8345635	116.5921408	109.6962579	100.8222455	104.6494244	102.0182389	106.9379047	90.38778567	103.9037162	114.9371289	110.5237639	99.38705337	96.18032734
AMP3CPR1ERY3DOX3TMP2	AMP	CPR	ERY	DOX	TMP	AMP+CPR	AMP+ERY	AMP+DOX	AMP+TMP	CPR+ERY	CPR+DOX	CPR+TMP	ERY+DOX	ERY+TMP	DOX+TMP	AMP+CPR+ERY	AMP+CPR+DOX	AMP+CPR+TMP	AMP+ERY+DOX	AMP+ERY+TMP	AMP+DOX+TMP	CPR+ERY+DOX	CPR+ERY+TMP	CPR+DOX+TMP	ERY+DOX+TMP	AMP+CPR+ERY+DOX	AMP+CPR+ERY+TMP	AMP+CPR+DOX+TMP	AMP+ERY+DOX+TMP	CPR+ERY+DOX+TMP	AMP+CPR+ERY+DOX+TMP
	96.07936508	91.65839126	95.82539683	104.1424316	95.97105972	91.25396825	95.06349206	97.34920635	97.34920635	98.69485033	102.0995886	96.87898993	90.49206349	89.73015873	99.37579799	90.74603175	96.84126984	101.6666667	95.82539683	102.4285714	100.1428571	100.9646758	98.24088523	89.38856575	103.4444444	96.84126984	105.4761905	96.07936508	102.6825397	103.2345013	106.4920635
	94.7205475	101.5085295	97.32768454	105.5688707	102.1852531	90.80984194	96.28482972	96.02411602	104.3669545	101.9596786	108.2757648	108.5013393	102.0205312	101.7598175	88.42520795	90.54912824	89.50627342	100.7169627	95.24197491	99.67410787	98.10982565	106.9223178	103.5387001	86.16946285	85.33485416	84.55271305	99.93482157	107.2348053	96.02411602	88.65078246	102.0205312
	100.0420218	92.45029152	99.21451583	103.6926297	110.1509942	91.49112694	102.800375	106.3862341	110.2479285	98.19105995	102.0182389	110.6293915	103.6278809	110.2479285	96.99506653	91.76696226	96.73199797	103.6278809	105.8345635	112.1787758	110.2479285	100.8222455	108.9550007	93.64628495	108.3170813	90.38778567	101.972869	104.1795516	114.3854583	102.2574376	99.49035115
AMP3CPR1ERY3DOX3TMP3	AMP	CPR	ERY	DOX	TMP	AMP+CPR	AMP+ERY	AMP+DOX	AMP+TMP	CPR+ERY	CPR+DOX	CPR+TMP	ERY+DOX	ERY+TMP	DOX+TMP	AMP+CPR+ERY	AMP+CPR+DOX	AMP+CPR+TMP	AMP+ERY+DOX	AMP+ERY+TMP	AMP+DOX+TMP	CPR+ERY+DOX	CPR+ERY+TMP	CPR+DOX+TMP	ERY+DOX+TMP	AMP+CPR+ERY+DOX	AMP+CPR+ERY+TMP	AMP+CPR+DOX+TMP	AMP+ERY+DOX+TMP	CPR+ERY+DOX+TMP	AMP+CPR+ERY+DOX+TMP
	96.07936508	91.65839126	95.82539683	104.1424316	98.69485033	91.25396825	95.06349206	97.34920635	95.06349206	98.69485033	102.0995886	101.4186409	90.49206349	95.06349206	97.55993758	90.74603175	96.84126984	100.6507937	95.82539683	95.57142857	100.9047619	100.9646758	109.1360477	98.69485033	100.9047619	96.84126984	97.6031746	93.03174603	102.6825397	100.0567456	82.61904762
	94.7205475	101.5085295	97.32768454	105.5688707	98.80163542	90.80984194	96.28482972	96.02411602	99.41339417	101.9596786	108.2757648	105.5688707	102.0205312	102.2812449	94.29014521	90.54912824	89.50627342	94.9812612	95.24197491	97.84911194	91.85269676	106.9223178	105.3432962	94.29014521	94.19912009	84.55271305	89.24555972	102.5419586	101.7598175	93.38784717	83.77057194
	100.0420218	92.45029152	99.21451583	103.6926297	109.1941994	91.49112694	102.800375	106.3862341	107.21374	98.19105995	102.0182389	109.6725968	103.6278809	109.6962579	97.71266258	91.76696226	96.73199797	104.4553869	105.8345635	105.5587281	104.1795516	100.8222455	109.9117955	96.03827179	106.3862341	90.38778567	94.24948012	95.35282139	103.6278809	96.27747047	97.00783329
AMP3CPR2ERY1DOX1TMP1	AMP	CPR	ERY	DOX	TMP	AMP+CPR	AMP+ERY	AMP+DOX	AMP+TMP	CPR+ERY	CPR+DOX	CPR+TMP	ERY+DOX	ERY+TMP	DOX+TMP	AMP+CPR+ERY	AMP+CPR+DOX	AMP+CPR+TMP	AMP+ERY+DOX	AMP+ERY+TMP	AMP+DOX+TMP	CPR+ERY+DOX	CPR+ERY+TMP	CPR+DOX+TMP	ERY+DOX+TMP	AMP+CPR+ERY+DOX	AMP+CPR+ERY+TMP	AMP+CPR+DOX+TMP	AMP+ERY+DOX+TMP	CPR+ERY+DOX+TMP	AMP+CPR+ERY+DOX+TMP
	96.07936508	101.5810872	90.29649596	98.24088523	91.88537381	91	91.25396825	94.04761905	102.4285714	96.03975305	102.5447975	100.3764493	83.71400199	90.29649596	92.79330401	85.15873016	98.87301587	110.3015873	86.42857143	102.9365079	104.2063492	97.2443909	101.5810872	102.3038699	80.99021138	84.14285714	101.1587302	108.015873	105.7301587	93.1486222	94.55555556
	94.7205475	97.67407187	96.99703933	105.1177217	100.1550825	91.85269676	91.07055565	93.93840639	102.2812449	91.71015357	102.2066498	105.7850007	95.19244325	100.1550825	99.25278444	84.81342676	93.67769268	95.76340231	83.24914453	97.58839824	102.5419586	93.14149396	106.0235575	95.76561801	90.45537854	81.68486231	98.10982565	105.6705231	94.9812612	95.28850455	91.33126935

	AMP	CPR	ERY	DOX	TMP	AMP+CPR	AMP+ERY	AMP+DOX	AMP+TMP	CPR+ERY	CPR+DOX	CPR+TMP	ERY+DOX	ERY+TMP	DOX+TMP	AMP+CPR+ERY	AMP+CPR+DOX	AMP+CPR+TMP	AMP+ERY+DOX	AMP+ERY+TMP	AMP+DOX+TMP	CPR+ERY+DOX	CPR+ERY+TMP	CPR+DOX+TMP	ERY+DOX+TMP	AMP+CPR+ERY+DOX	AMP+CPR+ERY+TMP	AMP+CPR+DOX+TMP	AMP+ERY+DOX+TMP	CPR+ERY+DOX+TMP	AMP+CPR+ERY+DOX+TMP
	100.0420218	97.65422696	94.12468231	98.66945732	106.8022126	99.21451583	95.07698607	107.21374	112.1787758	66.458492	105.6968774	110.0837776	94.363881	102.0182389	107.28061	89.2844444	100.8695277	114.3854583	90.9394563	108.3170813	113.8337877	94.24219345	104.7220107	111.5460777	96.03827179	87.62943249	103.9037162	113.5579524	108.5929166	97.41051028	101.972869
AMP3CPR2ERY1DOX1TMP2	AMP	CPR	ERY	DOX	TMP	AMP+CPR	AMP+ERY	AMP+DOX	AMP+TMP	CPR+ERY	CPR+DOX	CPR+TMP	ERY+DOX	ERY+TMP	DOX+TMP	AMP+CPR+ERY	AMP+CPR+DOX	AMP+CPR+TMP	AMP+ERY+DOX	AMP+ERY+TMP	AMP+DOX+TMP	CPR+ERY+DOX	CPR+ERY+TMP	CPR+DOX+TMP	ERY+DOX+TMP	AMP+CPR+ERY+DOX	AMP+CPR+ERY+TMP	AMP+CPR+DOX+TMP	AMP+ERY+DOX+TMP	CPR+ERY+DOX+TMP	AMP+CPR+ERY+DOX+TMP
	96.07936508	101.5810872	90.29649596	98.24088523	95.97105972	91	91.25396825	94.04761905	97.34920635	96.03975305	102.5447975	104.472218	83.71400199	88.48063555	95.51709462	85.15873016	98.87301587	109.031746	86.42857143	103.4444444	101.4126984	97.2443909	98.68995633	101.8220148	95.06312952	84.14285714	99.63492063	99.88888889	103.6984127	97.72624605	97.6031746
	94.7205475	97.67407187	96.99703933	105.1177217	102.1852531	91.85269676	91.07055565	93.93840639	104.3669545	91.71015357	102.2066498	106.7392277	95.19244325	100.6062315	102.8619766	84.81342676	93.67769268	98.10982565	83.24914453	94.4598338	103.3240997	93.14149396	100.0596392	92.90293723	99.02720993	81.68486231	96.80625713	103.8455271	92.63483787	95.52706128	93.67769268
	100.0420218	97.65422696	94.12468231	98.66945732	110.1509942	99.21451583	95.07698607	107.21374	110.2479285	66.458492	105.6968774	105.9405941	94.363881	103.453431	106.3238152	89.2844444	100.8695277	111.6271051	90.9394563	106.3862341	107.7654107	94.24219345	103.0159939	111.302361	106.8022126	87.62943249	101.972869	109.1445873	109.9720932	103.5034273	106.3862341
AMP3CPR2ERY1DOX1TMP3	AMP	CPR	ERY	DOX	TMP	AMP+CPR	AMP+ERY	AMP+DOX	AMP+TMP	CPR+ERY	CPR+DOX	CPR+TMP	ERY+DOX	ERY+TMP	DOX+TMP	AMP+CPR+ERY	AMP+CPR+DOX	AMP+CPR+TMP	AMP+ERY+DOX	AMP+ERY+TMP	AMP+DOX+TMP	CPR+ERY+DOX	CPR+ERY+TMP	CPR+DOX+TMP	ERY+DOX+TMP	AMP+CPR+ERY+DOX	AMP+CPR+ERY+TMP	AMP+CPR+DOX+TMP	AMP+ERY+DOX+TMP	CPR+ERY+DOX+TMP	AMP+CPR+ERY+DOX+TMP
	96.07936508	101.5810872	90.29649596	98.24088523	98.69485033	91	91.25396825	94.04761905	95.06349206	96.03975305	102.5447975	103.7494353	83.71400199	92.11235636	100.9646758	85.15873016	98.87301587	105.984127	86.42857143	101.4126984	99.12698413	97.2443909	98.9308839	101.3401596	96.65200738	84.14285714	94.80952381	97.0952381	94.55555556	99.41273905	102.6825397
	94.7205475	97.67407187	96.99703933	105.1177217	98.80163542	91.85269676	91.07055565	93.93840639	99.41339417	91.71015357	102.2066498	101.7295363	95.19244325	99.02720993	105.3432962	84.81342676	93.67769268	99.41339417	83.24914453	95.50268861	101.2383901	93.14149396	99.82108245	100.7753094	99.02720993	81.68486231	92.37412416	101.4991038	91.33126935	86.46190547	86.89913639
	100.0420218	97.65422696	94.12468231	98.66945732	109.1941994	99.21451583	95.07698607	107.21374	107.21374	66.458492	105.6968774	106.6717441	94.363881	105.6062192	109.4333981	89.2844444	100.8695277	108.868752	90.9394563	106.1103988	106.9379047	94.24219345	102.7722772	107.6466108	108.7158021	87.62943249	98.38700988	105.0070575	101.4211984	108.6214775	98.38700988
AMP3CPR2ERY1DOX2TMP1	AMP	CPR	ERY	DOX	TMP	AMP+CPR	AMP+ERY	AMP+DOX	AMP+TMP	CPR+ERY	CPR+DOX	CPR+TMP	ERY+DOX	ERY+TMP	DOX+TMP	AMP+CPR+ERY	AMP+CPR+DOX	AMP+CPR+TMP	AMP+ERY+DOX	AMP+ERY+TMP	AMP+DOX+TMP	CPR+ERY+DOX	CPR+ERY+TMP	CPR+DOX+TMP	ERY+DOX+TMP	AMP+CPR+ERY+DOX	AMP+CPR+ERY+TMP	AMP+CPR+DOX+TMP	AMP+ERY+DOX+TMP	CPR+ERY+DOX+TMP	AMP+CPR+ERY+DOX+TMP
	96.07936508	101.5810872	90.29649596	99.82976309	91.88537381	91	91.25396825	95.82539683	102.4285714	96.03975305	100.6173769	100.3764493	93.47425167	90.29649596	97.33295503	85.15873016	95.31746032	110.3015873	93.79365079	102.9365079	96.84126984	97.48531848	101.5810872	102.785725	95.97105972	89.98412698	101.1587302	105.2222222	97.6031746	95.79882548	107.7619048
	94.7205475	97.67407187	96.99703933	103.9898491	100.1550825	91.85269676	91.07055565	96.28482972	102.2812449	91.71015357	102.2066498	105.7850007	96.99703933	100.1550825	98.12491188	84.81342676	84.81342676	95.76340231	90.02770083	97.58839824	100.456249	96.95840167	106.0235575	100.7753094	96.99703933	81.42414861	98.10982565	103.5848134	100.7169627	86.93901894	99.93482157
	100.0420218	97.65422696	94.12468231	105.1278218	106.8022126	99.21451583	95.07698607	108.5929166	112.1787758	66.458492	94.24219345	110.0837776	96.27747047	102.0182389	99.86545074	89.2844444	106.1103988	114.3854583	97.55950393	108.3170813	108.3170813	97.1667936	104.7220107	106.4280274	100.3438481	87.35359717	103.9037162	99.49035115	109.4204226	108.8651942	106.3862341
AMP3CPR2ERY1DOX2TMP2	AMP	CPR	ERY	DOX	TMP	AMP+CPR	AMP+ERY	AMP+DOX	AMP+TMP	CPR+ERY	CPR+DOX	CPR+TMP	ERY+DOX	ERY+TMP	DOX+TMP	AMP+CPR+ERY	AMP+CPR+DOX	AMP+CPR+TMP	AMP+ERY+DOX	AMP+ERY+TMP	AMP+DOX+TMP	CPR+ERY+DOX	CPR+ERY+TMP	CPR+DOX+TMP	ERY+DOX+TMP	AMP+CPR+ERY+DOX	AMP+CPR+ERY+TMP	AMP+CPR+DOX+TMP	AMP+ERY+DOX+TMP	CPR+ERY+DOX+TMP	AMP+CPR+ERY+DOX+TMP
	96.07936508	101.5810872	90.29649596	99.82976309	95.97105972	91	91.25396825	95.82539683	97.34920635	96.03975305	100.6173769	104.472218	93.47425167	88.48063555	100.9646758	85.15873016	95.31746032	109.031746	93.79365079	103.4444444	105.2222222	97.48531848	98.68995633	98.20810119	97.55993758	89.98412698	99.63492063	105.2222222	98.11111111	96.03975305	100.9047619
	94.7205475	97.67407187	96.99703933	103.9898491	102.1852531	91.85269676	91.07055565	96.28482972	104.3669545	91.71015357	102.2066498	106.7392277	96.99703933	100.6062315	98.80163542	84.81342676	84.81342676	98.10982565	90.02770083	94.4598338	105.6705231	96.95840167	100.0596392	100.7753094	91.8088256	81.42414861	96.80625713	102.8026723	98.89196676	86.46190547	102.2812449
	100.0420218	97.65422696	94.12468231	105.1278218	110.1509942	99.21451583	95.07698607	108.5929166	110.2479285	66.458492	94.24219345	105.9405941	96.27747047	103.453431	99.14785469	89.2844444	106.1103988	111.6271051	97.55950393	106.3862341	108.3170813	97.1667936	103.0159939	111.5460777	93.88548363	87.35359717	101.972869	114.6612936	108.041246	108.3777609	108.5929166
AMP3CPR2ERY1DOX2TMP3	AMP	CPR	ERY	DOX	TMP	AMP+CPR	AMP+ERY	AMP+DOX	AMP+TMP	CPR+ERY	CPR+DOX	CPR+TMP	ERY+DOX	ERY+TMP	DOX+TMP	AMP+CPR+ERY	AMP+CPR+DOX	AMP+CPR+TMP	AMP+ERY+DOX	AMP+ERY+TMP	AMP+DOX+TMP	CPR+ERY+DOX	CPR+ERY+TMP	CPR+DOX+TMP	ERY+DOX+TMP	AMP+CPR+ERY+DOX	AMP+CPR+ERY+TMP	AMP+CPR+DOX+TMP	AMP+ERY+DOX+TMP	CPR+ERY+DOX+TMP	AMP+CPR+ERY+DOX+TMP
	96.07936508	101.5810872	90.29649596	99.82976309	98.69485033	91	91.25396825	95.82539683	95.06349206	96.03975305	100.6173769	103.7494353	93.47425167	92.11235636	100.7376933	85.15873016	95.31746032	105.984127	93.79365079	101.4126984	100.9047619	97.48531848	98.9308839	101.3401596	97.55993758	89.98412698	94.80952381	97.34920635	96.84126984	99.17181147	98.36507937
	94.7205475	97.67407187	96.99703933	103.9898491	98.80163542	91.85269676	91.07055565	96.28482972	99.41339417	91.71015357	102.2066498	101.7295363	96.99703933	99.02720993	98.80163542	84.81342676	84.81342676	99.41339417	90.02770083	95.50268861	100.1955353	96.95840167	99.82108245	96.71984494	90.45537854	81.42414861	92.37412416	94.9812612	91.33126935	90.75592664	96.28482972
	100.0420218	97.65422696	94.12468231	105.1278218	109.1941994	99.21451583	95.07698607	108.5929166	107.21374	66.458492	94.24219345	106.6717441	96.27747047	105.6062192	98.19105995	89.2844444	106.1103988	108.868752	97.55950393	106.1103988	91.21529162	97.1667936	102.7722772	104.9657273	91.73269547	87.35359717	98.38700988	101.4211984	101.6970337	87.41812643	98.93868052

[illegible]

AMP3CPR3ERY3DOX2TMP1	AMP	CPR	ERY	DOX	TMP	AMP+CPR	AMP+ERY	AMP+DOX	AMP+TMP	CPR+ERY	CPR+DOX	CPR+TMP	ERY+DOX	ERY+TMP	DOX+TMP	AMP+CPR+ERY	AMP+CPR+DOX	AMP+CPR+TMP	AMP+ERY+DOX	AMP+ERY+TMP	AMP+DOX+TMP	CPR+ERY+DOX	CPR+ERY+TMP	CPR+DOX+TMP	ERY+DOX+TMP	AMP+CPR+ERY+DOX	AMP+CPR+ERY+TMP	AMP+CPR+DOX+TMP	AMP+ERY+DOX+TMP	CPR+ERY+DOX+TMP	AMP+CPR+ERY+DOX+TMP
	96.07936508	97.34920635	95.82539683	99.82976309	91.88537381	98.11111111	95.06349206	95.82539683	102.4285714	96.84126984	91	99.12698413	86.17460317	92.26984127	97.33295503	94.80952381	99.63492063	108.5238095	96.07936508	104.2063492	96.84126984	90.74603175	94.55555556	105.2222222	106.2380952	95.31746032	107.5079365	104.968254	97.85714286	115.3809524	100.1428571
	94.7205475	102.8026723	97.32768454	103.9898491	100.1550825	98.10982565	96.28482972	96.28482972	102.2812449	98.63125306	101.7598175	109.8419423	102.8026723	104.8883819	98.12491188	96.80625713	100.1955353	107.495519	95.76340231	104.1062408	100.456249	100.1955353	110.102656	115.5776438	110.6240834	97.32768454	105.6705231	103.8455271	102.2812449	117.4026397	92.63483787
	100.0420218	101.972869	99.21451583	105.1278218	106.8022126	100.3178571	102.800375	108.5929166	112.1787758	106.9379047	101.4211984	117.4196468	99.21451583	113.282117	99.86545074	96.18032734	101.6970337	113.8337877	106.1103988	116.5921408	108.3170813	104.7312222	116.3163055	117.4196468	112.1787758	101.1453631	112.4546111	113.0062817	111.0754345	121.2813412	110.2479285

AMP3CPR3ERY3DOX2TMP2	AMP	CPR	ERY	DOX	TMP	AMP+CPR	AMP+ERY	AMP+DOX	AMP+TMP	CPR+ERY	CPR+DOX	CPR+TMP	ERY+DOX	ERY+TMP	DOX+TMP	AMP+CPR+ERY	AMP+CPR+DOX	AMP+CPR+TMP	AMP+ERY+DOX	AMP+ERY+TMP	AMP+DOX+TMP	CPR+ERY+DOX	CPR+ERY+TMP	CPR+DOX+TMP	ERY+DOX+TMP	AMP+CPR+ERY+DOX	AMP+CPR+ERY+TMP	AMP+CPR+DOX+TMP	AMP+ERY+DOX+TMP	CPR+ERY+DOX+TMP	AMP+CPR+ERY+DOX+TMP
	96.07936508	97.34920635	95.82539683	99.82976309	95.97105972	98.11111111	95.06349206	95.82539683	97.34920635	96.84126984	91	96.33333333	86.17460317	89.73015873	100.9646758	94.80952381	99.63492063	108.5238095	96.07936508	102.4285714	105.2222222	90.74603175	96.58730159	108.015873	108.5238095	95.31746032	107.2539683	104.4603175	94.80952381	111.5714286	100.1428571
	94.7205475	102.8026723	97.32768454	103.9898491	102.1852531	98.10982565	96.28482972	96.28482972	104.3669545	98.63125306	101.7598175	105.9312368	102.8026723	101.7598175	98.80163542	96.80625713	100.1955353	106.4526642	95.76340231	99.67410787	105.6705231	100.1955353	94.4598338	112.1883657	105.9312368	97.32768454	103.5848134	110.6240834	87.15985009	102.2812449	107.7562327
	100.0420218	101.972869	99.21451583	105.1278218	110.1509942	100.3178571	102.800375	108.5929166	110.2479285	106.9379047	101.4211984	113.8337877	99.21451583	110.2479285	99.14785469	96.18032734	101.6970337	112.4546111	106.1103988	112.1787758	108.3170813	104.7312222	112.4546111	114.3854583	107.7654107	101.1453631	108.3170813	114.109623	109.1445873	114.109623	85.97442058

AMP3CPR3ERY3DOX2TMP3	AMP	CPR	ERY	DOX	TMP	AMP+CPR	AMP+ERY	AMP+DOX	AMP+TMP	CPR+ERY	CPR+DOX	CPR+TMP	ERY+DOX	ERY+TMP	DOX+TMP	AMP+CPR+ERY	AMP+CPR+DOX	AMP+CPR+TMP	AMP+ERY+DOX	AMP+ERY+TMP	AMP+DOX+TMP	CPR+ERY+DOX	CPR+ERY+TMP	CPR+DOX+TMP	ERY+DOX+TMP	AMP+CPR+ERY+DOX	AMP+CPR+ERY+TMP	AMP+CPR+DOX+TMP	AMP+ERY+DOX+TMP	CPR+ERY+DOX+TMP	AMP+CPR+ERY+DOX+TMP
	96.07936508	97.34920635	95.82539683	99.82976309	98.69485033	98.11111111	95.06349206	95.82539683	95.06349206	96.84126984	91	93.03174603	86.17460317	95.06349206	100.7376933	94.80952381	99.63492063	103.4444444	96.07936508	95.57142857	100.9047619	90.74603175	93.28571429	104.2063492	105.984127	95.31746032	104.968254	101.4126984	95.31746032	107.5079365	101.1587302
	94.7205475	102.8026723	97.32768454	103.9898491	98.80163542	98.10982565	96.28482972	96.28482972	99.41339417	98.63125306	101.7598175	104.8883819	102.8026723	102.2812449	98.80163542	96.80625713	100.1955353	103.8455271	95.76340231	97.84911194	100.1955353	100.1955353	101.2383901	102.8026723	92.89555157	97.32768454	100.7169627	100.9776764	95.76340231	105.4098094	99.67410787
	100.0420218	101.972869	99.21451583	105.1278218	109.1941994	100.3178571	102.800375	108.5929166	107.21374	106.9379047	101.4211984	111.9029404	99.21451583	109.6962579	98.19105995	96.18032734	101.6970337	108.041246	106.1103988	105.5587281	91.21529162	104.7312222	107.7654107	112.1787758	101.4211984	101.1453631	107.7654107	104.1795516	102.2487043	116.0404702	103.0762103

AMP3CPR3ERY3DOX3TMP1	AMP	CPR	ERY	DOX	TMP	AMP+CPR	AMP+ERY	AMP+DOX	AMP+TMP	CPR+ERY	CPR+DOX	CPR+TMP	ERY+DOX	ERY+TMP	DOX+TMP	AMP+CPR+ERY	AMP+CPR+DOX	AMP+CPR+TMP	AMP+ERY+DOX	AMP+ERY+TMP	AMP+DOX+TMP	CPR+ERY+DOX	CPR+ERY+TMP	CPR+DOX+TMP	ERY+DOX+TMP	AMP+CPR+ERY+DOX	AMP+CPR+ERY+TMP	AMP+CPR+DOX+TMP	AMP+ERY+DOX+TMP	CPR+ERY+DOX+TMP	AMP+CPR+ERY+DOX+TMP
	96.07936508	97.34920635	95.82539683	104.1424316	91.88537381	98.11111111	95.06349206	97.34920635	102.4285714	96.84126984	90.23809524	99.12698413	90.49206349	92.26984127	104.1424316	94.80952381	100.1428571	108.5238095	95.82539683	104.2063492	102.1746032	92.52380952	94.55555556	108.7777778	108.2698413	97.34920635	107.5079365	111.8253968	104.4603175	103.952381	104.7142857
	94.7205475	102.8026723	97.32768454	105.5688707	100.1550825	98.10982565	96.28482972	96.02411602	102.2812449	98.63125306	98.89196676	109.8419423	102.0205312	104.8883819	92.25997462	96.80625713	98.37053935	107.495519	95.24197491	104.1062408	96.02411602	101.4991038	110.102656	107.2348053	89.50627342	95.76340231	105.6705231	104.1062408	98.37053935	105.1490956	105.6705231
	100.0420218	101.972869	99.21451583	103.6926297	106.8022126	100.3178571	102.800375	106.3862341	112.1787758	106.9379047	104.7312222	117.4196468	103.6278809	113.282117	93.16788758	96.18032734	105.0070575	113.8337877	105.8345635	116.5921408	109.6962579	105.0070575	116.3163055	106.6620694	106.9379047	104.7312222	112.4546111	110.5237639	110.5237639	119.9021647	110.2479285

AMP3CPR3ERY3DOX3TMP2	AMP	CPR	ERY	DOX	TMP	AMP+CPR	AMP+ERY	AMP+DOX	AMP+TMP	CPR+ERY	CPR+DOX	CPR+TMP	ERY+DOX	ERY+TMP	DOX+TMP	AMP+CPR+ERY	AMP+CPR+DOX	AMP+CPR+TMP	AMP+ERY+DOX	AMP+ERY+TMP	AMP+DOX+TMP	CPR+ERY+DOX	CPR+ERY+TMP	CPR+DOX+TMP	ERY+DOX+TMP	AMP+CPR+ERY+DOX	AMP+CPR+ERY+TMP	AMP+CPR+DOX+TMP	AMP+ERY+DOX+TMP	CPR+ERY+DOX+TMP	AMP+CPR+ERY+DOX+TMP
	96.07936508	97.34920635	95.82539683	104.1424316	95.97105972	98.11111111	95.06349206	97.34920635	97.34920635	96.84126984	90.23809524	96.33333333	90.49206349	89.73015873	99.37579799	94.80952381	100.1428571	108.5238095	95.82539683	102.4285714	100.1428571	92.52380952	96.58730159	101.4126984	103.4444444	97.34920635	107.2539683	107.7619048	102.6825397	103.4444444	106.4920635
	94.7205475	102.8026723	97.32768454	105.5688707	102.1852531	98.10982565	96.28482972	96.02411602	104.3669545	98.63125306	98.89196676	105.9312368	102.0205312	101.7598175	88.42520795	96.80625713	98.37053935	106.4526642	95.24197491	99.67410787	98.10982565	101.4991038	94.4598338	100.1955353	85.33485416	95.76340231	103.5848134	88.72413231	96.02411602	102.5419586	103.063386
	100.0420218	101.972869	99.21451583	103.6926297	110.1509942	100.3178571	102.800375	106.3862341	110.2479285	106.9379047	104.7312222	113.8337877	103.6278809	110.2479285	96.99506653	96.18032734	105.0070575	112.4546111	105.8345635	112.1787758	110.2479285	105.0070575	112.4546111	107.21374	108.3170813	104.7312222	108.3170813	119.350494	114.3854583	108.868752	113.0062817

AMP3CPR3ERY3DOX3TMP3	AMP	CPR	ERY	DOX	TMP	AMP+CPR	AMP+ERY	AMP+DOX	AMP+TMP	CPR+ERY	CPR+DOX	CPR+TMP	ERY+DOX	ERY+TMP	DOX+TMP	AMP+CPR+ERY	AMP+CPR+DOX	AMP+CPR+TMP	AMP+ERY+DOX	AMP+ERY+TMP	AMP+DOX+TMP	CPR+ERY+DOX	CPR+ERY+TMP	CPR+DOX+TMP	ERY+DOX+TMP	AMP+CPR+ERY+DOX	AMP+CPR+ERY+TMP	AMP+CPR+DOX+TMP	AMP+ERY+DOX+TMP	CPR+ERY+DOX+TMP	AMP+CPR+ERY+DOX+TMP
	96.07936508	97.34920635	95.82539683	104.1424316	98.69485033	98.11111111	95.06349206	97.34920635	95.06349206	96.84126984	90.23809524	93.03174603	90.49206349	95.06349206	97.55993758	94.80952381	100.1428571	103.4444444	95.82539683	95.57142857	100.9047619	92.52380952	93.28571429	99.38095238	100.9047619	97.34920635	104.968254	99.63492063	102.6825397	101.4126984	98.87301587
	94.7205475	102.8026723	97.32768454	105.5688707	98.80163542	98.10982565	96.28482972	96.02411602	99.41339417	98.63125306	98.89196676	104.8883819	102.0205312	102.2812449	94.29014521	96.80625713	98.37053935	103.8455271	95.24197491	97.84911194	91.85269676	101.4991038	101.2383901	97.06697083	94.19912009	95.76340231	100.7169627	90.02770083	101.7598175	101.7598175	99.15268046
	100.0420218	101.972869	99.21451583	103.6926297	109.1941994	100.3178571	102.800375	106.3862341	107.21374	106.9379047	104.7312222	111.9029404	103.6278809	109.6962579	97.71266258	96.18032734	105.0070575	108.041246	105.8345635	105.5587281	104.1795516	105.0070575	107.7654107	103.3520456	106.3862341	104.7312222	107.7654107	102.800375	103.6278809	108.5929166	99.21451583

AMP1CPR1ERY1FUS1DOX1	AMP	CPR	ERY	FUS	DOX	AMP+CPR	AMP+ERY	AMP+FUS	AMP+DOX	CPR+ERY	CPR+FUS	CPR+DOX	ERY+FUS	ERY+DOX	FUS+DOX	AMP+CPR+ERY	AMP+CPR+FUS	AMP+CPR+DOX	AMP+ERY+FUS	AMP+ERY+DOX	AMP+FUS+DOX	CPR+ERY+FUS	CPR+ERY+DOX	CPR+FUS+DOX	ERY+FUS+DOX	AMP+CPR+ERY+FUS	AMP+CPR+ERY+DOX	AMP+CPR+FUS+DOX	AMP+ERY+FUS+DOX	CPR+ERY+FUS+DOX	AMP+CPR+ERY+FUS+DOX
	60.31013476	89.73601625	94.46187927	65.33136422	108.9348348	59.01790659	52.51984493	3.599778475	73.4908621	87.07771829	66.40206757	105.9811704	61.19623408	105.0950711	58.27949049	67.58353332	4.818165036	72.60476278	5.113531475	71.71866347	1.827579841	74.48772383	93.28041351	55.3258261	49.71386376	9.839394499	73.19549566	0.978401329	2.12294628	45.28336718	1.864500646
	62.48760167	97.22277326	92.14441579	86.92719698	111.1882563	64.8482444	48.02618528	5.673477485	74.68756199	105.7925015	82.16623686	114.6796271	80.26185281	102.6185281	61.53540964	58.18290022	3.193810752	69.92660186	4.146002777	81.67030351	-0.039674668	83.43582622	97.5401706	59.05574291	47.6294386	21.28545923	69.60920452	-0.991866693	-0.35707201	45.40765721	0.595120016
	56.37453712	102.0454946	100.9169459	63.3926997	104.5847293	56.97407865	50.13225181	32.11073885	74.96032446	96.96702522	74.67818727	104.3025921	71.01040381	109.6631987	57.78522306	46.74660554	4.778698642	67.34262035	6.718391818	71.57467819	-0.335037912	50.76706048	99.50625992	56.09239993	47.62828425	8.446482102	58.03209311	5.589843061	-0.370305061	46.4997355	5.342973021

[illegible]

	56.37453712	92.58317025	95.40117417	101.1990831	99.22412273	47.09927702	52.38934932	40.29271733	74.39605008	91.33072407	91.33072407	122.6418787	98.84540117	111.0567515	93.58137895	48.50996297	17.40433786	45.0537824	40.01058014	71.57467819	0.229236466	84.12915851	121.0763209	81.3111546	105.1076321	18.8855581	47.87515429	16.55792629	1.886792453	77.92563601	14.61823312
AMP1CPR2ERY2FUS3DOX1	AMP	CPR	ERY	FUS	DOX	AMP+CPR	AMP+ERY	AMP+FUS	AMP+DOX	CPR+ERY	CPR+FUS	CPR+DOX	ERY+FUS	ERY+DOX	FUS+DOX	AMP+CPR+ERY	AMP+CPR+FUS	AMP+CPR+DOX	AMP+ERY+FUS	AMP+ERY+DOX	AMP+FUS+DOX	CPR+ERY+FUS	CPR+ERY+DOX	CPR+FUS+DOX	ERY+FUS+DOX	AMP+CPR+ERY+FUS	AMP+CPR+ERY+DOX	AMP+CPR+FUS+DOX	AMP+ERY+FUS+DOX	CPR+ERY+FUS+DOX	AMP+CPR+ERY+FUS+DOX
	60.31013476	88.33862829	92.2257522	103.6182389	108.9348348	58.72254015	49.2708141	39.81908806	73.4908621	97.30891422	105.9801906	105.9801906	102.0930667	101.7940572	79.69355732	46.61251615	36.71774045	66.10670113	44.10190142	73.78622854	47.20324903	102.0930667	111.6613717	90.43169501	79.06933283	43.65885176	78.51209156	33.3210264	41.59128669	71.59409456	34.50249215
	62.48760167	96.88521438	104.2684099	117.5362031	111.1882563	64.53084705	55.82225749	46.12180123	74.68756199	98.42338012	113.1897712	114.1126706	117.4966353	111.9592386	70.24399921	66.4352311	40.58718508	53.91787344	47.88732394	80.71811149	49.93056933	115.0355701	115.3432032	84.88752163	74.73562776	55.64372148	67.07002579	39.45645705	41.53937711	72.88982888	42.17417179
	56.37453712	92.58317025	95.40117417	113.6131194	104.5847293	47.09927702	52.38934932	49.56797743	74.96032446	91.33072407	111.0567515	107.6125245	112.6223092	103.5420744	58.59636748	48.50996297	59.16064186	69.88185505	42.5145477	80.03879386	48.43942867	106.0469667	113.2485323	92.27005871	88.51272016	35.46111797	60.57132781	39.41103862	55.21072121	81.3111546	32.0754717
AMP1CPR2ERY2FUS3DOX2	AMP	CPR	ERY	FUS	DOX	AMP+CPR	AMP+ERY	AMP+FUS	AMP+DOX	CPR+ERY	CPR+FUS	CPR+DOX	ERY+FUS	ERY+DOX	FUS+DOX	AMP+CPR+ERY	AMP+CPR+FUS	AMP+CPR+DOX	AMP+ERY+FUS	AMP+ERY+DOX	AMP+FUS+DOX	CPR+ERY+FUS	CPR+ERY+DOX	CPR+FUS+DOX	ERY+FUS+DOX	AMP+CPR+ERY+FUS	AMP+CPR+ERY+DOX	AMP+CPR+FUS+DOX	AMP+ERY+FUS+DOX	CPR+ERY+FUS+DOX	AMP+CPR+ERY+FUS+DOX
	60.31013476	88.33862829	92.2257522	103.6182389	113.9560642	58.72254015	49.2708141	39.81908806	82.64722171	97.30891422	105.9801906	117.3425528	102.0930667	107.1762287	86.78235186	46.61251615	36.71774045	61.3808381	44.10190142	74.9676943	35.09322503	102.0930667	113.4554289	90.73070454	86.54457111	43.65885176	73.19549566	57.24570796	35.68395791	80.56438049	48.08934835
	62.48760167	96.88521438	104.2684099	117.5362031	121.6623686	64.53084705	55.82225749	46.12180123	87.70085301	98.42338012	113.1897712	121.188233	117.4966353	117.4966353	76.90934338	66.4352311	40.58718508	56.45705217	47.88732394	88.33564769	38.50426503	115.0355701	113.8050375	89.1943857	78.11959239	55.64372148	63.57865503	53.10454275	51.83495338	77.50432609	42.31303313
	56.37453712	92.58317025	95.40117417	113.6131194	102.8919062	47.09927702	52.38934932	49.56797743	80.03879386	91.33072407	111.0567515	115.1272016	112.6223092	111.369863	76.93528478	48.50996297	59.16064186	57.18568154	42.5145477	74.67818727	32.0754717	106.0469667	114.81409	82.87671233	78.18003914	35.46111797	53.51789808	47.5930171	41.95027332	83.18982387	30.45318286
AMP1CPR2ERY2FUS3DOX3	AMP	CPR	ERY	FUS	DOX	AMP+CPR	AMP+ERY	AMP+FUS	AMP+DOX	CPR+ERY	CPR+FUS	CPR+DOX	ERY+FUS	ERY+DOX	FUS+DOX	AMP+CPR+ERY	AMP+CPR+FUS	AMP+CPR+DOX	AMP+ERY+FUS	AMP+ERY+DOX	AMP+FUS+DOX	CPR+ERY+FUS	CPR+ERY+DOX	CPR+FUS+DOX	ERY+FUS+DOX	AMP+CPR+ERY+FUS	AMP+CPR+ERY+DOX	AMP+CPR+FUS+DOX	AMP+ERY+FUS+DOX	CPR+ERY+FUS+DOX	AMP+CPR+ERY+FUS+DOX
	60.31013476	88.33862829	92.2257522	103.6182389	107.753369	58.72254015	49.2708141	39.81908806	73.4908621	97.30891422	105.9801906	94.91683797	102.0930667	109.8673145	97.7109101	46.61251615	36.71774045	58.72254015	44.10190142	76.14916005	39.81908806	102.0930667	112.5584003	107.1762287	109.8673145	43.65885176	61.97157098	50.30459664	40.40982093	97.90693328	48.97544766
	62.48760167	96.88521438	104.2684099	117.5362031	118.8057925	64.53084705	55.82225749	46.12180123	79.13112478	98.42338012	113.1897712	116.881369	117.4966353	114.4203038	108.3316802	66.4352311	40.58718508	61.2180123	47.88732394	87.06605832	53.10454275	115.0355701	113.1897712	107.9600077	102.1149779	55.64372148	60.72207895	51.51755604	50.24796667	103.0378773	52.78714541
	56.37453712	92.58317025	95.40117417	113.6131194	99.22412273	47.09927702	52.38934932	49.56797743	74.39605008	91.33072407	111.0567515	122.6418787	112.6223092	111.0567515	102.3276318	48.50996297	59.16064186	45.0537824	42.5145477	71.57467819	41.38599894	106.0469667	121.0763209	102.6027397	108.2387476	35.46111797	47.87515429	36.02539235	43.92523364	107.925636	43.36095927
AMP1CPR2ERY3FUS1DOX1	AMP	CPR	ERY	FUS	DOX	AMP+CPR	AMP+ERY	AMP+FUS	AMP+DOX	CPR+ERY	CPR+FUS	CPR+DOX	ERY+FUS	ERY+DOX	FUS+DOX	AMP+CPR+ERY	AMP+CPR+FUS	AMP+CPR+DOX	AMP+ERY+FUS	AMP+ERY+DOX	AMP+FUS+DOX	CPR+ERY+FUS	CPR+ERY+DOX	CPR+FUS+DOX	ERY+FUS+DOX	AMP+CPR+ERY+FUS	AMP+CPR+ERY+DOX	AMP+CPR+FUS+DOX	AMP+ERY+FUS+DOX	CPR+ERY+FUS+DOX	AMP+CPR+ERY+FUS+DOX
	60.31013476	88.33862829	99.06634883	65.33136422	108.9348348	58.72254015	54.69817242	3.599778475	73.4908621	98.80396188	70.39805644	105.9801906	105.4231228	106.0588002	58.27949049	51.92911205	17.81428835	66.10670113	11.31622669	71.71866347	1.827579841	71.89310409	110.4653336	59.12913474	59.51529599	20.76795274	72.90012922	38.48993908	4.485877792	58.83012521	21.35868562
	62.48760167	96.88521438	82.75002596	86.92719698	111.1882563	64.53084705	65.66157508	5.673477485	74.68756199	100.269179	88.27148625	114.1126706	94.38155572	94.71388514	61.53540964	65.16564174	10.49394961	53.91787344	4.721285459	72.14838326	-0.039674668	67.10248029	113.1897712	43.66467987	70.70308443	21.28545923	57.23070819	4.721285459	-0.674469351	41.81888098	5.990874826
	56.37453712	92.58317025	91.48265823	63.3926997	104.5847293	47.09927702	64.52124846	32.11073885	74.96032446	96.9667319	81.3111546	107.6125245	95.906837	103.3939088	57.78522306	52.74202081	17.75700935	69.88185505	10.10403809	69.88185505	-0.335037912	71.037182	108.5518591	60.33268102	74.70055701	16.91059778	63.67483689	6.683124669	0.476106507	55.00978474	5.836713102
AMP1CPR2ERY3FUS1DOX2	AMP	CPR	ERY	FUS	DOX	AMP+CPR	AMP+ERY	AMP+FUS	AMP+DOX	CPR+ERY	CPR+FUS	CPR+DOX	ERY+FUS	ERY+DOX	FUS+DOX	AMP+CPR+ERY	AMP+CPR+FUS	AMP+CPR+DOX	AMP+ERY+FUS	AMP+ERY+DOX	AMP+FUS+DOX	CPR+ERY+FUS	CPR+ERY+DOX	CPR+FUS+DOX	ERY+FUS+DOX	AMP+CPR+ERY+FUS	AMP+CPR+ERY+DOX	AMP+CPR+FUS+DOX	AMP+ERY+FUS+DOX	CPR+ERY+FUS+DOX	AMP+CPR+ERY+FUS+DOX
	60.31013476	88.33862829	99.06634883	65.33136422	113.9560642	58.72254015	54.69817242	3.599778475	82.64722171	98.80396188	70.39805644	117.3425528	105.4231228	116.2296385	61.19623408	51.92911205	17.81428835	61.3808381	11.31622669	81.76112239	0.941480524	71.89310409	117.0435433	57.03606802	65.55423123	20.76795274	70.53719771	1.532213402	13.97452464	54.34498225	14.86062396
	62.48760167	96.88521438	82.75002596	86.92719698	121.6623686	64.53084705	65.66157508	5.673477485	87.70085301	100.269179	88.27148625	121.188233	94.38155572	106.6777443	62.17020432	65.16564174	10.49394961	56.45705217	4.721285459	88.33564769	4.086490776	67.10248029	122.1111325	50.12497597	81.0052965	21.28545923	57.23070819	4.403888117	3.769093434	41.20361469	2.816901408
	56.37453712	92.58317025	91.48265823	63.3926997	102.8919062	47.09927702	64.52124846	32.11073885	80.03879386	96.9667319	81.3111546	115.1272016	95.906837	107.1374447	51.01393052	52.74202081	17.75700935	57.18568154	10.10403809	73.54963851	0.229236466	71.037182	112.6223092	55.00978474	71.29734257	16.91059778	59.72491624	7.282666196	-0.335037912	58.76712329	8.129077764
AMP1CPR2ERY3FUS1DOX3	AMP	CPR	ERY	FUS	DOX	AMP+CPR	AMP+ERY	AMP+FUS	AMP+DOX	CPR+ERY	CPR+FUS	CPR+DOX	ERY+FUS	ERY+DOX	FUS+DOX	AMP+CPR+ERY	AMP+CPR+FUS	AMP+CPR+DOX	AMP+ERY+FUS	AMP+ERY+DOX	AMP+FUS+DOX	CPR+ERY+FUS	CPR+ERY+DOX	CPR+FUS+DOX	ERY+FUS+DOX	AMP+CPR+ERY+FUS	AMP+CPR+ERY+DOX	AMP+CPR+FUS+DOX	AMP+ERY+FUS+DOX	CPR+ERY+FUS+DOX	AMP+CPR+ERY+FUS+DOX
	60.31013476	88.33862829	99.06634883	65.33136422	107.753369	58.72254015	54.69817242	3.599778475	73.4908621	98.80396188	70.39805644	94.91683797	105.4231228	104.151768	80.39505261	51.92911205	17.81428835	58.72254015	11.31622669	68.46963264	3.341332841	71.89310409	115.2494861	64.41786582	76.04290822	20.76795274	54.58741001	22.79859701	7.181096548	69.80003738	18.07273399
	62.48760167	96.88521438	82.75002596	86.92719698	118.8057925	64.53084705	65.66157508	5.673477485	79.13112478	100.269179	88.27148625	116.881369	94.38155572	120.9679094	82.16623686	65.16564174	10.49394961	61.2180123	4.721285459	79.76591946	5.038682801	67.10248029	121.8034993	70.79407806	77.68200228	21.28545923	58.99623091	5.732989486	6.62566951	72.94751009	24.39992065
	56.37453712	92.58317025	91.48265823	63.3926997	99.22412273	47.09927702	64.52124846	32.11073885	74.39605008	96.9667319	81.3111546	122.6418787	95.906837	103.0535873	74.74872157	52.74202081	17.75700935	45.0537824	10.10403809	66.49620878	5.025568683	71.037182	121.3894325	86.0665362	80.93269346	16.91059778	41.95027332	5.87198025	-0.088167872	79.17808219	19.41456533
AMP1CPR2ERY3FUS2DOX1	AMP	CPR	ERY	FUS	DOX	AMP+CPR	AMP+ERY	AMP+FUS	AMP+DOX	CPR+ERY	CPR+FUS	CPR+DOX	ERY+FUS	ERY+DOX	FUS+DOX	AMP+CPR+ERY	AMP+CPR+FUS	AMP+CPR+DOX	AMP+ERY+FUS	AMP+ERY+DOX	AMP+FUS+DOX	CPR+ERY+FUS	CPR+ERY+DOX	CPR+FUS+DOX	ERY+FUS+DOX	AMP+CPR+ERY+FUS	AMP+CPR+ERY+DOX	AMP+CPR+FUS+DOX	AMP+ERY+FUS+DOX	CPR+ERY+FUS+DOX	AMP+CPR+ERY+FUS+DOX
	60.31013476	88.33862829	99.06634883	92.3943142	108.9348348	58.72254015	54.69817242	21.61713125	73.4908621	98.80396188	82.35843768	105.9801906	110.1907032	106.0588002	74.19235739	51.92911205	24.60771645	66.10670113	25.45689496	71.71866347	12.49769245	85.34853298	110.4653336	69.80003738	75.40723083	26.23223186	72.90012922	31.95495662	20.4356655	60.53074192	32.84105594
	62.48760167	96.88521438	82.75002596	105.1577068	111.1882563	64.53084705	65.66157508	30.74786749	74.68756199	100.269179	98.73101327	114.1126706	105.3484266	94.71388514	87.24459433	65.16564174	20.41261654	53.91787344	27.89129141	72.14838326	7.577861535	92.57835032	113.1897712	73.56277639	80.00830824	33.7433049	57.23070819	27.89129141	38.36540369	67.41011344	31.38266217
	56.37453712	92.58317025	91.48265823	101.1990831	104.5847293	47.09927702	64.52124846	40.29271733	74.96032446	96.9667319	91.33072407	107.6125245	110.8809806	103.3939088	67.13101746	52.74202081	17.40433786	69.88185505	30.98219009	69.88185505	42.585082	96.65362035	108.5518591	77.61252446	82.18762878	17.96861224	63.67483689	19.94357256	15.21777464	72.91585127	15.99365191
AMP1CPR2ERY3FUS2DOX2	AMP	CPR	ERY	FUS	DOX	AMP+CPR	AMP+ERY	AMP+FUS	AMP+DOX	CPR+ERY	CPR+FUS	CPR+DOX	ERY+FUS	ERY+DOX	FUS+DOX	AMP+CPR+ERY	AMP+CPR+FUS	AMP+CPR+DOX	AMP+ERY+FUS	AMP+ERY+DOX	AMP+FUS+DOX	CPR+ERY+FUS	CPR+ERY+DOX	CPR+FUS+DOX	ERY+FUS+DOX	AMP+CPR+ERY+FUS	AMP+CPR+ERY+DOX	AMP+CPR+FUS+DOX	AMP+ERY+FUS+DOX	CPR+ERY+FUS+DOX	AMP+CPR+ERY+FUS+DOX
	60.31013476	88.33862829	99.06634883	92.3943142	113.9560642	58.72254015	54.69817242	21.61713125	82.64722171	98.80396188	82.35843768	117.3425528	110.1907032	116.2296385	77.14602178	51.92911205	24.60771645	61.3808381	25.45689496	81.76112239	2.713679158	85.34853298	117.0435433	72.49112315	69.36829559	26.23223186	70.53719771	11.61159313	30.77349086	67.1089516	24.2754292
	62.48760167	96.88521438	82.75002596	105.1577068	121.6623686	64.53084705	65.66157508	30.74786749	87.70085301	100.269179	98.73101327	121.188233	105.3484266	106.6777443	83.11842888	65.16564174	20.41261654	56.45705217	27.89129141	88.33564769	2.499504067	92.57835032	122.1111325	74.17804268	73.02939038	33.7433049	57.23070819	10.17655227	4.721285459	68.94827918	25.03471533
	56.37453712	92.58317025	91.48265823	101.1990831	102.8919062	47.09927702	64.52124846	40.29271733	80.03879386	96.9667319	91.33072407	115.1272016	110.8809806	107.1374447	64.30964557	52.74202081	17.40433786	57.18568154	30.98219009	73.54963851	3.050608358	96.65362035	112.6223092	82.30919765	75.72152134	17.96861224	59.72491624	5.025568683	1.886792453	79.80430528	21.42479281
AMP1CPR2ERY3FUS2DOX3	AMP	CPR	ERY	FUS	DOX	AMP+CPR	AMP+ERY	AMP+FUS	AMP+DOX	CPR+ERY	CPR+FUS	CPR+DOX	ERY+FUS	ERY+DOX	FUS+DOX	AMP+CPR+ERY	AMP+CPR+FUS	AMP+CPR+DOX	AMP+ERY+FUS	AMP+ERY+DOX	AMP+FUS+DOX	CPR+ERY+FUS	CPR+ERY+DOX	CPR+FUS+DOX	ERY+FUS+DOX	AMP+CPR+ERY+FUS	AMP+CPR+ERY+DOX	AMP+CPR+FUS+DOX	AMP+ERY+FUS+DOX	CPR+ERY+FUS+DOX	AMP+CPR+ERY+FUS+DOX
	60.31013476	88.33862829	99.06634883	92.3943142	107.753369	58.72254015	54.69817242	21.61713125	73.4908621	98.80396188	82.35843768	94.91683797	110.1907032	104.151768	102.7321396	51.92911205	24.60771645	58.72254015	25.45689496	68.46963264	5.999630792	85.34853298	115.2494861	79.6673519	111.462058	26.23223186	54.58741001	39.96677128	1.532213402	85.04952345	34.20712572
	62.48760167	96.88521438	82.75002596	105.1577068	118.8057925	64.53084705	65.66157508	30.74786749	79.13112478	100.269179	98.73101327	116.881369	105.3484266	120.9679094	98.80975997	65.16564174	20.41261654	61.2180123	27.89129141	79.76591946	4.086490776	92.57835032	121.8034993	80.58065757	118.9739329	33.7433049	58.99623091	33.28704622	9.799642928	93.80888291	47.25252926
	56.37453712	92.58317025	91.48265823	101.1990831	99.22412273	47.09927702	64.52124846	40.29271733	74.39605008	96.9667319	91.33072407	122.6418787	110.8809806	103.0535873	93.58137895	52.74202081	17.40433786	45.0537824	30.98219009	66.49620878	0.229236466	96.65362035	121.3894325	81.3111546	118.7083738	17.96861224	41.95027332	16.55792629	0.193969318	86.63405088	39.12890143
AMP1CPR2ERY3FUS3DOX1	AMP	CPR	ERY	FUS	DOX	AMP+CPR	AMP+ERY	AMP+FUS	AMP+DOX	CPR+ERY	CPR+FUS	CPR+DOX	ERY+FUS	ERY+DOX	FUS+DOX	AMP+CPR+ERY	AMP+CPR+FUS	AMP+CPR+DOX	AMP+ERY+FUS	AMP+ERY+DOX	AMP+FUS+DOX	CPR+ERY+FUS	CPR+ERY+DOX	CPR+FUS+DOX	ERY+FUS+DOX	AMP+CPR+ERY+FUS	AMP+CPR+ERY+DOX	AMP+CPR+FUS+DOX	AMP+ERY+FUS+DOX	CPR+ERY+FUS+DOX	AMP+CPR+ERY+FUS+DOX
	60.31013476	88.33862829	99.06634883	103.6182389	108.9348348	58.72254015	54.69817242	39.81908806	73.4908621	98.80396188	105.9801906	105.9801906	116.8653159	106.0588002	79.69355732	51.92911205	36.71774045	66.10670113	40.70518737	71.71866347	47.20324903	103.8871239	110.4653336	90.43169501	97.79499404	60.79010522	72.90012922	33.3210264	63.74376961	76.37824706	38.34225586
	62.48760167	96.88521438	82.75002596	117.5362031	111.1882563	64.53084705	65.66157508	46.12180123	74.68756199	100.269179	113.1897712	114.1126706	110.6656974	94.71388514	70.24399921	65.16564174	40.58718508	53.91787344	55.1874628	72.14838326	49.93056933	116.5737358	113.1897712	84.88752163	110.0010385	58.18290022	57.23070819	39.45645705	61.03947629	86.11805422	39.00019837
	56.37453712	92.58317025	91.48265823	113.6131194	104.5847293	47.09927702	64.52124846	49.56797743	74.96032446	96.9667319	111.0567515	107.6125245	124.4938383	103.3939088	58.59636748	52.74202081	59.16064186	69.88185505	45.33591959	69.88185505	48.43942867	107.925636	108.5518591	92.27005871	98.96973	47.31087992	63.67483689	39.41103862	60.28919062	86.32093933	37.1539411
AMP1CPR2ERY3FUS3DOX2	AMP	CPR	ERY	FUS	DOX	AMP+CPR	AMP+ERY	AMP+FUS	AMP+DOX	CPR+ERY	CPR+FUS	CPR+DOX	ERY+FUS	ERY+DOX	FUS+DOX	AMP+CPR+ERY	AMP+CPR+FUS	AMP+CPR+DOX	AMP+ERY+FUS	AMP+ERY+DOX	AMP+FUS+DOX	CPR+ERY+FUS	CPR+ERY+DOX	CPR+FUS+DOX	ERY+FUS+DOX	AMP+CPR+ERY+FUS	AMP+CPR+ERY+DOX	AMP+CPR+FUS+DOX	AMP+ERY+FUS+DOX	CPR+ERY+FUS+DOX	AMP+CPR+ERY+FUS+DOX
	60.31013476	88.33862829	99.06634883	103.6182389	113.9560642	58.72254015	54.69817242	39.81908806	82.64722171	98.80396188	105.9801906	117.3425528	116.8653159	116.2296385	86.78235186	51.92911205	36.71774045	61.3808381	40.70518737	81.76112239	35.09322503	103.8871239	117.0435433	90.73070454	92.70957489	60.79010522	70.53719771	57.24570796	37.16079011	90.43169501	58.72254015
	62.48760167	96.88521438	82.75002596	117.5362031	121.6623686	64.53084705	65.66157508	46.12180123	87.70085301	100.269179	113.1897712	121.188233	110.6656974	106.6777443	76.90934338	65.16564174	40.58718508	56.45705217	55.1874628	88.33564769	38.50426503	116.5737358	122.1111325	89.1943857	103.6867795	58.18290022	57.23070819	53.10454275	55.64372148	89.1943857	66.75262845
	56.37453712	92.58317025	91.48265823	113.6131194	102.8919062	47.09927702	64.52124846	49.56797743	80.03879386	96.9667319	111.0567515	115.1272016	124.4938383	107.1374447	76.93528478	52.74202081	59.16064186	57.18568154	45.33591959	73.54963851	32.0754717	107.925636	112.6223092	82.87671233	103.0535873	47.31087992	59.72491624	47.5930171	48.15729148	87.88649706	52.9536237
AMP1CPR2ERY3FUS3DOX3	AMP	CPR	ERY	FUS	DOX	AMP+CPR	AMP+ERY	AMP+FUS	AMP+DOX	CPR+ERY	CPR+FUS	CPR+DOX	ERY+FUS	ERY+DOX	FUS+DOX	AMP+CPR+ERY	AMP+CPR+FUS	AMP+CPR+DOX	AMP+ERY+FUS	AMP+ERY+DOX	AMP+FUS+DOX	CPR+ERY+FUS	CPR+ERY+DOX	CPR+FUS+DOX	ERY+FUS+DOX	AMP+CPR+ERY+FUS	AMP+CPR+ERY+DOX	AMP+CPR+FUS+DOX	AMP+ERY+FUS+DOX	CPR+ERY+FUS+DOX	AMP+CPR+ERY+FUS+DOX
	60.31013476	88.33862829	99.06634883	103.6182389	107.753369	58.72254015	54.69817242	39.81908806	73.4908621	98.80396188	105.9801906	94.91683797	116.8653159	104.151768	97.7109101	51.92911205	36.71774045	58.72254015	40.70518737	68.46963264	39.81908806	103.8871239	115.2494861	107.1762287	114.0047676	60.79010522	54.58741001	50.30459664	51.33837918	103.5881144	48.38471479
	62.48760167	96.88521438	82.75002596	117.5362031	118.8057925	64.53084705	65.66157508	46.12180123	79.13112478	100.269179	113.1897712	116.881369	110.6656974	120.9679094	108.3316802	65.16564174	40.58718508	61.2180123	55.1874628	79.76591946	53.10454275	116.5737358	121.8034993	107.9600077	116.3152975	58.18290022	58.99623091	51.51755604	76.59194604	82.7340896	47.70878794
	56.37453712	92.58317025	91.48265823	113.6131194	99.22412273	47.09927702	64.52124846	49.56797743	74.39605008	96.9667319	111.0567515	122.6418787	124.4938383	103.0535873	102.3276318	52.74202081	59.16064186	45.0537824	45.33591959	66.49620878	41.38599894	107.925636	121.3894325	102.6027397	124.8341598	47.31087992	41.95027332	36.02539235	49.00370305	82.56360078	41.66813613
AMP1CPR3ERY1FUS1DOX1	AMP	CPR	ERY	FUS	DOX	AMP+CPR	AMP+ERY	AMP+FUS	AMP+DOX	CPR+ERY	CPR+FUS	CPR+DOX	ERY+FUS	ERY+DOX	FUS+DOX	AMP+CPR+ERY	AMP+CPR+FUS	AMP+CPR+DOX	AMP+ERY+FUS	AMP+ERY+DOX	AMP+FUS+DOX	CPR+ERY+FUS	CPR+ERY+DOX	CPR+FUS+DOX	ERY+FUS+DOX	AMP+CPR+ERY+FUS	AMP+CPR+ERY+DOX	AMP+CPR+FUS+DOX	AMP+ERY+FUS+DOX	CPR+ERY+FUS+DOX	AMP+CPR+ERY+FUS+DOX
	60.31013476	92.70957489	94.46187927	65.33136422	108.9348348	82.05648883	52.51984493	3.599778475	73.4908621	91.7560588	102.2447358	104.4696067	61.19623408	105.0950711	58.27949049	77.92135869	24.86616208	72.30939634	5.113531475	71.71866347	1.827579841	72.22884386	105.1052841	63.32936035	49.71386376	22.09710172	66.10670113	3.009045597	2.12294628	40.58402861	2.12294628
	62.48760167	82.75002596	92.14441579	86.92719698	111.1882563	76.59194604	48.02618528	5.673477485	74.68756199	80.75604943	94.71388514	105.3484266	80.26185281	102.6185281	61.53540964	75.00495933	17.73457647	78.81372744	4.146002777	81.67030351	-0.039674668	81.0052965	102.0251324	70.70308443	47.6294386	16.78238445	62.62463	-0.61495735	-0.35707201	48.10468377	4.721285459
	56.37453712	88.76008668	100.9169459	63.3926997	104.5847293	70.44612943	50.13225181	32.11073885	74.96032446	87.73912234	94.54555123	102.3729444	71.01040381	109.6631987	57.78522306	63.95697408	25.90372068	82.86016576	6.718391818	71.57467819	-0.335037912	76.06184279	102.7132659	64.15059224	47.62828425	19.73196967	76.08887321	-0.335037912	-0.370305061	48.83612725	5.836713102
AMP1CPR3ERY1FUS1DOX2	AMP	CPR	ERY	FUS	DOX	AMP+CPR	AMP+ERY	AMP+FUS	AMP+DOX	CPR+ERY	CPR+FUS	CPR+DOX	ERY+FUS	ERY+DOX	FUS+DOX	AMP+CPR+ERY	AMP+CPR+FUS	AMP+CPR+DOX	AMP+ERY+FUS	AMP+ERY+DOX	AMP+FUS+DOX	CPR+ERY+FUS	CPR+ERY+DOX	CPR+FUS+DOX	ERY+FUS+DOX	AMP+CPR+ERY+FUS	AMP+CPR+ERY+DOX	AMP+CPR+FUS+DOX	AMP+ERY+FUS+DOX	CPR+ERY+FUS+DOX	AMP+CPR+ERY+FUS+DOX
	60.31013476	92.70957489	94.46187927	65.33136422	113.9560642	82.05648883	52.51984493	3.599778475	82.64722171	91.7560588	102.2447358	118.1366706	61.19623408	107.1626361	61.19623408	77.92135869	24.86616208	76.14916005	5.113531475	74.08159498	0.941480524	72.22884386	106.6944776	66.82558602	45.57873362	22.09710172	68.76499908	50.89532952	3.599778475	40.9018673	2.12294628
	62.48760167	82.75002596	92.14441579	86.92719698	121.6623686	76.59194604	48.02618528	5.673477485	87.70085301	80.75604943	94.71388514	107.3424032	80.26185281	118.1709978	62.17020432	75.00495933	17.73457647	87.38345566	4.146002777	89.9226344	4.086490776	81.0052965	95.04621456	80.67296708	44.13806784	16.78238445	68.6570125	2.182106725	7.002578853	56.74524873	2.816901408
	56.37453712	88.76008668	100.9169459	63.3926997	102.8919062	70.44612943	50.13225181	32.11073885	80.03879386	87.73912234	94.54555123	109.1793733	71.01040381	101.4812202	51.01393052	63.95697408	25.90372068	74.67818727	6.718391818	75.52459884	0.229236466	76.06184279	104.4148731	65.51187802	46.78187269	19.73196967	73.8317757	26.7853994	0.758243696	43.43352432	1.886792453
AMP1CPR3ERY1FUS1DOX3	AMP	CPR	ERY	FUS	DOX	AMP+CPR	AMP+ERY	AMP+FUS	AMP+DOX	CPR+ERY	CPR+FUS	CPR+DOX	ERY+FUS	ERY+DOX	FUS+DOX	AMP+CPR+ERY	AMP+CPR+FUS	AMP+CPR+DOX	AMP+ERY+FUS	AMP+ERY+DOX	AMP+FUS+DOX	CPR+ERY+FUS	CPR+ERY+DOX	CPR+FUS+DOX	ERY+FUS+DOX	AMP+CPR+ERY+FUS	AMP+CPR+ERY+DOX	AMP+CPR+FUS+DOX	AMP+ERY+FUS+DOX	CPR+ERY+FUS+DOX	AMP+CPR+ERY+FUS+DOX
	60.31013476	92.70957489	94.46187927	65.33136422	107.753369	82.05648883	52.51984493	3.599778475	73.4908621	91.7560588	102.2447358	105.4231228	61.19623408	103.6182389	80.39505261	77.92135869	24.86616208	66.99280044	5.113531475	64.92523537	3.341332841	72.22884386	102.8804132	80.49264998	73.89699095	22.09710172	66.99280044	30.77349086	1.532213402	68.7326182	13.6422374
	62.48760167	82.75002596	92.14441579	86.92719698	118.8057925	76.59194604	48.02618528	5.673477485	79.13112478	80.75604943	94.71388514	116.9799564	80.26185281	113.0926403	82.16623686	75.00495933	17.73457647	88.65304503	4.146002777	86.43126364	5.038682801	81.0052965	105.0160972	88.6488732	74.866098	16.78238445	65.16564174	34.55663559	2.182106725	73.3617198	4.403888117
	56.37453712	88.76008668	100.9169459	63.3926997	99.22412273	70.44612943	50.13225181	32.11073885	74.39605008	87.73912234	94.54555123	109.1793733	71.01040381	98.65984835	74.74872157	63.95697408	25.90372068	67.62475754	6.718391818	69.03544348	5.025568683	76.06184279	101.6923016	88.31341478	71.64521248	19.73196967	54.08217246	23.39975313	1.040380885	74.70055701	3.614882737
AMP1CPR3ERY1FUS2DOX1	AMP	CPR	ERY	FUS	DOX	AMP+CPR	AMP+ERY	AMP+FUS	AMP+DOX	CPR+ERY	CPR+FUS	CPR+DOX	ERY+FUS	ERY+DOX	FUS+DOX	AMP+CPR+ERY	AMP+CPR+FUS	AMP+CPR+DOX	AMP+ERY+FUS	AMP+ERY+DOX	AMP+FUS+DOX	CPR+ERY+FUS	CPR+ERY+DOX	CPR+FUS+DOX	ERY+FUS+DOX	AMP+CPR+ERY+FUS	AMP+CPR+ERY+DOX	AMP+CPR+FUS+DOX	AMP+ERY+FUS+DOX	CPR+ERY+FUS+DOX	AMP+CPR+ERY+FUS+DOX
	60.31013476	92.70957489	94.46187927	92.3943142	108.9348348	82.05648883	52.51984493	21.61713125	73.4908621	91.7560588	102.2447358	104.4696067	87.66845117	105.0950711	74.19235739	77.92135869	47.79398191	72.30939634	15.41443603	71.71866347	12.49769245	74.27493047	105.1052841	79.53913389	65.33136422	43.36348532	66.10670113	27.81982647	6.59036367	68.7326182	10.72549382
	62.48760167	82.75002596	92.14441579	105.1577068	111.1882563	76.59194604	48.02618528	30.74786749	74.68756199	80.75604943	95.04621456	105.3484266	89.9226344	102.6185281	87.24459433	75.00495933	46.75659591	78.81372744	11.70402698	81.67030351	7.577861535	73.44480216	102.0251324	83.89626129	64.39198572	37.23467566	62.62463	39.9523904	65.40368974	66.71513137	5.732989486
	56.37453712	88.76008668	100.9169459	101.1990831	104.5847293	70.44612943	50.13225181	40.29271733	74.96032446	87.73912234	109.5196948	102.3729444	91.88855581	109.6631987	67.13101746	63.95697408	54.08217246	82.86016576	12.3611356	71.57467819	42.585082	80.25205057	102.7132659	85.59084323	53.024158	35.46111797	76.08887321	21.70692999	1.604655264	77.08280712	3.897019926
AMP1CPR3ERY1FUS2DOX2	AMP	CPR	ERY	FUS	DOX	AMP+CPR	AMP+ERY	AMP+FUS	AMP+DOX	CPR+ERY	CPR+FUS	CPR+DOX	ERY+FUS	ERY+DOX	FUS+DOX	AMP+CPR+ERY	AMP+CPR+FUS	AMP+CPR+DOX	AMP+ERY+FUS	AMP+ERY+DOX	AMP+FUS+DOX	CPR+ERY+FUS	CPR+ERY+DOX	CPR+FUS+DOX	ERY+FUS+DOX	AMP+CPR+ERY+FUS	AMP+CPR+ERY+DOX	AMP+CPR+FUS+DOX	AMP+ERY+FUS+DOX	CPR+ERY+FUS+DOX	AMP+CPR+ERY+FUS+DOX
	60.31013476	92.70957489	94.46187927	92.3943142	113.9560642	82.05648883	52.51984493	21.61713125	82.64722171	91.7560588	102.2447358	118.1366706	87.66845117	107.1626361	77.14602178	77.92135869	47.79398191	76.14916005	15.41443603	74.08159498	2.713679158	74.27493047	106.6944776	79.22129519	67.3989293	43.36348532	68.76499908	21.91249769	9.839394499	82.71752086	24.01698357
	62.48760167	82.75002596	92.14441579	105.1577068	121.6623686	76.59194604	48.02618528	30.74786749	87.70085301	80.75604943	95.04621456	107.3424032	89.9226344	118.1709978	83.11842888	75.00495933	46.75659591	87.38345566	11.70402698	89.9226344	2.499504067	73.44480216	95.04621456	86.32256724	81.84883952	37.23467566	68.6570125	13.98532037	5.990874826	78.01433171	26.3638167
	56.37453712	88.76008668	100.9169459	101.1990831	102.8919062	70.44612943	50.13225181	40.29271733	80.03879386	87.73912234	109.5196948	109.1793733	91.88855581	101.4812202	64.30964557	63.95697408	54.08217246	74.67818727	12.3611356	75.52459884	3.050608358	80.25205057	104.4148731	93.41823644	61.48827367	35.46111797	73.8317757	25.33944631	3.897019926	75.3811999	20.54311409
AMP1CPR3ERY1FUS2DOX3	AMP	CPR	ERY	FUS	DOX	AMP+CPR	AMP+ERY	AMP+FUS	AMP+DOX	CPR+ERY	CPR+FUS	CPR+DOX	ERY+FUS	ERY+DOX	FUS+DOX	AMP+CPR+ERY	AMP+CPR+FUS	AMP+CPR+DOX	AMP+ERY+FUS	AMP+ERY+DOX	AMP+FUS+DOX	CPR+ERY+FUS	CPR+ERY+DOX	CPR+FUS+DOX	ERY+FUS+DOX	AMP+CPR+ERY+FUS	AMP+CPR+ERY+DOX	AMP+CPR+FUS+DOX	AMP+ERY+FUS+DOX	CPR+ERY+FUS+DOX	AMP+CPR+ERY+FUS+DOX
	60.31013476	92.70957489	94.46187927	92.3943142	107.753369	82.05648883	52.51984493	21.61713125	73.4908621	91.7560588	102.2447358	105.4231228	87.66845117	103.6182389	102.7321396	77.92135869	47.79398191	66.99280044	15.41443603	64.92523537	5.999630792	74.27493047	102.8804132	93.98092968	88.55455049	43.36348532	66.99280044	53.40594425	0.941480524	83.35319825	26.63836072
	62.48760167	82.75002596	92.14441579	105.1577068	118.8057925	76.59194604	48.02618528	30.74786749	79.13112478	80.75604943	95.04621456	116.9799564	89.9226344	113.0926403	98.80975997	75.00495933	46.75659591	88.65304503	11.70402698	86.43126364	4.086490776	73.44480216	105.0160972	107.0100737	83.25729022	37.23467566	65.16564174	48.66097996	3.769093434	79.01131997	36.46101964
	56.37453712	88.76008668	100.9169459	101.1990831	99.22412273	70.44612943	50.13225181	40.29271733	74.39605008	87.73912234	109.5196948	109.1793733	91.88855581	98.65984835	93.58137895	63.95697408	54.08217246	67.62475754	12.3611356	69.03544348	0.229236466	80.25205057	101.6923016	104.7551946	80.32093105	35.46111797	54.08217246	79.47451949	2.451066831	80.14570012	28.12555105
AMP1CPR3ERY1FUS3DOX1	AMP	CPR	ERY	FUS	DOX	AMP+CPR	AMP+ERY	AMP+FUS	AMP+DOX	CPR+ERY	CPR+FUS	CPR+DOX	ERY+FUS	ERY+DOX	FUS+DOX	AMP+CPR+ERY	AMP+CPR+FUS	AMP+CPR+DOX	AMP+ERY+FUS	AMP+ERY+DOX	AMP+FUS+DOX	CPR+ERY+FUS	CPR+ERY+DOX	CPR+FUS+DOX	ERY+FUS+DOX	AMP+CPR+ERY+FUS	AMP+CPR+ERY+DOX	AMP+CPR+FUS+DOX	AMP+ERY+FUS+DOX	CPR+ERY+FUS+DOX	AMP+CPR+ERY+FUS+DOX
	60.31013476	92.70957489	94.46187927	103.6182389	108.9348348	82.05648883	52.51984493	39.81908806	73.4908621	91.7560588	118.4545093	104.4696067	96.52944434	105.0950711	79.69355732	77.92135869	60.19937235	72.30939634	37.16079011	71.71866347	47.20324903	104.7874454	105.1052841	103.8339293	72.01402991	60.49473879	66.10670113	29.18589625	43.06811888	86.98847835	18.36810042
	62.48760167	82.75002596	92.14441579	117.5362031	111.1882563	76.59194604	48.02618528	46.12180123	74.68756199	80.75604943	110.0010385	105.3484266	110.870859	102.6185281	70.24399921	75.00495933	63.89605237	78.81372744	45.48700655	81.67030351	49.93056933	102.6897913	102.0251324	109.6687091	65.66157508	54.37413212	62.62463	30.56933148	39.77385439	91.72292034	26.3043047
	56.37453712	88.76008668	100.9169459	113.6131194	104.5847293	70.44612943	50.13225181	49.56797743	74.96032446	87.73912234	118.0277309	102.3729444	106.5596896	109.6631987	58.59636748	63.95697408	62.26415094	82.86016576	39.41103862	71.57467819	48.43942867	103.3939088	102.7132659	99.65037289	56.05713278	61.69987656	76.08887321	34.6147064	38.28248986	80.59237201	21.35425851
AMP1CPR3ERY1FUS3DOX2	AMP	CPR	ERY	FUS	DOX	AMP+CPR	AMP+ERY	AMP+FUS	AMP+DOX	CPR+ERY	CPR+FUS	CPR+DOX	ERY+FUS	ERY+DOX	FUS+DOX	AMP+CPR+ERY	AMP+CPR+FUS	AMP+CPR+DOX	AMP+ERY+FUS	AMP+ERY+DOX	AMP+FUS+DOX	CPR+ERY+FUS	CPR+ERY+DOX	CPR+FUS+DOX	ERY+FUS+DOX	AMP+CPR+ERY+FUS	AMP+CPR+ERY+DOX	AMP+CPR+FUS+DOX	AMP+ERY+FUS+DOX	CPR+ERY+FUS+DOX	AMP+CPR+ERY+FUS+DOX
	60.31013476	92.70957489	94.46187927	103.6182389	113.9560642	82.05648883	52.51984493	39.81908806	82.64722171	91.7560588	118.4545093	118.1366706	96.52944434	107.1626361	86.78235186	77.92135869	60.19937235	76.14916005	37.16079011	74.08159498	35.09322503	104.7874454	106.6944776	99.70202622	68.1742662	60.49473879	68.76499908	32.25032306	25.16152852	83.49225268	26.34299428
	62.48760167	82.75002596	92.14441579	117.5362031	121.6623686	76.59194604	48.02618528	46.12180123	87.70085301	80.75604943	110.0010385	107.3424032	110.870859	118.1709978	76.90934338	75.00495933	63.89605237	87.38345566	45.48700655	89.9226344	38.50426503	102.6897913	95.04621456	110.6656974	66.29636977	54.37413212	68.6570125	45.48700655	37.09581432	94.0492263	34.55663559
	56.37453712	88.76008668	100.9169459	113.6131194	102.8919062	70.44612943	50.13225181	49.56797743	80.03879386	87.73912234	118.0277309	109.1793733	106.5596896	101.4812202	76.93528478	63.95697408	62.26415094	74.67818727	39.41103862	75.52459884	32.0754717	103.3939088	104.4148731	102.032623	58.1026274	61.69987656	73.8317757	19.16769529	22.55334156	81.95365779	22.83547875
AMP1CPR3ERY1FUS3DOX3	AMP	CPR	ERY	FUS	DOX	AMP+CPR	AMP+ERY	AMP+FUS	AMP+DOX	CPR+ERY	CPR+FUS	CPR+DOX	ERY+FUS	ERY+DOX	FUS+DOX	AMP+CPR+ERY	AMP+CPR+FUS	AMP+CPR+DOX	AMP+ERY+FUS	AMP+ERY+DOX	AMP+FUS+DOX	CPR+ERY+FUS	CPR+ERY+DOX	CPR+FUS+DOX	ERY+FUS+DOX	AMP+CPR+ERY+FUS	AMP+CPR+ERY+DOX	AMP+CPR+FUS+DOX	AMP+ERY+FUS+DOX	CPR+ERY+FUS+DOX	AMP+CPR+ERY+FUS+DOX
	60.31013476	92.70957489	94.46187927	103.6182389	107.753369	82.05648883	52.51984493	39.81908806	73.4908621	91.7560588	118.4545093	105.4231228	96.52944434	103.6182389	97.7109101	77.92135869	60.19937235	66.99280044	37.16079011	64.92523537	39.81908806	104.7874454	102.8804132	107.0123162	90.03138268	60.49473879	66.99280044	37.8622854	44.54495108	92.70957489	34.35480893
	62.48760167	82.75002596	92.14441579	117.5362031	118.8057925	76.59194604	48.02618528	46.12180123	79.13112478	80.75604943	110.0010385	116.9799564	110.870859	113.0926403	108.3316802	75.00495933	63.89605237	88.65304503	45.48700655	86.43126364	53.10454275	102.6897913	105.0160972	124.6235331	94.36619718	54.37413212	65.16564174	39.45645705	51.83495338	103.0221207	25.80837135
	56.37453712	88.76008668	100.9169459	113.6131194	99.22412273	70.44612943	50.13225181	49.56797743	74.39605008	87.73912234	118.0277309	109.1793733	106.5596896	98.65984835	102.3276318	63.95697408	62.26415094	67.62475754	39.41103862	69.03544348	41.38599894	103.3939088	101.6923016	123.472874	88.22077235	61.69987656	54.08217246	35.17898078	41.95027332	103.7342302	19.09716099
AMP1CPR3ERY2FUS1DOX1	AMP	CPR	ERY	FUS	DOX	AMP+CPR	AMP+ERY	AMP+FUS	AMP+DOX	CPR+ERY	CPR+FUS	CPR+DOX	ERY+FUS	ERY+DOX	FUS+DOX	AMP+CPR+ERY	AMP+CPR+FUS	AMP+CPR+DOX	AMP+ERY+FUS	AMP+ERY+DOX	AMP+FUS+DOX	CPR+ERY+FUS	CPR+ERY+DOX	CPR+FUS+DOX	ERY+FUS+DOX	AMP+CPR+ERY+FUS	AMP+CPR+ERY+DOX	AMP+CPR+FUS+DOX	AMP+ERY+FUS+DOX	CPR+ERY+FUS+DOX	AMP+CPR+ERY+FUS+DOX
	60.31013476	92.70957489	92.2257522	65.33136422	108.9348348	82.05648883	49.2708141	3.599778475	73.4908621	91.12038141	102.2447358	104.4696067	70.39805644	101.7940572	58.27949049	81.46575595	24.86616208	72.30939634	3.304412036	73.78622854	1.827579841	66.50774732	109.5550258	63.32936035	56.43804896	22.68783459	64.92523537	3.009045597	8.03027506	63.32936035	7.181096548
	62.48760167	82.75002596	104.2684099	86.92719698	111.1882563	76.59194604	55.82225749	5.673477485	74.68756199	88.73195555	94.71388514	105.3484266	86.73332052	111.9592386	61.53540964	75.00495933	17.73457647	78.81372744	2.816901408	80.71811149	-0.039674668	81.0052965	105.3484266	70.70308443	36.89675062	20.90854989	74.37016465	-0.61495735	1.864709383	69.04143732	4.086490776
	56.37453712	88.76008668	95.40117417	63.3926997	104.5847293	70.44612943	52.38934932	32.11073885	74.96032446	86.03751512	94.54555123	102.3729444	81.93737769	103.5420744	57.78522306	64.23911127	25.90372068	82.86016576	34.64997355	80.03879386	-0.335037912	85.59084323	104.0745517	64.15059224	51.25244618	26.7148651	75.52459884	-0.335037912	16.02891906	68.91509246	11.79686122
AMP1CPR3ERY2FUS1DOX2	AMP	CPR	ERY	FUS	DOX	AMP+CPR	AMP+ERY	AMP+FUS	AMP+DOX	CPR+ERY	CPR+FUS	CPR+DOX	ERY+FUS	ERY+DOX	FUS+DOX	AMP+CPR+ERY	AMP+CPR+FUS	AMP+CPR+DOX	AMP+ERY+FUS	AMP+ERY+DOX	AMP+FUS+DOX	CPR+ERY+FUS	CPR+ERY+DOX	CPR+FUS+DOX	ERY+FUS+DOX	AMP+CPR+ERY+FUS	AMP+CPR+ERY+DOX	AMP+CPR+FUS+DOX	AMP+ERY+FUS+DOX	CPR+ERY+FUS+DOX	AMP+CPR+ERY+FUS+DOX
	60.31013476	92.70957489	92.2257522	65.33136422	113.9560642	82.05648883	49.2708141	3.599778475	82.64722171	91.12038141	102.2447358	118.1366706	70.39805644	107.1762287	61.19623408	81.46575595	24.86616208	76.14916005	3.304412036	74.9676943	0.941480524	66.50774732	112.0977354	66.82558602	49.85983928	22.68783459	73.4908621	50.89532952	21.35868562	63.32936035	1.827579841
	62.48760167	82.75002596	104.2684099	86.92719698	121.6623686	76.59194604	55.82225749	5.673477485	87.70085301	88.73195555	94.71388514	107.3424032	86.73332052	117.4966353	62.17020432	75.00495933	17.73457647	87.38345566	2.816901408	88.33564769	4.086490776	81.0052965	110.333368	80.67296708	40.03076331	20.90854989	74.05276731	2.182106725	9.85915493	70.37075501	2.182106725
	56.37453712	88.76008668	95.40117417	63.3926997	102.8919062	70.44612943	52.38934932	32.11073885	80.03879386	86.03751512	94.54555123	109.1793733	81.93737769	111.369863	51.01393052	64.23911127	25.90372068	74.67818727	34.64997355	74.67818727	0.229236466	85.59084323	102.032623	65.51187802	48.43444227	26.7148651	77.78169635	26.7853994	-0.335037912	72.99894979	5.025568683
AMP1CPR3ERY2FUS1DOX3	AMP	CPR	ERY	FUS	DOX	AMP+CPR	AMP+ERY	AMP+FUS	AMP+DOX	CPR+ERY	CPR+FUS	CPR+DOX	ERY+FUS	ERY+DOX	FUS+DOX	AMP+CPR+ERY	AMP+CPR+FUS	AMP+CPR+DOX	AMP+ERY+FUS	AMP+ERY+DOX	AMP+FUS+DOX	CPR+ERY+FUS	CPR+ERY+DOX	CPR+FUS+DOX	ERY+FUS+DOX	AMP+CPR+ERY+FUS	AMP+CPR+ERY+DOX	AMP+CPR+FUS+DOX	AMP+ERY+FUS+DOX	CPR+ERY+FUS+DOX	AMP+CPR+ERY+FUS+DOX
	60.31013476	92.70957489	92.2257522	65.33136422	107.753369	82.05648883	49.2708141	3.599778475	73.4908621	91.12038141	102.2447358	105.4231228	70.39805644	109.8673145	80.39505261	81.46575595	24.86616208	66.99280044	3.304412036	76.14916005	3.341332841	66.50774732	106.6944776	80.49264998	59.63771332	22.68783459	71.71866347	30.77349086	9.507107255	76.36074692	7.439542182
	62.48760167	82.75002596	104.2684099	86.92719698	118.8057925	76.59194604	55.82225749	5.673477485	79.13112478	88.73195555	94.71388514	116.9799564	86.73332052	114.4203038	82.16623686	75.00495933	17.73457647	88.65304503	2.816901408	87.06605832	5.038682801	81.0052965	109.0040503	88.6488732	63.71851567	20.90854989	72.78317794	34.55663559	4.086490776	82.66694361	4.086490776
	56.37453712	88.76008668	95.40117417	63.3926997	99.22412273	70.44612943	52.38934932	32.11073885	74.39605008	86.03751512	94.54555123	109.1793733	81.93737769	111.0567515	74.74872157	64.23911127	25.90372068	67.62475754	34.64997355	71.57467819	5.025568683	85.59084323	105.7761589	88.31341478	80.43052838	26.7148651	62.82842532	23.39975313	3.861752777	81.84730734	1.922059601
AMP1CPR3ERY2FUS2DOX1	AMP	CPR	ERY	FUS	DOX	AMP+CPR	AMP+ERY	AMP+FUS	AMP+DOX	CPR+ERY	CPR+FUS	CPR+DOX	ERY+FUS	ERY+DOX	FUS+DOX	AMP+CPR+ERY	AMP+CPR+FUS	AMP+CPR+DOX	AMP+ERY+FUS	AMP+ERY+DOX	AMP+FUS+DOX	CPR+ERY+FUS	CPR+ERY+DOX	CPR+FUS+DOX	ERY+FUS+DOX	AMP+CPR+ERY+FUS	AMP+CPR+ERY+DOX	AMP+CPR+FUS+DOX	AMP+ERY+FUS+DOX	CPR+ERY+FUS+DOX	AMP+CPR+ERY+FUS+DOX
	60.31013476	92.70957489	92.2257522	92.3943142	108.9348348	82.05648883	49.2708141	21.61713125	73.4908621	91.12038141	102.2447358	104.4696067	90.13268548	101.7940572	74.19235739	81.46575595	47.79398191	72.30939634	22.79859701	73.78622854	12.49769245	91.7560588	109.5550258	79.53913389	62.3247991	24.16466679	64.92523537	27.81982647	17.51892191	77.94994041	2.750599963
	62.48760167	82.75002596	104.2684099	105.1577068	111.1882563	76.59194604	55.82225749	30.74786749	74.68756199	88.73195555	95.04621456	105.3484266	101.8073447	111.9592386	87.24459433	75.00495933	46.75659591	78.81372744	25.98690736	80.71811149	7.577861535	90.39360266	105.3484266	83.89626129	64.64141511	24.39992065	74.37016465	39.9523904	6.62566951	79.01131997	2.499504067
	56.37453712	88.76008668	95.40117417	101.1990831	104.5847293	70.44612943	52.38934932	40.29271733	74.96032446	86.03751512	109.5196948	102.3729444	98.84540117	103.5420744	67.13101746	64.23911127	54.08217246	82.86016576	40.01058014	80.03879386	42.585082	97.60844423	104.0745517	85.59084323	64.46183953	23.32921883	75.52459884	21.70692999	4.179157115	80.48602156	3.897019926
AMP1CPR3ERY2FUS2DOX2	AMP	CPR	ERY	FUS	DOX	AMP+CPR	AMP+ERY	AMP+FUS	AMP+DOX	CPR+ERY	CPR+FUS	CPR+DOX	ERY+FUS	ERY+DOX	FUS+DOX	AMP+CPR+ERY	AMP+CPR+FUS	AMP+CPR+DOX	AMP+ERY+FUS	AMP+ERY+DOX	AMP+FUS+DOX	CPR+ERY+FUS	CPR+ERY+DOX	CPR+FUS+DOX	ERY+FUS+DOX	AMP+CPR+ERY+FUS	AMP+CPR+ERY+DOX	AMP+CPR+FUS+DOX	AMP+ERY+FUS+DOX	CPR+ERY+FUS+DOX	AMP+CPR+ERY+FUS+DOX
	60.31013476	92.70957489	92.2257522	92.3943142	113.9560642	82.05648883	49.2708141	21.61713125	82.64722171	91.12038141	102.2447358	118.1366706	90.13268548	107.1762287	77.14602178	81.46575595	47.79398191	76.14916005	22.79859701	74.9676943	2.713679158	91.7560588	112.0977354	79.22129519	71.29508503	24.16466679	73.4908621	21.91249769	17.77736755	82.08184346	11.02086025
	62.48760167	82.75002596	104.2684099	105.1577068	121.6623686	76.59194604	55.82225749	30.74786749	87.70085301	88.73195555	95.04621456	107.3424032	101.8073447	117.4966353	83.11842888	75.00495933	46.75659591	87.38345566	25.98690736	88.33564769	2.499504067	90.39360266	110.333368	86.32256724	71.7169775	24.39992065	74.05276731	13.98532037	6.943066852	79.34364939	12.39383366
	56.37453712	88.76008668	95.40117417	101.1990831	102.8919062	70.44612943	52.38934932	40.29271733	80.03879386	86.03751512	109.5196948	109.1793733	98.84540117	111.369863	64.30964557	64.23911127	54.08217246	74.67818727	40.01058014	74.67818727	3.050608358	97.60844423	102.032623	93.41823644	66.9667319	23.32921883	77.78169635	25.33944631	11.23258685	86.27148611	17.15746782
AMP1CPR3ERY2FUS2DOX3	AMP	CPR	ERY	FUS	DOX	AMP+CPR	AMP+ERY	AMP+FUS	AMP+DOX	CPR+ERY	CPR+FUS	CPR+DOX	ERY+FUS	ERY+DOX	FUS+DOX	AMP+CPR+ERY	AMP+CPR+FUS	AMP+CPR+DOX	AMP+ERY+FUS	AMP+ERY+DOX	AMP+FUS+DOX	CPR+ERY+FUS	CPR+ERY+DOX	CPR+FUS+DOX	ERY+FUS+DOX	AMP+CPR+ERY+FUS	AMP+CPR+ERY+DOX	AMP+CPR+FUS+DOX	AMP+ERY+FUS+DOX	CPR+ERY+FUS+DOX	AMP+CPR+ERY+FUS+DOX
	60.31013476	92.70957489	92.2257522	92.3943142	107.753369	82.05648883	49.2708141	21.61713125	73.4908621	91.12038141	102.2447358	105.4231228	90.13268548	109.8673145	102.7321396	81.46575595	47.79398191	66.99280044	22.79859701	76.14916005	5.999630792	91.7560588	106.6944776	93.98092968	106.2792001	24.16466679	71.71866347	53.40594425	1.236846963	77.31426301	35.38859147
	62.48760167	82.75002596	104.2684099	105.1577068	118.8057925	76.59194604	55.82225749	30.74786749	79.13112478	88.73195555	95.04621456	116.9799564	101.8073447	114.4203038	98.80975997	75.00495933	46.75659591	88.65304503	25.98690736	87.06605832	4.086490776	90.39360266	109.0040503	107.0100737	107.0371082	24.39992065	72.78317794	48.66097996	3.451696092	74.35870807	40.58718508
	56.37453712	88.76008668	95.40117417	101.1990831	99.22412273	70.44612943	52.38934932	40.29271733	74.39605008	86.03751512	109.5196948	109.1793733	98.84540117	111.0567515	93.58137895	64.23911127	54.08217246	67.62475754	40.01058014	71.57467819	0.229236466	97.60844423	105.7761589	104.7551946	105.1076321	23.32921883	62.82842532	79.47451949	1.886792453	88.99405767	38.56462705
AMP1CPR3ERY2FUS3DOX1	AMP	CPR	ERY	FUS	DOX	AMP+CPR	AMP+ERY	AMP+FUS	AMP+DOX	CPR+ERY	CPR+FUS	CPR+DOX	ERY+FUS	ERY+DOX	FUS+DOX	AMP+CPR+ERY	AMP+CPR+FUS	AMP+CPR+DOX	AMP+ERY+FUS	AMP+ERY+DOX	AMP+FUS+DOX	CPR+ERY+FUS	CPR+ERY+DOX	CPR+FUS+DOX	ERY+FUS+DOX	AMP+CPR+ERY+FUS	AMP+CPR+ERY+DOX	AMP+CPR+FUS+DOX	AMP+ERY+FUS+DOX	CPR+ERY+FUS+DOX	AMP+CPR+ERY+FUS+DOX
	60.31013476	92.70957489	92.2257522	103.6182389	108.9348348	82.05648883	49.2708141	39.81908806	73.4908621	91.12038141	118.4545093	104.4696067	102.0930667	101.7940572	79.69355732	81.46575595	60.19937235	72.30939634	44.10190142	73.78622854	47.20324903	110.8263806	109.5550258	103.8339293	79.06933283	57.83644083	64.92523537	29.18589625	41.59128669	93.98092968	20.91563596
	62.48760167	82.75002596	104.2684099	117.5362031	111.1882563	76.59194604	55.82225749	46.12180123	74.68756199	88.73195555	110.0010385	105.3484266	117.4966353	111.9592386	70.24399921	75.00495933	63.89605237	78.81372744	47.88732394	80.71811149	49.93056933	106.3454149	105.3484266	109.6687091	74.73562776	60.40468161	74.37016465	30.56933148	41.53937711	104.3514384	25.35211268
	56.37453712	88.76008668	95.40117417	113.6131194	104.5847293	70.44612943	52.38934932	49.56797743	74.96032446	86.03751512	118.0277309	102.3729444	112.6223092	103.5420744	58.59636748	64.23911127	62.26415094	82.86016576	42.5145477	80.03879386	48.43942867	109.5196948	104.0745517	99.65037289	88.51272016	69.31758067	75.52459884	34.6147064	55.21072121	93.86490834	25.30417916
AMP1CPR3ERY2FUS3DOX2	AMP	CPR	ERY	FUS	DOX	AMP+CPR	AMP+ERY	AMP+FUS	AMP+DOX	CPR+ERY	CPR+FUS	CPR+DOX	ERY+FUS	ERY+DOX	FUS+DOX	AMP+CPR+ERY	AMP+CPR+FUS	AMP+CPR+DOX	AMP+ERY+FUS	AMP+ERY+DOX	AMP+FUS+DOX	CPR+ERY+FUS	CPR+ERY+DOX	CPR+FUS+DOX	ERY+FUS+DOX	AMP+CPR+ERY+FUS	AMP+CPR+ERY+DOX	AMP+CPR+FUS+DOX	AMP+ERY+FUS+DOX	CPR+ERY+FUS+DOX	AMP+CPR+ERY+FUS+DOX
	60.31013476	92.70957489	92.2257522	103.6182389	113.9560642	82.05648883	49.2708141	39.81908806	82.64722171	91.12038141	118.4545093	118.1366706	102.0930667	107.1762287	86.78235186	81.46575595	60.19937235	76.14916005	44.10190142	74.9676943	35.09322503	110.8263806	112.0977354	99.70202622	86.54457111	57.83644083	73.4908621	32.25032306	35.68395791	82.85657529	18.9588333
	62.48760167	82.75002596	104.2684099	117.5362031	121.6623686	76.59194604	55.82225749	46.12180123	87.70085301	88.73195555	110.0010385	107.3424032	117.4966353	117.4966353	76.90934338	75.00495933	63.89605237	87.38345566	47.88732394	88.33564769	38.50426503	106.3454149	110.333368	110.6656974	78.11959239	60.40468161	74.05276731	45.48700655	51.83495338	99.69882646	34.37809958
	56.37453712	88.76008668	95.40117417	113.6131194	102.8919062	70.44612943	52.38934932	49.56797743	80.03879386	86.03751512	118.0277309	109.1793733	112.6223092	111.369863	76.93528478	64.23911127	62.26415094	74.67818727	42.5145477	74.67818727	32.0754717	109.5196948	102.032623	102.032623	78.18003914	69.31758067	77.78169635	19.16769529	41.95027332	90.4616939	22.27120437
AMP1CPR3ERY2FUS3DOX3	AMP	CPR	ERY	FUS	DOX	AMP+CPR	AMP+ERY	AMP+FUS	AMP+DOX	CPR+ERY	CPR+FUS	CPR+DOX	ERY+FUS	ERY+DOX	FUS+DOX	AMP+CPR+ERY	AMP+CPR+FUS	AMP+CPR+DOX	AMP+ERY+FUS	AMP+ERY+DOX	AMP+FUS+DOX	CPR+ERY+FUS	CPR+ERY+DOX	CPR+FUS+DOX	ERY+FUS+DOX	AMP+CPR+ERY+FUS	AMP+CPR+ERY+DOX	AMP+CPR+FUS+DOX	AMP+ERY+FUS+DOX	CPR+ERY+FUS+DOX	AMP+CPR+ERY+FUS+DOX
	60.31013476	92.70957489	92.2257522	103.6182389	107.753369	82.05648883	49.2708141	39.81908806	73.4908621	91.12038141	118.4545093	105.4231228	102.0930667	109.8673145	97.7109101	81.46575595	60.19937235	66.99280044	44.10190142	76.14916005	39.81908806	110.8263806	106.6944776	107.0123162	109.8673145	57.83644083	71.71866347	37.8622854	40.40982093	93.02741359	30.21967879
	62.48760167	82.75002596	104.2684099	117.5362031	118.8057925	76.59194604	55.82225749	46.12180123	79.13112478	88.73195555	110.0010385	116.9799564	117.4966353	114.4203038	108.3316802	75.00495933	63.89605237	88.65304503	47.88732394	87.06605832	53.10454275	106.3454149	109.0040503	124.6235331	102.1149779	60.40468161	72.78317794	39.45645705	50.24796667	108.007062	29.9345368
	56.37453712	88.76008668	95.40117417	113.6131194	99.22412273	70.44612943	52.38934932	49.56797743	74.39605008	86.03751512	118.0277309	109.1793733	112.6223092	111.0567515	102.3276318	64.23911127	62.26415094	67.62475754	42.5145477	71.57467819	41.38599894	109.5196948	105.7761589	123.472874	108.2387476	69.31758067	62.82842532	35.17898078	43.92523364	117.347088	28.40768824
AMP1CPR3ERY3FUS1DOX1	AMP	CPR	ERY	FUS	DOX	AMP+CPR	AMP+ERY	AMP+FUS	AMP+DOX	CPR+ERY	CPR+FUS	CPR+DOX	ERY+FUS	ERY+DOX	FUS+DOX	AMP+CPR+ERY	AMP+CPR+FUS	AMP+CPR+DOX	AMP+ERY+FUS	AMP+ERY+DOX	AMP+FUS+DOX	CPR+ERY+FUS	CPR+ERY+DOX	CPR+FUS+DOX	ERY+FUS+DOX	AMP+CPR+ERY+FUS	AMP+CPR+ERY+DOX	AMP+CPR+FUS+DOX	AMP+ERY+FUS+DOX	CPR+ERY+FUS+DOX	AMP+CPR+ERY+FUS+DOX
	60.31013476	92.70957489	99.06634883	65.33136422	108.9348348	82.05648883	54.69817242	3.599778475	73.4908621	97.47715534	102.2447358	104.4696067	105.4231228	106.0588002	58.27949049	84.1240539	24.86616208	72.30939634	11.31622669	71.71866347	1.827579841	68.7326182	110.1907032	63.32936035	59.51529599	20.32490308	67.87889976	3.009045597	4.485877792	63.64719905	20.4356655
	62.48760167	82.75002596	82.75002596	86.92719698	111.1882563	76.59194604	65.66157508	5.673477485	74.68756199	91.39059092	94.71388514	105.3484266	94.38155572	94.71388514	61.53540964	74.68756199	17.73457647	78.81372744	4.721285459	72.14838326	-0.039674668	87.31955551	103.0221207	70.70308443	70.70308443	21.22594723	74.37016465	-0.61495735	-0.674469351	62.72717832	-0.35707201
	56.37453712	88.76008668	91.48265823	63.3926997	104.5847293	70.44612943	64.52124846	32.11073885	74.96032446	90.4616939	94.54555123	102.3729444	95.906837	103.3939088	57.78522306	68.47116911	25.90372068	82.86016576	10.10403809	69.88185505	-0.335037912	86.27148611	101.6923016	64.15059224	74.70055701	27.87868101	74.39605008	-0.335037912	0.476106507	58.02480624	28.72509258
AMP1CPR3ERY3FUS1DOX2	AMP	CPR	ERY	FUS	DOX	AMP+CPR	AMP+ERY	AMP+FUS	AMP+DOX	CPR+ERY	CPR+FUS	CPR+DOX	ERY+FUS	ERY+DOX	FUS+DOX	AMP+CPR+ERY	AMP+CPR+FUS	AMP+CPR+DOX	AMP+ERY+FUS	AMP+ERY+DOX	AMP+FUS+DOX	CPR+ERY+FUS	CPR+ERY+DOX	CPR+FUS+DOX	ERY+FUS+DOX	AMP+CPR+ERY+FUS	AMP+CPR+ERY+DOX	AMP+CPR+FUS+DOX	AMP+ERY+FUS+DOX	CPR+ERY+FUS+DOX	AMP+CPR+ERY+FUS+DOX
	60.31013476	92.70957489	99.06634883	65.33136422	113.9560642	82.05648883	54.69817242	3.599778475	82.64722171	97.47715534	102.2447358	118.1366706	105.4231228	116.2296385	61.19623408	84.1240539	24.86616208	76.14916005	11.31622669	81.76112239	0.941480524	68.7326182	113.6869289	66.82558602	65.55423123	20.32490308	74.08159498	50.89532952	13.97452464	66.82558602	1.827579841
	62.48760167	82.75002596	82.75002596	86.92719698	121.6623686	76.59194604	65.66157508	5.673477485	87.70085301	91.39059092	94.71388514	107.3424032	94.38155572	106.6777443	62.17020432	74.68756199	17.73457647	87.38345566	4.721285459	88.33564769	4.086490776	87.31955551	107.0100737	80.67296708	81.0052965	21.22594723	81.35290617	2.182106725	3.769093434	75.02336691	26.3638167
	56.37453712	88.76008668	91.48265823	63.3926997	102.8919062	70.44612943	64.52124846	32.11073885	80.03879386	90.4616939	94.54555123	109.1793733	95.906837	107.1374447	51.01393052	68.47116911	25.90372068	74.67818727	10.10403809	73.54963851	0.229236466	86.27148611	103.7342302	65.51187802	71.29734257	27.87868101	82.01375419	26.7853994	-0.335037912	62.10866357	0.229236466
AMP1CPR3ERY3FUS1DOX3	AMP	CPR	ERY	FUS	DOX	AMP+CPR	AMP+ERY	AMP+FUS	AMP+DOX	CPR+ERY	CPR+FUS	CPR+DOX	ERY+FUS	ERY+DOX	FUS+DOX	AMP+CPR+ERY	AMP+CPR+FUS	AMP+CPR+DOX	AMP+ERY+FUS	AMP+ERY+DOX	AMP+FUS+DOX	CPR+ERY+FUS	CPR+ERY+DOX	CPR+FUS+DOX	ERY+FUS+DOX	AMP+CPR+ERY+FUS	AMP+CPR+ERY+DOX	AMP+CPR+FUS+DOX	AMP+ERY+FUS+DOX	CPR+ERY+FUS+DOX	AMP+CPR+ERY+FUS+DOX
	60.31013476	92.70957489	99.06634883	65.33136422	107.753369	82.05648883	54.69817242	3.599778475	73.4908621	97.47715534	102.2447358	105.4231228	105.4231228	104.151768	80.39505261	84.1240539	24.86616208	66.99280044	11.31622669	68.46963264	3.341332841	68.7326182	106.0588002	80.49264998	76.04290822	20.32490308	79.69355732	30.77349086	7.181096548	76.67858562	14.52833672
	62.48760167	82.75002596	82.75002596	86.92719698	118.8057925	76.59194604	65.66157508	5.673477485	79.13112478	91.39059092	94.71388514	116.9799564	94.38155572	120.9679094	82.16623686	74.68756199	17.73457647	88.65304503	4.721285459	79.76591946	5.038682801	87.31955551	109.3363797	88.6488732	77.68200228	21.22594723	76.2745487	34.55663559	6.62566951	81.66995534	30.1130728
	56.37453712	88.76008668	91.48265823	63.3926997	99.22412273	70.44612943	64.52124846	32.11073885	74.39605008	90.4616939	94.54555123	109.1793733	95.906837	103.0535873	74.74872157	68.47116911	25.90372068	67.62475754	10.10403809	66.49620878	5.025568683	86.27148611	108.8390519	88.31341478	80.93269346	27.87868101	78.34597073	23.39975313	-0.088167872	82.52795023	21.07212132
AMP1CPR3ERY3FUS2DOX1	AMP	CPR	ERY	FUS	DOX	AMP+CPR	AMP+ERY	AMP+FUS	AMP+DOX	CPR+ERY	CPR+FUS	CPR+DOX	ERY+FUS	ERY+DOX	FUS+DOX	AMP+CPR+ERY	AMP+CPR+FUS	AMP+CPR+DOX	AMP+ERY+FUS	AMP+ERY+DOX	AMP+FUS+DOX	CPR+ERY+FUS	CPR+ERY+DOX	CPR+FUS+DOX	ERY+FUS+DOX	AMP+CPR+ERY+FUS	AMP+CPR+ERY+DOX	AMP+CPR+FUS+DOX	AMP+ERY+FUS+DOX	CPR+ERY+FUS+DOX	AMP+CPR+ERY+FUS+DOX
	60.31013476	92.70957489	99.06634883	92.3943142	108.9348348	82.05648883	54.69817242	21.61713125	73.4908621	97.47715534	102.2447358	104.4696067	110.1907032	106.0588002	74.19235739	84.1240539	47.79398191	72.30939634	25.45689496	71.71866347	12.49769245	102.2447358	110.1907032	79.53913389	75.40723083	41.88665313	67.87889976	27.81982647	20.4356655	76.99642431	21.61713125
	62.48760167	82.75002596	82.75002596	105.1577068	111.1882563	76.59194604	65.66157508	30.74786749	74.68756199	91.39059092	95.04621456	105.3484266	105.3484266	94.71388514	87.24459433	74.68756199	46.75659591	78.81372744	27.89129141	72.14838326	7.577861535	96.04320282	103.0221207	83.89626129	80.00830824	36.46101964	74.37016465	39.9523904	38.36540369	86.32256724	26.93909939
	56.37453712	88.76008668	91.48265823	101.1990831	104.5847293	70.44612943	64.52124846	40.29271733	74.96032446	90.4616939	109.5196948	102.3729444	110.8809806	103.3939088	67.13101746	68.47116911	54.08217246	82.86016576	30.98219009	69.88185505	42.585082	106.4568018	101.6923016	85.59084323	82.18762878	43.36095927	74.39605008	21.70692999	15.21777464	81.16666445	11.54999118
AMP1CPR3ERY3FUS2DOX2	AMP	CPR	ERY	FUS	DOX	AMP+CPR	AMP+ERY	AMP+FUS	AMP+DOX	CPR+ERY	CPR+FUS	CPR+DOX	ERY+FUS	ERY+DOX	FUS+DOX	AMP+CPR+ERY	AMP+CPR+FUS	AMP+CPR+DOX	AMP+ERY+FUS	AMP+ERY+DOX	AMP+FUS+DOX	CPR+ERY+FUS	CPR+ERY+DOX	CPR+FUS+DOX	ERY+FUS+DOX	AMP+CPR+ERY+FUS	AMP+CPR+ERY+DOX	AMP+CPR+FUS+DOX	AMP+ERY+FUS+DOX	CPR+ERY+FUS+DOX	AMP+CPR+ERY+FUS+DOX
	60.31013476	92.70957489	99.06634883	92.3943142	113.9560642	82.05648883	54.69817242	21.61713125	82.64722171	97.47715534	102.2447358	118.1366706	110.1907032	116.2296385	77.14602178	84.1240539	47.79398191	76.14916005	25.45689496	81.76112239	2.713679158	102.2447358	113.6869289	79.22129519	69.36829559	41.88665313	74.08159498	21.91249769	30.77349086	77.31426301	21.9494185
	62.48760167	82.75002596	82.75002596	105.1577068	121.6623686	76.59194604	65.66157508	30.74786749	87.70085301	91.39059092	95.04621456	107.3424032	105.3484266	106.6777443	83.11842888	74.68756199	46.75659591	87.38345566	27.89129141	88.33564769	2.499504067	96.04320282	107.0100737	86.32256724	73.02939038	36.46101964	81.35290617	13.98532037	4.721285459	85.99023782	11.76353898
	56.37453712	88.76008668	91.48265823	101.1990831	102.8919062	70.44612943	64.52124846	40.29271733	80.03879386	90.4616939	109.5196948	109.1793733	110.8809806	107.1374447	64.30964557	68.47116911	54.08217246	74.67818727	30.98219009	73.54963851	3.050608358	106.4568018	103.7342302	93.41823644	75.72152134	43.36095927	82.01375419	25.33944631	1.886792453	86.952129	8.129077764
AMP1CPR3ERY3FUS2DOX3	AMP	CPR	ERY	FUS	DOX	AMP+CPR	AMP+ERY	AMP+FUS	AMP+DOX	CPR+ERY	CPR+FUS	CPR+DOX	ERY+FUS	ERY+DOX	FUS+DOX	AMP+CPR+ERY	AMP+CPR+FUS	AMP+CPR+DOX	AMP+ERY+FUS	AMP+ERY+DOX	AMP+FUS+DOX	CPR+ERY+FUS	CPR+ERY+DOX	CPR+FUS+DOX	ERY+FUS+DOX	AMP+CPR+ERY+FUS	AMP+CPR+ERY+DOX	AMP+CPR+FUS+DOX	AMP+ERY+FUS+DOX	CPR+ERY+FUS+DOX	AMP+CPR+ERY+FUS+DOX
	60.31013476	92.70957489	99.06634883	92.3943142	107.753369	82.05648883	54.69817242	21.61713125	73.4908621	97.47715534	102.2447358	105.4231228	110.1907032	104.151768	102.7321396	84.1240539	47.79398191	66.99280044	25.45689496	68.46963264	5.999630792	102.2447358	106.0588002	93.98092968	111.462058	41.88665313	79.69355732	53.40594425	1.532213402	103.5160906	51.4860624
	62.48760167	82.75002596	82.75002596	105.1577068	118.8057925	76.59194604	65.66157508	30.74786749	79.13112478	91.39059092	95.04621456	116.9799564	105.3484266	120.9679094	98.80975997	74.68756199	46.75659591	88.65304503	27.89129141	79.76591946	4.086490776	96.04320282	109.3363797	107.0100737	118.9739329	36.46101964	76.2745487	48.66097996	9.799642928	110.6656974	38.68280103
	56.37453712	88.76008668	91.48265823	101.1990831	99.22412273	70.44612943	64.52124846	40.29271733	74.39605008	90.4616939	109.5196948	109.1793733	110.8809806	103.0535873	93.58137895	68.47116911	54.08217246	67.62475754	30.98219009	66.49620878	0.229236466	106.4568018	108.8390519	104.7551946	118.7083738	43.36095927	78.34597073	79.47451949	0.193969318	106.1164803	46.18233116
AMP1CPR3ERY3FUS3DOX1	AMP	CPR	ERY	FUS	DOX	AMP+CPR	AMP+ERY	AMP+FUS	AMP+DOX	CPR+ERY	CPR+FUS	CPR+DOX	ERY+FUS	ERY+DOX	FUS+DOX	AMP+CPR+ERY	AMP+CPR+FUS	AMP+CPR+DOX	AMP+ERY+FUS	AMP+ERY+DOX	AMP+FUS+DOX	CPR+ERY+FUS	CPR+ERY+DOX	CPR+FUS+DOX	ERY+FUS+DOX	AMP+CPR+ERY+FUS	AMP+CPR+ERY+DOX	AMP+CPR+FUS+DOX	AMP+ERY+FUS+DOX	CPR+ERY+FUS+DOX	AMP+CPR+ERY+FUS+DOX
	60.31013476	92.70957489	99.06634883	103.6182389	108.9348348	82.05648883	54.69817242	39.81908806	73.4908621	97.47715534	118.4545093	104.4696067	116.8653159	106.0588002	79.69355732	84.1240539	60.19937235	72.30939634	40.70518737	71.71866347	47.20324903	111.462058	110.1907032	103.8339293	97.79499404	61.67620454	67.87889976	29.18589625	63.74376961	94.93444577	36.97618608
	62.48760167	82.75002596	82.75002596	117.5362031	111.1882563	76.59194604	65.66157508	46.12180123	74.68756199	91.39059092	110.0010385	105.3484266	110.6656974	94.71388514	70.24399921	74.68756199	63.89605237	78.81372744	55.1874628	72.14838326	49.93056933	112.6596739	103.0221207	109.6687091	110.0010385	58.50029756	74.37016465	30.56933148	61.03947629	101.0281441	35.96508629
	56.37453712	88.76008668	91.48265823	113.6131194	104.5847293	70.44612943	64.52124846	49.56797743	74.96032446	90.4616939	118.0277309	102.3729444	124.4938383	103.3939088	58.59636748	68.47116911	62.26415094	82.86016576	45.33591959	69.88185505	48.43942867	115.3051593	101.6923016	99.65037289	98.96973	68.18903192	74.39605008	34.6147064	60.28919062	91.48265823	33.20402045
AMP1CPR3ERY3FUS3DOX2	AMP	CPR	ERY	FUS	DOX	AMP+CPR	AMP+ERY	AMP+FUS	AMP+DOX	CPR+ERY	CPR+FUS	CPR+DOX	ERY+FUS	ERY+DOX	FUS+DOX	AMP+CPR+ERY	AMP+CPR+FUS	AMP+CPR+DOX	AMP+ERY+FUS	AMP+ERY+DOX	AMP+FUS+DOX	CPR+ERY+FUS	CPR+ERY+DOX	CPR+FUS+DOX	ERY+FUS+DOX	AMP+CPR+ERY+FUS	AMP+CPR+ERY+DOX	AMP+CPR+FUS+DOX	AMP+ERY+FUS+DOX	CPR+ERY+FUS+DOX	AMP+CPR+ERY+FUS+DOX
	60.31013476	92.70957489	99.06634883	103.6182389	113.9560642	82.05648883	54.69817242	39.81908806	82.64722171	97.47715534	118.4545093	118.1366706	116.8653159	116.2296385	86.78235186	84.1240539	60.19937235	76.14916005	40.70518737	81.76112239	35.09322503	111.462058	113.6869289	99.70202622	92.70957489	61.67620454	74.08159498	32.25032306	37.16079011	100.973381	36.97618608
	62.48760167	82.75002596	82.75002596	117.5362031	121.6623686	76.59194604	65.66157508	46.12180123	87.70085301	91.39059092	110.0010385	107.3424032	110.6656974	106.6777443	76.90934338	74.68756199	63.89605237	87.38345566	55.1874628	88.33564769	38.50426503	112.6596739	107.0100737	110.6656974	103.6867795	58.50029756	81.35290617	45.48700655	55.64372148	108.3393914	51.51755604
	56.37453712	88.76008668	91.48265823	113.6131194	102.8919062	70.44612943	64.52124846	49.56797743	80.03879386	90.4616939	118.0277309	109.1793733	124.4938383	107.1374447	76.93528478	68.47116911	62.26415094	74.67818727	45.33591959	73.54963851	32.0754717	115.3051593	103.7342302	102.032623	103.0535873	68.18903192	82.01375419	19.16769529	48.15729148	98.62940856	45.90019397
AMP1CPR3ERY3FUS3DOX3	AMP	CPR	ERY	FUS	DOX	AMP+CPR	AMP+ERY	AMP+FUS	AMP+DOX	CPR+ERY	CPR+FUS	CPR+DOX	ERY+FUS	ERY+DOX	FUS+DOX	AMP+CPR+ERY	AMP+CPR+FUS	AMP+CPR+DOX	AMP+ERY+FUS	AMP+ERY+DOX	AMP+FUS+DOX	CPR+ERY+FUS	CPR+ERY+DOX	CPR+FUS+DOX	ERY+FUS+DOX	AMP+CPR+ERY+FUS	AMP+CPR+ERY+DOX	AMP+CPR+FUS+DOX	AMP+ERY+FUS+DOX	CPR+ERY+FUS+DOX	AMP+CPR+ERY+FUS+DOX
	60.31013476	92.70957489	99.06634883	103.6182389	107.753369	82.05648883	54.69817242	39.81908806	73.4908621	97.47715534	118.4545093	105.4231228	116.8653159	104.151768	97.7109101	84.1240539	60.19937235	66.99280044	40.70518737	68.46963264	39.81908806	111.462058	106.0588002	107.0123162	114.0047676	61.67620454	79.69355732	37.8622854	51.33837918	100.6555423	34.61325457
	62.48760167	82.75002596	82.75002596	117.5362031	118.8057925	76.59194604	65.66157508	46.12180123	79.13112478	91.39059092	110.0010385	116.9799564	110.6656974	120.9679094	108.3316802	74.68756199	63.89605237	88.65304503	55.1874628	79.76591946	53.10454275	112.6596739	109.3363797	124.6235331	116.3152975	58.50029756	76.2745487	39.45645705	76.59194604	112.3273445	38.82166237
	56.37453712	88.76008668	91.48265823	113.6131194	99.22412273	70.44612943	64.52124846	49.56797743	74.39605008	90.4616939	118.0277309	109.1793733	124.4938383	103.0535873	102.3276318	68.47116911	62.26415094	67.62475754	45.33591959	66.49620878	41.38599894	115.3051593	108.8390519	123.472874	124.8341598	68.18903192	78.34597073	35.17898078	49.00370305	119.7293381	44.48950802
AMP2CPR1ERY1FUS1DOX1	AMP	CPR	ERY	FUS	DOX	AMP+CPR	AMP+ERY	AMP+FUS	AMP+DOX	CPR+ERY	CPR+FUS	CPR+DOX	ERY+FUS	ERY+DOX	FUS+DOX	AMP+CPR+ERY	AMP+CPR+FUS	AMP+CPR+DOX	AMP+ERY+FUS	AMP+ERY+DOX	AMP+FUS+DOX	CPR+ERY+FUS	CPR+ERY+DOX	CPR+FUS+DOX	ERY+FUS+DOX	AMP+CPR+ERY+FUS	AMP+CPR+ERY+DOX	AMP+CPR+FUS+DOX	AMP+ERY+FUS+DOX	CPR+ERY+FUS+DOX	AMP+CPR+ERY+FUS+DOX
	65.31489441	89.73601625	94.46187927	65.33136422	108.9348348	71.59409456	71.89310409	27.43412446	76.97626612	87.07771829	66.40206757	105.9811704	61.19623408	105.0950711	58.27949049	88.03961876	28.03214352	84.15249486	21.75294338	80.86339002	12.78265745	74.48772383	93.28041351	55.3258261	49.71386376	16.96879088	87.74060923	2.915342927	39.69351523	45.28336718	8.895533545
	83.96462219	97.22277326	92.14441579	86.92719698	111.1882563	85.15195478	78.42722553	23.97615843	91.04018458	105.7925015	82.16623686	114.6796271	80.26185281	102.6185281	61.53540964	74.12036147	5.82580273	87.65621996	8.594501057	93.80888291	3.36473755	83.43582622	97.5401706	59.05574291	47.6294386	10.44029994	71.04402999	1.826571813	4.902903288	45.40765721	23.66852528
	70.66536204	102.0454946	100.9169459	63.3926997	104.5847293	52.81800391	69.09980431	21.87866928	87.57338552	96.96702522	74.67818727	104.3025921	71.01040381	109.6631987	57.78522306	62.52446184	31.21330724	77.86692759	6.164383562	90.39138943	12.73972603	50.76706048	99.50625992	56.09239993	47.62828425	7.71037182	77.86692759	10.54794521	3.659491194	46.4997355	13.67906067
AMP2CPR1ERY1FUS1DOX2	AMP	CPR	ERY	FUS	DOX	AMP+CPR	AMP+ERY	AMP+FUS	AMP+DOX	CPR+ERY	CPR+FUS	CPR+DOX	ERY+FUS	ERY+DOX	FUS+DOX	AMP+CPR+ERY	AMP+CPR+FUS	AMP+CPR+DOX	AMP+ERY+FUS	AMP+ERY+DOX	AMP+FUS+DOX	CPR+ERY+FUS	CPR+ERY+DOX	CPR+FUS+DOX	ERY+FUS+DOX	AMP+CPR+ERY+FUS	AMP+CPR+ERY+DOX	AMP+CPR+FUS+DOX	AMP+ERY+FUS+DOX	CPR+ERY+FUS+DOX	AMP+CPR+ERY+FUS+DOX
	65.31489441	89.73601625	94.46187927	65.33136422	113.9560642	71.59409456	71.89310409	27.43412446	83.25546627	87.07771829	66.40206757	115.7282629	61.19623408	107.1626361	61.19623408	88.03961876	28.03214352	81.76041861	21.75294338	88.93664736	18.76284807	74.48772383	100.6645745	55.91655898	45.57873362	16.96879088	87.14259017	2.317323865	4.316950103	43.80653498	7.905064474
	83.96462219	97.22277326	92.14441579	86.92719698	121.6623686	85.15195478	78.42722553	23.97615843	95.96231494	105.7925015	82.16623686	119.7579845	80.26185281	118.1709978	62.17020432	74.12036147	5.82580273	77.81195924	8.594501057	106.421842	3.36473755	83.43582622	105.4751041	52.64828407	44.13806784	10.44029994	70.4287637	0.903672371	3.057104403	50.80341202	12.90136512
	70.66536204	102.0454946	100.9169459	63.3926997	102.8919062	52.81800391	69.09980431	21.87866928	91.64383562	96.96702522	74.67818727	108.53465	71.01040381	101.4812202	51.01393052	62.52446184	31.21330724	77.86692759	6.164383562	97.90606654	5.851272016	50.76706048	97.24916241	54.68171398	46.78187269	7.71037182	78.18003914	3.659491194	4.598825832	41.70340328	20.56751468
AMP2CPR1ERY1FUS1DOX3	AMP	CPR	ERY	FUS	DOX	AMP+CPR	AMP+ERY	AMP+FUS	AMP+DOX	CPR+ERY	CPR+FUS	CPR+DOX	ERY+FUS	ERY+DOX	FUS+DOX	AMP+CPR+ERY	AMP+CPR+FUS	AMP+CPR+DOX	AMP+ERY+FUS	AMP+ERY+DOX	AMP+FUS+DOX	CPR+ERY+FUS	CPR+ERY+DOX	CPR+FUS+DOX	ERY+FUS+DOX	AMP+CPR+ERY+FUS	AMP+CPR+ERY+DOX	AMP+CPR+FUS+DOX	AMP+ERY+FUS+DOX	CPR+ERY+FUS+DOX	AMP+CPR+ERY+FUS+DOX
	65.31489441	89.73601625	94.46187927	65.33136422	107.753369	71.59409456	71.89310409	27.43412446	86.54457111	87.07771829	66.40206757	111.0023999	61.19623408	103.6182389	80.39505261	88.03961876	28.03214352	73.38815175	21.75294338	76.67725659	12.18463839	74.48772383	108.9348348	82.46261769	73.89699095	16.96879088	85.94655205	32.21827696	14.2777051	60.31013476	37.80601757
	83.96462219	97.22277326	92.14441579	86.92719698	118.8057925	85.15195478	78.42722553	23.97615843	88.27148625	105.7925015	82.16623686	115.6318191	80.26185281	113.0926403	82.16623686	74.12036147	5.82580273	75.65852721	8.594501057	88.27148625	4.902903288	83.43582622	110.870859	84.70541559	74.866098	10.44029994	65.50663334	18.80407614	14.13189771	53.6004761	22.49567391
	70.66536204	102.0454946	100.9169459	63.3926997	99.22412273	52.81800391	69.09980431	21.87866928	93.52250489	96.96702522	74.67818727	109.0989244	71.01040381	98.65984835	74.74872157	62.52446184	31.21330724	90.39138943	6.164383562	78.49315068	17.12328767	50.76706048	101.4812202	79.26291659	71.64521248	7.71037182	74.42270059	17.43639922	10.23483366	61.20613648	20.31311155
AMP2CPR1ERY1FUS2DOX1	AMP	CPR	ERY	FUS	DOX	AMP+CPR	AMP+ERY	AMP+FUS	AMP+DOX	CPR+ERY	CPR+FUS	CPR+DOX	ERY+FUS	ERY+DOX	FUS+DOX	AMP+CPR+ERY	AMP+CPR+FUS	AMP+CPR+DOX	AMP+ERY+FUS	AMP+ERY+DOX	AMP+FUS+DOX	CPR+ERY+FUS	CPR+ERY+DOX	CPR+FUS+DOX	ERY+FUS+DOX	AMP+CPR+ERY+FUS	AMP+CPR+ERY+DOX	AMP+CPR+FUS+DOX	AMP+ERY+FUS+DOX	CPR+ERY+FUS+DOX	AMP+CPR+ERY+FUS+DOX
	65.31489441	89.73601625	94.46187927	92.3943142	108.9348348	71.59409456	71.89310409	28.23771258	76.97626612	87.07771829	81.17038951	105.9811704	87.66845117	105.0950711	74.19235739	88.03961876	30.12521024	84.15249486	16.57634087	80.86339002	26.44365539	62.67306627	93.28041351	74.78309027	65.33136422	19.36086713	87.74060923	24.05157914	19.0618576	62.08233339	44.08521772
	83.96462219	97.22277326	92.14441579	105.1577068	111.1882563	85.15195478	78.42722553	38.43491636	91.04018458	105.7925015	86.43126364	114.6796271	89.9226344	102.6185281	87.24459433	74.12036147	24.28379158	87.65621996	17.82349548	93.80888291	36.0315324	80.89664749	97.5401706	81.53144217	64.39198572	15.42011152	71.04402999	31.41703519	16.90059604	64.07458838	31.41703519
	70.66536204	102.0454946	100.9169459	101.1990831	104.5847293	52.81800391	69.09980431	29.02152642	87.57338552	96.96702522	85.11726327	104.3025921	91.88855581	109.6631987	67.13101746	62.52446184	23.38551859	77.86692759	20	90.39138943	26.88845401	48.50996297	99.50625992	63.463234	53.024158	18.74755382	77.86692759	17.49510763	12.11350294	52.17774643	31.27201566
AMP2CPR1ERY1FUS2DOX2	AMP	CPR	ERY	FUS	DOX	AMP+CPR	AMP+ERY	AMP+FUS	AMP+DOX	CPR+ERY	CPR+FUS	CPR+DOX	ERY+FUS	ERY+DOX	FUS+DOX	AMP+CPR+ERY	AMP+CPR+FUS	AMP+CPR+DOX	AMP+ERY+FUS	AMP+ERY+DOX	AMP+FUS+DOX	CPR+ERY+FUS	CPR+ERY+DOX	CPR+FUS+DOX	ERY+FUS+DOX	AMP+CPR+ERY+FUS	AMP+CPR+ERY+DOX	AMP+CPR+FUS+DOX	AMP+ERY+FUS+DOX	CPR+ERY+FUS+DOX	AMP+CPR+ERY+FUS+DOX
	65.31489441	89.73601625	94.46187927	92.3943142	113.9560642	71.59409456	71.89310409	28.23771258	83.25546627	87.07771829	81.17038951	115.7282629	87.66845117	107.1626361	77.14602178	88.03961876	30.12521024	81.76041861	16.57634087	88.93664736	18.76284807	62.67306627	100.6645745	81.87188481	67.3989293	19.36086713	87.14259017	46.77630349	27.73313399	63.85453203	35.41394132
	83.96462219	97.22277326	92.14441579	105.1577068	121.6623686	85.15195478	78.42722553	38.43491636	95.96231494	105.7925015	86.43126364	119.7579845	89.9226344	118.1709978	83.11842888	74.12036147	24.28379158	77.81195924	17.82349548	106.421842	24.03383965	80.89664749	105.4751041	78.99226344	81.84883952	15.42011152	70.4287637	33.512786	57.20053836	62.17020432	26.80253797
	70.66536204	102.0454946	100.9169459	101.1990831	102.8919062	52.81800391	69.09980431	29.02152642	91.64383562	96.96702522	85.11726327	108.53465	91.88855581	101.4812202	64.30964557	62.52446184	23.38551859	77.86692759	20	97.90606654	13.36594912	48.50996297	97.24916241	70.79880092	61.48827367	18.74755382	78.18003914	33.09197652	23.69863014	49.63851173	30.64579256
AMP2CPR1ERY1FUS2DOX3	AMP	CPR	ERY	FUS	DOX	AMP+CPR	AMP+ERY	AMP+FUS	AMP+DOX	CPR+ERY	CPR+FUS	CPR+DOX	ERY+FUS	ERY+DOX	FUS+DOX	AMP+CPR+ERY	AMP+CPR+FUS	AMP+CPR+DOX	AMP+ERY+FUS	AMP+ERY+DOX	AMP+FUS+DOX	CPR+ERY+FUS	CPR+ERY+DOX	CPR+FUS+DOX	ERY+FUS+DOX	AMP+CPR+ERY+FUS	AMP+CPR+ERY+DOX	AMP+CPR+FUS+DOX	AMP+ERY+FUS+DOX	CPR+ERY+FUS+DOX	AMP+CPR+ERY+FUS+DOX
	65.31489441	89.73601625	94.46187927	92.3943142	107.753369	71.59409456	71.89310409	28.23771258	86.54457111	87.07771829	81.17038951	111.0023999	87.66845117	103.6182389	102.7321396	88.03961876	30.12521024	73.38815175	16.57634087	76.67725659	16.96879088	62.67306627	108.9348348	68.1742662	88.55455049	19.36086713	85.94655205	22.9489815	4.615959634	73.89699095	34.81592226
	83.96462219	97.22277326	92.14441579	105.1577068	118.8057925	85.15195478	78.42722553	38.43491636	88.27148625	105.7925015	86.43126364	115.6318191	89.9226344	113.0926403	98.80975997	74.12036147	24.28379158	75.65852721	17.82349548	88.27148625	11.67083253	80.89664749	110.870859	77.08787939	83.25729022	15.42011152	65.50663334	18.13112863	1.211305518	79.62705812	21.57277447
	70.66536204	102.0454946	100.9169459	101.1990831	99.22412273	52.81800391	69.09980431	29.02152642	93.52250489	96.96702522	85.11726327	109.0989244	91.88855581	98.65984835	93.58137895	62.52446184	23.38551859	90.39138943	20	78.49315068	11.54598826	48.50996297	101.4812202	62.82842532	80.32093105	18.74755382	74.42270059	26.51663405	0.528375734	71.3630753	20
AMP2CPR1ERY1FUS3DOX1	AMP	CPR	ERY	FUS	DOX	AMP+CPR	AMP+ERY	AMP+FUS	AMP+DOX	CPR+ERY	CPR+FUS	CPR+DOX	ERY+FUS	ERY+DOX	FUS+DOX	AMP+CPR+ERY	AMP+CPR+FUS	AMP+CPR+DOX	AMP+ERY+FUS	AMP+ERY+DOX	AMP+FUS+DOX	CPR+ERY+FUS	CPR+ERY+DOX	CPR+FUS+DOX	ERY+FUS+DOX	AMP+CPR+ERY+FUS	AMP+CPR+ERY+DOX	AMP+CPR+FUS+DOX	AMP+ERY+FUS+DOX	CPR+ERY+FUS+DOX	AMP+CPR+ERY+FUS+DOX
	65.31489441	89.73601625	94.46187927	103.6182389	108.9348348	71.59409456	71.89310409	66.21192301	76.97626612	87.07771829	105.9811704	105.9811704	96.52944434	105.0950711	79.69355732	88.03961876	63.2218277	84.15249486	59.03569426	80.86339002	62.92281816	84.71478678	93.28041351	77.33062581	72.01402991	54.25154177	87.74060923	63.81984676	60.23173239	58.24256969	59.33470379
	83.96462219	97.22277326	92.14441579	117.5362031	111.1882563	85.15195478	78.42722553	79.04249183	91.04018458	105.7925015	118.1709978	114.6796271	110.870859	102.6185281	70.24399921	74.12036147	63.66083445	87.65621996	85.50278793	93.80888291	61.50740242	93.41400516	97.5401706	71.51358857	65.66157508	47.66391079	71.04402999	58.7387041	57.81580465	67.56595914	59.04633724
	70.66536204	102.0454946	100.9169459	113.6131194	104.5847293	52.81800391	69.09980431	71.60469667	87.57338552	96.96702522	104.3025921	104.3025921	106.5596896	109.6631987	58.59636748	62.52446184	52.81800391	77.86692759	71.91780822	90.39138943	70.97847358	94.14565332	99.50625992	66.77834597	56.05713278	43.73776908	77.86692759	71.91780822	51.56555773	56.40980427	62.21135029
AMP2CPR1ERY1FUS3DOX2	AMP	CPR	ERY	FUS	DOX	AMP+CPR	AMP+ERY	AMP+FUS	AMP+DOX	CPR+ERY	CPR+FUS	CPR+DOX	ERY+FUS	ERY+DOX	FUS+DOX	AMP+CPR+ERY	AMP+CPR+FUS	AMP+CPR+DOX	AMP+ERY+FUS	AMP+ERY+DOX	AMP+FUS+DOX	CPR+ERY+FUS	CPR+ERY+DOX	CPR+FUS+DOX	ERY+FUS+DOX	AMP+CPR+ERY+FUS	AMP+CPR+ERY+DOX	AMP+CPR+FUS+DOX	AMP+ERY+FUS+DOX	CPR+ERY+FUS+DOX	AMP+CPR+ERY+FUS+DOX
	65.31489441	89.73601625	94.46187927	103.6182389	113.9560642	71.59409456	71.89310409	66.21192301	83.25546627	87.07771829	105.9811704	115.7282629	96.52944434	107.1626361	86.78235186	88.03961876	63.2218277	81.76041861	59.03569426	88.93664736	59.33470379	84.71478678	100.6645745	79.39819088	68.1742662	54.25154177	87.14259017	65.31489441	52.75649411	62.67306627	63.2218277
	83.96462219	97.22277326	92.14441579	117.5362031	121.6623686	85.15195478	78.42722553	79.04249183	95.96231494	105.7925015	118.1709978	119.7579845	110.870859	118.1709978	76.90934338	74.12036147	63.66083445	77.81195924	85.50278793	106.421842	62.43030187	93.41400516	105.4751041	79.44852212	66.29636977	47.66391079	70.4287637	77.19669294	54.12420688	64.53084705	73.19746203
	70.66536204	102.0454946	100.9169459	113.6131194	102.8919062	52.81800391	69.09980431	71.60469667	91.64383562	96.96702522	104.3025921	108.53465	106.5596896	101.4812202	76.93528478	62.52446184	52.81800391	77.86692759	71.91780822	97.90606654	55.63600783	94.14565332	97.24916241	69.88185505	58.1026274	43.73776908	78.18003914	72.85714286	40.03913894	56.97407865	63.15068493

	AMP	CPR	ERY	FUS	DOX	AMP+CPR	AMP+ERY	AMP+FUS	AMP+DOX	CPR+ERY	CPR+FUS	CPR+DOX	ERY+FUS	ERY+DOX	FUS+DOX	AMP+CPR+ERY	AMP+CPR+FUS	AMP+CPR+DOX	AMP+ERY+FUS	AMP+ERY+DOX	AMP+FUS+DOX	CPR+ERY+FUS	CPR+ERY+DOX	CPR+FUS+DOX	ERY+FUS+DOX	AMP+CPR+ERY+FUS	AMP+CPR+ERY+DOX	AMP+CPR+FUS+DOX	AMP+ERY+FUS+DOX	CPR+ERY+FUS+DOX	AMP+CPR+ERY+FUS+DOX
AMP2CPR1ERY1FUS3DOX3	AMP	CPR	ERY	FUS	DOX	AMP+CPR	AMP+ERY	AMP+FUS	AMP+DOX	CPR+ERY	CPR+FUS	CPR+DOX	ERY+FUS	ERY+DOX	FUS+DOX	AMP+CPR+ERY	AMP+CPR+FUS	AMP+CPR+DOX	AMP+ERY+FUS	AMP+ERY+DOX	AMP+FUS+DOX	CPR+ERY+FUS	CPR+ERY+DOX	CPR+FUS+DOX	ERY+FUS+DOX	AMP+CPR+ERY+FUS	AMP+CPR+ERY+DOX	AMP+CPR+FUS+DOX	AMP+ERY+FUS+DOX	CPR+ERY+FUS+DOX	AMP+CPR+ERY+FUS+DOX
	65.31489441	89.73601625	94.46187927	103.6182389	107.753369	71.59409456	71.89310409	66.21192301	86.54457111	87.07771829	105.9811704	111.0023999	96.52944434	103.6182389	97.7109101	88.03961876	63.2218277	73.38815175	59.03569426	76.67725659	65.91291347	84.71478678	108.9348348	87.07771829	90.03138268	54.25154177	85.94655205	65.31489441	51.26144646	79.98892376	68.30498972
	83.96462219	97.22277326	92.14441579	117.5362031	118.8057925	85.19515478	78.42722553	79.04249183	88.27148625	105.7925015	118.1709978	115.6318191	110.870859	113.0926403	108.3316802	74.12036147	63.66083445	75.65852721	85.50278793	88.27148625	73.19746203	93.41400516	110.870859	101.3489387	94.36619718	47.66391079	65.50663334	72.88982888	50.12497597	94.04879984	67.96769852
	70.66536204	102.0454946	100.9169459	113.6131194	99.22412273	52.81800391	69.09980431	71.60469667	93.52250489	96.96702522	104.3025921	109.0989244	106.5596896	98.65984835	102.3276318	62.52446184	52.81800391	90.39138943	71.91780822	78.49315068	61.27201566	94.14565332	101.4812202	95.27420208	88.22077235	43.73776908	74.42270059	76.30136986	51.25244618	78.91024511	60.95890411
AMP2CPR1ERY2FUS1DOX1	AMP	CPR	ERY	FUS	DOX	AMP+CPR	AMP+ERY	AMP+FUS	AMP+DOX	CPR+ERY	CPR+FUS	CPR+DOX	ERY+FUS	ERY+DOX	FUS+DOX	AMP+CPR+ERY	AMP+CPR+FUS	AMP+CPR+DOX	AMP+ERY+FUS	AMP+ERY+DOX	AMP+FUS+DOX	CPR+ERY+FUS	CPR+ERY+DOX	CPR+FUS+DOX	ERY+FUS+DOX	AMP+CPR+ERY+FUS	AMP+CPR+ERY+DOX	AMP+CPR+FUS+DOX	AMP+ERY+FUS+DOX	CPR+ERY+FUS+DOX	AMP+CPR+ERY+FUS+DOX
	65.31489441	89.73601625	92.2257522	65.33136422	108.9348348	71.59409456	72.19211362	27.43412446	76.97626612	89.14528337	66.40206757	105.9811704	70.39805644	101.7940572	58.27949049	74.8831994	28.03214352	84.15249486	21.45393384	79.36834237	12.78265745	75.66918959	105.685804	55.3258261	56.43804896	26.14464586	83.25546627	2.915342927	46.77630349	45.57873362	7.7929359
	83.96462219	97.22277326	104.2684099	86.92719698	111.1882563	85.19515478	82.7340896	23.97615843	91.04018458	92.14441579	82.16623686	114.6796271	86.73332052	111.9592386	61.53540964	73.81272832	5.82580273	87.65621996	6.748702173	92.57835032	3.36473755	91.05336243	114.6796271	59.05574291	36.89675062	11.72851375	73.81272832	1.826571813	23.1109402	51.75560405	4.537588925
	70.66536204	102.0454946	95.40117417	63.3926997	104.5847293	52.81800391	72.23091977	21.87866928	87.57338552	96.40275084	74.67818727	104.3025921	81.93737769	103.5420744	57.78522306	63.15068493	31.21330724	77.86692759	16.81017613	91.01761252	12.73972603	52.74202081	102.609769	56.09239993	51.25244618	21.19373777	73.1702544	10.54794521	28.70841487	51.29606771	9.921722114
AMP2CPR1ERY2FUS1DOX2	AMP	CPR	ERY	FUS	DOX	AMP+CPR	AMP+ERY	AMP+FUS	AMP+DOX	CPR+ERY	CPR+FUS	CPR+DOX	ERY+FUS	ERY+DOX	FUS+DOX	AMP+CPR+ERY	AMP+CPR+FUS	AMP+CPR+DOX	AMP+ERY+FUS	AMP+ERY+DOX	AMP+FUS+DOX	CPR+ERY+FUS	CPR+ERY+DOX	CPR+FUS+DOX	ERY+FUS+DOX	AMP+CPR+ERY+FUS	AMP+CPR+ERY+DOX	AMP+CPR+FUS+DOX	AMP+ERY+FUS+DOX	CPR+ERY+FUS+DOX	AMP+CPR+ERY+FUS+DOX
	65.31489441	89.73601625	92.2257522	65.33136422	113.9560642	71.59409456	72.19211362	27.43412446	83.25546627	89.14528337	66.40206757	115.7282629	70.39805644	107.1762287	61.19623408	74.8831994	28.03214352	81.76041861	21.45393384	85.34853298	18.76284807	75.66918959	109.5255677	55.91655898	49.85983928	26.14464586	85.34853298	2.317323865	27.43412446	48.79084364	10.29714072
	83.96462219	97.22277326	104.2684099	86.92719698	121.6623686	85.19515478	82.7340896	23.97615843	95.96231494	92.14441579	82.16623686	119.7579845	86.73332052	117.4966353	62.17020432	73.81272832	5.82580273	77.81195924	6.748702173	96.57758123	3.36473755	91.05336243	113.727435	52.64828407	40.03076331	11.72851375	67.35243222	0.903672371	3.36473755	45.40765721	12.59373197
	70.66536204	102.0454946	95.40117417	63.3926997	102.8919062	52.81800391	72.23091977	21.87866928	91.64383562	96.40275084	74.67818727	108.53465	81.93737769	111.369863	51.01393052	63.15068493	31.21330724	77.86692759	16.81017613	92.27005871	5.851272016	52.74202081	106.2775525	54.68171398	48.43444227	21.19373777	72.85714286	3.659491194	53.75733855	44.80691236	12.73972603
AMP2CPR1ERY2FUS1DOX3	AMP	CPR	ERY	FUS	DOX	AMP+CPR	AMP+ERY	AMP+FUS	AMP+DOX	CPR+ERY	CPR+FUS	CPR+DOX	ERY+FUS	ERY+DOX	FUS+DOX	AMP+CPR+ERY	AMP+CPR+FUS	AMP+CPR+DOX	AMP+ERY+FUS	AMP+ERY+DOX	AMP+FUS+DOX	CPR+ERY+FUS	CPR+ERY+DOX	CPR+FUS+DOX	ERY+FUS+DOX	AMP+CPR+ERY+FUS	AMP+CPR+ERY+DOX	AMP+CPR+FUS+DOX	AMP+ERY+FUS+DOX	CPR+ERY+FUS+DOX	AMP+CPR+ERY+FUS+DOX
	65.31489441	89.73601625	92.2257522	65.33136422	107.753369	71.59409456	72.19211362	27.43412446	86.54457111	89.14528337	66.40206757	111.0023999	70.39805644	109.8673145	80.39505261	74.8831994	28.03214352	73.38815175	21.45393384	85.04952345	12.18463839	75.66918959	107.4580026	82.46261769	59.63371332	26.14464586	84.15249486	32.21827696	23.54700056	76.85065534	27.63969352
	83.96462219	97.22277326	104.2684099	86.92719698	118.8057925	85.19515478	82.7340896	23.97615843	88.27148625	92.14441579	82.16623686	115.6318191	86.73332052	114.4203038	82.16623686	73.81272832	5.82580273	75.65852721	6.748702173	95.34704864	4.902903288	91.05336243	118.1709978	84.70541559	63.71851567	11.72851375	67.35243222	18.80407614	3.672370698	83.75322357	32.28225341
	70.66536204	102.0454946	95.40117417	63.3926997	99.22412273	52.81800391	72.23091977	21.87866928	93.52250489	96.40275084	74.67818727	109.0989244	81.93737769	111.0567515	74.74872157	63.15068493	31.21330724	90.39138943	16.81017613	89.76516634	17.12328767	52.74202081	102.8919062	79.26291659	80.43052838	21.19373777	76.61448141	17.43639922	15.87084149	71.92734967	25.26418787
AMP2CPR1ERY2FUS2DOX1	AMP	CPR	ERY	FUS	DOX	AMP+CPR	AMP+ERY	AMP+FUS	AMP+DOX	CPR+ERY	CPR+FUS	CPR+DOX	ERY+FUS	ERY+DOX	FUS+DOX	AMP+CPR+ERY	AMP+CPR+FUS	AMP+CPR+DOX	AMP+ERY+FUS	AMP+ERY+DOX	AMP+FUS+DOX	CPR+ERY+FUS	CPR+ERY+DOX	CPR+FUS+DOX	ERY+FUS+DOX	AMP+CPR+ERY+FUS	AMP+CPR+ERY+DOX	AMP+CPR+FUS+DOX	AMP+ERY+FUS+DOX	CPR+ERY+FUS+DOX	AMP+CPR+ERY+FUS+DOX
	65.31489441	89.73601625	92.2257522	92.3943142	108.9348348	71.59409456	72.19211362	28.23771258	76.97626612	89.14528337	81.17038951	105.9811704	90.13268548	101.7940572	74.19235739	74.8831994	30.12521024	84.15249486	25.04204822	79.36834237	26.44365539	64.92523537	105.685804	74.78309027	62.3247991	24.14501962	83.25546627	24.05157914	28.92917212	68.58039505	25.84563633
	83.96462219	97.22277326	104.2684099	105.1577068	111.1882563	85.19515478	82.7340896	38.43491636	91.04018458	92.14441579	86.43126364	114.6796271	101.8073447	111.9592386	87.24459433	73.81272832	24.28379158	87.65621996	31.05172082	92.57835032	36.0315324	80.57925015	114.6796271	81.53144217	64.64141511	10.13266679	73.81272832	31.41703519	19.36166122	78.04007141	24.64910594
	70.66536204	102.0454946	95.40117417	101.1990831	104.5847293	52.81800391	72.23091977	29.02152642	87.57338552	96.40275084	85.11726327	104.3025921	98.84540117	103.5420744	67.13101746	63.15068493	23.38551859	77.86692759	24.01174168	91.01761252	26.88845401	69.03544348	102.609769	63.463234	64.46183953	17.49510763	73.1702544	17.49510763	64.71624266	60.35972492	22.19178082

[illegible]

	83.96462219	82.75002596	92.14441579	86.92719698	121.6623686	69.19823111	78.42722553	23.97615843	95.96231494	80.75604943	94.71388514	107.3424032	80.26185281	118.1709978	62.17020432	67.35243222	28.59065564	91.96308402	8.594501057	106.421842	3.36473755	81.0052965	95.04621456	80.67296708	44.13806784	14.43953086	80.88829071	23.1109402	3.057104403	56.74524873	3.672370698
	70.66536204	88.76008668	100.9169459	63.3926997	102.8919062	76.92759295	69.09980431	21.87866928	91.64383562	87.73912234	94.54555123	109.1793733	71.01040381	101.4812202	51.01393052	62.52446184	25.89041096	91.64383562	6.164383562	97.90606654	5.851272016	76.06184279	104.4148731	65.51187802	46.78187269	18.06262231	82.87671233	32.21135029	4.598825832	43.43352432	18.37573386

AMP2CPR3ERY1FUS1DOX3	AMP	CPR	ERY	FUS	DOX	AMP+CPR	AMP+ERY	AMP+FUS	AMP+DOX	CPR+ERY	CPR+FUS	CPR+DOX	ERY+FUS	ERY+DOX	FUS+DOX	AMP+CPR+ERY	AMP+CPR+FUS	AMP+CPR+DOX	AMP+ERY+FUS	AMP+ERY+DOX	AMP+FUS+DOX	CPR+ERY+FUS	CPR+ERY+DOX	CPR+FUS+DOX	ERY+FUS+DOX	AMP+CPR+ERY+FUS	AMP+CPR+ERY+DOX	AMP+CPR+FUS+DOX	AMP+ERY+FUS+DOX	CPR+ERY+FUS+DOX	AMP+CPR+ERY+FUS+DOX
	65.31489441	92.70957489	94.46187927	65.33136422	107.753369	82.05942814	71.89310409	27.43412446	86.54457111	91.7560588	102.2447358	105.4231228	61.19623408	103.6182389	80.39505261	68.00598019	31.02223883	80.86339002	21.75294338	76.67725659	12.18463839	72.22884386	102.8804132	80.49264998	73.89699095	17.86581947	86.84358064	43.18818912	14.2777051	68.7326182	40.79611288
	83.96462219	82.75002596	92.14441579	86.92719698	118.8057925	69.19823111	78.42722553	23.97615843	88.27148625	80.75604943	94.71388514	116.9799564	80.26185281	113.0926403	82.16623686	67.35243222	28.59065564	89.1943857	8.594501057	88.27148625	4.902903288	81.0052965	105.0160972	88.6488732	74.866098	14.43953086	83.34935589	48.58681023	14.13189771	73.3617198	27.36012305
	70.66536204	88.76008668	100.9169459	63.3926997	99.22412273	76.92759295	69.09980431	21.87866928	93.52250489	87.73912234	94.54555123	109.1793733	71.01040381	98.65984835	74.74872157	62.52446184	25.89041096	81.93737769	6.164383562	78.49315068	17.12328767	76.06184279	101.6923016	88.31341478	71.64521248	18.06262231	80.37181996	31.52641879	10.23483366	74.70055701	35.28375734

AMP2CPR3ERY1FUS2DOX1	AMP	CPR	ERY	FUS	DOX	AMP+CPR	AMP+ERY	AMP+FUS	AMP+DOX	CPR+ERY	CPR+FUS	CPR+DOX	ERY+FUS	ERY+DOX	FUS+DOX	AMP+CPR+ERY	AMP+CPR+FUS	AMP+CPR+DOX	AMP+ERY+FUS	AMP+ERY+DOX	AMP+FUS+DOX	CPR+ERY+FUS	CPR+ERY+DOX	CPR+FUS+DOX	ERY+FUS+DOX	AMP+CPR+ERY+FUS	AMP+CPR+ERY+DOX	AMP+CPR+FUS+DOX	AMP+ERY+FUS+DOX	CPR+ERY+FUS+DOX	AMP+CPR+ERY+FUS+DOX
	65.31489441	92.70957489	94.46187927	92.3943142	108.9348348	82.05942814	71.89310409	28.23771258	76.97626612	91.7560588	102.2447358	104.4696067	87.66845117	105.0950711	74.19235739	68.00598019	46.47729396	83.85348533	16.57634087	80.86339002	26.44365539	74.27493047	105.1052841	79.53913389	65.33136422	30.03176976	75.48121846	26.74266492	19.0618576	68.7326182	27.93870305
	83.96462219	82.75002596	92.14441579	105.1577068	111.1882563	69.19823111	78.42722553	38.43491636	91.04018458	80.75604943	95.04621456	105.3484266	89.9226344	102.6185281	87.24459433	67.35243222	56.58527206	100.269179	17.82349548	93.80888291	36.0315324	73.44480216	102.0251324	83.99626129	64.39198572	29.51355509	79.65775812	41.56892905	16.90059604	66.71513137	30.1865026
	70.66536204	88.76008668	100.9169459	101.1990831	104.5847293	76.92759295	69.09980431	29.02152642	87.57338552	87.73912234	109.5196948	102.3729444	91.88855581	109.6631987	67.13101746	62.52446184	55.00978474	88.19960861	20	90.39138943	26.88845401	80.25205057	102.7132659	85.59084323	53.024158	22.44618395	80.37181996	41.91780822	12.11350294	77.08280712	28.45401174

AMP2CPR3ERY1FUS2DOX2	AMP	CPR	ERY	FUS	DOX	AMP+CPR	AMP+ERY	AMP+FUS	AMP+DOX	CPR+ERY	CPR+FUS	CPR+DOX	ERY+FUS	ERY+DOX	FUS+DOX	AMP+CPR+ERY	AMP+CPR+FUS	AMP+CPR+DOX	AMP+ERY+FUS	AMP+ERY+DOX	AMP+FUS+DOX	CPR+ERY+FUS	CPR+ERY+DOX	CPR+FUS+DOX	ERY+FUS+DOX	AMP+CPR+ERY+FUS	AMP+CPR+ERY+DOX	AMP+CPR+FUS+DOX	AMP+ERY+FUS+DOX	CPR+ERY+FUS+DOX	AMP+CPR+ERY+FUS+DOX
	65.31489441	92.70957489	94.46187927	92.3943142	113.9560642	82.05942814	71.89310409	28.23771258	83.25546627	91.7560588	102.2447358	118.1366706	87.66845117	107.1626361	77.14602178	68.00598019	46.47729396	79.06933283	16.57634087	88.93664736	18.76284807	74.27493047	106.6944776	79.22129519	67.3989293	30.03176976	82.95645674	33.61988413	27.73313399	82.71752086	29.73276023
	83.96462219	82.75002596	92.14441579	105.1577068	121.6623686	69.19823111	78.42722553	38.43491636	95.96231494	80.75604943	95.04621456	107.3424032	89.9226344	118.1709978	83.11842888	67.35243222	56.58527206	91.96308402	17.82349548	106.421842	24.03383965	73.44480216	95.04621456	86.32256724	81.84883952	29.51355509	80.88829071	33.87810037	57.20053836	78.01433171	35.72389925
	70.66536204	88.76008668	100.9169459	101.1990831	102.8919062	76.92759295	69.09980431	29.02152642	91.64383562	87.73912234	109.5196948	109.1793733	91.88855581	101.4812202	64.30964557	62.52446184	55.00978474	91.64383562	20	97.90606654	13.36594912	80.25205057	104.4148731	93.41823644	61.48827367	22.44618395	82.87671233	37.22113503	23.69863014	75.3811999	47.2407045

AMP2CPR3ERY1FUS2DOX3	AMP	CPR	ERY	FUS	DOX	AMP+CPR	AMP+ERY	AMP+FUS	AMP+DOX	CPR+ERY	CPR+FUS	CPR+DOX	ERY+FUS	ERY+DOX	FUS+DOX	AMP+CPR+ERY	AMP+CPR+FUS	AMP+CPR+DOX	AMP+ERY+FUS	AMP+ERY+DOX	AMP+FUS+DOX	CPR+ERY+FUS	CPR+ERY+DOX	CPR+FUS+DOX	ERY+FUS+DOX	AMP+CPR+ERY+FUS	AMP+CPR+ERY+DOX	AMP+CPR+FUS+DOX	AMP+ERY+FUS+DOX	CPR+ERY+FUS+DOX	AMP+CPR+ERY+FUS+DOX
	65.31489441	92.70957489	94.46187927	92.3943142	107.753369	82.05942814	71.89310409	28.23771258	86.54457111	91.7560588	102.2447358	105.4231228	87.66845117	103.6182389	102.7321396	68.00598019	46.47729396	80.86339002	16.57634087	76.67725659	16.96879088	74.27493047	102.8804132	93.98092968	88.55455049	30.03176976	86.84358064	94.91683797	4.615959634	83.35319825	68.30498972
	83.96462219	82.75002596	92.14441579	105.1577068	118.8057925	69.19823111	78.42722553	38.43491636	88.27148625	80.75604943	95.04621456	116.9799564	89.9226344	113.0926403	98.80975997	67.35243222	56.58527206	89.1943857	17.82349548	88.27148625	11.67083253	73.44480216	105.0160972	107.0100737	83.25729022	29.51355509	83.34935589	91.04018458	1.211305518	79.01131997	69.19823111
	70.66536204	88.76008668	100.9169459	101.1990831	99.22412273	76.92759295	69.09980431	29.02152642	93.52250489	87.73912234	109.5196948	109.1793733	91.88855581	98.65984835	93.58137895	62.52446184	55.00978474	81.93737769	20	78.49315068	11.54598826	80.25205057	101.6923016	104.7551946	80.32093105	22.44618395	80.37181996	82.56360078	0.528375734	80.14570012	64.09001957

AMP2CPR3ERY1FUS3DOX1	AMP	CPR	ERY	FUS	DOX	AMP+CPR	AMP+ERY	AMP+FUS	AMP+DOX	CPR+ERY	CPR+FUS	CPR+DOX	ERY+FUS	ERY+DOX	FUS+DOX	AMP+CPR+ERY	AMP+CPR+FUS	AMP+CPR+DOX	AMP+ERY+FUS	AMP+ERY+DOX	AMP+FUS+DOX	CPR+ERY+FUS	CPR+ERY+DOX	CPR+FUS+DOX	ERY+FUS+DOX	AMP+CPR+ERY+FUS	AMP+CPR+ERY+DOX	AMP+CPR+FUS+DOX	AMP+ERY+FUS+DOX	CPR+ERY+FUS+DOX	AMP+CPR+ERY+FUS+DOX
	65.31489441	92.70957489	94.46187927	103.6182389	108.9348348	82.05942814	71.89310409	66.21192301	76.97626612	91.7560588	118.4545093	104.4696067	96.52944434	105.0950711	79.69355732	68.00598019	66.51093254	83.85348533	59.03569426	80.86339002	62.92281816	104.7874454	105.1052841	103.8339293	72.01402991	55.74658942	75.48121846	77.27527565	60.23173239	86.98847835	59.03569426
	83.96462219	82.75002596	92.14441579	117.5362031	111.1882563	69.19823111	78.42722553	79.04249183	91.04018458	80.75604943	110.0010385	105.3484266	110.870859	102.6185281	70.24399921	67.35243222	85.19515478	100.269179	85.50278793	93.80888291	61.50740242	102.6897913	102.0251324	109.6687091	65.66157508	71.96692944	79.65775812	68.58296481	57.81580465	91.72292034	47.97154393
	70.66536204	88.76008668	100.9169459	113.6131194	104.5847293	76.92759295	69.09980431	71.60469667	87.57338552	87.73912234	118.0277309	102.3729444	106.5596896	109.6631987	58.59636748	62.52446184	81.3111546	88.19960861	71.91780822	90.39138943	70.97847358	103.3939088	102.7132659	99.65037289	56.05713278	56.57534247	80.37181996	65.96868885	51.56555773	80.59237201	42.79843444

AMP2CPR3ERY1FUS3DOX2	AMP	CPR	ERY	FUS	DOX	AMP+CPR	AMP+ERY	AMP+FUS	AMP+DOX	CPR+ERY	CPR+FUS	CPR+DOX	ERY+FUS	ERY+DOX	FUS+DOX	AMP+CPR+ERY	AMP+CPR+FUS	AMP+CPR+DOX	AMP+ERY+FUS	AMP+ERY+DOX	AMP+FUS+DOX	CPR+ERY+FUS	CPR+ERY+DOX	CPR+FUS+DOX	ERY+FUS+DOX	AMP+CPR+ERY+FUS	AMP+CPR+ERY+DOX	AMP+CPR+FUS+DOX	AMP+ERY+FUS+DOX	CPR+ERY+FUS+DOX	AMP+CPR+ERY+FUS+DOX
	65.31489441	92.70957489	94.46187927	103.6182389	113.9560642	82.05942814	71.89310409	66.21192301	83.25546627	91.7560588	118.4545093	118.1366706	96.52944434	107.1626361	86.78235186	68.00598019	66.51093254	79.06933283	59.03569426	88.93664736	59.33470379	104.7874454	106.6944776	99.70202622	68.1742662	55.74658942	82.95645674	65.01588488	52.75649411	83.49225268	48.86937021
	83.96462219	82.75002596	92.14441579	117.5362031	121.6623686	69.19823111	78.42722553	79.04249183	95.96231494	80.75604943	110.0010385	107.3424032	110.870859	118.1709978	76.90934338	67.35243222	85.19515478	91.96308402	85.50278793	106.421842	62.43030187	102.6897913	95.04621456	110.6656974	66.29636977	71.96692944	80.88829071	58.1234378	54.12420688	94.0492263	48.89444338
	70.66536204	88.76008668	100.9169459	113.6131194	102.8919062	76.92759295	69.09980431	71.60469667	91.64383562	87.73912234	118.0277309	109.1793733	106.5596896	101.4812202	76.93528478	62.52446184	81.3111546	91.64383562	71.91780822	97.90606654	55.63600783	103.3939088	104.4148731	102.032623	58.1026274	56.57534247	82.87671233	62.52446184	40.03913894	81.95365779	52.50489237

AMP2CPR3ERY1FUS3DOX3	AMP	CPR	ERY	FUS	DOX	AMP+CPR	AMP+ERY	AMP+FUS	AMP+DOX	CPR+ERY	CPR+FUS	CPR+DOX	ERY+FUS	ERY+DOX	FUS+DOX	AMP+CPR+ERY	AMP+CPR+FUS	AMP+CPR+DOX	AMP+ERY+FUS	AMP+ERY+DOX	AMP+FUS+DOX	CPR+ERY+FUS	CPR+ERY+DOX	CPR+FUS+DOX	ERY+FUS+DOX	AMP+CPR+ERY+FUS	AMP+CPR+ERY+DOX	AMP+CPR+FUS+DOX	AMP+ERY+FUS+DOX	CPR+ERY+FUS+DOX	AMP+CPR+ERY+FUS+DOX
	65.31489441	92.70957489	94.46187927	103.6182389	107.753369	82.05942814	71.89310409	66.21192301	86.54457111	91.7560588	118.4545093	105.4231228	96.52944434	103.6182389	97.7109101	68.00598019	66.51093254	80.86339002	59.03569426	76.67725659	65.91291347	104.7874454	102.8804132	107.0123162	90.03138268	55.74658942	86.84358064	76.67725659	51.26144646	92.70957489	53.05550364
	83.96462219	82.75002596	92.14441579	117.5362031	118.8057925	69.19823111	78.42722553	79.04249183	88.27148625	80.75604943	110.0010385	116.9799564	110.870859	113.0926403	108.3316802	67.35243222	85.19515478	89.1943857	85.50278793	88.27148625	73.19746203	102.6897913	105.0160972	124.6235331	94.36619718	71.96692944	83.34935589	76.8890598	50.12497597	103.0221207	60.27686983
	70.66536204	88.76008668	100.9169459	113.6131194	99.22412273	76.92759295	69.09980431	71.60469667	93.52250489	87.73912234	118.0277309	109.1793733	106.5596896	98.65984835	102.3276318	62.52446184	81.3111546	81.93737769	71.91780822	78.49315068	61.27201566	103.3939088	101.6923016	123.472874	88.22077235	56.57534247	80.37181996	73.7964775	51.25244618	103.7342302	57.5146771

AMP2CPR3ERY2FUS1DOX1	AMP	CPR	ERY	FUS	DOX	AMP+CPR	AMP+ERY	AMP+FUS	AMP+DOX	CPR+ERY	CPR+FUS	CPR+DOX	ERY+FUS	ERY+DOX	FUS+DOX	AMP+CPR+ERY	AMP+CPR+FUS	AMP+CPR+DOX	AMP+ERY+FUS	AMP+ERY+DOX	AMP+FUS+DOX	CPR+ERY+FUS	CPR+ERY+DOX	CPR+FUS+DOX	ERY+FUS+DOX	AMP+CPR+ERY+FUS	AMP+CPR+ERY+DOX	AMP+CPR+FUS+DOX	AMP+ERY+FUS+DOX	CPR+ERY+FUS+DOX	AMP+CPR+ERY+FUS+DOX
	65.31489441	92.70957489	92.2257522	65.33136422	108.9348348	82.05942814	72.19211362	27.43412446	76.97626612	91.12038141	102.2447358	104.4696067	70.39805644	101.7940572	58.27949049	78.47131377	31.02223883	83.85348533	21.45393384	79.36834237	12.78265745	66.50774732	109.5550258	63.32936035	56.43804896	25.93907681	77.87329471	27.04167445	46.77630349	63.32936035	14.87572416
	83.96462219	82.75002596	104.2684099	86.92719698	111.1882563	69.19823111	82.7340896	23.97615843	91.04018458	88.73195555	94.71388514	105.3484266	86.73332052	111.9592386	61.53540964	64.27610075	28.59065564	100.269179	6.748702173	92.57835032	3.36473755	81.0052965	105.3484266	70.70308443	36.89675062	22.13035955	94.11651605	26.80253797	23.1109402	69.04143732	14.13189771
	70.66536204	88.76008668	95.40117417	63.3926997	104.5847293	76.92759295	72.23091977	21.87866928	87.57338552	86.03751512	94.54555123	102.3729444	81.93737769	103.5420744	57.78522306	67.53424658	25.89041096	88.19960861	16.81017613	91.01761252	12.73972603	85.59084323	104.0745517	64.15059224	51.25244618	21.50684932	86.00782779	17.74951076	28.70841487	68.91509246	6.790606654

AMP2CPR3ERY2FUS1DOX2	AMP	CPR	ERY	FUS	DOX	AMP+CPR	AMP+ERY	AMP+FUS	AMP+DOX	CPR+ERY	CPR+FUS	CPR+DOX	ERY+FUS	ERY+DOX	FUS+DOX	AMP+CPR+ERY	AMP+CPR+FUS	AMP+CPR+DOX	AMP+ERY+FUS	AMP+ERY+DOX	AMP+FUS+DOX	CPR+ERY+FUS	CPR+ERY+DOX	CPR+FUS+DOX	ERY+FUS+DOX	AMP+CPR+ERY+FUS	AMP+CPR+ERY+DOX	AMP+CPR+FUS+DOX	AMP+ERY+FUS+DOX	CPR+ERY+FUS+DOX	AMP+CPR+ERY+FUS+DOX
	65.31489441	92.70957489	92.2257522	65.33136422	113.9560642	82.05942814	72.19211362	27.43412446	83.25546627	91.12038141	102.2447358	118.1366706	70.39805644	107.1762287	61.19623408	78.47131377	31.02223883	79.06933283	21.45393384	85.34853298	18.76284807	66.50774732	112.0977354	66.82558602	49.85983928	25.93907681	84.75051392	44.08521772	27.43412446	63.32936035	21.15492431
	83.96462219	82.75002596	104.2684099	86.92719698	121.6623686	69.19823111	82.7340896	23.97615843	95.96231494	88.73195555	94.71388514	107.3424032	86.73332052	117.4966353	62.17020432	64.27610075	28.59065564	91.96308402	6.748702173	96.57758123	3.36473755	81.0052965	110.333368	80.67296708	40.03076331	22.13035955	90.11728514	23.1109402	3.36473755	70.37075501	38.18496443
	70.66536204	88.76008668	95.40117417	63.3926997	102.8919062	76.92759295	72.23091977	21.87866928	91.64383562	86.03751512	94.54555123	109.1793733	81.93737769	111.369863	51.01393052	67.53424658	25.89041096	91.64383562	16.81017613	92.27005871	5.851272016	85.59084323	102.032623	65.51187802	48.43444227	21.50684932	93.20939335	32.21135029	53.75733855	72.99894979	35.90998043

AMP2CPR3ERY2FUS1DOX3	AMP	CPR	ERY	FUS	DOX	AMP+CPR	AMP+ERY	AMP+FUS	AMP+DOX	CPR+ERY	CPR+FUS	CPR+DOX	ERY+FUS	ERY+DOX	FUS+DOX	AMP+CPR+ERY	AMP+CPR+FUS	AMP+CPR+DOX	AMP+ERY+FUS	AMP+ERY+DOX	AMP+FUS+DOX	CPR+ERY+FUS	CPR+ERY+DOX	CPR+FUS+DOX	ERY+FUS+DOX	AMP+CPR+ERY+FUS	AMP+CPR+ERY+DOX	AMP+CPR+FUS+DOX	AMP+ERY+FUS+DOX	CPR+ERY+FUS+DOX	AMP+CPR+ERY+FUS+DOX
	65.31489441	92.70957489	92.2257522	65.33136422	107.753369	82.05942814	72.19211362	27.43412446	86.54457111	91.12038141	102.2447358	105.4231228	70.39805644	109.8673145	80.39505261	78.47131377	31.02223883	80.86339002	21.45393384	85.04952345	12.18463839	66.50774732	106.6944776	80.49264998	59.63371332	25.93907681	86.54457111	43.18818912	23.54700056	76.36074692	24.74303868
	83.96462219	82.75002596	104.2684099	86.92719698	118.8057925	69.19823111	82.7340896	23.97615843	88.27148625	88.73195555	94.71388514	116.9799564	86.73332052	114.4203038	82.16623686	64.27610075	28.59065564	89.1943857	6.748702173	95.34704864	4.902903288	81.0052965	109.0040503	88.6488732	63.71851567	22.13035955	89.50201884	48.58681023	3.672370698	82.66694361	24.64910594
	70.66536204	88.76008668	95.40117417	63.3926997	99.22412273	76.92759295	72.23091977	21.87866928	93.52250489	86.03751512	94.54555123	109.1793733	81.93737769	111.0567515	74.74872157	67.53424658	25.89041096	81.93737769	16.81017613	89.76516634	17.12328767	85.59084323	105.7761589	88.31341478	80.43052838	21.50684932	88.19960861	31.52641879	15.87084149	81.84730734	27.20156556

AMP2CPR3ERY2FUS2DOX1	AMP	CPR	ERY	FUS	DOX	AMP+CPR	AMP+ERY	AMP+FUS	AMP+DOX	CPR+ERY	CPR+FUS	CPR+DOX	ERY+FUS	ERY+DOX	FUS+DOX	AMP+CPR+ERY	AMP+CPR+FUS	AMP+CPR+DOX	AMP+ERY+FUS	AMP+ERY+DOX	AMP+FUS+DOX	CPR+ERY+FUS	CPR+ERY+DOX	CPR+FUS+DOX	ERY+FUS+DOX	AMP+CPR+ERY+FUS	AMP+CPR+ERY+DOX	AMP+CPR+FUS+DOX	AMP+ERY+FUS+DOX	CPR+ERY+FUS+DOX	AMP+CPR+ERY+FUS+DOX
	65.31489441	92.70957489	92.2257522	92.3943142	108.9348348	82.05942814	72.19211362	28.23771258	76.97626612	91.12038141	102.2447358	104.4696067	90.13268548	101.7940572	74.19235739	78.47131377	46.47729396	83.85348533	25.04204822	79.36834237	26.44365539	91.7560588	109.5550258	79.53913389	62.3247991	41.992151	77.87329471	26.74266492	28.92917212	77.94994041	27.04167445
	83.96462219	82.75002596	104.2684099	105.1577068	111.1882563	69.19823111	82.7340896	38.43491636	91.04018458	88.73195555	95.04621456	105.3484266	101.8073447	111.9592386	87.24459433	64.27610075	56.58527206	100.269179	31.05172082	92.57835032	36.0315324	90.39360266	105.3484266	83.99626129	64.64141511	37.81965007	94.11651605	41.56892905	19.36166122	79.01131997	33.26283407
	70.66536204	88.76008668	95.40117417	101.1990831	104.5847293	76.92759295	72.23091977	29.02152642	87.57338552	86.03751512	109.5196948	102.3729444	98.84540117	103.5420744	67.13101746	67.53424658	55.00978474	88.19960861	24.01174168	91.01761252	26.88845401	97.60844423	104.0745517	85.59084323	64.46183953	33.09197652	86.00782779	41.91780822	64.71624266	80.48602156	39.41291585

AMP2CPR3ERY2FUS2DOX2	AMP	CPR	ERY	FUS	DOX	AMP+CPR	AMP+ERY	AMP+FUS	AMP+DOX	CPR+ERY	CPR+FUS	CPR+DOX	ERY+FUS	ERY+DOX	FUS+DOX	AMP+CPR+ERY	AMP+CPR+FUS	AMP+CPR+DOX	AMP+ERY+FUS	AMP+ERY+DOX	AMP+FUS+DOX	CPR+ERY+FUS	CPR+ERY+DOX	CPR+FUS+DOX	ERY+FUS+DOX	AMP+CPR+ERY+FUS	AMP+CPR+ERY+DOX	AMP+CPR+FUS+DOX	AMP+ERY+FUS+DOX	CPR+ERY+FUS+DOX	AMP+CPR+ERY+FUS+DOX
	65.31489441	92.70957489	92.2257522	92.3943142	113.9560642	82.05942814	72.19211362	28.23771258	83.25546627	91.12038141	102.2447358	118.1366706	90.13268548	107.1762287	77.14602178	78.47131377	46.47729396	79.06933283	25.04204822	85.34853298	18.76284807	91.7560588	112.0977354	79.22129519	71.29508503	41.992151	84.75051392	33.61988413	26.74266492	82.08184346	21.75294338
	83.96462219	82.75002596	104.2684099	105.1577068	121.6623686	69.19823111	82.7340896	38.43491636	95.96231494	88.73195555	95.04621456	107.3424032	101.8073447	117.4966353	83.11842888	64.27610075	56.58527206	91.96308402	31.05172082	96.57758123	24.03383965	90.39360266	110.333368	86.32256724	71.7169775	37.81965007	90.11728514	33.87810037	19.36166122	79.34364939	25.87963853
	70.66536204	88.76008668	95.40117417	101.1990831	102.8919062	76.92759295	72.23091977	29.02152642	91.64383562	86.03751512	109.5196948	109.1793733	98.84540117	111.369863	64.30964557	67.53424658	55.00978474	91.64383562	24.01174168	92.27005871	13.36594912	97.60844423	102.032623	93.41823644	66.9667319	33.09197652	93.20939335	37.22113503	26.51663405	86.27148611	36.22309198

AMP2CPR3ERY2FUS2DOX3	AMP	CPR	ERY	FUS	DOX	AMP+CPR	AMP+ERY	AMP+FUS	AMP+DOX	CPR+ERY	CPR+FUS	CPR+DOX	ERY+FUS	ERY+DOX	FUS+DOX	AMP+CPR+ERY	AMP+CPR+FUS	AMP+CPR+DOX	AMP+ERY+FUS	AMP+ERY+DOX	AMP+FUS+DOX	CPR+ERY+FUS	CPR+ERY+DOX	CPR+FUS+DOX	ERY+FUS+DOX	AMP+CPR+ERY+FUS	AMP+CPR+ERY+DOX	AMP+CPR+FUS+DOX	AMP+ERY+FUS+DOX	CPR+ERY+FUS+DOX	AMP+CPR+ERY+FUS+DOX
	65.31489441	92.70957489	92.2257522	92.3943142	107.753369	82.05942814	72.19211362	28.23771258	86.54457111	91.12038141	102.2447358	105.4231228	90.13268548	109.8673145	102.7321396	78.47131377	46.47729396	80.86339002	25.04204822	85.04952345	16.96879088	91.7560588	106.6944776	93.98092968	106.2792001	41.992151	86.54457111	94.91683797	16.96879088	77.31426301	73.98617081
	83.96462219	82.75002596	104.2684099	105.1577068	118.8057925	69.19823111	82.7340896	38.43491636	88.27148625	88.73195555	95.04621456	116.9799564	101.8073447	114.4203038	98.80975997	64.27610075	56.58527206	89.1943857	31.05172082	95.34704864	11.67083253	90.39360266	109.0040503	107.0100737	107.0371082	37.81965007	89.50201884	91.04018458	26.12959046	74.35870807	83.96462219
	70.66536204	88.76008668	95.40117417	101.1990831	99.22412273	76.92759295	72.23091977	29.02152642	93.52250489	86.03751512	109.5196948	109.1793733	98.84540117	111.0567515	93.58137895	67.53424658	55.00978474	81.93737769	24.01174168	89.76516634	11.54598826	97.60844423	105.7761589	104.7551946	105.1076321	33.09197652	88.19960861	82.56360078	2.152641879	88.99405767	75.36203523

AMP2CPR3ERY2FUS3DOX1	AMP	CPR	ERY	FUS	DOX	AMP+CPR	AMP+ERY	AMP+FUS	AMP+DOX	CPR+ERY	CPR+FUS	CPR+DOX	ERY+FUS	ERY+DOX	FUS+DOX	AMP+CPR+ERY	AMP+CPR+FUS	AMP+CPR+DOX	AMP+ERY+FUS	AMP+ERY+DOX	AMP+FUS+DOX	CPR+ERY+FUS	CPR+ERY+DOX	CPR+FUS+DOX	ERY+FUS+DOX	AMP+CPR+ERY+FUS	AMP+CPR+ERY+DOX	AMP+CPR+FUS+DOX	AMP+ERY+FUS+DOX	CPR+ERY+FUS+DOX	AMP+CPR+ERY+FUS+DOX
	65.31489441	92.70957489	92.2257522	103.6182389	108.9348348	82.05942814	72.19211362	66.21192301	76.97626612	91.12038141	118.4545093	104.4696067	102.0930667	101.7940572	79.69355732	78.47131377	66.51093254	83.85348533	56.64361802	79.36834237	62.92281816	110.8263806	109.5550258	103.8339293	79.06933283	59.93272286	77.87329471	77.27527565	60.53074192	93.98092968	58.73668473
	83.96462219	82.75002596	104.2684099	117.5362031	111.1882563	69.19823111	82.7340896	79.04249183	91.04018458	88.73195555	110.0010385	105.3484266	117.4966353	111.9592386	70.24399921	64.27610075	85.19515478	100.269179	78.11959239	92.57835032	61.50740242	106.3454149	105.3484266	109.6687091	74.73562776	71.35166314	94.11651605	68.58296481	45.8181119	104.3514384	54.12420688
	70.66536204	88.76008668	95.40117417	113.6131194	104.5847293	76.92759295	72.23091977	71.60469667	87.57338552	86.03751512	118.0277309	102.3729444	112.6223092	103.5420744	58.59636748	67.53424658	81.3111546	88.19960861	69.09980431	91.01761252	70.97847358	109.5196948	104.0745517	99.65037289	88.51272016	65.34246575	86.00782779	65.96868885	55.63600783	93.86490834	54.0704501

AMP2CPR3ERY2FUS3DOX2	AMP	CPR	ERY	FUS	DOX	AMP+CPR	AMP+ERY	AMP+FUS	AMP+DOX	CPR+ERY	CPR+FUS	CPR+DOX	ERY+FUS	ERY+DOX	FUS+DOX	AMP+CPR+ERY	AMP+CPR+FUS	AMP+CPR+DOX	AMP+ERY+FUS	AMP+ERY+DOX	AMP+FUS+DOX	CPR+ERY+FUS	CPR+ERY+DOX	CPR+FUS+DOX	ERY+FUS+DOX	AMP+CPR+ERY+FUS	AMP+CPR+ERY+DOX	AMP+CPR+FUS+DOX	AMP+ERY+FUS+DOX	CPR+ERY+FUS+DOX	AMP+CPR+ERY+FUS+DOX
	65.31489441	92.70957489	92.2257522	103.6182389	113.9560642	82.05942814	72.19211362	66.21192301	83.25546627	91.12038141	118.4545093	118.1366706	102.0930667	107.1762287	86.78235186	78.47131377	66.51093254	79.06933283	56.64361802	85.34853298	59.33470379	110.8263806	112.0977354	99.70202622	86.54457111	59.93272286	84.75051392	65.01588488	68.30498972	82.85657529	60.53074192
	83.96462219	82.75002596	104.2684099	117.5362031	121.6623686	69.19823111	82.7340896	79.04249183	95.96231494	88.73195555	110.0010385	107.3424032	117.4966353	117.4966353	76.90934338	64.27610075	85.19515478	91.96308402	78.11959239	96.57758123	62.43030187	106.3454149	110.333368	110.6656974	78.11959239	71.35166314	90.11728514	58.1234378	61.19976928	99.69882646	45.20284561
	70.66536204	88.76008668	95.40117417	113.6131194	102.8919062	76.92759295	72.23091977	71.60469667	91.64383562	86.03751512	118.0277309	109.1793733	112.6223092	111.369863	76.93528478	67.53424658	81.3111546	91.64383562	69.09980431	92.27005871	55.63600783	109.5196948	102.032623	102.032623	78.18003914	65.34246575	93.20939335	62.52446184	60.64579256	90.4616939	50.93933464

AMP2CPR3ERY2FUS3DOX3	AMP	CPR	ERY	FUS	DOX	AMP+CPR	AMP+ERY	AMP+FUS	AMP+DOX	CPR+ERY	CPR+FUS	CPR+DOX	ERY+FUS	ERY+DOX	FUS+DOX	AMP+CPR+ERY	AMP+CPR+FUS	AMP+CPR+DOX	AMP+ERY+FUS	AMP+ERY+DOX	AMP+FUS+DOX	CPR+ERY+FUS	CPR+ERY+DOX	CPR+FUS+DOX	ERY+FUS+DOX	AMP+CPR+ERY+FUS	AMP+CPR+ERY+DOX	AMP+CPR+FUS+DOX	AMP+ERY+FUS+DOX	CPR+ERY+FUS+DOX	AMP+CPR+ERY+FUS+DOX
	65.31489441	92.70957489	92.2257522	103.6182389	107.753369	82.05942814	72.19211362	66.21192301	86.54457111	91.12038141	118.4545093	105.4231228	102.0930667	109.8673145	97.7109101	78.47131377	66.51093254	80.86339002	56.64361802	85.04952345	65.91291347	110.8263806	106.6944776	107.0123162	109.8673145	59.93272286	86.54457111	76.67725659	65.01588488	93.02741359	65.61390394
	83.96462219	82.75002596	104.2684099	117.5362031	118.8057925	69.19823111	82.7340896	79.04249183	88.27148625	88.73195555	110.0010385	116.9799564	117.4966353	114.4203038	108.3316802	64.27610075	85.19515478	89.1943857	78.11959239	95.34704864	73.19746203	106.3454149	109.0040503	124.6235331	102.1149779	71.35166314	89.50201884	76.8890598	57.81580465	108.007062	62.12266872
	70.66536204	88.76008668	95.40117417	113.6131194	99.22412273	76.92759295	72.23091977	71.60469667	93.52250489	86.03751512	118.0277309	109.1793733	112.6223092	111.0567515	102.3276318	67.53424658	81.3111546	81.93737769	69.09980431	89.76516634	61.27201566	109.5196948	105.7761589	123.472874	108.2387476	65.34246575	88.19960861	73.7964775	61.27201566	117.347088	68.78669276

AMP2CPR3ERY3FUS1DOX1	AMP	CPR	ERY	FUS	DOX	AMP+CPR	AMP+ERY	AMP+FUS	AMP+DOX	CPR+ERY	CPR+FUS	CPR+DOX	ERY+FUS	ERY+DOX	FUS+DOX	AMP+CPR+ERY	AMP+CPR+FUS	AMP+CPR+DOX	AMP+ERY+FUS	AMP+ERY+DOX	AMP+FUS+DOX	CPR+ERY+FUS	CPR+ERY+DOX	CPR+FUS+DOX	ERY+FUS+DOX	AMP+CPR+ERY+FUS	AMP+CPR+ERY+DOX	AMP+CPR+FUS+DOX	AMP+ERY+FUS+DOX	CPR+ERY+FUS+DOX	AMP+CPR+ERY+FUS+DOX
	65.31489441	92.70957489	99.06634883	65.33136422	108.9348348	82.05942814	72.49112315	27.43412446	76.97626612	97.47715534	102.2447358	104.4696067	105.4231228	106.0588002	58.27949049	89.23565689	31.02223883	83.85348533	38.49747711	78.17230424	12.78265745	68.7326182	110.1907032	63.32936035	59.51529599	26.53709587	79.36834237	27.04167445	23.84601009	63.64719905	20.25789572
	83.96462219	82.75002596	82.75002596	86.92719698	111.1882563	69.19823111	89.1943857	23.97615843	91.04018458	91.39059092	94.71388514	105.3484266	94.38155572	94.71388514	61.53540964	68.58296481	28.59065564	100.269179	14.43953086	89.80965199	3.36473755	87.31955551	103.0221207	70.70308443	70.70308443	36.58911748	92.57835032	26.80253797	4.902903288	62.72717832	3.980003845
	70.66536204	88.76008668	91.48265823	63.3926997	104.5847293	76.92759295	75.04892368	21.87866928	87.57338552	90.4616939	94.54555123	102.3729444	95.906837	103.3939088	57.78522306	74.42270059	25.89041096	88.19960861	12.79843444	87.57338552	12.73972603	86.27148611	101.6923016	64.15059224	74.70055701	29.33463796	82.25048924	17.74951076	16.18395303	58.02480624	10.23483366

AMP2CPR3ERY3FUS1DOX2	AMP	CPR	ERY	FUS	DOX	AMP+CPR	AMP+ERY	AMP+FUS	AMP+DOX	CPR+ERY	CPR+FUS	CPR+DOX	ERY+FUS	ERY+DOX	FUS+DOX	AMP+CPR+ERY	AMP+CPR+FUS	AMP+CPR+DOX	AMP+ERY+FUS	AMP+ERY+DOX	AMP+FUS+DOX	CPR+ERY+FUS	CPR+ERY+DOX	CPR+FUS+DOX	ERY+FUS+DOX	AMP+CPR+ERY+FUS	AMP+CPR+ERY+DOX	AMP+CPR+FUS+DOX	AMP+ERY+FUS+DOX	CPR+ERY+FUS+DOX	AMP+CPR+ERY+FUS+DOX
	65.31489441	92.70957489	99.06634883	65.33136422	113.9560642	82.05942814	72.49112315	27.43412446	83.25546627	97.47715534	102.2447358	118.1366706	105.4231228	116.2296385	61.19623408	89.23565689	31.02223883	79.06933283	38.49747711	85.34853298	18.76284807	68.7326182	113.6869289	66.82558602	65.55423123	26.53709587	89.23565689	44.08521772	14.87572416	66.82558602	20.25789572
	83.96462219	82.75002596	82.75002596	86.92719698	121.6623686	69.19823111	89.1943857	23.97615843	95.96231494	91.39059092	94.71388514	107.3424032	94.38155572	106.6777443	62.17020432	68.58296481	28.59065564	91.96308402	14.43953086	96.26994809	3.36473755	87.31955551	107.0100737	80.67296708	81.0052965	36.58911748	90.73255143	23.1109402	5.210536435	75.02336691	22.13035955
	70.66536204	88.76008668	91.48265823	63.3926997	102.8919062	76.92759295	75.04892368	21.87866928	91.64383562	90.4616939	94.54555123	109.1793733	95.906837	107.1374447	51.01393052	74.42270059	25.89041096	91.64383562	12.79843444	87.88649706	5.851272016	86.27148611	103.7342302	65.51187802	71.29734257	29.33463796	91.95694716	32.21135029	13.99217221	62.10866357	3.659491194

AMP2CPR3ERY3FUS1DOX3	AMP	CPR	ERY	FUS	DOX	AMP+CPR	AMP+ERY	AMP+FUS	AMP+DOX	CPR+ERY	CPR+FUS	CPR+DOX	ERY+FUS	ERY+DOX	FUS+DOX	AMP+CPR+ERY	AMP+CPR+FUS	AMP+CPR+DOX	AMP+ERY+FUS	AMP+ERY+DOX	AMP+FUS+DOX	CPR+ERY+FUS	CPR+ERY+DOX	CPR+FUS+DOX	ERY+FUS+DOX	AMP+CPR+ERY+FUS	AMP+CPR+ERY+DOX	AMP+CPR+FUS+DOX	AMP+ERY+FUS+DOX	CPR+ERY+FUS+DOX	AMP+CPR+ERY+FUS+DOX
	65.31489441	92.70957489	99.06634883	65.33136422	107.753369	82.05942814	72.49112315	27.43412446	86.54457111	97.47715534	102.2447358	105.4231228	105.4231228	104.151768	80.39505261	89.23565689	31.02223883	80.86339002	38.49747711	92.2257522	12.18463839	68.7326182	106.0588002	80.49264998	76.04290822	26.53709587	82.65744721	43.18818912	21.15492431	76.67858562	46.47729396
	83.96462219	82.75002596	82.75002596	86.92719698	118.8057925	69.19823111	89.1943857	23.97615843	88.27148625	91.39059092	94.71388514	116.9799564	94.38155572	120.9679094	82.16623686	68.58296481	28.59065564	89.1943857	14.43953086	86.42568737	4.902903288	87.31955551	109.3363797	88.6488732	77.68200228	36.58911748	82.7340896	48.58681023	9.825033647	81.66995534	28.2830225
	70.66536204	88.76008668	91.48265823	63.3926997	99.22412273	76.92759295	75.04892368	21.87866928	93.52250489	90.4616939	94.54555123	109.1793733	95.906837	103.0535873	74.74872157	74.42270059	25.89041096	81.93737769	12.79843444	87.26027397	17.12328767	86.27148611	108.8390519	88.31341478	80.93269346	29.33463796	85.69471624	31.52641879	29.64774951	82.52795023	40.29354207

AMP2CPR3ERY3FUS2DOX1	AMP	CPR	ERY	FUS	DOX	AMP+CPR	AMP+ERY	AMP+FUS	AMP+DOX	CPR+ERY	CPR+FUS	CPR+DOX	ERY+FUS	ERY+DOX	FUS+DOX	AMP+CPR+ERY	AMP+CPR+FUS	AMP+CPR+DOX	AMP+ERY+FUS	AMP+ERY+DOX	AMP+FUS+DOX	CPR+ERY+FUS	CPR+ERY+DOX	CPR+FUS+DOX	ERY+FUS+DOX	AMP+CPR+ERY+FUS	AMP+CPR+ERY+DOX	AMP+CPR+FUS+DOX	AMP+ERY+FUS+DOX	CPR+ERY+FUS+DOX	AMP+CPR+ERY+FUS+DOX
	65.31489441	92.70957489	99.06634883	92.3943142	108.9348348	82.05942814	72.49112315	28.23771258	76.97626612	97.47715534	102.2447358	104.4696067	110.1907032	106.0588002	74.19235739	89.23565689	46.47729396	83.85348533	23.54700056	78.17230424	26.44365539	102.2447358	110.1907032	79.53913389	75.40723083	47.67333209	79.36834237	26.74266492	25.24761727	76.99642431	33.91889366
	83.96462219	82.75002596	82.75002596	105.1577068	111.1882563	69.19823111	89.1943857	38.43491636	91.04018458	91.39059092	95.04621456	105.3484266	105.3484266	94.71388514	87.24459433	68.58296481	56.58527206	100.269179	39.9730821	89.80965199	36.0315324	96.04320282	103.0221207	83.99626129	80.00830824	44.27994616	92.57835032	41.56892905	25.26437224	86.32256724	41.2612959
	70.66536204	88.76008668	91.48265823	101.1990831	104.5847293	76.92759295	75.04892368	29.02152642	87.57338552	90.4616939	109.5196948	102.3729444	110.8809806	103.3939088	67.13101746	74.42270059	55.00978474	88.19960861	20.56751468	87.57338552	26.88845401	106.4568018	101.6923016	85.59084323	82.18762878	45.30332681	82.25048924	41.91780822	38.72798434	81.16666445	37.22113503

AMP2CPR3ERY3FUS2DOX2	AMP	CPR	ERY	FUS	DOX	AMP+CPR	AMP+ERY	AMP+FUS	AMP+DOX	CPR+ERY	CPR+FUS	CPR+DOX	ERY+FUS	ERY+DOX	FUS+DOX	AMP+CPR+ERY	AMP+CPR+FUS	AMP+CPR+DOX	AMP+ERY+FUS	AMP+ERY+DOX	AMP+FUS+DOX	CPR+ERY+FUS	CPR+ERY+DOX	CPR+FUS+DOX	ERY+FUS+DOX	AMP+CPR+ERY+FUS	AMP+CPR+ERY+DOX	AMP+CPR+FUS+DOX	AMP+ERY+FUS+DOX	CPR+ERY+FUS+DOX	AMP+CPR+ERY+FUS+DOX
	65.31489441	92.70957489	99.06634883	92.3943142	113.9560642	82.05942814	72.49112315	28.23771258	83.25546627	97.47715534	102.2447358	118.1366706	110.1907032	116.2296385	77.14602178	89.23565689	46.47729396	79.06933283	23.54700056	85.34853298	18.76284807	102.2447358	113.6869289	79.22129519	69.36829559	47.67333209	89.23565689	33.61988413	20.46346477	77.31426301	35.71295085
	83.96462219	82.75002596	82.75002596	105.1577068	121.6623686	69.19823111	89.1943857	38.43491636	95.96231494	91.39059092	95.04621456	107.3424032	105.3484266	106.6777443	83.11842888	68.58296481	56.58527206	91.96308402	39.9730821	96.26994809	24.03383965	96.04320282	107.0100737	86.32256724	73.02939038	44.27994616	90.73255143	33.87810037	36.28148433	85.99023782	38.49259758
	70.66536204	88.76008668	91.48265823	101.1990831	102.8919062	76.92759295	75.04892368	29.02152642	91.64383562	90.4616939	109.5196948	109.1793733	110.8809806	107.1374447	64.30964557	74.42270059	55.00978474	91.64383562	20.56751468	87.88649706	13.36594912	106.4568018	103.7342302	93.41823644	75.72152134	45.30332681	91.95694716	37.22113503	25.32289628	86.952129	36.28180039

AMP2CPR3ERY3FUS2DOX3	AMP	CPR	ERY	FUS	DOX	AMP+CPR	AMP+ERY	AMP+FUS	AMP+DOX	CPR+ERY	CPR+FUS	CPR+DOX	ERY+FUS	ERY+DOX	FUS+DOX	AMP+CPR+ERY	AMP+CPR+FUS	AMP+CPR+DOX	AMP+ERY+FUS	AMP+ERY+DOX	AMP+FUS+DOX	CPR+ERY+FUS	CPR+ERY+DOX	CPR+FUS+DOX	ERY+FUS+DOX	AMP+CPR+ERY+FUS	AMP+CPR+ERY+DOX	AMP+CPR+FUS+DOX	AMP+ERY+FUS+DOX	CPR+ERY+FUS+DOX	AMP+CPR+ERY+FUS+DOX
	65.31489441	92.70957489	99.06634883	92.3943142	107.753369	82.05942814	72.49112315	28.23771258	86.54457111	97.47715534	102.2447358	105.4231228	110.1907032	104.151768	102.7321396	89.23565689	46.47729396	80.86339002	23.54700056	92.2257522	16.96879088	102.2447358	106.0588002	93.98092968	111.462058	47.67333209	82.65744721	94.91683797	30.42421977	103.5160906	84.75051392
	83.96462219	82.75002596	82.75002596	105.1577068	118.8057925	69.19823111	89.1943857	38.43491636	88.27148625	91.39059092	95.04621456	116.9799564	105.3484266	120.9679094	98.80975997	68.58296481	56.58527206	89.1943857	39.9730821	86.42568737	11.67083253	96.04320282	109.3363797	107.0100737	118.9739329	44.27994616	82.7340896	91.04018458	12.59373197	110.6656974	82.42645645
	70.66536204	88.76008668	91.48265823	101.1990831	99.22412273	76.92759295	75.04892368	29.02152642	93.52250489	90.4616939	109.5196948	109.1793733	110.8809806	103.0535873	93.58137895	74.42270059	55.00978474	81.93737769	20.56751468	87.26027397	11.54598826	106.4568018	108.8390519	104.7551946	118.7083738	45.30332681	85.69471624	82.56360078	16.86888454	106.1164803	75.04892368

AMP2CPR3ERY3FUS3DOX1	AMP	CPR	ERY	FUS	DOX	AMP+CPR	AMP+ERY	AMP+FUS	AMP+DOX	CPR+ERY	CPR+FUS	CPR+DOX	ERY+FUS	ERY+DOX	FUS+DOX	AMP+CPR+ERY	AMP+CPR+FUS	AMP+CPR+DOX	AMP+ERY+FUS	AMP+ERY+DOX	AMP+FUS+DOX	CPR+ERY+FUS	CPR+ERY+DOX	CPR+FUS+DOX	ERY+FUS+DOX	AMP+CPR+ERY+FUS	AMP+CPR+ERY+DOX	AMP+CPR+FUS+DOX	AMP+ERY+FUS+DOX	CPR+ERY+FUS+DOX	AMP+CPR+ERY+FUS+DOX
	65.31489441	92.70957489	99.06634883	103.6182389	108.9348348	82.05942814	72.49112315	66.21192301	76.97626612	97.47715534	118.4545093	104.4696067	116.8653159	106.0588002	79.69355732	89.23565689	66.51093254	83.85348533	59.93272286	78.17230424	62.92281816	111.462058	110.1907032	103.8339293	97.79499404	62.3247991	79.36834237	77.27527565	74.58418987	94.93444577	74.8831994
	83.96462219	82.75002596	82.75002596	117.5362031	111.1882563	69.19823111	89.1943857	79.04249183	91.04018458	91.39059092	110.0010385	105.3484266	110.6656974	94.71388514	70.24399921	68.58296481	85.19515478	100.269179	87.04095366	89.80965199	61.50740242	112.6596739	103.0221207	109.6687091	110.0010385	77.19669294	92.57835032	68.58296481	76.8890598	101.0281441	68.27533167
	70.66536204	88.76008668	91.48265823	113.6131194	104.5847293	76.92759295	75.04892368	71.60469667	87.57338552	90.4616939	118.0277309	102.3729444	124.4938383	103.3939088	58.59636748	74.42270059	81.3111546	88.19960861	76.30136986	87.57338552	70.97847358	115.3051593	101.6923016	99.65037289	98.96973	73.48336595	82.25048924	65.96868885	71.60469667	91.48265823	73.48336595

AMP2CPR3ERY3FUS3DOX2	AMP	CPR	ERY	FUS	DOX	AMP+CPR	AMP+ERY	AMP+FUS	AMP+DOX	CPR+ERY	CPR+FUS	CPR+DOX	ERY+FUS	ERY+DOX	FUS+DOX	AMP+CPR+ERY	AMP+CPR+FUS	AMP+CPR+DOX	AMP+ERY+FUS	AMP+ERY+DOX	AMP+FUS+DOX	CPR+ERY+FUS	CPR+ERY+DOX	CPR+FUS+DOX	ERY+FUS+DOX	AMP+CPR+ERY+FUS	AMP+CPR+ERY+DOX	AMP+CPR+FUS+DOX	AMP+ERY+FUS+DOX	CPR+ERY+FUS+DOX	AMP+CPR+ERY+FUS+DOX
	65.31489441	92.70957489	99.06634883	103.6182389	113.9560642	82.05942814	72.49112315	66.21192301	83.25546627	97.47715534	118.4545093	118.1366706	116.8653159	116.2296385	86.78235186	89.23565689	66.51093254	79.06933283	59.93272286	85.34853298	59.33470379	111.462058	113.6869289	99.70202622	92.70957489	62.3247991	89.23565689	65.01588488	65.01588488	100.973381	75.48121846
	83.96462219	82.75002596	82.75002596	117.5362031	121.6623686	69.19823111	89.1943857	79.04249183	95.96231494	91.39059092	110.0010385	107.3424032	110.6656974	106.6777443	76.90934338	68.58296481	85.19515478	91.96308402	87.04095366	96.26994809	62.43030187	112.6596739	107.0100737	110.6656974	103.6867795	77.19669294	90.73255143	58.1234378	64.5837339	108.3393914	62.12266872
	70.66536204	88.76008668	91.48265823	113.6131194	102.8919062	76.92759295	75.04892368	71.60469667	91.64383562	90.4616939	118.0277309	109.1793733	124.4938383	107.1374447	76.93528478	74.42270059	81.3111546	91.64383562	76.30136986	87.88649706	55.63600783	115.3051593	103.7342302	102.032623	103.0535873	73.48336595	91.95694716	62.52446184	59.39334638	98.62940856	69.09980431

AMP2CPR3ERY3FUS3DOX3	AMP	CPR	ERY	FUS	DOX	AMP+CPR	AMP+ERY	AMP+FUS	AMP+DOX	CPR+ERY	CPR+FUS	CPR+DOX	ERY+FUS	ERY+DOX	FUS+DOX	AMP+CPR+ERY	AMP+CPR+FUS	AMP+CPR+DOX	AMP+ERY+FUS	AMP+ERY+DOX	AMP+FUS+DOX	CPR+ERY+FUS	CPR+ERY+DOX	CPR+FUS+DOX	ERY+FUS+DOX	AMP+CPR+ERY+FUS	AMP+CPR+ERY+DOX	AMP+CPR+FUS+DOX	AMP+ERY+FUS+DOX	CPR+ERY+FUS+DOX	AMP+CPR+ERY+FUS+DOX
	65.31489441	92.70957489	99.06634883	103.6182389	107.753369	82.05942814	72.49112315	66.21192301	86.54457111	97.47715534	118.4545093	105.4231228	116.8653159	104.151768	97.7109101	89.23565689	66.51093254	80.86339002	59.93272286	92.2257522	65.91291347	111.462058	106.0588002	107.0123162	114.0047676	62.3247991	82.65744721	76.67725659	75.48121846	100.6555423	75.18220893
	83.96462219	82.75002596	82.75002596	117.5362031	118.8057925	69.19823111	89.1943857	79.04249183	88.27148625	91.39059092	110.0010385	116.9799564	110.6656974	120.9679094	108.3316802	68.58296481	85.19515478	89.1943857	87.04095366	86.42568737	73.19746203	112.6596739	109.3363797	124.6235331	116.3152975	77.19669294	82.7340896	76.8890598	79.96539127	112.3273445	71.04402999
	70.66536204	88.76008668	91.48265823	113.6131194	99.22412273	76.92759295	75.04892368	71.60469667	93.52250489	90.4616939	118.0277309	109.1793733	124.4938383	103.0535873	102.3276318	74.42270059	81.3111546	81.93737769	76.30136986	87.26027397	61.27201566	115.3051593	108.8390519	123.472874	124.8341598	73.48336595	85.69471624	73.7964775	68.47358121	119.7293381	67.53424658

AMP3CPR1ERY1FUS1DOX1	AMP	CPR	ERY	FUS	DOX	AMP+CPR	AMP+ERY	AMP+FUS	AMP+DOX	CPR+ERY	CPR+FUS	CPR+DOX	ERY+FUS	ERY+DOX	FUS+DOX	AMP+CPR+ERY	AMP+CPR+FUS	AMP+CPR+DOX	AMP+ERY+FUS	AMP+ERY+DOX	AMP+FUS+DOX	CPR+ERY+FUS	CPR+ERY+DOX	CPR+FUS+DOX	ERY+FUS+DOX	AMP+CPR+ERY+FUS	AMP+CPR+ERY+DOX	AMP+CPR+FUS+DOX	AMP+ERY+FUS+DOX	CPR+ERY+FUS+DOX	AMP+CPR+ERY+FUS+DOX
	75.22844656	89.73601625	94.46187927	65.33136422	108.9348348	73.63925308	71.0965435	31.0488677	96.84147795	87.07771829	66.40206757	105.9811704	61.19623408	105.0950711	58.27949049	90.16686532	25.96344855	80.31386571	24.55303933	96.84147795	37.26658721	74.48772383	93.28041351	55.3258261	49.71386376	33.27373858	100.973381	43.30552245	37.26658721	45.28336718	29.77751291
	71.78315505	97.22277326	92.14441579	86.92719698	111.1882563	96.37553225	70.45383737	46.44303666	89.72894382	105.7925015	82.16623686	114.6796271	80.26185281	102.6185281	61.53540964	95.7108734	38.88254232	85.40866134	28.58033025	86.4056496	50.43098972	83.43582622	97.5401706	59.05574291	47.6294386	36.55623637	79.75906117	46.77536608	36.14082459	45.40765721	39.13178939
	77.18915757	102.0454946	100.9169459	63.3926997	104.5847293	90.80201534	84.3359079	43.0506627	89.78105101	96.96702522	74.67818727	104.3025921	71.01040381	109.6631987	57.78522306	87.3988009	41.45540593	87.05847945	36.9248767	94.88587267	68.23444957	50.76706048	99.50625992	56.09239993	47.62828425	35.32961993	99.65037289	38.62648392	20.972309	46.4997355	37.94584103

AMP3CPR1ERY1FUS1DOX2	AMP	CPR	ERY	FUS	DOX	AMP+CPR	AMP+ERY	AMP+FUS	AMP+DOX	CPR+ERY	CPR+FUS	CPR+DOX	ERY+FUS	ERY+DOX	FUS+DOX	AMP+CPR+ERY	AMP+CPR+FUS	AMP+CPR+DOX	AMP+ERY+FUS	AMP+ERY+DOX	AMP+FUS+DOX	CPR+ERY+FUS	CPR+ERY+DOX	CPR+FUS+DOX	ERY+FUS+DOX	AMP+CPR+ERY+FUS	AMP+CPR+ERY+DOX	AMP+CPR+FUS+DOX	AMP+ERY+FUS+DOX	CPR+ERY+FUS+DOX	AMP+CPR+ERY+FUS+DOX
	75.22844656	89.73601625	94.46187927	65.33136422	113.9560642	73.63925308	71.0965435	31.0488677	98.43067143	87.07771829	66.40206757	115.7282629	61.19623408	107.1626361	61.19623408	90.16686532	25.96344855	81.9030592	24.55303933	103.1982519	37.9022646	74.48772383	100.6645745	55.91655898	45.57873362	33.27373858	94.93444577	38.85578069	25.50655542	43.80653498	33.77036154
	71.78315505	97.22277326	92.14441579	86.92719698	121.6623686	96.37553225	70.45383737	46.44303666	91.72292034	105.7925015	82.16623686	119.7579845	80.26185281	118.1709978	62.17020432	95.7108734	38.88254232	88.06729671	28.58033025	100.3634853	33.1498598	83.43582622	105.4751041	52.64828407	44.13806784	36.55623637	94.71388514	42.4550836	44.44906013	50.80341202	60.40087236
	77.18915757	102.0454946	100.9169459	63.3926997	102.8919062	90.80201534	84.3359079	43.0506627	96.92780134	96.96702522	74.67818727	108.53465	71.01040381	101.4812202	51.01393052	87.3988009	41.45540593	83.99558646	36.9248767	100.3310158	30.11844782	50.76706048	97.24916241	54.68171398	46.78187269	35.32961993	93.18426545	45.43291281	44.41194847	41.70340328	88.31341478

AMP3CPR1ERY1FUS1DOX3	AMP	CPR	ERY	FUS	DOX	AMP+CPR	AMP+ERY	AMP+FUS	AMP+DOX	CPR+ERY	CPR+FUS	CPR+DOX	ERY+FUS	ERY+DOX	FUS+DOX	AMP+CPR+ERY	AMP+CPR+FUS	AMP+CPR+DOX	AMP+ERY+FUS	AMP+ERY+DOX	AMP+FUS+DOX	CPR+ERY+FUS	CPR+ERY+DOX	CPR+FUS+DOX	ERY+FUS+DOX	AMP+CPR+ERY+FUS	AMP+CPR+ERY+DOX	AMP+CPR+FUS+DOX	AMP+ERY+FUS+DOX	CPR+ERY+FUS+DOX	AMP+CPR+ERY+FUS+DOX
	75.22844656	89.73601625	94.46187927	65.33136422	107.753369	73.63925308	71.0965435	31.0488677	94.29876838	87.07771829	66.40206757	111.0023999	61.19623408	103.6182389	80.39505261	90.16686532	25.96344855	85.08144617	24.55303933	93.02741359	28.36710369	74.48772383	108.9348348	82.46261769	73.89699095	33.27373858	92.0738975	44.89471593	29.63845848	60.31013476	43.30552245
	71.78315505	97.22277326	92.14441579	86.92719698	118.8057925	96.37553225	70.45383737	46.44303666	91.39059092	105.7925015	82.16623686	115.6318191	80.26185281	113.0926403	82.16623686	95.7108734	38.88254232	102.6897913	28.58033025	87.73496729	33.48218922	83.43582622	110.870859	84.70541559	74.866098	36.55623637	87.73496729	39.79644823	42.12275418	53.6004761	36.80548344
	77.18915757	102.0454946	100.9169459	63.3926997	99.22412273	90.80201534	84.3359079	43.0506627	94.54555123	96.96702522	74.67818727	109.0989244	71.01040381	98.65984835	74.74872157	87.3988009	41.45540593	98.62940856	36.9248767	86.71815801	41.34905548	50.76706048	101.4812202	79.26291659	71.64521248	35.32961993	85.69719368	55.64255613	42.02969836	61.20613648	44.75226992

AMP3CPR1ERY1FUS2DOX1	AMP	CPR	ERY	FUS	DOX	AMP+CPR	AMP+ERY	AMP+FUS	AMP+DOX	CPR+ERY	CPR+FUS	CPR+DOX	ERY+FUS	ERY+DOX	FUS+DOX	AMP+CPR+ERY	AMP+CPR+FUS	AMP+CPR+DOX	AMP+ERY+FUS	AMP+ERY+DOX	AMP+FUS+DOX	CPR+ERY+FUS	CPR+ERY+DOX	CPR+FUS+DOX	ERY+FUS+DOX	AMP+CPR+ERY+FUS	AMP+CPR+ERY+DOX	AMP+CPR+FUS+DOX	AMP+ERY+FUS+DOX	CPR+ERY+FUS+DOX	AMP+CPR+ERY+FUS+DOX
	75.22844656	89.73601625	94.46187927	92.3943142	108.9348348	73.63925308	71.0965435	52.34406039	96.84147795	87.07771829	81.17038951	105.9811704	87.66845117	105.0950711	74.19235739	90.16686532	45.98728645	80.31386571	37.72348033	96.84147795	53.15852205	62.67306627	93.28041351	74.78309027	65.33136422	40.26618991	100.973381	59.83313468	48.0731029	62.08233339	46.16607072
	71.78315505	97.22277326	92.14441579	105.1577068	111.1882563	96.37553225	70.45383737	55.4990134	89.72894382	105.7925015	86.43126364	114.6796271	89.9226344	102.6185281	87.24459433	95.7108734	52.5080486	85.40866134	41.54117769	86.4056496	45.11371897	80.89664749	97.5401706	81.53144217	64.39198572	49.51708381	79.75906117	48.76934261	52.75729567	64.07458838	45.11371897
	77.18915757	102.0454946	100.9169459	101.1990831	104.5847293	90.80201534	84.3359079	57.79083525	89.78105101	96.96702522	85.11726327	104.3025921	91.88855581	109.6631987	67.13101746	87.3988009	62.21501402	87.05847945	40.09412015	94.88587267	54.6215918	48.50996297	99.50625992	63.463234	53.024158	52.34569214	99.65037289	59.38609202	52.57966313	52.17774643	57.34416335

AMP3CPR1ERY1FUS2DOX2	AMP	CPR	ERY	FUS	DOX	AMP+CPR	AMP+ERY	AMP+FUS	AMP+DOX	CPR+ERY	CPR+FUS	CPR+DOX	ERY+FUS	ERY+DOX	FUS+DOX	AMP+CPR+ERY	AMP+CPR+FUS	AMP+CPR+DOX	AMP+ERY+FUS	AMP+ERY+DOX	AMP+FUS+DOX	CPR+ERY+FUS	CPR+ERY+DOX	CPR+FUS+DOX	ERY+FUS+DOX	AMP+CPR+ERY+FUS	AMP+CPR+ERY+DOX	AMP+CPR+FUS+DOX	AMP+ERY+FUS+DOX	CPR+ERY+FUS+DOX	AMP+CPR+ERY+FUS+DOX
	75.22844656	89.73601625	94.46187927	92.3943142	113.9560642	73.63925308	71.0965435	52.34406039	98.43067143	87.07771829	81.17038951	115.7282629	87.66845117	107.1626361	77.14602178	90.16686532	45.98728645	81.9030592	37.72348033	103.1982519	43.30552245	62.67306627	100.6645745	81.87188481	67.3989293	40.26618991	94.93444577	60.15097338	54.11203814	63.85453203	51.25148987
	71.78315505	97.22277326	92.14441579	105.1577068	121.6623686	96.37553225	70.45383737	55.4990134	91.72292034	105.7925015	86.43126364	119.7579845	89.9226344	118.1709978	83.11842888	95.7108734	52.5080486	88.06729671	41.54117769	100.3634853	46.11070724	80.89664749	105.4751041	78.99226344	81.84883952	49.51708381	94.71388514	60.81628414	47.44002492	62.17020432	45.77837782
	77.18915757	102.0454946	100.9169459	101.1990831	102.8919062	90.80201534	84.3359079	57.79083525	96.92780134	96.96702522	85.11726327	108.53465	91.88855581	101.4812202	64.30964557	87.3988009	62.21501402	83.99558646	40.09412015	100.3310158	51.89902025	48.50996297	97.24916241	70.79880092	61.48827367	52.34569214	93.18426545	61.76834213	44.07162703	49.63851173	47.81516292

AMP3CPR1ERY1FUS2DOX3	AMP	CPR	ERY	FUS	DOX	AMP+CPR	AMP+ERY	AMP+FUS	AMP+DOX	CPR+ERY	CPR+FUS	CPR+DOX	ERY+FUS	ERY+DOX	FUS+DOX	AMP+CPR+ERY	AMP+CPR+FUS	AMP+CPR+DOX	AMP+ERY+FUS	AMP+ERY+DOX	AMP+FUS+DOX	CPR+ERY+FUS	CPR+ERY+DOX	CPR+FUS+DOX	ERY+FUS+DOX	AMP+CPR+ERY+FUS	AMP+CPR+ERY+DOX	AMP+CPR+FUS+DOX	AMP+ERY+FUS+DOX	CPR+ERY+FUS+DOX	AMP+CPR+ERY+FUS+DOX
	75.22844656	89.73601625	94.46187927	92.3943142	107.753369	73.63925308	71.0965435	52.34406039	94.29876838	87.07771829	81.17038951	111.0023999	87.66845117	103.6182389	102.7321396	90.16686532	45.98728645	85.08144617	37.72348033	93.02741359	32.49900675	62.67306627	108.9348348	68.1742662	88.55455049	40.26618991	92.0738975	50.61581248	35.35955503	73.89699095	39.17361939
	71.78315505	97.22277326	92.14441579	105.1577068	118.8057925	96.37553225	70.45383737	55.4990134	91.39059092	105.7925015	86.43126364	115.6318191	89.9226344	113.0926403	98.80975997	95.7108734	52.5080486	102.6897913	41.54117769	87.73496729	30.24197736	80.89664749	110.870859	77.08787939	83.25729022	49.51708381	87.73496729	47.52310728	22.84764773	79.62705812	31.15588327
	77.18915757	102.0454946	100.9169459	101.1990831	99.22412273	90.80201534	84.3359079	57.79083525	94.54555123	96.96702522	85.11726327	109.0989244	91.88855581	98.65984835	93.58137895	87.3988009	62.21501402	98.62940856	40.09412015	86.71815801	34.54262659	48.50996297	101.4812202	62.82842532	80.32093105	52.34569214	85.69719368	46.79419858	25.3539476	71.3630753	63.81027079

AMP3CPR1ERY1FUS3DOX1	AMP	CPR	ERY	FUS	DOX	AMP+CPR	AMP+ERY	AMP+FUS	AMP+DOX	CPR+ERY	CPR+FUS	CPR+DOX	ERY+FUS	ERY+DOX	FUS+DOX	AMP+CPR+ERY	AMP+CPR+FUS	AMP+CPR+DOX	AMP+ERY+FUS	AMP+ERY+DOX	AMP+FUS+DOX	CPR+ERY+FUS	CPR+ERY+DOX	CPR+FUS+DOX	ERY+FUS+DOX	AMP+CPR+ERY+FUS	AMP+CPR+ERY+DOX	AMP+CPR+FUS+DOX	AMP+ERY+FUS+DOX	CPR+ERY+FUS+DOX	AMP+CPR+ERY+FUS+DOX
	75.22844656	89.73601625	94.46187927	103.6182389	108.9348348	73.63925308	71.0965435	87.30631704	96.84147795	87.07771829	105.9811704	105.9811704	96.52944434	105.0950711	79.69355732	90.16686532	72.68573699	80.31386571	83.49225268	96.84147795	70.14302741	84.71478678	93.28041351	77.33062581	72.01402991	80.94954311	100.973381	70.14302741	60.60786651	58.24256969	69.18951132
	71.78315505	97.22277326	92.14441579	117.5362031	111.1882563	96.37553225	70.45383737	82.75002596	89.72894382	105.7925015	118.1709978	114.6796271	110.870859	102.6185281	70.24399921	95.7108734	77.76508464	85.40866134	76.10343753	86.4056496	83.74701423	93.41400516	97.5401706	71.51358857	65.66157508	75.43877869	79.75906117	81.08837886	78.09741406	67.56595914	72.4478139
	77.18915757	102.0454946	100.9169459	113.6131194	104.5847293	90.80201534	84.3359079	85.01655079	89.78105101	96.96702522	104.3025921	104.3025921	106.5596896	109.6631987	58.59636748	87.3988009	82.29397924	87.05847945	83.99558646	94.88587267	77.18915757	94.14565332	99.50625992	66.77834597	56.05713278	85.35687223	99.65037289	72.08433591	58.47147814	56.40980427	69.36176435

AMP3CPR1ERY1FUS3DOX2	AMP	CPR	ERY	FUS	DOX	AMP+CPR	AMP+ERY	AMP+FUS	AMP+DOX	CPR+ERY	CPR+FUS	CPR+DOX	ERY+FUS	ERY+DOX	FUS+DOX	AMP+CPR+ERY	AMP+CPR+FUS	AMP+CPR+DOX	AMP+ERY+FUS	AMP+ERY+DOX	AMP+FUS+DOX	CPR+ERY+FUS	CPR+ERY+DOX	CPR+FUS+DOX	ERY+FUS+DOX	AMP+CPR+ERY+FUS	AMP+CPR+ERY+DOX	AMP+CPR+FUS+DOX	AMP+ERY+FUS+DOX	CPR+ERY+FUS+DOX	AMP+CPR+ERY+FUS+DOX
	75.22844656	89.73601625	94.46187927	103.6182389	113.9560642	73.63925308	71.0965435	87.30631704	98.43067143	87.07771829	105.9811704	115.7282629	96.52944434	107.1626361	86.78235186	90.16686532	72.68573699	81.9030592	83.49225268	103.1982519	66.96464044	84.71478678	100.6645745	79.39819088	68.1742662	80.94954311	94.93444577	66.50774732	61.5613826	62.67306627	65.69328566
	71.78315505	97.22277326	92.14441579	117.5362031	121.6623686	96.37553225	70.45383737	82.75002596	91.72292034	105.7925015	118.1709978	119.7579845	110.870859	118.1709978	76.90934338	95.7108734	77.76508464	88.06729671	76.10343753	100.3634853	86.4056496	93.41400516	105.4751041	79.44852212	66.29636977	75.43877869	94.71388514	82.41769654	71.11849621	64.53084705	79.75906117
	77.18915757	102.0454946	100.9169459	113.6131194	102.8919062	90.80201534	84.3359079	85.01655079	96.92780134	96.96702522	104.3025921	108.53465	106.5596896	101.4812202	76.93528478	87.3988009	82.29397924	83.99558646	83.99558646	100.3310158	69.7020858	94.14565332	97.24916241	69.88185505	58.1026274	85.35687223	93.18426545	110.5406591	57.79083525	56.97407865	65.95854991

AMP3CPR1ERY1FUS3DOX3	AMP	CPR	ERY	FUS	DOX	AMP+CPR	AMP+ERY	AMP+FUS	AMP+DOX	CPR+ERY	CPR+FUS	CPR+DOX	ERY+FUS	ERY+DOX	FUS+DOX	AMP+CPR+ERY	AMP+CPR+FUS	AMP+CPR+DOX	AMP+ERY+FUS	AMP+ERY+DOX	AMP+FUS+DOX	CPR+ERY+FUS	CPR+ERY+DOX	CPR+FUS+DOX	ERY+FUS+DOX	AMP+CPR+ERY+FUS	AMP+CPR+ERY+DOX	AMP+CPR+FUS+DOX	AMP+ERY+FUS+DOX	CPR+ERY+FUS+DOX	AMP+CPR+ERY+FUS+DOX
	75.22844656	89.73601625	94.46187927	103.6182389	107.753369	73.63925308	71.0965435	87.30631704	94.29876838	87.07771829	105.9811704	111.0023999	96.52944434	103.6182389	97.7109101	90.16686532	72.68573699	85.08144617	83.49225268	93.02741359	71.4143822	84.71478678	108.9348348	87.07771829	90.03138268	80.94954311	92.0738975	70.77870481	61.8792213	79.98892376	60.9257052
	71.78315505	97.22277326	92.14441579	117.5362031	118.8057925	96.37553225	70.45383737	82.75002596	91.39059092	105.7925015	118.1709978	115.6318191	110.870859	113.0926403	108.3316802	95.7108734	77.76508464	102.6897913	76.10343753	87.73496729	77.43275522	93.41400516	110.870859	101.3489387	94.36619718	75.43877869	87.73496729	90.06127324	75.77110811	94.04879984	73.11247274
	77.18915757	102.0454946	100.9169459	113.6131194	99.22412273	90.80201534	84.3359079	85.01655079	94.54555123	96.96702522	104.3025921	109.0989244	106.5596896	98.65984835	102.3276318	87.3988009	82.29397924	98.62940856	83.99558646	86.71815801	76.84883613	94.14565332	101.4812202	95.27420208	88.22077235	85.35687223	85.69719368	85.69719368	76.50851468	78.91024511	71.40369302

AMP3CPR1ERY2FUS1DOX1	AMP	CPR	ERY	FUS	DOX	AMP+CPR	AMP+ERY	AMP+FUS	AMP+DOX	CPR+ERY	CPR+FUS	CPR+DOX	ERY+FUS	ERY+DOX	FUS+DOX	AMP+CPR+ERY	AMP+CPR+FUS	AMP+CPR+DOX	AMP+ERY+FUS	AMP+ERY+DOX	AMP+FUS+DOX	CPR+ERY+FUS	CPR+ERY+DOX	CPR+FUS+DOX	ERY+FUS+DOX	AMP+CPR+ERY+FUS	AMP+CPR+ERY+DOX	AMP+CPR+FUS+DOX	AMP+ERY+FUS+DOX	CPR+ERY+FUS+DOX	AMP+CPR+ERY+FUS+DOX
	75.22844656	89.73601625	92.2257522	65.33136422	108.9348348	73.63925308	74.59276917	31.0488677	96.84147795	89.14528337	66.40206757	105.9811704	70.39805644	101.7940572	58.27949049	83.17441398	25.96344855	80.31386571	25.50655542	102.2447358	37.26658721	75.66918959	105.685804	55.3258261	56.43804896	31.0488677	93.34525228	43.30552245	66.82558602	45.57873362	28.36710369
	71.78315505	97.22277326	104.2684099	86.92719698	111.1882563	96.37553225	68.45986084	46.44303666	89.72894382	92.14441579	82.16623686	114.6796271	86.73332052	111.9592386	61.53540964	96.37553225	38.88254232	85.40866134	54.41894278	98.36950878	50.43098972	91.05336243	114.6796271	59.05574291	36.89675062	29.90964794	93.38456745	46.77536608	53.08962509	51.75560405	35.47616575
	77.18915757	102.0454946	95.40117417	63.3926997	104.5847293	90.80201534	78.2101219	43.0506627	89.78105101	96.40275084	74.67818727	104.3025921	81.93737769	103.5420744	57.78522306	89.10040812	41.45540593	87.05847945	42.37001981	97.26812278	68.23444957	52.74202081	102.609769	56.09239993	51.25244618	39.41347726	95.56651556	38.62648392	65.51187802	51.29606771	43.39098414

AMP3CPR1ERY2FUS1DOX2	AMP	CPR	ERY	FUS	DOX	AMP+CPR	AMP+ERY	AMP+FUS	AMP+DOX	CPR+ERY	CPR+FUS	CPR+DOX	ERY+FUS	ERY+DOX	FUS+DOX	AMP+CPR+ERY	AMP+CPR+FUS	AMP+CPR+DOX	AMP+ERY+FUS	AMP+ERY+DOX	AMP+FUS+DOX	CPR+ERY+FUS	CPR+ERY+DOX	CPR+FUS+DOX	ERY+FUS+DOX	AMP+CPR+ERY+FUS	AMP+CPR+ERY+DOX	AMP+CPR+FUS+DOX	AMP+ERY+FUS+DOX	CPR+ERY+FUS+DOX	AMP+CPR+ERY+FUS+DOX
	75.22844656	89.73601625	92.2257522	65.33136422	113.9560642	73.63925308	74.59276917	31.0488677	98.43067143	89.14528337	66.40206757	115.7282629	70.39805644	107.1762287	61.19623408	83.17441398	25.96344855	81.9030592	25.50655542	95.25228447	37.9022646	75.66918959	109.5255677	55.91655898	49.85983928	31.0488677	83.17441398	38.85578069	43.62336114	48.79084364	39.49145809
	71.78315505	97.22277326	104.2684099	86.92719698	121.6623686	96.37553225	68.45986084	46.44303666	91.72292034	92.14441579	82.16623686	119.7579845	86.73332052	117.4966353	62.17020432	96.37553225	38.88254232	88.06729671	54.41894278	94.38155572	33.1498598	91.05336243	113.727435	52.64828407	40.03076331	29.90964794	86.73797902	42.4550836	53.75428393	45.40765721	39.46411881
	77.18915757	102.0454946	95.40117417	63.3926997	102.8919062	90.80201534	78.2101219	43.0506627	96.92780134	96.40275084	74.67818727	108.53465	81.93737769	111.369863	51.01393052	89.10040812	41.45540593	83.99558646	42.37001981	106.7971232	30.11844782	52.74202081	106.2775525	54.68171398	48.43444227	39.41347726	89.44072956	45.43291281	64.15059224	44.80691236	93.077915

AMP3CPR1ERY2FUS1DOX3	AMP	CPR	ERY	FUS	DOX	AMP+CPR	AMP+ERY	AMP+FUS	AMP+DOX	CPR+ERY	CPR+FUS	CPR+DOX	ERY+FUS	ERY+DOX	FUS+DOX	AMP+CPR+ERY	AMP+CPR+FUS	AMP+CPR+DOX	AMP+ERY+FUS	AMP+ERY+DOX	AMP+FUS+DOX	CPR+ERY+FUS	CPR+ERY+DOX	CPR+FUS+DOX	ERY+FUS+DOX	AMP+CPR+ERY+FUS	AMP+CPR+ERY+DOX	AMP+CPR+FUS+DOX	AMP+ERY+FUS+DOX	CPR+ERY+FUS+DOX	AMP+CPR+ERY+FUS+DOX
	75.22844656	89.73601625	92.2257522	65.33136422	107.753369	73.63925308	74.59276917	31.0488677	94.29876838	89.14528337	66.40206757	111.0023999	70.39805644	109.8673145	80.39505261	83.17441398	25.96344855	85.08144617	25.50655542	96.20580056	28.36710369	75.66918959	107.4580026	82.46261769	59.63371332	31.0488677	85.08144617	44.89471593	46.16607072	76.85065534	44.25903854
	71.78315505	97.22277326	104.2684099	86.92719698	118.8057925	96.37553225	68.45986084	46.44303666	91.39059092	92.14441579	82.16623686	115.6318191	86.73332052	114.4203038	82.16623686	96.37553225	38.88254232	102.6897913	54.41894278	93.38456745	33.48218922	91.05336243	118.1709978	84.70541559	63.71851567	29.90964794	97.37252051	39.79644823	45.11371897	83.75322357	32.15287153
	77.18915757	102.0454946	95.40117417	63.3926997	99.22412273	90.80201534	78.2101219	43.0506627	94.54555123	96.40275084	74.67818727	109.0989244	81.93737769	111.0567515	74.74872157	89.10040812	41.45540593	98.62940856	42.37001981	98.28908711	41.34905548	52.74202081	102.8919062	79.26291659	80.43052838	39.41347726	96.92780134	55.64255613	50.19741303	71.92734967	48.83612725

AMP3CPR1ERY2FUS2DOX1	AMP	CPR	ERY	FUS	DOX	AMP+CPR	AMP+ERY	AMP+FUS	AMP+DOX	CPR+ERY	CPR+FUS	CPR+DOX	ERY+FUS	ERY+DOX	FUS+DOX	AMP+CPR+ERY	AMP+CPR+FUS	AMP+CPR+DOX	AMP+ERY+FUS	AMP+ERY+DOX	AMP+FUS+DOX	CPR+ERY+FUS	CPR+ERY+DOX	CPR+FUS+DOX	ERY+FUS+DOX	AMP+CPR+ERY+FUS	AMP+CPR+ERY+DOX	AMP+CPR+FUS+DOX	AMP+ERY+FUS+DOX	CPR+ERY+FUS+DOX	AMP+CPR+ERY+FUS+DOX
	75.22844656	89.73601625	92.2257522	92.3943142	108.9348348	73.63925308	74.59276917	52.34406039	96.84147795	89.14528337	81.17038951	105.9811704	90.13268548	101.7940572	74.19235739	83.17441398	45.98728645	80.31386571	48.52999603	102.2447358	53.15852205	64.92523537	105.685804	74.78309027	62.3247991	45.98728645	93.34525228	59.83313468	49.34445769	68.58039505	51.56932857
	71.78315505	97.22277326	104.2684099	105.1577068	111.1882563	96.37553225	68.45986084	55.4990134	89.72894382	92.14441579	86.43126364	114.6796271	101.8073447	111.9592386	87.24459433	96.37553225	52.5080486	85.40866134	47.19077786	98.36950878	45.11371897	80.57925015	114.6796271	81.53144217	64.64141511	44.86447191	93.38456745	48.76934261	49.43400145	78.04007141	40.46110707
	77.18915757	102.0454946	95.40117417	101.1990831	104.5847293	90.80201534	78.2101219	57.79083525	89.78105101	96.40275084	85.11726327	104.3025921	98.84540117	103.5420744	67.13101746	89.10040812	62.21501402	87.05847945	48.60215626	97.26812278	54.6215918	69.03544348	102.609769	63.463234	64.46183953	57.11019236	95.56651556	59.38609202	50.53773447	60.35972492	52.91998458

AMP3CPR1ERY2FUS2DOX2	AMP	CPR	ERY	FUS	DOX	AMP+CPR	AMP+ERY	AMP+FUS	AMP+DOX	CPR+ERY	CPR+FUS	CPR+DOX	ERY+FUS	ERY+DOX	FUS+DOX	AMP+CPR+ERY	AMP+CPR+FUS	AMP+CPR+DOX	AMP+ERY+FUS	AMP+ERY+DOX	AMP+FUS+DOX	CPR+ERY+FUS	CPR+ERY+DOX	CPR+FUS+DOX	ERY+FUS+DOX	AMP+CPR+ERY+FUS	AMP+CPR+ERY+DOX	AMP+CPR+FUS+DOX	AMP+ERY+FUS+DOX	CPR+ERY+FUS+DOX	AMP+CPR+ERY+FUS+DOX
	75.22844656	89.73601625	92.2257522	92.3943142	113.9560642	73.63925308	74.59276917	52.34406039	98.43067143	89.14528337	81.17038951	115.7282629	90.13268548	107.1762287	77.14602178	83.17441398	45.98728645	81.9030592	48.52999603	95.25228447	43.30552245	64.92523537	109.5255677	81.87188481	71.29508503	45.98728645	83.17441398	60.15097338	53.47636075	73.60162452	55.70123162
	71.78315505	97.22277326	104.2684099	105.1577068	121.6623686	96.37553225	68.45986084	55.4990134	91.72292034	92.14441579	86.43126364	119.7579845	101.8073447	117.4966353	83.11842888	96.37553225	52.5080486	88.06729671	47.19077786	94.38155572	46.11070724	80.57925015	113.727435	78.99226344	71.7169775	44.86447191	86.73797902	60.81628414	50.76331914	73.59650863	45.77837782
	77.18915757	102.0454946	95.40117417	101.1990831	102.8919062	90.80201534	78.2101219	57.79083525	96.92780134	96.40275084	85.11726327	108.53465	98.84540117	111.369863	64.30964557	89.10040812	62.21501402	83.99558646	48.60215626	106.7971232	51.89902025	69.03544348	106.2775525	70.79880092	66.9667319	57.11019236	89.44072956	61.76834213	50.87805591	60.92399929	50.19741303

AMP3CPR1ERY2FUS2DOX3	AMP	CPR	ERY	FUS	DOX	AMP+CPR	AMP+ERY	AMP+FUS	AMP+DOX	CPR+ERY	CPR+FUS	CPR+DOX	ERY+FUS	ERY+DOX	FUS+DOX	AMP+CPR+ERY	AMP+CPR+FUS	AMP+CPR+DOX	AMP+ERY+FUS	AMP+ERY+DOX	AMP+FUS+DOX	CPR+ERY+FUS	CPR+ERY+DOX	CPR+FUS+DOX	ERY+FUS+DOX	AMP+CPR+ERY+FUS	AMP+CPR+ERY+DOX	AMP+CPR+FUS+DOX	AMP+ERY+FUS+DOX	CPR+ERY+FUS+DOX	AMP+CPR+ERY+FUS+DOX
	75.22844656	89.73601625	92.2257522	92.3943142	107.753369	73.63925308	74.59276917	52.34406039	94.29876838	89.14528337	81.17038951	111.0023999	90.13268548	109.8673145	102.7321396	83.17441398	45.98728645	85.08144617	48.52999603	96.20580056	32.49900675	64.92523537	107.4580026	68.1742662	106.2792001	45.98728645	85.08144617	50.61581248	37.5844259	74.19235739	52.52284466
	71.78315505	97.22277326	104.2684099	105.1577068	118.8057925	96.37553225	68.45986084	55.4990134	91.39059092	92.14441579	86.43126364	115.6318191	101.8073447	114.4203038	98.80975997	96.37553225	52.5080486	102.6897913	47.19077786	93.38456745	30.24197736	80.57925015	118.1709978	77.08787939	107.0371082	44.86447191	97.37252051	47.52310728	35.47616575	83.11842888	40.79343649
	77.18915757	102.0454946	95.40117417	101.1990831	99.22412273	90.80201534	78.2101219	57.79083525	94.54555123	96.40275084	85.11726327	109.0989244	98.84540117	111.0567515	93.58137895	89.10040812	62.21501402	98.62940856	48.60215626	98.28908711	34.54262659	69.03544348	102.8919062	62.82842532	105.1076321	57.11019236	96.92780134	46.79419858	26.71523337	68.25956621	42.71034125

AMP3CPR1ERY2FUS3DOX1	AMP	CPR	ERY	FUS	DOX	AMP+CPR	AMP+ERY	AMP+FUS	AMP+DOX	CPR+ERY	CPR+FUS	CPR+DOX	ERY+FUS	ERY+DOX	FUS+DOX	AMP+CPR+ERY	AMP+CPR+FUS	AMP+CPR+DOX	AMP+ERY+FUS	AMP+ERY+DOX	AMP+FUS+DOX	CPR+ERY+FUS	CPR+ERY+DOX	CPR+FUS+DOX	ERY+FUS+DOX	AMP+CPR+ERY+FUS	AMP+CPR+ERY+DOX	AMP+CPR+FUS+DOX	AMP+ERY+FUS+DOX	CPR+ERY+FUS+DOX	AMP+CPR+ERY+FUS+DOX
	75.22844656	89.73601625	92.2257522	103.6182389	108.9348348	73.63925308	74.59276917	87.30631704	96.84147795	89.14528337	105.9811704	105.9811704	102.0930667	101.7940572	79.69355732	83.17441398	72.68573699	80.31386571	81.9030592	102.2447358	70.14302741	97.41554366	105.685804	77.33062581	79.06933283	80.31386571	93.34525228	70.14302741	54.56893127	74.08159498	75.22844656
	71.78315505	97.22277326	104.2684099	117.5362031	111.1882563	96.37553225	68.45986084	82.75002596	89.72894382	92.14441579	118.1709978	114.6796271	117.4966353	111.9592386	70.24399921	96.37553225	77.76508464	85.40866134	80.09139059	98.36950878	83.74701423	101.666336	114.6796271	71.51358857	74.73562776	75.43877869	93.38456745	81.08837886	83.08235538	65.48303908	78.09741406
	77.18915757	102.0454946	95.40117417	113.6131194	104.5847293	90.80201534	78.2101219	85.01655079	89.78105101	96.40275084	104.3025921	104.3025921	112.6223092	103.5420744	58.59636748	89.10040812	82.29397924	87.05847945	81.2730149	97.26812278	77.18915757	102.0454946	102.609769	66.77834597	88.51272016	86.03751512	95.56651556	72.08433591	64.93758558	65.08552284	75.48755035

AMP3CPR1ERY2FUS3DOX2	AMP	CPR	ERY	FUS	DOX	AMP+CPR	AMP+ERY	AMP+FUS	AMP+DOX	CPR+ERY	CPR+FUS	CPR+DOX	ERY+FUS	ERY+DOX	FUS+DOX	AMP+CPR+ERY	AMP+CPR+FUS	AMP+CPR+DOX	AMP+ERY+FUS	AMP+ERY+DOX	AMP+FUS+DOX	CPR+ERY+FUS	CPR+ERY+DOX	CPR+FUS+DOX	ERY+FUS+DOX	AMP+CPR+ERY+FUS	AMP+CPR+ERY+DOX	AMP+CPR+FUS+DOX	AMP+ERY+FUS+DOX	CPR+ERY+FUS+DOX	AMP+CPR+ERY+FUS+DOX
	75.22844656	89.73601625	92.2257522	103.6182389	113.9560642	73.63925308	74.59276917	87.30631704	98.43067143	89.14528337	105.9811704	115.7282629	102.0930667	107.1762287	86.78235186	83.17441398	72.68573699	81.9030592	81.9030592	95.25228447	66.96464044	97.41554366	109.5255677	79.39819088	86.54457111	80.31386571	83.17441398	66.50774732	59.33651172	67.87889976	59.01867302
	71.78315505	97.22277326	104.2684099	117.5362031	121.6623686	96.37553225	68.45986084	82.75002596	91.72292034	92.14441579	118.1709978	119.7579845	117.4966353	117.4966353	76.90934338	96.37553225	77.76508464	88.06729671	80.09139059	94.38155572	86.4056496	101.666336	113.727435	79.44852212	78.11959239	75.43877869	86.73797902	82.41769654	78.09741406	76.59194604	75.10644927
	77.18915757	102.0454946	95.40117417	113.6131194	102.8919062	90.80201534	78.2101219	85.01655079	96.92780134	96.40275084	104.3025921	108.53465	112.6223092	111.369863	76.93528478	89.10040812	82.29397924	83.99558646	81.2730149	106.7971232	69.7020858	102.0454946	106.2775525	69.88185505	78.18003914	86.03751512	89.44072956	110.5406591	69.36176435	72.13895257	97.94876567

AMP3CPR1ERY2FUS3DOX3	AMP	CPR	ERY	FUS	DOX	AMP+CPR	AMP+ERY	AMP+FUS	AMP+DOX	CPR+ERY	CPR+FUS	CPR+DOX	ERY+FUS	ERY+DOX	FUS+DOX	AMP+CPR+ERY	AMP+CPR+FUS	AMP+CPR+DOX	AMP+ERY+FUS	AMP+ERY+DOX	AMP+FUS+DOX	CPR+ERY+FUS	CPR+ERY+DOX	CPR+FUS+DOX	ERY+FUS+DOX	AMP+CPR+ERY+FUS	AMP+CPR+ERY+DOX	AMP+CPR+FUS+DOX	AMP+ERY+FUS+DOX	CPR+ERY+FUS+DOX	AMP+CPR+ERY+FUS+DOX
	75.22844656	89.73601625	92.2257522	103.6182389	107.753369	73.63925308	74.59276917	87.30631704	94.29876838	89.14528337	105.9811704	111.0023999	102.0930667	109.8673145	97.7109101	83.17441398	72.68573699	85.08144617	81.9030592	96.20580056	71.4143822	97.41554366	107.4580026	87.07771829	109.8673145	80.31386571	85.08144617	70.77870481	66.64680175	82.35185527	62.51489869
	71.78315505	97.22277326	104.2684099	117.5362031	118.8057925	96.37553225	68.45986084	82.75002596	91.39059092	92.14441579	118.1709978	115.6318191	117.4966353	114.4203038	108.3316802	96.37553225	77.76508464	102.6897913	80.09139059	93.38456745	77.43275522	101.666336	118.1709978	101.3489387	102.1149779	75.43877869	97.37252051	90.06127324	79.42673175	89.28783971	71.78315505
	77.18915757	102.0454946	95.40117417	113.6131194	99.22412273	90.80201534	78.2101219	85.01655079	94.54555123	96.40275084	104.3025921	109.0989244	112.6223092	111.0567515	102.3276318	89.10040812	82.29397924	98.62940856	81.2730149	98.28908711	76.84883613	102.0454946	102.8919062	95.27420208	108.2387476	86.03751512	96.92780134	85.69719368	77.52947902	81.16734262	73.78594313

AMP3CPR1ERY3FUS1DOX1	AMP	CPR	ERY	FUS	DOX	AMP+CPR	AMP+ERY	AMP+FUS	AMP+DOX	CPR+ERY	CPR+FUS	CPR+DOX	ERY+FUS	ERY+DOX	FUS+DOX	AMP+CPR+ERY	AMP+CPR+FUS	AMP+CPR+DOX	AMP+ERY+FUS	AMP+ERY+DOX	AMP+FUS+DOX	CPR+ERY+FUS	CPR+ERY+DOX	CPR+FUS+DOX	ERY+FUS+DOX	AMP+CPR+ERY+FUS	AMP+CPR+ERY+DOX	AMP+CPR+FUS+DOX	AMP+ERY+FUS+DOX	CPR+ERY+FUS+DOX	AMP+CPR+ERY+FUS+DOX
	75.22844656	89.73601625	99.06634883	65.33136422	108.9348348	73.63925308	75.22844656	31.0488677	96.84147795	92.68968064	66.40206757	105.9811704	105.4231228	106.0588002	58.27949049	81.2673818	25.96344855	80.31386571	35.04171633	91.7560588	37.26658721	71.238693	107.753369	55.3258261	59.51529599	36.13428685	79.36034962	43.30552245	46.16607072	50.0092302	41.39849027
	71.78315505	97.22277326	82.75002596	86.92719698	111.1882563	96.37553225	71.78315505	46.44303666	89.72894382	96.90537592	82.16623686	114.6796271	94.38155572	94.71388514	61.53540964	95.7108734	38.88254232	85.40866134	37.8024717	82.75002596	50.43098972	91.37075977	111.5056536	59.05574291	70.70308443	28.91265967	83.41468481	46.77536608	51.09564856	53.34259076	50.43098972
	77.18915757	102.0454946	91.48265823	63.3926997	104.5847293	90.80201534	74.80690746	43.0506627	89.78105101	105.7132781	74.67818727	104.3025921	95.906837	103.3939088	57.78522306	87.3988009	41.45540593	87.05847945	51.5586988	86.37783657	68.23444957	52.45988362	100.3526715	56.09239993	74.70055701	40.77476304	88.41976523	38.62648392	50.87805591	57.78522306	49.51677014

AMP3CPR1ERY3FUS1DOX2	AMP	CPR	ERY	FUS	DOX	AMP+CPR	AMP+ERY	AMP+FUS	AMP+DOX	CPR+ERY	CPR+FUS	CPR+DOX	ERY+FUS	ERY+DOX	FUS+DOX	AMP+CPR+ERY	AMP+CPR+FUS	AMP+CPR+DOX	AMP+ERY+FUS	AMP+ERY+DOX	AMP+FUS+DOX	CPR+ERY+FUS	CPR+ERY+DOX	CPR+FUS+DOX	ERY+FUS+DOX	AMP+CPR+ERY+FUS	AMP+CPR+ERY+DOX	AMP+CPR+FUS+DOX	AMP+ERY+FUS+DOX	CPR+ERY+FUS+DOX	AMP+CPR+ERY+FUS+DOX
	75.22844656	89.73601625	99.06634883	65.33136422	113.9560642	73.63925308	75.22844656	31.0488677	98.43067143	92.68968064	66.40206757	115.7282629	105.4231228	116.2296385	61.19623408	81.2673818	25.96344855	81.9030592	35.04171633	97.79499404	37.9022646	71.238693	111.8884992	55.91655898	65.55423123	36.13428685	79.67818832	38.85578069	52.20500596	48.23703157	38.85578069
	71.78315505	97.22277326	82.75002596	86.92719698	121.6623686	96.37553225	71.78315505	46.44303666	91.72292034	96.90537592	82.16623686	119.7579845	94.38155572	106.6777443	62.17020432	95.7108734	38.88254232	88.06729671	37.8024717	94.0492263	33.1498598	91.37075977	119.1231898	52.64828407	81.0052965	28.91265967	96.37553225	42.4550836	45.11371897	47.6294386	55.41593104
	77.18915757	102.0454946	91.48265823	63.3926997	102.8919062	90.80201534	74.80690746	43.0506627	96.92780134	105.7132781	74.67818727	108.53465	95.906837	107.1374447	51.01393052	87.3988009	41.45540593	83.99558646	51.5586988	93.86490834	30.11844782	52.45988362	109.6631987	54.68171398	71.29734257	40.77476304	85.69719368	45.43291281	54.6215918	54.11743961	97.84241522

AMP3CPR1ERY3FUS1DOX3	AMP	CPR	ERY	FUS	DOX	AMP+CPR	AMP+ERY	AMP+FUS	AMP+DOX	CPR+ERY	CPR+FUS	CPR+DOX	ERY+FUS	ERY+DOX	FUS+DOX	AMP+CPR+ERY	AMP+CPR+FUS	AMP+CPR+DOX	AMP+ERY+FUS	AMP+ERY+DOX	AMP+FUS+DOX	CPR+ERY+FUS	CPR+ERY+DOX	CPR+FUS+DOX	ERY+FUS+DOX	AMP+CPR+ERY+FUS	AMP+CPR+ERY+DOX	AMP+CPR+FUS+DOX	AMP+ERY+FUS+DOX	CPR+ERY+FUS+DOX	AMP+CPR+ERY+FUS+DOX
	75.22844656	89.73601625	99.06634883	65.33136422	107.753369	73.63925308	75.22844656	31.0488677	94.29876838	92.68968064	66.40206757	111.0023999	105.4231228	104.151768	80.39505261	81.2673818	25.96344855	85.08144617	35.04171633	84.76360747	28.36710369	71.238693	110.1163005	82.46261769	76.04290822	36.13428685	79.04251093	44.89471593	34.08820024	80.69041905	44.89471593
	71.78315505	97.22277326	82.75002596	86.92719698	118.8057925	96.37553225	71.78315505	46.44303666	91.39059092	96.90537592	82.16623686	115.6318191	94.38155572	120.9679094	82.16623686	95.7108734	38.88254232	102.6897913	37.8024717	86.4056496	33.48218922	91.37075977	118.1709978	84.70541559	77.68200228	28.91265967	105.0160972	39.79644823	39.46411881	89.14897838	37.8024717
	77.18915757	102.0454946	91.48265823	63.3926997	99.22412273	90.80201534	74.80690746	43.0506627	94.54555123	105.7132781	74.67818727	109.0989244	95.906837	103.0535873	74.74872157	87.3988009	41.45540593	98.62940856	51.5586988	90.80201534	41.34905548	52.45988362	104.5847293	79.26291659	80.93269346	40.77476304	88.41976523	55.64255613	52.57966313	76.15940751	52.23934169

AMP3CPR1ERY3FUS2DOX1	AMP	CPR	ERY	FUS	DOX	AMP+CPR	AMP+ERY	AMP+FUS	AMP+DOX	CPR+ERY	CPR+FUS	CPR+DOX	ERY+FUS	ERY+DOX	FUS+DOX	AMP+CPR+ERY	AMP+CPR+FUS	AMP+CPR+DOX	AMP+ERY+FUS	AMP+ERY+DOX	AMP+FUS+DOX	CPR+ERY+FUS	CPR+ERY+DOX	CPR+FUS+DOX	ERY+FUS+DOX	AMP+CPR+ERY+FUS	AMP+CPR+ERY+DOX	AMP+CPR+FUS+DOX	AMP+ERY+FUS+DOX	CPR+ERY+FUS+DOX	AMP+CPR+ERY+FUS+DOX
	75.22844656	89.73601625	99.06634883	92.3943142	108.9348348	73.63925308	75.22844656	52.34406039	96.84147795	92.68968064	81.17038951	105.9811704	110.1907032	106.0588002	74.19235739	81.2673818	45.98728645	80.31386571	48.84783472	91.7560588	53.15852205	78.51209156	107.753369	74.78309027	75.40723083	45.03377036	79.36034962	59.83313468	53.15852205	69.46649437	56.33690902
	71.78315505	97.22277326	82.75002596	105.1577068	111.1882563	96.37553225	71.78315505	55.4990134	89.72894382	96.90537592	86.43126364	114.6796271	105.3484266	94.71388514	87.24459433	95.7108734	52.5080486	85.40866134	50.18174265	82.75002596	45.11371897	79.76591946	111.5056536	81.53144217	80.00830824	42.53816596	83.41468481	48.76934261	55.74826046	75.50089268	46.44303666
	77.18915757	102.0454946	91.48265823	101.1990831	104.5847293	90.80201534	74.80690746	57.79083525	89.78105101	105.7132781	85.11726327	104.3025921	110.8809806	103.3939088	67.13101746	87.3988009	62.21501402	87.05847945	54.72794225	86.37783657	54.6215918	78.06383354	100.3526715	63.463234	82.18762878	57.79083525	88.41976523	59.38609202	60.06673491	58.1026274	75.04087845

AMP3CPR1ERY3FUS2DOX2	AMP	CPR	ERY	FUS	DOX	AMP+CPR	AMP+ERY	AMP+FUS	AMP+DOX	CPR+ERY	CPR+FUS	CPR+DOX	ERY+FUS	ERY+DOX	FUS+DOX	AMP+CPR+ERY	AMP+CPR+FUS	AMP+CPR+DOX	AMP+ERY+FUS	AMP+ERY+DOX	AMP+FUS+DOX	CPR+ERY+FUS	CPR+ERY+DOX	CPR+FUS+DOX	ERY+FUS+DOX	AMP+CPR+ERY+FUS	AMP+CPR+ERY+DOX	AMP+CPR+FUS+DOX	AMP+ERY+FUS+DOX	CPR+ERY+FUS+DOX	AMP+CPR+ERY+FUS+DOX
	75.22844656	89.73601625	99.06634883	92.3943142	113.9560642	73.63925308	75.22844656	52.34406039	98.43067143	92.68968064	81.17038951	115.7282629	110.1907032	116.2296385	77.14602178	81.2673818	45.98728645	81.9030592	48.84783472	97.79499404	43.30552245	78.51209156	111.8884992	81.87188481	69.36829559	45.03377036	79.67818832	60.15097338	56.97258641	71.238693	57.29042511
	71.78315505	97.22277326	82.75002596	105.1577068	121.6623686	96.37553225	71.78315505	55.4990134	91.72292034	96.90537592	86.43126364	119.7579845	105.3484266	106.6777443	83.11842888	95.7108734	52.5080486	88.06729671	50.18174265	94.0492263	46.11070724	79.76591946	119.1231898	78.99226344	73.02939038	42.53816596	96.37553225	60.81628414	47.1076955	78.6748661	50.0986603
	77.18915757	102.0454946	91.48265823	101.1990831	102.8919062	90.80201534	74.80690746	57.79083525	96.92780134	105.7132781	85.11726327	108.53465	110.8809806	107.1374447	64.30964557	87.3988009	62.21501402	83.99558646	54.72794225	93.86490834	51.89902025	78.06383354	109.6631987	70.79880092	75.72152134	57.79083525	85.69719368	61.76834213	59.72641346	65.72033151	53.94094891

AMP3CPR1ERY3FUS2DOX3	AMP	CPR	ERY	FUS	DOX	AMP+CPR	AMP+ERY	AMP+FUS	AMP+DOX	CPR+ERY	CPR+FUS	CPR+DOX	ERY+FUS	ERY+DOX	FUS+DOX	AMP+CPR+ERY	AMP+CPR+FUS	AMP+CPR+DOX	AMP+ERY+FUS	AMP+ERY+DOX	AMP+FUS+DOX	CPR+ERY+FUS	CPR+ERY+DOX	CPR+FUS+DOX	ERY+FUS+DOX	AMP+CPR+ERY+FUS	AMP+CPR+ERY+DOX	AMP+CPR+FUS+DOX	AMP+ERY+FUS+DOX	CPR+ERY+FUS+DOX	AMP+CPR+ERY+FUS+DOX
	75.22844656	89.73601625	99.06634883	92.3943142	107.753369	73.63925308	75.22844656	52.34406039	94.29876838	92.68968064	81.17038951	111.0023999	110.1907032	104.151768	102.7321396	81.2673818	45.98728645	85.08144617	48.84783472	84.76360747	32.49900675	78.51209156	110.1163005	68.1742662	111.462058	45.03377036	79.04251093	50.61581248	31.54549066	76.14916005	50.61581248
	71.78315505	97.22277326	82.75002596	105.1577068	118.8057925	96.37553225	71.78315505	55.4990134	91.39059092	96.90537592	86.43126364	115.6318191	105.3484266	120.9679094	98.80975997	95.7108734	52.5080486	102.6897913	50.18174265	86.4056496	30.24197736	79.76591946	118.1709978	77.08787939	118.9739329	42.53816596	105.0160972	47.52310728	30.49122443	70.73993255	40.21186001
	77.18915757	102.0454946	91.48265823	101.1990831	99.22412273	90.80201534	74.80690746	57.79083525	94.54555123	105.7132781	85.11726327	109.0989244	110.8809806	103.0535873	93.58137895	87.3988009	62.21501402	98.62940856	54.72794225	90.80201534	34.54262659	78.06383354	104.5847293	62.82842532	118.7083738	57.79083525	88.41976523	46.79419858	33.8619837	69.59971786	58.36512769

AMP3CPR1ERY3FUS3DOX1	AMP	CPR	ERY	FUS	DOX	AMP+CPR	AMP+ERY	AMP+FUS	AMP+DOX	CPR+ERY	CPR+FUS	CPR+DOX	ERY+FUS	ERY+DOX	FUS+DOX	AMP+CPR+ERY	AMP+CPR+FUS	AMP+CPR+DOX	AMP+ERY+FUS	AMP+ERY+DOX	AMP+FUS+DOX	CPR+ERY+FUS	CPR+ERY+DOX	CPR+FUS+DOX	ERY+FUS+DOX	AMP+CPR+ERY+FUS	AMP+CPR+ERY+DOX	AMP+CPR+FUS+DOX	AMP+ERY+FUS+DOX	CPR+ERY+FUS+DOX	AMP+CPR+ERY+FUS+DOX
	75.22844656	89.73601625	99.06634883	103.6182389	108.9348348	73.63925308	75.22844656	87.30631704	96.84147795	92.68968064	105.9811704	105.9811704	116.8653159	106.0588002	79.69355732	81.2673818	72.68573699	80.31386571	79.99602702	91.7560588	70.14302741	104.2089718	107.753369	77.33062581	97.79499404	74.59276917	79.36034962	70.14302741	75.22844656	68.76499908	73.00357569
	71.78315505	97.22277326	82.75002596	117.5362031	111.1882563	96.37553225	71.78315505	82.75002596	89.72894382	96.90537592	118.1709978	114.6796271	110.6656974	94.71388514	70.24399921	95.7108734	77.76508464	85.40866134	82.08536712	82.75002596	83.74701423	109.9186669	111.5056536	71.51358857	110.0010385	72.4478139	83.41468481	81.08837886	92.05524977	65.80043642	86.73797902
	77.18915757	102.0454946	91.48265823	113.6131194	104.5847293	90.80201534	74.80690746	85.01655079	89.78105101	105.7132781	104.3025921	104.3025921	124.4938383	103.3939088	58.59636748	87.3988009	82.29397924	87.05847945	86.03751512	86.37783657	77.18915757	99.78839711	100.3526715	66.77834597	98.96973	80.93269346	88.41976523	72.08433591	79.57140768	68.18903192	80.93269346

AMP3CPR1ERY3FUS3DOX2	AMP	CPR	ERY	FUS	DOX	AMP+CPR	AMP+ERY	AMP+FUS	AMP+DOX	CPR+ERY	CPR+FUS	CPR+DOX	ERY+FUS	ERY+DOX	FUS+DOX	AMP+CPR+ERY	AMP+CPR+FUS	AMP+CPR+DOX	AMP+ERY+FUS	AMP+ERY+DOX	AMP+FUS+DOX	CPR+ERY+FUS	CPR+ERY+DOX	CPR+FUS+DOX	ERY+FUS+DOX	AMP+CPR+ERY+FUS	AMP+CPR+ERY+DOX	AMP+CPR+FUS+DOX	AMP+ERY+FUS+DOX	CPR+ERY+FUS+DOX	AMP+CPR+ERY+FUS+DOX
	75.22844656	89.73601625	99.06634883	103.6182389	113.9560642	73.63925308	75.22844656	87.30631704	98.43067143	92.68968064	105.9811704	115.7282629	116.8653159	116.2296385	86.78235186	81.2673818	72.68573699	81.9030592	79.99602702	97.79499404	66.96464044	104.2089718	111.8884992	79.39819088	92.70957489	74.59276917	79.67818832	66.50774732	72.05005959	77.03525937	80.31386571
	71.78315505	97.22277326	82.75002596	117.5362031	121.6623686	96.37553225	71.78315505	82.75002596	91.72292034	96.90537592	118.1709978	119.7579845	110.6656974	106.6777443	76.90934338	95.7108734	77.76508464	88.06729671	82.08536712	94.0492263	86.4056496	109.9186669	119.1231898	79.44852212	103.6867795	72.4478139	96.37553225	82.41769654	102.0251324	71.83098592	83.41468481
	77.18915757	102.0454946	91.48265823	113.6131194	102.8919062	90.80201534	74.80690746	85.01655079	96.92780134	105.7132781	104.3025921	108.53465	124.4938383	107.1374447	76.93528478	87.3988009	82.29397924	83.99558646	86.03751512	93.86490834	69.7020858	99.78839711	109.6631987	69.88185505	103.0535873	80.93269346	85.69719368	110.5406591	73.44562168	70.72826662	77.18915757

AMP3CPR1ERY3FUS3DOX3	AMP	CPR	ERY	FUS	DOX	AMP+CPR	AMP+ERY	AMP+FUS	AMP+DOX	CPR+ERY	CPR+FUS	CPR+DOX	ERY+FUS	ERY+DOX	FUS+DOX	AMP+CPR+ERY	AMP+CPR+FUS	AMP+CPR+DOX	AMP+ERY+FUS	AMP+ERY+DOX	AMP+FUS+DOX	CPR+ERY+FUS	CPR+ERY+DOX	CPR+FUS+DOX	ERY+FUS+DOX	AMP+CPR+ERY+FUS	AMP+CPR+ERY+DOX	AMP+CPR+FUS+DOX	AMP+ERY+FUS+DOX	CPR+ERY+FUS+DOX	AMP+CPR+ERY+FUS+DOX
	75.22844656	89.73601625	99.06634883	103.6182389	107.753369	73.63925308	75.22844656	87.30631704	94.29876838	92.68968064	105.9811704	111.0023999	116.8653159	104.151768	97.7109101	81.2673818	72.68573699	85.08144617	79.99602702	84.76360747	71.4143822	104.2089718	110.1163005	87.07771829	114.0047676	74.59276917	79.04251093	70.77870481	67.28247914	91.21284844	69.82518872
	71.78315505	97.22277326	82.75002596	117.5362031	118.8057925	96.37553225	71.78315505	82.75002596	91.39059092	96.90537592	118.1709978	115.6318191	110.6656974	120.9679094	108.3316802	95.7108734	77.76508464	102.6897913	82.08536712	86.4056496	77.43275522	109.9186669	118.1709978	101.3489387	116.3152975	72.4478139	105.0160972	90.06127324	84.07934365	91.50962111	79.75906117
	77.18915757	102.0454946	91.48265823	113.6131194	99.22412273	90.80201534	74.80690746	85.01655079	94.54555123	105.7132781	104.3025921	109.0989244	124.4938383	103.0535873	102.3276318	87.3988009	82.29397924	98.62940856	86.03751512	90.80201534	76.84883613	99.78839711	104.5847293	95.27420208	124.8341598	80.93269346	88.41976523	85.69719368	86.37783657	84.55298889	81.61333635

AMP3CPR2ERY1FUS1DOX1	AMP	CPR	ERY	FUS	DOX	AMP+CPR	AMP+ERY	AMP+FUS	AMP+DOX	CPR+ERY	CPR+FUS	CPR+DOX	ERY+FUS	ERY+DOX	FUS+DOX	AMP+CPR+ERY	AMP+CPR+FUS	AMP+CPR+DOX	AMP+ERY+FUS	AMP+ERY+DOX	AMP+FUS+DOX	CPR+ERY+FUS	CPR+ERY+DOX	CPR+FUS+DOX	ERY+FUS+DOX	AMP+CPR+ERY+FUS	AMP+CPR+ERY+DOX	AMP+CPR+FUS+DOX	AMP+ERY+FUS+DOX	CPR+ERY+FUS+DOX	AMP+CPR+ERY+FUS+DOX
	75.22844656	88.33862829	94.46187927	65.33136422	108.9348348	75.54628526	71.0965435	31.0488677	96.84147795	96.71089516	70.39805644	105.9801906	61.19623408	105.0950711	58.27949049	65.37544696	36.13428685	89.53118792	24.55303933	96.84147795	37.26658721	62.02578957	102.3920762	59.12913474	49.71386376	23.10290028	74.91060787	44.57687723	37.26658721	51.35488694	33.13468415
	71.78315505	96.88521438	92.14441579	86.92719698	111.1882563	82.41769654	70.45383737	46.44303666	89.72894382	99.65391271	88.27148625	114.1126706	80.26185281	102.6185281	61.53540964	93.38456745	42.53816596	90.72593208	28.58033025	86.4056496	50.43098972	57.25821957	106.421842	43.66467987	47.6294386	32.56828331	86.07332018	37.8024717	36.14082459	30.43645453	25.34011839
	77.18915757	92.58317025	100.9169459	63.3926997	104.5847293	86.71815801	84.3359079	43.0506627	89.78105101	92.58317025	81.3111546	107.6125245	71.01040381	109.6631987	57.78522306	79.91172913	48.60215626	86.71815801	36.9248767	94.88587267	68.23444957	65.08806262	107.2994129	60.33268102	47.62828425	34.98929849	85.69719368	44.41194847	20.972309	47.49510763	33.18134081

AMP3CPR2ERY1FUS1DOX2	AMP	CPR	ERY	FUS	DOX	AMP+CPR	AMP+ERY	AMP+FUS	AMP+DOX	CPR+ERY	CPR+FUS	CPR+DOX	ERY+FUS	ERY+DOX	FUS+DOX	AMP+CPR+ERY	AMP+CPR+FUS	AMP+CPR+DOX	AMP+ERY+FUS	AMP+ERY+DOX	AMP+FUS+DOX	CPR+ERY+FUS	CPR+ERY+DOX	CPR+FUS+DOX	ERY+FUS+DOX	AMP+CPR+ERY+FUS	AMP+CPR+ERY+DOX	AMP+CPR+FUS+DOX	AMP+ERY+FUS+DOX	CPR+ERY+FUS+DOX	AMP+CPR+ERY+FUS+DOX
	75.22844656	88.33862829	94.46187927	65.33136422	113.9560642	75.54628526	71.0965435	31.0488677	98.43067143	96.71089516	70.39805644	117.3425528	61.19623408	107.1626361	61.19623408	65.37544696	36.13428685	97.15931665	24.55303933	103.1982519	37.9022646	62.02578957	106.8772192	57.03606802	45.57873362	23.10290028	75.86412396	46.16607072	25.50655542	53.14894412	31.54549066
	71.78315505	96.88521438	92.14441579	86.92719698	121.6623686	82.41769654	70.45383737	46.44303666	91.72292034	99.65391271	88.27148625	121.188233	80.26185281	118.1709978	62.17020432	93.38456745	42.53816596	95.7108734	28.58033025	100.3634853	33.1498598	57.25821957	117.8042684	50.12497597	44.13806784	32.56828331	89.06428497	50.76331914	44.44906013	33.57046722	28.82957732
	77.18915757	92.58317025	100.9169459	63.3926997	102.8919062	86.71815801	84.3359079	43.0506627	96.92780134	92.58317025	81.3111546	115.1272016	71.01040381	101.4812202	51.01393052	79.91172913	48.60215626	89.78105101	36.9248767	100.3310158	30.11844782	65.08806262	108.8649706	55.00978474	46.78187269	34.98929849	88.07944379	53.60062747	44.41194847	60.95890411	71.97798546

AMP3CPR2ERY1FUS1DOX3	AMP	CPR	ERY	FUS	DOX	AMP+CPR	AMP+ERY	AMP+FUS	AMP+DOX	CPR+ERY	CPR+FUS	CPR+DOX	ERY+FUS	ERY+DOX	FUS+DOX	AMP+CPR+ERY	AMP+CPR+FUS	AMP+CPR+DOX	AMP+ERY+FUS	AMP+ERY+DOX	AMP+FUS+DOX	CPR+ERY+FUS	CPR+ERY+DOX	CPR+FUS+DOX	ERY+FUS+DOX	AMP+CPR+ERY+FUS	AMP+CPR+ERY+DOX	AMP+CPR+FUS+DOX	AMP+ERY+FUS+DOX	CPR+ERY+FUS+DOX	AMP+CPR+ERY+FUS+DOX
	75.22844656	88.33862829	94.46187927	65.33136422	107.753369	75.54628526	71.0965435	31.0488677	94.29876838	96.71089516	70.39805644	94.91683797	61.19623408	103.6182389	80.39505261	65.37544696	36.13428685	103.1982519	24.55303933	93.02741359	28.36710369	62.02578957	105.6811811	64.41786582	73.89699095	23.10290028	74.27493047	47.43742551	29.63845848	52.45748458	41.71632896
	71.78315505	96.88521438	92.14441579	86.92719698	118.8057925	82.41769654	70.45383737	46.44303666	91.39059092	99.65391271	88.27148625	116.881369	80.26185281	113.0926403	82.16623686	93.38456745	42.53816596	96.70786167	28.58033025	87.73496729	33.48218922	57.25821957	111.0363392	70.79407806	74.866098	32.56828331	90.06127324	44.86447191	42.12275418	59.10401846	28.24800083
	77.18915757	92.58317025	100.9169459	63.3926997	99.22412273	86.71815801	84.3359079	43.0506627	94.54555123	92.58317025	81.3111546	122.6418787	71.01040381	98.65984835	74.74872157	79.91172913	48.60215626	93.86490834	36.9248767	86.71815801	41.34905548	65.08806262	110.1174168	86.0665362	71.64521248	34.98929849	84.67622934	43.0506627	42.02969836	56.57534247	42.71034125

AMP3CPR2ERY1FUS2DOX1	AMP	CPR	ERY	FUS	DOX	AMP+CPR	AMP+ERY	AMP+FUS	AMP+DOX	CPR+ERY	CPR+FUS	CPR+DOX	ERY+FUS	ERY+DOX	FUS+DOX	AMP+CPR+ERY	AMP+CPR+FUS	AMP+CPR+DOX	AMP+ERY+FUS	AMP+ERY+DOX	AMP+FUS+DOX	CPR+ERY+FUS	CPR+ERY+DOX	CPR+FUS+DOX	ERY+FUS+DOX	AMP+CPR+ERY+FUS	AMP+CPR+ERY+DOX	AMP+CPR+FUS+DOX	AMP+ERY+FUS+DOX	CPR+ERY+FUS+DOX	AMP+CPR+ERY+FUS+DOX
	75.22844656	88.33862829	94.46187927	92.3943142	108.9348348	75.54628526	71.0965435	52.34406039	96.84147795	96.71089516	82.35843768	105.9801906	87.66845117	105.0950711	74.19235739	65.37544696	53.29757648	89.53118792	37.72348033	96.84147795	53.15852205	68.60399925	102.3920762	69.80003738	65.33136422	41.219706	74.91060787	49.02661899	48.0731029	48.86937021	48.70878029
	71.78315505	96.88521438	92.14441579	105.1577068	111.1882563	82.41769654	70.45383737	55.4990134	89.72894382	99.65391271	98.73101327	114.1126706	89.9226344	102.6185281	87.24459433	93.38456745	55.4990134	90.72593208	41.54117769	86.4056496	45.11371897	62.12266872	106.421842	73.56277639	64.39198572	44.53214249	86.07332018	49.76633088	52.75729567	54.1818881	47.77235435
	77.18915757	92.58317025	100.9169459	101.1990831	104.5847293	86.71815801	84.3359079	57.79083525	89.78105101	92.58317025	91.33072407	107.6125245	91.88855581	109.6631987	67.13101746	79.91172913	60.85372825	86.71815801	40.09412015	94.88587267	54.6215918	62.83757339	107.2994129	77.61252446	53.024158	50.30376348	85.69719368	58.70544913	52.57966313	57.57338552	95.46016511

AMP3CPR2ERY1FUS2DOX2	AMP	CPR	ERY	FUS	DOX	AMP+CPR	AMP+ERY	AMP+FUS	AMP+DOX	CPR+ERY	CPR+FUS	CPR+DOX	ERY+FUS	ERY+DOX	FUS+DOX	AMP+CPR+ERY	AMP+CPR+FUS	AMP+CPR+DOX	AMP+ERY+FUS	AMP+ERY+DOX	AMP+FUS+DOX	CPR+ERY+FUS	CPR+ERY+DOX	CPR+FUS+DOX	ERY+FUS+DOX	AMP+CPR+ERY+FUS	AMP+CPR+ERY+DOX	AMP+CPR+FUS+DOX	AMP+ERY+FUS+DOX	CPR+ERY+FUS+DOX	AMP+CPR+ERY+FUS+DOX
	75.22844656	88.33862829	94.46187927	92.3943142	113.9560642	75.54628526	71.0965435	52.34406039	98.43067143	96.71089516	82.35843768	117.3425528	87.66845117	107.1626361	77.14602178	65.37544696	53.29757648	97.15931665	37.72348033	103.1982519	43.30552245	68.60399925	106.8772192	72.49112315	67.3989293	41.219706	75.86412396	52.20500596	54.11203814	58.4376752	59.19745729
	71.78315505	96.88521438	92.14441579	105.1577068	121.6623686	82.41769654	70.45383737	55.4990134	91.72292034	99.65391271	98.73101327	121.188233	89.9226344	118.1709978	83.11842888	93.38456745	55.4990134	95.7108734	41.54117769	100.3634853	46.11070724	62.12266872	117.8042684	74.17804268	81.84883952	44.53214249	89.06428497	46.11070724	47.44002492	52.64372236	41.79042476
	77.18915757	92.58317025	100.9169459	101.1990831	102.8919062	86.71815801	84.3359079	57.79083525	96.92780134	92.58317025	91.33072407	115.1272016	91.88855581	101.4812202	64.30964557	79.91172913	60.85372825	89.78105101	40.09412015	100.3310158	51.89902025	62.83757339	108.8649706	82.30919765	61.48827367	50.30376348	88.07944379	50.87805591	44.07162703	63.20939335	54.96191324

AMP3CPR2ERY1FUS2DOX3	AMP	CPR	ERY	FUS	DOX	AMP+CPR	AMP+ERY	AMP+FUS	AMP+DOX	CPR+ERY	CPR+FUS	CPR+DOX	ERY+FUS	ERY+DOX	FUS+DOX	AMP+CPR+ERY	AMP+CPR+FUS	AMP+CPR+DOX	AMP+ERY+FUS	AMP+ERY+DOX	AMP+FUS+DOX	CPR+ERY+FUS	CPR+ERY+DOX	CPR+FUS+DOX	ERY+FUS+DOX	AMP+CPR+ERY+FUS	AMP+CPR+ERY+DOX	AMP+CPR+FUS+DOX	AMP+ERY+FUS+DOX	CPR+ERY+FUS+DOX	AMP+CPR+ERY+FUS+DOX
	75.22844656	88.33862829	94.46187927	92.3943142	107.753369	75.54628526	71.0965435	52.34406039	94.29876838	96.71089516	82.35843768	94.91683797	87.66845117	103.6182389	102.7321396	65.37544696	53.29757648	103.1982519	37.72348033	93.02741359	32.49900675	68.60399925	105.6811811	79.6673519	88.55455049	41.219706	74.27493047	69.50735002	35.35955503	68.00598019	42.98768375
	71.78315505	96.88521438	92.14441579	105.1577068	118.8057925	82.41769654	70.45383737	55.4990134	91.39059092	99.65391271	98.73101327	116.881369	89.9226344	113.0926403	98.80975997	93.38456745	55.4990134	96.70786167	41.54117769	87.73496729	30.24197736	62.12266872	111.0363392	80.58065757	83.25729022	44.53214249	90.06127324	85.40866134	22.84764773	59.71928475	48.52009554
	77.18915757	92.58317025	100.9169459	101.1990831	99.22412273	86.71815801	84.3359079	57.79083525	94.54555123	92.58317025	91.33072407	122.6418787	91.88855581	98.65984835	93.58137895	79.91172913	60.85372825	93.86490834	40.09412015	86.71815801	34.54262659	62.83757339	110.1174168	81.3111546	80.32093105	50.30376348	84.67622934	75.82787179	25.3539476	77.92563601	63.12962791

AMP3CPR2ERY1FUS3DOX1	AMP	CPR	ERY	FUS	DOX	AMP+CPR	AMP+ERY	AMP+FUS	AMP+DOX	CPR+ERY	CPR+FUS	CPR+DOX	ERY+FUS	ERY+DOX	FUS+DOX	AMP+CPR+ERY	AMP+CPR+FUS	AMP+CPR+DOX	AMP+ERY+FUS	AMP+ERY+DOX	AMP+FUS+DOX	CPR+ERY+FUS	CPR+ERY+DOX	CPR+FUS+DOX	ERY+FUS+DOX	AMP+CPR+ERY+FUS	AMP+CPR+ERY+DOX	AMP+CPR+FUS+DOX	AMP+ERY+FUS+DOX	CPR+ERY+FUS+DOX	AMP+CPR+ERY+FUS+DOX
	75.22844656	88.33862829	94.46187927	103.6182389	108.9348348	75.54628526	71.0965435	87.30631704	96.84147795	96.71089516	105.9801906	105.9801906	96.52944434	105.0950711	79.69355732	65.37544696	85.08144617	89.53118792	83.49225268	96.84147795	70.14302741	94.61782844	102.3920762	90.43169501	72.01402991	65.69328566	74.91060787	70.14302741	60.60786651	59.93272286	61.8792213
	71.78315505	96.88521438	92.14441579	117.5362031	111.1882563	82.41769654	70.45383737	82.75002596	89.72894382	99.65391271	113.1897712	114.1126706	110.870859	102.6185281	70.24399921	93.38456745	78.42974348	90.72593208	76.10343753	86.4056496	83.74701423	103.9607768	106.421842	84.88752163	65.66157508	80.09139059	86.07332018	79.09440233	78.09741406	67.66006537	74.44179042
	77.18915757	92.58317025	100.9169459	113.6131194	104.5847293	86.71815801	84.3359079	85.01655079	89.78105101	92.58317025	111.0567515	107.6125245	106.5596896	109.6631987	58.59636748	79.91172913	79.57140768	86.71815801	83.99558646	94.88587267	77.18915757	99.78473581	107.2994129	92.27005871	56.05713278	97.60844423	85.69719368	72.42465735	58.47147814	68.16046967	68.00047858

AMP3CPR2ERY1FUS3DOX2	AMP	CPR	ERY	FUS	DOX	AMP+CPR	AMP+ERY	AMP+FUS	AMP+DOX	CPR+ERY	CPR+FUS	CPR+DOX	ERY+FUS	ERY+DOX	FUS+DOX	AMP+CPR+ERY	AMP+CPR+FUS	AMP+CPR+DOX	AMP+ERY+FUS	AMP+ERY+DOX	AMP+FUS+DOX	CPR+ERY+FUS	CPR+ERY+DOX	CPR+FUS+DOX	ERY+FUS+DOX	AMP+CPR+ERY+FUS	AMP+CPR+ERY+DOX	AMP+CPR+FUS+DOX	AMP+ERY+FUS+DOX	CPR+ERY+FUS+DOX	AMP+CPR+ERY+FUS+DOX
	75.22844656	88.33862829	94.46187927	103.6182389	113.9560642	75.54628526	71.0965435	87.30631704	98.43067143	96.71089516	105.9801906	117.3425528	96.52944434	107.1626361	86.78235186	65.37544696	85.08144617	97.15931665	83.49225268	103.1982519	66.96464044	94.61782844	106.8772192	90.73070454	68.1742662	65.69328566	75.86412396	74.91060787	61.5613826	60.23173239	63.15057608
	71.78315505	96.88521438	92.14441579	117.5362031	121.6623686	82.41769654	70.45383737	82.75002596	91.72292034	99.65391271	113.1897712	121.188233	110.870859	118.1709978	76.90934338	93.38456745	78.42974348	95.7108734	76.10343753	100.3634853	86.4056496	103.9607768	117.8042684	89.1943857	66.29636977	80.09139059	89.06428497	100.0311559	71.11849621	62.43030187	90.06127324
	77.18915757	92.58317025	100.9169459	113.6131194	102.8919062	86.71815801	84.3359079	85.01655079	96.92780134	92.58317025	111.0567515	115.1272016	106.5596896	101.4812202	76.93528478	79.91172913	79.57140768	89.78105101	83.99558646	100.3310158	69.7020858	99.78473581	108.8649706	82.87671233	58.1026274	97.60844423	88.07944379	73.44562168	57.79083525	67.84735812	60.85372825

AMP3CPR2ERY1FUS3DOX3	AMP	CPR	ERY	FUS	DOX	AMP+CPR	AMP+ERY	AMP+FUS	AMP+DOX	CPR+ERY	CPR+FUS	CPR+DOX	ERY+FUS	ERY+DOX	FUS+DOX	AMP+CPR+ERY	AMP+CPR+FUS	AMP+CPR+DOX	AMP+ERY+FUS	AMP+ERY+DOX	AMP+FUS+DOX	CPR+ERY+FUS	CPR+ERY+DOX	CPR+FUS+DOX	ERY+FUS+DOX	AMP+CPR+ERY+FUS	AMP+CPR+ERY+DOX	AMP+CPR+FUS+DOX	AMP+ERY+FUS+DOX	CPR+ERY+FUS+DOX	AMP+CPR+ERY+FUS+DOX
	75.22844656	88.33862829	94.46187927	103.6182389	107.753369	75.54628526	71.0965435	87.30631704	94.29876838	96.71089516	105.9801906	94.91683797	96.52944434	103.6182389	97.7109101	65.37544696	85.08144617	103.1982519	83.49225268	93.02741359	71.4143822	94.61782844	105.6811811	107.1762287	90.03138268	65.69328566	74.27493047	72.36789829	61.8792213	91.02971407	65.69328566
	71.78315505	96.88521438	92.14441579	117.5362031	118.8057925	82.41769654	70.45383737	82.75002596	91.39059092	99.65391271	113.1897712	116.881369	110.870859	113.0926403	108.3316802	93.38456745	78.42974348	96.70786167	76.10343753	87.73496729	77.43275522	103.9607768	111.0363392	107.9600077	94.36619718	80.09139059	90.06127324	86.73797902	75.77110811	99.03864641	76.43576695
	77.18915757	92.58317025	100.9169459	113.6131194	99.22412273	86.71815801	84.3359079	85.01655079	94.54555123	92.58317025	111.0567515	122.6418787	106.5596896	98.65984835	102.3276318	79.91172913	79.57140768	93.86490834	83.99558646	86.71815801	76.84883613	99.78473581	110.1174168	102.6027397	88.22077235	97.60844423	84.67622934	78.2101219	76.50851468	89.45205479	76.50851468

AMP3CPR2ERY2FUS1DOX1	AMP	CPR	ERY	FUS	DOX	AMP+CPR	AMP+ERY	AMP+FUS	AMP+DOX	CPR+ERY	CPR+FUS	CPR+DOX	ERY+FUS	ERY+DOX	FUS+DOX	AMP+CPR+ERY	AMP+CPR+FUS	AMP+CPR+DOX	AMP+ERY+FUS	AMP+ERY+DOX	AMP+FUS+DOX	CPR+ERY+FUS	CPR+ERY+DOX	CPR+FUS+DOX	ERY+FUS+DOX	AMP+CPR+ERY+FUS	AMP+CPR+ERY+DOX	AMP+CPR+FUS+DOX	AMP+ERY+FUS+DOX	CPR+ERY+FUS+DOX	AMP+CPR+ERY+FUS+DOX
	75.22844656	88.33862829	92.2257522	65.33136422	108.9348348	75.54628526	74.59276917	31.0488677	96.84147795	97.30891422	70.39805644	105.9801906	70.39805644	101.7940572	58.27949049	64.73976957	36.13428685	89.53118792	25.50655542	102.2447358	37.26658721	71.29508503	111.6613717	59.12913474	56.43804896	35.49860946	84.44576877	44.57687723	66.82558602	54.34498225	45.53039333
	71.78315505	96.88521438	104.2684099	86.92719698	111.1882563	82.41769654	68.45986084	46.44303666	89.72894382	98.42338012	88.27148625	114.1126706	86.73332052	111.9592386	61.53540964	93.05223803	42.53816596	90.72593208	54.41894278	98.36950878	50.43098972	64.02614882	115.3432032	43.66467987	36.89675062	36.22390695	89.39661439	37.8024717	53.08962509	37.26206499	40.46110707
	77.18915757	92.58317025	95.40117417	63.3926997	104.5847293	86.71815801	78.2101219	43.0506627	89.78105101	91.33072407	81.3111546	107.6125245	81.93737769	103.5420744	57.78522306	80.25205057	48.60215626	86.71815801	42.37001981	97.26812278	68.23444957	68.21917808	113.2485323	60.33268102	51.25244618	36.24423381	92.84394401	44.41194847	65.51187802	51.87866928	43.0506627

AMP3CPR2ERY2FUS1DOX2	AMP	CPR	ERY	FUS	DOX	AMP+CPR	AMP+ERY	AMP+FUS	AMP+DOX	CPR+ERY	CPR+FUS	CPR+DOX	ERY+FUS	ERY+DOX	FUS+DOX	AMP+CPR+ERY	AMP+CPR+FUS	AMP+CPR+DOX	AMP+ERY+FUS	AMP+ERY+DOX	AMP+FUS+DOX	CPR+ERY+FUS	CPR+ERY+DOX	CPR+FUS+DOX	ERY+FUS+DOX	AMP+CPR+ERY+FUS	AMP+CPR+ERY+DOX	AMP+CPR+FUS+DOX	AMP+ERY+FUS+DOX	CPR+ERY+FUS+DOX	AMP+CPR+ERY+FUS+DOX
	75.22844656	88.33862829	92.2257522	65.33136422	113.9560642	75.54628526	74.59276917	31.0488677	98.43067143	97.30891422	70.39805644	117.3425528	70.39805644	107.1762287	61.19623408	64.73976957	36.13428685	97.15931665	25.50655542	95.25228447	37.9022646	71.29508503	113.4554289	57.03606802	49.85983928	35.49860946	87.94199444	46.16607072	43.62336114	48.66380116	31.54549066
	71.78315505	96.88521438	104.2684099	86.92719698	121.6623686	82.41769654	68.45986084	46.44303666	91.72292034	98.42338012	88.27148625	121.188233	86.73332052	117.4966353	62.17020432	93.05223803	42.53816596	95.7108734	54.41894278	94.38155572	33.1498598	64.02614882	113.8050375	50.12497597	40.03076331	36.22390695	92.71990861	50.76331914	53.75428393	37.81965007	34.81150691
	77.18915757	92.58317025	95.40117417	63.3926997	102.8919062	86.71815801	78.2101219	43.0506627	96.92780134	91.33072407	81.3111546	115.1272016	81.93737769	111.369863	51.01393052	80.25205057	48.60215626	89.78105101	42.37001981	106.7971232	30.11844782	68.21917808	114.81409	55.00978474	48.43444227	36.24423381	89.78105101	53.60062747	64.15059224	58.45401174	23.31201893

AMP3CPR2ERY2FUS1DOX3	AMP	CPR	ERY	FUS	DOX	AMP+CPR	AMP+ERY	AMP+FUS	AMP+DOX	CPR+ERY	CPR+FUS	CPR+DOX	ERY+FUS	ERY+DOX	FUS+DOX	AMP+CPR+ERY	AMP+CPR+FUS	AMP+CPR+DOX	AMP+ERY+FUS	AMP+ERY+DOX	AMP+FUS+DOX	CPR+ERY+FUS	CPR+ERY+DOX	CPR+FUS+DOX	ERY+FUS+DOX	AMP+CPR+ERY+FUS	AMP+CPR+ERY+DOX	AMP+CPR+FUS+DOX	AMP+ERY+FUS+DOX	CPR+ERY+FUS+DOX	AMP+CPR+ERY+FUS+DOX
	75.22844656	88.33862829	92.2257522	65.33136422	107.753369	75.54628526	74.59276917	31.0488677	94.29876838	97.30891422	70.39805644	94.91683797	70.39805644	109.8673145	80.39505261	64.73976957	36.13428685	103.1982519	25.50655542	96.20580056	28.36710369	71.29508503	112.5584003	64.41786582	59.63371332	35.49860946	87.62415574	47.43742551	46.16607072	66.80994207	42.98768375
	71.78315505	96.88521438	104.2684099	86.92719698	118.8057925	82.41769654	68.45986084	46.44303666	91.39059092	98.42338012	88.27148625	116.881369	86.73332052	114.4203038	82.16623686	93.05223803	42.53816596	96.70786167	54.41894278	93.38456745	33.48218922	64.02614882	113.1897712	70.79407806	63.71851567	36.22390695	90.06127324	44.86447191	45.11371897	63.41088252	43.78440129
	77.18915757	92.58317025	95.40117417	63.3926997	99.22412273	86.71815801	78.2101219	43.0506627	94.54555123	91.33072407	81.3111546	122.6418787	81.93737769	111.0567515	74.74872157	80.25205057	48.60215626	93.86490834	42.37001981	98.28908711	41.34905548	68.21917808	121.0763209	86.0665362	80.43052838	36.24423381	87.73912234	43.0506627	50.19741303	71.66340509	50.19741303

AMP3CPR2ERY2FUS2DOX1	AMP	CPR	ERY	FUS	DOX	AMP+CPR	AMP+ERY	AMP+FUS	AMP+DOX	CPR+ERY	CPR+FUS	CPR+DOX	ERY+FUS	ERY+DOX	FUS+DOX	AMP+CPR+ERY	AMP+CPR+FUS	AMP+CPR+DOX	AMP+ERY+FUS	AMP+ERY+DOX	AMP+FUS+DOX	CPR+ERY+FUS	CPR+ERY+DOX	CPR+FUS+DOX	ERY+FUS+DOX	AMP+CPR+ERY+FUS	AMP+CPR+ERY+DOX	AMP+CPR+FUS+DOX	AMP+ERY+FUS+DOX	CPR+ERY+FUS+DOX	AMP+CPR+ERY+FUS+DOX
	75.22844656	88.33862829	92.2257522	92.3943142	108.9348348	75.54628526	74.59276917	52.34406039	96.84147795	97.30891422	82.35843768	105.9801906	90.13268548	101.7940572	74.19235739	64.73976957	53.29757648	89.53118792	48.52999603	102.2447358	53.15852205	72.49112315	111.6613717	69.80003738	62.3247991	39.94835121	84.44576877	49.02661899	49.34445769	66.51093254	51.56932857
	71.78315505	96.88521438	104.2684099	105.1577068	111.1882563	82.41769654	68.45986084	55.4990134	89.72894382	98.42338012	98.73101327	114.1126706	101.8073447	111.9592386	87.24459433	93.05223803	55.4990134	90.72593208	47.19077786	98.36950878	45.11371897	79.96539127	115.3432032	73.56277639	64.64141511	47.19077786	89.39661439	49.76633088	49.43400145	67.71774659	46.77536608
	77.18915757	92.58317025	95.40117417	101.1990831	104.5847293	86.71815801	78.2101219	57.79083525	89.78105101	91.33072407	91.33072407	107.6125245	98.84540117	103.5420744	67.13101746	80.25205057	60.85372825	86.71815801	48.60215626	97.26812278	54.6215918	84.12915851	113.2485323	77.61252446	64.46183953	48.26183481	92.84394401	58.70544913	50.53773447	76.67318982	51.89902025

AMP3CPR2ERY2FUS2DOX2	AMP	CPR	ERY	FUS	DOX	AMP+CPR	AMP+ERY	AMP+FUS	AMP+DOX	CPR+ERY	CPR+FUS	CPR+DOX	ERY+FUS	ERY+DOX	FUS+DOX	AMP+CPR+ERY	AMP+CPR+FUS	AMP+CPR+DOX	AMP+ERY+FUS	AMP+ERY+DOX	AMP+FUS+DOX	CPR+ERY+FUS	CPR+ERY+DOX	CPR+FUS+DOX	ERY+FUS+DOX	AMP+CPR+ERY+FUS	AMP+CPR+ERY+DOX	AMP+CPR+FUS+DOX	AMP+ERY+FUS+DOX	CPR+ERY+FUS+DOX	AMP+CPR+ERY+FUS+DOX
	75.22844656	88.33862829	92.2257522	92.3943142	113.9560642	75.54628526	74.59276917	52.34406039	98.43067143	97.30891422	82.35843768	117.3425528	90.13268548	107.1762287	77.14602178	64.73976957	53.29757648	97.15931665	48.52999603	95.25228447	43.30552245	72.49112315	113.4554289	72.49112315	71.29508503	39.94835121	87.94199444	52.20500596	53.47636075	67.70697066	57.9261025
	71.78315505	96.88521438	104.2684099	105.1577068	121.6623686	82.41769654	68.45986084	55.4990134	91.72292034	98.42338012	98.73101327	121.188233	101.8073447	117.4966353	83.11842888	93.05223803	55.4990134	95.7108734	47.19077786	94.38155572	46.11070724	79.96539127	113.8050375	74.17804268	71.7169775	47.19077786	92.71990861	46.11070724	50.76331914	59.41165161	42.4550836
	77.18915757	92.58317025	95.40117417	101.1990831	102.8919062	86.71815801	78.2101219	57.79083525	96.92780134	91.33072407	91.33072407	115.1272016	98.84540117	111.369863	64.30964557	80.25205057	60.85372825	89.78105101	48.60215626	106.7971232	51.89902025	84.12915851	114.81409	82.30919765	66.9667319	48.26183481	89.78105101	50.87805591	50.87805591	72.28962818	51.5586988

AMP3CPR2ERY2FUS2DOX3	AMP	CPR	ERY	FUS	DOX	AMP+CPR	AMP+ERY	AMP+FUS	AMP+DOX	CPR+ERY	CPR+FUS	CPR+DOX	ERY+FUS	ERY+DOX	FUS+DOX	AMP+CPR+ERY	AMP+CPR+FUS	AMP+CPR+DOX	AMP+ERY+FUS	AMP+ERY+DOX	AMP+FUS+DOX	CPR+ERY+FUS	CPR+ERY+DOX	CPR+FUS+DOX	ERY+FUS+DOX	AMP+CPR+ERY+FUS	AMP+CPR+ERY+DOX	AMP+CPR+FUS+DOX	AMP+ERY+FUS+DOX	CPR+ERY+FUS+DOX	AMP+CPR+ERY+FUS+DOX
	75.22844656	88.33862829	92.2257522	92.3943142	107.753369	75.54628526	74.59276917	52.34406039	94.29876838	97.30891422	82.35843768	94.91683797	90.13268548	109.8673145	102.7321396	64.73976957	53.29757648	103.1982519	48.52999603	96.20580056	32.49900675	72.49112315	112.5584003	79.6673519	106.2792001	39.94835121	87.62415574	69.50735002	37.5844259	73.98617081	50.61581248
	71.78315505	96.88521438	104.2684099	105.1577068	118.8057925	82.41769654	68.45986084	55.4990134	91.39059092	98.42338012	98.73101327	116.881369	101.8073447	114.4203038	98.80975997	93.05223803	55.4990134	96.70786167	47.19077786	93.38456745	30.24197736	79.96539127	113.1897712	80.58065757	107.0371082	47.19077786	90.06127324	85.40866134	35.47616575	69.56354547	70.45383737
	77.18915757	92.58317025	95.40117417	101.1990831	99.22412273	86.71815801	78.2101219	57.79083525	94.54555123	91.33072407	91.33072407	122.6418787	98.84540117	111.0567515	93.58137895	80.25205057	60.85372825	93.86490834	48.60215626	98.28908711	34.54262659	84.12915851	121.0763209	81.3111546	105.1076321	48.26183481	87.73912234	75.82787179	26.71523337	77.92563601	44.75226992

AMP3CPR2ERY2FUS3DOX1	AMP	CPR	ERY	FUS	DOX	AMP+CPR	AMP+ERY	AMP+FUS	AMP+DOX	CPR+ERY	CPR+FUS	CPR+DOX	ERY+FUS	ERY+DOX	FUS+DOX	AMP+CPR+ERY	AMP+CPR+FUS	AMP+CPR+DOX	AMP+ERY+FUS	AMP+ERY+DOX	AMP+FUS+DOX	CPR+ERY+FUS	CPR+ERY+DOX	CPR+FUS+DOX	ERY+FUS+DOX	AMP+CPR+ERY+FUS	AMP+CPR+ERY+DOX	AMP+CPR+FUS+DOX	AMP+ERY+FUS+DOX	CPR+ERY+FUS+DOX	AMP+CPR+ERY+FUS+DOX
	75.22844656	88.33862829	92.2257522	103.6182389	108.9348348	75.54628526	74.59276917	87.30631704	96.84147795	97.30891422	105.9801906	105.9801906	102.0930667	101.7940572	79.69355732	64.73976957	85.08144617	89.53118792	81.9030592	102.2447358	70.14302741	102.0930667	111.6613717	90.43169501	79.06933283	70.46086611	84.44576877	70.14302741	54.56893127	71.59409456	62.19705999
	71.78315505	96.88521438	104.2684099	117.5362031	111.1882563	82.41769654	68.45986084	82.75002596	89.72894382	98.42338012	113.1897712	114.1126706	117.4966353	111.9592386	70.24399921	93.05223803	78.42974348	90.72593208	80.09139059	98.36950878	83.74701423	115.0355701	115.3432032	84.88752163	74.73562776	76.10343753	89.39661439	79.09440233	83.08235538	72.88982888	81.08837886
	77.18915757	92.58317025	95.40117417	113.6131194	104.5847293	86.71815801	78.2101219	85.01655079	89.78105101	91.33072407	111.0567515	107.6125245	112.6223092	103.5420744	58.59636748	80.25205057	79.57140768	86.71815801	81.2730149	97.26812278	77.18915757	106.0469667	113.2485323	92.27005871	88.51272016	101.3519801	92.84394401	72.42465735	64.93758558	81.3111546	67.66015713

AMP3CPR2ERY2FUS3DOX2	AMP	CPR	ERY	FUS	DOX	AMP+CPR	AMP+ERY	AMP+FUS	AMP+DOX	CPR+ERY	CPR+FUS	CPR+DOX	ERY+FUS	ERY+DOX	FUS+DOX	AMP+CPR+ERY	AMP+CPR+FUS	AMP+CPR+DOX	AMP+ERY+FUS	AMP+ERY+DOX	AMP+FUS+DOX	CPR+ERY+FUS	CPR+ERY+DOX	CPR+FUS+DOX	ERY+FUS+DOX	AMP+CPR+ERY+FUS	AMP+CPR+ERY+DOX	AMP+CPR+FUS+DOX	AMP+ERY+FUS+DOX	CPR+ERY+FUS+DOX	AMP+CPR+ERY+FUS+DOX
	75.22844656	88.33862829	92.2257522	103.6182389	113.9560642	75.54628526	74.59276917	87.30631704	98.43067143	97.30891422	105.9801906	117.3425528	102.0930667	107.1762287	86.78235186	64.73976957	85.08144617	97.15931665	81.9030592	95.25228447	66.96464044	102.0930667	113.4554289	90.73070454	86.54457111	70.46086611	87.94199444	74.91060787	59.33651172	80.56438049	72.68573699
	71.78315505	96.88521438	104.2684099	117.5362031	121.6623686	82.41769654	68.45986084	82.75002596	91.72292034	98.42338012	113.1897712	121.188233	117.4966353	117.4966353	76.90934338	93.05223803	78.42974348	95.7108734	80.09139059	94.38155572	86.4056496	115.0355701	113.8050375	89.1943857	78.11959239	76.10343753	92.71990861	100.0311559	78.09741406	77.50432609	77.43275522
	77.18915757	92.58317025	95.40117417	113.6131194	102.8919062	86.71815801	78.2101219	85.01655079	96.92780134	91.33072407	111.0567515	115.1272016	112.6223092	111.369863	76.93528478	80.25205057	79.57140768	89.78105101	81.2730149	106.7971232	69.7020858	106.0469667	114.81409	82.87671233	78.18003914	101.3519801	89.78105101	73.44562168	69.36176435	83.18982387	63.91662125

AMP3CPR2ERY2FUS3DOX3	AMP	CPR	ERY	FUS	DOX	AMP+CPR	AMP+ERY	AMP+FUS	AMP+DOX	CPR+ERY	CPR+FUS	CPR+DOX	ERY+FUS	ERY+DOX	FUS+DOX	AMP+CPR+ERY	AMP+CPR+FUS	AMP+CPR+DOX	AMP+ERY+FUS	AMP+ERY+DOX	AMP+FUS+DOX	CPR+ERY+FUS	CPR+ERY+DOX	CPR+FUS+DOX	ERY+FUS+DOX	AMP+CPR+ERY+FUS	AMP+CPR+ERY+DOX	AMP+CPR+FUS+DOX	AMP+ERY+FUS+DOX	CPR+ERY+FUS+DOX	AMP+CPR+ERY+FUS+DOX
	75.22844656	88.33862829	92.2257522	103.6182389	107.753369	75.54628526	74.59276917	87.30631704	94.29876838	97.30891422	105.9801906	94.91683797	102.0930667	109.8673145	97.7109101	64.73976957	85.08144617	103.1982519	81.9030592	96.20580056	71.4143822	102.0930667	112.5584003	107.1762287	109.8673145	70.46086611	87.62415574	72.36789829	66.64680175	97.90693328	67.60031784
	71.78315505	96.88521438	104.2684099	117.5362031	118.8057925	82.41769654	68.45986084	82.75002596	91.39059092	98.42338012	113.1897712	116.881369	117.4966353	114.4203038	108.3316802	93.05223803	78.42974348	96.70786167	80.09139059	93.38456745	77.43275522	115.0355701	113.1897712	107.9600077	102.1149779	76.10343753	90.06127324	86.73797902	79.42673175	103.0378773	84.07934365
	77.18915757	92.58317025	95.40117417	113.6131194	99.22412273	86.71815801	78.2101219	85.01655079	94.54555123	91.33072407	111.0567515	122.6418787	112.6223092	111.0567515	102.3276318	80.25205057	79.57140768	93.86490834	81.2730149	98.28908711	76.84883613	106.0469667	121.0763209	102.6027397	108.2387476	101.3519801	87.73912234	78.2101219	77.52947902	107.925636	84.3359079

AMP3CPR2ERY3FUS1DOX1	AMP	CPR	ERY	FUS	DOX	AMP+CPR	AMP+ERY	AMP+FUS	AMP+DOX	CPR+ERY	CPR+FUS	CPR+DOX	ERY+FUS	ERY+DOX	FUS+DOX	AMP+CPR+ERY	AMP+CPR+FUS	AMP+CPR+DOX	AMP+ERY+FUS	AMP+ERY+DOX	AMP+FUS+DOX	CPR+ERY+FUS	CPR+ERY+DOX	CPR+FUS+DOX	ERY+FUS+DOX	AMP+CPR+ERY+FUS	AMP+CPR+ERY+DOX	AMP+CPR+FUS+DOX	AMP+ERY+FUS+DOX	CPR+ERY+FUS+DOX	AMP+CPR+ERY+FUS+DOX
	75.22844656	88.33862829	99.06634883	65.33136422	108.9348348	75.54628526	75.22844656	31.0488677	96.84147795	98.80396188	70.39805644	105.9801906	105.4231228	106.0588002	58.27949049	68.55383393	36.13428685	89.53118792	35.04171633	91.7560588	37.26658721	71.89310409	110.4653336	59.12913474	59.51529599	42.17322209	87.94199444	44.57687723	46.16607072	58.83012521	43.30552245
	71.78315505	96.88521438	82.75002596	86.92719698	111.1882563	82.41769654	71.78315505	46.44303666	89.72894382	100.269179	88.27148625	114.1126706	94.38155572	94.71388514	61.53540964	86.73797902	42.53816596	90.72593208	37.8024717	82.75002596	50.43098972	67.10248029	113.1897712	43.66467987	70.70308443	49.84941323	87.73496729	37.8024717	51.09564856	41.81888098	43.45207187
	77.18915757	92.58317025	91.48265823	63.3926997	104.5847293	86.71815801	74.80690746	43.0506627	89.78105101	96.9667319	81.3111546	107.6125245	95.906837	103.3939088	57.78522306	79.57140768	48.60215626	86.71815801	51.5586988	86.37783657	68.23444957	71.037182	108.5518591	60.33268102	74.70055701	53.70697792	82.63430068	44.41194847	50.87805591	55.00978474	59.72641346

AMP3CPR2ERY3FUS1DOX2	AMP	CPR	ERY	FUS	DOX	AMP+CPR	AMP+ERY	AMP+FUS	AMP+DOX	CPR+ERY	CPR+FUS	CPR+DOX	ERY+FUS	ERY+DOX	FUS+DOX	AMP+CPR+ERY	AMP+CPR+FUS	AMP+CPR+DOX	AMP+ERY+FUS	AMP+ERY+DOX	AMP+FUS+DOX	CPR+ERY+FUS	CPR+ERY+DOX	CPR+FUS+DOX	ERY+FUS+DOX	AMP+CPR+ERY+FUS	AMP+CPR+ERY+DOX	AMP+CPR+FUS+DOX	AMP+ERY+FUS+DOX	CPR+ERY+FUS+DOX	AMP+CPR+ERY+FUS+DOX
	75.22844656	88.33862829	99.06634883	65.33136422	113.9560642	75.54628526	75.22844656	31.0488677	98.43067143	98.80396188	70.39805644	117.3425528	105.4231228	116.2296385	61.19623408	68.55383393	36.13428685	97.15931665	35.04171633	97.79499404	37.9022646	71.89310409	117.0435433	57.03606802	65.55423123	42.17322209	97.79499404	46.16607072	52.20500596	54.34498225	49.66229638
	71.78315505	96.88521438	82.75002596	86.92719698	121.6623686	82.41769654	71.78315505	46.44303666	91.72292034	100.269179	88.27148625	121.188233	94.38155572	106.6777443	62.17020432	86.73797902	42.53816596	95.7108734	37.8024717	94.0492263	33.1498598	67.10248029	122.1111325	50.12497597	81.0052965	49.84941323	89.06428497	50.76331914	45.11371897	41.20361469	81.7530377
	77.18915757	92.58317025	91.48265823	63.3926997	102.8919062	86.71815801	74.80690746	43.0506627	96.92780134	96.9667319	81.3111546	115.1272016	95.906837	107.1374447	51.01393052	79.57140768	48.60215626	89.78105101	51.5586988	93.86490834	30.11844782	71.037182	112.6223092	55.00978474	71.29734257	53.70697792	99.99069434	53.60062747	54.6215918	58.76712329	69.59573535

AMP3CPR2ERY3FUS1DOX3	AMP	CPR	ERY	FUS	DOX	AMP+CPR	AMP+ERY	AMP+FUS	AMP+DOX	CPR+ERY	CPR+FUS	CPR+DOX	ERY+FUS	ERY+DOX	FUS+DOX	AMP+CPR+ERY	AMP+CPR+FUS	AMP+CPR+DOX	AMP+ERY+FUS	AMP+ERY+DOX	AMP+FUS+DOX	CPR+ERY+FUS	CPR+ERY+DOX	CPR+FUS+DOX	ERY+FUS+DOX	AMP+CPR+ERY+FUS	AMP+CPR+ERY+DOX	AMP+CPR+FUS+DOX	AMP+ERY+FUS+DOX	CPR+ERY+FUS+DOX	AMP+CPR+ERY+FUS+DOX
	75.22844656	88.33862829	99.06634883	65.33136422	107.753369	75.54628526	75.22844656	31.0488677	94.29876838	98.80396188	70.39805644	94.91683797	105.4231228	104.151768	80.39505261	68.55383393	36.13428685	103.1982519	35.04171633	84.76360747	28.36710369	71.89310409	115.2494861	64.41786582	76.04290822	42.17322209	87.94199444	47.43742551	34.08820024	69.80003738	45.21255463
	71.78315505	96.88521438	82.75002596	86.92719698	118.8057925	82.41769654	71.78315505	46.44303666	91.39059092	100.269179	88.27148625	116.881369	94.38155572	120.9679094	82.16623686	86.73797902	42.53816596	96.70786167	37.8024717	86.4056496	33.48218922	67.10248029	121.8034993	70.79407806	77.68200228	49.84941323	95.04621456	44.86447191	39.46411881	72.94751009	44.19981306
	77.18915757	92.58317025	91.48265823	63.3926997	99.22412273	86.71815801	74.80690746	43.0506627	94.54555123	96.9667319	81.3111546	122.6418787	95.906837	103.0535873	74.74872157	79.57140768	48.60215626	93.86490834	51.5586988	90.80201534	41.34905548	71.037182	121.3894325	86.0665362	80.93269346	53.70697792	81.61333635	43.0506627	52.57966313	79.17808219	47.13452003

AMP3CPR2ERY3FUS2DOX1	AMP	CPR	ERY	FUS	DOX	AMP+CPR	AMP+ERY	AMP+FUS	AMP+DOX	CPR+ERY	CPR+FUS	CPR+DOX	ERY+FUS	ERY+DOX	FUS+DOX	AMP+CPR+ERY	AMP+CPR+FUS	AMP+CPR+DOX	AMP+ERY+FUS	AMP+ERY+DOX	AMP+FUS+DOX	CPR+ERY+FUS	CPR+ERY+DOX	CPR+FUS+DOX	ERY+FUS+DOX	AMP+CPR+ERY+FUS	AMP+CPR+ERY+DOX	AMP+CPR+FUS+DOX	AMP+ERY+FUS+DOX	CPR+ERY+FUS+DOX	AMP+CPR+ERY+FUS+DOX
	75.22844656	88.33862829	99.06634883	92.3943142	108.9348348	75.54628526	75.22844656	52.34406039	96.84147795	98.80396188	82.35843768	105.9801906	110.1907032	106.0588002	74.19235739	68.55383393	53.29757648	89.53118792	48.84783472	91.7560588	53.15852205	85.34853298	110.4653336	69.80003738	75.40723083	60.60786651	87.94199444	49.02661899	53.15852205	60.53074192	51.25148987
	71.78315505	96.88521438	82.75002596	105.1577068	111.1882563	82.41769654	71.78315505	55.4990134	89.72894382	100.269179	98.73101327	114.1126706	105.3484266	94.71388514	87.24459433	86.73797902	55.4990134	90.72593208	50.18174265	82.75002596	45.11371897	92.57835032	113.1897712	73.56277639	80.00830824	61.81327241	87.73496729	49.76633088	55.74826046	67.41011344	50.76331914
	77.18915757	92.58317025	91.48265823	101.1990831	104.5847293	86.71815801	74.80690746	57.79083525	89.78105101	96.9667319	91.33072407	107.6125245	110.8809806	103.3939088	67.13101746	79.57140768	60.85372825	86.71815801	54.72794225	86.37783657	54.6215918	96.65362035	108.5518591	77.61252446	82.18762878	65.61822847	82.63430068	58.70544913	60.06673491	72.91585127	83.20859312

AMP3CPR2ERY3FUS2DOX2	AMP	CPR	ERY	FUS	DOX	AMP+CPR	AMP+ERY	AMP+FUS	AMP+DOX	CPR+ERY	CPR+FUS	CPR+DOX	ERY+FUS	ERY+DOX	FUS+DOX	AMP+CPR+ERY	AMP+CPR+FUS	AMP+CPR+DOX	AMP+ERY+FUS	AMP+ERY+DOX	AMP+FUS+DOX	CPR+ERY+FUS	CPR+ERY+DOX	CPR+FUS+DOX	ERY+FUS+DOX	AMP+CPR+ERY+FUS	AMP+CPR+ERY+DOX	AMP+CPR+FUS+DOX	AMP+ERY+FUS+DOX	CPR+ERY+FUS+DOX	AMP+CPR+ERY+FUS+DOX
	75.22844656	88.33862829	99.06634883	92.3943142	113.9560642	75.54628526	75.22844656	52.34406039	98.43067143	98.80396188	82.35843768	117.3425528	110.1907032	116.2296385	77.14602178	68.55383393	53.29757648	97.15931665	48.84783472	97.79499404	43.30552245	85.34853298	117.0435433	72.49112315	69.36829559	60.60786651	97.79499404	52.20500596	56.97258641	67.1089516	54.74771553
	71.78315505	96.88521438	82.75002596	105.1577068	121.6623686	82.41769654	71.78315505	55.4990134	91.72292034	100.269179	98.73101327	121.188233	105.3484266	106.6777443	83.11842888	86.73797902	55.4990134	95.7108734	50.18174265	94.0492263	46.11070724	92.57835032	122.1111325	74.17804268	73.02939038	61.81327241	89.06428497	46.11070724	47.1076955	68.94827918	47.77235435
	77.18915757	92.58317025	91.48265823	101.1990831	102.8919062	86.71815801	74.80690746	57.79083525	96.92780134	96.9667319	91.33072407	115.1272016	110.8809806	107.1374447	64.30964557	79.57140768	60.85372825	89.78105101	54.72794225	93.86490834	51.89902025	96.65362035	112.6223092	82.30919765	75.72152134	65.61822847	99.99069434	50.87805591	59.72641346	79.80430528	70.27637824

AMP3CPR2ERY3FUS2DOX3	AMP	CPR	ERY	FUS	DOX	AMP+CPR	AMP+ERY	AMP+FUS	AMP+DOX	CPR+ERY	CPR+FUS	CPR+DOX	ERY+FUS	ERY+DOX	FUS+DOX	AMP+CPR+ERY	AMP+CPR+FUS	AMP+CPR+DOX	AMP+ERY+FUS	AMP+ERY+DOX	AMP+FUS+DOX	CPR+ERY+FUS	CPR+ERY+DOX	CPR+FUS+DOX	ERY+FUS+DOX	AMP+CPR+ERY+FUS	AMP+CPR+ERY+DOX	AMP+CPR+FUS+DOX	AMP+ERY+FUS+DOX	CPR+ERY+FUS+DOX	AMP+CPR+ERY+FUS+DOX
	75.22844656	88.33862829	99.06634883	92.3943142	107.753369	75.54628526	75.22844656	52.34406039	94.29876838	98.80396188	82.35843768	94.91683797	110.1907032	104.151768	102.7321396	68.55383393	53.29757648	103.1982519	48.84783472	84.76360747	32.49900675	85.34853298	115.2494861	79.6673519	111.462058	60.60786651	87.94199444	69.50735002	31.54549066	85.04952345	68.55383393
	71.78315505	96.88521438	82.75002596	105.1577068	118.8057925	82.41769654	71.78315505	55.4990134	91.39059092	100.269179	98.73101327	116.881369	105.3484266	120.9679094	98.80975997	86.73797902	55.4990134	96.70786167	50.18174265	86.4056496	30.24197736	92.57835032	121.8034993	80.58065757	118.9739329	61.81327241	95.04621456	85.40866134	30.49122443	93.80888291	85.07633191
	77.18915757	92.58317025	91.48265823	101.1990831	99.22412273	86.71815801	74.80690746	57.79083525	94.54555123	96.9667319	91.33072407	122.6418787	110.8809806	103.0535873	93.58137895	79.57140768	60.85372825	93.86490834	54.72794225	90.80201534	34.54262659	96.65362035	121.3894325	81.3111546	118.7083738	65.61822847	81.61333635	75.82787179	33.8619837	86.63405088	69.36176435

AMP3CPR2ERY3FUS3DOX1	AMP	CPR	ERY	FUS	DOX	AMP+CPR	AMP+ERY	AMP+FUS	AMP+DOX	CPR+ERY	CPR+FUS	CPR+DOX	ERY+FUS	ERY+DOX	FUS+DOX	AMP+CPR+ERY	AMP+CPR+FUS	AMP+CPR+DOX	AMP+ERY+FUS	AMP+ERY+DOX	AMP+FUS+DOX	CPR+ERY+FUS	CPR+ERY+DOX	CPR+FUS+DOX	ERY+FUS+DOX	AMP+CPR+ERY+FUS	AMP+CPR+ERY+DOX	AMP+CPR+FUS+DOX	AMP+ERY+FUS+DOX	CPR+ERY+FUS+DOX	AMP+CPR+ERY+FUS+DOX
	75.22844656	88.33862829	99.06634883	103.6182389	108.9348348	75.54628526	75.22844656	87.30631704	96.84147795	98.80396188	105.9801906	105.9801906	116.8653159	106.0588002	79.69355732	68.55383393	85.08144617	89.53118792	79.99602702	91.7560588	70.14302741	103.8871239	110.4653336	90.43169501	97.79499404	84.76360747	87.94199444	70.14302741	75.22844656	76.37824706	70.77870481
	71.78315505	96.88521438	82.75002596	117.5362031	111.1882563	82.41769654	71.78315505	82.75002596	89.72894382	100.269179	113.1897712	114.1126706	110.6656974	94.71388514	70.24399921	86.73797902	78.42974348	90.72593208	82.08536712	82.75002596	83.74701423	116.5737358	113.1897712	84.88752163	110.0010385	80.75604943	87.73496729	79.09440233	92.05524977	86.11805422	85.07633191
	77.18915757	92.58317025	91.48265823	113.6131194	104.5847293	86.71815801	74.80690746	85.01655079	89.78105101	96.9667319	111.0567515	107.6125245	124.4938383	103.3939088	58.59636748	79.57140768	79.57140768	86.71815801	86.03751512	86.37783657	77.18915757	107.925636	108.5518591	92.27005871	98.96973	93.18426545	82.63430068	72.42465735	79.57140768	86.32093933	71.40369302

AMP3CPR2ERY3FUS3DOX2	AMP	CPR	ERY	FUS	DOX	AMP+CPR	AMP+ERY	AMP+FUS	AMP+DOX	CPR+ERY	CPR+FUS	CPR+DOX	ERY+FUS	ERY+DOX	FUS+DOX	AMP+CPR+ERY	AMP+CPR+FUS	AMP+CPR+DOX	AMP+ERY+FUS	AMP+ERY+DOX	AMP+FUS+DOX	CPR+ERY+FUS	CPR+ERY+DOX	CPR+FUS+DOX	ERY+FUS+DOX	AMP+CPR+ERY+FUS	AMP+CPR+ERY+DOX	AMP+CPR+FUS+DOX	AMP+ERY+FUS+DOX	CPR+ERY+FUS+DOX	AMP+CPR+ERY+FUS+DOX
	75.22844656	88.33862829	99.06634883	103.6182389	113.9560642	75.54628526	75.22844656	87.30631704	98.43067143	98.80396188	105.9801906	117.3425528	116.8653159	116.2296385	86.78235186	68.55383393	85.08144617	97.15931665	79.99602702	97.79499404	66.96464044	103.8871239	117.0435433	90.73070454	92.70957489	84.76360747	97.79499404	74.91060787	72.05005959	90.43169501	65.37544696
	71.78315505	96.88521438	82.75002596	117.5362031	121.6623686	82.41769654	71.78315505	82.75002596	91.72292034	100.269179	113.1897712	121.188233	110.6656974	106.6777443	76.90934338	86.73797902	78.42974348	95.7108734	82.08536712	94.0492263	86.4056496	116.5737358	122.1111325	89.1943857	103.6867795	80.75604943	89.06428497	100.0311559	102.0251324	89.1943857	95.37854398
	77.18915757	92.58317025	91.48265823	113.6131194	102.8919062	86.71815801	74.80690746	85.01655079	96.92780134	96.9667319	111.0567515	115.1272016	124.4938383	107.1374447	76.93528478	79.57140768	79.57140768	89.78105101	86.03751512	93.86490834	69.7020858	107.925636	112.6223092	82.87671233	103.0535873	93.18426545	99.99069434	73.44562168	73.44562168	87.88649706	79.91172913

AMP3CPR2ERY3FUS3DOX3	AMP	CPR	ERY	FUS	DOX	AMP+CPR	AMP+ERY	AMP+FUS	AMP+DOX	CPR+ERY	CPR+FUS	CPR+DOX	ERY+FUS	ERY+DOX	FUS+DOX	AMP+CPR+ERY	AMP+CPR+FUS	AMP+CPR+DOX	AMP+ERY+FUS	AMP+ERY+DOX	AMP+FUS+DOX	CPR+ERY+FUS	CPR+ERY+DOX	CPR+FUS+DOX	ERY+FUS+DOX	AMP+CPR+ERY+FUS	AMP+CPR+ERY+DOX	AMP+CPR+FUS+DOX	AMP+ERY+FUS+DOX	CPR+ERY+FUS+DOX	AMP+CPR+ERY+FUS+DOX
	75.22844656	88.33862829	99.06634883	103.6182389	107.753369	75.54628526	75.22844656	87.30631704	94.29876838	98.80396188	105.9801906	94.91683797	116.8653159	104.151768	97.7109101	68.55383393	85.08144617	103.1982519	79.99602702	84.76360747	71.4143822	103.8871239	115.2494861	107.1762287	114.0047676	84.76360747	87.94199444	72.36789829	67.28247914	103.5881144	67.60031784
	71.78315505	96.88521438	82.75002596	117.5362031	118.8057925	82.41769654	71.78315505	82.75002596	91.39059092	100.269179	113.1897712	116.881369	110.6656974	120.9679094	108.3316802	86.73797902	78.42974348	96.70786167	82.08536712	86.4056496	77.43275522	116.5737358	121.8034993	107.9600077	116.3152975	80.75604943	95.04621456	86.73797902	84.07934365	82.7340896	88.06729671
	77.18915757	92.58317025	91.48265823	113.6131194	99.22412273	86.71815801	74.80690746	85.01655079	94.54555123	96.9667319	111.0567515	122.6418787	124.4938383	103.0535873	102.3276318	79.57140768	79.57140768	93.86490834	86.03751512	90.80201534	76.84883613	107.925636	121.3894325	102.6027397	124.8341598	93.18426545	81.61333635	78.2101219	86.37783657	82.56360078	77.86980046

AMP3CPR3ERY1FUS1DOX1	AMP	CPR	ERY	FUS	DOX	AMP+CPR	AMP+ERY	AMP+FUS	AMP+DOX	CPR+ERY	CPR+FUS	CPR+DOX	ERY+FUS	ERY+DOX	FUS+DOX	AMP+CPR+ERY	AMP+CPR+FUS	AMP+CPR+DOX	AMP+ERY+FUS	AMP+ERY+DOX	AMP+FUS+DOX	CPR+ERY+FUS	CPR+ERY+DOX	CPR+FUS+DOX	ERY+FUS+DOX	AMP+CPR+ERY+FUS	AMP+CPR+ERY+DOX	AMP+CPR+FUS+DOX	AMP+ERY+FUS+DOX	CPR+ERY+FUS+DOX	AMP+CPR+ERY+FUS+DOX
	75.22844656	92.70957489	94.46187927	65.33136422	108.9348348	74.91060787	71.0965435	31.0488677	96.84147795	91.7560588	102.2447358	104.4696067	61.19623408	105.0950711	58.27949049	64.42193087	38.35915773	97.15931665	24.55303933	96.84147795	37.26658721	72.22884386	105.1052841	63.32936035	49.71386376	33.27373858	91.12038141	40.76281287	37.26658721	40.58402861	38.2201033
	71.78315505	82.75002596	92.14441579	86.92719698	111.1882563	74.109461	70.45383737	46.44303666	89.72894382	80.75604943	94.71388514	105.3484266	80.26185281	102.6185281	61.53540964	78.7620729	54.16969571	91.72292034	28.58033025	86.4056496	50.43098972	81.0052965	102.0251324	70.70308443	47.6294386	40.21186001	83.74701423	61.06553121	36.14082459	48.10468377	34.14684806
	77.18915757	88.76008668	100.9169459	63.3926997	104.5847293	81.95365779	84.3359079	43.0506627	89.78105101	87.73912234	94.54555123	102.3729444	71.01040381	109.6631987	57.78522306	77.18915757	52.23934169	102.7132659	36.9248767	94.88587267	68.23444957	76.06184279	102.7132659	64.15059224	47.62828425	52.23934169	86.37783657	67.21348524	20.972309	48.83612725	47.47484147

AMP3CPR3ERY1FUS1DOX2	AMP	CPR	ERY	FUS	DOX	AMP+CPR	AMP+ERY	AMP+FUS	AMP+DOX	CPR+ERY	CPR+FUS	CPR+DOX	ERY+FUS	ERY+DOX	FUS+DOX	AMP+CPR+ERY	AMP+CPR+FUS	AMP+CPR+DOX	AMP+ERY+FUS	AMP+ERY+DOX	AMP+FUS+DOX	CPR+ERY+FUS	CPR+ERY+DOX	CPR+FUS+DOX	ERY+FUS+DOX	AMP+CPR+ERY+FUS	AMP+CPR+ERY+DOX	AMP+CPR+FUS+DOX	AMP+ERY+FUS+DOX	CPR+ERY+FUS+DOX	AMP+CPR+ERY+FUS+DOX
	75.22844656	92.70957489	94.46187927	65.33136422	113.9560642	74.91060787	71.0965435	31.0488677	98.43067143	91.7560588	102.2447358	118.1366706	61.19623408	107.1626361	61.19623408	64.42193087	38.35915773	107.0123162	24.55303933	103.1982519	37.9022646	72.22884386	106.6944776	66.82558602	45.57873362	33.27373858	93.66309098	48.70878029	25.50655542	40.9018673	29.95629718
	71.78315505	82.75002596	92.14441579	86.92719698	121.6623686	74.109461	70.45383737	46.44303666	91.72292034	80.75604943	94.71388514	107.3424032	80.26185281	118.1709978	62.17020432	78.7620729	54.16969571	99.03416762	28.58033025	100.3634853	33.1498598	81.0052965	95.04621456	80.67296708	44.13806784	40.21186001	87.73496729	57.74223699	44.44906013	56.74524873	36.14082459
	77.18915757	88.76008668	100.9169459	63.3926997	102.8919062	81.95365779	84.3359079	43.0506627	96.92780134	87.73912234	94.54555123	109.1793733	71.01040381	101.4812202	51.01393052	77.18915757	52.23934169	96.92780134	36.9248767	100.3310158	30.11844782	76.06184279	104.4148731	65.51187802	46.78187269	52.23934169	87.73912234	60.7473778	44.41194847	43.43352432	38.96680537

AMP3CPR3ERY1FUS1DOX3	AMP	CPR	ERY	FUS	DOX	AMP+CPR	AMP+ERY	AMP+FUS	AMP+DOX	CPR+ERY	CPR+FUS	CPR+DOX	ERY+FUS	ERY+DOX	FUS+DOX	AMP+CPR+ERY	AMP+CPR+FUS	AMP+CPR+DOX	AMP+ERY+FUS	AMP+ERY+DOX	AMP+FUS+DOX	CPR+ERY+FUS	CPR+ERY+DOX	CPR+FUS+DOX	ERY+FUS+DOX	AMP+CPR+ERY+FUS	AMP+CPR+ERY+DOX	AMP+CPR+FUS+DOX	AMP+ERY+FUS+DOX	CPR+ERY+FUS+DOX	AMP+CPR+ERY+FUS+DOX
	75.22844656	92.70957489	94.46187927	65.33136422	107.753369	74.91060787	71.0965435	31.0488677	94.29876838	91.7560588	102.2447358	105.4231228	61.19623408	103.6182389	80.39505261	64.42193087	38.35915773	91.4382201	24.55303933	93.02741359	28.36710369	72.22884386	102.8804132	80.49264998	73.89699095	33.27373858	104.151768	39.63051251	29.63845848	68.7326182	49.02661899
	71.78315505	82.75002596	92.14441579	86.92719698	118.8057925	74.109461	70.45383737	46.44303666	91.39059092	80.75604943	94.71388514	116.9799564	80.26185281	113.0926403	82.16623686	78.7620729	54.16969571	91.72292034	28.58033025	87.73496729	33.48218922	81.0052965	105.0160972	88.6488732	74.866098	40.21186001	100.6958147	43.86748364	42.12275418	73.3617198	54.16969571
	77.18915757	88.76008668	100.9169459	63.3926997	99.22412273	81.95365779	84.3359079	43.0506627	94.54555123	87.73912234	94.54555123	109.1793733	71.01040381	98.65984835	74.74872157	77.18915757	52.23934169	93.52458689	36.9248767	86.71815801	41.34905548	76.06184279	101.6923016	88.31341478	71.64521248	52.23934169	98.28908711	50.53773447	42.02969836	74.70055701	49.17644869

AMP3CPR3ERY1FUS2DOX1	AMP	CPR	ERY	FUS	DOX	AMP+CPR	AMP+ERY	AMP+FUS	AMP+DOX	CPR+ERY	CPR+FUS	CPR+DOX	ERY+FUS	ERY+DOX	FUS+DOX	AMP+CPR+ERY	AMP+CPR+FUS	AMP+CPR+DOX	AMP+ERY+FUS	AMP+ERY+DOX	AMP+FUS+DOX	CPR+ERY+FUS	CPR+ERY+DOX	CPR+FUS+DOX	ERY+FUS+DOX	AMP+CPR+ERY+FUS	AMP+CPR+ERY+DOX	AMP+CPR+FUS+DOX	AMP+ERY+FUS+DOX	CPR+ERY+FUS+DOX	AMP+CPR+ERY+FUS+DOX
	75.22844656	92.70957489	94.46187927	92.3943142	108.9348348	74.91060787	71.0965435	52.34406039	96.84147795	91.7560588	102.2447358	104.4696067	87.66845117	105.0950711	74.19235739	64.42193087	71.7322209	97.15931665	37.72348033	96.84147795	53.15852205	74.27493047	105.1052841	79.53913389	65.33136422	55.20460866	91.12038141	60.15097338	48.0731029	68.7326182	51.25148987
	71.78315505	82.75002596	92.14441579	105.1577068	111.1882563	74.109461	70.45383737	55.4990134	89.72894382	80.75604943	95.04621456	105.3484266	89.9226344	102.6185281	87.24459433	78.7620729	70.78616679	91.72292034	41.54117769	86.4056496	45.11371897	73.44480216	102.0251324	83.99626129	64.39198572	57.49298993	83.74701423	60.73320179	52.75729567	66.71513137	46.44303666
	77.18915757	88.76008668	100.9169459	101.1990831	104.5847293	81.95365779	84.3359079	57.79083525	89.78105101	87.73912234	109.5196948	102.3729444	91.88855581	109.6631987	67.13101746	77.18915757	73.44562168	102.7132659	40.09412015	94.88587267	54.6215918	80.25205057	102.7132659	85.59084323	53.024158	58.47147814	86.37783657	59.38609202	52.57966313	77.08280712	57.34416335

AMP3CPR3ERY1FUS2DOX2	AMP	CPR	ERY	FUS	DOX	AMP+CPR	AMP+ERY	AMP+FUS	AMP+DOX	CPR+ERY	CPR+FUS	CPR+DOX	ERY+FUS	ERY+DOX	FUS+DOX	AMP+CPR+ERY	AMP+CPR+FUS	AMP+CPR+DOX	AMP+ERY+FUS	AMP+ERY+DOX	AMP+FUS+DOX	CPR+ERY+FUS	CPR+ERY+DOX	CPR+FUS+DOX	ERY+FUS+DOX	AMP+CPR+ERY+FUS	AMP+CPR+ERY+DOX	AMP+CPR+FUS+DOX	AMP+ERY+FUS+DOX	CPR+ERY+FUS+DOX	AMP+CPR+ERY+FUS+DOX
	75.22844656	92.70957489	94.46187927	92.3943142	113.9560642	74.91060787	71.0965435	52.34406039	98.43067143	91.7560588	102.2447358	118.1366706	87.66845117	107.1626361	77.14602178	64.42193087	71.7322209	107.0123162	37.72348033	103.1982519	43.30552245	74.27493047	106.6944776	79.22129519	67.3989293	55.20460866	93.66309098	56.01907032	54.11203814	82.71752086	49.02661899
	71.78315505	82.75002596	92.14441579	105.1577068	121.6623686	74.109461	70.45383737	55.4990134	91.72292034	80.75604943	95.04621456	107.3424032	89.9226344	118.1709978	83.11842888	78.7620729	70.78616679	99.03416762	41.54117769	100.3634853	46.11070724	73.44480216	95.04621456	86.32256724	81.84883952	57.49298993	87.73496729	52.09263683	47.44002492	78.01433171	46.77536608
	77.18915757	88.76008668	100.9169459	101.1990831	102.8919062	81.95365779	84.3359079	57.79083525	96.92780134	87.73912234	109.5196948	109.1793733	91.88855581	101.4812202	64.30964557	77.18915757	73.44562168	96.92780134	40.09412015	100.3310158	51.89902025	80.25205057	104.4148731	93.41823644	61.48827367	58.47147814	87.73912234	49.17644869	44.07162703	75.3811999	48.83612725

AMP3CPR3ERY1FUS2DOX3	AMP	CPR	ERY	FUS	DOX	AMP+CPR	AMP+ERY	AMP+FUS	AMP+DOX	CPR+ERY	CPR+FUS	CPR+DOX	ERY+FUS	ERY+DOX	FUS+DOX	AMP+CPR+ERY	AMP+CPR+FUS	AMP+CPR+DOX	AMP+ERY+FUS	AMP+ERY+DOX	AMP+FUS+DOX	CPR+ERY+FUS	CPR+ERY+DOX	CPR+FUS+DOX	ERY+FUS+DOX	AMP+CPR+ERY+FUS	AMP+CPR+ERY+DOX	AMP+CPR+FUS+DOX	AMP+ERY+FUS+DOX	CPR+ERY+FUS+DOX	AMP+CPR+ERY+FUS+DOX
	75.22844656	92.70957489	94.46187927	92.3943142	107.753369	74.91060787	71.0965435	52.34406039	94.29876838	91.7560588	102.2447358	105.4231228	87.66845117	103.6182389	102.7321396	64.42193087	71.7322209	91.4382201	37.72348033	93.02741359	32.49900675	74.27493047	102.8804132	93.98092968	88.55455049	55.20460866	104.151768	87.30631704	35.35955503	83.35319825	75.54628526
	71.78315505	82.75002596	92.14441579	105.1577068	118.8057925	74.109461	70.45383737	55.4990134	91.39059092	80.75604943	95.04621456	116.9799564	89.9226344	113.0926403	98.80975997	78.7620729	70.78616679	91.72292034	41.54117769	87.73496729	30.24197736	73.44480216	105.0160972	107.0100737	83.25729022	57.49298993	100.6958147	108.3393914	22.84764773	79.01131997	88.73195555
	77.18915757	88.76008668	100.9169459	101.1990831	99.22412273	81.95365779	84.3359079	57.79083525	94.54555123	87.73912234	109.5196948	109.1793733	91.88855581	98.65984835	93.58137895	77.18915757	73.44562168	93.52458689	40.09412015	86.71815801	34.54262659	80.25205057	101.6923016	104.7551946	80.32093105	58.47147814	98.28908711	98.28908711	25.3539476	80.14570012	79.23108624

AMP3CPR3ERY1FUS3DOX1	AMP	CPR	ERY	FUS	DOX	AMP+CPR	AMP+ERY	AMP+FUS	AMP+DOX	CPR+ERY	CPR+FUS	CPR+DOX	ERY+FUS	ERY+DOX	FUS+DOX	AMP+CPR+ERY	AMP+CPR+FUS	AMP+CPR+DOX	AMP+ERY+FUS	AMP+ERY+DOX	AMP+FUS+DOX	CPR+ERY+FUS	CPR+ERY+DOX	CPR+FUS+DOX	ERY+FUS+DOX	AMP+CPR+ERY+FUS	AMP+CPR+ERY+DOX	AMP+CPR+FUS+DOX	AMP+ERY+FUS+DOX	CPR+ERY+FUS+DOX	AMP+CPR+ERY+FUS+DOX
	75.22844656	92.70957489	94.46187927	103.6182389	108.9348348	74.91060787	71.0965435	87.30631704	96.84147795	91.7560588	118.4545093	104.4696067	96.52944434	105.0950711	79.69355732	64.42193087	85.39928486	97.15931665	83.49225268	96.84147795	70.14302741	104.7874454	105.1052841	103.8339293	72.01402991	77.13547874	91.12038141	65.69328566	60.60786651	86.98847835	56.79380215
	71.78315505	82.75002596	92.14441579	117.5362031	111.1882563	74.109461	70.45383737	82.75002596	89.72894382	80.75604943	110.0010385	105.3484266	110.870859	102.6185281	70.24399921	78.7620729	82.41769654	91.72292034	76.10343753	86.4056496	83.74701423	102.6897913	102.0251324	109.6687091	65.66157508	76.76809638	83.74701423	92.38757919	78.09741406	91.72292034	69.78917852
	77.18915757	88.76008668	100.9169459	113.6131194	104.5847293	81.95365779	84.3359079	85.01655079	89.78105101	87.73912234	118.0277309	102.3729444	106.5596896	109.6631987	58.59636748	77.18915757	90.4616939	102.7132659	83.99558646	94.88587267	77.18915757	103.3939088	102.7132659	99.65037289	56.05713278	77.52947902	86.37783657	74.12626457	58.47147814	80.59237201	63.91662125

AMP3CPR3ERY1FUS3DOX2	AMP	CPR	ERY	FUS	DOX	AMP+CPR	AMP+ERY	AMP+FUS	AMP+DOX	CPR+ERY	CPR+FUS	CPR+DOX	ERY+FUS	ERY+DOX	FUS+DOX	AMP+CPR+ERY	AMP+CPR+FUS	AMP+CPR+DOX	AMP+ERY+FUS	AMP+ERY+DOX	AMP+FUS+DOX	CPR+ERY+FUS	CPR+ERY+DOX	CPR+FUS+DOX	ERY+FUS+DOX	AMP+CPR+ERY+FUS	AMP+CPR+ERY+DOX	AMP+CPR+FUS+DOX	AMP+ERY+FUS+DOX	CPR+ERY+FUS+DOX	AMP+CPR+ERY+FUS+DOX
	75.22844656	92.70957489	94.46187927	103.6182389	113.9560642	74.91060787	71.0965435	87.30631704	98.43067143	91.7560588	118.4545093	118.1366706	96.52944434	107.1626361	86.78235186	64.42193087	85.39928486	107.0123162	83.49225268	103.1982519	66.96464044	104.7874454	106.6944776	99.70202622	68.1742662	77.13547874	93.66309098	63.96503774	61.5613826	83.49225268	52.52284466
	71.78315505	82.75002596	92.14441579	117.5362031	121.6623686	74.109461	70.45383737	82.75002596	91.72292034	80.75604943	110.0010385	107.3424032	110.870859	118.1709978	76.90934338	78.7620729	82.41769654	99.03416762	76.10343753	100.3634853	86.4056496	102.6897913	95.04621456	110.6656974	66.29636977	76.76809638	87.73496729	82.08536712	71.11849621	94.0492263	79.09440233
	77.18915757	88.76008668	100.9169459	113.6131194	102.8919062	81.95365779	84.3359079	85.01655079	96.92780134	87.73912234	118.0277309	109.1793733	106.5596896	101.4812202	76.93528478	77.18915757	90.4616939	96.92780134	83.99558646	100.3310158	69.7020858	103.3939088	104.4148731	102.032623	58.1026274	77.52947902	87.73912234	77.86980046	57.79083525	81.95365779	59.15212103

AMP3CPR3ERY1FUS3DOX3	AMP	CPR	ERY	FUS	DOX	AMP+CPR	AMP+ERY	AMP+FUS	AMP+DOX	CPR+ERY	CPR+FUS	CPR+DOX	ERY+FUS	ERY+DOX	FUS+DOX	AMP+CPR+ERY	AMP+CPR+FUS	AMP+CPR+DOX	AMP+ERY+FUS	AMP+ERY+DOX	AMP+FUS+DOX	CPR+ERY+FUS	CPR+ERY+DOX	CPR+FUS+DOX	ERY+FUS+DOX	AMP+CPR+ERY+FUS	AMP+CPR+ERY+DOX	AMP+CPR+FUS+DOX	AMP+ERY+FUS+DOX	CPR+ERY+FUS+DOX	AMP+CPR+ERY+FUS+DOX
	75.22844656	92.70957489	94.46187927	103.6182389	107.753369	74.91060787	71.0965435	87.30631704	94.29876838	91.7560588	118.4545093	105.4231228	96.52944434	103.6182389	97.7109101	64.42193087	85.39928486	91.4382201	83.49225268	93.02741359	71.4143822	104.7874454	102.8804132	107.0123162	90.03138268	77.13547874	104.151768	66.50774732	61.8792213	92.70957489	61.8792213
	71.78315505	82.75002596	92.14441579	117.5362031	118.8057925	74.109461	70.45383737	82.75002596	91.39059092	80.75604943	110.0010385	116.9799564	110.870859	113.0926403	108.3316802	78.7620729	82.41769654	91.72292034	76.10343753	87.73496729	77.43275522	102.6897913	105.0160972	124.6235331	94.36619718	76.76809638	100.6958147	85.74099076	75.77110811	103.0221207	80.75604943
	77.18915757	88.76008668	100.9169459	113.6131194	99.22412273	81.95365779	84.3359079	85.01655079	94.54555123	87.73912234	118.0277309	109.1793733	106.5596896	98.65984835	102.3276318	77.18915757	90.4616939	93.52458689	83.99558646	86.71815801	76.84883613	103.3939088	101.6923016	123.472874	88.22077235	77.52947902	98.28908711	84.67622934	76.50851468	103.7342302	72.42465735

AMP3CPR3ERY2FUS1DOX1	AMP	CPR	ERY	FUS	DOX	AMP+CPR	AMP+ERY	AMP+FUS	AMP+DOX	CPR+ERY	CPR+FUS	CPR+DOX	ERY+FUS	ERY+DOX	FUS+DOX	AMP+CPR+ERY	AMP+CPR+FUS	AMP+CPR+DOX	AMP+ERY+FUS	AMP+ERY+DOX	AMP+FUS+DOX	CPR+ERY+FUS	CPR+ERY+DOX	CPR+FUS+DOX	ERY+FUS+DOX	AMP+CPR+ERY+FUS	AMP+CPR+ERY+DOX	AMP+CPR+FUS+DOX	AMP+ERY+FUS+DOX	CPR+ERY+FUS+DOX	AMP+CPR+ERY+FUS+DOX
	75.22844656	92.70957489	92.2257522	65.33136422	108.9348348	74.91060787	74.59276917	31.0488677	96.84147795	91.12038141	102.2447358	104.4696067	70.39805644	101.7940572	58.27949049	70.77870481	38.35915773	97.15931665	25.50655542	102.2447358	37.26658721	66.50774732	109.5550258	63.32936035	56.43804896	38.04131903	90.48470401	40.76281287	66.82558602	63.32936035	27.87048073
	71.78315505	82.75002596	104.2684099	86.92719698	111.1882563	74.109461	68.45986084	46.44303666	89.72894382	88.73195555	94.71388514	105.3484266	86.73332052	111.9592386	61.53540964	76.10343753	54.16969571	91.72292034	54.41894278	98.36950878	50.43098972	81.0052965	105.3484266	70.70308443	36.89675062	46.52611902	90.39360266	61.06553121	53.08962509	69.04143732	36.47315401
	77.18915757	88.76008668	95.40117417	63.3926997	104.5847293	81.95365779	78.2101219	43.0506627	89.78105101	86.03751512	94.54555123	102.3729444	81.93737769	103.5420744	57.78522306	81.61333635	52.23934169	102.7132659	42.37001981	97.26812278	68.23444957	85.59084323	104.0745517	64.15059224	51.25244618	45.8795847	91.14233679	67.21348524	65.51187802	68.91509246	34.88294803

AMP3CPR3ERY2FUS1DOX2	AMP	CPR	ERY	FUS	DOX	AMP+CPR	AMP+ERY	AMP+FUS	AMP+DOX	CPR+ERY	CPR+FUS	CPR+DOX	ERY+FUS	ERY+DOX	FUS+DOX	AMP+CPR+ERY	AMP+CPR+FUS	AMP+CPR+DOX	AMP+ERY+FUS	AMP+ERY+DOX	AMP+FUS+DOX	CPR+ERY+FUS	CPR+ERY+DOX	CPR+FUS+DOX	ERY+FUS+DOX	AMP+CPR+ERY+FUS	AMP+CPR+ERY+DOX	AMP+CPR+FUS+DOX	AMP+ERY+FUS+DOX	CPR+ERY+FUS+DOX	AMP+CPR+ERY+FUS+DOX
	75.22844656	92.70957489	92.2257522	65.33136422	113.9560642	74.91060787	74.59276917	31.0488677	98.43067143	91.12038141	102.2447358	118.1366706	70.39805644	107.1762287	61.19623408	70.77870481	38.35915773	107.0123162	25.50655542	95.25228447	37.9022646	66.50774732	112.0977354	66.82558602	49.85983928	38.04131903	101.6090584	48.70878029	43.62336114	63.32936035	32.18116806
	71.78315505	82.75002596	104.2684099	86.92719698	121.6623686	74.109461	68.45986084	46.44303666	91.72292034	88.73195555	94.71388514	107.3424032	86.73332052	117.4966353	62.17020432	76.10343753	54.16969571	99.03416762	54.41894278	94.38155572	33.1498598	81.0052965	110.333368	80.67296708	40.03076331	46.52611902	94.0492263	57.74223699	53.75428393	70.37075501	43.11974244
	77.18915757	88.76008668	95.40117417	63.3926997	102.8919062	81.95365779	78.2101219	43.0506627	96.92780134	86.03751512	94.54555123	109.1793733	81.93737769	111.369863	51.01393052	81.61333635	52.23934169	96.92780134	42.37001981	106.7971232	30.11844782	85.59084323	102.032623	65.51187802	48.43444227	45.8795847	98.28908711	60.7473778	64.15059224	72.99894979	36.24423381

AMP3CPR3ERY2FUS1DOX3	AMP	CPR	ERY	FUS	DOX	AMP+CPR	AMP+ERY	AMP+FUS	AMP+DOX	CPR+ERY	CPR+FUS	CPR+DOX	ERY+FUS	ERY+DOX	FUS+DOX	AMP+CPR+ERY	AMP+CPR+FUS	AMP+CPR+DOX	AMP+ERY+FUS	AMP+ERY+DOX	AMP+FUS+DOX	CPR+ERY+FUS	CPR+ERY+DOX	CPR+FUS+DOX	ERY+FUS+DOX	AMP+CPR+ERY+FUS	AMP+CPR+ERY+DOX	AMP+CPR+FUS+DOX	AMP+ERY+FUS+DOX	CPR+ERY+FUS+DOX	AMP+CPR+ERY+FUS+DOX
	75.22844656	92.70957489	92.2257522	65.33136422	107.753369	74.91060787	74.59276917	31.0488677	94.29876838	91.12038141	102.2447358	105.4231228	70.39805644	109.8673145	80.39505261	70.77870481	38.35915773	91.4382201	25.50655542	96.20580056	28.36710369	66.50774732	106.6944776	80.49264998	59.63371332	38.04131903	100.0198649	39.63051251	46.16607072	76.36074692	48.70878029
	71.78315505	82.75002596	104.2684099	86.92719698	118.8057925	74.109461	68.45986084	46.44303666	91.39059092	88.73195555	94.71388514	116.9799564	86.73332052	114.4203038	82.16623686	76.10343753	54.16969571	91.72292034	54.41894278	93.38456745	33.48218922	81.0052965	109.0040503	88.6488732	63.71851567	46.52611902	92.38757919	43.86748364	45.11371897	82.66694361	50.76331914
	77.18915757	88.76008668	95.40117417	63.3926997	99.22412273	81.95365779	78.2101219	43.0506627	94.54555123	86.03751512	94.54555123	109.1793733	81.93737769	111.0567515	74.74872157	81.61333635	52.23934169	93.52458689	42.37001981	98.28908711	41.34905548	85.59084323	105.7761589	88.31341478	80.43052838	45.8795847	98.62940856	50.53773447	50.19741303	81.84730734	55.30223469

AMP3CPR3ERY2FUS2DOX1	AMP	CPR	ERY	FUS	DOX	AMP+CPR	AMP+ERY	AMP+FUS	AMP+DOX	CPR+ERY	CPR+FUS	CPR+DOX	ERY+FUS	ERY+DOX	FUS+DOX	AMP+CPR+ERY	AMP+CPR+FUS	AMP+CPR+DOX	AMP+ERY+FUS	AMP+ERY+DOX	AMP+FUS+DOX	CPR+ERY+FUS	CPR+ERY+DOX	CPR+FUS+DOX	ERY+FUS+DOX	AMP+CPR+ERY+FUS	AMP+CPR+ERY+DOX	AMP+CPR+FUS+DOX	AMP+ERY+FUS+DOX	CPR+ERY+FUS+DOX	AMP+CPR+ERY+FUS+DOX
	75.22844656	92.70957489	92.2257522	92.3943142	108.9348348	74.91060787	74.59276917	52.34406039	96.84147795	91.12038141	102.2447358	104.4696067	90.13268548	101.7940572	74.19235739	70.77870481	71.7322209	97.15931665	48.52999603	102.2447358	53.15852205	91.7560588	109.5550258	79.53913389	62.3247991	59.65435042	90.48470401	60.15097338	49.34445769	77.94994041	52.84068335
	71.78315505	82.75002596	104.2684099	105.1577068	111.1882563	74.109461	68.45986084	55.4990134	89.72894382	88.73195555	95.04621456	105.3484266	101.8073447	111.9592386	87.24459433	76.10343753	70.78616679	91.72292034	47.19077786	98.36950878	45.11371897	90.39360266	105.3484266	83.99626129	64.64141511	57.1606605	90.39360266	60.73320179	49.43400145	79.01131997	47.44002492
	77.18915757	88.76008668	95.40117417	101.1990831	104.5847293	81.95365779	78.2101219	57.79083525	89.78105101	86.03751512	109.5196948	102.3729444	98.84540117	103.5420744	67.13101746	81.61333635	73.44562168	102.7132659	48.60215626	97.26812278	54.6215918	97.60844423	104.0745517	85.59084323	64.46183953	67.31983569	91.14233679	59.38609202	50.53773447	80.48602156	56.66352047

AMP3CPR3ERY2FUS2DOX2	AMP	CPR	ERY	FUS	DOX	AMP+CPR	AMP+ERY	AMP+FUS	AMP+DOX	CPR+ERY	CPR+FUS	CPR+DOX	ERY+FUS	ERY+DOX	FUS+DOX	AMP+CPR+ERY	AMP+CPR+FUS	AMP+CPR+DOX	AMP+ERY+FUS	AMP+ERY+DOX	AMP+FUS+DOX	CPR+ERY+FUS	CPR+ERY+DOX	CPR+FUS+DOX	ERY+FUS+DOX	AMP+CPR+ERY+FUS	AMP+CPR+ERY+DOX	AMP+CPR+FUS+DOX	AMP+ERY+FUS+DOX	CPR+ERY+FUS+DOX	AMP+CPR+ERY+FUS+DOX
	75.22844656	92.70957489	92.2257522	92.3943142	113.9560642	74.91060787	74.59276917	52.34406039	98.43067143	91.12038141	102.2447358	118.1366706	90.13268548	107.1762287	77.14602178	70.77870481	71.7322209	107.0123162	48.52999603	95.25228447	43.30552245	91.7560588	112.0977354	79.22129519	71.29508503	59.65435042	101.6090584	56.01907032	53.47636075	82.08184346	57.60826381
	71.78315505	82.75002596	104.2684099	105.1577068	121.6623686	74.109461	68.45986084	55.4990134	91.72292034	88.73195555	95.04621456	107.3424032	101.8073447	117.4966353	83.11842888	76.10343753	70.78616679	99.03416762	47.19077786	94.38155572	46.11070724	90.39360266	110.333368	86.32256724	71.7169775	57.1606605	94.0492263	52.09263683	50.76331914	79.34364939	48.43701319
	77.18915757	88.76008668	95.40117417	101.1990831	102.8919062	81.95365779	78.2101219	57.79083525	96.92780134	86.03751512	109.5196948	109.1793733	98.84540117	111.369863	64.30964557	81.61333635	73.44562168	96.92780134	48.60215626	106.7971232	51.89902025	97.60844423	102.032623	93.41823644	66.9667319	67.31983569	98.28908711	49.17644869	50.87805591	86.27148611	50.87805591

AMP3CPR3ERY2FUS2DOX3	AMP	CPR	ERY	FUS	DOX	AMP+CPR	AMP+ERY	AMP+FUS	AMP+DOX	CPR+ERY	CPR+FUS	CPR+DOX	ERY+FUS	ERY+DOX	FUS+DOX	AMP+CPR+ERY	AMP+CPR+FUS	AMP+CPR+DOX	AMP+ERY+FUS	AMP+ERY+DOX	AMP+FUS+DOX	CPR+ERY+FUS	CPR+ERY+DOX	CPR+FUS+DOX	ERY+FUS+DOX	AMP+CPR+ERY+FUS	AMP+CPR+ERY+DOX	AMP+CPR+FUS+DOX	AMP+ERY+FUS+DOX	CPR+ERY+FUS+DOX	AMP+CPR+ERY+FUS+DOX
	75.22844656	92.70957489	92.2257522	92.3943142	107.753369	74.91060787	74.59276917	52.34406039	94.29876838	91.12038141	102.2447358	105.4231228	90.13268548	109.8673145	102.7321396	70.77870481	71.7322209	91.4382201	48.52999603	96.20580056	32.49900675	91.7560588	106.6944776	93.98092968	106.2792001	59.65435042	100.0198649	87.30631704	37.5844259	77.31426301	81.5852205
	71.78315505	82.75002596	104.2684099	105.1577068	118.8057925	74.109461	68.45986084	55.4990134	91.39059092	88.73195555	95.04621456	116.9799564	101.8073447	114.4203038	98.80975997	76.10343753	70.78616679	91.72292034	47.19077786	93.38456745	30.24197736	90.39360266	109.0040503	107.0100737	107.0371082	57.1606605	92.38757919	108.3393914	35.47616575	74.35870807	102.0251324
	77.18915757	88.76008668	95.40117417	101.1990831	99.22412273	81.95365779	78.2101219	57.79083525	94.54555123	86.03751512	109.5196948	109.1793733	98.84540117	111.0567515	93.58137895	81.61333635	73.44562168	93.52458689	48.60215626	98.28908711	34.54262659	97.60844423	105.7761589	104.7551946	105.1076321	67.31983569	98.62940856	98.28908711	26.71523337	88.99405767	92.50362256

AMP3CPR3ERY2FUS3DOX1	AMP	CPR	ERY	FUS	DOX	AMP+CPR	AMP+ERY	AMP+FUS	AMP+DOX	CPR+ERY	CPR+FUS	CPR+DOX	ERY+FUS	ERY+DOX	FUS+DOX	AMP+CPR+ERY	AMP+CPR+FUS	AMP+CPR+DOX	AMP+ERY+FUS	AMP+ERY+DOX	AMP+FUS+DOX	CPR+ERY+FUS	CPR+ERY+DOX	CPR+FUS+DOX	ERY+FUS+DOX	AMP+CPR+ERY+FUS	AMP+CPR+ERY+DOX	AMP+CPR+FUS+DOX	AMP+ERY+FUS+DOX	CPR+ERY+FUS+DOX	AMP+CPR+ERY+FUS+DOX

	75.22844656	92.70957489	92.2257522	103.6182389	108.9348348	74.91060787	74.59276917	87.30631704	96.84147795	91.12038141	118.4545093	104.4696067	102.0930667	101.7940572	79.69355732	70.77870481	85.39928486	97.15931665	81.9030592	102.2447358	70.14302741	110.8263806	109.5550258	103.8339293	79.06933283	79.99602702	90.48470401	65.69328566	54.56893127	93.98092968	67.60031784
	71.78315505	82.75002596	104.2684099	117.5362031	111.1882563	74.109461	68.45986084	82.75002596	89.72894382	88.73195555	110.0010385	105.3484266	117.4966353	111.9592386	70.24399921	76.10343753	82.41769654	91.72292034	80.09139059	98.36950878	83.74701423	106.3454149	105.3484266	109.6687091	74.73562776	80.42372001	90.39360266	92.38757919	83.08235538	104.3514384	80.42372001
	77.18915757	88.76008668	95.40117417	113.6131194	104.5847293	81.95365779	78.2101219	85.01655079	89.78105101	86.03751512	118.0277309	102.3729444	112.6223092	103.5420744	58.59636748	81.61333635	90.4616939	102.7132659	81.2730149	97.26812278	77.18915757	109.5196948	104.0745517	99.65037289	88.51272016	96.92780134	91.14233679	74.12626457	64.93758558	93.86490834	65.61822847

AMP3CPR3ERY2FUS3DOX2	AMP	CPR	ERY	FUS	DOX	AMP+CPR	AMP+ERY	AMP+FUS	AMP+DOX	CPR+ERY	CPR+FUS	CPR+DOX	ERY+FUS	ERY+DOX	FUS+DOX	AMP+CPR+ERY	AMP+CPR+FUS	AMP+CPR+DOX	AMP+ERY+FUS	AMP+ERY+DOX	AMP+FUS+DOX	CPR+ERY+FUS	CPR+ERY+DOX	CPR+FUS+DOX	ERY+FUS+DOX	AMP+CPR+ERY+FUS	AMP+CPR+ERY+DOX	AMP+CPR+FUS+DOX	AMP+ERY+FUS+DOX	CPR+ERY+FUS+DOX	AMP+CPR+ERY+FUS+DOX
	75.22844656	92.70957489	92.2257522	103.6182389	113.9560642	74.91060787	74.59276917	87.30631704	98.43067143	91.12038141	118.4545093	118.1366706	102.0930667	107.1762287	86.78235186	70.77870481	85.39928486	107.0123162	81.9030592	95.25228447	66.96464044	110.8263806	112.0977354	99.70202622	86.54457111	79.99602702	101.6090584	63.96503774	59.33651172	82.85657529	66.96464044
	71.78315505	82.75002596	104.2684099	117.5362031	121.6623686	74.109461	68.45986084	82.75002596	91.72292034	88.73195555	110.0010385	107.3424032	117.4966353	117.4966353	76.90934338	76.10343753	82.41769654	99.03416762	80.09139059	94.38155572	86.4056496	106.3454149	110.333368	110.6656974	78.11959239	80.42372001	94.0492263	82.08536712	78.09741406	99.69882646	86.07332018
	77.18915757	88.76008668	95.40117417	113.6131194	102.8919062	81.95365779	78.2101219	85.01655079	96.92780134	86.03751512	118.0277309	109.1793733	112.6223092	111.369863	76.93528478	81.61333635	90.4616939	96.92780134	81.2730149	106.7971232	69.7020858	109.5196948	102.032623	102.032623	78.18003914	96.92780134	98.28908711	77.86980046	69.36176435	90.4616939	73.44562168

AMP3CPR3ERY2FUS3DOX3	AMP	CPR	ERY	FUS	DOX	AMP+CPR	AMP+ERY	AMP+FUS	AMP+DOX	CPR+ERY	CPR+FUS	CPR+DOX	ERY+FUS	ERY+DOX	FUS+DOX	AMP+CPR+ERY	AMP+CPR+FUS	AMP+CPR+DOX	AMP+ERY+FUS	AMP+ERY+DOX	AMP+FUS+DOX	CPR+ERY+FUS	CPR+ERY+DOX	CPR+FUS+DOX	ERY+FUS+DOX	AMP+CPR+ERY+FUS	AMP+CPR+ERY+DOX	AMP+CPR+FUS+DOX	AMP+ERY+FUS+DOX	CPR+ERY+FUS+DOX	AMP+CPR+ERY+FUS+DOX
	75.22844656	92.70957489	92.2257522	103.6182389	107.753369	74.91060787	74.59276917	87.30631704	94.29876838	91.12038141	118.4545093	105.4231228	102.0930667	109.8673145	97.7109101	70.77870481	85.39928486	91.4382201	81.9030592	96.20580056	71.4143822	110.8263806	106.6944776	107.0123162	109.8673145	79.99602702	100.0198649	66.50774732	66.64680175	93.02741359	72.36789829
	71.78315505	82.75002596	104.2684099	117.5362031	118.8057925	74.109461	68.45986084	82.75002596	91.39059092	88.73195555	110.0010385	116.9799564	117.4966353	114.4203038	108.3316802	76.10343753	82.41769654	91.72292034	80.09139059	93.38456745	77.43275522	106.3454149	109.0040503	124.6235331	102.1149779	80.42372001	92.38757919	85.74099076	79.42673175	108.007062	88.73195555
	77.18915757	88.76008668	95.40117417	113.6131194	99.22412273	81.95365779	78.2101219	85.01655079	94.54555123	86.03751512	118.0277309	109.1793733	112.6223092	111.0567515	102.3276318	81.61333635	90.4616939	93.52458689	81.2730149	98.28908711	76.84883613	109.5196948	105.7761589	123.472874	108.2387476	96.92780134	98.62940856	84.67622934	77.52947902	117.347088	77.86980046

AMP3CPR3ERY3FUS1DOX1	AMP	CPR	ERY	FUS	DOX	AMP+CPR	AMP+ERY	AMP+FUS	AMP+DOX	CPR+ERY	CPR+FUS	CPR+DOX	ERY+FUS	ERY+DOX	FUS+DOX	AMP+CPR+ERY	AMP+CPR+FUS	AMP+CPR+DOX	AMP+ERY+FUS	AMP+ERY+DOX	AMP+FUS+DOX	CPR+ERY+FUS	CPR+ERY+DOX	CPR+FUS+DOX	ERY+FUS+DOX	AMP+CPR+ERY+FUS	AMP+CPR+ERY+DOX	AMP+CPR+FUS+DOX	AMP+ERY+FUS+DOX	CPR+ERY+FUS+DOX	AMP+CPR+ERY+FUS+DOX
	75.22844656	92.70957489	99.06634883	65.33136422	108.9348348	74.91060787	75.22844656	31.0488677	96.84147795	97.47715534	102.2447358	104.4696067	105.4231228	106.0588002	58.27949049	76.18196265	38.35915773	97.15931665	35.04171633	91.7560588	37.26658721	68.7326182	110.1907032	63.32936035	59.51529599	40.9018673	86.35280095	40.76281287	46.16607072	63.64719905	40.76281287
	71.78315505	82.75002596	82.75002596	86.92719698	111.1882563	74.109461	71.78315505	46.44303666	89.72894382	91.39059092	94.71388514	105.3484266	94.38155572	94.71388514	61.53540964	77.43275522	54.16969571	91.72292034	37.8024717	82.75002596	50.43098972	87.31955551	103.0221207	70.70308443	70.70308443	48.85242497	88.73195555	61.06553121	51.09564856	62.72717832	60.06854294
	77.18915757	88.76008668	91.48265823	63.3926997	104.5847293	81.95365779	74.80690746	43.0506627	89.78105101	90.4616939	94.54555123	102.3729444	95.906837	103.3939088	57.78522306	82.97462212	52.23934169	102.7132659	51.5586988	86.37783657	68.23444957	86.27148611	101.6923016	64.15059224	74.70055701	46.56022759	89.78105101	67.21348524	50.87805591	58.02480624	55.30223469

AMP3CPR3ERY3FUS1DOX2	AMP	CPR	ERY	FUS	DOX	AMP+CPR	AMP+ERY	AMP+FUS	AMP+DOX	CPR+ERY	CPR+FUS	CPR+DOX	ERY+FUS	ERY+DOX	FUS+DOX	AMP+CPR+ERY	AMP+CPR+FUS	AMP+CPR+DOX	AMP+ERY+FUS	AMP+ERY+DOX	AMP+FUS+DOX	CPR+ERY+FUS	CPR+ERY+DOX	CPR+FUS+DOX	ERY+FUS+DOX	AMP+CPR+ERY+FUS	AMP+CPR+ERY+DOX	AMP+CPR+FUS+DOX	AMP+ERY+FUS+DOX	CPR+ERY+FUS+DOX	AMP+CPR+ERY+FUS+DOX
	75.22844656	92.70957489	99.06634883	65.33136422	113.9560642	74.91060787	75.22844656	31.0488677	98.43067143	97.47715534	102.2447358	118.1366706	105.4231228	116.2296385	61.19623408	76.18196265	38.35915773	107.0123162	35.04171633	97.79499404	37.9022646	68.7326182	113.6869289	66.82558602	65.55423123	40.9018673	99.70202622	48.70878029	52.20500596	66.82558602	44.25903854
	71.78315505	82.75002596	82.75002596	86.92719698	121.6623686	74.109461	71.78315505	46.44303666	91.72292034	91.39059092	94.71388514	107.3424032	94.38155572	106.6777443	62.17020432	77.43275522	54.16969571	99.03416762	37.8024717	94.0492263	33.1498598	87.31955551	107.0100737	80.67296708	81.0052965	48.85242497	99.36649704	57.74223699	45.11371897	75.02336691	55.41593104
	77.18915757	88.76008668	91.48265823	63.3926997	102.8919062	81.95365779	74.80690746	43.0506627	96.92780134	90.4616939	94.54555123	109.1793733	95.906837	107.1374447	51.01393052	82.97462212	52.23934169	96.92780134	51.5586988	93.86490834	30.11844782	86.27148611	103.7342302	65.51187802	71.29734257	46.56022759	99.65037289	60.7473778	54.6215918	62.10866357	58.36512769

AMP3CPR3ERY3FUS1DOX3	AMP	CPR	ERY	FUS	DOX	AMP+CPR	AMP+ERY	AMP+FUS	AMP+DOX	CPR+ERY	CPR+FUS	CPR+DOX	ERY+FUS	ERY+DOX	FUS+DOX	AMP+CPR+ERY	AMP+CPR+FUS	AMP+CPR+DOX	AMP+ERY+FUS	AMP+ERY+DOX	AMP+FUS+DOX	CPR+ERY+FUS	CPR+ERY+DOX	CPR+FUS+DOX	ERY+FUS+DOX	AMP+CPR+ERY+FUS	AMP+CPR+ERY+DOX	AMP+CPR+FUS+DOX	AMP+ERY+FUS+DOX	CPR+ERY+FUS+DOX	AMP+CPR+ERY+FUS+DOX
	75.22844656	92.70957489	99.06634883	65.33136422	107.753369	74.91060787	75.22844656	31.0488677	94.29876838	97.47715534	102.2447358	105.4231228	105.4231228	104.151768	80.39505261	76.18196265	38.35915773	91.4382201	35.04171633	84.76360747	28.36710369	68.7326182	106.0588002	80.49264998	76.04290822	40.9018673	94.29876838	39.63051251	34.08820024	76.67858562	43.12673818
	71.78315505	82.75002596	82.75002596	86.92719698	118.8057925	74.109461	71.78315505	46.44303666	91.39059092	91.39059092	94.71388514	116.9799564	94.38155572	120.9679094	82.16623686	77.43275522	54.16969571	91.72292034	37.8024717	86.4056496	33.48218922	87.31955551	109.3363797	88.6488732	77.68200228	48.85242497	88.73195555	43.86748364	39.46411881	81.66995534	54.83435455
	77.18915757	88.76008668	91.48265823	63.3926997	99.22412273	81.95365779	74.80690746	43.0506627	94.54555123	90.4616939	94.54555123	109.1793733	95.906837	103.0535873	74.74872157	82.97462212	52.23934169	93.52458689	51.5586988	90.80201534	41.34905548	86.27148611	108.8390519	88.31341478	80.93269346	46.56022759	95.906837	50.53773447	52.57966313	82.52795023	52.57966313

AMP3CPR3ERY3FUS2DOX1	AMP	CPR	ERY	FUS	DOX	AMP+CPR	AMP+ERY	AMP+FUS	AMP+DOX	CPR+ERY	CPR+FUS	CPR+DOX	ERY+FUS	ERY+DOX	FUS+DOX	AMP+CPR+ERY	AMP+CPR+FUS	AMP+CPR+DOX	AMP+ERY+FUS	AMP+ERY+DOX	AMP+FUS+DOX	CPR+ERY+FUS	CPR+ERY+DOX	CPR+FUS+DOX	ERY+FUS+DOX	AMP+CPR+ERY+FUS	AMP+CPR+ERY+DOX	AMP+CPR+FUS+DOX	AMP+ERY+FUS+DOX	CPR+ERY+FUS+DOX	AMP+CPR+ERY+FUS+DOX
	75.22844656	92.70957489	99.06634883	92.3943142	108.9348348	74.91060787	75.22844656	52.34406039	96.84147795	97.47715534	102.2447358	104.4696067	110.1907032	106.0588002	74.19235739	76.18196265	71.7322209	97.15931665	48.84783472	91.7560588	53.15852205	102.2447358	110.1907032	79.53913389	75.40723083	61.8792213	86.35280095	60.15097338	53.15852205	76.99642431	51.25148987
	71.78315505	82.75002596	82.75002596	105.1577068	111.1882563	74.109461	71.78315505	55.4990134	89.72894382	91.39059092	95.04621456	105.3484266	105.3484266	94.71388514	87.24459433	77.43275522	70.78616679	91.72292034	50.18174265	82.75002596	45.11371897	96.04320282	103.0221207	83.99626129	80.00830824	68.12753142	88.73195555	60.73320179	55.74826046	86.32256724	50.76331914
	77.18915757	88.76008668	91.48265823	101.1990831	104.5847293	81.95365779	74.80690746	57.79083525	89.78105101	90.4616939	109.5196948	102.3729444	110.8809806	103.3939088	67.13101746	82.97462212	73.44562168	102.7132659	54.72794225	86.37783657	54.6215918	106.4568018	101.6923016	85.59084323	82.18762878	71.74401446	89.78105101	59.38609202	60.06673491	81.16666445	57.6844848

AMP3CPR3ERY3FUS2DOX2	AMP	CPR	ERY	FUS	DOX	AMP+CPR	AMP+ERY	AMP+FUS	AMP+DOX	CPR+ERY	CPR+FUS	CPR+DOX	ERY+FUS	ERY+DOX	FUS+DOX	AMP+CPR+ERY	AMP+CPR+FUS	AMP+CPR+DOX	AMP+ERY+FUS	AMP+ERY+DOX	AMP+FUS+DOX	CPR+ERY+FUS	CPR+ERY+DOX	CPR+FUS+DOX	ERY+FUS+DOX	AMP+CPR+ERY+FUS	AMP+CPR+ERY+DOX	AMP+CPR+FUS+DOX	AMP+ERY+FUS+DOX	CPR+ERY+FUS+DOX	AMP+CPR+ERY+FUS+DOX
	75.22844656	92.70957489	99.06634883	92.3943142	113.9560642	74.91060787	75.22844656	52.34406039	98.43067143	97.47715534	102.2447358	118.1366706	110.1907032	116.2296385	77.14602178	76.18196265	71.7322209	107.0123162	48.84783472	97.79499404	43.30552245	102.2447358	113.6869289	79.22129519	69.36829559	61.8792213	99.70202622	56.01907032	56.97258641	77.31426301	57.9261025
	71.78315505	82.75002596	82.75002596	105.1577068	121.6623686	74.109461	71.78315505	55.4990134	91.72292034	91.39059092	95.04621456	107.3424032	105.3484266	106.6777443	83.11842888	77.43275522	70.78616679	99.03416762	50.18174265	94.0492263	46.11070724	96.04320282	107.0100737	86.32256724	73.02939038	68.12753142	99.36649704	52.09263683	47.1076955	85.99023782	52.42496625
	77.18915757	88.76008668	91.48265823	101.1990831	102.8919062	81.95365779	74.80690746	57.79083525	96.92780134	90.4616939	109.5196948	109.1793733	110.8809806	107.1374447	64.30964557	82.97462212	73.44562168	96.92780134	54.72794225	93.86490834	51.89902025	106.4568018	103.7342302	93.41823644	75.72152134	71.74401446	99.65037289	49.17644869	59.72641346	86.952129	51.21837736

AMP3CPR3ERY3FUS2DOX3	AMP	CPR	ERY	FUS	DOX	AMP+CPR	AMP+ERY	AMP+FUS	AMP+DOX	CPR+ERY	CPR+FUS	CPR+DOX	ERY+FUS	ERY+DOX	FUS+DOX	AMP+CPR+ERY	AMP+CPR+FUS	AMP+CPR+DOX	AMP+ERY+FUS	AMP+ERY+DOX	AMP+FUS+DOX	CPR+ERY+FUS	CPR+ERY+DOX	CPR+FUS+DOX	ERY+FUS+DOX	AMP+CPR+ERY+FUS	AMP+CPR+ERY+DOX	AMP+CPR+FUS+DOX	AMP+ERY+FUS+DOX	CPR+ERY+FUS+DOX	AMP+CPR+ERY+FUS+DOX
	75.22844656	92.70957489	99.06634883	92.3943142	107.753369	74.91060787	75.22844656	52.34406039	94.29876838	97.47715534	102.2447358	105.4231228	110.1907032	104.151768	102.7321396	76.18196265	71.7322209	91.4382201	48.84783472	84.76360747	32.49900675	102.2447358	106.0588002	93.98092968	111.462058	61.8792213	94.29876838	87.30631704	31.54549066	103.5160906	79.04251093
	71.78315505	82.75002596	82.75002596	105.1577068	118.8057925	74.109461	71.78315505	55.4990134	91.39059092	91.39059092	95.04621456	116.9799564	105.3484266	120.9679094	98.80975997	77.43275522	70.78616679	91.72292034	50.18174265	86.4056496	30.24197736	96.04320282	109.3363797	107.0100737	118.9739329	68.12753142	88.73195555	108.3393914	30.49122443	110.6656974	101.3604736
	77.18915757	88.76008668	91.48265823	101.1990831	99.22412273	81.95365779	74.80690746	57.79083525	94.54555123	90.4616939	109.5196948	109.1793733	110.8809806	103.0535873	93.58137895	82.97462212	73.44562168	93.52458689	54.72794225	90.80201534	34.54262659	106.4568018	108.8390519	104.7551946	118.7083738	71.74401446	95.906837	98.28908711	33.8619837	106.1164803	91.48265823

AMP3CPR3ERY3FUS3DOX1	AMP	CPR	ERY	FUS	DOX	AMP+CPR	AMP+ERY	AMP+FUS	AMP+DOX	CPR+ERY	CPR+FUS	CPR+DOX	ERY+FUS	ERY+DOX	FUS+DOX	AMP+CPR+ERY	AMP+CPR+FUS	AMP+CPR+DOX	AMP+ERY+FUS	AMP+ERY+DOX	AMP+FUS+DOX	CPR+ERY+FUS	CPR+ERY+DOX	CPR+FUS+DOX	ERY+FUS+DOX	AMP+CPR+ERY+FUS	AMP+CPR+ERY+DOX	AMP+CPR+FUS+DOX	AMP+ERY+FUS+DOX	CPR+ERY+FUS+DOX	AMP+CPR+ERY+FUS+DOX
	75.22844656	92.70957489	99.06634883	103.6182389	108.9348348	74.91060787	75.22844656	87.30631704	96.84147795	97.47715534	118.4545093	104.4696067	116.8653159	106.0588002	79.69355732	76.18196265	85.39928486	97.15931665	79.99602702	91.7560588	70.14302741	111.462058	110.1907032	103.8339293	97.79499404	80.63170441	86.35280095	65.69328566	75.22844656	94.93444577	65.69328566
	71.78315505	82.75002596	82.75002596	117.5362031	111.1882563	74.109461	71.78315505	82.75002596	89.72894382	91.39059092	110.0010385	105.3484266	110.6656974	94.71388514	70.24399921	77.43275522	82.41769654	91.72292034	82.08536712	82.75002596	83.74701423	112.6596739	103.0221207	109.6687091	110.0010385	81.08837886	88.73195555	92.38757919	92.05524977	101.0281441	88.73195555
	77.18915757	88.76008668	91.48265823	113.6131194	104.5847293	81.95365779	74.80690746	85.01655079	89.78105101	90.4616939	118.0277309	102.3729444	124.4938383	103.3939088	58.59636748	82.97462212	90.4616939	102.7132659	86.03751512	86.37783657	77.18915757	115.3051593	101.6923016	99.65037289	98.96973	86.37783657	89.78105101	74.12626457	79.57140768	91.48265823	66.97951424

AMP3CPR3ERY3FUS3DOX2	AMP	CPR	ERY	FUS	DOX	AMP+CPR	AMP+ERY	AMP+FUS	AMP+DOX	CPR+ERY	CPR+FUS	CPR+DOX	ERY+FUS	ERY+DOX	FUS+DOX	AMP+CPR+ERY	AMP+CPR+FUS	AMP+CPR+DOX	AMP+ERY+FUS	AMP+ERY+DOX	AMP+FUS+DOX	CPR+ERY+FUS	CPR+ERY+DOX	CPR+FUS+DOX	ERY+FUS+DOX	AMP+CPR+ERY+FUS	AMP+CPR+ERY+DOX	AMP+CPR+FUS+DOX	AMP+ERY+FUS+DOX	CPR+ERY+FUS+DOX	AMP+CPR+ERY+FUS+DOX
	75.22844656	92.70957489	99.06634883	103.6182389	113.9560642	74.91060787	75.22844656	87.30631704	98.43067143	97.47715534	118.4545093	118.1366706	116.8653159	116.2296385	86.78235186	76.18196265	85.39928486	107.0123162	79.99602702	97.79499404	66.96464044	111.462058	113.6869289	99.70202622	92.70957489	80.63170441	99.70202622	63.96503774	72.05005959	100.973381	64.42193087
	71.78315505	82.75002596	82.75002596	117.5362031	121.6623686	74.109461	71.78315505	82.75002596	91.72292034	91.39059092	110.0010385	107.3424032	110.6656974	106.6777443	76.90934338	77.43275522	82.41769654	99.03416762	82.08536712	94.0492263	86.4056496	112.6596739	107.0100737	110.6656974	103.6867795	81.08837886	99.36649704	82.08536712	102.0251324	108.3393914	90.06127324
	77.18915757	88.76008668	91.48265823	113.6131194	102.8919062	81.95365779	74.80690746	85.01655079	96.92780134	90.4616939	118.0277309	109.1793733	124.4938383	107.1374447	76.93528478	82.97462212	90.4616939	96.92780134	86.03751512	93.86490834	69.7020858	115.3051593	103.7342302	102.032623	103.0535873	86.37783657	99.65037289	77.86980046	73.44562168	98.62940856	76.50851468

AMP3CPR3ERY3FUS3DOX3	AMP	CPR	ERY	FUS	DOX	AMP+CPR	AMP+ERY	AMP+FUS	AMP+DOX	CPR+ERY	CPR+FUS	CPR+DOX	ERY+FUS	ERY+DOX	FUS+DOX	AMP+CPR+ERY	AMP+CPR+FUS	AMP+CPR+DOX	AMP+ERY+FUS	AMP+ERY+DOX	AMP+FUS+DOX	CPR+ERY+FUS	CPR+ERY+DOX	CPR+FUS+DOX	ERY+FUS+DOX	AMP+CPR+ERY+FUS	AMP+CPR+ERY+DOX	AMP+CPR+FUS+DOX	AMP+ERY+FUS+DOX	CPR+ERY+FUS+DOX	AMP+CPR+ERY+FUS+DOX
	75.22844656	92.70957489	99.06634883	103.6182389	107.753369	74.91060787	75.22844656	87.30631704	94.29876838	97.47715534	118.4545093	105.4231228	116.8653159	104.151768	97.7109101	76.18196265	85.39928486	91.4382201	79.99602702	84.76360747	71.4143822	111.462058	106.0588002	107.0123162	114.0047676	80.63170441	94.29876838	66.50774732	67.28247914	100.6555423	65.05760826
	71.78315505	82.75002596	82.75002596	117.5362031	118.8057925	74.109461	71.78315505	82.75002596	91.39059092	91.39059092	110.0010385	116.9799564	110.6656974	120.9679094	108.3316802	77.43275522	82.41769654	91.72292034	82.08536712	86.4056496	77.43275522	112.6596739	109.3363797	124.6235331	116.3152975	81.08837886	88.73195555	85.74099076	84.07934365	112.3273445	87.73496729
	77.18915757	88.76008668	91.48265823	113.6131194	99.22412273	81.95365779	74.80690746	85.01655079	94.54555123	90.4616939	118.0277309	109.1793733	124.4938383	103.0535873	102.3276318	82.97462212	90.4616939	93.52458689	86.03751512	90.80201534	76.84883613	115.3051593	108.8390519	123.472874	124.8341598	86.37783657	95.906837	84.67622934	86.37783657	119.7293381	84.67622934

AMP1CPR1ERY1FUS1TMP1	AMP	CPR	ERY	FUS	TMP	AMP+CPR	AMP+ERY	AMP+FUS	AMP+TMP	CPR+ERY	CPR+FUS	CPR+TMP	ERY+FUS	ERY+TMP	FUS+TMP	AMP+CPR+ERY	AMP+CPR+FUS	AMP+CPR+TMP	AMP+ERY+FUS	AMP+ERY+TMP	AMP+FUS+TMP	CPR+ERY+FUS	CPR+ERY+TMP	CPR+FUS+TMP	ERY+FUS+TMP	AMP+CPR+ERY+FUS	AMP+CPR+ERY+TMP	AMP+CPR+FUS+TMP	AMP+ERY+FUS+TMP	CPR+ERY+FUS+TMP	AMP+CPR+ERY+FUS+TMP
	68.54228943	98.3646914	95.26991384	70.23034992	103.9915597	62.35273431	52.54088271	13.18797257	75.85721822	90.48707579	67.17074028	80.92139968	60.3833304	89.36170213	64.91999297	70.23034992	7.842447688	84.57886408	6.400562687	78.38930895	10.09319501	55.07297345	72.48109724	67.17074028	55.91700369	36.539476	65.16616845	21.3469316	57.92157552	55.07297345	26.97379989
	60.57180851	96.74202128	101.2632979	82.6462766	100.4654255	45.67819149	47.7393617	3.390957447	84.77393617	85.57180851	51.7287234	98.86968085	63.49734043	96.21010638	54.38829787	52.59308511	2.859042553	67.75265957	7.313829787	74.1356383	10.92087766	45.07978723	78.125	55.71808511	55.98404255	1.462765957	59.24202128	11.71875	6.848404255	49.33510638	5.06981383
	49.54289049	90.62439214	94.35907411	74.01283797	112.410037	60.1244894	51.09900798	2.120210076	98.40497958	106.808014	70.5893795	124.8589769	74.94650846	109.297802	71.21182649	52.34390196	3.053880568	89.69072165	3.053880568	87.51215717	5.23244505	59.69655709	84.0886987	68.41081502	72.45672048	8.169616806	84.71114569	2.742657071	3.365104065	47.2476172	3.501264345
	60.15533981	91.51456311	97.72815534	81.26213592	110.776699	47.08737864	51.74757282	5.475728155	94.93203883	100.5242718	85.00970874	113.5728155	76	112.6407767	72.89320388	42.73786408	2.368932039	90.89320388	3.300970874	89.65048544	1.747572816	75.06796117	95.24271845	69.16504854	71.33980583	10.17475728	75.98058252	6.757281553	-0.116504854	49.90291262	6.446601942

AMP1CPR1ERY1FUS1TMP2	AMP	CPR	ERY	FUS	TMP	AMP+CPR	AMP+ERY	AMP+FUS	AMP+TMP	CPR+ERY	CPR+FUS	CPR+TMP	ERY+FUS	ERY+TMP	FUS+TMP	AMP+CPR+ERY	AMP+CPR+FUS	AMP+CPR+TMP	AMP+ERY+FUS	AMP+ERY+TMP	AMP+FUS+TMP	CPR+ERY+FUS	CPR+ERY+TMP	CPR+FUS+TMP	ERY+FUS+TMP	AMP+CPR+ERY+FUS	AMP+CPR+ERY+TMP	AMP+CPR+FUS+TMP	AMP+ERY+FUS+TMP	CPR+ERY+FUS+TMP	AMP+CPR+ERY+FUS+TMP
	68.54228943	98.3646914	95.26991384	70.23034992	99.49006506	62.35273431	52.54088271	13.18797257	83.73483383	90.48707579	67.17074028	79.79602602	60.3833304	87.95498505	64.63864955	70.23034992	7.842447688	82.04677334	6.400562687	89.36170213	9.530508177	55.07297345	89.64304554	72.79760858	58.44909443	36.539476	69.38631968	3.340953051	7.561104273	54.54545455	3.587128539
	60.57180851	96.74202128	101.2632979	82.6462766	101.5292553	45.67819149	47.7393617	3.390957447	88.76329787	85.57180851	51.7287234	94.34840426	63.49734043	94.6143617	55.25265957	52.59308511	2.859042553	73.07180851	7.313829787	83.97606383	8.377659574	45.07978723	93.55053191	62.10106383	53.85638298	1.462765957	64.82712766	4.005984043	6.582446809	37.36702128	2.410239362
	49.54289049	90.62439214	94.35907411	74.01283797	109.297802	60.1244894	51.09900798	2.120210076	102.1396615	106.808014	70.5893795	110.2314725	74.94650846	109.920249	60.94145108	52.34390196	3.053880568	81.59891072	3.053880568	97.16008559	2.120210076	59.69655709	105.8743435	73.39039097	69.03326201	8.169616806	92.18050963	2.742657071	1.808986578	50.67107567	2.742657071
	60.15533981	91.51456311	97.72815534	81.26213592	115.4368932	47.08737864	51.74757282	5.475728155	103.631068	100.5242718	85.00970874	114.815534	76	119.1650485	72.58252427	42.73786408	2.368932039	86.54368932	3.300970874	91.2038835	2.058252427	75.06796117	107.6699029	72.27184466	65.74757282	10.17475728	84.99029126	30.67961165	1.747572816	47.00970874	-0.077669903

AMP1CPR1ERY1FUS1TMP3	AMP	CPR	ERY	FUS	TMP	AMP+CPR	AMP+ERY	AMP+FUS	AMP+TMP	CPR+ERY	CPR+FUS	CPR+TMP	ERY+FUS	ERY+TMP	FUS+TMP	AMP+CPR+ERY	AMP+CPR+FUS	AMP+CPR+TMP	AMP+ERY+FUS	AMP+ERY+TMP	AMP+FUS+TMP	CPR+ERY+FUS	CPR+ERY+TMP	CPR+FUS+TMP	ERY+FUS+TMP	AMP+CPR+ERY+FUS	AMP+CPR+ERY+TMP	AMP+CPR+FUS+TMP	AMP+ERY+FUS+TMP	CPR+ERY+FUS+TMP	AMP+CPR+ERY+FUS+TMP
	68.54228943	98.3646914	95.26991384	70.23034992	108.2117109	62.35273431	52.54088271	13.18797257	75.5758748	90.48707579	67.17074028	90.7684192	60.3833304	91.04976262	73.92298224	70.23034992	7.842447688	64.60348162	6.400562687	79.23333919	7.279760858	55.07297345	93.30050993	73.078952	67.4520837	36.539476	82.60946017	3.340953051	5.556532442	65.4826798	13.43414806
	60.57180851	96.74202128	101.2632979	82.6462766	100.4654255	45.67819149	47.7393617	3.390957447	76.26329787	85.57180851	51.7287234	98.07180851	63.49734043	94.88031915	59.50797872	52.59308511	2.859042553	63.23138298	7.313829787	80.25265957	15.97406915	45.07978723	91.95478723	69.28191489	51.99468085	1.462765957	60.03989362	2.144281915	2.32712766	45.61170213	0.748005319
	49.54289049	90.62439214	94.35907411	74.01283797	115.8334954	60.1244894	51.09900798	2.120210076	74.12954678	106.808014	70.5893795	111.4763665	74.94650846	108.3641315	68.41081502	52.34390196	3.053880568	74.12954678	3.053880568	73.81832328	1.186539584	59.69655709	107.7416845	78.68119043	70.278156	8.169616806	66.66018284	9.725734293	1.808986578	55.02820463	2.256370356
	60.15533981	91.51456311	97.72815534	81.26213592	108.2912621	47.08737864	51.74757282	5.475728155	82.19417476	100.5242718	85.00970874	117.9223301	76	111.7087379	74.75728155	42.73786408	2.368932039	92.13592233	3.300970874	64.48543689	4.54368932	75.06796117	116.368932	77.55339806	61.08737864	10.17475728	75.98058252	5.786407767	2.67961165	49.80582524	1.029126214

AMP1CPR1ERY1FUS2TMP1	AMP	CPR	ERY	FUS	TMP	AMP+CPR	AMP+ERY	AMP+FUS	AMP+TMP	CPR+ERY	CPR+FUS	CPR+TMP	ERY+FUS	ERY+TMP	FUS+TMP	AMP+CPR+ERY	AMP+CPR+FUS	AMP+CPR+TMP	AMP+ERY+FUS	AMP+ERY+TMP	AMP+FUS+TMP	CPR+ERY+FUS	CPR+ERY+TMP	CPR+FUS+TMP	ERY+FUS+TMP	AMP+CPR+ERY+FUS	AMP+CPR+ERY+TMP	AMP+CPR+FUS+TMP	AMP+ERY+FUS+TMP	CPR+ERY+FUS+TMP	AMP+CPR+ERY+FUS+TMP
	68.54228943	98.3646914	95.26991384	85.70423774	103.9915597	62.35273431	52.54088271	32.03798136	75.85721822	90.48707579	76.41990505	80.92139968	68.82363285	89.36170213	66.0101987	70.23034992	29.78723404	84.57886408	20.78424477	78.38930895	28.34534904	57.32372077	72.48109724	64.91999297	65.20133638	35.13275892	65.16616845	35.94162124	24.16036575	66.60805346	32.28415685
	60.57180851	96.74202128	101.2632979	95.94414894	100.4654255	45.67819149	47.7393617	24.48470745	84.77393617	85.57180851	77.59308511	98.86968085	67.48670213	96.21010638	62.96542553	52.59308511	23.42087766	67.75265957	15.82446809	74.1356383	34.85704787	50.13297872	78.125	50.13297872	44.88031915	11.30319149	59.24202128	26.19680851	31.78191489	47.20744681	34.97340426
	49.54289049	90.62439214	94.35907411	96.53763859	112.410037	60.1244894	51.09900798	11.76813849	98.40497958	106.808014	67.28262984	124.8589769	63.74246256	109.297802	67.47714452	52.34390196	26.39564287	89.69072165	6.788562536	87.51215717	34.4874538	77.74751994	84.0886987	61.87512157	63.74246256	8.480840303	84.71114569	32.62011282	1.808986578	72.45672048	50.04862867
	60.15533981	91.51456311	97.72815534	100.5242718	110.776699	47.08737864	51.74757282	8.893203883	94.93203883	100.5242718	74.13592233	113.5728155	71.33980583	112.6407767	66.36893204	42.73786408	22.87378641	90.89320388	25.04854369	89.65048544	39.33980583	79.41747573	95.24271845	63.26213592	62.01941748	12.66019417	75.98058252	51.45631068	2.058252427	65.4368932	35.9223301

AMP1CPR1ERY1FUS2TMP2	AMP	CPR	ERY	FUS	TMP	AMP+CPR	AMP+ERY	AMP+FUS	AMP+TMP	CPR+ERY	CPR+FUS	CPR+TMP	ERY+FUS	ERY+TMP	FUS+TMP	AMP+CPR+ERY	AMP+CPR+FUS	AMP+CPR+TMP	AMP+ERY+FUS	AMP+ERY+TMP	AMP+FUS+TMP	CPR+ERY+FUS	CPR+ERY+TMP	CPR+FUS+TMP	ERY+FUS+TMP	AMP+CPR+ERY+FUS	AMP+CPR+ERY+TMP	AMP+CPR+FUS+TMP	AMP+ERY+FUS+TMP	CPR+ERY+FUS+TMP	AMP+CPR+ERY+FUS+TMP
	68.54228943	98.3646914	95.26991384	85.70423774	99.49006506	62.35273431	52.54088271	32.03798136	83.73483383	90.48707579	76.41990505	79.79602602	68.82363285	87.95498505	67.17074028	70.23034992	29.78723404	82.04677334	20.78424477	89.36170213	27.78266221	57.32372077	89.64304554	71.95357834	69.70283102	35.13275892	69.38631968	39.56391771	29.18937929	64.07596272	26.93863197
	60.57180851	96.74202128	101.2632979	95.94414894	101.5292553	45.67819149	47.7393617	24.48470745	88.76329787	85.57180851	77.59308511	94.34840426	67.48670213	94.6143617	62.43351064	52.59308511	23.42087766	73.07180851	15.82446809	83.97606383	29.27194149	50.13297872	93.55053191	61.03723404	52.32712766	11.30319149	64.82712766	35.30585106	25.66489362	58.11170213	14.7606383
	49.54289049	90.62439214	94.35907411	96.53763859	109.297802	60.1244894	51.09900798	11.76813849	102.1396615	106.808014	67.28262984	110.2314725	63.74246256	109.920249	74.63528496	52.34390196	26.39564287	81.59891072	6.788562536	97.16008559	21.10484342	77.74751994	105.8743435	67.47714452	70.900603	8.480840303	92.18050963	31.37521883	22.03851391	87.70667185	27.32931336
	60.15533981	91.51456311	97.72815534	100.5242718	115.4368932	47.08737864	51.74757282	8.893203883	103.631068	100.5242718	74.13592233	114.815534	71.33980583	119.1650485	63.86407767	42.73786408	22.87378641	86.54368932	25.04854369	91.2038835	25.26213592	79.41747573	107.6699029	71.96116505	63.88349515	12.66019417	84.99029126	46.7961165	11.90291262	71.65048544	34.67961165

AMP1CPR1ERY1FUS2TMP3	AMP	CPR	ERY	FUS	TMP	AMP+CPR	AMP+ERY	AMP+FUS	AMP+TMP	CPR+ERY	CPR+FUS	CPR+TMP	ERY+FUS	ERY+TMP	FUS+TMP	AMP+CPR+ERY	AMP+CPR+FUS	AMP+CPR+TMP	AMP+ERY+FUS	AMP+ERY+TMP	AMP+FUS+TMP	CPR+ERY+FUS	CPR+ERY+TMP	CPR+FUS+TMP	ERY+FUS+TMP	AMP+CPR+ERY+FUS	AMP+CPR+ERY+TMP	AMP+CPR+FUS+TMP	AMP+ERY+FUS+TMP	CPR+ERY+FUS+TMP	AMP+CPR+ERY+FUS+TMP
	68.54228943	98.3646914	95.26991384	85.70423774	108.2117109	62.35273431	52.54088271	32.03798136	75.5758748	90.48707579	76.41990505	90.7684192	68.82363285	91.04976262	81.48408651	70.23034992	29.78723404	64.60348162	20.78424477	79.23333919	1.89906805	57.32372077	93.30050993	83.20731493	67.41691577	35.13275892	82.60946017	29.22454721	3.340953051	71.39089151	16.00140672
	60.57180851	96.74202128	101.2632979	95.94414894	100.4654255	45.67819149	47.7393617	24.48470745	76.26329787	85.57180851	77.59308511	98.07180851	67.48670213	94.88031915	78.65691489	52.59308511	23.42087766	63.23138298	15.82446809	80.25265957	14.91023936	50.13297872	91.95478723	65.55851064	60.57180851	11.30319149	60.03989362	21.02726064	7.197473404	52.2606383	6.931515957
	49.54289049	90.62439214	94.35907411	96.53763859	115.8334954	60.1244894	51.09900798	11.76813849	74.12954678	106.808014	67.28262984	111.4763665	63.74246256	108.3641315	83.4662517	52.34390196	26.39564287	74.12954678	6.788562536	73.81832328	9.589574013	77.74751994	107.7416845	66.54347403	62.30305388	8.480840303	66.66018284	9.414510796	0.564092589	59.0741101	3.676327563
	60.15533981	91.51456311	97.72815534	100.5242718	108.2912621	47.08737864	51.74757282	8.893203883	82.19417476	100.5242718	74.13592233	117.9223301	71.33980583	111.7087379	97.72815534	42.73786408	22.87378641	92.13592233	25.04854369	64.48543689	7.650485437	79.41747573	116.368932	81.59223301	58.27184466	12.66019417	75.98058252	4.27184466	1.436893204	75.68932039	3.339805825

AMP1CPR1ERY1FUS3TMP1	AMP	CPR	ERY	FUS	TMP	AMP+CPR	AMP+ERY	AMP+FUS	AMP+TMP	CPR+ERY	CPR+FUS	CPR+TMP	ERY+FUS	ERY+TMP	FUS+TMP	AMP+CPR+ERY	AMP+CPR+FUS	AMP+CPR+TMP	AMP+ERY+FUS	AMP+ERY+TMP	AMP+FUS+TMP	CPR+ERY+FUS	CPR+ERY+TMP	CPR+FUS+TMP	ERY+FUS+TMP	AMP+CPR+ERY+FUS	AMP+CPR+ERY+TMP	AMP+CPR+FUS+TMP	AMP+ERY+FUS+TMP	CPR+ERY+FUS+TMP	AMP+CPR+ERY+FUS+TMP
	68.54228943	98.3646914	95.26991384	98.64603482	103.9915597	62.35273431	52.54088271	46.66783893	75.85721822	90.48707579	84.86020749	80.92139968	88.23632847	89.36170213	75.01318797	70.23034992	50.60664674	84.57886408	36.46914014	78.38930895	57.85123967	85.98558115	72.48109724	60.10198699	68.26094602	31.96764551	65.16616845	34.49973624	53.91243186	59.29312467	53.06840162
	60.57180851	96.74202128	101.2632979	101.7952128	100.4654255	45.67819149	47.7393617	52.06117021	84.77393617	85.57180851	97.53989362	98.86968085	89.82712766	96.21010638	69.6143617	52.59308511	32.38031915	67.75265957	41.95478723	74.1356383	66.68882979	81.05053191	78.125	58.17819149	58.17819149	25.73138298	59.24202128	51.52925532	57.38031915	52.59308511	53.39095745
	49.54289049	90.62439214	94.35907411	112.410037	112.410037	60.1244894	51.09900798	34.91538611	98.40497958	106.808014	107.7416845	124.8589769	103.073332	109.297802	78.48667574	52.34390196	29.62458666	89.69072165	29.00213966	87.51215717	56.38980743	82.22135771	84.0886987	62.92550088	68.21630033	36.4715036	84.71114569	44.25209103	44.25209103	51.91596966	48.60922
	60.15533981	91.51456311	97.72815534	108.2912621	110.776699	47.08737864	51.74757282	36.83495146	94.93203883	100.5242718	107.0485437	113.5728155	100.2135922	112.6407767	83.12621359	42.73786408	31.86407767	90.89320388	43.04854369	89.65048544	49.26213592	82.50485437	95.24271845	75.04854369	66.97087379	31.24271845	75.98058252	41.18446602	43.04854369	61.39805825	57.96116505

AMP1CPR1ERY1FUS3TMP2	AMP	CPR	ERY	FUS	TMP	AMP+CPR	AMP+ERY	AMP+FUS	AMP+TMP	CPR+ERY	CPR+FUS	CPR+TMP	ERY+FUS	ERY+TMP	FUS+TMP	AMP+CPR+ERY	AMP+CPR+FUS	AMP+CPR+TMP	AMP+ERY+FUS	AMP+ERY+TMP	AMP+FUS+TMP	CPR+ERY+FUS	CPR+ERY+TMP	CPR+FUS+TMP	ERY+FUS+TMP	AMP+CPR+ERY+FUS	AMP+CPR+ERY+TMP	AMP+CPR+FUS+TMP	AMP+ERY+FUS+TMP	CPR+ERY+FUS+TMP	AMP+CPR+ERY+FUS+TMP
	68.54228943	98.3646914	95.26991384	98.64603482	99.49006506	62.35273431	52.54088271	46.66783893	83.73483383	90.48707579	84.86020749	79.79602602	88.23632847	87.95498505	86.26692456	70.23034992	50.60664674	82.04677334	36.46914014	89.36170213	42.13117637	85.98558115	89.64304554	77.54527871	83.172147	31.96764551	69.38631968	34.49973624	28.62669246	59.82064357	53.63108845
	60.57180851	96.74202128	101.2632979	101.7952128	101.5292553	45.67819149	47.7393617	52.06117021	88.76329787	85.57180851	97.53989362	94.34840426	89.82712766	94.6143617	82.6462766	52.59308511	32.38031915	73.07180851	41.95478723	83.97606383	52.85904255	81.05053191	93.55053191	76.52925532	77.06117021	25.73138298	64.82712766	54.18882979	36.6356383	53.92287234	51.52925532
	49.54289049	90.62439214	94.35907411	112.410037	109.297802	60.1244894	51.09900798	34.91538611	102.1396615	106.808014	107.7416845	110.2314725	103.073332	109.920249	95.2927446	52.34390196	29.62458666	81.59891072	29.00213966	97.16008559	45.49698502	82.22135771	105.8743435	83.4662517	80.66524023	36.4715036	92.18050963	41.13985606	38.33884458	75.88017895	41.45107956
	60.15533981	91.51456311	97.72815534	108.2912621	115.4368932	47.08737864	51.74757282	36.83495146	103.631068	100.5242718	107.0485437	114.815534	100.2135922	119.1650485	89.65048544	42.73786408	31.86407767	86.54368932	43.04854369	91.2038835	46.77669903	82.50485437	107.6699029	88.7184466	75.98058252	31.24271845	84.99029126	39.02912621	40.58252427	71.02912621	51.74757282

AMP1CPR1ERY1FUS3TMP3	AMP	CPR	ERY	FUS	TMP	AMP+CPR	AMP+ERY	AMP+FUS	AMP+TMP	CPR+ERY	CPR+FUS	CPR+TMP	ERY+FUS	ERY+TMP	FUS+TMP	AMP+CPR+ERY	AMP+CPR+FUS	AMP+CPR+TMP	AMP+ERY+FUS	AMP+ERY+TMP	AMP+FUS+TMP	CPR+ERY+FUS	CPR+ERY+TMP	CPR+FUS+TMP	ERY+FUS+TMP	AMP+CPR+ERY+FUS	AMP+CPR+ERY+TMP	AMP+CPR+FUS+TMP	AMP+ERY+FUS+TMP	CPR+ERY+FUS+TMP	AMP+CPR+ERY+FUS+TMP
	68.54228943	98.3646914	95.26991384	98.64603482	108.2117109	62.35273431	52.54088271	46.66783893	75.5758748	90.48707579	84.86020749	90.7684192	88.23632847	91.04976262	105.9609636	70.23034992	50.60664674	64.60348162	36.46914014	79.23333919	34.00738526	85.98558115	93.30050993	94.14454018	95.55125725	31.96764551	82.60946017	38.5088799	20.46773343	67.9796026	22.75364867
	60.57180851	96.74202128	101.2632979	101.7952128	100.4654255	45.67819149	47.7393617	52.06117021	76.26329787	85.57180851	97.53989362	98.07180851	89.82712766	94.88031915	105.2526596	52.59308511	32.38031915	63.23138298	41.95478723	80.25265957	37.25066489	81.05053191	91.95478723	94.88031915	97.00797872	25.73138298	60.03989362	23.07180851	39.29521277	71.47606383	17.75265957
	49.54289049	90.62439214	94.35907411	112.410037	115.8334954	60.1244894	51.09900798	34.91538611	74.12954678	106.808014	107.7416845	111.4763665	103.073332	108.3641315	109.920249	52.34390196	29.62458666	74.12954678	29.00213966	73.81832328	41.33437074	82.22135771	107.7416845	100.5835441	106.808014	36.4715036	66.66018284	45.38027621	22.03851391	61.99183038	24.52830189
	60.15533981	91.51456311	97.72815534	108.2912621	108.2912621	47.08737864	51.74757282	36.83495146	82.19417476	100.5242718	107.0485437	117.9223301	100.2135922	111.7087379	111.0873786	42.73786408	31.86407767	92.13592233	43.04854369	64.48543689	28.77669903	82.50485437	116.368932	107.9805825	105.184466	31.24271845	75.98058252	30.95145631	26.60194175	70.69902913	21.22330097

AMP1CPR1ERY2FUS1TMP1	AMP	CPR	ERY	FUS	TMP	AMP+CPR	AMP+ERY	AMP+FUS	AMP+TMP	CPR+ERY	CPR+FUS	CPR+TMP	ERY+FUS	ERY+TMP	FUS+TMP	AMP+CPR+ERY	AMP+CPR+FUS	AMP+CPR+TMP	AMP+ERY+FUS	AMP+ERY+TMP	AMP+FUS+TMP	CPR+ERY+FUS	CPR+ERY+TMP	CPR+FUS+TMP	ERY+FUS+TMP	AMP+CPR+ERY+FUS	AMP+CPR+ERY+TMP	AMP+CPR+FUS+TMP	AMP+ERY+FUS+TMP	CPR+ERY+FUS+TMP	AMP+CPR+ERY+FUS+TMP
	68.54228943	98.3646914	103.8353601	70.23034992	103.9915597	62.35273431	48.56690698	13.18797257	75.85721822	97.52066116	67.17074028	80.92139968	69.71000935	113.414406	64.91999297	69.94900651	7.842447688	84.57886408	5.591700369	82.89080359	10.09319501	57.8864076	82.32811676	67.17074028	52.66604303	4.184983295	80.64005627	21.3469316	8.123791103	62.95058906	10.65588184
	60.57180851	96.74202128	102.1828909	82.6462766	100.4654255	45.67819149	46.40957447	3.390957447	84.77393617	87.96542553	51.7287234	98.86968085	77.95476893	110.993117	54.38829787	49.66755319	2.859042553	67.75265957	8.377659574	71.74202128	10.92087766	48.00531915	86.6356383	55.71808511	72.86135693	8.178191489	59.24202128	11.71875	11.71875	52.52659574	5.06981383
	49.54289049	90.62439214	88.62788963	74.01283797	112.410037	60.1244894	57.01225443	2.120210076	98.40497958	106.185567	70.5893795	124.8589769	82.06562267	107.7181208	71.21182649	49.54289049	3.053880568	89.69072165	34.7986773	90.00194515	5.23244505	58.4516631	101.2059911	68.41081502	56.7114094	13.77163976	92.80295662	2.742657071	2.742657071	59.0741101	44.582766
	60.15533981	91.51456311	0	81.26213592	110.776699	47.08737864	52.05825243	5.475728155	94.93203883	98.03883495	85.00970874	113.5728155	0	0	72.89320388	43.3592233	2.368932039	90.89320388	4.233009709	91.2038835	1.747572816	65.12621359	114.1941748	69.16504854	0	48.38834951	94.62135922	6.757281553	2.990291262	63.26213592	5.825242718

AMP1CPR1ERY2FUS1TMP2	AMP	CPR	ERY	FUS	TMP	AMP+CPR	AMP+ERY	AMP+FUS	AMP+TMP	CPR+ERY	CPR+FUS	CPR+TMP	ERY+FUS	ERY+TMP	FUS+TMP	AMP+CPR+ERY	AMP+CPR+FUS	AMP+CPR+TMP	AMP+ERY+FUS	AMP+ERY+TMP	AMP+FUS+TMP	CPR+ERY+FUS	CPR+ERY+TMP	CPR+FUS+TMP	ERY+FUS+TMP	AMP+CPR+ERY+FUS	AMP+CPR+ERY+TMP	AMP+CPR+FUS+TMP	AMP+ERY+FUS+TMP	CPR+ERY+FUS+TMP	AMP+CPR+ERY+FUS+TMP
	68.54228943	98.3646914	103.8353601	70.23034992	99.49006506	62.35273431	48.56690698	13.18797257	83.73483383	97.52066116	67.17074028	79.79602602	69.71000935	120.2993452	64.63864955	69.94900651	7.842447688	82.04677334	5.591700369	82.04677334	9.530508177	57.8864076	86.26692456	72.79760858	62.84377923	4.184983295	73.32512748	3.340953051	9.811851591	59.01178126	5.02901354
	60.57180851	96.74202128	102.1828909	82.6462766	101.5292553	45.67819149	46.40957447	3.390957447	88.76329787	87.96542553	51.7287234	94.34840426	77.95476893	119.1740413	55.25265957	49.66755319	2.859042553	73.07180851	8.377659574	83.44414894	8.377659574	48.00531915	95.67819149	62.10106383	76.32251721	8.178191489	65.625	4.005984043	4.803856383	44.96343085	2.942154255
	49.54289049	90.62439214	88.62788963	74.01283797	109.297802	60.1244894	57.01225443	2.120210076	102.1396615	106.185567	70.5893795	110.2314725	82.06562267	112.7889635	60.94145108	49.54289049	3.053880568	81.59891072	34.7986773	100.8947676	2.120210076	58.4516631	102.1396615	73.39039097	63.87024609	13.77163976	97.16008559	2.742657071	2.120210076	54.09453414	7.235946314
	60.15533981	91.51456311	0	81.26213592	115.4368932	47.08737864	52.05825243	5.475728155	103.631068	98.03883495	85.00970874	114.815534	0	0	72.58252427	43.3592233	2.368932039	86.54368932	4.233009709	98.34951456	2.058252427	65.12621359	114.815534	72.27184466	0	48.38834951	94.62135922	30.67961165	1.126213592	48.5631068	5.514563107

AMP1CPR1ERY2FUS1TMP3	AMP	CPR	ERY	FUS	TMP	AMP+CPR	AMP+ERY	AMP+FUS	AMP+TMP	CPR+ERY	CPR+FUS	CPR+TMP	ERY+FUS	ERY+TMP	FUS+TMP	AMP+CPR+ERY	AMP+CPR+FUS	AMP+CPR+TMP	AMP+ERY+FUS	AMP+ERY+TMP	AMP+FUS+TMP	CPR+ERY+FUS	CPR+ERY+TMP	CPR+FUS+TMP	ERY+FUS+TMP	AMP+CPR+ERY+FUS	AMP+CPR+ERY+TMP	AMP+CPR+FUS+TMP	AMP+ERY+FUS+TMP	CPR+ERY+FUS+TMP	AMP+CPR+ERY+FUS+TMP
	68.54228943	98.3646914	103.8353601	70.23034992	108.2117109	62.35273431	48.56690698	13.18797257	75.5758748	97.52066116	67.17074028	90.7684192	69.71000935	121.7960711	73.92298224	69.94900651	7.842447688	64.60348162	5.591700369	79.79602602	7.279760858	57.8864076	90.48707579	73.078952	66.41721235	4.184983295	74.73184456	3.340953051	7.561104273	69.14014419	14.2781783
	60.57180851	96.74202128	102.1828909	82.6462766	100.4654255	45.67819149	46.40957447	3.390957447	76.26329787	87.96542553	51.7287234	98.07180851	77.95476893	124.5231072	59.50797872	49.66755319	2.859042553	63.23138298	8.377659574	80.78457447	15.97406915	48.00531915	98.07180851	69.28191489	78.89872173	8.178191489	64.29521277	2.144281915	7.04787234	50.66489362	1.811835106
	49.54289049	90.62439214	88.62788963	74.01283797	115.8334954	60.1244894	57.01225443	2.120210076	74.12954678	106.185567	70.5893795	111.4763665	82.06562267	112.7889635	68.41081502	49.54289049	3.053880568	74.12954678	34.7986773	93.11418012	1.186539584	58.4516631	105.8743435	78.68119043	80.87248322	13.77163976	75.68566427	9.725734293	3.676327563	49.73740517	0.700252869
	60.15533981	91.51456311	0	81.26213592	108.2912621	47.08737864	52.05825243	5.475728155	82.19417476	98.03883495	85.00970874	117.9223301	0	0	74.75728155	43.3592233	2.368932039	92.13592233	4.233009709	75.98058252	4.54368932	65.12621359	116.6796117	77.55339806	0	48.38834951	66.66019417	5.786407767	1.436893204	53.00970874	2.990291262

AMP1CPR1ERY2FUS2TMP1	AMP	CPR	ERY	FUS	TMP	AMP+CPR	AMP+ERY	AMP+FUS	AMP+TMP	CPR+ERY	CPR+FUS	CPR+TMP	ERY+FUS	ERY+TMP	FUS+TMP	AMP+CPR+ERY	AMP+CPR+FUS	AMP+CPR+TMP	AMP+ERY+FUS	AMP+ERY+TMP	AMP+FUS+TMP	CPR+ERY+FUS	CPR+ERY+TMP	CPR+FUS+TMP	ERY+FUS+TMP	AMP+CPR+ERY+FUS	AMP+CPR+ERY+TMP	AMP+CPR+FUS+TMP	AMP+ERY+FUS+TMP	CPR+ERY+FUS+TMP	AMP+CPR+ERY+FUS+TMP
	68.54228943	98.3646914	103.8353601	85.70423774	103.9915597	62.35273431	48.56690698	32.03798136	75.85721822	97.52066116	76.41990505	80.92139968	76.59494855	113.414406	66.0101987	69.94900651	29.78723404	84.57886408	25.56708282	82.89080359	28.34534904	57.28855284	82.32811676	64.91999297	58.3536015	21.90961843	80.64005627	35.94162124	36.50430807	71.10954809	28.66186038
	60.57180851	96.74202128	102.1828909	95.94414894	100.4654255	45.67819149	46.40957447	24.48470745	84.77393617	87.96542553	77.59308511	98.86968085	89.91150442	110.993117	62.96542553	49.66755319	23.42087766	67.75265957	18.48404255	71.74202128	34.85704787	65.35904255	86.6356383	50.13297872	66.56833825	11.30319149	59.24202128	26.19680851	25.66489362	54.65425532	23.80319149
	49.54289049	90.62439214	88.62788963	96.53763859	112.410037	60.1244894	57.01225443	11.76813849	98.40497958	106.185567	67.28262984	124.8589769	83.55704698	107.7181208	67.47714452	49.54289049	26.39564287	89.69072165	12.70180899	90.00194515	34.4874538	71.21182649	101.2059911	61.87512157	74.01193139	15.50282046	92.80295662	32.62011282	42.57926473	82.1046489	32.62011282
	60.15533981	91.51456311	0	100.5242718	110.776699	47.08737864	52.05825243	8.893203883	94.93203883	98.03883495	74.13592233	113.5728155	0	0	66.36893204	43.3592233	22.87378641	90.89320388	21.00970874	91.2038835	39.33980583	84.69902913	114.1941748	63.26213592	0	12.97087379	94.62135922	51.45631068	27.22330097	74.13592233	25.04854369

AMP1CPR1ERY2FUS2TMP2	AMP	CPR	ERY	FUS	TMP	AMP+CPR	AMP+ERY	AMP+FUS	AMP+TMP	CPR+ERY	CPR+FUS	CPR+TMP	ERY+FUS	ERY+TMP	FUS+TMP	AMP+CPR+ERY	AMP+CPR+FUS	AMP+CPR+TMP	AMP+ERY+FUS	AMP+ERY+TMP	AMP+FUS+TMP	CPR+ERY+FUS	CPR+ERY+TMP	CPR+FUS+TMP	ERY+FUS+TMP	AMP+CPR+ERY+FUS	AMP+CPR+ERY+TMP	AMP+CPR+FUS+TMP	AMP+ERY+FUS+TMP	CPR+ERY+FUS+TMP	AMP+CPR+ERY+FUS+TMP
	68.54228943	98.3646914	103.8353601	85.70423774	99.49006506	62.35273431	48.56690698	32.03798136	83.73483383	97.52066116	76.41990505	79.79602602	76.59494855	120.2993452	67.17074028	69.94900651	29.78723404	82.04677334	25.56708282	82.04677334	27.78266221	57.28855284	86.26692456	71.95357834	64.02245089	21.90961843	73.32512748	39.56391771	36.50430807	68.85880077	26.09460172
	60.57180851	96.74202128	102.1828909	95.94414894	101.5292553	45.67819149	46.40957447	24.48470745	88.76329787	87.96542553	77.59308511	94.34840426	89.91150442	119.1740413	62.43351064	49.66755319	23.42087766	73.07180851	18.48404255	83.44414894	29.27194149	65.35904255	95.67819149	61.03723404	71.03244838	11.30319149	65.625	35.30585106	24.66755319	60.7712766	16.77194149
	49.54289049	90.62439214	88.62788963	96.53763859	109.297802	60.1244894	57.01225443	11.76813849	102.1396615	106.185567	67.28262984	110.2314725	83.55704698	112.7889635	74.63528496	49.54289049	26.39564287	81.59891072	12.70180899	100.8947676	21.10484342	71.21182649	102.1396615	67.47714452	82.96047726	15.50282046	97.16008559	31.37521883	46.9363937	74.01283797	28.26298386
	60.15533981	91.51456311	0	100.5242718	115.4368932	47.08737864	52.05825243	8.893203883	103.631068	98.03883495	74.13592233	114.815534	0	0	63.86407767	43.3592233	22.87378641	86.54368932	21.00970874	98.34951456	25.26213592	84.69902913	114.815534	71.96116505	0	12.97087379	94.62135922	46.7961165	28.77669903	74.44660194	19.3592233

AMP1CPR1ERY2FUS2TMP3	AMP	CPR	ERY	FUS	TMP	AMP+CPR	AMP+ERY	AMP+FUS	AMP+TMP	CPR+ERY	CPR+FUS	CPR+TMP	ERY+FUS	ERY+TMP	FUS+TMP	AMP+CPR+ERY	AMP+CPR+FUS	AMP+CPR+TMP	AMP+ERY+FUS	AMP+ERY+TMP	AMP+FUS+TMP	CPR+ERY+FUS	CPR+ERY+TMP	CPR+FUS+TMP	ERY+FUS+TMP	AMP+CPR+ERY+FUS	AMP+CPR+ERY+TMP	AMP+CPR+FUS+TMP	AMP+ERY+FUS+TMP	CPR+ERY+FUS+TMP	AMP+CPR+ERY+FUS+TMP
	68.54228943	98.3646914	103.8353601	85.70423774	108.2117109	62.35273431	48.56690698	32.03798136	75.5758748	97.52066116	76.41990505	90.7684192	76.59494855	121.7960711	81.48408651	69.94900651	29.78723404	64.60348162	25.56708282	79.79602602	1.89906805	57.28855284	90.48707579	83.20731493	81.68381665	21.90961843	74.73184456	29.22454721	2.215579392	78.70582029	26.97379989
	60.57180851	96.74202128	102.1828909	95.94414894	100.4654255	45.67819149	46.40957447	24.48470745	76.26329787	87.96542553	77.59308511	98.07180851	89.91150442	124.5231072	78.65691489	49.66755319	23.42087766	63.23138298	18.48404255	80.78457447	14.91023936	65.35904255	98.07180851	65.55851064	94.3166175	11.30319149	64.29521277	21.02726064	0.997340426	56.78191489	14.64428191
	49.54289049	90.62439214	88.62788963	96.53763859	115.8334954	60.1244894	57.01225443	11.76813849	74.12954678	106.185567	67.28262984	111.4763665	83.55704698	112.7889635	83.4662517	49.54289049	26.39564287	74.12954678	12.70180899	93.11418012	9.589574013	71.21182649	105.8743435	66.54347403	91.90902312	15.50282046	75.68566427	9.414510796	7.722233029	70.900603	25.15074888
	60.15533981	91.51456311	0	100.5242718	108.2912621	47.08737864	52.05825243	8.893203883	82.19417476	98.03883495	74.13592233	117.9223301	0	0	97.72815534	43.3592233	22.87378641	92.13592233	21.00970874	75.98058252	7.650485437	84.69902913	116.6796117	81.59223301	0	12.97087379	66.66019417	4.27184466	5.786407767	73.51456311	24.11650485

AMP1CPR1ERY2FUS3TMP1	AMP	CPR	ERY	FUS	TMP	AMP+CPR	AMP+ERY	AMP+FUS	AMP+TMP	CPR+ERY	CPR+FUS	CPR+TMP	ERY+FUS	ERY+TMP	FUS+TMP	AMP+CPR+ERY	AMP+CPR+FUS	AMP+CPR+TMP	AMP+ERY+FUS	AMP+ERY+TMP	AMP+FUS+TMP	CPR+ERY+FUS	CPR+ERY+TMP	CPR+FUS+TMP	ERY+FUS+TMP	AMP+CPR+ERY+FUS	AMP+CPR+ERY+TMP	AMP+CPR+FUS+TMP	AMP+ERY+FUS+TMP	CPR+ERY+FUS+TMP	AMP+CPR+ERY+FUS+TMP
	68.54228943	98.3646914	103.8353601	98.64603482	103.9915597	62.35273431	48.56690698	46.66783893	75.85721822	97.52066116	84.86020749	80.92139968	106.828812	113.414406	75.01318797	69.94900651	50.60664674	84.57886408	35.97678917	82.89080359	57.85123967	82.89080359	82.32811676	60.10198699	85.8746492	31.19395112	80.64005627	34.49973624	38.15720063	58.44909443	57.56989625
	60.57180851	96.74202128	102.1828909	101.7952128	100.4654255	45.67819149	46.40957447	52.06117021	84.77393617	87.96542553	97.53989362	98.86968085	105.6440511	110.993117	69.6143617	49.66755319	32.38031915	67.75265957	46.47606383	71.74202128	66.68882979	88.49734043	86.6356383	58.17819149	72.92035398	29.18882979	59.24202128	51.52925532	54.45478723	51.79521277	54.45478723
	49.54289049	90.62439214	88.62788963	112.410037	112.410037	60.1244894	57.01225443	34.91538611	98.40497958	106.185567	107.7416845	124.8589769	102.945563	107.7181208	78.48667574	49.54289049	29.62458666	89.69072165	34.29293912	90.00194515	56.38980743	91.86928613	101.2059911	62.92550088	77.59134974	32.62011282	92.80295662	44.25209103	45.80820852	59.38533359	51.72145497
	60.15533981	91.51456311	0	108.2912621	110.776699	47.08737864	52.05825243	36.83495146	94.93203883	98.03883495	107.0485437	113.5728155	0	0	83.12621359	43.3592233	31.86407767	90.89320388	34.34951456	91.2038835	49.26213592	94.93203883	114.1941748	75.04854369	0	32.40776699	94.62135922	41.18446602	44.60194175	61.68932039	44.60194175

AMP1CPR1ERY2FUS3TMP2	AMP	CPR	ERY	FUS	TMP	AMP+CPR	AMP+ERY	AMP+FUS	AMP+TMP	CPR+ERY	CPR+FUS	CPR+TMP	ERY+FUS	ERY+TMP	FUS+TMP	AMP+CPR+ERY	AMP+CPR+FUS	AMP+CPR+TMP	AMP+ERY+FUS	AMP+ERY+TMP	AMP+FUS+TMP	CPR+ERY+FUS	CPR+ERY+TMP	CPR+FUS+TMP	ERY+FUS+TMP	AMP+CPR+ERY+FUS	AMP+CPR+ERY+TMP	AMP+CPR+FUS+TMP	AMP+ERY+FUS+TMP	CPR+ERY+FUS+TMP	AMP+CPR+ERY+FUS+TMP
	68.54228943	98.3646914	103.8353601	98.64603482	99.49006506	62.35273431	48.56690698	46.66783893	83.73483383	97.52066116	84.86020749	79.79602602	106.828812	120.2993452	86.26692456	69.94900651	50.60664674	82.04677334	35.97678917	82.04677334	42.13117637	82.89080359	86.26692456	77.54527871	93.95696913	31.19395112	73.32512748	34.49973624	34.53490417	65.72885528	37.03182697
	60.57180851	96.74202128	102.1828909	101.7952128	101.5292553	45.67819149	46.40957447	52.06117021	88.76329787	87.96542553	97.53989362	94.34840426	105.6440511	119.1740413	82.6462766	49.66755319	32.38031915	73.07180851	46.47606383	83.44414894	52.85904255	88.49734043	95.67819149	76.52925532	89.28220256	29.18882979	65.625	54.18882979	47.27393617	65.625	37.69946809
	49.54289049	90.62439214	88.62788963	112.410037	109.297802	60.1244894	57.01225443	34.91538611	102.1396615	106.185567	107.7416845	110.2314725	102.945563	112.7889635	95.2927446	49.54289049	29.62458666	81.59891072	34.29293912	100.8947676	45.49698502	91.86928613	102.1396615	83.4662517	99.66442953	32.62011282	97.16008559	41.13985606	34.29293912	75.68566427	38.84458277
	60.15533981	91.51456311	0	108.2912621	115.4368932	47.08737864	52.05825243	36.83495146	103.631068	98.03883495	107.0485437	114.815534	0	0	89.65048544	43.3592233	31.86407767	86.54368932	34.34951456	98.34951456	46.77669903	94.93203883	114.815534	88.7184466	0	32.40776699	92.13592233	39.02912621	37.47572816	73.18446602	34.34951456

AMP1CPR1ERY2FUS3TMP3	AMP	CPR	ERY	FUS	TMP	AMP+CPR	AMP+ERY	AMP+FUS	AMP+TMP	CPR+ERY	CPR+FUS	CPR+TMP	ERY+FUS	ERY+TMP	FUS+TMP	AMP+CPR+ERY	AMP+CPR+FUS	AMP+CPR+TMP	AMP+ERY+FUS	AMP+ERY+TMP	AMP+FUS+TMP	CPR+ERY+FUS	CPR+ERY+TMP	CPR+FUS+TMP	ERY+FUS+TMP	AMP+CPR+ERY+FUS	AMP+CPR+ERY+TMP	AMP+CPR+FUS+TMP	AMP+ERY+FUS+TMP	CPR+ERY+FUS+TMP	AMP+CPR+ERY+FUS+TMP
	68.54228943	98.3646914	103.8353601	98.64603482	108.2117109	62.35273431	48.56690698	46.66783893	75.5758748	97.52066116	84.86020749	90.7684192	106.828812	121.7960711	105.9609636	69.94900651	50.60664674	64.60348162	35.97678917	79.79602602	34.00738526	82.89080359	90.48707579	94.14454018	119.1019645	31.19395112	74.73184456	38.5088799	22.47230526	73.6064709	26.69245648
	60.57180851	96.74202128	102.1828909	101.7952128	100.4654255	45.67819149	46.40957447	52.06117021	76.26329787	87.96542553	97.53989362	98.07180851	105.6440511	124.5231072	105.2526596	49.66755319	32.38031915	63.23138298	46.47606383	80.78457447	37.25066489	88.49734043	98.07180851	94.88031915	109.4198623	29.18882979	64.29521277	23.07180851	28.47406915	77.06117021	22.00797872
	49.54289049	90.62439214	88.62788963	112.410037	115.8334954	60.1244894	57.01225443	34.91538611	74.12954678	106.185567	107.7416845	111.4763665	102.945563	112.7889635	109.920249	49.54289049	29.62458666	74.12954678	34.29293912	93.11418012	41.33437074	91.86928613	105.8743435	100.5835441	111.8941089	32.62011282	75.68566427	45.38027621	27.32931336	64.79284186	26.70686637
	60.15533981	91.51456311	0	108.2912621	108.2912621	47.08737864	52.05825243	36.83495146	82.19417476	98.03883495	107.0485437	117.9223301	0	0	111.0873786	43.3592233	31.86407767	92.13592233	34.34951456	75.98058252	28.77669903	94.93203883	116.6796117	107.9805825	0	32.40776699	66.66019417	30.95145631	17.59223301	75.04854369	24.73786408

AMP1CPR1ERY3FUS1TMP1	AMP	CPR	ERY	FUS	TMP	AMP+CPR	AMP+ERY	AMP+FUS	AMP+TMP	CPR+ERY	CPR+FUS	CPR+TMP	ERY+FUS	ERY+TMP	FUS+TMP	AMP+CPR+ERY	AMP+CPR+FUS	AMP+CPR+TMP	AMP+ERY+FUS	AMP+ERY+TMP	AMP+FUS+TMP	CPR+ERY+FUS	CPR+ERY+TMP	CPR+FUS+TMP	ERY+FUS+TMP	AMP+CPR+ERY+FUS	AMP+CPR+ERY+TMP	AMP+CPR+FUS+TMP	AMP+ERY+FUS+TMP	CPR+ERY+FUS+TMP	AMP+CPR+ERY+FUS+TMP
	68.54228943	98.3646914	95.34686971	70.23034992	103.9915597	62.35273431	54.47511869	13.18797257	75.85721822	99.20872165	67.17074028	80.92139968	80.79526227	114.6362098	64.91999297	64.88482504	7.842447688	84.57886408	4.184983295	76.13856163	10.09319501	60.98118516	86.54826798	67.17074028	60.08883249	13.18797257	84.57886408	21.3469316	24.44170916	64.91999297	7.842447688
	60.57180851	96.74202128	99.12713472	82.6462766	100.4654255	45.67819149	50.73138298	3.390957447	84.77393617	96.74202128	51.7287234	98.86968085	90.32258065	114.914611	54.38829787	41.42287234	2.859042553	67.75265957	8.97606383	74.1356383	10.92087766	54.65425532	97.27393617	55.71808511	56.31878558	2.526595745	62.16755319	11.71875	20.49534574	55.98404255	9.059175532
	49.54289049	90.62439214	108.9759282	74.01283797	112.410037	60.1244894	53.78331064	2.120210076	98.40497958	98.71620307	70.5893795	124.8589769	93.63525092	120.3998368	71.21182649	50.78778448	3.053880568	89.69072165	2.742657071	110.2314725	5.23244505	80.85975491	110.8539195	68.41081502	62.95389637	1.808986578	85.02236919	2.742657071	4.921221552	64.98735655	3.053880568
	60.15533981	91.51456311	0	81.26213592	110.776699	47.08737864	53.9223301	5.475728155	94.93203883	100.2135922	85.00970874	113.5728155	0	0	72.89320388	44.29126214	2.368932039	90.89320388	3.300970874	100.2135922	1.747572816	83.14563107	110.4660194	69.16504854	0	7.067961165	99.2815534	6.757281553	4.854368932	70.09708738	12.03883495

AMP1CPR1ERY3FUS1TMP2	AMP	CPR	ERY	FUS	TMP	AMP+CPR	AMP+ERY	AMP+FUS	AMP+TMP	CPR+ERY	CPR+FUS	CPR+TMP	ERY+FUS	ERY+TMP	FUS+TMP	AMP+CPR+ERY	AMP+CPR+FUS	AMP+CPR+TMP	AMP+ERY+FUS	AMP+ERY+TMP	AMP+FUS+TMP	CPR+ERY+FUS	CPR+ERY+TMP	CPR+FUS+TMP	ERY+FUS+TMP	AMP+CPR+ERY+FUS	AMP+CPR+ERY+TMP	AMP+CPR+FUS+TMP	AMP+ERY+FUS+TMP	CPR+ERY+FUS+TMP	AMP+CPR+ERY+FUS+TMP
	68.54228943	98.3646914	95.34686971	70.23034992	99.49006506	62.35273431	54.47511869	13.18797257	83.73483383	99.20872165	67.17074028	79.79602602	80.79526227	114.9746193	64.63864955	64.88482504	7.842447688	82.04677334	4.184983295	85.98558115	9.530508177	60.98118516	87.11095481	72.79760858	72.2715736	13.18797257	79.23333919	3.340953051	7.561104273	66.88939687	2.496922806
	60.57180851	96.74202128	99.12713472	82.6462766	101.5292553	45.67819149	50.73138298	3.390957447	88.76329787	96.74202128	51.7287234	94.34840426	90.32258065	115.2182163	55.25265957	41.42287234	2.859042553	73.07180851	8.97606383	84.24202128	8.377659574	54.65425532	101.5292553	62.10106383	65.73055028	2.526595745	67.48670213	4.005984043	6.931515957	56.78191489	4.454787234
	49.54289049	90.62439214	108.9759282	74.01283797	109.297802	60.1244894	53.78331064	2.120210076	102.1396615	98.71620307	70.5893795	110.2314725	93.63525092	125.2957976	60.94145108	50.78778448	3.053880568	81.59891072	2.742657071	109.297802	2.120210076	80.85975491	109.6090255	73.39039097	80.57935537	1.808986578	96.22641509	2.742657071	2.120210076	79.92608442	8.792063801
	60.15533981	91.51456311	0	81.26213592	115.4368932	47.08737864	53.9223301	5.475728155	103.631068	100.2135922	85.00970874	114.815534	0	0	72.58252427	44.29126214	2.368932039	86.54368932	3.300970874	107.3592233	2.058252427	83.14563107	111.3980583	72.27184466	0	7.067961165	92.13592233	30.67961165	1.72815534	71.65048544	6.757281553

AMP1CPR1ERY3FUS1TMP3	AMP	CPR	ERY	FUS	TMP	AMP+CPR	AMP+ERY	AMP+FUS	AMP+TMP	CPR+ERY	CPR+FUS	CPR+TMP	ERY+FUS	ERY+TMP	FUS+TMP	AMP+CPR+ERY	AMP+CPR+FUS	AMP+CPR+TMP	AMP+ERY+FUS	AMP+ERY+TMP	AMP+FUS+TMP	CPR+ERY+FUS	CPR+ERY+TMP	CPR+FUS+TMP	ERY+FUS+TMP	AMP+CPR+ERY+FUS	AMP+CPR+ERY+TMP	AMP+CPR+FUS+TMP	AMP+ERY+FUS+TMP	CPR+ERY+FUS+TMP	AMP+CPR+ERY+FUS+TMP
	68.54228943	98.3646914	95.34686971	70.23034992	108.2117109	62.35273431	54.47511869	13.18797257	75.5758748	99.20872165	67.17074028	90.7684192	80.79526227	115.6514382	73.92298224	64.88482504	7.842447688	64.60348162	4.184983295	76.13856163	7.279760858	60.98118516	88.51767188	73.078952	83.84094755	13.18797257	66.0101987	3.340953051	8.123791103	75.32969931	9.49534025
	60.57180851	96.74202128	99.12713472	82.6462766	100.4654255	45.67819149	50.73138298	3.390957447	76.26329787	96.74202128	51.7287234	98.07180851	90.32258065	107.0208729	59.50797872	41.42287234	2.859042553	63.23138298	8.97606383	79.45478723	15.97406915	54.65425532	103.3909574	69.28191489	81.82163188	2.526595745	62.69946809	2.144281915	12.10106383	66.35638298	8.527260638
	49.54289049	90.62439214	108.9759282	74.01283797	115.8334954	60.1244894	53.78331064	2.120210076	74.12954678	98.71620307	70.5893795	111.4763665	93.63525092	117.1358629	68.41081502	50.78778448	3.053880568	74.12954678	2.742657071	98.40497958	1.186539584	80.85975491	108.052908	78.68119043	90.04487964	1.808986578	66.34895935	9.725734293	2.431433573	86.46177786	7.235946314
	60.15533981	91.51456311	0	81.26213592	108.2912621	47.08737864	53.9223301	5.475728155	82.19417476	100.2135922	85.00970874	117.9223301	0	0	74.75728155	44.29126214	2.368932039	92.13592233	3.300970874	82.19417476	4.54368932	83.14563107	112.0194175	77.55339806	0	7.067961165	58.89320388	5.786407767	2.349514563	71.96116505	5.203883495

AMP1CPR1ERY3FUS2TMP1	AMP	CPR	ERY	FUS	TMP	AMP+CPR	AMP+ERY	AMP+FUS	AMP+TMP	CPR+ERY	CPR+FUS	CPR+TMP	ERY+FUS	ERY+TMP	FUS+TMP	AMP+CPR+ERY	AMP+CPR+FUS	AMP+CPR+TMP	AMP+ERY+FUS	AMP+ERY+TMP	AMP+FUS+TMP	CPR+ERY+FUS	CPR+ERY+TMP	CPR+FUS+TMP	ERY+FUS+TMP	AMP+CPR+ERY+FUS	AMP+CPR+ERY+TMP	AMP+CPR+FUS+TMP	AMP+ERY+FUS+TMP	CPR+ERY+FUS+TMP	AMP+CPR+ERY+FUS+TMP
	68.54228943	98.3646914	95.34686971	85.70423774	103.9915597	62.35273431	54.47511869	32.03798136	75.85721822	99.20872165	76.41990505	80.92139968	84.17935702	114.6362098	66.0101987	64.88482504	29.78723404	84.57886408	26.41111306	76.13856163	28.34534904	72.48109724	86.54826798	64.91999297	69.2893401	32.31932478	84.57886408	35.94162124	22.47230526	63.79461931	32.84684368
	60.57180851	96.74202128	99.12713472	95.94414894	100.4654255	45.67819149	50.73138298	24.48470745	84.77393617	96.74202128	77.59308511	98.86968085	93.05502846	114.914611	62.96542553	41.42287234	23.42087766	67.75265957	15.7081117	74.1356383	34.85704787	69.08244681	97.27393617	50.13297872	74.23149905	17.03789894	62.16755319	26.19680851	27.14428191	43.21808511	20.61170213
	49.54289049	90.62439214	108.9759282	96.53763859	112.410037	60.1244894	53.78331064	11.76813849	98.40497958	98.71620307	67.28262984	124.8589769	103.75357	120.3998368	67.47714452	50.78778448	26.39564287	89.69072165	6.788562536	110.2314725	34.4874538	71.21182649	110.8539195	61.87512157	78.29457364	9.278350515	85.02236919	32.62011282	31.37521883	71.21182649	28.88543085
	60.15533981	91.51456311	0	100.5242718	110.776699	47.08737864	53.9223301	8.893203883	94.93203883	100.2135922	74.13592233	113.5728155	0	0	66.36893204	44.29126214	22.87378641	90.89320388	21.94174757	100.2135922	39.33980583	73.51456311	110.4660194	63.26213592	0	15.32038835	99.2815534	51.45631068	19.66990291	61.39805825	21.00970874

AMP1CPR1ERY3FUS2TMP2	AMP	CPR	ERY	FUS	TMP	AMP+CPR	AMP+ERY	AMP+FUS	AMP+TMP	CPR+ERY	CPR+FUS	CPR+TMP	ERY+FUS	ERY+TMP	FUS+TMP	AMP+CPR+ERY	AMP+CPR+FUS	AMP+CPR+TMP	AMP+ERY+FUS	AMP+ERY+TMP	AMP+FUS+TMP	CPR+ERY+FUS	CPR+ERY+TMP	CPR+FUS+TMP	ERY+FUS+TMP	AMP+CPR+ERY+FUS	AMP+CPR+ERY+TMP	AMP+CPR+FUS+TMP	AMP+ERY+FUS+TMP	CPR+ERY+FUS+TMP	AMP+CPR+ERY+FUS+TMP
	68.54228943	98.3646914	95.34686971	85.70423774	99.49006506	62.35273431	54.47511869	32.03798136	83.73483383	99.20872165	76.41990505	79.79602602	84.17935702	114.9746193	67.17074028	64.88482504	29.78723404	82.04677334	26.41111306	85.98558115	27.78266221	72.48109724	87.11095481	71.95357834	85.8714044	32.31932478	79.23333919	39.56391771	36.78565149	53.34974503	25.28573941
	60.57180851	96.74202128	99.12713472	95.94414894	101.5292553	45.67819149	50.73138298	24.48470745	88.76329787	96.74202128	77.59308511	94.34840426	93.05502846	115.2182163	62.43351064	41.42287234	23.42087766	73.07180851	15.7081117	84.24202128	29.27194149	69.08244681	101.5292553	61.03723404	88.19734345	17.03789894	67.48670213	35.30585106	25.81449468	60.50531915	19.6974734
	49.54289049	90.62439214	108.9759282	96.53763859	109.297802	60.1244894	53.78331064	11.76813849	102.1396615	98.71620307	67.28262984	110.2314725	103.75357	125.2957976	74.63528496	50.78778448	26.39564287	81.59891072	6.788562536	109.297802	21.10484342	71.21182649	109.6090255	67.47714452	85.14891881	9.278350515	96.22641509	31.37521883	41.02314725	73.39039097	31.68644233
	60.15533981	91.51456311	0	100.5242718	115.4368932	47.08737864	53.9223301	8.893203883	103.631068	100.2135922	74.13592233	114.815534	0	0	63.86407767	44.29126214	22.87378641	86.54368932	21.94174757	107.3592233	25.26213592	73.51456311	111.3980583	71.96116505	0	15.32038835	92.13592233	46.7961165	24.01941748	68.23300971	23.49514563

AMP1CPR1ERY3FUS2TMP3	AMP	CPR	ERY	FUS	TMP	AMP+CPR	AMP+ERY	AMP+FUS	AMP+TMP	CPR+ERY	CPR+FUS	CPR+TMP	ERY+FUS	ERY+TMP	FUS+TMP	AMP+CPR+ERY	AMP+CPR+FUS	AMP+CPR+TMP	AMP+ERY+FUS	AMP+ERY+TMP	AMP+FUS+TMP	CPR+ERY+FUS	CPR+ERY+TMP	CPR+FUS+TMP	ERY+FUS+TMP	AMP+CPR+ERY+FUS	AMP+CPR+ERY+TMP	AMP+CPR+FUS+TMP	AMP+ERY+FUS+TMP	CPR+ERY+FUS+TMP	AMP+CPR+ERY+FUS+TMP
	68.54228943	98.3646914	95.34686971	85.70423774	108.2117109	62.35273431	54.47511869	32.03798136	75.5758748	99.20872165	76.41990505	90.7684192	84.17935702	115.6514382	81.48408651	64.88482504	29.78723404	64.60348162	26.41111306	76.13856163	1.89906805	72.48109724	88.51767188	83.20731493	98.39255499	32.31932478	66.0101987	29.22454721	3.305785124	73.36029541	19.65887111
	60.57180851	96.74202128	99.12713472	95.94414894	100.4654255	45.67819149	50.73138298	24.48470745	76.26329787	96.74202128	77.59308511	98.07180851	93.05502846	107.0208729	78.65691489	41.42287234	23.42087766	63.23138298	15.7081117	79.45478723	14.91023936	69.08244681	103.3909574	65.55851064	93.9658444	17.03789894	62.69946809	21.02726064	6.399601064	59.70744681	14.64428191
	49.54289049	90.62439214	108.9759282	96.53763859	115.8334954	60.1244894	53.78331064	11.76813849	74.12954678	98.71620307	67.28262984	111.4763665	103.75357	117.1358629	83.4662517	50.78778448	26.39564287	74.12954678	6.788562536	98.40497958	9.589574013	71.21182649	108.052908	66.54347403	92.65605875	9.278350515	66.34895935	9.414510796	6.166115542	71.21182649	8.169616806
	60.15533981	91.51456311	0	100.5242718	108.2912621	47.08737864	53.9223301	8.893203883	82.19417476	100.2135922	74.13592233	117.9223301	0	0	97.72815534	44.29126214	22.87378641	92.13592233	21.94174757	82.19417476	7.650485437	73.51456311	112.0194175	81.59223301	0	15.32038835	58.89320388	4.27184466	12	77.55339806	7.378640777

AMP1CPR1ERY3FUS3TMP1	AMP	CPR	ERY	FUS	TMP	AMP+CPR	AMP+ERY	AMP+FUS	AMP+TMP	CPR+ERY	CPR+FUS	CPR+TMP	ERY+FUS	ERY+TMP	FUS+TMP	AMP+CPR+ERY	AMP+CPR+FUS	AMP+CPR+TMP	AMP+ERY+FUS	AMP+ERY+TMP	AMP+FUS+TMP	CPR+ERY+FUS	CPR+ERY+TMP	CPR+FUS+TMP	ERY+FUS+TMP	AMP+CPR+ERY+FUS	AMP+CPR+ERY+TMP	AMP+CPR+FUS+TMP	AMP+ERY+FUS+TMP	CPR+ERY+FUS+TMP	AMP+CPR+ERY+FUS+TMP
	68.54228943	98.3646914	95.34686971	98.64603482	103.9915597	62.35273431	54.47511869	46.66783893	75.85721822	99.20872165	84.86020749	80.92139968	102.1150592	114.6362098	75.01318797	64.88482504	50.60664674	84.57886408	40.12660454	76.13856163	57.85123967	84.29752066	86.54826798	60.10198699	79.44162437	43.57306137	84.57886408	34.49973624	57.28855284	56.72586601	49.12959381
	60.57180851	96.74202128	99.12713472	101.7952128	100.4654255	45.67819149	50.73138298	52.06117021	84.77393617	96.74202128	97.53989362	98.86968085	107.9316888	114.914611	69.6143617	41.42287234	32.38031915	67.75265957	43.28457447	74.1356383	66.68882979	97.00797872	97.27393617	58.17819149	84.25047438	28.85638298	62.16755319	51.52925532	66.42287234	59.24202128	66.42287234
	49.54289049	90.62439214	108.9759282	112.410037	112.410037	60.1244894	53.78331064	34.91538611	98.40497958	98.71620307	107.7416845	124.8589769	115.1774786	120.3998368	78.48667574	50.78778448	29.62458666	89.69072165	32.11437464	110.2314725	56.38980743	107.1192375	110.8539195	62.92550088	94.94084047	33.24255981	85.02236919	44.25209103	72.88465279	64.48161836	55.45613694
	60.15533981	91.51456311	0	108.2912621	110.776699	47.08737864	53.9223301	36.83495146	94.93203883	100.2135922	107.0485437	113.5728155	0	0	83.12621359	44.29126214	31.86407767	90.89320388	34.03883495	100.2135922	49.26213592	107.3592233	110.4660194	75.04854369	0	37.14563107	99.2815534	41.18446602	60.13592233	70.69902913	52.05825243

AMP1CPR1ERY3FUS3TMP2	AMP	CPR	ERY	FUS	TMP	AMP+CPR	AMP+ERY	AMP+FUS	AMP+TMP	CPR+ERY	CPR+FUS	CPR+TMP	ERY+FUS	ERY+TMP	FUS+TMP	AMP+CPR+ERY	AMP+CPR+FUS	AMP+CPR+TMP	AMP+ERY+FUS	AMP+ERY+TMP	AMP+FUS+TMP	CPR+ERY+FUS	CPR+ERY+TMP	CPR+FUS+TMP	ERY+FUS+TMP	AMP+CPR+ERY+FUS	AMP+CPR+ERY+TMP	AMP+CPR+FUS+TMP	AMP+ERY+FUS+TMP	CPR+ERY+FUS+TMP	AMP+CPR+ERY+FUS+TMP
	68.54228943	98.3646914	95.34686971	98.64603482	99.49006506	62.35273431	54.47511869	46.66783893	83.73483383	99.20872165	84.86020749	79.79602602	102.1150592	114.9746193	86.26692456	64.88482504	50.60664674	82.04677334	40.12660454	85.98558115	42.13117637	84.29752066	87.11095481	77.54527871	106.1759729	43.57306137	79.23333919	34.49973624	35.66027783	76.13856163	46.59750308
	60.57180851	96.74202128	99.12713472	101.7952128	101.5292553	45.67819149	50.73138298	52.06117021	88.76329787	96.74202128	97.53989362	94.34840426	107.9316888	115.2182163	82.6462766	41.42287234	32.38031915	73.07180851	43.28457447	84.24202128	52.85904255	97.00797872	101.5292553	76.52925532	101.8595825	28.85638298	67.48670213	54.18882979	43.81648936	65.89095745	57.91223404
	49.54289049	90.62439214	108.9759282	112.410037	109.297802	60.1244894	53.78331064	34.91538611	102.1396615	98.71620307	107.7416845	110.2314725	115.1774786	125.2957976	95.2927446	50.78778448	29.62458666	81.59891072	32.11437464	109.297802	45.49698502	107.1192375	109.6090255	83.4662517	106.6911465	33.24255981	96.22641509	41.13985606	43.00719704	75.37444077	50.78778448
	60.15533981	91.51456311	0	108.2912621	115.4368932	47.08737864	53.9223301	36.83495146	103.631068	100.2135922	107.0485437	114.815534	0	0	89.65048544	44.29126214	31.86407767	86.54368932	34.03883495	107.3592233	46.77669903	107.3592233	111.3980583	88.7184466	0	37.14563107	92.13592233	39.02912621	46.77669903	77.22330097	50.81553398

AMP1CPR1ERY3FUS3TMP3	AMP	CPR	ERY	FUS	TMP	AMP+CPR	AMP+ERY	AMP+FUS	AMP+TMP	CPR+ERY	CPR+FUS	CPR+TMP	ERY+FUS	ERY+TMP	FUS+TMP	AMP+CPR+ERY	AMP+CPR+FUS	AMP+CPR+TMP	AMP+ERY+FUS	AMP+ERY+TMP	AMP+FUS+TMP	CPR+ERY+FUS	CPR+ERY+TMP	CPR+FUS+TMP	ERY+FUS+TMP	AMP+CPR+ERY+FUS	AMP+CPR+ERY+TMP	AMP+CPR+FUS+TMP	AMP+ERY+FUS+TMP	CPR+ERY+FUS+TMP	AMP+CPR+ERY+FUS+TMP
	68.54228943	98.3646914	95.34686971	98.64603482	108.2117109	62.35273431	54.47511869	46.66783893	75.5758748	99.20872165	84.86020749	90.7684192	102.1150592	115.6514382	105.9609636	64.88482504	50.60664674	64.60348162	40.12660454	76.13856163	34.00738526	84.29752066	88.51767188	94.14454018	114.2978003	43.57306137	66.0101987	38.5088799	34.00738526	87.67364164	41.88500088
	60.57180851	96.74202128	99.12713472	101.7952128	100.4654255	45.67819149	50.73138298	52.06117021	76.26329787	96.74202128	97.53989362	98.07180851	107.9316888	107.0208729	105.2526596	41.42287234	32.38031915	63.23138298	43.28457447	79.45478723	37.25066489	97.00797872	103.3909574	94.88031915	114.3074004	28.85638298	62.69946809	23.07180851	48.6037234	87.16755319	32.91223404
	49.54289049	90.62439214	108.9759282	112.410037	115.8334954	60.1244894	53.78331064	34.91538611	74.12954678	98.71620307	107.7416845	111.4763665	115.1774786	117.1358629	109.920249	50.78778448	29.62458666	74.12954678	32.11437464	98.40497958	41.33437074	107.1192375	108.052908	100.5835441	128.5597715	33.24255981	66.34895935	45.38027621	35.73234779	99.33865007	31.99766582
	60.15533981	91.51456311	0	108.2912621	108.2912621	47.08737864	53.9223301	36.83495146	82.19417476	100.2135922	107.0485437	117.9223301	0	0	111.0873786	44.29126214	31.86407767	92.13592233	34.03883495	82.19417476	28.77669903	107.3592233	112.0194175	107.9805825	0	37.14563107	58.89320388	30.95145631	29.70873786	106.7378641	31.57281553

AMP1CPR2ERY1FUS1TMP1	AMP	CPR	ERY	FUS	TMP	AMP+CPR	AMP+ERY	AMP+FUS	AMP+TMP	CPR+ERY	CPR+FUS	CPR+TMP	ERY+FUS	ERY+TMP	FUS+TMP	AMP+CPR+ERY	AMP+CPR+FUS	AMP+CPR+TMP	AMP+ERY+FUS	AMP+ERY+TMP	AMP+FUS+TMP	CPR+ERY+FUS	CPR+ERY+TMP	CPR+FUS+TMP	ERY+FUS+TMP	AMP+CPR+ERY+FUS	AMP+CPR+ERY+TMP	AMP+CPR+FUS+TMP	AMP+ERY+FUS+TMP	CPR+ERY+FUS+TMP	AMP+CPR+ERY+FUS+TMP
	68.54228943	109.8222638	95.26991384	70.23034992	103.9915597	64.32213821	52.54088271	13.18797257	75.85721822	86.47333957	70.92609916	110.121609	60.3833304	89.36170213	64.91999297	60.3833304	19.65887111	78.38930895	6.400562687	78.38930895	10.09319501	52.06735267	84.97661366	62.84377923	55.91700369	4.747670125	83.73483383	39.88042905	57.92157552	46.67913938	19.65887111
	60.57180851	103.7561455	101.2632979	82.6462766	100.4654255	58.11170213	47.7393617	3.390957447	84.77393617	99.98033432	72.546706	109.4198623	63.49734043	96.21010638	54.38829787	53.125	39.7606383	78.39095745	7.313829787	74.1356383	10.92087766	62.79252704	89.59685349	84.18879056	55.98404255	8.527260638	70.94414894	44.34840426	6.848404255	68.77089479	6.848404255
	49.54289049	83.55704698	94.35907411	74.01283797	112.410037	52.03267847	51.09900798	2.120210076	98.40497958	87.73303505	56.54362416	113.3855332	74.94650846	109.297802	71.21182649	63.85917137	3.365104065	102.7621085	3.053880568	87.51215717	5.23244505	56.2453393	95.78672632	56.54362416	72.45672048	2.742657071	88.75705116	46.00272321	3.365104065	49.38478747	1.186539584
	60.15533981	0	97.72815534	81.26213592	110.776699	61.06796117	51.74757282	5.475728155	94.93203883	0	0	0	76	112.6407767	72.89320388	58.89320388	4.233009709	101.1456311	3.300970874	89.65048544	1.747572816	0	0	0	71.33980583	2.349514563	86.85436893	34.05825243	-0.116504854	0	1.436893204

AMP1CPR2ERY1FUS1TMP2	AMP	CPR	ERY	FUS	TMP	AMP+CPR	AMP+ERY	AMP+FUS	AMP+TMP	CPR+ERY	CPR+FUS	CPR+TMP	ERY+FUS	ERY+TMP	FUS+TMP	AMP+CPR+ERY	AMP+CPR+FUS	AMP+CPR+TMP	AMP+ERY+FUS	AMP+ERY+TMP	AMP+FUS+TMP	CPR+ERY+FUS	CPR+ERY+TMP	CPR+FUS+TMP	ERY+FUS+TMP	AMP+CPR+ERY+FUS	AMP+CPR+ERY+TMP	AMP+CPR+FUS+TMP	AMP+ERY+FUS+TMP	CPR+ERY+FUS+TMP	AMP+CPR+ERY+FUS+TMP
	68.54228943	109.8222638	95.26991384	70.23034992	99.49006506	64.32213821	52.54088271	13.18797257	83.73483383	86.47333957	70.92609916	111.3189897	60.3833304	87.95498505	64.63864955	60.3833304	19.65887111	75.01318797	6.400562687	89.36170213	9.530508177	52.06735267	106.828812	72.42282507	58.44909443	4.747670125	75.29453139	6.963249516	7.561104273	51.61833489	4.747670125
	60.57180851	103.7561455	101.2632979	82.6462766	101.5292553	58.11170213	47.7393617	3.390957447	88.76329787	99.98033432	72.546706	116.027532	63.49734043	94.6143617	55.25265957	53.125	39.7606383	85.30585106	7.313829787	83.97606383	8.377659574	62.79252704	104.7000983	86.3913471	53.85638298	8.527260638	73.33776596	16.77194149	6.582446809	59.33136676	2.676196809
	49.54289049	83.55704698	94.35907411	74.01283797	109.297802	52.03267847	51.09900798	2.120210076	102.1396615	87.73303505	56.54362416	119.0529456	74.94650846	109.920249	60.94145108	63.85917137	3.365104065	95.2927446	3.053880568	97.16008559	2.120210076	56.2453393	108.3146905	63.10589113	69.03326201	2.742657071	86.57848668	3.98755106	1.808986578	45.84265474	1.808986578
	60.15533981	0	97.72815534	81.26213592	115.4368932	61.06796117	51.74757282	5.475728155	103.631068	0	0	0	76	119.1650485	72.58252427	58.89320388	4.233009709	97.10679612	3.300970874	91.2038835	2.058252427	0	0	0	65.74757282	2.349514563	88.09708738	2.058252427	1.747572816	0	16.69902913

AMP1CPR2ERY1FUS1TMP3	AMP	CPR	ERY	FUS	TMP	AMP+CPR	AMP+ERY	AMP+FUS	AMP+TMP	CPR+ERY	CPR+FUS	CPR+TMP	ERY+FUS	ERY+TMP	FUS+TMP	AMP+CPR+ERY	AMP+CPR+FUS	AMP+CPR+TMP	AMP+ERY+FUS	AMP+ERY+TMP	AMP+FUS+TMP	CPR+ERY+FUS	CPR+ERY+TMP	CPR+FUS+TMP	ERY+FUS+TMP	AMP+CPR+ERY+FUS	AMP+CPR+ERY+TMP	AMP+CPR+FUS+TMP	AMP+ERY+FUS+TMP	CPR+ERY+FUS+TMP	AMP+CPR+ERY+FUS+TMP
	68.54228943	109.8222638	95.26991384	70.23034992	108.2117109	64.32213821	52.54088271	13.18797257	75.5758748	86.47333957	70.92609916	124.4901777	60.3833304	91.04976262	73.92298224	60.3833304	19.65887111	62.91542114	6.400562687	79.23333919	7.279760858	52.06735267	117.9045837	77.81103835	67.4520837	4.747670125	53.91243186	17.12678038	5.556532442	59.55098223	1.617724635
	60.57180851	103.7561455	101.2632979	82.6462766	100.4654255	58.11170213	47.7393617	3.390957447	76.26329787	99.98033432	72.546706	123.2645034	63.49734043	94.88031915	59.50797872	53.125	39.7606383	74.93351064	7.313829787	80.25265957	15.97406915	62.79252704	113.5103245	90.79646018	51.99468085	8.527260638	60.03989362	22.62300532	2.32712766	68.14159292	5.984042553
	49.54289049	83.55704698	94.35907411	74.01283797	115.8334954	52.03267847	51.09900798	2.120210076	74.12954678	87.73303505	56.54362416	119.3512304	74.94650846	108.3641315	68.41081502	63.85917137	3.365104065	63.23672437	3.053880568	73.81832328	1.186539584	56.2453393	110.7009694	64.00074571	70.278156	2.742657071	71.3285353	4.921221552	1.808986578	55.05219985	3.053880568
	60.15533981	0	97.72815534	81.26213592	108.2912621	61.06796117	51.74757282	5.475728155	82.19417476	0	0	0	76	111.7087379	74.75728155	58.89320388	4.233009709	75.04854369	3.300970874	64.48543689	4.54368932	0	0	0	61.08737864	2.349514563	73.18446602	1.747572816	2.67961165	0	8.932038835

AMP1CPR2ERY1FUS2TMP1	AMP	CPR	ERY	FUS	TMP	AMP+CPR	AMP+ERY	AMP+FUS	AMP+TMP	CPR+ERY	CPR+FUS	CPR+TMP	ERY+FUS	ERY+TMP	FUS+TMP	AMP+CPR+ERY	AMP+CPR+FUS	AMP+CPR+TMP	AMP+ERY+FUS	AMP+ERY+TMP	AMP+FUS+TMP	CPR+ERY+FUS	CPR+ERY+TMP	CPR+FUS+TMP	ERY+FUS+TMP	AMP+CPR+ERY+FUS	AMP+CPR+ERY+TMP	AMP+CPR+FUS+TMP	AMP+ERY+FUS+TMP	CPR+ERY+FUS+TMP	AMP+CPR+ERY+FUS+TMP
	68.54228943	109.8222638	95.26991384	85.70423774	103.9915597	64.32213821	52.54088271	32.03798136	75.85721822	86.47333957	81.98316183	110.121609	68.82363285	89.36170213	66.0101987	60.3833304	35.13275892	78.38930895	20.78424477	78.38930895	28.34534904	62.84377923	84.97661366	59.85032741	65.20133638	20.15122209	83.73483383	53.38491296	24.16036575	63.74181478	41.00580271
	60.57180851	103.7561455	101.2632979	95.94414894	100.4654255	58.11170213	47.7393617	24.48470745	84.77393617	99.98033432	85.50639135	109.4198623	67.48670213	96.21010638	62.96542553	53.125	23.80319149	78.39095745	15.82446809	74.1356383	34.85704787	68.14159292	89.59685349	61.84857424	44.88031915	18.48404255	70.94414894	36.1037234	31.78191489	63.10717797	24.06914894
	49.54289049	83.55704698	94.35907411	96.53763859	112.410037	52.03267847	51.09900798	11.76813849	98.40497958	87.73303505	79.08277405	113.3855332	63.74246256	109.297802	67.47714452	63.85917137	30.13032484	102.7621085	6.788562536	87.51215717	34.4874538	58.50111857	95.78672632	60.5891126	63.74246256	21.72729041	88.75705116	36.04357129	1.808986578	57.43847875	36.97724178
	60.15533981	0	97.72815534	100.5242718	110.776699	61.06796117	51.74757282	8.893203883	94.93203883	0	0	0	71.33980583	112.6407767	66.36893204	58.89320388	23.80582524	101.1456311	25.04854369	89.65048544	39.33980583	0	0	0	62.01941748	16.97087379	86.85436893	42.75728155	2.058252427	0	27.84466019

AMP1CPR2ERY1FUS2TMP2	AMP	CPR	ERY	FUS	TMP	AMP+CPR	AMP+ERY	AMP+FUS	AMP+TMP	CPR+ERY	CPR+FUS	CPR+TMP	ERY+FUS	ERY+TMP	FUS+TMP	AMP+CPR+ERY	AMP+CPR+FUS	AMP+CPR+TMP	AMP+ERY+FUS	AMP+ERY+TMP	AMP+FUS+TMP	CPR+ERY+FUS	CPR+ERY+TMP	CPR+FUS+TMP	ERY+FUS+TMP	AMP+CPR+ERY+FUS	AMP+CPR+ERY+TMP	AMP+CPR+FUS+TMP	AMP+ERY+FUS+TMP	CPR+ERY+FUS+TMP	AMP+CPR+ERY+FUS+TMP
	68.54228943	109.8222638	95.26991384	85.70423774	99.49006506	64.32213821	52.54088271	32.03798136	83.73483383	86.47333957	81.98316183	111.3189897	68.82363285	87.95498505	67.17074028	60.3833304	35.13275892	75.01318797	20.78424477	89.36170213	27.78266221	62.84377923	106.828812	64.34050514	69.70283102	20.15122209	75.29453139	33.69087392	29.18937929	74.21889616	36.78565149
	60.57180851	103.7561455	101.2632979	95.94414894	101.5292553	58.11170213	47.7393617	24.48470745	88.76329787	99.98033432	85.50639135	116.027532	67.48670213	94.6143617	62.43351064	53.125	23.80319149	85.30585106	15.82446809	83.97606383	29.27194149	68.14159292	104.7000983	73.23500492	52.32712766	18.48404255	73.33776596	24.66755319	25.66489362	64.99508358	21.55917553
	49.54289049	83.55704698	94.35907411	96.53763859	109.297802	52.03267847	51.09900798	11.76813849	102.1396615	87.73303505	79.08277405	119.0529456	63.74246256	109.920249	74.63528496	63.85917137	30.13032484	95.2927446	6.788562536	97.16008559	21.10484342	58.50111857	108.3146905	76.39821029	70.900603	21.72729041	86.57848668	21.72729041	22.03851391	56.54362416	22.6609609
	60.15533981	0	97.72815534	100.5242718	115.4368932	61.06796117	51.74757282	8.893203883	103.631068	0	0	0	71.33980583	119.1650485	63.86407767	58.89320388	23.80582524	97.10679612	25.04854369	91.2038835	25.26213592	0	0	0	63.88349515	16.97087379	88.09708738	28.15533981	11.90291262	0	31.88349515

AMP1CPR2ERY1FUS2TMP3	AMP	CPR	ERY	FUS	TMP	AMP+CPR	AMP+ERY	AMP+FUS	AMP+TMP	CPR+ERY	CPR+FUS	CPR+TMP	ERY+FUS	ERY+TMP	FUS+TMP	AMP+CPR+ERY	AMP+CPR+FUS	AMP+CPR+TMP	AMP+ERY+FUS	AMP+ERY+TMP	AMP+FUS+TMP	CPR+ERY+FUS	CPR+ERY+TMP	CPR+FUS+TMP	ERY+FUS+TMP	AMP+CPR+ERY+FUS	AMP+CPR+ERY+TMP	AMP+CPR+FUS+TMP	AMP+ERY+FUS+TMP	CPR+ERY+FUS+TMP	AMP+CPR+ERY+FUS+TMP
	68.54228943	109.8222638	95.26991384	85.70423774	108.2117109	64.32213821	52.54088271	32.03798136	75.5758748	86.47333957	81.98316183	124.4901777	68.82363285	91.04976262	81.48408651	60.3833304	35.13275892	62.91542114	20.78424477	79.23333919	1.89906805	62.84377923	117.9045837	68.83068288	67.41691577	20.15122209	53.91243186	8.93265342	3.340953051	75.11693171	13.46931598
	60.57180851	103.7561455	101.2632979	95.94414894	100.4654255	58.11170213	47.7393617	24.48470745	76.26329787	99.98033432	85.50639135	123.2645034	67.48670213	94.88031915	78.65691489	53.125	23.80319149	74.93351064	15.82446809	80.25265957	14.91023936	68.14159292	113.5103245	77.26647001	60.57180851	18.48404255	60.03989362	13.31449468	7.197473404	75.69321534	17.03789894
	49.54289049	83.55704698	94.35907411	96.53763859	115.8334954	52.03267847	51.09900798	11.76813849	74.12954678	87.73303505	79.08277405	119.3512304	63.74246256	108.3641315	83.4662517	63.85917137	30.13032484	63.23672437	6.788562536	73.81832328	9.589574013	58.50111857	110.7009694	79.38105891	62.30305388	21.72729041	71.3285353	20.48239642	0.564092589	61.01789709	24.52830189
	60.15533981	0	97.72815534	100.5242718	108.2912621	61.06796117	51.74757282	8.893203883	82.19417476	0	0	0	71.33980583	111.7087379	97.72815534	58.89320388	23.80582524	75.04854369	25.04854369	64.48543689	7.650485437	0	0	0	58.27184466	16.97087379	73.18446602	15.00970874	1.436893204	0	7.86407767

AMP1CPR2ERY1FUS3TMP1	AMP	CPR	ERY	FUS	TMP	AMP+CPR	AMP+ERY	AMP+FUS	AMP+TMP	CPR+ERY	CPR+FUS	CPR+TMP	ERY+FUS	ERY+TMP	FUS+TMP	AMP+CPR+ERY	AMP+CPR+FUS	AMP+CPR+TMP	AMP+ERY+FUS	AMP+ERY+TMP	AMP+FUS+TMP	CPR+ERY+FUS	CPR+ERY+TMP	CPR+FUS+TMP	ERY+FUS+TMP	AMP+CPR+ERY+FUS	AMP+CPR+ERY+TMP	AMP+CPR+FUS+TMP	AMP+ERY+FUS+TMP	CPR+ERY+FUS+TMP	AMP+CPR+ERY+FUS+TMP
	68.54228943	109.8222638	95.26991384	98.64603482	103.9915597	64.32213821	52.54088271	46.66783893	75.85721822	86.47333957	104.7333957	110.121609	88.23632847	89.36170213	75.01318797	60.3833304	53.34974503	78.38930895	36.46914014	78.38930895	57.85123967	86.47333957	84.97661366	71.20673527	68.26094602	29.50589063	83.73483383	56.16317918	53.91243186	67.01590271	41.81466503
	60.57180851	103.7561455	101.2632979	101.7952128	100.4654255	58.11170213	47.7393617	52.06117021	84.77393617	99.98033432	103.7561455	109.4198623	89.82712766	96.21010638	69.6143617	53.125	53.59042553	78.39095745	41.95478723	74.1356383	66.68882979	89.28220256	89.59685349	76.06686332	58.17819149	28.59042553	70.94414894	58.97606383	57.38031915	69.14454277	57.38031915
	49.54289049	83.55704698	94.35907411	112.410037	112.410037	52.03267847	51.09900798	34.91538611	98.40497958	87.73303505	99.06785981	113.3855332	103.073332	109.297802	78.48667574	63.85917137	43.62964404	102.7621085	29.00213966	87.51215717	56.38980743	90.41759881	95.78672632	83.85533184	68.21630033	21.53277572	88.75705116	66.66018284	44.25209103	81.76733781	54.83368994
	60.15533981	0	97.72815534	108.2912621	110.776699	61.06796117	51.74757282	36.83495146	94.93203883	0	0	0	100.2135922	112.6407767	83.12621359	58.89320388	43.3592233	101.1456311	43.04854369	89.65048544	49.26213592	0	0	0	66.97087379	27.2038835	86.85436893	61.06796117	43.04854369	0	54.54368932

AMP1CPR2ERY1FUS3TMP2	AMP	CPR	ERY	FUS	TMP	AMP+CPR	AMP+ERY	AMP+FUS	AMP+TMP	CPR+ERY	CPR+FUS	CPR+TMP	ERY+FUS	ERY+TMP	FUS+TMP	AMP+CPR+ERY	AMP+CPR+FUS	AMP+CPR+TMP	AMP+ERY+FUS	AMP+ERY+TMP	AMP+FUS+TMP	CPR+ERY+FUS	CPR+ERY+TMP	CPR+FUS+TMP	ERY+FUS+TMP	AMP+CPR+ERY+FUS	AMP+CPR+ERY+TMP	AMP+CPR+FUS+TMP	AMP+ERY+FUS+TMP	CPR+ERY+FUS+TMP	AMP+CPR+ERY+FUS+TMP
	68.54228943	109.8222638	95.26991384	98.64603482	99.49006506	64.32213821	52.54088271	46.66783893	83.73483383	86.47333957	104.7333957	111.3189897	88.23632847	87.95498505	86.26692456	60.3833304	53.34974503	75.01318797	36.46914014	89.36170213	42.13117637	86.47333957	106.828812	85.27595884	83.172147	29.50589063	75.29453139	55.60049235	28.62669246	73.30215154	44.06541234
	60.57180851	103.7561455	101.2632979	101.7952128	101.5292553	58.11170213	47.7393617	52.06117021	88.76329787	99.98033432	103.7561455	116.027532	89.82712766	94.6143617	82.6462766	53.125	53.59042553	85.30585106	41.95478723	83.97606383	52.85904255	89.28220256	104.7000983	85.19174041	77.06117021	28.59042553	73.33776596	64.56117021	36.6356383	84.87708948	58.97606383
	49.54289049	83.55704698	94.35907411	112.410037	109.297802	52.03267847	51.09900798	34.91538611	102.1396615	87.73303505	99.06785981	119.0529456	103.073332	109.920249	95.2927446	63.85917137	43.62964404	95.2927446	29.00213966	97.16008559	45.49698502	90.41759881	108.3146905	92.50559284	80.66524023	21.53277572	86.57848668	69.77241782	38.33884458	88.03131991	59.19081891
	60.15533981	0	97.72815534	108.2912621	115.4368932	61.06796117	51.74757282	36.83495146	103.631068	0	0	0	100.2135922	119.1650485	89.65048544	58.89320388	43.3592233	97.10679612	43.04854369	91.2038835	46.77669903	0	0	0	75.98058252	27.2038835	88.09708738	83.4368932	40.58252427	0	45.22330097

AMP1CPR2ERY1FUS3TMP3	AMP	CPR	ERY	FUS	TMP	AMP+CPR	AMP+ERY	AMP+FUS	AMP+TMP	CPR+ERY	CPR+FUS	CPR+TMP	ERY+FUS	ERY+TMP	FUS+TMP	AMP+CPR+ERY	AMP+CPR+FUS	AMP+CPR+TMP	AMP+ERY+FUS	AMP+ERY+TMP	AMP+FUS+TMP	CPR+ERY+FUS	CPR+ERY+TMP	CPR+FUS+TMP	ERY+FUS+TMP	AMP+CPR+ERY+FUS	AMP+CPR+ERY+TMP	AMP+CPR+FUS+TMP	AMP+ERY+FUS+TMP	CPR+ERY+FUS+TMP	AMP+CPR+ERY+FUS+TMP
	68.54228943	109.8222638	95.26991384	98.64603482	108.2117109	64.32213821	52.54088271	46.66783893	75.5758748	86.47333957	104.7333957	124.4901777	88.23632847	91.04976262	105.9609636	60.3833304	53.34974503	62.91542114	36.46914014	79.23333919	34.00738526	86.47333957	117.9045837	105.9307764	95.55125725	29.50589063	53.91243186	36.50430807	20.46773343	86.17399439	24.65271672
	60.57180851	103.7561455	101.2632979	101.7952128	100.4654255	58.11170213	47.7393617	52.06117021	76.26329787	99.98033432	103.7561455	123.2645034	89.82712766	94.88031915	105.2526596	53.125	53.59042553	74.93351064	41.95478723	80.25265957	37.25066489	89.28220256	113.5103245	102.8121927	97.00797872	28.59042553	60.03989362	42.42021277	39.29521277	94.00196657	33.17819149
	49.54289049	83.55704698	94.35907411	112.410037	115.8334954	52.03267847	51.09900798	34.91538611	74.12954678	87.73303505	99.06785981	119.3512304	103.073332	108.3641315	109.920249	63.85917137	43.62964404	63.23672437	29.00213966	73.81832328	41.33437074	90.41759881	110.7009694	111.2975391	106.808014	21.53277572	71.3285353	45.69149971	22.03851391	98.17300522	28.06846917
	60.15533981	0	97.72815534	108.2912621	108.2912621	61.06796117	51.74757282	36.83495146	82.19417476	0	0	0	100.2135922	111.7087379	111.0873786	58.89320388	43.3592233	75.04854369	43.04854369	64.48543689	28.77669903	0	0	0	105.184466	27.2038835	73.18446602	29.70873786	26.60194175	0	38.09708738

AMP1CPR2ERY2FUS1TMP1	AMP	CPR	ERY	FUS	TMP	AMP+CPR	AMP+ERY	AMP+FUS	AMP+TMP	CPR+ERY	CPR+FUS	CPR+TMP	ERY+FUS	ERY+TMP	FUS+TMP	AMP+CPR+ERY	AMP+CPR+FUS	AMP+CPR+TMP	AMP+ERY+FUS	AMP+ERY+TMP	AMP+FUS+TMP	CPR+ERY+FUS	CPR+ERY+TMP	CPR+FUS+TMP	ERY+FUS+TMP	AMP+CPR+ERY+FUS	AMP+CPR+ERY+TMP	AMP+CPR+FUS+TMP	AMP+ERY+FUS+TMP	CPR+ERY+FUS+TMP	AMP+CPR+ERY+FUS+TMP
	68.54228943	109.8222638	103.8353601	70.23034992	103.9915597	64.32213821	48.56690698	13.18797257	75.85721822	99.34518241	70.92609916	110.121609	69.71000935	113.414406	64.91999297	52.22437137	19.65887111	78.38930895	5.591700369	82.89080359	10.09319501	55.36014967	99.94387278	62.84377923	52.66604303	4.993845613	76.98259188	39.88042905	8.123791103	55.36014967	25.84842624
	60.57180851	103.7561455	102.1828909	82.6462766	100.4654255	58.11170213	46.40957447	3.390957447	84.77393617	106.5880039	72.546706	109.4198623	77.95476893	110.993117	54.38829787	50.99734043	39.7606383	78.39095745	8.377659574	71.74202128	10.92087766	62.47787611	99.03638151	84.18879056	72.86135693	2.32712766	75.73138298	44.34840426	11.71875	70.34414946	3.92287234
	49.54289049	83.55704698	88.62788963	74.01283797	112.410037	52.03267847	57.01225443	2.120210076	98.40497958	97.87472036	56.54362416	113.3855332	82.06562267	107.7181208	71.21182649	59.19081891	3.365104065	102.7621085	34.7986773	90.00194515	5.23244505	55.94705444	105.033557	56.54362416	56.7114094	3.365104065	92.80295662	46.00272321	2.742657071	54.15734526	2.431433573
	60.15533981	0	0	81.26213592	110.776699	61.06796117	52.05825243	5.475728155	94.93203883	0	0	0	0	0	72.89320388	48.01941748	4.233009709	101.1456311	4.233009709	91.2038835	1.747572816	0	0	0	0	17.26213592	98.34951456	34.05825243	2.990291262	0	2.058252427

AMP1CPR2ERY2FUS1TMP2	AMP	CPR	ERY	FUS	TMP	AMP+CPR	AMP+ERY	AMP+FUS	AMP+TMP	CPR+ERY	CPR+FUS	CPR+TMP	ERY+FUS	ERY+TMP	FUS+TMP	AMP+CPR+ERY	AMP+CPR+FUS	AMP+CPR+TMP	AMP+ERY+FUS	AMP+ERY+TMP	AMP+FUS+TMP	CPR+ERY+FUS	CPR+ERY+TMP	CPR+FUS+TMP	ERY+FUS+TMP	AMP+CPR+ERY+FUS	AMP+CPR+ERY+TMP	AMP+CPR+FUS+TMP	AMP+ERY+FUS+TMP	CPR+ERY+FUS+TMP	AMP+CPR+ERY+FUS+TMP
	68.54228943	109.8222638	103.8353601	70.23034992	99.49006506	64.32213821	48.56690698	13.18797257	83.73483383	99.34518241	70.92609916	111.3189897	69.71000935	120.2993452	64.63864955	52.22437137	19.65887111	75.01318797	5.591700369	82.04677334	9.530508177	55.36014967	99.34518241	72.42282507	62.84377923	4.993845613	80.92139968	6.963249516	9.811851591	56.40785781	3.059609636
	60.57180851	103.7561455	102.1828909	82.6462766	101.5292553	58.11170213	46.40957447	3.390957447	88.76329787	106.5880039	72.546706	116.027532	77.95476893	119.1740413	55.25265957	50.99734043	39.7606383	85.30585106	8.377659574	83.44414894	8.377659574	62.47787611	105.3294002	86.3913471	76.32251721	2.32712766	74.66755319	16.77194149	4.803856383	72.23205506	2.859042553
	49.54289049	83.55704698	88.62788963	74.01283797	109.297802	52.03267847	57.01225443	2.120210076	102.1396615	97.87472036	56.54362416	119.0529456	82.06562267	112.7889635	60.94145108	59.19081891	3.365104065	95.2927446	34.7986773	100.8947676	2.120210076	55.94705444	108.9112603	63.10589113	63.87024609	3.365104065	86.57848668	3.98755106	2.120210076	52.06935123	1.808986578
	60.15533981	0	0	81.26213592	115.4368932	61.06796117	52.05825243	5.475728155	103.631068	0	0	0	0	0	72.58252427	48.01941748	4.233009709	97.10679612	4.233009709	98.34951456	2.058252427	0	0	0	0	17.26213592	90.89320388	2.058252427	1.126213592	0	1.126213592

AMP1CPR2ERY2FUS1TMP3	AMP	CPR	ERY	FUS	TMP	AMP+CPR	AMP+ERY	AMP+FUS	AMP+TMP	CPR+ERY	CPR+FUS	CPR+TMP	ERY+FUS	ERY+TMP	FUS+TMP	AMP+CPR+ERY	AMP+CPR+FUS	AMP+CPR+TMP	AMP+ERY+FUS	AMP+ERY+TMP	AMP+FUS+TMP	CPR+ERY+FUS	CPR+ERY+TMP	CPR+FUS+TMP	ERY+FUS+TMP	AMP+CPR+ERY+FUS	AMP+CPR+ERY+TMP	AMP+CPR+FUS+TMP	AMP+ERY+FUS+TMP	CPR+ERY+FUS+TMP	AMP+CPR+ERY+FUS+TMP
	68.54228943	109.8222638	103.8353601	70.23034992	108.2117109	64.32213821	48.56690698	13.18797257	75.5758748	99.34518241	70.92609916	124.4901777	69.71000935	121.7960711	73.92298224	52.22437137	19.65887111	62.91542114	5.591700369	79.79602602	7.279760858	55.36014967	112.2170253	77.81103835	66.41721235	4.993845613	52.7870582	17.12678038	7.561104273	64.63985033	4.431158783
	60.57180851	103.7561455	102.1828909	82.6462766	100.4654255	58.11170213	46.40957447	3.390957447	76.26329787	106.5880039	72.546706	123.2645034	77.95476893	124.5231072	59.50797872	50.99734043	39.7606383	74.93351064	8.377659574	80.78457447	15.97406915	62.47787611	112.5663717	90.79646018	78.89872173	2.32712766	71.74202128	22.62300532	7.04787234	76.95181908	7.845744681
	49.54289049	83.55704698	88.62788963	74.01283797	115.8334954	52.03267847	57.01225443	2.120210076	74.12954678	97.87472036	56.54362416	119.3512304	82.06562267	112.7889635	68.41081502	59.19081891	3.365104065	63.23672437	34.7986773	93.11418012	1.186539584	55.94705444	105.3318419	64.00074571	80.87248322	3.365104065	72.57342929	4.921221552	3.676327563	57.73676361	1.497763081
	60.15533981	0	0	81.26213592	108.2912621	61.06796117	52.05825243	5.475728155	82.19417476	0	0	0	0	0	74.75728155	48.01941748	4.233009709	75.04854369	4.233009709	75.98058252	4.54368932	0	0	0	0	17.26213592	70.0776699	1.747572816	1.436893204	0	4.233009709

AMP1CPR2ERY2FUS2TMP1	AMP	CPR	ERY	FUS	TMP	AMP+CPR	AMP+ERY	AMP+FUS	AMP+TMP	CPR+ERY	CPR+FUS	CPR+TMP	ERY+FUS	ERY+TMP	FUS+TMP	AMP+CPR+ERY	AMP+CPR+FUS	AMP+CPR+TMP	AMP+ERY+FUS	AMP+ERY+TMP	AMP+FUS+TMP	CPR+ERY+FUS	CPR+ERY+TMP	CPR+FUS+TMP	ERY+FUS+TMP	AMP+CPR+ERY+FUS	AMP+CPR+ERY+TMP	AMP+CPR+FUS+TMP	AMP+ERY+FUS+TMP	CPR+ERY+FUS+TMP	AMP+CPR+ERY+FUS+TMP
	68.54228943	109.8222638	103.8353601	85.70423774	103.9915597	64.32213821	48.56690698	32.03798136	75.85721822	99.34518241	81.98316183	110.121609	76.59494855	113.414406	66.0101987	52.22437137	35.13275892	78.38930895	25.56708282	82.89080359	28.34534904	70.30869972	99.94387278	59.85032741	58.3536015	24.37137331	76.98259188	53.38491296	36.50430807	72.42282507	45.50729734
	60.57180851	103.7561455	102.1828909	95.94414894	100.4654255	58.11170213	46.40957447	24.48470745	84.77393617	106.5880039	85.50639135	109.4198623	89.91150442	110.993117	62.96542553	50.99734043	23.80319149	78.39095745	18.48404255	71.74202128	34.85704787	64.7394297	99.03638151	61.84857424	66.56833825	19.01595745	75.73138298	36.1037234	25.66489362	76.00786627	19.01595745
	49.54289049	83.55704698	88.62788963	96.53763859	112.410037	52.03267847	57.01225443	11.76813849	98.40497958	97.87472036	79.08277405	113.3855332	83.55704698	107.7181208	67.47714452	59.19081891	30.13032484	102.7621085	12.70180899	90.00194515	34.4874538	71.92393736	105.033557	60.5891126	74.01193139	11.456915	92.80295662	36.04357129	42.57926473	56.84190902	32.30888932
	60.15533981	0	0	100.5242718	110.776699	61.06796117	52.05825243	8.893203883	94.93203883	0	0	0	0	0	66.36893204	48.01941748	23.80582524	101.1456311	21.00970874	91.2038835	39.33980583	0	0	0	0	7.029126214	98.34951456	42.75728155	27.22330097	0	25.3592233

AMP1CPR2ERY2FUS2TMP2	AMP	CPR	ERY	FUS	TMP	AMP+CPR	AMP+ERY	AMP+FUS	AMP+TMP	CPR+ERY	CPR+FUS	CPR+TMP	ERY+FUS	ERY+TMP	FUS+TMP	AMP+CPR+ERY	AMP+CPR+FUS	AMP+CPR+TMP	AMP+ERY+FUS	AMP+ERY+TMP	AMP+FUS+TMP	CPR+ERY+FUS	CPR+ERY+TMP	CPR+FUS+TMP	ERY+FUS+TMP	AMP+CPR+ERY+FUS	AMP+CPR+ERY+TMP	AMP+CPR+FUS+TMP	AMP+ERY+FUS+TMP	CPR+ERY+FUS+TMP	AMP+CPR+ERY+FUS+TMP
	68.54228943	109.8222638	103.8353601	85.70423774	99.49006506	64.32213821	48.56690698	32.03798136	83.73483383	99.34518241	81.98316183	111.3189897	76.59494855	120.2993452	67.17074028	52.22437137	35.13275892	75.01318797	25.56708282	82.04677334	27.78266221	70.30869972	99.34518241	64.34050514	64.02245089	24.37137331	80.92139968	33.69087392	36.50430807	75.71562208	32.56550026
	60.57180851	103.7561455	102.1828909	95.94414894	101.5292553	58.11170213	46.40957447	24.48470745	88.76329787	106.5880039	85.50639135	116.027532	89.91150442	119.1740413	62.43351064	50.99734043	23.80319149	85.30585106	18.48404255	83.44414894	29.27194149	64.7394297	105.3294002	73.23500492	71.03244838	19.01595745	74.66755319	24.66755319	24.66755319	76.32251721	25.01662234
	49.54289049	83.55704698	88.62788963	96.53763859	109.297802	52.03267847	57.01225443	11.76813849	102.1396615	97.87472036	79.08277405	119.0529456	83.55704698	112.7889635	74.63528496	59.19081891	30.13032484	95.2927446	12.70180899	100.8947676	21.10484342	71.92393736	108.9112603	76.39821029	82.96047726	11.456915	86.57848668	21.72729041	46.9363937	60.71961223	29.50787784
	60.15533981	0	0	100.5242718	115.4368932	61.06796117	52.05825243	8.893203883	103.631068	0	0	0	0	0	63.86407767	48.01941748	23.80582524	97.10679612	21.00970874	98.34951456	25.26213592	0	0	0	0	7.029126214	90.89320388	28.15533981	28.77669903	0	9.514563107

AMP1CPR2ERY2FUS2TMP3	AMP	CPR	ERY	FUS	TMP	AMP+CPR	AMP+ERY	AMP+FUS	AMP+TMP	CPR+ERY	CPR+FUS	CPR+TMP	ERY+FUS	ERY+TMP	FUS+TMP	AMP+CPR+ERY	AMP+CPR+FUS	AMP+CPR+TMP	AMP+ERY+FUS	AMP+ERY+TMP	AMP+FUS+TMP	CPR+ERY+FUS	CPR+ERY+TMP	CPR+FUS+TMP	ERY+FUS+TMP	AMP+CPR+ERY+FUS	AMP+CPR+ERY+TMP	AMP+CPR+FUS+TMP	AMP+ERY+FUS+TMP	CPR+ERY+FUS+TMP	AMP+CPR+ERY+FUS+TMP
	68.54228943	109.8222638	103.8353601	85.70423774	108.2117109	64.32213821	48.56690698	32.03798136	75.5758748	99.34518241	81.98316183	124.4901777	76.59494855	121.7960711	81.48408651	52.22437137	35.13275892	62.91542114	25.56708282	79.79602602	1.89906805	70.30869972	112.2170253	68.83068288	81.68381665	24.37137331	52.7870582	8.93265342	2.215579392	76.61365762	6.119219272
	60.57180851	103.7561455	102.1828909	95.94414894	100.4654255	58.11170213	46.40957447	24.48470745	76.26329787	106.5880039	85.50639135	123.2645034	89.91150442	124.5231072	78.65691489	50.99734043	23.80319149	74.93351064	18.48404255	80.78457447	14.91023936	64.7394297	112.5663717	77.26647001	94.3166175	19.01595745	71.74202128	13.31449468	0.997340426	75.69321534	32.1974734
	49.54289049	83.55704698	88.62788963	96.53763859	115.8334954	52.03267847	57.01225443	11.76813849	74.12954678	97.87472036	79.08277405	119.3512304	83.55704698	112.7889635	83.4662517	59.19081891	30.13032484	63.23672437	12.70180899	93.11418012	9.589574013	71.92393736	105.3318419	79.38105891	91.90902312	11.456915	72.57342929	20.48239642	7.722233029	65.19388516	15.01653375
	60.15533981	0	0	100.5242718	108.2912621	61.06796117	52.05825243	8.893203883	82.19417476	0	0	0	0	0	97.72815534	48.01941748	23.80582524	75.04854369	21.00970874	75.98058252	7.650485437	0	0	0	0	7.029126214	70.0776699	15.00970874	5.786407767	0	7.067961165

AMP1CPR2ERY2FUS3TMP1	AMP	CPR	ERY	FUS	TMP	AMP+CPR	AMP+ERY	AMP+FUS	AMP+TMP	CPR+ERY	CPR+FUS	CPR+TMP	ERY+FUS	ERY+TMP	FUS+TMP	AMP+CPR+ERY	AMP+CPR+FUS	AMP+CPR+TMP	AMP+ERY+FUS	AMP+ERY+TMP	AMP+FUS+TMP	CPR+ERY+FUS	CPR+ERY+TMP	CPR+FUS+TMP	ERY+FUS+TMP	AMP+CPR+ERY+FUS	AMP+CPR+ERY+TMP	AMP+CPR+FUS+TMP	AMP+ERY+FUS+TMP	CPR+ERY+FUS+TMP	AMP+CPR+ERY+FUS+TMP
	68.54228943	109.8222638	103.8353601	98.64603482	103.9915597	64.32213821	48.56690698	46.66783893	75.85721822	99.34518241	104.7333957	110.121609	106.828812	113.414406	75.01318797	52.22437137	53.34974503	78.38930895	35.97678917	82.89080359	57.85123967	95.45369504	99.94387278	71.20673527	85.8746492	37.5945138	76.98259188	56.16317918	38.15720063	68.51262862	35.097591
	60.57180851	103.7561455	102.1828909	101.7952128	100.4654255	58.11170213	46.40957447	52.06117021	84.77393617	106.5880039	103.7561455	109.4198623	105.6440511	110.993117	69.6143617	50.99734043	53.59042553	78.39095745	46.47606383	71.74202128	66.68882979	92.4287119	99.03638151	76.06686332	72.92035398	37.43351064	75.73138298	58.97606383	54.45478723	75.12291052	40.89095745
	49.54289049	83.55704698	88.62788963	112.410037	112.410037	52.03267847	57.01225443	34.91538611	98.40497958	97.87472036	99.06785981	113.3855332	102.945563	107.7181208	78.48667574	59.19081891	43.62964404	102.7621085	34.29293912	90.00194515	56.38980743	95.48844146	105.033557	83.85533184	77.59134974	24.3337872	92.80295662	66.66018284	45.80820852	87.73305505	39.27251507
	60.15533981	0	0	108.2912621	110.776699	61.06796117	52.05825243	36.83495146	94.93203883	0	0	0	0	0	83.12621359	48.01941748	43.3592233	101.1456311	34.34951456	91.2038835	49.26213592	0	0	0	0	26.27184466	98.34951456	61.06796117	44.60194175	0	37.76699029

AMP1CPR2ERY2FUS3TMP2	AMP	CPR	ERY	FUS	TMP	AMP+CPR	AMP+ERY	AMP+FUS	AMP+TMP	CPR+ERY	CPR+FUS	CPR+TMP	ERY+FUS	ERY+TMP	FUS+TMP	AMP+CPR+ERY	AMP+CPR+FUS	AMP+CPR+TMP	AMP+ERY+FUS	AMP+ERY+TMP	AMP+FUS+TMP	CPR+ERY+FUS	CPR+ERY+TMP	CPR+FUS+TMP	ERY+FUS+TMP	AMP+CPR+ERY+FUS	AMP+CPR+ERY+TMP	AMP+CPR+FUS+TMP	AMP+ERY+FUS+TMP	CPR+ERY+FUS+TMP	AMP+CPR+ERY+FUS+TMP
	68.54228943	109.8222638	103.8353601	98.64603482	99.49006506	64.32213821	48.56690698	46.66783893	83.73483383	99.34518241	104.7333957	111.3189897	106.828812	120.2993452	86.26692456	52.22437137	53.34974503	75.01318797	35.97678917	82.04677334	42.13117637	95.45369504	99.34518241	85.27595884	93.95696913	37.5945138	80.92139968	55.60049235	34.53490417	79.28905519	40.16177246
	60.57180851	103.7561455	102.1828909	101.7952128	101.5292553	58.11170213	46.40957447	52.06117021	88.76329787	106.5880039	103.7561455	116.027532	105.6440511	119.1740413	82.6462766	50.99734043	53.59042553	85.30585106	46.47606383	83.44414894	52.85904255	92.4287119	105.3294002	85.19174041	89.28220256	37.43351064	74.66755319	64.56117021	47.27393617	84.87708948	58.97606383
	49.54289049	83.55704698	88.62788963	112.410037	109.297802	52.03267847	57.01225443	34.91538611	102.1396615	97.87472036	99.06785981	119.0529456	102.945563	112.7889635	95.2927446	59.19081891	43.62964404	95.2927446	34.29293912	100.8947676	45.49698502	95.48844146	108.9112603	92.50559284	99.66442953	24.3337872	86.57848668	69.77241782	34.29293912	93.10216257	51.09900798
	60.15533981	0	0	108.2912621	115.4368932	61.06796117	52.05825243	36.83495146	103.631068	0	0	0	0	0	89.65048544	48.01941748	43.3592233	97.10679612	34.34951456	98.34951456	46.77669903	0	0	0	0	26.27184466	90.89320388	83.4368932	37.47572816	0	41.49514563

AMP1CPR2ERY2FUS3TMP3	AMP	CPR	ERY	FUS	TMP	AMP+CPR	AMP+ERY	AMP+FUS	AMP+TMP	CPR+ERY	CPR+FUS	CPR+TMP	ERY+FUS	ERY+TMP	FUS+TMP	AMP+CPR+ERY	AMP+CPR+FUS	AMP+CPR+TMP	AMP+ERY+FUS	AMP+ERY+TMP	AMP+FUS+TMP	CPR+ERY+FUS	CPR+ERY+TMP	CPR+FUS+TMP	ERY+FUS+TMP	AMP+CPR+ERY+FUS	AMP+CPR+ERY+TMP	AMP+CPR+FUS+TMP	AMP+ERY+FUS+TMP	CPR+ERY+FUS+TMP	AMP+CPR+ERY+FUS+TMP
	68.54228943	109.8222638	103.8353601	98.64603482	108.2117109	64.32213821	48.56690698	46.66783893	75.5758748	99.34518241	104.7333957	124.4901777	106.828812	121.7960711	105.9609636	52.22437137	53.34974503	62.91542114	35.97678917	79.79602602	34.00738526	95.45369504	112.2170253	105.9307764	119.1019645	37.5945138	52.7870582	36.50430807	22.47230526	86.47333957	30.59609636
	60.57180851	103.7561455	102.1828909	101.7952128	100.4654255	58.11170213	46.40957447	52.06117021	76.26329787	106.5880039	103.7561455	123.2645034	105.6440511	124.5231072	105.2526596	50.99734043	53.59042553	74.93351064	46.47606383	80.78457447	37.25066489	92.4287119	112.5663717	102.8121927	109.4198623	37.43351064	71.74202128	42.42021277	28.47406915	89.28220256	50.71338298
	49.54289049	83.55704698	88.62788963	112.410037	115.8334954	52.03267847	57.01225443	34.91538611	74.12954678	97.87472036	99.06785981	119.3512304	102.945563	112.7889635	109.920249	59.19081891	43.62964404	63.23672437	34.29293912	93.11418012	41.33437074	95.48844146	105.3318419	111.2975391	111.8941089	24.3337872	72.57342929	45.69149971	27.32931336	97.27815063	25.77319588
	60.15533981	0	0	108.2912621	108.2912621	61.06796117	52.05825243	36.83495146	82.19417476	0	0	0	0	0	111.0873786	48.01941748	43.3592233	75.04854369	34.34951456	75.98058252	28.77669903	0	0	0	0	26.27184466	70.0776699	29.70873786	17.59223301	0	21.63106796

AMP1CPR2ERY3FUS1TMP1	AMP	CPR	ERY	FUS	TMP	AMP+CPR	AMP+ERY	AMP+FUS	AMP+TMP	CPR+ERY	CPR+FUS	CPR+TMP	ERY+FUS	ERY+TMP	FUS+TMP	AMP+CPR+ERY	AMP+CPR+FUS	AMP+CPR+TMP	AMP+ERY+FUS	AMP+ERY+TMP	AMP+FUS+TMP	CPR+ERY+FUS	CPR+ERY+TMP	CPR+FUS+TMP	ERY+FUS+TMP	AMP+CPR+ERY+FUS	AMP+CPR+ERY+TMP	AMP+CPR+FUS+TMP	AMP+ERY+FUS+TMP	CPR+ERY+FUS+TMP	AMP+CPR+ERY+FUS+TMP
	68.54228943	109.8222638	95.34686971	70.23034992	103.9915597	64.32213821	54.47511869	13.18797257	75.85721822	95.75304022	70.92609916	110.121609	80.79526227	114.6362098	64.91999297	48.03938808	19.65887111	78.38930895	4.184983295	76.13856163	10.09319501	60.74836296	104.4340505	62.84377923	60.08833249	21.31176367	80.64005627	39.88042905	24.44170916	57.75491113	33.12818709
	60.57180851	103.7561455	99.12713472	82.6462766	100.4654255	58.11170213	50.73138298	3.390957447	84.77393617	102.4975418	72.546706	109.4198623	90.32258065	114.914611	54.38829787	44.88031915	39.7606383	78.39095745	8.97606383	74.1356383	10.92087766	67.82694199	104.0707965	84.18879056	56.31878558	17.15425532	74.66755319	44.34840426	20.49534574	71.97140413	41.15691489
	49.54289049	83.55704698	108.9759282	74.01283797	112.410037	52.03267847	53.78331064	2.120210076	98.40497958	95.78672632	56.54362416	113.3855332	93.63525092	120.3998368	71.21182649	58.25714841	3.365104065	102.7621085	2.742657071	110.2314725	5.23244505	56.41312453	110.9992543	56.54362416	62.95389637	4.921221552	101.828438	46.00272321	4.921221552	57.14019389	36.04357129
	60.15533981	0	0	81.26213592	110.776699	61.06796117	53.9223301	5.475728155	94.93203883	0	0	0	0	0	72.89320388	48.97087379	4.233009709	101.1456311	3.300970874	100.2135922	1.747572816	0	0	0	0	2.990291262	94	34.05825243	4.854368932	0	35.30097087

AMP1CPR2ERY3FUS1TMP2	AMP	CPR	ERY	FUS	TMP	AMP+CPR	AMP+ERY	AMP+FUS	AMP+TMP	CPR+ERY	CPR+FUS	CPR+TMP	ERY+FUS	ERY+TMP	FUS+TMP	AMP+CPR+ERY	AMP+CPR+FUS	AMP+CPR+TMP	AMP+ERY+FUS	AMP+ERY+TMP	AMP+FUS+TMP	CPR+ERY+FUS	CPR+ERY+TMP	CPR+FUS+TMP	ERY+FUS+TMP	AMP+CPR+ERY+FUS	AMP+CPR+ERY+TMP	AMP+CPR+FUS+TMP	AMP+ERY+FUS+TMP	CPR+ERY+FUS+TMP	AMP+CPR+ERY+FUS+TMP
	68.54228943	109.8222638	95.34686971	70.23034992	99.49006506	64.32213821	54.47511869	13.18797257	83.73483383	95.75304022	70.92609916	111.3189897	80.79526227	114.9746193	64.63864955	48.03938808	19.65887111	75.01318797	4.184983295	85.98558115	9.530508177	60.74836296	115.8091674	72.42282507	72.2715736	21.31176367	75.01318797	6.963249516	7.561104273	66.43592142	3.024441709
	60.57180851	103.7561455	99.12713472	82.6462766	101.5292553	58.11170213	50.73138298	3.390957447	88.76329787	102.4975418	72.546706	116.027532	90.32258065	115.2182163	55.25265957	44.88031915	39.7606383	85.30585106	8.97606383	84.24202128	8.377659574	67.82694199	107.8466077	86.3913471	65.73055028	17.15425532	79.18882979	16.77194149	6.931515957	79.15437561	11.6356383
	49.54289049	83.55704698	108.9759282	74.01283797	109.297802	52.03267847	53.78331064	2.120210076	102.1396615	95.78672632	56.54362416	119.0529456	93.63525092	125.2957976	60.94145108	58.25714841	3.365104065	95.2927446	2.742657071	109.297802	2.120210076	56.41312453	117.2632364	63.10589113	80.57935537	4.921221552	94.9815211	3.98755106	2.120210076	57.73676361	2.120210076
	60.15533981	0	0	81.26213592	115.4368932	61.06796117	53.9223301	5.475728155	103.631068	0	0	0	0	0	72.58252427	48.97087379	4.233009709	97.10679612	3.300970874	107.3592233	2.058252427	0	0	0	0	2.990291262	86.85436893	2.058252427	1.72815534	0	2.67961165

AMP1CPR2ERY3FUS1TMP3	AMP	CPR	ERY	FUS	TMP	AMP+CPR	AMP+ERY	AMP+FUS	AMP+TMP	CPR+ERY	CPR+FUS	CPR+TMP	ERY+FUS	ERY+TMP	FUS+TMP	AMP+CPR+ERY	AMP+CPR+FUS	AMP+CPR+TMP	AMP+ERY+FUS	AMP+ERY+TMP	AMP+FUS+TMP	CPR+ERY+FUS	CPR+ERY+TMP	CPR+FUS+TMP	ERY+FUS+TMP	AMP+CPR+ERY+FUS	AMP+CPR+ERY+TMP	AMP+CPR+FUS+TMP	AMP+ERY+FUS+TMP	CPR+ERY+FUS+TMP	AMP+CPR+ERY+FUS+TMP
	68.54228943	109.8222638	95.34686971	70.23034992	108.2117109	64.32213821	54.47511869	13.18797257	75.5758748	95.75304022	70.92609916	124.4901777	80.79526227	115.6514382	73.92298224	48.03938808	19.65887111	62.91542114	4.184983295	76.13856163	7.279760858	60.74836296	116.707203	77.81103835	83.60494755	21.31176367	54.19377528	17.12678038	8.123791103	72.42282507	13.43414806
	60.57180851	103.7561455	99.12713472	82.6462766	100.4654255	58.11170213	50.73138298	3.390957447	76.26329787	102.4975418	72.546706	123.2645034	90.32258065	107.0208729	59.50797872	44.88031915	39.7606383	74.93351064	8.97606383	79.45478723	15.97406915	67.82694199	110.6784661	90.79646018	81.82163188	17.15425532	68.28457447	22.62300532	12.10106383	72.546706	9.973404255
	49.54289049	83.55704698	108.9759282	74.01283797	115.8334954	52.03267847	53.78331064	2.120210076	74.12954678	95.78672632	56.54362416	119.3512304	93.63525092	117.1358629	68.41081502	58.25714841	3.365104065	63.23672437	2.742657071	98.40497958	1.186539584	56.41312453	116.3683818	64.00074571	90.04487964	4.921221552	73.19587629	4.921221552	2.431433573	58.79940343	23.2834079
	60.15533981	0	0	81.26213592	108.2912621	61.06796117	53.9223301	5.475728155	82.19417476	0	0	0	0	0	74.75728155	48.97087379	4.233009709	75.04854369	3.300970874	82.19417476	4.54368932	0	0	0	0	2.990291262	57.33980583	1.747572816	2.349514563	0	3.514563107

AMP1CPR2ERY3FUS2TMP1	AMP	CPR	ERY	FUS	TMP	AMP+CPR	AMP+ERY	AMP+FUS	AMP+TMP	CPR+ERY	CPR+FUS	CPR+TMP	ERY+FUS	ERY+TMP	FUS+TMP	AMP+CPR+ERY	AMP+CPR+FUS	AMP+CPR+TMP	AMP+ERY+FUS	AMP+ERY+TMP	AMP+FUS+TMP	CPR+ERY+FUS	CPR+ERY+TMP	CPR+FUS+TMP	ERY+FUS+TMP	AMP+CPR+ERY+FUS	AMP+CPR+ERY+TMP	AMP+CPR+FUS+TMP	AMP+ERY+FUS+TMP	CPR+ERY+FUS+TMP	AMP+CPR+ERY+FUS+TMP
	68.54228943	109.8222638	95.34686971	85.70423774	103.9915597	64.32213821	54.47511869	32.03798136	75.85721822	95.75304022	81.98316183	110.121609	84.17935702	114.6362098	66.0101987	48.03938808	35.13275892	78.38930895	26.41111306	76.13856163	28.34534904	72.70346118	104.4340505	59.85032741	69.2893401	27.53648672	80.64005627	53.38491296	22.47230526	57.15622077	53.38491296
	60.57180851	103.7561455	99.12713472	95.94414894	100.4654255	58.11170213	50.73138298	24.48470745	84.77393617	102.4975418	85.50639135	109.4198623	93.05502846	114.914611	62.96542553	44.88031915	23.80319149	78.39095745	15.7081117	74.1356383	34.85704787	69.45919371	104.0707965	61.84857424	74.23149905	20.07978723	74.66755319	36.1037234	27.14428191	61.21927237	37.69946809
	49.54289049	83.55704698	108.9759282	96.53763859	112.410037	52.03267847	53.78331064	11.76813849	98.40497958	95.78672632	79.08277405	113.3855332	103.75357	120.3998368	67.47714452	58.25714841	30.13032484	102.7621085	6.788562536	110.2314725	34.4874538	79.97762864	110.9992543	60.5891126	78.29457364	24.83952538	101.828438	36.04357129	31.37521883	54.3251305	39.27251507
	60.15533981	0	0	100.5242718	110.776699	61.06796117	53.9223301	8.893203883	94.93203883	0	0	0	0	0	66.36893204	48.97087379	23.80582524	101.1456311	21.94174757	100.2135922	39.33980583	0	0	0	0	21.32038835	94	42.75728155	19.66990291	0	49.59223301

AMP1CPR2ERY3FUS2TMP2	AMP	CPR	ERY	FUS	TMP	AMP+CPR	AMP+ERY	AMP+FUS	AMP+TMP	CPR+ERY	CPR+FUS	CPR+TMP	ERY+FUS	ERY+TMP	FUS+TMP	AMP+CPR+ERY	AMP+CPR+FUS	AMP+CPR+TMP	AMP+ERY+FUS	AMP+ERY+TMP	AMP+FUS+TMP	CPR+ERY+FUS	CPR+ERY+TMP	CPR+FUS+TMP	ERY+FUS+TMP	AMP+CPR+ERY+FUS	AMP+CPR+ERY+TMP	AMP+CPR+FUS+TMP	AMP+ERY+FUS+TMP	CPR+ERY+FUS+TMP	AMP+CPR+ERY+FUS+TMP
	68.54228943	109.8222638	95.34686971	85.70423774	99.49006506	64.32213821	54.47511869	32.03798136	83.73483383	95.75304022	81.98316183	111.3189897	84.17935702	114.9746193	67.17074028	48.03938808	35.13275892	75.01318797	26.41111306	85.98558115	27.78266221	72.70346118	115.8091674	64.34050514	85.8714044	27.53648672	75.01318797	33.69087392	36.78565149	69.42937325	38.47371197
	60.57180851	103.7561455	99.12713472	95.94414894	101.5292553	58.11170213	50.73138298	24.48470745	88.76329787	102.4975418	85.50639135	116.027532	93.05502846	115.2182163	62.43351064	44.88031915	23.80319149	85.30585106	15.7081117	84.24202128	29.27194149	69.45919371	107.8466077	73.23500492	88.19734345	20.07978723	79.18882979	24.66755319	25.81449468	70.97345133	52.85904255
	49.54289049	83.55704698	108.9759282	96.53763859	109.297802	52.03267847	53.78331064	11.76813849	102.1396615	95.78672632	79.08277405	119.0529456	103.75357	125.2957976	74.63528496	58.25714841	30.13032484	95.2927446	6.788562536	109.297802	21.10484342	79.97762864	117.2632364	76.39821029	85.14891881	24.83952538	94.9815211	21.72729041	41.02314725	61.78225205	31.18070414
	60.15533981	0	0	100.5242718	115.4368932	61.06796117	53.9223301	8.893203883	103.631068	0	0	0	0	0	63.86407767	48.97087379	23.80582524	97.10679612	21.94174757	107.3592233	25.26213592	0	0	0	0	21.32038835	86.85436893	28.15533981	24.01941748	0	33.74757282

AMP1CPR2ERY3FUS2TMP3	AMP	CPR	ERY	FUS	TMP	AMP+CPR	AMP+ERY	AMP+FUS	AMP+TMP	CPR+ERY	CPR+FUS	CPR+TMP	ERY+FUS	ERY+TMP	FUS+TMP	AMP+CPR+ERY	AMP+CPR+FUS	AMP+CPR+TMP	AMP+ERY+FUS	AMP+ERY+TMP	AMP+FUS+TMP	CPR+ERY+FUS	CPR+ERY+TMP	CPR+FUS+TMP	ERY+FUS+TMP	AMP+CPR+ERY+FUS	AMP+CPR+ERY+TMP	AMP+CPR+FUS+TMP	AMP+ERY+FUS+TMP	CPR+ERY+FUS+TMP	AMP+CPR+ERY+FUS+TMP
	68.54228943	109.8222638	95.34686971	85.70423774	108.2117109	64.32213821	54.47511869	32.03798136	75.5758748	95.75304022	81.98316183	124.4901777	84.17935702	115.6514382	81.48408651	48.03938808	35.13275892	62.91542114	26.41111306	76.13856163	1.89906805	72.70346118	116.707203	68.83068288	98.39255499	27.53648672	54.19377528	8.93265342	3.305785124	67.63330215	26.93863197
	60.57180851	103.7561455	99.12713472	95.94414894	100.4654255	58.11170213	50.73138298	24.48470745	76.26329787	102.4975418	85.50639135	123.2645034	93.05502846	107.0208729	78.65691489	44.88031915	23.80319149	74.93351064	15.7081117	79.45478723	14.91023936	69.45919371	110.6784661	77.26647001	93.9658444	20.07978723	68.28457447	13.31449468	6.399601064	68.82989184	27.67619681
	49.54289049	83.55704698	108.9759282	96.53763859	115.8334954	52.03267847	53.78331064	11.76813849	74.12954678	95.78672632	79.08277405	119.3512304	103.75357	117.1358629	83.4662517	58.25714841	30.13032484	63.23672437	6.788562536	98.40497958	9.589574013	79.97762864	116.3683818	79.38105891	92.65605875	24.83952538	73.19587629	20.48239642	6.166115542	77.59134974	21.41606691
	60.15533981	0	0	100.5242718	108.2912621	61.06796117	53.9223301	8.893203883	82.19417476	0	0	0	0	0	97.72815534	48.97087379	23.80582524	75.04854369	21.94174757	82.19417476	7.650485437	0	0	0	0	21.32038835	57.33980583	15.00970874	12	0	29.70873786

AMP1CPR2ERY3FUS3TMP1	AMP	CPR	ERY	FUS	TMP	AMP+CPR	AMP+ERY	AMP+FUS	AMP+TMP	CPR+ERY	CPR+FUS	CPR+TMP	ERY+FUS	ERY+TMP	FUS+TMP	AMP+CPR+ERY	AMP+CPR+FUS	AMP+CPR+TMP	AMP+ERY+FUS	AMP+ERY+TMP	AMP+FUS+TMP	CPR+ERY+FUS	CPR+ERY+TMP	CPR+FUS+TMP	ERY+FUS+TMP	AMP+CPR+ERY+FUS	AMP+CPR+ERY+TMP	AMP+CPR+FUS+TMP	AMP+ERY+FUS+TMP	CPR+ERY+FUS+TMP	AMP+CPR+ERY+FUS+TMP
	68.54228943	109.8222638	95.34686971	98.64603482	103.9915597	64.32213821	54.47511869	46.66783893	75.85721822	95.75304022	104.7333957	110.121609	102.1150592	114.6362098	75.01318797	48.03938808	53.34974503	78.38930895	40.12660454	76.13856163	57.85123967	104.1347053	104.4340505	71.20673527	79.44162437	48.56690698	80.64005627	56.16317918	57.28855284	68.21328344	57.28855284
	60.57180851	103.7561455	99.12713472	101.7952128	100.4654255	58.11170213	50.73138298	52.06117021	84.77393617	102.4975418	103.7561455	109.4198623	107.9316888	114.914611	69.6143617	44.88031915	53.59042553	78.39095745	43.28457447	74.1356383	66.68882979	101.553589	104.0707965	76.06686332	84.25047438	51.19680851	74.66755319	58.97606383	66.42287234	73.86430678	56.05053191
	49.54289049	83.55704698	108.9759282	112.410037	112.410037	52.03267847	53.78331064	34.91538611	98.40497958	95.78672632	99.06785981	113.3855332	115.1774786	120.3998368	78.48667574	58.25714841	43.62964404	102.7621085	32.11437464	110.2314725	56.38980743	105.3318419	110.9992543	83.85533184	94.94084047	47.36432601	101.828438	66.66018284	72.88465279	86.5398956	74.75199378
	60.15533981	0	0	108.2912621	110.776699	61.06796117	53.9223301	36.83495146	94.93203883	0	0	0	0	0	83.12621359	48.97087379	43.3592233	101.1456311	34.03883495	100.2135922	49.26213592	0	0	0	0	43.66990291	94	61.06796117	60.13592233	0	63.55339806

AMP1CPR2ERY3FUS3TMP2	AMP	CPR	ERY	FUS	TMP	AMP+CPR	AMP+ERY	AMP+FUS	AMP+TMP	CPR+ERY	CPR+FUS	CPR+TMP	ERY+FUS	ERY+TMP	FUS+TMP	AMP+CPR+ERY	AMP+CPR+FUS	AMP+CPR+TMP	AMP+ERY+FUS	AMP+ERY+TMP	AMP+FUS+TMP	CPR+ERY+FUS	CPR+ERY+TMP	CPR+FUS+TMP	ERY+FUS+TMP	AMP+CPR+ERY+FUS	AMP+CPR+ERY+TMP	AMP+CPR+FUS+TMP	AMP+ERY+FUS+TMP	CPR+ERY+FUS+TMP	AMP+CPR+ERY+FUS+TMP
	68.54228943	109.8222638	95.34686971	98.64603482	99.49006506	64.32213821	54.47511869	46.66783893	83.73483383	95.75304022	104.7333957	111.3189897	102.1150592	114.9746193	86.26692456	48.03938808	53.34974503	75.01318797	40.12660454	85.98558115	42.13117637	104.1347053	115.8091674	85.27595884	106.1759729	48.56690698	75.01318797	55.60049235	35.66027783	81.08512629	61.79004748
	60.57180851	103.7561455	99.12713472	101.7952128	101.5292553	58.11170213	50.73138298	52.06117021	88.76329787	102.4975418	103.7561455	116.027532	107.9316888	115.2182163	82.6462766	44.88031915	53.59042553	85.30585106	43.28457447	84.24202128	52.85904255	101.553589	107.8466077	85.19174041	101.8595825	51.19680851	79.18882979	64.56117021	43.81648936	85.50639135	66.42287234
	49.54289049	83.55704698	108.9759282	112.410037	109.297802	52.03267847	53.78331064	34.91538611	102.1396615	95.78672632	99.06785981	119.0529456	115.1774786	125.2957976	95.2927446	58.25714841	43.62964404	95.2927446	32.11437464	109.297802	45.49698502	105.3318419	117.2632364	92.50559284	106.6911465	47.36432601	94.9815211	69.77241782	43.00719704	96.68158091	79.10912274
	60.15533981	0	0	108.2912621	115.4368932	61.06796117	53.9223301	36.83495146	103.631068	0	0	0	0	0	89.65048544	48.97087379	43.3592233	97.10679612	34.03883495	107.3592233	46.77669903	0	0	0	0	43.66990291	86.85436893	83.4368932	46.77669903	0	76.29126214

AMP1CPR2ERY3FUS3TMP3	AMP	CPR	ERY	FUS	TMP	AMP+CPR	AMP+ERY	AMP+FUS	AMP+TMP	CPR+ERY	CPR+FUS	CPR+TMP	ERY+FUS	ERY+TMP	FUS+TMP	AMP+CPR+ERY	AMP+CPR+FUS	AMP+CPR+TMP	AMP+ERY+FUS	AMP+ERY+TMP	AMP+FUS+TMP	CPR+ERY+FUS	CPR+ERY+TMP	CPR+FUS+TMP	ERY+FUS+TMP	AMP+CPR+ERY+FUS	AMP+CPR+ERY+TMP	AMP+CPR+FUS+TMP	AMP+ERY+FUS+TMP	CPR+ERY+FUS+TMP	AMP+CPR+ERY+FUS+TMP
	68.54228943	109.8222638	95.34686971	98.64603482	108.2117109	64.32213821	54.47511869	46.66783893	75.5758748	95.75304022	104.7333957	124.4901777	102.1150592	115.6514382	105.9609636	48.03938808	53.34974503	62.91542114	40.12660454	76.13856163	34.00738526	104.1347053	116.707203	105.9307764	114.2978003	48.56690698	54.19377528	36.50430807	34.00738526	108.9242283	55.03780552
	60.57180851	103.7561455	99.12713472	101.7952128	100.4654255	58.11170213	50.73138298	52.06117021	76.26329787	102.4975418	103.7561455	123.2645034	107.9316888	107.0208729	105.2526596	44.88031915	53.59042553	74.93351064	43.28457447	79.45478723	37.25066489	101.553589	110.6784661	102.8121927	114.3074004	51.19680851	68.28457447	42.42021277	48.6037234	110.3638151	46.21010638
	49.54289049	83.55704698	108.9759282	112.410037	115.8334954	52.03267847	53.78331064	34.91538611	74.12954678	95.78672632	99.06785981	119.3512304	115.1774786	117.1358629	109.920249	58.25714841	43.62964404	63.23672437	32.11437464	98.40497958	41.33437074	105.3318419	116.3683818	111.2975391	128.5597715	47.36432601	73.19587629	45.69149971	35.73234779	114.2803878	53.27757246
	60.15533981	0	0	108.2912621	108.2912621	61.06796117	53.9223301	36.83495146	82.19417476	0	0	0	0	0	111.0873786	48.97087379	43.3592233	75.04854369	34.03883495	82.19417476	28.77669903	0	0	0	0	43.66990291	57.33980583	29.70873786	29.70873786	0	46.77669903

AMP1CPR3ERY1FUS1TMP1	AMP	CPR	ERY	FUS	TMP	AMP+CPR	AMP+ERY	AMP+FUS	AMP+TMP	CPR+ERY	CPR+FUS	CPR+TMP	ERY+FUS	ERY+TMP	FUS+TMP	AMP+CPR+ERY	AMP+CPR+FUS	AMP+CPR+TMP	AMP+ERY+FUS	AMP+ERY+TMP	AMP+FUS+TMP	CPR+ERY+FUS	CPR+ERY+TMP	CPR+FUS+TMP	ERY+FUS+TMP	AMP+CPR+ERY+FUS	AMP+CPR+ERY+TMP	AMP+CPR+FUS+TMP	AMP+ERY+FUS+TMP	CPR+ERY+FUS+TMP	AMP+CPR+ERY+FUS+TMP
	68.54228943	94.67005076	95.26991384	70.23034992	103.9915597	77.82662212	52.54088271	13.18797257	75.85721822	104.4839255	83.43908629	112.605753	60.3833304	89.36170213	64.91999297	74.16915773	30.31475295	83.45349042	6.400562687	78.38930895	10.09319501	48.9213198	84.85617597	69.90270728	55.91700369	23.03499209	78.67065237	45.78864076	57.92157552	68.2106599	49.72744857
	60.57180851	95.18026565	101.2632979	82.6462766	100.4654255	58.64361702	47.7393617	3.390957447	84.77393617	88.19734345	54.00379507	109.4497154	63.49734043	96.21010638	54.38829787	55.25265957	25.93085106	91.95478723	7.313829787	74.1356383	10.92087766	50.66413662	94.57305503	63.41555977	55.98404255	26.99468085	78.125	53.39095745	6.848404255	51.57495256	40.29255319
	49.54289049	104.0799674	94.35907411	74.01283797	112.410037	78.48667574	51.09900798	2.120210076	98.40497958	92.98245614	67.19706242	125.2957976	74.94650846	109.297802	71.21182649	75.06321727	31.18070414	103.073332	3.053880568	87.51215717	5.23244505	50.04079967	106.3647491	71.58302734	72.45672048	21.41606691	102.7621085	60.63022758	3.365104065	53.95756834	37.28846528
	60.15533981	0	97.72815534	81.26213592	110.776699	89.02912621	51.74757282	5.475728155	94.93203883	0	0	0	76	112.6407767	72.89320388	70.38834951	40.25242718	105.184466	3.300970874	89.65048544	1.747572816	0	0	0	71.33980583	23.18446602	103.0097087	52.69902913	-0.116504854	0	52.0776699

AMP1CPR3ERY1FUS1TMP2	AMP	CPR	ERY	FUS	TMP	AMP+CPR	AMP+ERY	AMP+FUS	AMP+TMP	CPR+ERY	CPR+FUS	CPR+TMP	ERY+FUS	ERY+TMP	FUS+TMP	AMP+CPR+ERY	AMP+CPR+FUS	AMP+CPR+TMP	AMP+ERY+FUS	AMP+ERY+TMP	AMP+FUS+TMP	CPR+ERY+FUS	CPR+ERY+TMP	CPR+FUS+TMP	ERY+FUS+TMP	AMP+CPR+ERY+FUS	AMP+CPR+ERY+TMP	AMP+CPR+FUS+TMP	AMP+ERY+FUS+TMP	CPR+ERY+FUS+TMP	AMP+CPR+ERY+FUS+TMP
	68.54228943	94.67005076	95.26991384	70.23034992	99.49006506	77.82662212	52.54088271	13.18797257	83.73483383	104.4839255	83.43908629	113.9593909	60.3833304	87.95498505	64.63864955	74.16915773	30.31475295	89.64304554	6.400562687	89.36170213	9.530508177	48.9213198	104.822335	83.10067682	58.49909443	23.03499209	75.85721822	41.00580271	7.561104273	51.96700508	27.78266221
	60.57180851	95.18026565	101.2632979	82.6462766	101.5292553	58.64361702	47.7393617	3.390957447	88.76329787	88.19734345	54.00379507	112.7893738	63.49734043	94.6143617	55.25265957	55.25265957	25.93085106	83.44414894	7.313829787	83.97606383	8.377659574	50.66413662	99.43074004	71.30929791	53.85638298	26.99468085	86.1037234	44.6143617	6.582446809	44.85768501	26.19680851
	49.54289049	104.0799674	94.35907411	74.01283797	109.297802	78.48667574	51.09900798	2.120210076	102.1396615	92.98245614	67.19706242	127.5805794	74.94650846	109.920249	60.94145108	75.06321727	31.18070414	117.7008364	3.053880568	97.16008559	2.120210076	50.04079967	118.7678499	67.66625867	69.03326201	21.41606691	95.9151916	29.19665435	1.808986578	46.63402693	3.676327563
	60.15533981	0	97.72815534	81.26213592	115.4368932	89.02912621	51.74757282	5.475728155	103.631068	0	0	0	76	119.1650485	72.58252427	70.38834951	40.25242718	93.68932039	3.300970874	91.2038835	2.058252427	0	0	0	65.74757282	23.18446602	101.7669903	27.22330097	1.747572816	0	6.621359223

AMP1CPR3ERY1FUS1TMP3	AMP	CPR	ERY	FUS	TMP	AMP+CPR	AMP+ERY	AMP+FUS	AMP+TMP	CPR+ERY	CPR+FUS	CPR+TMP	ERY+FUS	ERY+TMP	FUS+TMP	AMP+CPR+ERY	AMP+CPR+FUS	AMP+CPR+TMP	AMP+ERY+FUS	AMP+ERY+TMP	AMP+FUS+TMP	CPR+ERY+FUS	CPR+ERY+TMP	CPR+FUS+TMP	ERY+FUS+TMP	AMP+CPR+ERY+FUS	AMP+CPR+ERY+TMP	AMP+CPR+FUS+TMP	AMP+ERY+FUS+TMP	CPR+ERY+FUS+TMP	AMP+CPR+ERY+FUS+TMP
	68.54228943	94.67005076	95.26991384	70.23034992	108.2117109	77.82662212	52.54088271	13.18797257	75.5758748	104.4839255	83.43908629	121.0659898	60.3833304	91.04976262	73.92298224	74.16915773	30.31475295	76.41990505	6.400562687	79.23333919	7.279760858	48.9213198	104.822335	75.31725888	67.4520837	23.03499209	69.10497626	50.88799015	5.556532442	62.45769882	22.71848075
	60.57180851	95.18026565	101.2632979	82.6462766	100.4654255	58.64361702	47.7393617	3.390957447	76.26329787	88.19734345	54.00379507	106.7172676	63.49734043	94.88031915	59.50797872	55.25265957	25.93085106	74.93351064	7.313829787	80.25265957	15.97406915	50.66413662	99.12713472	67.66603416	51.99468085	26.99468085	75.19946809	50.19946809	2.32712766	55.52182163	17.03789894
	49.54289049	104.0799674	94.35907411	74.01283797	115.8334954	78.48667574	51.09900798	2.120210076	74.12954678	92.98245614	67.19706242	123.0110159	74.94650846	108.3641315	68.41081502	75.06321727	31.18070414	74.75199378	3.053880568	73.81832328	1.186539584	50.04079967	108.3231334	70.7874337	70.278156	21.41606691	70.70608831	35.1099008	1.808986578	51.67278662	3.365104065
	60.15533981	0	97.72815534	81.26213592	108.2912621	89.02912621	51.74757282	5.475728155	82.19417476	0	0	0	76	111.7087379	74.75728155	70.38834951	40.25242718	65.41747573	3.300970874	64.48543689	4.54368932	0	0	0	61.08737864	23.18446602	75.3592233	38.31067961	2.67961165	0	15.63106796

AMP1CPR3ERY1FUS2TMP1	AMP	CPR	ERY	FUS	TMP	AMP+CPR	AMP+ERY	AMP+FUS	AMP+TMP	CPR+ERY	CPR+FUS	CPR+TMP	ERY+FUS	ERY+TMP	FUS+TMP	AMP+CPR+ERY	AMP+CPR+FUS	AMP+CPR+TMP	AMP+ERY+FUS	AMP+ERY+TMP	AMP+FUS+TMP	CPR+ERY+FUS	CPR+ERY+TMP	CPR+FUS+TMP	ERY+FUS+TMP	AMP+CPR+ERY+FUS	AMP+CPR+ERY+TMP	AMP+CPR+FUS+TMP	AMP+ERY+FUS+TMP	CPR+ERY+FUS+TMP	AMP+CPR+ERY+FUS+TMP
	68.54228943	94.67005076	95.26991384	85.70423774	103.9915597	77.82662212	52.54088271	32.03798136	75.85721822	104.4839255	80.79526227	112.605753	68.82363285	89.36170213	66.0101987	74.16915773	44.34675576	83.45349042	20.78424477	78.38930895	28.34534904	56.02791878	84.85617597	60.49069374	65.20133638	39.56391771	78.67065237	46.59750308	24.16036575	65.16497462	54.2289432
	60.57180851	95.18026565	101.2632979	95.94414894	100.4654255	58.64361702	47.7393617	24.48470745	84.77393617	88.19734345	90.62618596	109.4497154	67.48670213	96.21010638	62.96542553	55.25265957	46.47606383	91.95478723	15.82446809	74.1356383	34.85704787	51.76470588	94.57305503	59.65844402	44.88031915	27.79255319	78.125	47.00797872	31.78191489	54.91461101	32.57978723
	49.54289049	104.0799674	94.35907411	96.53763859	112.410037	78.48667574	51.09900798	11.76813849	98.40497958	92.98245614	95.59363525	125.2957976	63.74246256	109.297802	67.47714452	75.06321727	48.60922	103.073332	6.788562536	87.51215717	34.4874538	61.64830681	106.3647491	79.92656059	63.74246256	25.46197238	102.7621085	46.00272321	1.808986578	51.34638923	41.95681774
	60.15533981	0	97.72815534	100.5242718	110.776699	89.02912621	51.74757282	8.893203883	94.93203883	0	0	0	71.33980583	112.6407767	66.36893204	70.38834951	58.89320388	105.184466	25.04854369	89.65048544	39.33980583	0	0	0	62.01941748	22.87378641	103.0097087	51.76699029	2.058252427	0	45.55339806

AMP1CPR3ERY1FUS2TMP2	AMP	CPR	ERY	FUS	TMP	AMP+CPR	AMP+ERY	AMP+FUS	AMP+TMP	CPR+ERY	CPR+FUS	CPR+TMP	ERY+FUS	ERY+TMP	FUS+TMP	AMP+CPR+ERY	AMP+CPR+FUS	AMP+CPR+TMP	AMP+ERY+FUS	AMP+ERY+TMP	AMP+FUS+TMP	CPR+ERY+FUS	CPR+ERY+TMP	CPR+FUS+TMP	ERY+FUS+TMP	AMP+CPR+ERY+FUS	AMP+CPR+ERY+TMP	AMP+CPR+FUS+TMP	AMP+ERY+FUS+TMP	CPR+ERY+FUS+TMP	AMP+CPR+ERY+FUS+TMP
	68.54228943	94.67005076	95.26991384	85.70423774	99.49006506	77.82662212	52.54088271	32.03798136	83.73483383	104.4839255	80.79526227	113.9593909	68.82363285	87.95498505	67.17074028	74.16915773	44.34675576	89.64304554	20.78424477	89.36170213	27.78266221	56.02791878	104.822335	66.5820643	69.70283102	39.56391771	75.85721822	46.03481625	29.18937929	74.30203046	35.66027783
	60.57180851	95.18026565	101.2632979	95.94414894	101.5292553	58.64361702	47.7393617	24.48470745	88.76329787	88.19734345	90.62618596	112.7893738	67.48670213	94.6143617	62.43351064	55.25265957	46.47606383	83.44414894	15.82446809	83.97606383	29.27194149	51.76470588	99.43074004	59.0512334	52.32712766	27.79255319	86.1037234	56.31648936	25.66489362	54.54079696	32.38031915
	49.54289049	104.0799674	94.35907411	96.53763859	109.297802	78.48667574	51.09900798	11.76813849	102.1396615	92.98245614	95.59363525	127.5805794	63.74246256	109.920249	74.63528496	75.06321727	48.60922	117.7008364	6.788562536	97.16008559	21.10484342	61.64830681	118.7678499	75.03059976	70.900603	25.46197238	95.9151916	44.75782922	22.03851391	59.50632395	33.24255981
	60.15533981	0	97.72815534	100.5242718	115.4368932	89.02912621	51.74757282	8.893203883	103.631068	0	0	0	71.33980583	119.1650485	63.86407767	70.38834951	58.89320388	93.68932039	25.04854369	91.2038835	25.26213592	0	0	0	63.88349515	22.87378641	101.7669903	39.02912621	11.90291262	0	34.05825243

AMP1CPR3ERY1FUS2TMP3	AMP	CPR	ERY	FUS	TMP	AMP+CPR	AMP+ERY	AMP+FUS	AMP+TMP	CPR+ERY	CPR+FUS	CPR+TMP	ERY+FUS	ERY+TMP	FUS+TMP	AMP+CPR+ERY	AMP+CPR+FUS	AMP+CPR+TMP	AMP+ERY+FUS	AMP+ERY+TMP	AMP+FUS+TMP	CPR+ERY+FUS	CPR+ERY+TMP	CPR+FUS+TMP	ERY+FUS+TMP	AMP+CPR+ERY+FUS	AMP+CPR+ERY+TMP	AMP+CPR+FUS+TMP	AMP+ERY+FUS+TMP	CPR+ERY+FUS+TMP	AMP+CPR+ERY+FUS+TMP
	68.54228943	94.67005076	95.26991384	85.70423774	108.2117109	77.82662212	52.54088271	32.03798136	75.5758748	104.4839255	80.79526227	121.0659898	68.82363285	91.04976262	81.48408651	74.16915773	44.34675576	76.41990505	20.78424477	79.23333919	1.89906805	56.02791878	104.822335	68.2106599	67.41691577	39.56391771	69.10497626	48.56690698	3.340953051	76.33248731	31.72147002
	60.57180851	95.18026565	101.2632979	95.94414894	100.4654255	58.64361702	47.7393617	24.48470745	76.26329787	88.19734345	90.62618596	106.7172676	67.48670213	94.88031915	78.65691489	55.25265957	46.47606383	74.93351064	15.82446809	80.25265957	14.91023936	51.76470588	99.12713472	65.12333966	60.57180851	27.79255319	75.19946809	54.72074468	7.197473404	72.22011385	22.80585106
	49.54289049	104.0799674	94.35907411	96.53763859	115.8334954	78.48667574	51.09900798	11.76813849	74.12954678	92.98245614	95.59363525	123.0110159	63.74246256	108.3641315	83.4662517	75.06321727	48.60922	74.75199378	6.788562536	73.81832328	9.589574013	61.64830681	108.3231334	61.55854753	62.30305388	25.46197238	70.70608831	52.03267847	0.564092589	67.99265606	29.62458866
	60.15533981	0	97.72815534	100.5242718	108.2912621	89.02912621	51.74757282	8.893203883	82.19417476	0	0	0	71.33980583	111.7087379	97.72815534	70.38834951	58.89320388	65.41747573	25.04854369	64.48543689	7.650485437	0	0	0	58.27184466	22.87378641	75.3592233	53.30097087	1.436893204	0	16.5631068

AMP1CPR3ERY1FUS3TMP1	AMP	CPR	ERY	FUS	TMP	AMP+CPR	AMP+ERY	AMP+FUS	AMP+TMP	CPR+ERY	CPR+FUS	CPR+TMP	ERY+FUS	ERY+TMP	FUS+TMP	AMP+CPR+ERY	AMP+CPR+FUS	AMP+CPR+TMP	AMP+ERY+FUS	AMP+ERY+TMP	AMP+FUS+TMP	CPR+ERY+FUS	CPR+ERY+TMP	CPR+FUS+TMP	ERY+FUS+TMP	AMP+CPR+ERY+FUS	AMP+CPR+ERY+TMP	AMP+CPR+FUS+TMP	AMP+ERY+FUS+TMP	CPR+ERY+FUS+TMP	AMP+CPR+ERY+FUS+TMP
	68.54228943	94.67005076	95.26991384	98.64603482	103.9915597	77.82662212	52.54088271	46.66783893	75.85721822	104.4839255	100.0846024	112.605753	88.23632847	89.36170213	75.01318797	74.16915773	58.69526991	83.45349042	36.46914014	78.38930895	57.85123967	105.499154	84.85617597	76.05752961	68.26094602	60.66467382	78.67065237	68.54228943	53.91243186	87.2250423	53.91243186
	60.57180851	95.18026565	101.2632979	101.7952128	100.4654255	58.64361702	47.7393617	52.06117021	84.77393617	88.19734345	101.2523719	109.4497154	89.82712766	96.21010638	69.6143617	55.25265957	85.57180851	91.95478723	41.95478723	74.1356383	66.68882979	94.87666034	94.57305503	90.01897533	58.17819149	65.625	78.125	69.6143617	57.38031915	78.48197343	55.25265957
	49.54289049	104.0799674	94.35907411	112.410037	112.410037	78.48667574	51.09900798	34.91538611	98.40497958	92.98245614	117.4622603	125.2957976	103.073332	109.297802	78.48667574	75.06321727	82.22135771	103.073332	29.00213966	87.51215717	56.38980743	102.7743778	106.3647491	92.32966136	68.21630033	69.14997082	102.7621085	78.79789924	44.25209103	81.88494492	49.54289049
	60.15533981	0	97.72815534	108.2912621	110.776699	89.02912621	51.74757282	36.83495146	94.93203883	0	0	0	100.2135922	112.6407767	83.12621359	70.38834951	56.40776699	105.184466	43.04854369	89.65048544	49.26213592	0	0	0	66.97087379	65.41747573	103.0097087	80.95145631	43.04854369	0	44.91262136

AMP1CPR3ERY1FUS3TMP2	AMP	CPR	ERY	FUS	TMP	AMP+CPR	AMP+ERY	AMP+FUS	AMP+TMP	CPR+ERY	CPR+FUS	CPR+TMP	ERY+FUS	ERY+TMP	FUS+TMP	AMP+CPR+ERY	AMP+CPR+FUS	AMP+CPR+TMP	AMP+ERY+FUS	AMP+ERY+TMP	AMP+FUS+TMP	CPR+ERY+FUS	CPR+ERY+TMP	CPR+FUS+TMP	ERY+FUS+TMP	AMP+CPR+ERY+FUS	AMP+CPR+ERY+TMP	AMP+CPR+FUS+TMP	AMP+ERY+FUS+TMP	CPR+ERY+FUS+TMP	AMP+CPR+ERY+FUS+TMP
	68.54228943	94.67005076	95.26991384	98.64603482	99.49006506	77.82662212	52.54088271	46.66783893	83.73483383	104.4839255	100.0846024	113.9593909	88.23632847	87.95498505	86.26692456	74.16915773	58.69526991	89.64304554	36.46914014	89.36170213	42.13117637	105.499154	104.822335	105.8375635	83.172147	60.66467382	75.85721822	66.0101987	28.62669246	83.50253807	55.88183577
	60.57180851	95.18026565	101.2632979	101.7952128	101.5292553	58.64361702	47.7393617	52.06117021	88.76329787	88.19734345	101.2523719	112.7893738	89.82712766	94.6143617	82.6462766	55.25265957	85.57180851	83.44414894	41.95478723	83.97606383	52.85904255	94.87666034	99.43074004	101.5559772	77.06117021	65.625	86.1037234	66.68882979	36.6356383	79.69639469	62.16755319
	49.54289049	104.0799674	94.35907411	112.410037	109.297802	78.48667574	51.09900798	34.91538611	102.1396615	92.98245614	117.4622603	127.5805794	103.073332	109.920249	95.2927446	75.06321727	82.22135771	117.7008364	29.00213966	97.16008559	45.49698502	102.7743778	118.7678499	102.121583	80.66524023	69.14997082	95.9151916	78.17545225	38.33884458	92.65605875	59.19081891
	60.15533981	0	97.72815534	108.2912621	115.4368932	89.02912621	51.74757282	36.83495146	103.631068	0	0	0	100.2135922	119.1650485	89.65048544	70.38834951	56.40776699	93.68932039	43.04854369	91.2038835	46.77669903	0	0	0	75.98058252	65.41747573	101.7669903	78.15533981	40.58252427	0	52.99029126

AMP1CPR3ERY1FUS3TMP3	AMP	CPR	ERY	FUS	TMP	AMP+CPR	AMP+ERY	AMP+FUS	AMP+TMP	CPR+ERY	CPR+FUS	CPR+TMP	ERY+FUS	ERY+TMP	FUS+TMP	AMP+CPR+ERY	AMP+CPR+FUS	AMP+CPR+TMP	AMP+ERY+FUS	AMP+ERY+TMP	AMP+FUS+TMP	CPR+ERY+FUS	CPR+ERY+TMP	CPR+FUS+TMP	ERY+FUS+TMP	AMP+CPR+ERY+FUS	AMP+CPR+ERY+TMP	AMP+CPR+FUS+TMP	AMP+ERY+FUS+TMP	CPR+ERY+FUS+TMP	AMP+CPR+ERY+FUS+TMP
	68.54228943	94.67005076	95.26991384	98.64603482	108.2117109	77.82662212	52.54088271	46.66783893	75.5758748	104.4839255	100.0846024	121.0659898	88.23632847	91.04976262	105.9609636	74.16915773	58.69526991	76.41990505	36.46914014	79.23333919	34.00738526	105.499154	104.822335	123.0964467	95.55125725	60.66467382	69.10497626	66.85422894	20.46773343	91.28595601	47.72287674
	60.57180851	95.18026565	101.2632979	101.7952128	100.4654255	58.64361702	47.7393617	52.06117021	76.26329787	88.19734345	101.2523719	106.7172676	89.82712766	94.88031915	105.2526596	55.25265957	85.57180851	74.93351064	41.95478723	80.25265957	37.25066489	94.87666034	99.12713472	111.2713472	97.00797872	65.625	75.19946809	65.95478723	39.29521277	87.28652751	57.91223404
	49.54289049	104.0799674	94.35907411	112.410037	115.8334954	78.48667574	51.09900798	34.91538611	74.12954678	92.98245614	117.4622603	123.0110159	103.073332	108.3641315	109.920249	75.06321727	82.22135771	74.75199378	29.00213966	73.81832328	41.33437074	102.7743778	108.3231334	113.5454916	106.808014	69.14997082	70.70608831	83.7774752	22.03851391	97.87841697	65.41528885
	60.15533981	0	97.72815534	108.2912621	108.2912621	89.02912621	51.74757282	36.83495146	82.19417476	0	0	0	100.2135922	111.7087379	111.0873786	70.38834951	56.40776699	65.41747573	43.04854369	64.48543689	28.77669903	0	0	0	105.184466	65.41747573	75.3592233	65.10679612	26.60194175	0	56.40776699

AMP1CPR3ERY2FUS1TMP1	AMP	CPR	ERY	FUS	TMP	AMP+CPR	AMP+ERY	AMP+FUS	AMP+TMP	CPR+ERY	CPR+FUS	CPR+TMP	ERY+FUS	ERY+TMP	FUS+TMP	AMP+CPR+ERY	AMP+CPR+FUS	AMP+CPR+TMP	AMP+ERY+FUS	AMP+ERY+TMP	AMP+FUS+TMP	CPR+ERY+FUS	CPR+ERY+TMP	CPR+FUS+TMP	ERY+FUS+TMP	AMP+CPR+ERY+FUS	AMP+CPR+ERY+TMP	AMP+CPR+FUS+TMP	AMP+ERY+FUS+TMP	CPR+ERY+FUS+TMP	AMP+CPR+ERY+FUS+TMP
	68.54228943	94.67005076	103.8353601	70.23034992	103.9915597	77.82662212	48.56690698	13.18797257	75.85721822	96.70050761	83.43908629	112.605753	69.71000935	113.414406	64.91999297	69.38631968	30.31475295	83.45349042	5.591700369	82.89080359	10.09319501	58.73519459	100.7614213	69.90270728	52.66604303	22.19096184	78.38930895	45.78864076	8.123791103	67.87225042	49.72744857
	60.57180851	95.18026565	102.1828909	82.6462766	100.4654255	58.64361702	46.40957447	3.390957447	84.77393617	93.05502846	54.00379507	109.4497154	77.95476893	110.993117	54.38829787	56.58244681	25.93085106	91.95478723	8.377659574	71.74202128	10.92087766	50.36053131	107.6280835	63.41555977	72.86135693	26.7287234	83.71010638	53.39095745	11.71875	57.0398482	39.7606383
	49.54289049	104.0799674	88.62788963	74.01283797	112.410037	78.48667574	57.01225443	2.120210076	98.40497958	97.2256222	67.19706242	125.2957976	82.06562267	107.7181208	71.21182649	75.99688777	31.18070414	103.073332	34.7986773	90.00194515	5.23244505	55.91595267	118.4414525	71.58302734	56.7114094	16.12526746	113.6549309	60.63022758	2.742657071	57.87433701	66.54347403
	60.15533981	0	0	81.26213592	110.776699	89.02912621	52.05825243	5.475728155	94.93203883	0	0	0	0	0	72.89320388	71.94174757	40.25242718	105.184466	4.233009709	91.2038835	1.747572816	0	0	0	0	14.17475728	103.3203883	52.69902913	2.990291262	0	54.5631068

AMP1CPR3ERY2FUS1TMP2	AMP	CPR	ERY	FUS	TMP	AMP+CPR	AMP+ERY	AMP+FUS	AMP+TMP	CPR+ERY	CPR+FUS	CPR+TMP	ERY+FUS	ERY+TMP	FUS+TMP	AMP+CPR+ERY	AMP+CPR+FUS	AMP+CPR+TMP	AMP+ERY+FUS	AMP+ERY+TMP	AMP+FUS+TMP	CPR+ERY+FUS	CPR+ERY+TMP	CPR+FUS+TMP	ERY+FUS+TMP	AMP+CPR+ERY+FUS	AMP+CPR+ERY+TMP	AMP+CPR+FUS+TMP	AMP+ERY+FUS+TMP	CPR+ERY+FUS+TMP	AMP+CPR+ERY+FUS+TMP
	68.54228943	94.67005076	103.8353601	70.23034992	99.49006506	77.82662212	48.56690698	13.18797257	83.73483383	96.70050761	83.43908629	113.9593909	69.71000935	120.2993452	64.63864955	69.38631968	30.31475295	89.64304554	5.591700369	82.04677334	9.530508177	58.73519459	108.8832487	83.10067682	62.84377923	22.19096184	77.26393529	41.00580271	9.811851591	58.05837563	31.15878319
	60.57180851	95.18026565	102.1828909	82.6462766	101.5292553	58.64361702	46.40957447	3.390957447	88.76329787	93.05502846	54.00379507	112.7893738	77.95476893	119.1740413	55.25265957	56.58244681	25.93085106	83.44414894	8.377659574	83.44414894	8.377659574	50.36053131	110.6641366	71.30929791	76.32251721	26.7287234	91.95478723	44.6143617	4.803856383	50.66413662	21.14361702
	49.54289049	104.0799674	88.62788963	74.01283797	109.297802	78.48667574	57.01225443	2.120210076	102.1396615	97.2256222	67.19706242	127.5805794	82.06562267	112.7889635	60.94145108	75.99688777	31.18070414	117.7008364	34.7986773	100.8947676	2.120210076	55.91595267	120.0734394	67.66625867	63.87024609	16.12526746	97.47130908	29.19665435	2.120210076	56.89514484	3.053880568
	60.15533981	0	0	81.26213592	115.4368932	89.02912621	52.05825243	5.475728155	103.631068	0	0	0	0	0	72.58252427	71.94174757	40.25242718	93.68932039	4.233009709	98.34951456	2.058252427	0	0	0	0	14.17475728	93.68932039	27.22330097	1.126213592	0	3.611650485

AMP1CPR3ERY2FUS1TMP3	AMP	CPR	ERY	FUS	TMP	AMP+CPR	AMP+ERY	AMP+FUS	AMP+TMP	CPR+ERY	CPR+FUS	CPR+TMP	ERY+FUS	ERY+TMP	FUS+TMP	AMP+CPR+ERY	AMP+CPR+FUS	AMP+CPR+TMP	AMP+ERY+FUS	AMP+ERY+TMP	AMP+FUS+TMP	CPR+ERY+FUS	CPR+ERY+TMP	CPR+FUS+TMP	ERY+FUS+TMP	AMP+CPR+ERY+FUS	AMP+CPR+ERY+TMP	AMP+CPR+FUS+TMP	AMP+ERY+FUS+TMP	CPR+ERY+FUS+TMP	AMP+CPR+ERY+FUS+TMP
	68.54228943	94.67005076	103.8353601	70.23034992	108.2117109	77.82662212	48.56690698	13.18797257	75.5758748	96.70050761	83.43908629	121.0659898	69.71000935	121.7960711	73.92298224	69.38631968	30.31475295	76.41990505	5.591700369	79.79602602	7.279760858	58.73519459	113.6209814	75.31725888	66.41721235	22.19096184	55.31914894	50.88799015	7.561104273	67.19543147	28.06400563
	60.57180851	95.18026565	102.1828909	82.6462766	100.4654255	58.64361702	46.40957447	3.390957447	76.26329787	93.05502846	54.00379507	106.7172676	77.95476893	124.5231072	59.50797872	56.58244681	25.93085106	74.93351064	8.377659574	80.78457447	15.97406915	50.36053131	110.6641366	67.66603416	78.89872173	26.7287234	76.79521277	50.19946809	7.04787234	65.23719165	19.16555851
	49.54289049	104.0799674	88.62788963	74.01283797	115.8334954	78.48667574	57.01225443	2.120210076	74.12954678	97.2256222	67.19706242	123.0110159	82.06562267	112.7889635	68.41081502	75.99688777	31.18070414	74.75199378	34.7986773	93.11418012	1.186539584	55.91595267	115.8302734	70.7874337	80.87248322	16.12526746	72.88465279	35.1099008	3.676327563	65.70787434	8.967127018
	60.15533981	0	0	81.26213592	108.2912621	89.02912621	52.05825243	5.475728155	82.19417476	0	0	0	0	0	74.75728155	71.94174757	40.25242718	65.41747573	4.233009709	75.98058252	4.54368932	0	0	0	0	14.17475728	61.06796117	38.31067961	1.436893204	0	18.21359223

AMP1CPR3ERY2FUS2TMP1	AMP	CPR	ERY	FUS	TMP	AMP+CPR	AMP+ERY	AMP+FUS	AMP+TMP	CPR+ERY	CPR+FUS	CPR+TMP	ERY+FUS	ERY+TMP	FUS+TMP	AMP+CPR+ERY	AMP+CPR+FUS	AMP+CPR+TMP	AMP+ERY+FUS	AMP+ERY+TMP	AMP+FUS+TMP	CPR+ERY+FUS	CPR+ERY+TMP	CPR+FUS+TMP	ERY+FUS+TMP	AMP+CPR+ERY+FUS	AMP+CPR+ERY+TMP	AMP+CPR+FUS+TMP	AMP+ERY+FUS+TMP	CPR+ERY+FUS+TMP	AMP+CPR+ERY+FUS+TMP
	68.54228943	94.67005076	103.8353601	85.70423774	103.9915597	77.82662212	48.56690698	32.03798136	75.85721822	96.70050761	80.79526227	112.605753	76.59494855	113.414406	66.0101987	69.38631968	44.34675576	83.45349042	25.56708282	82.89080359	28.34534904	64.55160745	100.7614213	60.49069374	58.3536015	40.47828381	78.38930895	46.59750308	36.50430807	65.50338409	50.00879198
	60.57180851	95.18026565	102.1828909	95.94414894	100.4654255	58.64361702	46.40957447	24.48470745	84.77393617	93.05502846	90.62618596	109.4497154	89.91150442	110.993117	62.96542553	56.58244681	46.47606383	91.95478723	18.48404255	71.74202128	34.85704787	76.96394687	107.6280835	59.65844402	66.56833825	33.11170213	83.71010638	47.00797872	25.66489362	59.77229602	37.36702128
	49.54289049	104.0799674	88.62788963	96.53763859	112.410037	78.48667574	57.01225443	11.76813849	98.40497958	97.2256222	95.59363525	125.2957976	83.55704698	107.7181208	67.47714452	75.99688777	48.60922	103.073332	12.70180899	90.00194515	34.4874538	81.23215014	118.4414525	79.92656059	74.01193139	26.51235168	113.6549309	46.00272321	42.57926473	61.79110567	52.53841665
	60.15533981	0	0	100.5242718	110.776699	89.02912621	52.05825243	8.893203883	94.93203883	0	0	0	0	0	66.36893204	71.94174757	58.89320388	105.184466	21.00970874	91.2038835	39.33980583	0	0	0	0	29.06796117	103.3203883	51.76699029	27.22330097	0	47.41747573

AMP1CPR3ERY2FUS2TMP2	AMP	CPR	ERY	FUS	TMP	AMP+CPR	AMP+ERY	AMP+FUS	AMP+TMP	CPR+ERY	CPR+FUS	CPR+TMP	ERY+FUS	ERY+TMP	FUS+TMP	AMP+CPR+ERY	AMP+CPR+FUS	AMP+CPR+TMP	AMP+ERY+FUS	AMP+ERY+TMP	AMP+FUS+TMP	CPR+ERY+FUS	CPR+ERY+TMP	CPR+FUS+TMP	ERY+FUS+TMP	AMP+CPR+ERY+FUS	AMP+CPR+ERY+TMP	AMP+CPR+FUS+TMP	AMP+ERY+FUS+TMP	CPR+ERY+FUS+TMP	AMP+CPR+ERY+FUS+TMP
	68.54228943	94.67005076	103.8353601	85.70423774	99.49006506	77.82662212	48.56690698	32.03798136	83.73483383	96.70050761	80.79526227	113.9593909	76.59494855	120.2993452	67.17074028	69.38631968	44.34675576	89.64304554	25.56708282	82.04677334	27.78266221	64.55160745	108.8832487	66.5820643	64.02245089	40.47828381	77.26393529	46.03481625	36.50430807	71.25634518	38.19236856
	60.57180851	95.18026565	102.1828909	95.94414894	101.5292553	58.64361702	46.40957447	24.48470745	88.76329787	93.05502846	90.62618596	112.7893738	89.91150442	119.1740413	62.43351064	56.58244681	46.47606383	83.44414894	18.48404255	83.44414894	29.27194149	76.96394687	110.6641366	59.0512334	71.03244838	33.11170213	91.95478723	56.31648936	24.66755319	59.77229602	27.06117021
	49.54289049	104.0799674	88.62788963	96.53763859	109.297802	78.48667574	57.01225443	11.76813849	102.1396615	97.2256222	95.59363525	127.5805794	83.55704698	112.7889635	74.63528496	75.99688777	48.60922	117.7008364	12.70180899	100.8947676	21.10484342	81.23215014	120.0734394	75.03059976	82.96047726	26.51235168	97.47130908	44.75782922	46.9363937	66.36066911	30.44154834
	60.15533981	0	0	100.5242718	115.4368932	89.02912621	52.05825243	8.893203883	103.631068	0	0	0	0	0	63.86407767	71.94174757	58.89320388	93.68932039	21.00970874	98.34951456	25.26213592	0	0	0	0	29.06796117	93.68932039	39.02912621	28.77669903	0	28.15533981

AMP1CPR3ERY2FUS2TMP3	AMP	CPR	ERY	FUS	TMP	AMP+CPR	AMP+ERY	AMP+FUS	AMP+TMP	CPR+ERY	CPR+FUS	CPR+TMP	ERY+FUS	ERY+TMP	FUS+TMP	AMP+CPR+ERY	AMP+CPR+FUS	AMP+CPR+TMP	AMP+ERY+FUS	AMP+ERY+TMP	AMP+FUS+TMP	CPR+ERY+FUS	CPR+ERY+TMP	CPR+FUS+TMP	ERY+FUS+TMP	AMP+CPR+ERY+FUS	AMP+CPR+ERY+TMP	AMP+CPR+FUS+TMP	AMP+ERY+FUS+TMP	CPR+ERY+FUS+TMP	AMP+CPR+ERY+FUS+TMP
	68.54228943	94.67005076	103.8353601	85.70423774	108.2117109	77.82662212	48.56690698	32.03798136	75.5758748	96.70050761	80.79526227	121.0659898	76.59494855	121.7960711	81.48408651	69.38631968	44.34675576	76.41990505	25.56708282	79.79602602	1.89906805	64.55160745	113.6209814	68.2106599	81.68381665	40.47828381	55.31914894	48.56690698	2.215579392	71.59475465	33.69087392
	60.57180851	95.18026565	102.1828909	95.94414894	100.4654255	58.64361702	46.40957447	24.48470745	76.26329787	93.05502846	90.62618596	106.7172676	89.91150442	124.5231072	78.65691489	56.58244681	46.47606383	74.93351064	18.48404255	80.78457447	14.91023936	76.96394687	110.6641366	65.12333966	94.3166175	33.11170213	76.79521277	54.72074468	0.997340426	67.66603416	19.6974734
	49.54289049	104.0799674	88.62788963	96.53763859	115.8334954	78.48667574	57.01225443	11.76813849	74.12954678	97.2256222	95.59363525	123.0110159	83.55704698	112.7889635	83.4662517	75.99688777	48.60922	74.75199378	12.70180899	93.11418012	9.589574013	81.23215014	115.8302734	61.55854753	91.90902312	26.51235168	72.88465279	52.03267847	7.722233029	69.624643	18.92627893
	60.15533981	0	0	100.5242718	108.2912621	89.02912621	52.05825243	8.893203883	82.19417476	0	0	0	0	0	97.72815534	71.94174757	58.89320388	65.41747573	21.00970874	75.98058252	7.650485437	0	0	0	0	29.06796117	61.06796117	53.30097087	5.786407767	0	35.30097087

AMP1CPR3ERY2FUS3TMP1	AMP	CPR	ERY	FUS	TMP	AMP+CPR	AMP+ERY	AMP+FUS	AMP+TMP	CPR+ERY	CPR+FUS	CPR+TMP	ERY+FUS	ERY+TMP	FUS+TMP	AMP+CPR+ERY	AMP+CPR+FUS	AMP+CPR+TMP	AMP+ERY+FUS	AMP+ERY+TMP	AMP+FUS+TMP	CPR+ERY+FUS	CPR+ERY+TMP	CPR+FUS+TMP	ERY+FUS+TMP	AMP+CPR+ERY+FUS	AMP+CPR+ERY+TMP	AMP+CPR+FUS+TMP	AMP+ERY+FUS+TMP	CPR+ERY+FUS+TMP	AMP+CPR+ERY+FUS+TMP
	68.54228943	94.67005076	103.8353601	98.64603482	103.9915597	77.82662212	48.56690698	46.66783893	75.85721822	96.70050761	100.0846024	112.605753	106.828812	113.414406	75.01318797	69.38631968	58.69526991	83.45349042	35.97678917	82.89080359	57.85123967	97.03891709	100.7614213	76.05752961	85.8746492	66.29154211	78.38930895	68.54228943	38.15720063	84.5177665	53.63108845

	AMP	CPR	ERY	FUS	TMP	AMP+CPR	AMP+ERY	AMP+FUS	AMP+TMP	CPR+ERY	CPR+FUS	CPR+TMP	ERY+FUS	ERY+TMP	FUS+TMP	AMP+CPR+ERY	AMP+CPR+FUS	AMP+CPR+TMP	…
	60.57180851	95.18026565	102.1828909	101.7952128	100.4654255	58.64361702	46.40957447	52.06117021	84.77393617	93.05502846	101.2523719	109.4497154	105.6440511	110.993117	69.6143617	56.58244681	85.57180851	91.95478723	[illegible]
	49.54289049	104.0799674	88.62788963	112.410037	112.410037	78.48667574	57.01225443	34.91538611	98.40497958	97.2256222	117.4622603	125.2957976	102.945563	107.7181208	78.48667574	75.99688777	82.22135771	103.073332	[illegible]
	60.15533981	0	0	108.2912621	110.776699	89.02912621	52.05825243	36.83495146	94.93203883	0	0	0	0	0	83.12621359	71.94174757	56.40776699	105.184466	[illegible]
AMP1CPR3ERY2FUS3TMP2	AMP	CPR	ERY	FUS	TMP	AMP+CPR	AMP+ERY	AMP+FUS	AMP+TMP	CPR+ERY	CPR+FUS	CPR+TMP	ERY+FUS	ERY+TMP	FUS+TMP	AMP+CPR+ERY	AMP+CPR+FUS	AMP+CPR+TMP	[illegible]
	68.54228943	94.67005076	103.8353601	98.64603482	99.49006506	77.82662212	48.56690698	46.66783893	83.73483383	96.70050761	100.0846024	113.9593909	106.828812	120.2993452	86.26692456	69.38631968	58.69526991	89.64304554	[illegible]
	60.57180851	95.18026565	102.1828909	101.7952128	101.5292553	58.64361702	46.40957447	52.06117021	88.76329787	93.05502846	101.2523719	112.7893738	105.6440511	119.1740413	82.6462766	56.58244681	85.57180851	83.44414894	[illegible]
	49.54289049	104.0799674	88.62788963	112.410037	109.297802	78.48667574	57.01225443	34.91538611	102.1396615	97.2256222	117.4622603	127.5805794	102.945563	112.7889635	95.2927446	75.99688777	82.22135771	117.7008364	[illegible]
	60.15533981	0	0	108.2912621	115.4368932	89.02912621	52.05825243	36.83495146	103.631068	0	0	0	0	0	89.65048544	71.94174757	56.40776699	93.68932039	[illegible]
AMP1CPR3ERY2FUS3TMP3	AMP	CPR	ERY	FUS	TMP	AMP+CPR	AMP+ERY	AMP+FUS	AMP+TMP	CPR+ERY	CPR+FUS	CPR+TMP	ERY+FUS	ERY+TMP	FUS+TMP	AMP+CPR+ERY	AMP+CPR+FUS	AMP+CPR+TMP	[illegible]
	68.54228943	94.67005076	103.8353601	98.64603482	108.2117109	77.82662212	48.56690698	46.66783893	75.5758748	96.70050761	100.0846024	121.0659898	106.828812	121.7960711	105.9609636	69.38631968	58.69526991	76.41990505	[illegible]
	60.57180851	95.18026565	102.1828909	101.7952128	100.4654255	58.64361702	46.40957447	52.06117021	76.26329787	93.05502846	101.2523719	106.7172676	105.6440511	124.5231072	105.2526596	56.58244681	85.57180851	74.93351064	[illegible]
	49.54289049	104.0799674	88.62788963	112.410037	115.8334954	78.48667574	57.01225443	34.91538611	74.12954678	97.2256222	117.4622603	123.0110159	102.945563	112.7889635	109.920249	75.99688777	82.22135771	74.75199378	[illegible]
	60.15533981	0	0	108.2912621	108.2912621	89.02912621	52.05825243	36.83495146	82.19417476	0	0	0	0	0	111.0873786	71.94174757	56.40776699	65.41747573	[illegible]
AMP1CPR3ERY3FUS1TMP1	AMP	CPR	ERY	FUS	TMP	AMP+CPR	AMP+ERY	AMP+FUS	AMP+TMP	CPR+ERY	CPR+FUS	CPR+TMP	ERY+FUS	ERY+TMP	FUS+TMP	AMP+CPR+ERY	AMP+CPR+FUS	AMP+CPR+TMP	[illegible]
	68.54228943	94.67005076	95.34686971	70.23034992	103.9915597	77.82662212	54.47511869	13.18797257	75.85721822	93.31641286	83.43908629	112.605753	80.79526227	114.6362098	64.91999297	80.35871285	30.31475295	83.45349042	[illegible]
	60.57180851	95.18026565	99.12713472	82.6462766	100.4654255	58.64361702	50.73138298	3.390957447	84.77393617	95.18026565	54.00379507	109.4497154	90.32258065	114.914611	54.38829787	58.44414894	25.93085106	91.95478723	[illegible]
	49.54289049	104.0799674	108.9759282	74.01283797	112.410037	78.48667574	53.78331064	2.120210076	98.40497958	100.4895961	67.19706242	125.2957976	93.63525092	120.3998368	71.21182649	67.28262984	31.18070414	103.073332	[illegible]
	60.15533981	0	0	81.26213592	110.776699	89.02912621	53.9223301	5.475728155	94.93203883	0	0	0	0	0	72.89320388	77.22330097	40.25242718	105.184466	[illegible]
AMP1CPR3ERY3FUS1TMP2	AMP	CPR	ERY	FUS	TMP	AMP+CPR	AMP+ERY	AMP+FUS	AMP+TMP	CPR+ERY	CPR+FUS	CPR+TMP	ERY+FUS	ERY+TMP	FUS+TMP	AMP+CPR+ERY	AMP+CPR+FUS	AMP+CPR+TMP	[illegible]
	68.54228943	94.67005076	95.34686971	70.23034992	99.49006506	77.82662212	54.47511869	13.18797257	83.73483383	93.31641286	83.43908629	113.9593909	80.79526227	114.9746193	64.63864955	80.35871285	30.31475295	89.64304554	[illegible]
	60.57180851	95.18026565	99.12713472	82.6462766	101.5292553	58.64361702	50.73138298	3.390957447	88.76329787	95.18026565	54.00379507	112.7893738	90.32258065	115.2182163	55.25265957	58.44414894	25.93085106	83.44414894	[illegible]
	49.54289049	104.0799674	108.9759282	74.01283797	109.297802	78.48667574	53.78331064	2.120210076	102.1396615	100.4895961	67.19706242	127.5805794	93.63525092	125.2957976	60.94145108	67.28262984	31.18070414	117.7008364	[illegible]
	60.15533981	0	0	81.26213592	115.4368932	89.02912621	53.9223301	5.475728155	103.631068	0	0	0	0	0	72.58252427	77.22330097	40.25242718	93.68932039	[illegible]
AMP1CPR3ERY3FUS1TMP3	AMP	CPR	ERY	FUS	TMP	AMP+CPR	AMP+ERY	AMP+FUS	AMP+TMP	CPR+ERY	CPR+FUS	CPR+TMP	ERY+FUS	ERY+TMP	FUS+TMP	AMP+CPR+ERY	AMP+CPR+FUS	AMP+CPR+TMP	[illegible]
	68.54228943	94.67005076	95.34686971	70.23034992	108.2117109	77.82662212	54.47511869	13.18797257	75.5758748	93.31641286	83.43908629	121.0659898	80.79526227	115.6514382	73.92298224	80.35871285	30.31475295	76.41990505	[illegible]
	60.57180851	95.18026565	99.12713472	82.6462766	100.4654255	58.64361702	50.73138298	3.390957447	76.26329787	95.18026565	54.00379507	106.7172676	90.32258065	107.0208729	59.50797872	58.44414894	25.93085106	74.93351064	[illegible]
	49.54289049	104.0799674	108.9759282	74.01283797	115.8334954	78.48667574	53.78331064	2.120210076	74.12954678	100.4895961	67.19706242	123.0110159	93.63525092	117.1358629	68.41081502	67.28262984	31.18070414	74.75199378	[illegible]
	60.15533981	0	0	81.26213592	108.2912621	89.02912621	53.9223301	5.475728155	82.19417476	0	0	0	0	0	74.75728155	77.22330097	40.25242718	65.41747573	[illegible]
AMP1CPR3ERY3FUS2TMP1	AMP	CPR	ERY	FUS	TMP	AMP+CPR	AMP+ERY	AMP+FUS	AMP+TMP	CPR+ERY	CPR+FUS	CPR+TMP	ERY+FUS	ERY+TMP	FUS+TMP	AMP+CPR+ERY	AMP+CPR+FUS	AMP+CPR+TMP	[illegible]
	68.54228943	94.67005076	95.34686971	85.70423774	103.9915597	77.82662212	54.47511869	32.03798136	75.85721822	93.31641286	80.79526227	112.605753	84.17935702	114.6362098	66.0101987	80.35871285	44.34675576	83.45349042	[illegible]
	60.57180851	95.18026565	99.12713472	95.94414894	100.4654255	58.64361702	50.73138298	24.48470745	84.77393617	95.18026565	90.62618596	109.4497154	93.05502846	114.914611	62.96542553	58.44414894	46.47606383	91.95478723	[illegible]
	49.54289049	104.0799674	108.9759282	96.53763859	112.410037	78.48667574	53.78331064	11.76813849	98.40497958	100.4895961	95.59363525	125.2957976	103.75357	120.3998368	67.47714452	67.28262984	48.60922	103.073332	[illegible]
	60.15533981	0	0	100.5242718	110.776699	89.02912621	53.9223301	8.893203883	94.93203883	0	0	0	0	0	66.36893204	77.22330097	58.89320388	105.184466	[illegible]
AMP1CPR3ERY3FUS2TMP2	AMP	CPR	ERY	FUS	TMP	AMP+CPR	AMP+ERY	AMP+FUS	AMP+TMP	CPR+ERY	CPR+FUS	CPR+TMP	ERY+FUS	ERY+TMP	FUS+TMP	AMP+CPR+ERY	AMP+CPR+FUS	AMP+CPR+TMP	[illegible]
	68.54228943	94.67005076	95.34686971	85.70423774	99.49006506	77.82662212	54.47511869	32.03798136	83.73483383	93.31641286	80.79526227	113.9593909	84.17935702	114.9746193	67.17074028	80.35871285	44.34675576	89.64304554	[illegible]
	60.57180851	95.18026565	99.12713472	95.94414894	101.5292553	58.64361702	50.73138298	24.48470745	88.76329787	95.18026565	90.62618596	112.7893738	93.05502846	115.2182163	62.43351064	58.44414894	46.47606383	83.44414894	[illegible]
	49.54289049	104.0799674	108.9759282	96.53763859	109.297802	78.48667574	53.78331064	11.76813849	102.1396615	100.4895961	95.59363525	127.5805794	103.75357	125.2957976	74.63528496	67.28262984	48.60922	117.7008364	[illegible]
	60.15533981	0	0	100.5242718	115.4368932	89.02912621	53.9223301	8.893203883	103.631068	0	0	0	0	0	63.86407767	77.22330097	58.89320388	93.68932039	[illegible]
AMP1CPR3ERY3FUS2TMP3	AMP	CPR	ERY	FUS	TMP	AMP+CPR	AMP+ERY	AMP+FUS	AMP+TMP	CPR+ERY	CPR+FUS	CPR+TMP	ERY+FUS	ERY+TMP	FUS+TMP	AMP+CPR+ERY	AMP+CPR+FUS	AMP+CPR+TMP	[illegible]
	68.54228943	94.67005076	95.34686971	85.70423774	108.2117109	77.82662212	54.47511869	32.03798136	75.5758748	93.31641286	80.79526227	121.0659898	84.17935702	115.6514382	81.48408651	80.35871285	44.34675576	76.41990505	[illegible]

[illegible]

AMP2CPR2ERY1FUS1TMP2	AMP	CPR	ERY	FUS	TMP	AMP+CPR	AMP+ERY	AMP+FUS	AMP+TMP	CPR+ERY	CPR+FUS	CPR+TMP	ERY+FUS	ERY+TMP	FUS+TMP	AMP+CPR+ERY	AMP+CPR+FUS	AMP+CPR+TMP	[illegible]
	70.30869972	109.8222638	95.26991384	70.23034992	99.49006506	70.6080449	85.57530402	16.59494855	100.243218	86.47333957	70.92609916	111.3189897	60.3833304	87.95498505	64.63864955	60.4303087	27.6707203	91.26286249	[illegible]
	87.07964602	103.7561455	101.2632979	82.6462766	101.5292553	73.54965585	79.21337266	14.04129794	94.94591937	99.98033432	72.546706	116.027532	63.49734043	94.6143617	55.25265957	60.33431662	29.75417896	89.91150442	[illegible]
	70.43251305	83.55704698	94.35907411	74.01283797	109.297802	62.37882177	61.48396719	26.4541387	105.6301268	87.73303505	56.54362416	119.0529456	74.94650846	109.920249	60.94145108	71.6256525	25.68978374	98.76957494	[illegible]
	0	0	97.72815534	81.26213592	115.4368932	0	0	0	0	0	0	0	76	119.1650485	72.58252427	0	0	0	[illegible]

AMP2CPR2ERY1FUS1TMP3	AMP	CPR	ERY	FUS	TMP	AMP+CPR	AMP+ERY	AMP+FUS	AMP+TMP	CPR+ERY	CPR+FUS	CPR+TMP	ERY+FUS	ERY+TMP	FUS+TMP	AMP+CPR+ERY	AMP+CPR+FUS	AMP+CPR+TMP	[illegible]
	70.30869972	109.8222638	95.26991384	70.23034992	108.2117109	70.6080449	85.57530402	16.59494855	104.1347053	86.47333957	70.92609916	124.4901777	60.3833304	91.04976262	73.92298224	60.4303087	27.6707203	66.11786717	[illegible]
	87.07964602	103.7561455	101.2632979	82.6462766	100.4654255	73.54965585	79.21337266	14.04129794	99.03638151	99.98033432	72.546706	123.2645034	63.49734043	94.88031915	59.50797872	60.33431662	29.75417896	83.93313668	[illegible]
	70.43251305	83.55704698	94.35907411	74.01283797	115.8334954	62.37882177	61.48396719	26.4541387	92.20730798	87.73303505	56.54362416	119.3512304	74.94650846	108.3641315	68.41081502	71.6256525	25.68978374	88.9261745	[illegible]
	0	0	97.72815534	81.26213592	108.2912621	0	0	0	0	0	0	0	76	111.7087379	74.75728155	0	0	0	[illegible]

AMP2CPR2ERY1FUS2TMP1	AMP	CPR	ERY	FUS	TMP	AMP+CPR	AMP+ERY	AMP+FUS	AMP+TMP	CPR+ERY	CPR+FUS	CPR+TMP	ERY+FUS	ERY+TMP	FUS+TMP	AMP+CPR+ERY	AMP+CPR+FUS	AMP+CPR+TMP	[illegible]
	70.30869972	109.8222638	95.26991384	85.70423774	103.9915597	70.6080449	85.57530402	28.26941066	93.05893358	86.47333957	81.98316183	110.121609	68.82363285	89.36170213	66.0101987	60.4303087	35.88400374	106.5294668	[illegible]
	87.07964602	103.7561455	101.2632979	95.94414894	100.4654255	73.54965585	79.21337266	37.64011799	89.91150442	99.98033432	85.50639135	109.4198623	67.48670213	96.21010638	62.96542553	60.33431662	26.9813176	95.88987217	[illegible]
	70.43251305	83.55704698	94.35907411	96.53763859	112.410037	62.37882177	61.48396719	38.08724832	95.1901566	87.73303505	79.08277405	113.3855332	63.74246256	109.297802	67.47714452	71.6256525	39.4108874	94.29530201	[illegible]
	0	0	97.72815534	100.5242718	110.776699	0	0	0	0	0	0	0	71.33980583	112.6407767	66.36893204	0	0	0	[illegible]

AMP2CPR2ERY1FUS2TMP2	AMP	CPR	ERY	FUS	TMP	AMP+CPR	AMP+ERY	AMP+FUS	AMP+TMP	CPR+ERY	CPR+FUS	CPR+TMP	ERY+FUS	ERY+TMP	FUS+TMP	AMP+CPR+ERY	AMP+CPR+FUS	AMP+CPR+TMP	[illegible]
	70.30869972	109.8222638	95.26991384	85.70423774	99.49006506	70.6080449	85.57530402	28.26941066	100.243218	86.47333957	81.98316183	111.3189897	68.82363285	87.95498505	67.17074028	60.4303087	35.88400374	91.26286249	[illegible]
	87.07964602	103.7561455	101.2632979	95.94414894	101.5292553	73.54965585	79.21337266	37.64011799	94.94591937	99.98033432	85.50639135	116.027532	67.48670213	94.6143617	62.43351064	60.33431662	26.9813176	89.91150442	[illegible]
	70.43251305	83.55704698	94.35907411	96.53763859	109.297802	62.37882177	61.48396719	38.08724832	105.6301268	87.73303505	79.08277405	119.0529456	63.74246256	109.920249	74.63528496	71.6256525	39.4108874	98.76957494	[illegible]
	0	0	97.72815534	100.5242718	115.4368932	0	0	0	0	0	0	0	71.33980583	119.1650485	63.86407767	0	0	0	[illegible]

[illegible]

	87.07964602	95.18026565	102.1828909	95.94414894	101.5292553	78.5840708	79.84267453	37.64011799	94.94591937	93.05502846	90.62618596	112.7893738	89.91150442	119.1740413	62.43351064	68.82989184	54.3166175	104.0707965	38.5840708	89.91150442	34.49360865	76.96394687	110.6641366	59.0512334	71.03244838	31.66175025	97.46312684	73.86430678	40.45231072	59.77229602	50.26548673
	70.43251305	104.0799674	88.62788963	96.53763859	109.297802	70.73079791	66.85309471	38.08724832	105.6301268	97.2256222	95.59363525	127.5805794	83.55704698	112.7889635	74.63528496	82.36390753	55.81655481	97.87472036	53.43027591	89.82102908	43.15809098	81.23215014	120.0734394	75.03059976	82.96047726	35.23489933	98.76957494	69.83594333	51.9388516	66.36066911	55.81655481
	0	0	0	100.5242718	115.4368932	0	0	0	0	0	0	0	0	0	63.86407767	0	0	0	0	0	0	0	0	0	0	0	0	0	0	0	0

AMP2CPR3ERY2FUS2TMP3	AMP	CPR	ERY	FUS	TMP	AMP+CPR	AMP+ERY	AMP+FUS	AMP+TMP	CPR+ERY	CPR+FUS	CPR+TMP	ERY+FUS	ERY+TMP	FUS+TMP	AMP+CPR+ERY	AMP+CPR+FUS	AMP+CPR+TMP	AMP+ERY+FUS	AMP+ERY+TMP	AMP+FUS+TMP	CPR+ERY+FUS	CPR+ERY+TMP	CPR+FUS+TMP	ERY+FUS+TMP	AMP+CPR+ERY+FUS	AMP+CPR+ERY+TMP	AMP+CPR+FUS+TMP	AMP+ERY+FUS+TMP	CPR+ERY+FUS+TMP	AMP+CPR+ERY+FUS+TMP
	70.30869972	94.67005076	103.8353601	85.70423774	108.2117109	84.37792329	73.60149673	28.26941066	104.1347053	96.70050761	80.79526227	121.0659898	76.59494855	121.7960711	81.48408651	78.98971001	44.88306829	97.24976614	31.56220767	95.45369504	19.73807297	64.55160745	113.6209814	68.2106599	81.68381665	36.48269411	85.8746492	76.89429373	21.38447147	71.59475465	45.46304958
	87.07964602	95.18026565	102.1828909	95.94414894	100.4654255	78.5840708	79.84267453	37.64011799	99.03638151	93.05502846	90.62618596	106.7172676	89.91150442	124.5231072	78.65691489	68.82989184	54.3166175	86.76499508	38.5840708	93.68731563	29.77384464	76.96394687	110.6641366	65.12333966	94.3166175	31.66175025	87.07964602	87.70894789	21.27826942	67.66603416	44.91642085
	70.43251305	104.0799674	88.62788963	96.53763859	115.8334954	70.73079791	66.85309471	38.08724832	92.20730798	97.2256222	95.59363525	123.0110159	83.55704698	112.7889635	83.4662517	82.36390753	55.81655481	82.06562267	53.43027591	91.31245339	37.4906786	81.23215014	115.8302734	81.55854753	91.90902312	35.23489933	99.36614467	89.22445936	22.53914989	69.624643	52.83370619
	0	0	0	100.5242718	108.2912621	0	0	0	0	0	0	0	0	0	97.72815534	0	0	0	0	0	0	0	0	0	0	0	0	0	0	0	0

AMP2CPR3ERY2FUS3TMP1	AMP	CPR	ERY	FUS	TMP	AMP+CPR	AMP+ERY	AMP+FUS	AMP+TMP	CPR+ERY	CPR+FUS	CPR+TMP	ERY+FUS	ERY+TMP	FUS+TMP	AMP+CPR+ERY	AMP+CPR+FUS	AMP+CPR+TMP	AMP+ERY+FUS	AMP+ERY+TMP	AMP+FUS+TMP	CPR+ERY+FUS	CPR+ERY+TMP	CPR+FUS+TMP	ERY+FUS+TMP	AMP+CPR+ERY+FUS	AMP+CPR+ERY+TMP	AMP+CPR+FUS+TMP	AMP+ERY+FUS+TMP	CPR+ERY+FUS+TMP	AMP+CPR+ERY+FUS+TMP
	70.30869972	94.67005076	103.8353601	98.64603482	103.9915597	84.37792329	73.60149673	66.41721235	93.05893358	96.70050761	100.0846024	112.605753	106.828812	113.414406	75.01318797	78.98971001	68.81197381	88.26941066	59.23292797	81.68381665	73.00280636	97.03891709	100.7614213	76.05752961	85.8746492	83.77923293	89.16744621	71.80542563	68.81197381	84.5177665	67.01590271
	87.07964602	95.18026565	102.1828909	101.7952128	100.4654255	78.5840708	79.84267453	65.05408063	89.91150442	93.05502846	101.2523719	109.4497154	105.6440511	110.993117	69.6143617	68.82989184	87.07964602	114.1396264	69.77384464	88.02359882	78.5840708	101.5559772	107.6280835	90.01897533	72.92035398	79.21337266	98.40707965	82.67453294	71.97640118	81.21442125	65.05408063
	70.43251305	104.0799674	88.62788963	112.410037	112.410037	70.73079791	66.85309471	68.64280388	95.1901566	97.2256222	117.4622603	125.2957976	102.945563	107.7181208	78.48667574	82.36390753	80.2759135	102.6472782	70.73079791	95.1901566	79.97762864	108.9759282	118.4414525	92.32966136	77.59134974	75.5033557	99.06785981	82.96047726	73.71364653	83.84332925	74.90678598
	0	0	0	108.2912621	110.776699	0	0	0	0	0	0	0	0	0	83.12621359	0	0	0	0	0	0	0	0	0	0	0	0	0	0	0	0

AMP2CPR3ERY2FUS3TMP2	AMP	CPR	ERY	FUS	TMP	AMP+CPR	AMP+ERY	AMP+FUS	AMP+TMP	CPR+ERY	CPR+FUS	CPR+TMP	ERY+FUS	ERY+TMP	FUS+TMP	AMP+CPR+ERY	AMP+CPR+FUS	AMP+CPR+TMP	AMP+ERY+FUS	AMP+ERY+TMP	AMP+FUS+TMP	CPR+ERY+FUS	CPR+ERY+TMP	CPR+FUS+TMP	ERY+FUS+TMP	AMP+CPR+ERY+FUS	AMP+CPR+ERY+TMP	AMP+CPR+FUS+TMP	AMP+ERY+FUS+TMP	CPR+ERY+FUS+TMP	AMP+CPR+ERY+FUS+TMP
	70.30869972	94.67005076	103.8353601	98.64603482	99.49006506	84.37792329	73.60149673	66.41721235	100.243218	96.70050761	100.0846024	113.9593909	106.828812	120.2993452	86.26692456	78.98971001	68.81197381	100.5425631	59.23292797	104.1347053	69.41066417	97.03891709	108.8832487	105.8375635	93.95696913	83.77923293	92.16089804	78.39101964	55.94013096	101.0998308	68.51262862
	87.07964602	95.18026565	102.1828909	101.7952128	101.5292553	78.5840708	79.84267453	65.05408063	94.94591937	93.05502846	101.2523719	112.7893738	105.6440511	119.1740413	82.6462766	68.82989184	87.07964602	104.0707965	69.77384464	89.91150442	86.13569322	101.5559772	110.6641366	101.5559772	89.28220256	79.21337266	97.46312684	80.78662734	59.07571288	93.35863378	64.7394297
	70.43251305	104.0799674	88.62788963	112.410037	109.297802	70.73079791	66.85309471	68.64280388	105.6301268	97.2256222	117.4622603	127.5805794	102.945563	112.7889635	95.2927446	82.36390753	80.2759135	97.87472036	70.73079791	89.82102908	85.34675615	108.9759282	120.0734394	102.121583	99.66442953	75.5033557	98.76957494	82.66219239	71.92393736	89.06568747	80.2759135
	0	0	0	108.2912621	115.4368932	0	0	0	0	0	0	0	0	0	89.65048544	0	0	0	0	0	0	0	0	0	0	0	0	0	0	0	0

AMP2CPR3ERY2FUS3TMP3	AMP	CPR	ERY	FUS	TMP	AMP+CPR	AMP+ERY	AMP+FUS	AMP+TMP	CPR+ERY	CPR+FUS	CPR+TMP	ERY+FUS	ERY+TMP	FUS+TMP	AMP+CPR+ERY	AMP+CPR+FUS	AMP+CPR+TMP	AMP+ERY+FUS	AMP+ERY+TMP	AMP+FUS+TMP	CPR+ERY+FUS	CPR+ERY+TMP	CPR+FUS+TMP	ERY+FUS+TMP	AMP+CPR+ERY+FUS	AMP+CPR+ERY+TMP	AMP+CPR+FUS+TMP	AMP+ERY+FUS+TMP	CPR+ERY+FUS+TMP	AMP+CPR+ERY+FUS+TMP
	70.30869972	94.67005076	103.8353601	98.64603482	108.2117109	84.37792329	73.60149673	66.41721235	104.1347053	96.70050761	100.0846024	121.0659898	106.828812	121.7960711	105.9609636	78.98971001	68.81197381	97.24976614	59.23292797	95.45369504	34.85500468	97.03891709	113.6209814	123.0964467	119.1019645	83.77923293	85.8746492	93.65762395	24.97661366	98.73096447	80.7857811
	87.07964602	95.18026565	102.1828909	101.7952128	100.4654255	78.5840708	79.84267453	65.05408063	99.03638151	93.05502846	101.2523719	106.7172676	105.6440511	124.5231072	105.2526596	68.82989184	87.07964602	86.76499508	69.77384464	93.68731563	42.98918387	101.5559772	110.6641366	111.2713472	109.4198623	79.21337266	87.07964602	88.96755162	36.69616519	91.23339658	77.01081613
	70.43251305	104.0799674	88.62788963	112.410037	115.8334954	70.73079791	66.85309471	68.64280388	92.20730798	97.2256222	117.4622603	123.0110159	102.945563	112.7889635	109.920249	82.36390753	80.2759135	82.06562267	70.73079791	91.31245339	47.63236391	108.9759282	115.8302734	113.5454916	111.8941089	75.5033557	99.36614467	90.71588367	40.60402685	98.53121175	87.13646532
	0	0	0	108.2912621	108.2912621	0	0	0	0	0	0	0	0	0	111.0873786	0	0	0	0	0	0	0	0	0	0	0	0	0	0	0	0

AMP2CPR3ERY3FUS1TMP1	AMP	CPR	ERY	FUS	TMP	AMP+CPR	AMP+ERY	AMP+FUS	AMP+TMP	CPR+ERY	CPR+FUS	CPR+TMP	ERY+FUS	ERY+TMP	FUS+TMP	AMP+CPR+ERY	AMP+CPR+FUS	AMP+CPR+TMP	AMP+ERY+FUS	AMP+ERY+TMP	AMP+FUS+TMP	CPR+ERY+FUS	CPR+ERY+TMP	CPR+FUS+TMP	ERY+FUS+TMP	AMP+CPR+ERY+FUS	AMP+CPR+ERY+TMP	AMP+CPR+FUS+TMP	AMP+ERY+FUS+TMP	CPR+ERY+FUS+TMP	AMP+CPR+ERY+FUS+TMP
	70.30869972	94.67005076	95.34686971	70.23034992	103.9915597	84.37792329	76.29560337	16.59494855	93.05893358	93.31641286	83.43908629	112.605753	80.79526227	114.6362098	64.91999297	75.99625819	36.65107577	88.26941066	14.79887746	86.77268475	34.85500468	72.60998308	108.2064298	69.90270728	60.08883249	39.17680075	94.85500468	48.15715622	40.84190833	74.64043993	53.84471469
	87.07964602	95.18026565	99.12713472	82.6462766	100.4654255	78.5840708	84.87708948	14.04129794	89.91150442	95.18026565	54.00379507	109.4497154	90.32258065	114.914611	54.38829787	74.80825959	36.38151426	114.1396264	19.70501475	86.13569322	21.8879056	70.89184061	111.2713472	63.41555977	56.31878558	41.14060964	100.6096362	59.33136676	42.65486726	58.55787476	58.07276303
	70.43251305	104.0799674	108.9759282	74.01283797	112.410037	70.73079791	73.11707681	26.4541387	95.1901566	100.4895961	67.19706242	125.2957976	93.63525092	120.3998368	71.21182649	77.8896346	31.95376585	102.6472782	18.10216257	92.50559284	36.59582401	65.23867809	124.9694002	71.58302734	62.95389637	37.32289336	96.38329605	64.46681581	42.22595078	56.89514484	65.65995526
	0	0	0	81.26213592	110.776699	0	0	0	0	0	0	0	0	0	72.89320388	0	0	0	0	0	0	0	0	0	0	0	0	0	0	0	0

AMP2CPR3ERY3FUS1TMP2	AMP	CPR	ERY	FUS	TMP	AMP+CPR	AMP+ERY	AMP+FUS	AMP+TMP	CPR+ERY	CPR+FUS	CPR+TMP	ERY+FUS	ERY+TMP	FUS+TMP	AMP+CPR+ERY	AMP+CPR+FUS	AMP+CPR+TMP	AMP+ERY+FUS	AMP+ERY+TMP	AMP+FUS+TMP	CPR+ERY+FUS	CPR+ERY+TMP	CPR+FUS+TMP	ERY+FUS+TMP	AMP+CPR+ERY+FUS	AMP+CPR+ERY+TMP	AMP+CPR+FUS+TMP	AMP+ERY+FUS+TMP	CPR+ERY+FUS+TMP	AMP+CPR+ERY+FUS+TMP
	70.30869972	94.67005076	95.34686971	70.23034992	99.49006506	84.37792329	76.29560337	16.59494855	100.243218	93.31641286	83.43908629	113.9593909	80.79526227	114.9746193	64.63864955	75.99625819	36.65107577	100.5425631	14.79887746	106.2301216	10.90739008	72.60998308	112.9441624	83.10067682	72.2715736	39.17680075	97.54911132	49.95322732	16.89429373	80.05499154	42.76894294
	87.07964602	95.18026565	99.12713472	82.6462766	101.5292553	78.5840708	84.87708948	14.04129794	94.94591937	95.18026565	54.00379507	112.7893738	90.32258065	115.2182163	55.25265957	74.80825959	36.38151426	104.0707965	19.70501475	93.68731563	8.987217306	70.89184061	112.1821632	71.30929791	65.73055028	41.14060964	99.35103245	54.04129794	7.728613569	65.54079696	45.48672566
	70.43251305	104.0799674	108.9759282	74.01283797	109.297802	70.73079791	73.11707681	26.4541387	105.6301268	100.4895961	67.19706242	127.5805794	93.63525092	125.2957976	60.94145108	77.8896346	31.95376585	97.87472036	18.10216257	99.36614467	12.43475019	65.23867809	122.3582211	67.66625867	80.57935537	37.32289336	99.96271439	66.55480984	7.662192394	61.46470828	62.08053691
	0	0	0	81.26213592	115.4368932	0	0	0	0	0	0	0	0	0	72.58252427	0	0	0	0	0	0	0	0	0	0	0	0	0	0	0	0

AMP2CPR3ERY3FUS1TMP3	AMP	CPR	ERY	FUS	TMP	AMP+CPR	AMP+ERY	AMP+FUS	AMP+TMP	CPR+ERY	CPR+FUS	CPR+TMP	ERY+FUS	ERY+TMP	FUS+TMP	AMP+CPR+ERY	AMP+CPR+FUS	AMP+CPR+TMP	AMP+ERY+FUS	AMP+ERY+TMP	AMP+FUS+TMP	CPR+ERY+FUS	CPR+ERY+TMP	CPR+FUS+TMP	ERY+FUS+TMP	AMP+CPR+ERY+FUS	AMP+CPR+ERY+TMP	AMP+CPR+FUS+TMP	AMP+ERY+FUS+TMP	CPR+ERY+FUS+TMP	AMP+CPR+ERY+FUS+TMP
	70.30869972	94.67005076	95.34686971	70.23034992	108.2117109	84.37792329	76.29560337	16.59494855	104.1347053	93.31641286	83.43908629	121.0659898	80.79526227	115.6514382	73.92298224	75.99625819	36.65107577	97.24976614	14.79887746	100.243218	9.710009355	72.60998308	117.3434856	75.31725888	83.84094755	39.17680075	89.46679139	62.22637979	31.26286249	70.9179357	70.30869972
	87.07964602	95.18026565	99.12713472	82.6462766	100.4654255	78.5840708	84.87708948	14.04129794	99.03638151	95.18026565	54.00379507	106.7172676	90.32258065	107.0208729	59.50797872	74.80825959	36.38151426	86.76499508	19.70501475	97.14847591	22.53687316	70.89184061	105.5028463	67.66603416	81.82163188	41.14060964	79.84267453	77.95476893	14.65093412	64.32637571	69.14454277
	70.43251305	104.0799674	108.9759282	74.01283797	115.8334954	70.73079791	73.11707681	26.4541387	92.20730798	100.4895961	67.19706242	123.0110159	93.63525092	117.1358629	68.41081502	77.8896346	31.95376585	82.06562267	18.10216257	90.11931394	10.64504101	65.23867809	117.7886577	70.7874337	90.04487964	37.32289336	92.20730798	78.78448919	12.73303505	70.7874337	75.5033557
	0	0	0	81.26213592	108.2912621	0	0	0	0	0	0	0	0	0	74.75728155	0	0	0	0	0	0	0	0	0	0	0	0	0	0	0	0

AMP2CPR3ERY3FUS2TMP1	AMP	CPR	ERY	FUS	TMP	AMP+CPR	AMP+ERY	AMP+FUS	AMP+TMP	CPR+ERY	CPR+FUS	CPR+TMP	ERY+FUS	ERY+TMP	FUS+TMP	AMP+CPR+ERY	AMP+CPR+FUS	AMP+CPR+TMP	AMP+ERY+FUS	AMP+ERY+TMP	AMP+FUS+TMP	CPR+ERY+FUS	CPR+ERY+TMP	CPR+FUS+TMP	ERY+FUS+TMP	AMP+CPR+ERY+FUS	AMP+CPR+ERY+TMP	AMP+CPR+FUS+TMP	AMP+ERY+FUS+TMP	CPR+ERY+FUS+TMP	AMP+CPR+ERY+FUS+TMP
	70.30869972	94.67005076	95.34686971	85.70423774	103.9915597	84.37792329	76.29560337	28.26941066	93.05893358	93.31641286	80.79526227	112.605753	84.17935702	114.6362098	66.0101987	75.99625819	44.88306829	88.26941066	40.54256314	86.77268475	42.78765201	79.78003384	108.2064298	60.49069374	69.2893401	45.76239476	94.85500468	52.34798877	37.10009355	51.3536379	60.4303087
	87.07964602	95.18026565	99.12713472	95.94414894	100.4654255	78.5840708	84.87708948	37.64011799	89.91150442	95.18026565	90.62618596	109.4497154	93.05502846	114.914611	62.96542553	74.80825959	54.3166175	114.1396264	42.35988201	86.13569322	32.9006883	86.07210626	111.2713472	59.65844402	74.23149905	37.36479843	100.6096362	66.31268437	35.41789577	54.4971537	59.39036382
	70.43251305	104.0799674	108.9759282	96.53763859	112.410037	70.73079791	73.11707681	38.08724832	95.1901566	100.4895961	95.59363525	125.2957976	103.75357	120.3998368	67.47714452	77.8896346	55.81655481	102.6472782	36.89410887	92.50559284	40.30574198	86.78090575	124.9694002	79.92656059	78.29457364	53.72856078	96.38329605	66.25652498	38.38553318	60.66911465	65.06338553
	0	0	0	100.5242718	110.776699	0	0	0	0	0	0	0	0	0	66.36893204	0	0	0	0	0	0	0	0	0	0	0	0	0	0	0	0

AMP2CPR3ERY3FUS2TMP2	AMP	CPR	ERY	FUS	TMP	AMP+CPR	AMP+ERY	AMP+FUS	AMP+TMP	CPR+ERY	CPR+FUS	CPR+TMP	ERY+FUS	ERY+TMP	FUS+TMP	AMP+CPR+ERY	AMP+CPR+FUS	AMP+CPR+TMP	AMP+ERY+FUS	AMP+ERY+TMP	AMP+FUS+TMP	CPR+ERY+FUS	CPR+ERY+TMP	CPR+FUS+TMP	ERY+FUS+TMP	AMP+CPR+ERY+FUS	AMP+CPR+ERY+TMP	AMP+CPR+FUS+TMP	AMP+ERY+FUS+TMP	CPR+ERY+FUS+TMP	AMP+CPR+ERY+FUS+TMP
	70.30869972	94.67005076	95.34686971	85.70423774	99.49006506	84.37792329	76.29560337	28.26941066	100.243218	93.31641286	80.79526227	113.9593909	84.17935702	114.9746193	67.17074028	75.99625819	44.88306829	100.5425631	40.54256314	106.2301216	29.91580917	79.78003384	112.9441624	66.5820643	85.8714044	45.76239476	97.54911132	64.32179607	37.97942002	61.44247039	62.52572498
	87.07964602	95.18026565	99.12713472	95.94414894	101.5292553	78.5840708	84.87708948	37.64011799	94.94591937	95.18026565	90.62618596	112.7893738	93.05502846	115.2182163	62.43351064	74.80825959	54.3166175	104.0707965	42.35988201	93.68731563	34.49360865	86.07210626	112.1821632	59.0512334	88.19734345	37.36479843	99.35103245	73.86430678	39.25270403	63.90891841	60.96361849
	70.43251305	104.0799674	108.9759282	96.53763859	109.297802	70.73079791	73.11707681	38.08724832	105.6301268	100.4895961	95.59363525	127.5805794	103.75357	125.2957976	74.63528496	77.8896346	55.81655481	97.87472036	36.89410887	99.36614467	43.15809098	86.78090575	122.3582211	75.03059976	85.14891881	53.72856078	99.96271439	69.83594333	48.52721849	74.70420237	73.71364653
	0	0	0	100.5242718	115.4368932	0	0	0	0	0	0	0	0	0	63.86407767	0	0	0	0	0	0	0	0	0	0	0	0	0	0	0	0

AMP2CPR3ERY3FUS2TMP3	AMP	CPR	ERY	FUS	TMP	AMP+CPR	AMP+ERY	AMP+FUS	AMP+TMP	CPR+ERY	CPR+FUS	CPR+TMP	ERY+FUS	ERY+TMP	FUS+TMP	AMP+CPR+ERY	AMP+CPR+FUS	AMP+CPR+TMP	AMP+ERY+FUS	AMP+ERY+TMP	AMP+FUS+TMP	CPR+ERY+FUS	CPR+ERY+TMP	CPR+FUS+TMP	ERY+FUS+TMP	AMP+CPR+ERY+FUS	AMP+CPR+ERY+TMP	AMP+CPR+FUS+TMP	AMP+ERY+FUS+TMP	CPR+ERY+FUS+TMP	AMP+CPR+ERY+FUS+TMP
	70.30869972	94.67005076	95.34686971	85.70423774	108.2117109	84.37792329	76.29560337	28.26941066	104.1347053	93.31641286	80.79526227	121.0659898	84.17935702	115.6514382	81.48408651	75.99625819	44.88306829	97.24976614	40.54256314	100.243218	19.73807297	79.78003384	117.3434856	68.2106599	98.39255499	45.76239476	89.46679139	76.89429373	33.65762395	60.82910321	77.4929841
	87.07964602	95.18026565	99.12713472	95.94414894	100.4654255	78.5840708	84.87708948	37.64011799	99.03638151	95.18026565	90.62618596	106.7172676	93.05502846	107.0208729	78.65691489	74.80825959	54.3166175	86.76499508	42.35988201	97.14847591	29.77384464	86.07210626	105.5028463	65.12333966	93.9658444	37.36479843	79.84267453	87.70894789	31.97640118	69.07020873	78.26941986
	70.43251305	104.0799674	108.9759282	96.53763859	115.8334954	70.73079791	73.11707681	38.08724832	92.20730798	100.4895961	95.59363525	123.0110159	103.75357	117.1358629	83.4662517	77.8896346	55.81655481	82.06562267	36.89410887	90.11931394	37.4906786	86.78090575	117.7886577	81.55854753	92.65605875	53.72856078	92.20730798	89.22445936	33.31469053	70.46103631	82.36390753
	0	0	0	100.5242718	108.2912621	0	0	0	0	0	0	0	0	0	97.72815534	0	0	0	0	0	0	0	0	0	0	0	0	0	0	0	0

AMP2CPR3ERY3FUS3TMP1	AMP	CPR	ERY	FUS	TMP	AMP+CPR	AMP+ERY	AMP+FUS	AMP+TMP	CPR+ERY	CPR+FUS	CPR+TMP	ERY+FUS	ERY+TMP	FUS+TMP	AMP+CPR+ERY	AMP+CPR+FUS	AMP+CPR+TMP	AMP+ERY+FUS	AMP+ERY+TMP	AMP+FUS+TMP	CPR+ERY+FUS	CPR+ERY+TMP	CPR+FUS+TMP	ERY+FUS+TMP	AMP+CPR+ERY+FUS	AMP+CPR+ERY+TMP	AMP+CPR+FUS+TMP	AMP+ERY+FUS+TMP	CPR+ERY+FUS+TMP	AMP+CPR+ERY+FUS+TMP
	70.30869972	94.67005076	95.34686971	98.64603482	103.9915597	84.37792329	76.29560337	66.41721235	93.05893358	93.31641286	100.0846024	112.605753	102.1150592	114.6362098	75.01318797	75.99625819	68.81197381	88.26941066	60.13096352	86.77268475	73.00280636	101.0998308	108.2064298	76.05752961	79.44162437	70.90739008	94.85500468	71.80542563	81.68381665	78.42639594	71.50608045
	87.07964602	95.18026565	99.12713472	101.7952128	100.4654255	78.5840708	84.87708948	65.05408063	89.91150442	95.18026565	101.2523719	109.4497154	107.9316888	114.914611	69.6143617	74.80825959	87.07964602	114.1396264	69.14454277	86.13569322	78.5840708	101.8595825	111.2713472	90.01897533	84.25047438	87.70894789	100.6096362	82.67453294	88.96755162	88.19734345	74.17895772
	70.43251305	104.0799674	108.9759282	112.410037	112.410037	70.73079791	73.11707681	68.64280388	95.1901566	100.4895961	117.4622603	125.2957976	115.1774786	120.3998368	78.48667574	77.8896346	80.2759135	102.6472782	82.96047726	92.50559284	79.97762864	111.2607099	124.9694002	92.32966136	94.94084047	77.8896346	96.38329605	82.96047726	80.87248322	88.08649531	76.69649515
	0	0	0	108.2912621	110.776699	0	0	0	0	0	0	0	0	0	83.12621359	0	0	0	0	0	0	0	0	0	0	0	0	0	0	0	0

AMP2CPR3ERY3FUS3TMP2	AMP	CPR	ERY	FUS	TMP	AMP+CPR	AMP+ERY	AMP+FUS	AMP+TMP	CPR+ERY	CPR+FUS	CPR+TMP	ERY+FUS	ERY+TMP	FUS+TMP	AMP+CPR+ERY	AMP+CPR+FUS	AMP+CPR+TMP	AMP+ERY+FUS	AMP+ERY+TMP	AMP+FUS+TMP	CPR+ERY+FUS	CPR+ERY+TMP	CPR+FUS+TMP	ERY+FUS+TMP	AMP+CPR+ERY+FUS	AMP+CPR+ERY+TMP	AMP+CPR+FUS+TMP	AMP+ERY+FUS+TMP	CPR+ERY+FUS+TMP	AMP+CPR+ERY+FUS+TMP
	70.30869972	94.67005076	95.34686971	98.64603482	99.49006506	84.37792329	76.29560337	66.41721235	100.243218	93.31641286	100.0846024	113.9593909	102.1150592	114.9746193	86.26692456	75.99625819	68.81197381	100.5425631	60.13096352	106.2301216	69.41066417	101.0998308	112.9441624	105.8375635	106.1759729	70.90739008	97.54911132	78.39101964	60.13096352	93.31641286	82.5818522
	87.07964602	95.18026565	99.12713472	101.7952128	101.5292553	78.5840708	84.87708948	65.05408063	94.94591937	95.18026565	101.2523719	112.7893738	107.9316888	115.2182163	82.6462766	74.80825959	87.07964602	104.0707965	69.14454277	93.68731563	86.13569322	101.8595825	112.1821632	101.5559772	101.8595825	87.70894789	99.35103245	80.78662734	72.60570305	94.87666034	77.01081613
	70.43251305	104.0799674	108.9759282	112.410037	109.297802	70.73079791	73.11707681	68.64280388	105.6301268	100.4895961	117.4622603	127.5805794	115.1774786	125.2957976	95.2927446	77.8896346	80.2759135	97.87472036	82.96047726	99.36614467	85.34675615	111.2607099	122.3582211	102.121583	106.6911465	77.8896346	99.96271439	82.66219239	77.8896346	103.4271726	78.48620433
	0	0	0	108.2912621	115.4368932	0	0	0	0	0	0	0	0	0	89.65048544	0	0	0	0	0	0	0	0	0	0	0	0	0	0	0	0

AMP2CPR3ERY3FUS3TMP3	AMP	CPR	ERY	FUS	TMP	AMP+CPR	AMP+ERY	AMP+FUS	AMP+TMP	CPR+ERY	CPR+FUS	CPR+TMP	ERY+FUS	ERY+TMP	FUS+TMP	AMP+CPR+ERY	AMP+CPR+FUS	AMP+CPR+TMP	AMP+ERY+FUS	AMP+ERY+TMP	AMP+FUS+TMP	CPR+ERY+FUS	CPR+ERY+TMP	CPR+FUS+TMP	ERY+FUS+TMP	AMP+CPR+ERY+FUS	AMP+CPR+ERY+TMP	AMP+CPR+FUS+TMP	AMP+ERY+FUS+TMP	CPR+ERY+FUS+TMP	AMP+CPR+ERY+FUS+TMP
	70.30869972	94.67005076	95.34686971	98.64603482	108.2117109	84.37792329	76.29560337	66.41721235	104.1347053	93.31641286	100.0846024	121.0659898	102.1150592	115.6514382	105.9609636	75.99625819	68.81197381	97.24976614	60.13096352	100.243218	34.85500468	101.0998308	117.3434856	123.0964467	114.2978003	70.90739008	89.46679139	93.65762395	48.4565014	126.142132	88.26941066
	87.07964602	95.18026565	99.12713472	101.7952128	100.4654255	78.5840708	84.87708948	65.05408063	99.03638151	95.18026565	101.2523719	106.7172676	107.9316888	107.0208729	105.2526596	74.80825959	87.07964602	86.76499508	69.14454277	97.14847591	42.98918387	101.8595825	105.5028463	111.2713472	114.3074004	87.70894789	79.84267453	88.96755162	69.45919371	110.6641366	91.17010816
	70.43251305	104.0799674	108.9759282	112.410037	115.8334954	70.73079791	73.11707681	68.64280388	92.20730798	100.4895961	117.4622603	123.0110159	115.1774786	117.1358629	109.920249	77.8896346	80.2759135	82.06562267	82.96047726	90.11931394	47.63236391	111.2607099	117.7886577	113.5454916	128.5597715	77.8896346	92.20730798	90.71588367	77.59134974	114.8510812	98.17300522
	0	0	0	108.2912621	108.2912621	0	0	0	0	0	0	0	0	0	111.0873786	0	0	0	0	0	0	0	0	0	0	0	0	0	0	0	0

AMP3CPR1ERY1FUS1TMP1	AMP	CPR	ERY	FUS	TMP	AMP+CPR	AMP+ERY	AMP+FUS	AMP+TMP	CPR+ERY	CPR+FUS	CPR+TMP	ERY+FUS	ERY+TMP	FUS+TMP	AMP+CPR+ERY	AMP+CPR+FUS	AMP+CPR+TMP	AMP+ERY+FUS	AMP+ERY+TMP	AMP+FUS+TMP	CPR+ERY+FUS	CPR+ERY+TMP	CPR+FUS+TMP	ERY+FUS+TMP	AMP+CPR+ERY+FUS	AMP+CPR+ERY+TMP	AMP+CPR+FUS+TMP	AMP+ERY+FUS+TMP	CPR+ERY+FUS+TMP	AMP+CPR+ERY+FUS+TMP
	96.36209814	98.3646914	95.26991384	70.23034992	103.9915597	84.17935702	92.97800338	11.06175973	89.59390863	90.48707579	67.17074028	80.92139968	60.3833304	89.36170213	64.91999297	78.42639594	16.09560068	100.4230118	14.78426396	97.37732657	43.50676819	55.07297345	72.48109724	67.17074028	55.91700369	17.78764805	91.96277496	36.73857868	44.18358714	55.07297345	22.92724196
	86.9829222	96.74202128	101.2632979	82.6462766	100.4654255	68.15939279	80	16.66034156	103.3776091	85.57180851	51.7287234	98.86968085	63.49734043	96.21010638	54.38829787	71.19544592	14.80075901	79.69639469	22.4288425	89.10815939	27.55218216	45.07978723	78.125	55.71808511	55.98404255	12.97912713	87.28652751	47.9316888	29.67741935	49.33510638	44.59203036
	71.44022848	90.62439214	94.35907411	74.01283797	112.410037	86.78090575	82.5377397	19.54304366	114.1982864	106.808014	70.5893795	124.8589769	74.94650846	109.297802	71.21182649	61.64830681	22.48062016	95.26723786	19.86944104	96.89922481	31.94614443	59.69655709	84.0886987	68.41081502	72.45672048	14.97348021	76.6625867	32.27254182	35.02651979	47.2476172	35.35291718
	0	91.51456311	97.72815534	81.26213592	110.776699	0	0	0	0	100.5242718	85.00970874	113.5728155	76	112.6407767	72.89320388	0	0	0	0	0	0	75.06796117	95.24271845	69.16504854	71.33980583	0	0	0	0	49.90291262	0

AMP3CPR1ERY1FUS1TMP2	AMP	CPR	ERY	FUS	TMP	AMP+CPR	AMP+ERY	AMP+FUS	AMP+TMP	CPR+ERY	CPR+FUS	CPR+TMP	ERY+FUS	ERY+TMP	FUS+TMP	AMP+CPR+ERY	AMP+CPR+FUS	AMP+CPR+TMP	AMP+ERY+FUS	AMP+ERY+TMP	AMP+FUS+TMP	CPR+ERY+FUS	CPR+ERY+TMP	CPR+FUS+TMP	ERY+FUS+TMP	AMP+CPR+ERY+FUS	AMP+CPR+ERY+TMP	AMP+CPR+FUS+TMP	AMP+ERY+FUS+TMP	CPR+ERY+FUS+TMP	AMP+CPR+ERY+FUS+TMP
	96.36209814	98.3646914	95.26991384	70.23034992	99.49006506	84.17935702	92.97800338	11.06175973	98.05414552	90.48707579	67.17074028	79.79602602	60.3833304	87.95498505	64.63864955	78.42639594	16.09560068	88.91708968	14.78426396	101.4382403	18.80287648	55.07297345	89.64304554	72.79760858	58.44909443	17.78764805	100.0846024	17.82994924	2.897631134	54.54545455	10.72335025
	86.9829222	96.74202128	101.2632979	82.6462766	101.5292553	68.15939279	80	16.66034156	97.30550285	85.57180851	51.7287234	94.34840426	63.49734043	94.6143617	55.25265957	71.19544592	14.80075901	85.16129032	22.4288425	93.9658444	20.26565465	45.07978723	93.55053191	62.10106383	53.85638298	12.97912713	98.82352941	18.48197343	11.76470588	37.36702128	9.373814042
	71.44022848	90.62439214	94.35907411	74.01283797	109.297802	86.78090575	82.5377397	19.54304366	112.8926969	106.808014	70.5893795	110.2314725	74.94650846	109.920249	60.94145108	61.64830681	22.48062016	91.67686659	19.86944104	112.2399021	23.78620971	59.69655709	105.8743435	73.39039097	69.03326201	14.97348021	78.62097103	27.0501836	17.25826193	50.67107567	14.78988168
	0	91.51456311	97.72815534	81.26213592	115.4368932	0	0	0	0	100.5242718	85.00970874	114.815534	76	119.1650485	72.58252427	0	0	0	0	0	0	75.06796117	107.6699029	72.27184466	65.74757282	0	0	0	0	47.00970874	0

AMP3CPR1ERY1FUS1TMP3	AMP	CPR	ERY	FUS	TMP	AMP+CPR	AMP+ERY	AMP+FUS	AMP+TMP	CPR+ERY	CPR+FUS	CPR+TMP	ERY+FUS	ERY+TMP	FUS+TMP	AMP+CPR+ERY	AMP+CPR+FUS	AMP+CPR+TMP	AMP+ERY+FUS	AMP+ERY+TMP	AMP+FUS+TMP	CPR+ERY+FUS	CPR+ERY+TMP	CPR+FUS+TMP	ERY+FUS+TMP	AMP+CPR+ERY+FUS	AMP+CPR+ERY+TMP	AMP+CPR+FUS+TMP	AMP+ERY+FUS+TMP	CPR+ERY+FUS+TMP	AMP+CPR+ERY+FUS+TMP
	96.36209814	98.3646914	95.26991384	70.23034992	108.2117109	84.17935702	92.97800338	11.06175973	103.4686971	90.48707579	67.17074028	90.7684192	60.3833304	91.04976262	73.92298224	78.42639594	16.09560068	106.1759729	14.78426396	99.06937394	18.12605753	55.07297345	93.30050993	73.078952	67.4520837	17.78764805	61.50592217	20.55837563	1.924703892	65.4826798	9.708121827
	86.9829222	96.74202128	101.2632979	82.6462766	100.4654255	68.15939279	80	16.66034156	89.10815939	85.57180851	51.7287234	98.07180851	63.49734043	94.88031915	59.50797872	71.19544592	14.80075901	81.51802657	22.4288425	88.50094877	23.90891841	45.07978723	91.95478723	69.28191489	51.99468085	12.97912713	83.64326376	37.00189753	28.15939279	45.61170213	32.75142315
	71.44022848	90.62439214	94.35907411	74.01283797	115.8334954	86.78090575	82.5377397	19.54304366	108.9759282	106.808014	70.5893795	111.4763665	74.94650846	108.3641315	68.41081502	61.64830681	22.48062016	84.82252142	19.86944104	103.4271726	22.48062016	59.69655709	107.7416845	78.68119043	70.278156	14.97348021	62.30110159	30.64055488	16.60546716	55.02820463	22.9498164
	0	91.51456311	97.72815534	81.26213592	108.2912621	0	0	0	0	100.5242718	85.00970874	117.9223301	76	111.7087379	74.75728155	0	0	0	0	0	0	75.06796117	116.368932	77.55339806	61.08737864	0	0	0	0	49.80582524	0

AMP3CPR1ERY1FUS2TMP1	AMP	CPR	ERY	FUS	TMP	AMP+CPR	AMP+ERY	AMP+FUS	AMP+TMP	CPR+ERY	CPR+FUS	CPR+TMP	ERY+FUS	ERY+TMP	FUS+TMP	AMP+CPR+ERY	AMP+CPR+FUS	AMP+CPR+TMP	AMP+ERY+FUS	AMP+ERY+TMP	AMP+FUS+TMP	CPR+ERY+FUS	CPR+ERY+TMP	CPR+FUS+TMP	ERY+FUS+TMP	AMP+CPR+ERY+FUS	AMP+CPR+ERY+TMP	AMP+CPR+FUS+TMP	AMP+ERY+FUS+TMP	CPR+ERY+FUS+TMP	AMP+CPR+ERY+FUS+TMP
	96.36209814	98.3646914	95.26991384	85.70423774	103.9915597	84.17935702	92.97800338	57.78341794	89.59390863	90.48707579	76.41990505	80.92139968	68.82363285	89.36170213	66.0101987	78.42639594	43.57021997	100.4230118	53.72250423	97.37732657	55.68950931	57.32372077	72.48109724	64.91999297	65.20133638	40.18612521	91.96277496	40.52453469	52.64382403	66.60805346	29.69543147
	86.9829222	96.74202128	101.2632979	95.94414894	100.4654255	68.15939279	80	48.72865275	103.3776091	85.57180851	77.59308511	98.86968085	67.48670213	96.21010638	62.96542553	71.19544592	39.92409867	79.69639469	42.9601518	89.10815939	42.04933586	50.13297872	78.125	50.13297872	44.88031915	40.5313093	87.28652751	50.66413662	37.60910816	47.20744681	37.60910816
	71.44022848	90.62439214	94.35907411	96.53763859	112.410037	86.78090575	82.5377397	56.75234598	114.1982864	106.808014	67.28262984	124.8589769	63.74246256	109.297802	67.47714452	61.64830681	48.91880865	95.26723786	49.24520604	96.89922481	35.53651571	77.74751994	84.0886987	61.87512157	63.74246256	27.37658099	76.6625867	51.20359037	48.08241534	72.45672048	37.6376989
	0	91.51456311	97.72815534	100.5242718	110.776699	0	0	0	0	100.5242718	74.13592233	113.5728155	71.33980583	112.6407767	66.36893204	0	0	0	0	0	0	79.41747573	95.24271845	63.26213592	62.01941748	0	0	0	0	65.4368932	0

AMP3CPR1ERY1FUS2TMP2	AMP	CPR	ERY	FUS	TMP	AMP+CPR	AMP+ERY	AMP+FUS	AMP+TMP	CPR+ERY	CPR+FUS	CPR+TMP	ERY+FUS	ERY+TMP	FUS+TMP	AMP+CPR+ERY	AMP+CPR+FUS	AMP+CPR+TMP	AMP+ERY+FUS	AMP+ERY+TMP	AMP+FUS+TMP	CPR+ERY+FUS	CPR+ERY+TMP	CPR+FUS+TMP	ERY+FUS+TMP	AMP+CPR+ERY+FUS	AMP+CPR+ERY+TMP	AMP+CPR+FUS+TMP	AMP+ERY+FUS+TMP	CPR+ERY+FUS+TMP	AMP+CPR+ERY+FUS+TMP
	96.36209814	98.3646914	95.26991384	85.70423774	99.49006506	84.17935702	92.97800338	57.78341794	98.05414552	90.48707579	76.41990505	79.79602602	68.82363285	87.95498505	67.17074028	78.42639594	43.57021997	88.91708968	53.72250423	101.4382403	55.68950931	57.32372077	89.64304554	71.95357834	69.70283102	40.18612521	100.0846024	58.79864636	45.19881557	64.07596272	42.55499154
	86.9829222	96.74202128	101.2632979	95.94414894	101.5292553	68.15939279	80	48.72865275	97.30550285	85.57180851	77.59308511	94.34840426	67.48670213	94.6143617	62.43351064	71.19544592	39.92409867	85.16129032	42.9601518	93.9658444	47.81783681	50.13297872	93.55053191	61.03723404	52.32712766	40.5313093	98.82352941	55.21821632	37.60910816	58.11170213	40.9487666
	71.44022848	90.62439214	94.35907411	96.53763859	109.297802	86.78090575	82.5377397	56.75234598	112.8926969	106.808014	67.28262984	110.2314725	63.74246256	109.920249	74.63528496	61.64830681	48.91880865	91.67686659	49.24520604	112.2399021	49.57160343	77.74751994	105.8743435	67.47714452	70.900603	27.37658099	78.62097103	65.89147287	39.77968176	87.70667185	44.34924521
	0	91.51456311	97.72815534	100.5242718	115.4368932	0	0	0	0	100.5242718	74.13592233	114.815534	71.33980583	119.1650485	63.86407767	0	0	0	0	0	0	79.41747573	107.6699029	71.96116505	63.88349515	0	0	0	0	71.65048544	0

AMP3CPR1ERY1FUS2TMP3	AMP	CPR	ERY	FUS	TMP	AMP+CPR	AMP+ERY	AMP+FUS	AMP+TMP	CPR+ERY	CPR+FUS	CPR+TMP	ERY+FUS	ERY+TMP	FUS+TMP	AMP+CPR+ERY	AMP+CPR+FUS	AMP+CPR+TMP	AMP+ERY+FUS	AMP+ERY+TMP	AMP+FUS+TMP	CPR+ERY+FUS	CPR+ERY+TMP	CPR+FUS+TMP	ERY+FUS+TMP	AMP+CPR+ERY+FUS	AMP+CPR+ERY+TMP	AMP+CPR+FUS+TMP	AMP+ERY+FUS+TMP	CPR+ERY+FUS+TMP	AMP+CPR+ERY+FUS+TMP
	96.36209814	98.3646914	95.26991384	85.70423774	108.2117109	84.17935702	92.97800338	57.78341794	103.4686971	90.48707579	76.41990505	90.7684192	68.82363285	91.04976262	81.48408651	78.42639594	43.57021997	106.1759729	53.72250423	99.06937394	32.00084602	57.32372077	93.30050993	83.20731493	67.41691577	40.18612521	61.50592217	35.38494078	26.92470389	71.39089151	23.94247039
	86.9829222	96.74202128	101.2632979	95.94414894	100.4654255	68.15939279	80	48.72865275	89.10815939	85.57180851	77.59308511	98.07180851	67.48670213	94.88031915	78.65691489	71.19544592	39.92409867	81.51802657	42.9601518	88.50094877	35.4459203	50.13297872	91.95478723	65.55851064	60.57180851	40.5313093	83.64326376	36.66034156	37.30550285	52.2606383	27.59013283
	71.44022848	90.62439214	94.35907411	96.53763859	115.8334954	86.78090575	82.5377397	56.75234598	108.9759282	106.808014	67.28262984	111.4763665	63.74246256	108.3641315	83.4662517	61.64830681	48.91880865	84.82252142	49.24520604	103.4271726	40.10607915	77.74751994	107.7416845	66.54347403	62.30305388	27.37658099	62.30110159	54.46756426	30.96695226	59.0741101	25.74459404
	0	91.51456311	97.72815534	100.5242718	108.2912621	0	0	0	0	100.5242718	74.13592233	117.9223301	71.33980583	111.7087379	97.72815534	0	0	0	0	0	0	79.41747573	116.368932	81.59223301	58.27184466	0	0	0	0	75.68932039	0

AMP3CPR1ERY1FUS3TMP1	AMP	CPR	ERY	FUS	TMP	AMP+CPR	AMP+ERY	AMP+FUS	AMP+TMP	CPR+ERY	CPR+FUS	CPR+TMP	ERY+FUS	ERY+TMP	FUS+TMP	AMP+CPR+ERY	AMP+CPR+FUS	AMP+CPR+TMP	AMP+ERY+FUS	AMP+ERY+TMP	AMP+FUS+TMP	CPR+ERY+FUS	CPR+ERY+TMP	CPR+FUS+TMP	ERY+FUS+TMP	AMP+CPR+ERY+FUS	AMP+CPR+ERY+TMP	AMP+CPR+FUS+TMP	AMP+ERY+FUS+TMP	CPR+ERY+FUS+TMP	AMP+CPR+ERY+FUS+TMP
	96.36209814	98.3646914	95.26991384	98.64603482	103.9915597	84.17935702	92.97800338	82.82571912	89.59390863	90.48707579	84.86020749	80.92139968	88.23632847	89.36170213	75.01318797	78.42639594	82.14890017	100.4230118	87.90186125	97.37732657	86.88663283	85.98558115	72.48109724	60.10198699	68.26094602	84.17935702	91.96277496	77.41116751	87.56345178	59.29312467	57.10659898
	86.9829222	96.74202128	101.2632979	101.7952128	100.4654255	68.15939279	80	76.66034156	103.3776091	85.57180851	97.53989362	98.86968085	89.82712766	96.21010638	69.6143617	71.19544592	79.08918406	79.69639469	79.08918406	89.10815939	76.05313093	81.05053191	78.125	58.17819149	58.17819149	75.4459203	87.28652751	63.30170778	76.05313093	52.59308511	65.73055028
	71.44022848	90.62439214	94.35907411	112.410037	112.410037	86.78090575	82.5377397	91.67686659	114.1982864	106.808014	107.7416845	124.8589769	103.073332	109.297802	78.48667574	61.64830681	74.70420237	95.26723786	80.25295798	96.89922481	87.10730314	82.22135771	84.0886987	62.92550088	68.21630033	80.90575275	76.6625867	70.46103631	80.25295798	51.91596966	77.96817625
	0	91.51456311	97.72815534	108.2912621	110.776699	0	0	0	0	100.5242718	107.0485437	113.5728155	100.2135922	112.6407767	83.12621359	0	0	0	0	0	0	82.50485437	95.24271845	75.04854369	66.97087379	0	0	0	0	61.39805825	0

AMP3CPR1ERY1FUS3TMP2	AMP	CPR	ERY	FUS	TMP	AMP+CPR	AMP+ERY	AMP+FUS	AMP+TMP	CPR+ERY	CPR+FUS	CPR+TMP	ERY+FUS	ERY+TMP	FUS+TMP	AMP+CPR+ERY	AMP+CPR+FUS	AMP+CPR+TMP	AMP+ERY+FUS	AMP+ERY+TMP	AMP+FUS+TMP	CPR+ERY+FUS	CPR+ERY+TMP	CPR+FUS+TMP	ERY+FUS+TMP	AMP+CPR+ERY+FUS	AMP+CPR+ERY+TMP	AMP+CPR+FUS+TMP	AMP+ERY+FUS+TMP	CPR+ERY+FUS+TMP	AMP+CPR+ERY+FUS+TMP
	96.36209814	98.3646914	95.26991384	98.64603482	99.49006506	84.17935702	92.97800338	82.82571912	98.05414552	90.48707579	84.86020749	79.79602602	88.23632847	87.95498505	86.26692456	78.42639594	82.14890017	88.91708968	87.90186125	101.4382403	81.47208122	85.98558115	89.64304554	77.54527871	83.172147	84.17935702	100.0846024	82.48730964	75.38071066	59.82064357	62.85956007
	86.9829222	96.74202128	101.2632979	101.7952128	101.5292553	68.15939279	80	76.66034156	97.30550285	85.57180851	97.53989362	94.34840426	89.82712766	94.6143617	82.6462766	71.19544592	79.08918406	85.16129032	79.08918406	93.9658444	74.23149905	81.05053191	93.55053191	76.52925532	77.06117021	75.4459203	98.82352941	67.55218216	66.0341556	53.92287234	62.69449715
	71.44022848	90.62439214	94.35907411	112.410037	109.297802	86.78090575	82.5377397	91.67686659	112.8926969	106.808014	107.7416845	110.2314725	103.073332	109.920249	95.2927446	61.64830681	74.70420237	91.67686659	80.25295798	112.2399021	81.55854753	82.22135771	105.8743435	83.4662517	80.66524023	80.90575275	78.62097103	82.5377397	71.76662587	75.88017895	74.37780498
	0	91.51456311	97.72815534	108.2912621	115.4368932	0	0	0	0	100.5242718	107.0485437	114.815534	100.2135922	119.1650485	89.65048544	0	0	0	0	0	0	82.50485437	107.6699029	88.7184466	75.98058252	0	0	0	0	71.02912621	0

AMP3CPR1ERY1FUS3TMP3	AMP	CPR	ERY	FUS	TMP	AMP+CPR	AMP+ERY	AMP+FUS	AMP+TMP	CPR+ERY	CPR+FUS	CPR+TMP	ERY+FUS	ERY+TMP	FUS+TMP	AMP+CPR+ERY	AMP+CPR+FUS	AMP+CPR+TMP	AMP+ERY+FUS	AMP+ERY+TMP	AMP+FUS+TMP	CPR+ERY+FUS	CPR+ERY+TMP	CPR+FUS+TMP	ERY+FUS+TMP	AMP+CPR+ERY+FUS	AMP+CPR+ERY+TMP	AMP+CPR+FUS+TMP	AMP+ERY+FUS+TMP	CPR+ERY+FUS+TMP	AMP+CPR+ERY+FUS+TMP
	96.36209814	98.3646914	95.26991384	98.64603482	108.2117109	84.17935702	92.97800338	82.82571912	103.4686971	90.48707579	84.86020749	90.7684192	88.23632847	91.04976262	105.9609636	78.42639594	82.14890017	106.1759729	87.90186125	99.06937394	55.68950931	85.98558115	93.30050993	94.14454018	95.55125725	84.17935702	61.50592217	74.02707276	50.67681895	67.9796026	51.69204738
	86.9829222	96.74202128	101.2632979	101.7952128	100.4654255	68.15939279	80	76.66034156	89.10815939	85.57180851	97.53989362	98.07180851	89.82712766	94.88031915	105.2526596	71.19544592	79.08918406	81.51802657	79.08918406	88.50094877	56.9259962	81.05053191	91.95478723	94.88031915	97.00797872	75.4459203	83.64326376	52.97912713	58.74762808	71.47606383	45.38899431
	71.44022848	90.62439214	94.35907411	112.410037	115.8334954	86.78090575	82.5377397	91.67686659	108.9759282	106.808014	107.7416845	111.4763665	103.073332	108.3641315	109.920249	61.64830681	74.70420237	84.82252142	80.25295798	103.4271726	73.07221542	82.22135771	107.7416845	100.5835441	106.808014	80.90575275	62.30110159	76.00979192	64.9122807	61.99183038	56.75234598
	0	91.51456311	97.72815534	108.2912621	108.2912621	0	0	0	0	100.5242718	107.0485437	117.9223301	100.2135922	111.7087379	111.0873786	0	0	0	0	0	0	82.50485437	116.368932	107.9805825	105.184466	0	0	0	0	70.69902913	0

AMP3CPR1ERY2FUS1TMP1	AMP	CPR	ERY	FUS	TMP	AMP+CPR	AMP+ERY	AMP+FUS	AMP+TMP	CPR+ERY	CPR+FUS	CPR+TMP	ERY+FUS	ERY+TMP	FUS+TMP	AMP+CPR+ERY	AMP+CPR+FUS	AMP+CPR+TMP	AMP+ERY+FUS	AMP+ERY+TMP	AMP+FUS+TMP	CPR+ERY+FUS	CPR+ERY+TMP	CPR+FUS+TMP	ERY+FUS+TMP	AMP+CPR+ERY+FUS	AMP+CPR+ERY+TMP	AMP+CPR+FUS+TMP	AMP+ERY+FUS+TMP	CPR+ERY+FUS+TMP	AMP+CPR+ERY+FUS+TMP
	96.36209814	98.3646914	103.8353601	70.23034992	103.9915597	84.17935702	106.8527919	11.06175973	89.59390863	97.52066116	67.17074028	80.92139968	69.71000935	113.414406	64.91999297	84.85617597	16.09560068	100.4230118	18.84517766	90.94754653	43.50676819	57.8864076	82.32811676	67.17074028	52.66604303	11.4001692	95.00846024	36.73857868	44.86040609	62.95058906	32.0642978
	86.9829222	96.74202128	102.1828909	82.6462766	100.4654255	68.15939279	76.35673624	16.66034156	103.3776091	87.96542553	51.7287234	98.86968085	77.95476893	110.993117	54.38829787	76.35673624	14.80075901	79.69639469	27.28652751	92.75142315	27.55218216	48.00531915	86.6356383	55.71808511	72.86135693	12.37191651	77.5711575	47.9316888	23.64326376	52.52659574	54.00379507
	71.44022848	90.62439214	88.62788963	74.01283797	112.410037	86.78090575	70.13463892	19.54304366	114.1982864	106.185567	70.5893795	124.8589769	82.06562267	107.7181208	71.21182649	60.99551204	22.48062016	95.26723786	16.27906977	98.85760914	31.94614443	58.4516631	101.2059911	68.41081502	56.7114094	17.58465932	80.57935537	32.27254182	27.19298246	59.0741101	36.33210934
	0	91.51456311	0	81.26213592	110.776699	0	0	0	0	98.03883495	85.00970874	113.5728155	0	0	72.89320388	0	0	0	0	0	0	65.12621359	114.1941748	69.16504854	0	0	0	0	0	63.26213592	0

AMP3CPR1ERY2FUS1TMP2	AMP	CPR	ERY	FUS	TMP	AMP+CPR	AMP+ERY	AMP+FUS	AMP+TMP	CPR+ERY	CPR+FUS	CPR+TMP	ERY+FUS	ERY+TMP	FUS+TMP	AMP+CPR+ERY	AMP+CPR+FUS	AMP+CPR+TMP	AMP+ERY+FUS	AMP+ERY+TMP	AMP+FUS+TMP	CPR+ERY+FUS	CPR+ERY+TMP	CPR+FUS+TMP	ERY+FUS+TMP	AMP+CPR+ERY+FUS	AMP+CPR+ERY+TMP	AMP+CPR+FUS+TMP	AMP+ERY+FUS+TMP	CPR+ERY+FUS+TMP	AMP+CPR+ERY+FUS+TMP
	96.36209814	98.3646914	103.8353601	70.23034992	99.49006506	84.17935702	106.8527919	11.06175973	98.05414552	97.52066116	67.17074028	79.79602602	69.71000935	120.2993452	64.63864955	84.85617597	16.09560068	88.91708968	18.84517766	99.74619289	18.80287648	57.8864076	86.26692456	72.79760858	62.84377923	11.4001692	115.3130288	17.82994924	14.06514382	59.01178126	9.031302876
	86.9829222	96.74202128	102.1828909	82.6462766	101.5292553	68.15939279	76.35673624	16.66034156	97.30550285	87.96542553	51.7287234	94.34840426	77.95476893	119.1740413	55.25265957	76.35673624	14.80075901	85.16129032	27.28652751	91.5370019	20.26565465	48.00531915	95.67819149	62.10106383	76.32251721	12.37191651	97.60910816	18.48197343	21.4800759	44.96343085	16.01518027
	71.44022848	90.62439214	88.62788963	74.01283797	109.297802	86.78090575	70.13463892	19.54304366	112.8926969	106.185567	70.5893795	110.2314725	82.06562267	112.7889635	60.94145108	60.99551204	22.48062016	91.67686659	16.27906977	108.6495308	23.78620971	58.4516631	102.1396615	73.39039097	63.87024609	17.58465932	81.55854753	27.0501836	17.58465932	54.09453414	18.05385557
	0	91.51456311	0	81.26213592	115.4368932	0	0	0	0	98.03883495	85.00970874	114.815534	0	0	72.58252427	0	0	0	0	0	0	65.12621359	114.815534	72.27184466	0	0	0	0	0	48.5631068	0

AMP3CPR1ERY2FUS1TMP3	AMP	CPR	ERY	FUS	TMP	AMP+CPR	AMP+ERY	AMP+FUS	AMP+TMP	CPR+ERY	CPR+FUS	CPR+TMP	ERY+FUS	ERY+TMP	FUS+TMP	AMP+CPR+ERY	AMP+CPR+FUS	AMP+CPR+TMP	AMP+ERY+FUS	AMP+ERY+TMP	AMP+FUS+TMP	CPR+ERY+FUS	CPR+ERY+TMP	CPR+FUS+TMP	ERY+FUS+TMP	AMP+CPR+ERY+FUS	AMP+CPR+ERY+TMP	AMP+CPR+FUS+TMP	AMP+ERY+FUS+TMP	CPR+ERY+FUS+TMP	AMP+CPR+ERY+FUS+TMP
	96.36209814	98.3646914	103.8353601	70.23034992	108.2117109	84.17935702	106.8527919	11.06175973	103.4686971	97.52066116	67.17074028	90.7684192	69.71000935	121.7960711	73.92298224	84.85617597	16.09560068	106.1759729	18.84517766	95.34686971	18.12605753	57.8864076	90.48707579	73.078952	66.41721235	11.4001692	60.82910321	20.55837563	15.7571912	69.14014419	24.93654822
	86.9829222	96.74202128	102.1828909	82.6462766	100.4654255	68.15939279	76.35673624	16.66034156	89.10815939	87.96542553	51.7287234	98.07180851	77.95476893	124.5231072	59.50797872	76.35673624	14.80075901	81.51802657	27.28652751	89.71537002	23.90891841	48.00531915	98.07180851	69.28191489	78.89872173	12.37191651	89.71537002	37.00189753	27.24857685	50.66489362	37.60910816
	71.44022848	90.62439214	88.62788963	74.01283797	115.8334954	86.78090575	70.13463892	19.54304366	108.9759282	106.185567	70.5893795	111.4763665	82.06562267	112.7889635	68.41081502	60.99551204	22.48062016	84.82252142	16.27906977	104.0799674	22.48062016	58.4516631	105.8743435	78.68119043	80.87248322	17.58465932	68.82904937	30.64055488	15.95267238	49.73740517	16.93186455
	0	91.51456311	0	81.26213592	108.2912621	0	0	0	0	98.03883495	85.00970874	117.9223301	0	0	74.75728155	0	0	0	0	0	0	65.12621359	116.6796117	77.55339806	0	0	0	0	0	53.00970874	0

AMP3CPR1ERY2FUS2TMP1	AMP	CPR	ERY	FUS	TMP	AMP+CPR	AMP+ERY	AMP+FUS	AMP+TMP	CPR+ERY	CPR+FUS	CPR+TMP	ERY+FUS	ERY+TMP	FUS+TMP	AMP+CPR+ERY	AMP+CPR+FUS	AMP+CPR+TMP	AMP+ERY+FUS	AMP+ERY+TMP	AMP+FUS+TMP	CPR+ERY+FUS	CPR+ERY+TMP	CPR+FUS+TMP	ERY+FUS+TMP	AMP+CPR+ERY+FUS	AMP+CPR+ERY+TMP	AMP+CPR+FUS+TMP	AMP+ERY+FUS+TMP	CPR+ERY+FUS+TMP	AMP+CPR+ERY+FUS+TMP
	96.36209814	98.3646914	103.8353601	85.70423774	103.9915597	84.17935702	106.8527919	57.78341794	89.59390863	97.52066116	76.41990505	80.92139968	76.59494855	113.414406	66.0101987	84.85617597	43.57021997	100.4230118	65.2284264	90.94754653	55.68950931	57.28855284	82.32811676	64.91999297	58.3536015	50	95.00846024	40.52453469	50.2749577	71.10954809	41.53976311
	86.9829222	96.74202128	102.1828909	95.94414894	100.4654255	68.15939279	76.35673624	48.72865275	103.3776091	87.96542553	77.59308511	98.86968085	89.91150442	110.993117	62.96542553	76.35673624	39.92409867	79.69639469	59.0512334	92.75142315	42.04933586	65.35904255	86.6356383	50.13297872	66.56833825	47.21062619	77.5711575	50.66413662	43.26375712	54.65425532	44.89563567
	71.44022848	90.62439214	88.62788963	96.53763859	112.410037	86.78090575	70.13463892	56.75234598	114.1982864	106.185567	67.28262984	124.8589769	83.55704698	107.7181208	67.47714452	60.99551204	48.91880865	95.26723786	58.05793554	98.85760914	35.53651571	71.21182649	101.2059911	61.87512157	74.01193139	40.90167279	80.57935537	51.20359037	47.28682171	82.1046489	39.77968176
	0	91.51456311	0	100.5242718	110.776699	0	0	0	0	98.03883495	74.13592233	113.5728155	0	0	66.36893204	0	0	0	0	0	0	84.69902913	114.1941748	63.26213592	0	0	0	0	0	74.13592233	0

AMP3CPR1ERY2FUS2TMP2	AMP	CPR	ERY	FUS	TMP	AMP+CPR	AMP+ERY	AMP+FUS	AMP+TMP	CPR+ERY	CPR+FUS	CPR+TMP	ERY+FUS	ERY+TMP	FUS+TMP	AMP+CPR+ERY	AMP+CPR+FUS	AMP+CPR+TMP	AMP+ERY+FUS	AMP+ERY+TMP	AMP+FUS+TMP	CPR+ERY+FUS	CPR+ERY+TMP	CPR+FUS+TMP	ERY+FUS+TMP	AMP+CPR+ERY+FUS	AMP+CPR+ERY+TMP	AMP+CPR+FUS+TMP	AMP+ERY+FUS+TMP	CPR+ERY+FUS+TMP	AMP+CPR+ERY+FUS+TMP
	96.36209814	98.3646914	103.8353601	85.70423774	99.49006506	84.17935702	106.8527919	57.78341794	98.05414552	97.52066116	76.41990505	79.79602602	76.59494855	120.2993452	67.17074028	84.85617597	43.57021997	88.91708968	65.2284264	99.74619289	55.68950931	57.28855284	86.26692456	71.95357834	64.02245089	50	115.3130288	58.79864636	46.27749577	68.85880077	44.18358714
	86.9829222	96.74202128	102.1828909	95.94414894	101.5292553	68.15939279	76.35673624	48.72865275	97.30550285	87.96542553	77.59308511	94.34840426	89.91150442	119.1740413	62.43351064	76.35673624	39.92409867	85.16129032	59.0512334	91.5370019	47.81783681	65.35904255	95.67819149	61.03723404	71.03244838	47.21062619	97.60910816	55.21821632	42.16318786	60.7712766	43.98481973
	71.44022848	90.62439214	88.62788963	96.53763859	109.297802	86.78090575	70.13463892	56.75234598	112.8926969	106.185567	67.28262984	110.2314725	83.55704698	112.7889635	74.63528496	60.99551204	48.91880865	91.67686659	58.05793554	108.6495308	49.57160343	71.21182649	102.1396615	67.47714452	82.96047726	40.90167279	81.55854753	65.89147287	46.96042432	74.01283797	48.91880865
	0	91.51456311	0	100.5242718	115.4368932	0	0	0	0	98.03883495	74.13592233	114.815534	0	0	63.86407767	0	0	0	0	0	0	84.69902913	114.815534	71.96116505	0	0	0	0	0	74.44660194	0

AMP3CPR1ERY2FUS2TMP3	AMP	CPR	ERY	FUS	TMP	AMP+CPR	AMP+ERY	AMP+FUS	AMP+TMP	CPR+ERY	CPR+FUS	CPR+TMP	ERY+FUS	ERY+TMP	FUS+TMP	AMP+CPR+ERY	AMP+CPR+FUS	AMP+CPR+TMP	AMP+ERY+FUS	AMP+ERY+TMP	AMP+FUS+TMP	CPR+ERY+FUS	CPR+ERY+TMP	CPR+FUS+TMP	ERY+FUS+TMP	AMP+CPR+ERY+FUS	AMP+CPR+ERY+TMP	AMP+CPR+FUS+TMP	AMP+ERY+FUS+TMP	CPR+ERY+FUS+TMP	AMP+CPR+ERY+FUS+TMP
	96.36209814	98.3646914	103.8353601	85.70423774	108.2117109	84.17935702	106.8527919	57.78341794	103.4686971	97.52066116	76.41990505	90.7684192	76.59494855	121.7960711	81.48408651	84.85617597	43.57021997	106.1759729	65.2284264	95.34686971	32.00084602	57.28855284	90.48707579	83.20731493	81.68381665	50	60.82910321	35.38494078	33.35448393	78.70582029	26.31133672
	86.9829222	96.74202128	102.1828909	95.94414894	100.4654255	68.15939279	76.35673624	48.72865275	89.10815939	87.96542553	77.59308511	98.07180851	89.91150442	124.5231072	78.65691489	76.35673624	39.92409867	81.51802657	59.0512334	89.71537002	35.4459203	65.35904255	98.07180851	65.55851064	94.3166175	47.21062619	89.71537002	36.66034156	30.01897533	56.78191489	33.9658444
	71.44022848	90.62439214	88.62788963	96.53763859	115.8334954	86.78090575	70.13463892	56.75234598	108.9759282	106.185567	67.28262984	111.4763665	83.55704698	112.7889635	83.4662517	60.99551204	48.91880865	84.82252142	58.05793554	104.0799674	40.10607915	71.21182649	105.8743435	66.54347403	91.90902312	40.90167279	68.82904937	54.46756426	36.51570787	70.900603	37.16850265
	0	91.51456311	0	100.5242718	108.2912621	0	0	0	0	98.03883495	74.13592233	117.9223301	0	0	97.72815534	0	0	0	0	0	0	84.69902913	116.6796117	81.59223301	0	0	0	0	0	73.51456311	0

AMP3CPR1ERY2FUS3TMP1	AMP	CPR	ERY	FUS	TMP	AMP+CPR	AMP+ERY	AMP+FUS	AMP+TMP	CPR+ERY	CPR+FUS	CPR+TMP	ERY+FUS	ERY+TMP	FUS+TMP	AMP+CPR+ERY	AMP+CPR+FUS	AMP+CPR+TMP	AMP+ERY+FUS	AMP+ERY+TMP	AMP+FUS+TMP	CPR+ERY+FUS	CPR+ERY+TMP	CPR+FUS+TMP	ERY+FUS+TMP	AMP+CPR+ERY+FUS	AMP+CPR+ERY+TMP	AMP+CPR+FUS+TMP	AMP+ERY+FUS+TMP	CPR+ERY+FUS+TMP	AMP+CPR+ERY+FUS+TMP
	96.36209814	98.3646914	103.8353601	98.64603482	103.9915597	84.17935702	106.8527919	82.82571912	89.59390863	97.52066116	84.86020749	80.92139968	106.828812	113.414406	75.01318797	84.85617597	82.14890017	100.4230118	89.9323181	90.94754653	86.88663283	82.89080359	82.32811676	60.10198699	85.8746492	83.1641286	95.00846024	77.41116751	73.01184433	58.44909443	66.5820643
	86.9829222	96.74202128	102.1828909	101.7952128	100.4654255	68.15939279	76.35673624	76.66034156	103.3776091	87.96542553	97.53989362	98.86968085	105.6440511	110.993117	69.6143617	76.35673624	79.08918406	79.69639469	77.87476281	92.75142315	76.05313093	88.49734043	86.6356383	58.17819149	72.92035398	78.78557875	77.5711575	63.30170778	69.07020873	51.79521277	69.98102467
	71.44022848	90.62439214	88.62788963	112.410037	112.410037	86.78090575	70.13463892	91.67686659	114.1982864	106.185567	107.7416845	124.8589769	102.945563	107.7181208	78.48667574	60.99551204	74.70420237	95.26723786	80.90575275	98.85760914	87.10730314	91.86928613	101.2059911	62.92550088	77.59134974	68.50265198	80.57935537	70.46103631	74.70420237	59.38533359	73.7250102
	0	91.51456311	0	108.2912621	110.776699	0	0	0	0	98.03883495	107.0485437	113.5728155	0	0	83.12621359	0	0	0	0	0	0	94.93203883	114.1941748	75.04854369	0	0	0	0	0	61.68932039	0

AMP3CPR1ERY2FUS3TMP2	AMP	CPR	ERY	FUS	TMP	AMP+CPR	AMP+ERY	AMP+FUS	AMP+TMP	CPR+ERY	CPR+FUS	CPR+TMP	ERY+FUS	ERY+TMP	FUS+TMP	AMP+CPR+ERY	AMP+CPR+FUS	AMP+CPR+TMP	AMP+ERY+FUS	AMP+ERY+TMP	AMP+FUS+TMP	CPR+ERY+FUS	CPR+ERY+TMP	CPR+FUS+TMP	ERY+FUS+TMP	AMP+CPR+ERY+FUS	AMP+CPR+ERY+TMP	AMP+CPR+FUS+TMP	AMP+ERY+FUS+TMP	CPR+ERY+FUS+TMP	AMP+CPR+ERY+FUS+TMP
	96.36209814	98.3646914	103.8353601	98.64603482	99.49006506	84.17935702	106.8527919	82.82571912	98.05414552	97.52066116	84.86020749	79.79602602	106.828812	120.2993452	86.26692456	84.85617597	82.14890017	88.91708968	89.9323181	99.74619289	81.47208122	82.89080359	86.26692456	77.54527871	93.95696913	83.1641286	115.3130288	82.48730964	64.89001692	65.72885528	68.61252115
	86.9829222	96.74202128	102.1828909	101.7952128	101.5292553	68.15939279	76.35673624	76.66034156	97.30550285	87.96542553	97.53989362	94.34840426	105.6440511	119.1740413	82.6462766	76.35673624	79.08918406	85.16129032	77.87476281	91.5370019	74.23149905	88.49734043	95.67819149	76.52925532	89.28220256	78.78557875	97.60910816	67.55218216	70.58823529	65.625	60.87286528
	71.44022848	90.62439214	88.62788963	112.410037	109.297802	86.78090575	70.13463892	91.67686659	112.8926969	106.185567	107.7416845	110.2314725	102.945563	112.7889635	95.2927446	60.99551204	74.70420237	91.67686659	80.90575275	108.6495308	81.55854753	91.86928613	102.1396615	83.4662517	99.66442953	68.50265198	81.55854753	82.5377397	75.35699714	75.68566427	64.58588331
	0	91.51456311	0	108.2912621	115.4368932	0	0	0	0	98.03883495	107.0485437	114.815534	0	0	89.65048544	0	0	0	0	0	0	94.93203883	114.815534	88.7184466	0	0	0	0	0	73.18446602	0

AMP3CPR1ERY2FUS3TMP3	AMP	CPR	ERY	FUS	TMP	AMP+CPR	AMP+ERY	AMP+FUS	AMP+TMP	CPR+ERY	CPR+FUS	CPR+TMP	ERY+FUS	ERY+TMP	FUS+TMP	AMP+CPR+ERY	AMP+CPR+FUS	AMP+CPR+TMP	AMP+ERY+FUS	AMP+ERY+TMP	AMP+FUS+TMP	CPR+ERY+FUS	CPR+ERY+TMP	CPR+FUS+TMP	ERY+FUS+TMP	AMP+CPR+ERY+FUS	AMP+CPR+ERY+TMP	AMP+CPR+FUS+TMP	AMP+ERY+FUS+TMP	CPR+ERY+FUS+TMP	AMP+CPR+ERY+FUS+TMP
	96.36209814	98.3646914	103.8353601	98.64603482	108.2117109	84.17935702	106.8527919	82.82571912	103.4686971	97.52066116	84.86020749	90.7684192	106.828812	121.7960711	105.9609636	84.85617597	82.14890017	106.1759729	89.9323181	95.34686971	55.68950931	82.89080359	90.48707579	94.14454018	119.1019645	83.1641286	60.82910321	74.02707276	46.21404399	73.6064709	59.13705584
	86.9829222	96.74202128	102.1828909	101.7952128	100.4654255	68.15939279	76.35673624	76.66034156	89.10815939	87.96542553	97.53989362	98.07180851	105.6440511	124.5231072	105.2526596	76.35673624	79.08918406	81.51802657	77.87476281	89.71537002	56.9259962	88.49734043	98.07180851	94.88031915	109.4198623	78.78557875	89.71537002	52.97912713	52.67552182	77.06117021	69.67741935
	71.44022848	90.62439214	88.62788963	112.410037	115.8334954	86.78090575	70.13463892	91.67686659	108.9759282	106.185567	107.7416845	111.4763665	102.945563	112.7889635	109.920249	60.99551204	74.70420237	84.82252142	80.90575275	104.0799674	73.07221542	91.86928613	105.8743435	100.5835441	111.8941089	68.50265198	68.82904937	76.00979192	69.15544676	64.79284186	60.01631987
	0	91.51456311	0	108.2912621	108.2912621	0	0	0	0	98.03883495	107.0485437	117.9223301	0	0	111.0873786	0	0	0	0	0	0	94.93203883	116.6796117	107.9805825	0	0	0	0	0	75.04854369	0

AMP3CPR1ERY3FUS1TMP1	AMP	CPR	ERY	FUS	TMP	AMP+CPR	AMP+ERY	AMP+FUS	AMP+TMP	CPR+ERY	CPR+FUS	CPR+TMP	ERY+FUS	ERY+TMP	FUS+TMP	AMP+CPR+ERY	AMP+CPR+FUS	AMP+CPR+TMP	AMP+ERY+FUS	AMP+ERY+TMP	AMP+FUS+TMP	CPR+ERY+FUS	CPR+ERY+TMP	CPR+FUS+TMP	ERY+FUS+TMP	AMP+CPR+ERY+FUS	AMP+CPR+ERY+TMP	AMP+CPR+FUS+TMP	AMP+ERY+FUS+TMP	CPR+ERY+FUS+TMP	AMP+CPR+ERY+FUS+TMP
	96.36209814	98.3646914	95.34686971	70.23034992	103.9915597	84.17935702	109.5600677	11.06175973	89.59390863	99.20872165	67.17074028	80.92139968	80.79526227	114.6362098	64.91999297	84.85617597	16.09560068	100.4230118	21.89086294	89.9323181	43.50676819	60.98118516	86.54826798	67.17074028	60.08883249	19.47969543	97.03891709	36.73857868	58.05837563	64.91999297	32.40270728
	86.9829222	96.74202128	99.12713472	82.6462766	100.4654255	68.15939279	84.25047438	16.66034156	103.3776091	96.74202128	51.7287234	98.86968085	90.32258065	114.914611	54.38829787	70.58823529	14.80075901	79.69639469	23.83301708	98.82352941	27.55218216	54.65425532	97.27393617	55.71808511	56.31878558	20.79696395	83.33965844	47.9316888	41.51802657	55.98404255	47.62808349
	71.44022848	90.62439214	108.9759282	74.01283797	112.410037	86.78090575	68.50265198	19.54304366	114.1982864	98.71620307	70.5893795	124.8589769	93.63525092	120.3998368	71.21182649	62.30110159	22.48062016	95.26723786	32.27254182	100.4895961	31.94614443	80.85975491	110.8539195	68.41081502	62.95389637	24.03100775	88.08649531	32.27254182	50.36719706	64.98735655	30.96695226
	0	91.51456311	0	81.26213592	110.776699	0	0	0	0	100.2135922	85.00970874	113.5728155	0	0	72.89320388	0	0	0	0	0	0	83.14563107	110.4660194	69.16504854	0	0	0	0	0	70.09708738	0

AMP3CPR1ERY3FUS1TMP2	AMP	CPR	ERY	FUS	TMP	AMP+CPR	AMP+ERY	AMP+FUS	AMP+TMP	CPR+ERY	CPR+FUS	CPR+TMP	ERY+FUS	ERY+TMP	FUS+TMP	AMP+CPR+ERY	AMP+CPR+FUS	AMP+CPR+TMP	AMP+ERY+FUS	AMP+ERY+TMP	AMP+FUS+TMP	CPR+ERY+FUS	CPR+ERY+TMP	CPR+FUS+TMP	ERY+FUS+TMP	AMP+CPR+ERY+FUS	AMP+CPR+ERY+TMP	AMP+CPR+FUS+TMP	AMP+ERY+FUS+TMP	CPR+ERY+FUS+TMP	AMP+CPR+ERY+FUS+TMP
	96.36209814	98.3646914	95.34686971	70.23034992	99.49006506	84.17935702	109.5600677	11.06175973	98.05414552	99.20872165	67.17074028	79.79602602	80.79526227	114.9746193	64.63864955	84.85617597	16.09560068	88.91708968	21.89086294	99.06937394	18.80287648	60.98118516	87.11095481	72.79760858	72.2715736	19.47969543	126.4805415	17.82994924	17.78764805	66.88939687	28.34179357
	86.9829222	96.74202128	99.12713472	82.6462766	101.5292553	68.15939279	84.25047438	16.66034156	97.30550285	96.74202128	51.7287234	94.34840426	90.32258065	115.2182163	55.25265957	70.58823529	14.80075901	85.16129032	23.83301708	102.1631879	20.26565465	54.65425532	101.5292553	62.10106383	65.73055028	20.79696395	90.62618596	18.48197343	25.12333966	56.78191489	16.96394687
	71.44022848	90.62439214	108.9759282	74.01283797	109.297802	86.78090575	68.50265198	19.54304366	112.8926969	98.71620307	70.5893795	110.2314725	93.63525092	125.2957976	60.94145108	62.30110159	22.48062016	91.67686659	32.27254182	110.2815177	23.78620971	80.85975491	109.6090255	73.39039097	80.57935537	24.03100775	84.49612403	27.0501836	21.82782538	79.92608442	25.09179927
	0	91.51456311	0	81.26213592	115.4368932	0	0	0	0	100.2135922	85.00970874	114.815534	0	0	72.58252427	0	0	0	0	0	0	83.14563107	111.3980583	72.27184466	0	0	0	0	0	71.65048544	0

AMP3CPR1ERY3FUS1TMP3	AMP	CPR	ERY	FUS	TMP	AMP+CPR	AMP+ERY	AMP+FUS	AMP+TMP	CPR+ERY	CPR+FUS	CPR+TMP	ERY+FUS	ERY+TMP	FUS+TMP	AMP+CPR+ERY	AMP+CPR+FUS	AMP+CPR+TMP	AMP+ERY+FUS	AMP+ERY+TMP	AMP+FUS+TMP	CPR+ERY+FUS	CPR+ERY+TMP	CPR+FUS+TMP	ERY+FUS+TMP	AMP+CPR+ERY+FUS	AMP+CPR+ERY+TMP	AMP+CPR+FUS+TMP	AMP+ERY+FUS+TMP	CPR+ERY+FUS+TMP	AMP+CPR+ERY+FUS+TMP
	96.36209814	98.3646914	95.34686971	70.23034992	108.2117109	84.17935702	109.5600677	11.06175973	103.4686971	99.20872165	67.17074028	90.7684192	80.79526227	115.6514382	73.92298224	84.85617597	16.09560068	106.1759729	21.89086294	94.67005076	18.12605753	60.98118516	88.51767188	73.078952	83.84094755	19.47969543	91.96277496	20.55837563	20.83333333	75.32969931	25.61336717
	86.9829222	96.74202128	99.12713472	82.6462766	100.4654255	68.15939279	84.25047438	16.66034156	89.10815939	96.74202128	51.7287234	98.07180851	90.32258065	107.0208729	59.50797872	70.58823529	14.80075901	81.51802657	23.83301708	93.35863378	23.90891841	54.65425532	103.3909574	69.28191489	81.82163188	20.79696395	85.16129032	37.00189753	21.78368121	66.35638298	33.9658444
	71.44022848	90.62439214	108.9759282	74.01283797	115.8334954	86.78090575	68.50265198	19.54304366	108.9759282	98.71620307	70.5893795	111.4763665	93.63525092	117.1358629	68.41081502	62.30110159	22.48062016	84.82252142	32.27254182	104.4063647	22.48062016	80.85975491	108.052908	78.68119043	90.04487964	24.03100775	76.6625867	30.64055488	23.78620971	86.46177786	29.00856793
	0	91.51456311	0	81.26213592	108.2912621	0	0	0	0	100.2135922	85.00970874	117.9223301	0	0	74.75728155	0	0	0	0	0	0	83.14563107	112.0194175	77.55339806	0	0	0	0	0	71.96116505	0

AMP3CPR1ERY3FUS2TMP1	AMP	CPR	ERY	FUS	TMP	AMP+CPR	AMP+ERY	AMP+FUS	AMP+TMP	CPR+ERY	CPR+FUS	CPR+TMP	ERY+FUS	ERY+TMP	FUS+TMP	AMP+CPR+ERY	AMP+CPR+FUS	AMP+CPR+TMP	AMP+ERY+FUS	AMP+ERY+TMP	AMP+FUS+TMP	CPR+ERY+FUS	CPR+ERY+TMP	CPR+FUS+TMP	ERY+FUS+TMP	AMP+CPR+ERY+FUS	AMP+CPR+ERY+TMP	AMP+CPR+FUS+TMP	AMP+ERY+FUS+TMP	CPR+ERY+FUS+TMP	AMP+CPR+ERY+FUS+TMP
	96.36209814	98.3646914	95.34686971	85.70423774	103.9915597	84.17935702	109.5600677	57.78341794	89.59390863	99.20872165	76.41990505	80.92139968	84.17935702	114.6362098	66.0101987	84.85617597	43.57021997	100.4230118	51.69204738	89.9323181	55.68950931	72.48109724	86.54826798	64.91999297	69.2893401	44.58544839	97.03891709	40.52453469	48.58291032	63.79461931	40.18612521
	86.9829222	96.74202128	99.12713472	95.94414894	100.4654255	68.15939279	84.25047438	48.72865275	103.3776091	96.74202128	77.59308511	98.86968085	93.05502846	114.914611	62.96542553	70.58823529	39.92409867	79.69639469	55.10436433	98.82352941	42.04933586	69.08244681	97.27393617	50.13297872	74.23149905	43.26375712	83.33965844	50.66413662	37.5711575	43.21808511	49.44971537
	71.44022848	90.62439214	108.9759282	96.53763859	112.410037	86.78090575	68.50265198	56.75234598	114.1982864	98.71620307	67.28262984	124.8589769	103.75357	120.3998368	67.47714452	62.30110159	48.91880865	95.26723786	53.81476948	100.4895961	35.53651571	71.21182649	110.8539195	61.87512157	78.29457364	51.20359037	88.08649531	51.20359037	32.9253366	71.21182649	46.30762954
	0	91.51456311	0	100.5242718	110.776699	0	0	0	0	100.2135922	74.13592233	113.5728155	0	0	66.36893204	0	0	0	0	0	0	73.51456311	110.4660194	63.26213592	0	0	0	0	0	61.39805825	0

AMP3CPR1ERY3FUS2TMP2	AMP	CPR	ERY	FUS	TMP	AMP+CPR	AMP+ERY	AMP+FUS	AMP+TMP	CPR+ERY	CPR+FUS	CPR+TMP	ERY+FUS	ERY+TMP	FUS+TMP	AMP+CPR+ERY	AMP+CPR+FUS	AMP+CPR+TMP	AMP+ERY+FUS	AMP+ERY+TMP	AMP+FUS+TMP	CPR+ERY+FUS	CPR+ERY+TMP	CPR+FUS+TMP	ERY+FUS+TMP	AMP+CPR+ERY+FUS	AMP+CPR+ERY+TMP	AMP+CPR+FUS+TMP	AMP+ERY+FUS+TMP	CPR+ERY+FUS+TMP	AMP+CPR+ERY+FUS+TMP
	96.36209814	98.3646914	95.34686971	85.70423774	99.49006506	84.17935702	109.5600677	57.78341794	98.05414552	99.20872165	76.41990505	79.79602602	84.17935702	114.9746193	67.17074028	84.85617597	43.57021997	88.91708968	51.69204738	99.06937394	55.68950931	72.48109724	87.11095481	71.95357834	85.8714044	44.58544839	126.4805415	58.79864636	44.24703892	53.34974503	40.18612521
	86.9829222	96.74202128	99.12713472	95.94414894	101.5292553	68.15939279	84.25047438	48.72865275	97.30550285	96.74202128	77.59308511	94.34840426	93.05502846	115.2182163	62.43351064	70.58823529	39.92409867	85.16129032	55.10436433	102.1631879	47.81783681	69.08244681	101.5292553	61.03723404	88.19734345	43.26375712	90.62618596	55.21821632	44.17457306	60.50531915	45.80645161
	71.44022848	90.62439214	108.9759282	96.53763859	109.297802	86.78090575	68.50265198	56.75234598	112.8926969	98.71620307	67.28262984	110.2314725	103.75357	125.2957976	74.63528496	62.30110159	48.91880865	91.67686659	53.81476948	110.2815177	49.57160343	71.21182649	109.6090255	67.47714452	85.14891881	51.20359037	84.49612403	65.89147287	59.68992248	73.39039097	58.05793554
	0	91.51456311	0	100.5242718	115.4368932	0	0	0	0	100.2135922	74.13592233	114.815534	0	0	63.86407767	0	0	0	0	0	0	73.51456311	111.3980583	71.96116505	0	0	0	0	0	68.23300971	0

AMP3CPR1ERY3FUS2TMP3	AMP	CPR	ERY	FUS	TMP	AMP+CPR	AMP+ERY	AMP+FUS	AMP+TMP	CPR+ERY	CPR+FUS	CPR+TMP	ERY+FUS	ERY+TMP	FUS+TMP	AMP+CPR+ERY	AMP+CPR+FUS	AMP+CPR+TMP	AMP+ERY+FUS	AMP+ERY+TMP	AMP+FUS+TMP	CPR+ERY+FUS	CPR+ERY+TMP	CPR+FUS+TMP	ERY+FUS+TMP	AMP+CPR+ERY+FUS	AMP+CPR+ERY+TMP	AMP+CPR+FUS+TMP	AMP+ERY+FUS+TMP	CPR+ERY+FUS+TMP	AMP+CPR+ERY+FUS+TMP
	96.36209814	98.3646914	95.34686971	85.70423774	108.2117109	84.17935702	109.5600677	57.78341794	103.4686971	99.20872165	76.41990505	90.7684192	84.17935702	115.6514382	81.48408651	84.85617597	43.57021997	106.1759729	51.69204738	94.67005076	32.00084602	72.48109724	88.51767188	83.20731493	98.39255499	44.58544839	91.96277496	35.38494078	29.6319797	73.36029541	46.55245347
	86.9829222	96.74202128	99.12713472	95.94414894	100.4654255	68.15939279	84.25047438	48.72865275	89.10815939	96.74202128	77.59308511	98.07180851	93.05502846	107.0208729	78.65691489	70.58823529	39.92409867	81.51802657	55.10436433	93.35863378	35.4459203	69.08244681	103.3909574	65.55851064	93.9658444	43.26375712	85.16129032	36.66034156	39.69639469	59.70744681	34.53510436
	71.44022848	90.62439214	108.9759282	96.53763859	115.8334954	86.78090575	68.50265198	56.75234598	108.9759282	98.71620307	67.28262984	111.4763665	103.75357	117.1358629	83.4662517	62.30110159	48.91880865	84.82252142	53.81476948	104.4063647	40.10607915	71.21182649	108.052908	66.54347403	92.65605875	51.20359037	76.6625867	54.46756426	35.8629131	71.21182649	49.57160343
	0	91.51456311	0	100.5242718	108.2912621	0	0	0	0	100.2135922	74.13592233	117.9223301	0	0	97.72815534	0	0	0	0	0	0	73.51456311	112.0194175	81.59223301	0	0	0	0	0	77.55339806	0

AMP3CPR1ERY3FUS3TMP1	AMP	CPR	ERY	FUS	TMP	AMP+CPR	AMP+ERY	AMP+FUS	AMP+TMP	CPR+ERY	CPR+FUS	CPR+TMP	ERY+FUS	ERY+TMP	FUS+TMP	AMP+CPR+ERY	AMP+CPR+FUS	AMP+CPR+TMP	AMP+ERY+FUS	AMP+ERY+TMP	AMP+FUS+TMP	CPR+ERY+FUS	CPR+ERY+TMP	CPR+FUS+TMP	ERY+FUS+TMP	AMP+CPR+ERY+FUS	AMP+CPR+ERY+TMP	AMP+CPR+FUS+TMP	AMP+ERY+FUS+TMP	CPR+ERY+FUS+TMP	AMP+CPR+ERY+FUS+TMP
	96.36209814	98.3646914	95.34686971	98.64603482	103.9915597	84.17935702	109.5600677	82.82571912	89.59390863	99.20872165	84.86020749	80.92139968	102.1150592	114.6362098	75.01318797	84.85617597	82.14890017	100.4230118	85.19458545	89.9323181	86.88663283	84.29752066	86.54826798	60.10198699	79.44162437	82.14890017	97.03891709	77.41116751	82.48730964	56.72586601	68.61252115
	86.9829222	96.74202128	99.12713472	101.7952128	100.4654255	68.15939279	84.25047438	76.66034156	103.3776091	96.74202128	97.53989362	98.86968085	107.9316888	114.914611	69.6143617	70.58823529	79.08918406	79.69639469	82.73244782	98.82352941	76.05313093	97.00797872	97.27393617	58.17819149	84.25047438	76.05313093	83.33965844	63.30170778	83.64326376	59.24202128	71.49905123
	71.44022848	90.62439214	108.9759282	112.410037	112.410037	86.78090575	68.50265198	91.67686659	114.1982864	98.71620307	107.7416845	124.8589769	115.1774786	120.3998368	78.48667574	62.30110159	74.70420237	95.26723786	82.86413709	100.4895961	87.10730314	107.1192375	110.8539195	62.92550088	94.94084047	76.98898409	88.08649531	70.46103631	83.84332925	64.48161836	72.41942064
	0	91.51456311	0	108.2912621	110.776699	0	0	0	0	100.2135922	107.0485437	113.5728155	0	0	83.12621359	0	0	0	0	0	0	107.3592233	110.4660194	75.04854369	0	0	0	0	0	70.69902913	0

AMP3CPR1ERY3FUS3TMP2	AMP	CPR	ERY	FUS	TMP	AMP+CPR	AMP+ERY	AMP+FUS	AMP+TMP	CPR+ERY	CPR+FUS	CPR+TMP	ERY+FUS	ERY+TMP	FUS+TMP	AMP+CPR+ERY	AMP+CPR+FUS	AMP+CPR+TMP	AMP+ERY+FUS	AMP+ERY+TMP	AMP+FUS+TMP	CPR+ERY+FUS	CPR+ERY+TMP	CPR+FUS+TMP	ERY+FUS+TMP	AMP+CPR+ERY+FUS	AMP+CPR+ERY+TMP	AMP+CPR+FUS+TMP	AMP+ERY+FUS+TMP	CPR+ERY+FUS+TMP	AMP+CPR+ERY+FUS+TMP
	96.36209814	98.3646914	95.34686971	98.64603482	99.49006506	84.17935702	109.5600677	82.82571912	98.05414552	99.20872165	84.86020749	79.79602602	102.1150592	114.9746193	86.26692456	84.85617597	82.14890017	88.91708968	85.19458545	99.06937394	81.47208122	84.29752066	87.11095481	77.54527871	106.1759729	82.14890017	126.4805415	82.48730964	66.92047377	76.13856163	77.74957699
	86.9829222	96.74202128	99.12713472	101.7952128	101.5292553	68.15939279	84.25047438	76.66034156	97.30550285	96.74202128	97.53989362	94.34840426	107.9316888	115.2182163	82.6462766	70.58823529	79.08918406	85.16129032	82.73244782	102.1631879	74.23149905	97.00797872	101.5292553	76.52925532	101.8595825	76.05313093	90.62618596	67.55218216	69.07020873	65.89095745	65.12333966
	71.44022848	90.62439214	108.9759282	112.410037	109.297802	86.78090575	68.50265198	91.67686659	112.8926969	98.71620307	107.7416845	110.2314725	115.1774786	125.2957976	95.2927446	62.30110159	74.70420237	91.67686659	82.86413709	110.2815177	81.55854753	107.1192375	109.6090255	83.4662517	106.6911465	76.98898409	84.49612403	82.5377397	76.33618931	75.37444077	72.74581803
	0	91.51456311	0	108.2912621	115.4368932	0	0	0	0	100.2135922	107.0485437	114.815534	0	0	89.65048544	0	0	0	0	0	0	107.3592233	111.3980583	88.7184466	0	0	0	0	0	77.22330097	0

AMP3CPR1ERY3FUS3TMP3	AMP	CPR	ERY	FUS	TMP	AMP+CPR	AMP+ERY	AMP+FUS	AMP+TMP	CPR+ERY	CPR+FUS	CPR+TMP	ERY+FUS	ERY+TMP	FUS+TMP	AMP+CPR+ERY	AMP+CPR+FUS	AMP+CPR+TMP	AMP+ERY+FUS	AMP+ERY+TMP	AMP+FUS+TMP	CPR+ERY+FUS	CPR+ERY+TMP	CPR+FUS+TMP	ERY+FUS+TMP	AMP+CPR+ERY+FUS	AMP+CPR+ERY+TMP	AMP+CPR+FUS+TMP	AMP+ERY+FUS+TMP	CPR+ERY+FUS+TMP	AMP+CPR+ERY+FUS+TMP
	96.36209814	98.3646914	95.34686971	98.64603482	108.2117109	84.17935702	109.5600677	82.82571912	103.4686971	99.20872165	84.86020749	90.7684192	102.1150592	115.6514382	105.9609636	84.85617597	82.14890017	106.1759729	85.19458545	94.67005076	55.68950931	84.29752066	88.51767188	94.14454018	114.2978003	82.14890017	91.96277496	74.02707276	67.19543147	87.67364164	76.05752961
	86.9829222	96.74202128	99.12713472	101.7952128	100.4654255	68.15939279	84.25047438	76.66034156	89.10815939	96.74202128	97.53989362	98.07180851	107.9316888	107.0208729	105.2526596	70.58823529	79.08918406	81.51802657	82.73244782	93.35863378	56.9259962	97.00797872	103.3909574	94.88031915	114.3074004	76.05313093	85.16129032	52.97912713	70.89184061	87.16755319	51.15749526
	71.44022848	90.62439214	108.9759282	112.410037	115.8334954	86.78090575	68.50265198	91.67686659	108.9759282	98.71620307	107.7416845	111.4763665	115.1774786	117.1358629	109.920249	62.30110159	74.70420237	84.82252142	82.86413709	104.4063647	73.07221542	107.1192375	108.052908	100.5835441	128.5597715	76.98898409	76.6625867	76.00979192	84.16972664	99.33865007	66.87066503
	0	91.51456311	0	108.2912621	108.2912621	0	0	0	0	100.2135922	107.0485437	117.9223301	0	0	111.0873786	0	0	0	0	0	0	107.3592233	112.0194175	107.9805825	0	0	0	0	0	106.7378641	0

AMP3CPR2ERY1FUS1TMP1	AMP	CPR	ERY	FUS	TMP	AMP+CPR	AMP+ERY	AMP+FUS	AMP+TMP	CPR+ERY	CPR+FUS	CPR+TMP	ERY+FUS	ERY+TMP	FUS+TMP	AMP+CPR+ERY	AMP+CPR+FUS	AMP+CPR+TMP	AMP+ERY+FUS	AMP+ERY+TMP	AMP+FUS+TMP	CPR+ERY+FUS	CPR+ERY+TMP	CPR+FUS+TMP	ERY+FUS+TMP	AMP+CPR+ERY+FUS	AMP+CPR+ERY+TMP	AMP+CPR+FUS+TMP	AMP+ERY+FUS+TMP	CPR+ERY+FUS+TMP	AMP+CPR+ERY+FUS+TMP
	96.36209814	109.8222638	95.26991384	70.23034992	103.9915597	99.06937394	92.97800338	11.06175973	89.59390863	86.47333957	70.92609916	110.121609	60.3833304	89.36170213	64.91999297	84.17935702	38.15566836	117.3434856	14.78426396	97.37732657	43.50676819	52.06735267	84.97661366	62.84377923	55.91700369	25.57106599	103.1302876	49.59813875	44.18358714	46.67913938	39.78426396
	86.9829222	103.7561455	101.2632979	82.6462766	100.4654255	70.89184061	80	16.66034156	103.3776091	99.98033432	72.546706	109.4198623	63.49734043	96.21010638	54.38829787	58.14041746	39.08918406	84.85768501	22.4288425	89.10815939	27.55218216	62.79252704	89.59685349	84.18879056	55.98404255	32.14421252	78.48197343	50.66413662	29.67741935	68.77089479	45.19924099
	71.44022848	83.55704698	94.35907411	74.01283797	112.410037	75.35699714	82.5377397	19.54304366	114.1982864	87.73303505	56.54362416	113.3855332	74.94650846	109.297802	71.21182649	62.30110159	27.37658099	104.4063647	19.86944104	96.89922481	31.94614443	56.2453393	95.78672632	56.54362416	72.45672048	20.84863321	100.8159935	51.52998776	35.02651979	49.38478747	28.17217462
	0	0	97.72815534	81.26213592	110.776699	0	0	0	0	0	0	0	76	112.6407767	72.89320388	0	0	0	0	0	0	0	0	0	71.33980583	0	0	0	0	0	0

AMP3CPR2ERY1FUS1TMP2	AMP	CPR	ERY	FUS	TMP	AMP+CPR	AMP+ERY	AMP+FUS	AMP+TMP	CPR+ERY	CPR+FUS	CPR+TMP	ERY+FUS	ERY+TMP	FUS+TMP	AMP+CPR+ERY	AMP+CPR+FUS	AMP+CPR+TMP	AMP+ERY+FUS	AMP+ERY+TMP	AMP+FUS+TMP	CPR+ERY+FUS	CPR+ERY+TMP	CPR+FUS+TMP	ERY+FUS+TMP	AMP+CPR+ERY+FUS	AMP+CPR+ERY+TMP	AMP+CPR+FUS+TMP	AMP+ERY+FUS+TMP	CPR+ERY+FUS+TMP	AMP+CPR+ERY+FUS+TMP
	96.36209814	109.8222638	95.26991384	70.23034992	99.49006506	99.06937394	92.97800338	11.06175973	98.05414552	86.47333957	70.92609916	111.3189897	60.3833304	87.95498505	64.63864955	84.17935702	38.15566836	114.2978003	14.78426396	101.4382403	18.80287648	52.06735267	106.828812	72.42282507	58.44909443	25.57106599	113.9593909	38.43062606	2.897631134	51.61833489	22.18697124
	86.9829222	103.7561455	101.2632979	82.6462766	101.5292553	70.89184061	80	16.66034156	97.30550285	99.98033432	72.546706	116.027532	63.49734043	94.6143617	55.25265957	58.14041746	39.08918406	85.46489564	22.4288425	93.9658444	20.26565465	62.79252704	104.7000983	86.3913471	53.85638298	32.14421252	83.64326376	41.25237192	11.76470588	59.33136676	18.48197343
	71.44022848	83.55704698	94.35907411	74.01283797	109.297802	75.35699714	82.5377397	19.54304366	112.8926969	87.73303505	56.54362416	119.0529456	74.94650846	109.920249	60.94145108	62.30110159	27.37658099	101.4687882	19.86944104	112.2399021	23.78620971	56.2453393	108.3146905	63.10589113	69.03326201	20.84863321	94.94084047	29.00856793	17.25826193	45.84265474	24.76540188
	0	0	97.72815534	81.26213592	115.4368932	0	0	0	0	0	0	0	76	119.1650485	72.58252427	0	0	0	0	0	0	0	0	0	65.74757282	0	0	0	0	0	0

AMP3CPR2ERY1FUS1TMP3	AMP	CPR	ERY	FUS	TMP	AMP+CPR	AMP+ERY	AMP+FUS	AMP+TMP	CPR+ERY	CPR+FUS	CPR+TMP	ERY+FUS	ERY+TMP	FUS+TMP	AMP+CPR+ERY	AMP+CPR+FUS	AMP+CPR+TMP	AMP+ERY+FUS	AMP+ERY+TMP	AMP+FUS+TMP	CPR+ERY+FUS	CPR+ERY+TMP	CPR+FUS+TMP	ERY+FUS+TMP	AMP+CPR+ERY+FUS	AMP+CPR+ERY+TMP	AMP+CPR+FUS+TMP	AMP+ERY+FUS+TMP	CPR+ERY+FUS+TMP	AMP+CPR+ERY+FUS+TMP
	96.36209814	109.8222638	95.26991384	70.23034992	108.2117109	99.06937394	92.97800338	11.06175973	103.4686971	86.47333957	70.92609916	124.4901777	60.3833304	91.04976262	73.92298224	84.17935702	38.15566836	92.97800338	14.78426396	99.06937394	18.12605753	52.06735267	117.9045837	77.81103835	67.4520837	25.57106599	100.7614213	39.44585448	1.924703892	59.55098223	20.49492386
	86.9829222	103.7561455	101.2632979	82.6462766	100.4654255	70.89184061	80	16.66034156	89.10815939	99.98033432	72.546706	123.2645034	63.49734043	94.88031915	59.50797872	58.14041746	39.08918406	90.62618596	22.4288425	88.50094877	23.90891841	62.79252704	113.5103245	90.79646018	51.99468085	32.14421252	75.4459203	32.75142315	28.15939279	68.14159292	45.80645161
	71.44022848	83.55704698	94.35907411	74.01283797	115.8334954	75.35699714	82.5377397	19.54304366	108.9759282	87.73303505	56.54362416	119.3512304	74.94650846	108.3641315	68.41081502	62.30110159	27.37658099	100.8159935	19.86944104	103.4271726	22.48062016	56.2453393	110.7009694	64.00074571	70.278156	20.84863321	75.03059976	46.96042432	16.60546716	55.05219985	20.84863321
	0	0	97.72815534	81.26213592	108.2912621	0	0	0	0	0	0	0	76	111.7087379	74.75728155	0	0	0	0	0	0	0	0	0	61.08737864	0	0	0	0	0	0

AMP3CPR2ERY1FUS2TMP1	AMP	CPR	ERY	FUS	TMP	AMP+CPR	AMP+ERY	AMP+FUS	AMP+TMP	CPR+ERY	CPR+FUS	CPR+TMP	ERY+FUS	ERY+TMP	FUS+TMP	AMP+CPR+ERY	AMP+CPR+FUS	AMP+CPR+TMP	AMP+ERY+FUS	AMP+ERY+TMP	AMP+FUS+TMP	CPR+ERY+FUS	CPR+ERY+TMP	CPR+FUS+TMP	ERY+FUS+TMP	AMP+CPR+ERY+FUS	AMP+CPR+ERY+TMP	AMP+CPR+FUS+TMP	AMP+ERY+FUS+TMP	CPR+ERY+FUS+TMP	AMP+CPR+ERY+FUS+TMP
	96.36209814	109.8222638	95.26991384	85.70423774	103.9915597	99.06937394	92.97800338	57.78341794	89.59390863	86.47333957	81.98316183	110.121609	68.82363285	89.36170213	66.0101987	84.17935702	66.5820643	117.3434856	53.72250423	97.37732657	55.68950931	62.84377923	84.97661366	59.85032741	65.20133638	45.93908629	103.1302876	60.42724196	52.64382403	63.74181478	40.12267343
	86.9829222	103.7561455	101.2632979	95.94414894	100.4654255	70.89184061	80	48.72865275	103.3776091	99.98033432	85.50639135	109.4198623	67.48670213	96.21010638	62.96542553	58.14041746	59.65844402	84.85768501	42.9601518	89.10815939	42.04933586	68.14159292	89.59685349	61.84857424	44.88031915	38.82352941	78.48197343	47.5142315	37.60910816	63.10717797	58.25426945
	71.44022848	83.55704698	94.35907411	96.53763859	112.410037	75.35699714	82.5377397	56.75234598	114.1982864	87.73303505	79.08277405	113.3855332	63.74246256	109.297802	67.47714452	62.30110159	59.68992248	104.4063647	49.24520604	96.89922481	35.53651571	58.50111857	95.78672632	60.5891126	63.74246256	43.37005304	100.8159935	57.73153815	48.08241534	57.43847875	32.9253366
	0	0	97.72815534	100.5242718	110.776699	0	0	0	0	0	0	0	71.33980583	112.6407767	66.36893204	0	0	0	0	0	0	0	0	0	62.01941748	0	0	0	0	0	0

AMP3CPR2ERY1FUS2TMP2	AMP	CPR	ERY	FUS	TMP	AMP+CPR	AMP+ERY	AMP+FUS	AMP+TMP	CPR+ERY	CPR+FUS	CPR+TMP	ERY+FUS	ERY+TMP	FUS+TMP	AMP+CPR+ERY	AMP+CPR+FUS	AMP+CPR+TMP	AMP+ERY+FUS	AMP+ERY+TMP	AMP+FUS+TMP	CPR+ERY+FUS	CPR+ERY+TMP	CPR+FUS+TMP	ERY+FUS+TMP	AMP+CPR+ERY+FUS	AMP+CPR+ERY+TMP	AMP+CPR+FUS+TMP	AMP+ERY+FUS+TMP	CPR+ERY+FUS+TMP	AMP+CPR+ERY+FUS+TMP
	96.36209814	109.8222638	95.26991384	85.70423774	99.49006506	99.06937394	92.97800338	57.78341794	98.05414552	86.47333957	81.98316183	111.3189897	68.82363285	87.95498505	67.17074028	84.17935702	66.5820643	114.2978003	53.72250423	101.4382403	55.68950931	62.84377923	106.828812	64.34050514	69.70283102	45.93908629	113.9593909	53.65905245	45.19881557	74.21889616	52.30541455
	86.9829222	103.7561455	101.2632979	95.94414894	101.5292553	70.89184061	80	48.72865275	97.30550285	99.98033432	85.50639135	116.027532	67.48670213	94.6143617	62.43351064	58.14041746	59.65844402	85.46489564	42.9601518	93.9658444	47.81783681	68.14159292	104.7000983	73.23500492	52.32712766	38.82352941	83.64326376	48.42504744	37.60910816	64.99508358	39.62049336
	71.44022848	83.55704698	94.35907411	96.53763859	109.297802	75.35699714	82.5377397	56.75234598	112.8926969	87.73303505	79.08277405	119.0529456	63.74246256	109.920249	74.63528496	62.30110159	59.68992248	101.4687882	49.24520604	112.2399021	49.57160343	58.50111857	108.3146905	76.39821029	70.900603	43.37005304	94.94084047	62.95389637	39.77968176	56.54362416	48.91880865
	0	0	97.72815534	100.5242718	115.4368932	0	0	0	0	0	0	0	71.33980583	119.1650485	63.86407767	0	0	0	0	0	0	0	0	0	63.88349515	0	0	0	0	0	0

AMP3CPR2ERY1FUS2TMP3	AMP	CPR	ERY	FUS	TMP	AMP+CPR	AMP+ERY	AMP+FUS	AMP+TMP	CPR+ERY	CPR+FUS	CPR+TMP	ERY+FUS	ERY+TMP	FUS+TMP	AMP+CPR+ERY	AMP+CPR+FUS	AMP+CPR+TMP	AMP+ERY+FUS	AMP+ERY+TMP	AMP+FUS+TMP	CPR+ERY+FUS	CPR+ERY+TMP	CPR+FUS+TMP	ERY+FUS+TMP	AMP+CPR+ERY+FUS	AMP+CPR+ERY+TMP	AMP+CPR+FUS+TMP	AMP+ERY+FUS+TMP	CPR+ERY+FUS+TMP	AMP+CPR+ERY+FUS+TMP
	96.36209814	109.8222638	95.26991384	85.70423774	108.2117109	99.06937394	92.97800338	57.78341794	103.4686971	86.47333957	81.98316183	124.4901777	68.82363285	91.04976262	81.48408651	84.17935702	66.5820643	92.97800338	53.72250423	99.06937394	32.00084602	62.84377923	117.9045837	68.83068288	67.41691577	45.93908629	100.7614213	40.86294416	26.92470389	75.11693171	32.3392555
	86.9829222	103.7561455	101.2632979	95.94414894	100.4654255	70.89184061	80	48.72865275	89.10815939	99.98033432	85.50639135	123.2645034	67.48670213	94.88031915	78.65691489	58.14041746	59.65844402	90.62618596	42.9601518	88.50094877	35.4459203	68.14159292	113.5103245	77.26647001	60.57180851	38.82352941	75.4459203	43.0740038	37.30550285	75.69321534	30.89184061
	71.44022848	83.55704698	94.35907411	96.53763859	115.8334954	75.35699714	82.5377397	56.75234598	108.9759282	87.73303505	79.08277405	119.3512304	63.74246256	108.3641315	83.4662517	62.30110159	59.68992248	100.8159935	49.24520604	103.4271726	40.10607915	58.50111857	110.7009694	79.38105891	62.30305388	43.37005304	75.03059976	44.02284782	30.96695226	61.01789709	31.29334965
	0	0	97.72815534	100.5242718	108.2912621	0	0	0	0	0	0	0	71.33980583	111.7087379	97.72815534	0	0	0	0	0	0	0	0	0	58.27184466	0	0	0	0	0	0

AMP3CPR2ERY1FUS3TMP1	AMP	CPR	ERY	FUS	TMP	AMP+CPR	AMP+ERY	AMP+FUS	AMP+TMP	CPR+ERY	CPR+FUS	CPR+TMP	ERY+FUS	ERY+TMP	FUS+TMP	AMP+CPR+ERY	AMP+CPR+FUS	AMP+CPR+TMP	AMP+ERY+FUS	AMP+ERY+TMP	AMP+FUS+TMP	CPR+ERY+FUS	CPR+ERY+TMP	CPR+FUS+TMP	ERY+FUS+TMP	AMP+CPR+ERY+FUS	AMP+CPR+ERY+TMP	AMP+CPR+FUS+TMP	AMP+ERY+FUS+TMP	CPR+ERY+FUS+TMP	AMP+CPR+ERY+FUS+TMP
	96.36209814	109.8222638	95.26991384	98.64603482	103.9915597	99.06937394	92.97800338	82.82571912	89.59390863	86.47333957	104.7333957	110.121609	88.23632847	89.36170213	75.01318797	84.17935702	95.00846024	117.3434856	87.90186125	97.37732657	86.88663283	86.47333957	84.97661366	71.20673527	68.26094602	80.45685279	103.1302876	99.06937394	87.56345178	67.01590271	80.11844332
	86.9829222	103.7561455	101.2632979	101.7952128	100.4654255	70.89184061	80	76.66034156	103.3776091	99.98033432	103.7561455	109.4198623	89.82712766	96.21010638	69.6143617	58.14041746	67.55218216	84.85768501	79.08918406	89.10815939	76.05313093	89.28220256	89.59685349	76.06686332	58.17819149	68.76660342	78.48197343	73.0170778	76.05313093	69.14454277	62.99810247
	71.44022848	83.55704698	94.35907411	112.410037	112.410037	75.35699714	82.5377397	91.67686659	114.1982864	87.73303505	99.06785981	113.3855332	103.073332	109.297802	78.48667574	62.30110159	77.64177887	104.4063647	80.25295798	96.89922481	87.10730314	90.41759881	95.78672632	83.85533184	68.21630033	69.80824153	100.8159935	78.94736842	80.25295798	81.76733781	71.44022848
	0	0	97.72815534	108.2912621	110.776699	0	0	0	0	0	0	0	100.2135922	112.6407767	83.12621359	0	0	0	0	0	0	0	0	0	66.97087379	0	0	0	0	0	0

AMP3CPR2ERY1FUS3TMP2	AMP	CPR	ERY	FUS	TMP	AMP+CPR	AMP+ERY	AMP+FUS	AMP+TMP	CPR+ERY	CPR+FUS	CPR+TMP	ERY+FUS	ERY+TMP	FUS+TMP	AMP+CPR+ERY	AMP+CPR+FUS	AMP+CPR+TMP	AMP+ERY+FUS	AMP+ERY+TMP	AMP+FUS+TMP	CPR+ERY+FUS	CPR+ERY+TMP	CPR+FUS+TMP	ERY+FUS+TMP	AMP+CPR+ERY+FUS	AMP+CPR+ERY+TMP	AMP+CPR+FUS+TMP	AMP+ERY+FUS+TMP	CPR+ERY+FUS+TMP	AMP+CPR+ERY+FUS+TMP
	96.36209814	109.8222638	95.26991384	98.64603482	99.49006506	99.06937394	92.97800338	82.82571912	98.05414552	86.47333957	104.7333957	111.3189897	88.23632847	87.95498505	86.26692456	84.17935702	95.00846024	114.2978003	87.90186125	101.4382403	81.47208122	86.47333957	106.828812	85.27595884	83.172147	80.45685279	113.9593909	94.67005076	75.38071066	73.30215154	79.44162437
	86.9829222	103.7561455	101.2632979	101.7952128	101.5292553	70.89184061	80	76.66034156	97.30550285	99.98033432	103.7561455	116.027532	89.82712766	94.6143617	82.6462766	58.14041746	67.55218216	85.46489564	79.08918406	93.9658444	74.23149905	89.28220256	104.7000983	85.19174041	77.06117021	68.76660342	83.64326376	68.76660342	66.0341556	84.87708948	62.69449715
	71.44022848	83.55704698	94.35907411	112.410037	109.297802	75.35699714	82.5377397	91.67686659	112.8926969	87.73303505	99.06785981	119.0529456	103.073332	109.920249	95.2927446	62.30110159	77.64177887	101.4687882	80.25295798	112.2399021	81.55854753	90.41759881	108.3146905	92.50559284	80.66524023	69.80824153	94.94084047	78.29457364	71.76662587	88.03131991	70.7874337
	0	0	97.72815534	108.2912621	115.4368932	0	0	0	0	0	0	0	100.2135922	119.1650485	89.65048544	0	0	0	0	0	0	0	0	0	75.98058252	0	0	0	0	0	0

AMP3CPR2ERY1FUS3TMP3	AMP	CPR	ERY	FUS	TMP	AMP+CPR	AMP+ERY	AMP+FUS	AMP+TMP	CPR+ERY	CPR+FUS	CPR+TMP	ERY+FUS	ERY+TMP	FUS+TMP	AMP+CPR+ERY	AMP+CPR+FUS	AMP+CPR+TMP	AMP+ERY+FUS	AMP+ERY+TMP	AMP+FUS+TMP	CPR+ERY+FUS	CPR+ERY+TMP	CPR+FUS+TMP	ERY+FUS+TMP	AMP+CPR+ERY+FUS	AMP+CPR+ERY+TMP	AMP+CPR+FUS+TMP	AMP+ERY+FUS+TMP	CPR+ERY+FUS+TMP	AMP+CPR+ERY+FUS+TMP
	96.36209814	109.8222638	95.26991384	98.64603482	108.2117109	99.06937394	92.97800338	82.82571912	103.4686971	86.47333957	104.7333957	124.4901777	88.23632847	91.04976262	105.9609636	84.17935702	95.00846024	92.97800338	87.90186125	99.06937394	55.68950931	86.47333957	117.9045837	105.9307764	95.55125725	80.45685279	100.7614213	86.54822335	50.67681895	86.17399439	74.36548223
	86.9829222	103.7561455	101.2632979	101.7952128	100.4654255	70.89184061	80	76.66034156	89.10815939	99.98033432	103.7561455	123.2645034	89.82712766	94.88031915	105.2526596	58.14041746	67.55218216	90.62618596	79.08918406	88.50094877	56.9259962	89.28220256	113.5103245	102.8121927	97.00797872	68.76660342	75.4459203	64.62998102	58.74762808	94.00196657	51.15749526
	71.44022848	83.55704698	94.35907411	112.410037	115.8334954	75.35699714	82.5377397	91.67686659	108.9759282	87.73303505	99.06785981	119.3512304	103.073332	108.3641315	109.920249	62.30110159	77.64177887	100.8159935	80.25295798	103.4271726	73.07221542	90.41759881	110.7009694	111.2975391	106.808014	69.80824153	75.03059976	74.37780498	64.9122807	98.17300522	69.80824153
	0	0	97.72815534	108.2912621	108.2912621	0	0	0	0	0	0	0	100.2135922	111.7087379	111.0873786	0	0	0	0	0	0	0	0	0	105.184466	0	0	0	0	0	0

AMP3CPR2ERY2FUS1TMP1	AMP	CPR	ERY	FUS	TMP	AMP+CPR	AMP+ERY	AMP+FUS	AMP+TMP	CPR+ERY	CPR+FUS	CPR+TMP	ERY+FUS	ERY+TMP	FUS+TMP	AMP+CPR+ERY	AMP+CPR+FUS	AMP+CPR+TMP	AMP+ERY+FUS	AMP+ERY+TMP	AMP+FUS+TMP	CPR+ERY+FUS	CPR+ERY+TMP	CPR+FUS+TMP	ERY+FUS+TMP	AMP+CPR+ERY+FUS	AMP+CPR+ERY+TMP	AMP+CPR+FUS+TMP	AMP+ERY+FUS+TMP	CPR+ERY+FUS+TMP	AMP+CPR+ERY+FUS+TMP
	96.36209814	109.8222638	103.8353601	70.23034992	103.9915597	99.06937394	106.8527919	11.06175973	89.59390863	99.34518241	70.92609916	110.121609	69.71000935	113.414406	64.91999297	86.54822335	38.15566836	117.3434856	18.84517766	90.94754653	43.50676819	55.36014967	99.94387278	62.84377923	52.66604303	17.78764805	109.5600677	49.59813875	44.86040609	55.36014967	42.15313029
	86.9829222	103.7561455	102.1828909	82.6462766	100.4654255	70.89184061	76.35673624	16.66034156	103.3776091	106.5880039	72.546706	109.4198623	77.95476893	110.993117	54.38829787	59.35483871	39.08918406	84.85768501	27.28652751	92.75142315	27.55218216	62.47787611	99.03638151	84.18879056	72.86135693	24.25047438	88.19734345	50.66413662	23.64326376	70.34414946	60.37950664
	71.44022848	83.55704698	88.62788963	74.01283797	112.410037	75.35699714	70.13463892	19.54304366	114.1982864	97.87472036	56.54362416	113.3855332	82.06562267	107.7181208	71.21182649	60.34271726	27.37658099	104.4063647	16.27906977	98.85760914	31.94614443	55.94705444	105.033557	56.54362416	56.7114094	18.2374541	104.7327621	51.52998776	27.19298246	54.15734526	35.67931457
	0	0	0	81.26213592	110.776699	0	0	0	0	0	0	0	0	0	72.89320388	0	0	0	0	0	0	0	0	0	0	0	0	0	0	0	0

AMP3CPR2ERY2FUS1TMP2	AMP	CPR	ERY	FUS	TMP	AMP+CPR	AMP+ERY	AMP+FUS	AMP+TMP	CPR+ERY	CPR+FUS	CPR+TMP	ERY+FUS	ERY+TMP	FUS+TMP	AMP+CPR+ERY	AMP+CPR+FUS	AMP+CPR+TMP	AMP+ERY+FUS	AMP+ERY+TMP	AMP+FUS+TMP	CPR+ERY+FUS	CPR+ERY+TMP	CPR+FUS+TMP	ERY+FUS+TMP	AMP+CPR+ERY+FUS	AMP+CPR+ERY+TMP	AMP+CPR+FUS+TMP	AMP+ERY+FUS+TMP	CPR+ERY+FUS+TMP	AMP+CPR+ERY+FUS+TMP
	96.36209814	109.8222638	103.8353601	70.23034992	99.49006506	99.06937394	106.8527919	11.06175973	98.05414552	99.34518241	70.92609916	111.3189897	69.71000935	120.2993452	64.63864955	86.54822335	38.15566836	114.2978003	18.84517766	99.74619289	18.80287648	55.36014967	99.34518241	72.42282507	62.84377923	17.78764805	110.5752961	38.43062606	14.06514382	56.40785781	19.18358714
	86.9829222	103.7561455	102.1828909	82.6462766	101.5292553	70.89184061	76.35673624	16.66034156	97.30550285	106.5880039	72.546706	116.027532	77.95476893	119.1740413	55.25265957	59.35483871	39.08918406	85.46489564	27.28652751	91.5370019	20.26565465	62.47787611	105.3294002	86.3913471	76.32251721	24.25047438	93.9658444	41.25237192	21.4800759	72.23205506	17.87476281
	71.44022848	83.55704698	88.62788963	74.01283797	109.297802	75.35699714	70.13463892	19.54304366	112.8926969	97.87472036	56.54362416	119.0529456	82.06562267	112.7889635	60.94145108	60.34271726	27.37658099	101.4687882	16.27906977	108.6495308	23.78620971	55.94705444	108.9112603	63.10589113	63.87024609	18.2374541	101.4687882	29.00856793	17.58465932	52.06935123	23.78620971
	0	0	0	81.26213592	115.4368932	0	0	0	0	0	0	0	0	0	72.58252427	0	0	0	0	0	0	0	0	0	0	0	0	0	0	0	0

AMP3CPR2ERY2FUS1TMP3	AMP	CPR	ERY	FUS	TMP	AMP+CPR	AMP+ERY	AMP+FUS	AMP+TMP	CPR+ERY	CPR+FUS	CPR+TMP	ERY+FUS	ERY+TMP	FUS+TMP	AMP+CPR+ERY	AMP+CPR+FUS	AMP+CPR+TMP	AMP+ERY+FUS	AMP+ERY+TMP	AMP+FUS+TMP	CPR+ERY+FUS	CPR+ERY+TMP	CPR+FUS+TMP	ERY+FUS+TMP	AMP+CPR+ERY+FUS	AMP+CPR+ERY+TMP	AMP+CPR+FUS+TMP	AMP+ERY+FUS+TMP	CPR+ERY+FUS+TMP	AMP+CPR+ERY+FUS+TMP
	96.36209814	109.8222638	103.8353601	70.23034992	108.2117109	99.06937394	106.8527919	11.06175973	103.4686971	99.34518241	70.92609916	124.4901777	69.71000935	121.7960711	73.92298224	86.54822335	38.15566836	92.97800338	18.84517766	95.34686971	18.12605753	55.36014967	112.2170253	77.81103835	66.41721235	17.78764805	85.8714044	39.44585448	15.7571912	64.63985033	36.4001692
	86.9829222	103.7561455	102.1828909	82.6462766	100.4654255	70.89184061	76.35673624	16.66034156	89.10815939	106.5880039	72.546706	123.2645034	77.95476893	124.5231072	59.50797872	59.35483871	39.08918406	90.62618596	27.28652751	89.71537002	23.90891841	62.47787611	112.5663717	90.79646018	78.89872173	24.25047438	81.21442125	32.75142315	27.24857685	76.95181908	36.39468691
	71.44022848	83.55704698	88.62788963	74.01283797	115.8334954	75.35699714	70.13463892	19.54304366	108.9759282	97.87472036	56.54362416	119.3512304	82.06562267	112.7889635	68.41081502	60.34271726	27.37658099	100.8159935	16.27906977	104.0799674	22.48062016	55.94705444	105.3318419	64.00074571	80.87248322	18.2374541	83.51693186	46.96042432	15.95267238	57.73676361	28.02937576
	0	0	0	81.26213592	108.2912621	0	0	0	0	0	0	0	0	0	74.75728155	0	0	0	0	0	0	0	0	0	0	0	0	0	0	0	0

AMP3CPR2ERY2FUS2TMP1	AMP	CPR	ERY	FUS	TMP	AMP+CPR	AMP+ERY	AMP+FUS	AMP+TMP	CPR+ERY	CPR+FUS	CPR+TMP	ERY+FUS	ERY+TMP	FUS+TMP	AMP+CPR+ERY	AMP+CPR+FUS	AMP+CPR+TMP	AMP+ERY+FUS	AMP+ERY+TMP	AMP+FUS+TMP	CPR+ERY+FUS	CPR+ERY+TMP	CPR+FUS+TMP	ERY+FUS+TMP	AMP+CPR+ERY+FUS	AMP+CPR+ERY+TMP	AMP+CPR+FUS+TMP	AMP+ERY+FUS+TMP	CPR+ERY+FUS+TMP	AMP+CPR+ERY+FUS+TMP
	96.36209814	109.8222638	103.8353601	85.70423774	103.9915597	99.06937394	106.8527919	57.78341794	89.59390863	99.34518241	81.98316183	110.121609	76.59494855	113.414406	66.0101987	86.54822335	66.5820643	117.3434856	65.2284264	90.94754653	55.68950931	70.30869972	99.94387278	59.85032741	58.3536015	46.61590525	109.5600677	60.42724196	50.2749577	72.42282507	45.26226734
	86.9829222	103.7561455	102.1828909	95.94414894	100.4654255	70.89184061	76.35673624	48.72865275	103.3776091	106.5880039	85.50639135	109.4198623	89.91150442	110.993117	62.96542553	59.35483871	59.65844402	84.85768501	59.0512334	92.75142315	42.04933586	64.7394297	99.03638151	61.84857424	66.56833825	43.37760911	88.19734345	47.5142315	43.26375712	76.00786627	51.27134725
	71.44022848	83.55704698	88.62788963	96.53763859	112.410037	75.35699714	70.13463892	56.75234598	114.1982864	97.87472036	79.08277405	113.3855332	83.55704698	107.7181208	67.47714452	60.34271726	59.68992248	104.4063647	58.05793554	98.85760914	35.53651571	71.92393736	105.033557	60.5891126	74.01193139	59.0371277	104.7327621	57.73153815	47.28682171	56.84190902	42.39086087
	0	0	0	100.5242718	110.776699	0	0	0	0	0	0	0	0	0	66.36893204	0	0	0	0	0	0	0	0	0	0	0	0	0	0	0	0

AMP3CPR2ERY2FUS2TMP2	AMP	CPR	ERY	FUS	TMP	AMP+CPR	AMP+ERY	AMP+FUS	AMP+TMP	CPR+ERY	CPR+FUS	CPR+TMP	ERY+FUS	ERY+TMP	FUS+TMP	AMP+CPR+ERY	AMP+CPR+FUS	AMP+CPR+TMP	AMP+ERY+FUS	AMP+ERY+TMP	AMP+FUS+TMP	CPR+ERY+FUS	CPR+ERY+TMP	CPR+FUS+TMP	ERY+FUS+TMP	AMP+CPR+ERY+FUS	AMP+CPR+ERY+TMP	AMP+CPR+FUS+TMP	AMP+ERY+FUS+TMP	CPR+ERY+FUS+TMP	AMP+CPR+ERY+FUS+TMP
	96.36209814	109.8222638	103.8353601	85.70423774	99.49006506	99.06937394	106.8527919	57.78341794	98.05414552	99.34518241	81.98316183	111.3189897	76.59494855	120.2993452	67.17074028	86.54822335	66.5820643	114.2978003	65.2284264	99.74619289	55.68950931	70.30869972	99.34518241	64.34050514	64.02245089	46.61590525	110.5752961	53.65905245	46.27749577	75.71562208	48.24450085
	86.9829222	103.7561455	102.1828909	95.94414894	101.5292553	70.89184061	76.35673624	48.72865275	97.30550285	106.5880039	85.50639135	116.027532	89.91150442	119.1740413	62.43351064	59.35483871	59.65844402	85.46489564	59.0512334	91.5370019	47.81783681	64.7394297	105.3294002	73.23500492	71.03244838	43.37760911	93.9658444	48.42504744	42.16318786	76.32251721	42.46679317
	71.44022848	83.55704698	88.62788963	96.53763859	109.297802	75.35699714	70.13463892	56.75234598	112.8926969	97.87472036	79.08277405	119.0529456	83.55704698	112.7889635	74.63528496	60.34271726	59.68992248	101.4687882	58.05793554	108.6495308	49.57160343	71.92393736	108.9112603	76.39821029	82.96047726	59.0371277	101.4687882	62.95389637	46.96042432	60.71961223	51.52998776
	0	0	0	100.5242718	115.4368932	0	0	0	0	0	0	0	0	0	63.86407767	0	0	0	0	0	0	0	0	0	0	0	0	0	0	0	0

AMP3CPR2ERY2FUS2TMP3	AMP	CPR	ERY	FUS	TMP	AMP+CPR	AMP+ERY	AMP+FUS	AMP+TMP	CPR+ERY	CPR+FUS	CPR+TMP	ERY+FUS	ERY+TMP	FUS+TMP	AMP+CPR+ERY	AMP+CPR+FUS	AMP+CPR+TMP	AMP+ERY+FUS	AMP+ERY+TMP	AMP+FUS+TMP	CPR+ERY+FUS	CPR+ERY+TMP	CPR+FUS+TMP	ERY+FUS+TMP	AMP+CPR+ERY+FUS	AMP+CPR+ERY+TMP	AMP+CPR+FUS+TMP	AMP+ERY+FUS+TMP	CPR+ERY+FUS+TMP	AMP+CPR+ERY+FUS+TMP
	96.36209814	109.8222638	103.8353601	85.70423774	108.2117109	99.06937394	106.8527919	57.78341794	103.4686971	99.34518241	81.98316183	124.4901777	76.59494855	121.7960711	81.48408651	86.54822335	66.5820643	92.97800338	65.2284264	95.34686971	32.00084602	70.30869972	112.2170253	68.83068288	81.68381665	46.61590525	85.8714044	40.86294416	33.35448393	76.61365762	43.16835871
	86.9829222	103.7561455	102.1828909	95.94414894	100.4654255	70.89184061	76.35673624	48.72865275	89.10815939	106.5880039	85.50639135	123.2645034	89.91150442	124.5231072	78.65691489	59.35483871	59.65844402	90.62618596	59.0512334	89.71537002	35.4459203	64.7394297	112.5663717	77.26647001	94.3166175	43.37760911	81.21442125	43.0740038	30.01897533	75.69321534	36.66034156
	71.44022848	83.55704698	88.62788963	96.53763859	115.8334954	75.35699714	70.13463892	56.75234598	108.9759282	97.87472036	79.08277405	119.3512304	83.55704698	112.7889635	83.4662517	60.34271726	59.68992248	100.8159935	58.05793554	104.0799674	40.10607915	71.92393736	105.3318419	79.38105891	91.90902312	59.0371277	83.51693186	44.02284782	36.51570787	65.19388516	39.45328437
	0	0	0	100.5242718	108.2912621	0	0	0	0	0	0	0	0	0	97.72815534	0	0	0	0	0	0	0	0	0	0	0	0	0	0	0	0

AMP3CPR2ERY2FUS3TMP1	AMP	CPR	ERY	FUS	TMP	AMP+CPR	AMP+ERY	AMP+FUS	AMP+TMP	CPR+ERY	CPR+FUS	CPR+TMP	ERY+FUS	ERY+TMP	FUS+TMP	AMP+CPR+ERY	AMP+CPR+FUS	AMP+CPR+TMP	AMP+ERY+FUS	AMP+ERY+TMP	AMP+FUS+TMP	CPR+ERY+FUS	CPR+ERY+TMP	CPR+FUS+TMP	ERY+FUS+TMP	AMP+CPR+ERY+FUS	AMP+CPR+ERY+TMP	AMP+CPR+FUS+TMP	AMP+ERY+FUS+TMP	CPR+ERY+FUS+TMP	AMP+CPR+ERY+FUS+TMP
	96.36209814	109.8222638	103.8353601	98.64603482	103.9915597	99.06937394	106.8527919	82.82571912	89.59390863	99.34518241	104.7333957	110.121609	106.828812	113.414406	75.01318797	86.54822335	95.00846024	117.3434856	89.9323181	90.94754653	86.88663283	95.45369504	99.94387278	71.20673527	85.8746492	81.13367174	109.5600677	99.06937394	73.01184433	68.51262862	79.44162437
	86.9829222	103.7561455	102.1828909	101.7952128	100.4654255	70.89184061	76.35673624	76.66034156	103.3776091	106.5880039	103.7561455	109.4198623	105.6440511	110.993117	69.6143617	59.35483871	67.55218216	84.85768501	77.87476281	92.75142315	76.05313093	92.4287119	99.03638151	76.06686332	72.92035398	61.4800759	88.19734345	73.0170778	69.07020873	75.12291052	58.14041746
	71.44022848	83.55704698	88.62788963	112.410037	112.410037	75.35699714	70.13463892	91.67686659	114.1982864	97.87472036	99.06785981	113.3855332	102.945563	107.7181208	78.48667574	60.34271726	77.64177887	104.4063647	80.90575275	98.85760914	87.10730314	95.48844146	105.033557	83.85533184	77.59134974	67.8498572	104.7327621	78.94736842	74.70420237	87.73303505	66.87066503
	0	0	0	108.2912621	110.776699	0	0	0	0	0	0	0	0	0	83.12621359	0	0	0	0	0	0	0	0	0	0	0	0	0	0	0	0

AMP3CPR2ERY2FUS3TMP2	AMP	CPR	ERY	FUS	TMP	AMP+CPR	AMP+ERY	AMP+FUS	AMP+TMP	CPR+ERY	CPR+FUS	CPR+TMP	ERY+FUS	ERY+TMP	FUS+TMP	AMP+CPR+ERY	AMP+CPR+FUS	AMP+CPR+TMP	AMP+ERY+FUS	AMP+ERY+TMP	AMP+FUS+TMP	CPR+ERY+FUS	CPR+ERY+TMP	CPR+FUS+TMP	ERY+FUS+TMP	AMP+CPR+ERY+FUS	AMP+CPR+ERY+TMP	AMP+CPR+FUS+TMP	AMP+ERY+FUS+TMP	CPR+ERY+FUS+TMP	AMP+CPR+ERY+FUS+TMP
	96.36209814	109.8222638	103.8353601	98.64603482	99.49006506	99.06937394	106.8527919	82.82571912	98.05414552	99.34518241	104.7333957	111.3189897	106.828812	120.2993452	86.26692456	86.54822335	95.00846024	114.2978003	89.9323181	99.74619289	81.47208122	95.45369504	99.34518241	85.27595884	93.95696913	81.13367174	110.5752961	94.67005076	64.89001692	79.28905519	73.01184433
	86.9829222	103.7561455	102.1828909	101.7952128	101.5292553	70.89184061	76.35673624	76.66034156	97.30550285	106.5880039	103.7561455	116.027532	105.6440511	119.1740413	82.6462766	59.35483871	67.55218216	85.46489564	77.87476281	91.5370019	74.23149905	92.4287119	105.3294002	85.19174041	89.28220256	61.4800759	93.9658444	68.76660342	70.58823529	84.87708948	60.26565465
	71.44022848	83.55704698	88.62788963	112.410037	109.297802	75.35699714	70.13463892	91.67686659	112.8926969	97.87472036	99.06785981	119.0529456	102.945563	112.7889635	95.2927446	60.34271726	77.64177887	101.4687882	80.90575275	108.6495308	81.55854753	95.48844146	108.9112603	92.50559284	99.66442953	67.8498572	101.4687882	78.29457364	75.35699714	93.10216257	63.93308854
	0	0	0	108.2912621	115.4368932	0	0	0	0	0	0	0	0	0	89.65048544	0	0	0	0	0	0	0	0	0	0	0	0	0	0	0	0

AMP3CPR2ERY2FUS3TMP3	AMP	CPR	ERY	FUS	TMP	AMP+CPR	AMP+ERY	AMP+FUS	AMP+TMP	CPR+ERY	CPR+FUS	CPR+TMP	ERY+FUS	ERY+TMP	FUS+TMP	AMP+CPR+ERY	AMP+CPR+FUS	AMP+CPR+TMP	AMP+ERY+FUS	AMP+ERY+TMP	AMP+FUS+TMP	CPR+ERY+FUS	CPR+ERY+TMP	CPR+FUS+TMP	ERY+FUS+TMP	AMP+CPR+ERY+FUS	AMP+CPR+ERY+TMP	AMP+CPR+FUS+TMP	AMP+ERY+FUS+TMP	CPR+ERY+FUS+TMP	AMP+CPR+ERY+FUS+TMP
	96.36209814	109.8222638	103.8353601	98.64603482	108.2117109	99.06937394	106.8527919	82.82571912	103.4686971	99.34518241	104.7333957	124.4901777	106.828812	121.7960711	105.9609636	86.54822335	95.00846024	92.97800338	89.9323181	95.34686971	55.68950931	95.45369504	112.2170253	105.9307764	119.1019645	81.13367174	85.8714044	86.54822335	46.21404399	86.47333957	68.61252115
	86.9829222	103.7561455	102.1828909	101.7952128	100.4654255	70.89184061	76.35673624	76.66034156	89.10815939	106.5880039	103.7561455	123.2645034	105.6440511	124.5231072	105.2526596	59.35483871	67.55218216	90.62618596	77.87476281	89.71537002	56.9259962	92.4287119	112.5663717	102.8121927	109.4198623	61.4800759	81.21442125	64.62998102	52.67552182	89.28220256	56.31878558
	71.44022848	83.55704698	88.62788963	112.410037	115.8334954	75.35699714	70.13463892	91.67686659	108.9759282	97.87472036	99.06785981	119.3512304	102.945563	112.7889635	109.920249	60.34271726	77.64177887	100.8159935	80.90575275	104.0799674	73.07221542	95.48844146	105.3318419	111.2975391	111.8941089	67.8498572	83.51693186	74.37780498	69.15544676	97.27815063	65.23867809
	0	0	0	108.2912621	108.2912621	0	0	0	0	0	0	0	0	0	111.0873786	0	0	0	0	0	0	0	0	0	0	0	0	0	0	0	0

AMP3CPR2ERY3FUS1TMP1	AMP	CPR	ERY	FUS	TMP	AMP+CPR	AMP+ERY	AMP+FUS	AMP+TMP	CPR+ERY	CPR+FUS	CPR+TMP	ERY+FUS	ERY+TMP	FUS+TMP	AMP+CPR+ERY	AMP+CPR+FUS	AMP+CPR+TMP	AMP+ERY+FUS	AMP+ERY+TMP	AMP+FUS+TMP	CPR+ERY+FUS	CPR+ERY+TMP	CPR+FUS+TMP	ERY+FUS+TMP	AMP+CPR+ERY+FUS	AMP+CPR+ERY+TMP	AMP+CPR+FUS+TMP	AMP+ERY+FUS+TMP	CPR+ERY+FUS+TMP	AMP+CPR+ERY+FUS+TMP
	96.36209814	109.8222638	95.34686971	70.23034992	103.9915597	99.06937394	109.5600677	11.06175973	89.59390863	95.75304022	70.92609916	110.121609	80.79526227	114.6362098	64.91999297	95.34686971	38.15566836	117.3434856	21.89086294	89.9323181	43.50676819	60.74836296	104.4340505	62.84377923	60.08883249	32.3392555	110.5752961	49.59813875	58.05837563	57.75491113	48.9213198
	86.9829222	103.7561455	99.12713472	82.6462766	100.4654255	70.89184061	84.25047438	16.66034156	103.3776091	102.4975418	72.546706	109.4198623	90.32258065	114.914611	54.38829787	67.55218216	39.08918406	84.85768501	23.83301708	98.82352941	27.55218216	67.82694199	104.0707965	84.18879056	56.31878558	39.31688805	81.82163188	50.66413662	41.51802657	71.91740413	50.056926
	71.44022848	83.55704698	108.9759282	74.01283797	112.410037	75.35699714	68.50265198	19.54304366	114.1982864	95.78672632	56.54362416	113.3855332	93.63525092	120.3998368	71.21182649	67.52345981	27.37658099	104.4063647	32.27254182	100.4895961	31.94614443	56.41312453	110.9992543	56.54362416	62.95389637	50.55079559	97.55201958	51.52998776	50.36719706	57.14019389	46.63402693
	0	0	0	81.26213592	110.776699	0	0	0	0	0	0	0	0	0	72.89320388	0	0	0	0	0	0	0	0	0	0	0	0	0	0	0	0

AMP3CPR2ERY3FUS1TMP2	AMP	CPR	ERY	FUS	TMP	AMP+CPR	AMP+ERY	AMP+FUS	AMP+TMP	CPR+ERY	CPR+FUS	CPR+TMP	ERY+FUS	ERY+TMP	FUS+TMP	AMP+CPR+ERY	AMP+CPR+FUS	AMP+CPR+TMP	AMP+ERY+FUS	AMP+ERY+TMP	AMP+FUS+TMP	CPR+ERY+FUS	CPR+ERY+TMP	CPR+FUS+TMP	ERY+FUS+TMP	AMP+CPR+ERY+FUS	AMP+CPR+ERY+TMP	AMP+CPR+FUS+TMP	AMP+ERY+FUS+TMP	CPR+ERY+FUS+TMP	AMP+CPR+ERY+FUS+TMP
	96.36209814	109.8222638	95.34686971	70.23034992	99.49006506	99.06937394	109.5600677	11.06175973	98.05414552	95.75304022	70.92609916	111.3189897	80.79526227	114.9746193	64.63864955	95.34686971	38.15566836	114.2978003	21.89086294	99.06937394	18.80287648	60.74836296	115.8091674	72.42282507	72.2715736	32.3392555	113.6209814	38.43062606	17.78764805	66.43592142	26.24788494
	86.9829222	103.7561455	99.12713472	82.6462766	101.5292553	70.89184061	84.25047438	16.66034156	97.30550285	102.4975418	72.546706	116.027532	90.32258065	115.2182163	55.25265957	67.55218216	39.08918406	85.46489564	23.83301708	102.1631879	20.26565465	67.82694199	107.8466077	86.3913471	65.73055028	39.31688805	85.16129032	41.25237192	25.12333966	79.15437561	38.21631879
	71.44022848	83.55704698	108.9759282	74.01283797	109.297802	75.35699714	68.50265198	19.54304366	112.8926969	95.78672632	56.54362416	119.0529456	93.63525092	125.2957976	60.94145108	67.52345981	27.37658099	101.4687882	32.27254182	110.2815177	23.78620971	56.41312453	117.2632364	63.10589113	80.57935537	50.55079559	109.9551204	29.00856793	21.82782538	57.73676361	29.66136271
	0	0	0	81.26213592	115.4368932	0	0	0	0	0	0	0	0	0	72.58252427	0	0	0	0	0	0	0	0	0	0	0	0	0	0	0	0

AMP3CPR2ERY3FUS1TMP3	AMP	CPR	ERY	FUS	TMP	AMP+CPR	AMP+ERY	AMP+FUS	AMP+TMP	CPR+ERY	CPR+FUS	CPR+TMP	ERY+FUS	ERY+TMP	FUS+TMP	AMP+CPR+ERY	AMP+CPR+FUS	AMP+CPR+TMP	AMP+ERY+FUS	AMP+ERY+TMP	AMP+FUS+TMP	CPR+ERY+FUS	CPR+ERY+TMP	CPR+FUS+TMP	ERY+FUS+TMP	AMP+CPR+ERY+FUS	AMP+CPR+ERY+TMP	AMP+CPR+FUS+TMP	AMP+ERY+FUS+TMP	CPR+ERY+FUS+TMP	AMP+CPR+ERY+FUS+TMP
	96.36209814	109.8222638	95.34686971	70.23034992	108.2117109	99.06937394	109.5600677	11.06175973	103.4686971	95.75304022	70.92609916	124.4901777	80.79526227	115.6514382	73.92298224	95.34686971	38.15566836	92.97800338	21.89086294	94.67005076	18.12605753	60.74836296	116.707203	77.81103835	83.84094755	32.3392555	103.4686971	39.44585448	20.83333333	72.42282507	38.09221658
	86.9829222	103.7561455	99.12713472	82.6462766	100.4654255	70.89184061	84.25047438	16.66034156	89.10815939	102.4975418	72.546706	123.2645034	90.32258065	107.0208729	59.50797872	67.55218216	39.08918406	90.62618596	23.83301708	93.35863378	23.90891841	67.82694199	110.6784661	90.79646018	81.82163188	39.31688805	87.89373814	32.75142315	21.78368121	72.546706	47.32447818
	71.44022848	83.55704698	108.9759282	74.01283797	115.8334954	75.35699714	68.50265198	19.54304366	108.9759282	95.78672632	56.54362416	119.3512304	93.63525092	117.1358629	68.41081502	67.52345981	27.37658099	100.8159935	32.27254182	104.4063647	22.48062016	56.41312453	116.3683818	64.00074571	90.04487964	50.55079559	94.2880457	46.96042432	23.78620971	58.79940343	36.51570787
	0	0	0	81.26213592	108.2912621	0	0	0	0	0	0	0	0	0	74.75728155	0	0	0	0	0	0	0	0	0	0	0	0	0	0	0	0

AMP3CPR2ERY3FUS2TMP1	AMP	CPR	ERY	FUS	TMP	AMP+CPR	AMP+ERY	AMP+FUS	AMP+TMP	CPR+ERY	CPR+FUS	CPR+TMP	ERY+FUS	ERY+TMP	FUS+TMP	AMP+CPR+ERY	AMP+CPR+FUS	AMP+CPR+TMP	AMP+ERY+FUS	AMP+ERY+TMP	AMP+FUS+TMP	CPR+ERY+FUS	CPR+ERY+TMP	CPR+FUS+TMP	ERY+FUS+TMP	AMP+CPR+ERY+FUS	AMP+CPR+ERY+TMP	AMP+CPR+FUS+TMP	AMP+ERY+FUS+TMP	CPR+ERY+FUS+TMP	AMP+CPR+ERY+FUS+TMP
	96.36209814	109.8222638	95.34686971	85.70423774	103.9915597	99.06937394	109.5600677	57.78341794	89.59390863	95.75304022	81.98316183	110.121609	84.17935702	114.6362098	66.0101987	95.34686971	66.5820643	117.3434856	51.69204738	89.9323181	55.68950931	72.70346118	104.4340505	59.85032741	69.2893401	64.89001692	110.5752961	60.42724196	48.58291032	57.15622077	73.2680203
	86.9829222	103.7561455	99.12713472	95.94414894	100.4654255	70.89184061	84.25047438	48.72865275	103.3776091	102.4975418	85.50639135	109.4198623	93.05502846	114.914611	62.96542553	67.55218216	59.65844402	84.85768501	55.10436433	98.82352941	42.04933586	69.45919371	104.0707965	61.84857424	74.23149905	45.69259962	81.82163188	47.5142315	37.5711575	61.21927237	45.38899431
	71.44022848	83.55704698	108.9759282	96.53763859	112.410037	75.35699714	68.50265198	56.75234598	114.1982864	95.78672632	79.08277405	113.3855332	103.75357	120.3998368	67.47714452	67.52345981	59.68992248	104.4063647	53.81476948	100.4895961	35.53651571	79.97762864	110.9992543	60.5891126	78.29457364	59.68992248	97.55201958	57.73153815	32.9253366	54.3251305	53.1619747
	0	0	0	100.5242718	110.776699	0	0	0	0	0	0	0	0	0	66.36893204	0	0	0	0	0	0	0	0	0	0	0	0	0	0	0	0

AMP3CPR2ERY3FUS2TMP2	AMP	CPR	ERY	FUS	TMP	AMP+CPR	AMP+ERY	AMP+FUS	AMP+TMP	CPR+ERY	CPR+FUS	CPR+TMP	ERY+FUS	ERY+TMP	FUS+TMP	AMP+CPR+ERY	AMP+CPR+FUS	AMP+CPR+TMP	AMP+ERY+FUS	AMP+ERY+TMP	AMP+FUS+TMP	CPR+ERY+FUS	CPR+ERY+TMP	CPR+FUS+TMP	ERY+FUS+TMP	AMP+CPR+ERY+FUS	AMP+CPR+ERY+TMP	AMP+CPR+FUS+TMP	AMP+ERY+FUS+TMP	CPR+ERY+FUS+TMP	AMP+CPR+ERY+FUS+TMP
	96.36209814	109.8222638	95.34686971	85.70423774	99.49006506	99.06937394	109.5600677	57.78341794	98.05414552	95.75304022	81.98316183	111.3189897	84.17935702	114.9746193	67.17074028	95.34686971	66.5820643	114.2978003	51.69204738	99.06937394	55.68950931	72.70346118	115.8091674	64.34050514	85.8714044	64.89001692	113.6209814	53.65905245	44.24703892	69.42937325	70.30456853
	86.9829222	103.7561455	99.12713472	95.94414894	101.5292553	70.89184061	84.25047438	48.72865275	97.30550285	102.4975418	85.50639135	116.027532	93.05502846	115.2182163	62.43351064	67.55218216	59.65844402	85.46489564	55.10436433	102.1631879	47.81783681	69.45919371	107.8466077	73.23500492	88.19734345	45.69259962	85.16129032	48.42504744	44.17457306	70.97345133	55.71157495
	71.44022848	83.55704698	108.9759282	96.53763859	109.297802	75.35699714	68.50265198	56.75234598	112.8926969	95.78672632	79.08277405	119.0529456	103.75357	125.2957976	74.63528496	67.52345981	59.68992248	101.4687882	53.81476948	110.2815177	49.57160343	79.97762864	117.2632364	76.39821029	85.14891881	59.68992248	109.9551204	62.95389637	59.68992248	61.78225205	68.50265198
	0	0	0	100.5242718	115.4368932	0	0	0	0	0	0	0	0	0	63.86407767	0	0	0	0	0	0	0	0	0	0	0	0	0	0	0	0

AMP3CPR2ERY3FUS2TMP3	AMP	CPR	ERY	FUS	TMP	AMP+CPR	AMP+ERY	AMP+FUS	AMP+TMP	CPR+ERY	CPR+FUS	CPR+TMP	ERY+FUS	ERY+TMP	FUS+TMP	AMP+CPR+ERY	AMP+CPR+FUS	AMP+CPR+TMP	AMP+ERY+FUS	AMP+ERY+TMP	AMP+FUS+TMP	CPR+ERY+FUS	CPR+ERY+TMP	CPR+FUS+TMP	ERY+FUS+TMP	AMP+CPR+ERY+FUS	AMP+CPR+ERY+TMP	AMP+CPR+FUS+TMP	AMP+ERY+FUS+TMP	CPR+ERY+FUS+TMP	AMP+CPR+ERY+FUS+TMP
	96.36209814	109.8222638	95.34686971	85.70423774	108.2117109	99.06937394	109.5600677	57.78341794	103.4686971	95.75304022	81.98316183	124.4901777	84.17935702	115.6514382	81.48408651	95.34686971	66.5820643	92.97800338	51.69204738	94.67005076	32.00084602	72.70346118	116.707203	68.83068288	98.39255499	64.89001692	103.4686971	40.86294416	29.6319797	67.63330215	48.30795262
	86.9829222	103.7561455	99.12713472	95.94414894	100.4654255	70.89184061	84.25047438	48.72865275	89.10815939	102.4975418	85.50639135	123.2645034	93.05502846	107.0208729	78.65691489	67.55218216	59.65844402	90.62618596	55.10436433	93.35863378	35.4459203	69.45919371	110.6784661	77.26647001	93.9658444	45.69259962	87.89373814	43.0740038	39.69639469	68.82989184	46.67931689
	71.44022848	83.55704698	108.9759282	96.53763859	115.8334954	75.35699714	68.50265198	56.75234598	108.9759282	95.78672632	79.08277405	119.3512304	103.75357	117.1358629	83.4662517	67.52345981	59.68992248	100.8159935	53.81476948	104.4063647	40.10607915	79.97762864	116.3683818	79.38105891	92.65605875	59.68992248	94.2880457	44.02284782	35.8629131	77.59134974	45.65483476
	0	0	0	100.5242718	108.2912621	0	0	0	0	0	0	0	0	0	97.72815534	0	0	0	0	0	0	0	0	0	0	0	0	0	0	0	0

AMP3CPR2ERY3FUS3TMP1	AMP	CPR	ERY	FUS	TMP	AMP+CPR	AMP+ERY	AMP+FUS	AMP+TMP	CPR+ERY	CPR+FUS	CPR+TMP	ERY+FUS	ERY+TMP	FUS+TMP	AMP+CPR+ERY	AMP+CPR+FUS	AMP+CPR+TMP	AMP+ERY+FUS	AMP+ERY+TMP	AMP+FUS+TMP	CPR+ERY+FUS	CPR+ERY+TMP	CPR+FUS+TMP	ERY+FUS+TMP	AMP+CPR+ERY+FUS	AMP+CPR+ERY+TMP	AMP+CPR+FUS+TMP	AMP+ERY+FUS+TMP	CPR+ERY+FUS+TMP	AMP+CPR+ERY+FUS+TMP
	96.36209814	109.8222638	95.34686971	98.64603482	103.9915597	99.06937394	109.5600677	82.82571912	89.59390863	95.75304022	104.7333957	110.121609	102.1150592	114.6362098	75.01318797	95.34686971	95.00846024	117.3434856	85.19458545	89.9323181	86.88663283	104.1347053	104.4340505	71.20673527	79.44162437	90.60913706	110.5752961	99.06937394	82.48730964	68.21328344	92.30118443
	86.9829222	103.7561455	99.12713472	101.7952128	100.4654255	70.89184061	84.25047438	76.66034156	103.3776091	102.4975418	103.7561455	109.4198623	107.9316888	114.914611	69.6143617	67.55218216	67.55218216	84.85768501	82.73244782	98.82352941	76.05313093	101.553589	104.0707965	76.06686332	84.25047438	70.58823529	81.82163188	73.0170778	83.64326376	73.86430678	69.67741935
	71.44022848	83.55704698	108.9759282	112.410037	112.410037	75.35699714	68.50265198	91.67686659	114.1982864	95.78672632	99.06785981	113.3855332	115.1774786	120.3998368	78.48667574	67.52345981	77.64177887	104.4063647	82.86413709	100.4895961	87.10730314	105.3318419	110.9992543	83.85533184	94.94084047	78.29457364	97.55201958	78.94736842	83.84332925	86.5398956	81.23215014
	0	0	0	108.2912621	110.776699	0	0	0	0	0	0	0	0	0	83.12621359	0	0	0	0	0	0	0	0	0	0	0	0	0	0	0	0

AMP3CPR2ERY3FUS3TMP2	AMP	CPR	ERY	FUS	TMP	AMP+CPR	AMP+ERY	AMP+FUS	AMP+TMP	CPR+ERY	CPR+FUS	CPR+TMP	ERY+FUS	ERY+TMP	FUS+TMP	AMP+CPR+ERY	AMP+CPR+FUS	AMP+CPR+TMP	AMP+ERY+FUS	AMP+ERY+TMP	AMP+FUS+TMP	CPR+ERY+FUS	CPR+ERY+TMP	CPR+FUS+TMP	ERY+FUS+TMP	AMP+CPR+ERY+FUS	AMP+CPR+ERY+TMP	AMP+CPR+FUS+TMP	AMP+ERY+FUS+TMP	CPR+ERY+FUS+TMP	AMP+CPR+ERY+FUS+TMP
	96.36209814	109.8222638	95.34686971	98.64603482	99.49006506	99.06937394	109.5600677	82.82571912	98.05414552	95.75304022	104.7333957	111.3189897	102.1150592	114.9746193	86.26692456	95.34686971	95.00846024	114.2978003	85.19458545	99.06937394	81.47208122	104.1347053	115.8091674	85.27595884	106.1759729	90.60913706	113.6209814	94.67005076	66.92047377	81.08512629	102.7918782
	86.9829222	103.7561455	99.12713472	101.7952128	101.5292553	70.89184061	84.25047438	76.66034156	97.30550285	102.4975418	103.7561455	116.027532	107.9316888	115.2182163	82.6462766	67.55218216	67.55218216	85.46489564	82.73244782	102.1631879	74.23149905	101.553589	107.8466077	85.19174041	101.8595825	70.58823529	85.16129032	68.76660342	69.07020873	85.50639135	72.40986717
	71.44022848	83.55704698	108.9759282	112.410037	109.297802	75.35699714	68.50265198	91.67686659	112.8926969	95.78672632	99.06785981	119.0529456	115.1774786	125.2957976	95.2927446	67.52345981	77.64177887	101.4687882	82.86413709	110.2815177	81.55854753	105.3318419	117.2632364	92.50559284	106.6911465	78.29457364	109.9551204	78.29457364	76.33618931	96.68158091	82.86413709
	0	0	0	108.2912621	115.4368932	0	0	0	0	0	0	0	0	0	89.65048544	0	0	0	0	0	0	0	0	0	0	0	0	0	0	0	0

AMP3CPR2ERY3FUS3TMP3	AMP	CPR	ERY	FUS	TMP	AMP+CPR	AMP+ERY	AMP+FUS	AMP+TMP	CPR+ERY	CPR+FUS	CPR+TMP	ERY+FUS	ERY+TMP	FUS+TMP	AMP+CPR+ERY	AMP+CPR+FUS	AMP+CPR+TMP	AMP+ERY+FUS	AMP+ERY+TMP	AMP+FUS+TMP	CPR+ERY+FUS	CPR+ERY+TMP	CPR+FUS+TMP	ERY+FUS+TMP	AMP+CPR+ERY+FUS	AMP+CPR+ERY+TMP	AMP+CPR+FUS+TMP	AMP+ERY+FUS+TMP	CPR+ERY+FUS+TMP	AMP+CPR+ERY+FUS+TMP
	96.36209814	109.8222638	95.34686971	98.64603482	108.2117109	99.06937394	109.5600677	82.82571912	103.4686971	95.75304022	104.7333957	124.4901777	102.1150592	115.6514382	105.9609636	95.34686971	95.00846024	92.97800338	85.19458545	94.67005076	55.68950931	104.1347053	116.707203	105.9307764	114.2978003	90.60913706	103.4686971	86.54822335	67.19543147	108.9242283	93.65482234
	86.9829222	103.7561455	99.12713472	101.7952128	100.4654255	70.89184061	84.25047438	76.66034156	89.10815939	102.4975418	103.7561455	123.2645034	107.9316888	107.0208729	105.2526596	67.55218216	67.55218216	90.62618596	82.73244782	93.35863378	56.9259962	101.553589	110.6784661	102.8121927	114.3074004	70.58823529	87.89373814	64.62998102	70.89184061	110.3638151	70.58823529
	71.44022848	83.55704698	108.9759282	112.410037	115.8334954	75.35699714	68.50265198	91.67686659	108.9759282	95.78672632	99.06785981	119.3512304	115.1774786	117.1358629	109.920249	67.52345981	77.64177887	100.8159935	82.86413709	104.4063647	73.07221542	105.3318419	116.3683818	111.2975391	128.5597715	78.29457364	94.2880457	74.37780498	84.16972664	114.2803878	86.78090575
	0	0	0	108.2912621	108.2912621	0	0	0	0	0	0	0	0	0	111.0873786	0	0	0	0	0	0	0	0	0	0	0	0	0	0	0	0

AMP3CPR3ERY1FUS1TMP1	AMP	CPR	ERY	FUS	TMP	AMP+CPR	AMP+ERY	AMP+FUS	AMP+TMP	CPR+ERY	CPR+FUS	CPR+TMP	ERY+FUS	ERY+TMP	FUS+TMP	AMP+CPR+ERY	AMP+CPR+FUS	AMP+CPR+TMP	AMP+ERY+FUS	AMP+ERY+TMP	AMP+FUS+TMP	CPR+ERY+FUS	CPR+ERY+TMP	CPR+FUS+TMP	ERY+FUS+TMP	AMP+CPR+ERY+FUS	AMP+CPR+ERY+TMP	AMP+CPR+FUS+TMP	AMP+ERY+FUS+TMP	CPR+ERY+FUS+TMP	AMP+CPR+ERY+FUS+TMP
	96.36209814	94.67005076	95.26991384	70.23034992	103.9915597	105.8375635	92.97800338	11.06175973	89.59390863	104.4839255	83.43908629	112.605753	60.3833304	89.36170213	64.91999297	87.56345178	58.79864636	113.6209814	14.78426396	97.37732657	43.50676819	48.9213198	84.85617597	69.90270728	55.91700369	35.10998308	107.1912014	59.75042301	44.18358714	68.2106599	51.6285956
	86.9829222	95.18026565	101.2632979	82.6462766	100.4654255	85.76850095	80	16.66034156	103.3776091	88.19734345	54.00379507	109.4497154	63.49734043	96.21010638	54.38829787	74.83870968	45.38899431	101.8595825	22.4288425	89.10815939	27.55218216	50.66413662	94.57305503	63.41555977	55.98404255	33.85199241	84.85768501	59.0512334	29.67741935	51.57495256	59.77229602
	71.44022848	104.0799674	94.35907411	74.01283797	112.410037	84.49612403	82.5377397	19.54304366	114.1982864	92.98245614	67.19706242	125.2957976	74.94650846	109.297802	71.21182649	66.21787026	47.93961648	113.2190942	19.86944104	96.89922481	31.94614443	50.04079967	106.3647491	71.58302734	72.45672048	29.66136271	99.51040392	60.99551204	35.02651979	53.95756834	49.3880049
	0	0	97.72815534	81.26213592	110.776699	0	0	0	0	0	0	0	76	112.6407767	72.89320388	0	0	0	0	0	0	0	0	0	71.33980583	0	0	0	0	0	0

AMP3CPR3ERY1FUS1TMP2	AMP	CPR	ERY	FUS	TMP	AMP+CPR	AMP+ERY	AMP+FUS	AMP+TMP	CPR+ERY	CPR+FUS	CPR+TMP	ERY+FUS	ERY+TMP	FUS+TMP	AMP+CPR+ERY	AMP+CPR+FUS	AMP+CPR+TMP	AMP+ERY+FUS	AMP+ERY+TMP	AMP+FUS+TMP	CPR+ERY+FUS	CPR+ERY+TMP	CPR+FUS+TMP	ERY+FUS+TMP	AMP+CPR+ERY+FUS	AMP+CPR+ERY+TMP	AMP+CPR+FUS+TMP	AMP+ERY+FUS+TMP	CPR+ERY+FUS+TMP	AMP+CPR+ERY+FUS+TMP
	96.36209814	94.67005076	95.26991384	70.23034992	99.49006506	105.8375635	92.97800338	11.06175973	98.05414552	104.4839255	83.43908629	113.9593909	60.3833304	87.95498505	64.63864955	87.56345178	58.79864636	102.4534687	14.78426396	101.4382403	18.80287648	48.9213198	104.822335	83.10067682	58.44909443	35.10998308	109.5600677	59.13705584	2.897631134	51.96700508	52.9822335
	86.9829222	95.18026565	101.2632979	82.6462766	101.5292553	85.76850095	80	16.66034156	97.30550285	88.19734345	54.00379507	112.7893738	63.49734043	94.6143617	55.25265957	74.83870968	45.38899431	108.2352941	22.4288425	93.9658444	20.26565465	50.66413662	99.43074004	71.30929791	53.85638298	33.85199241	86.37571157	56.31878558	11.76470588	44.85768501	46.71726755
	71.44022848	104.0799674	94.35907411	74.01283797	109.297802	84.49612403	82.5377397	19.54304366	112.8926969	92.98245614	67.19706242	127.5805794	74.94650846	109.920249	60.94145108	66.21787026	47.93961648	113.2190942	19.86944104	112.2399021	23.78620971	50.04079967	118.7678499	67.66625867	69.03326201	29.66136271	98.53121175	60.66911465	17.25826193	46.63402693	33.72093023
	0	0	97.72815534	81.26213592	115.4368932	0	0	0	0	0	0	0	76	119.1650485	72.58252427	0	0	0	0	0	0	0	0	0	65.74757282	0	0	0	0	0	0

AMP3CPR3ERY1FUS1TMP3	AMP	CPR	ERY	FUS	TMP	AMP+CPR	AMP+ERY	AMP+FUS	AMP+TMP	CPR+ERY	CPR+FUS	CPR+TMP	ERY+FUS	ERY+TMP	FUS+TMP	AMP+CPR+ERY	AMP+CPR+FUS	AMP+CPR+TMP	AMP+ERY+FUS	AMP+ERY+TMP	AMP+FUS+TMP	CPR+ERY+FUS	CPR+ERY+TMP	CPR+FUS+TMP	ERY+FUS+TMP	AMP+CPR+ERY+FUS	AMP+CPR+ERY+TMP	AMP+CPR+FUS+TMP	AMP+ERY+FUS+TMP	CPR+ERY+FUS+TMP	AMP+CPR+ERY+FUS+TMP
	96.36209814	94.67005076	95.26991384	70.23034992	108.2117109	105.8375635	92.97800338	11.06175973	103.4686971	104.4839255	83.43908629	121.0659898	60.3833304	91.04976262	73.92298224	87.56345178	58.79864636	100.0846024	14.78426396	99.06937394	18.12605753	48.9213198	104.822335	75.31725888	67.4520837	35.10998308	89.59390863	74.36548223	1.924703892	62.45769882	49.59813875
	86.9829222	95.18026565	101.2632979	82.6462766	100.4654255	85.76850095	80	16.66034156	89.10815939	88.19734345	54.00379507	106.7172676	63.49734043	94.88031915	59.50797872	74.83870968	45.38899431	100.6451613	22.4288425	88.50094877	23.90891841	50.66413662	99.12713472	67.66603416	51.99468085	33.85199241	80.60721063	73.0170778	28.15939279	55.52182163	39.62049336
	71.44022848	104.0799674	94.35907411	74.01283797	115.8334954	84.49612403	82.5377397	19.54304366	108.9759282	92.98245614	67.19706242	123.0110159	74.94650846	108.3641315	68.41081502	66.21787026	47.93961648	92.32966136	19.86944104	103.4271726	22.48062016	50.04079967	108.3231334	70.7874337	70.278156	29.66136271	96.89922481	78.94736842	16.60546716	51.67278662	53.48837209
	0	0	97.72815534	81.26213592	108.2912621	0	0	0	0	0	0	0	76	111.7087379	74.75728155	0	0	0	0	0	0	0	0	0	61.08737864	0	0	0	0	0	0

AMP3CPR3ERY1FUS2TMP1	AMP	CPR	ERY	FUS	TMP	AMP+CPR	AMP+ERY	AMP+FUS	AMP+TMP	CPR+ERY	CPR+FUS	CPR+TMP	ERY+FUS	ERY+TMP	FUS+TMP	AMP+CPR+ERY	AMP+CPR+FUS	AMP+CPR+TMP	AMP+ERY+FUS	AMP+ERY+TMP	AMP+FUS+TMP	CPR+ERY+FUS	CPR+ERY+TMP	CPR+FUS+TMP	ERY+FUS+TMP	AMP+CPR+ERY+FUS	AMP+CPR+ERY+TMP	AMP+CPR+FUS+TMP	AMP+ERY+FUS+TMP	CPR+ERY+FUS+TMP	AMP+CPR+ERY+FUS+TMP
	96.36209814	94.67005076	95.26991384	85.70423774	103.9915597	105.8375635	92.97800338	57.78341794	89.59390863	104.4839255	80.79526227	112.605753	68.82363285	89.36170213	66.0101987	87.56345178	74.36548223	113.6209814	53.72250423	97.37732657	55.68950931	56.02791878	84.85617597	60.49069374	65.20133638	53.72250423	107.1912014	70.98138748	52.64382403	65.16497462	64.82656514
	86.9829222	95.18026565	101.2632979	95.94414894	100.4654255	85.76850095	80	48.72865275	103.3776091	88.19734345	90.62618596	109.4497154	67.48670213	96.21010638	62.96542553	74.83870968	68.76660342	101.8595825	42.9601518	89.10815939	42.04933586	51.76470588	94.57305503	59.65844402	44.88031915	51.15749526	84.85768501	59.35483871	37.60910816	54.91461101	45.08538899
	71.44022848	104.0799674	94.35907411	96.53763859	112.410037	84.49612403	82.5377397	56.75234598	114.1982864	92.98245614	95.59363525	125.2957976	63.74246256	109.297802	67.47714452	66.21787026	77.64177887	113.2190942	49.24520604	96.89922481	35.53651571	61.64830681	106.3647491	79.92656059	63.74246256	45.00203998	99.51040392	73.7250102	48.08241534	51.34638923	56.42594859
	0	0	97.72815534	100.5242718	110.776699	0	0	0	0	0	0	0	71.33980583	112.6407767	66.36893204	0	0	0	0	0	0	0	0	0	62.01941748	0	0	0	0	0	0

AMP3CPR3ERY1FUS2TMP2	AMP	CPR	ERY	FUS	TMP	AMP+CPR	AMP+ERY	AMP+FUS	AMP+TMP	CPR+ERY	CPR+FUS	CPR+TMP	ERY+FUS	ERY+TMP	FUS+TMP	AMP+CPR+ERY	AMP+CPR+FUS	AMP+CPR+TMP	AMP+ERY+FUS	AMP+ERY+TMP	AMP+FUS+TMP	CPR+ERY+FUS	CPR+ERY+TMP	CPR+FUS+TMP	ERY+FUS+TMP	AMP+CPR+ERY+FUS	AMP+CPR+ERY+TMP	AMP+CPR+FUS+TMP	AMP+ERY+FUS+TMP	CPR+ERY+FUS+TMP	AMP+CPR+ERY+FUS+TMP
	96.36209814	94.67005076	95.26991384	85.70423774	99.49006506	105.8375635	92.97800338	57.78341794	98.05414552	104.4839255	80.79526227	113.9593909	68.82363285	87.95498505	67.17074028	87.56345178	74.36548223	102.4534687	53.72250423	101.4382403	55.68950931	56.02791878	104.822335	66.5820643	69.70283102	53.72250423	109.5600677	80.11844332	45.19881557	74.30203046	55.07614213
	86.9829222	95.18026565	101.2632979	95.94414894	101.5292553	85.76850095	80	48.72865275	97.30550285	88.19734345	90.62618596	112.7893738	67.48670213	94.6143617	62.43351064	74.83870968	68.76660342	108.2352941	42.9601518	93.9658444	47.81783681	51.76470588	99.43074004	59.0512334	52.32712766	51.15749526	86.37571157	61.17647059	37.60910816	65.54079696	53.88994307
	71.44022848	104.0799674	94.35907411	96.53763859	109.297802	84.49612403	82.5377397	56.75234598	112.8926969	92.98245614	95.59363525	127.5805794	63.74246256	109.920249	74.63528496	66.21787026	77.64177887	113.2190942	49.24520604	112.2399021	49.57160343	61.64830681	118.7678499	75.03059976	70.900603	45.00203998	98.53121175	83.19053448	39.77968176	59.50632395	58.38433293
	0	0	97.72815534	100.5242718	115.4368932	0	0	0	0	0	0	0	71.33980583	119.1650485	63.86407767	0	0	0	0	0	0	0	0	0	63.88349515	0	0	0	0	0	0

AMP3CPR3ERY1FUS2TMP3	AMP	CPR	ERY	FUS	TMP	AMP+CPR	AMP+ERY	AMP+FUS	AMP+TMP	CPR+ERY	CPR+FUS	CPR+TMP	ERY+FUS	ERY+TMP	FUS+TMP	AMP+CPR+ERY	AMP+CPR+FUS	AMP+CPR+TMP	AMP+ERY+FUS	AMP+ERY+TMP	AMP+FUS+TMP	CPR+ERY+FUS	CPR+ERY+TMP	CPR+FUS+TMP	ERY+FUS+TMP	AMP+CPR+ERY+FUS	AMP+CPR+ERY+TMP	AMP+CPR+FUS+TMP	AMP+ERY+FUS+TMP	CPR+ERY+FUS+TMP	AMP+CPR+ERY+FUS+TMP
	96.36209814	94.67005076	95.26991384	85.70423774	108.2117109	105.8375635	92.97800338	57.78341794	103.4686971	104.4839255	80.79526227	121.0659898	68.82363285	91.04976262	81.48408651	87.56345178	74.36548223	100.0846024	53.72250423	99.06937394	32.00084602	56.02791878	104.822335	68.2106599	67.41691577	53.72250423	89.59390863	99.74619289	26.92470389	76.33248731	68.61252115
	86.9829222	95.18026565	101.2632979	95.94414894	100.4654255	85.76850095	80	48.72865275	89.10815939	88.19734345	90.62618596	106.7172676	67.48670213	94.88031915	78.65691489	74.83870968	68.76660342	100.6451613	42.9601518	88.50094877	35.4459203	51.76470588	99.12713472	65.12333966	60.57180851	51.15749526	80.60721063	79.69639469	37.30550285	72.22011385	55.10436433
	71.44022848	104.0799674	94.35907411	96.53763859	115.8334954	84.49612403	82.5377397	56.75234598	108.9759282	92.98245614	95.59363525	123.0110159	63.74246256	108.3641315	83.4662517	66.21787026	77.64177887	92.32966136	49.24520604	103.4271726	40.10607915	61.64830681	108.3231334	81.55854753	62.30305388	45.00203998	96.89922481	92.65605875	30.96695226	67.99265606	64.9122807
	0	0	97.72815534	100.5242718	108.2912621	0	0	0	0	0	0	0	71.33980583	111.7087379	97.72815534	0	0	0	0	0	0	0	0	0	58.27184466	0	0	0	0	0	0

AMP3CPR3ERY1FUS3TMP1	AMP	CPR	ERY	FUS	TMP	AMP+CPR	AMP+ERY	AMP+FUS	AMP+TMP	CPR+ERY	CPR+FUS	CPR+TMP	ERY+FUS	ERY+TMP	FUS+TMP	AMP+CPR+ERY	AMP+CPR+FUS	AMP+CPR+TMP	AMP+ERY+FUS	AMP+ERY+TMP	AMP+FUS+TMP	CPR+ERY+FUS	CPR+ERY+TMP	CPR+FUS+TMP	ERY+FUS+TMP	AMP+CPR+ERY+FUS	AMP+CPR+ERY+TMP	AMP+CPR+FUS+TMP	AMP+ERY+FUS+TMP	CPR+ERY+FUS+TMP	AMP+CPR+ERY+FUS+TMP
	96.36209814	94.67005076	95.26991384	98.64603482	103.9915597	105.8375635	92.97800338	82.82571912	89.59390863	104.4839255	100.0846024	112.605753	88.23632847	89.36170213	75.01318797	87.56345178	93.65482234	113.6209814	87.90186125	97.37732657	86.88663283	105.499154	84.85617597	76.05752961	68.26094602	80.45685279	107.1912014	89.59390863	87.56345178	87.2250423	87.2250423
	86.9829222	95.18026565	101.2632979	101.7952128	100.4654255	85.76850095	80	76.66034156	103.3776091	88.19734345	101.2523719	109.4497154	89.82712766	96.21010638	69.6143617	74.83870968	82.73244782	101.8595825	79.08918406	89.10815939	76.05313093	94.87666034	94.57305503	90.01897533	58.17819149	73.92789374	84.85768501	83.94686907	76.05313093	78.48197343	74.83870968
	71.44022848	104.0799674	94.35907411	112.410037	112.410037	84.49612403	82.5377397	91.67686659	114.1982864	92.98245614	117.4622603	125.2957976	103.073332	109.297802	78.48667574	66.21787026	89.06568747	113.2190942	80.25295798	96.89922481	87.10730314	102.7743778	106.3647491	92.32966136	68.21630033	70.13463892	99.51040392	88.73929009	80.25295798	81.88494492	77.31538148
	0	0	97.72815534	108.2912621	110.776699	0	0	0	0	0	0	0	100.2135922	112.6407767	83.12621359	0	0	0	0	0	0	0	0	0	66.97087379	0	0	0	0	0	0

AMP3CPR3ERY1FUS3TMP2	AMP	CPR	ERY	FUS	TMP	AMP+CPR	AMP+ERY	AMP+FUS	AMP+TMP	CPR+ERY	CPR+FUS	CPR+TMP	ERY+FUS	ERY+TMP	FUS+TMP	AMP+CPR+ERY	AMP+CPR+FUS	AMP+CPR+TMP	AMP+ERY+FUS	AMP+ERY+TMP	AMP+FUS+TMP	CPR+ERY+FUS	CPR+ERY+TMP	CPR+FUS+TMP	ERY+FUS+TMP	AMP+CPR+ERY+FUS	AMP+CPR+ERY+TMP	AMP+CPR+FUS+TMP	AMP+ERY+FUS+TMP	CPR+ERY+FUS+TMP	AMP+CPR+ERY+FUS+TMP
	96.36209814	94.67005076	95.26991384	98.64603482	99.49006506	105.8375635	92.97800338	82.82571912	98.05414552	104.4839255	100.0846024	113.9593909	88.23632847	87.95498505	86.26692456	87.56345178	93.65482234	102.4534687	87.90186125	101.4382403	81.47208122	105.499154	104.822335	105.8375635	83.172147	80.45685279	109.5600677	99.74619289	75.38071066	83.50253807	97.03891709
	86.9829222	95.18026565	101.2632979	101.7952128	101.5292553	85.76850095	80	76.66034156	97.30550285	88.19734345	101.2523719	112.7893738	89.82712766	94.6143617	82.6462766	74.83870968	82.73244782	108.2352941	79.08918406	93.9658444	74.23149905	94.87666034	99.43074004	101.5559772	77.06117021	73.92789374	86.37571157	80.60721063	66.0341556	79.69639469	72.40986717
	71.44022848	104.0799674	94.35907411	112.410037	109.297802	84.49612403	82.5377397	91.67686659	112.8926969	92.98245614	117.4622603	127.5805794	103.073332	109.920249	95.2927446	66.21787026	89.06568747	113.2190942	80.25295798	112.2399021	81.55854753	102.7743778	118.7678499	102.121583	80.66524023	70.13463892	98.53121175	95.26723786	71.76662587	92.65605875	81.55854753
	0	0	97.72815534	108.2912621	115.4368932	0	0	0	0	0	0	0	100.2135922	119.1650485	89.65048544	0	0	0	0	0	0	0	0	0	75.98058252	0	0	0	0	0	0

AMP3CPR3ERY1FUS3TMP3	AMP	CPR	ERY	FUS	TMP	AMP+CPR	AMP+ERY	AMP+FUS	AMP+TMP	CPR+ERY	CPR+FUS	CPR+TMP	ERY+FUS	ERY+TMP	FUS+TMP	AMP+CPR+ERY	AMP+CPR+FUS	AMP+CPR+TMP	AMP+ERY+FUS	AMP+ERY+TMP	AMP+FUS+TMP	CPR+ERY+FUS	CPR+ERY+TMP	CPR+FUS+TMP	ERY+FUS+TMP	AMP+CPR+ERY+FUS	AMP+CPR+ERY+TMP	AMP+CPR+FUS+TMP	AMP+ERY+FUS+TMP	CPR+ERY+FUS+TMP	AMP+CPR+ERY+FUS+TMP
	96.36209814	94.67005076	95.26991384	98.64603482	108.2117109	105.8375635	92.97800338	82.82571912	103.4686971	104.4839255	100.0846024	121.0659898	88.23632847	91.04976262	105.9609636	87.56345178	93.65482234	100.0846024	87.90186125	99.06937394	55.68950931	105.499154	104.822335	123.0964467	95.55125725	80.45685279	89.59390863	113.9593909	50.67681895	91.28595601	103.8071066
	86.9829222	95.18026565	101.2632979	101.7952128	100.4654255	85.76850095	80	76.66034156	89.10815939	88.19734345	101.2523719	106.7172676	89.82712766	94.88031915	105.2526596	74.83870968	82.73244782	100.6451613	79.08918406	88.50094877	56.9259962	94.87666034	99.12713472	111.2713472	97.00797872	73.92789374	80.60721063	94.57305503	58.74762808	87.28652751	75.14231499
	71.44022848	104.0799674	94.35907411	112.410037	115.8334954	84.49612403	82.5377397	91.67686659	108.9759282	92.98245614	117.4622603	123.0110159	103.073332	108.3641315	109.920249	66.21787026	89.06568747	92.32966136	80.25295798	103.4271726	73.07221542	102.7743778	108.3231334	113.5454916	106.808014	70.13463892	96.89922481	100.1631987	64.9122807	97.87841697	98.53121175
	0	0	97.72815534	108.2912621	108.2912621	0	0	0	0	0	0	0	100.2135922	111.7087379	111.0873786	0	0	0	0	0	0	0	0	0	105.184466	0	0	0	0	0	0

AMP3CPR3ERY2FUS1TMP1	AMP	CPR	ERY	FUS	TMP	AMP+CPR	AMP+ERY	AMP+FUS	AMP+TMP	CPR+ERY	CPR+FUS	CPR+TMP	ERY+FUS	ERY+TMP	FUS+TMP	AMP+CPR+ERY	AMP+CPR+FUS	AMP+CPR+TMP	AMP+ERY+FUS	AMP+ERY+TMP	AMP+FUS+TMP	CPR+ERY+FUS	CPR+ERY+TMP	CPR+FUS+TMP	ERY+FUS+TMP	AMP+CPR+ERY+FUS	AMP+CPR+ERY+TMP	AMP+CPR+FUS+TMP	AMP+ERY+FUS+TMP	CPR+ERY+FUS+TMP	AMP+CPR+ERY+FUS+TMP
	96.36209814	94.67005076	103.8353601	70.23034992	103.9915597	105.8375635	106.8527919	11.06175973	89.59390863	96.70050761	83.43908629	112.605753	69.71000935	113.414406	64.91999297	96.36209814	58.79864636	113.6209814	18.84517766	90.94754653	43.50676819	58.73519459	100.7614213	69.90270728	52.66604303	31.72588832	101.7766497	59.75042301	44.86040609	67.87225042	45.19881557
	86.9829222	95.18026565	102.1828909	82.6462766	100.4654255	85.76850095	76.35673624	16.66034156	103.3776091	93.05502846	54.00379507	109.4497154	77.95476893	110.993117	54.38829787	83.33965844	45.38899431	101.8595825	27.28652751	92.75142315	27.55218216	50.36053131	107.6280835	63.41555977	72.86135693	26.86907021	106.1100569	59.0512334	23.64326376	57.0398482	46.60341556
	71.44022848	104.0799674	88.62788963	74.01283797	112.410037	84.49612403	70.13463892	19.54304366	114.1982864	97.2256222	67.19706242	125.2957976	82.06562267	107.7181208	71.21182649	71.44022848	47.93961648	113.2190942	16.27906977	98.85760914	31.94614443	55.91595267	118.4414525	71.58302734	56.7114094	28.68217054	100.8159935	60.99551204	27.19298246	57.87433701	52.50917993
	0	0	0	81.26213592	110.776699	0	0	0	0	0	0	0	0	0	72.89320388	0	0	0	0	0	0	0	0	0	0	0	0	0	0	0	0

AMP3CPR3ERY2FUS1TMP2	AMP	CPR	ERY	FUS	TMP	AMP+CPR	AMP+ERY	AMP+FUS	AMP+TMP	CPR+ERY	CPR+FUS	CPR+TMP	ERY+FUS	ERY+TMP	FUS+TMP	AMP+CPR+ERY	AMP+CPR+FUS	AMP+CPR+TMP	AMP+ERY+FUS	AMP+ERY+TMP	AMP+FUS+TMP	CPR+ERY+FUS	CPR+ERY+TMP	CPR+FUS+TMP	ERY+FUS+TMP	AMP+CPR+ERY+FUS	AMP+CPR+ERY+TMP	AMP+CPR+FUS+TMP	AMP+ERY+FUS+TMP	CPR+ERY+FUS+TMP	AMP+CPR+ERY+FUS+TMP
	96.36209814	94.67005076	103.8353601	70.23034992	99.49006506	105.8375635	106.8527919	11.06175973	98.05414552	96.70050761	83.43908629	113.9593909	69.71000935	120.2993452	64.63864955	96.36209814	58.79864636	102.4534687	18.84517766	99.74619289	18.80287648	58.73519459	108.8832487	83.10067682	62.84377923	31.72588832	109.5600677	59.13705584	14.06514382	58.05837563	43.84517766
	86.9829222	95.18026565	102.1828909	82.6462766	101.5292553	85.76850095	76.35673624	16.66034156	97.30550285	93.05502846	54.00379507	112.7893738	77.95476893	119.1740413	55.25265957	83.33965844	45.38899431	108.2352941	27.28652751	91.5370019	20.26565465	50.36053131	110.6641366	71.30929791	76.32251721	26.86907021	99.73434535	56.31878558	21.4800759	50.66413662	35.78747628
	71.44022848	104.0799674	88.62788963	74.01283797	109.297802	84.49612403	70.13463892	19.54304366	112.8926969	97.2256222	67.19706242	127.5805794	82.06562267	112.7889635	60.94145108	71.44022848	47.93961648	113.2190942	16.27906977	108.6495308	23.78620971	55.91595267	120.0734394	67.66625867	63.87024609	28.68217054	107.996736	60.66911465	17.58465932	56.89514484	37.96409629
	0	0	0	81.26213592	115.4368932	0	0	0	0	0	0	0	0	0	72.58252427	0	0	0	0	0	0	0	0	0	0	0	0	0	0	0	0

AMP3CPR3ERY2FUS1TMP3	AMP	CPR	ERY	FUS	TMP	AMP+CPR	AMP+ERY	AMP+FUS	AMP+TMP	CPR+ERY	CPR+FUS	CPR+TMP	ERY+FUS	ERY+TMP	FUS+TMP	AMP+CPR+ERY	AMP+CPR+FUS	AMP+CPR+TMP	AMP+ERY+FUS	AMP+ERY+TMP	AMP+FUS+TMP	CPR+ERY+FUS	CPR+ERY+TMP	CPR+FUS+TMP	ERY+FUS+TMP	AMP+CPR+ERY+FUS	AMP+CPR+ERY+TMP	AMP+CPR+FUS+TMP	AMP+ERY+FUS+TMP	CPR+ERY+FUS+TMP	AMP+CPR+ERY+FUS+TMP
	96.36209814	94.67005076	103.8353601	70.23034992	108.2117109	105.8375635	106.8527919	11.06175973	103.4686971	96.70050761	83.43908629	121.0659898	69.71000935	121.7960711	73.92298224	96.36209814	58.79864636	100.0846024	18.84517766	95.34686971	18.12605753	58.73519459	113.6209814	75.31725888	66.41721235	31.72588832	93.65482234	74.36548223	15.7571912	67.19543147	42.82994924

	AMP	CPR	ERY	FUS	TMP	AMP+CPR	AMP+ERY	AMP+FUS	AMP+TMP	CPR+ERY	CPR+FUS	CPR+TMP	ERY+FUS	ERY+TMP	FUS+TMP	AMP+CPR+ERY	AMP+CPR+FUS	…
	86.9829222	95.18026565	102.1828909	82.6462766	100.4654255	85.76850095	76.35673624	16.66034156	89.10815939	93.05502846	54.00379507	106.7172676	77.95476893	124.5231072	59.50797872	83.33965844	45.38899431	[illegible]
	71.44022848	104.0799674	88.62788963	74.01283797	115.8334954	84.49612403	70.13463892	19.54304366	108.9759282	97.2256222	67.19706242	123.0110159	82.06562267	112.7889635	68.41081502	71.44022848	47.93961648	[illegible]
	0	0	0	81.26213592	108.2912621	0	0	0	0	0	0	0	0	0	74.75728155	0	0	[illegible]
AMP3CPR3ERY2FUS2TMP1	AMP	CPR	ERY	FUS	TMP	AMP+CPR	AMP+ERY	AMP+FUS	AMP+TMP	CPR+ERY	CPR+FUS	CPR+TMP	ERY+FUS	ERY+TMP	FUS+TMP	AMP+CPR+ERY	AMP+CPR+FUS	[illegible]
	96.36209814	94.67005076	103.8353601	85.70423774	103.9915597	105.8375635	106.8527919	57.78341794	89.59390863	96.70050761	80.79526227	112.605753	76.59494855	113.414406	66.0101987	96.36209814	74.36548223	[illegible]
	86.9829222	95.18026565	102.1828909	95.94414894	100.4654255	85.76850095	76.35673624	48.72865275	103.3776091	93.05502846	90.62618596	109.4497154	89.91150442	110.993117	62.96542553	83.33965844	68.76660342	[illegible]
	71.44022848	104.0799674	88.62788963	96.53763859	112.410037	84.49612403	70.13463892	56.75234598	114.1982864	97.2256222	95.59363525	125.2957976	83.55704698	107.7181208	67.47714452	71.44022848	77.64177887	[illegible]
	0	0	0	100.5242718	110.776699	0	0	0	0	0	0	0	0	0	66.36893204	0	0	[illegible]
AMP3CPR3ERY2FUS2TMP2	AMP	CPR	ERY	FUS	TMP	AMP+CPR	AMP+ERY	AMP+FUS	AMP+TMP	CPR+ERY	CPR+FUS	CPR+TMP	ERY+FUS	ERY+TMP	FUS+TMP	AMP+CPR+ERY	AMP+CPR+FUS	[illegible]
	96.36209814	94.67005076	103.8353601	85.70423774	99.49006506	105.8375635	106.8527919	57.78341794	98.05414552	96.70050761	80.79526227	113.9593909	76.59494855	120.2993452	67.17074028	96.36209814	74.36548223	[illegible]
	86.9829222	95.18026565	102.1828909	95.94414894	101.5292553	85.76850095	76.35673624	48.72865275	97.30550285	93.05502846	90.62618596	112.7893738	89.91150442	119.1740413	62.43351064	83.33965844	68.76660342	[illegible]
	71.44022848	104.0799674	88.62788963	96.53763859	109.297802	84.49612403	70.13463892	56.75234598	112.8926969	97.2256222	95.59363525	127.5805794	83.55704698	112.7889635	74.63528496	71.44022848	77.64177887	[illegible]
	0	0	0	100.5242718	115.4368932	0	0	0	0	0	0	0	0	0	63.86407767	0	0	[illegible]
AMP3CPR3ERY2FUS2TMP3	AMP	CPR	ERY	FUS	TMP	AMP+CPR	AMP+ERY	AMP+FUS	AMP+TMP	CPR+ERY	CPR+FUS	CPR+TMP	ERY+FUS	ERY+TMP	FUS+TMP	AMP+CPR+ERY	AMP+CPR+FUS	[illegible]
	96.36209814	94.67005076	103.8353601	85.70423774	108.2117109	105.8375635	106.8527919	57.78341794	103.4686971	96.70050761	80.79526227	121.0659898	76.59494855	121.7960711	81.48408651	96.36209814	74.36548223	[illegible]
	86.9829222	95.18026565	102.1828909	95.94414894	100.4654255	85.76850095	76.35673624	48.72865275	89.10815939	93.05502846	90.62618596	106.7172676	89.91150442	124.5231072	78.65691489	83.33965844	68.76660342	[illegible]
	71.44022848	104.0799674	88.62788963	96.53763859	115.8334954	84.49612403	70.13463892	56.75234598	108.9759282	97.2256222	95.59363525	123.0110159	83.55704698	112.7889635	83.4662517	71.44022848	77.64177887	[illegible]
	0	0	0	100.5242718	108.2912621	0	0	0	0	0	0	0	0	0	97.72815534	0	0	[illegible]
AMP3CPR3ERY2FUS3TMP1	AMP	CPR	ERY	FUS	TMP	AMP+CPR	AMP+ERY	AMP+FUS	AMP+TMP	CPR+ERY	CPR+FUS	CPR+TMP	ERY+FUS	ERY+TMP	FUS+TMP	AMP+CPR+ERY	AMP+CPR+FUS	[illegible]
	96.36209814	94.67005076	103.8353601	98.64603482	103.9915597	105.8375635	106.8527919	82.82571912	89.59390863	96.70050761	100.0846024	112.605753	106.828812	113.414406	75.01318797	96.36209814	93.65482234	[illegible]
	86.9829222	95.18026565	102.1828909	101.7952128	100.4654255	85.76850095	76.35673624	76.66034156	103.3776091	93.05502846	101.2523719	109.4497154	105.6440511	110.993117	69.6143617	83.33965844	82.73244782	[illegible]
	71.44022848	104.0799674	88.62788963	112.410037	112.410037	84.49612403	70.13463892	91.67686659	114.1982864	97.2256222	117.4622603	125.2957976	102.945563	107.7181208	78.48667574	71.44022848	89.06568747	[illegible]
	0	0	0	108.2912621	110.776699	0	0	0	0	0	0	0	0	0	83.12621359	0	0	[illegible]
AMP3CPR3ERY2FUS3TMP2	AMP	CPR	ERY	FUS	TMP	AMP+CPR	AMP+ERY	AMP+FUS	AMP+TMP	CPR+ERY	CPR+FUS	CPR+TMP	ERY+FUS	ERY+TMP	FUS+TMP	AMP+CPR+ERY	AMP+CPR+FUS	[illegible]
	96.36209814	94.67005076	103.8353601	98.64603482	99.49006506	105.8375635	106.8527919	82.82571912	98.05414552	96.70050761	100.0846024	113.9593909	106.828812	120.2993452	86.26692456	96.36209814	93.65482234	[illegible]
	86.9829222	95.18026565	102.1828909	101.7952128	101.5292553	85.76850095	76.35673624	76.66034156	97.30550285	93.05502846	101.2523719	112.7893738	105.6440511	119.1740413	82.6462766	83.33965844	82.73244782	[illegible]
	71.44022848	104.0799674	88.62788963	112.410037	109.297802	84.49612403	70.13463892	91.67686659	112.8926969	97.2256222	117.4622603	127.5805794	102.945563	112.7889635	95.2927446	71.44022848	89.06568747	[illegible]
	0	0	0	108.2912621	115.4368932	0	0	0	0	0	0	0	0	0	89.65048544	0	0	[illegible]
AMP3CPR3ERY2FUS3TMP3	AMP	CPR	ERY	FUS	TMP	AMP+CPR	AMP+ERY	AMP+FUS	AMP+TMP	CPR+ERY	CPR+FUS	CPR+TMP	ERY+FUS	ERY+TMP	FUS+TMP	AMP+CPR+ERY	AMP+CPR+FUS	[illegible]
	96.36209814	94.67005076	103.8353601	98.64603482	108.2117109	105.8375635	106.8527919	82.82571912	103.4686971	96.70050761	100.0846024	121.0659898	106.828812	121.7960711	105.9609636	96.36209814	93.65482234	[illegible]
	86.9829222	95.18026565	102.1828909	101.7952128	100.4654255	85.76850095	76.35673624	76.66034156	89.10815939	93.05502846	101.2523719	106.7172676	105.6440511	124.5231072	105.2526596	83.33965844	82.73244782	[illegible]
	71.44022848	104.0799674	88.62788963	112.410037	115.8334954	84.49612403	70.13463892	91.67686659	108.9759282	97.2256222	117.4622603	123.0110159	102.945563	112.7889635	109.920249	71.44022848	89.06568747	[illegible]
	0	0	0	108.2912621	108.2912621	0	0	0	0	0	0	0	0	0	111.0873786	0	0	[illegible]
AMP3CPR3ERY3FUS1TMP1	AMP	CPR	ERY	FUS	TMP	AMP+CPR	AMP+ERY	AMP+FUS	AMP+TMP	CPR+ERY	CPR+FUS	CPR+TMP	ERY+FUS	ERY+TMP	FUS+TMP	AMP+CPR+ERY	AMP+CPR+FUS	[illegible]
	96.36209814	94.67005076	95.34686971	70.23034992	103.9915597	105.8375635	109.5600677	11.06175973	89.59390863	93.31641286	83.43908629	112.605753	80.79526227	114.6362098	64.91999297	96.70050761	58.79864636	[illegible]
	86.9829222	95.18026565	99.12713472	82.6462766	100.4654255	85.76850095	84.25047438	16.66034156	103.3776091	95.18026565	54.00379507	109.4497154	90.32258065	114.914611	54.38829787	84.85768501	45.38899431	[illegible]
	71.44022848	104.0799674	108.9759282	74.01283797	112.410037	84.49612403	68.50265198	19.54304366	114.1982864	100.4895961	67.19706242	125.2957976	93.63525092	120.3998368	71.21182649	78.29457364	47.93961648	[illegible]
	0	0	0	81.26213592	110.776699	0	0	0	0	0	0	0	0	0	72.89320388	0	0	[illegible]

[illegible]

AMP1CPR2FUS1DOX1TMP3	AMP	CPR	FUS	DOX	TMP	AMP+CPR	AMP+FUS	AMP+DOX	AMP+TMP	CPR+FUS	CPR+DOX	CPR+TMP	FUS+DOX	FUS+TMP	DOX+TMP	AMP+CPR+FUS	AMP+CPR+DOX	AMP+CPR+TMP	AMP+FUS+DOX	AMP+FUS+TMP	AMP+DOX+TMP	CPR+FUS+DOX	CPR+FUS+TMP	CPR+DOX+TMP	FUS+DOX+TMP	AMP+CPR+FUS+DOX	AMP+CPR+FUS+TMP	AMP+CPR+DOX+TMP	AMP+FUS+DOX+TMP	CPR+FUS+DOX+TMP	AMP+CPR+FUS+DOX+TMP
	78.14815665	86.38437378	72.56876735	88.42597903	98.11649728	49.9575581	39.12913809	66.98937805	81.96563353	87.66821798	81.50576584	76.5171141	74.33067976	73.74337562	99.29110555	20.62905779	59.06077221	66.40207392	15.04966849	2.97322719	71.98146321	76.5171141	72.70226278	69.76776176	95.76728074	27.97035949	42.94661497	61.11633669	16.77487439	77.69091451	27.97035949
	72.2704949	98.12693389	80.73290518	90.40423121	99.16887042	64.11031356	6.384586324	65.92368719	71.96826596	86.20355717	89.52925337	76.52880459	65.01700038	98.56441254	99.16887042	29.31620703	46.2788062	40.8386853	10.9180204	2.417831507	69.55043445	72.46616435	92.25027754	76.96341262	111.258028	14.54476766	23.27162826	74.99055535	6.384586324	82.57552495	29.01397809
	82.62638001	85.26818516	93.47278714	95.95196591	102.4598102	73.63935696	9.18070889	75.80863839	77.35812512	76.10213079	89.08890522	69.10360029	63.10284718	94.09258183	104.938989	28.37497579	72.70966492	87.27484021	7.321324811	19.07805539	79.83730389	78.74724467	84.62527553	77.92064658	95.33217122	27.7551811	49.44799535	82.00658532	30.54425721	86.68258633	25.56653109
AMP1CPR2FUS1DOX2TMP1	AMP	CPR	FUS	DOX	TMP	AMP+CPR	AMP+FUS	AMP+DOX	AMP+TMP	CPR+FUS	CPR+DOX	CPR+TMP	FUS+DOX	FUS+TMP	DOX+TMP	AMP+CPR+FUS	AMP+CPR+DOX	AMP+CPR+TMP	AMP+FUS+DOX	AMP+FUS+TMP	AMP+DOX+TMP	CPR+FUS+DOX	CPR+FUS+TMP	CPR+DOX+TMP	FUS+DOX+TMP	AMP+CPR+FUS+DOX	AMP+CPR+FUS+TMP	AMP+CPR+DOX+TMP	AMP+FUS+DOX+TMP	CPR+FUS+DOX+TMP	AMP+CPR+FUS+DOX+TMP
	78.14815665	86.38437378	72.56876735	92.53710799	99.29110555	49.9575581	39.12913809	66.69572599	83.1402418	87.66821798	80.91886563	81.50576584	82.2592856	84.90215421	100.7593659	20.62905779	56.4179036	39.97338778	10.64488747	54.98634977	70.51320287	84.73371696	85.32061716	74.16951329	96.0609328	36.1926174	36.48626947	54.94964326	12.99410402	75.34331369	31.49418431
	72.2704949	98.12693389	80.73290518	89.19531545	98.2621836	64.11031356	6.384586324	81.94182093	86.47525501	86.20355717	70.48208423	75.31946052	86.77748394	76.80392898	97.35549679	29.31620703	48.09217983	36.30525123	54.70343785	44.12542501	67.43483189	94.66896568	86.50589319	78.3428207	101.8889309	38.98753306	33.8496411	85.87079713	0.642236494	87.11056523	43.21873819
	82.62638001	85.26818516	93.47278714	100.9103235	93.47278714	73.63935696	9.18070889	84.79566144	81.07689328	76.10213079	72.86921381	76.39603233	100.6004261	101.2202208	104.0092969	28.37497579	73.32945962	71.77997288	9.490606237	44.17974046	88.20453225	95.20573108	66.40337987	64.05216752	73.32945962	37.67189618	52.85686616	79.2175092	6.682161534	81.09845702	31.45458067
AMP1CPR2FUS1DOX2TMP2	AMP	CPR	FUS	DOX	TMP	AMP+CPR	AMP+FUS	AMP+DOX	AMP+TMP	CPR+FUS	CPR+DOX	CPR+TMP	FUS+DOX	FUS+TMP	DOX+TMP	AMP+CPR+FUS	AMP+CPR+DOX	AMP+CPR+TMP	AMP+FUS+DOX	AMP+FUS+TMP	AMP+DOX+TMP	CPR+FUS+DOX	CPR+FUS+TMP	CPR+DOX+TMP	FUS+DOX+TMP	AMP+CPR+FUS+DOX	AMP+CPR+FUS+TMP	AMP+CPR+DOX+TMP	AMP+FUS+DOX+TMP	CPR+FUS+DOX+TMP	AMP+CPR+FUS+DOX+TMP
	78.14815665	86.38437378	72.56876735	92.53710799	94.59267246	49.9575581	39.12913809	66.69572599	81.96563353	87.66821798	80.91886563	73.58261308	82.2592856	94.59267246	103.6958866	20.62905779	56.4179036	55.83059946	10.64488747	60.85939113	86.95771869	84.73371696	77.69091451	83.55991655	70.80685494	36.1926174	31.49418431	49.9575581	11.81949574	85.32061716	17.39888504
	72.2704949	98.12693389	80.73290518	89.19531545	92.51983377	64.11031356	6.384586324	81.94182093	78.61730261	86.20355717	70.48208423	78.94749274	86.77748394	102.7956177	107.0268228	29.31620703	48.09217983	33.58519078	54.70343785	55.61012467	63.80808462	94.66896568	78.04048468	81.6685169	82.24404987	38.98753306	25.42500944	75.59501322	10.27578391	74.11011645	29.61843597
	82.62638001	85.26818516	93.47278714	100.9103235	99.36083672	73.63935696	9.18070889	84.79566144	75.80863839	76.10213079	72.86921381	72.63041881	100.6004261	102.1499128	89.13422429	28.37497579	73.32945962	75.80863839	9.490606237	65.56265737	86.96494286	95.20573108	71.69360764	84.09257899	84.79566144	37.67189618	66.80224676	70.23048615	6.99205888	73.14474651	12.5895797
AMP1CPR2FUS1DOX2TMP3	AMP	CPR	FUS	DOX	TMP	AMP+CPR	AMP+FUS	AMP+DOX	AMP+TMP	CPR+FUS	CPR+DOX	CPR+TMP	FUS+DOX	FUS+TMP	DOX+TMP	AMP+CPR+FUS	AMP+CPR+DOX	AMP+CPR+TMP	AMP+FUS+DOX	AMP+FUS+TMP	AMP+DOX+TMP	CPR+FUS+DOX	CPR+FUS+TMP	CPR+DOX+TMP	FUS+DOX+TMP	AMP+CPR+FUS+DOX	AMP+CPR+FUS+TMP	AMP+CPR+DOX+TMP	AMP+FUS+DOX+TMP	CPR+FUS+DOX+TMP	AMP+CPR+FUS+DOX+TMP
	78.14815665	86.38437378	72.56876735	92.53710799	98.11649728	49.9575581	39.12913809	66.69572599	81.96563353	87.66821798	80.91886563	76.5171141	82.2592856	73.74337562	103.9895386	20.62905779	56.4179036	66.40207392	10.64488747	2.97322719	72.86241942	84.73371696	72.70226278	96.17827094	79.32276492	36.1926174	42.94661497	67.87033426	30.61322811	103.2210734	39.71644222
	72.2704949	98.12693389	80.73290518	89.19531545	99.16887042	64.11031356	6.384586324	81.94182093	71.96826596	86.20355717	70.48208423	76.52880459	86.77748394	98.56441254	98.56441254	29.31620703	48.09217983	40.8386853	54.70343785	2.417831507	65.62145825	94.66896568	92.25027754	96.48298179	99.47109936	38.98753306	23.27162826	81.03513411	1.208915754	88.01757328	34.75632792
	82.62638001	85.26818516	93.47278714	100.9103235	102.4598102	73.63935696	9.18070889	84.79566144	77.35812512	76.10213079	72.86921381	69.10360029	100.6004261	94.09258183	91.92330041	28.37497579	73.32945962	87.27484021	9.490606237	19.07805539	84.79566144	95.20573108	84.62527553	72.86921381	94.71237653	37.67189618	49.44799535	82.62638001	6.682161534	82.86186627	30.23435987
AMP1CPR2FUS1DOX3TMP1	AMP	CPR	FUS	DOX	TMP	AMP+CPR	AMP+FUS	AMP+DOX	AMP+TMP	CPR+FUS	CPR+DOX	CPR+TMP	FUS+DOX	FUS+TMP	DOX+TMP	AMP+CPR+FUS	AMP+CPR+DOX	AMP+CPR+TMP	AMP+FUS+DOX	AMP+FUS+TMP	AMP+DOX+TMP	CPR+FUS+DOX	CPR+FUS+TMP	CPR+DOX+TMP	FUS+DOX+TMP	AMP+CPR+FUS+DOX	AMP+CPR+FUS+TMP	AMP+CPR+DOX+TMP	AMP+FUS+DOX+TMP	CPR+FUS+DOX+TMP	AMP+CPR+FUS+DOX+TMP
	78.14815665	86.38437378	72.56876735	103.4022345	99.29110555	49.9575581	39.12913809	80.20372112	83.1402418	87.66821798	79.04812123	81.50576584	87.8386749	84.90215421	106.6324073	20.62905779	47.31468949	39.97338778	23.2719264	54.98634977	69.63224667	75.6367638	85.32061716	98.81932186	81.96563353	35.60531327	36.48626947	70.2195508	36.1926174	98.23242165	27.38305536
	72.2704949	98.12693389	80.73290518	94.93766528	98.2621836	64.11031356	6.384586324	88.89308651	86.47525501	86.20355717	75.62179654	75.31946052	94.93766528	76.80392898	100.6800151	29.31620703	42.34983	36.30525123	24.48054401	44.12542501	60.78579524	71.55915629	86.50589319	89.22691735	94.93766528	29.9206649	33.8496411	85.56856819	41.40536456	85.59888514	47.14771439
	82.62638001	85.26818516	93.47278714	112.6864226	93.47278714	73.63935696	9.18070889	83.55607205	81.07689328	76.10213079	75.5143277	76.39603233	106.1785783	101.2202208	108.6577571	28.37497579	82.00658532	71.77997288	14.44896378	44.17974046	83.8659694	73.75091844	66.40337987	85.50698016	80.76699593	35.50261476	52.85686616	84.48576409	52.85686616	81.98016165	39.53128026
AMP1CPR2FUS1DOX3TMP2	AMP	CPR	FUS	DOX	TMP	AMP+CPR	AMP+FUS	AMP+DOX	AMP+TMP	CPR+FUS	CPR+DOX	CPR+TMP	FUS+DOX	FUS+TMP	DOX+TMP	AMP+CPR+FUS	AMP+CPR+DOX	AMP+CPR+TMP	AMP+FUS+DOX	AMP+FUS+TMP	AMP+DOX+TMP	CPR+FUS+DOX	CPR+FUS+TMP	CPR+DOX+TMP	FUS+DOX+TMP	AMP+CPR+FUS+DOX	AMP+CPR+FUS+TMP	AMP+CPR+DOX+TMP	AMP+FUS+DOX+TMP	CPR+FUS+DOX+TMP	AMP+CPR+FUS+DOX+TMP
	78.14815665	86.38437378	72.56876735	103.4022345	94.59267246	49.9575581	39.12913809	80.20372112	81.96563353	87.66821798	79.04812123	73.58261308	87.8386749	94.59267246	102.5212783	20.62905779	47.31468949	55.83059946	23.2719264	60.85939113	67.87033426	75.6367638	77.69091451	86.78786767	98.11649728	35.60531327	31.49418431	86.95771869	0.954369222	95.59137073	33.51304228
	72.2704949	98.12693389	80.73290518	94.93766528	92.51983377	64.11031356	6.384586324	88.89308651	78.61730261	86.20355717	75.62179654	78.94749274	94.93766528	102.7956177	92.21760484	29.31620703	42.34983	33.58519078	24.48054401	55.61012467	57.46127692	71.55915629	78.04048468	78.64515672	89.79977333	29.9206649	25.42500944	76.50170004	44.12542501	83.48253301	51.68114847
	82.62638001	85.26818516	93.47278714	112.6864226	99.36083672	73.63935696	9.18070889	83.55607205	75.80863839	76.10213079	75.5143277	72.63041881	106.1785783	102.1499128	101.5301181	28.37497579	82.00658532	75.80863839	14.44896378	65.56265737	77.66802247	73.75091844	71.69360764	89.97060985	95.64206856	35.50261476	66.80224676	83.2461747	15.99845051	94.32402645	36.74220414
AMP1CPR2FUS1DOX3TMP3	AMP	CPR	FUS	DOX	TMP	AMP+CPR	AMP+FUS	AMP+DOX	AMP+TMP	CPR+FUS	CPR+DOX	CPR+TMP	FUS+DOX	FUS+TMP	DOX+TMP	AMP+CPR+FUS	AMP+CPR+DOX	AMP+CPR+TMP	AMP+FUS+DOX	AMP+FUS+TMP	AMP+DOX+TMP	CPR+FUS+DOX	CPR+FUS+TMP	CPR+DOX+TMP	FUS+DOX+TMP	AMP+CPR+FUS+DOX	AMP+CPR+FUS+TMP	AMP+CPR+DOX+TMP	AMP+FUS+DOX+TMP	CPR+FUS+DOX+TMP	AMP+CPR+FUS+DOX+TMP
	78.14815665	86.38437378	72.56876735	103.4022345	98.11649728	49.9575581	39.12913809	80.20372112	81.96563353	87.66821798	79.04812123	76.5171141	87.8386749	73.74337562	91.65615178	20.62905779	47.31468949	66.40207392	23.2719264	2.97322719	78.14815665	75.6367638	72.70226278	86.09092368	76.97354837	35.60531327	42.94661497	76.09259217	16.22427677	100.5800225	14.75601643
	72.2704949	98.12693389	80.73290518	94.93766528	99.16887042	64.11031356	6.384586324	88.89308651	71.96826596	86.20355717	75.62179654	76.52880459	94.93766528	98.56441254	94.03097847	29.31620703	42.34983	40.8386853	24.48054401	2.417831507	84.35965244	71.55915629	92.25027754	100.8479581	82.84850774	29.9206649	23.27162826	67.13260295	32.33849641	74.11011645	35.66301473
	82.62638001	85.26818516	93.47278714	112.6864226	102.4598102	73.63935696	9.18070889	83.55607205	77.35812512	76.10213079	75.5143277	69.10360029	106.1785783	94.09258183	101.5301181	28.37497579	82.00658532	87.27484021	14.44896378	19.07805539	81.38679063	73.75091844	84.62527553	96.43644379	80.45709859	35.50261476	49.44799535	71.16017819	22.17702886	86.09478325	22.15766028
AMP1CPR2FUS2DOX1TMP1	AMP	CPR	FUS	DOX	TMP	AMP+CPR	AMP+FUS	AMP+DOX	AMP+TMP	CPR+FUS	CPR+DOX	CPR+TMP	FUS+DOX	FUS+TMP	DOX+TMP	AMP+CPR+FUS	AMP+CPR+DOX	AMP+CPR+TMP	AMP+FUS+DOX	AMP+FUS+TMP	AMP+DOX+TMP	CPR+FUS+DOX	CPR+FUS+TMP	CPR+DOX+TMP	FUS+DOX+TMP	AMP+CPR+FUS+DOX	AMP+CPR+FUS+TMP	AMP+CPR+DOX+TMP	AMP+FUS+DOX+TMP	CPR+FUS+DOX+TMP	AMP+CPR+FUS+DOX+TMP
	78.14815665	86.38437378	97.64552145	88.42597903	99.29110555	49.9575581	43.24026704	66.98937805	83.1402418	96.47172104	81.50576584	81.50576584	90.8961691	99.11277196	96.94188901	30.31957604	59.06077221	39.97338778	21.21636193	49.70061254	63.7592053	96.17827094	88.84201839	78.27781471	97.64552145	32.37514052	37.66087774	51.42581844	39.97338778	92.95031981	31.20053224
	72.2704949	98.12693389	108.8787585	90.40423121	98.2621836	64.11031356	52.58783529	65.92368719	86.47525501	98.59933392	89.52925337	75.31946052	79.24982875	90.43626143	92.82206271	33.24518323	46.2788062	36.30525123	22.40272006	32.03626747	74.99055535	88.3199093	97.99466188	80.59144484	91.34326948	25.9916887	43.52096713	59.87910842	29.01397809	108.5764225	32.37627503
	82.62638001	85.26818516	96.6752388	95.95196591	93.47278714	73.63935696	57.19542901	75.80863839	81.07689328	80.21675239	89.08890522	76.39603233	56.39235856	90.79720794	108.9676545	30.54425721	72.70966492	71.77997288	31.16405191	34.26302537	82.93627736	96.08743571	81.09845702	73.51212344	101.0837619	29.92446252	61.22409452	78.90761185	49.13809801	73.4570169	36.12240945
AMP1CPR2FUS2DOX1TMP2	AMP	CPR	FUS	DOX	TMP	AMP+CPR	AMP+FUS	AMP+DOX	AMP+TMP	CPR+FUS	CPR+DOX	CPR+TMP	FUS+DOX	FUS+TMP	DOX+TMP	AMP+CPR+FUS	AMP+CPR+DOX	AMP+CPR+TMP	AMP+FUS+DOX	AMP+FUS+TMP	AMP+DOX+TMP	CPR+FUS+DOX	CPR+FUS+TMP	CPR+DOX+TMP	FUS+DOX+TMP	AMP+CPR+FUS+DOX	AMP+CPR+FUS+TMP	AMP+CPR+DOX+TMP	AMP+FUS+DOX+TMP	CPR+FUS+DOX+TMP	AMP+CPR+FUS+DOX+TMP
	78.14815665	86.38437378	97.64552145	88.42597903	94.59267246	49.9575581	43.24026704	66.98937805	81.96563353	96.47172104	81.50576584	73.58261308	90.8961691	92.65686971	99.58475762	30.31957604	59.06077221	55.83059946	21.21636193	57.04191424	62.29094496	96.17827094	92.06996951	80.33196543	101.7538229	32.37514052	47.64504806	64.64016151	43.4972126	88.54856828	32.96244465
	72.2704949	98.12693389	108.8787585	90.40423121	92.51983377	64.11031356	52.58783529	65.92368719	78.61730261	98.59933392	89.52925337	78.94749274	79.24982875	89.52925337	96.14658104	33.24518323	46.2788062	33.58519078	22.40272006	52.89006422	71.66603702	88.3199093	107.3670784	68.49800411	88.3199093	25.9916887	49.5655459	68.3415187	37.7786173	96.78531781	26.6339252
	82.62638001	85.26818516	96.6752388	95.95196591	99.36083672	73.63935696	57.19542901	75.80863839	75.80863839	80.21675239	89.08890522	72.63041881	56.39235856	83.44966936	100.6004261	30.54425721	72.70966492	75.80863839	31.16405191	60.91419717	76.42843308	96.08743571	87.85819251	69.39750184	101.0837619	29.92446252	50.99748208	88.20453225	66.18245206	106.6678913	33.02343599
AMP1CPR2FUS2DOX1TMP3	AMP	CPR	FUS	DOX	TMP	AMP+CPR	AMP+FUS	AMP+DOX	AMP+TMP	CPR+FUS	CPR+DOX	CPR+TMP	FUS+DOX	FUS+TMP	DOX+TMP	AMP+CPR+FUS	AMP+CPR+DOX	AMP+CPR+TMP	AMP+FUS+DOX	AMP+FUS+TMP	AMP+DOX+TMP	CPR+FUS+DOX	CPR+FUS+TMP	CPR+DOX+TMP	FUS+DOX+TMP	AMP+CPR+FUS+DOX	AMP+CPR+FUS+TMP	AMP+CPR+DOX+TMP	AMP+FUS+DOX+TMP	CPR+FUS+DOX+TMP	AMP+CPR+FUS+DOX+TMP
	78.14815665	86.38437378	97.64552145	88.42597903	98.11649728	49.9575581	43.24026704	66.98937805	81.96563353	96.47172104	81.50576584	76.5171141	90.8961691	101.1669227	99.29110555	30.31957604	59.06077221	66.40207392	21.21636193	58.51017459	71.98146321	96.17827094	92.65686971	69.76776176	94.41757032	32.37514052	52.93078529	61.11633669	47.64504806	95.59137073	20.92270986
	72.2704949	98.12693389	108.8787585	90.40423121	99.16887042	64.11031356	52.58783529	65.92368719	71.96826596	98.59933392	89.52925337	76.52880459	79.24982875	94.06429364	99.16887042	33.24518323	46.2788062	40.8386853	22.40272006	45.33434076	69.55043445	88.3199093	109.1810946	76.96341262	73.50544441	25.9916887	52.28560635	74.99055535	64.07253495	99.20400595	21.19380431
	82.62638001	85.26818516	96.6752388	95.95196591	102.4598102	73.63935696	57.19542901	75.80863839	77.35812512	80.21675239	89.08890522	69.10360029	56.39235856	96.96914034	104.938989	30.54425721	72.70966492	87.27484021	31.16405191	58.12512105	79.83730389	96.08743571	87.27038942	77.92064658	87.85819251	29.92446252	59.67460779	82.00658532	64.94286268	108.4313005	14.11969785
AMP1CPR2FUS2DOX2TMP1	AMP	CPR	FUS	DOX	TMP	AMP+CPR	AMP+FUS	AMP+DOX	AMP+TMP	CPR+FUS	CPR+DOX	CPR+TMP	FUS+DOX	FUS+TMP	DOX+TMP	AMP+CPR+FUS	AMP+CPR+DOX	AMP+CPR+TMP	AMP+FUS+DOX	AMP+FUS+TMP	AMP+DOX+TMP	CPR+FUS+DOX	CPR+FUS+TMP	CPR+DOX+TMP	FUS+DOX+TMP	AMP+CPR+FUS+DOX	AMP+CPR+FUS+TMP	AMP+CPR+DOX+TMP	AMP+FUS+DOX+TMP	CPR+FUS+DOX+TMP	AMP+CPR+FUS+DOX+TMP
	78.14815665	86.38437378	97.64552145	92.53710799	99.29110555	49.9575581	43.24026704	66.69572599	83.1402418	96.47172104	80.91886563	81.50576584	107.6228249	99.11277196	100.7593659	30.31957604	56.4179036	39.97338778	50.87522081	49.70061254	70.51320287	99.11277196	88.84201839	74.16951329	96.76517114	25.62114295	37.66087774	54.94964326	42.94661497	94.71102043	28.26401156
	72.2704949	98.12693389	108.8787585	89.19531545	98.2621836	64.11031356	52.58783529	81.94182093	86.47525501	98.59933392	70.48208423	75.31946052	105.5530623	90.43626143	97.35549679	33.24518323	48.09217983	36.30525123	33.88741972	32.03626747	67.43483189	104.0413822	97.99466188	78.3428207	101.3203581	24.17831507	43.52096713	85.87079713	34.75632792	78.64515672	33.8496411
	82.62638001	85.26818516	96.6752388	100.9103235	93.47278714	73.63935696	57.19542901	84.79566144	81.07689328	80.21675239	72.86921381	76.39603233	90.79720794	90.79720794	104.0092969	30.54425721	73.32945962	71.77997288	17.52856866	34.26302537	88.20453225	96.96914034	81.09845702	64.05216752	96.6752388	43.25004842	61.22409452	79.2175092	59.67460779	94.91182954	27.44528375
AMP1CPR2FUS2DOX2TMP2	AMP	CPR	FUS	DOX	TMP	AMP+CPR	AMP+FUS	AMP+DOX	AMP+TMP	CPR+FUS	CPR+DOX	CPR+TMP	FUS+DOX	FUS+TMP	DOX+TMP	AMP+CPR+FUS	AMP+CPR+DOX	AMP+CPR+TMP	AMP+FUS+DOX	AMP+FUS+TMP	AMP+DOX+TMP	CPR+FUS+DOX	CPR+FUS+TMP	CPR+DOX+TMP	FUS+DOX+TMP	AMP+CPR+FUS+DOX	AMP+CPR+FUS+TMP	AMP+CPR+DOX+TMP	AMP+FUS+DOX+TMP	CPR+FUS+DOX+TMP	AMP+CPR+FUS+DOX+TMP
	78.14815665	86.38437378	97.64552145	92.53710799	94.59267246	49.9575581	43.24026704	66.69572599	81.96563353	96.47172104	80.91886563	73.58261308	107.6228249	92.65686971	103.6958866	30.31957604	56.4179036	55.83059946	50.87522081	57.04191424	86.95771869	99.11277196	92.06996951	83.55991655	99.11277196	25.62114295	47.64504806	49.9575581	57.04191424	102.3407231	34.43070499
	72.2704949	98.12693389	108.8787585	89.19531545	92.51983377	64.11031356	52.58783529	81.94182093	78.61730261	98.59933392	70.48208423	78.94749274	105.5530623	89.52925337	107.0268228	33.24518323	48.09217983	33.58519078	33.88741972	52.89006422	63.80808462	104.0413822	107.3670784	81.6685169	113.4137988	24.17831507	49.5655459	75.59501322	59.53910087	96.48298179	48.96108802
	82.62638001	85.26818516	96.6752388	100.9103235	99.36083672	73.63935696	57.19542901	84.79566144	75.80863839	80.21675239	72.86921381	72.63041881	90.79720794	83.44966936	89.13422429	30.54425721	73.32945962	75.80863839	17.52856866	60.91419717	86.96494286	96.96914034	87.85819251	84.09257899	102.2593681	43.25004842	50.99748208	70.23048615	76.71896184	70.22409993	42.63025373
AMP1CPR2FUS2DOX2TMP3	AMP	CPR	FUS	DOX	TMP	AMP+CPR	AMP+FUS	AMP+DOX	AMP+TMP	CPR+FUS	CPR+DOX	CPR+TMP	FUS+DOX	FUS+TMP	DOX+TMP	AMP+CPR+FUS	AMP+CPR+DOX	AMP+CPR+TMP	AMP+FUS+DOX	AMP+FUS+TMP	AMP+DOX+TMP	CPR+FUS+DOX	CPR+FUS+TMP	CPR+DOX+TMP	FUS+DOX+TMP	AMP+CPR+FUS+DOX	AMP+CPR+FUS+TMP	AMP+CPR+DOX+TMP	AMP+FUS+DOX+TMP	CPR+FUS+DOX+TMP	AMP+CPR+FUS+DOX+TMP
	78.14815665	86.38437378	97.64552145	92.53710799	98.11649728	49.9575581	43.24026704	66.69572599	81.96563353	96.47172104	80.91886563	76.5171141	107.6228249	101.1669227	103.9895386	30.31957604	56.4179036	66.40207392	50.87522081	58.51017459	72.86241942	99.11277196	92.65686971	96.17827094	110.850776	25.62114295	52.93078529	67.87033426	45.58948359	107.916275	33.54974879
	72.2704949	98.12693389	108.8787585	89.19531545	99.16887042	64.11031356	52.58783529	81.94182093	71.96826596	98.59933392	70.48208423	76.52880459	105.5530623	94.06429364	98.56441254	33.24518323	48.09217983	40.8386853	33.88741972	45.33434076	65.62145825	104.0413822	109.1810946	96.48298179	109.1810946	24.17831507	52.28560635	81.03513411	46.84548546	85.29654912	50.47223272
	82.62638001	85.26818516	96.6752388	100.9103235	102.4598102	73.63935696	57.19542901	84.79566144	77.35812512	80.21675239	72.86921381	69.10360029	90.79720794	96.96914034	91.92330041	30.54425721	73.32945962	87.27484021	17.52856866	58.12512105	84.79566144	96.96914034	87.27038942	72.86921381	105.1983835	43.25004842	59.67460779	82.62638001	63.39337594	86.68258633	48.82820066
AMP1CPR2FUS2DOX3TMP1	AMP	CPR	FUS	DOX	TMP	AMP+CPR	AMP+FUS	AMP+DOX	AMP+TMP	CPR+FUS	CPR+DOX	CPR+TMP	FUS+DOX	FUS+TMP	DOX+TMP	AMP+CPR+FUS	AMP+CPR+DOX	AMP+CPR+TMP	AMP+FUS+DOX	AMP+FUS+TMP	AMP+DOX+TMP	CPR+FUS+DOX	CPR+FUS+TMP	CPR+DOX+TMP	FUS+DOX+TMP	AMP+CPR+FUS+DOX	AMP+CPR+FUS+TMP	AMP+CPR+DOX+TMP	AMP+FUS+DOX+TMP	CPR+FUS+DOX+TMP	AMP+CPR+FUS+DOX+TMP
	78.14815665	86.38437378	97.64552145	103.4022345	99.29110555	49.9575581	43.24026704	80.20372112	83.1402418	96.47172104	79.04812123	81.50576584	98.52587175	99.11277196	106.6324073	30.31957604	47.31468949	39.97338778	63.79591181	49.70061254	69.63224667	90.3092689	88.84201839	98.81932186	117.3066783	29.43861984	37.66087774	70.2195508	48.52600427	114.3721773	35.60531327
	72.2704949	98.12693389	108.8787585	94.93766528	98.2621836	64.11031356	52.58783529	88.89308651	86.47525501	98.59933392	75.62179654	75.31946052	100.715686	90.43626143	100.6800151	33.24518323	42.34983	36.30525123	50.47223272	32.03626747	60.78579524	110.0881026	97.99466188	89.22691735	103.7390462	36.87193049	43.52096713	85.56856819	49.5655459	104.0413822	49.60332452
	82.62638001	85.26818516	96.6752388	112.6864226	93.47278714	73.63935696	57.19542901	83.55607205	81.07689328	80.21675239	75.5143277	76.39603233	95.49963262	90.79720794	108.6577571	30.54425721	82.00658532	71.77997288	23.41661825	34.26302537	83.8659694	97.85084497	81.09845702	85.50698016	92.26671565	44.48963781	61.22409452	84.48576409	66.49234941	93.73622337	43.57931435
AMP1CPR2FUS2DOX3TMP2	AMP	CPR	FUS	DOX	TMP	AMP+CPR	AMP+FUS	AMP+DOX	AMP+TMP	CPR+FUS	CPR+DOX	CPR+TMP	FUS+DOX	FUS+TMP	DOX+TMP	AMP+CPR+FUS	AMP+CPR+DOX	AMP+CPR+TMP	AMP+FUS+DOX	AMP+FUS+TMP	AMP+DOX+TMP	CPR+FUS+DOX	CPR+FUS+TMP	CPR+DOX+TMP	FUS+DOX+TMP	AMP+CPR+FUS+DOX	AMP+CPR+FUS+TMP	AMP+CPR+DOX+TMP	AMP+FUS+DOX+TMP	CPR+FUS+DOX+TMP	AMP+CPR+FUS+DOX+TMP
	78.14815665	86.38437378	97.64552145	103.4022345	94.59267246	49.9575581	43.24026704	80.20372112	81.96563353	96.47172104	79.04812123	73.58261308	98.52587175	92.65686971	102.5212783	30.31957604	47.31468949	55.83059946	63.79591181	57.04191424	67.87033426	90.3092689	92.06996951	86.78786767	117.8935785	29.43861984	47.64504806	86.95771869	47.64504806	101.7538229	0.330358577
	72.2704949	98.12693389	108.8787585	94.93766528	92.51983377	64.11031356	52.58783529	88.89308651	78.61730261	98.59933392	75.62179654	78.94749274	100.715686	89.52925337	92.21760484	33.24518323	42.34983	33.58519078	50.47223272	52.89006422	57.46127692	110.0881026	107.3670784	78.64515672	106.4600704	36.87193049	49.5655459	76.50170004	53.4945221	91.04093346	4.835663015
	82.62638001	85.26818516	96.6752388	112.6864226	99.36083672	73.63935696	57.19542901	83.55607205	75.80863839	80.21675239	75.5143277	72.63041881	95.49963262	83.44966936	101.5301181	30.54425721	82.00658532	75.80863839	23.41661825	60.91419717	77.66802247	97.85084497	87.85819251	89.97060985	87.85819251	44.48963781	50.99748208	83.2461747	69.59132287	102.8471712	20.64691071
AMP1CPR2FUS2DOX3TMP3	AMP	CPR	FUS	DOX	TMP	AMP+CPR	AMP+FUS	AMP+DOX	AMP+TMP	CPR+FUS	CPR+DOX	CPR+TMP	FUS+DOX	FUS+TMP	DOX+TMP	AMP+CPR+FUS	AMP+CPR+DOX	AMP+CPR+TMP	AMP+FUS+DOX	AMP+FUS+TMP	AMP+DOX+TMP	CPR+FUS+DOX	CPR+FUS+TMP	CPR+DOX+TMP	FUS+DOX+TMP	AMP+CPR+FUS+DOX	AMP+CPR+FUS+TMP	AMP+CPR+DOX+TMP	AMP+FUS+DOX+TMP	CPR+FUS+DOX+TMP	AMP+CPR+FUS+DOX+TMP
	78.14815665	86.38437378	97.64552145	103.4022345	98.11649728	49.9575581	43.24026704	80.20372112	81.96563353	96.47172104	79.04812123	76.5171141	98.52587175	101.1669227	91.65615178	30.31957604	47.31468949	66.40207392	63.79591181	58.51017459	78.14815665	90.3092689	92.65686971	86.09092368	115.2525276	29.43861984	52.93078529	76.09259217	40.30374636	109.0900754	42.32260433
	72.2704949	98.12693389	108.8787585	94.93766528	99.16887042	64.11031356	52.58783529	88.89308651	71.96826596	98.59933392	75.62179654	76.52880459	100.715686	94.06429364	94.03097847	33.24518323	42.34983	40.8386853	50.47223272	45.33434076	84.35965244	110.0881026	109.1810946	100.8479581	107.0647424	36.87193049	52.28560635	67.13260295	58.93464299	90.73859744	46.58103513
	82.62638001	85.26818516	96.6752388	112.6864226	102.4598102	73.63935696	57.19542901	83.55607205	77.35812512	80.21675239	75.5143277	69.10360029	95.49963262	96.96914034	101.5301181	30.54425721	82.00658532	87.27484021	23.41661825	58.12512105	81.38679063	97.85084497	87.27038942	96.43644379	94.03012491	44.48963781	59.67460779	71.16017819	61.53399187	101.671565	45.72922719
AMP1CPR2FUS3DOX1TMP1	AMP	CPR	FUS	DOX	TMP	AMP+CPR	AMP+FUS	AMP+DOX	AMP+TMP	CPR+FUS	CPR+DOX	CPR+TMP	FUS+DOX	FUS+TMP	DOX+TMP	AMP+CPR+FUS	AMP+CPR+DOX	AMP+CPR+TMP	AMP+FUS+DOX	AMP+FUS+TMP	AMP+DOX+TMP	CPR+FUS+DOX	CPR+FUS+TMP	CPR+DOX+TMP	FUS+DOX+TMP	AMP+CPR+FUS+DOX	AMP+CPR+FUS+TMP	AMP+CPR+DOX+TMP	AMP+FUS+DOX+TMP	CPR+FUS+DOX+TMP	AMP+CPR+FUS+DOX+TMP
	78.14815665	86.38437378	95.90014577	88.42597903	99.29110555	49.9575581	63.50225974	66.98937805	83.1402418	92.06996951	81.50576584	81.50576584	77.53279883	72.8680758	96.94188901	51.42581844	59.06077221	39.97338778	59.97843493	69.3385946	63.7592053	92.65686971	90.8961691	78.27781471	76.94970845	45.58948359	44.12122324	51.42581844	45.55277708	94.41757032	48.19564569
	72.2704949	98.12693389	92.29679476	90.40423121	98.2621836	64.11031356	74.95277673	65.92368719	86.47525501	104.9483903	89.52925337	75.31946052	78.69665309	54.89640517	92.82206271	45.06989044	46.2788062	36.30525123	36.87193049	61.39025312	74.99055535	97.38998984	107.0647424	80.59144484	83.51336993	37.21193804	45.97657726	59.87910842	52.6256139	100.111014	63.80808462
	82.62638001	85.26818516	84.78536497	95.95196591	93.47278714	73.63935696	49.73852411	75.80863839	81.07689328	79.04114622	89.08890522	76.39603233	94.63865242	89.42220612	108.9676545	47.56924269	72.70966492	71.77997288	63.06411001	75.1888437	82.93627736	92.26671565	86.68258633	73.51212344	93.47944213	59.69397637	44.17974046	78.90761185	63.10284718	97.26304188	72.70966492
AMP1CPR2FUS3DOX1TMP2	AMP	CPR	FUS	DOX	TMP	AMP+CPR	AMP+FUS	AMP+DOX	AMP+TMP	CPR+FUS	CPR+DOX	CPR+TMP	FUS+DOX	FUS+TMP	DOX+TMP	AMP+CPR+FUS	AMP+CPR+DOX	AMP+CPR+TMP	AMP+FUS+DOX	AMP+FUS+TMP	AMP+DOX+TMP	CPR+FUS+DOX	CPR+FUS+TMP	CPR+DOX+TMP	FUS+DOX+TMP	AMP+CPR+FUS+DOX	AMP+CPR+FUS+TMP	AMP+CPR+DOX+TMP	AMP+FUS+DOX+TMP	CPR+FUS+DOX+TMP	AMP+CPR+FUS+DOX+TMP
	78.14815665	86.38437378	95.90014577	88.42597903	94.59267246	49.9575581	63.50225974	66.98937805	81.96563353	92.06996951	81.50576584	73.58261308	77.53279883	57.99927114	99.58475762	51.42581844	59.06077221	55.83059946	59.97843493	60.56573906	62.29094496	92.65686971	91.4830693	80.33196543	87.44533528	45.58948359	62.58459703	64.64016151	60.23538049	95.00447053	52.30677464
	72.2704949	98.12693389	92.29679476	90.40423121	92.51983377	64.11031356	74.95277673	65.92368719	78.61730261	104.9483903	89.52925337	78.94749274	78.69665309	56.59642288	96.14658104	45.06989044	46.2788062	33.58519078	36.87193049	53.53230072	71.66603702	97.38998984	107.0647424	68.49800411	71.0465734	37.21193804	51.11446921	68.3415187	64.71477144	91.04093346	49.60332452
	82.62638001	85.26818516	84.78536497	95.95196591	99.36083672	73.63935696	49.73852411	75.80863839	75.80863839	79.04114622	89.08890522	72.63041881	94.63865242	90.87121898	100.6004261	47.56924269	72.70966492	75.80863839	63.06411001	64.03253922	76.42843308	92.26671565	98.7325496	69.39750184	90.87121898	59.69397637	61.8632578	88.20453225	56.55626574	93.73622337	52.21770289
AMP1CPR2FUS3DOX1TMP3	AMP	CPR	FUS	DOX	TMP	AMP+CPR	AMP+FUS	AMP+DOX	AMP+TMP	CPR+FUS	CPR+DOX	CPR+TMP	FUS+DOX	FUS+TMP	DOX+TMP	AMP+CPR+FUS	AMP+CPR+DOX	AMP+CPR+TMP	AMP+FUS+DOX	AMP+FUS+TMP	AMP+DOX+TMP	CPR+FUS+DOX	CPR+FUS+TMP	CPR+DOX+TMP	FUS+DOX+TMP	AMP+CPR+FUS+DOX	AMP+CPR+FUS+TMP	AMP+CPR+DOX+TMP	AMP+FUS+DOX+TMP	CPR+FUS+DOX+TMP	AMP+CPR+FUS+DOX+TMP
	78.14815665	86.38437378	95.90014577	88.42597903	98.11649728	49.9575581	63.50225974	66.98937805	81.96563353	92.06996951	81.50576584	76.5171141	77.53279883	66.74562682	99.29110555	51.42581844	59.06077221	66.40207392	59.97843493	59.06077221	71.98146321	92.65686971	94.12412022	69.76776176	83.65524781	45.58948359	56.4179036	61.11633669	39.67973571	79.45161512	41.18470256
	72.2704949	98.12693389	92.29679476	90.40423121	99.16887042	64.11031356	74.95277673	65.92368719	71.96826596	104.9483903	89.52925337	76.52880459	78.69665309	65.09651142	99.16887042	45.06989044	46.2788062	40.8386853	36.87193049	55.64790329	69.55043445	97.38998984	100.715686	76.96341262	73.59659996	37.21193804	42.04760106	74.99055535	46.58103513	82.57552495	57.15904798
	82.62638001	85.26818516	84.78536497	95.95196591	102.4598102	73.63935696	49.73852411	75.80863839	77.35812512	79.04114622	89.08890522	69.10360029	94.63865242	81.88733925	104.938989	47.56924269	72.70966492	87.27484021	63.06411001	53.80592679	79.83730389	92.26671565	96.38133725	77.92064658	75.07697881	59.69397637	52.87623475	82.00658532	44.50900639	95.79353417	67.44141003
AMP1CPR2FUS3DOX2TMP1	AMP	CPR	FUS	DOX	TMP	AMP+CPR	AMP+FUS	AMP+DOX	AMP+TMP	CPR+FUS	CPR+DOX	CPR+TMP	FUS+DOX	FUS+TMP	DOX+TMP	AMP+CPR+FUS	AMP+CPR+DOX	AMP+CPR+TMP	AMP+FUS+DOX	AMP+FUS+TMP	AMP+DOX+TMP	CPR+FUS+DOX	CPR+FUS+TMP	CPR+DOX+TMP	FUS+DOX+TMP	AMP+CPR+FUS+DOX	AMP+CPR+FUS+TMP	AMP+CPR+DOX+TMP	AMP+FUS+DOX+TMP	CPR+FUS+DOX+TMP	AMP+CPR+FUS+DOX+TMP
	78.14815665	86.38437378	95.90014577	92.53710799	99.29110555	49.9575581	63.50225974	66.69572599	83.1402418	92.06996951	80.91886563	81.50576584	91.52696793	72.8680758	100.7593659	51.42581844	56.4179036	39.97338778	59.09747872	69.3385946	70.51320287	98.23242165	90.8961691	74.16951329	64.59548105	43.24026704	44.12122324	54.94964326	58.17981601	92.65686971	61.70364083
	72.2704949	98.12693389	92.29679476	89.19531545	98.2621836	64.11031356	74.95277673	81.94182093	86.47525501	104.9483903	70.48208423	75.31946052	68.49654684	54.89640517	97.35549679	45.06989044	48.09217983	36.30525123	62.86361919	61.39025312	67.43483189	82.57552495	107.0647424	78.3428207	73.0299274	42.00982244	45.97657726	85.87079713	59.27465055	87.71523726	70.45712127
	82.62638001	85.26818516	84.78536497	100.9103235	93.47278714	73.63935696	49.73852411	84.79566144	81.07689328	79.04114622	72.86921381	76.39603233	73.62796595	89.42220612	104.0092969	47.56924269	73.32945962	71.77997288	48.84756924	75.1888437	88.20453225	94.61792799	86.68258633	64.05216752	101.8837167	42.64962231	44.17974046	79.2175092	70.5403835	84.62527553	56.59500291
AMP1CPR2FUS3DOX2TMP2	AMP	CPR	FUS	DOX	TMP	AMP+CPR	AMP+FUS	AMP+DOX	AMP+TMP	CPR+FUS	CPR+DOX	CPR+TMP	FUS+DOX	FUS+TMP	DOX+TMP	AMP+CPR+FUS	AMP+CPR+DOX	AMP+CPR+TMP	AMP+FUS+DOX	AMP+FUS+TMP	AMP+DOX+TMP	CPR+FUS+DOX	CPR+FUS+TMP	CPR+DOX+TMP	FUS+DOX+TMP	AMP+CPR+FUS+DOX	AMP+CPR+FUS+TMP	AMP+CPR+DOX+TMP	AMP+FUS+DOX+TMP	CPR+FUS+DOX+TMP	AMP+CPR+FUS+DOX+TMP
	78.14815665	86.38437378	95.90014577	92.53710799	94.59267246	49.9575581	63.50225974	66.69572599	81.96563353	92.06996951	80.91886563	73.58261308	91.52696793	57.99927114	103.6958866	51.42581844	56.4179036	55.83059946	59.09747872	60.56573906	86.95771869	98.23242165	91.4830693	83.55991655	81.61443149	43.24026704	62.58459703	49.9575581	81.96563353	98.23242165	63.46555324
	72.2704949	98.12693389	92.29679476	89.19531545	92.51983377	64.11031356	74.95277673	81.94182093	78.61730261	104.9483903	70.48208423	78.94749274	68.49654684	56.59642288	107.0268228	45.06989044	48.09217983	33.58519078	62.86361919	53.53230072	63.80808462	82.57552495	107.0647424	81.6685169	98.24685674	42.00982244	51.11446921	75.59501322	70.45712127	93.76195763	61.08802418
	82.62638001	85.26818516	84.78536497	100.9103235	99.36083672	73.63935696	49.73852411	84.79566144	75.80863839	79.04114622	72.86921381	72.63041881	73.62796595	90.87121898	89.13422429	47.56924269	73.32945962	75.80863839	48.84756924	64.03253922	86.96494286	94.61792799	98.7325496	84.09257899	99.85509871	42.64962231	61.8632578	70.23048615	56.86616308	61.99485672	63.37400736
AMP1CPR2FUS3DOX2TMP3	AMP	CPR	FUS	DOX	TMP	AMP+CPR	AMP+FUS	AMP+DOX	AMP+TMP	CPR+FUS	CPR+DOX	CPR+TMP	FUS+DOX	FUS+TMP	DOX+TMP	AMP+CPR+FUS	AMP+CPR+DOX	AMP+CPR+TMP	AMP+FUS+DOX	AMP+FUS+TMP	AMP+DOX+TMP	CPR+FUS+DOX	CPR+FUS+TMP	CPR+DOX+TMP	FUS+DOX+TMP	AMP+CPR+FUS+DOX	AMP+CPR+FUS+TMP	AMP+CPR+DOX+TMP	AMP+FUS+DOX+TMP	CPR+FUS+DOX+TMP	AMP+CPR+FUS+DOX+TMP
	78.14815665	86.38437378	95.90014577	92.53710799	98.11649728	49.9575581	63.50225974	66.69572599	81.96563353	92.06996951	80.91886563	76.5171141	91.52696793	66.74562682	103.9895386	51.42581844	56.4179036	66.40207392	59.09747872	59.06077221	72.86241942	98.23242165	94.12412022	96.17827094	108.4365889	43.24026704	56.4179036	67.87033426	56.45461011	108.5031752	47.60834155
	72.2704949	98.12693389	92.29679476	89.19531545	99.16887042	64.11031356	74.95277673	81.94182093	71.96826596	104.9483903	70.48208423	76.52880459	68.49654684	65.09651142	98.56441254	45.06989044	48.09217983	40.8386853	62.86361919	55.64790329	65.62145825	82.57552495	100.715686	96.48298179	88.61342306	42.00982244	42.04760106	81.03513411	51.37891953	99.20400595	61.99471099
	82.62638001	85.26818516	84.78536497	100.9103235	102.4598102	73.63935696	49.73852411	84.79566144	77.35812512	79.04114622	72.86921381	69.10360029	73.62796595	81.88733925	91.92330041	47.56924269	73.32945962	87.27484021	48.84756924	53.80592679	84.79566144	94.61792799	96.38133725	72.86921381	99.56529614	42.64962231	52.87623475	82.62638001	58.4350184	85.21307862	56.59500291
AMP1CPR2FUS3DOX3TMP1	AMP	CPR	FUS	DOX	TMP	AMP+CPR	AMP+FUS	AMP+DOX	AMP+TMP	CPR+FUS	CPR+DOX	CPR+TMP	FUS+DOX	FUS+TMP	DOX+TMP	AMP+CPR+FUS	AMP+CPR+DOX	AMP+CPR+TMP	AMP+FUS+DOX	AMP+FUS+TMP	AMP+DOX+TMP	CPR+FUS+DOX	CPR+FUS+TMP	CPR+DOX+TMP	FUS+DOX+TMP	AMP+CPR+FUS+DOX	AMP+CPR+FUS+TMP	AMP+CPR+DOX+TMP	AMP+FUS+DOX+TMP	CPR+FUS+DOX+TMP	AMP+CPR+FUS+DOX+TMP
	78.14815665	86.38437378	95.90014577	103.4022345	99.29110555	49.9575581	63.50225974	80.20372112	83.1402418	92.06996951	79.04812123	81.50576584	48.66982507	72.8680758	106.6324073	51.42581844	47.31468949	39.97338778	57.00520774	69.3385946	69.63224667	91.77651941	90.8961691	98.81932186	102.3141399	49.37025396	44.12122324	70.2195508	54.94964326	112.3180265	59.35442428
	72.2704949	98.12693389	92.29679476	94.93766528	98.2621836	64.11031356	74.95277673	88.89308651	86.47525501	104.9483903	75.62179654	75.31946052	61.41313972	54.89640517	100.6800151	45.06989044	42.34983	36.30525123	58.33018512	61.39025312	60.78579524	106.1577344	107.0647424	89.22691735	85.78006021	48.09217983	45.97657726	85.56856819	59.57687949	108.5764225	63.50585569
	82.62638001	85.26818516	84.78536497	112.6864226	93.47278714	73.63935696	49.73852411	83.55607205	81.07689328	79.04114622	75.5143277	76.39603233	68.41151965	89.42220612	108.6577571	47.56924269	82.00658532	71.77997288	53.76718962	75.1888437	83.8659694	99.02645114	86.68258633	85.50698016	101.5939141	56.28510556	44.17974046	84.48576409	76.42843308	89.91550331	45.39996126
AMP1CPR2FUS3DOX3TMP2	AMP	CPR	FUS	DOX	TMP	AMP+CPR	AMP+FUS	AMP+DOX	AMP+TMP	CPR+FUS	CPR+DOX	CPR+TMP	FUS+DOX	FUS+TMP	DOX+TMP	AMP+CPR+FUS	AMP+CPR+DOX	AMP+CPR+TMP	AMP+FUS+DOX	AMP+FUS+TMP	AMP+DOX+TMP	CPR+FUS+DOX	CPR+FUS+TMP	CPR+DOX+TMP	FUS+DOX+TMP	AMP+CPR+FUS+DOX	AMP+CPR+FUS+TMP	AMP+CPR+DOX+TMP	AMP+FUS+DOX+TMP	CPR+FUS+DOX+TMP	AMP+CPR+FUS+DOX+TMP
	78.14815665	86.38437378	95.90014577	103.4022345	94.59267246	49.9575581	63.50225974	80.20372112	81.96563353	92.06996951	79.04812123	73.58261308	48.66982507	57.99927114	102.5212783	51.42581844	47.31468949	55.83059946	57.00520774	60.56573906	67.87033426	91.77651941	91.4830693	86.78786767	98.52405248	49.37025396	62.58459703	86.95771869	62.58459703	103.8079736	71.10050701
	72.2704949	98.12693389	92.29679476	94.93766528	92.51983377	64.11031356	74.95277673	88.89308651	78.61730261	104.9483903	75.62179654	78.94749274	61.41313972	56.59642288	92.21760484	45.06989044	42.34983	33.58519078	58.33018512	53.53230072	57.46127692	106.1577344	107.0647424	78.64515672	88.89675934	48.09217983	51.11446921	76.50170004	55.34567435	88.01757328	71.36380808
	82.62638001	85.26818516	84.78536497	112.6864226	99.36083672	73.63935696	49.73852411	83.55607205	75.80863839	79.04114622	75.5143277	72.63041881	68.41151965	90.87121898	101.5301181	47.56924269	82.00658532	75.80863839	53.76718962	64.03253922	77.66802247	99.02645114	98.7325496	89.97060985	98.69588843	56.28510556	61.8632578	83.2461747	59.36471044	98.14474651	55.6265737
AMP1CPR2FUS3DOX3TMP3	AMP	CPR	FUS	DOX	TMP	AMP+CPR	AMP+FUS	AMP+DOX	AMP+TMP	CPR+FUS	CPR+DOX	CPR+TMP	FUS+DOX	FUS+TMP	DOX+TMP	AMP+CPR+FUS	AMP+CPR+DOX	AMP+CPR+TMP	AMP+FUS+DOX	AMP+FUS+TMP	AMP+DOX+TMP	CPR+FUS+DOX	CPR+FUS+TMP	CPR+DOX+TMP	FUS+DOX+TMP	AMP+CPR+FUS+DOX	AMP+CPR+FUS+TMP	AMP+CPR+DOX+TMP	AMP+FUS+DOX+TMP	CPR+FUS+DOX+TMP	AMP+CPR+FUS+DOX+TMP
	78.14815665	86.38437378	95.90014577	103.4022345	98.11649728	49.9575581	63.50225974	80.20372112	81.96563353	92.06996951	79.04812123	76.5171141	48.66982507	66.74562682	91.65615178	51.42581844	47.31468949	66.40207392	57.00520774	59.06077221	78.14815665	91.77651941	94.12412022	86.09092368	96.48323615	49.37025396	56.4179036	76.09259217	77.56085251	109.6769756	42.90990846
	72.2704949	98.12693389	92.29679476	94.93766528	99.16887042	64.11031356	74.95277673	88.89308651	71.96826596	104.9483903	75.62179654	76.52880459	61.41313972	65.09651142	94.03097847	45.06989044	42.34983	40.8386853	58.33018512	55.64790329	84.35965244	106.1577344	100.715686	100.8479581	85.78006021	48.09217983	42.04760106	67.13260295	68.3415187	99.20400595	62.29693993
	82.62638001	85.26818516	84.78536497	112.6864226	102.4598102	73.63935696	49.73852411	83.55607205	77.35812512	79.04114622	75.5143277	69.10360029	68.41151965	81.88733925	101.5301181	47.56924269	82.00658532	87.27484021	53.76718962	53.80592679	81.38679063	99.02645114	96.38133725	96.43644379	94.34884985	56.28510556	52.87623475	71.16017819	76.42843308	97.26304188	60.2750339
AMP1CPR3FUS1DOX1TMP1	AMP	CPR	FUS	DOX	TMP	AMP+CPR	AMP+FUS	AMP+DOX	AMP+TMP	CPR+FUS	CPR+DOX	CPR+TMP	FUS+DOX	FUS+TMP	DOX+TMP	AMP+CPR+FUS	AMP+CPR+DOX	AMP+CPR+TMP	AMP+FUS+DOX	AMP+FUS+TMP	AMP+DOX+TMP	CPR+FUS+DOX	CPR+FUS+TMP	CPR+DOX+TMP	FUS+DOX+TMP	AMP+CPR+FUS+DOX	AMP+CPR+FUS+TMP	AMP+CPR+DOX+TMP	AMP+FUS+DOX+TMP	CPR+FUS+DOX+TMP	AMP+CPR+FUS+DOX+TMP
	78.14815665	86.75291545	72.56876735	88.42597903	99.29110555	74.9179839	39.12913809	66.98937805	83.1402418	76.65816327	96.6654519	65.47011662	74.33067976	84.90215421	96.94188901	44.12122324	96.0609328	47.31468949	15.04966849	54.98634977	63.7592053	96.77478134	56.35932945	78.88119534	75.7989401	16.8115809	45.29583152	66.69572599	26.20844709	79.28206997	25.62114295
	72.2704949	84.36337879	80.73290518	90.40423121	98.2621836	70.75935021	6.384586324	65.92368719	86.47525501	68.21321055	80.59146449	61.8912697	65.01700038	76.80392898	92.82206271	42.61428032	81.94182093	40.8386853	10.9180204	44.12542501	74.99055535	97.39684788	81.81335222	67.5579954	98.56441254	20.85379675	33.8496411	52.6256139	32.33849641	57.30476359	30.22289384
	82.62638001	88.11809455	93.47278714	95.95196591	93.47278714	79.52740655	9.18070889	75.80863839	81.07689328	84.78536497	93.33454084	90.14671255	63.10284718	101.2202208	108.9676545	42.01045904	88.5144296	86.96494286	7.321324811	44.17974046	82.93627736	83.33635211	119.8514762	104.9266437	86.34514817	28.08444703	42.32035638	69.61069146	39.84117761	87.68339069	40.15107496
AMP1CPR3FUS1DOX1TMP2	AMP	CPR	FUS	DOX	TMP	AMP+CPR	AMP+FUS	AMP+DOX	AMP+TMP	CPR+FUS	CPR+DOX	CPR+TMP	FUS+DOX	FUS+TMP	DOX+TMP	AMP+CPR+FUS	AMP+CPR+DOX	AMP+CPR+TMP	AMP+FUS+DOX	AMP+FUS+TMP	AMP+DOX+TMP	CPR+FUS+DOX	CPR+FUS+TMP	CPR+DOX+TMP	FUS+DOX+TMP	AMP+CPR+FUS+DOX	AMP+CPR+FUS+TMP	AMP+CPR+DOX+TMP	AMP+FUS+DOX+TMP	CPR+FUS+DOX+TMP	AMP+CPR+FUS+DOX+TMP
	78.14815665	86.75291545	72.56876735	88.42597903	94.59267246	74.9179839	39.12913809	66.98937805	81.96563353	76.65816327	96.6654519	81.79664723	74.33067976	94.59267246	99.58475762	44.12122324	96.0609328	53.48138292	15.04966849	60.85939113	62.29094496	96.77478134	61.20626822	71.30102041	69.92589874	16.8115809	31.49418431	62.58459703	4.735139599	78.99052478	9.763931267
	72.2704949	84.36337879	80.73290518	90.40423121	92.51983377	70.75935021	6.384586324	65.92368719	78.61730261	68.21321055	80.59146449	66.70798654	65.01700038	102.7956177	96.14658104	42.61428032	81.94182093	67.73706082	10.9180204	55.61012467	71.66603702	97.39684788	74.44660882	63.87462369	77.40838685	20.85379675	44.42765395	46.58103513	0.944465433	61.97981229	4.231205138
	82.62638001	88.11809455	93.47278714	95.95196591	99.36083672	79.52740655	9.18070889	75.80863839	75.80863839	84.78536497	93.33454084	94.49375113	63.10284718	102.1499128	100.6004261	42.01045904	88.5144296	84.79566144	7.321324811	65.56265737	76.42843308	83.33635211	100.1449013	106.9552617	73.94925431	28.08444703	54.71625024	79.83730389	4.822777455	84.20575983	7.301956227
AMP1CPR3FUS1DOX1TMP3	AMP	CPR	FUS	DOX	TMP	AMP+CPR	AMP+FUS	AMP+DOX	AMP+TMP	CPR+FUS	CPR+DOX	CPR+TMP	FUS+DOX	FUS+TMP	DOX+TMP	AMP+CPR+FUS	AMP+CPR+DOX	AMP+CPR+TMP	AMP+FUS+DOX	AMP+FUS+TMP	AMP+DOX+TMP	CPR+FUS+DOX	CPR+FUS+TMP	CPR+DOX+TMP	FUS+DOX+TMP	AMP+CPR+FUS+DOX	AMP+CPR+FUS+TMP	AMP+CPR+DOX+TMP	AMP+FUS+DOX+TMP	CPR+FUS+DOX+TMP	AMP+CPR+FUS+DOX+TMP
	78.14815665	86.75291545	72.56876735	88.42597903	98.11649728	74.9179839	39.12913809	66.98937805	81.96563353	76.65816327	96.6654519	76.25728863	74.33067976	73.74337562	99.29110555	44.12122324	96.0609328	56.12425153	15.04966849	2.97322719	71.98146321	96.77478134	64.41326531	82.96282799	95.76728074	16.8115809	36.1926174	79.91006905	16.77487439	84.23833819	46.76409186
	72.2704949	84.36337879	80.73290518	90.40423121	99.16887042	70.75935021	6.384586324	65.92368719	71.96826596	68.21321055	80.59146449	61.32459713	65.01700038	98.56441254	99.16887042	42.61428032	81.94182093	57.46127692	10.9180204	2.417831507	69.55043445	97.39684788	63.96316628	67.84133168	111.258028	20.85379675	38.08084624	65.01700038	6.384586324	82.38002479	42.31205138
	82.62638001	88.11809455	93.47278714	95.95196591	102.4598102	79.52740655	9.18070889	75.80863839	77.35812512	84.78536497	93.33454084	106.9552617	63.10284718	94.09258183	104.938989	42.01045904	88.5144296	76.73833043	7.321324811	19.07805539	79.83730389	83.33635211	85.65477269	106.085854	95.33217122	28.08444703	57.81522371	90.68371102	30.54425721	87.39358812	53.16676351
AMP1CPR3FUS1DOX2TMP1	AMP	CPR	FUS	DOX	TMP	AMP+CPR	AMP+FUS	AMP+DOX	AMP+TMP	CPR+FUS	CPR+DOX	CPR+TMP	FUS+DOX	FUS+TMP	DOX+TMP	AMP+CPR+FUS	AMP+CPR+DOX	AMP+CPR+TMP	AMP+FUS+DOX	AMP+FUS+TMP	AMP+DOX+TMP	CPR+FUS+DOX	CPR+FUS+TMP	CPR+DOX+TMP	FUS+DOX+TMP	AMP+CPR+FUS+DOX	AMP+CPR+FUS+TMP	AMP+CPR+DOX+TMP	AMP+FUS+DOX+TMP	CPR+FUS+DOX+TMP	AMP+CPR+FUS+DOX+TMP
	78.14815665	86.75291545	72.56876735	92.53710799	99.29110555	74.9179839	39.12913809	66.69572599	83.1402418	76.65816327	79.17274052	65.47011662	82.2592856	84.90215421	100.7593659	44.12122324	91.94980385	47.31468949	10.64488747	54.98634977	70.51320287	73.45116618	56.35932945	68.31268222	96.0609328	30.90688018	45.29583152	64.34650944	12.99410402	62.95553936	2.128977494
	72.2704949	84.36337879	80.73290518	89.19531545	98.2621836	70.75935021	6.384586324	81.94182093	86.47525501	68.21321055	67.5579954	61.8912697	86.77748394	76.80392898	97.35549679	42.61428032	64.71477144	40.8386853	54.70343785	44.12542501	67.43483189	82.94669736	81.81335222	44.69629892	101.8889309	22.96939932	33.8496411	55.04344541	0.642236494	84.36337879	3.626747261
	82.62638001	88.11809455	93.47278714	100.9103235	93.47278714	79.52740655	9.18070889	84.79566144	81.07689328	84.78536497	91.30592284	90.14671255	100.6004261	101.2202208	104.0092969	42.01045904	89.44412163	86.96494286	9.490606237	44.17974046	88.20453225	83.33635211	119.8514762	101.1592103	73.32945962	55.95583963	42.32035638	83.2461747	6.682161534	69.86053251	11.33062173
AMP1CPR3FUS1DOX2TMP2	AMP	CPR	FUS	DOX	TMP	AMP+CPR	AMP+FUS	AMP+DOX	AMP+TMP	CPR+FUS	CPR+DOX	CPR+TMP	FUS+DOX	FUS+TMP	DOX+TMP	AMP+CPR+FUS	AMP+CPR+DOX	AMP+CPR+TMP	AMP+FUS+DOX	AMP+FUS+TMP	AMP+DOX+TMP	CPR+FUS+DOX	CPR+FUS+TMP	CPR+DOX+TMP	FUS+DOX+TMP	AMP+CPR+FUS+DOX	AMP+CPR+FUS+TMP	AMP+CPR+DOX+TMP	AMP+FUS+DOX+TMP	CPR+FUS+DOX+TMP	AMP+CPR+FUS+DOX+TMP
	78.14815665	86.75291545	72.56876735	92.53710799	94.59267246	74.9179839	39.12913809	66.69572599	81.96563353	76.65816327	79.17274052	81.79664723	82.2592856	94.59267246	103.6958866	44.12122324	91.94980385	53.48138292	10.64488747	60.85939113	86.95771869	73.45116618	61.20626822	82.6712828	70.80685494	30.90688018	31.49418431	42.32260433	11.81949574	69.47886297	1.541673358
	72.2704949	84.36337879	80.73290518	89.19531545	92.51983377	70.75935021	6.384586324	81.94182093	78.61730261	68.21321055	67.5579954	66.70798654	86.77748394	102.7956177	107.0268228	42.61428032	64.71477144	67.73706082	54.70343785	55.61012467	63.80808462	82.94669736	74.44660882	54.89640517	82.24404987	22.96939932	44.42765395	57.15904798	10.27578391	72.74659111	34.75632792
	82.62638001	88.11809455	93.47278714	100.9103235	99.36083672	79.52740655	9.18070889	84.79566144	75.80863839	84.78536497	91.30592284	94.49375113	100.6004261	102.1499128	89.13422429	42.01045904	89.44412163	84.79566144	9.490606237	65.56265737	86.96494286	83.33635211	100.1449013	103.4776309	84.79566144	55.95583963	54.71625024	77.35812512	6.99205888	77.2504981	32.71353864
AMP1CPR3FUS1DOX2TMP3	AMP	CPR	FUS	DOX	TMP	AMP+CPR	AMP+FUS	AMP+DOX	AMP+TMP	CPR+FUS	CPR+DOX	CPR+TMP	FUS+DOX	FUS+TMP	DOX+TMP	AMP+CPR+FUS	AMP+CPR+DOX	AMP+CPR+TMP	AMP+FUS+DOX	AMP+FUS+TMP	AMP+DOX+TMP	CPR+FUS+DOX	CPR+FUS+TMP	CPR+DOX+TMP	FUS+DOX+TMP	AMP+CPR+FUS+DOX	AMP+CPR+FUS+TMP	AMP+CPR+DOX+TMP	AMP+FUS+DOX+TMP	CPR+FUS+DOX+TMP	AMP+CPR+FUS+DOX+TMP
	78.14815665	86.75291545	72.56876735	92.53710799	98.11649728	74.9179839	39.12913809	66.69572599	81.96563353	76.65816327	79.17274052	76.25728863	82.2592856	73.74337562	103.9895386	44.12122324	91.94980385	56.12425153	10.64488747	2.97322719	72.86241942	73.45116618	64.41326531	80.63046647	79.32276492	30.90688018	36.1926174	66.10842185	30.61322811	82.78061224	1.24802129
	72.2704949	84.36337879	80.73290518	89.19531545	99.16887042	70.75935021	6.384586324	81.94182093	71.96826596	68.21321055	67.5579954	61.32459713	86.77748394	98.56441254	98.56441254	42.61428032	64.71477144	57.46127692	54.70343785	2.417831507	65.62145825	82.94669736	63.96316628	95.41349389	99.47109936	22.96939932	38.08084624	66.22591613	1.208915754	100.7968833	3.664525878
	82.62638001	88.11809455	93.47278714	100.9103235	102.4598102	79.52740655	9.18070889	84.79566144	77.35812512	84.78536497	91.30592284	106.9552617	100.6004261	94.09258183	91.92330041	42.01045904	89.44412163	76.73833043	9.490606237	19.07805539	84.79566144	83.33635211	85.65477269	97.68157942	94.71237653	55.95583963	57.81522371	82.93627736	6.682161534	96.37746785	6.372264187
AMP1CPR3FUS1DOX3TMP1	AMP	CPR	FUS	DOX	TMP	AMP+CPR	AMP+FUS	AMP+DOX	AMP+TMP	CPR+FUS	CPR+DOX	CPR+TMP	FUS+DOX	FUS+TMP	DOX+TMP	AMP+CPR+FUS	AMP+CPR+DOX	AMP+CPR+TMP	AMP+FUS+DOX	AMP+FUS+TMP	AMP+DOX+TMP	CPR+FUS+DOX	CPR+FUS+TMP	CPR+DOX+TMP	FUS+DOX+TMP	AMP+CPR+FUS+DOX	AMP+CPR+FUS+TMP	AMP+CPR+DOX+TMP	AMP+FUS+DOX+TMP	CPR+FUS+DOX+TMP	AMP+CPR+FUS+DOX+TMP
	78.14815665	86.75291545	72.56876735	103.4022345	99.29110555	74.9179839	39.12913809	80.20372112	83.1402418	76.65816327	68.96865889	65.47011662	87.8386749	84.90215421	106.6324073	44.12122324	63.7592053	47.31468949	23.2719264	54.98634977	69.63224667	60.04008746	56.35932945	87.44533528	81.96563353	44.12122324	45.29583152	60.23538049	36.1926174	73.74271137	25.32749088
	72.2704949	84.36337879	80.73290518	94.93766528	98.2621836	70.75935021	6.384586324	88.89308651	86.47525501	68.21321055	68.40800425	61.8912697	94.93766528	76.80392898	100.6800151	42.61428032	51.11446921	40.8386853	24.48054401	44.12542501	60.78579524	87.19674163	81.81335222	79.26332566	94.93766528	36.56970155	33.8496411	64.11031356	41.40536456	96.26350274	39.89421987
	82.62638001	88.11809455	93.47278714	112.6864226	93.47278714	79.52740655	9.18070889	83.55607205	81.07689328	84.78536497	93.04473827	90.14671255	106.1785783	101.2202208	108.6577571	42.01045904	95.95196591	86.96494286	14.44896378	44.17974046	83.8659694	92.89983699	119.8514762	99.71019743	80.76699593	31.80321518	42.32035638	68.37110207	52.85686616	93.7692447	26.20569436
AMP1CPR3FUS1DOX3TMP2	AMP	CPR	FUS	DOX	TMP	AMP+CPR	AMP+FUS	AMP+DOX	AMP+TMP	CPR+FUS	CPR+DOX	CPR+TMP	FUS+DOX	FUS+TMP	DOX+TMP	AMP+CPR+FUS	AMP+CPR+DOX	AMP+CPR+TMP	AMP+FUS+DOX	AMP+FUS+TMP	AMP+DOX+TMP	CPR+FUS+DOX	CPR+FUS+TMP	CPR+DOX+TMP	FUS+DOX+TMP	AMP+CPR+FUS+DOX	AMP+CPR+FUS+TMP	AMP+CPR+DOX+TMP	AMP+FUS+DOX+TMP	CPR+FUS+DOX+TMP	AMP+CPR+FUS+DOX+TMP
	78.14815665	86.75291545	72.56876735	103.4022345	94.59267246	74.9179839	39.12913809	80.20372112	81.96563353	76.65816327	68.96865889	81.79664723	87.8386749	94.59267246	102.5212783	44.12122324	63.7592053	53.48138292	23.2719264	60.85939113	67.87033426	60.04008746	61.20626822	87.33600583	98.11649728	44.12122324	31.49418431	58.47346808	0.954369222	100.856414	8.589322994
	72.2704949	84.36337879	80.73290518	94.93766528	92.51983377	70.75935021	6.384586324	88.89308651	78.61730261	68.21321055	68.40800425	66.70798654	94.93766528	102.7956177	92.21760484	42.61428032	51.11446921	67.73706082	24.48054401	55.61012467	57.46127692	87.19674163	74.44660882	78.97998937	89.79977333	36.56970155	44.42765395	70.45712127	44.12542501	90.88011333	9.709104647
	82.62638001	88.11809455	93.47278714	112.6864226	99.36083672	79.52740655	9.18070889	83.55607205	75.80863839	84.78536497	93.04473827	94.49375113	106.1785783	102.1499128	101.5301181	42.01045904	95.95196591	84.79566144	14.44896378	65.56265737	77.66802247	92.89983699	100.1449013	77.97500453	95.64206856	31.80321518	54.71625024	65.8919233	15.99845051	106.5205579	41.080767
AMP1CPR3FUS1DOX3TMP3	AMP	CPR	FUS	DOX	TMP	AMP+CPR	AMP+FUS	AMP+DOX	AMP+TMP	CPR+FUS	CPR+DOX	CPR+TMP	FUS+DOX	FUS+TMP	DOX+TMP	AMP+CPR+FUS	AMP+CPR+DOX	AMP+CPR+TMP	AMP+FUS+DOX	AMP+FUS+TMP	AMP+DOX+TMP	CPR+FUS+DOX	CPR+FUS+TMP	CPR+DOX+TMP	FUS+DOX+TMP	AMP+CPR+FUS+DOX	AMP+CPR+FUS+TMP	AMP+CPR+DOX+TMP	AMP+FUS+DOX+TMP	CPR+FUS+DOX+TMP	AMP+CPR+FUS+DOX+TMP
	78.14815665	86.75291545	72.56876735	103.4022345	98.11649728	74.9179839	39.12913809	80.20372112	81.96563353	76.65816327	68.96865889	76.25728863	87.8386749	73.74337562	91.65615178	44.12122324	63.7592053	56.12425153	23.2719264	2.97322719	78.14815665	60.04008746	64.41326531	101.9132653	76.97354837	44.12122324	36.1926174	90.18789144	16.22427677	89.19460641	1.24802129
	72.2704949	84.36337879	80.73290518	94.93766528	99.16887042	70.75935021	6.384586324	88.89308651	71.96826596	68.21321055	68.40800425	61.32459713	94.93766528	98.56441254	94.03097847	42.61428032	51.11446921	57.46127692	24.48054401	2.417831507	84.35965244	87.19674163	63.96316628	85.9748539	82.84850774	36.56970155	38.08084624	76.80392898	32.33849641	88.33008677	9.406875708
	82.62638001	88.11809455	93.47278714	112.6864226	102.4598102	79.52740655	9.18070889	83.55607205	77.35812512	84.78536497	93.04473827	106.9552617	106.1785783	94.09258183	101.5301181	42.01045904	95.95196591	76.73833043	14.44896378	19.07805539	81.38679063	92.89983699	85.65477269	86.66908169	80.45709859	31.80321518	57.81522371	72.08987023	22.17702886	100.4347039	8.870811544
AMP1CPR3FUS2DOX1TMP1	AMP	CPR	FUS	DOX	TMP	AMP+CPR	AMP+FUS	AMP+DOX	AMP+TMP	CPR+FUS	CPR+DOX	CPR+TMP	FUS+DOX	FUS+TMP	DOX+TMP	AMP+CPR+FUS	AMP+CPR+DOX	AMP+CPR+TMP	AMP+FUS+DOX	AMP+FUS+TMP	AMP+DOX+TMP	CPR+FUS+DOX	CPR+FUS+TMP	CPR+DOX+TMP	FUS+DOX+TMP	AMP+CPR+FUS+DOX	AMP+CPR+FUS+TMP	AMP+CPR+DOX+TMP	AMP+FUS+DOX+TMP	CPR+FUS+DOX+TMP	AMP+CPR+FUS+DOX+TMP
	78.14815665	86.75291545	97.64552145	88.42597903	99.29110555	74.9179839	43.24026704	66.98937805	83.1402418	80.15670554	96.6654519	65.47011662	90.8961691	99.11277196	96.94188901	55.5736539	96.0609328	47.31468949	21.21636193	49.70061254	63.7592053	83.07215743	79.86516035	78.88119534	97.64552145	38.54183395	23.2719264	66.69572599	39.97338778	80.44825073	27.67670743
	72.2704949	84.36337879	108.8787585	90.40423121	98.2621836	70.75935021	52.58783529	65.92368719	86.47525501	78.12998052	80.59146449	61.8912697	79.24982875	90.43626143	92.82206271	58.63241405	81.94182093	40.8386853	22.40272006	32.03626747	74.99055535	93.14680361	84.0800425	67.5579954	91.34326948	36.87193049	41.7075935	52.6256139	29.01397809	64.67150699	34.75632792
	82.62638001	88.11809455	96.6752388	95.95196591	93.47278714	79.52740655	57.19542901	75.80863839	81.07689328	78.69951096	93.33454084	90.14671255	56.39235856	90.79720794	108.9676545	52.54696882	88.5144296	86.96494286	31.16405191	34.26302537	82.93627736	91.7406267	97.82648071	104.9266437	101.0837619	46.98818516	38.29169088	69.61069146	49.13809801	98.11628328	46.96881658
AMP1CPR3FUS2DOX1TMP2	AMP	CPR	FUS	DOX	TMP	AMP+CPR	AMP+FUS	AMP+DOX	AMP+TMP	CPR+FUS	CPR+DOX	CPR+TMP	FUS+DOX	FUS+TMP	DOX+TMP	AMP+CPR+FUS	AMP+CPR+DOX	AMP+CPR+TMP	AMP+FUS+DOX	AMP+FUS+TMP	AMP+DOX+TMP	CPR+FUS+DOX	CPR+FUS+TMP	CPR+DOX+TMP	FUS+DOX+TMP	AMP+CPR+FUS+DOX	AMP+CPR+FUS+TMP	AMP+CPR+DOX+TMP	AMP+FUS+DOX+TMP	CPR+FUS+DOX+TMP	AMP+CPR+FUS+DOX+TMP
	78.14815665	86.75291545	97.64552145	88.42597903	94.59267246	74.9179839	43.24026704	66.98937805	81.96563353	80.15670554	96.6654519	81.79664723	90.8961691	92.65686971	99.58475762	55.5736539	96.0609328	53.48138292	21.21636193	57.04191424	62.29094496	83.07215743	82.78061224	71.30102041	101.7538229	38.54183395	44.12122324	62.58459703	43.4972126	85.98760933	30.61322811
	72.2704949	84.36337879	108.8787585	90.40423121	92.51983377	70.75935021	52.58783529	65.92368719	78.61730261	78.12998052	80.59146449	66.70798654	79.24982875	89.52925337	96.14658104	58.63241405	81.94182093	67.73706082	22.40272006	52.89006422	71.66603702	93.14680361	82.09668851	63.87462369	88.3199093	36.87193049	52.89006422	46.58103513	37.7786173	63.67983	30.82735172
	82.62638001	88.11809455	96.6752388	95.95196591	99.36083672	79.52740655	57.19542901	75.80863839	75.80863839	78.69951096	93.33454084	94.49375113	56.39235856	83.44966936	100.6004261	52.54696882	88.5144296	84.79566144	31.16405191	60.91419717	76.42843308	91.7406267	102.7531244	106.9552617	101.0837619	46.98818516	55.95583963	79.83730389	66.18245206	93.47944213	59.98450513
AMP1CPR3FUS2DOX1TMP3	AMP	CPR	FUS	DOX	TMP	AMP+CPR	AMP+FUS	AMP+DOX	AMP+TMP	CPR+FUS	CPR+DOX	CPR+TMP	FUS+DOX	FUS+TMP	DOX+TMP	AMP+CPR+FUS	AMP+CPR+DOX	AMP+CPR+TMP	AMP+FUS+DOX	AMP+FUS+TMP	AMP+DOX+TMP	CPR+FUS+DOX	CPR+FUS+TMP	CPR+DOX+TMP	FUS+DOX+TMP	AMP+CPR+FUS+DOX	AMP+CPR+FUS+TMP	AMP+CPR+DOX+TMP	AMP+FUS+DOX+TMP	CPR+FUS+DOX+TMP	AMP+CPR+FUS+DOX+TMP
	78.14815665	86.75291545	97.64552145	88.42597903	98.11649728	74.9179839	43.24026704	66.98937805	81.96563353	80.15670554	96.6654519	76.25728863	90.8961691	101.1669227	99.29110555	55.5736539	96.0609328	56.12425153	21.21636193	58.51017459	71.98146321	83.07215743	77.24125364	82.96282799	94.41757032	38.54183395	49.37025396	79.91006905	47.64504806	95.31705539	29.14496777
	72.2704949	84.36337879	108.8787585	90.40423121	99.16887042	70.75935021	52.58783529	65.92368719	71.96826596	78.12998052	80.59146449	61.32459713	79.24982875	94.06429364	99.16887042	58.63241405	81.94182093	57.46127692	22.40272006	45.33434076	69.55043445	93.14680361	66.51319285	67.84133168	73.50544441	36.87193049	44.42765395	65.01700038	64.07253495	75.01328139	24.48054401
	82.62638001	88.11809455	96.6752388	95.95196591	102.4598102	79.52740655	57.19542901	75.80863839	77.35812512	78.69951096	93.33454084	106.9552617	56.39235856	96.96914034	104.938989	52.54696882	88.5144296	76.73833043	31.16405191	58.12512105	79.83730389	91.7406267	99.56529614	106.085854	87.85819251	46.98818516	51.30737943	90.68371102	64.94286268	93.47944213	49.7578927
AMP1CPR3FUS2DOX2TMP1	AMP	CPR	FUS	DOX	TMP	AMP+CPR	AMP+FUS	AMP+DOX	AMP+TMP	CPR+FUS	CPR+DOX	CPR+TMP	FUS+DOX	FUS+TMP	DOX+TMP	AMP+CPR+FUS	AMP+CPR+DOX	AMP+CPR+TMP	AMP+FUS+DOX	AMP+FUS+TMP	AMP+DOX+TMP	CPR+FUS+DOX	CPR+FUS+TMP	CPR+DOX+TMP	FUS+DOX+TMP	AMP+CPR+FUS+DOX	AMP+CPR+FUS+TMP	AMP+CPR+DOX+TMP	AMP+FUS+DOX+TMP	CPR+FUS+DOX+TMP	AMP+CPR+FUS+DOX+TMP
	78.14815665	86.75291545	97.64552145	92.53710799	99.29110555	74.9179839	43.24026704	66.69572599	83.1402418	80.15670554	79.17274052	65.47011662	107.6228249	99.11277196	100.7593659	55.5736539	91.94980385	47.31468949	50.87522081	49.70061254	70.51320287	76.07507289	79.86516035	68.31268222	96.76517114	39.12913809	23.2719264	64.34650944	42.94661497	69.36953353	34.13705293
	72.2704949	84.36337879	108.8787585	89.19531545	98.2621836	70.75935021	52.58783529	81.94182093	86.47525501	78.12998052	67.5579954	61.8912697	105.5530623	90.43626143	97.35549679	58.63241405	64.71477144	40.8386853	33.88741972	32.03626747	67.43483189	90.59677705	84.0800425	44.69629892	101.3203581	35.96524367	41.7075935	55.04344541	34.75632792	93.14680361	34.15187004
	82.62638001	88.11809455	96.6752388	100.9103235	93.47278714	79.52740655	57.19542901	84.79566144	81.07689328	78.69951096	91.30592284	90.14671255	90.79720794	90.79720794	104.0092969	52.54696882	89.44412163	86.96494286	17.52856866	34.26302537	88.20453225	92.89983699	97.82648071	101.1592103	96.6752388	37.05210149	38.29169088	83.2461747	59.67460779	90.00181127	19.38795274
AMP1CPR3FUS2DOX2TMP2	AMP	CPR	FUS	DOX	TMP	AMP+CPR	AMP+FUS	AMP+DOX	AMP+TMP	CPR+FUS	CPR+DOX	CPR+TMP	FUS+DOX	FUS+TMP	DOX+TMP	AMP+CPR+FUS	AMP+CPR+DOX	AMP+CPR+TMP	AMP+FUS+DOX	AMP+FUS+TMP	AMP+DOX+TMP	CPR+FUS+DOX	CPR+FUS+TMP	CPR+DOX+TMP	FUS+DOX+TMP	AMP+CPR+FUS+DOX	AMP+CPR+FUS+TMP	AMP+CPR+DOX+TMP	AMP+FUS+DOX+TMP	CPR+FUS+DOX+TMP	AMP+CPR+FUS+DOX+TMP
	78.14815665	86.75291545	97.64552145	92.53710799	94.59267246	74.9179839	43.24026704	66.69572599	81.96563353	80.15670554	79.17274052	81.79664723	107.6228249	92.65686971	103.6958866	55.5736539	91.94980385	53.48138292	50.87522081	57.04191424	86.95771869	76.07507289	82.78061224	82.6712828	99.11277196	39.12913809	44.12122324	42.32260433	57.04191424	78.4074344	35.89896534
	72.2704949	84.36337879	108.8787585	89.19531545	92.51983377	70.75935021	52.58783529	81.94182093	78.61730261	78.12998052	67.5579954	66.70798654	105.5530623	89.52925337	107.0268228	58.63241405	64.71477144	67.73706082	33.88741972	52.89006422	63.80808462	90.59677705	82.09668851	54.89640517	113.4137988	35.96524367	52.89006422	57.15904798	59.53910087	97.96352045	47.75217227
	82.62638001	88.11809455	96.6752388	100.9103235	99.36083672	79.52740655	57.19542901	84.79566144	75.80863839	78.69951096	91.30592284	94.49375113	90.79720794	83.44966936	89.13422429	52.54696882	89.44412163	84.79566144	17.52856866	60.91419717	86.96494286	92.89983699	102.7531244	103.4776309	102.2593681	37.05210149	55.95583963	77.35812512	76.71896184	100.1449013	57.50532636
AMP1CPR3FUS2DOX2TMP3	AMP	CPR	FUS	DOX	TMP	AMP+CPR	AMP+FUS	AMP+DOX	AMP+TMP	CPR+FUS	CPR+DOX	CPR+TMP	FUS+DOX	FUS+TMP	DOX+TMP	AMP+CPR+FUS	AMP+CPR+DOX	AMP+CPR+TMP	AMP+FUS+DOX	AMP+FUS+TMP	AMP+DOX+TMP	CPR+FUS+DOX	CPR+FUS+TMP	CPR+DOX+TMP	FUS+DOX+TMP	AMP+CPR+FUS+DOX	AMP+CPR+FUS+TMP	AMP+CPR+DOX+TMP	AMP+FUS+DOX+TMP	CPR+FUS+DOX+TMP	AMP+CPR+FUS+DOX+TMP
	78.14815665	86.75291545	97.64552145	92.53710799	98.11649728	74.9179839	43.24026704	66.69572599	81.96563353	80.15670554	79.17274052	76.25728863	107.6228249	101.1669227	103.9895386	55.5736539	91.94980385	56.12425153	50.87522081	58.51017459	72.86241942	76.07507289	77.24125364	80.63046647	110.850776	39.12913809	49.37025396	66.10842185	45.58948359	76.36661808	23.2719264
	72.2704949	84.36337879	108.8787585	89.19531545	99.16887042	70.75935021	52.58783529	81.94182093	71.96826596	78.12998052	67.5579954	61.32459713	105.5530623	94.06429364	98.56441254	58.63241405	64.71477144	57.46127692	33.88741972	45.33434076	65.62145825	90.59677705	66.51319285	95.41349389	109.1810946	35.96524367	44.42765395	66.22591613	46.84548546	96.26350274	12.08915754
	82.62638001	88.11809455	96.6752388	100.9103235	102.4598102	79.52740655	57.19542901	84.79566144	77.35812512	78.69951096	91.30592284	106.9552617	90.79720794	96.96914034	91.92330041	52.54696882	89.44412163	76.73833043	17.52856866	58.12512105	84.79566144	92.89983699	99.56529614	97.68157942	105.1983835	37.05210149	51.30737943	82.93627736	63.39337594	94.34884985	46.96881658
AMP1CPR3FUS2DOX3TMP1	AMP	CPR	FUS	DOX	TMP	AMP+CPR	AMP+FUS	AMP+DOX	AMP+TMP	CPR+FUS	CPR+DOX	CPR+TMP	FUS+DOX	FUS+TMP	DOX+TMP	AMP+CPR+FUS	AMP+CPR+DOX	AMP+CPR+TMP	AMP+FUS+DOX	AMP+FUS+TMP	AMP+DOX+TMP	CPR+FUS+DOX	CPR+FUS+TMP	CPR+DOX+TMP	FUS+DOX+TMP	AMP+CPR+FUS+DOX	AMP+CPR+FUS+TMP	AMP+CPR+DOX+TMP	AMP+FUS+DOX+TMP	CPR+FUS+DOX+TMP	AMP+CPR+FUS+DOX+TMP
	78.14815665	86.75291545	97.64552145	103.4022345	99.29110555	74.9179839	43.24026704	80.20372112	83.1402418	80.15670554	68.96865889	65.47011662	98.52587175	99.11277196	106.6324073	55.5736539	63.7592053	47.31468949	63.79591181	49.70061254	69.63224667	74.90889213	79.86516035	87.44533528	117.3066783	48.52600427	23.2719264	60.23538049	48.52600427	89.4861516	33.25609672
	72.2704949	84.36337879	108.8787585	94.93766528	98.2621836	70.75935021	52.58783529	88.89308651	86.47525501	78.12998052	68.40800425	61.8912697	100.715686	90.43626143	100.6800151	58.63241405	51.11446921	40.8386853	50.47223272	32.03626747	60.78579524	87.7634142	84.0800425	79.26332566	103.7390462	47.75217227	41.7075935	64.11031356	49.5655459	99.38020188	42.61428032
	82.62638001	88.11809455	96.6752388	112.6864226	93.47278714	79.52740655	57.19542901	83.55607205	81.07689328	78.69951096	93.04473827	90.14671255	95.49963262	90.79720794	108.6577571	52.54696882	95.95196591	86.96494286	23.41661825	34.26302537	83.8659694	101.3041116	97.82648071	99.71019743	92.26671565	49.13809801	38.29169088	68.37110207	66.49234941	106.2307553	62.77358125
AMP1CPR3FUS2DOX3TMP2	AMP	CPR	FUS	DOX	TMP	AMP+CPR	AMP+FUS	AMP+DOX	AMP+TMP	CPR+FUS	CPR+DOX	CPR+TMP	FUS+DOX	FUS+TMP	DOX+TMP	AMP+CPR+FUS	AMP+CPR+DOX	AMP+CPR+TMP	AMP+FUS+DOX	AMP+FUS+TMP	AMP+DOX+TMP	CPR+FUS+DOX	CPR+FUS+TMP	CPR+DOX+TMP	FUS+DOX+TMP	AMP+CPR+FUS+DOX	AMP+CPR+FUS+TMP	AMP+CPR+DOX+TMP	AMP+FUS+DOX+TMP	CPR+FUS+DOX+TMP	AMP+CPR+FUS+DOX+TMP
	78.14815665	86.75291545	97.64552145	103.4022345	94.59267246	74.9179839	43.24026704	80.20372112	81.96563353	80.15670554	68.96865889	81.79664723	98.52587175	92.65686971	102.5212783	55.5736539	63.7592053	53.48138292	63.79591181	57.04191424	67.87033426	74.90889213	82.78061224	87.33600583	117.8935785	48.52600427	44.12122324	58.47346808	47.64504806	100.5648688	21.80366606
	72.2704949	84.36337879	108.8787585	94.93766528	92.51983377	70.75935021	52.58783529	88.89308651	78.61730261	78.12998052	68.40800425	66.70798654	100.715686	89.52925337	92.21760484	58.63241405	51.11446921	67.73706082	50.47223272	52.89006422	57.46127692	87.7634142	82.09668851	78.97998937	106.4600704	47.75217227	52.89006422	70.45712127	53.4945221	98.81352931	44.12542501
	82.62638001	88.11809455	96.6752388	112.6864226	99.36083672	79.52740655	57.19542901	83.55607205	75.80863839	78.69951096	93.04473827	94.49375113	95.49963262	83.44966936	101.5301181	52.54696882	95.95196591	84.79566144	23.41661825	60.91419717	77.66802247	101.3041116	102.7531244	77.97500453	87.85819251	49.13809801	55.95583963	65.8919233	69.59132287	105.9409527	38.91148557
AMP1CPR3FUS2DOX3TMP3	AMP	CPR	FUS	DOX	TMP	AMP+CPR	AMP+FUS	AMP+DOX	AMP+TMP	CPR+FUS	CPR+DOX	CPR+TMP	FUS+DOX	FUS+TMP	DOX+TMP	AMP+CPR+FUS	AMP+CPR+DOX	AMP+CPR+TMP	AMP+FUS+DOX	AMP+FUS+TMP	AMP+DOX+TMP	CPR+FUS+DOX	CPR+FUS+TMP	CPR+DOX+TMP	FUS+DOX+TMP	AMP+CPR+FUS+DOX	AMP+CPR+FUS+TMP	AMP+CPR+DOX+TMP	AMP+FUS+DOX+TMP	CPR+FUS+DOX+TMP	AMP+CPR+FUS+DOX+TMP
	78.14815665	86.75291545	97.64552145	103.4022345	98.11649728	74.9179839	43.24026704	80.20372112	81.96563353	80.15670554	68.96865889	76.25728863	98.52587175	101.1669227	91.65615178	55.5736539	63.7592053	56.12425153	63.79591181	58.51017459	78.14815665	74.90889213	77.24125364	101.9132653	115.2525276	48.52600427	49.37025396	90.18789144	40.30374636	102.8972303	13.87506022
	72.2704949	84.36337879	108.8787585	94.93766528	99.16887042	70.75935021	52.58783529	88.89308651	71.96826596	78.12998052	68.40800425	61.32459713	100.715686	94.06429364	94.03097847	58.63241405	51.11446921	57.46127692	50.47223272	45.33434076	84.35965244	87.7634142	66.51319285	85.9748539	107.0647424	47.75217227	44.42765395	76.80392898	58.93464299	95.41349389	44.42765395
	82.62638001	88.11809455	96.6752388	112.6864226	102.4598102	79.52740655	57.19542901	83.55607205	77.35812512	78.69951096	93.04473827	106.9552617	95.49963262	96.96914034	101.5301181	52.54696882	95.95196591	76.73833043	23.41661825	58.12512105	81.38679063	101.3041116	99.56529614	86.66908169	94.03012491	49.13809801	51.30737943	72.08987023	61.53399187	106.5205579	27.44528375
AMP1CPR3FUS3DOX1TMP1	AMP	CPR	FUS	DOX	TMP	AMP+CPR	AMP+FUS	AMP+DOX	AMP+TMP	CPR+FUS	CPR+DOX	CPR+TMP	FUS+DOX	FUS+TMP	DOX+TMP	AMP+CPR+FUS	AMP+CPR+DOX	AMP+CPR+TMP	AMP+FUS+DOX	AMP+FUS+TMP	AMP+DOX+TMP	CPR+FUS+DOX	CPR+FUS+TMP	CPR+DOX+TMP	FUS+DOX+TMP	AMP+CPR+FUS+DOX	AMP+CPR+FUS+TMP	AMP+CPR+DOX+TMP	AMP+FUS+DOX+TMP	CPR+FUS+DOX+TMP	AMP+CPR+FUS+DOX+TMP
	78.14815665	86.75291545	95.90014577	88.42597903	99.29110555	74.9179839	63.50225974	66.98937805	83.1402418	78.4074344	96.6654519	65.47011662	77.53279883	72.8680758	96.94188901	57.2988598	96.0609328	47.31468949	59.97843493	69.3385946	63.7592053	87.44533528	84.82142857	78.88119534	76.94970845	70.2195508	46.72738535	66.69572599	45.55277708	63.24708455	46.14008121
	72.2704949	84.36337879	92.29679476	90.40423121	98.2621836	70.75935021	74.95277673	65.92368719	86.47525501	81.53001594	80.59146449	61.8912697	78.69665309	54.89640517	92.82206271	78.01284473	81.94182093	40.8386853	36.87193049	61.39025312	74.99055535	85.49672392	75.86329024	67.5579954	83.51336993	64.11031356	48.09217983	52.6256139	52.6256139	63.67983	43.25651681
	82.62638001	88.11809455	84.78536497	95.95196591	93.47278714	79.52740655	49.73852411	75.80863839	81.07689328	73.19326209	93.33454084	90.14671255	94.63865242	89.42220612	108.9676545	77.35812512	88.5144296	86.96494286	63.06411001	75.1888437	82.93627736	89.42220612	92.89983699	104.9266437	93.47944213	74.569049	63.41274453	69.61069146	63.10284718	76.09128781	83.2461747
AMP1CPR3FUS3DOX1TMP2	AMP	CPR	FUS	DOX	TMP	AMP+CPR	AMP+FUS	AMP+DOX	AMP+TMP	CPR+FUS	CPR+DOX	CPR+TMP	FUS+DOX	FUS+TMP	DOX+TMP	AMP+CPR+FUS	AMP+CPR+DOX	AMP+CPR+TMP	AMP+FUS+DOX	AMP+FUS+TMP	AMP+DOX+TMP	CPR+FUS+DOX	CPR+FUS+TMP	CPR+DOX+TMP	FUS+DOX+TMP	AMP+CPR+FUS+DOX	AMP+CPR+FUS+TMP	AMP+CPR+DOX+TMP	AMP+FUS+DOX+TMP	CPR+FUS+DOX+TMP	AMP+CPR+FUS+DOX+TMP
	78.14815665	86.75291545	95.90014577	88.42597903	94.59267246	74.9179839	63.50225974	66.98937805	81.96563353	78.4074344	96.6654519	81.79664723	77.53279883	57.99927114	99.58475762	57.2988598	96.0609328	53.48138292	59.97843493	60.56573906	62.29094496	87.44533528	66.16253644	71.30102041	87.44533528	70.2195508	54.06868705	62.58459703	60.23538049	71.70189504	54.98634977
	72.2704949	84.36337879	92.29679476	90.40423121	92.51983377	70.75935021	74.95277673	65.92368719	78.61730261	81.53001594	80.59146449	66.70798654	78.69665309	56.59642288	96.14658104	78.01284473	81.94182093	67.73706082	36.87193049	53.53230072	71.66603702	85.49672392	70.76323712	63.87462369	71.0465734	64.11031356	63.20362675	46.58103513	64.71477144	63.11315743	32.33849641
	82.62638001	88.11809455	84.78536497	95.95196591	99.36083672	79.52740655	49.73852411	75.80863839	75.80863839	73.19326209	93.33454084	94.49375113	94.63865242	90.87121898	100.6004261	77.35812512	88.5144296	84.79566144	63.06411001	64.03253922	76.42843308	89.42220612	96.37746785	106.9552617	90.87121898	74.569049	88.82432694	79.83730389	56.55626574	90.29161384	68.04183614
AMP1CPR3FUS3DOX1TMP3	AMP	CPR	FUS	DOX	TMP	AMP+CPR	AMP+FUS	AMP+DOX	AMP+TMP	CPR+FUS	CPR+DOX	CPR+TMP	FUS+DOX	FUS+TMP	DOX+TMP	AMP+CPR+FUS	AMP+CPR+DOX	AMP+CPR+TMP	AMP+FUS+DOX	AMP+FUS+TMP	AMP+DOX+TMP	CPR+FUS+DOX	CPR+FUS+TMP	CPR+DOX+TMP	FUS+DOX+TMP	AMP+CPR+FUS+DOX	AMP+CPR+FUS+TMP	AMP+CPR+DOX+TMP	AMP+FUS+DOX+TMP	CPR+FUS+DOX+TMP	AMP+CPR+FUS+DOX+TMP
	78.14815665	86.75291545	95.90014577	88.42597903	98.11649728	74.9179839	63.50225974	66.98937805	81.96563353	78.4074344	96.6654519	76.25728863	77.53279883	66.74562682	99.29110555	57.2988598	96.0609328	56.12425153	59.97843493	59.06077221	71.98146321	87.44533528	61.49781341	82.96282799	83.65524781	70.2195508	68.16398633	79.91006905	39.67973571	91.52696793	47.05774393
	72.2704949	84.36337879	92.29679476	90.40423121	99.16887042	70.75935021	74.95277673	65.92368719	71.96826596	81.53001594	80.59146449	61.32459713	78.69665309	65.09651142	99.16887042	78.01284473	81.94182093	57.46127692	36.87193049	55.64790329	69.55043445	85.49672392	63.96316628	67.84133168	73.59659996	64.11031356	67.73706082	65.01700038	46.58103513	71.0465734	38.4208538
	82.62638001	88.11809455	84.78536497	95.95196591	102.4598102	79.52740655	49.73852411	75.80863839	77.35812512	73.19326209	93.33454084	106.9552617	94.63865242	81.88733925	104.938989	77.35812512	88.5144296	76.73833043	63.06411001	53.80592679	79.83730389	89.42220612	98.11628328	106.085854	75.07697881	74.569049	51.90780554	90.68371102	44.50900639	94.20394856	50.04842146
AMP1CPR3FUS3DOX2TMP1	AMP	CPR	FUS	DOX	TMP	AMP+CPR	AMP+FUS	AMP+DOX	AMP+TMP	CPR+FUS	CPR+DOX	CPR+TMP	FUS+DOX	FUS+TMP	DOX+TMP	AMP+CPR+FUS	AMP+CPR+DOX	AMP+CPR+TMP	AMP+FUS+DOX	AMP+FUS+TMP	AMP+DOX+TMP	CPR+FUS+DOX	CPR+FUS+TMP	CPR+DOX+TMP	FUS+DOX+TMP	AMP+CPR+FUS+DOX	AMP+CPR+FUS+TMP	AMP+CPR+DOX+TMP	AMP+FUS+DOX+TMP	CPR+FUS+DOX+TMP	AMP+CPR+FUS+DOX+TMP
	78.14815665	86.75291545	95.90014577	92.53710799	99.29110555	74.9179839	63.50225974	66.69572599	83.1402418	78.4074344	79.17274052	65.47011662	91.52696793	72.8680758	100.7593659	57.2988598	91.94980385	47.31468949	59.09747872	69.3385946	70.51320287	79.57361516	84.82142857	68.31268222	64.59548105	56.71155567	46.72738535	64.34650944	58.17981601	69.77040816	54.98634977
	72.2704949	84.36337879	92.29679476	89.19531545	98.2621836	70.75935021	74.95277673	81.94182093	86.47525501	81.53001594	67.5579954	61.8912697	68.49654684	54.89640517	97.35549679	78.01284473	64.71477144	40.8386853	62.86361919	61.39025312	67.43483189	84.64671507	75.86329024	44.69629892	73.0299274	54.13675859	48.09217983	55.04344541	59.27465055	90.03010448	58.97242161
	82.62638001	88.11809455	84.78536497	100.9103235	93.47278714	79.52740655	49.73852411	84.79566144	81.07689328	73.19326209	91.30592284	90.14671255	73.62796595	89.42220612	104.0092969	77.35812512	89.44412163	86.96494286	48.84756924	75.1888437	88.20453225	98.40608585	92.89983699	101.1592103	101.8837167	69.61069146	63.41274453	83.2461747	70.5403835	91.45082413	65.54328879
AMP1CPR3FUS3DOX2TMP2	AMP	CPR	FUS	DOX	TMP	AMP+CPR	AMP+FUS	AMP+DOX	AMP+TMP	CPR+FUS	CPR+DOX	CPR+TMP	FUS+DOX	FUS+TMP	DOX+TMP	AMP+CPR+FUS	AMP+CPR+DOX	AMP+CPR+TMP	AMP+FUS+DOX	AMP+FUS+TMP	AMP+DOX+TMP	CPR+FUS+DOX	CPR+FUS+TMP	CPR+DOX+TMP	FUS+DOX+TMP	AMP+CPR+FUS+DOX	AMP+CPR+FUS+TMP	AMP+CPR+DOX+TMP	AMP+FUS+DOX+TMP	CPR+FUS+DOX+TMP	AMP+CPR+FUS+DOX+TMP

	78.14815665	86.75291545	95.90014577	92.53710799	94.59267246	74.9179839	63.50225974	66.69572599	81.96563353	78.4074344	79.17274052	81.79664723	91.52696793	57.99927114	103.6958866	57.2988598	91.94980385	53.48138292	59.09747872	60.56573906	86.95771869	79.57361516	66.16253644	82.6712828	81.61443149	56.71155567	54.06868705	42.32260433	81.96563353	71.11880466	40.01009429
	72.2704949	84.36337879	92.29679476	89.19531545	92.51983377	70.75935021	74.95277673	81.94182093	78.61730261	81.53001594	67.5579954	66.70798654	68.49654684	56.59642288	107.0268228	78.01284473	64.71477144	67.73706082	62.86361919	53.53230072	63.80808462	84.64671507	70.76323712	54.89640517	98.24685674	54.13675859	63.20362675	57.15904798	70.45712127	92.29679476	46.88326407
	82.62638001	88.11809455	84.78536497	100.9103235	99.36083672	79.52740655	49.73852411	84.79566144	75.80863839	73.19326209	91.30592284	94.49375113	73.62796595	90.87121898	89.13422429	77.35812512	89.44412163	84.79566144	48.84756924	64.03253922	86.96494286	98.40608585	96.37746785	103.4776309	99.85509871	69.61069146	88.82432694	77.35812512	56.86616308	97.82648071	50.68758474
AMP1CPR3FUS3DOX2TMP3	AMP	CPR	FUS	DOX	TMP	AMP+CPR	AMP+FUS	AMP+DOX	AMP+TMP	CPR+FUS	CPR+DOX	CPR+TMP	FUS+DOX	FUS+TMP	DOX+TMP	AMP+CPR+FUS	AMP+CPR+DOX	AMP+CPR+TMP	AMP+FUS+DOX	AMP+FUS+TMP	AMP+DOX+TMP	CPR+FUS+DOX	CPR+FUS+TMP	CPR+DOX+TMP	FUS+DOX+TMP	AMP+CPR+FUS+DOX	AMP+CPR+FUS+TMP	AMP+CPR+DOX+TMP	AMP+FUS+DOX+TMP	CPR+FUS+DOX+TMP	AMP+CPR+FUS+DOX+TMP
	78.14815665	86.75291545	95.90014577	92.53710799	98.11649728	74.9179839	63.50225974	66.69572599	81.96563353	78.4074344	79.17274052	76.25728863	91.52696793	66.74562682	103.9895386	57.2988598	91.94980385	56.12425153	59.09747872	59.06077221	72.86241942	79.57361516	61.49781341	80.63046647	108.4365889	56.71155567	68.16398633	66.10842185	56.45461011	73.15962099	51.46252495
	72.2704949	84.36337879	92.29679476	89.19531545	99.16887042	70.75935021	74.95277673	81.94182093	71.96826596	81.53001594	67.5579954	61.32459713	68.49654684	65.09651142	98.56441254	78.01284473	64.71477144	57.46127692	62.86361919	55.64790329	65.62145825	84.64671507	63.96316628	95.41349389	88.61342306	54.13675859	67.73706082	66.22591613	51.37891953	100.2302107	63.16584813
	82.62638001	88.11809455	84.78536497	100.9103235	102.4598102	79.52740655	49.73852411	84.79566144	77.35812512	73.19326209	91.30592284	106.9552617	73.62796595	81.88733925	91.92330041	77.35812512	89.44412163	76.73833043	48.84756924	53.80592679	84.79566144	98.40608585	98.11628328	97.68157942	99.56529614	69.61069146	51.90780554	82.93627736	58.4350184	95.21825756	60.60429983
AMP1CPR3FUS3DOX3TMP1	AMP	CPR	FUS	DOX	TMP	AMP+CPR	AMP+FUS	AMP+DOX	AMP+TMP	CPR+FUS	CPR+DOX	CPR+TMP	FUS+DOX	FUS+TMP	DOX+TMP	AMP+CPR+FUS	AMP+CPR+DOX	AMP+CPR+TMP	AMP+FUS+DOX	AMP+FUS+TMP	AMP+DOX+TMP	CPR+FUS+DOX	CPR+FUS+TMP	CPR+DOX+TMP	FUS+DOX+TMP	AMP+CPR+FUS+DOX	AMP+CPR+FUS+TMP	AMP+CPR+DOX+TMP	AMP+FUS+DOX+TMP	CPR+FUS+DOX+TMP	AMP+CPR+FUS+DOX+TMP
	78.14815665	86.75291545	95.90014577	103.4022345	99.29110555	74.9179839	63.50225974	80.20372112	83.1402418	78.4074344	68.96865889	65.47011662	48.66982507	72.8680758	106.6324073	57.2988598	63.7592053	47.31468949	57.00520774	69.3385946	69.63224667	84.23833819	84.82142857	87.44533528	102.3141399	57.59251187	46.72738535	60.23538049	54.94964326	83.946793	58.47346808
	72.2704949	84.36337879	92.29679476	94.93766528	98.2621836	70.75935021	74.95277673	88.89308651	86.47525501	81.53001594	68.40800425	61.8912697	61.41313972	54.89640517	100.6800151	78.01284473	51.11446921	40.8386853	58.33018512	61.39025312	60.78579524	80.11333451	75.86329024	79.26332566	85.78006021	63.20362675	48.09217983	64.11031356	59.57687949	90.59677705	45.37211938
	82.62638001	88.11809455	84.78536497	112.6864226	93.47278714	79.52740655	49.73852411	83.55607205	81.07689328	73.19326209	93.04473827	90.14671255	68.41151965	89.42220612	108.6577571	77.35812512	95.95196591	86.96494286	53.76718962	75.1888437	83.8659694	94.63865242	92.89983699	99.71019743	101.5939141	83.55607205	63.41274453	68.37110207	76.42843308	99.56529614	49.44799535
AMP1CPR3FUS3DOX3TMP2	AMP	CPR	FUS	DOX	TMP	AMP+CPR	AMP+FUS	AMP+DOX	AMP+TMP	CPR+FUS	CPR+DOX	CPR+TMP	FUS+DOX	FUS+TMP	DOX+TMP	AMP+CPR+FUS	AMP+CPR+DOX	AMP+CPR+TMP	AMP+FUS+DOX	AMP+FUS+TMP	AMP+DOX+TMP	CPR+FUS+DOX	CPR+FUS+TMP	CPR+DOX+TMP	FUS+DOX+TMP	AMP+CPR+FUS+DOX	AMP+CPR+FUS+TMP	AMP+CPR+DOX+TMP	AMP+FUS+DOX+TMP	CPR+FUS+DOX+TMP	AMP+CPR+FUS+DOX+TMP
	78.14815665	86.75291545	95.90014577	103.4022345	94.59267246	74.9179839	63.50225974	80.20372112	81.96563353	78.4074344	68.96865889	81.79664723	48.66982507	57.99927114	102.5212783	57.2988598	63.7592053	53.48138292	57.00520774	60.56573906	67.87033426	84.23833819	66.16253644	87.33600583	98.52405248	57.59251187	54.06868705	58.47346808	62.58459703	100.856414	42.61625639
	72.2704949	84.36337879	92.29679476	94.93766528	92.51983377	70.75935021	74.95277673	88.89308651	78.61730261	81.53001594	68.40800425	66.70798654	61.41313972	56.59642288	92.21760484	78.01284473	51.11446921	67.73706082	58.33018512	53.53230072	57.46127692	80.11333451	70.76323712	78.97998937	88.89675934	63.20362675	63.20362675	70.45712127	55.34567435	93.71347618	54.43898753
	82.62638001	88.11809455	84.78536497	112.6864226	99.36083672	79.52740655	49.73852411	83.55607205	75.80863839	73.19326209	93.04473827	94.49375113	68.41151965	90.87121898	101.5301181	77.35812512	95.95196591	84.79566144	53.76718962	64.03253922	77.66802247	94.63865242	96.37746785	77.97500453	98.69588843	83.55607205	88.82432694	65.8919233	59.36471044	103.3327296	59.96513655
AMP1CPR3FUS3DOX3TMP3	AMP	CPR	FUS	DOX	TMP	AMP+CPR	AMP+FUS	AMP+DOX	AMP+TMP	CPR+FUS	CPR+DOX	CPR+TMP	FUS+DOX	FUS+TMP	DOX+TMP	AMP+CPR+FUS	AMP+CPR+DOX	AMP+CPR+TMP	AMP+FUS+DOX	AMP+FUS+TMP	AMP+DOX+TMP	CPR+FUS+DOX	CPR+FUS+TMP	CPR+DOX+TMP	FUS+DOX+TMP	AMP+CPR+FUS+DOX	AMP+CPR+FUS+TMP	AMP+CPR+DOX+TMP	AMP+FUS+DOX+TMP	CPR+FUS+DOX+TMP	AMP+CPR+FUS+DOX+TMP
	78.14815665	86.75291545	95.90014577	103.4022345	98.11649728	74.9179839	63.50225974	80.20372112	81.96563353	78.4074344	68.96865889	76.25728863	48.66982507	66.74562682	91.65615178	57.2988598	63.7592053	56.12425153	57.00520774	59.06077221	78.14815665	84.23833819	61.49781341	101.9132653	96.48323615	57.59251187	68.16398633	90.18789144	77.56085251	95.31705539	49.66390603
	72.2704949	84.36337879	92.29679476	94.93766528	99.16887042	70.75935021	74.95277673	88.89308651	71.96826596	81.53001594	68.40800425	61.32459713	61.41313972	65.09651142	94.03097847	78.01284473	51.11446921	57.46127692	58.33018512	55.64790329	84.35965244	80.11333451	63.96316628	85.9748539	85.78006021	63.20362675	67.73706082	76.80392898	68.3415187	88.61342306	68.03928976
	82.62638001	88.11809455	84.78536497	112.6864226	102.4598102	79.52740655	49.73852411	83.55607205	77.35812512	73.19326209	93.04473827	106.9552617	68.41151965	81.88733925	101.5301181	77.35812512	95.95196591	76.73833043	53.76718962	53.80592679	81.38679063	94.63865242	98.11628328	86.66908169	94.34884985	83.55607205	51.90780554	72.08987023	76.42843308	101.8837167	67.71257021
AMP2CPR1FUS1DOX1TMP1	AMP	CPR	FUS	DOX	TMP	AMP+CPR	AMP+FUS	AMP+DOX	AMP+TMP	CPR+FUS	CPR+DOX	CPR+TMP	FUS+DOX	FUS+TMP	DOX+TMP	AMP+CPR+FUS	AMP+CPR+DOX	AMP+CPR+TMP	AMP+FUS+DOX	AMP+FUS+TMP	AMP+DOX+TMP	CPR+FUS+DOX	CPR+FUS+TMP	CPR+DOX+TMP	FUS+DOX+TMP	AMP+CPR+FUS+DOX	AMP+CPR+FUS+TMP	AMP+CPR+DOX+TMP	AMP+FUS+DOX+TMP	CPR+FUS+DOX+TMP	AMP+CPR+FUS+DOX+TMP
	72.70226278	87.25137076	72.56876735	88.42597903	99.29110555	65.25596644	21.09172608	74.46296339	82.67956624	69.63224667	87.8386749	91.06884765	74.33067976	84.90215421	96.94188901	38.6987322	60.67080859	54.80180655	23.86116142	38.4052821	74.75641349	65.81476978	67.87033426	95.47362867	75.7989401	32.82973016	33.83846489	61.5511589	39.28563241	76.09259217	46.75026938
	69.27274016	87.38194182	80.73290518	90.40423121	98.2621836	68.66806812	41.81685051	63.3582918	73.80778043	79.52398942	90.10200227	85.56856819	65.01700038	76.80392898	92.82206271	49.979923	78.3428207	49.75317099	24.69707348	38.3021943	64.73769988	67.13260295	94.93766528	97.95995467	98.56441254	39.39816236	45.44488273	56.10222737	48.2792829	88.89308651	45.14254671
	75.20205731	92.5430951	93.47278714	95.95196591	93.47278714	64.63997061	30.52902278	71.45481264	60.21307862	99.98063142	96.5717606	99.36083672	63.10284718	101.2202208	108.9676545	38.17046289	83.21087436	48.1814842	31.41072741	46.10580456	59.64364438	99.98063142	103.3895022	84.79566144	86.34514817	30.82292432	34.34974284	66.40337987	31.70462895	93.78268449	34.95591477
AMP2CPR1FUS1DOX1TMP2	AMP	CPR	FUS	DOX	TMP	AMP+CPR	AMP+FUS	AMP+DOX	AMP+TMP	CPR+FUS	CPR+DOX	CPR+TMP	FUS+DOX	FUS+TMP	DOX+TMP	AMP+CPR+FUS	AMP+CPR+DOX	AMP+CPR+TMP	AMP+FUS+DOX	AMP+FUS+TMP	AMP+DOX+TMP	CPR+FUS+DOX	CPR+FUS+TMP	CPR+DOX+TMP	FUS+DOX+TMP	AMP+CPR+FUS+DOX	AMP+CPR+FUS+TMP	AMP+CPR+DOX+TMP	AMP+FUS+DOX+TMP	CPR+FUS+DOX+TMP	AMP+CPR+FUS+DOX+TMP
	72.70226278	87.25137076	72.56876735	88.42597903	94.59267246	65.25596644	21.09172608	74.46296339	72.40881267	69.63224667	87.8386749	95.1799766	74.33067976	94.59267246	99.58475762	38.6987322	60.67080859	55.68215686	23.86116142	41.04633302	80.03851533	65.81476978	79.32276492	98.70380142	69.92589874	32.82973016	38.111832	66.53981063	17.99215938	65.81476978	53.79307183
	69.27274016	87.38194182	80.73290518	90.40423121	92.51983377	68.66806812	41.81685051	63.3582918	66.85405201	79.52398942	90.10200227	90.70646014	65.01700038	102.7956177	96.14658104	49.979923	78.3428207	81.06384486	24.69707348	51.00030706	65.34237192	67.13260295	107.9335096	106.120136	77.40838685	39.39816236	46.04955476	60.20265961	23.67668942	82.24404987	43.93320264
	75.20205731	92.5430951	93.47278714	95.95196591	99.36083672	64.63997061	30.52902278	71.45481264	60.23144747	99.98063142	96.5717606	90.68371102	63.10284718	102.1499128	100.6004261	38.17046289	83.21087436	81.09845702	31.41072741	51.10213079	61.09478325	99.98063142	97.81134999	91.61340306	73.94925431	30.82292432	28.76561352	51.12049963	40.52167524	82.93627736	45.24246877
AMP2CPR1FUS1DOX1TMP3	AMP	CPR	FUS	DOX	TMP	AMP+CPR	AMP+FUS	AMP+DOX	AMP+TMP	CPR+FUS	CPR+DOX	CPR+TMP	FUS+DOX	FUS+TMP	DOX+TMP	AMP+CPR+FUS	AMP+CPR+DOX	AMP+CPR+TMP	AMP+FUS+DOX	AMP+FUS+TMP	AMP+DOX+TMP	CPR+FUS+DOX	CPR+FUS+TMP	CPR+DOX+TMP	FUS+DOX+TMP	AMP+CPR+FUS+DOX	AMP+CPR+FUS+TMP	AMP+CPR+DOX+TMP	AMP+FUS+DOX+TMP	CPR+FUS+DOX+TMP	AMP+CPR+FUS+DOX+TMP
	72.70226278	87.25137076	72.56876735	88.42597903	98.11649728	65.25596644	21.09172608	74.46296339	78.57126482	69.63224667	87.8386749	89.89423937	74.33067976	73.74337562	99.29110555	38.6987322	60.67080859	53.92145625	23.86116142	28.84981315	75.9302139	65.81476978	73.15607149	99.29110555	95.76728074	32.82973016	37.65331622	61.73456521	18.87250969	62.29094496	34.13191499
	69.27274016	87.38194182	80.73290518	90.40423121	99.16887042	68.66806812	41.81685051	63.3582918	67.45872405	79.52398942	90.10200227	93.72874953	65.01700038	98.56441254	99.16887042	49.979923	78.3428207	65.47464393	24.69707348	41.02321846	65.94704396	67.13260295	87.07971288	104.9112202	111.258028	39.39816236	41.81685051	57.78397147	5.536528332	87.68417076	26.94569762
	75.20205731	92.5430951	93.47278714	95.95196591	102.4598102	64.63997061	30.52902278	71.45481264	66.40337987	99.98063142	96.5717606	85.41545613	63.10284718	94.09258183	104.938989	38.17046289	83.21087436	77.5716385	31.41072741	20.24246877	62.56429096	99.98063142	89.44412163	86.03525082	95.33217122	30.82292432	25.238795	57.29243204	49.63262307	81.07689328	32.60470242
AMP2CPR1FUS1DOX2TMP1	AMP	CPR	FUS	DOX	TMP	AMP+CPR	AMP+FUS	AMP+DOX	AMP+TMP	CPR+FUS	CPR+DOX	CPR+TMP	FUS+DOX	FUS+TMP	DOX+TMP	AMP+CPR+FUS	AMP+CPR+DOX	AMP+CPR+TMP	AMP+FUS+DOX	AMP+FUS+TMP	AMP+DOX+TMP	CPR+FUS+DOX	CPR+FUS+TMP	CPR+DOX+TMP	FUS+DOX+TMP	AMP+CPR+FUS+DOX	AMP+CPR+FUS+TMP	AMP+CPR+DOX+TMP	AMP+FUS+DOX+TMP	CPR+FUS+DOX+TMP	AMP+CPR+FUS+DOX+TMP
	72.70226278	87.25137076	72.56876735	92.53710799	99.29110555	65.25596644	21.09172608	71.23501227	82.67956624	69.63224667	94.59267246	91.06884765	82.2592856	84.90215421	100.7593659	38.6987322	75.6367638	54.80180655	29.01487884	38.4052821	76.5171141	76.6798963	67.87033426	101.053018	96.0609328	18.45067516	33.83846489	58.91010798	20.50482588	69.04494253	44.69611866
	69.27274016	87.38194182	80.73290518	89.19531545	98.2621836	68.66806812	41.81685051	69.87741219	73.80778043	79.52398942	87.38194182	85.56856819	86.77748394	76.80392898	97.35549679	49.979923	69.57507617	49.75317099	30.74379384	38.3021943	65.34237192	71.06157915	94.93766528	107.9335096	101.8889309	27.60705766	45.44488273	54.89288329	27.00238562	80.73290518	58.86104353
	75.20205731	92.5430951	93.47278714	100.9103235	93.47278714	64.63997061	30.52902278	64.93387215	60.21307862	99.98063142	99.36083672	99.36083672	100.6004261	101.2202208	104.0092969	38.17046289	75.86333578	48.1814842	44.63629684	46.10580456	70.49963262	99.98063142	103.3895022	92.5430951	73.32945962	29.05951506	34.34974284	59.93754592	23.1814842	84.79566144	51.70830272
AMP2CPR1FUS1DOX2TMP2	AMP	CPR	FUS	DOX	TMP	AMP+CPR	AMP+FUS	AMP+DOX	AMP+TMP	CPR+FUS	CPR+DOX	CPR+TMP	FUS+DOX	FUS+TMP	DOX+TMP	AMP+CPR+FUS	AMP+CPR+DOX	AMP+CPR+TMP	AMP+FUS+DOX	AMP+FUS+TMP	AMP+DOX+TMP	CPR+FUS+DOX	CPR+FUS+TMP	CPR+DOX+TMP	FUS+DOX+TMP	AMP+CPR+FUS+DOX	AMP+CPR+FUS+TMP	AMP+CPR+DOX+TMP	AMP+FUS+DOX+TMP	CPR+FUS+DOX+TMP	AMP+CPR+FUS+DOX+TMP
	72.70226278	87.25137076	72.56876735	92.53710799	94.59267246	65.25596644	21.09172608	71.23501227	72.40881267	69.63224667	94.59267246	95.1799766	82.2592856	94.59267246	103.6958866	38.6987322	75.6367638	55.68215686	29.01487884	41.04633302	72.11536257	76.6798963	79.32276492	86.07676249	70.80685494	18.45067516	38.111832	72.00531878	35.0122653	75.21163596	28.84981315
	69.27274016	87.38194182	80.73290518	89.19531545	92.51983377	68.66806812	41.81685051	69.87741219	66.85405201	79.52398942	87.38194182	90.70646014	86.77748394	102.7956177	107.0268228	49.979923	69.57507617	81.06384486	30.74379384	51.00030706	76.52880459	71.06157915	107.9335096	97.35549679	82.24404987	27.60705766	46.04955476	69.70734818	32.14209793	57.15904798	29.53444977
	75.20205731	92.5430951	93.47278714	100.9103235	99.36083672	64.63997061	30.52902278	64.93387215	60.23144747	99.98063142	99.36083672	90.68371102	100.6004261	102.1499128	89.13422429	38.17046289	75.86333578	81.09845702	44.63629684	51.10213079	79.02277737	99.98063142	97.81134999	86.03525082	84.79566144	29.05951506	28.76561352	71.98750918	31.11682586	72.08987023	19.0668626
AMP2CPR1FUS1DOX2TMP3	AMP	CPR	FUS	DOX	TMP	AMP+CPR	AMP+FUS	AMP+DOX	AMP+TMP	CPR+FUS	CPR+DOX	CPR+TMP	FUS+DOX	FUS+TMP	DOX+TMP	AMP+CPR+FUS	AMP+CPR+DOX	AMP+CPR+TMP	AMP+FUS+DOX	AMP+FUS+TMP	AMP+DOX+TMP	CPR+FUS+DOX	CPR+FUS+TMP	CPR+DOX+TMP	FUS+DOX+TMP	AMP+CPR+FUS+DOX	AMP+CPR+FUS+TMP	AMP+CPR+DOX+TMP	AMP+FUS+DOX+TMP	CPR+FUS+DOX+TMP	AMP+CPR+FUS+DOX+TMP
	72.70226278	87.25137076	72.56876735	92.53710799	98.11649728	65.25596644	21.09172608	71.23501227	78.57126482	69.63224667	94.59267246	89.89423937	82.2592856	73.74337562	103.9895386	38.6987322	75.6367638	53.92145625	29.01487884	28.84981315	61.844609	76.6798963	73.15607149	86.95771869	79.32276492	18.45067516	37.65331622	60.85421491	26.50221234	77.26720044	51.86730553
	69.27274016	87.38194182	80.73290518	89.19531545	99.16887042	68.66806812	41.81685051	69.87741219	67.45872405	79.52398942	87.38194182	93.72874953	86.77748394	98.56441254	98.56441254	49.979923	69.57507617	65.47464393	30.74379384	41.02321846	80.45917283	71.06157915	87.07971288	91.00868908	99.47109936	27.60705766	41.81685051	64.73769988	29.11873775	66.83037401	48.4115549
	75.20205731	92.5430951	93.47278714	100.9103235	102.4598102	64.63997061	30.52902278	64.93387215	66.40337987	99.98063142	99.36083672	85.41545613	100.6004261	94.09258183	91.92330041	38.17046289	75.86333578	77.5716385	44.63629684	20.24246877	62.56429096	99.98063142	89.44412163	86.34514817	94.71237653	29.05951506	25.238795	60.87435709	37.58265981	95.95196591	54.33504776
AMP2CPR1FUS1DOX3TMP1	AMP	CPR	FUS	DOX	TMP	AMP+CPR	AMP+FUS	AMP+DOX	AMP+TMP	CPR+FUS	CPR+DOX	CPR+TMP	FUS+DOX	FUS+TMP	DOX+TMP	AMP+CPR+FUS	AMP+CPR+DOX	AMP+CPR+TMP	AMP+FUS+DOX	AMP+FUS+TMP	AMP+DOX+TMP	CPR+FUS+DOX	CPR+FUS+TMP	CPR+DOX+TMP	FUS+DOX+TMP	AMP+CPR+FUS+DOX	AMP+CPR+FUS+TMP	AMP+CPR+DOX+TMP	AMP+FUS+DOX+TMP	CPR+FUS+DOX+TMP	AMP+CPR+FUS+DOX+TMP
	72.70226278	87.25137076	72.56876735	103.4022345	99.29110555	65.25596644	21.09172608	71.82191247	82.67956624	69.63224667	98.41014935	91.06884765	87.8386749	84.90215421	106.6324073	38.6987322	78.27781471	54.80180655	27.54762833	38.4052821	73.28916298	75.21163596	67.87033426	88.13232696	81.96563353	28.26291295	33.83846489	66.42976684	27.54762833	68.4576384	30.77557945
	69.27274016	87.38194182	80.73290518	94.93766528	98.2621836	68.66806812	41.81685051	64.73769988	73.80778043	79.52398942	84.0574235	85.56856819	94.93766528	76.80392898	100.6800151	49.979923	67.76106007	49.75317099	31.95313792	38.3021943	82.87786097	81.03513411	94.93766528	87.68417076	94.93766528	31.53742589	45.44488273	75.92413256	40.90984245	71.06157915	34.86312209
	75.20205731	92.5430951	93.47278714	112.6864226	93.47278714	64.63997061	30.52902278	58.81704629	60.21307862	99.98063142	95.95196591	99.36083672	106.1785783	101.2202208	108.6577571	38.17046289	68.16678913	48.1814842	44.04849375	46.10580456	62.56429096	92.82599245	103.3895022	95.95196591	80.76699593	29.64731815	34.34974284	59.40484938	28.47171198	81.38679063	38.75826598
AMP2CPR1FUS1DOX3TMP2	AMP	CPR	FUS	DOX	TMP	AMP+CPR	AMP+FUS	AMP+DOX	AMP+TMP	CPR+FUS	CPR+DOX	CPR+TMP	FUS+DOX	FUS+TMP	DOX+TMP	AMP+CPR+FUS	AMP+CPR+DOX	AMP+CPR+TMP	AMP+FUS+DOX	AMP+FUS+TMP	AMP+DOX+TMP	CPR+FUS+DOX	CPR+FUS+TMP	CPR+DOX+TMP	FUS+DOX+TMP	AMP+CPR+FUS+DOX	AMP+CPR+FUS+TMP	AMP+CPR+DOX+TMP	AMP+FUS+DOX+TMP	CPR+FUS+DOX+TMP	AMP+CPR+FUS+DOX+TMP
	72.70226278	87.25137076	72.56876735	103.4022345	94.59267246	65.25596644	21.09172608	71.82191247	72.40881267	69.63224667	98.41014935	95.1799766	87.8386749	94.59267246	102.5212783	38.6987322	78.27781471	55.68215686	27.54762833	41.04633302	69.76776176	75.21163596	79.32276492	86.95771869	98.11649728	28.26291295	38.111832	61.5511589	35.76423118	76.38624424	53.33455604
	69.27274016	87.38194182	80.73290518	94.93766528	92.51983377	68.66806812	41.81685051	64.73769988	66.85405201	79.52398942	84.0574235	90.70646014	94.93766528	102.7956177	92.21760484	49.979923	67.76106007	81.06384486	31.95313792	51.00030706	74.41245246	81.03513411	107.9335096	89.19531545	89.79977333	31.53742589	46.04955476	66.24937997	36.97947422	70.15489233	55.0629473
	75.20205731	92.5430951	93.47278714	112.6864226	99.36083672	64.63997061	30.52902278	58.81704629	60.23144747	99.98063142	95.95196591	90.68371102	106.1785783	102.1499128	101.5301181	38.17046289	68.16678913	81.09845702	44.04849375	51.10213079	77.27773696	92.82599245	97.81134999	95.33217122	95.64206856	29.64731815	28.76561352	54.70242469	24.65099192	81.07689328	41.71565026
AMP2CPR1FUS1DOX3TMP3	AMP	CPR	FUS	DOX	TMP	AMP+CPR	AMP+FUS	AMP+DOX	AMP+TMP	CPR+FUS	CPR+DOX	CPR+TMP	FUS+DOX	FUS+TMP	DOX+TMP	AMP+CPR+FUS	AMP+CPR+DOX	AMP+CPR+TMP	AMP+FUS+DOX	AMP+FUS+TMP	AMP+DOX+TMP	CPR+FUS+DOX	CPR+FUS+TMP	CPR+DOX+TMP	FUS+DOX+TMP	AMP+CPR+FUS+DOX	AMP+CPR+FUS+TMP	AMP+CPR+DOX+TMP	AMP+FUS+DOX+TMP	CPR+FUS+DOX+TMP	AMP+CPR+FUS+DOX+TMP
	72.70226278	87.25137076	72.56876735	103.4022345	98.11649728	65.25596644	21.09172608	71.82191247	78.57126482	69.63224667	98.41014935	89.89423937	87.8386749	73.74337562	91.65615178	38.6987322	78.27781471	53.92145625	27.54762833	28.84981315	76.5171141	75.21163596	73.15607149	104.8704948	76.97354837	28.26291295	37.65331622	72.99571288	27.21749696	86.95771869	31.65592976
	69.27274016	87.38194182	80.73290518	94.93766528	99.16887042	68.66806812	41.81685051	64.73769988	67.45872405	79.52398942	84.0574235	93.72874953	94.93766528	98.56441254	94.03097847	49.979923	67.76106007	65.47464393	31.95313792	41.02321846	70.48208423	81.03513411	87.07971288	97.95995467	82.84850774	31.53742589	41.81685051	75.92413256	21.86267331	86.47525501	22.16500933
	75.20205731	92.5430951	93.47278714	112.6864226	102.4598102	64.63997061	30.52902278	58.81704629	66.40337987	99.98063142	95.95196591	85.41545613	106.1785783	94.09258183	101.5301181	38.17046289	68.16678913	77.5716385	44.04849375	20.24246877	72.92432035	92.82599245	89.44412163	93.16288979	80.45709859	29.64731815	25.238795	55.58412932	20.31594416	88.82432694	53.45334313
AMP2CPR1FUS2DOX1TMP1	AMP	CPR	FUS	DOX	TMP	AMP+CPR	AMP+FUS	AMP+DOX	AMP+TMP	CPR+FUS	CPR+DOX	CPR+TMP	FUS+DOX	FUS+TMP	DOX+TMP	AMP+CPR+FUS	AMP+CPR+DOX	AMP+CPR+TMP	AMP+FUS+DOX	AMP+FUS+TMP	AMP+DOX+TMP	CPR+FUS+DOX	CPR+FUS+TMP	CPR+DOX+TMP	FUS+DOX+TMP	AMP+CPR+FUS+DOX	AMP+CPR+FUS+TMP	AMP+CPR+DOX+TMP	AMP+FUS+DOX+TMP	CPR+FUS+DOX+TMP	AMP+CPR+FUS+DOX+TMP
	72.70226278	87.25137076	97.64552145	88.42597903	99.29110555	65.25596644	41.76161764	74.46296339	82.67956624	91.36249971	87.8386749	91.06884765	90.8961691	99.11277196	96.94188901	41.46816754	60.67080859	54.80180655	27.96946285	45.15463445	74.75641349	99.29110555	92.83076005	95.47362867	97.64552145	38.07515074	41.17471744	61.5511589	50.85857081	100.1720618	30.61051377
	69.27274016	87.38194182	108.8787585	90.40423121	98.2621836	68.66806812	70.53877223	63.3582918	73.80778043	87.38194182	90.10200227	85.56856819	79.24982875	90.43626143	92.82206271	51.79393911	78.3428207	49.75317099	40.24848241	46.76760281	64.73769988	95.54212316	102.1911598	97.95995467	91.34326948	49.79096299	39.9461464	56.10222737	61.58206769	121.8360408	41.21217847
	75.20205731	92.5430951	96.6752388	95.95196591	93.47278714	64.63997061	59.62527553	71.45481264	60.21307862	107.1082704	96.5717606	99.36083672	56.39235856	90.79720794	108.9676545	39.93387215	83.21087436	48.1814842	28.17781043	35.81925055	59.64364438	114.5458067	106.1785783	84.79566144	101.0837619	49.33872153	47.29977957	66.40337987	64.93387215	111.7567306	37.8949302
AMP2CPR1FUS2DOX1TMP2	AMP	CPR	FUS	DOX	TMP	AMP+CPR	AMP+FUS	AMP+DOX	AMP+TMP	CPR+FUS	CPR+DOX	CPR+TMP	FUS+DOX	FUS+TMP	DOX+TMP	AMP+CPR+FUS	AMP+CPR+DOX	AMP+CPR+TMP	AMP+FUS+DOX	AMP+FUS+TMP	AMP+DOX+TMP	CPR+FUS+DOX	CPR+FUS+TMP	CPR+DOX+TMP	FUS+DOX+TMP	AMP+CPR+FUS+DOX	AMP+CPR+FUS+TMP	AMP+CPR+DOX+TMP	AMP+FUS+DOX+TMP	CPR+FUS+DOX+TMP	AMP+CPR+FUS+DOX+TMP
	72.70226278	87.25137076	97.64552145	88.42597903	94.59267246	65.25596644	41.76161764	74.46296339	72.40881267	91.36249971	87.8386749	95.1799766	90.8961691	92.65686971	99.58475762	41.46816754	60.67080859	55.68215686	27.96946285	47.63061968	80.03851533	99.29110555	97.52919315	98.70380142	101.7538229	38.07515074	51.73892111	66.53981063	51.73892111	89.30693524	51.15202091
	69.27274016	87.38194182	108.8787585	90.40423121	92.51983377	68.66806812	70.53877223	63.3582918	66.85405201	87.38194182	90.10200227	90.70646014	79.24982875	89.52925337	96.14658104	51.79393911	78.3428207	81.06384486	40.24848241	57.95403548	65.34237192	95.54212316	112.7691726	106.120136	88.3199093	49.79096299	41.21217847	60.20265961	55.23301131	107.3290518	42.83723457
	75.20205731	92.5430951	96.6752388	95.95196591	99.36083672	64.63997061	59.62527553	71.45481264	60.23144747	107.1082704	96.5717606	90.68371102	56.39235856	83.44966936	100.6004261	39.93387215	83.21087436	81.09845702	28.17781043	46.71197649	61.09478325	114.5458067	105.2488863	91.61340306	101.0837619	49.33872153	35.81925055	51.12049963	70.49963262	100.2905288	50.53269655
AMP2CPR1FUS2DOX1TMP3	AMP	CPR	FUS	DOX	TMP	AMP+CPR	AMP+FUS	AMP+DOX	AMP+TMP	CPR+FUS	CPR+DOX	CPR+TMP	FUS+DOX	FUS+TMP	DOX+TMP	AMP+CPR+FUS	AMP+CPR+DOX	AMP+CPR+TMP	AMP+FUS+DOX	AMP+FUS+TMP	AMP+DOX+TMP	CPR+FUS+DOX	CPR+FUS+TMP	CPR+DOX+TMP	FUS+DOX+TMP	AMP+CPR+FUS+DOX	AMP+CPR+FUS+TMP	AMP+CPR+DOX+TMP	AMP+FUS+DOX+TMP	CPR+FUS+DOX+TMP	AMP+CPR+FUS+DOX+TMP
	72.70226278	87.25137076	97.64552145	88.42597903	98.11649728	65.25596644	41.76161764	74.46296339	78.57126482	91.36249971	87.8386749	89.89423937	90.8961691	101.1669227	99.29110555	41.46816754	60.67080859	53.92145625	27.96946285	58.78172356	75.9302139	99.29110555	114.267361	99.29110555	94.41757032	38.07515074	58.78172356	61.73456521	57.90137325	101.34667	53.79307183
	69.27274016	87.38194182	108.8787585	90.40423121	99.16887042	68.66806812	70.53877223	63.3582918	67.45872405	87.38194182	90.10200227	93.72874953	79.24982875	94.06429364	99.16887042	51.79393911	78.3428207	65.47464393	40.24848241	59.76805159	65.94704396	95.54212316	119.1159804	104.9112202	73.50544441	49.79096299	48.77057893	57.78397147	70.95448426	111.8624858	60.37272362
	75.20205731	92.5430951	96.6752388	95.95196591	102.4598102	64.63997061	59.62527553	71.45481264	66.40337987	107.1082704	96.5717606	85.41545613	56.39235856	96.96914034	104.938989	39.93387215	83.21087436	77.5716385	28.17781043	45.83027186	62.56429096	114.5458067	102.4598102	86.03525082	87.85819251	49.33872153	35.52534901	57.29243204	66.09110948	106.1785783	57.29243204
AMP2CPR1FUS2DOX2TMP1	AMP	CPR	FUS	DOX	TMP	AMP+CPR	AMP+FUS	AMP+DOX	AMP+TMP	CPR+FUS	CPR+DOX	CPR+TMP	FUS+DOX	FUS+TMP	DOX+TMP	AMP+CPR+FUS	AMP+CPR+DOX	AMP+CPR+TMP	AMP+FUS+DOX	AMP+FUS+TMP	AMP+DOX+TMP	CPR+FUS+DOX	CPR+FUS+TMP	CPR+DOX+TMP	FUS+DOX+TMP	AMP+CPR+FUS+DOX	AMP+CPR+FUS+TMP	AMP+CPR+DOX+TMP	AMP+FUS+DOX+TMP	CPR+FUS+DOX+TMP	AMP+CPR+FUS+DOX+TMP
	72.70226278	87.25137076	97.64552145	92.53710799	99.29110555	65.25596644	41.76161764	71.23501227	82.67956624	91.36249971	94.59267246	91.06884765	107.6228249	99.11277196	100.7593659	41.46816754	75.6367638	54.80180655	28.13452853	45.15463445	76.5171141	91.36249971	92.83076005	101.053018	96.76517114	38.111832	41.17471744	58.91010798	50.5651207	86.37041455	46.75026938
	69.27274016	87.38194182	108.8787585	89.19531545	98.2621836	68.66806812	70.53877223	69.87741219	73.80778043	87.38194182	87.38194182	85.56856819	105.5530623	90.43626143	97.35549679	51.79393911	69.57507617	49.75317099	36.79051421	46.76760281	65.34237192	90.10200227	102.1911598	107.9335096	101.3203581	52.81432317	39.9461464	54.89288329	55.11963531	80.73290518	64.30309186
	75.20205731	92.5430951	96.6752388	100.9103235	93.47278714	64.63997061	59.62527553	64.93387215	60.21307862	107.1082704	99.36083672	99.36083672	90.79720794	90.79720794	104.0092969	39.93387215	75.86333578	48.1814842	31.11682586	35.81925055	70.49963262	113.6161147	106.1785783	92.5430951	96.6752388	34.34974284	47.29977957	59.93754592	69.03012491	89.75401898	34.36811168
AMP2CPR1FUS2DOX2TMP2	AMP	CPR	FUS	DOX	TMP	AMP+CPR	AMP+FUS	AMP+DOX	AMP+TMP	CPR+FUS	CPR+DOX	CPR+TMP	FUS+DOX	FUS+TMP	DOX+TMP	AMP+CPR+FUS	AMP+CPR+DOX	AMP+CPR+TMP	AMP+FUS+DOX	AMP+FUS+TMP	AMP+DOX+TMP	CPR+FUS+DOX	CPR+FUS+TMP	CPR+DOX+TMP	FUS+DOX+TMP	AMP+CPR+FUS+DOX	AMP+CPR+FUS+TMP	AMP+CPR+DOX+TMP	AMP+FUS+DOX+TMP	CPR+FUS+DOX+TMP	AMP+CPR+FUS+DOX+TMP
	72.70226278	87.25137076	97.64552145	92.53710799	94.59267246	65.25596644	41.76161764	71.23501227	72.40881267	91.36249971	94.59267246	95.1799766	107.6228249	92.65686971	103.6958866	41.46816754	75.6367638	55.68215686	28.13452853	47.63061968	72.11536257	91.36249971	97.52919315	86.07676249	99.11277196	38.111832	51.73892111	72.00531878	53.20617162	88.42597903	40.71620166
	69.27274016	87.38194182	108.8787585	89.19531545	92.51983377	68.66806812	70.53877223	69.87741219	66.85405201	87.38194182	87.38194182	90.70646014	105.5530623	89.52925337	107.0268228	51.79393911	69.57507617	81.06384486	36.79051421	57.95403548	76.52880459	90.10200227	112.7691726	97.35549679	113.4137988	52.81432317	41.21217847	69.70734818	61.58206769	93.72874953	52.03958712
	75.20205731	92.5430951	96.6752388	100.9103235	99.36083672	64.63997061	59.62527553	64.93387215	60.23144747	107.1082704	99.36083672	90.68371102	90.79720794	83.44966936	89.13422429	39.93387215	75.86333578	81.09845702	31.11682586	46.71197649	79.02277737	113.6161147	105.2488863	86.03525082	102.2593681	34.34974284	35.81925055	71.98750918	64.32770022	97.81134999	48.47538575
AMP2CPR1FUS2DOX2TMP3	AMP	CPR	FUS	DOX	TMP	AMP+CPR	AMP+FUS	AMP+DOX	AMP+TMP	CPR+FUS	CPR+DOX	CPR+TMP	FUS+DOX	FUS+TMP	DOX+TMP	AMP+CPR+FUS	AMP+CPR+DOX	AMP+CPR+TMP	AMP+FUS+DOX	AMP+FUS+TMP	AMP+DOX+TMP	CPR+FUS+DOX	CPR+FUS+TMP	CPR+DOX+TMP	FUS+DOX+TMP	AMP+CPR+FUS+DOX	AMP+CPR+FUS+TMP	AMP+CPR+DOX+TMP	AMP+FUS+DOX+TMP	CPR+FUS+DOX+TMP	AMP+CPR+FUS+DOX+TMP
	72.70226278	87.25137076	97.64552145	92.53710799	98.11649728	65.25596644	41.76161764	71.23501227	78.57126482	91.36249971	94.59267246	89.89423937	107.6228249	101.1669227	103.9895386	41.46816754	75.6367638	53.92145625	28.13452853	58.78172356	61.844609	91.36249971	114.267361	86.95771869	110.850776	38.111832	58.78172356	60.85421491	48.96948577	74.62433183	53.20617162
	69.27274016	87.38194182	108.8787585	89.19531545	99.16887042	68.66806812	70.53877223	69.87741219	67.45872405	87.38194182	87.38194182	93.72874953	105.5530623	94.06429364	98.56441254	51.79393911	69.57507617	65.47464393	36.79051421	59.76805159	80.45917283	90.10200227	119.1159804	91.00868908	109.1810946	52.81432317	48.77057893	64.73769988	52.09627513	90.10200227	43.44190661
	75.20205731	92.5430951	96.6752388	100.9103235	102.4598102	64.63997061	59.62527553	64.93387215	66.40337987	107.1082704	99.36083672	85.41545613	90.79720794	96.96914034	91.92330041	39.93387215	75.86333578	77.5716385	31.11682586	45.83027186	62.56429096	113.6161147	102.4598102	86.34514817	105.1983835	34.34974284	35.52534901	60.87435709	43.16678913	96.5717606	35.81925055
AMP2CPR1FUS2DOX3TMP1	AMP	CPR	FUS	DOX	TMP	AMP+CPR	AMP+FUS	AMP+DOX	AMP+TMP	CPR+FUS	CPR+DOX	CPR+TMP	FUS+DOX	FUS+TMP	DOX+TMP	AMP+CPR+FUS	AMP+CPR+DOX	AMP+CPR+TMP	AMP+FUS+DOX	AMP+FUS+TMP	AMP+DOX+TMP	CPR+FUS+DOX	CPR+FUS+TMP	CPR+DOX+TMP	FUS+DOX+TMP	AMP+CPR+FUS+DOX	AMP+CPR+FUS+TMP	AMP+CPR+DOX+TMP	AMP+FUS+DOX+TMP	CPR+FUS+DOX+TMP	AMP+CPR+FUS+DOX+TMP
	72.70226278	87.25137076	97.64552145	103.4022345	99.29110555	65.25596644	41.76161764	71.82191247	82.67956624	91.36249971	98.41014935	91.06884765	98.52587175	99.11277196	106.6324073	41.46816754	78.27781471	54.80180655	57.02102295	45.15463445	73.28916298	95.76728074	92.83076005	88.13232696	117.3066783	40.00091703	41.17471744	66.42976684	57.73630757	58.47346808	45.11795319
	69.27274016	87.38194182	108.8787585	94.93766528	98.2621836	68.66806812	70.53877223	64.73769988	73.80778043	87.38194182	84.0574235	85.56856819	100.715686	90.43626143	100.6800151	51.79393911	67.76106007	49.75317099	54.02366724	46.76760281	82.87786097	103.4000756	102.1911598	87.68417076	103.7390462	41.6278905	39.9461464	75.92413256	51.30264308	78.61730261	70.48208423
	75.20205731	92.5430951	96.6752388	112.6864226	93.47278714	64.63997061	59.62527553	58.81704629	60.21307862	107.1082704	95.95196591	99.36083672	95.49963262	90.79720794	108.6577571	39.93387215	68.16678913	48.1814842	53.15944159	35.81925055	62.56429096	107.4181677	106.1785783	95.95196591	92.26671565	70.79353417	47.29977957	59.40484938	53.45334313	103.3895022	49.35709037
AMP2CPR1FUS2DOX3TMP2	AMP	CPR	FUS	DOX	TMP	AMP+CPR	AMP+FUS	AMP+DOX	AMP+TMP	CPR+FUS	CPR+DOX	CPR+TMP	FUS+DOX	FUS+TMP	DOX+TMP	AMP+CPR+FUS	AMP+CPR+DOX	AMP+CPR+TMP	AMP+FUS+DOX	AMP+FUS+TMP	AMP+DOX+TMP	CPR+FUS+DOX	CPR+FUS+TMP	CPR+DOX+TMP	FUS+DOX+TMP	AMP+CPR+FUS+DOX	AMP+CPR+FUS+TMP	AMP+CPR+DOX+TMP	AMP+FUS+DOX+TMP	CPR+FUS+DOX+TMP	AMP+CPR+FUS+DOX+TMP
	72.70226278	87.25137076	97.64552145	103.4022345	94.59267246	65.25596644	41.76161764	71.82191247	72.40881267	91.36249971	98.41014935	95.1799766	98.52587175	92.65686971	102.5212783	41.46816754	78.27781471	55.68215686	57.02102295	47.63061968	69.76776176	95.76728074	97.52919315	86.95771869	117.8935785	40.00091703	51.73892111	61.5511589	36.64458149	71.98146321	31.49086407
	69.27274016	87.38194182	108.8787585	94.93766528	92.51983377	68.66806812	70.53877223	64.73769988	66.85405201	87.38194182	84.0574235	90.70646014	100.715686	89.52925337	92.21760484	51.79393911	67.76106007	81.06384486	54.02366724	57.95403548	74.41245246	103.4000756	112.7691726	89.19531545	106.4600704	41.6278905	41.21217847	66.24937997	42.42152255	85.56856819	38.43446631
	75.20205731	92.5430951	96.6752388	112.6864226	99.36083672	64.63997061	59.62527553	58.81704629	60.23144747	107.1082704	95.95196591	90.68371102	95.49963262	83.44966936	101.5301181	39.93387215	68.16678913	81.09845702	53.15944159	46.71197649	77.27773696	107.4181677	105.2488863	95.33217122	87.85819251	70.79353417	35.81925055	54.70242469	42.87288758	113.926012	45.51800147
AMP2CPR1FUS2DOX3TMP3	AMP	CPR	FUS	DOX	TMP	AMP+CPR	AMP+FUS	AMP+DOX	AMP+TMP	CPR+FUS	CPR+DOX	CPR+TMP	FUS+DOX	FUS+TMP	DOX+TMP	AMP+CPR+FUS	AMP+CPR+DOX	AMP+CPR+TMP	AMP+FUS+DOX	AMP+FUS+TMP	AMP+DOX+TMP	CPR+FUS+DOX	CPR+FUS+TMP	CPR+DOX+TMP	FUS+DOX+TMP	AMP+CPR+FUS+DOX	AMP+CPR+FUS+TMP	AMP+CPR+DOX+TMP	AMP+FUS+DOX+TMP	CPR+FUS+DOX+TMP	AMP+CPR+FUS+DOX+TMP
	72.70226278	87.25137076	97.64552145	103.4022345	98.11649728	65.25596644	41.76161764	71.82191247	78.57126482	91.36249971	98.41014935	89.89423937	98.52587175	101.1669227	91.65615178	41.46816754	78.27781471	53.92145625	57.02102295	58.78172356	76.5171141	95.76728074	114.267361	104.8704948	115.2525276	40.00091703	58.78172356	72.99571288	61.88129026	101.34667	48.51096999
	69.27274016	87.38194182	108.8787585	94.93766528	99.16887042	68.66806812	70.53877223	64.73769988	67.45872405	87.38194182	84.0574235	93.72874953	100.715686	94.06429364	94.03097847	51.79393911	67.76106007	65.47464393	54.02366724	59.76805159	70.48208423	103.4000756	119.1159804	97.95995467	107.0647424	41.6278905	48.77057893	75.92413256	53.11665919	107.0268228	54.76061129
	75.20205731	92.5430951	96.6752388	112.6864226	102.4598102	64.63997061	59.62527553	58.81704629	66.40337987	107.1082704	95.95196591	85.41545613	95.49963262	96.96914034	101.5301181	39.93387215	68.16678913	77.5716385	53.15944159	45.83027186	72.92432035	107.4181677	102.4598102	93.16288979	94.03012491	70.79353417	35.52534901	55.58412932	49.04481999	117.6447802	48.75091844
AMP2CPR1FUS3DOX1TMP1	AMP	CPR	FUS	DOX	TMP	AMP+CPR	AMP+FUS	AMP+DOX	AMP+TMP	CPR+FUS	CPR+DOX	CPR+TMP	FUS+DOX	FUS+TMP	DOX+TMP	AMP+CPR+FUS	AMP+CPR+DOX	AMP+CPR+TMP	AMP+FUS+DOX	AMP+FUS+TMP	AMP+DOX+TMP	CPR+FUS+DOX	CPR+FUS+TMP	CPR+DOX+TMP	FUS+DOX+TMP	AMP+CPR+FUS+DOX	AMP+CPR+FUS+TMP	AMP+CPR+DOX+TMP	AMP+FUS+DOX+TMP	CPR+FUS+DOX+TMP	AMP+CPR+FUS+DOX+TMP
	72.70226278	87.25137076	95.90014577	88.42597903	99.29110555	65.25596644	60.54242417	74.46296339	82.67956624	96.64823694	87.8386749	91.06884765	77.53279883	72.8680758	96.94188901	63.01840941	60.67080859	54.80180655	65.23762581	72.70226278	74.75641349	98.70380142	102.8149304	95.47362867	76.94970845	65.65946033	64.48565992	61.5511589	69.47431165	99.87840969	61.2577088
	69.27274016	87.38194182	92.29679476	90.40423121	98.2621836	68.66806812	69.14046815	63.3582918	73.80778043	99.773283	90.10200227	85.56856819	78.69665309	54.89640517	92.82206271	61.7143397	78.3428207	49.75317099	66.41944399	77.73814866	64.73769988	93.42652059	106.7245939	97.95995467	83.51336993	58.86104353	62.31901174	56.10222737	71.6914283	120.627125	60.50499563
	75.20205731	92.5430951	84.78536497	95.95196591	93.47278714	64.63997061	64.91550331	71.45481264	60.21307862	106.4884757	96.5717606	99.36083672	94.63865242	89.42220612	108.9676545	56.11682586	83.21087436	48.1814842	66.67891256	54.35341661	59.64364438	101.2202208	104.938989	84.79566144	93.47944213	64.93387215	56.09845702	66.40337987	69.63629684	102.4598102	86.38868479
AMP2CPR1FUS3DOX1TMP2	AMP	CPR	FUS	DOX	TMP	AMP+CPR	AMP+FUS	AMP+DOX	AMP+TMP	CPR+FUS	CPR+DOX	CPR+TMP	FUS+DOX	FUS+TMP	DOX+TMP	AMP+CPR+FUS	AMP+CPR+DOX	AMP+CPR+TMP	AMP+FUS+DOX	AMP+FUS+TMP	AMP+DOX+TMP	CPR+FUS+DOX	CPR+FUS+TMP	CPR+DOX+TMP	FUS+DOX+TMP	AMP+CPR+FUS+DOX	AMP+CPR+FUS+TMP	AMP+CPR+DOX+TMP	AMP+FUS+DOX+TMP	CPR+FUS+DOX+TMP	AMP+CPR+FUS+DOX+TMP
	72.70226278	87.25137076	95.90014577	88.42597903	94.59267246	65.25596644	60.54242417	74.46296339	72.40881267	96.64823694	87.8386749	95.1799766	77.53279883	57.99927114	99.58475762	63.01840941	60.67080859	55.68215686	65.23762581	72.40881267	80.03851533	98.70380142	95.1799766	98.70380142	87.44533528	65.65946033	52.61927142	66.53981063	60.54242417	109.5689279	48.05245421
	69.27274016	87.38194182	92.29679476	90.40423121	92.51983377	68.66806812	69.14046815	63.3582918	66.85405201	99.773283	90.10200227	90.70646014	78.69665309	56.59642288	96.14658104	61.7143397	78.3428207	81.06384486	66.41944399	63.52835581	65.34237192	93.42652059	109.7468833	106.120136	71.0465734	58.86104353	50.22557101	60.20265961	62.18673973	122.7427276	52.64425916
	75.20205731	92.5430951	84.78536497	95.95196591	99.36083672	64.63997061	64.91550331	71.45481264	60.23144747	106.4884757	96.5717606	90.68371102	94.63865242	90.87121898	100.6004261	56.11682586	83.21087436	81.09845702	66.67891256	60.8008817	61.09478325	101.2202208	88.82432694	91.61340306	90.87121898	64.93387215	70.22409993	51.12049963	69.32402645	108.0379624	69.63629684
AMP2CPR1FUS3DOX1TMP3	AMP	CPR	FUS	DOX	TMP	AMP+CPR	AMP+FUS	AMP+DOX	AMP+TMP	CPR+FUS	CPR+DOX	CPR+TMP	FUS+DOX	FUS+TMP	DOX+TMP	AMP+CPR+FUS	AMP+CPR+DOX	AMP+CPR+TMP	AMP+FUS+DOX	AMP+FUS+TMP	AMP+DOX+TMP	CPR+FUS+DOX	CPR+FUS+TMP	CPR+DOX+TMP	FUS+DOX+TMP	AMP+CPR+FUS+DOX	AMP+CPR+FUS+TMP	AMP+CPR+DOX+TMP	AMP+FUS+DOX+TMP	CPR+FUS+DOX+TMP	AMP+CPR+FUS+DOX+TMP
	72.70226278	87.25137076	95.90014577	88.42597903	98.11649728	65.25596644	60.54242417	74.46296339	78.57126482	96.64823694	87.8386749	89.89423937	77.53279883	66.74562682	99.29110555	63.01840941	60.67080859	53.92145625	65.23762581	58.91010798	75.9302139	98.70380142	108.3943197	99.29110555	83.65524781	65.65946033	57.90137325	61.73456521	70.51972764	106.3387552	47.04371948
	69.27274016	87.38194182	92.29679476	90.40423121	99.16887042	68.66806812	69.14046815	63.3582918	67.45872405	99.773283	90.10200227	93.72874953	78.69665309	65.09651142	99.16887042	61.7143397	78.3428207	65.47464393	66.41944399	63.22601979	65.94704396	93.42652059	119.4182093	104.9112202	73.59659996	58.86104353	49.31856296	57.78397147	64.60542788	114.5825463	57.65169946
	75.20205731	92.5430951	84.78536497	95.95196591	102.4598102	64.63997061	64.91550331	71.45481264	66.40337987	106.4884757	96.5717606	85.41545613	94.63865242	81.88733925	104.938989	56.11682586	83.21087436	77.5716385	66.67891256	58.17413666	62.56429096	101.2202208	109.2775518	86.03525082	75.07697881	64.93387215	79.04114622	57.29243204	67.85451874	105.868681	42.2850845
AMP2CPR1FUS3DOX2TMP1	AMP	CPR	FUS	DOX	TMP	AMP+CPR	AMP+FUS	AMP+DOX	AMP+TMP	CPR+FUS	CPR+DOX	CPR+TMP	FUS+DOX	FUS+TMP	DOX+TMP	AMP+CPR+FUS	AMP+CPR+DOX	AMP+CPR+TMP	AMP+FUS+DOX	AMP+FUS+TMP	AMP+DOX+TMP	CPR+FUS+DOX	CPR+FUS+TMP	CPR+DOX+TMP	FUS+DOX+TMP	AMP+CPR+FUS+DOX	AMP+CPR+FUS+TMP	AMP+CPR+DOX+TMP	AMP+FUS+DOX+TMP	CPR+FUS+DOX+TMP	AMP+CPR+FUS+DOX+TMP
	72.70226278	87.25137076	95.90014577	92.53710799	99.29110555	65.25596644	60.54242417	71.23501227	82.67956624	96.64823694	94.59267246	91.06884765	91.52696793	72.8680758	100.7593659	63.01840941	75.6367638	54.80180655	66.53981063	72.70226278	76.5171141	103.6958866	102.8149304	101.053018	64.59548105	46.29175359	64.48565992	58.91010798	65.53107591	93.41806419	55.97560696
	69.27274016	87.38194182	92.29679476	89.19531545	98.2621836	68.66806812	69.14046815	69.87741219	73.80778043	99.773283	87.38194182	85.56856819	68.49654684	54.89640517	97.35549679	61.7143397	69.57507617	49.75317099	78.3428207	77.73814866	65.34237192	95.23989422	106.7245939	107.9335096	73.0299274	52.34192314	62.31901174	54.89288329	66.11710797	115.791462	55.66761934
	75.20205731	92.5430951	84.78536497	100.9103235	93.47278714	64.63997061	64.91550331	64.93387215	60.21307862	106.4884757	99.36083672	99.36083672	73.62796595	89.42220612	104.0092969	56.11682586	75.86333578	48.1814842	47.88758266	54.35341661	70.49963262	106.4884757	104.938989	92.5430951	101.8837167	56.41072741	56.09845702	59.93754592	83.72520206	107.7280651	57.88023512
AMP2CPR1FUS3DOX2TMP2	AMP	CPR	FUS	DOX	TMP	AMP+CPR	AMP+FUS	AMP+DOX	AMP+TMP	CPR+FUS	CPR+DOX	CPR+TMP	FUS+DOX	FUS+TMP	DOX+TMP	AMP+CPR+FUS	AMP+CPR+DOX	AMP+CPR+TMP	AMP+FUS+DOX	AMP+FUS+TMP	AMP+DOX+TMP	CPR+FUS+DOX	CPR+FUS+TMP	CPR+DOX+TMP	FUS+DOX+TMP	AMP+CPR+FUS+DOX	AMP+CPR+FUS+TMP	AMP+CPR+DOX+TMP	AMP+FUS+DOX+TMP	CPR+FUS+DOX+TMP	AMP+CPR+FUS+DOX+TMP
	72.70226278	87.25137076	95.90014577	92.53710799	94.59267246	65.25596644	60.54242417	71.23501227	72.40881267	96.64823694	94.59267246	95.1799766	91.52696793	57.99927114	103.6958866	63.01840941	75.6367638	55.68215686	66.53981063	72.40881267	72.11536257	103.6958866	95.1799766	86.07676249	81.61443149	46.29175359	52.61927142	72.00531878	69.47431165	87.54502283	61.5511589
	69.27274016	87.38194182	92.29679476	89.19531545	92.51983377	68.66806812	69.14046815	69.87741219	66.85405201	99.773283	87.38194182	90.70646014	68.49654684	56.59642288	107.0268228	61.7143397	69.57507617	81.06384486	78.3428207	63.52835581	76.52880459	95.23989422	109.7468833	97.35549679	98.24685674	52.34192314	50.22557101	69.70734818	64.13302785	96.44880997	61.7143397
	75.20205731	92.5430951	84.78536497	100.9103235	99.36083672	64.63997061	64.91550331	64.93387215	60.23144747	106.4884757	99.36083672	90.68371102	73.62796595	90.87121898	89.13422429	56.11682586	75.86333578	81.09845702	47.88758266	60.8008817	79.02277737	106.4884757	88.82432694	86.03525082	99.85509871	56.41072741	70.22409993	71.98750918	70.20573108	103.3895022	50.53269655
AMP2CPR1FUS3DOX2TMP3	AMP	CPR	FUS	DOX	TMP	AMP+CPR	AMP+FUS	AMP+DOX	AMP+TMP	CPR+FUS	CPR+DOX	CPR+TMP	FUS+DOX	FUS+TMP	DOX+TMP	AMP+CPR+FUS	AMP+CPR+DOX	AMP+CPR+TMP	AMP+FUS+DOX	AMP+FUS+TMP	AMP+DOX+TMP	CPR+FUS+DOX	CPR+FUS+TMP	CPR+DOX+TMP	FUS+DOX+TMP	AMP+CPR+FUS+DOX	AMP+CPR+FUS+TMP	AMP+CPR+DOX+TMP	AMP+FUS+DOX+TMP	CPR+FUS+DOX+TMP	AMP+CPR+FUS+DOX+TMP
	72.70226278	87.25137076	95.90014577	92.53710799	98.11649728	65.25596644	60.54242417	71.23501227	78.57126482	96.64823694	94.59267246	89.89423937	91.52696793	66.74562682	103.9895386	63.01840941	75.6367638	53.92145625	66.53981063	58.91010798	61.844609	103.6958866	108.3943197	86.95771869	108.4365889	46.29175359	57.90137325	60.85421491	68.00706114	93.12441212	59.79045829
	69.27274016	87.38194182	92.29679476	89.19531545	99.16887042	68.66806812	69.14046815	69.87741219	67.45872405	99.773283	87.38194182	93.72874953	68.49654684	65.09651142	98.56441254	61.7143397	69.57507617	65.47464393	78.3428207	63.22601979	80.45917283	95.23989422	119.4182093	91.00868908	88.61342306	52.34192314	49.31856296	64.73769988	63.83069183	102.7956177	76.83114061
	75.20205731	92.5430951	84.78536497	100.9103235	102.4598102	64.63997061	64.91550331	64.93387215	66.40337987	106.4884757	99.36083672	85.41545613	73.62796595	81.88733925	91.92330041	56.11682586	75.86333578	77.5716385	47.88758266	58.17413666	62.56429096	106.4884757	109.2775518	86.34514817	99.56529614	56.41072741	79.04114622	60.87435709	64.32770022	104.3191943	61.11315209
AMP2CPR1FUS3DOX3TMP1	AMP	CPR	FUS	DOX	TMP	AMP+CPR	AMP+FUS	AMP+DOX	AMP+TMP	CPR+FUS	CPR+DOX	CPR+TMP	FUS+DOX	FUS+TMP	DOX+TMP	AMP+CPR+FUS	AMP+CPR+DOX	AMP+CPR+TMP	AMP+FUS+DOX	AMP+FUS+TMP	AMP+DOX+TMP	CPR+FUS+DOX	CPR+FUS+TMP	CPR+DOX+TMP	FUS+DOX+TMP	AMP+CPR+FUS+DOX	AMP+CPR+FUS+TMP	AMP+CPR+DOX+TMP	AMP+FUS+DOX+TMP	CPR+FUS+DOX+TMP	AMP+CPR+FUS+DOX+TMP
	72.70226278	87.25137076	95.90014577	103.4022345	99.29110555	65.25596644	60.54242417	71.82191247	82.67956624	96.64823694	98.41014935	91.06884765	48.66982507	72.8680758	106.6324073	63.01840941	78.27781471	54.80180655	69.47431165	72.70226278	73.28916298	106.9260593	102.8149304	88.13232696	102.3141399	53.33455604	64.48565992	66.42976684	86.20096747	91.94980385	52.45420574
	69.27274016	87.38194182	92.29679476	94.93766528	98.2621836	68.66806812	69.14046815	64.73769988	73.80778043	99.773283	84.0574235	85.56856819	61.41313972	54.89640517	100.6800151	61.7143397	67.76106007	49.75317099	70.78442025	77.73814866	82.87786097	99.47109936	106.7245939	87.68417076	85.78006021	68.83813213	62.31901174	75.92413256	92.25027754	95.8443521	67.15638803
	75.20205731	92.5430951	84.78536497	112.6864226	93.47278714	64.63997061	64.91550331	58.81704629	60.21307862	106.4884757	95.95196591	99.36083672	68.41151965	89.42220612	108.6577571	56.11682586	68.16678913	48.1814842	69.91182954	54.35341661	62.56429096	104.938989	104.938989	95.95196591	101.5939141	56.6862601	56.09845702	59.40484938	65.50330639	112.99632	48.75091844
AMP2CPR1FUS3DOX3TMP2	AMP	CPR	FUS	DOX	TMP	AMP+CPR	AMP+FUS	AMP+DOX	AMP+TMP	CPR+FUS	CPR+DOX	CPR+TMP	FUS+DOX	FUS+TMP	DOX+TMP	AMP+CPR+FUS	AMP+CPR+DOX	AMP+CPR+TMP	AMP+FUS+DOX	AMP+FUS+TMP	AMP+DOX+TMP	CPR+FUS+DOX	CPR+FUS+TMP	CPR+DOX+TMP	FUS+DOX+TMP	AMP+CPR+FUS+DOX	AMP+CPR+FUS+TMP	AMP+CPR+DOX+TMP	AMP+FUS+DOX+TMP	CPR+FUS+DOX+TMP	AMP+CPR+FUS+DOX+TMP
	72.70226278	87.25137076	95.90014577	103.4022345	94.59267246	65.25596644	60.54242417	71.82191247	72.40881267	96.64823694	98.41014935	95.1799766	48.66982507	57.99927114	102.5212783	63.01840941	78.27781471	55.68215686	69.47431165	72.40881267	69.76776176	106.9260593	95.1799766	86.95771869	98.52405248	53.33455604	52.61927142	61.5511589	65.65946033	92.24345592	55.38870676
	69.27274016	87.38194182	92.29679476	94.93766528	92.51983377	68.66806812	69.14046815	64.73769988	66.85405201	99.773283	84.0574235	90.70646014	61.41313972	56.59642288	92.21760484	61.7143397	67.76106007	81.06384486	70.78442025	63.52835581	74.41245246	99.47109936	109.7468833	89.19531545	88.89675934	68.83813213	50.22557101	66.24937997	61.7143397	111.258028	60.80733165
	75.20205731	92.5430951	84.78536497	112.6864226	99.36083672	64.63997061	64.91550331	58.81704629	60.23144747	106.4884757	95.95196591	90.68371102	68.41151965	90.87121898	101.5301181	56.11682586	68.16678913	81.09845702	69.91182954	60.8008817	77.27773696	104.938989	88.82432694	95.33217122	98.69588843	56.6862601	70.22409993	54.70242469	59.62527553	106.798373	57.86186627
AMP2CPR1FUS3DOX3TMP3	AMP	CPR	FUS	DOX	TMP	AMP+CPR	AMP+FUS	AMP+DOX	AMP+TMP	CPR+FUS	CPR+DOX	CPR+TMP	FUS+DOX	FUS+TMP	DOX+TMP	AMP+CPR+FUS	AMP+CPR+DOX	AMP+CPR+TMP	AMP+FUS+DOX	AMP+FUS+TMP	AMP+DOX+TMP	CPR+FUS+DOX	CPR+FUS+TMP	CPR+DOX+TMP	FUS+DOX+TMP	AMP+CPR+FUS+DOX	AMP+CPR+FUS+TMP	AMP+CPR+DOX+TMP	AMP+FUS+DOX+TMP	CPR+FUS+DOX+TMP	AMP+CPR+FUS+DOX+TMP
	72.70226278	87.25137076	95.90014577	103.4022345	98.11649728	65.25596644	60.54242417	71.82191247	78.57126482	96.64823694	98.41014935	89.89423937	48.66982507	66.74562682	91.65615178	63.01840941	78.27781471	53.92145625	69.47431165	58.91010798	76.5171141	106.9260593	108.3943197	104.8704948	96.48323615	53.33455604	57.90137325	72.99571288	71.82191247	117.2038817	58.32320778
	69.27274016	87.38194182	92.29679476	94.93766528	99.16887042	68.66806812	69.14046815	64.73769988	67.45872405	99.773283	84.0574235	93.72874953	61.41313972	65.09651142	94.03097847	61.7143397	67.76106007	65.47464393	70.78442025	63.22601979	70.48208423	99.47109936	119.4182093	97.95995467	85.78006021	68.83813213	49.31856296	75.92413256	79.55216477	108.8401964	68.06339608
	75.20205731	92.5430951	84.78536497	112.6864226	102.4598102	64.63997061	64.91550331	58.81704629	66.40337987	106.4884757	95.95196591	85.41545613	68.41151965	81.88733925	101.5301181	56.11682586	68.16678913	77.5716385	69.91182954	58.17413666	72.92432035	104.938989	109.2775518	93.16288979	94.34884985	56.6862601	79.04114622	55.58412932	73.16311536	117.3348828	57.27406319
AMP2CPR2FUS1DOX1TMP1	AMP	CPR	FUS	DOX	TMP	AMP+CPR	AMP+FUS	AMP+DOX	AMP+TMP	CPR+FUS	CPR+DOX	CPR+TMP	FUS+DOX	FUS+TMP	DOX+TMP	AMP+CPR+FUS	AMP+CPR+DOX	AMP+CPR+TMP	AMP+FUS+DOX	AMP+FUS+TMP	AMP+DOX+TMP	CPR+FUS+DOX	CPR+FUS+TMP	CPR+DOX+TMP	FUS+DOX+TMP	AMP+CPR+FUS+DOX	AMP+CPR+FUS+TMP	AMP+CPR+DOX+TMP	AMP+FUS+DOX+TMP	CPR+FUS+DOX+TMP	AMP+CPR+FUS+DOX+TMP
	72.70226278	86.38437378	72.56876735	88.42597903	99.29110555	71.23501227	21.09172608	74.46296339	82.67956624	87.66821798	81.50576584	81.50576584	74.33067976	84.90215421	96.94188901	13.71879227	71.82191247	61.5511589	23.86116142	38.4052821	74.75641349	76.5171141	85.32061716	78.27781471	75.7989401	29.01487884	11.37119145	69.76776176	39.28563241	81.50576584	37.94676632
	69.27274016	98.12693389	80.73290518	90.40423121	98.2621836	71.21902827	41.81685051	63.3582918	73.80778043	86.20355717	89.52925337	75.31946052	65.01700038	76.80392898	92.82206271	28.21172969	76.05640456	53.85360323	24.69707348	38.3021943	64.73769988	72.46616435	86.50589319	80.59144884	98.56441254	31.04612986	46.76760281	82.57552495	48.2792829	88.01757328	33.46481801
	75.20205731	85.26818516	93.47278714	95.95196591	93.47278714	67.63409258	30.52902278	71.45481264	60.21307862	76.10213079	89.08890522	76.39603233	63.10284718	101.2202208	108.9676545	11.42542248	83.21087436	45.53637032	31.41072741	46.10580456	59.64364438	78.74724467	66.40337987	73.51212344	86.34514817	37.58265981	47.86921381	73.4570169	31.70462895	62.58265981	28.47171198
AMP2CPR2FUS1DOX1TMP2	AMP	CPR	FUS	DOX	TMP	AMP+CPR	AMP+FUS	AMP+DOX	AMP+TMP	CPR+FUS	CPR+DOX	CPR+TMP	FUS+DOX	FUS+TMP	DOX+TMP	AMP+CPR+FUS	AMP+CPR+DOX	AMP+CPR+TMP	AMP+FUS+DOX	AMP+FUS+TMP	AMP+DOX+TMP	CPR+FUS+DOX	CPR+FUS+TMP	CPR+DOX+TMP	FUS+DOX+TMP	AMP+CPR+FUS+DOX	AMP+CPR+FUS+TMP	AMP+CPR+DOX+TMP	AMP+FUS+DOX+TMP	CPR+FUS+DOX+TMP	AMP+CPR+FUS+DOX+TMP
	72.70226278	86.38437378	72.56876735	88.42597903	94.59267246	71.23501227	21.09172608	74.46296339	72.40881267	87.66821798	81.50576584	73.58261308	74.33067976	94.59267246	99.58475762	13.71879227	71.82191247	62.72495931	23.86116142	41.04633302	80.03851533	76.5171141	77.69091451	80.33196543	69.92589874	29.01487884	46.45681928	71.82191247	17.99215938	83.26646645	49.3913203
	69.27274016	98.12693389	80.73290518	90.40423121	92.51983377	71.21902827	41.81685051	63.3582918	66.85405201	86.20355717	89.52925337	78.94749274	65.01700038	102.7956177	96.14658104	28.21172969	76.05640456	68.06339608	24.69707348	51.00030706	65.34237192	72.46616435	78.04048468	68.49800411	77.40838685	31.04612986	44.23553865	88.62224532	23.67668942	84.69187708	47.67461086
	75.20205731	85.26818516	93.47278714	95.95196591	99.36083672	67.63409258	30.52902278	71.45481264	60.23144747	76.10213079	89.08890522	72.63041881	63.10284718	102.1499128	100.6004261	11.42542248	83.21087436	65.81557678	31.41072741	51.10213079	61.09478325	78.74724467	71.69360764	69.39750184	73.94925431	37.58265981	43.46069067	73.4570169	40.52167524	81.6862601	54.33504776
AMP2CPR2FUS1DOX1TMP3	AMP	CPR	FUS	DOX	TMP	AMP+CPR	AMP+FUS	AMP+DOX	AMP+TMP	CPR+FUS	CPR+DOX	CPR+TMP	FUS+DOX	FUS+TMP	DOX+TMP	AMP+CPR+FUS	AMP+CPR+DOX	AMP+CPR+TMP	AMP+FUS+DOX	AMP+FUS+TMP	AMP+DOX+TMP	CPR+FUS+DOX	CPR+FUS+TMP	CPR+DOX+TMP	FUS+DOX+TMP	AMP+CPR+FUS+DOX	AMP+CPR+FUS+TMP	AMP+CPR+DOX+TMP	AMP+FUS+DOX+TMP	CPR+FUS+DOX+TMP	AMP+CPR+FUS+DOX+TMP
	72.70226278	86.38437378	72.56876735	88.42597903	98.11649728	71.23501227	21.09172608	74.46296339	78.57126482	87.66821798	81.50576584	76.5171141	74.33067976	73.74337562	99.29110555	13.71879227	71.82191247	63.01840941	23.86116142	28.84981315	75.9302139	76.5171141	72.70226278	69.76776176	95.76728074	29.01487884	23.56771132	71.23501227	18.87250969	77.69091451	41.33978312
	69.27274016	98.12693389	80.73290518	90.40423121	99.16887042	71.21902827	41.81685051	63.3582918	67.45872405	86.20355717	89.52925337	76.52880459	65.01700038	98.56441254	99.16887042	28.21172969	76.05640456	67.15638803	24.69707348	41.02321846	65.94704396	72.46616435	92.25027754	76.96341262	111.258028	31.04612986	24.88603349	84.9942131	5.536528332	82.57552495	43.02619458
	75.20205731	85.26818516	93.47278714	95.95196591	102.4598102	67.63409258	30.52902278	71.45481264	66.40337987	76.10213079	89.08890522	69.10360029	63.10284718	94.09258183	104.938989	11.42542248	83.21087436	58.7435709	31.41072741	20.24246877	62.56429096	78.74724467	84.62527553	77.92064658	95.33217122	37.58265981	17.89125643	66.40337987	49.63262307	86.68258633	44.93019838
AMP2CPR2FUS1DOX2TMP1	AMP	CPR	FUS	DOX	TMP	AMP+CPR	AMP+FUS	AMP+DOX	AMP+TMP	CPR+FUS	CPR+DOX	CPR+TMP	FUS+DOX	FUS+TMP	DOX+TMP	AMP+CPR+FUS	AMP+CPR+DOX	AMP+CPR+TMP	AMP+FUS+DOX	AMP+FUS+TMP	AMP+DOX+TMP	CPR+FUS+DOX	CPR+FUS+TMP	CPR+DOX+TMP	FUS+DOX+TMP	AMP+CPR+FUS+DOX	AMP+CPR+FUS+TMP	AMP+CPR+DOX+TMP	AMP+FUS+DOX+TMP	CPR+FUS+DOX+TMP	AMP+CPR+FUS+DOX+TMP
	72.70226278	86.38437378	72.56876735	92.53710799	99.29110555	71.23501227	21.09172608	71.23501227	82.67956624	87.66821798	80.91886563	81.50576584	82.2592856	84.90215421	100.7593659	13.71879227	65.65946033	61.5511589	29.01487884	38.4052821	76.5171141	84.73371696	85.32061716	74.16951329	96.0609328	39.87253261	11.37119145	70.35466196	20.50482588	75.34331369	33.83846489
	69.27274016	98.12693389	80.73290518	89.19531545	98.2621836	71.21902827	41.81685051	69.87741219	73.80778043	86.20355717	70.48208423	75.31946052	86.77748394	76.80392898	97.35549679	28.21172969	57.78397147	53.85360323	30.74379384	38.3021943	65.34237192	94.66896568	86.50589319	78.3428207	101.8889309	35.46779413	46.76760281	87.71523726	27.00238562	87.11056523	39.81387439
	75.20205731	85.26818516	93.47278714	100.9103235	93.47278714	67.63409258	30.52902278	64.93387215	60.21307862	76.10213079	72.86921381	76.39603233	100.6004261	101.2202208	104.0092969	11.42542248	66.16458486	45.53637032	44.63629684	46.10580456	70.49963262	95.20573108	66.40337987	64.05216752	73.32945962	39.05216752	47.86921381	71.3997061	23.1814842	81.09845702	26.12049963
AMP2CPR2FUS1DOX2TMP2	AMP	CPR	FUS	DOX	TMP	AMP+CPR	AMP+FUS	AMP+DOX	AMP+TMP	CPR+FUS	CPR+DOX	CPR+TMP	FUS+DOX	FUS+TMP	DOX+TMP	AMP+CPR+FUS	AMP+CPR+DOX	AMP+CPR+TMP	AMP+FUS+DOX	AMP+FUS+TMP	AMP+DOX+TMP	CPR+FUS+DOX	CPR+FUS+TMP	CPR+DOX+TMP	FUS+DOX+TMP	AMP+CPR+FUS+DOX	AMP+CPR+FUS+TMP	AMP+CPR+DOX+TMP	AMP+FUS+DOX+TMP	CPR+FUS+DOX+TMP	AMP+CPR+FUS+DOX+TMP
	72.70226278	86.38437378	72.56876735	92.53710799	94.59267246	71.23501227	21.09172608	71.23501227	72.40881267	87.66821798	80.91886563	73.58261308	82.2592856	94.59267246	103.6958866	13.71879227	65.65946033	62.72495931	29.01487884	41.04633302	72.11536257	84.73371696	77.69091451	83.55991655	70.80685494	39.87253261	46.45681928	73.58261308	35.0122653	85.32061716	32.95811458
	69.27274016	98.12693389	80.73290518	89.19531545	92.51983377	71.21902827	41.81685051	69.87741219	66.85405201	86.20355717	70.48208423	78.94749274	86.77748394	102.7956177	107.0268228	28.21172969	57.78397147	68.06339608	30.74379384	51.00030706	76.52880459	94.66896568	78.04048468	81.685169	82.24404987	35.46779413	44.23553865	83.18019699	32.14209793	74.11011645	31.95313792
	75.20205731	85.26818516	93.47278714	100.9103235	99.36083672	67.63409258	30.52902278	64.93387215	60.23144747	76.10213079	72.86921381	72.63041881	100.6004261	102.1499128	89.13422429	11.42542248	66.16458486	65.81557678	44.63629684	51.10213079	79.02277737	95.20573108	71.69360764	84.09257899	84.79566144	39.05216752	43.46069067	70.81190301	31.11682586	73.14474651	28.17781043
AMP2CPR2FUS1DOX2TMP3	AMP	CPR	FUS	DOX	TMP	AMP+CPR	AMP+FUS	AMP+DOX	AMP+TMP	CPR+FUS	CPR+DOX	CPR+TMP	FUS+DOX	FUS+TMP	DOX+TMP	AMP+CPR+FUS	AMP+CPR+DOX	AMP+CPR+TMP	AMP+FUS+DOX	AMP+FUS+TMP	AMP+DOX+TMP	CPR+FUS+DOX	CPR+FUS+TMP	CPR+DOX+TMP	FUS+DOX+TMP	AMP+CPR+FUS+DOX	AMP+CPR+FUS+TMP	AMP+CPR+DOX+TMP	AMP+FUS+DOX+TMP	CPR+FUS+DOX+TMP	AMP+CPR+FUS+DOX+TMP
	72.70226278	86.38437378	72.56876735	92.53710799	98.11649728	71.23501227	21.09172608	71.23501227	78.57126482	87.66821798	80.91886563	76.5171141	82.2592856	73.74337562	103.9895386	13.71879227	65.65946033	63.01840941	29.01487884	28.84981315	61.844609	84.73371696	72.70226278	96.17827094	79.32276492	39.87253261	23.56771132	82.67956624	26.50221234	103.2210734	32.07776428
	69.27274016	98.12693389	80.73290518	89.19531545	99.16887042	71.21902827	41.81685051	69.87741219	67.45872405	86.20355717	70.48208423	76.52880459	86.77748394	98.56441254	98.56441254	28.21172969	57.78397147	67.15638803	30.74379384	41.02321846	80.45917283	94.66896568	92.25027754	96.48298179	99.47109936	35.46779413	24.88603349	84.08720505	29.11873775	88.01757328	27.55036965
	75.20205731	85.26818516	93.47278714	100.9103235	102.4598102	67.63409258	30.52902278	64.93387215	66.40337987	76.10213079	72.86921381	69.10360029	100.6004261	94.09258183	91.92330041	11.42542248	66.16458486	58.7435709	44.63629684	20.24246877	62.56429096	95.20573108	84.62527553	72.86921381	94.71237653	39.05216752	17.89125643	57.88023512	37.58265981	82.86186627	24.35709037
AMP2CPR2FUS1DOX3TMP1	AMP	CPR	FUS	DOX	TMP	AMP+CPR	AMP+FUS	AMP+DOX	AMP+TMP	CPR+FUS	CPR+DOX	CPR+TMP	FUS+DOX	FUS+TMP	DOX+TMP	AMP+CPR+FUS	AMP+CPR+DOX	AMP+CPR+TMP	AMP+FUS+DOX	AMP+FUS+TMP	AMP+DOX+TMP	CPR+FUS+DOX	CPR+FUS+TMP	CPR+DOX+TMP	FUS+DOX+TMP	AMP+CPR+FUS+DOX	AMP+CPR+FUS+TMP	AMP+CPR+DOX+TMP	AMP+FUS+DOX+TMP	CPR+FUS+DOX+TMP	AMP+CPR+FUS+DOX+TMP
	72.70226278	86.38437378	72.56876735	103.4022345	99.29110555	71.23501227	21.09172608	71.82191247	82.67956624	87.66821798	79.04812123	81.50576584	87.8386749	84.90215421	106.6324073	13.71879227	75.04986359	61.5511589	27.54762833	38.4052821	73.28916298	75.6367638	85.32061716	98.81932186	81.96563353	35.76423118	11.37119145	72.70226278	27.54762833	98.23242165	49.09787019
	69.27274016	98.12693389	80.73290518	94.93766528	98.2621836	71.21902827	41.81685051	64.73769988	73.80778043	86.20355717	75.62179654	75.31946052	94.93766528	76.80392898	100.6800151	28.21172969	62.31901174	53.85360323	31.95313792	38.3021943	82.87786097	71.15915629	86.50589319	89.22691735	94.93766528	23.48772941	46.76760281	77.73814866	40.90984245	85.59888514	43.74424262
	75.20205731	85.26818516	93.47278714	112.6864226	93.47278714	67.63409258	30.52902278	58.81704629	60.21307862	76.10213079	75.5143277	76.39603233	106.1785783	101.2202208	108.6577571	11.42542248	55.29022777	45.53637032	44.04849375	46.10580456	62.56429096	73.75091844	66.40337987	85.50698016	80.76699593	45.81190301	47.86921381	64.69507715	28.47171198	81.98016165	67.26671565
AMP2CPR2FUS1DOX3TMP2	AMP	CPR	FUS	DOX	TMP	AMP+CPR	AMP+FUS	AMP+DOX	AMP+TMP	CPR+FUS	CPR+DOX	CPR+TMP	FUS+DOX	FUS+TMP	DOX+TMP	AMP+CPR+FUS	AMP+CPR+DOX	AMP+CPR+TMP	AMP+FUS+DOX	AMP+FUS+TMP	AMP+DOX+TMP	CPR+FUS+DOX	CPR+FUS+TMP	CPR+DOX+TMP	FUS+DOX+TMP	AMP+CPR+FUS+DOX	AMP+CPR+FUS+TMP	AMP+CPR+DOX+TMP	AMP+FUS+DOX+TMP	CPR+FUS+DOX+TMP	AMP+CPR+FUS+DOX+TMP
	72.70226278	86.38437378	72.56876735	103.4022345	94.59267246	71.23501227	21.09172608	71.82191247	72.40881267	87.66821798	79.04812123	73.58261308	87.8386749	94.59267246	102.5212783	13.71879227	75.04986359	62.72495931	27.54762833	41.04633302	69.76776176	75.6367638	77.69091451	86.78786767	98.11649728	35.76423118	46.45681928	67.12671084	35.76423118	95.59137073	43.06380247
	69.27274016	98.12693389	80.73290518	94.93766528	92.51983377	71.21902827	41.81685051	64.73769988	66.85405201	86.20355717	75.62179654	78.94749274	94.93766528	102.7956177	92.21760484	28.21172969	62.31901174	68.06339608	31.95313792	51.00030706	74.41245246	71.15915629	78.04048468	78.64515672	89.79977333	23.48772941	44.23553865	74.11011645	36.97947422	83.48253301	46.95656282
	75.20205731	85.26818516	93.47278714	112.6864226	99.36083672	67.63409258	30.52902278	58.81704629	60.23144747	76.10213079	75.5143277	72.63041881	106.1785783	102.1499128	101.5301181	11.42542248	55.29022777	65.81557678	44.04849375	51.10213079	77.27773696	73.75091844	71.69360764	89.97060985	95.64206856	45.81190301	43.46069067	67.2850845	24.65099192	94.32402645	46.69360764
AMP2CPR2FUS1DOX3TMP3	AMP	CPR	FUS	DOX	TMP	AMP+CPR	AMP+FUS	AMP+DOX	AMP+TMP	CPR+FUS	CPR+DOX	CPR+TMP	FUS+DOX	FUS+TMP	DOX+TMP	AMP+CPR+FUS	AMP+CPR+DOX	AMP+CPR+TMP	AMP+FUS+DOX	AMP+FUS+TMP	AMP+DOX+TMP	CPR+FUS+DOX	CPR+FUS+TMP	CPR+DOX+TMP	FUS+DOX+TMP	AMP+CPR+FUS+DOX	AMP+CPR+FUS+TMP	AMP+CPR+DOX+TMP	AMP+FUS+DOX+TMP	CPR+FUS+DOX+TMP	AMP+CPR+FUS+DOX+TMP
	72.70226278	86.38437378	72.56876735	103.4022345	98.11649728	71.23501227	21.09172608	71.82191247	78.57126482	87.66821798	79.04812123	76.5171141	87.8386749	73.74337562	91.65615178	13.71879227	75.04986359	63.01840941	27.54762833	28.84981315	76.5171141	75.6367638	72.70226278	86.09032368	76.97354837	35.76423118	23.56771132	66.72321695	27.21749696	100.5800225	39.24895115
	69.27274016	98.12693389	80.73290518	94.93766528	99.16887042	71.21902827	41.81685051	64.73769988	67.45872405	86.20355717	75.62179654	76.52880459	94.93766528	98.56441254	94.03097847	28.21172969	62.31901174	67.15638803	31.95313792	41.02321846	70.48208423	71.15915629	92.25027754	100.8479581	82.84850774	23.48772941	24.88603349	94.49890167	21.86267331	74.11011645	51.79393911
	75.20205731	85.26818516	93.47278714	112.6864226	102.4598102	67.63409258	30.52902278	58.81704629	66.40337987	76.10213079	75.5143277	69.10360029	106.1785783	94.09258183	101.5301181	11.42542248	55.29022777	58.7435709	44.04849375	20.24246877	72.92432035	73.75091844	84.62527553	96.43644379	80.45709859	45.81190301	17.89125643	76.39603233	20.31594416	86.09478325	47.28141073
AMP2CPR2FUS2DOX1TMP1	AMP	CPR	FUS	DOX	TMP	AMP+CPR	AMP+FUS	AMP+DOX	AMP+TMP	CPR+FUS	CPR+DOX	CPR+TMP	FUS+DOX	FUS+TMP	DOX+TMP	AMP+CPR+FUS	AMP+CPR+DOX	AMP+CPR+TMP	AMP+FUS+DOX	AMP+FUS+TMP	AMP+DOX+TMP	CPR+FUS+DOX	CPR+FUS+TMP	CPR+DOX+TMP	FUS+DOX+TMP	AMP+CPR+FUS+DOX	AMP+CPR+FUS+TMP	AMP+CPR+DOX+TMP	AMP+FUS+DOX+TMP	CPR+FUS+DOX+TMP	AMP+CPR+FUS+DOX+TMP
	72.70226278	86.38437378	97.64552145	88.42597903	99.29110555	71.23501227	41.76161764	74.46296339	82.67956624	96.47172104	81.50576584	81.50576584	90.8961691	99.11277196	96.94188901	46.32843486	71.82191247	61.5511589	27.96946285	45.15463445	74.75641349	96.17827094	88.84201839	78.27781471	97.64552145	34.59043078	54.37997203	69.76776176	50.85857081	92.95031981	35.5991655
	69.27274016	98.12693389	108.8787585	90.40423121	98.2621836	71.21902827	70.53877223	63.3582918	73.80778043	98.59933392	89.52925337	75.31946052	79.24982875	90.43626143	92.82206271	45.14254671	76.05640456	53.85360323	40.24848241	46.76760281	64.73769988	88.3199093	97.99466188	80.59144884	91.34326948	41.15549047	54.32600326	82.57552495	61.58206769	108.5764225	43.2718426
	75.20205731	85.26818516	96.6752388	95.95196591	93.47278714	67.63409258	59.62527553	71.45481264	60.21307862	80.21675239	89.08890522	76.39603233	56.39235856	90.79720794	108.9676545	31.99853049	83.21087436	45.53637032	28.17781043	35.81925055	59.64364438	96.08743571	81.09845702	73.51212344	101.0837619	53.45334313	68.44232182	73.4570169	64.93387215	73.4570169	65.79720794
AMP2CPR2FUS2DOX1TMP2	AMP	CPR	FUS	DOX	TMP	AMP+CPR	AMP+FUS	AMP+DOX	AMP+TMP	CPR+FUS	CPR+DOX	CPR+TMP	FUS+DOX	FUS+TMP	DOX+TMP	AMP+CPR+FUS	AMP+CPR+DOX	AMP+CPR+TMP	AMP+FUS+DOX	AMP+FUS+TMP	AMP+DOX+TMP	CPR+FUS+DOX	CPR+FUS+TMP	CPR+DOX+TMP	FUS+DOX+TMP	AMP+CPR+FUS+DOX	AMP+CPR+FUS+TMP	AMP+CPR+DOX+TMP	AMP+FUS+DOX+TMP	CPR+FUS+DOX+TMP	AMP+CPR+FUS+DOX+TMP
	72.70226278	86.38437378	97.64552145	88.42597903	94.59267246	71.23501227	41.76161764	74.46296339	72.40881267	96.47172104	81.50576584	73.58261308	90.8961691	92.65686971	99.58475762	46.32843486	71.82191247	62.72495931	27.96946285	47.63061968	80.03851533	96.17827094	92.06996951	80.33196543	101.7538229	34.59043078	55.26032234	71.82191247	51.73892111	88.54856828	40.75288292
	69.27274016	98.12693389	108.8787585	90.40423121	92.51983377	71.21902827	70.53877223	63.3582918	66.85405201	98.59933392	89.52925337	78.94749274	79.24982875	89.52925337	96.14658104	45.14254671	76.05640456	68.06339608	40.24848241	57.95403548	65.34237192	88.3199093	107.3670784	68.49800411	88.3199093	41.15549047	61.58206769	88.62224532	55.23301131	96.78531781	40.0028344
	75.20205731	85.26818516	96.6752388	95.95196591	99.36083672	67.63409258	59.62527553	71.45481264	60.23144747	80.21675239	89.08890522	72.63041881	56.39235856	83.44966936	100.6004261	31.99853049	83.21087436	65.81557678	28.17781043	46.71197649	61.09478325	96.08743571	87.85819251	69.39750184	101.0837619	53.45334313	86.66421749	73.4570169	70.49963262	106.6678913	47.86921381
AMP2CPR2FUS2DOX1TMP3	AMP	CPR	FUS	DOX	TMP	AMP+CPR	AMP+FUS	AMP+DOX	AMP+TMP	CPR+FUS	CPR+DOX	CPR+TMP	FUS+DOX	FUS+TMP	DOX+TMP	AMP+CPR+FUS	AMP+CPR+DOX	AMP+CPR+TMP	AMP+FUS+DOX	AMP+FUS+TMP	AMP+DOX+TMP	CPR+FUS+DOX	CPR+FUS+TMP	CPR+DOX+TMP	FUS+DOX+TMP	AMP+CPR+FUS+DOX	AMP+CPR+FUS+TMP	AMP+CPR+DOX+TMP	AMP+FUS+DOX+TMP	CPR+FUS+DOX+TMP	AMP+CPR+FUS+DOX+TMP
	72.70226278	86.38437378	97.64552145	88.42597903	98.11649728	71.23501227	41.76161764	74.46296339	78.57126482	96.47172104	81.50576584	76.5171141	90.8961691	101.1669227	99.29110555	46.32843486	71.82191247	63.01840941	27.96946285	58.78172356	75.9302139	96.17827094	92.65686971	69.76776176	94.41757032	34.59043078	61.42277448	71.23501227	57.90137325	95.59137073	35.0122653
	69.27274016	98.12693389	108.8787585	90.40423121	99.16887042	71.21902827	70.53877223	63.3582918	67.45872405	98.59933392	89.52925337	76.52880459	79.24982875	94.06429364	99.16887042	45.14254671	76.05640456	67.15638803	40.24848241	59.76805159	65.94704396	88.3199093	109.1810946	76.96341262	73.50544441	41.15549047	54.32600326	84.9942131	70.95448426	99.20400595	37.69752226
	75.20205731	85.26818516	96.6752388	95.95196591	102.4598102	67.63409258	59.62527553	71.45481264	66.40337987	80.21675239	89.08890522	69.10360029	56.39235856	96.96914034	104.938989	31.99853049	83.21087436	58.7435709	28.17781043	45.83027186	62.56429096	96.08743571	87.27038942	77.92064658	87.85819251	53.45334313	69.32402645	66.40337987	66.09110948	108.4313005	56.09845702
AMP2CPR2FUS2DOX2TMP1	AMP	CPR	FUS	DOX	TMP	AMP+CPR	AMP+FUS	AMP+DOX	AMP+TMP	CPR+FUS	CPR+DOX	CPR+TMP	FUS+DOX	FUS+TMP	DOX+TMP	AMP+CPR+FUS	AMP+CPR+DOX	AMP+CPR+TMP	AMP+FUS+DOX	AMP+FUS+TMP	AMP+DOX+TMP	CPR+FUS+DOX	CPR+FUS+TMP	CPR+DOX+TMP	FUS+DOX+TMP	AMP+CPR+FUS+DOX	AMP+CPR+FUS+TMP	AMP+CPR+DOX+TMP	AMP+FUS+DOX+TMP	CPR+FUS+DOX+TMP	AMP+CPR+FUS+DOX+TMP
	72.70226278	86.38437378	97.64552145	92.53710799	99.29110555	71.23501227	41.76161764	71.23501227	82.67956624	96.47172104	80.91886563	81.50576584	107.6228249	99.11277196	100.7593659	46.32843486	65.65946033	61.5511589	28.13452853	45.15463445	76.5171141	99.11277196	88.84201839	74.16951329	96.76517114	47.79568537	54.37997203	70.35466196	50.5651207	94.71102043	57.31447305
	69.27274016	98.12693389	108.8787585	89.19531545	98.2621836	71.21902827	70.53877223	69.87741219	73.80778043	98.59933392	70.48208423	75.31946052	105.5530623	90.43626143	97.35549679	45.14254671	57.78397147	53.85360323	36.79051421	46.76760281	65.34237192	104.0413822	97.99466188	78.3428207	101.3203581	59.76805159	54.32600326	87.71523726	55.11963531	78.64515672	44.65125068
	75.20205731	85.26818516	96.6752388	100.9103235	93.47278714	67.63409258	59.62527553	64.93387215	60.21307862	80.21675239	72.86921381	76.39603233	90.79720794	90.79720794	104.0092969	31.99853049	66.16458486	45.53637032	31.11682586	35.81925055	70.49963262	96.96914034	81.09845702	64.05216752	96.6752388	45.22409993	68.44232182	71.3997061	69.03012491	94.91182954	39.05216752
AMP2CPR2FUS2DOX2TMP2	AMP	CPR	FUS	DOX	TMP	AMP+CPR	AMP+FUS	AMP+DOX	AMP+TMP	CPR+FUS	CPR+DOX	CPR+TMP	FUS+DOX	FUS+TMP	DOX+TMP	AMP+CPR+FUS	AMP+CPR+DOX	AMP+CPR+TMP	AMP+FUS+DOX	AMP+FUS+TMP	AMP+DOX+TMP	CPR+FUS+DOX	CPR+FUS+TMP	CPR+DOX+TMP	FUS+DOX+TMP	AMP+CPR+FUS+DOX	AMP+CPR+FUS+TMP	AMP+CPR+DOX+TMP	AMP+FUS+DOX+TMP	CPR+FUS+DOX+TMP	AMP+CPR+FUS+DOX+TMP
	72.70226278	86.38437378	97.64552145	92.53710799	94.59267246	71.23501227	41.76161764	71.23501227	72.40881267	96.47172104	80.91886563	73.58261308	107.6228249	92.65686971	103.6958866	46.32843486	65.65946033	62.72495931	28.13452853	47.63061968	72.11536257	99.11277196	92.06996951	83.55991655	99.11277196	47.79568537	55.26032234	73.58261308	53.20617162	102.3407231	46.45681928
	69.27274016	98.12693389	108.8787585	89.19531545	92.51983377	71.21902827	70.53877223	69.87741219	66.85405201	98.59933392	70.48208423	78.94749274	105.5530623	89.52925337	107.0268228	45.14254671	57.78397147	68.06339608	36.79051421	57.95403548	76.52880459	104.0413822	107.3670784	81.685169	113.4137988	59.76805159	61.58206769	83.18019699	61.58206769	96.48298179	35.27883412
	75.20205731	85.26818516	96.6752388	100.9103235	99.36083672	67.63409258	59.62527553	64.93387215	60.23144747	80.21675239	72.86921381	72.63041881	90.79720794	83.44966936	89.13422429	31.99853049	66.16458486	65.81557678	31.11682586	46.71197649	79.02277737	96.96914034	87.85819251	84.09257899	102.2593681	45.22409993	86.66421749	70.81190301	64.32770022	70.22409993	49.33872153
AMP2CPR2FUS2DOX2TMP3	AMP	CPR	FUS	DOX	TMP	AMP+CPR	AMP+FUS	AMP+DOX	AMP+TMP	CPR+FUS	CPR+DOX	CPR+TMP	FUS+DOX	FUS+TMP	DOX+TMP	AMP+CPR+FUS	AMP+CPR+DOX	AMP+CPR+TMP	AMP+FUS+DOX	AMP+FUS+TMP	AMP+DOX+TMP	CPR+FUS+DOX	CPR+FUS+TMP	CPR+DOX+TMP	FUS+DOX+TMP	AMP+CPR+FUS+DOX	AMP+CPR+FUS+TMP	AMP+CPR+DOX+TMP	AMP+FUS+DOX+TMP	CPR+FUS+DOX+TMP	AMP+CPR+FUS+DOX+TMP
	72.70226278	86.38437378	97.64552145	92.53710799	98.11649728	71.23501227	41.76161764	71.23501227	78.57126482	96.47172104	80.91886563	76.5171141	107.6228249	101.1669227	103.9895386	46.32843486	65.65946033	63.01840941	28.13452853	58.78172356	61.844609	99.11277196	92.65686971	96.17827094	110.850776	47.79568537	61.42277448	82.67956624	48.96948577	107.916275	52.91272152
	69.27274016	98.12693389	108.8787585	89.19531545	99.16887042	71.21902827	70.53877223	69.87741219	67.45872405	98.59933392	70.48208423	76.52880459	105.5530623	94.06429364	98.56441254	45.14254671	57.78397147	67.15638803	36.79051421	59.76805159	80.45917283	104.0413822	109.1810946	96.48298179	109.1810946	59.76805159	54.32600326	84.08720505	52.09627513	85.29654912	63.52835581
	75.20205731	85.26818516	96.6752388	100.9103235	102.4598102	67.63409258	59.62527553	64.93387215	66.40337987	80.21675239	72.86921381	69.10360029	90.79720794	96.96914034	91.92330041	31.99853049	66.16458486	58.7435709	31.11682586	45.83027186	62.56429096	96.96914034	87.27038942	72.86921381	105.1983835	45.22409993	69.32402645	57.88023512	43.16678913	86.68258633	39.93387215
AMP2CPR2FUS2DOX3TMP1	AMP	CPR	FUS	DOX	TMP	AMP+CPR	AMP+FUS	AMP+DOX	AMP+TMP	CPR+FUS	CPR+DOX	CPR+TMP	FUS+DOX	FUS+TMP	DOX+TMP	AMP+CPR+FUS	AMP+CPR+DOX	AMP+CPR+TMP	AMP+FUS+DOX	AMP+FUS+TMP	AMP+DOX+TMP	CPR+FUS+DOX	CPR+FUS+TMP	CPR+DOX+TMP	FUS+DOX+TMP	AMP+CPR+FUS+DOX	AMP+CPR+FUS+TMP	AMP+CPR+DOX+TMP	AMP+FUS+DOX+TMP	CPR+FUS+DOX+TMP	AMP+CPR+FUS+DOX+TMP
	72.70226278	86.38437378	97.64552145	103.4022345	99.29110555	71.23501227	41.76161764	71.82191247	82.67956624	96.47172104	79.04812123	81.50576584	98.52587175	99.11277196	106.6324073	46.32843486	75.04986359	61.5511589	57.02102295	45.15463445	73.28916298	90.3092689	88.84201839	98.81932186	117.3066783	38.99218231	54.37997203	72.70226278	57.73630757	114.3721773	55.68215686
	69.27274016	98.12693389	108.8787585	94.93766528	98.2621836	71.21902827	70.53877223	64.73769988	73.80778043	98.59933392	75.62179654	75.31946052	100.715686	90.43626143	100.6800151	45.14254671	62.31901174	53.85360323	54.02366724	46.76760281	82.87786097	110.0881026	97.99466188	89.22691735	103.7390462	51.90731511	54.32600326	77.73814866	51.30264308	104.0413822	52.34192314

	75.20205731	85.26818516	96.6752388	112.6864226	93.47278714	67.63409258	59.62527553	58.81704629	60.21307862	80.21675239	75.5143277
AMP2CPR2FUS2DOX3TMP2	AMP	CPR	FUS	DOX	TMP	AMP+CPR	AMP+FUS	AMP+DOX	AMP+TMP	CPR+FUS	CPR+DOX
	72.70226278	86.38437378	97.64552145	103.4022345	94.59267246	71.23501227	41.76161764	71.82191247	72.40881267	96.47172104	79.04812123
	69.27274016	98.12693389	108.8787585	94.93766528	92.51983377	71.21902827	70.53877223	64.73769988	66.85405201	98.59933392	75.62179654
	75.20205731	85.26818516	96.6752388	112.6864226	99.36083672	67.63409258	59.62527553	58.81704629	60.23144747	80.21675239	75.5143277
AMP2CPR2FUS2DOX3TMP3	AMP	CPR	FUS	DOX	TMP	AMP+CPR	AMP+FUS	AMP+DOX	AMP+TMP	CPR+FUS	CPR+DOX
	72.70226278	86.38437378	97.64552145	103.4022345	98.11649728	71.23501227	41.76161764	71.82191247	78.57126482	96.47172104	79.04812123
	69.27274016	98.12693389	108.8787585	94.93766528	99.16887042	71.21902827	70.53877223	64.73769988	67.45872405	98.59933392	75.62179654
	75.20205731	85.26818516	96.6752388	112.6864226	102.4598102	67.63409258	59.62527553	58.81704629	66.40337987	80.21675239	75.5143277
AMP2CPR2FUS3DOX1TMP1	AMP	CPR	FUS	DOX	TMP	AMP+CPR	AMP+FUS	AMP+DOX	AMP+TMP	CPR+FUS	CPR+DOX
	72.70226278	86.38437378	95.90014577	88.42597903	99.29110555	71.23501227	60.54242417	74.46296339	82.67956624	92.06996951	81.50576584
	69.27274016	98.12693389	92.29679476	90.40423121	98.2621836	71.21902827	69.14046815	63.3582918	73.80778043	104.9483903	89.52925337
	75.20205731	85.26818516	84.78536497	95.95196591	93.47278714	67.63409258	64.91550331	71.45481264	60.21307862	79.04114622	89.08890522
AMP2CPR2FUS3DOX1TMP2	AMP	CPR	FUS	DOX	TMP	AMP+CPR	AMP+FUS	AMP+DOX	AMP+TMP	CPR+FUS	CPR+DOX
	72.70226278	86.38437378	95.90014577	88.42597903	94.59267246	71.23501227	60.54242417	74.46296339	72.40881267	92.06996951	81.50576584
	69.27274016	98.12693389	92.29679476	90.40423121	92.51983377	71.21902827	69.14046815	63.3582918	66.85405201	104.9483903	89.52925337
	75.20205731	85.26818516	84.78536497	95.95196591	99.36083672	67.63409258	64.91550331	71.45481264	60.23144747	79.04114622	89.08890522
AMP2CPR2FUS3DOX1TMP3	AMP	CPR	FUS	DOX	TMP	AMP+CPR	AMP+FUS	AMP+DOX	AMP+TMP	CPR+FUS	CPR+DOX
	72.70226278	86.38437378	95.90014577	88.42597903	98.11649728	71.23501227	60.54242417	74.46296339	78.57126482	92.06996951	81.50576584
	69.27274016	98.12693389	92.29679476	90.40423121	99.16887042	71.21902827	69.14046815	63.3582918	67.45872405	104.9483903	89.52925337
	75.20205731	85.26818516	84.78536497	95.95196591	102.4598102	67.63409258	64.91550331	71.45481264	66.40337987	79.04114622	89.08890522

AMP3CPR1FUS2DOX1TMP1	AMP	CPR	FUS	DOX	TMP	AMP+CPR	AMP+FUS	AMP+DOX	AMP+TMP	CPR+FUS	CPR+DOX	CPR+TMP	FUS+DOX	FUS+TMP	DOX+TMP	AMP+CPR+FUS	AMP+CPR+DOX	AMP+CPR+TMP	AMP+FUS+DOX	AMP+FUS+TMP	AMP+DOX+TMP	CPR+FUS+DOX	CPR+FUS+TMP	CPR+DOX+TMP	FUS+DOX+TMP	AMP+CPR+FUS+DOX	AMP+CPR+FUS+TMP	AMP+CPR+DOX+TMP	AMP+FUS+DOX+TMP	CPR+FUS+DOX+TMP	AMP+CPR+FUS+DOX+TMP
	82.08819242	87.25137076	97.64552145	88.42597903	99.29110555	76.84037901	53.46209913	85.58673469	64.30393586	91.36249971	87.8386749	91.06884765	90.8961691	99.11277196	96.94188901	56.94241983	87.91909621	68.20335277	65.28790087	40.32434402	55.26603499	99.29110555	92.83076005	95.47362867	97.64552145	61.20626822	67.3287172	73.15962099	60.4409621	100.1720618	54.20918367
	85.40818134	87.38194182	108.8787585	90.40423121	98.2621836	78.32477422	67.15069949	73.59659996	48.66300691	87.38194182	90.10200227	85.56856819	79.24982875	90.43626143	92.82206271	55.3214096	72.46325483	57.16309545	46.11298034	56.59642288	53.3380556	95.54212316	102.1911598	97.95995467	91.34326948	48.94634319	77.27997167	68.49654684	63.96316628	121.8360408	59.71312201
	77.10559681	92.5430951	96.6752388	95.95196591	93.47278714	69.57072994	73.01213548	77.68520196	82.46694439	107.1082704	96.5717606	99.36083672	56.39235856	90.79720794	108.9676545	59.86234378	73.62796595	73.62796595	59.10161203	54.93570005	86.08947654	114.5458067	106.1785783	84.79566144	101.0837619	58.99293606	65.65839522	79.71381996	73.19326209	111.7567306	58.70313349
AMP3CPR1FUS2DOX1TMP2	AMP	CPR	FUS	DOX	TMP	AMP+CPR	AMP+FUS	AMP+DOX	AMP+TMP	CPR+FUS	CPR+DOX	CPR+TMP	FUS+DOX	FUS+TMP	DOX+TMP	AMP+CPR+FUS	AMP+CPR+DOX	AMP+CPR+TMP	AMP+FUS+DOX	AMP+FUS+TMP	AMP+DOX+TMP	CPR+FUS+DOX	CPR+FUS+TMP	CPR+DOX+TMP	FUS+DOX+TMP	AMP+CPR+FUS+DOX	AMP+CPR+FUS+TMP	AMP+CPR+DOX+TMP	AMP+FUS+DOX+TMP	CPR+FUS+DOX+TMP	AMP+CPR+FUS+DOX+TMP
	82.08819242	87.25137076	97.64552145	88.42597903	94.59267246	76.84037901	53.46209913	85.58673469	71.00947522	91.36249971	87.8386749	95.1799766	90.8961691	92.65686971	99.58475762	56.94241983	87.91909621	74.90889213	65.28790087	46.62900875	55.66690962	99.29110555	97.52919315	98.70380142	101.7538229	61.20626822	55.08381924	77.24125364	76.18440233	89.30693524	69.07798834
	85.40818134	87.38194182	108.8787585	90.40423121	92.51983377	78.32477422	67.15069949	73.59659996	52.82450859	87.38194182	90.10200227	90.70646014	79.24982875	89.52925337	96.14658104	55.3214096	72.46325483	55.46307774	46.11298034	56.10058438	57.44643173	95.54212316	112.7691726	106.120136	88.3199093	48.94634319	59.9964583	51.69116345	55.17974146	107.3290518	58.0131043
	77.10559681	92.5430951	96.6752388	95.95196591	99.36083672	69.57072994	73.01213548	77.68520196	83.33635211	107.1082704	96.5717606	90.68371102	56.39235856	83.44966936	100.6004261	59.86234378	73.62796595	78.2648071	59.10161203	67.39721065	80.00362253	114.5458067	105.2488863	91.61340306	101.0837619	58.99293606	53.30556059	74.78717624	60.84042746	100.2905288	57.83372577
AMP3CPR1FUS2DOX1TMP3	AMP	CPR	FUS	DOX	TMP	AMP+CPR	AMP+FUS	AMP+DOX	AMP+TMP	CPR+FUS	CPR+DOX	CPR+TMP	FUS+DOX	FUS+TMP	DOX+TMP	AMP+CPR+FUS	AMP+CPR+DOX	AMP+CPR+TMP	AMP+FUS+DOX	AMP+FUS+TMP	AMP+DOX+TMP	CPR+FUS+DOX	CPR+FUS+TMP	CPR+DOX+TMP	FUS+DOX+TMP	AMP+CPR+FUS+DOX	AMP+CPR+FUS+TMP	AMP+CPR+DOX+TMP	AMP+FUS+DOX+TMP	CPR+FUS+DOX+TMP	AMP+CPR+FUS+DOX+TMP
	82.08819242	87.25137076	97.64552145	88.42597903	98.11649728	76.84037901	53.46209913	85.58673469	65.17857143	91.36249971	87.8386749	89.89423937	90.8961691	101.1669227	99.29110555	56.94241983	87.91909621	78.11588921	65.28790087	59.85787172	64.88702624	99.29110555	114.267361	99.29110555	94.41757032	61.20626822	57.41618076	71.70189504	62.48177843	101.34667	78.11588921
	85.40818134	87.38194182	108.8787585	90.40423121	99.16887042	78.32477422	67.15069949	73.59659996	53.62139189	87.38194182	90.10200227	93.72874953	79.24982875	94.06429364	99.16887042	55.3214096	72.46325483	67.64653798	46.11298034	51.6380379	56.31308659	95.54212316	119.1159804	104.9112202	73.50544441	48.94634319	49.51301576	63.87462369	60.84646715	111.8624858	70.76323712
	77.10559681	92.5430951	96.6752388	95.95196591	102.4598102	69.57072994	73.01213548	77.68520196	92.17533056	107.1082704	96.5717606	85.41545613	56.39235856	96.96914034	104.938989	59.86234378	73.62796595	88.11809455	59.10161203	81.30773411	90.43651512	114.5458067	102.4598102	86.03525082	87.85819251	58.99293606	44.64770875	86.95888426	68.84622351	106.1785783	85.36497011
AMP3CPR1FUS2DOX2TMP1	AMP	CPR	FUS	DOX	TMP	AMP+CPR	AMP+FUS	AMP+DOX	AMP+TMP	CPR+FUS	CPR+DOX	CPR+TMP	FUS+DOX	FUS+TMP	DOX+TMP	AMP+CPR+FUS	AMP+CPR+DOX	AMP+CPR+TMP	AMP+FUS+DOX	AMP+FUS+TMP	AMP+DOX+TMP	CPR+FUS+DOX	CPR+FUS+TMP	CPR+DOX+TMP	FUS+DOX+TMP	AMP+CPR+FUS+DOX	AMP+CPR+FUS+TMP	AMP+CPR+DOX+TMP	AMP+FUS+DOX+TMP	CPR+FUS+DOX+TMP	AMP+CPR+FUS+DOX+TMP
	82.08819242	87.25137076	97.64552145	92.53710799	99.29110555	76.84037901	53.46209913	80.92201166	64.30393586	91.36249971	94.59267246	91.06884765	107.6228249	99.11277196	100.7593659	56.94241983	88.2106414	68.20335277	50.41909621	40.32434402	59.34766764	91.36249971	92.83076005	101.053018	96.76517114	59.27478134	67.3287172	69.84329446	53.44387755	86.37041455	72.28498542
	85.40818134	87.38194182	108.8787585	89.19531545	98.2621836	78.32477422	67.15069949	63.39649371	48.66300691	87.38194182	87.38194182	85.56856819	105.5530623	90.43626143	97.35549679	55.3214096	79.26332566	57.16309545	39.59624579	56.59642288	72.46325483	90.10200227	102.1911598	107.9335096	101.3203581	48.94634319	77.27997167	63.30795112	56.38392067	80.73290518	69.91322826
	77.10559681	92.5430951	96.6752388	100.9103235	93.47278714	69.57072994	73.01213548	78.55460967	82.46694439	107.1082704	99.36083672	99.36083672	90.79720794	90.79720794	104.0092969	59.86234378	77.10559681	73.62796595	49.82792972	54.93570005	90.14671255	113.6161147	106.1785783	92.5430951	96.6752388	49.24832458	65.65839522	76.2361891	54.1749683	89.75401898	64.20938236
AMP3CPR1FUS2DOX2TMP2	AMP	CPR	FUS	DOX	TMP	AMP+CPR	AMP+FUS	AMP+DOX	AMP+TMP	CPR+FUS	CPR+DOX	CPR+TMP	FUS+DOX	FUS+TMP	DOX+TMP	AMP+CPR+FUS	AMP+CPR+DOX	AMP+CPR+TMP	AMP+FUS+DOX	AMP+FUS+TMP	AMP+DOX+TMP	CPR+FUS+DOX	CPR+FUS+TMP	CPR+DOX+TMP	FUS+DOX+TMP	AMP+CPR+FUS+DOX	AMP+CPR+FUS+TMP	AMP+CPR+DOX+TMP	AMP+FUS+DOX+TMP	CPR+FUS+DOX+TMP	AMP+CPR+FUS+DOX+TMP
	82.08819242	87.25137076	97.64552145	92.53710799	94.59267246	76.84037901	53.46209913	80.92201166	71.00947522	91.36249971	94.59267246	95.1799766	107.6228249	92.65686971	103.6958866	56.94241983	88.2106414	74.90889213	50.41909621	46.62900875	66.63629738	91.36249971	97.52919315	86.07676249	99.11277196	59.27478134	55.08381924	82.78061224	70.82725948	88.42597903	78.69897959
	85.40818134	87.38194182	108.8787585	89.19531545	92.51983377	78.32477422	67.15069949	63.39649371	52.82450859	87.38194182	87.38194182	90.70646014	105.5530623	89.52925337	107.0268228	55.3214096	79.26332566	55.46307774	39.59624579	56.10058438	85.49672392	90.10200227	112.7691726	97.35549679	113.4137988	48.94634319	59.9964583	66.70798654	38.17956437	93.72874953	74.44660882
	77.10559681	92.5430951	96.6752388	100.9103235	99.36083672	69.57072994	73.01213548	78.55460967	83.33635211	107.1082704	99.36083672	90.68371102	90.79720794	83.44966936	89.13422429	59.86234378	77.10559681	78.2648071	49.82792972	67.39721065	99.71019743	113.6161147	105.2488863	86.03525082	102.2593681	49.24832458	53.30556059	91.88552798	69.13602608	97.81134999	59.57254121
AMP3CPR1FUS2DOX2TMP3	AMP	CPR	FUS	DOX	TMP	AMP+CPR	AMP+FUS	AMP+DOX	AMP+TMP	CPR+FUS	CPR+DOX	CPR+TMP	FUS+DOX	FUS+TMP	DOX+TMP	AMP+CPR+FUS	AMP+CPR+DOX	AMP+CPR+TMP	AMP+FUS+DOX	AMP+FUS+TMP	AMP+DOX+TMP	CPR+FUS+DOX	CPR+FUS+TMP	CPR+DOX+TMP	FUS+DOX+TMP	AMP+CPR+FUS+DOX	AMP+CPR+FUS+TMP	AMP+CPR+DOX+TMP	AMP+FUS+DOX+TMP	CPR+FUS+DOX+TMP	AMP+CPR+FUS+DOX+TMP
	82.08819242	87.25137076	97.64552145	92.53710799	98.11649728	76.84037901	53.46209913	80.92201166	65.17857143	91.36249971	94.59267246	89.89423937	107.6228249	101.1669227	103.9895386	56.94241983	88.2106414	78.11588921	50.41909621	59.85787172	70.2441691	91.36249971	114.267361	86.95771869	110.850776	59.27478134	57.41618076	73.15962099	59.1654519	74.62433183	73.74271137
	85.40818134	87.38194182	108.8787585	89.19531545	99.16887042	78.32477422	67.15069949	63.39649371	53.62139189	87.38194182	87.38194182	93.72874953	105.5530623	94.06429364	98.56441254	55.3214096	79.26332566	67.64653798	39.59624579	51.6380379	84.64671507	90.10200227	119.1159804	91.00868908	109.1810946	48.94634319	49.51301576	76.05808394	57.72976802	90.10200227	45.82964406
	77.10559681	92.5430951	96.6752388	100.9103235	102.4598102	69.57072994	73.01213548	78.55460967	92.17533056	107.1082704	99.36083672	85.41545613	90.79720794	96.96914034	91.92330041	59.86234378	77.10559681	88.11809455	49.82792972	81.30773411	85.79967397	113.6161147	102.4598102	86.34514817	105.1983835	49.24832458	44.64770875	89.56710741	53.88516573	96.5717606	58.70313349
AMP3CPR1FUS2DOX3TMP1	AMP	CPR	FUS	DOX	TMP	AMP+CPR	AMP+FUS	AMP+DOX	AMP+TMP	CPR+FUS	CPR+DOX	CPR+TMP	FUS+DOX	FUS+TMP	DOX+TMP	AMP+CPR+FUS	AMP+CPR+DOX	AMP+CPR+TMP	AMP+FUS+DOX	AMP+FUS+TMP	AMP+DOX+TMP	CPR+FUS+DOX	CPR+FUS+TMP	CPR+DOX+TMP	FUS+DOX+TMP	AMP+CPR+FUS+DOX	AMP+CPR+FUS+TMP	AMP+CPR+DOX+TMP	AMP+FUS+DOX+TMP	CPR+FUS+DOX+TMP	AMP+CPR+FUS+DOX+TMP
	82.08819242	87.25137076	97.64552145	103.4022345	99.29110555	76.84037901	53.46209913	86.46137026	64.30393586	91.36249971	98.41014935	91.06884765	98.52587175	99.11277196	106.6324073	56.94241983	74.03425656	68.20335277	49.38046647	40.32434402	72.75874636	95.76728074	92.83076005	88.13232696	117.3066783	72.57653061	67.3287172	78.29810496	71.99344023	58.47346808	78.69897959
	85.40818134	87.38194182	108.8787585	94.93766528	98.2621836	78.32477422	67.15069949	63.02461484	48.66300691	87.38194182	84.0574235	85.56856819	100.715686	90.43626143	100.6800151	55.3214096	75.29661767	57.16309545	48.94634319	56.59642288	92.86346733	103.4000756	102.1911598	87.68417076	103.7390462	58.57977687	77.27997167	92.20825217	69.34655569	78.61730261	61.41313972
	77.10559681	92.5430951	96.6752388	112.6864226	93.47278714	69.57072994	73.01213548	83.33635211	82.46694439	107.1082704	95.95196591	99.36083672	95.49963262	90.79720794	108.6577571	59.86234378	82.61184568	73.62796595	62.8509328	54.93570005	88.98750226	107.4181677	106.1785783	95.95196591	92.26671565	65.65839522	65.65839522	89.27730484	72.0340518	103.3895022	74.64227495
AMP3CPR1FUS2DOX3TMP2	AMP	CPR	FUS	DOX	TMP	AMP+CPR	AMP+FUS	AMP+DOX	AMP+TMP	CPR+FUS	CPR+DOX	CPR+TMP	FUS+DOX	FUS+TMP	DOX+TMP	AMP+CPR+FUS	AMP+CPR+DOX	AMP+CPR+TMP	AMP+FUS+DOX	AMP+FUS+TMP	AMP+DOX+TMP	CPR+FUS+DOX	CPR+FUS+TMP	CPR+DOX+TMP	FUS+DOX+TMP	AMP+CPR+FUS+DOX	AMP+CPR+FUS+TMP	AMP+CPR+DOX+TMP	AMP+FUS+DOX+TMP	CPR+FUS+DOX+TMP	AMP+CPR+FUS+DOX+TMP
	82.08819242	87.25137076	97.64552145	103.4022345	94.59267246	76.84037901	53.46209913	86.46137026	71.00947522	91.36249971	98.41014935	95.1799766	98.52587175	92.65686971	102.5212783	56.94241983	74.03425656	74.90889213	49.38046647	46.62900875	86.8622449	95.76728074	97.52919315	86.95771869	117.8935785	72.57653061	55.08381924	69.07798834	62.08090379	71.98146321	64.12172012
	85.40818134	87.38194182	108.8787585	94.93766528	92.51983377	78.32477422	67.15069949	63.02461484	52.82450859	87.38194182	84.0574235	90.70646014	100.715686	89.52925337	92.21760484	55.3214096	75.29661767	55.46307774	48.94634319	56.10058438	83.51336993	103.4000756	112.7691726	89.19531545	106.4600704	58.57977687	59.9964583	64.44129626	65.94652028	85.56856819	61.27147158
	77.10559681	92.5430951	96.6752388	112.6864226	99.36083672	69.57072994	73.01213548	83.33635211	83.33635211	107.1082704	95.95196591	90.68371102	95.49963262	83.44966936	101.5301181	59.86234378	82.61184568	78.2648071	62.8509328	67.39721065	79.2791161	107.4181677	105.2488863	95.33217122	87.85819251	65.65839522	53.30556059	76.81579424	56.76507879	113.926012	74.93207752
AMP3CPR1FUS2DOX3TMP3	AMP	CPR	FUS	DOX	TMP	AMP+CPR	AMP+FUS	AMP+DOX	AMP+TMP	CPR+FUS	CPR+DOX	CPR+TMP	FUS+DOX	FUS+TMP	DOX+TMP	AMP+CPR+FUS	AMP+CPR+DOX	AMP+CPR+TMP	AMP+FUS+DOX	AMP+FUS+TMP	AMP+DOX+TMP	CPR+FUS+DOX	CPR+FUS+TMP	CPR+DOX+TMP	FUS+DOX+TMP	AMP+CPR+FUS+DOX	AMP+CPR+FUS+TMP	AMP+CPR+DOX+TMP	AMP+FUS+DOX+TMP	CPR+FUS+DOX+TMP	AMP+CPR+FUS+DOX+TMP
	82.08819242	87.25137076	97.64552145	103.4022345	98.11649728	76.84037901	53.46209913	86.46137026	65.17857143	91.36249971	98.41014935	89.89423937	98.52587175	101.1669227	91.65615178	56.94241983	74.03425656	78.11588921	49.38046647	59.85787172	90.25145773	95.76728074	114.267361	104.8704948	115.2525276	72.57653061	57.41618076	76.36661808	55.66690962	101.34667	74.90889213
	85.40818134	87.38194182	108.8787585	94.93766528	99.16887042	78.32477422	67.15069949	63.02461484	53.62139189	87.38194182	84.0574235	93.72874953	100.715686	94.06429364	94.03097847	55.3214096	75.29661767	67.64653798	48.94634319	51.6380379	80.68000708	103.4000756	119.1159804	97.95995467	107.0647424	58.57977687	49.51301576	74.64140251	54.61306889	107.0268228	56.73809102
	77.10559681	92.5430951	96.6752388	112.6864226	102.4598102	69.57072994	73.01213548	83.33635211	92.17533056	107.1082704	95.95196591	85.41545613	95.49963262	96.96914034	101.5301181	59.86234378	82.61184568	88.11809455	62.8509328	81.30773411	83.91595725	107.4181677	102.4598102	93.16288979	94.03012491	65.65839522	44.64770875	83.4812534	59.86234378	117.6447802	57.25412063
AMP3CPR1FUS3DOX1TMP1	AMP	CPR	FUS	DOX	TMP	AMP+CPR	AMP+FUS	AMP+DOX	AMP+TMP	CPR+FUS	CPR+DOX	CPR+TMP	FUS+DOX	FUS+TMP	DOX+TMP	AMP+CPR+FUS	AMP+CPR+DOX	AMP+CPR+TMP	AMP+FUS+DOX	AMP+FUS+TMP	AMP+DOX+TMP	CPR+FUS+DOX	CPR+FUS+TMP	CPR+DOX+TMP	FUS+DOX+TMP	AMP+CPR+FUS+DOX	AMP+CPR+FUS+TMP	AMP+CPR+DOX+TMP	AMP+FUS+DOX+TMP	CPR+FUS+DOX+TMP	AMP+CPR+FUS+DOX+TMP
	82.08819242	87.25137076	95.90014577	88.42597903	99.29110555	76.84037901	67.80247813	85.58673469	64.30393586	96.64823694	87.8386749	91.06884765	77.53279883	72.8680758	96.94188901	62.66399417	87.91909621	68.20335277	92.11005831	50.81997085	55.26603499	98.70380142	102.8149304	95.47362867	76.94970845	58.29081633	84.23833819	73.15962099	67.03717201	99.87840969	75.7835277
	85.40818134	87.38194182	92.29679476	90.40423121	98.2621836	78.32477422	62.54648486	73.59659996	48.66300691	99.7733283	90.10200227	85.56856819	78.69665309	54.89640517	92.82206271	61.12980344	72.46325483	57.16309545	72.46325483	60.84646715	53.3380556	93.42652059	106.7245939	97.95995467	83.51336993	63.39649371	36.39100407	68.49654684	42.57127678	120.627125	90.88011333
	77.10559681	92.5430951	84.78536497	95.95196591	93.47278714	69.57072994	71.56312262	77.68520196	82.46694439	106.4884757	96.5717606	99.36083672	94.63865242	89.42220612	108.9676545	56.38471291	73.62796595	73.62796595	73.48306466	82.75674697	86.08947654	101.2202208	104.938989	84.79566144	93.47944213	49.57435247	66.81760551	79.71381996	58.99293606	102.4598102	73.19326209
AMP3CPR1FUS3DOX1TMP2	AMP	CPR	FUS	DOX	TMP	AMP+CPR	AMP+FUS	AMP+DOX	AMP+TMP	CPR+FUS	CPR+DOX	CPR+TMP	FUS+DOX	FUS+TMP	DOX+TMP	AMP+CPR+FUS	AMP+CPR+DOX	AMP+CPR+TMP	AMP+FUS+DOX	AMP+FUS+TMP	AMP+DOX+TMP	CPR+FUS+DOX	CPR+FUS+TMP	CPR+DOX+TMP	FUS+DOX+TMP	AMP+CPR+FUS+DOX	AMP+CPR+FUS+TMP	AMP+CPR+DOX+TMP	AMP+FUS+DOX+TMP	CPR+FUS+DOX+TMP	AMP+CPR+FUS+DOX+TMP
	82.08819242	87.25137076	95.90014577	88.42597903	94.59267246	76.84037901	67.80247813	85.58673469	71.00947522	96.64823694	87.8386749	95.1799766	77.53279883	57.99927114	99.58475762	62.66399417	87.91909621	74.90889213	92.11005831	61.02405248	55.66690962	98.70380142	95.1799766	98.70380142	87.44533528	58.29081633	77.53279883	77.24125364	62.77332362	109.5689279	81.03134111
	85.40818134	87.38194182	92.29679476	90.40423121	92.51983377	78.32477422	62.54648486	73.59659996	52.82450859	99.7733283	90.10200227	90.70646014	78.69665309	56.59642288	96.14658104	61.12980344	72.46325483	55.46307774	72.46325483	45.40463963	57.44643173	93.42652059	109.7468833	106.120136	71.0465734	63.39649371	77.56330795	51.69116345	67.92987427	122.7427276	70.47990083
	77.10559681	92.5430951	84.78536497	95.95196591	99.36083672	69.57072994	71.56312262	77.68520196	83.33635211	106.4884757	96.5717606	90.68371102	94.63865242	90.87121898	100.6004261	56.38471291	73.62796595	78.2648071	73.48306466	83.62615468	80.00362253	101.2202208	88.82432694	91.61340306	90.87121898	49.57435247	72.61365695	74.78717624	72.0340518	108.0379624	77.2504981
AMP3CPR1FUS3DOX1TMP3	AMP	CPR	FUS	DOX	TMP	AMP+CPR	AMP+FUS	AMP+DOX	AMP+TMP	CPR+FUS	CPR+DOX	CPR+TMP	FUS+DOX	FUS+TMP	DOX+TMP	AMP+CPR+FUS	AMP+CPR+DOX	AMP+CPR+TMP	AMP+FUS+DOX	AMP+FUS+TMP	AMP+DOX+TMP	CPR+FUS+DOX	CPR+FUS+TMP	CPR+DOX+TMP	FUS+DOX+TMP	AMP+CPR+FUS+DOX	AMP+CPR+FUS+TMP	AMP+CPR+DOX+TMP	AMP+FUS+DOX+TMP	CPR+FUS+DOX+TMP	AMP+CPR+FUS+DOX+TMP
	82.08819242	87.25137076	95.90014577	88.42597903	98.11649728	76.84037901	67.80247813	85.58673469	65.17857143	96.64823694	87.8386749	89.89423937	77.53279883	66.74562682	99.29110555	62.66399417	87.91909621	78.11588921	92.11005831	66.56341108	64.88702624	98.70380142	108.3943197	99.29110555	83.65524781	58.29081633	75.20043732	71.70189504	61.02405248	106.3387552	89.4861516
	85.40818134	87.38194182	92.29679476	90.40423121	99.16887042	78.32477422	62.54648486	73.59659996	53.62139189	99.7733283	90.10200227	93.72874953	78.69665309	65.09651142	99.16887042	61.12980344	72.46325483	67.64653798	72.46325483	59.28811758	56.31308659	93.42652059	119.4182093	104.9112202	73.59659996	63.39649371	74.44660882	63.87462369	73.31326368	114.5825463	71.32990969
	77.10559681	92.5430951	84.78536497	95.95196591	102.4598102	69.57072994	71.56312262	77.68520196	92.17533056	106.4884757	96.5717606	85.41545613	94.63865242	81.88733925	104.938989	56.38471291	73.62796595	88.11809455	73.48306466	79.56891867	90.43651512	101.2202208	109.2775518	86.03525082	75.07697881	49.57435247	76.09128781	86.95888426	71.88915052	105.868681	80.87303025
AMP3CPR1FUS3DOX2TMP1	AMP	CPR	FUS	DOX	TMP	AMP+CPR	AMP+FUS	AMP+DOX	AMP+TMP	CPR+FUS	CPR+DOX	CPR+TMP	FUS+DOX	FUS+TMP	DOX+TMP	AMP+CPR+FUS	AMP+CPR+DOX	AMP+CPR+TMP	AMP+FUS+DOX	AMP+FUS+TMP	AMP+DOX+TMP	CPR+FUS+DOX	CPR+FUS+TMP	CPR+DOX+TMP	FUS+DOX+TMP	AMP+CPR+FUS+DOX	AMP+CPR+FUS+TMP	AMP+CPR+DOX+TMP	AMP+FUS+DOX+TMP	CPR+FUS+DOX+TMP	AMP+CPR+FUS+DOX+TMP
	82.08819242	87.25137076	95.90014577	92.53710799	99.29110555	76.84037901	67.80247813	80.92201166	64.30393586	96.64823694	94.59267246	91.06884765	91.52696793	72.8680758	100.7593659	62.66399417	88.2106414	68.20335277	80.73979592	50.81997085	59.34766764	103.6958866	102.8149304	101.053018	64.59548105	77.53279883	84.23833819	69.84329446	60.33163265	93.41806419	84.82142857
	85.40818134	87.38194182	92.29679476	89.19531545	98.2621836	78.32477422	62.54648486	63.39649371	48.66300691	99.7733283	87.38194182	85.56856819	68.49654684	54.89640517	97.35549679	61.12980344	79.26332566	57.16309545	82.38002479	60.84646715	72.46325483	95.23989422	106.7245939	107.9335096	73.0299274	66.51319285	36.39100407	63.30795112	81.53001594	115.791462	67.64653798
	77.10559681	92.5430951	84.78536497	100.9103235	93.47278714	69.57072994	71.56312262	78.55460967	82.46694439	106.4884757	99.36083672	99.36083672	73.62796595	89.42220612	104.0092969	56.38471291	77.10559681	73.62796595	72.0340518	82.75674697	90.14671255	106.4884757	104.938989	92.5430951	101.8837167	58.41333092	66.81760551	76.2361891	77.2504981	107.7280651	73.0483608
AMP3CPR1FUS3DOX2TMP2	AMP	CPR	FUS	DOX	TMP	AMP+CPR	AMP+FUS	AMP+DOX	AMP+TMP	CPR+FUS	CPR+DOX	CPR+TMP	FUS+DOX	FUS+TMP	DOX+TMP	AMP+CPR+FUS	AMP+CPR+DOX	AMP+CPR+TMP	AMP+FUS+DOX	AMP+FUS+TMP	AMP+DOX+TMP	CPR+FUS+DOX	CPR+FUS+TMP	CPR+DOX+TMP	FUS+DOX+TMP	AMP+CPR+FUS+DOX	AMP+CPR+FUS+TMP	AMP+CPR+DOX+TMP	AMP+FUS+DOX+TMP	CPR+FUS+DOX+TMP	AMP+CPR+FUS+DOX+TMP
	82.08819242	87.25137076	95.90014577	92.53710799	94.59267246	76.84037901	67.80247813	80.92201166	71.00947522	96.64823694	94.59267246	95.1799766	91.52696793	57.99927114	103.6958866	62.66399417	88.2106414	74.90889213	80.73979592	61.02405248	66.63629738	103.6958866	95.1799766	86.07676249	81.61443149	77.53279883	77.53279883	82.78061224	66.16253644	87.54502283	84.23833819
	85.40818134	87.38194182	92.29679476	89.19531545	92.51983377	78.32477422	62.54648486	63.39649371	52.82450859	99.7733283	87.38194182	90.70646014	68.49654684	56.59642288	107.0268228	61.12980344	79.26332566	55.46307774	82.38002479	45.40463963	85.49672392	95.23989422	109.7468833	97.35549679	98.24685674	66.51319285	77.56330795	66.70798654	70.76323712	96.44880997	63.39649371
	77.10559681	92.5430951	84.78536497	100.9103235	99.36083672	69.57072994	71.56312262	78.55460967	83.33635211	106.4884757	99.36083672	90.68371102	73.62796595	90.87121898	89.13422429	56.38471291	77.10559681	78.2648071	72.0340518	83.62615468	99.71019743	106.4884757	88.82432694	86.03525082	99.85509871	58.41333092	72.61365695	91.88552798	74.64227495	103.3895022	66.23800036
AMP3CPR1FUS3DOX2TMP3	AMP	CPR	FUS	DOX	TMP	AMP+CPR	AMP+FUS	AMP+DOX	AMP+TMP	CPR+FUS	CPR+DOX	CPR+TMP	FUS+DOX	FUS+TMP	DOX+TMP	AMP+CPR+FUS	AMP+CPR+DOX	AMP+CPR+TMP	AMP+FUS+DOX	AMP+FUS+TMP	AMP+DOX+TMP	CPR+FUS+DOX	CPR+FUS+TMP	CPR+DOX+TMP	FUS+DOX+TMP	AMP+CPR+FUS+DOX	AMP+CPR+FUS+TMP	AMP+CPR+DOX+TMP	AMP+FUS+DOX+TMP	CPR+FUS+DOX+TMP	AMP+CPR+FUS+DOX+TMP
	82.08819242	87.25137076	95.90014577	92.53710799	98.11649728	76.84037901	67.80247813	80.92201166	65.17857143	96.64823694	94.59267246	89.89423937	91.52696793	66.74562682	103.9895386	62.66399417	88.2106414	78.11588921	80.73979592	66.56341108	70.2441691	103.6958866	108.3943197	86.95771869	108.4365889	77.53279883	75.20043732	73.15962099	77.24125364	93.12441212	85.11297376
	85.40818134	87.38194182	92.29679476	89.19531545	99.16887042	78.32477422	62.54648486	63.39649371	53.62139189	99.7733283	87.38194182	93.72874953	68.49654684	65.09651142	98.56441254	61.12980344	79.26332566	67.64653798	82.38002479	59.28811758	84.64671507	95.23989422	119.4182093	91.00868908	88.61342306	66.51319285	74.44660882	76.05808394	72.74659111	102.7956177	65.66318399
	77.10559681	92.5430951	84.78536497	100.9103235	102.4598102	69.57072994	71.56312262	78.55460967	92.17533056	106.4884757	99.36083672	85.41545613	73.62796595	81.88733925	91.92330041	56.38471291	77.10559681	88.11809455	72.0340518	79.56891867	85.79967397	106.4884757	109.2775518	86.34514817	99.56529614	58.41333092	76.09128781	89.56710741	76.96069553	104.3191943	69.71563123
AMP3CPR1FUS3DOX3TMP1	AMP	CPR	FUS	DOX	TMP	AMP+CPR	AMP+FUS	AMP+DOX	AMP+TMP	CPR+FUS	CPR+DOX	CPR+TMP	FUS+DOX	FUS+TMP	DOX+TMP	AMP+CPR+FUS	AMP+CPR+DOX	AMP+CPR+TMP	AMP+FUS+DOX	AMP+FUS+TMP	AMP+DOX+TMP	CPR+FUS+DOX	CPR+FUS+TMP	CPR+DOX+TMP	FUS+DOX+TMP	AMP+CPR+FUS+DOX	AMP+CPR+FUS+TMP	AMP+CPR+DOX+TMP	AMP+FUS+DOX+TMP	CPR+FUS+DOX+TMP	AMP+CPR+FUS+DOX+TMP
	82.08819242	87.25137076	95.90014577	103.4022345	99.29110555	76.84037901	67.80247813	86.46137026	64.30393586	96.64823694	98.41014935	91.06884765	48.66982507	72.8680758	106.6324073	62.66399417	74.03425656	68.20335277	54.50072886	50.81997085	72.75874636	106.9260593	102.8149304	88.13232696	102.3141399	56.06778426	84.23833819	78.29810496	95.90014577	91.94980385	75.7835277
	85.40818134	87.38194182	92.29679476	94.93766528	98.2621836	78.32477422	62.54648486	63.02461484	48.66300691	99.7733283	84.0574235	85.56856819	61.41313972	54.89640517	100.6800151	61.12980344	75.29661767	57.16309545	72.74659111	60.84646715	92.86346733	99.47109936	106.7245939	87.68417076	85.78006021	73.0299274	36.39100407	92.20825217	91.16344962	95.8443521	56.02975031
	77.10559681	92.5430951	84.78536497	112.6864226	93.47278714	69.57072994	71.56312262	83.33635211	82.46694439	106.4884757	95.95196591	99.36083672	68.41151965	89.42220612	108.6577571	56.38471291	82.61184568	73.62796595	79.2791161	82.75674697	88.98750226	104.938989	104.938989	95.95196591	101.5939141	68.26661837	66.81760551	89.27730484	86.81398297	112.99632	63.19507336
AMP3CPR1FUS3DOX3TMP2	AMP	CPR	FUS	DOX	TMP	AMP+CPR	AMP+FUS	AMP+DOX	AMP+TMP	CPR+FUS	CPR+DOX	CPR+TMP	FUS+DOX	FUS+TMP	DOX+TMP	AMP+CPR+FUS	AMP+CPR+DOX	AMP+CPR+TMP	AMP+FUS+DOX	AMP+FUS+TMP	AMP+DOX+TMP	CPR+FUS+DOX	CPR+FUS+TMP	CPR+DOX+TMP	FUS+DOX+TMP	AMP+CPR+FUS+DOX	AMP+CPR+FUS+TMP	AMP+CPR+DOX+TMP	AMP+FUS+DOX+TMP	CPR+FUS+DOX+TMP	AMP+CPR+FUS+DOX+TMP
	82.08819242	87.25137076	95.90014577	103.4022345	94.59267246	76.84037901	67.80247813	86.46137026	71.00947522	96.64823694	98.41014935	95.1799766	48.66982507	57.99927114	102.5212783	62.66399417	74.03425656	74.90889213	54.50072886	61.02405248	86.8622449	106.9260593	95.1799766	86.95771869	98.52405248	56.06778426	77.53279883	69.07798834	85.98760933	92.24345592	70.53571429
	85.40818134	87.38194182	92.29679476	94.93766528	92.51983377	78.32477422	62.54648486	63.02461484	52.82450859	99.7733283	84.0574235	90.70646014	61.41313972	56.59642288	92.21760484	61.12980344	75.29661767	55.46307774	72.74659111	45.40463963	83.51336993	99.47109936	109.7468833	89.19531545	88.89675934	73.0299274	77.56330795	64.44129626	71.0465734	111.258028	54.61306889
	77.10559681	92.5430951	84.78536497	112.6864226	99.36083672	69.57072994	71.56312262	83.33635211	83.33635211	106.4884757	95.95196591	90.68371102	68.41151965	90.87121898	101.5301181	56.38471291	82.61184568	78.2648071	79.2791161	83.62615468	79.2791161	104.938989	88.82432694	95.33217122	98.69588843	68.26661837	72.61365695	76.81579424	82.75674697	106.798373	68.55642094
AMP3CPR1FUS3DOX3TMP3	AMP	CPR	FUS	DOX	TMP	AMP+CPR	AMP+FUS	AMP+DOX	AMP+TMP	CPR+FUS	CPR+DOX	CPR+TMP	FUS+DOX	FUS+TMP	DOX+TMP	AMP+CPR+FUS	AMP+CPR+DOX	AMP+CPR+TMP	AMP+FUS+DOX	AMP+FUS+TMP	AMP+DOX+TMP	CPR+FUS+DOX	CPR+FUS+TMP	CPR+DOX+TMP	FUS+DOX+TMP	AMP+CPR+FUS+DOX	AMP+CPR+FUS+TMP	AMP+CPR+DOX+TMP	AMP+FUS+DOX+TMP	CPR+FUS+DOX+TMP	AMP+CPR+FUS+DOX+TMP
	82.08819242	87.25137076	95.90014577	103.4022345	98.11649728	76.84037901	67.80247813	86.46137026	65.17857143	96.64823694	98.41014935	89.89423937	48.66982507	66.74562682	91.65615178	62.66399417	74.03425656	78.11588921	54.50072886	66.56341108	90.25145773	106.9260593	108.3943197	104.8704948	96.48323615	56.06778426	75.20043732	76.36661808	82.19752187	117.2038817	83.36370262
	85.40818134	87.38194182	92.29679476	94.93766528	99.16887042	78.32477422	62.54648486	63.02461484	53.62139189	99.7733283	84.0574235	93.72874953	61.41313972	65.09651142	94.03097847	61.12980344	75.29661767	67.64653798	72.74659111	59.28811758	80.68000708	99.47109936	119.4182093	97.95995467	85.78006021	73.0299274	74.44660882	74.64140251	85.21338764	108.8401964	48.94634319
	77.10559681	92.5430951	84.78536497	112.6864226	102.4598102	69.57072994	71.56312262	83.33635211	92.17533056	106.4884757	95.95196591	85.41545613	68.41151965	81.88733925	101.5301181	56.38471291	82.61184568	88.11809455	79.2791161	79.56891867	83.91595725	104.938989	109.2775518	93.16288979	94.34884985	68.26661837	76.09128781	83.4812534	90.29161384	117.3348828	54.21119362
AMP3CPR2FUS1DOX1TMP1	AMP	CPR	FUS	DOX	TMP	AMP+CPR	AMP+FUS	AMP+DOX	AMP+TMP	CPR+FUS	CPR+DOX	CPR+TMP	FUS+DOX	FUS+TMP	DOX+TMP	AMP+CPR+FUS	AMP+CPR+DOX	AMP+CPR+TMP	AMP+FUS+DOX	AMP+FUS+TMP	AMP+DOX+TMP	CPR+FUS+DOX	CPR+FUS+TMP	CPR+DOX+TMP	FUS+DOX+TMP	AMP+CPR+FUS+DOX	AMP+CPR+FUS+TMP	AMP+CPR+DOX+TMP	AMP+FUS+DOX+TMP	CPR+FUS+DOX+TMP	AMP+CPR+FUS+DOX+TMP
	82.08819242	86.38437378	72.56876735	88.42597903	99.29110555	86.46137026	36.24271137	85.58673469	64.30393586	87.66821798	81.50576584	81.50576584	74.33067976	84.90215421	96.94188901	47.33965015	90.54300292	59.74854227	44.40597668	42.83892128	55.26603499	76.5171141	85.32061716	78.27781471	75.7989401	40.92565598	50.81997085	76.36661808	43.25801749	81.50576584	38.88483965
	85.40818134	98.12693389	80.73290518	90.40423121	98.2621836	68.40800425	44.12962635	73.59659996	48.66300691	86.20355717	89.52925337	75.31946052	65.01700038	76.80392898	92.82206271	32.58367275	79.17478307	62.54648486	39.31290951	49.22967948	53.3380556	72.46616435	86.50589319	80.59144484	98.56441254	36.55038073	56.59642288	71.61324597	46.4671507	88.01757328	43.70462192
	77.10559681	85.26818516	93.47278714	95.95196591	93.47278714	73.33816338	62.28944032	77.68520196	82.46694439	76.10213079	89.08890522	76.39603233	63.10284718	101.2202208	108.9676545	36.78681398	71.88915052	79.42401739	55.04437602	58.41333092	86.08947654	78.74724467	66.40337987	73.51212344	86.34514817	52.1463503	36.78681398	85.22006883	42.58286542	62.58265981	58.99293606
AMP3CPR2FUS1DOX1TMP2	AMP	CPR	FUS	DOX	TMP	AMP+CPR	AMP+FUS	AMP+DOX	AMP+TMP	CPR+FUS	CPR+DOX	CPR+TMP	FUS+DOX	FUS+TMP	DOX+TMP	AMP+CPR+FUS	AMP+CPR+DOX	AMP+CPR+TMP	AMP+FUS+DOX	AMP+FUS+TMP	AMP+DOX+TMP	CPR+FUS+DOX	CPR+FUS+TMP	CPR+DOX+TMP	FUS+DOX+TMP	AMP+CPR+FUS+DOX	AMP+CPR+FUS+TMP	AMP+CPR+DOX+TMP	AMP+FUS+DOX+TMP	CPR+FUS+DOX+TMP	AMP+CPR+FUS+DOX+TMP
	82.08819242	86.38437378	72.56876735	88.42597903	94.59267246	86.46137026	36.24271137	85.58673469	71.00947522	87.66821798	81.50576584	73.58261308	74.33067976	94.59267246	99.58475762	47.33965015	90.54300292	66.16253644	44.40597668	46.04591837	55.66690962	76.5171141	77.69091451	80.33196543	69.92589874	40.92565598	47.61297376	89.4861516	57.5255102	83.26646645	66.74562682
	85.40818134	98.12693389	80.73290518	90.40423121	92.51983377	68.40800425	44.12962635	73.59659996	52.82450859	86.20355717	89.52925337	78.94749274	65.01700038	102.7956177	96.14658104	32.58367275	79.17478307	72.46325483	39.31290951	54.11723039	57.44643173	72.46616435	78.04048468	68.49800411	77.40838685	36.55038073	41.01292722	49.6546839	49.01717726	84.69187708	51.77970604
	77.10559681	85.26818516	93.47278714	95.95196591	99.36083672	73.33816338	62.28944032	77.68520196	83.33635211	76.10213079	89.08890522	72.63041881	63.10284718	102.1499128	100.6004261	36.78681398	71.88915052	84.64046368	55.04437602	66.81760551	80.00362253	78.74724467	71.69360764	69.39750184	73.94925431	52.1463503	66.34667633	77.68520196	47.50950915	81.6862601	58.99293606
AMP3CPR2FUS1DOX1TMP3	AMP	CPR	FUS	DOX	TMP	AMP+CPR	AMP+FUS	AMP+DOX	AMP+TMP	CPR+FUS	CPR+DOX	CPR+TMP	FUS+DOX	FUS+TMP	DOX+TMP	AMP+CPR+FUS	AMP+CPR+DOX	AMP+CPR+TMP	AMP+FUS+DOX	AMP+FUS+TMP	AMP+DOX+TMP	CPR+FUS+DOX	CPR+FUS+TMP	CPR+DOX+TMP	FUS+DOX+TMP	AMP+CPR+FUS+DOX	AMP+CPR+FUS+TMP	AMP+CPR+DOX+TMP	AMP+FUS+DOX+TMP	CPR+FUS+DOX+TMP	AMP+CPR+FUS+DOX+TMP
	82.08819242	86.38437378	72.56876735	88.42597903	98.11649728	86.46137026	36.24271137	85.58673469	65.17857143	87.66821798	81.50576584	76.5171141	74.33067976	73.74337562	99.29110555	47.33965015	90.54300292	70.82725948	44.40597668	40.03279883	64.88702624	76.5171141	72.70226278	69.76776176	95.76728074	40.92565598	39.44970845	88.31997085	41.78206997	77.69091451	52.58746356
	85.40818134	98.12693389	80.73290518	90.40423121	99.16887042	68.40800425	44.12962635	73.59659996	53.62139189	86.20355717	89.52925337	76.52880459	65.01700038	98.56441254	99.16887042	32.58367275	79.17478307	66.51319285	39.31290951	58.36727466	56.31308659	72.46616435	92.25027754	76.96341262	111.258028	36.55038073	34.21285638	62.12148043	43.2796175	82.57552495	53.05471932
	77.10559681	85.26818516	93.47278714	95.95196591	102.4598102	73.33816338	62.28944032	77.68520196	92.17533056	76.10213079	89.08890522	69.10360029	63.10284718	94.09258183	104.938989	36.78681398	71.88915052	96.81217171	55.04437602	48.94040935	90.43651512	78.74724467	84.62527553	77.92064658	95.33217122	52.1463503	45.46277848	79.71381996	40.30067017	86.68258633	51.27694258
AMP3CPR2FUS1DOX2TMP1	AMP	CPR	FUS	DOX	TMP	AMP+CPR	AMP+FUS	AMP+DOX	AMP+TMP	CPR+FUS	CPR+DOX	CPR+TMP	FUS+DOX	FUS+TMP	DOX+TMP	AMP+CPR+FUS	AMP+CPR+DOX	AMP+CPR+TMP	AMP+FUS+DOX	AMP+FUS+TMP	AMP+DOX+TMP	CPR+FUS+DOX	CPR+FUS+TMP	CPR+DOX+TMP	FUS+DOX+TMP	AMP+CPR+FUS+DOX	AMP+CPR+FUS+TMP	AMP+CPR+DOX+TMP	AMP+FUS+DOX+TMP	CPR+FUS+DOX+TMP	AMP+CPR+FUS+DOX+TMP
	82.08819242	86.38437378	72.56876735	92.53710799	99.29110555	86.46137026	36.24271137	80.92201166	64.30393586	87.66821798	80.91886563	81.50576584	82.2592856	84.90215421	100.7593659	47.33965015	75.96574344	59.74854227	50.54664723	42.83892128	59.34766764	84.73371696	85.32061716	74.16951329	96.0609328	54.79227405	50.81997085	80.44825073	40.32434402	75.34331369	56.83309038
	85.40818134	98.12693389	80.73290518	89.19531545	98.2621836	68.40800425	44.12962635	63.39649371	48.66300691	86.20355717	70.48208423	75.31946052	86.77748394	76.80392898	97.35549679	32.58367275	56.22454401	62.54648486	33.78785196	49.22967948	72.46325483	94.66896568	86.50589319	78.3428207	101.8889309	64.81317514	56.59642288	57.44643173	47.52966177	87.11056523	61.83814415
	77.10559681	85.26818516	93.47278714	100.9103235	93.47278714	73.33816338	62.28944032	78.55460967	82.46694439	76.10213079	72.86921381	76.39603233	100.6004261	101.2202208	104.0092969	36.78681398	70.44013766	79.42401739	73.30193805	58.41333092	90.14671255	95.20573108	66.40337987	64.05216752	73.32945962	43.92320232	36.78681398	74.78717624	51.83843507	81.09845702	50.40753487
AMP3CPR2FUS1DOX2TMP2	AMP	CPR	FUS	DOX	TMP	AMP+CPR	AMP+FUS	AMP+DOX	AMP+TMP	CPR+FUS	CPR+DOX	CPR+TMP	FUS+DOX	FUS+TMP	DOX+TMP	AMP+CPR+FUS	AMP+CPR+DOX	AMP+CPR+TMP	AMP+FUS+DOX	AMP+FUS+TMP	AMP+DOX+TMP	CPR+FUS+DOX	CPR+FUS+TMP	CPR+DOX+TMP	FUS+DOX+TMP	AMP+CPR+FUS+DOX	AMP+CPR+FUS+TMP	AMP+CPR+DOX+TMP	AMP+FUS+DOX+TMP	CPR+FUS+DOX+TMP	AMP+CPR+FUS+DOX+TMP
	82.08819242	86.38437378	72.56876735	92.53710799	94.59267246	86.46137026	36.24271137	80.92201166	71.00947522	87.66821798	80.91886563	73.58261308	82.2592856	94.59267246	103.6958866	47.33965015	75.96574344	66.16253644	50.54664723	46.04591837	66.63629738	84.73371696	77.69091451	83.55991655	70.80685494	54.79227405	47.61297376	69.36953353	34.20189504	85.32061716	44.13265306
	85.40818134	98.12693389	80.73290518	89.19531545	92.51983377	68.40800425	44.12962635	63.39649371	52.82450859	86.20355717	70.48208423	78.94749274	86.77748394	102.7956177	107.0268228	32.58367275	56.22454401	72.46325483	33.78785196	54.11723039	85.49672392	94.66896568	78.04048468	81.6685169	82.24404987	64.81317514	41.01292722	66.51319285	28.82946697	74.11011645	45.9713122
	77.10559681	85.26818516	93.47278714	100.9103235	99.36083672	73.33816338	62.28944032	78.55460967	83.33635211	76.10213079	72.86921381	72.63041881	100.6004261	102.1499128	89.13422429	36.78681398	70.44013766	84.64046368	73.30193805	66.81760551	99.71019743	95.20573108	71.69360764	84.09257899	84.79566144	43.92320232	66.34667633	70.44013766	52.99764535	73.14474651	50.69733744
AMP3CPR2FUS1DOX2TMP3	AMP	CPR	FUS	DOX	TMP	AMP+CPR	AMP+FUS	AMP+DOX	AMP+TMP	CPR+FUS	CPR+DOX	CPR+TMP	FUS+DOX	FUS+TMP	DOX+TMP	AMP+CPR+FUS	AMP+CPR+DOX	AMP+CPR+TMP	AMP+FUS+DOX	AMP+FUS+TMP	AMP+DOX+TMP	CPR+FUS+DOX	CPR+FUS+TMP	CPR+DOX+TMP	FUS+DOX+TMP	AMP+CPR+FUS+DOX	AMP+CPR+FUS+TMP	AMP+CPR+DOX+TMP	AMP+FUS+DOX+TMP	CPR+FUS+DOX+TMP	AMP+CPR+FUS+DOX+TMP
	82.08819242	86.38437378	72.56876735	92.53710799	98.11649728	86.46137026	36.24271137	80.92201166	65.17857143	87.66821798	80.91886563	76.5171141	82.2592856	73.74337562	103.9895386	47.33965015	75.96574344	70.82725948	50.54664723	40.03279883	70.2441691	84.73371696	72.70226278	96.17827094	79.32276492	54.79227405	39.44970845	70.13483965	39.75947522	103.2210734	45.29883382
	85.40818134	98.12693389	80.73290518	89.19531545	99.16887042	68.40800425	44.12962635	63.39649371	53.62139189	86.20355717	70.48208423	76.52880459	86.77748394	98.56441254	98.56441254	32.58367275	56.22454401	66.51319285	33.78785196	58.36727466	84.64671507	94.66896568	92.25027754	96.48298179	99.47109936	64.81317514	34.21285638	73.0299274	43.35045157	88.01757328	58.72144502
	77.10559681	85.26818516	93.47278714	100.9103235	102.4598102	73.33816338	62.28944032	78.55460967	92.17533056	76.10213079	72.86921381	69.10360029	100.6004261	94.09258183	91.92330041	36.78681398	70.44013766	96.81217171	73.30193805	48.94040935	85.79967397	95.20573108	84.62527553	72.86921381	94.71237653	43.92320232	45.46277848	88.69769969	54.15685564	82.86186627	51.56674515
AMP3CPR2FUS1DOX3TMP1	AMP	CPR	FUS	DOX	TMP	AMP+CPR	AMP+FUS	AMP+DOX	AMP+TMP	CPR+FUS	CPR+DOX	CPR+TMP	FUS+DOX	FUS+TMP	DOX+TMP	AMP+CPR+FUS	AMP+CPR+DOX	AMP+CPR+TMP	AMP+FUS+DOX	AMP+FUS+TMP	AMP+DOX+TMP	CPR+FUS+DOX	CPR+FUS+TMP	CPR+DOX+TMP	FUS+DOX+TMP	AMP+CPR+FUS+DOX	AMP+CPR+FUS+TMP	AMP+CPR+DOX+TMP	AMP+FUS+DOX+TMP	CPR+FUS+DOX+TMP	AMP+CPR+FUS+DOX+TMP
	82.08819242	86.38437378	72.56876735	103.4022345	99.29110555	86.46137026	36.24271137	86.46137026	64.30393586	87.66821798	79.04812123	81.50576584	87.8386749	84.90215421	106.6324073	47.33965015	64.30393586	59.74854227	47.04810496	42.83892128	72.75874636	75.6367638	85.32061716	98.81932186	81.96563353	42.54737609	50.81997085	76.25728863	46.04591837	98.23242165	51.12973761
	85.40818134	98.12693389	80.73290518	94.93766528	98.2621836	68.40800425	44.12962635	63.02461484	48.66300691	86.20355717	75.62179654	75.31946052	94.93766528	76.80392898	100.6800151	32.58367275	51.77970604	62.54648486	37.40038959	49.22967948	92.86346733	71.55915629	86.50589319	89.22691735	94.93766528	39.59624579	56.59642288	70.76323712	59.71312201	85.59888514	51.21303347
	77.10559681	85.26818516	93.47278714	112.6864226	93.47278714	73.33816338	62.28944032	83.33635211	82.46694439	76.10213079	75.5143277	76.39603233	106.1785783	101.2202208	108.6577571	36.78681398	74.49737366	79.42401739	53.01575801	58.41333092	88.98750226	73.75091844	66.40337987	85.50698016	80.76699593	60.55062489	36.78681398	87.5384894	62.27132766	81.98016165	48.66871943
AMP3CPR2FUS1DOX3TMP2	AMP	CPR	FUS	DOX	TMP	AMP+CPR	AMP+FUS	AMP+DOX	AMP+TMP	CPR+FUS	CPR+DOX	CPR+TMP	FUS+DOX	FUS+TMP	DOX+TMP	AMP+CPR+FUS	AMP+CPR+DOX	AMP+CPR+TMP	AMP+FUS+DOX	AMP+FUS+TMP	AMP+DOX+TMP	CPR+FUS+DOX	CPR+FUS+TMP	CPR+DOX+TMP	FUS+DOX+TMP	AMP+CPR+FUS+DOX	AMP+CPR+FUS+TMP	AMP+CPR+DOX+TMP	AMP+FUS+DOX+TMP	CPR+FUS+DOX+TMP	AMP+CPR+FUS+DOX+TMP
	82.08819242	86.38437378	72.56876735	103.4022345	94.59267246	86.46137026	36.24271137	86.46137026	71.00947522	87.66821798	79.04812123	73.58261308	87.8386749	94.59267246	102.5212783	47.33965015	64.30393586	66.16253644	47.04810496	46.04591837	86.8622449	75.6367638	77.69091451	86.78786767	98.11649728	42.54737609	47.61297376	69.55174927	41.21720117	95.59137073	54.62827988
	85.40818134	98.12693389	80.73290518	94.93766528	92.51983377	68.40800425	44.12962635	63.02461484	52.82450859	86.20355717	75.62179654	78.94749274	94.93766528	102.7956177	92.21760484	32.58367275	51.77970604	72.46325483	37.40038959	54.11723039	83.51336993	71.55915629	78.04048468	78.64515672	89.79977333	39.59624579	41.01292722	95.60828759	45.61714185	83.48253301	46.75048698
	77.10559681	85.26818516	93.47278714	112.6864226	99.36083672	73.33816338	62.28944032	83.33635211	83.33635211	76.10213079	75.5143277	72.63041881	106.1785783	102.1499128	101.5301181	36.78681398	74.49737366	84.64046368	53.01575801	66.81760551	79.2791161	73.75091844	71.69360764	89.97060985	95.64206856	60.55062489	66.34667633	75.65658395	69.22658939	94.32402645	44.79261003
AMP3CPR2FUS1DOX3TMP3	AMP	CPR	FUS	DOX	TMP	AMP+CPR	AMP+FUS	AMP+DOX	AMP+TMP	CPR+FUS	CPR+DOX	CPR+TMP	FUS+DOX	FUS+TMP	DOX+TMP	AMP+CPR+FUS	AMP+CPR+DOX	AMP+CPR+TMP	AMP+FUS+DOX	AMP+FUS+TMP	AMP+DOX+TMP	CPR+FUS+DOX	CPR+FUS+TMP	CPR+DOX+TMP	FUS+DOX+TMP	AMP+CPR+FUS+DOX	AMP+CPR+FUS+TMP	AMP+CPR+DOX+TMP	AMP+FUS+DOX+TMP	CPR+FUS+DOX+TMP	AMP+CPR+FUS+DOX+TMP
	82.08819242	86.38437378	72.56876735	103.4022345	98.11649728	86.46137026	36.24271137	86.46137026	65.17857143	87.66821798	79.04812123	76.5171141	87.8386749	73.74337562	91.65615178	47.33965015	64.30393586	70.82725948	47.04810496	40.03279883	90.25145773	75.6367638	72.70226278	86.09092368	76.97354837	42.54737609	39.44970845	92.00072886	46.15524781	100.5800225	57.54373178
	85.40818134	98.12693389	80.73290518	94.93766528	99.16887042	68.40800425	44.12962635	63.02461484	53.62139189	86.20355717	75.62179654	76.52880459	94.93766528	98.56441254	94.03097847	32.58367275	51.77970604	66.51319285	37.40038959	58.36727466	80.68000708	71.55915629	92.25027754	100.8479581	82.84850774	39.59624579	34.21285638	79.17478307	57.23392952	74.11011645	40.51708872
	77.10559681	85.26818516	93.47278714	112.6864226	102.4598102	73.33816338	62.28944032	83.33635211	92.17533056	76.10213079	75.5143277	69.10360029	106.1785783	94.09258183	101.5301181	36.78681398	74.49737366	96.81217171	53.01575801	48.94040935	83.91595725	73.75091844	84.62527553	96.43644379	80.45709859	60.55062489	45.46277848	80.87303025	71.83481253	86.09478325	47.69063575
AMP3CPR2FUS2DOX1TMP1	AMP	CPR	FUS	DOX	TMP	AMP+CPR	AMP+FUS	AMP+DOX	AMP+TMP	CPR+FUS	CPR+DOX	CPR+TMP	FUS+DOX	FUS+TMP	DOX+TMP	AMP+CPR+FUS	AMP+CPR+DOX	AMP+CPR+TMP	AMP+FUS+DOX	AMP+FUS+TMP	AMP+DOX+TMP	CPR+FUS+DOX	CPR+FUS+TMP	CPR+DOX+TMP	FUS+DOX+TMP	AMP+CPR+FUS+DOX	AMP+CPR+FUS+TMP	AMP+CPR+DOX+TMP	AMP+FUS+DOX+TMP	CPR+FUS+DOX+TMP	AMP+CPR+FUS+DOX+TMP
	82.08819242	86.38437378	97.64552145	88.42597903	99.29110555	86.46137026	53.46209913	85.58673469	64.30393586	96.47172104	81.50576584	81.50576584	90.8961691	99.11277196	96.94188901	49.67201166	90.54300292	59.74854227	65.28790087	40.32434402	55.26603499	96.17827094	88.84201839	78.27781471	97.64552145	43.42201166	59.1654519	76.36661808	60.4409621	92.95031981	91.23542274
	85.40818134	98.12693389	108.8787585	90.40423121	98.2621836	68.40800425	67.15069949	73.59659996	48.66300691	98.59933392	89.52925337	75.31946052	79.24982875	90.43626143	92.82206271	41.01292722	79.17478307	62.54648486	46.11298034	56.59642288	53.3380556	88.3199093	97.99466188	80.59144484	91.34326948	53.05471932	68.21321055	71.61324597	63.96316628	108.5764225	62.26314857
	77.10559681	85.26818516	96.6752388	95.95196591	93.47278714	73.33816338	73.01213548	77.68520196	82.46694439	80.21675239	89.08890522	76.39603233	56.39235856	90.79720794	108.9676545	66.05687375	71.88915052	79.42401739	59.10161203	54.93570005	86.08947654	96.08743571	81.09845702	73.51212344	101.0837619	37.07661655	64.31805832	85.22006883	73.19326209	73.4570169	74.06266981
AMP3CPR2FUS2DOX1TMP2	AMP	CPR	FUS	DOX	TMP	AMP+CPR	AMP+FUS	AMP+DOX	AMP+TMP	CPR+FUS	CPR+DOX	CPR+TMP	FUS+DOX	FUS+TMP	DOX+TMP	AMP+CPR+FUS	AMP+CPR+DOX	AMP+CPR+TMP	AMP+FUS+DOX	AMP+FUS+TMP	AMP+DOX+TMP	CPR+FUS+DOX	CPR+FUS+TMP	CPR+DOX+TMP	FUS+DOX+TMP	AMP+CPR+FUS+DOX	AMP+CPR+FUS+TMP	AMP+CPR+DOX+TMP	AMP+FUS+DOX+TMP	CPR+FUS+DOX+TMP	AMP+CPR+FUS+DOX+TMP
	82.08819242	86.38437378	97.64552145	88.42597903	94.59267246	86.46137026	53.46209913	85.58673469	71.00947522	96.47172104	81.50576584	73.58261308	90.8961691	92.65686971	99.58475762	49.67201166	90.54300292	66.16253644	65.28790087	46.62900875	55.66690962	96.17827094	92.06996951	80.33196543	101.7538229	43.42201166	41.78206997	89.4861516	76.18440233	88.54856828	48.5058309
	85.40818134	98.12693389	108.8787585	90.40423121	92.51983377	68.40800425	67.15069949	73.59659996	52.82450859	98.59933392	89.52925337	78.94749274	79.24982875	89.52925337	96.14658104	41.01292722	79.17478307	72.46325483	46.11298034	56.10058438	57.44643173	88.3199093	107.3670784	68.49800411	88.3199093	53.05471932	48.37967062	49.6546839	55.17974146	96.78531781	50.78802904
	77.10559681	85.26818516	96.6752388	95.95196591	99.36083672	73.33816338	73.01213548	77.68520196	83.33635211	80.21675239	89.08890522	72.63041881	56.39235856	83.44966936	100.6004261	66.05687375	71.88915052	84.64046368	59.10161203	67.39721065	80.00362253	96.08743571	87.85819251	69.39750184	101.0837619	37.07661655	65.47726861	77.68520196	60.84042746	106.6678913	65.47726861
AMP3CPR2FUS2DOX1TMP3	AMP	CPR	FUS	DOX	TMP	AMP+CPR	AMP+FUS	AMP+DOX	AMP+TMP	CPR+FUS	CPR+DOX	CPR+TMP	FUS+DOX	FUS+TMP	DOX+TMP	AMP+CPR+FUS	AMP+CPR+DOX	AMP+CPR+TMP	AMP+FUS+DOX	AMP+FUS+TMP	AMP+DOX+TMP	CPR+FUS+DOX	CPR+FUS+TMP	CPR+DOX+TMP	FUS+DOX+TMP	AMP+CPR+FUS+DOX	AMP+CPR+FUS+TMP	AMP+CPR+DOX+TMP	AMP+FUS+DOX+TMP	CPR+FUS+DOX+TMP	AMP+CPR+FUS+DOX+TMP
	82.08819242	86.38437378	97.64552145	88.42597903	98.11649728	86.46137026	53.46209913	85.58673469	65.17857143	96.47172104	81.50576584	76.5171141	90.8961691	101.1669227	99.29110555	49.67201166	90.54300292	70.82725948	65.28790087	59.85787172	64.88702624	96.17827094	92.65686971	69.76776176	94.41757032	43.42201166	54.50072886	88.31997085	62.48177843	95.59137073	57.12463557
	85.40818134	98.12693389	108.8787585	90.40423121	99.16887042	68.40800425	67.15069949	73.59659996	53.62139189	98.59933392	89.52925337	76.52880459	79.24982875	94.06429364	99.16887042	41.01292722	79.17478307	66.51319285	46.11298034	51.6380379	56.31308659	88.3199093	109.1810946	76.96341262	73.50544441	53.05471932	54.04639632	62.12148043	60.84646715	99.20400595	72.88825925
	77.10559681	85.26818516	96.6752388	95.95196591	102.4598102	73.33816338	73.01213548	77.68520196	92.17533056	80.21675239	89.08890522	69.10360029	56.39235856	96.96914034	104.938989	66.05687375	71.88915052	96.81217171	59.10161203	81.30773411	90.43651512	96.08743571	87.27038942	77.92064658	87.85819251	37.07661655	62.47056693	79.71381996	68.84622351	108.4313005	62.57924289
AMP3CPR2FUS2DOX2TMP1	AMP	CPR	FUS	DOX	TMP	AMP+CPR	AMP+FUS	AMP+DOX	AMP+TMP	CPR+FUS	CPR+DOX	CPR+TMP	FUS+DOX	FUS+TMP	DOX+TMP	AMP+CPR+FUS	AMP+CPR+DOX	AMP+CPR+TMP	AMP+FUS+DOX	AMP+FUS+TMP	AMP+DOX+TMP	CPR+FUS+DOX	CPR+FUS+TMP	CPR+DOX+TMP	FUS+DOX+TMP	AMP+CPR+FUS+DOX	AMP+CPR+FUS+TMP	AMP+CPR+DOX+TMP	AMP+FUS+DOX+TMP	CPR+FUS+DOX+TMP	AMP+CPR+FUS+DOX+TMP
	82.08819242	86.38437378	97.64552145	92.53710799	99.29110555	86.46137026	53.46209913	80.92201166	64.30393586	96.47172104	80.91886563	81.50576584	107.6228249	99.11277196	100.7593659	49.67201166	75.96574344	59.74854227	50.41909621	40.32434402	59.34766764	99.11277196	88.84201839	74.16951329	96.76517114	42.65670554	59.1654519	80.44825073	53.44387755	94.71102043	40.50655977
	85.40818134	98.12693389	108.8787585	89.19531545	98.2621836	68.40800425	67.15069949	63.39649371	48.66300691	98.59933392	70.48208423	75.31946052	105.5530623	90.43626143	97.35549679	41.01292722	56.22454401	62.54648486	39.59624579	56.59642288	72.46325483	104.0413822	97.99466188	78.3428207	101.3203581	43.63378785	68.21321055	57.44643173	56.38392067	78.64515672	70.90490526
	77.10559681	85.26818516	96.6752388	100.9103235	93.47278714	73.33816338	73.01213548	78.55460967	82.46694439	80.21675239	72.86921381	76.39603233	90.79720794	90.79720794	104.0092969	66.05687375	70.44013766	79.42401739	49.82792972	54.93570005	90.14671255	96.96914034	81.09845702	64.05216752	96.6752388	63.73845318	64.31805832	74.78717624	54.1749683	94.91182954	50.11773229
AMP3CPR2FUS2DOX2TMP2	AMP	CPR	FUS	DOX	TMP	AMP+CPR	AMP+FUS	AMP+DOX	AMP+TMP	CPR+FUS	CPR+DOX	CPR+TMP	FUS+DOX	FUS+TMP	DOX+TMP	AMP+CPR+FUS	AMP+CPR+DOX	AMP+CPR+TMP	AMP+FUS+DOX	AMP+FUS+TMP	AMP+DOX+TMP	CPR+FUS+DOX	CPR+FUS+TMP	CPR+DOX+TMP	FUS+DOX+TMP	AMP+CPR+FUS+DOX	AMP+CPR+FUS+TMP	AMP+CPR+DOX+TMP	AMP+FUS+DOX+TMP	CPR+FUS+DOX+TMP	AMP+CPR+FUS+DOX+TMP
	82.08819242	86.38437378	97.64552145	92.53710799	94.59267246	86.46137026	53.46209913	80.92201166	71.00947522	96.47172104	80.91886563	73.58261308	107.6228249	92.65686971	103.6958866	49.67201166	75.96574344	66.16253644	50.41909621	46.62900875	66.63629738	99.11277196	92.06996951	83.55991655	99.11277196	42.65670554	41.78206997	69.36953353	70.82725948	102.3407231	48.08673469
	85.40818134	98.12693389	108.8787585	89.19531545	92.51983377	68.40800425	67.15069949	63.39649371	52.82450859	98.59933392	70.48208423	78.94749274	105.5530623	89.52925337	107.0268228	41.01292722	56.22454401	72.46325483	39.59624579	56.10058438	85.49672392	104.0413822	107.3670784	81.6685169	113.4137988	43.63378785	48.37967062	66.51319285	38.17956437	96.48298179	58.15477245
	77.10559681	85.26818516	96.6752388	100.9103235	99.36083672	73.33816338	73.01213548	78.55460967	83.33635211	80.21675239	72.86921381	72.63041881	90.79720794	83.44966936	89.13422429	66.05687375	70.44013766	84.64046368	49.82792972	67.39721065	99.71019743	96.96914034	87.85819251	84.09257899	102.2593681	63.73845318	65.47726861	70.44013766	69.13602608	70.22409993	46.64010143
AMP3CPR2FUS2DOX2TMP3	AMP	CPR	FUS	DOX	TMP	AMP+CPR	AMP+FUS	AMP+DOX	AMP+TMP	CPR+FUS	CPR+DOX	CPR+TMP	FUS+DOX	FUS+TMP	DOX+TMP	AMP+CPR+FUS	AMP+CPR+DOX	AMP+CPR+TMP	AMP+FUS+DOX	AMP+FUS+TMP	AMP+DOX+TMP	CPR+FUS+DOX	CPR+FUS+TMP	CPR+DOX+TMP	FUS+DOX+TMP	AMP+CPR+FUS+DOX	AMP+CPR+FUS+TMP	AMP+CPR+DOX+TMP	AMP+FUS+DOX+TMP	CPR+FUS+DOX+TMP	AMP+CPR+FUS+DOX+TMP
	82.08819242	86.38437378	97.64552145	92.53710799	98.11649728	86.46137026	53.46209913	80.92201166	65.17857143	96.47172104	80.91886563	76.5171141	107.6228249	101.1669227	103.9895386	49.67201166	75.96574344	70.82725948	50.41909621	59.85787172	70.2441691	99.11277196	92.65686971	96.17827094	110.850776	42.65670554	54.50072886	70.13483965	59.1654519	107.916275	50.41909621
	85.40818134	98.12693389	108.8787585	89.19531545	99.16887042	68.40800425	67.15069949	63.39649371	53.62139189	98.59933392	70.48208423	76.52880459	105.5530623	94.06429364	98.56441254	41.01292722	56.22454401	66.51319285	39.59624579	51.6380379	84.64671507	104.0413822	109.1810946	96.48298179	109.1810946	43.63378785	54.04639632	73.0299274	57.72976802	85.29654912	51.07136533
	77.10559681	85.26818516	96.6752388	100.9103235	102.4598102	73.33816338	73.01213548	78.55460967	92.17533056	80.21675239	72.86921381	69.10360029	90.79720794	96.96914034	91.92330041	66.05687375	70.44013766	96.81217171	49.82792972	81.30773411	85.79967397	96.96914034	87.27038942	72.86921381	105.1983835	63.73845318	62.47056693	88.69769969	53.88516573	86.68258633	51.56674515
AMP3CPR2FUS2DOX3TMP1	AMP	CPR	FUS	DOX	TMP	AMP+CPR	AMP+FUS	AMP+DOX	AMP+TMP	CPR+FUS	CPR+DOX	CPR+TMP	FUS+DOX	FUS+TMP	DOX+TMP	AMP+CPR+FUS	AMP+CPR+DOX	AMP+CPR+TMP	AMP+FUS+DOX	AMP+FUS+TMP	AMP+DOX+TMP	CPR+FUS+DOX	CPR+FUS+TMP	CPR+DOX+TMP	FUS+DOX+TMP	AMP+CPR+FUS+DOX	AMP+CPR+FUS+TMP	AMP+CPR+DOX+TMP	AMP+FUS+DOX+TMP	CPR+FUS+DOX+TMP	AMP+CPR+FUS+DOX+TMP
	82.08819242	86.38437378	97.64552145	103.4022345	99.29110555	86.46137026	53.46209913	86.46137026	64.30393586	96.47172104	79.04812123	81.50576584	98.52587175	99.11277196	106.6324073	49.67201166	64.30393586	59.74854227	49.38046647	40.32434402	72.75874636	90.3092689	88.84201839	98.81932186	117.3066783	55.66690962	59.1654519	76.25728863	71.99344023	114.3721773	46.92055394
	85.40818134	98.12693389	108.8787585	94.93766528	98.2621836	68.40800425	67.15069949	63.02461484	48.66300691	98.59933392	75.62179654	75.31946052	100.715686	90.43626143	100.6800151	41.01292722	51.77970604	62.54648486	48.94634319	56.59642288	92.86346733	110.0881026	97.99466188	89.22691735	103.7390462	60.27979458	68.21321055	70.76323712	69.34655569	104.0413822	74.7299451
	77.10559681	85.26818516	96.6752388	112.6864226	93.47278714	73.33816338	73.01213548	83.33635211	82.46694439	80.21675239	75.5143277	76.39603233	95.49963262	90.79720794	108.6577571	66.05687375	74.49737366	79.42401739	62.8509328	54.93570005	88.98750226	97.85084497	81.09845702	85.50698016	92.26671565	63.15884803	64.31805832	87.5384894	72.0340518	93.73622337	32.14997283
AMP3CPR2FUS2DOX3TMP2	AMP	CPR	FUS	DOX	TMP	AMP+CPR	AMP+FUS	AMP+DOX	AMP+TMP	CPR+FUS	CPR+DOX	CPR+TMP	FUS+DOX	FUS+TMP	DOX+TMP	AMP+CPR+FUS	AMP+CPR+DOX	AMP+CPR+TMP	AMP+FUS+DOX	AMP+FUS+TMP	AMP+DOX+TMP	CPR+FUS+DOX	CPR+FUS+TMP	CPR+DOX+TMP	FUS+DOX+TMP	AMP+CPR+FUS+DOX	AMP+CPR+FUS+TMP	AMP+CPR+DOX+TMP	AMP+FUS+DOX+TMP	CPR+FUS+DOX+TMP	AMP+CPR+FUS+DOX+TMP
	82.08819242	86.38437378	97.64552145	103.4022345	94.59267246	86.46137026	53.46209913	86.46137026	71.00947522	96.47172104	79.04812123	73.58261308	98.52587175	92.65686971	102.5212783	49.67201166	64.30393586	66.16253644	49.38046647	46.62900875	86.8622449	90.3092689	92.06996951	86.78786767	117.8935785	55.66690962	41.78206997	69.55174927	62.08090379	101.7538229	45.1712828
	85.40818134	98.12693389	108.8787585	94.93766528	92.51983377	68.40800425	67.15069949	63.02461484	52.82450859	98.59933392	75.62179654	78.94749274	100.715686	89.52925337	92.21760484	41.01292722	51.77970604	72.46325483	48.94634319	56.10058438	83.51336993	110.0881026	107.3670784	78.64515672	106.4600704	60.27979458	48.37967062	95.60828759	65.94652028	91.04093346	56.31308659
	77.10559681	85.26818516	96.6752388	112.6864226	99.36083672	73.33816338	73.01213548	83.33635211	83.33635211	80.21675239	75.5143277	72.63041881	95.49963262	83.44966936	101.5301181	66.05687375	74.49737366	84.64046368	62.8509328	67.39721065	79.2791161	97.85084497	87.85819251	89.97060985	87.85819251	63.15884803	65.47726861	75.65658395	56.76507879	102.8471712	61.89096178
AMP3CPR2FUS2DOX3TMP3	AMP	CPR	FUS	DOX	TMP	AMP+CPR	AMP+FUS	AMP+DOX	AMP+TMP	CPR+FUS	CPR+DOX	CPR+TMP	FUS+DOX	FUS+TMP	DOX+TMP	AMP+CPR+FUS	AMP+CPR+DOX	AMP+CPR+TMP	AMP+FUS+DOX	AMP+FUS+TMP	AMP+DOX+TMP	CPR+FUS+DOX	CPR+FUS+TMP	CPR+DOX+TMP	FUS+DOX+TMP	AMP+CPR+FUS+DOX	AMP+CPR+FUS+TMP	AMP+CPR+DOX+TMP	AMP+FUS+DOX+TMP	CPR+FUS+DOX+TMP	AMP+CPR+FUS+DOX+TMP
	82.08819242	86.38437378	97.64552145	103.4022345	98.11649728	86.46137026	53.46209913	86.46137026	65.17857143	96.47172104	79.04812123	76.5171141	98.52587175	101.1669227	91.65615178	49.67201166	64.30393586	70.82725948	49.38046647	59.85787172	90.25145773	90.3092689	92.65686971	86.09092368	115.2525276	55.66690962	54.50072886	92.00072886	55.66690962	109.0900754	50.7106414
	85.40818134	98.12693389	108.8787585	94.93766528	99.16887042	68.40800425	67.15069949	63.02461484	53.62139189	98.59933392	75.62179654	76.52880459	100.715686	94.06429364	94.03097847	41.01292722	51.77970604	66.51319285	48.94634319	51.6380379	80.68000708	110.0881026	109.1810946	100.8479581	107.0647424	60.27979458	54.04639632	79.17478307	54.61306889	90.73859744	59.42978573
	77.10559681	85.26818516	96.6752388	112.6864226	102.4598102	73.33816338	73.01213548	83.33635211	92.17533056	80.21675239	75.5143277	69.10360029	95.49963262	96.96914034	101.5301181	66.05687375	74.49737366	96.81217171	62.8509328	81.30773411	83.91595725	97.85084497	87.27038942	96.43644379	94.03012491	63.15884803	62.47056693	80.87303025	59.86234378	101.671565	65.36859265
AMP3CPR2FUS3DOX1TMP1	AMP	CPR	FUS	DOX	TMP	AMP+CPR	AMP+FUS	AMP+DOX	AMP+TMP	CPR+FUS	CPR+DOX	CPR+TMP	FUS+DOX	FUS+TMP	DOX+TMP	AMP+CPR+FUS	AMP+CPR+DOX	AMP+CPR+TMP	AMP+FUS+DOX	AMP+FUS+TMP	AMP+DOX+TMP	CPR+FUS+DOX	CPR+FUS+TMP	CPR+DOX+TMP	FUS+DOX+TMP	AMP+CPR+FUS+DOX	AMP+CPR+FUS+TMP	AMP+CPR+DOX+TMP	AMP+FUS+DOX+TMP	CPR+FUS+DOX+TMP	AMP+CPR+FUS+DOX+TMP
	82.08819242	86.38437378	95.90014577	88.42597903	99.29110555	86.46137026	67.80247813	85.58673469	64.30393586	92.06996951	81.50576584	81.50576584	77.53279883	72.8680758	96.94188901	66.45408163	90.54300292	59.74854227	92.11005831	50.81997085	55.26603499	92.65686971	90.8961691	78.27781471	76.94970845	69.95262391	72.8680758	76.36661808	67.03717201	94.41757032	90.65233236
	85.40818134	98.12693389	92.29679476	90.40423121	98.2621836	68.40800425	62.54648486	73.59659996	48.66300691	104.9483903	89.52925337	75.31946052	78.69665309	54.89640517	92.82206271	63.96316628	79.17478307	62.54648486	72.46325483	60.84646715	53.3380556	97.38998984	107.0647424	80.59144484	83.51336993	54.80786258	78.69665309	71.61324597	42.57127678	100.111014	46.39631663
	77.10559681	85.26818516	84.78536497	95.95196591	93.47278714	73.33816338	71.56312262	77.68520196	82.46694439	79.04114622	89.08890522	76.39603233	94.63865242	89.42220612	108.9676545	47.2559319	71.88915052	79.42401739	73.48306466	82.75674697	86.08947654	92.26671565	86.68258633	73.51212344	93.47944213	72.61365695	49.86415504	85.22006883	58.99293606	97.26304188	66.81760551
AMP3CPR2FUS3DOX1TMP2	AMP	CPR	FUS	DOX	TMP	AMP+CPR	AMP+FUS	AMP+DOX	AMP+TMP	CPR+FUS	CPR+DOX	CPR+TMP	FUS+DOX	FUS+TMP	DOX+TMP	AMP+CPR+FUS	AMP+CPR+DOX	AMP+CPR+TMP	AMP+FUS+DOX	AMP+FUS+TMP	AMP+DOX+TMP	CPR+FUS+DOX	CPR+FUS+TMP	CPR+DOX+TMP	FUS+DOX+TMP	AMP+CPR+FUS+DOX	AMP+CPR+FUS+TMP	AMP+CPR+DOX+TMP	AMP+FUS+DOX+TMP	CPR+FUS+DOX+TMP	AMP+CPR+FUS+DOX+TMP
	82.08819242	86.38437378	95.90014577	88.42597903	94.59267246	86.46137026	67.80247813	85.58673469	71.00947522	92.06996951	81.50576584	73.58261308	77.53279883	57.99927114	99.58475762	66.45408163	90.54300292	66.16253644	92.11005831	61.02405248	55.66690962	92.65686971	91.4830693	80.33196543	87.44533528	69.95262391	58.58236152	89.4861516	62.77332362	95.00447053	93.27623907
	85.40818134	98.12693389	92.29679476	90.40423121	92.51983377	68.40800425	62.54648486	73.59659996	52.82450859	104.9483903	89.52925337	78.94749274	78.69665309	56.59642288	96.14658104	63.96316628	79.17478307	72.46325483	72.46325483	45.40463963	57.44643173	97.38998984	107.0647424	68.49800411	71.0465734	54.80786258	86.91340535	49.6546839	67.92987427	91.04093346	66.79652913
	77.10559681	85.26818516	84.78536497	95.95196591	99.36083672	73.33816338	71.56312262	77.68520196	83.33635211	79.04114622	89.08890522	72.63041881	94.63865242	90.87121898	100.6004261	47.2559319	71.88915052	84.64046368	73.48306466	83.62615468	80.00362253	92.26671565	98.7325496	69.39750184	90.87121898	72.61365695	70.29523637	77.68520196	72.0340518	93.73622337	69.42582865
AMP3CPR2FUS3DOX1TMP3	AMP	CPR	FUS	DOX	TMP	AMP+CPR	AMP+FUS	AMP+DOX	AMP+TMP	CPR+FUS	CPR+DOX	CPR+TMP	FUS+DOX	FUS+TMP	DOX+TMP	AMP+CPR+FUS	AMP+CPR+DOX	AMP+CPR+TMP	AMP+FUS+DOX	AMP+FUS+TMP	AMP+DOX+TMP	CPR+FUS+DOX	CPR+FUS+TMP	CPR+DOX+TMP	FUS+DOX+TMP	AMP+CPR+FUS+DOX	AMP+CPR+FUS+TMP	AMP+CPR+DOX+TMP	AMP+FUS+DOX+TMP	CPR+FUS+DOX+TMP	AMP+CPR+FUS+DOX+TMP
	82.08819242	86.38437378	95.90014577	88.42597903	98.11649728	86.46137026	67.80247813	85.58673469	65.17857143	92.06996951	81.50576584	76.5171141	77.53279883	66.74562682	99.29110555	66.45408163	90.54300292	70.82725948	92.11005831	66.56341108	64.88702624	92.65686971	94.12412022	69.76776176	83.65524781	69.95262391	76.07507289	88.31997085	61.02405248	79.45161512	90.94387755
	85.40818134	98.12693389	92.29679476	90.40423121	99.16887042	68.40800425	62.54648486	73.59659996	53.62139189	104.9483903	89.52925337	76.52880459	78.69665309	65.09651142	99.16887042	63.96316628	79.17478307	66.51319285	72.46325483	59.28811758	56.31308659	97.38998984	100.715686	76.96341262	73.59659996	54.80786258	67.3632017	62.12148043	73.31326368	82.57552495	77.70497609
	77.10559681	85.26818516	84.78536497	95.95196591	102.4598102	73.33816338	71.56312262	77.68520196	92.17533056	79.04114622	89.08890522	69.10360029	94.63865242	81.88733925	104.938989	47.2559319	71.88915052	96.81217171	73.48306466	79.56891867	90.43651512	92.26671565	96.38133725	77.92064658	75.07697881	72.61365695	84.4955624	79.71381996	71.88915052	95.79353417	68.9911248
AMP3CPR2FUS3DOX2TMP1	AMP	CPR	FUS	DOX	TMP	AMP+CPR	AMP+FUS	AMP+DOX	AMP+TMP	CPR+FUS	CPR+DOX	CPR+TMP	FUS+DOX	FUS+TMP	DOX+TMP	AMP+CPR+FUS	AMP+CPR+DOX	AMP+CPR+TMP	AMP+FUS+DOX	AMP+FUS+TMP	AMP+DOX+TMP	CPR+FUS+DOX	CPR+FUS+TMP	CPR+DOX+TMP	FUS+DOX+TMP	AMP+CPR+FUS+DOX	AMP+CPR+FUS+TMP	AMP+CPR+DOX+TMP	AMP+FUS+DOX+TMP	CPR+FUS+DOX+TMP	AMP+CPR+FUS+DOX+TMP
	82.08819242	86.38437378	95.90014577	92.53710799	99.29110555	86.46137026	67.80247813	80.92201166	64.30393586	92.06996951	80.91886563	81.50576584	91.52696793	72.8680758	100.7593659	66.45408163	75.96574344	59.74854227	80.73979592	50.81997085	59.34766764	98.23242165	90.8961691	74.16951329	64.59548105	66.74562682	72.8680758	80.44825073	60.33163265	92.65686971	79.57361516
	85.40818134	98.12693389	92.29679476	89.19531545	98.2621836	68.40800425	62.54648486	63.39649371	48.66300691	104.9483903	70.48208423	75.31946052	68.49654684	54.89640517	97.35549679	63.96316628	56.22454401	62.54648486	82.38002479	60.84646715	72.46325483	82.57552495	107.0647424	78.3428207	73.0299274	47.38799362	78.69665309	57.44643173	81.53001594	87.71523726	64.10483443
	77.10559681	85.26818516	84.78536497	100.9103235	93.47278714	73.33816338	71.56312262	78.55460967	82.46694439	79.04114622	72.86921381	76.39603233	73.62796595	89.42220612	104.0092969	47.2559319	70.44013766	79.42401739	72.0340518	82.75674697	90.14671255	94.61792799	86.68258633	64.05216752	101.8837167	67.10740808	49.86415504	74.78717624	77.2504981	84.62527553	70.29523637
AMP3CPR2FUS3DOX2TMP2	AMP	CPR	FUS	DOX	TMP	AMP+CPR	AMP+FUS	AMP+DOX	AMP+TMP	CPR+FUS	CPR+DOX	CPR+TMP	FUS+DOX	FUS+TMP	DOX+TMP	AMP+CPR+FUS	AMP+CPR+DOX	AMP+CPR+TMP	AMP+FUS+DOX	AMP+FUS+TMP	AMP+DOX+TMP	CPR+FUS+DOX	CPR+FUS+TMP	CPR+DOX+TMP	FUS+DOX+TMP	AMP+CPR+FUS+DOX	AMP+CPR+FUS+TMP	AMP+CPR+DOX+TMP	AMP+FUS+DOX+TMP	CPR+FUS+DOX+TMP	AMP+CPR+FUS+DOX+TMP
	82.08819242	86.38437378	95.90014577	92.53710799	94.59267246	86.46137026	67.80247813	80.92201166	71.00947522	92.06996951	80.91886563	73.58261308	91.52696793	57.99927114	103.6958866	66.45408163	75.96574344	66.16253644	80.73979592	61.02405248	66.63629738	98.23242165	91.4830693	83.55991655	81.61443149	66.74562682	58.58236152	69.36953353	66.16253644	98.23242165	74.90889213
	85.40818134	98.12693389	92.29679476	89.19531545	92.51983377	68.40800425	62.54648486	63.39649371	52.82450859	104.9483903	70.48208423	78.94749274	68.49654684	56.59642288	107.0268228	63.96316628	56.22454401	72.46325483	82.38002479	45.40463963	85.49672392	82.57552495	107.0647424	81.6685169	98.24685674	47.38799362	86.91340535	66.51319285	70.76323712	93.76195763	72.46325483
	77.10559681	85.26818516	84.78536497	100.9103235	99.36083672	73.33816338	71.56312262	78.55460967	83.33635211	79.04114622	72.86921381	72.63041881	73.62796595	90.87121898	89.13422429	47.2559319	70.44013766	84.64046368	72.0340518	83.62615468	99.71019743	94.61792799	98.7325496	84.09257899	99.85509871	67.10740808	70.29523637	70.44013766	74.64227495	61.99485672	82.17714182
AMP3CPR2FUS3DOX2TMP3	AMP	CPR	FUS	DOX	TMP	AMP+CPR	AMP+FUS	AMP+DOX	AMP+TMP	CPR+FUS	CPR+DOX	CPR+TMP	FUS+DOX	FUS+TMP	DOX+TMP	AMP+CPR+FUS	AMP+CPR+DOX	AMP+CPR+TMP	AMP+FUS+DOX	AMP+FUS+TMP	AMP+DOX+TMP	CPR+FUS+DOX	CPR+FUS+TMP	CPR+DOX+TMP	FUS+DOX+TMP	AMP+CPR+FUS+DOX	AMP+CPR+FUS+TMP	AMP+CPR+DOX+TMP	AMP+FUS+DOX+TMP	CPR+FUS+DOX+TMP	AMP+CPR+FUS+DOX+TMP
	82.08819242	86.38437378	95.90014577	92.53710799	98.11649728	86.46137026	67.80247813	80.92201166	65.17857143	92.06996951	80.91886563	76.5171141	91.52696793	66.74562682	103.9895386	66.45408163	75.96574344	70.82725948	80.73979592	66.56341108	70.2441691	98.23242165	94.12412022	96.17827094	108.4365889	66.74562682	76.07507289	70.13483965	77.24125364	108.5031752	80.15670554
	85.40818134	98.12693389	92.29679476	89.19531545	99.16887042	68.40800425	62.54648486	63.39649371	53.62139189	104.9483903	70.48208423	76.52880459	68.49654684	65.09651142	98.56441254	63.96316628	56.22454401	66.51319285	82.38002479	59.28811758	84.64671507	82.57552495	100.715686	96.48298179	88.61342306	47.38799362	67.3632017	73.0299274	72.74659111	99.20400595	75.29661767
	77.10559681	85.26818516	84.78536497	100.9103235	102.4598102	73.33816338	71.56312262	78.55460967	92.17533056	79.04114622	72.86921381	69.10360029	73.62796595	81.88733925	91.92330041	47.2559319	70.44013766	96.81217171	72.0340518	79.56891867	85.79967397	94.61792799	96.38133725	72.86921381	99.56529614	67.10740808	84.4955624	88.69769969	76.96069553	85.21307862	82.75674697
AMP3CPR2FUS3DOX3TMP1	AMP	CPR	FUS	DOX	TMP	AMP+CPR	AMP+FUS	AMP+DOX	AMP+TMP	CPR+FUS	CPR+DOX	CPR+TMP	FUS+DOX	FUS+TMP	DOX+TMP	AMP+CPR+FUS	AMP+CPR+DOX	AMP+CPR+TMP	AMP+FUS+DOX	AMP+FUS+TMP	AMP+DOX+TMP	CPR+FUS+DOX	CPR+FUS+TMP	CPR+DOX+TMP	FUS+DOX+TMP	AMP+CPR+FUS+DOX	AMP+CPR+FUS+TMP	AMP+CPR+DOX+TMP	AMP+FUS+DOX+TMP	CPR+FUS+DOX+TMP	AMP+CPR+FUS+DOX+TMP
	82.08819242	86.38437378	95.90014577	103.4022345	99.29110555	86.46137026	67.80247813	86.46137026	64.30393586	92.06996951	79.04812123	81.50576584	48.66982507	72.8680758	106.6324073	66.45408163	64.30393586	59.74854227	54.50072886	50.81997085	72.75874636	91.77651941	90.8961691	98.81932186	102.3141399	75.49198251	72.8680758	76.25728863	95.90014577	112.3180265	76.07507289
	85.40818134	98.12693389	92.29679476	94.93766528	98.2621836	68.40800425	62.54648486	63.02461484	48.66300691	104.9483903	75.62179654	75.31946052	61.41313972	54.89640517	100.6800151	63.96316628	51.77970604	62.54648486	72.74659111	60.84646715	92.86346733	106.1577344	107.0647424	89.22691735	85.78006021	73.31326368	78.69665309	70.76323712	91.16344962	108.5764225	83.23003365
	77.10559681	85.26818516	84.78536497	112.6864226	93.47278714	73.33816338	71.56312262	83.33635211	82.46694439	79.04114622	75.5143277	76.39603233	68.41151965	89.42220612	108.6577571	47.2559319	74.49737366	79.42401739	79.2791161	82.75674697	88.98750226	99.02645114	86.68258633	85.50698016	101.5939141	69.42582865	49.86415504	87.5384894	86.81398297	89.91550331	82.75674697
AMP3CPR2FUS3DOX3TMP2	AMP	CPR	FUS	DOX	TMP	AMP+CPR	AMP+FUS	AMP+DOX	AMP+TMP	CPR+FUS	CPR+DOX	CPR+TMP	FUS+DOX	FUS+TMP	DOX+TMP	AMP+CPR+FUS	AMP+CPR+DOX	AMP+CPR+TMP	AMP+FUS+DOX	AMP+FUS+TMP	AMP+DOX+TMP	CPR+FUS+DOX	CPR+FUS+TMP	CPR+DOX+TMP	FUS+DOX+TMP	AMP+CPR+FUS+DOX	AMP+CPR+FUS+TMP	AMP+CPR+DOX+TMP	AMP+FUS+DOX+TMP	CPR+FUS+DOX+TMP	AMP+CPR+FUS+DOX+TMP
	82.08819242	86.38437378	95.90014577	103.4022345	94.59267246	86.46137026	67.80247813	86.46137026	71.00947522	92.06996951	79.04812123	73.58261308	48.66982507	57.99927114	102.5212783	66.45408163	64.30393586	66.16253644	54.50072886	61.02405248	86.8622449	91.77651941	91.4830693	86.78786767	98.52405248	75.49198251	58.58236152	69.55174927	85.98760933	103.8079736	66.92784257
	85.40818134	98.12693389	92.29679476	94.93766528	92.51983377	68.40800425	62.54648486	63.02461484	52.82450859	104.9483903	75.62179654	78.94749274	61.41313972	56.59642288	92.21760484	63.96316628	51.77970604	72.46325483	72.74659111	45.40463963	83.51336993	106.1577344	107.0647424	78.64515672	88.89675934	73.31326368	86.91340535	95.60828759	71.0465734	88.01757328	82.09668851
	77.10559681	85.26818516	84.78536497	112.6864226	99.36083672	73.33816338	71.56312262	83.33635211	83.33635211	79.04114622	75.5143277	72.63041881	68.41151965	90.87121898	101.5301181	47.2559319	74.49737366	84.64046368	79.2791161	83.62615468	79.2791161	99.02645114	98.7325496	89.97060985	98.69588843	69.42582865	70.29523637	75.65658395	82.75674697	98.14474651	72.90345952
AMP3CPR2FUS3DOX3TMP3	AMP	CPR	FUS	DOX	TMP	AMP+CPR	AMP+FUS	AMP+DOX	AMP+TMP	CPR+FUS	CPR+DOX	CPR+TMP	FUS+DOX	FUS+TMP	DOX+TMP	AMP+CPR+FUS	AMP+CPR+DOX	AMP+CPR+TMP	AMP+FUS+DOX	AMP+FUS+TMP	AMP+DOX+TMP	CPR+FUS+DOX	CPR+FUS+TMP	CPR+DOX+TMP	FUS+DOX+TMP	AMP+CPR+FUS+DOX	AMP+CPR+FUS+TMP	AMP+CPR+DOX+TMP	AMP+FUS+DOX+TMP	CPR+FUS+DOX+TMP	AMP+CPR+FUS+DOX+TMP
	82.08819242	86.38437378	95.90014577	103.4022345	98.11649728	86.46137026	67.80247813	86.46137026	65.17857143	92.06996951	79.04812123	76.5171141	48.66982507	66.74562682	91.65615178	66.45408163	64.30393586	70.82725948	54.50072886	66.56341108	90.25145773	91.77651941	94.12412022	86.09092368	96.48323615	75.49198251	76.07507289	92.00072886	82.19752187	109.6769756	67.91180758
	85.40818134	98.12693389	92.29679476	94.93766528	99.16887042	68.40800425	62.54648486	63.02461484	53.62139189	104.9483903	75.62179654	76.52880459	61.41313972	65.09651142	94.03097847	63.96316628	51.77970604	66.51319285	72.74659111	59.28811758	80.68000708	106.1577344	100.715686	100.8479581	85.78006021	73.31326368	67.3632017	79.17478307	85.21338764	99.20400595	68.49654684
	77.10559681	85.26818516	84.78536497	112.6864226	102.4598102	73.33816338	71.56312262	83.33635211	92.17533056	79.04114622	75.5143277	69.10360029	68.41151965	81.88733925	101.5301181	47.2559319	74.49737366	96.81217171	79.2791161	79.56891867	83.91595725	99.02645114	96.38133725	96.43644379	94.34884985	69.42582865	84.4955624	80.87303025	90.29161384	97.26304188	78.11990581
AMP3CPR3FUS1DOX1TMP1	AMP	CPR	FUS	DOX	TMP	AMP+CPR	AMP+FUS	AMP+DOX	AMP+TMP	CPR+FUS	CPR+DOX	CPR+TMP	FUS+DOX	FUS+TMP	DOX+TMP	AMP+CPR+FUS	AMP+CPR+DOX	AMP+CPR+TMP	AMP+FUS+DOX	AMP+FUS+TMP	AMP+DOX+TMP	CPR+FUS+DOX	CPR+FUS+TMP	CPR+DOX+TMP	FUS+DOX+TMP	AMP+CPR+FUS+DOX	AMP+CPR+FUS+TMP	AMP+CPR+DOX+TMP	AMP+FUS+DOX+TMP	CPR+FUS+DOX+TMP	AMP+CPR+FUS+DOX+TMP
	82.08819242	86.75291545	72.56876735	88.42597903	99.29110555	73.34183673	36.24271137	85.58673469	64.30393586	76.65816327	96.6654519	65.47011662	74.33067976	84.90215421	96.94188901	54.62827988	92.00072886	70.42638484	44.40597668	42.83892128	55.26603499	96.77478134	56.35932945	78.88119534	75.7989401	37.59110787	49.54446064	44.29664723	43.25801749	79.28206997	57.54373178
	85.40818134	84.36337879	80.73290518	90.40423121	98.2621836	70.95803081	44.12962635	73.59659996	48.66300691	68.21321055	80.59146449	61.8912697	65.01700038	76.80392898	92.82206271	47.31715955	77.27997167	49.6546839	39.31290951	49.22967948	53.3380556	97.39684788	81.81335222	67.5579954	98.56441254	46.11298034	48.66300691	68.21321055	46.4671507	57.30476359	43.98795821
	77.10559681	88.11809455	93.47278714	95.95196591	93.47278714	80.2934251	62.28944032	77.68520196	82.46694439	84.78536497	93.33454084	90.14671255	63.10284718	101.2202208	108.9676545	40.55424742	87.82829198	77.68520196	55.04437602	58.41333092	86.08947654	83.33635211	119.8514762	104.9266437	86.34514817	47.50950915	51.85654773	81.01793153	42.58286542	87.68339069	46.82122804
AMP3CPR3FUS1DOX1TMP2	AMP	CPR	FUS	DOX	TMP	AMP+CPR	AMP+FUS	AMP+DOX	AMP+TMP	CPR+FUS	CPR+DOX	CPR+TMP	FUS+DOX	FUS+TMP	DOX+TMP	AMP+CPR+FUS	AMP+CPR+DOX	AMP+CPR+TMP	AMP+FUS+DOX	AMP+FUS+TMP	AMP+DOX+TMP	CPR+FUS+DOX	CPR+FUS+TMP	CPR+DOX+TMP	FUS+DOX+TMP	AMP+CPR+FUS+DOX	AMP+CPR+FUS+TMP	AMP+CPR+DOX+TMP	AMP+FUS+DOX+TMP	CPR+FUS+DOX+TMP	AMP+CPR+FUS+DOX+TMP
	82.08819242	86.75291545	72.56876735	88.42597903	94.59267246	73.34183673	36.24271137	85.58673469	71.00947522	76.65816327	96.6654519	81.79664723	74.33067976	94.59267246	99.58475762	54.62827988	92.00072886	65.47011662	44.40597668	46.04591837	55.66690962	96.77478134	61.20626822	71.30102041	69.92589874	37.59110787	38.17419825	57.12463557	57.5255102	78.99052478	56.9606414
	85.40818134	84.36337879	80.73290518	90.40423121	92.51983377	70.95803081	44.12962635	73.59659996	52.82450859	68.21321055	80.59146449	66.70798654	65.01700038	102.7956177	96.14658104	47.31715955	77.27997167	54.04639632	39.31290951	54.11723039	57.44643173	97.39684788	74.44660882	63.87462369	77.40838685	46.11298034	46.75048698	80.11333451	49.01717726	61.97981229	43.13794935
	77.10559681	88.11809455	93.47278714	95.95196591	99.36083672	80.2934251	62.28944032	77.68520196	83.33635211	84.78536497	93.33454084	94.49375113	63.10284718	102.1499128	100.6004261	40.55424742	87.82829198	81.88733925	55.04437602	66.81760551	80.00362253	83.33635211	100.1449013	106.9552617	73.94925431	47.50950915	55.91378373	80.72812896	47.50950915	84.20575983	42.58286542
AMP3CPR3FUS1DOX1TMP3	AMP	CPR	FUS	DOX	TMP	AMP+CPR	AMP+FUS	AMP+DOX	AMP+TMP	CPR+FUS	CPR+DOX	CPR+TMP	FUS+DOX	FUS+TMP	DOX+TMP	AMP+CPR+FUS	AMP+CPR+DOX	AMP+CPR+TMP	AMP+FUS+DOX	AMP+FUS+TMP	AMP+DOX+TMP	CPR+FUS+DOX	CPR+FUS+TMP	CPR+DOX+TMP	FUS+DOX+TMP	AMP+CPR+FUS+DOX	AMP+CPR+FUS+TMP	AMP+CPR+DOX+TMP	AMP+FUS+DOX+TMP	CPR+FUS+DOX+TMP	AMP+CPR+FUS+DOX+TMP
	82.08819242	86.75291545	72.56876735	88.42597903	98.11649728	73.34183673	36.24271137	85.58673469	65.17857143	76.65816327	96.6654519	76.25728863	74.33067976	73.74337562	99.29110555	54.62827988	92.00072886	45.35349854	44.40597668	40.03279883	64.88702624	96.77478134	64.41326531	82.96282799	95.76728074	37.59110787	49.96355685	55.66690962	41.78206997	84.23833819	88.31997085

	85.40818134	84.36337879	80.73290518	90.40423121	99.16887042	70.95803081	44.12962635	73.59659996	53.62139189	68.21321055	80.59146449	[illegible]
	77.10559681	88.11809455	93.47278714	95.95196591	102.4598102	80.2934251	62.28944032	77.68520196	92.17533056	84.78536497	93.33454084	[illegible]
[illegible]S1DOX2TMP1	AMP	CPR	FUS	DOX	TMP	AMP+CPR	AMP+FUS	AMP+DOX	AMP+TMP	CPR+FUS	CPR+DOX	[illegible]
	82.08819242	86.75291545	72.56876735	92.53710799	99.29110555	73.34183673	36.24271137	80.92201166	64.30393586	76.65816327	79.17274052	[illegible]
	85.40818134	84.36337879	80.73290518	89.19531545	98.2621836	70.95803081	44.12962635	63.39649371	48.66300691	68.21321055	67.5579954	[illegible]
	77.10559681	88.11809455	93.47278714	100.9103235	93.47278714	80.2934251	62.28944032	78.55460967	82.46694439	84.78536497	91.30592284	[illegible]
[illegible]S1DOX2TMP2	AMP	CPR	FUS	DOX	TMP	AMP+CPR	AMP+FUS	AMP+DOX	AMP+TMP	CPR+FUS	CPR+DOX	[illegible]
	82.08819242	86.75291545	72.56876735	92.53710799	94.59267246	73.34183673	36.24271137	80.92201166	71.00947522	76.65816327	79.17274052	[illegible]
	85.40818134	84.36337879	80.73290518	89.19531545	92.51983377	70.95803081	44.12962635	63.39649371	52.82450859	68.21321055	67.5579954	[illegible]
	77.10559681	88.11809455	93.47278714	100.9103235	99.36083672	80.2934251	62.28944032	78.55460967	83.33635211	84.78536497	91.30592284	[illegible]
[illegible]S1DOX2TMP3	AMP	CPR	FUS	DOX	TMP	AMP+CPR	AMP+FUS	AMP+DOX	AMP+TMP	CPR+FUS	CPR+DOX	[illegible]
	82.08819242	86.75291545	72.56876735	92.53710799	98.11649728	73.34183673	36.24271137	80.92201166	65.17857143	76.65816327	79.17274052	[illegible]
	85.40818134	84.36337879	80.73290518	89.19531545	99.16887042	70.95803081	44.12962635	63.39649371	53.62139189	68.21321055	67.5579954	[illegible]
	77.10559681	88.11809455	93.47278714	100.9103235	102.4598102	80.2934251	62.28944032	78.55460967	92.17533056	84.78536497	91.30592284	[illegible]
[illegible]S1DOX3TMP1	AMP	CPR	FUS	DOX	TMP	AMP+CPR	AMP+FUS	AMP+DOX	AMP+TMP	CPR+FUS	CPR+DOX	[illegible]
	82.08819242	86.75291545	72.56876735	103.4022345	99.29110555	73.34183673	36.24271137	86.46137026	64.30393586	76.65816327	68.96865889	[illegible]

[illegible]

AMP1CPR1STR3DOX2TMP3	AMP	CPR	STR	DOX	TMP	AMP+CPR	AMP+STR	AMP+DOX	AMP+TMP	CPR+STR	CPR+DOX	CPR+TMP	STR+DOX
	88.89160156	101.7822266	93.55176933	100.6103516	88.11035156	83.81347656	34.79003906	88.11035156	83.03222656	70.72753906	96.50878906	89.08691406	95.64875491
	94.15696955	102.4758604	85.80206563	102.2777915	91.97821243	93.1666254	29.38846249	83.06511513	86.23421639	52.36444665	99.90096559	82.47090864	96.67930448
	93.03614154	101.4985518	94.53113607	98.07329052	96.6628888	84.57373127	40.64979222	86.99156278	89.00642236	48.1047727	95.05100113	92.02871175	99.19789996
AMP1CPR1STR3DOX3TMP1	AMP	CPR	STR	DOX	TMP	AMP+CPR	AMP+STR	AMP+DOX	AMP+TMP	CPR+STR	CPR+DOX	CPR+TMP	STR+DOX
	88.89160156	101.7822266	93.55176933	99.04785156	83.22753906	83.81347656	34.79003906	91.23535156	88.50097656	70.72753906	102.1728516	90.06347656	91.03538663
	94.15696955	102.4758604	85.80206563	101.2874474	87.62069819	93.1666254	29.38846249	86.63035405	84.45159693	52.36444665	102.2777915	81.4805645	94.37835011
	93.03614154	101.4985518	94.53113607	100.0881501	85.17818914	84.57373127	40.64979222	86.79007682	80.54401209	48.1047727	97.46883264	78.93212442	96.86451801
AMP1CPR1STR3DOX3TMP2	AMP	CPR	STR	DOX	TMP	AMP+CPR	AMP+STR	AMP+DOX	AMP+TMP	CPR+STR	CPR+DOX	CPR+TMP	STR+DOX
	88.89160156	101.7822266	93.55176933	99.04785156	91.04003906	83.81347656	34.79003906	91.23535156	91.82128906	70.72753906	102.1728516	88.89160156	91.03538663
	94.15696955	102.4758604	85.80206563	101.2874474	93.36469423	93.1666254	29.38846249	86.63035405	87.42262936	52.36444665	102.2777915	85.83807873	94.37835011
	93.03614154	101.4985518	94.53113607	100.0881501	93.84208538	84.57373127	40.64979222	86.79007682	89.20790832	48.1047727	97.46883264	92.02871175	96.86451801
AMP1CPR1STR3DOX3TMP3	AMP	CPR	STR	DOX	TMP	AMP+CPR	AMP+STR	AMP+DOX	AMP+TMP	CPR+STR	CPR+DOX	CPR+TMP	STR+DOX
	88.89160156	101.7822266	93.55176933	99.04785156	88.11035156	83.81347656	34.79003906	91.23535156	83.03222656	70.72753906	102.1728516	89.08691406	91.03538663
	94.15696955	102.4758604	85.80206563	101.2874474	91.97821243	93.1666254	29.38846249	86.63035405	86.23421639	52.36444665	102.2777915	82.47090864	94.37835011
	93.03614154	101.4985518	94.53113607	100.0881501	96.6628888	84.57373127	40.64979222	86.79007682	89.00642236	48.1047727	97.46883264	92.02871175	96.86451801
AMP1CPR2STR1DOX1TMP1	AMP	CPR	STR	DOX	TMP	AMP+CPR	AMP+STR	AMP+DOX	AMP+TMP	CPR+STR	CPR+DOX	CPR+TMP	STR+DOX
	88.89160156	104.1507103	97.48535156	103.9306641	83.22753906	81.46972656	46.19140625	90.06347656	88.50097656	9.894996912	104.3483632	89.3267449	84.20410156
	94.15696955	81.77863702	89.60138648	102.6739292	87.62069819	90.19559297	4.827927705	90.59173063	84.45159693	4.386176897	103.8182697	92.41179314	80.29215152
	93.03614154	103.2440056	86.58859086	98.47626244	85.17818914	89.61088024	4.508248331	89.40939428	80.54401209	9.462751635	102.2182331	87.4471086	67.85039668
AMP1CPR2STR1DOX1TMP2	AMP	CPR	STR	DOX	TMP	AMP+CPR	AMP+STR	AMP+DOX	AMP+TMP	CPR+STR	CPR+DOX	CPR+TMP	STR+DOX
	88.89160156	104.1507103	97.48535156	103.9306641	91.04003906	81.46972656	46.19140625	90.06347656	91.82128906	9.894996912	104.3483632	90.90796788	84.20410156
	94.15696955	81.77863702	89.60138648	102.6739292	93.36469423	90.19559297	4.827927705	90.59173063	87.42262936	4.386176897	103.8182697	91.25181247	80.29215152
	93.03614154	103.2440056	86.58859086	98.47626244	93.84208538	89.61088024	4.508248331	89.40939428	89.20790832	9.462751635	102.2182331	99.1409155	67.85039668
AMP1CPR2STR1DOX1TMP3	AMP	CPR	STR	DOX	TMP	AMP+CPR	AMP+STR	AMP+DOX	AMP+TMP	CPR+STR	CPR+DOX	CPR+TMP	STR+DOX
	88.89160156	104.1507103	97.48535156	103.9306641	88.11035156	81.46972656	46.19140625	90.06347656	83.03222656	9.894996912	104.3483632	90.90796788	84.20410156
	94.15696955	81.77863702	89.60138648	102.6739292	91.97821243	90.19559297	4.827927705	90.59173063	86.23421639	4.386176897	103.8182697	91.25181247	80.29215152
	93.03614154	103.2440056	86.58859086	98.47626244	96.6628888	89.61088024	4.508248331	89.40939428	89.00642236	9.462751635	102.2182331	97.70483395	67.85039668
AMP1CPR2STR1DOX2TMP1	AMP	CPR	STR	DOX	TMP	AMP+CPR	AMP+STR	AMP+DOX	AMP+TMP	CPR+STR	CPR+DOX	CPR+TMP	STR+DOX
	88.89160156	104.1507103	97.48535156	100.6103516	83.22753906	81.46972656	46.19140625	88.11035156	88.50097656	9.894996912	98.22112415	89.3267449	90.45410156
	94.15696955	81.77863702	89.60138648	102.2777915	87.62069819	90.19559297	4.827927705	83.06511513	84.45159693	4.386176897	98.21169647	92.41179314	92.37435009
	93.03614154	103.2440056	86.58859086	98.07329052	85.17818914	89.61088024	4.508248331	86.99156278	80.54401209	9.462751635	101.1924606	87.4471086	86.79007682
AMP1CPR2STR1DOX2TMP2	AMP	CPR	STR	DOX	TMP	AMP+CPR	AMP+STR	AMP+DOX	AMP+TMP	CPR+STR	CPR+DOX	CPR+TMP	STR+DOX
	88.89160156	104.1507103	97.48535156	100.6103516	91.04003906	81.46972656	46.19140625	88.11035156	91.82128906	9.894996912	98.22112415	90.90796788	90.45410156
	94.15696955	81.77863702	89.60138648	102.2777915	93.36469423	90.19559297	4.827927705	83.06511513	87.42262936	4.386176897	98.21169647	91.25181247	92.37435009
	93.03614154	103.2440056	86.58859086	98.07329052	93.84208538	89.61088024	4.508248331	86.99156278	89.20790832	9.462751635	101.1924606	99.1409155	86.79007682
AMP1CPR2STR1DOX2TMP3	AMP	CPR	STR	DOX	TMP	AMP+CPR	AMP+STR	AMP+DOX	AMP+TMP	CPR+STR	CPR+DOX	CPR+TMP	STR+DOX
	88.89160156	104.1507103	97.48535156	100.6103516	88.11035156	81.46972656	46.19140625	88.11035156	83.03222656	9.894996912	98.22112415	90.90796788	90.45410156
	94.15696955	81.77863702	89.60138648	102.2777915	91.97821243	90.19559297	4.827927705	83.06511513	86.23421639	4.386176897	98.21169647	91.25181247	92.37435009
	93.03614154	103.2440056	86.58859086	98.07329052	96.6628888	89.61088024	4.508248331	86.99156278	89.00642236	9.462751635	101.1924606	97.70483395	86.79007682
AMP1CPR2STR1DOX3TMP1	AMP	CPR	STR	DOX	TMP	AMP+CPR	AMP+STR	AMP+DOX	AMP+TMP	CPR+STR	CPR+DOX	CPR+TMP	STR+DOX
	88.89160156	104.1507103	97.48535156	99.04785156	83.22753906	81.46972656	46.19140625	91.23535156	88.50097656	9.894996912	102.3718345	89.3267449	81.86035156
	94.15696955	81.77863702	89.60138648	101.2874474	87.62069819	90.19559297	4.827927705	86.63035405	84.45159693	4.386176897	102.0782987	92.41179314	87.22456053
	93.03614154	103.2440056	86.58859086	100.0881501	85.17818914	89.61088024	4.508248331	86.79007682	80.54401209	9.462751635	103.4491601	87.4471086	86.18561894
AMP1CPR2STR1DOX3TMP2	AMP	CPR	STR	DOX	TMP	AMP+CPR	AMP+STR	AMP+DOX	AMP+TMP	CPR+STR	CPR+DOX	CPR+TMP	STR+DOX
	88.89160156	104.1507103	97.48535156	99.04785156	91.04003906	81.46972656	46.19140625	91.23535156	91.82128906	9.894996912	102.3718345	90.90796788	81.86035156
	94.15696955	81.77863702	89.60138648	101.2874474	93.36469423	90.19559297	4.827927705	86.63035405	87.42262936	4.386176897	102.0782987	91.25181247	87.22456053
	93.03614154	103.2440056	86.58859086	100.0881501	93.84208538	89.61088024	4.508248331	86.79007682	89.20790832	9.462751635	103.4491601	99.1409155	86.18561894
AMP1CPR2STR1DOX3TMP3	AMP	CPR	STR	DOX	TMP	AMP+CPR	AMP+STR	AMP+DOX	AMP+TMP	CPR+STR	CPR+DOX	CPR+TMP	STR+DOX
	88.89160156	104.1507103	97.48535156	99.04785156	88.11035156	81.46972656	46.19140625	91.23535156	83.03222656	9.894996912	102.3718345	90.90796788	81.86035156
	94.15696955	81.77863702	89.60138648	101.2874474	91.97821243	90.19559297	4.827927705	86.63035405	86.23421639	4.386176897	102.0782987	91.25181247	87.22456053
	93.03614154	103.2440056	86.58859086	100.0881501	96.6628888	89.61088024	4.508248331	86.79007682	89.00642236	9.462751635	103.4491601	97.70483395	86.18561894
AMP1CPR2STR2DOX1TMP1	AMP	CPR	STR	DOX	TMP	AMP+CPR	AMP+STR	AMP+DOX	AMP+TMP	CPR+STR	CPR+DOX	CPR+TMP	STR+DOX
	88.89160156	104.1507103	46.67078443	103.9306641	83.22753906	81.46972656	4.907226563	90.06347656	88.50097656	46.26312539	104.3483632	89.3267449	45.07720815
	94.15696955	81.77863702	50.16916385	102.6739292	87.62069819	90.19559297	4.827927705	90.59173063	84.45159693	12.69937168	103.8182697	92.41179314	3.28661189
	93.03614154	103.2440056	59.93076035	98.47626244	85.17818914	89.61088024	3.097846619	89.40939428	80.54401209	46.39056289	102.2182331	87.4471086	6.475189127
AMP1CPR2STR2DOX1TMP2	AMP	CPR	STR	DOX	TMP	AMP+CPR	AMP+STR	AMP+DOX	AMP+TMP	CPR+STR	CPR+DOX	CPR+TMP	STR+DOX
	88.89160156	104.1507103	46.67078443	103.9306641	91.04003906	81.46972656	4.907226563	90.06347656	91.82128906	46.26312539	104.3483632	90.90796788	45.07720815
	94.15696955	81.77863702	50.16916385	102.6739292	93.36469423	90.19559297	4.827927705	90.59173063	87.42262936	12.69937168	103.8182697	91.25181247	3.28661189
	93.03614154	103.2440056	59.93076035	98.47626244	93.84208538	89.61088024	3.097846619	89.40939428	89.20790832	46.39056289	102.2182331	99.1409155	6.475189127
AMP1CPR2STR2DOX1TMP3	AMP	CPR	STR	DOX	TMP	AMP+CPR	AMP+STR	AMP+DOX	AMP+TMP	CPR+STR	CPR+DOX	CPR+TMP	STR+DOX
	88.89160156	104.1507103	46.67078443	103.9306641	88.11035156	81.46972656	4.907226563	90.06347656	83.03222656	46.26312539	104.3483632	90.90796788	45.07720815
	94.15696955	81.77863702	50.16916385	102.6739292	91.97821243	90.19559297	4.827927705	90.59173063	86.23421639	12.69937168	103.8182697	91.25181247	3.28661189
	93.03614154	103.2440056	59.93076035	98.47626244	96.6628888	89.61088024	3.097846619	89.40939428	89.00642236	46.39056289	102.2182331	97.70483395	6.475189127
AMP1CPR2STR2DOX2TMP1	AMP	CPR	STR	DOX	TMP	AMP+CPR	AMP+STR	AMP+DOX	AMP+TMP	CPR+STR	CPR+DOX	CPR+TMP	STR+DOX
	88.89160156	104.1507103	46.67078443	100.6103516	83.22753906	81.46972656	4.907226563	88.11035156	88.50097656	46.26312539	98.22112415	89.3267449	61.6553428
	94.15696955	81.77863702	50.16916385	102.2777915	87.62069819	90.19559297	4.827927705	83.06511513	84.45159693	12.69937168	98.21169647	92.41179314	48.5258579
	93.03614154	103.2440056	59.93076035	98.07329052	85.17818914	89.61088024	3.097846619	86.99156278	80.54401209	46.39056289	101.1924606	87.4471086	67.13681241
AMP1CPR2STR2DOX2TMP2	AMP	CPR	STR	DOX	TMP	AMP+CPR	AMP+STR	AMP+DOX	AMP+TMP	CPR+STR	CPR+DOX	CPR+TMP	STR+DOX
	88.89160156	104.1507103	46.67078443	100.6103516	91.04003906	81.46972656	4.907226563	88.11035156	91.82128906	46.26312539	98.22112415	90.90796788	61.6553428
	94.15696955	81.77863702	50.16916385	102.2777915	93.36469423	90.19559297	4.827927705	83.06511513	87.42262936	12.69937168	98.21169647	91.25181247	48.5258579
	93.03614154	103.2440056	59.93076035	98.07329052	93.84208538	89.61088024	3.097846619	86.99156278	89.20790832	46.39056289	101.1924606	99.1409155	67.13681241
AMP1CPR2STR2DOX2TMP3	AMP	CPR	STR	DOX	TMP	AMP+CPR	AMP+STR	AMP+DOX	AMP+TMP	CPR+STR	CPR+DOX	CPR+TMP	STR+DOX
	88.89160156	104.1507103	46.67078443	100.6103516	88.11035156	81.46972656	4.907226563	88.11035156	83.03222656	46.26312539	98.22112415	90.90796788	61.6553428
	94.15696955	81.77863702	50.16916385	102.2777915	91.97821243	90.19559297	4.827927705	83.06511513	86.23421639	12.69937168	98.21169647	91.25181247	48.5258579
	93.03614154	103.2440056	59.93076035	98.07329052	96.6628888	89.61088024	3.097846619	86.99156278	89.00642236	46.39056289	101.1924606	97.70483395	67.13681241
AMP1CPR2STR2DOX3TMP1	AMP	CPR	STR	DOX	TMP	AMP+CPR	AMP+STR	AMP+DOX	AMP+TMP	CPR+STR	CPR+DOX	CPR+TMP	STR+DOX
	88.89160156	104.1507103	46.67078443	99.04785156	83.22753906	81.46972656	4.907226563	91.23535156	88.50097656	46.26312539	102.3718345	89.3267449	62.44595429
	94.15696955	81.77863702	50.16916385	101.2874474	87.62069819	90.19559297	4.827927705	86.63035405	84.45159693	12.69937168	102.0782987	92.41179314	54.22909618
	93.03614154	103.2440056	59.93076035	100.0881501	85.17818914	89.61088024	3.097846619	86.79007682	80.54401209	46.39056289	103.4491601	87.4471086	58.93063213
AMP1CPR2STR2DOX3TMP2	AMP	CPR	STR	DOX	TMP	AMP+CPR	AMP+STR	AMP+DOX	AMP+TMP	CPR+STR	CPR+DOX	CPR+TMP	STR+DOX
	88.89160156	104.1507103	46.67078443	99.04785156	91.04003906	81.46972656	4.907226563	91.23535156	91.82128906	46.26312539	102.3718345	90.90796788	62.44595429
	94.15696955	81.77863702	50.16916385	101.2874474	93.36469423	90.19559297	4.827927705	86.63035405	87.42262936	12.69937168	102.0782987	91.25181247	54.22909618
	93.03614154	103.2440056	59.93076035	100.0881501	93.84208538	89.61088024	3.097846619	86.79007682	89.20790832	46.39056289	103.4491601	99.1409155	58.93063213
AMP1CPR2STR2DOX3TMP3	AMP	CPR	STR	DOX	TMP	AMP+CPR	AMP+STR	AMP+DOX	AMP+TMP	CPR+STR	CPR+DOX	CPR+TMP	STR+DOX
	88.89160156	104.1507103	46.67078443	99.04785156	88.11035156	81.46972656	4.907226563	91.23535156	83.03222656	46.26312539	102.3718345	90.90796788	62.44595429
	94.15696955	81.77863702	50.16916385	101.2874474	91.97821243	90.19559297	4.827927705	86.63035405	86.23421639	12.69937168	102.0782987	91.25181247	54.22909618
	93.03614154	103.2440056	59.93076035	100.0881501	96.6628888	89.61088024	3.097846619	86.79007682	89.00642236	46.39056289	103.4491601	97.70483395	58.93063213
AMP1CPR2STR3DOX1TMP1	AMP	CPR	STR	DOX	TMP	AMP+CPR	AMP+STR	AMP+DOX	AMP+TMP	CPR+STR	CPR+DOX	CPR+TMP	STR+DOX
	88.89160156	104.1507103	93.55176933	103.9306641	83.22753906	81.46972656	34.79003906	90.06347656	88.50097656	61.26003706	104.3483632	89.3267449	83.48623853
	94.15696955	81.77863702	85.80206563	102.6739292	87.62069819	90.19559297	29.38846249	90.59173063	84.45159693	58.48235863	103.8182697	92.41179314	93.33246176
	93.03614154	103.2440056	94.53113607	98.47626244	85.17818914	89.61088024	40.64979222	89.40939428	80.54401209	66.52134889	102.2182331	87.4471086	92.43109231
AMP1CPR2STR3DOX1TMP2	AMP	CPR	STR	DOX	TMP	AMP+CPR	AMP+STR	AMP+DOX	AMP+TMP	CPR+STR	CPR+DOX	CPR+TMP	STR+DOX
	88.89160156	104.1507103	93.55176933	103.9306641	91.04003906	81.46972656	34.79003906	90.06347656	91.82128906	61.26003706	104.3483632	90.90796788	83.48623853
	94.15696955	81.77863702	85.80206563	102.6739292	93.36469423	90.19559297	29.38846249	90.59173063	87.42262936	58.48235863	103.8182697	91.25181247	93.33246176
	93.03614154	103.2440056	94.53113607	98.47626244	93.84208538	89.61088024	40.64979222	89.40939428	89.20790832	66.52134889	102.2182331	99.1409155	92.43109231
AMP1CPR2STR3DOX1TMP3	AMP	CPR	STR	DOX	TMP	AMP+CPR	AMP+STR	AMP+DOX	AMP+TMP	CPR+STR	CPR+DOX	CPR+TMP	STR+DOX
	88.89160156	104.1507103	93.55176933	103.9306641	88.11035156	81.46972656	34.79003906	90.06347656	83.03222656	61.26003706	104.3483632	90.90796788	83.48623853
	94.15696955	81.77863702	85.80206563	102.6739292	91.97821243	90.19559297	29.38846249	90.59173063	86.23421639	58.48235863	103.8182697	91.25181247	93.33246176
	93.03614154	103.2440056	94.53113607	98.47626244	96.6628888	89.61088024	40.64979222	89.40939428	89.00642236	66.52134889	102.2182331	97.70483395	92.43109231
AMP1CPR2STR3DOX2TMP1	AMP	CPR	STR	DOX	TMP	AMP+CPR	AMP+STR	AMP+DOX	AMP+TMP	CPR+STR	CPR+DOX	CPR+TMP	STR+DOX
	88.89160156	104.1507103	93.55176933	100.6103516	83.22753906	81.46972656	34.79003906	88.11035156	88.50097656	61.26003706	98.22112415	89.3267449	95.64875491
	94.15696955	81.77863702	85.80206563	102.2777915	87.62069819	90.19559297	29.38846249	83.06511513	84.45159693	58.48235863	98.21169647	92.41179314	96.67930448
	93.03614154	103.2440056	94.53113607	98.07329052	85.17818914	89.61088024	40.64979222	86.99156278	80.54401209	66.52134889	101.1924606	87.4471086	99.19789996
AMP1CPR2STR3DOX2TMP2	AMP	CPR	STR	DOX	TMP	AMP+CPR	AMP+STR	AMP+DOX	AMP+TMP	CPR+STR	CPR+DOX	CPR+TMP	STR+DOX
	88.89160156	104.1507103	93.55176933	100.6103516	91.04003906	81.46972656	34.79003906	88.11035156	91.82128906	61.26003706	98.22112415	90.90796788	95.64875491
	94.15696955	81.77863702	85.80206563	102.2777915	93.36469423	90.19559297	29.38846249	83.06511513	87.42262936	58.48235863	98.21169647	91.25181247	96.67930448
	93.03614154	103.2440056	94.53113607	98.07329052	93.84208538	89.61088024	40.64979222	86.99156278	89.20790832	66.52134889	101.1924606	99.1409155	99.19789996
AMP1CPR2STR3DOX2TMP3	AMP	CPR	STR	DOX	TMP	AMP+CPR	AMP+STR	AMP+DOX	AMP+TMP	CPR+STR	CPR+DOX	CPR+TMP	STR+DOX
	88.89160156	104.1507103	93.55176933	100.6103516	88.11035156	81.46972656	34.79003906	88.11035156	83.03222656	61.26003706	98.22112415	90.90796788	95.64875491
	94.15696955	81.77863702	85.80206563	102.2777915	91.97821243	90.19559297	29.38846249	83.06511513	86.23421639	58.48235863	98.21169647	91.25181247	96.67930448
	93.03614154	103.2440056	94.53113607	98.07329052	96.6628888	89.61088024	40.64979222	86.99156278	89.00642236	66.52134889	101.1924606	97.70483395	99.19789996
AMP1CPR2STR3DOX3TMP1	AMP	CPR	STR	DOX	TMP	AMP+CPR	AMP+STR	AMP+DOX	AMP+TMP	CPR+STR	CPR+DOX	CPR+TMP	STR+DOX
	88.89160156	104.1507103	93.55176933	99.04785156	83.22753906	81.46972656	34.79003906	91.23535156	88.50097656	61.26003706	102.3718345	89.3267449	91.03538663
	94.15696955	81.77863702	85.80206563	101.2874474	87.62069819	90.19559297	29.38846249	86.63035405	84.45159693	58.48235863	102.0782987	92.41179314	94.37835011
	93.03614154	103.2440056	94.53113607	100.0881501	85.17818914	89.61088024	40.64979222	86.79007682	80.54401209	66.52134889	103.4491601	87.4471086	96.86451801
AMP1CPR2STR3DOX3TMP2	AMP	CPR	STR	DOX	TMP	AMP+CPR	AMP+STR	AMP+DOX	AMP+TMP	CPR+STR	CPR+DOX	CPR+TMP	STR+DOX
	88.89160156	104.1507103	93.55176933	99.04785156	91.04003906	81.46972656	34.79003906	91.23535156	91.82128906	61.26003706	102.3718345	90.90796788	91.03538663
	94.15696955	81.77863702	85.80206563	101.2874474	93.36469423	90.19559297	29.38846249	86.63035405	87.42262936	58.48235863	102.0782987	91.25181247	94.37835011
	93.03614154	103.2440056	94.53113607	100.0881501	93.84208538	89.61088024	40.64979222	86.79007682	89.20790832	66.52134889	103.4491601	99.1409155	96.86451801
AMP1CPR2STR3DOX3TMP3	AMP	CPR	STR	DOX	TMP	AMP+CPR	AMP+STR	AMP+DOX	AMP+TMP	CPR+STR	CPR+DOX	CPR+TMP	STR+DOX
	88.89160156	104.1507103	93.55176933	99.04785156	88.11035156	81.46972656	34.79003906	91.23535156	83.03222656	61.26003706	102.3718345	90.90796788	91.03538663
	94.15696955	81.77863702	85.80206563	101.2874474	91.97821243	90.19559297	29.38846249	86.63035405	86.23421639	58.48235863	102.0782987	91.25181247	94.37835011
	93.03614154	103.2440056	94.53113607	100.0881501	96.6628888	89.61088024	40.64979222	86.79007682	89.00642236	66.52134889	103.4491601	97.70483395	96.86451801
AMP1CPR3STR1DOX1TMP1	AMP	CPR	STR	DOX	TMP	AMP+CPR	AMP+STR	AMP+DOX	AMP+TMP	CPR+STR	CPR+DOX	CPR+TMP	STR+DOX
	88.89160156	86.21231979	97.48535156	103.9306641	83.22753906	88.30566406	46.19140625	90.06347656	88.50097656	58.33551769	101.5203145	95.64875491	84.20410156
	94.15696955	102.3271016	89.60138648	102.6739292	87.62069819	99.50482793	4.827927705	90.59173063	84.45159693	45.28696562	106.0922996	97.09765982	80.29215152
	93.03614154	104.3313402	86.58859086	98.47626244	85.17818914	89.8123662	4.508248331	89.40939428	80.54401209	59.10748141	103.8646638	108.0647513	67.85039668
AMP1CPR3STR1DOX1TMP2	AMP	CPR	STR	DOX	TMP	AMP+CPR	AMP+STR	AMP+DOX	AMP+TMP	CPR+STR	CPR+DOX	CPR+TMP	STR+DOX
	88.89160156	86.21231979	97.48535156	103.9306641	91.04003906	88.30566406	46.19140625	90.06347656	91.82128906	58.33551769	101.5203145	101.1009174	84.20410156
	94.15696955	102.3271016	89.60138648	102.6739292	93.36469423	99.50482793	4.827927705	90.59173063	87.42262936	45.28696562	106.0922996	103.1638123	80.29215152
	93.03614154	104.3313402	86.58859086	98.47626244	93.84208538	89.8123662	4.508248331	89.40939428	89.20790832	59.10748141	103.8646638	106.6647222	67.85039668
AMP1CPR3STR1DOX1TMP3	AMP	CPR	STR	DOX	TMP	AMP+CPR	AMP+STR	AMP+DOX	AMP+TMP	CPR+STR	CPR+DOX	CPR+TMP	STR+DOX
	88.89160156	86.21231979	97.48535156	103.9306641	88.11035156	88.30566406	46.19140625	90.06347656	83.03222656	58.33551769	101.5203145	100.2621232	84.20410156
	94.15696955	102.3271016	89.60138648	102.6739292	91.97821243	99.50482793	4.827927705	90.59173063	86.23421639	45.28696562	106.0922996	98.77108119	80.29215152
	93.03614154	104.3313402	86.58859086	98.47626244	96.6628888	89.8123662	4.508248331	89.40939428	89.00642236	59.10748141	103.8646638	99.66457635	67.85039668
AMP1CPR3STR1DOX2TMP1	AMP	CPR	STR	DOX	TMP	AMP+CPR	AMP+STR	AMP+DOX	AMP+TMP	CPR+STR	CPR+DOX	CPR+TMP	STR+DOX
	88.89160156	86.21231979	97.48535156	100.6103516	83.22753906	88.30566406	46.19140625	88.11035156	88.50097656	58.33551769	96.69724771	95.64875491	90.45410156
	94.15696955	102.3271016	89.60138648	102.2777915	87.62069819	99.50482793	4.827927705	83.06511513	84.45159693	45.28696562	105.255589	97.09765982	92.37435009
	93.03614154	104.3313402	86.58859086	98.07329052	85.17818914	89.8123662	4.508248331	86.99156278	80.54401209	59.10748141	104.098002	108.0647513	86.79007682
AMP1CPR3STR1DOX2TMP2	AMP	CPR	STR	DOX	TMP	AMP+CPR	AMP+STR	AMP+DOX	AMP+TMP	CPR+STR	CPR+DOX	CPR+TMP	STR+DOX
	88.89160156	86.21231979	97.48535156	100.6103516	91.04003906	88.30566406	46.19140625	88.11035156	91.82128906	58.33551769	96.69724771	101.1009174	90.45410156
	94.15696955	102.3271016	89.60138648	102.2777915	93.36469423	99.50482793	4.827927705	83.06511513	87.42262936	45.28696562	105.255589	103.1638123	92.37435009
	93.03614154	104.3313402	86.58859086	98.07329052	93.84208538	89.8123662	4.508248331	86.99156278	89.20790832	59.10748141	104.098002	106.6647222	86.79007682
AMP1CPR3STR1DOX2TMP3	AMP	CPR	STR	DOX	TMP	AMP+CPR	AMP+STR	AMP+DOX	AMP+TMP	CPR+STR	CPR+DOX	CPR+TMP	STR+DOX
	88.89160156	86.21231979	97.48535156	100.6103516	88.11035156	88.30566406	46.19140625	88.11035156	83.03222656	58.33551769	96.69724771	100.2621232	90.45410156
	94.15696955	102.3271016	89.60138648	102.2777915	91.97821243	99.50482793	4.827927705	83.06511513	86.23421639	45.28696562	105.255589	98.77108119	92.37435009
	93.03614154	104.3313402	86.58859086	98.07329052	96.6628888	89.8123662	4.508248331	86.99156278	89.00642236	59.10748141	104.098002	99.66457635	86.79007682
AMP1CPR3STR1DOX3TMP1	AMP	CPR	STR	DOX	TMP	AMP+CPR	AMP+STR	AMP+DOX	AMP+TMP	CPR+STR	CPR+DOX	CPR+TMP	STR+DOX
	88.89160156	86.21231979	97.48535156	99.04785156	83.22753906	88.30566406	46.19140625	91.23535156	88.50097656	58.33551769	103.8269987	95.64875491	81.86035156
	94.15696955	102.3271016	89.60138648	101.2874474	87.62069819	99.50482793	4.827927705	86.63035405	84.45159693	45.28696562	105.4647666	97.09765982	87.22456053
	93.03614154	104.3313402	86.58859086	100.0881501	85.17818914	89.8123662	4.508248331	86.79007682	80.54401209	59.10748141	113.8982062	108.0647513	86.18561894
AMP1CPR3STR1DOX3TMP2	AMP	CPR	STR	DOX	TMP	AMP+CPR	AMP+STR	AMP+DOX	AMP+TMP	CPR+STR	CPR+DOX	CPR+TMP	STR+DOX
	88.89160156	86.21231979	97.48535156	99.04785156	91.04003906	88.30566406	46.19140625	91.23535156	91.82128906	58.33551769	103.8269987	101.1009174	81.86035156
	94.15696955	102.3271016	89.60138648	101.2874474	93.36469423	99.50482793	4.827927705	86.63035405	87.42262936	45.28696562	105.4647666	103.1638123	87.22456053
	93.03614154	104.3313402	86.58859086	100.0881501	93.84208538	89.8123662	4.508248331	86.79007682	89.20790832	59.10748141	113.8982062	106.6647222	86.18561894
AMP1CPR3STR1DOX3TMP3	AMP	CPR	STR	DOX	TMP	AMP+CPR	AMP+STR	AMP+DOX	AMP+TMP	CPR+STR	CPR+DOX	CPR+TMP	STR+DOX
	88.89160156	86.21231979	97.48535156	99.04785156	88.11035156	88.30566406	46.19140625	91.23535156	83.03222656	58.33551769	103.8269987	100.2621232	81.86035156
	94.15696955	102.3271016	89.60138648	101.2874474	91.97821243	99.50482793	4.827927705	86.63035405	86.23421639	45.28696562	105.4647666	98.77108119	87.22456053
	93.03614154	104.3313402	86.58859086	100.0881501	96.6628888	89.8123662	4.508248331	86.79007682	89.00642236	59.10748141	113.8982062	99.66457635	86.18561894
AMP1CPR3STR2DOX1TMP1	AMP	CPR	STR	DOX	TMP	AMP+CPR	AMP+STR	AMP+DOX	AMP+TMP	CPR+STR	CPR+DOX	CPR+TMP	STR+DOX
	88.89160156	86.21231979	46.67078443	103.9306641	83.22753906	88.30566406	4.907226563	90.06347656	88.50097656	58.15203145	101.5203145	95.64875491	45.07720815
	94.15696955	102.3271016	50.16916385	102.6739292	87.62069819	99.50482793	4.827927705	90.59173063	84.45159693	56.92247353	106.0922996	97.09765982	3.28661189
	93.03614154	104.3313402	59.93076035	98.47626244	85.17818914	89.8123662	3.097846619	89.40939428	80.54401209	64.13883623	103.8646638	108.0647513	6.475189127
AMP1CPR3STR2DOX1TMP2	AMP	CPR	STR	DOX	TMP	AMP+CPR	AMP+STR	AMP+DOX	AMP+TMP	CPR+STR	CPR+DOX	CPR+TMP	STR+DOX
	88.89160156	86.21231979	46.67078443	103.9306641	91.04003906	88.30566406	4.907226563	90.06347656	91.82128906	58.15203145	101.5203145	101.1009174	45.07720815
	94.15696955	102.3271016	50.16916385	102.6739292	93.36469423	99.50482793	4.827927705	90.59173063	87.42262936	56.92247353	106.0922996	103.1638123	3.28661189
	93.03614154	104.3313402	59.93076035	98.47626244	93.84208538	89.8123662	3.097846619	89.40939428	89.20790832	64.13883623	103.8646638	106.6647222	6.475189127
AMP1CPR3STR2DOX1TMP3	AMP	CPR	STR	DOX	TMP	AMP+CPR	AMP+STR	AMP+DOX	AMP+TMP	CPR+STR	CPR+DOX	CPR+TMP	STR+DOX
	88.89160156	86.21231979	46.67078443	103.9306641	88.11035156	88.30566406	4.907226563	90.06347656	83.03222656	58.15203145	101.5203145	100.2621232	45.07720815
	94.15696955	102.3271016	50.16916385	102.6739292	91.97821243	99.50482793	4.827927705	90.59173063	86.23421639	56.92247353	106.0922996	98.77108119	3.28661189
	93.03614154	104.3313402	59.93076035	98.47626244	96.6628888	89.8123662	3.097846619	89.40939428	89.00642236	64.13883623	103.8646638	99.66457635	6.475189127
AMP1CPR3STR2DOX2TMP1	AMP	CPR	STR	DOX	TMP	AMP+CPR	AMP+STR	AMP+DOX	AMP+TMP	CPR+STR	CPR+DOX	CPR+TMP	STR+DOX
	88.89160156	86.21231979	46.67078443	100.6103516	83.22753906	88.30566406	4.907226563	88.11035156	88.50097656	58.15203145	96.69724771	95.64875491	61.6553428
	94.15696955	102.3271016	50.16916385	102.2777915	87.62069819	99.50482793	4.827927705	83.06511513	84.45159693	56.92247353	105.255589	97.09765982	48.5258579
	93.03614154	104.3313402	59.93076035	98.07329052	85.17818914	89.8123662	3.097846619	86.99156278	80.54401209	64.13883623	104.098002	108.0647513	67.13681241
AMP1CPR3STR2DOX2TMP2	AMP	CPR	STR	DOX	TMP	AMP+CPR	AMP+STR	AMP+DOX	AMP+TMP	CPR+STR	CPR+DOX	CPR+TMP	STR+DOX
	88.89160156	86.21231979	46.67078443	100.6103516	91.04003906	88.30566406	4.907226563	88.11035156	91.82128906	58.15203145	96.69724771	101.1009174	61.6553428
	94.15696955	102.3271016	50.16916385	102.2777915	93.36469423	99.50482793	4.827927705	83.06511513	87.42262936	56.92247353	105.255589	103.1638123	48.5258579
	93.03614154	104.3313402	59.93076035	98.07329052	93.84208538	89.8123662	3.097846619	86.99156278	89.20790832	64.13883623	104.098002	106.6647222	67.13681241
AMP1CPR3STR2DOX2TMP3	AMP	CPR	STR	DOX	TMP	AMP+CPR	AMP+STR	AMP+DOX	AMP+TMP	CPR+STR	CPR+DOX	CPR+TMP	STR+DOX
	88.89160156	86.21231979	46.67078443	100.6103516	88.11035156	88.30566406	4.907226563	88.11035156	83.03222656	58.15203145	96.69724771	100.2621232	61.6553428
	94.15696955	102.3271016	50.16916385	102.2777915	91.97821243	99.50482793	4.827927705	83.06511513	86.23421639	56.92247353	105.255589	98.77108119	48.5258579
	93.03614154	104.3313402	59.93076035	98.07329052	96.6628888	89.8123662	3.097846619	86.99156278	89.00642236	64.13883623	104.098002	99.66457635	67.13681241
AMP1CPR3STR2DOX3TMP1	AMP	CPR	STR	DOX	TMP	AMP+CPR	AMP+STR	AMP+DOX	AMP+TMP	CPR+STR	CPR+DOX	CPR+TMP	STR+DOX
	88.89160156	86.21231979	46.67078443	99.04785156	83.22753906	88.30566406	4.907226563	91.23535156	88.50097656	58.15203145	103.8269987	95.64875491	62.44595429
	94.15696955	102.3271016	50.16916385	101.2874474	87.62069819	99.50482793	4.827927705	86.63035405	84.45159693	56.92247353	105.4647666	97.09765982	54.22909618
	93.03614154	104.3313402	59.93076035	100.0881501	85.17818914	89.8123662	3.097846619	86.79007682	80.54401209	64.13883623	113.8982062	108.0647513	58.93063213
AMP1CPR3STR2DOX3TMP2	AMP	CPR	STR	DOX	TMP	AMP+CPR	AMP+STR	AMP+DOX	AMP+TMP	CPR+STR	CPR+DOX	CPR+TMP	STR+DOX
	88.89160156	86.21231979	46.67078443	99.04785156	91.04003906	88.30566406	4.907226563	91.23535156	91.82128906	58.15203145	103.8269987	101.1009174	62.44595429

AMP1CPR1STR3DOX2TMP3	STR+TMP	DOX+TMP	AMP+CPR+STR	AMP+CPR+DOX	AMP+CPR+TMP	AMP+STR+DOX	AMP+STR+TMP	AMP+DOX+TMP	CPR+STR+DOX
	94.60026212	94.55566406	24.85351563	66.04003906	87.52441406	52.36816406	25.61035156	74.82910156	51.58691406
	94.16917244	86.03614756	20.63629611	78.1133944	90.78979946	27.45729141	34.14211438	87.02649171	54.19658331
	93.1311069	97.46883264	20.66490366	89.20790832	96.46140285	41.05276414	29.16509256	97.6703186	56.93237628
AMP1CPR1STR3DOX3TMP1	STR+TMP	DOX+TMP	AMP+CPR+STR	AMP+CPR+DOX	AMP+CPR+TMP	AMP+STR+DOX	AMP+STR+TMP	AMP+DOX+TMP	CPR+STR+DOX
	89.56749672	79.51660156	24.85351563	73.07128906	79.12597656	33.22753906	32.47070313	84.59472656	55.32226563
	96.88848215	82.47090864	20.63629611	87.81876702	81.67863333	24.48625897	40.72790295	94.75117603	60.9309235
	96.63117982	84.37224531	20.66490366	85.17818914	93.84208538	37.35045964	40.6120136	100.0881501	51.08928347
AMP1CPR1STR3DOX3TMP2	STR+TMP	DOX+TMP	AMP+CPR+STR	AMP+CPR+DOX	AMP+CPR+TMP	AMP+STR+DOX	AMP+STR+TMP	AMP+DOX+TMP	CPR+STR+DOX
	88.30930537	77.56347656	24.85351563	73.07128906	90.25878906	33.22753906	19.16503906	86.15722656	55.32226563
	92.91410642	85.83807873	20.63629611	87.81876702	86.03614756	24.48625897	43.10472889	90.19559297	60.9309235
	96.86451801	95.65545901	20.66490366	85.17818914	97.06586072	37.35045964	34.36594887	97.26734668	51.08928347
AMP1CPR1STR3DOX3TMP3	STR+TMP	DOX+TMP	AMP+CPR+STR	AMP+CPR+DOX	AMP+CPR+TMP	AMP+STR+DOX	AMP+STR+TMP	AMP+DOX+TMP	CPR+STR+DOX
	94.60026212	93.96972656	24.85351563	73.07128906	87.52441406	33.22753906	25.61035156	89.28222656	55.32226563
	94.16917244	101.0893786	20.63629611	87.81876702	90.78979946	24.48625897	34.14211438	96.13765784	60.9309235
	93.1311069	104.5208412	20.66490366	85.17818914	96.46140285	37.35045964	29.16509256	90.61831004	51.08928347
AMP1CPR2STR1DOX1TMP1	STR+TMP	DOX+TMP	AMP+CPR+STR	AMP+CPR+DOX	AMP+CPR+TMP	AMP+STR+DOX	AMP+STR+TMP	AMP+DOX+TMP	CPR+STR+DOX
	81.07910156	83.61816406	4.00390625	86.74316406	89.67285156	3.61328125	7.397460938	77.56347656	34.40395306
	82.66897747	76.7269126	7.798960139	83.65932161	93.36469423	3.639514731	0.235206734	80.68828918	2.126631223
	82.15589976	85.17818914	3.097846619	94.44654326	98.67774839	5.717164085	4.306762372	82.76035764	3.923579946
AMP1CPR2STR1DOX1TMP2	STR+TMP	DOX+TMP	AMP+CPR+STR	AMP+CPR+DOX	AMP+CPR+TMP	AMP+STR+DOX	AMP+STR+TMP	AMP+DOX+TMP	CPR+STR+DOX
	81.07910156	84.20410156	4.00390625	86.74316406	72.29003906	3.61328125	6.811523438	76.00097656	34.40395306
	81.08442684	80.49022035	7.798960139	83.65932161	88.01683585	3.639514731	2.017826195	90.3936618	2.126631223
	85.17818914	87.79750661	3.097846619	94.44654326	88.80493641	5.717164085	4.306762372	86.99156278	3.923579946
AMP1CPR2STR1DOX1TMP3	STR+TMP	DOX+TMP	AMP+CPR+STR	AMP+CPR+DOX	AMP+CPR+TMP	AMP+STR+DOX	AMP+STR+TMP	AMP+DOX+TMP	CPR+STR+DOX
	86.54785156	94.36035156	4.00390625	86.74316406	64.86816406	3.61328125	7.788085938	85.96191406	34.40395306
	84.45159693	99.10869027	7.798960139	83.65932161	85.24387225	3.639514731	4.394652142	95.54345135	2.126631223
	90.01385216	109.1550183	3.097846619	94.44654326	80.74549805	5.717164085	3.173403853	93.2376275	3.923579946
AMP1CPR2STR1DOX2TMP1	STR+TMP	DOX+TMP	AMP+CPR+STR	AMP+CPR+DOX	AMP+CPR+TMP	AMP+STR+DOX	AMP+STR+TMP	AMP+DOX+TMP	CPR+STR+DOX
	81.07910156	83.81347656	4.00390625	90.64941406	89.67285156	3.61328125	7.397460938	83.42285156	6.114885732
	82.66897747	84.84773459	7.798960139	89.40331765	93.36469423	0.866551127	0.235206734	91.18593711	3.866602223
	82.15589976	95.65545901	3.097846619	95.05100113	98.67774839	5.918650044	4.306762372	89.61088024	7.706116169
AMP1CPR2STR1DOX2TMP2	STR+TMP	DOX+TMP	AMP+CPR+STR	AMP+CPR+DOX	AMP+CPR+TMP	AMP+STR+DOX	AMP+STR+TMP	AMP+DOX+TMP	CPR+STR+DOX
	81.07910156	88.69628906	4.00390625	90.64941406	72.29003906	3.61328125	6.811523438	78.93066406	6.114885732
	81.08442684	83.26318396	7.798960139	89.40331765	88.01683585	0.866551127	2.017826195	91.38400594	3.866602223
	85.17818914	96.6628888	3.097846619	95.05100113	88.80493641	5.918650044	4.306762372	97.46883264	7.706116169
AMP1CPR2STR1DOX2TMP3	STR+TMP	DOX+TMP	AMP+CPR+STR	AMP+CPR+DOX	AMP+CPR+TMP	AMP+STR+DOX	AMP+STR+TMP	AMP+DOX+TMP	CPR+STR+DOX
	86.54785156	94.55566406	4.00390625	90.64941406	64.86816406	3.61328125	7.788085938	74.82910156	6.114885732
	84.45159693	86.03614756	7.798960139	89.40331765	85.24387225	0.866551127	4.394652142	87.02649171	3.866602223
	90.01385216	97.46883264	3.097846619	95.05100113	80.74549805	5.918650044	3.173403853	97.6703186	7.706116169
AMP1CPR2STR1DOX3TMP1	STR+TMP	DOX+TMP	AMP+CPR+STR	AMP+CPR+DOX	AMP+CPR+TMP	AMP+STR+DOX	AMP+STR+TMP	AMP+DOX+TMP	CPR+STR+DOX
	81.07910156	79.51660156	4.00390625	89.08691406	89.67285156	5.053710938	7.397460938	84.59472656	8.906732551
	82.66897747	82.47090864	7.798960139	97.32607081	93.36469423	0.829413221	0.235206734	94.75117603	3.093281779
	82.15589976	84.37224531	3.097846619	96.86437476	98.67774839	5.717164085	4.306762372	100.0881501	1.461725862
AMP1CPR2STR1DOX3TMP2	STR+TMP	DOX+TMP	AMP+CPR+STR	AMP+CPR+DOX	AMP+CPR+TMP	AMP+STR+DOX	AMP+STR+TMP	AMP+DOX+TMP	CPR+STR+DOX
	81.07910156	77.56347656	4.00390625	89.08691406	72.29003906	5.053710938	6.811523438	86.15722656	8.906732551
	81.08442684	85.83807873	7.798960139	97.32607081	88.01683585	0.829413221	2.017826195	90.19559297	3.093281779
	85.17818914	95.65545901	3.097846619	96.86437476	88.80493641	5.717164085	4.306762372	97.26734668	1.461725862
AMP1CPR2STR1DOX3TMP3	STR+TMP	DOX+TMP	AMP+CPR+STR	AMP+CPR+DOX	AMP+CPR+TMP	AMP+STR+DOX	AMP+STR+TMP	AMP+DOX+TMP	CPR+STR+DOX
	86.54785156	93.96972656	4.00390625	89.08691406	64.86816406	5.053710938	7.788085938	89.28222656	8.906732551
	84.45159693	101.0893786	7.798960139	97.32607081	85.24387225	0.829413221	4.394652142	96.13765784	3.093281779
	90.01385216	104.5208412	3.097846619	96.86437476	80.74549805	5.717164085	3.173403853	90.61831004	1.461725862
AMP1CPR2STR2DOX1TMP1	STR+TMP	DOX+TMP	AMP+CPR+STR	AMP+CPR+DOX	AMP+CPR+TMP	AMP+STR+DOX	AMP+STR+TMP	AMP+DOX+TMP	CPR+STR+DOX
	49.20321186	83.61816406	3.61328125	86.74316406	89.67285156	3.41796875	7.983398438	77.56347656	53.77393453
	54.03576607	76.7269126	4.827927705	83.65932161	93.36469423	3.83758356	0.631344392	80.68828918	31.06573224
	57.08424157	85.17818914	2.694874701	94.44654326	98.67774839	4.911220249	42.99206649	82.76035764	45.45454545
AMP1CPR2STR2DOX1TMP2	STR+TMP	DOX+TMP	AMP+CPR+STR	AMP+CPR+DOX	AMP+CPR+TMP	AMP+STR+DOX	AMP+STR+TMP	AMP+DOX+TMP	CPR+STR+DOX
	63.03891291	84.20410156	3.61328125	86.74316406	72.29003906	3.41796875	9.66796875	76.00097656	53.77393453
	54.80908652	80.49022035	4.827927705	83.65932161	88.01683585	3.83758356	1.02748205	90.3936618	31.06573224
	73.08629311	87.79750661	2.694874701	94.44654326	88.80493641	4.911220249	1.322251606	86.99156278	45.45454545
AMP1CPR2STR2DOX1TMP3	STR+TMP	DOX+TMP	AMP+CPR+STR	AMP+CPR+DOX	AMP+CPR+TMP	AMP+STR+DOX	AMP+STR+TMP	AMP+DOX+TMP	CPR+STR+DOX
	69.75911056	94.36035156	3.61328125	86.74316406	64.86816406	3.41796875	8.374023437	85.96191406	53.77393453
	54.71242146	99.10869027	4.827927705	83.65932161	85.24387225	3.83758356	4.592720971	95.54345135	31.06573224
	71.65021157	109.1550183	2.694874701	94.44654326	80.74549805	4.911220249	2.165974059	93.2376275	45.45454545
AMP1CPR2STR2DOX2TMP1	STR+TMP	DOX+TMP	AMP+CPR+STR	AMP+CPR+DOX	AMP+CPR+TMP	AMP+STR+DOX	AMP+STR+TMP	AMP+DOX+TMP	CPR+STR+DOX
	49.20321186	83.81347656	3.61328125	90.64941406	89.67285156	3.22265625	7.983398438	83.42285156	51.61210624
	54.03576607	84.84773459	4.827927705	89.40331765	93.36469423	0.074275811	0.631344392	91.18593711	51.75205413
	57.08424157	95.65545901	2.694874701	95.05100113	98.67774839	4.508248331	42.99206649	89.61088024	54.59674317
AMP1CPR2STR2DOX2TMP2	STR+TMP	DOX+TMP	AMP+CPR+STR	AMP+CPR+DOX	AMP+CPR+TMP	AMP+STR+DOX	AMP+STR+TMP	AMP+DOX+TMP	CPR+STR+DOX
	63.03891291	88.69628906	3.61328125	90.64941406	72.29003906	3.22265625	9.66796875	78.93066406	51.61210624
	54.80908652	83.26318396	4.827927705	89.40331765	88.01683585	0.074275811	1.02748205	91.38400594	51.75205413
	73.08629311	96.6628888	2.694874701	95.05100113	88.80493641	4.508248331	1.322251606	97.46883264	54.59674317
AMP1CPR2STR2DOX2TMP3	STR+TMP	DOX+TMP	AMP+CPR+STR	AMP+CPR+DOX	AMP+CPR+TMP	AMP+STR+DOX	AMP+STR+TMP	AMP+DOX+TMP	CPR+STR+DOX
	69.75911056	94.55566406	3.61328125	90.64941406	64.86816406	3.22265625	8.374023437	74.82910156	51.61210624
	54.71242146	86.03614756	4.827927705	89.40331765	85.24387225	0.074275811	4.592720971	87.02649171	51.75205413
	71.65021157	97.46883264	2.694874701	95.05100113	80.74549805	4.508248331	2.165974059	97.6703186	54.59674317
AMP1CPR2STR2DOX3TMP1	STR+TMP	DOX+TMP	AMP+CPR+STR	AMP+CPR+DOX	AMP+CPR+TMP	AMP+STR+DOX	AMP+STR+TMP	AMP+DOX+TMP	CPR+STR+DOX
	49.20321186	79.51660156	3.61328125	89.08691406	89.67285156	7.397460938	7.983398438	84.59472656	44.69425571
	54.03576607	82.47090864	4.827927705	97.32607081	93.36469423	0.433275563	0.631344392	94.75117603	58.67568874
	57.08424157	84.37224531	2.694874701	96.86437476	98.67774839	3.903790455	42.99206649	100.0881501	54.80189768
AMP1CPR2STR2DOX3TMP2	STR+TMP	DOX+TMP	AMP+CPR+STR	AMP+CPR+DOX	AMP+CPR+TMP	AMP+STR+DOX	AMP+STR+TMP	AMP+DOX+TMP	CPR+STR+DOX
	63.03891291	77.56347656	3.61328125	89.08691406	72.29003906	7.397460938	9.66796875	86.15722656	44.69425571
	54.80908652	85.83807873	4.827927705	97.32607081	88.01683585	0.433275563	1.02748205	90.19559297	58.67568874
	73.08629311	95.65545901	2.694874701	96.86437476	88.80493641	3.903790455	1.322251606	97.26734668	54.80189768
AMP1CPR2STR2DOX3TMP3	STR+TMP	DOX+TMP	AMP+CPR+STR	AMP+CPR+DOX	AMP+CPR+TMP	AMP+STR+DOX	AMP+STR+TMP	AMP+DOX+TMP	CPR+STR+DOX
	69.75911056	93.96972656	3.61328125	89.08691406	64.86816406	7.397460938	8.374023437	89.28222656	44.69425571
	54.71242146	101.0893786	4.827927705	97.32607081	85.24387225	0.433275563	4.592720971	96.13765784	58.67568874
	71.65021157	104.5208412	2.694874701	96.86437476	80.74549805	3.903790455	2.165974059	90.61831004	54.80189768
AMP1CPR2STR3DOX1TMP1	STR+TMP	DOX+TMP	AMP+CPR+STR	AMP+CPR+DOX	AMP+CPR+TMP	AMP+STR+DOX	AMP+STR+TMP	AMP+DOX+TMP	CPR+STR+DOX
	89.56749672	83.61816406	21.53320313	86.74316406	89.67285156	22.16796875	32.47070313	77.56347656	48.44966028
	96.88848215	76.7269126	20.92102005	83.65932161	93.36469423	6.808615994	40.72790295	80.68828918	54.42242629
	96.63117982	85.17818914	21.03009697	94.44654326	98.67774839	37.14897368	40.6120136	82.76035764	58.2895243
AMP1CPR2STR3DOX1TMP2	STR+TMP	DOX+TMP	AMP+CPR+STR	AMP+CPR+DOX	AMP+CPR+TMP	AMP+STR+DOX	AMP+STR+TMP	AMP+DOX+TMP	CPR+STR+DOX
	88.30930537	84.20410156	21.53320313	86.74316406	72.29003906	22.16796875	19.16503906	76.00097656	48.44966028
	92.91410642	80.49022035	20.92102005	83.65932161	88.01683585	6.808615994	43.10472889	90.3936618	54.42242629
	96.86451801	87.79750661	21.03009697	94.44654326	88.80493641	37.14897368	34.36594887	86.99156278	58.2895243
AMP1CPR2STR3DOX1TMP3	STR+TMP	DOX+TMP	AMP+CPR+STR	AMP+CPR+DOX	AMP+CPR+TMP	AMP+STR+DOX	AMP+STR+TMP	AMP+DOX+TMP	CPR+STR+DOX
	94.60026212	94.36035156	21.53320313	86.74316406	64.86816406	22.16796875	25.61035156	85.96191406	48.44966028
	94.16917244	99.10869027	20.92102005	83.65932161	85.24387225	6.808615994	34.14211438	95.54345135	54.42242629
	93.1311069	109.1550183	21.03009697	94.44654326	80.74549805	37.14897368	29.16509256	93.2376275	58.2895243
AMP1CPR2STR3DOX2TMP1	STR+TMP	DOX+TMP	AMP+CPR+STR	AMP+CPR+DOX	AMP+CPR+TMP	AMP+STR+DOX	AMP+STR+TMP	AMP+DOX+TMP	CPR+STR+DOX
	89.56749672	83.81347656	21.53320313	90.64941406	89.67285156	52.36816406	32.47070313	83.42285156	59.08585547
	96.88848215	84.84773459	20.92102005	89.40331765	93.36469423	27.45729141	40.72790295	91.18593711	53.26244563
	96.63117982	95.65545901	21.03009697	95.05100113	98.67774839	41.05276414	40.6120136	89.61088024	51.33991537
AMP1CPR2STR3DOX2TMP2	STR+TMP	DOX+TMP	AMP+CPR+STR	AMP+CPR+DOX	AMP+CPR+TMP	AMP+STR+DOX	AMP+STR+TMP	AMP+DOX+TMP	CPR+STR+DOX
	88.30930537	88.69628906	21.53320313	90.64941406	72.29003906	52.36816406	19.16503906	78.93066406	59.08585547
	92.91410642	83.26318396	20.92102005	89.40331765	88.01683585	27.45729141	43.10472889	91.38400594	53.26244563
	96.86451801	96.6628888	21.03009697	95.05100113	88.80493641	41.05276414	34.36594887	97.46883264	51.33991537
AMP1CPR2STR3DOX2TMP3	STR+TMP	DOX+TMP	AMP+CPR+STR	AMP+CPR+DOX	AMP+CPR+TMP	AMP+STR+DOX	AMP+STR+TMP	AMP+DOX+TMP	CPR+STR+DOX
	94.60026212	94.55566406	21.53320313	90.64941406	64.86816406	52.36816406	25.61035156	74.82910156	59.08585547
	94.16917244	86.03614756	20.92102005	89.40331765	85.24387225	27.45729141	34.14211438	87.02649171	53.26244563
	93.1311069	97.46883264	21.03009697	95.05100113	80.74549805	41.05276414	29.16509256	97.6703186	51.33991537
AMP1CPR2STR3DOX3TMP1	STR+TMP	DOX+TMP	AMP+CPR+STR	AMP+CPR+DOX	AMP+CPR+TMP	AMP+STR+DOX	AMP+STR+TMP	AMP+DOX+TMP	CPR+STR+DOX
	89.56749672	79.51660156	21.53320313	89.08691406	89.67285156	33.22753906	32.47070313	84.59472656	38.56701668
	96.88848215	82.47090864	20.92102005	97.32607081	93.36469423	24.48625897	40.72790295	94.75117603	59.83566941
	96.63117982	84.37224531	21.03009697	96.86437476	98.67774839	37.35045964	40.6120136	100.0881501	53.16066162
AMP1CPR2STR3DOX3TMP2	STR+TMP	DOX+TMP	AMP+CPR+STR	AMP+CPR+DOX	AMP+CPR+TMP	AMP+STR+DOX	AMP+STR+TMP	AMP+DOX+TMP	CPR+STR+DOX
	88.30930537	77.56347656	21.53320313	89.08691406	72.29003906	33.22753906	19.16503906	86.15722656	38.56701668
	92.91410642	85.83807873	20.92102005	97.32607081	88.01683585	24.48625897	43.10472889	90.19559297	59.83566941
	96.86451801	95.65545901	21.03009697	96.86437476	88.80493641	37.35045964	34.36594887	97.26734668	53.16066162
AMP1CPR2STR3DOX3TMP3	STR+TMP	DOX+TMP	AMP+CPR+STR	AMP+CPR+DOX	AMP+CPR+TMP	AMP+STR+DOX	AMP+STR+TMP	AMP+DOX+TMP	CPR+STR+DOX
	94.60026212	93.96972656	21.53320313	89.08691406	64.86816406	33.22753906	25.61035156	89.28222656	38.56701668
	94.16917244	101.0893786	20.92102005	97.32607081	85.24387225	24.48625897	34.14211438	96.13765784	59.83566941
	93.1311069	104.5208412	21.03009697	96.86437476	80.74549805	37.35045964	29.16509256	90.61831004	53.16066162
AMP1CPR3STR1DOX1TMP1	STR+TMP	DOX+TMP	AMP+CPR+STR	AMP+CPR+DOX	AMP+CPR+TMP	AMP+STR+DOX	AMP+STR+TMP	AMP+DOX+TMP	CPR+STR+DOX
	81.07910156	83.61816406	3.149414063	95.33691406	90.45410156	3.61328125	7.397460938	77.56347656	1.323722149
	82.66897747	76.7269126	4.431790047	90.59173063	91.18593711	3.639514731	0.235206734	80.68828918	3.804418878
	82.15589976	85.17818914	3.097846619	91.82722579	95.65545901	5.717164085	4.306762372	82.76035764	20.14000292
AMP1CPR3STR1DOX1TMP2	STR+TMP	DOX+TMP	AMP+CPR+STR	AMP+CPR+DOX	AMP+CPR+TMP	AMP+STR+DOX	AMP+STR+TMP	AMP+DOX+TMP	CPR+STR+DOX
	81.07910156	84.20410156	3.149414063	95.33691406	85.96191406	3.61328125	6.811523438	76.00097656	1.323722149
	81.08442684	80.49022035	4.431790047	90.59173063	86.43228522	3.639514731	2.017826195	90.3936618	3.804418878
	85.17818914	87.79750661	3.097846619	91.82722579	93.2376275	5.717164085	4.306762372	86.99156278	20.14000292
AMP1CPR3STR1DOX1TMP3	STR+TMP	DOX+TMP	AMP+CPR+STR	AMP+CPR+DOX	AMP+CPR+TMP	AMP+STR+DOX	AMP+STR+TMP	AMP+DOX+TMP	CPR+STR+DOX
	86.54785156	94.36035156	3.149414063	95.33691406	81.27441406	3.61328125	7.788085938	85.96191406	1.323722149
	84.45159693	99.10869027	4.431790047	90.59173063	81.67863333	3.639514731	4.394652142	95.54345135	3.804418878
	90.01385216	109.1550183	3.097846619	91.82722579	77.92469462	5.717164085	3.173403853	93.2376275	20.14000292
AMP1CPR3STR1DOX2TMP1	STR+TMP	DOX+TMP	AMP+CPR+STR	AMP+CPR+DOX	AMP+CPR+TMP	AMP+STR+DOX	AMP+STR+TMP	AMP+DOX+TMP	CPR+STR+DOX
	81.07910156	83.81347656	3.149414063	93.18847656	90.45410156	3.61328125	7.397460938	83.42285156	2.346002621
	82.66897747	84.84773459	4.431790047	85.83807873	91.18593711	0.866551127	0.235206734	91.18593711	7.151261603
	82.15589976	95.65545901	3.097846619	92.02871175	95.65545901	5.918650044	4.306762372	89.61088024	38.80705848
AMP1CPR3STR1DOX2TMP2	STR+TMP	DOX+TMP	AMP+CPR+STR	AMP+CPR+DOX	AMP+CPR+TMP	AMP+STR+DOX	AMP+STR+TMP	AMP+DOX+TMP	CPR+STR+DOX
	81.07910156	88.69628906	3.149414063	93.18847656	85.96191406	3.61328125	6.811523438	78.93066406	2.346002621
	81.08442684	83.26318396	4.431790047	85.83807873	86.43228522	0.866551127	2.017826195	91.38400594	7.151261603
	85.17818914	96.6628888	3.097846619	92.02871175	93.2376275	5.918650044	4.306762372	97.46883264	38.80705848
AMP1CPR3STR1DOX2TMP3	STR+TMP	DOX+TMP	AMP+CPR+STR	AMP+CPR+DOX	AMP+CPR+TMP	AMP+STR+DOX	AMP+STR+TMP	AMP+DOX+TMP	CPR+STR+DOX
	86.54785156	94.55566406	3.149414063	93.18847656	81.27441406	3.61328125	7.788085938	74.82910156	2.346002621
	84.45159693	86.03614756	4.431790047	85.83807873	81.67863333	0.866551127	4.394652142	87.02649171	7.151261603
	90.01385216	97.46883264	3.097846619	92.02871175	77.92469462	5.918650044	3.173403853	97.6703186	38.80705848
AMP1CPR3STR1DOX3TMP1	STR+TMP	DOX+TMP	AMP+CPR+STR	AMP+CPR+DOX	AMP+CPR+TMP	AMP+STR+DOX	AMP+STR+TMP	AMP+DOX+TMP	CPR+STR+DOX
	81.07910156	79.51660156	3.149414063	87.91503906	90.45410156	5.053710938	7.397460938	84.59472656	1.703800786
	82.66897747	82.47090864	4.431790047	88.61104234	91.18593711	0.829413221	0.235206734	94.75117603	4.431951889
	82.15589976	84.37224531	3.097846619	95.65545901	95.65545901	5.717164085	4.306762372	100.0881501	3.106314715
AMP1CPR3STR1DOX3TMP2	STR+TMP	DOX+TMP	AMP+CPR+STR	AMP+CPR+DOX	AMP+CPR+TMP	AMP+STR+DOX	AMP+STR+TMP	AMP+DOX+TMP	CPR+STR+DOX
	81.07910156	77.56347656	3.149414063	87.91503906	85.96191406	5.053710938	6.811523438	86.15722656	1.703800786
	81.08442684	85.83807873	4.431790047	88.61104234	86.43228522	0.829413221	2.017826195	90.19559297	4.431951889
	85.17818914	95.65545901	3.097846619	95.65545901	93.2376275	5.717164085	4.306762372	97.26734668	3.106314715
AMP1CPR3STR1DOX3TMP3	STR+TMP	DOX+TMP	AMP+CPR+STR	AMP+CPR+DOX	AMP+CPR+TMP	AMP+STR+DOX	AMP+STR+TMP	AMP+DOX+TMP	CPR+STR+DOX
	86.54785156	93.96972656	3.149414063	87.91503906	81.27441406	5.053710938	7.788085938	89.28222656	1.703800786
	84.45159693	101.0893786	4.431790047	88.61104234	81.67863333	0.829413221	4.394652142	96.13765784	4.431951889
	90.01385216	104.5208412	3.097846619	95.65545901	77.92469462	5.717164085	3.173403853	90.61831004	3.106314715
AMP1CPR3STR2DOX1TMP1	STR+TMP	DOX+TMP	AMP+CPR+STR	AMP+CPR+DOX	AMP+CPR+TMP	AMP+STR+DOX	AMP+STR+TMP	AMP+DOX+TMP	CPR+STR+DOX
	49.20321186	83.61816406	22.94921875	95.33691406	90.45410156	3.41796875	7.983398438	77.56347656	17.0249017
	54.03576607	76.7269126	4.827927705	90.59173063	91.18593711	3.83758356	0.631344392	80.68828918	54.28160544
	57.08424157	85.17818914	3.903790455	91.82722579	95.65545901	4.911220249	42.99206649	82.76035764	32.27358903
AMP1CPR3STR2DOX1TMP2	STR+TMP	DOX+TMP	AMP+CPR+STR	AMP+CPR+DOX	AMP+CPR+TMP	AMP+STR+DOX	AMP+STR+TMP	AMP+DOX+TMP	CPR+STR+DOX
	63.03891291	84.20410156	22.94921875	95.33691406	85.96191406	3.41796875	9.66796875	76.00097656	17.0249017
	54.80908652	80.49022035	4.827927705	90.59173063	86.43228522	3.83758356	1.02748205	90.3936618	54.28160544
	73.08629311	87.79750661	3.903790455	91.82722579	93.2376275	4.911220249	1.322251606	86.99156278	32.27358903
AMP1CPR3STR2DOX1TMP3	STR+TMP	DOX+TMP	AMP+CPR+STR	AMP+CPR+DOX	AMP+CPR+TMP	AMP+STR+DOX	AMP+STR+TMP	AMP+DOX+TMP	CPR+STR+DOX
	69.75911056	94.36035156	22.94921875	95.33691406	81.27441406	3.41796875	8.374023437	85.96191406	17.0249017
	54.71242146	99.10869027	4.827927705	90.59173063	81.67863333	3.83758356	4.592720971	95.54345135	54.28160544
	71.65021157	109.1550183	3.903790455	91.82722579	77.92469462	4.911220249	2.165974059	93.2376275	32.27358903
AMP1CPR3STR2DOX2TMP1	STR+TMP	DOX+TMP	AMP+CPR+STR	AMP+CPR+DOX	AMP+CPR+TMP	AMP+STR+DOX	AMP+STR+TMP	AMP+DOX+TMP	CPR+STR+DOX
	49.20321186	83.81347656	22.94921875	93.18847656	90.45410156	3.22265625	7.983398438	83.42285156	56.47444299
	54.03576607	84.84773459	4.827927705	85.83807873	91.18593711	0.074275811	0.631344392	91.18593711	56.92247353
	57.08424157	95.65545901	3.903790455	92.02871175	95.65545901	4.508248331	42.99206649	89.61088024	67.87224734
AMP1CPR3STR2DOX2TMP2	STR+TMP	DOX+TMP	AMP+CPR+STR	AMP+CPR+DOX	AMP+CPR+TMP	AMP+STR+DOX	AMP+STR+TMP	AMP+DOX+TMP	CPR+STR+DOX
	63.03891291	88.69628906	22.94921875	93.18847656	85.96191406	3.22265625	9.66796875	78.93066406	56.47444299
	54.80908652	83.26318396	4.827927705	85.83807873	86.43228522	0.074275811	1.02748205	91.38400594	56.92247353
	73.08629311	96.6628888	3.903790455	92.02871175	93.2376275	4.508248331	1.322251606	97.46883264	67.87224734
AMP1CPR3STR2DOX2TMP3	STR+TMP	DOX+TMP	AMP+CPR+STR	AMP+CPR+DOX	AMP+CPR+TMP	AMP+STR+DOX	AMP+STR+TMP	AMP+DOX+TMP	CPR+STR+DOX
	69.75911056	94.55566406	22.94921875	93.18847656	81.27441406	3.22265625	8.374023437	74.82910156	56.47444299
	54.71242146	86.03614756	4.827927705	85.83807873	81.67863333	0.074275811	4.592720971	87.02649171	56.92247353
	71.65021157	97.46883264	3.903790455	92.02871175	77.92469462	4.508248331	2.165974059	97.6703186	67.87224734
AMP1CPR3STR2DOX3TMP1	STR+TMP	DOX+TMP	AMP+CPR+STR	AMP+CPR+DOX	AMP+CPR+TMP	AMP+STR+DOX	AMP+STR+TMP	AMP+DOX+TMP	CPR+STR+DOX
	49.20321186	79.51660156	22.94921875	87.91503906	90.45410156	7.397460938	7.983398438	84.59472656	66.74967235
	54.03576607	82.47090864	4.827927705	88.61104234	91.18593711	0.433275563	0.631344392	94.75117603	67.79971238
	57.08424157	84.37224531	3.903790455	95.65545901	95.65545901	3.903790455	42.99206649	100.0881501	70.20562928
AMP1CPR3STR2DOX3TMP2	STR+TMP	DOX+TMP	AMP+CPR+STR	AMP+CPR+DOX	AMP+CPR+TMP	AMP+STR+DOX	AMP+STR+TMP	AMP+DOX+TMP	CPR+STR+DOX
	63.03891291	77.56347656	22.94921875	87.91503906	85.96191406	7.397460938	9.66796875	86.15722656	66.74967235

AMP1CPR1STR3DOX2TMP3	CPR+STR+TMP	CPR+DOX+TMP	STR+DOX+TMP	AMP+CPR+STR+DOX	AMP+CPR+STR+TMP	AMP+CPR+DOX+TMP	AMP+STR+DOX+TMP	CPR+STR+DOX+TMP	AMP+CPR+STR+DOX+TMP
	50.80566406	96.89941406	70.90432503	19.18945313	33.88671875	75.02441406	21.53320313	52.39257813	33.83789063
	64.10002476	101.683585	67.81278599	19.05174548	18.94033177	87.22456053	37.95493934	48.45258727	32.60708096
	62.37249717	93.64059942	70.73064022	16.03072661	17.60483566	87.39453469	25.09759476	44.44024682	28.72434202
AMP1CPR1STR3DOX3TMP1	CPR+STR+TMP	CPR+DOX+TMP	STR+DOX+TMP	AMP+CPR+STR+DOX	AMP+CPR+STR+TMP	AMP+CPR+DOX+TMP	AMP+STR+DOX+TMP	CPR+STR+DOX+TMP	AMP+CPR+STR+DOX+TMP
	60.40039063	75.41503906	72.16251638	13.72070313	18.99414063	82.05566406	43.60351563	46.19140625	34.27734375
	51.81975737	80.68828918	74.50647143	14.78088636	2.104481307	77.71725675	54.39465214	42.70859124	0.631344392
	53.91008689	99.68517819	78.66413884	19.41820929	25.66427402	84.77521723	42.42538723	42.02241531	35.73857197
AMP1CPR1STR3DOX3TMP2	CPR+STR+TMP	CPR+DOX+TMP	STR+DOX+TMP	AMP+CPR+STR+DOX	AMP+CPR+STR+TMP	AMP+CPR+DOX+TMP	AMP+STR+DOX+TMP	CPR+STR+DOX+TMP	AMP+CPR+STR+DOX+TMP
	56.10351563	93.96972656	72.37221494	13.72070313	40.91796875	89.67285156	37.35351563	59.61914063	36.81640625
	53.20623917	81.08442684	85.38371029	14.78088636	1.708343649	87.02649171	54.98885863	65.68457539	41.82966081
	48.26848004	96.86437476	97.09785621	19.41820929	15.42626873	87.59602065	41.01498552	53.50711497	4.747512908
AMP1CPR1STR3DOX3TMP3	CPR+STR+TMP	CPR+DOX+TMP	STR+DOX+TMP	AMP+CPR+STR+DOX	AMP+CPR+STR+TMP	AMP+CPR+DOX+TMP	AMP+STR+DOX+TMP	CPR+STR+DOX+TMP	AMP+CPR+STR+DOX+TMP
	50.80566406	94.16503906	66.50065531	13.72070313	33.88671875	91.43066406	35.37597656	58.44726563	49.26757813
	64.10002476	99.10869027	73.25140541	14.78088636	18.94033177	97.32607081	42.90666006	63.90195593	36.2837336
	62.37249717	103.5134114	80.064168	19.41820929	17.60483566	96.05843093	41.41795744	57.73832011	57.49905553
AMP1CPR2STR1DOX1TMP1	CPR+STR+TMP	CPR+DOX+TMP	STR+DOX+TMP	AMP+CPR+STR+DOX	AMP+CPR+STR+TMP	AMP+CPR+DOX+TMP	AMP+STR+DOX+TMP	CPR+STR+DOX+TMP	AMP+CPR+STR+DOX+TMP
	4.360716492	78.85114268	59.59472656	2.63671875	7.397460938	92.99316406	6.811523438	4.928968499	5.834960938
	3.866602223	76.1720638	50.97796484	7.006684823	3.045308245	95.93958901	3.602376826	6.959884002	1.856895271
	1.487370176	81.90793691	59.18650044	0.919279688	2.971917894	94.44654326	4.583805566	4.359533273	4.785291525
AMP1CPR2STR1DOX1TMP2	CPR+STR+TMP	CPR+DOX+TMP	STR+DOX+TMP	AMP+CPR+STR+DOX	AMP+CPR+STR+TMP	AMP+CPR+DOX+TMP	AMP+STR+DOX+TMP	CPR+STR+DOX+TMP	AMP+CPR+STR+DOX+TMP
	3.15009265	82.80420012	46.72851563	2.63671875	6.93359375	87.13378906	9.741210938	4.731315627	7.592773437
	3.866602223	85.25857902	56.77147809	7.006684823	3.83758356	82.27283981	2.847239416	6.186563557	0.829413221
	2.513142711	80.26670086	51.69374134	0.919279688	2.568945977	84.37224531	5.994207279	4.56468778	5.188263443
AMP1CPR2STR1DOX1TMP3	CPR+STR+TMP	CPR+DOX+TMP	STR+DOX+TMP	AMP+CPR+STR+DOX	AMP+CPR+STR+TMP	AMP+CPR+DOX+TMP	AMP+STR+DOX+TMP	CPR+STR+DOX+TMP	AMP+CPR+STR+DOX+TMP
	2.359481161	107.3131563	48.68164063	2.63671875	8.764648437	73.07128906	10.52246094	4.731315627	5.834960937
	4.059932334	104.2049299	1.262688784	7.006684823	4.431790047	86.82842288	3.045308245	5.799903335	1.819757366
	4.33388896	106.5264778	62.57398313	0.919279688	5.188263443	83.56630147	6.195693238	3.744069753	4.583805566
AMP1CPR2STR1DOX2TMP1	CPR+STR+TMP	CPR+DOX+TMP	STR+DOX+TMP	AMP+CPR+STR+DOX	AMP+CPR+STR+TMP	AMP+CPR+DOX+TMP	AMP+STR+DOX+TMP	CPR+STR+DOX+TMP	AMP+CPR+STR+DOX+TMP
	4.360716492	95.84928968	53.34472656	3.881835938	7.397460938	79.32128906	6.225585938	3.940704138	7.91015625
	3.866602223	81.19864669	56.37534043	2.698687794	3.045308245	79.10373855	4.233721218	6.379893668	1.658826442
	1.487370176	96.2687524	54.71603073	1.158544264	2.971917894	81.75292784	5.79272132	3.538915246	3.173403853
AMP1CPR2STR1DOX2TMP2	CPR+STR+TMP	CPR+DOX+TMP	STR+DOX+TMP	AMP+CPR+STR+DOX	AMP+CPR+STR+TMP	AMP+CPR+DOX+TMP	AMP+STR+DOX+TMP	CPR+STR+DOX+TMP	AMP+CPR+STR+DOX+TMP
	3.15009265	94.66337245	50.24414063	3.881835938	6.93359375	88.11035156	4.858398438	2.779493515	10.13183594
	3.866602223	80.42532624	62.11933647	2.698687794	3.83758356	91.7801436	1.856895271	6.959884002	-0.074275811
	2.513142711	98.52545198	64.58884272	1.158544264	2.568945977	96.46140285	4.583805566	1.282215669	5.918650044
AMP1CPR2STR1DOX2TMP3	CPR+STR+TMP	CPR+DOX+TMP	STR+DOX+TMP	AMP+CPR+STR+DOX	AMP+CPR+STR+TMP	AMP+CPR+DOX+TMP	AMP+STR+DOX+TMP	CPR+STR+DOX+TMP	AMP+CPR+STR+DOX+TMP
	2.359481161	91.89623224	50.83007813	3.881835938	8.764648437	86.35253906	7.006835938	1.766522545	9.545898438
	4.059932334	86.99855002	54.39465214	2.698687794	4.431790047	88.21490468	2.896756623	6.766553891	1.510274821
	4.33388896	92.98628029	51.8952273	1.158544264	5.188263443	95.65545901	3.77786173	10.16797025	2.291902783
AMP1CPR2STR1DOX3TMP1	CPR+STR+TMP	CPR+DOX+TMP	STR+DOX+TMP	AMP+CPR+STR+DOX	AMP+CPR+STR+TMP	AMP+CPR+DOX+TMP	AMP+STR+DOX+TMP	CPR+STR+DOX+TMP	AMP+CPR+STR+DOX+TMP
	4.360716492	94.07041384	43.01757813	7.202148438	7.397460938	95.33691406	19.70214844	1.964175417	4.272460938
	3.866602223	84.67858869	60.13864818	3.045308245	3.045308245	93.76083189	2.896756623	6.57322378	-1.460757613
	1.487370176	91.34504424	47.90328674	1.888930865	2.971917894	97.87180456	2.770431936	14.06590589	0.755572346
AMP1CPR2STR1DOX3TMP2	CPR+STR+TMP	CPR+DOX+TMP	STR+DOX+TMP	AMP+CPR+STR+DOX	AMP+CPR+STR+TMP	AMP+CPR+DOX+TMP	AMP+STR+DOX+TMP	CPR+STR+DOX+TMP	AMP+CPR+STR+DOX+TMP
	3.15009265	94.46571958	47.29003906	7.202148438	6.93359375	91.23535156	46.58203125	0.975911056	7.397460938
	3.866602223	85.06524891	51.57217133	3.045308245	3.83758356	90.59173063	1.708343649	7.733204447	0.037137905
	2.513142711	89.08834466	70.26822818	1.888930865	2.568945977	90.01385216	2.694874701	12.42466983	2.732653318
AMP1CPR2STR1DOX3TMP3	CPR+STR+TMP	CPR+DOX+TMP	STR+DOX+TMP	AMP+CPR+STR+DOX	AMP+CPR+STR+TMP	AMP+CPR+DOX+TMP	AMP+STR+DOX+TMP	CPR+STR+DOX+TMP	AMP+CPR+STR+DOX+TMP
	2.359481161	95.25633107	52.00195313	7.202148438	8.764648437	90.84472656	4.98046875	7.91846819	5.639648438
	4.059932334	97.0517158	42.42386729	3.045308245	4.431790047	90.19559297	14.24857638	3.28661189	5.731616737
	4.33388896	99.34607001	48.26848004	1.888930865	5.188263443	88.40196449	2.934139277	2.28234389	1.687444906
AMP1CPR2STR2DOX1TMP1	CPR+STR+TMP	CPR+DOX+TMP	STR+DOX+TMP	AMP+CPR+STR+DOX	AMP+CPR+STR+TMP	AMP+CPR+DOX+TMP	AMP+STR+DOX+TMP	CPR+STR+DOX+TMP	AMP+CPR+STR+DOX+TMP
	4.360716492	78.85114268	3.347745522	2.978515625	9.741210938	92.99316406	7.397460937	2.977146387	8.764648437
	1.739971	76.1720638	2.319961334	5.224065363	2.847239416	95.93958901	4.035652389	6.186563557	33.90690765
	10.98858828	81.90793691	47.50609052	2.165974059	5.188263443	94.44654326	7.60609495	12.83497884	7.001637073
AMP1CPR2STR2DOX1TMP2	CPR+STR+TMP	CPR+DOX+TMP	STR+DOX+TMP	AMP+CPR+STR+DOX	AMP+CPR+STR+TMP	AMP+CPR+DOX+TMP	AMP+STR+DOX+TMP	CPR+STR+DOX+TMP	AMP+CPR+STR+DOX+TMP
	12.46448425	82.80420012	5.126621371	2.978515625	8.374023438	87.13378906	46.97265625	3.940704138	7.202148438
	4.253262446	85.25857902	1.739971	5.224065363	-1.547412726	82.27283981	4.431790047	6.766553891	1.856895271
	45.45454545	80.26670086	1.051416848	2.165974059	6.397179197	84.37224531	8.613524745	5.769970509	6.800151114
AMP1CPR2STR2DOX1TMP3	CPR+STR+TMP	CPR+DOX+TMP	STR+DOX+TMP	AMP+CPR+STR+DOX	AMP+CPR+STR+TMP	AMP+CPR+DOX+TMP	AMP+STR+DOX+TMP	CPR+STR+DOX+TMP	AMP+CPR+STR+DOX+TMP
	56.73872761	107.3131563	5.719579988	2.978515625	7.202148438	73.07128906	9.741210937	4.138357011	10.20507813
	3.612856452	104.2049299	3.093281779	5.224065363	3.83758356	86.82842288	2.500618965	9.473175447	5.583065115
	65.76484165	106.5264778	1.051416848	2.165974059	6.800151114	83.56630147	7.60609495	5.975125016	6.397179197
AMP1CPR2STR2DOX2TMP1	CPR+STR+TMP	CPR+DOX+TMP	STR+DOX+TMP	AMP+CPR+STR+DOX	AMP+CPR+STR+TMP	AMP+CPR+DOX+TMP	AMP+STR+DOX+TMP	CPR+STR+DOX+TMP	AMP+CPR+STR+DOX+TMP
	4.360716492	95.84928968	5.324274243	5.95703125	9.741210938	79.32128906	6.616210938	2.557134033	5.56640625
	1.739971	81.19864669	39.37892702	3.441445902	2.847239416	79.10373855	3.441445902	6.959884002	0.829413221
	10.98858828	96.2687524	15.00192332	2.367460018	5.188263443	81.75292784	6.800151114	4.33388896	3.979347689
AMP1CPR2STR2DOX2TMP2	CPR+STR+TMP	CPR+DOX+TMP	STR+DOX+TMP	AMP+CPR+STR+DOX	AMP+CPR+STR+TMP	AMP+CPR+DOX+TMP	AMP+STR+DOX+TMP	CPR+STR+DOX+TMP	AMP+CPR+STR+DOX+TMP
	12.46448425	94.66337245	12.43977764	5.95703125	8.374023438	88.11035156	2.514648438	47.84434836	8.764648438
	4.253262446	80.42532624	25.0362494	3.441445902	-1.547412726	91.7801436	3.639514731	15.21266312	3.094825452
	45.45454545	98.52545198	24.02872163	2.367460018	6.397179197	96.46140285	5.591235361	13.56584177	3.979347689
AMP1CPR2STR2DOX2TMP3	CPR+STR+TMP	CPR+DOX+TMP	STR+DOX+TMP	AMP+CPR+STR+DOX	AMP+CPR+STR+TMP	AMP+CPR+DOX+TMP	AMP+STR+DOX+TMP	CPR+STR+DOX+TMP	AMP+CPR+STR+DOX+TMP
	56.73872761	91.89623224	13.23038913	5.95703125	7.202148438	86.35253906	7.592773437	11.87152563	6.811523438
	3.612856452	86.99855002	24.45625906	3.441445902	3.83758356	88.21490468	2.104481307	10.63315611	0.717999505
	65.76484165	92.98628029	23.0029491	2.367460018	6.800151114	95.65545901	7.807580909	57.96897038	2.367460018
AMP1CPR2STR2DOX3TMP1	CPR+STR+TMP	CPR+DOX+TMP	STR+DOX+TMP	AMP+CPR+STR+DOX	AMP+CPR+STR+TMP	AMP+CPR+DOX+TMP	AMP+STR+DOX+TMP	CPR+STR+DOX+TMP	AMP+CPR+STR+DOX+TMP
	4.360716492	94.07041384	12.0444719	11.23046875	9.741210938	95.33691406	9.936523437	3.174799259	9.741210938
	1.739971	84.67858869	12.27646206	14.89230007	2.847239416	93.76083189	2.896756623	8.506524891	1.510274821
	10.98858828	91.34504424	28.13181177	5.188263443	5.188263443	97.87180456	3.979347689	42.78753686	13.6128951
AMP1CPR2STR2DOX3TMP2	CPR+STR+TMP	CPR+DOX+TMP	STR+DOX+TMP	AMP+CPR+STR+DOX	AMP+CPR+STR+TMP	AMP+CPR+DOX+TMP	AMP+STR+DOX+TMP	CPR+STR+DOX+TMP	AMP+CPR+STR+DOX+TMP
	12.46448425	94.46571958	52.40271773	11.23046875	8.374023438	91.23535156	14.30664063	6.93020383	6.225585938
	4.253262446	85.06524891	51.71580474	14.89230007	-1.547412726	90.59173063	2.896756623	5.606573224	-0.866551127
	45.45454545	89.08834466	59.52045134	5.188263443	6.397179197	90.01385216	2.694874701	24.52878574	14.41883894
AMP1CPR2STR2DOX3TMP3	CPR+STR+TMP	CPR+DOX+TMP	STR+DOX+TMP	AMP+CPR+STR+DOX	AMP+CPR+STR+TMP	AMP+CPR+DOX+TMP	AMP+STR+DOX+TMP	CPR+STR+DOX+TMP	AMP+CPR+STR+DOX+TMP
	56.73872761	95.25633107	45.28721433	11.23046875	7.202148438	90.84472656	5.883789063	58.51760346	56.73828125
	3.612856452	97.0517158	52.13871435	14.89230007	3.83758356	90.19559297	4.543203763	0.193330111	2.364446645
	65.76484165	99.34607001	64.239005	5.188263443	6.800151114	88.40196449	2.304495655	6.475189127	1.485958947
AMP1CPR2STR3DOX1TMP1	CPR+STR+TMP	CPR+DOX+TMP	STR+DOX+TMP	AMP+CPR+STR+DOX	AMP+CPR+STR+TMP	AMP+CPR+DOX+TMP	AMP+STR+DOX+TMP	CPR+STR+DOX+TMP	AMP+CPR+STR+DOX+TMP
	42.91537986	78.85114268	49.97378768	13.57421875	34.61914063	92.99316406	34.81445313	41.32180358	45.36132813
	58.86901885	76.1720638	53.15727546	2.649170587	28.64570438	95.93958901	53.00817034	5.413243113	3.639514731
	53.16066162	81.90793691	57.83870497	20.02266717	18.61226546	94.44654326	47.4625362	43.40300038	32.14960332
AMP1CPR2STR3DOX1TMP2	CPR+STR+TMP	CPR+DOX+TMP	STR+DOX+TMP	AMP+CPR+STR+DOX	AMP+CPR+STR+TMP	AMP+CPR+DOX+TMP	AMP+STR+DOX+TMP	CPR+STR+DOX+TMP	AMP+CPR+STR+DOX+TMP
	52.79802347	82.80420012	42.1887287	13.57421875	26.07421875	87.13378906	32.86132813	48.04200124	34.08203125
	34.35234413	85.25857902	37.33821415	2.649170587	33.31270116	82.27283981	29.4379797	46.91880135	4.035652389
	53.77612514	80.26670086	54.44071751	20.02266717	34.32817026	84.37224531	18.20929354	52.95550712	6.800151114
AMP1CPR2STR3DOX1TMP3	CPR+STR+TMP	CPR+DOX+TMP	STR+DOX+TMP	AMP+CPR+STR+DOX	AMP+CPR+STR+TMP	AMP+CPR+DOX+TMP	AMP+STR+DOX+TMP	CPR+STR+DOX+TMP	AMP+CPR+STR+DOX+TMP
	53.19332922	107.3131563	56.68414155	13.57421875	15.86914063	73.07128906	39.11132813	44.49660284	28.41796875
	58.09569841	104.2049299	58.59589489	2.649170587	13.90195593	86.82842288	27.56870513	59.09859836	10.53478584
	52.95550712	106.5264778	45.93845705	20.02266717	18.20929354	83.56630147	7.203123032	50.17309912	5.389749402
AMP1CPR2STR3DOX2TMP1	CPR+STR+TMP	CPR+DOX+TMP	STR+DOX+TMP	AMP+CPR+STR+DOX	AMP+CPR+STR+TMP	AMP+CPR+DOX+TMP	AMP+STR+DOX+TMP	CPR+STR+DOX+TMP	AMP+CPR+STR+DOX+TMP
	42.91537986	95.84928968	51.86107471	33.03222656	34.61914063	79.32128906	41.06445313	61.49474985	35.00976563
	58.86901885	81.19864669	51.69303177	19.33646942	28.64570438	79.10373855	50.23520673	54.42242629	36.17231988
	53.16066162	96.2687524	49.20519177	30.77698023	18.61226546	81.75292784	45.0447047	72.03487627	24.65684423
AMP1CPR2STR3DOX2TMP2	CPR+STR+TMP	CPR+DOX+TMP	STR+DOX+TMP	AMP+CPR+STR+DOX	AMP+CPR+STR+TMP	AMP+CPR+DOX+TMP	AMP+STR+DOX+TMP	CPR+STR+DOX+TMP	AMP+CPR+STR+DOX+TMP
	52.79802347	94.66337245	59.82961992	33.03222656	26.07421875	88.11035156	33.05664063	52.99567634	34.86328125
	34.35234413	80.42532624	61.73355994	19.33646942	33.31270116	91.7801436	46.66996781	37.63895602	2.017826195
	53.77612514	98.52545198	62.50546886	30.77698023	34.32817026	96.46140285	37.35045964	61.98230542	12.76917265
AMP1CPR2STR3DOX2TMP3	CPR+STR+TMP	CPR+DOX+TMP	STR+DOX+TMP	AMP+CPR+STR+DOX	AMP+CPR+STR+TMP	AMP+CPR+DOX+TMP	AMP+STR+DOX+TMP	CPR+STR+DOX+TMP	AMP+CPR+STR+DOX+TMP
	53.19332922	91.89623224	70.90432503	33.03222656	15.86914063	86.35253906	21.53320313	51.01914762	31.49414063
	58.09569841	86.99855002	67.81278599	19.33646942	13.90195593	88.21490468	37.95493934	51.1358144	33.99356276
	52.95550712	92.98628029	70.73064022	30.77698023	18.20929354	95.65545901	25.09759476	56.85344275	42.02241531
AMP1CPR2STR3DOX3TMP1	CPR+STR+TMP	CPR+DOX+TMP	STR+DOX+TMP	AMP+CPR+STR+DOX	AMP+CPR+STR+TMP	AMP+CPR+DOX+TMP	AMP+STR+DOX+TMP	CPR+STR+DOX+TMP	AMP+CPR+STR+DOX+TMP
	42.91537986	94.07041384	72.16251638	18.79882813	34.61914063	95.33691406	43.60351563	52.6003706	32.08007813
	58.86901885	84.67858869	74.50647143	16.36543699	28.64570438	93.76083189	54.39465214	42.66553891	29.04184204
	53.16066162	91.34504424	78.66413884	25.46278806	18.61226546	97.87180456	42.42538723	48.64726247	50.28333963
AMP1CPR2STR3DOX3TMP2	CPR+STR+TMP	CPR+DOX+TMP	STR+DOX+TMP	AMP+CPR+STR+DOX	AMP+CPR+STR+TMP	AMP+CPR+DOX+TMP	AMP+STR+DOX+TMP	CPR+STR+DOX+TMP	AMP+CPR+STR+DOX+TMP
	52.79802347	94.46571958	72.37221494	18.79882813	26.07421875	91.23535156	37.35351563	54.37924645	39.69726563
	34.35234413	85.06524891	85.38371029	16.36543699	33.31270116	90.59173063	54.98885863	65.05558241	48.05644962
	53.77612514	89.08834466	97.09785621	25.46278806	34.32817026	90.01385216	41.01498552	51.10911655	41.01498552
AMP1CPR2STR3DOX3TMP3	CPR+STR+TMP	CPR+DOX+TMP	STR+DOX+TMP	AMP+CPR+STR+DOX	AMP+CPR+STR+TMP	AMP+CPR+DOX+TMP	AMP+STR+DOX+TMP	CPR+STR+DOX+TMP	AMP+CPR+STR+DOX+TMP
	53.19332922	95.25633107	66.50065531	18.79882813	15.86914063	90.84472656	35.37597656	50.03088326	26.41601563
	58.09569841	97.0517158	73.25140541	16.36543699	13.90195593	90.19559297	42.90666006	52.68245529	34.98390691
	52.95550712	99.34607001	80.064168	25.46278806	18.20929354	88.40196449	41.41795744	60.95653289	26.67170382
AMP1CPR3STR1DOX1TMP1	CPR+STR+TMP	CPR+DOX+TMP	STR+DOX+TMP	AMP+CPR+STR+DOX	AMP+CPR+STR+TMP	AMP+CPR+DOX+TMP	AMP+STR+DOX+TMP	CPR+STR+DOX+TMP	AMP+CPR+STR+DOX+TMP
	2.752293578	90.40629096	59.59472656	12.52441406	6.030273438	72.68066406	6.811523438	8.204456094	10.71777344
	6.314550922	86.22042097	50.97796484	1.819757366	1.621688537	75.53849963	3.602376826	9.452215976	0.829413221
	1.006270964	82.86422634	59.18650044	3.903790455	4.508248331	88.60345045	4.583805566	4.404258422	5.188263443
AMP1CPR3STR1DOX1TMP2	CPR+STR+TMP	CPR+DOX+TMP	STR+DOX+TMP	AMP+CPR+STR+DOX	AMP+CPR+STR+TMP	AMP+CPR+DOX+TMP	AMP+STR+DOX+TMP	CPR+STR+DOX+TMP	AMP+CPR+STR+DOX+TMP
	60.01310616	89.35779817	46.72851563	12.52441406	5.834960938	80.68847656	9.741210938	6.526867628	11.69433594
	8.406327625	94.16917244	56.77147809	1.819757366	1.225550879	81.08442684	2.847239416	8.615505295	4.035652389
	1.706285548	85.43094648	51.69374134	3.903790455	4.180833648	88.20047853	5.994207279	4.170920228	3.576375771
AMP1CPR3STR1DOX1TMP3	CPR+STR+TMP	CPR+DOX+TMP	STR+DOX+TMP	AMP+CPR+STR+DOX	AMP+CPR+STR+TMP	AMP+CPR+DOX+TMP	AMP+STR+DOX+TMP	CPR+STR+DOX+TMP	AMP+CPR+STR+DOX+TMP
	7.798165138	110.9567497	48.68164063	12.52441406	9.155273438	78.93066406	10.52246094	5.688073394	12.86621094
	6.732906262	107.138188	1.262688784	1.819757366	0.235206734	86.82842288	3.045308245	7.360439273	1.262688784
	53.74070293	112.498177	62.57398313	3.903790455	4.986777484	88.80493641	6.195693238	4.870934811	2.568945977
AMP1CPR3STR1DOX2TMP1	CPR+STR+TMP	CPR+DOX+TMP	STR+DOX+TMP	AMP+CPR+STR+DOX	AMP+CPR+STR+TMP	AMP+CPR+DOX+TMP	AMP+STR+DOX+TMP	CPR+STR+DOX+TMP	AMP+CPR+STR+DOX+TMP
	2.752293578	103.197903	53.34472656	4.39453125	6.030273438	84.00878906	6.225585938	7.994757536	14.03808594
	6.314550922	92.70492875	56.37534043	3.441445902	1.621688537	78.1133944	4.233721218	7.151261603	2.215895024
	1.006270964	105.7313694	54.71603073	3.702304496	4.508248331	82.35738572	5.79272132	26.67347236	1.9644881
AMP1CPR3STR1DOX2TMP2	CPR+STR+TMP	CPR+DOX+TMP	STR+DOX+TMP	AMP+CPR+STR+DOX	AMP+CPR+STR+TMP	AMP+CPR+DOX+TMP	AMP+STR+DOX+TMP	CPR+STR+DOX+TMP	AMP+CPR+STR+DOX+TMP
	60.01310616	104.6657929	50.24414063	4.39453125	5.834960938	86.93847656	4.858398438	6.107470511	9.155273438
	8.406327625	105.0464113	62.11933647	3.441445902	1.225550879	93.36469423	1.856895271	6.942083933	1.819757366
	1.706285548	110.6314715	64.58884272	3.702304496	4.180833648	98.07329052	4.583805566	58.87414321	0.113335852
AMP1CPR3STR1DOX2TMP3	CPR+STR+TMP	CPR+DOX+TMP	STR+DOX+TMP	AMP+CPR+STR+DOX	AMP+CPR+STR+TMP	AMP+CPR+DOX+TMP	AMP+STR+DOX+TMP	CPR+STR+DOX+TMP	AMP+CPR+STR+DOX+TMP
	7.798165138	101.7300131	50.83007813	4.39453125	9.155273438	80.29785156	7.006835938	5.268676278	15.40527344
	6.732906262	102.1179239	54.39465214	3.441445902	0.235206734	81.87670215	2.896756623	5.26866257	2.302550136
	53.74070293	103.1646493	51.8952273	3.702304496	4.986777484	95.85694497	3.77786173	1.370861893	0.352600428
AMP1CPR3STR1DOX3TMP1	CPR+STR+TMP	CPR+DOX+TMP	STR+DOX+TMP	AMP+CPR+STR+DOX	AMP+CPR+STR+TMP	AMP+CPR+DOX+TMP	AMP+STR+DOX+TMP	CPR+STR+DOX+TMP	AMP+CPR+STR+DOX+TMP
	2.752293578	97.7457405	43.01757813	5.17578125	6.030273438	84.39941406	19.70214844	23.34207077	10.32714844
	6.314550922	93.12328409	60.13864818	1.262688784	1.621688537	79.10373855	2.896756623	5.477840241	1.906412478
	1.006270964	94.29779787	47.90328674	4.508248331	4.508248331	92.02871175	2.770431936	5.570949395	-0.251857449
AMP1CPR3STR1DOX3TMP2	CPR+STR+TMP	CPR+DOX+TMP	STR+DOX+TMP	AMP+CPR+STR+DOX	AMP+CPR+STR+TMP	AMP+CPR+DOX+TMP	AMP+STR+DOX+TMP	CPR+STR+DOX+TMP	AMP+CPR+STR+DOX+TMP
	60.01310616	97.7457405	47.29003906	5.17578125	5.834960938	76.00097656	46.58203125	23.76146789	14.81933594
	8.406327625	93.33246176	51.57217133	1.262688784	1.225550879	76.33077494	1.708343649	4.418878285	0.916068334
	1.706285548	99.19789996	70.26822818	4.508248331	4.180833648	80.94698401	2.694874701	1.137523698	-0.125928724
AMP1CPR3STR1DOX3TMP3	CPR+STR+TMP	CPR+DOX+TMP	STR+DOX+TMP	AMP+CPR+STR+DOX	AMP+CPR+STR+TMP	AMP+CPR+DOX+TMP	AMP+STR+DOX+TMP	CPR+STR+DOX+TMP	AMP+CPR+STR+DOX+TMP
	7.798165138	93.55176933	52.00195313	5.17578125	9.155273438	84.39941406	4.98046875	5.307994758	3.344726563
	6.732906262	98.77108119	42.42386729	1.262688784	0.235206734	97.72220847	14.24857638	7.556543339	2.562515474
	53.74070293	105.264693	48.26848004	4.508248331	4.986777484	82.55887168	2.934139277	5.3376112	-0.125928724
AMP1CPR3STR2DOX1TMP1	CPR+STR+TMP	CPR+DOX+TMP	STR+DOX+TMP	AMP+CPR+STR+DOX	AMP+CPR+STR+TMP	AMP+CPR+DOX+TMP	AMP+STR+DOX+TMP	CPR+STR+DOX+TMP	AMP+CPR+STR+DOX+TMP
	28.55832241	90.40629096	3.347745522	48.33984375	23.92578125	72.68066406	7.397460937	8.427260813	8.764648437
	5.0594849	86.22042097	2.319961334	2.847239416	2.253032929	75.53849963	4.035652389	7.569616943	1.225550879
	62.14087793	82.86422634	47.50609052	8.53796751	4.70973429	88.60345045	7.60609495	10.10646055	3.979347689
AMP1CPR3STR2DOX1TMP2	CPR+STR+TMP	CPR+DOX+TMP	STR+DOX+TMP	AMP+CPR+STR+DOX	AMP+CPR+STR+TMP	AMP+CPR+DOX+TMP	AMP+STR+DOX+TMP	CPR+STR+DOX+TMP	AMP+CPR+STR+DOX+TMP
	58.12581913	89.35779817	5.126621371	48.33984375	7.397460938	80.68847656	46.97265625	3.171690695	9.936523437
	5.26866257	94.16917244	1.739971	2.847239416	2.017826195	81.08442684	4.431790047	8.406327625	1.262688784
	39.04039667	85.43094648	1.051416848	8.53796751	15.99294799	88.20047853	8.613524745	4.870934811	3.173403853
AMP1CPR3STR2DOX1TMP3	CPR+STR+TMP	CPR+DOX+TMP	STR+DOX+TMP	AMP+CPR+STR+DOX	AMP+CPR+STR+TMP	AMP+CPR+DOX+TMP	AMP+STR+DOX+TMP	CPR+STR+DOX+TMP	AMP+CPR+STR+DOX+TMP
	67.77195282	110.9567497	5.719579988	48.33984375	6.811523438	78.93066406	9.741210937	4.639580603	12.28027344
	23.32331024	107.138188	3.093281779	2.847239416	0.235206734	86.82842288	2.500618965	28.34357432	3.243377074
	43.24048418	112.498177	1.051416848	8.53796751	3.77786173	88.80493641	7.60609495	6.737640368	2.165974059
AMP1CPR3STR2DOX2TMP1	CPR+STR+TMP	CPR+DOX+TMP	STR+DOX+TMP	AMP+CPR+STR+DOX	AMP+CPR+STR+TMP	AMP+CPR+DOX+TMP	AMP+STR+DOX+TMP	CPR+STR+DOX+TMP	AMP+CPR+STR+DOX+TMP
	28.55832241	103.197903	5.324274243	5.56640625	23.92578125	84.00878906	6.616210938	5.478374836	9.545898437
	5.0594849	92.70492875	39.37892702	5.620203021	2.253032929	78.1133944	3.441445902	6.105373251	3.008170339
	62.14087793	105.7313694	15.00192332	14.9855182	4.70973429	82.35738572	6.800151114	7.306402217	0.51630777
AMP1CPR3STR2DOX2TMP2	CPR+STR+TMP	CPR+DOX+TMP	STR+DOX+TMP	AMP+CPR+STR+DOX	AMP+CPR+STR+TMP	AMP+CPR+DOX+TMP	AMP+STR+DOX+TMP	CPR+STR+DOX+TMP	AMP+CPR+STR+DOX+TMP
	58.12581913	104.6657929	12.43977764	5.56640625	7.397460938	86.93847656	2.514648438	28.34862385	8.959960938
	5.26866257	105.0464113	25.0362494	5.620203021	2.017826195	93.36469423	3.639514731	5.0594849	3.998514484
	39.04039667	110.6314715	24.02872163	14.9855182	15.99294799	98.07329052	5.591235361	4.870934811	-0.088150107
AMP1CPR3STR2DOX2TMP3	CPR+STR+TMP	CPR+DOX+TMP	STR+DOX+TMP	AMP+CPR+STR+DOX	AMP+CPR+STR+TMP	AMP+CPR+DOX+TMP	AMP+STR+DOX+TMP	CPR+STR+DOX+TMP	AMP+CPR+STR+DOX+TMP
	67.77195282	101.7300131	13.23038913	5.56640625	6.811523438	80.29785156	7.592773437	32.54259502	1.342773437
	23.32331024	102.1179239	24.45625906	5.620203021	0.235206734	81.87670215	2.104481307	5.255588966	4.988858628
	43.24048418	103.1646493	23.0029491	14.9855182	3.77786173	95.85694497	7.807580909	20.37334111	0.554086387
AMP1CPR3STR2DOX3TMP1	CPR+STR+TMP	CPR+DOX+TMP	STR+DOX+TMP	AMP+CPR+STR+DOX	AMP+CPR+STR+TMP	AMP+CPR+DOX+TMP	AMP+STR+DOX+TMP	CPR+STR+DOX+TMP	AMP+CPR+STR+DOX+TMP
	28.55832241	97.7457405	12.0444719	27.78320313	23.92578125	84.39941406	9.936523437	31.07470511	10.91308594
	5.0594849	93.12328409	12.27646206	3.441445902	2.253032929	79.10373855	2.896756623	7.765721009	2.413963852
	62.14087793	94.29779787	28.13181177	39.36531923	4.70973429	92.02871175	3.979347689	69.84103835	-0.251857449
AMP1CPR3STR2DOX3TMP2	CPR+STR+TMP	CPR+DOX+TMP	STR+DOX+TMP	AMP+CPR+STR+DOX	AMP+CPR+STR+TMP	AMP+CPR+DOX+TMP	AMP+STR+DOX+TMP	CPR+STR+DOX+TMP	AMP+CPR+STR+DOX+TMP
	58.12581913	97.7457405	52.40271773	27.78320313	7.397460938	76.00097656	14.30664063	44.94102228	9.545898438

	AMP	CPR	STR	DOX	TMP	AMP+CPR	AMP+STR	AMP+DOX	AMP+TMP	CPR+STR	CPR+DOX
	94.15696955	102.3271016	50.16916385	101.2874474	93.36469423	99.50482793	4.827927705	86.63035405	87.42262936	56.92247353	105.4647666
	93.03614154	104.3313402	59.93076035	100.0881501	93.84208538	89.8123662	3.097846619	86.79007682	89.20790832	64.13883623	113.8982062
AMP1CPR3STR2DOX3TMP3	AMP	CPR	STR	DOX	TMP	AMP+CPR	AMP+STR	AMP+DOX	AMP+TMP	CPR+STR	CPR+DOX
	88.89160156	86.21231979	46.67078443	99.04785156	88.11035156	88.30566406	4.907226563	91.23535156	83.03222656	58.15203145	103.8269987
	94.15696955	102.3271016	50.16916385	101.2874474	91.97821243	99.50482793	4.827927705	86.63035405	86.23421639	56.92247353	105.4647666
	93.03614154	104.3313402	59.93076035	100.0881501	96.6628888	89.8123662	3.097846619	86.79007682	89.00642236	64.13883623	113.8982062
AMP1CPR3STR3DOX1TMP1	AMP	CPR	STR	DOX	TMP	AMP+CPR	AMP+STR	AMP+DOX	AMP+TMP	CPR+STR	CPR+DOX
	88.89160156	86.21231979	93.55176933	103.9306641	83.22753906	88.30566406	34.79003906	90.06347656	88.50097656	72.5819135	101.5203145
	94.15696955	102.3271016	85.80206563	102.6739292	87.62069819	99.50482793	29.38846249	90.59173063	84.45159693	68.64949667	106.0922996
	93.03614154	104.3313402	94.53113607	98.47626244	85.17818914	89.8123662	40.64979222	89.40939428	80.54401209	74.46405133	103.8646638
AMP1CPR3STR3DOX1TMP2	AMP	CPR	STR	DOX	TMP	AMP+CPR	AMP+STR	AMP+DOX	AMP+TMP	CPR+STR	CPR+DOX
	88.89160156	86.21231979	93.55176933	103.9306641	91.04003906	88.30566406	34.79003906	90.06347656	91.82128906	72.5819135	101.5203145
	94.15696955	102.3271016	85.80206563	102.6739292	93.36469423	99.50482793	29.38846249	90.59173063	87.42262936	68.64949667	106.0922996
	93.03614154	104.3313402	94.53113607	98.47626244	93.84208538	89.8123662	40.64979222	89.40939428	89.20790832	74.46405133	103.8646638
AMP1CPR3STR3DOX1TMP3	AMP	CPR	STR	DOX	TMP	AMP+CPR	AMP+STR	AMP+DOX	AMP+TMP	CPR+STR	CPR+DOX
	88.89160156	86.21231979	93.55176933	103.9306641	88.11035156	88.30566406	34.79003906	90.06347656	83.03222656	72.5819135	101.5203145
	94.15696955	102.3271016	85.80206563	102.6739292	91.97821243	99.50482793	29.38846249	90.59173063	86.23421639	68.64949667	106.0922996
	93.03614154	104.3313402	94.53113607	98.47626244	96.6628888	89.8123662	40.64979222	89.40939428	89.00642236	74.46405133	103.8646638

[illegible]

AMP2CPR2STR2DOX1TMP2	AMP	CPR	STR	DOX	TMP	AMP+CPR	AMP+STR	AMP+DOX	AMP+TMP	CPR+STR	CPR+DOX	CPR+TMP	STR+DOX	STR+TMP	DOX+TMP	AMP+CPR+STR	AMP+CPR+DOX	AMP+CPR+TMP	AMP+STR+DOX	AMP+STR+TMP	AMP+DOX+TMP	CPR+STR+DOX	CPR+STR+TMP	CPR+DOX+TMP	STR+DOX+TMP	AMP+CPR+STR+DOX	AMP+CPR+STR+TMP	AMP+CPR+DOX+TMP	AMP+STR+DOX+TMP	CPR+STR+DOX+TMP	AMP+CPR+STR+DOX+TMP
	91.69857937	104.1507103	46.67078443	103.9306641	91.04003906	90.31500926	6.337245213	101.9765287	104.3483632	46.26312539	104.3483632	90.90796788	45.07720815	63.03891291	84.20410156	4.731315627	98.81408277	92.29153799	4.731315627	9.277331686	85.76899321	53.77393453	12.46448425	82.80420012	5.126621371	5.941939469	4.138357011	80.23471279	10.85855466	3.940704138	4.731315627
	86.6118898	81.77863702	50.16916385	102.6739292	93.36469423	86.99855002	1.449975834	88.3518608	102.0782987	12.69937168	103.8182697	91.25181247	3.28661189	54.80908652	80.49022035	3.673272112	97.0517158	89.70517158	3.28661189	6.379893668	83.90526825	31.06573224	4.253262446	85.25857902	1.739971	3.866602223	3.866602223	91.05848236	6.766553891	6.766553891	0.966650556
	92.37081677	103.2440056	59.93076035	98.47626244	93.84208538	94.83267086	3.513270932	95.44813438	96.47390691	46.39056289	102.2182331	99.1409155	6.475189127	73.08629311	87.79750661	4.769842287	100.3718425	95.24297987	2.897807411	5.359661495	90.52442621	45.45454545	45.45454545	80.26670086	1.051416848	3.397871522	3.94922426	83.95948198	42.78753686	5.769970509	6.590588537
AMP2CPR2STR2DOX1TMP3	AMP	CPR	STR	DOX	TMP	AMP+CPR	AMP+STR	AMP+DOX	AMP+TMP	CPR+STR	CPR+DOX	CPR+TMP	STR+DOX	STR+TMP	DOX+TMP	AMP+CPR+STR	AMP+CPR+DOX	AMP+CPR+TMP	AMP+STR+DOX	AMP+STR+TMP	AMP+DOX+TMP	CPR+STR+DOX	CPR+STR+TMP	CPR+DOX+TMP	STR+DOX+TMP	AMP+CPR+STR+DOX	AMP+CPR+STR+TMP	AMP+CPR+DOX+TMP	AMP+STR+DOX+TMP	CPR+STR+DOX+TMP	AMP+CPR+STR+DOX+TMP
	91.69857937	104.1507103	46.67078443	103.9306641	88.11035156	90.31500926	6.337245213	101.9765287	101.1859172	46.26312539	104.3483632	90.90796788	45.07720815	69.75911056	94.36035156	4.731315627	98.81408277	88.14082767	4.731315627	8.486720198	97.82581841	53.77393453	56.73872761	107.3131563	5.719579988	5.941939469	2.952439778	98.61642989	6.312538604	4.138357011	4.360716492
	86.6118898	81.77863702	50.16916385	102.6739292	91.97821243	86.99855002	1.449975834	88.3518608	98.79168681	12.69937168	103.8182697	91.25181247	3.28661189	54.71242146	99.10869027	3.673272112	97.0517158	81.00531658	3.28661189	5.02658289	99.56500725	31.06573224	3.612856452	104.2049299	3.093281779	3.866602223	2.319961334	82.16529725	5.799903335	9.473175447	1.933301112
	92.37081677	103.2440056	59.93076035	98.47626244	96.6628888	94.83267086	3.513270932	95.44813438	99.34607001	46.39056289	102.2182331	97.70483395	6.475189127	71.65021157	109.1550183	4.769842287	100.3718425	84.78010001	2.897807411	5.359661495	93.60174381	45.45454545	65.76484165	106.5264778	1.051416848	3.397871522	4.56468778	86.21618156	7.231696371	5.975125016	7.411206565
AMP2CPR2STR2DOX2TMP1	AMP	CPR	STR	DOX	TMP	AMP+CPR	AMP+STR	AMP+DOX	AMP+TMP	CPR+STR	CPR+DOX	CPR+TMP	STR+DOX	STR+TMP	DOX+TMP	AMP+CPR+STR	AMP+CPR+DOX	AMP+CPR+TMP	AMP+STR+DOX	AMP+STR+TMP	AMP+DOX+TMP	CPR+STR+DOX	CPR+STR+TMP	CPR+DOX+TMP	STR+DOX+TMP	AMP+CPR+STR+DOX	AMP+CPR+STR+TMP	AMP+CPR+DOX+TMP	AMP+STR+DOX+TMP	CPR+STR+DOX+TMP	AMP+CPR+STR+DOX+TMP
	91.69857937	104.1507103	46.67078443	100.6103516	83.22753906	90.31500926	6.337245213	101.3835701	91.89623224	46.26312539	98.22112415	89.3267449	61.6553428	49.20321186	83.81347656	4.731315627	100.5929586	99.40704138	6.510191476	4.928968499	88.14082767	51.61210624	4.360716492	95.84928968	5.324274243	3.545398394	5.719579988	78.65348981	41.32180358	2.557134033	2.198888203
	86.6118898	81.77863702	50.16916385	102.2777915	87.62069819	86.99855002	1.449975834	98.40502658	96.27839536	12.69937168	98.21169647	92.41179314	48.5258579	54.03576607	84.84773459	3.673272112	93.18511358	98.59835669	4.639922668	4.446592557	97.0517158	51.75205413	1.739971	81.19864669	39.37892702	4.833252779	4.446592557	88.73852102	6.57322378	6.959884002	3.479942001
	92.37081677	103.2440056	59.93076035	98.07329052	85.17818914	94.83267086	3.513270932	97.90998846	85.60071804	46.39056289	101.1924606	87.4471086	67.13681241	57.08424157	95.65545901	4.769842287	99.55122452	104.6800872	3.308116425	2.28234389	89.90896269	54.59674317	10.98858828	96.2687524	15.00192332	3.538915246	2.987562508	79.85639185	6.821387357	4.33388896	8.231824593
AMP2CPR2STR2DOX2TMP2	AMP	CPR	STR	DOX	TMP	AMP+CPR	AMP+STR	AMP+DOX	AMP+TMP	CPR+STR	CPR+DOX	CPR+TMP	STR+DOX	STR+TMP	DOX+TMP	AMP+CPR+STR	AMP+CPR+DOX	AMP+CPR+TMP	AMP+STR+DOX	AMP+STR+TMP	AMP+DOX+TMP	CPR+STR+DOX	CPR+STR+TMP	CPR+DOX+TMP	STR+DOX+TMP	AMP+CPR+STR+DOX	AMP+CPR+STR+TMP	AMP+CPR+DOX+TMP	AMP+STR+DOX+TMP	CPR+STR+DOX+TMP	AMP+CPR+STR+DOX+TMP
	91.69857937	104.1507103	46.67078443	100.6103516	91.04003906	90.31500926	6.337245213	101.3835701	104.3483632	46.26312539	98.22112415	90.90796788	61.6553428	63.03891291	88.69628906	4.731315627	100.5929586	92.29153799	6.510191476	9.277331686	87.54786905	51.61210624	12.46448425	94.66337245	12.43977764	3.545398394	4.138357011	93.47745522	5.324274243	47.84434836	1.000617665
	86.6118898	81.77863702	50.16916385	102.2777915	93.36469423	86.99855002	1.449975834	98.40502658	102.0782987	12.69937168	98.21169647	91.25181247	48.5258579	54.80908652	83.26318396	3.673272112	93.18511358	89.70517158	4.639922668	6.379893668	101.6916385	51.75205413	4.253262446	80.42532624	25.0362494	4.833252779	3.866602223	95.50507492	6.57322378	15.21266312	1.159980667
	92.37081677	103.2440056	59.93076035	98.07329052	93.84208538	94.83267086	3.513270932	97.90998846	96.47390691	46.39056289	101.1924606	99.1409155	67.13681241	73.08629311	96.6628888	4.769842287	99.55122452	95.24297987	3.308116425	5.359661495	94.83267086	54.59674317	45.45454545	98.52545198	24.02872163	3.538915246	3.94922426	95.85844339	6.064880113	13.56584177	0.23079882
AMP2CPR2STR2DOX2TMP3	AMP	CPR	STR	DOX	TMP	AMP+CPR	AMP+STR	AMP+DOX	AMP+TMP	CPR+STR	CPR+DOX	CPR+TMP	STR+DOX	STR+TMP	DOX+TMP	AMP+CPR+STR	AMP+CPR+DOX	AMP+CPR+TMP	AMP+STR+DOX	AMP+STR+TMP	AMP+DOX+TMP	CPR+STR+DOX	CPR+STR+TMP	CPR+DOX+TMP	STR+DOX+TMP	AMP+CPR+STR+DOX	AMP+CPR+STR+TMP	AMP+CPR+DOX+TMP	AMP+STR+DOX+TMP	CPR+STR+DOX+TMP	AMP+CPR+STR+DOX+TMP
	91.69857937	104.1507103	46.67078443	100.6103516	88.11035156	90.31500926	6.337245213	101.3835701	101.1859172	46.26312539	98.22112415	90.90796788	61.6553428	69.75911056	94.55566406	4.731315627	100.5929586	88.14082767	6.510191476	8.486720198	86.75725757	51.61210624	56.73872761	91.89623224	13.23038913	3.545398394	2.952439778	93.67510809	13.45274861	11.87152563	2.38418777
	86.6118898	81.77863702	50.16916385	102.2777915	91.97821243	86.99855002	1.449975834	98.40502658	98.79168681	12.69937168	98.21169647	91.25181247	48.5258579	54.71242146	86.03614756	3.673272112	93.18511358	81.00531658	4.639922668	5.02658289	94.73175447	51.75205413	3.612856452	86.99855002	24.45625906	4.833252779	2.319961334	92.21846303	7.346544224	10.63315611	0.386660222
	92.37081677	103.2440056	59.93076035	98.07329052	96.6628888	94.83267086	3.513270932	97.90998846	99.34607001	46.39056289	101.1924606	97.70483395	67.13681241	71.65021157	97.46883264	4.769842287	99.55122452	84.78010001	3.308116425	5.359661495	98.73060649	54.59674317	65.76484165	92.98628029	23.0029491	3.538915246	4.56468778	91.34504424	2.782408001	57.96897038	-1.000128222
AMP2CPR2STR2DOX3TMP1	AMP	CPR	STR	DOX	TMP	AMP+CPR	AMP+STR	AMP+DOX	AMP+TMP	CPR+STR	CPR+DOX	CPR+TMP	STR+DOX	STR+TMP	DOX+TMP	AMP+CPR+STR	AMP+CPR+DOX	AMP+CPR+TMP	AMP+STR+DOX	AMP+STR+TMP	AMP+DOX+TMP	CPR+STR+DOX	CPR+STR+TMP	CPR+DOX+TMP	STR+DOX+TMP	AMP+CPR+STR+DOX	AMP+CPR+STR+TMP	AMP+CPR+DOX+TMP	AMP+STR+DOX+TMP	CPR+STR+DOX+TMP	AMP+CPR+STR+DOX+TMP
	91.69857937	104.1507103	46.67078443	99.04785156	83.22753906	90.31500926	6.337245213	101.9765287	91.89623224	46.26312539	102.3718345	89.3267449	62.44595429	49.20321186	79.51660156	4.731315627	103.7554046	99.40704138	5.91723286	4.928968499	95.0586782	44.69425571	4.360716492	94.07041384	12.0444719	5.91723286	5.719579988	99.60469426	23.13773935	3.174799259	1.198270537
	86.6118898	81.77863702	50.16916385	101.2874474	87.62069819	86.99855002	1.449975834	98.98501692	96.27839536	12.69937168	102.0782987	92.41179314	54.22909618	54.03576607	82.47090864	3.673272112	98.98501692	98.59835669	4.253262446	4.446592557	91.44514258	58.67568874	1.739971	84.67858869	12.27646206	4.639922668	4.446592557	94.73175447	5.606573224	8.506524891	2.452875785
	92.37081677	103.2440056	59.93076035	100.0881501	85.17818914	94.83267086	3.513270932	95.03782536	85.60071804	46.39056289	103.4491601	87.4471086	58.93063213	57.08424157	84.37224531	4.769842287	102.8336966	104.6800872	4.33388896	2.28234389	99.96153353	54.80189768	10.98858828	91.34504424	28.13181177	20.63085011	2.987562508	91.96050776	16.73291448	42.78753686	0.256443134
AMP2CPR2STR2DOX3TMP2	AMP	CPR	STR	DOX	TMP	AMP+CPR	AMP+STR	AMP+DOX	AMP+TMP	CPR+STR	CPR+DOX	CPR+TMP	STR+DOX	STR+TMP	DOX+TMP	AMP+CPR+STR	AMP+CPR+DOX	AMP+CPR+TMP	AMP+STR+DOX	AMP+STR+TMP	AMP+DOX+TMP	CPR+STR+DOX	CPR+STR+TMP	CPR+DOX+TMP	STR+DOX+TMP	AMP+CPR+STR+DOX	AMP+CPR+STR+TMP	AMP+CPR+DOX+TMP	AMP+STR+DOX+TMP	CPR+STR+DOX+TMP	AMP+CPR+STR+DOX+TMP
	91.69857937	104.1507103	46.67078443	99.04785156	91.04003906	90.31500926	6.337245213	101.9765287	104.3483632	46.26312539	102.3718345	90.90796788	62.44595429	63.03891291	77.56347656	4.731315627	103.7554046	92.29153799	5.91723286	9.277331686	94.26806671	44.69425571	12.46448425	94.46571958	52.40271773	5.91723286	4.138357011	93.87276096	7.91846819	6.93020383	1.000617665
	86.6118898	81.77863702	50.16916385	101.2874474	93.36469423	86.99855002	1.449975834	98.98501692	102.0782987	12.69937168	102.0782987	91.25181247	54.22909618	54.80908652	85.83807873	3.673272112	98.98501692	89.70517158	4.253262446	6.379893668	97.63170614	58.67568874	4.253262446	85.06524891	51.71580474	4.639922668	3.866602223	92.99178347	1.933301112	5.606573224	2.066215563
	92.37081677	103.2440056	59.93076035	100.0881501	93.84208538	94.83267086	3.513270932	95.03782536	96.47390691	46.39056289	103.4491601	99.1409155	58.93063213	73.08629311	95.65545901	4.769842287	102.8336966	95.24297987	4.33388896	5.359661495	96.67906142	54.80189768	45.45454545	89.08834466	59.52045134	20.63085011	3.94922426	90.72958072	4.833953071	24.52878574	8.642133607
AMP2CPR2STR2DOX3TMP3	AMP	CPR	STR	DOX	TMP	AMP+CPR	AMP+STR	AMP+DOX	AMP+TMP	CPR+STR	CPR+DOX	CPR+TMP	STR+DOX	STR+TMP	DOX+TMP	AMP+CPR+STR	AMP+CPR+DOX	AMP+CPR+TMP	AMP+STR+DOX	AMP+STR+TMP	AMP+DOX+TMP	CPR+STR+DOX	CPR+STR+TMP	CPR+DOX+TMP	STR+DOX+TMP	AMP+CPR+STR+DOX	AMP+CPR+STR+TMP	AMP+CPR+DOX+TMP	AMP+STR+DOX+TMP	CPR+STR+DOX+TMP	AMP+CPR+STR+DOX+TMP
	91.69857937	104.1507103	46.67078443	99.04785156	88.11035156	90.31500926	6.337245213	101.9765287	101.1859172	46.26312539	102.3718345	90.90796788	62.44595429	69.75911056	93.96972656	4.731315627	103.7554046	88.14082767	5.91723286	8.486720198	91.10562075	44.69425571	56.73872761	95.25633107	45.28721433	5.91723286	2.952439778	93.67510809	4.953675108	58.51760346	3.965410747
	86.6118898	81.77863702	50.16916385	101.2874474	91.97821243	86.99855002	1.449975834	98.98501692	98.79168681	12.69937168	102.0782987	91.25181247	54.22909618	54.71242146	101.0893786	3.673272112	98.98501692	81.00531658	4.253262446	5.02658289	92.41179314	58.67568874	3.612856452	97.0517158	52.13871435	4.639922668	2.319961334	91.44514258	3.588690188	0.193330111	5.02658289
	92.37081677	103.2440056	59.93076035	100.0881501	96.6628888	94.83267086	3.513270932	95.03782536	99.34607001	46.39056289	103.4491601	97.70483395	58.93063213	71.65021157	104.5208412	4.769842287	102.8336966	84.78010001	4.33388896	5.359661495	81.70278241	54.80189768	65.76484165	99.34607001	64.239005	20.63085011	4.56468778	96.2687524	6.61623285	6.475189127	8.872932427
AMP2CPR2STR3DOX1TMP1	AMP	CPR	STR	DOX	TMP	AMP+CPR	AMP+STR	AMP+DOX	AMP+TMP	CPR+STR	CPR+DOX	CPR+TMP	STR+DOX	STR+TMP	DOX+TMP	AMP+CPR+STR	AMP+CPR+DOX	AMP+CPR+TMP	AMP+STR+DOX	AMP+STR+TMP	AMP+DOX+TMP	CPR+STR+DOX	CPR+STR+TMP	CPR+DOX+TMP	STR+DOX+TMP	AMP+CPR+STR+DOX	AMP+CPR+STR+TMP	AMP+CPR+DOX+TMP	AMP+STR+DOX+TMP	CPR+STR+DOX+TMP	AMP+CPR+STR+DOX+TMP
	91.69857937	104.1507103	93.55176933	103.9306641	83.22753906	90.31500926	55.3304509	101.9765287	91.89623224	61.26003706	104.3483632	89.3267449	83.48623853	89.56749672	83.61816406	19.75293391	98.81408277	99.40704138	34.41630636	47.06609018	85.17603459	48.44966028	42.91537986	78.85114268	49.97378768	45.67016677	32.0444719	94.26806671	45.4848672	41.32180358	36.38048178
	86.6118898	81.77863702	85.80206563	102.6739292	87.62069819	86.99855002	35.57274045	88.3518608	96.27839536	58.48235863	103.8182697	92.41179314	93.33246176	96.88848215	76.7269126	17.33929435	97.0517158	98.59835669	20.23924601	45.33591107	93.95843403	54.42242629	58.86901885	76.1720638	53.15727546	4.446592557	43.59594007	94.73175447	11.59980667	5.413243113	12.89270179
	92.37081677	103.2440056	94.53113607	98.47626244	85.17818914	94.83267086	68.36773945	95.44813438	85.60071804	66.52134889	102.2182331	87.4471086	92.43109231	96.63117982	85.17818914	38.00487242	100.3718425	104.6800872	47.30093602	45.56994486	87.85741762	58.2895243	53.16066162	81.90793691	57.83870497	34.08129247	43.92870881	94.83267086	49.87818951	43.40300038	55.4173612
AMP2CPR2STR3DOX1TMP2	AMP	CPR	STR	DOX	TMP	AMP+CPR	AMP+STR	AMP+DOX	AMP+TMP	CPR+STR	CPR+DOX	CPR+TMP	STR+DOX	STR+TMP	DOX+TMP	AMP+CPR+STR	AMP+CPR+DOX	AMP+CPR+TMP	AMP+STR+DOX	AMP+STR+TMP	AMP+DOX+TMP	CPR+STR+DOX	CPR+STR+TMP	CPR+DOX+TMP	STR+DOX+TMP	AMP+CPR+STR+DOX	AMP+CPR+STR+TMP	AMP+CPR+DOX+TMP	AMP+STR+DOX+TMP	CPR+STR+DOX+TMP	AMP+CPR+STR+DOX+TMP
	91.69857937	104.1507103	93.55176933	103.9306641	91.04003906	90.31500926	55.3304509	101.9765287	104.3483632	61.26003706	104.3483632	90.90796788	83.48623853	88.30930537	84.20410156	19.75293391	98.81408277	92.29153799	34.41630636	40.34589253	85.76899321	48.44966028	52.79802347	82.80420012	42.1887287	45.67016677	32.03211859	80.23471279	43.90364422	48.04200124	45.07720815
	86.6118898	81.77863702	85.80206563	102.6739292	93.36469423	86.99855002	35.57274045	88.3518608	102.0782987	58.48235863	103.8182697	91.25181247	93.33246176	92.91410642	80.49022035	17.33929435	97.0517158	89.70517158	20.23924601	30.4857419	83.90526825	54.42242629	34.35234413	85.25857902	37.33821415	4.446592557	4.639922668	91.05848236	34.35234413	46.91880135	2.029966167
	92.37081677	103.2440056	94.53113607	98.47626244	93.84208538	94.83267086	68.36773945	95.44813438	96.47390691	66.52134889	102.2182331	99.1409155	92.43109231	96.86451801	87.79750661	38.00487242	100.3718425	95.24297987	47.30093602	49.26272599	90.52442621	58.2895243	53.77612514	80.26670086	54.44071751	34.08129247	18.98961405	83.95948198	37.24836518	52.95550712	24.32363123
AMP2CPR2STR3DOX1TMP3	AMP	CPR	STR	DOX	TMP	AMP+CPR	AMP+STR	AMP+DOX	AMP+TMP	CPR+STR	CPR+DOX	CPR+TMP	STR+DOX	STR+TMP	DOX+TMP	AMP+CPR+STR	AMP+CPR+DOX	AMP+CPR+TMP	AMP+STR+DOX	AMP+STR+TMP	AMP+DOX+TMP	CPR+STR+DOX	CPR+STR+TMP	CPR+DOX+TMP	STR+DOX+TMP	AMP+CPR+STR+DOX	AMP+CPR+STR+TMP	AMP+CPR+DOX+TMP	AMP+STR+DOX+TMP	CPR+STR+DOX+TMP	AMP+CPR+STR+DOX+TMP
	91.69857937	104.1507103	93.55176933	103.9306641	88.11035156	90.31500926	55.3304509	101.9765287	101.1859172	61.26003706	104.3483632	90.90796788	83.48623853	94.60026212	94.36035156	19.75293391	98.81408277	88.14082767	34.41630636	43.07597282	97.82581841	48.44966028	53.19332922	107.3131563	56.68414155	45.67016677	12.06917851	98.61642989	35.58987029	44.49660284	20.96355775
	86.6118898	81.77863702	85.80206563	102.6739292	91.97821243	86.99855002	35.57274045	88.3518608	98.79168681	58.48235863	103.8182697	91.25181247	93.33246176	94.16917244	99.10869027	17.33929435	97.0517158	81.00531658	20.23924601	36.92605123	99.56500725	54.42242629	58.09569841	104.2049299	58.59589489	4.446592557	3.866602223	82.16529725	7.926534558	59.09859836	2.126631223
	92.37081677	103.2440056	94.53113607	98.47626244	96.6628888	94.83267086	68.36773945	95.44813438	99.34607001	66.52134889	102.2182331	97.70483395	92.43109231	93.1311069	109.1550183	38.00487242	100.3718425	84.78010001	47.30093602	42.72342608	93.60174381	58.2895243	52.95550712	106.5264778	45.93845705	34.08129247	25.05449417	86.21618156	20.63085011	50.17309912	5.975125016
AMP2CPR2STR3DOX2TMP1	AMP	CPR	STR	DOX	TMP	AMP+CPR	AMP+STR	AMP+DOX	AMP+TMP	CPR+STR	CPR+DOX	CPR+TMP	STR+DOX	STR+TMP	DOX+TMP	AMP+CPR+STR	AMP+CPR+DOX	AMP+CPR+TMP	AMP+STR+DOX	AMP+STR+TMP	AMP+DOX+TMP	CPR+STR+DOX	CPR+STR+TMP	CPR+DOX+TMP	STR+DOX+TMP	AMP+CPR+STR+DOX	AMP+CPR+STR+TMP	AMP+CPR+DOX+TMP	AMP+STR+DOX+TMP	CPR+STR+DOX+TMP	AMP+CPR+STR+DOX+TMP
	91.69857937	104.1507103	93.55176933	100.6103516	83.22753906	90.31500926	55.3304509	101.3835701	91.89623224	61.26003706	98.22112415	89.3267449	95.64875491	89.56749672	83.81347656	19.75293391	100.5929586	99.40704138	53.35392217	47.06609018	88.14082767	59.08585547	42.91537986	95.84928968	51.86107471	42.87831995	32.0444719	78.65348981	44.69425571	61.49474985	45.27486103
	86.6118898	81.77863702	85.80206563	102.2777915	87.62069819	86.99855002	35.57274045	98.40502658	96.27839536	58.48235863	98.21169647	92.41179314	96.67930448	96.88848215	84.84773459	17.33929435	93.18511358	98.59835669	45.62590623	45.33591107	97.0517158	53.26244563	58.86901885	81.19864669	51.69303177	36.44272595	43.59594007	88.73852102	48.62252296	54.42242629	27.97245046
	92.37081677	103.2440056	94.53113607	98.07329052	85.17818914	94.83267086	68.36773945	97.90998846	85.60071804	66.52134889	101.1924606	87.4471086	99.19789996	96.63117982	95.65545901	38.00487242	99.55122452	104.6800872	43.31324529	45.56994486	89.90896269	51.33991537	53.16066162	96.2687524	49.20519177	47.03167073	43.92870881	79.85639185	57.26375176	72.03487627	51.72458007
AMP2CPR2STR3DOX2TMP2	AMP	CPR	STR	DOX	TMP	AMP+CPR	AMP+STR	AMP+DOX	AMP+TMP	CPR+STR	CPR+DOX	CPR+TMP	STR+DOX	STR+TMP	DOX+TMP	AMP+CPR+STR	AMP+CPR+DOX	AMP+CPR+TMP	AMP+STR+DOX	AMP+STR+TMP	AMP+DOX+TMP	CPR+STR+DOX	CPR+STR+TMP	CPR+DOX+TMP	STR+DOX+TMP	AMP+CPR+STR+DOX	AMP+CPR+STR+TMP	AMP+CPR+DOX+TMP	AMP+STR+DOX+TMP	CPR+STR+DOX+TMP	AMP+CPR+STR+DOX+TMP
	91.69857937	104.1507103	93.55176933	100.6103516	91.04003906	90.31500926	55.3304509	101.3835701	104.3483632	61.26003706	98.22112415	90.90796788	95.64875491	88.30930537	88.69628906	19.75293391	100.5929586	92.29153799	53.35392217	40.34589253	87.54786905	59.08585547	52.79802347	94.66337245	59.82961992	42.87831995	32.03211859	93.47745522	49.0426189	52.99567634	25.31192094
	86.6118898	81.77863702	85.80206563	102.2777915	93.36469423	86.99855002	35.57274045	98.40502658	102.0782987	58.48235863	98.21169647	91.25181247	96.67930448	92.91410642	83.26318396	17.33929435	93.18511358	89.70517158	45.62590623	30.4857419	101.6916385	53.26244563	34.35234413	80.42532624	61.73355994	36.44272595	4.639922668	95.50507492	45.17883035	37.63895602	6.379893668
	92.37081677	103.2440056	94.53113607	98.07329052	93.84208538	94.83267086	68.36773945	97.90998846	96.47390691	66.52134889	101.1924606	99.1409155	99.19789996	96.86451801	96.6628888	38.00487242	99.55122452	95.24297987	43.31324529	49.26272599	94.83267086	51.33991537	53.77612514	98.52545198	62.50546886	47.03167073	18.98961405	95.85844339	34.58135658	61.98230542	2.28234389
AMP2CPR2STR3DOX2TMP3	AMP	CPR	STR	DOX	TMP	AMP+CPR	AMP+STR	AMP+DOX	AMP+TMP	CPR+STR	CPR+DOX	CPR+TMP	STR+DOX	STR+TMP	DOX+TMP	AMP+CPR+STR	AMP+CPR+DOX	AMP+CPR+TMP	AMP+STR+DOX	AMP+STR+TMP	AMP+DOX+TMP	CPR+STR+DOX	CPR+STR+TMP	CPR+DOX+TMP	STR+DOX+TMP	AMP+CPR+STR+DOX	AMP+CPR+STR+TMP	AMP+CPR+DOX+TMP	AMP+STR+DOX+TMP	CPR+STR+DOX+TMP	AMP+CPR+STR+DOX+TMP
	91.69857937	104.1507103	93.55176933	100.6103516	88.11035156	90.31500926	55.3304509	101.3835701	101.1859172	61.26003706	98.22112415	90.90796788	95.64875491	94.60026212	94.55566406	19.75293391	100.5929586	88.14082767	53.35392217	43.07597282	86.75725757	59.08585547	53.19332922	91.89623224	70.90432503	42.87831995	12.06917851	93.67510809	30.26559605	51.01914762	36.78814083
	86.6118898	81.77863702	85.80206563	102.2777915	91.97821243	86.99855002	35.57274045	98.40502658	98.79168681	58.48235863	98.21169647	91.25181247	96.67930448	94.16917244	86.03614756	17.33929435	93.18511358	81.00531658	45.62590623	36.92605123	94.73175447	53.26244563	58.09569841	86.99855002	67.81278599	36.44272595	3.866602223	92.21846303	31.9961334	51.1358144	39.34267762
	92.37081677	103.2440056	94.53113607	98.07329052	96.6628888	94.83267086	68.36773945	97.90998846	99.34607001	66.52134889	101.1924606	97.70483395	99.19789996	93.1311069	97.46883264	38.00487242	99.55122452	84.78010001	43.31324529	42.72342608	98.73060649	51.33991537	52.95550712	92.98628029	70.73064022	47.03167073	25.05449417	91.34504424	36.13283754	56.85344275	39.82561867
AMP2CPR2STR3DOX3TMP1	AMP	CPR	STR	DOX	TMP	AMP+CPR	AMP+STR	AMP+DOX	AMP+TMP	CPR+STR	CPR+DOX	CPR+TMP	STR+DOX	STR+TMP	DOX+TMP	AMP+CPR+STR	AMP+CPR+DOX	AMP+CPR+TMP	AMP+STR+DOX	AMP+STR+TMP	AMP+DOX+TMP	CPR+STR+DOX	CPR+STR+TMP	CPR+DOX+TMP	STR+DOX+TMP	AMP+CPR+STR+DOX	AMP+CPR+STR+TMP	AMP+CPR+DOX+TMP	AMP+STR+DOX+TMP	CPR+STR+DOX+TMP	AMP+CPR+STR+DOX+TMP
	91.69857937	104.1507103	93.55176933	99.04785156	83.22753906	90.31500926	55.3304509	101.9765287	91.89623224	61.26003706	102.3718345	89.3267449	91.03538663	89.56749672	79.51660156	19.75293391	103.7554046	99.40704138	37.18344657	47.06609018	95.0586782	38.56701668	42.91537986	94.07041384	72.16251638	34.21865349	32.0444719	99.60469426	35.20691785	52.6003706	49.24027177
	86.6118898	81.77863702	85.80206563	101.2874474	87.62069819	86.99855002	35.57274045	98.98501692	96.27839536	58.48235863	102.0782987	92.41179314	94.37835011	96.88848215	82.47090864	17.33929435	98.98501692	98.59835669	30.83615273	45.33591107	91.44514258	59.83566941	58.86901885	84.67858869	74.50647143	35.4760754	43.59594007	94.73175447	36.24939584	42.66553891	7.479458676
	92.37081677	103.2440056	94.53113607	100.0881501	85.17818914	94.83267086	68.36773945	95.03782536	85.60071804	66.52134889	103.4491601	87.4471086	96.86451801	96.63117982	84.37224531	38.00487242	102.8336966	104.6800872	37.7740736	45.56994486	99.96153353	53.16066162	53.16066162	91.34504424	78.66413884	41.05654571	43.92870881	91.96050776	49.05757148	48.64726247	54.59674317
AMP2CPR2STR3DOX3TMP2	AMP	CPR	STR	DOX	TMP	AMP+CPR	AMP+STR	AMP+DOX	AMP+TMP	CPR+STR	CPR+DOX	CPR+TMP	STR+DOX	STR+TMP	DOX+TMP	AMP+CPR+STR	AMP+CPR+DOX	AMP+CPR+TMP	AMP+STR+DOX	AMP+STR+TMP	AMP+DOX+TMP	CPR+STR+DOX	CPR+STR+TMP	CPR+DOX+TMP	STR+DOX+TMP	AMP+CPR+STR+DOX	AMP+CPR+STR+TMP	AMP+CPR+DOX+TMP	AMP+STR+DOX+TMP	CPR+STR+DOX+TMP	AMP+CPR+STR+DOX+TMP
	91.69857937	104.1507103	93.55176933	99.04785156	91.04003906	90.31500926	55.3304509	101.9765287	104.3483632	61.26003706	102.3718345	90.90796788	91.03538663	88.30930537	77.56347656	19.75293391	103.7554046	92.29153799	37.18344657	40.34589253	94.26806671	38.56701668	52.79802347	94.46571958	72.37221494	34.21865349	32.03211859	93.87276096	58.72760964	54.37924645	45.68252007
	86.6118898	81.77863702	85.80206563	101.2874474	93.36469423	86.99855002	35.57274045	98.98501692	102.0782987	58.48235863	102.0782987	91.25181247	94.37835011	92.91410642	85.83807873	17.33929435	98.98501692	89.70517158	30.83615273	30.4857419	97.63170614	59.83566941	34.35234413	85.06524891	85.38371029	35.4760754	4.639922668	92.99178347	38.37602707	65.05558241	49.20251329
	92.37081677	103.2440056	94.53113607	100.0881501	93.84208538	94.83267086	68.36773945	95.03782536	96.47390691	66.52134889	103.4491601	99.1409155	96.86451801	96.86451801	95.65545901	38.00487242	102.8336966	95.24297987	37.7740736	49.26272599	96.67906142	53.16066162	53.77612514	89.08834466	97.09785621	41.05654571	18.98961405	90.72958072	39.29991024	51.10911655	47.00602641
AMP2CPR2STR3DOX3TMP3	AMP	CPR	STR	DOX	TMP	AMP+CPR	AMP+STR	AMP+DOX	AMP+TMP	CPR+STR	CPR+DOX	CPR+TMP	STR+DOX	STR+TMP	DOX+TMP	AMP+CPR+STR	AMP+CPR+DOX	AMP+CPR+TMP	AMP+STR+DOX	AMP+STR+TMP	AMP+DOX+TMP	CPR+STR+DOX	CPR+STR+TMP	CPR+DOX+TMP	STR+DOX+TMP	AMP+CPR+STR+DOX	AMP+CPR+STR+TMP	AMP+CPR+DOX+TMP	AMP+STR+DOX+TMP	CPR+STR+DOX+TMP	AMP+CPR+STR+DOX+TMP
	91.69857937	104.1507103	93.55176933	99.04785156	88.11035156	90.31500926	55.3304509	101.9765287	101.1859172	61.26003706	102.3718345	90.90796788	91.03538663	94.60026212	93.96972656	19.75293391	103.7554046	88.14082767	37.18344657	43.07597282	91.10562075	38.56701668	53.19332922	95.25633107	66.50065531	34.21865349	12.06917851	93.67510809	32.63743051	50.03088326	35.60222359
	86.6118898	81.77863702	85.80206563	101.2874474	91.97821243	86.99855002	35.57274045	98.98501692	98.79168681	58.48235863	102.0782987	91.25181247	94.37835011	94.16917244	101.0893786	17.33929435	98.98501692	81.00531658	30.83615273	36.92605123	92.41179314	59.83566941	58.09569841	97.0517158	73.25140541	35.4760754	3.866602223	91.44514258	38.18269696	52.68245529	26.42580957
	92.37081677	103.2440056	94.53113607	100.0881501	96.6628888	94.83267086	68.36773945	95.03782536	99.34607001	66.52134889	103.4491601	97.70483395	96.86451801	93.1311069	104.5208412	38.00487242	102.8336966	84.78010001	37.7740736	42.72342608	81.70278241	53.16066162	52.95550712	99.34607001	80.064168	41.05654571	25.05449417	96.2687524	37.97922811	60.95653289	40.87703552
AMP2CPR3STR1DOX1TMP1	AMP	CPR	STR	DOX	TMP	AMP+CPR	AMP+STR	AMP+DOX	AMP+TMP	CPR+STR	CPR+DOX	CPR+TMP	STR+DOX	STR+TMP	DOX+TMP	AMP+CPR+STR	AMP+CPR+DOX	AMP+CPR+TMP	AMP+STR+DOX	AMP+STR+TMP	AMP+DOX+TMP	CPR+STR+DOX	CPR+STR+TMP	CPR+DOX+TMP	STR+DOX+TMP	AMP+CPR+STR+DOX	AMP+CPR+STR+TMP	AMP+CPR+DOX+TMP	AMP+STR+DOX+TMP	CPR+STR+DOX+TMP	AMP+CPR+STR+DOX+TMP
	91.69857937	86.21231979	97.48535156	103.9306641	83.22753906	91.30327363	12.26683138	101.9765287	91.89623224	58.33551769	101.5203145	95.64875491	84.20410156	81.07910156	83.61816406	3.15009265	90.11735639	100.1976529	4.533662755	6.905497221	85.17603459	1.323722149	2.752293578	90.40629096	59.59472656	3.940704138	8.906732551	86.75725757	9.870290303	8.204456094	3.545398394
	86.6118898	102.3271016	89.60138648	102.6739292	87.62069819	84.67858869	2.223296278	88.3518608	96.27839536	45.28696562	106.0922996	97.09765982	80.29215152	82.66897747	76.7269126	3.673272112	94.73175447	99.95166747	4.059932334	4.639922668	93.95843403	3.804418878	6.314550922	86.22042097	50.97796484	2.899951667	2.513291445	82.74528758	5.799903335	9.452215976	2.029966167
	92.37081677	104.3313402	86.58859086	98.47626244	85.17818914	95.65328888	1.872034876	95.44813438	85.60071804	59.10748141	103.8646638	108.0647513	67.85039668	82.15589976	85.17818914	1.961789973	103.2440056	97.29452494	1.692524683	5.154506988	87.85741762	20.14000292	1.006270964	82.86422634	59.18650044	2.923451725	4.974996794	87.4471086	3.333760739	4.404258422	6.38543403
AMP2CPR3STR1DOX1TMP2	AMP	CPR	STR	DOX	TMP	AMP+CPR	AMP+STR	AMP+DOX	AMP+TMP	CPR+STR	CPR+DOX	CPR+TMP	STR+DOX	STR+TMP	DOX+TMP	AMP+CPR+STR	AMP+CPR+DOX	AMP+CPR+TMP	AMP+STR+DOX	AMP+STR+TMP	AMP+DOX+TMP	CPR+STR+DOX	CPR+STR+TMP	CPR+DOX+TMP	STR+DOX+TMP	AMP+CPR+STR+DOX	AMP+CPR+STR+TMP	AMP+CPR+DOX+TMP	AMP+STR+DOX+TMP	CPR+STR+DOX+TMP	AMP+CPR+STR+DOX+TMP
	91.69857937	86.21231979	97.48535156	103.9306641	91.04003906	91.30327363	12.26683138	101.9765287	104.3483632	58.33551769	101.5203145	101.1009174	84.20410156	81.07910156	84.20410156	3.15009265	90.11735639	95.84928968	4.533662755	4.336009883	85.76899321	1.323722149	60.01310616	89.35779817	46.72851563	3.940704138	3.743051266	97.82581841	9.474984558	6.526867628	3.15009265
	86.6118898	102.3271016	89.60138648	102.6739292	93.36469423	84.67858869	2.223296278	88.3518608	102.0782987	45.28696562	106.0922996	103.1638123	80.29215152	81.08442684	80.49022035	3.673272112	94.73175447	90.28516191	4.059932334	7.346544224	83.90526825	3.804418878	8.406327625	94.16917244	56.77147809	2.899951667	2.899951667	84.09859836	5.799903335	8.615505295	1.643305945
	92.37081677	104.3313402	86.58859086	98.47626244	93.84208538	95.65328888	1.872034876	95.44813438	96.47390691	59.10748141	103.8646638	106.6647222	67.85039668	85.17818914	87.79750661	1.961789973	103.2440056	97.49967945	1.692524683	8.052314399	90.52442621	20.14000292	1.706285548	85.43094648	51.69374134	2.923451725	4.974996794	90.11411719	8.052314399	4.170920228	5.359661495
AMP2CPR3STR1DOX1TMP3	AMP	CPR	STR	DOX	TMP	AMP+CPR	AMP+STR	AMP+DOX	AMP+TMP	CPR+STR	CPR+DOX	CPR+TMP	STR+DOX	STR+TMP	DOX+TMP	AMP+CPR+STR	AMP+CPR+DOX	AMP+CPR+TMP	AMP+STR+DOX	AMP+STR+TMP	AMP+DOX+TMP	CPR+STR+DOX	CPR+STR+TMP	CPR+DOX+TMP	STR+DOX+TMP	AMP+CPR+STR+DOX	AMP+CPR+STR+TMP	AMP+CPR+DOX+TMP	AMP+STR+DOX+TMP	CPR+STR+DOX+TMP	AMP+CPR+STR+DOX+TMP
	91.69857937	86.21231979	97.48535156	103.9306641	88.11035156	91.30327363	12.26683138	101.9765287	101.1859172	58.33551769	101.5203145	100.2621232	84.20410156	86.54785156	94.36035156	3.15009265	90.11735639	88.14082767	4.533662755	7.893761581	97.82581841	1.323722149	7.798165138	110.9567497	48.68164063	3.940704138	3.15009265	88.93143916	8.091414453	5.688073394	3.347745522
	86.6118898	102.3271016	89.60138648	102.6739292	91.97821243	84.67858869	2.223296278	88.3518608	98.79168681	45.28696562	106.0922996	98.77108119	80.29215152	84.45159693	99.10869027	3.673272112	94.73175447	91.63847269	4.059932334	6.379893668	99.56500725	3.804418878	6.732906262	107.138188	1.262688784	2.899951667	3.28661189	92.02513291	7.539874335	7.360439273	1.933301112
	92.37081677	104.3313402	86.58859086	98.47626244	96.6628888	95.65328888	1.872034876	95.44813438	99.34607001	59.10748141	103.8646638	99.66457635	67.85039668	90.01385216	109.1550183	1.961789973	103.2440056	92.57597128	1.692524683	4.744197974	93.60174381	20.14000292	53.74070293	112.498177	62.57398313	2.923451725	5.975125016	101.3976151	5.385305808	4.870934811	5.975125016
AMP2CPR3STR1DOX2TMP1	AMP	CPR	STR	DOX	TMP	AMP+CPR	AMP+STR	AMP+DOX	AMP+TMP	CPR+STR	CPR+DOX	CPR+TMP	STR+DOX	STR+TMP	DOX+TMP	AMP+CPR+STR	AMP+CPR+DOX	AMP+CPR+TMP	AMP+STR+DOX	AMP+STR+TMP	AMP+DOX+TMP	CPR+STR+DOX	CPR+STR+TMP	CPR+DOX+TMP	STR+DOX+TMP	AMP+CPR+STR+DOX	AMP+CPR+STR+TMP	AMP+CPR+DOX+TMP	AMP+STR+DOX+TMP	CPR+STR+DOX+TMP	AMP+CPR+STR+DOX+TMP
	91.69857937	86.21231979	97.48535156	100.6103516	83.22753906	91.30327363	12.26683138	101.3835701	91.89623224	58.33551769	96.69724771	95.64875491	90.45410156	81.07910156	83.81347656	3.15009265	94.26806671	100.1976529	31.83446572	6.905497221	88.14082767	2.346002621	2.752293578	103.197903	53.34472656	6.312538604	8.906732551	80.43236566	7.103150093	7.994757536	16.02223595
	86.6118898	102.3271016	89.60138648	102.2777915	87.62069819	84.67858869	2.223296278	98.40502658	96.27839536	45.28696562	105.255589	97.09765982	92.37435009	82.66897747	84.84773459	3.673272112	98.59835669	99.95166747	5.993233446	4.639922668	97.0517158	7.151261603	6.314550922	92.70492875	56.37534043	36.09231513	2.513291445	88.15853069	7.346544224	7.151261603	2.513291445
	92.37081677	104.3313402	86.58859086	98.07329052	85.17818914	95.65328888	1.872034876	97.90998846	85.60071804	59.10748141	104.098002	108.0647513	86.79007682	82.15589976	95.65545901	1.961789973	102.0130786	97.29452494	2.513142711	5.154506988	89.90896269	38.80705848	1.006270964	105.7313694	54.71603073	2.102833697	4.974996794	86.01102705	4.769842287	26.67347236	7.411206565
AMP2CPR3STR1DOX2TMP2	AMP	CPR	STR	DOX	TMP	AMP+CPR	AMP+STR	AMP+DOX	AMP+TMP	CPR+STR	CPR+DOX	CPR+TMP	STR+DOX	STR+TMP	DOX+TMP	AMP+CPR+STR	AMP+CPR+DOX	AMP+CPR+TMP	AMP+STR+DOX	AMP+STR+TMP	AMP+DOX+TMP	CPR+STR+DOX	CPR+STR+TMP	CPR+DOX+TMP	STR+DOX+TMP	AMP+CPR+STR+DOX	AMP+CPR+STR+TMP	AMP+CPR+DOX+TMP	AMP+STR+DOX+TMP	CPR+STR+DOX+TMP	AMP+CPR+STR+DOX+TMP
	91.69857937	86.21231979	97.48535156	100.6103516	91.04003906	91.30327363	12.26683138	101.3835701	104.3483632	58.33551769	96.69724771	101.1009174	90.45410156	81.07910156	88.69628906	3.15009265	94.26806671	95.84928968	31.83446572	4.336009883	87.54786905	2.346002621	60.01310616	104.6657929	50.24414063	6.312538604	3.743051266	100.5929586	6.312538604	6.107470511	5.15132798
	86.6118898	102.3271016	89.60138648	102.2777915	93.36469423	84.67858869	2.223296278	98.40502658	102.0782987	45.28696562	105.255589	103.1638123	92.37435009	81.08442684	83.26318396	3.673272112	98.59835669	90.28516191	5.993233446	7.346544224	101.6916385	7.151261603	8.406327625	105.0464113	62.11933647	36.09231513	2.899951667	100.7249879	5.219913001	6.942083933	3.28661189
	92.37081677	104.3313402	86.58859086	98.07329052	93.84208538	95.65328888	1.872034876	97.90998846	96.47390691	59.10748141	104.098002	106.6647222	86.79007682	85.17818914	96.6628888	1.961789973	102.0130786	97.49967945	2.513142711	8.052314399	94.83267086	38.80705848	1.706285548	110.6314715	64.58884272	2.102833697	4.974996794	100.5769971	3.192717015	58.87414321	2.897807411
AMP2CPR3STR1DOX2TMP3	AMP	CPR	STR	DOX	TMP	AMP+CPR	AMP+STR	AMP+DOX	AMP+TMP	CPR+STR	CPR+DOX	CPR+TMP	STR+DOX	STR+TMP	DOX+TMP	AMP+CPR+STR	AMP+CPR+DOX	AMP+CPR+TMP	AMP+STR+DOX	AMP+STR+TMP	AMP+DOX+TMP	CPR+STR+DOX	CPR+STR+TMP	CPR+DOX+TMP	STR+DOX+TMP	AMP+CPR+STR+DOX	AMP+CPR+STR+TMP	AMP+CPR+DOX+TMP	AMP+STR+DOX+TMP	CPR+STR+DOX+TMP	AMP+CPR+STR+DOX+TMP
	91.69857937	86.21231979	97.48535156	100.6103516	88.11035156	91.30327363	12.26683138	101.3835701	101.1859172	58.33551769	96.69724771	100.2621232	90.45410156	86.54785156	94.55566406	3.15009265	94.26806671	88.14082767	31.83446572	7.893761581	86.75725757	2.346002621	7.798165138	101.7300131	50.83007813	6.312538604	3.15009265	97.82581841	5.15132798	5.268676278	7.127856702
	86.6118898	102.3271016	89.60138648	102.2777915	91.97821243	84.67858869	2.223296278	98.40502658	98.79168681	45.28696562	105.255589	98.77108119	92.37435009	84.45159693	86.03614756	3.673272112	98.59835669	91.63847269	5.993233446	6.379893668	94.73175447	7.151261603	6.732906262	102.1179239	54.39465214	36.09231513	3.28661189	97.43837603	6.766553891	5.26866257	3.28661189
	92.37081677	104.3313402	86.58859086	98.07329052	96.6628888	95.65328888	1.872034876	97.90998846	99.34607001	59.10748141	104.098002	99.66457635	86.79007682	90.01385216	97.46883264	1.961789973	102.0130786	92.57597128	2.513142711	4.744197974	98.73060649	38.80705848	53.74070293	103.1646493	51.8952273	2.102833697	5.975125016	95.03782536	8.526734197	1.370861893	0.730862931
AMP2CPR3STR1DOX3TMP1	AMP	CPR	STR	DOX	TMP	AMP+CPR	AMP+STR	AMP+DOX	AMP+TMP	CPR+STR	CPR+DOX	CPR+TMP	STR+DOX	STR+TMP	DOX+TMP	AMP+CPR+STR	AMP+CPR+DOX	AMP+CPR+TMP	AMP+STR+DOX	AMP+STR+TMP	AMP+DOX+TMP	CPR+STR+DOX	CPR+STR+TMP	CPR+DOX+TMP	STR+DOX+TMP	AMP+CPR+STR+DOX	AMP+CPR+STR+TMP	AMP+CPR+DOX+TMP	AMP+STR+DOX+TMP	CPR+STR+DOX+TMP	AMP+CPR+STR+DOX+TMP
	91.69857937	86.21231979	97.48535156	99.04785156	83.22753906	91.30327363	12.26683138	101.9765287	91.89623224	58.33551769	103.8269987	95.64875491	81.86035156	81.07910156	79.51660156	3.15009265	95.45398394	100.1976529	6.312538604	6.905497221	95.0586782	1.703800786	2.752293578	97.7457405	43.01757813	4.928968499	8.906732551	91.89623224	9.499691167	23.34207077	4.360716492
	86.6118898	102.3271016	89.60138648	101.2874474	87.62069819	84.67858869	2.223296278	98.98501692	96.27839536	45.28696562	105.4647666	97.09765982	87.22456053	82.66897747	82.47090864	3.673272112	103.2382794	99.95166747	5.799903335	4.639922668	91.44514258	4.431951889	6.314550922	93.12328409	60.13864818	2.223296278	2.513291445	100.918318	4.639922668	5.477840241	0.966650556
	92.37081677	104.3313402	86.58859086	100.0881501	85.17818914	95.65328888	1.872034876	95.03782536	85.60071804	59.10748141	113.8982062	108.0647513	86.18561894	82.15589976	84.37224531	1.961789973	101.8079241	97.29452494	2.692652904	5.154506988	99.96153353	3.106314715	1.006270964	94.29779787	47.90328674	2.077189383	4.974996794	96.0635979	1.346326452	5.570949395	1.346326452
AMP2CPR3STR1DOX3TMP2	AMP	CPR	STR	DOX	TMP	AMP+CPR	AMP+STR	AMP+DOX	AMP+TMP	CPR+STR	CPR+DOX	CPR+TMP	STR+DOX	STR+TMP	DOX+TMP	AMP+CPR+STR	AMP+CPR+DOX	AMP+CPR+TMP	AMP+STR+DOX	AMP+STR+TMP	AMP+DOX+TMP	CPR+STR+DOX	CPR+STR+TMP	CPR+DOX+TMP	STR+DOX+TMP	AMP+CPR+STR+DOX	AMP+CPR+STR+TMP	AMP+CPR+DOX+TMP	AMP+STR+DOX+TMP	CPR+STR+DOX+TMP	AMP+CPR+STR+DOX+TMP
	91.69857937	86.21231979	97.48535156	99.04785156	91.04003906	91.30327363	12.26683138	101.9765287	104.3483632	58.33551769	103.8269987	101.1009174	81.86035156	81.07910156	77.56347656	3.15009265	95.45398394	95.84928968	6.312538604	4.336009883	94.26806671	1.703800786	60.01310616	97.7457405	47.29003906	4.928968499	3.743051266	84.3854231	48.04200124	23.76146789	3.965410747
	86.6118898	102.3271016	89.60138648	101.2874474	93.36469423	84.67858869	2.223296278	98.98501692	102.0782987	45.28696562	105.4647666	103.1638123	87.22456053	81.08442684	85.83807873	3.673272112	103.2382794	90.28516191	5.799903335	7.346544224	97.63170614	4.431951889	8.406327625	93.33246176	51.57217133	2.223296278	2.899951667	86.22522958	2.126631223	4.418878285	2.609956501
	92.37081677	104.3313402	86.58859086	100.0881501	93.84208538	95.65328888	1.872034876	95.03782536	96.47390691	59.10748141	113.8982062	106.6647222	86.18561894	85.17818914	95.65545901	1.961789973	101.8079241	97.49967945	2.692652904	8.052314399	96.67906142	3.106314715	1.706285548	99.19789996	70.26822818	2.077189383	4.974996794	92.98628029	1.756635466	1.137523698	5.975125016
AMP2CPR3STR1DOX3TMP3	AMP	CPR	STR	DOX	TMP	AMP+CPR	AMP+STR	AMP+DOX	AMP+TMP	CPR+STR	CPR+DOX	CPR+TMP	STR+DOX	STR+TMP	DOX+TMP	AMP+CPR+STR	AMP+CPR+DOX	AMP+CPR+TMP	AMP+STR+DOX	AMP+STR+TMP	AMP+DOX+TMP	CPR+STR+DOX	CPR+STR+TMP	CPR+DOX+TMP	STR+DOX+TMP	AMP+CPR+STR+DOX	AMP+CPR+STR+TMP	AMP+CPR+DOX+TMP	AMP+STR+DOX+TMP	CPR+STR+DOX+TMP	AMP+CPR+STR+DOX+TMP
	91.69857937	86.21231979	97.48535156	99.04785156	88.11035156	91.30327363	12.26683138	101.9765287	101.1859172	58.33551769	103.8269987	100.2621232	81.86035156	86.54785156	93.96972656	3.15009265	95.45398394	88.14082767	6.312538604	7.893761581	91.10562075	1.703800786	7.798165138	93.55176933	52.00195313	4.928968499	3.15009265	88.53613342	6.732550957	5.307994758	1.39592341
	86.6118898	102.3271016	89.60138648	101.2874474	91.97821243	84.67858869	2.223296278	98.98501692	98.79168681	45.28696562	105.4647666	98.77108119	87.22456053	84.45159693	101.0893786	3.673272112	103.2382794	91.63847269	5.799903335	6.379893668	92.41179314	4.431951889	6.732906262	98.77108119	42.42386729	2.223296278	3.28661189	91.25181247	1.933301112	7.556543339	2.029966167
	92.37081677	104.3313402	86.58859086	100.0881501	96.6628888	95.65328888	1.872034876	95.03782536	99.34607001	59.10748141	113.8982062	99.66457635	86.18561894	90.01385216	104.5208412	1.961789973	101.8079241	92.57597128	2.692652904	4.744197974	81.70278241	3.106314715	53.74070293	105.264693	48.26848004	2.077189383	5.975125016	102.8336966	6.000769329	5.3376112	11.95025003
AMP2CPR3STR2DOX1TMP1	AMP	CPR	STR	DOX	TMP	AMP+CPR	AMP+STR	AMP+DOX	AMP+TMP	CPR+STR	CPR+DOX	CPR+TMP	STR+DOX	STR+TMP	DOX+TMP	AMP+CPR+STR	AMP+CPR+DOX	AMP+CPR+TMP	AMP+STR+DOX	AMP+STR+TMP	AMP+DOX+TMP	CPR+STR+DOX	CPR+STR+TMP	CPR+DOX+TMP	STR+DOX+TMP	AMP+CPR+STR+DOX	AMP+CPR+STR+TMP	AMP+CPR+DOX+TMP	AMP+STR+DOX+TMP	CPR+STR+DOX+TMP	AMP+CPR+STR+DOX+TMP
	91.69857937	86.21231979	46.67078443	103.9306641	83.22753906	91.30327363	6.337245213	101.9765287	91.89623224	58.15203145	101.5203145	95.64875491	45.07720815	49.20321186	83.61816406	2.952439778	90.11735639	100.1976529	4.731315627	4.928968499	85.17603459	17.0249017	28.55832241	90.40629096	3.347745522	5.126621371	5.756639901	86.75725757	13.84805435	8.427260813	7.103150093
	86.6118898	102.3271016	50.16916385	102.6739292	87.62069819	84.67858869	1.449975834	88.3518608	96.27839536	56.92247353	106.0922996	97.09765982	3.28661189	54.03576607	76.7269126	2.513291445	94.73175447	99.95166747	3.28661189	4.446592557	93.95843403	54.28160544	5.0594849	86.22042097	2.319961334	3.28661189	1.643305945	82.74528758	7.539874335	7.569616943	2.899951667
	92.37081677	104.3313402	59.93076035	98.47626244	85.17818914	95.65328888	3.513270932	95.44813438	85.60071804	64.13883623	103.8646638	108.0647513	6.475189127	57.08424157	85.17818914	1.487370176	103.2440056	97.29452494	2.897807411	2.28234389	87.85741762	32.27358903	62.14087793	82.86422634	47.50609052	45.86485447	2.307988204	87.4471086	7.231696371	10.10646055	6.795743044
AMP2CPR3STR2DOX1TMP2	AMP	CPR	STR	DOX	TMP	AMP+CPR	AMP+STR	AMP+DOX	AMP+TMP	CPR+STR	CPR+DOX	CPR+TMP	STR+DOX	STR+TMP	DOX+TMP	AMP+CPR+STR	AMP+CPR+DOX	AMP+CPR+TMP	AMP+STR+DOX	AMP+STR+TMP	AMP+DOX+TMP	CPR+STR+DOX	CPR+STR+TMP	CPR+DOX+TMP	STR+DOX+TMP	AMP+CPR+STR+DOX	AMP+CPR+STR+TMP	AMP+CPR+DOX+TMP	AMP+STR+DOX+TMP	CPR+STR+DOX+TMP	AMP+CPR+STR+DOX+TMP
	91.69857937	86.21231979	46.67078443	103.9306641	91.04003906	91.30327363	6.337245213	101.9765287	104.3483632	58.15203145	101.5203145	101.1009174	45.07720815	63.03891291	84.20410156	2.952439778	90.11735639	95.84928968	4.731315627	9.277331686	85.76899321	17.0249017	58.12581913	89.35779817	5.126621371	5.126621371	3.545398394	97.82581841	10.85855466	3.171690695	4.533662755
	86.6118898	102.3271016	50.16916385	102.6739292	93.36469423	84.67858869	1.449975834	88.3518608	102.0782987	56.92247353	106.0922996	103.1638123	3.28661189	54.80908652	80.49022035	2.513291445	94.73175447	90.28516191	3.28661189	6.379893668	83.90526825	54.28160544	5.26866257	94.16917244	1.739971	3.28661189	3.673272112	84.09859836	6.766553891	8.406327625	2.319961334
	92.37081677	104.3313402	59.93076035	98.47626244	93.84208538	95.65328888	3.513270932	95.44813438	96.47390691	64.13883623	103.8646638	106.6647222	6.475189127	73.08629311	87.79750661	1.487370176	103.2440056	97.49967945	2.897807411	5.359661495	90.52442621	32.27358903	39.04039667	85.43094648	1.051416848	45.86485447	6.411078343	90.11411719	42.78753686	4.870934811	7.411206565
AMP2CPR3STR2DOX1TMP3	AMP	CPR	STR	DOX	TMP	AMP+CPR	AMP+STR	AMP+DOX	AMP+TMP	CPR+STR	CPR+DOX	CPR+TMP	STR+DOX	STR+TMP	DOX+TMP	AMP+CPR+STR	AMP+CPR+DOX	AMP+CPR+TMP	AMP+STR+DOX	AMP+STR+TMP	AMP+DOX+TMP	CPR+STR+DOX	CPR+STR+TMP	CPR+DOX+TMP	STR+DOX+TMP	AMP+CPR+STR+DOX	AMP+CPR+STR+TMP	AMP+CPR+DOX+TMP	AMP+STR+DOX+TMP	CPR+STR+DOX+TMP	AMP+CPR+STR+DOX+TMP
	91.69857937	86.21231979	46.67078443	103.9306641	88.11035156	91.30327363	6.337245213	101.9765287	101.1859172	58.15203145	101.5203145	100.2621232	45.07720815	69.75911056	94.36035156	2.952439778	90.11735639	88.14082767	4.731315627	8.486720198	97.82581841	17.0249017	67.77195282	110.9567497	5.719579988	5.126621371	5.521927116	88.93143916	6.312538604	4.639580603	5.126621371
	86.6118898	102.3271016	50.16916385	102.6739292	91.97821243	84.67858869	1.449975834	88.3518608	98.79168681	56.92247353	106.0922996	98.77108119	3.28661189	54.71242146	99.10869027	2.513291445	94.73175447	91.63847269	3.28661189	5.02658289	99.56500725	54.28160544	23.32331024	107.138188	3.093281779	3.28661189	2.126631223	92.02513291	5.799903335	28.34357432	6.379893668
	92.37081677	104.3313402	59.93076035	98.47626244	96.6628888	95.65328888	3.513270932	95.44813438	99.34607001	64.13883623	103.8646638	99.66457635	6.475189127	71.65021157	109.1550183	1.487370176	103.2440056	92.57597128	2.897807411	5.359661495	93.60174381	32.27358903	43.24048418	112.498177	1.051416848	45.86485447	7.000897551	101.3976151	7.231696371	6.737640368	7.821515579
AMP2CPR3STR2DOX2TMP1	AMP	CPR	STR	DOX	TMP	AMP+CPR	AMP+STR	AMP+DOX	AMP+TMP	CPR+STR	CPR+DOX	CPR+TMP	STR+DOX	STR+TMP	DOX+TMP	AMP+CPR+STR	AMP+CPR+DOX	AMP+CPR+TMP	AMP+STR+DOX	AMP+STR+TMP	AMP+DOX+TMP	CPR+STR+DOX	CPR+STR+TMP	CPR+DOX+TMP	STR+DOX+TMP	AMP+CPR+STR+DOX	AMP+CPR+STR+TMP	AMP+CPR+DOX+TMP	AMP+STR+DOX+TMP	CPR+STR+DOX+TMP	AMP+CPR+STR+DOX+TMP
	91.69857937	86.21231979	46.67078443	100.6103516	83.22753906	91.30327363	6.337245213	101.3835701	91.89623224	58.15203145	96.69724771	95.64875491	61.6553428	49.20321186	83.81347656	2.952439778	94.26806671	100.1976529	6.510191476	4.928968499	88.14082767	56.47444299	28.55832241	103.197903	5.324274243	5.546633725	5.756639901	80.43236566	41.32180358	5.478374836	4.756022236
	86.6118898	102.3271016	50.16916385	102.2777915	87.62069819	84.67858869	1.449975834	98.40502658	96.27839536	56.92247353	105.255589	97.09765982	48.5258579	54.03576607	84.84773459	2.513291445	98.59835669	99.95166747	4.639922668	4.446592557	97.0517158	56.92247353	5.0594849	92.70492875	39.37892702	3.673272112	1.643305945	88.15853069	6.57322378	6.105373251	5.993233446
	92.37081677	104.3313402	59.93076035	98.07329052	85.17818914	95.65328888	3.513270932	97.90998846	85.60071804	64.13883623	104.098002	108.0647513	67.13681241	57.08424157	95.65545901	1.487370176	102.0130786	97.29452494	3.308116425	2.28234389	89.90896269	67.87224734	62.14087793	105.7313694	15.00192332	1.487370176	2.307988204	86.01102705	6.821387357	7.306402217	5.564816002
AMP2CPR3STR2DOX2TMP2	AMP	CPR	STR	DOX	TMP	AMP+CPR	AMP+STR	AMP+DOX	AMP+TMP	CPR+STR	CPR+DOX	CPR+TMP	STR+DOX	STR+TMP	DOX+TMP	AMP+CPR+STR	AMP+CPR+DOX	AMP+CPR+TMP	AMP+STR+DOX	AMP+STR+TMP	AMP+DOX+TMP	CPR+STR+DOX	CPR+STR+TMP	CPR+DOX+TMP	STR+DOX+TMP	AMP+CPR+STR+DOX	AMP+CPR+STR+TMP	AMP+CPR+DOX+TMP	AMP+STR+DOX+TMP	CPR+STR+DOX+TMP	AMP+CPR+STR+DOX+TMP
	91.69857937	86.21231979	46.67078443	100.6103516	91.04003906	91.30327363	6.337245213	101.3835701	104.3483632	58.15203145	96.69724771	101.1009174	61.6553428	63.03891291	88.69628906	2.952439778	94.26806671	95.84928968	6.510191476	9.277331686	87.54786905	56.47444299	58.12581913	104.6657929	12.43977764	5.546633725	3.545398394	100.5929586	5.324274243	28.34862385	7.325509574
	86.6118898	102.3271016	50.16916385	102.2777915	93.36469423	84.67858869	1.449975834	98.40502658	102.0782987	56.92247353	105.255589	103.1638123	48.5258579	54.80908652	83.26318396	2.513291445	98.59835669	90.28516191	4.639922668	6.379893668	101.6916385	56.92247353	5.26866257	105.0464113	25.0362494	3.673272112	3.673272112	100.7249879	6.57322378	5.0594849	4.446592557
	92.37081677	104.3313402	59.93076035	98.07329052	93.84208538	95.65328888	3.513270932	97.90998846	96.47390691	64.13883623	104.098002	106.6647222	67.13681241	73.08629311	96.6628888	1.487370176	102.0130786	97.49967945	3.308116425	5.359661495	94.83267086	67.87224734	39.04039667	110.6314715	24.02872163	1.487370176	6.411078343	100.5769971	6.064880113	4.870934811	3.718425439
AMP2CPR3STR2DOX2TMP3	AMP	CPR	STR	DOX	TMP	AMP+CPR	AMP+STR	AMP+DOX	AMP+TMP	CPR+STR	CPR+DOX	CPR+TMP	STR+DOX	STR+TMP	DOX+TMP	AMP+CPR+STR	AMP+CPR+DOX	AMP+CPR+TMP	AMP+STR+DOX	AMP+STR+TMP	AMP+DOX+TMP	CPR+STR+DOX	CPR+STR+TMP	CPR+DOX+TMP	STR+DOX+TMP	AMP+CPR+STR+DOX	AMP+CPR+STR+TMP	AMP+CPR+DOX+TMP	AMP+STR+DOX+TMP	CPR+STR+DOX+TMP	AMP+CPR+STR+DOX+TMP
	91.69857937	86.21231979	46.67078443	100.6103516	88.11035156	91.30327363	6.337245213	101.3835701	101.1859172	58.15203145	96.69724771	100.2621232	61.6553428	69.75911056	94.55566406	2.952439778	94.26806671	88.14082767	6.510191476	8.486720198	86.75725757	56.47444299	67.77195282	101.7300131	13.23038913	5.546633725	5.521927116	97.82581841	13.45274861	32.54259502	7.127856702
	86.6118898	102.3271016	50.16916385	102.2777915	91.97821243	84.67858869	1.449975834	98.40502658	98.79168681	56.92247353	105.255589	98.77108119	48.5258579	54.71242146	86.03614756	2.513291445	98.59835669	91.63847269	4.639922668	5.02658289	94.73175447	56.92247353	23.32331024	102.1179239	24.45625906	3.673272112	2.126631223	97.43837603	7.346544224	5.255588966	2.513291445
	92.37081677	104.3313402	59.93076035	98.07329052	96.6628888	95.65328888	3.513270932	97.90998846	99.34607001	64.13883623	104.098002	99.66457635	67.13681241	71.65021157	97.46883264	1.487370176	102.0130786	92.57597128	3.308116425	5.359661495	98.73060649	67.87224734	43.24048418	103.1646493	23.0029491	1.487370176	7.000897551	95.03782536	2.782408001	20.37334111	5.449416592
AMP2CPR3STR2DOX3TMP1	AMP	CPR	STR	DOX	TMP	AMP+CPR	AMP+STR	AMP+DOX	AMP+TMP	CPR+STR	CPR+DOX	CPR+TMP	STR+DOX	STR+TMP	DOX+TMP	AMP+CPR+STR	AMP+CPR+DOX	AMP+CPR+TMP	AMP+STR+DOX	AMP+STR+TMP	AMP+DOX+TMP	CPR+STR+DOX	CPR+STR+TMP	CPR+DOX+TMP	STR+DOX+TMP	AMP+CPR+STR+DOX	AMP+CPR+STR+TMP	AMP+CPR+DOX+TMP	AMP+STR+DOX+TMP	CPR+STR+DOX+TMP	AMP+CPR+STR+DOX+TMP
	91.69857937	86.21231979	46.67078443	99.04785156	83.22753906	91.30327363	6.337245213	101.9765287	91.89623224	58.15203145	103.8269987	95.64875491	62.44595429	49.20321186	79.51660156	2.952439778	95.45398394	100.1976529	5.91723286	4.928968499	95.0586782	66.74967235	28.55832241	97.7457405	12.0444719	7.325509574	5.756639901	91.89623224	23.13773935	31.07470511	5.744286597
	86.6118898	102.3271016	50.16916385	101.2874474	87.62069819	84.67858869	1.449975834	98.98501692	96.27839536	56.92247353	105.4647666	97.09765982	54.22909618	54.03576607	82.47090864	2.513291445	103.2382794	99.95166747	4.253262446	4.446592557	91.44514258	67.79971238	5.0594849	93.12328409	12.27646206	2.996616723	1.643305945	100.918318	5.606573224	7.765721009	8.31319478
	92.37081677	104.3313402	59.93076035	100.0881501	85.17818914	95.65328888	3.513270932	95.03782536	85.60071804	64.13883623	113.8982062	108.0647513	58.93063213	57.08424157	84.37224531	1.487370176	101.8079241	97.29452494	4.33388896	2.28234389	99.96153353	70.20562928	62.14087793	94.29779787	28.13181177	1.692524683	2.307988204	96.0635979	16.73291448	69.84103835	5.244262085
AMP2CPR3STR2DOX3TMP2	AMP	CPR	STR	DOX	TMP	AMP+CPR	AMP+STR	AMP+DOX	AMP+TMP	CPR+STR	CPR+DOX	CPR+TMP	STR+DOX	STR+TMP	DOX+TMP	AMP+CPR+STR	AMP+CPR+DOX	AMP+CPR+TMP	AMP+STR+DOX	AMP+STR+TMP	AMP+DOX+TMP	CPR+STR+DOX	CPR+STR+TMP	CPR+DOX+TMP	STR+DOX+TMP	AMP+CPR+STR+DOX	AMP+CPR+STR+TMP	AMP+CPR+DOX+TMP	AMP+STR+DOX+TMP	CPR+STR+DOX+TMP	AMP+CPR+STR+DOX+TMP
	91.69857937	86.21231979	46.67078443	99.04785156	91.04003906	91.30327363	6.337245213	101.9765287	104.3483632	58.15203145	103.8269987	101.1009174	62.44595429	63.03891291	77.56347656	2.952439778	95.45398394	95.84928968	5.91723286	9.277331686	94.26806671	66.74967235	58.12581913	97.7457405	52.40271773	7.325509574	3.545398394	84.3854231	7.91846819	44.94102228	3.965410747
	86.6118898	102.3271016	50.16916385	101.2874474	93.36469423	84.67858869	1.449975834	98.98501692	102.0782987	56.92247353	105.4647666	103.1638123	54.22909618	54.80908652	85.83807873	2.513291445	103.2382794	90.28516191	4.253262446	6.379893668	97.63170614	67.79971238	5.26866257	93.33246176	51.71580474	2.996616723	3.673272112	86.22522958	1.933301112	38.80245784	-0.386660222
	92.37081677	104.3313402	59.93076035	100.0881501	93.84208538	95.65328888	3.513270932	95.03782536	96.47390691	64.13883623	113.8982062	106.6647222	58.93063213	73.08629311	95.65545901	1.487370176	101.8079241	97.49967945	4.33388896	5.359661495	96.67906142	70.20562928	39.04039667	99.19789996	59.52045134	1.692524683	6.411078343	92.98628029	4.833953071	1.837538282	0.641107834
AMP2CPR3STR2DOX3TMP3	AMP	CPR	STR	DOX	TMP	AMP+CPR	AMP+STR	AMP+DOX	AMP+TMP	CPR+STR	CPR+DOX	CPR+TMP	STR+DOX	STR+TMP	DOX+TMP	AMP+CPR+STR	AMP+CPR+DOX	AMP+CPR+TMP	AMP+STR+DOX	AMP+STR+TMP	AMP+DOX+TMP	CPR+STR+DOX	CPR+STR+TMP	CPR+DOX+TMP	STR+DOX+TMP	AMP+CPR+STR+DOX	AMP+CPR+STR+TMP	AMP+CPR+DOX+TMP	AMP+STR+DOX+TMP	CPR+STR+DOX+TMP	AMP+CPR+STR+DOX+TMP
	91.69857937	86.21231979	46.67078443	99.04785156	88.11035156	91.30327363	6.337245213	101.9765287	101.1859172	58.15203145	103.8269987	100.2621232	62.44595429	69.75911056	93.96972656	2.952439778	95.45398394	88.14082767	5.91723286	8.486720198	91.10562075	66.74967235	67.77195282	93.55176933	45.28721433	7.325509574	5.521927116	88.53613342	4.953675108	65.88466579	24.32365658
	86.6118898	102.3271016	50.16916385	101.2874474	91.97821243	84.67858869	1.449975834	98.98501692	98.79168681	56.92247353	105.4647666	98.77108119	54.22909618	54.71242146	101.0893786	2.513291445	103.2382794	91.63847269	4.253262446	5.02658289	92.41179314	67.79971238	23.32331024	98.77108119	52.13871435	2.996616723	2.126631223	91.25181247	3.588690188	69.13322003	2.029966167
	92.37081677	104.3313402	59.93076035	100.0881501	96.6628888	95.65328888	3.513270932	95.03782536	99.34607001	64.13883623	113.8982062	99.66457635	58.93063213	71.65021157	104.5208412	1.487370176	101.8079241	92.57597128	4.33388896	5.359661495	81.70278241	70.20562928	43.24048418	105.264693	64.239005	1.692524683	7.000897551	102.8336966	6.61623285	57.00743765	1.756635466
AMP2CPR3STR3DOX1TMP1	AMP	CPR	STR	DOX	TMP	AMP+CPR	AMP+STR	AMP+DOX	AMP+TMP	CPR+STR	CPR+DOX	CPR+TMP	STR+DOX	STR+TMP	DOX+TMP	AMP+CPR+STR	AMP+CPR+DOX	AMP+CPR+TMP	AMP+STR+DOX	AMP+STR+TMP	AMP+DOX+TMP	CPR+STR+DOX	CPR+STR+TMP	CPR+DOX+TMP	STR+DOX+TMP	AMP+CPR+STR+DOX	AMP+CPR+STR+TMP	AMP+CPR+DOX+TMP	AMP+STR+DOX+TMP	CPR+STR+DOX+TMP	AMP+CPR+STR+DOX+TMP
	91.69857937	86.21231979	93.55176933	103.9306641	83.22753906	91.30327363	55.3304509	101.9765287	91.89623224	72.5819135	101.5203145	95.64875491	83.48623853	89.56749672	83.61816406	38.72760964	90.11735639	100.1976529	34.41630636	47.06609018	85.17603459	53.32896461	60.66841415	90.40629096	49.97378768	5.126621371	40.14823965	86.75725757	45.4848672	44.52162516	46.856084
	86.6118898	102.3271016	85.80206563	102.6739292	87.62069819	84.67858869	35.57274045	88.3518608	96.27839536	68.64949667	106.0922996	97.09765982	93.33246176	96.88848215	76.7269126	28.70952151	94.73175447	99.95166747	20.23924601	45.33591107	93.95843403	58.38671722	64.45286966	86.22042097	53.15727546	2.899951667	55.19574674	82.74528758	11.59980667	21.4407112	9.279845336
	92.37081677	104.3313402	94.53113607	98.47626244	85.17818914	95.65328888	68.36773945	95.44813438	85.60071804	74.46405133	103.8646638	108.0647513	92.43109231	96.63117982	85.17818914	48.46775228	103.2440056	97.29452494	47.30093602	45.56994486	87.85741762	65.77220359	71.37232026	82.86422634	57.83870497	1.077061162	47.21118092	87.4471086	49.87818951	73.47236401	7.821515579
AMP2CPR3STR3DOX1TMP2	AMP	CPR	STR	DOX	TMP	AMP+CPR	AMP+STR	AMP+DOX	AMP+TMP	CPR+STR	CPR+DOX	CPR+TMP	STR+DOX	STR+TMP	DOX+TMP	AMP+CPR+STR	AMP+CPR+DOX	AMP+CPR+TMP	AMP+STR+DOX	AMP+STR+TMP	AMP+DOX+TMP	CPR+STR+DOX	CPR+STR+TMP	CPR+DOX+TMP	STR+DOX+TMP	AMP+CPR+STR+DOX	AMP+CPR+STR+TMP	AMP+CPR+DOX+TMP	AMP+STR+DOX+TMP	CPR+STR+DOX+TMP	AMP+CPR+STR+DOX+TMP
	91.69857937	86.21231979	93.55176933	103.9306641	91.04003906	91.30327363	55.3304509	101.9765287	104.3483632	72.5819135	101.5203145	101.1009174	83.48623853	88.30930537	84.20410156	38.72760964	90.11735639	95.84928968	34.41630636	40.34589253	85.76899321	53.32896461	59.61992136	89.35779817	42.1887287	5.126621371	26.3125386	97.82581841	43.90364422	46.40891219	18.78937616
	86.6118898	102.3271016	85.80206563	102.6739292	93.36469423	84.67858869	35.57274045	88.3518608	102.0782987	68.64949667	106.0922996	103.1638123	93.33246176	92.91410642	80.49022035	28.70952151	94.73175447	90.28516191	20.23924601	30.4857419	83.90526825	58.38671722	66.33546869	94.16917244	37.33821415	2.899951667	24.10584824	84.09859836	34.35234413	40.60661524	6.959884002
	92.37081677	104.3313402	94.53113607	98.47626244	93.84208538	95.65328888	68.36773945	95.44813438	96.47390691	74.46405133	103.8646638	106.6647222	92.43109231	96.86451801	87.79750661	48.46775228	103.2440056	97.49967945	47.30093602	49.26272599	90.52442621	65.77220359	73.23902581	85.43094648	54.44071751	1.077061162	25.14424926	90.11411719	37.24836518	55.27198483	7.206052058
AMP2CPR3STR3DOX1TMP3	AMP	CPR	STR	DOX	TMP	AMP+CPR	AMP+STR	AMP+DOX	AMP+TMP	CPR+STR	CPR+DOX	CPR+TMP	STR+DOX	STR+TMP	DOX+TMP	AMP+CPR+STR	AMP+CPR+DOX	AMP+CPR+TMP	AMP+STR+DOX	AMP+STR+TMP	AMP+DOX+TMP	CPR+STR+DOX	CPR+STR+TMP	CPR+DOX+TMP	STR+DOX+TMP	AMP+CPR+STR+DOX	AMP+CPR+STR+TMP	AMP+CPR+DOX+TMP	AMP+STR+DOX+TMP	CPR+STR+DOX+TMP	AMP+CPR+STR+DOX+TMP
	91.69857937	86.21231979	93.55176933	103.9306641	88.11035156	91.30327363	55.3304509	101.9765287	101.1859172	72.5819135	101.5203145	100.2621232	83.48623853	94.60026212	94.36035156	38.72760964	90.11735639	88.14082767	34.41630636	43.07597282	97.82581841	53.32896461	80.1310616	110.9567497	56.68414155	5.126621371	40.70413836	88.93143916	35.58987029	68.84665793	5.324274243
	86.6118898	102.3271016	85.80206563	102.6739292	91.97821243	84.67858869	35.57274045	88.3518608	98.79168681	68.64949667	106.0922996	98.77108119	93.33246176	94.16917244	99.10869027	28.70952151	94.73175447	91.63847269	20.23924601	36.92605123	99.56500725	58.38671722	56.08576284	107.138188	58.59589489	2.899951667	37.2160464	92.02513291	7.926534558	58.17753955	9.666505558
	92.37081677	104.3313402	94.53113607	98.47626244	96.6628888	95.65328888	68.36773945	95.44813438	99.34607001	74.46405133	103.8646638	99.66457635	92.43109231	93.1311069	109.1550183	48.46775228	103.2440056	92.57597128	47.30093602	42.72342608	93.60174381	65.77220359	57.37202858	112.498177	45.93845705	1.077061162	37.56891909	101.3976151	20.63085011	74.87239317	4.154378767
AMP2CPR3STR3DOX2TMP1	AMP	CPR	STR	DOX	TMP	AMP+CPR	AMP+STR	AMP+DOX	AMP+TMP	CPR+STR	CPR+DOX	CPR+TMP	STR+DOX	STR+TMP	DOX+TMP	AMP+CPR+STR	AMP+CPR+DOX	AMP+CPR+TMP	AMP+STR+DOX	AMP+STR+TMP	AMP+DOX+TMP	CPR+STR+DOX	CPR+STR+TMP	CPR+DOX+TMP	STR+DOX+TMP	AMP+CPR+STR+DOX	AMP+CPR+STR+TMP	AMP+CPR+DOX+TMP	AMP+STR+DOX+TMP	CPR+STR+DOX+TMP	AMP+CPR+STR+DOX+TMP
	91.69857937	86.21231979	93.55176933	100.6103516	83.22753906	91.30327363	55.3304509	101.3835701	91.89623224	72.5819135	96.69724771	95.64875491	95.64875491	89.56749672	83.81347656	38.72760964	94.26806671	100.1976529	53.35392217	47.06609018	88.14082767	64.82306684	60.66841415	103.197903	51.86107471	43.70599135	40.14823965	80.43236566	44.69425571	68.0078637	56.55342804
	86.6118898	102.3271016	85.80206563	102.2777915	87.62069819	84.67858869	35.57274045	98.40502658	96.27839536	68.64949667	105.255589	97.09765982	96.67930448	96.88848215	84.84773459	28.70952151	98.59835669	99.95166747	45.62590623	45.33591107	97.0517158	64.8842986	64.45286966	92.70492875	51.69303177	44.94925085	55.19574674	88.15853069	48.62252296	62.98862596	33.77235379
	92.37081677	104.3313402	94.53113607	98.07329052	85.17818914	95.65328888	68.36773945	97.90998846	85.60071804	74.46405133	104.098002	108.0647513	99.19789996	96.63117982	95.65545901	48.46775228	102.0130786	97.29452494	43.31324529	45.56994486	89.90896269	71.19731661	71.37232026	105.7313694	49.20519177	45.56994486	47.21118092	86.01102705	57.26375176	72.07233484	51.51942557
AMP2CPR3STR3DOX2TMP2	AMP	CPR	STR	DOX	TMP	AMP+CPR	AMP+STR	AMP+DOX	AMP+TMP	CPR+STR	CPR+DOX	CPR+TMP	STR+DOX	STR+TMP	DOX+TMP	AMP+CPR+STR	AMP+CPR+DOX	AMP+CPR+TMP	AMP+STR+DOX	AMP+STR+TMP	AMP+DOX+TMP	CPR+STR+DOX	CPR+STR+TMP	CPR+DOX+TMP	STR+DOX+TMP	AMP+CPR+STR+DOX	AMP+CPR+STR+TMP	AMP+CPR+DOX+TMP	AMP+STR+DOX+TMP	CPR+STR+DOX+TMP	AMP+CPR+STR+DOX+TMP
	91.69857937	86.21231979	93.55176933	100.6103516	91.04003906	91.30327363	55.3304509	101.3835701	104.3483632	72.5819135	96.69724771	101.1009174	95.64875491	88.30930537	88.69628906	38.72760964	94.26806671	95.84928968	53.35392217	40.34589253	87.54786905	64.82306684	59.61992136	104.6657929	59.82961992	43.70599135	26.3125386	100.5929586	49.0426189	67.37876802	34.20630019
	86.6118898	102.3271016	85.80206563	102.2777915	93.36469423	84.67858869	35.57274045	98.40502658	102.0782987	68.64949667	105.255589	103.1638123	96.67930448	92.91410642	83.26318396	28.70952151	98.59835669	90.28516191	45.62590623	30.4857419	101.6916385	64.8842986	66.33546869	105.0464113	61.73355994	44.94925085	24.10584824	100.7249879	45.17883035	68.00889005	6.379893668
	92.37081677	104.3313402	94.53113607	98.07329052	93.84208538	95.65328888	68.36773945	97.90998846	96.47390691	74.46405133	104.098002	106.6647222	99.19789996	96.86451801	96.6628888	48.46775228	102.0130786	97.49967945	43.31324529	49.26272599	94.83267086	71.19731661	73.23902581	110.6314715	62.50546886	45.56994486	25.14424926	100.5769971	34.58135658	74.63905498	34.78651109
AMP2CPR3STR3DOX2TMP3	AMP	CPR	STR	DOX	TMP	AMP+CPR	AMP+STR	AMP+DOX	AMP+TMP	CPR+STR	CPR+DOX	CPR+TMP	STR+DOX	STR+TMP	DOX+TMP	AMP+CPR+STR	AMP+CPR+DOX	AMP+CPR+TMP	AMP+STR+DOX	AMP+STR+TMP	AMP+DOX+TMP	CPR+STR+DOX	CPR+STR+TMP	CPR+DOX+TMP	STR+DOX+TMP	AMP+CPR+STR+DOX	AMP+CPR+STR+TMP	AMP+CPR+DOX+TMP	AMP+STR+DOX+TMP	CPR+STR+DOX+TMP	AMP+CPR+STR+DOX+TMP
	91.69857937	86.21231979	93.55176933	100.6103516	88.11035156	91.30327363	55.3304509	101.3835701	101.1859172	72.5819135	96.69724771	100.2621232	95.64875491	94.60026212	94.55566406	38.72760964	94.26806671	88.14082767	53.35392217	43.07597282	86.75725757	64.82306684	80.1310616	101.7300131	70.90432503	43.70599135	40.70413836	97.82581841	30.26559605	63.18479685	29.83323039
	86.6118898	102.3271016	85.80206563	102.2777915	91.97821243	84.67858869	35.57274045	98.40502658	98.79168681	68.64949667	105.255589	98.77108119	96.67930448	94.16917244	86.03614756	28.70952151	98.59835669	91.63847269	45.62590623	36.92605123	94.73175447	64.8842986	56.08576284	102.1179239	67.81278599	44.94925085	37.2160464	97.43837603	31.9961334	61.3152046	26.7762204
	92.37081677	104.3313402	94.53113607	98.07329052	96.6628888	95.65328888	68.36773945	97.90998846	99.34607001	74.46405133	104.098002	99.66457635	99.19789996	93.1311069	97.46883264	48.46775228	102.0130786	92.57597128	43.31324529	42.72342608	98.73060649	71.19731661	57.37202858	103.1646493	70.73064022	45.56994486	37.56891909	95.03782536	36.13283754	75.33906956	32.44005642
AMP2CPR3STR3DOX3TMP1	AMP	CPR	STR	DOX	TMP	AMP+CPR	AMP+STR	AMP+DOX	AMP+TMP	CPR+STR	CPR+DOX	CPR+TMP	STR+DOX	STR+TMP	DOX+TMP	AMP+CPR+STR	AMP+CPR+DOX	AMP+CPR+TMP	AMP+STR+DOX	AMP+STR+TMP	AMP+DOX+TMP	CPR+STR+DOX	CPR+STR+TMP	CPR+DOX+TMP	STR+DOX+TMP	AMP+CPR+STR+DOX	AMP+CPR+STR+TMP	AMP+CPR+DOX+TMP	AMP+STR+DOX+TMP	CPR+STR+DOX+TMP	AMP+CPR+STR+DOX+TMP
	91.69857937	86.21231979	93.55176933	99.04785156	83.22753906	91.30327363	55.3304509	101.9765287	91.89623224	72.5819135	103.8269987	95.64875491	91.03538663	89.56749672	79.51660156	38.72760964	95.45398394	100.1976529	37.18344657	47.06609018	95.0586782	60.87811271	60.66841415	97.7457405	72.16251638	27.26374305	40.14823965	91.89623224	35.20691785	71.36304063	48.43730698
	86.6118898	102.3271016	85.80206563	101.2874474	87.62069819	84.67858869	35.57274045	98.98501692	96.27839536	68.64949667	105.4647666	97.09765982	94.37835011	96.88848215	82.47090864	28.70952151	103.2382794	99.95166747	30.83615273	45.33591107	91.44514258	59.85096091	64.45286966	93.12328409	74.50647143	42.62928951	55.19574674	100.918318	36.24939584	54.20316381	40.15224746
	92.37081677	104.3313402	94.53113607	100.0881501	85.17818914	95.65328888	68.36773945	95.03782536	85.60071804	74.46405133	113.8982062	108.0647513	96.86451801	96.63117982	84.37224531	48.46775228	101.8079241	97.29452494	37.7740736	45.56994486	99.96153353	63.90549803	71.37232026	94.29779787	78.66413884	36.54314656	47.21118092	96.0635979	49.05757148	52.4719265	4.628798564
AMP2CPR3STR3DOX3TMP2	AMP	CPR	STR	DOX	TMP	AMP+CPR	AMP+STR	AMP+DOX	AMP+TMP	CPR+STR	CPR+DOX	CPR+TMP	STR+DOX	STR+TMP	DOX+TMP	AMP+CPR+STR	AMP+CPR+DOX	AMP+CPR+TMP	AMP+STR+DOX	AMP+STR+TMP	AMP+DOX+TMP	CPR+STR+DOX	CPR+STR+TMP	CPR+DOX+TMP	STR+DOX+TMP	AMP+CPR+STR+DOX	AMP+CPR+STR+TMP	AMP+CPR+DOX+TMP	AMP+STR+DOX+TMP	CPR+STR+DOX+TMP	AMP+CPR+STR+DOX+TMP
	91.69857937	86.21231979	93.55176933	99.04785156	91.04003906	91.30327363	55.3304509	101.9765287	104.3483632	72.5819135	103.8269987	101.1009174	91.03538663	88.30930537	77.56347656	38.72760964	95.45398394	95.84928968	37.18344657	40.34589253	94.26806671	60.87811271	59.61992136	97.7457405	72.37221494	27.26374305	26.3125386	84.3854231	58.72760964	67.58846658	32.63743051
	86.6118898	102.3271016	85.80206563	101.2874474	93.36469423	84.67858869	35.57274045	98.98501692	102.0782987	68.64949667	105.4647666	103.1638123	94.37835011	92.91410642	85.83807873	28.70952151	103.2382794	90.28516191	30.83615273	30.4857419	97.63170614	59.85096091	66.33546869	93.33246176	85.38371029	42.62928951	24.10584824	86.22522958	38.37602707	63.82533665	1.739971
	92.37081677	104.3313402	94.53113607	100.0881501	93.84208538	95.65328888	68.36773945	95.03782536	96.47390691	74.46405133	113.8982062	106.6647222	96.86451801	96.86451801	95.65545901	48.46775228	101.8079241	97.49967945	37.7740736	49.26272599	96.67906142	63.90549803	73.23902581	99.19789996	97.09785621	36.54314656	25.14424926	92.98628029	39.29991024	71.83899665	34.37620208
AMP2CPR3STR3DOX3TMP3	AMP	CPR	STR	DOX	TMP	AMP+CPR	AMP+STR	AMP+DOX	AMP+TMP	CPR+STR	CPR+DOX	CPR+TMP	STR+DOX	STR+TMP	DOX+TMP	AMP+CPR+STR	AMP+CPR+DOX	AMP+CPR+TMP	AMP+STR+DOX	AMP+STR+TMP	AMP+DOX+TMP	CPR+STR+DOX	CPR+STR+TMP	CPR+DOX+TMP	STR+DOX+TMP	AMP+CPR+STR+DOX	AMP+CPR+STR+TMP	AMP+CPR+DOX+TMP	AMP+STR+DOX+TMP	CPR+STR+DOX+TMP	AMP+CPR+STR+DOX+TMP
	91.69857937	86.21231979	93.55176933	99.04785156	88.11035156	91.30327363	55.3304509	101.9765287	101.1859172	72.5819135	103.8269987	100.2621232	91.03538663	94.60026212	93.96972656	38.72760964	95.45398394	88.14082767	37.18344657	43.07597282	91.10562075	60.87811271	80.1310616	93.55176933	66.50065531	27.26374305	40.70413836	88.53613342	32.63743051	58.15203145	33.82334775
	86.6118898	102.3271016	85.80206563	101.2874474	91.97821243	84.67858869	35.57274045	98.98501692	98.79168681	68.64949667	105.4647666	98.77108119	94.37835011	94.16917244	101.0893786	28.70952151	103.2382794	91.63847269	30.83615273	36.92605123	92.41179314	59.85096091	56.08576284	98.77108119	73.25140541	42.62928951	37.2160464	91.25181247	38.18269696	66.33546869	45.17883035
	92.37081677	104.3313402	94.53113607	100.0881501	96.6628888	95.65328888	68.36773945	95.03782536	99.34607001	74.46405133	113.8982062	99.66457635	96.86451801	93.1311069	104.5208412	48.46775228	101.8079241	92.57597128	37.7740736	42.72342608	81.70278241	63.90549803	57.37202858	105.264693	80.064168	36.54314656	37.56891909	102.8336966	37.97922811	63.90549803	36.33799205
AMP3CPR1STR1DOX1TMP1	AMP	CPR	STR	DOX	TMP	AMP+CPR	AMP+STR	AMP+DOX	AMP+TMP	CPR+STR	CPR+DOX	CPR+TMP	STR+DOX	STR+TMP	DOX+TMP	AMP+CPR+STR	AMP+CPR+DOX	AMP+CPR+TMP	AMP+STR+DOX	AMP+STR+TMP	AMP+DOX+TMP	CPR+STR+DOX	CPR+STR+TMP	CPR+DOX+TMP	STR+DOX+TMP	AMP+CPR+STR+DOX	AMP+CPR+STR+TMP	AMP+CPR+DOX+TMP	AMP+STR+DOX+TMP	CPR+STR+DOX+TMP	AMP+CPR+STR+DOX+TMP
	100.0524246	101.7822266	97.48535156	103.9306641	83.22753906	97.32634338	3.630406291	98.37483617	88.51900393	6.665039062	98.65722656	90.06347656	84.20410156	81.07910156	83.61816406	3.70904325	97.95543906	96.69724771	1.743119266	2.162516383	96.27785059	4.907226562	3.735351563	76.19628906	59.59472656	4.9672346	9.475753604	93.76146789	10.09174312	6.274414063	2.752293578
	81.40933455	102.4758604	89.60138648	102.6739292	87.62069819	95.42423846	5.961563603	105.4647666	105.255589	6.214409507	100.0990344	81.4805645	80.29215152	82.66897747	76.7269126	1.568832527	99.18943653	88.73055301	5.26866257	6.523728592	97.3068375	7.60089131	0.668482298	61.47561278	50.97796484	2.340175186	2.340175186	92.28657341	34.8280821	4.085169596	5.961563603
	101.7646201	101.4985518	86.58859086	98.47626244	85.17818914	101.9979583	15.93991541	104.3313402	101.0646055	5.188263443	98.47626244	78.93212442	67.85039668	82.15589976	85.17818914	3.937582033	94.29779787	88.9310194	3.106314715	3.470905644	89.63103398	3.097846619	0.113335852	75.70834907	59.18650044	3.470905644	3.120898352	88.46434301	4.637596617	3.979347689	3.820912936
AMP3CPR1STR1DOX1TMP2	AMP	CPR	STR	DOX	TMP	AMP+CPR	AMP+STR	AMP+DOX	AMP+TMP	CPR+STR	CPR+DOX	CPR+TMP	STR+DOX	STR+TMP	DOX+TMP	AMP+CPR+STR	AMP+CPR+DOX	AMP+CPR+TMP	AMP+STR+DOX	AMP+STR+TMP	AMP+DOX+TMP	CPR+STR+DOX	CPR+STR+TMP	CPR+DOX+TMP	STR+DOX+TMP	AMP+CPR+STR+DOX	AMP+CPR+STR+TMP	AMP+CPR+DOX+TMP	AMP+STR+DOX+TMP	CPR+STR+DOX+TMP	AMP+CPR+STR+DOX+TMP
	100.0524246	101.7822266	97.48535156	103.9306641	91.04003906	97.32634338	3.630406291	98.37483617	104.2463958	6.665039062	98.65722656	88.89160156	84.20410156	81.07910156	84.20410156	3.70904325	97.95543906	90.82568807	1.743119266	5.727391874	98.16513761	4.907226562	2.83203125	81.46972656	46.72851563	4.9672346	3.499344692	93.55176933	12.1887287	2.954101563	4.429882045
	81.40933455	102.4758604	89.60138648	102.6739292	93.36469423	95.42423846	5.961563603	105.4647666	107.5565433	6.214409507	100.0990344	85.83807873	80.29215152	81.08442684	80.49022035	1.568832527	99.18943653	101.9087462	5.26866257	7.778794614	96.26094914	7.60089131	1.312205992	69.00222827	56.77147809	2.340175186	1.921819846	84.54699961	16.56425677	15.17702402	7.634984965
	101.7646201	101.4985518	86.58859086	98.47626244	93.84208538	101.9979583	15.93991541	104.3313402	111.7981625	5.188263443	98.47626244	92.02871175	67.85039668	85.17818914	87.79750661	3.937582033	94.29779787	96.63117982	3.106314715	3.704243838	91.73107773	3.097846619	2.531167359	83.56630147	51.69374134	3.470905644	4.520927519	100.8312673	2.129211025	3.173403853	4.520927519
AMP3CPR1STR1DOX1TMP3	AMP	CPR	STR	DOX	TMP	AMP+CPR	AMP+STR	AMP+DOX	AMP+TMP	CPR+STR	CPR+DOX	CPR+TMP	STR+DOX	STR+TMP	DOX+TMP	AMP+CPR+STR	AMP+CPR+DOX	AMP+CPR+TMP	AMP+STR+DOX	AMP+STR+TMP	AMP+DOX+TMP	CPR+STR+DOX	CPR+STR+TMP	CPR+DOX+TMP	STR+DOX+TMP	AMP+CPR+STR+DOX	AMP+CPR+STR+TMP	AMP+CPR+DOX+TMP	AMP+STR+DOX+TMP	CPR+STR+DOX+TMP	AMP+CPR+STR+DOX+TMP
	100.0524246	101.7822266	97.48535156	103.9306641	88.11035156	97.32634338	3.630406291	98.37483617	103.8269987	6.665039062	98.65722656	89.08691406	84.20410156	86.54785156	94.36035156	3.70904325	97.95543906	93.76146789	1.743119266	7.365661861	101.310616	4.907226562	4.907226563	100.2197266	48.68164063	4.9672346	2.870249017	102.9882045	13.44692005	6.811523437	4.338138925
	81.40933455	102.4758604	89.60138648	102.6739292	91.97821243	95.42423846	5.961563603	105.4647666	106.9290103	6.214409507	100.0990344	82.47090864	80.29215152	84.45159693	99.10869027	1.568832527	99.18943653	95.00588312	5.26866257	19.91109949	106.0922996	7.60089131	2.104481307	98.11834613	1.262688784	2.340175186	1.987187868	91.44986273	15.10001307	6.412478336	4.288142241
	101.7646201	101.4985518	86.58859086	98.47626244	96.6628888	101.9979583	15.93991541	104.3313402	108.9981041	5.188263443	98.47626244	92.02871175	67.85039668	90.01385216	109.1550183	3.937582033	94.29779787	98.96456176	3.106314715	6.329298527	114.1315444	3.097846619	4.105276414	90.01385216	62.57398313	3.470905644	4.987603908	93.1311069	4.404258422	5.591235361	1.895872831
AMP3CPR1STR1DOX2TMP1	AMP	CPR	STR	DOX	TMP	AMP+CPR	AMP+STR	AMP+DOX	AMP+TMP	CPR+STR	CPR+DOX	CPR+TMP	STR+DOX	STR+TMP	DOX+TMP	AMP+CPR+STR	AMP+CPR+DOX	AMP+CPR+TMP	AMP+STR+DOX	AMP+STR+TMP	AMP+DOX+TMP	CPR+STR+DOX	CPR+STR+TMP	CPR+DOX+TMP	STR+DOX+TMP	AMP+CPR+STR+DOX	AMP+CPR+STR+TMP	AMP+CPR+DOX+TMP	AMP+STR+DOX+TMP	CPR+STR+DOX+TMP	AMP+CPR+STR+DOX+TMP

	100.0524246	101.7822266	97.48535156	100.6103516	83.22753906	97.32634338	3.630406291	92.50327654	88.51900393	6.665039062	96.50878906	90.06347656	90.45410156	81.07910156	83.81347656	3.70904325	86.42201835	96.69724771	4.259501966	2.162516383	105.2948886	33.69140625	3.735351563	90.06347656	53.34472656	4.338138925	9.475753604	81.59895151	12.39842726	2.63671875	6.854521625
	81.40933455	102.4758604	89.60138648	102.2777915	87.62069819	95.42423846	5.961563603	106.7198327	105.255589	6.214409507	99.90096559	81.4805645	92.37435009	82.66897747	84.84773459	1.568832527	88.52137534	88.73055301	5.26866257	6.523728592	106.3014773	5.224065363	0.668482298	85.24387225	56.37534043	2.340175186	2.340175186	82.24604523	15.30919074	3.441445902	6.170741273
	101.7646201	101.4985518	86.58859086	98.07329052	85.17818914	101.9979583	15.93991541	102.4646347	101.0646055	5.188263443	95.05100113	78.93212442	86.79007682	82.15589976	95.65545901	3.937582033	96.63117982	88.9310194	1.939623742	3.470905644	108.0647513	1.284472988	0.113335852	68.45485455	54.71603073	5.206358466	3.120898352	91.03106315	2.304214671	6.800151114	4.273005688
AMP3CPR1STR1DOX2TMP2	AMP	CPR	STR	DOX	TMP	AMP+CPR	AMP+STR	AMP+DOX	AMP+TMP	CPR+STR	CPR+DOX	CPR+TMP	STR+DOX	STR+TMP	DOX+TMP	AMP+CPR+STR	AMP+CPR+DOX	AMP+CPR+TMP	AMP+STR+DOX	AMP+STR+TMP	AMP+DOX+TMP	CPR+STR+DOX	CPR+STR+TMP	CPR+DOX+TMP	STR+DOX+TMP	AMP+CPR+STR+DOX	AMP+CPR+STR+TMP	AMP+CPR+DOX+TMP	AMP+STR+DOX+TMP	CPR+STR+DOX+TMP	AMP+CPR+STR+DOX+TMP
	100.0524246	101.7822266	97.48535156	100.6103516	91.04003906	97.32634338	3.630406291	92.50327654	104.2463958	6.665039062	96.50878906	88.89160156	90.45410156	81.07910156	88.69628906	3.70904325	86.42201835	90.82568807	4.259501966	5.727391874	105.9239843	33.69140625	2.83203125	96.89941406	50.24414063	4.338138925	3.499344692	105.5045872	8.623853211	3.881835938	5.806028834
	81.40933455	102.4758604	89.60138648	102.2777915	93.36469423	95.42423846	5.961563603	106.7198327	107.5565433	6.214409507	99.90096559	85.83807873	92.37435009	81.08442684	83.26318396	1.568832527	88.52137534	101.9087462	5.26866257	7.778794614	107.5565433	5.224065363	1.312205992	97.12800198	62.11933647	2.340175186	1.921819846	101.0720356	10.49810433	3.639514731	6.798274284
	101.7646201	101.4985518	86.58859086	98.07329052	93.84208538	101.9979583	15.93991541	102.4646347	111.7981625	5.188263443	95.05100113	92.02871175	86.79007682	85.17818914	96.6628888	3.937582033	96.63117982	96.63117982	1.939623742	3.704243838	107.598075	1.284472988	2.531167359	76.11132099	64.58884272	5.206358466	4.520927519	106.6647222	2.87297652	5.389749402	3.529240193
AMP3CPR1STR1DOX2TMP3	AMP	CPR	STR	DOX	TMP	AMP+CPR	AMP+STR	AMP+DOX	AMP+TMP	CPR+STR	CPR+DOX	CPR+TMP	STR+DOX	STR+TMP	DOX+TMP	AMP+CPR+STR	AMP+CPR+DOX	AMP+CPR+TMP	AMP+STR+DOX	AMP+STR+TMP	AMP+DOX+TMP	CPR+STR+DOX	CPR+STR+TMP	CPR+DOX+TMP	STR+DOX+TMP	AMP+CPR+STR+DOX	AMP+CPR+STR+TMP	AMP+CPR+DOX+TMP	AMP+STR+DOX+TMP	CPR+STR+DOX+TMP	AMP+CPR+STR+DOX+TMP
	100.0524246	101.7822266	97.48535156	100.6103516	88.11035156	97.32634338	3.630406291	92.50327654	103.8269987	6.665039062	96.50878906	89.08691406	90.45410156	86.54785156	94.55566406	3.70904325	86.42201835	93.76146789	4.259501966	7.365661861	104.2463958	33.69140625	4.907226563	96.89941406	50.83007813	4.338138925	2.870249017	89.77719528	6.526867628	4.858398438	5.05897772
	81.40933455	102.4758604	89.60138648	102.2777915	91.97821243	95.42423846	5.961563603	106.7198327	106.9290103	6.214409507	99.90096559	82.47090864	92.37435009	84.45159693	86.03614756	1.568832527	88.52137534	95.00588312	5.26866257	19.91109949	104.2097006	5.224065363	2.104481307	101.683585	54.39465214	2.340175186	1.987187868	85.38371029	11.12563734	5.025996534	3.242253889
	101.7646201	101.4985518	86.58859086	98.07329052	96.6628888	101.9979583	15.93991541	102.4646347	108.9981041	5.188263443	95.05100113	92.02871175	86.79007682	90.01385216	97.46883264	3.937582033	96.63117982	98.96456176	1.939623742	6.329298527	103.8646638	1.284472988	4.105276414	93.64059942	51.8952273	5.206358466	4.987603908	97.56453259	1.837538282	8.613524745	3.820912936
AMP3CPR1STR1DOX3TMP1	AMP	CPR	STR	DOX	TMP	AMP+CPR	AMP+STR	AMP+DOX	AMP+TMP	CPR+STR	CPR+DOX	CPR+TMP	STR+DOX	STR+TMP	DOX+TMP	AMP+CPR+STR	AMP+CPR+DOX	AMP+CPR+TMP	AMP+STR+DOX	AMP+STR+TMP	AMP+DOX+TMP	CPR+STR+DOX	CPR+STR+TMP	CPR+DOX+TMP	STR+DOX+TMP	AMP+CPR+STR+DOX	AMP+CPR+STR+TMP	AMP+CPR+DOX+TMP	AMP+STR+DOX+TMP	CPR+STR+DOX+TMP	AMP+CPR+STR+DOX+TMP
	100.0524246	101.7822266	97.48535156	99.04785156	83.22753906	97.32634338	3.630406291	93.76146789	88.51900393	6.665039062	102.1728516	90.06347656	81.86035156	81.07910156	79.51660156	3.70904325	97.11664482	96.69724771	4.259501966	2.162516383	88.72870249	8.374023438	3.735351563	75.41503906	43.01757813	6.22542595	9.475753604	86.21231979	4.010484928	8.374023438	3.80078637
	81.40933455	102.4758604	89.60138648	101.2874474	87.62069819	95.42423846	5.961563603	110.2758531	105.255589	6.214409507	102.2777915	81.4805645	87.22456053	82.66897747	82.47090864	1.568832527	90.61315205	88.73055301	5.477840241	6.523728592	108.8116094	0.829413221	0.668482298	80.68828918	60.13864818	2.758530527	2.340175186	89.77644136	10.07974899	1.856895271	2.549352857
	101.7646201	101.4985518	86.58859086	100.0881501	85.17818914	101.9979583	15.93991541	110.3981333	101.0646055	5.188263443	97.46883264	78.93212442	86.18561894	82.15589976	84.37224531	3.937582033	89.39769579	88.9310194	59.3408196	3.470905644	104.3313402	-0.528900642	0.113335852	99.68517819	47.90328674	6.606387633	3.120898352	103.6313257	6.270963978	5.389749402	5.43969666
AMP3CPR1STR1DOX3TMP2	AMP	CPR	STR	DOX	TMP	AMP+CPR	AMP+STR	AMP+DOX	AMP+TMP	CPR+STR	CPR+DOX	CPR+TMP	STR+DOX	STR+TMP	DOX+TMP	AMP+CPR+STR	AMP+CPR+DOX	AMP+CPR+TMP	AMP+STR+DOX	AMP+STR+TMP	AMP+DOX+TMP	CPR+STR+DOX	CPR+STR+TMP	CPR+DOX+TMP	STR+DOX+TMP	AMP+CPR+STR+DOX	AMP+CPR+STR+TMP	AMP+CPR+DOX+TMP	AMP+STR+DOX+TMP	CPR+STR+DOX+TMP	AMP+CPR+STR+DOX+TMP
	100.0524246	101.7822266	97.48535156	99.04785156	91.04003906	97.32634338	3.630406291	93.76146789	104.2463958	6.665039062	102.1728516	88.89160156	81.86035156	81.07910156	77.56347656	3.70904325	97.11664482	90.82568807	4.259501966	5.727391874	86.63171691	8.374023438	2.83203125	93.96972656	47.29003906	6.22542595	3.499344692	87.88990826	3.001310616	9.350585938	10.51114024
	81.40933455	102.4758604	89.60138648	101.2874474	93.36469423	95.42423846	5.961563603	110.2758531	107.5565433	6.214409507	102.2777915	85.83807873	87.22456053	81.08442684	85.83807873	1.568832527	90.61315205	101.9087462	5.477840241	7.778794614	104.8372336	0.829413221	1.312205992	81.08442684	51.57217133	2.758530527	1.921819846	105.883122	6.105373251	5.867789057	5.046411296
	101.7646201	101.4985518	86.58859086	100.0881501	93.84208538	101.9979583	15.93991541	110.3981333	111.7981625	5.188263443	97.46883264	92.02871175	86.18561894	85.17818914	95.65545901	3.937582033	89.39769579	96.63117982	59.3408196	3.704243838	105.7313694	-0.528900642	2.531167359	96.86437476	70.26822818	6.606387633	4.520927519	107.1313986	5.104273006	5.188263443	6.139711244
AMP3CPR1STR1DOX3TMP3	AMP	CPR	STR	DOX	TMP	AMP+CPR	AMP+STR	AMP+DOX	AMP+TMP	CPR+STR	CPR+DOX	CPR+TMP	STR+DOX	STR+TMP	DOX+TMP	AMP+CPR+STR	AMP+CPR+DOX	AMP+CPR+TMP	AMP+STR+DOX	AMP+STR+TMP	AMP+DOX+TMP	CPR+STR+DOX	CPR+STR+TMP	CPR+DOX+TMP	STR+DOX+TMP	AMP+CPR+STR+DOX	AMP+CPR+STR+TMP	AMP+CPR+DOX+TMP	AMP+STR+DOX+TMP	CPR+STR+DOX+TMP	AMP+CPR+STR+DOX+TMP
	100.0524246	101.7822266	97.48535156	99.04785156	88.11035156	97.32634338	3.630406291	93.76146789	103.8269987	6.665039062	102.1728516	89.08691406	81.86035156	86.54785156	93.96972656	3.70904325	97.11664482	93.76146789	4.259501966	7.365661861	97.11664482	8.374023438	4.907226563	94.16503906	52.00195313	6.22542595	2.870249017	107.1821756	54.79685452	14.42871094	7.575360419
	81.40933455	102.4758604	89.60138648	101.2874474	91.97821243	95.42423846	5.961563603	110.2758531	106.9290103	6.214409507	102.2777915	82.47090864	87.22456053	84.45159693	101.0893786	1.568832527	90.61315205	95.00588312	5.477840241	19.91109949	102.9546346	0.829413221	2.104481307	99.10869027	42.42386729	2.758530527	1.987187868	99.81696954	10.07974899	6.660064372	4.418878285
	101.7646201	101.4985518	86.58859086	100.0881501	96.6628888	101.9979583	15.93991541	110.3981333	108.9981041	5.188263443	97.46883264	92.02871175	86.18561894	90.01385216	104.5208412	3.937582033	89.39769579	98.96456176	59.3408196	6.329298527	109.2314423	-0.528900642	4.105276414	103.5134114	48.26848004	6.606387633	4.987603908	108.9981041	4.404258422	68.17781136	6.387633076
AMP3CPR1STR2DOX1TMP1	AMP	CPR	STR	DOX	TMP	AMP+CPR	AMP+STR	AMP+DOX	AMP+TMP	CPR+STR	CPR+DOX	CPR+TMP	STR+DOX	STR+TMP	DOX+TMP	AMP+CPR+STR	AMP+CPR+DOX	AMP+CPR+TMP	AMP+STR+DOX	AMP+STR+TMP	AMP+DOX+TMP	CPR+STR+DOX	CPR+STR+TMP	CPR+DOX+TMP	STR+DOX+TMP	AMP+CPR+STR+DOX	AMP+CPR+STR+TMP	AMP+CPR+DOX+TMP	AMP+STR+DOX+TMP	CPR+STR+DOX+TMP	AMP+CPR+STR+DOX+TMP
	100.0524246	101.7822266	46.67078443	103.9306641	83.22753906	97.32634338	44.07601573	98.37483617	88.51900393	56.29882813	98.65722656	90.06347656	45.07720815	49.20321186	83.61816406	46.80209699	97.95543906	96.69724771	5.688073394	21.42857143	96.27785059	43.60351563	50.09765625	76.19628906	3.347745522	5.806028834	28.76802097	93.76146789	11.55963303	7.250976562	3.840104849
	81.40933455	102.4758604	50.16916385	102.6739292	87.62069819	95.42423846	36.71068113	105.4647666	105.255589	56.28868532	100.0990344	81.4805645	3.28661189	54.03576607	76.7269126	3.582167604	99.18943653	88.73055301	4.431951889	7.778794614	97.3068375	39.45283486	0.037137905	61.47561278	2.319961334	6.170741273	4.209700614	92.28657341	14.8908354	4.629858876	3.582167604
	101.7646201	101.4985518	59.93076035	98.47626244	85.17818914	101.9979583	39.50707306	104.3313402	101.0646055	64.95403602	98.47626244	78.93212442	6.475189127	57.08424157	85.17818914	6.037625784	94.29779787	88.9310194	62.37421613	0.904185504	89.63103398	3.097846619	1.120765647	75.70834907	47.50609052	3.470905644	3.004229255	88.46434301	6.504302173	4.583805566	3.004229255
AMP3CPR1STR2DOX1TMP2	AMP	CPR	STR	DOX	TMP	AMP+CPR	AMP+STR	AMP+DOX	AMP+TMP	CPR+STR	CPR+DOX	CPR+TMP	STR+DOX	STR+TMP	DOX+TMP	AMP+CPR+STR	AMP+CPR+DOX	AMP+CPR+TMP	AMP+STR+DOX	AMP+STR+TMP	AMP+DOX+TMP	CPR+STR+DOX	CPR+STR+TMP	CPR+DOX+TMP	STR+DOX+TMP	AMP+CPR+STR+DOX	AMP+CPR+STR+TMP	AMP+CPR+DOX+TMP	AMP+STR+DOX+TMP	CPR+STR+DOX+TMP	AMP+CPR+STR+DOX+TMP
	100.0524246	101.7822266	46.67078443	103.9306641	91.04003906	97.32634338	44.07601573	98.37483617	104.2463958	56.29882813	98.65722656	88.89160156	45.07720815	63.03891291	84.20410156	46.80209699	97.95543906	90.82568807	5.688073394	3.211009174	98.16513761	43.60351563	50.09765625	81.46972656	5.126621371	5.806028834	12.22804718	93.55176933	13.23722149	6.665039062	3.001310616
	81.40933455	102.4758604	50.16916385	102.6739292	93.36469423	95.42423846	36.71068113	105.4647666	107.5565433	56.28868532	100.0990344	85.83807873	3.28661189	54.80908652	80.49022035	3.582167604	99.18943653	101.9087462	4.431951889	7.778794614	96.26094914	39.45283486	0.235206734	69.00222827	1.739971	6.170741273	4.418878285	84.54699961	14.68165773	5.422134192	4.837233625
	101.7646201	101.4985518	59.93076035	98.47626244	93.84208538	101.9979583	39.50707306	104.3313402	111.7981625	64.95403602	98.47626244	92.02871175	6.475189127	73.08629311	87.79750661	6.037625784	94.29779787	96.63117982	62.37421613	40.67376404	91.73107773	3.097846619	32.91776854	83.56630147	1.051416848	3.470905644	5.104273006	100.8312673	3.806329299	3.173403853	2.77089106
AMP3CPR1STR2DOX1TMP3	AMP	CPR	STR	DOX	TMP	AMP+CPR	AMP+STR	AMP+DOX	AMP+TMP	CPR+STR	CPR+DOX	CPR+TMP	STR+DOX	STR+TMP	DOX+TMP	AMP+CPR+STR	AMP+CPR+DOX	AMP+CPR+TMP	AMP+STR+DOX	AMP+STR+TMP	AMP+DOX+TMP	CPR+STR+DOX	CPR+STR+TMP	CPR+DOX+TMP	STR+DOX+TMP	AMP+CPR+STR+DOX	AMP+CPR+STR+TMP	AMP+CPR+DOX+TMP	AMP+STR+DOX+TMP	CPR+STR+DOX+TMP	AMP+CPR+STR+DOX+TMP
	100.0524246	101.7822266	46.67078443	103.9306641	88.11035156	97.32634338	44.07601573	98.37483617	103.8269987	56.29882813	98.65722656	89.08691406	45.07720815	69.75911056	94.36035156	46.80209699	97.95543906	93.76146789	5.688073394	22.0576671	101.310616	43.60351563	48.87695313	100.2197266	5.719579988	5.806028834	4.049803408	102.9882045	13.44692005	58.49609375	3.813892529
	81.40933455	102.4758604	50.16916385	102.6739292	91.97821243	95.42423846	36.71068113	105.4647666	106.9290103	56.28868532	100.0990344	82.47090864	3.28661189	54.71242146	99.10869027	3.582167604	99.18943653	95.00588312	4.431951889	51.27467643	106.0922996	39.45283486	13.10968061	98.11834613	3.093281779	6.170741273	2.536279252	91.44986273	13.84494705	2.847239416	4.209700614
	101.7646201	101.4985518	59.93076035	98.47626244	96.6628888	101.9979583	39.50707306	104.3313402	108.9981041	64.95403602	98.47626244	92.02871175	6.475189127	71.65021157	109.1550183	6.037625784	94.29779787	98.96456176	62.37421613	3.704243838	114.1315444	3.097846619	57.29756958	90.01385216	1.051416848	3.470905644	3.704243838	93.1311069	1.939623742	8.17277421	8.473093189
AMP3CPR1STR2DOX2TMP1	AMP	CPR	STR	DOX	TMP	AMP+CPR	AMP+STR	AMP+DOX	AMP+TMP	CPR+STR	CPR+DOX	CPR+TMP	STR+DOX	STR+TMP	DOX+TMP	AMP+CPR+STR	AMP+CPR+DOX	AMP+CPR+TMP	AMP+STR+DOX	AMP+STR+TMP	AMP+DOX+TMP	CPR+STR+DOX	CPR+STR+TMP	CPR+DOX+TMP	STR+DOX+TMP	AMP+CPR+STR+DOX	AMP+CPR+STR+TMP	AMP+CPR+DOX+TMP	AMP+STR+DOX+TMP	CPR+STR+DOX+TMP	AMP+CPR+STR+DOX+TMP
	100.0524246	101.7822266	46.67078443	100.6103516	83.22753906	97.32634338	44.07601573	92.50327654	88.51900393	56.29882813	96.50878906	90.06347656	61.6553428	49.20321186	83.81347656	46.80209699	86.42201835	96.69724771	5.701179554	21.42857143	105.2948886	55.51757813	50.09765625	90.06347656	5.324274243	4.338138925	28.76802097	81.59895151	13.02752294	7.32421875	3.171690695
	81.40933455	102.4758604	50.16916385	102.2777915	87.62069819	95.42423846	36.71068113	106.7198327	105.255589	56.28868532	99.90096559	81.4805645	48.5258579	54.03576607	84.84773459	3.582167604	88.52137534	88.73055301	5.0594849	7.778794614	106.3014773	42.82000495	0.037137905	85.24387225	39.37892702	2.327101582	4.209700614	82.24604523	13.21741404	3.243377074	5.464766636
	101.7646201	101.4985518	59.93076035	98.07329052	85.17818914	101.9979583	39.50707306	102.4646347	101.0646055	64.95403602	95.05100113	78.93212442	67.13681241	57.08424157	95.65545901	6.037625784	96.63117982	88.9310194	57.70745224	0.904185504	108.0647513	60.15615162	1.120765647	68.45485455	15.00192332	4.404258422	3.004229255	91.03106315	4.404258422	12.36620073	6.606387633
AMP3CPR1STR2DOX2TMP2	AMP	CPR	STR	DOX	TMP	AMP+CPR	AMP+STR	AMP+DOX	AMP+TMP	CPR+STR	CPR+DOX	CPR+TMP	STR+DOX	STR+TMP	DOX+TMP	AMP+CPR+STR	AMP+CPR+DOX	AMP+CPR+TMP	AMP+STR+DOX	AMP+STR+TMP	AMP+DOX+TMP	CPR+STR+DOX	CPR+STR+TMP	CPR+DOX+TMP	STR+DOX+TMP	AMP+CPR+STR+DOX	AMP+CPR+STR+TMP	AMP+CPR+DOX+TMP	AMP+STR+DOX+TMP	CPR+STR+DOX+TMP	AMP+CPR+STR+DOX+TMP
	100.0524246	101.7822266	46.67078443	100.6103516	91.04003906	97.32634338	44.07601573	92.50327654	104.2463958	56.29882813	96.50878906	88.89160156	61.6553428	63.03891291	88.69628906	46.80209699	86.42201835	90.82568807	5.701179554	3.211009174	105.9239843	55.51757813	50.09765625	96.89941406	12.43977764	4.338138925	12.22804718	105.5045872	63.99737877	17.87109375	3.171690695
	81.40933455	102.4758604	50.16916385	102.2777915	93.36469423	95.42423846	36.71068113	106.7198327	107.5565433	56.28868532	99.90096559	85.83807873	48.5258579	54.80908652	83.26318396	3.582167604	88.52137534	101.9087462	5.0594849	7.778794614	107.5565433	42.82000495	0.235206734	97.12800198	25.0362494	2.327101582	4.418878285	101.0720356	12.38070336	8.640752662	5.334030592
	101.7646201	101.4985518	59.93076035	98.07329052	93.84208538	101.9979583	39.50707306	102.4646347	111.7981625	64.95403602	95.05100113	92.02871175	67.13681241	73.08629311	96.6628888	6.037625784	96.63117982	96.63117982	57.70745224	40.67376404	107.598075	60.15615162	32.91776854	76.11132099	24.02872163	4.404258422	5.104273006	106.6647222	2.304214671	20.02266717	11.28773516
AMP3CPR1STR2DOX2TMP3	AMP	CPR	STR	DOX	TMP	AMP+CPR	AMP+STR	AMP+DOX	AMP+TMP	CPR+STR	CPR+DOX	CPR+TMP	STR+DOX	STR+TMP	DOX+TMP	AMP+CPR+STR	AMP+CPR+DOX	AMP+CPR+TMP	AMP+STR+DOX	AMP+STR+TMP	AMP+DOX+TMP	CPR+STR+DOX	CPR+STR+TMP	CPR+DOX+TMP	STR+DOX+TMP	AMP+CPR+STR+DOX	AMP+CPR+STR+TMP	AMP+CPR+DOX+TMP	AMP+STR+DOX+TMP	CPR+STR+DOX+TMP	AMP+CPR+STR+DOX+TMP
	100.0524246	101.7822266	46.67078443	100.6103516	88.11035156	97.32634338	44.07601573	92.50327654	103.8269987	56.29882813	96.50878906	89.08691406	61.6553428	69.75911056	94.55566406	46.80209699	86.42201835	93.76146789	5.701179554	22.0576671	104.2463958	55.51757813	48.87695313	96.89941406	13.23038913	4.338138925	4.049803408	89.77719528	7.365661861	28.61328125	6.22542595
	81.40933455	102.4758604	50.16916385	102.2777915	91.97821243	95.42423846	36.71068113	106.7198327	106.9290103	56.28868532	99.90096559	82.47090864	48.5258579	54.71242146	86.03614756	3.582167604	88.52137534	95.00588312	5.0594849	51.27467643	104.2097006	42.82000495	13.10968061	101.683585	24.45625906	2.327101582	2.536279252	85.38371029	9.870571317	16.27878188	10.35429468
	101.7646201	101.4985518	59.93076035	98.07329052	96.6628888	101.9979583	39.50707306	102.4646347	108.9981041	64.95403602	95.05100113	92.02871175	67.13681241	71.65021157	97.46883264	6.037625784	96.63117982	98.96456176	57.70745224	3.704243838	103.8646638	60.15615162	57.29756958	93.64059942	23.0029491	4.404258422	3.704243838	97.56453259	88.40600846	29.08953532	9.887705994
AMP3CPR1STR2DOX3TMP1	AMP	CPR	STR	DOX	TMP	AMP+CPR	AMP+STR	AMP+DOX	AMP+TMP	CPR+STR	CPR+DOX	CPR+TMP	STR+DOX	STR+TMP	DOX+TMP	AMP+CPR+STR	AMP+CPR+DOX	AMP+CPR+TMP	AMP+STR+DOX	AMP+STR+TMP	AMP+DOX+TMP	CPR+STR+DOX	CPR+STR+TMP	CPR+DOX+TMP	STR+DOX+TMP	AMP+CPR+STR+DOX	AMP+CPR+STR+TMP	AMP+CPR+DOX+TMP	AMP+STR+DOX+TMP	CPR+STR+DOX+TMP	AMP+CPR+STR+DOX+TMP
	100.0524246	101.7822266	46.67078443	99.04785156	83.22753906	97.32634338	44.07601573	93.76146789	88.51900393	56.29882813	102.1728516	90.06347656	62.44595429	49.20321186	79.51660156	46.80209699	97.11664482	96.69724771	21.42857143	21.42857143	88.72870249	58.44726563	50.09765625	75.41503906	12.0444719	6.015727392	28.76802097	86.21231979	33.38138925	14.23339844	6.854521625
	81.40933455	102.4758604	50.16916385	101.2874474	87.62069819	95.42423846	36.71068113	110.2758531	105.255589	56.28868532	102.2777915	81.4805645	54.22909618	54.03576607	82.47090864	3.582167604	90.61315205	88.73055301	48.215453	7.778794614	108.8116094	62.91161179	0.037137905	80.68828918	12.27646206	4.418878285	4.209700614	89.77644136	9.099228657	3.639514731	4.431951889
	101.7646201	101.4985518	59.93076035	100.0881501	85.17818914	101.9979583	39.50707306	110.3981333	101.0646055	64.95403602	97.46883264	78.93212442	58.93063213	57.08424157	84.37224531	6.037625784	89.39769579	88.9310194	34.37363278	0.904185504	104.3313402	68.82004785	1.120765647	99.68517819	28.13181177	21.54003208	3.004229255	103.6313257	49.07393904	20.62712505	7.087647659
AMP3CPR1STR2DOX3TMP2	AMP	CPR	STR	DOX	TMP	AMP+CPR	AMP+STR	AMP+DOX	AMP+TMP	CPR+STR	CPR+DOX	CPR+TMP	STR+DOX	STR+TMP	DOX+TMP	AMP+CPR+STR	AMP+CPR+DOX	AMP+CPR+TMP	AMP+STR+DOX	AMP+STR+TMP	AMP+DOX+TMP	CPR+STR+DOX	CPR+STR+TMP	CPR+DOX+TMP	STR+DOX+TMP	AMP+CPR+STR+DOX	AMP+CPR+STR+TMP	AMP+CPR+DOX+TMP	AMP+STR+DOX+TMP	CPR+STR+DOX+TMP	AMP+CPR+STR+DOX+TMP
	100.0524246	101.7822266	46.67078443	99.04785156	91.04003906	97.32634338	44.07601573	93.76146789	104.2463958	56.29882813	102.1728516	88.89160156	62.44595429	63.03891291	77.56347656	46.80209699	97.11664482	90.82568807	21.42857143	3.211009174	86.63171691	58.44726563	50.09765625	93.96972656	52.40271773	6.015727392	12.22804718	87.88990826	2.791612058	10.91308594	10.30144168
	81.40933455	102.4758604	50.16916385	101.2874474	93.36469423	95.42423846	36.71068113	110.2758531	107.5565433	56.28868532	102.2777915	85.83807873	54.22909618	54.80908652	85.83807873	3.582167604	90.61315205	101.9087462	48.215453	7.778794614	104.8372336	62.91161179	0.235206734	81.08442684	51.71580474	4.418878285	4.418878285	105.883122	4.431951889	1.708343649	3.595241208
	101.7646201	101.4985518	59.93076035	100.0881501	93.84208538	101.9979583	39.50707306	110.3981333	111.7981625	64.95403602	97.46883264	92.02871175	58.93063213	73.08629311	95.65545901	6.037625784	89.39769579	96.63117982	34.37363278	40.67376404	105.7313694	68.82004785	32.91776854	96.86437476	59.52045134	21.54003208	5.104273006	107.1313986	5.104273006	5.79272132	6.854309465
AMP3CPR1STR2DOX3TMP3	AMP	CPR	STR	DOX	TMP	AMP+CPR	AMP+STR	AMP+DOX	AMP+TMP	CPR+STR	CPR+DOX	CPR+TMP	STR+DOX	STR+TMP	DOX+TMP	AMP+CPR+STR	AMP+CPR+DOX	AMP+CPR+TMP	AMP+STR+DOX	AMP+STR+TMP	AMP+DOX+TMP	CPR+STR+DOX	CPR+STR+TMP	CPR+DOX+TMP	STR+DOX+TMP	AMP+CPR+STR+DOX	AMP+CPR+STR+TMP	AMP+CPR+DOX+TMP	AMP+STR+DOX+TMP	CPR+STR+DOX+TMP	AMP+CPR+STR+DOX+TMP
	100.0524246	101.7822266	46.67078443	99.04785156	88.11035156	97.32634338	44.07601573	93.76146789	103.8269987	56.29882813	102.1728516	89.08691406	62.44595429	69.75911056	93.96972656	46.80209699	97.11664482	93.76146789	21.42857143	22.0576671	97.11664482	58.44726563	48.87695313	94.16503906	45.28721433	6.015727392	4.049803408	107.1821756	5.897771953	68.65234375	22.0576671
	81.40933455	102.4758604	50.16916385	101.2874474	91.97821243	95.42423846	36.71068113	110.2758531	106.9290103	56.28868532	102.2777915	82.47090864	54.22909618	54.71242146	101.0893786	3.582167604	90.61315205	95.00588312	48.215453	51.27467643	102.9546346	62.91161179	13.10968061	99.10869027	52.13871435	4.418878285	2.536279252	99.81696954	6.523728592	50.14855162	41.31258988
	101.7646201	101.4985518	59.93076035	100.0881501	96.6628888	101.9979583	39.50707306	110.3981333	108.9981041	64.95403602	97.46883264	92.02871175	58.93063213	71.65021157	104.5208412	6.037625784	89.39769579	98.96456176	34.37363278	3.704243838	109.2314423	68.82004785	57.29756958	103.5134114	64.239005	21.54003208	3.704243838	108.9981041	7.437654951	43.19355245	7.554324048
AMP3CPR1STR3DOX1TMP1	AMP	CPR	STR	DOX	TMP	AMP+CPR	AMP+STR	AMP+DOX	AMP+TMP	CPR+STR	CPR+DOX	CPR+TMP	STR+DOX	STR+TMP	DOX+TMP	AMP+CPR+STR	AMP+CPR+DOX	AMP+CPR+TMP	AMP+STR+DOX	AMP+STR+TMP	AMP+DOX+TMP	CPR+STR+DOX	CPR+STR+TMP	CPR+DOX+TMP	STR+DOX+TMP	AMP+CPR+STR+DOX	AMP+CPR+STR+TMP	AMP+CPR+DOX+TMP	AMP+STR+DOX+TMP	CPR+STR+DOX+TMP	AMP+CPR+STR+DOX+TMP
	100.0524246	101.7822266	93.55176933	103.9306641	83.22753906	97.32634338	62.5163827	98.37483617	88.51900393	70.72753906	98.65722656	90.06347656	83.48623853	89.56749672	83.61816406	44.9017038	97.95543906	96.69724771	33.38138925	53.11926606	96.27785059	54.73632813	60.40039063	76.19628906	49.97378768	43.23722149	48.92529489	93.76146789	51.23197903	42.28515625	26.88073394
	81.40933455	102.4758604	85.80206563	102.6739292	87.62069819	95.42423846	61.74663355	105.4647666	105.255589	52.36444665	100.0990344	81.4805645	93.33246176	96.88848215	76.7269126	41.87475487	99.18943653	88.73055301	47.37874232	48.34618904	97.3068375	53.99851448	51.81975737	61.47561278	53.15727546	47.09112302	36.08314812	92.28657341	51.4838541	14.29809359	3.791345274
	101.7646201	101.4985518	94.53113607	98.47626244	85.17818914	101.9979583	74.23071314	104.3313402	101.0646055	48.1047727	98.47626244	78.93212442	92.43109231	96.63117982	85.17818914	57.89703952	94.29779787	88.9310194	37.64036751	56.672014	89.63103398	57.13386223	53.91008689	75.70834907	57.83870497	45.70511886	66.70555637	88.46434301	64.60551261	44.4024682	62.73880706
AMP3CPR1STR3DOX1TMP2	AMP	CPR	STR	DOX	TMP	AMP+CPR	AMP+STR	AMP+DOX	AMP+TMP	CPR+STR	CPR+DOX	CPR+TMP	STR+DOX	STR+TMP	DOX+TMP	AMP+CPR+STR	AMP+CPR+DOX	AMP+CPR+TMP	AMP+STR+DOX	AMP+STR+TMP	AMP+DOX+TMP	CPR+STR+DOX	CPR+STR+TMP	CPR+DOX+TMP	STR+DOX+TMP	AMP+CPR+STR+DOX	AMP+CPR+STR+TMP	AMP+CPR+DOX+TMP	AMP+STR+DOX+TMP	CPR+STR+DOX+TMP	AMP+CPR+STR+DOX+TMP
	100.0524246	101.7822266	93.55176933	103.9306641	91.04003906	97.32634338	62.5163827	98.37483617	104.2463958	70.72753906	98.65722656	88.89160156	83.48623853	88.30930537	84.20410156	44.9017038	97.95543906	90.82568807	33.38138925	52.49017038	98.16513761	54.73632813	56.10351563	81.46972656	42.1887287	43.23722149	53.11926606	93.55176933	37.57536042	49.85351563	41.97903014
	81.40933455	102.4758604	85.80206563	102.6739292	93.36469423	95.42423846	61.74663355	105.4647666	107.5565433	52.36444665	100.0990344	85.83807873	93.33246176	92.91410642	80.49022035	41.87475487	99.18943653	101.9087462	47.37874232	52.94809779	96.26094914	53.99851448	53.20623917	69.00222827	37.33821415	47.09112302	52.52974245	84.54699961	23.53248791	52.52537757	29.180285
	101.7646201	101.4985518	94.53113607	98.47626244	93.84208538	101.9979583	74.23071314	104.3313402	111.7981625	48.1047727	98.47626244	92.02871175	92.43109231	96.86451801	87.79750661	57.89703952	94.29779787	96.63117982	37.64036751	54.33863205	91.73107773	57.13386223	48.26848004	83.56630147	54.44071751	45.70511886	70.43896748	100.8312673	58.77205775	45.409898	25.2734432
AMP3CPR1STR3DOX1TMP3	AMP	CPR	STR	DOX	TMP	AMP+CPR	AMP+STR	AMP+DOX	AMP+TMP	CPR+STR	CPR+DOX	CPR+TMP	STR+DOX	STR+TMP	DOX+TMP	AMP+CPR+STR	AMP+CPR+DOX	AMP+CPR+TMP	AMP+STR+DOX	AMP+STR+TMP	AMP+DOX+TMP	CPR+STR+DOX	CPR+STR+TMP	CPR+DOX+TMP	STR+DOX+TMP	AMP+CPR+STR+DOX	AMP+CPR+STR+TMP	AMP+CPR+DOX+TMP	AMP+STR+DOX+TMP	CPR+STR+DOX+TMP	AMP+CPR+STR+DOX+TMP
	100.0524246	101.7822266	93.55176933	103.9306641	88.11035156	97.32634338	62.5163827	98.37483617	103.8269987	70.72753906	98.65722656	89.08691406	83.48623853	94.60026212	94.36035156	44.9017038	97.95543906	93.76146789	33.38138925	54.75753604	101.310616	54.73632813	50.80566406	100.2197266	56.68414155	43.23722149	51.86107471	102.9882045	46.40891219	62.54882813	22.47706422
	81.40933455	102.4758604	85.80206563	102.6739292	91.97821243	95.42423846	61.74663355	105.4647666	106.9290103	52.36444665	100.0990344	82.47090864	93.33246176	94.16917244	99.10869027	41.87475487	99.18943653	95.00588312	47.37874232	57.98143548	106.0922996	53.99851448	64.10002476	98.11834613	58.59589489	47.09112302	52.73892012	91.44986273	35.45561511	48.45258727	15.08693947
	101.7646201	101.4985518	94.53113607	98.47626244	96.6628888	101.9979583	74.23071314	104.3313402	108.9981041	48.1047727	98.47626244	92.02871175	92.43109231	93.1311069	109.1550183	57.89703952	94.29779787	98.96456176	37.64036751	49.67186816	114.1315444	57.13386223	62.37249717	90.01385216	45.93845705	45.70511886	61.33877789	93.1311069	67.63890914	46.45510641	24.34009042
AMP3CPR1STR3DOX2TMP1	AMP	CPR	STR	DOX	TMP	AMP+CPR	AMP+STR	AMP+DOX	AMP+TMP	CPR+STR	CPR+DOX	CPR+TMP	STR+DOX	STR+TMP	DOX+TMP	AMP+CPR+STR	AMP+CPR+DOX	AMP+CPR+TMP	AMP+STR+DOX	AMP+STR+TMP	AMP+DOX+TMP	CPR+STR+DOX	CPR+STR+TMP	CPR+DOX+TMP	STR+DOX+TMP	AMP+CPR+STR+DOX	AMP+CPR+STR+TMP	AMP+CPR+DOX+TMP	AMP+STR+DOX+TMP	CPR+STR+DOX+TMP	AMP+CPR+STR+DOX+TMP
	100.0524246	101.7822266	93.55176933	100.6103516	83.22753906	97.32634338	62.5163827	92.50327654	88.51900393	70.72753906	96.50878906	90.06347656	95.64875491	89.56749672	83.81347656	44.9017038	86.42201835	96.69724771	56.22542595	53.11926606	105.2948886	51.58691406	60.40039063	90.06347656	51.86107471	44.73132372	48.92529489	81.59895151	48.71559633	61.37695313	35.05897772
	81.40933455	102.4758604	85.80206563	102.2777915	87.62069819	95.42423846	61.74663355	106.7198327	105.255589	52.36444665	99.90096559	81.4805645	96.67930448	96.88848215	84.84773459	41.87475487	88.52137534	88.73055301	58.39979082	48.34618904	106.3014773	54.19658331	51.81975737	85.24387225	51.69303177	44.58099098	36.08314812	82.24604523	47.71865603	53.80044565	43.32592496
	101.7646201	101.4985518	94.53113607	98.07329052	85.17818914	101.9979583	74.23071314	102.4646347	101.0646055	48.1047727	95.05100113	78.93212442	99.19789996	96.63117982	95.65545901	57.89703952	96.63117982	88.9310194	72.83068397	56.672014	108.0647513	56.93237628	53.91008689	68.45485455	49.20519177	53.40527928	66.70555637	91.03106315	57.13869039	51.29076942	34.60697098
AMP3CPR1STR3DOX2TMP2	AMP	CPR	STR	DOX	TMP	AMP+CPR	AMP+STR	AMP+DOX	AMP+TMP	CPR+STR	CPR+DOX	CPR+TMP	STR+DOX	STR+TMP	DOX+TMP	AMP+CPR+STR	AMP+CPR+DOX	AMP+CPR+TMP	AMP+STR+DOX	AMP+STR+TMP	AMP+DOX+TMP	CPR+STR+DOX	CPR+STR+TMP	CPR+DOX+TMP	STR+DOX+TMP	AMP+CPR+STR+DOX	AMP+CPR+STR+TMP	AMP+CPR+DOX+TMP	AMP+STR+DOX+TMP	CPR+STR+DOX+TMP	AMP+CPR+STR+DOX+TMP
	100.0524246	101.7822266	93.55176933	100.6103516	91.04003906	97.32634338	62.5163827	92.50327654	104.2463958	70.72753906	96.50878906	88.89160156	95.64875491	88.30930537	88.69628906	44.9017038	86.42201835	90.82568807	56.22542595	52.49017038	105.9239843	51.58691406	56.10351563	96.89941406	59.82961992	44.73132372	53.11926606	105.5045872	48.29619921	61.76757813	4.049803408
	81.40933455	102.4758604	85.80206563	102.2777915	93.36469423	95.42423846	61.74663355	106.7198327	107.5565433	52.36444665	99.90096559	85.83807873	96.67930448	92.91410642	83.26318396	41.87475487	88.52137534	101.9087462	58.39979082	52.94809779	107.5565433	54.19658331	53.20623917	97.12800198	61.73355994	44.58099098	52.52974245	101.0720356	48.55536671	41.52017826	13.7011374
	101.7646201	101.4985518	94.53113607	98.07329052	93.84208538	101.9979583	74.23071314	102.4646347	111.7981625	48.1047727	95.05100113	92.02871175	99.19789996	96.86451801	96.6628888	57.89703952	96.63117982	96.63117982	72.83068397	54.33863205	107.598075	56.93237628	48.26848004	76.11132099	62.50546886	53.40527928	70.43896748	106.6647222	60.63876331	42.79058053	31.80691264
AMP3CPR1STR3DOX2TMP3	AMP	CPR	STR	DOX	TMP	AMP+CPR	AMP+STR	AMP+DOX	AMP+TMP	CPR+STR	CPR+DOX	CPR+TMP	STR+DOX	STR+TMP	DOX+TMP	AMP+CPR+STR	AMP+CPR+DOX	AMP+CPR+TMP	AMP+STR+DOX	AMP+STR+TMP	AMP+DOX+TMP	CPR+STR+DOX	CPR+STR+TMP	CPR+DOX+TMP	STR+DOX+TMP	AMP+CPR+STR+DOX	AMP+CPR+STR+TMP	AMP+CPR+DOX+TMP	AMP+STR+DOX+TMP	CPR+STR+DOX+TMP	AMP+CPR+STR+DOX+TMP
	100.0524246	101.7822266	93.55176933	100.6103516	88.11035156	97.32634338	62.5163827	92.50327654	103.8269987	70.72753906	96.50878906	89.08691406	95.64875491	94.60026212	94.55566406	44.9017038	86.42201835	93.76146789	56.22542595	54.75753604	104.2463958	51.58691406	50.80566406	96.89941406	70.90432503	44.73132372	51.86107471	89.77719528	45.95019659	52.39257813	49.76408912
	81.40933455	102.4758604	85.80206563	102.2777915	91.97821243	95.42423846	61.74663355	106.7198327	106.9290103	52.36444665	99.90096559	82.47090864	96.67930448	94.16917244	86.03614756	41.87475487	88.52137534	95.00588312	58.39979082	57.98143548	104.2097006	54.19658331	64.10002476	101.683585	67.81278599	44.58099098	52.73892012	85.38371029	45.62687933	48.45258727	32.86704144
	101.7646201	101.4985518	94.53113607	98.07329052	96.6628888	101.9979583	74.23071314	102.4646347	108.9981041	48.1047727	95.05100113	92.02871175	99.19789996	93.1311069	97.46883264	57.89703952	96.63117982	98.96456176	72.83068397	49.67186816	103.8646638	56.93237628	62.37249717	93.64059942	70.73064022	53.40527928	61.33877789	97.56453259	55.50532303	44.44024682	42.26338049
AMP3CPR1STR3DOX3TMP1	AMP	CPR	STR	DOX	TMP	AMP+CPR	AMP+STR	AMP+DOX	AMP+TMP	CPR+STR	CPR+DOX	CPR+TMP	STR+DOX	STR+TMP	DOX+TMP	AMP+CPR+STR	AMP+CPR+DOX	AMP+CPR+TMP	AMP+STR+DOX	AMP+STR+TMP	AMP+DOX+TMP	CPR+STR+DOX	CPR+STR+TMP	CPR+DOX+TMP	STR+DOX+TMP	AMP+CPR+STR+DOX	AMP+CPR+STR+TMP	AMP+CPR+DOX+TMP	AMP+STR+DOX+TMP	CPR+STR+DOX+TMP	AMP+CPR+STR+DOX+TMP
	100.0524246	101.7822266	93.55176933	99.04785156	83.22753906	97.32634338	62.5163827	93.76146789	88.51900393	70.72753906	102.1728516	90.06347656	91.03538663	89.56749672	79.51660156	44.9017038	97.11664482	96.69724771	50.81258191	53.11926606	88.72870249	55.32226563	60.40039063	75.41503906	72.16251638	52.9095675	48.92529489	86.21231979	51.86107471	46.19140625	35.68807339
	81.40933455	102.4758604	85.80206563	101.2874474	87.62069819	95.42423846	61.74663355	110.2758531	105.255589	52.36444665	102.2777915	81.4805645	94.37835011	96.88848215	82.47090864	41.87475487	90.61315205	88.73055301	43.95345797	48.34618904	108.8116094	60.9309235	51.81975737	80.68828918	74.50647143	45.41770166	36.08314812	89.77644136	51.06549876	42.70859124	44.03189959
	101.7646201	101.4985518	94.53113607	100.0881501	85.17818914	101.9979583	74.23071314	110.3981333	101.0646055	48.1047727	97.46883264	78.93212442	96.86451801	96.63117982	84.37224531	57.89703952	89.39769579	88.9310194	46.69680618	56.672014	104.3313402	51.08928347	53.91008689	99.68517819	78.66413884	59.23873414	66.70555637	103.6313257	64.83885081	42.02241531	28.07350153
AMP3CPR1STR3DOX3TMP2	AMP	CPR	STR	DOX	TMP	AMP+CPR	AMP+STR	AMP+DOX	AMP+TMP	CPR+STR	CPR+DOX	CPR+TMP	STR+DOX	STR+TMP	DOX+TMP	AMP+CPR+STR	AMP+CPR+DOX	AMP+CPR+TMP	AMP+STR+DOX	AMP+STR+TMP	AMP+DOX+TMP	CPR+STR+DOX	CPR+STR+TMP	CPR+DOX+TMP	STR+DOX+TMP	AMP+CPR+STR+DOX	AMP+CPR+STR+TMP	AMP+CPR+DOX+TMP	AMP+STR+DOX+TMP	CPR+STR+DOX+TMP	AMP+CPR+STR+DOX+TMP
	100.0524246	101.7822266	93.55176933	99.04785156	91.04003906	97.32634338	62.5163827	93.76146789	104.2463958	70.72753906	102.1728516	88.89160156	91.03538663	88.30930537	77.56347656	44.9017038	97.11664482	90.82568807	50.81258191	52.49017038	86.63171691	55.32226563	56.10351563	93.96972656	72.37221494	52.9095675	53.11926606	87.88990826	50.3931848	59.61914063	36.76277851
	81.40933455	102.4758604	85.80206563	101.2874474	93.36469423	95.42423846	61.74663355	110.2758531	107.5565433	52.36444665	102.2777915	85.83807873	94.37835011	92.91410642	85.83807873	41.87475487	90.61315205	101.9087462	43.95345797	52.94809779	104.8372336	60.9309235	53.20623917	81.08442684	85.38371029	45.41770166	52.52974245	105.883122	50.43796575	65.68457539	48.97372206
	101.7646201	101.4985518	94.53113607	100.0881501	93.84208538	101.9979583	74.23071314	110.3981333	111.7981625	48.1047727	97.46883264	92.02871175	96.86451801	96.86451801	95.65545901	57.89703952	89.39769579	96.63117982	46.69680618	54.33863205	105.7313694	51.08928347	48.26848004	96.86437476	97.09785621	59.23873414	70.43896748	107.1313986	58.07204317	53.50711497	49.43852997
AMP3CPR1STR3DOX3TMP3	AMP	CPR	STR	DOX	TMP	AMP+CPR	AMP+STR	AMP+DOX	AMP+TMP	CPR+STR	CPR+DOX	CPR+TMP	STR+DOX	STR+TMP	DOX+TMP	AMP+CPR+STR	AMP+CPR+DOX	AMP+CPR+TMP	AMP+STR+DOX	AMP+STR+TMP	AMP+DOX+TMP	CPR+STR+DOX	CPR+STR+TMP	CPR+DOX+TMP	STR+DOX+TMP	AMP+CPR+STR+DOX	AMP+CPR+STR+TMP	AMP+CPR+DOX+TMP	AMP+STR+DOX+TMP	CPR+STR+DOX+TMP	AMP+CPR+STR+DOX+TMP
	100.0524246	101.7822266	93.55176933	99.04785156	88.11035156	97.32634338	62.5163827	93.76146789	103.8269987	70.72753906	102.1728516	89.08691406	91.03538663	94.60026212	93.96972656	44.9017038	97.11664482	93.76146789	50.81258191	54.75753604	97.11664482	55.32226563	50.80566406	94.16503906	66.50065531	52.9095675	51.86107471	107.1821756	53.07994758	58.44726563	50.81258191
	81.40933455	102.4758604	85.80206563	101.2874474	91.97821243	95.42423846	61.74663355	110.2758531	106.9290103	52.36444665	102.2777915	82.47090864	94.37835011	94.16917244	101.0893786	41.87475487	90.61315205	95.00588312	43.95345797	57.98143548	102.9546346	60.9309235	64.10002476	99.10869027	73.25140541	45.41770166	52.73892012	99.81696954	46.88194535	63.90195593	52.73892012
	101.7646201	101.4985518	94.53113607	100.0881501	96.6628888	101.9979583	74.23071314	110.3981333	108.9981041	48.1047727	97.46883264	92.02871175	96.86451801	93.1311069	104.5208412	57.89703952	89.39769579	98.96456176	46.69680618	49.67186816	109.2314423	51.08928347	62.37249717	103.5134114	80.064168	59.23873414	61.33877789	108.9981041	51.77191191	57.73832011	58.30538136
AMP3CPR2STR1DOX1TMP1	AMP	CPR	STR	DOX	TMP	AMP+CPR	AMP+STR	AMP+DOX	AMP+TMP	CPR+STR	CPR+DOX	CPR+TMP	STR+DOX	STR+TMP	DOX+TMP	AMP+CPR+STR	AMP+CPR+DOX	AMP+CPR+TMP	AMP+STR+DOX	AMP+STR+TMP	AMP+DOX+TMP	CPR+STR+DOX	CPR+STR+TMP	CPR+DOX+TMP	STR+DOX+TMP	AMP+CPR+STR+DOX	AMP+CPR+STR+TMP	AMP+CPR+DOX+TMP	AMP+STR+DOX+TMP	CPR+STR+DOX+TMP	AMP+CPR+STR+DOX+TMP
	100.0524246	104.1507103	97.48535156	103.9306641	83.22753906	96.90694626	3.630406291	98.37483617	88.51900393	9.894996912	104.3483632	89.3267449	84.20410156	81.07910156	83.61816406	5.386631717	101.1009174	103.197903	1.743119266	2.162516383	96.27785059	34.40395306	4.360716492	78.85114268	59.59472656	5.281782438	2.765399738	100.2621232	10.09174312	4.928968499	2.581913499
	81.40933455	81.77863702	89.60138648	102.6739292	87.62069819	97.09765982	5.961563603	105.4647666	105.255589	4.386176897	103.8182697	92.41179314	80.29215152	82.66897747	76.7269126	3.45143156	103.3729899	95.21506079	5.26866257	6.523728592	97.3068375	2.126631223	3.866602223	76.1720638	50.97796484	3.033076219	2.536279252	100.6536802	34.8280821	6.959884002	0.86285789
	101.7646201	103.2440056	86.58859086	98.47626244	85.17818914	103.8646638	15.93991541	104.3313402	101.0646055	9.462751635	102.2182331	87.4471086	67.85039668	82.15589976	85.17818914	6.270963978	107.3647368	109.6981187	3.106314715	3.470905644	89.63103398	3.923579946	1.487370176	81.90793691	59.18650044	7.437654951	7.204316757	106.6647222	4.637596617	4.359533273	7.90433134
AMP3CPR2STR1DOX1TMP2	AMP	CPR	STR	DOX	TMP	AMP+CPR	AMP+STR	AMP+DOX	AMP+TMP	CPR+STR	CPR+DOX	CPR+TMP	STR+DOX	STR+TMP	DOX+TMP	AMP+CPR+STR	AMP+CPR+DOX	AMP+CPR+TMP	AMP+STR+DOX	AMP+STR+TMP	AMP+DOX+TMP	CPR+STR+DOX	CPR+STR+TMP	CPR+DOX+TMP	STR+DOX+TMP	AMP+CPR+STR+DOX	AMP+CPR+STR+TMP	AMP+CPR+DOX+TMP	AMP+STR+DOX+TMP	CPR+STR+DOX+TMP	AMP+CPR+STR+DOX+TMP
	100.0524246	104.1507103	97.48535156	103.9306641	91.04003906	96.90694626	3.630406291	98.37483617	104.2463958	9.894996912	104.3483632	90.90796788	84.20410156	81.07910156	84.20410156	5.386631717	101.1009174	96.48754915	1.743119266	5.727391874	98.16513761	34.40395306	3.15009265	82.80420012	46.72851563	5.281782438	1.952817824	102.9882045	12.1887287	4.731315627	2.765399738
	81.40933455	81.77863702	89.60138648	102.6739292	93.36469423	97.09765982	5.961563603	105.4647666	107.5565433	4.386176897	103.8182697	91.25181247	80.29215152	81.08442684	80.49022035	3.45143156	103.3729899	94.58752778	5.26866257	7.778794614	96.26094914	2.126631223	3.866602223	85.25857902	56.77147809	3.033076219	11.54399268	100.2353249	16.56425677	6.186563557	8.197149954
	101.7646201	103.2440056	86.58859086	98.47626244	93.84208538	103.8646638	15.93991541	104.3313402	111.7981625	9.462751635	102.2182331	99.1409155	67.85039668	85.17818914	87.79750661	6.270963978	107.3647368	102.2312965	3.106314715	3.704243838	91.73107773	3.923579946	2.513142711	80.26670086	51.69374134	7.437654951	8.837684118	97.79787079	2.129211025	4.56468778	6.970978562
AMP3CPR2STR1DOX1TMP3	AMP	CPR	STR	DOX	TMP	AMP+CPR	AMP+STR	AMP+DOX	AMP+TMP	CPR+STR	CPR+DOX	CPR+TMP	STR+DOX	STR+TMP	DOX+TMP	AMP+CPR+STR	AMP+CPR+DOX	AMP+CPR+TMP	AMP+STR+DOX	AMP+STR+TMP	AMP+DOX+TMP	CPR+STR+DOX	CPR+STR+TMP	CPR+DOX+TMP	STR+DOX+TMP	AMP+CPR+STR+DOX	AMP+CPR+STR+TMP	AMP+CPR+DOX+TMP	AMP+STR+DOX+TMP	CPR+STR+DOX+TMP	AMP+CPR+STR+DOX+TMP
	100.0524246	104.1507103	97.48535156	103.9306641	88.11035156	96.90694626	3.630406291	98.37483617	103.8269987	9.894996912	104.3483632	90.90796788	84.20410156	86.54785156	94.36035156	5.386631717	101.1009174	95.2293578	1.743119266	7.365661861	101.310616	34.40395306	2.359481161	107.3131563	48.68164063	5.281782438	2.581913499	98.58453473	13.44692005	4.731315627	2.55570118
	81.40933455	81.77863702	89.60138648	102.6739292	91.97821243	97.09765982	5.961563603	105.4647666	106.9290103	4.386176897	103.8182697	91.25181247	80.29215152	84.45159693	99.10869027	3.45143156	103.3729899	94.58752778	5.26866257	19.91109949	106.0922996	2.126631223	4.059932334	104.2049299	1.262688784	3.033076219	4.209700614	89.14890835	15.10001307	5.799903335	46.88194535
	101.7646201	103.2440056	86.58859086	98.47626244	96.6628888	103.8646638	15.93991541	104.3313402	108.9981041	9.462751635	102.2182331	97.70483395	67.85039668	90.01385216	109.1550183	6.270963978	107.3647368	102.9313111	3.106314715	6.329298527	114.1315444	3.923579946	4.33388896	106.5264778	62.57398313	7.437654951	10.47105148	104.7980166	4.404258422	3.744069753	3.004229255
AMP3CPR2STR1DOX2TMP1	AMP	CPR	STR	DOX	TMP	AMP+CPR	AMP+STR	AMP+DOX	AMP+TMP	CPR+STR	CPR+DOX	CPR+TMP	STR+DOX	STR+TMP	DOX+TMP	AMP+CPR+STR	AMP+CPR+DOX	AMP+CPR+TMP	AMP+STR+DOX	AMP+STR+TMP	AMP+DOX+TMP	CPR+STR+DOX	CPR+STR+TMP	CPR+DOX+TMP	STR+DOX+TMP	AMP+CPR+STR+DOX	AMP+CPR+STR+TMP	AMP+CPR+DOX+TMP	AMP+STR+DOX+TMP	CPR+STR+DOX+TMP	AMP+CPR+STR+DOX+TMP
	100.0524246	104.1507103	97.48535156	100.6103516	83.22753906	96.90694626	3.630406291	92.50327654	88.51900393	9.894996912	98.22112415	89.3267449	90.45410156	81.07910156	83.81347656	5.386631717	99.84272608	103.197903	4.259501966	2.162516383	105.2948886	6.114885732	4.360716492	95.84928968	53.34472656	4.652686763	2.765399738	87.26081258	12.39842726	3.940704138	6.566186107
	81.40933455	81.77863702	89.60138648	102.2777915	87.62069819	97.09765982	5.961563603	106.7198327	105.255589	4.386176897	98.21169647	92.41179314	92.37435009	82.66897747	84.84773459	3.45143156	105.4647666	95.21506079	5.26866257	6.523728592	106.3014773	3.866602223	3.866602223	81.19864669	56.37534043	5.543208263	2.536279252	80.78180154	15.30919074	6.379893668	8.890050987
	101.7646201	103.2440056	86.58859086	98.07329052	85.17818914	103.8646638	15.93991541	102.4646347	101.0646055	9.462751635	101.1924606	87.4471086	86.79007682	82.15589976	95.65545901	6.270963978	106.6647222	109.6981187	1.939623742	3.470905644	108.0647513	7.706116169	1.487370176	96.2687524	54.71603073	9.537698702	7.204316757	109.4647805	2.304214671	3.538915246	-0.160420009
AMP3CPR2STR1DOX2TMP2	AMP	CPR	STR	DOX	TMP	AMP+CPR	AMP+STR	AMP+DOX	AMP+TMP	CPR+STR	CPR+DOX	CPR+TMP	STR+DOX	STR+TMP	DOX+TMP	AMP+CPR+STR	AMP+CPR+DOX	AMP+CPR+TMP	AMP+STR+DOX	AMP+STR+TMP	AMP+DOX+TMP	CPR+STR+DOX	CPR+STR+TMP	CPR+DOX+TMP	STR+DOX+TMP	AMP+CPR+STR+DOX	AMP+CPR+STR+TMP	AMP+CPR+DOX+TMP	AMP+STR+DOX+TMP	CPR+STR+DOX+TMP	AMP+CPR+STR+DOX+TMP
	100.0524246	104.1507103	97.48535156	100.6103516	91.04003906	96.90694626	3.630406291	92.50327654	104.2463958	9.894996912	98.22112415	90.90796788	90.45410156	81.07910156	88.69628906	5.386631717	99.84272608	96.48754915	4.259501966	5.727391874	105.9239843	6.114885732	3.15009265	94.66337245	50.24414063	4.652686763	1.952817824	100.4718218	8.623853211	2.779493515	6.566186107
	81.40933455	81.77863702	89.60138648	102.2777915	93.36469423	97.09765982	5.961563603	106.7198327	107.5565433	4.386176897	98.21169647	91.25181247	92.37435009	81.08442684	83.26318396	3.45143156	105.4647666	94.58752778	5.26866257	7.778794614	107.5565433	3.866602223	3.866602223	80.42532624	62.11933647	5.543208263	11.54399268	107.5565433	10.49810433	6.959884002	47.37874232
	101.7646201	103.2440056	86.58859086	98.07329052	93.84208538	103.8646638	15.93991541	102.4646347	111.7981625	9.462751635	101.1924606	99.1409155	86.79007682	85.17818914	96.6628888	6.270963978	106.6647222	102.2312965	1.939623742	3.704243838	107.598075	7.706116169	2.513142711	98.52545198	64.58884272	9.537698702	8.837684118	111.5648243	2.87297652	1.282215669	1.706285548
AMP3CPR2STR1DOX2TMP3	AMP	CPR	STR	DOX	TMP	AMP+CPR	AMP+STR	AMP+DOX	AMP+TMP	CPR+STR	CPR+DOX	CPR+TMP	STR+DOX	STR+TMP	DOX+TMP	AMP+CPR+STR	AMP+CPR+DOX	AMP+CPR+TMP	AMP+STR+DOX	AMP+STR+TMP	AMP+DOX+TMP	CPR+STR+DOX	CPR+STR+TMP	CPR+DOX+TMP	STR+DOX+TMP	AMP+CPR+STR+DOX	AMP+CPR+STR+TMP	AMP+CPR+DOX+TMP	AMP+STR+DOX+TMP	CPR+STR+DOX+TMP	AMP+CPR+STR+DOX+TMP
	100.0524246	104.1507103	97.48535156	100.6103516	88.11035156	96.90694626	3.630406291	92.50327654	103.8269987	9.894996912	98.22112415	90.90796788	90.45410156	86.54785156	94.55566406	5.386631717	99.84272608	95.2293578	4.259501966	7.365661861	104.2463958	6.114885732	2.359481161	91.89623224	50.83007813	4.652686763	2.581913499	97.11664482	6.526867628	1.766522545	3.840104849
	81.40933455	81.77863702	89.60138648	102.2777915	91.97821243	97.09765982	5.961563603	106.7198327	106.9290103	4.386176897	98.21169647	91.25181247	92.37435009	84.45159693	86.03614756	3.45143156	105.4647666	94.58752778	5.26866257	19.91109949	104.2097006	3.866602223	4.059932334	86.99855002	54.39465214	5.543208263	4.209700614	106.7198327	11.12563734	6.766553891	1.490390901
	101.7646201	103.2440056	86.58859086	98.07329052	96.6628888	103.8646638	15.93991541	102.4646347	108.9981041	9.462751635	101.1924606	97.70483395	86.79007682	90.01385216	97.46883264	6.270963978	106.6647222	102.9313111	1.939623742	6.329298527	103.8646638	7.706116169	4.33388896	92.98628029	51.8952273	9.537698702	10.47105148	103.6313257	1.837538282	10.16797025	1.472947353
AMP3CPR2STR1DOX3TMP1	AMP	CPR	STR	DOX	TMP	AMP+CPR	AMP+STR	AMP+DOX	AMP+TMP	CPR+STR	CPR+DOX	CPR+TMP	STR+DOX	STR+TMP	DOX+TMP	AMP+CPR+STR	AMP+CPR+DOX	AMP+CPR+TMP	AMP+STR+DOX	AMP+STR+TMP	AMP+DOX+TMP	CPR+STR+DOX	CPR+STR+TMP	CPR+DOX+TMP	STR+DOX+TMP	AMP+CPR+STR+DOX	AMP+CPR+STR+TMP	AMP+CPR+DOX+TMP	AMP+STR+DOX+TMP	CPR+STR+DOX+TMP	AMP+CPR+STR+DOX+TMP
	100.0524246	104.1507103	97.48535156	99.04785156	83.22753906	96.90694626	3.630406291	93.76146789	88.51900393	9.894996912	102.3718345	89.3267449	81.86035156	81.07910156	79.51660156	5.386631717	103.6173001	103.197903	4.259501966	2.162516383	88.72870249	8.906732551	4.360716492	94.07041384	43.01757813	3.604193971	2.765399738	105.5045872	4.010484928	1.964175417	2.162516383
	81.40933455	81.77863702	89.60138648	101.2874474	87.62069819	97.09765982	5.961563603	110.2758531	105.255589	4.386176897	102.0782987	92.41179314	87.22456053	82.66897747	82.47090864	3.45143156	107.9748987	95.21506079	5.477840241	6.523728592	108.8116094	3.093281779	3.866602223	84.67858869	60.13864818	2.405543208	2.536279252	110.2758531	10.07974899	6.57322378	2.745456922
	101.7646201	103.2440056	86.58859086	100.0881501	85.17818914	103.8646638	15.93991541	110.3981333	101.0646055	9.462751635	103.4491601	87.4471086	86.18561894	82.15589976	84.37224531	6.270963978	112.7315152	109.6981187	59.3408196	3.470905644	104.3313402	1.461725862	1.487370176	91.34504424	47.90328674	7.204316757	7.204316757	91.96441593	6.270963978	14.06590589	0.20417092
AMP3CPR2STR1DOX3TMP2	AMP	CPR	STR	DOX	TMP	AMP+CPR	AMP+STR	AMP+DOX	AMP+TMP	CPR+STR	CPR+DOX	CPR+TMP	STR+DOX	STR+TMP	DOX+TMP	AMP+CPR+STR	AMP+CPR+DOX	AMP+CPR+TMP	AMP+STR+DOX	AMP+STR+TMP	AMP+DOX+TMP	CPR+STR+DOX	CPR+STR+TMP	CPR+DOX+TMP	STR+DOX+TMP	AMP+CPR+STR+DOX	AMP+CPR+STR+TMP	AMP+CPR+DOX+TMP	AMP+STR+DOX+TMP	CPR+STR+DOX+TMP	AMP+CPR+STR+DOX+TMP
	100.0524246	104.1507103	97.48535156	99.04785156	91.04003906	96.90694626	3.630406291	93.76146789	104.2463958	9.894996912	102.3718345	90.90796788	81.86035156	81.07910156	77.56347656	5.386631717	103.6173001	96.48754915	4.259501966	5.727391874	86.63171691	8.906732551	3.15009265	94.46571958	47.29003906	3.604193971	1.952817824	103.8269987	3.001310616	0.975911056	1.743119266
	81.40933455	81.77863702	89.60138648	101.2874474	93.36469423	97.09765982	5.961563603	110.2758531	107.5565433	4.386176897	102.0782987	91.25181247	87.22456053	81.08442684	85.83807873	3.45143156	107.9748987	94.58752778	5.477840241	7.778794614	104.8372336	3.093281779	3.866602223	85.06524891	51.57217133	2.405543208	11.54399268	107.765721	6.105373251	7.733204447	7.216629625
	101.7646201	103.2440056	86.58859086	100.0881501	93.84208538	103.8646638	15.93991541	110.3981333	111.7981625	9.462751635	103.4491601	99.1409155	86.18561894	85.17818914	95.65545901	6.270963978	112.7315152	102.2312965	59.3408196	3.704243838	105.7313694	1.461725862	2.513142711	89.08834466	70.26822818	7.204316757	8.837684118	98.26454718	5.104273006	12.42466983	12.92110252
AMP3CPR2STR1DOX3TMP3	AMP	CPR	STR	DOX	TMP	AMP+CPR	AMP+STR	AMP+DOX	AMP+TMP	CPR+STR	CPR+DOX	CPR+TMP	STR+DOX	STR+TMP	DOX+TMP	AMP+CPR+STR	AMP+CPR+DOX	AMP+CPR+TMP	AMP+STR+DOX	AMP+STR+TMP	AMP+DOX+TMP	CPR+STR+DOX	CPR+STR+TMP	CPR+DOX+TMP	STR+DOX+TMP	AMP+CPR+STR+DOX	AMP+CPR+STR+TMP	AMP+CPR+DOX+TMP	AMP+STR+DOX+TMP	CPR+STR+DOX+TMP	AMP+CPR+STR+DOX+TMP
	100.0524246	104.1507103	97.48535156	99.04785156	88.11035156	96.90694626	3.630406291	93.76146789	103.8269987	9.894996912	102.3718345	90.90796788	81.86035156	86.54785156	93.96972656	5.386631717	103.6173001	95.2293578	4.259501966	7.365661861	97.11664482	8.906732551	2.359481161	95.25633107	52.00195313	3.604193971	2.581913499	104.0366972	54.79685452	7.91846819	8.833551769
	81.40933455	81.77863702	89.60138648	101.2874474	91.97821243	97.09765982	5.961563603	110.2758531	106.9290103	4.386176897	102.0782987	91.25181247	87.22456053	84.45159693	101.0893786	3.45143156	107.9748987	94.58752778	5.477840241	19.91109949	102.9546346	3.093281779	4.059932334	97.0517158	42.42386729	2.405543208	4.209700614	109.2299647	10.07974899	3.28661189	10.69420839
	101.7646201	103.2440056	86.58859086	100.0881501	96.6628888	103.8646638	15.93991541	110.3981333	108.9981041	9.462751635	103.4491601	97.70483395	86.18561894	90.01385216	104.5208412	6.270963978	112.7315152	102.9313111	59.3408196	6.329298527	109.2314423	1.461725862	4.33388896	99.34607001	48.26848004	7.204316757	10.47105148	107.8314132	4.404258422	2.28234389	7.787662243
AMP3CPR2STR2DOX1TMP1	AMP	CPR	STR	DOX	TMP	AMP+CPR	AMP+STR	AMP+DOX	AMP+TMP	CPR+STR	CPR+DOX	CPR+TMP	STR+DOX	STR+TMP	DOX+TMP	AMP+CPR+STR	AMP+CPR+DOX	AMP+CPR+TMP	AMP+STR+DOX	AMP+STR+TMP	AMP+DOX+TMP	CPR+STR+DOX	CPR+STR+TMP	CPR+DOX+TMP	STR+DOX+TMP	AMP+CPR+STR+DOX	AMP+CPR+STR+TMP	AMP+CPR+DOX+TMP	AMP+STR+DOX+TMP	CPR+STR+DOX+TMP	AMP+CPR+STR+DOX+TMP
	100.0524246	104.1507103	46.67078443	103.9306641	83.22753906	96.90694626	44.07601573	98.37483617	88.51900393	46.26312539	104.3483632	89.3267449	45.07720815	49.20321186	83.61816406	4.259501966	101.1009174	103.197903	5.688073394	21.42857143	96.27785059	53.77393453	4.360716492	78.85114268	3.347745522	3.840104849	2.765399738	100.2621232	11.55963303	2.977146387	2.581913499
	81.40933455	81.77863702	50.16916385	102.6739292	87.62069819	97.09765982	36.71068113	105.4647666	105.255589	12.69937168	103.8182697	92.41179314	3.28661189	54.03576607	76.7269126	3.033076219	103.3729899	95.21506079	4.431951889	7.778794614	97.3068375	31.06573224	1.739971	76.1720638	2.319961334	49.88887436	11.19100536	100.6536802	14.8908354	6.186563557	2.758530527
	101.7646201	103.2440056	59.93076035	98.47626244	85.17818914	103.8646638	39.50707306	104.3313402	101.0646055	46.39056289	102.2182331	87.4471086	6.475189127	57.08424157	85.17818914	8.239754995	107.3647368	109.6981187	62.37421613	0.904185504	89.63103398	45.45454545	10.98858828	81.90793691	47.50609052	8.706431384	5.3376112	106.6647222	6.504302173	12.83497884	8.837684118
AMP3CPR2STR2DOX1TMP2	AMP	CPR	STR	DOX	TMP	AMP+CPR	AMP+STR	AMP+DOX	AMP+TMP	CPR+STR	CPR+DOX	CPR+TMP	STR+DOX	STR+TMP	DOX+TMP	AMP+CPR+STR	AMP+CPR+DOX	AMP+CPR+TMP	AMP+STR+DOX	AMP+STR+TMP	AMP+DOX+TMP	CPR+STR+DOX	CPR+STR+TMP	CPR+DOX+TMP	STR+DOX+TMP	AMP+CPR+STR+DOX	AMP+CPR+STR+TMP	AMP+CPR+DOX+TMP	AMP+STR+DOX+TMP	CPR+STR+DOX+TMP	AMP+CPR+STR+DOX+TMP
	100.0524246	104.1507103	46.67078443	103.9306641	91.04003906	96.90694626	44.07601573	98.37483617	104.2463958	46.26312539	104.3483632	90.90796788	45.07720815	63.03891291	84.20410156	4.259501966	101.1009174	96.48754915	5.688073394	3.211009174	98.16513761	53.77393453	12.46448425	82.80420012	5.126621371	3.840104849	1.743119266	102.9882045	13.23722149	3.940704138	4.652686763
	81.40933455	81.77863702	50.16916385	102.6739292	93.36469423	97.09765982	36.71068113	105.4647666	107.5565433	12.69937168	103.8182697	91.25181247	3.28661189	54.80908652	80.49022035	3.033076219	103.3729899	94.58752778	4.431951889	7.778794614	96.26094914	31.06573224	4.253262446	85.25857902	1.739971	49.88887436	4.641129559	100.2353249	14.68165773	6.766553891	5.0594849
	101.7646201	103.2440056	59.93076035	98.47626244	93.84208538	103.8646638	39.50707306	104.3313402	111.7981625	46.39056289	102.2182331	99.1409155	6.475189127	73.08629311	87.79750661	8.239754995	107.3647368	102.2312965	62.37421613	40.67376404	91.73107773	45.45454545	45.45454545	80.26670086	1.051416848	8.706431384	6.970978562	97.79787079	3.806329299	5.769970509	6.270963978
AMP3CPR2STR2DOX1TMP3	AMP	CPR	STR	DOX	TMP	AMP+CPR	AMP+STR	AMP+DOX	AMP+TMP	CPR+STR	CPR+DOX	CPR+TMP	STR+DOX	STR+TMP	DOX+TMP	AMP+CPR+STR	AMP+CPR+DOX	AMP+CPR+TMP	AMP+STR+DOX	AMP+STR+TMP	AMP+DOX+TMP	CPR+STR+DOX	CPR+STR+TMP	CPR+DOX+TMP	STR+DOX+TMP	AMP+CPR+STR+DOX	AMP+CPR+STR+TMP	AMP+CPR+DOX+TMP	AMP+STR+DOX+TMP	CPR+STR+DOX+TMP	AMP+CPR+STR+DOX+TMP
	100.0524246	104.1507103	46.67078443	103.9306641	88.11035156	96.90694626	44.07601573	98.37483617	103.8269987	46.26312539	104.3483632	90.90796788	45.07720815	69.75911056	94.36035156	4.259501966	101.1009174	95.2293578	5.688073394	22.0576671	101.310616	53.77393453	56.73872761	107.3131563	5.719579988	3.840104849	12.83093054	98.58453473	13.44692005	4.138357011	3.840104849
	81.40933455	81.77863702	50.16916385	102.6739292	91.97821243	97.09765982	36.71068113	105.4647666	106.9290103	12.69937168	103.8182697	91.25181247	3.28661189	54.71242146	99.10869027	3.033076219	103.3729899	94.58752778	4.431951889	51.27467643	106.0922996	31.06573224	3.612856452	104.2049299	3.093281779	49.88887436	2.327101582	89.14890835	13.84494705	9.473175447	1.778010197
	101.7646201	103.2440056	59.93076035	98.47626244	96.6628888	103.8646638	39.50707306	104.3313402	108.9981041	46.39056289	102.2182331	97.70483395	6.475189127	71.65021157	109.1550183	8.239754995	107.3647368	102.9313111	62.37421613	3.704243838	114.1315444	45.45454545	65.76484165	106.5264778	1.051416848	8.706431384	7.437654951	104.7980166	1.939623742	5.975125016	8.371007729
AMP3CPR2STR2DOX2TMP1	AMP	CPR	STR	DOX	TMP	AMP+CPR	AMP+STR	AMP+DOX	AMP+TMP	CPR+STR	CPR+DOX	CPR+TMP	STR+DOX	STR+TMP	DOX+TMP	AMP+CPR+STR	AMP+CPR+DOX	AMP+CPR+TMP	AMP+STR+DOX	AMP+STR+TMP	AMP+DOX+TMP	CPR+STR+DOX	CPR+STR+TMP	CPR+DOX+TMP	STR+DOX+TMP	AMP+CPR+STR+DOX	AMP+CPR+STR+TMP	AMP+CPR+DOX+TMP	AMP+STR+DOX+TMP	CPR+STR+DOX+TMP	AMP+CPR+STR+DOX+TMP
	100.0524246	104.1507103	46.67078443	100.6103516	83.22753906	96.90694626	44.07601573	92.50327654	88.51900393	46.26312539	98.22112415	89.3267449	61.6553428	49.20321186	83.81347656	4.259501966	99.84272608	103.197903	5.701179554	21.42857143	105.2948886	51.61210624	4.360716492	95.84928968	5.324274243	3.420707733	2.765399738	87.26081258	13.02752294	2.557134033	6.775884666
	81.40933455	81.77863702	50.16916385	102.2777915	87.62069819	97.09765982	36.71068113	106.7198327	105.255589	12.69937168	98.21169647	92.41179314	48.5258579	54.03576607	84.84773459	3.033076219	105.4647666	95.21506079	5.0594849	7.778794614	106.3014773	51.75205413	1.739971	81.19864669	39.37892702	2.614720879	11.19100536	80.78180154	13.21741404	6.959884002	9.308406328
	101.7646201	103.2440056	59.93076035	98.07329052	85.17818914	103.8646638	39.50707306	102.4646347	101.0646055	46.39056289	101.1924606	87.4471086	67.13681241	57.08424157	95.65545901	8.239754995	106.6647222	109.6981187	57.70745224	0.904185504	108.0647513	54.59674317	10.98858828	96.2687524	15.00192332	59.57415779	5.3376112	109.4647805	4.404258422	4.33388896	0.670847309
AMP3CPR2STR2DOX2TMP2	AMP	CPR	STR	DOX	TMP	AMP+CPR	AMP+STR	AMP+DOX	AMP+TMP	CPR+STR	CPR+DOX	CPR+TMP	STR+DOX	STR+TMP	DOX+TMP	AMP+CPR+STR	AMP+CPR+DOX	AMP+CPR+TMP	AMP+STR+DOX	AMP+STR+TMP	AMP+DOX+TMP	CPR+STR+DOX	CPR+STR+TMP	CPR+DOX+TMP	STR+DOX+TMP	AMP+CPR+STR+DOX	AMP+CPR+STR+TMP	AMP+CPR+DOX+TMP	AMP+STR+DOX+TMP	CPR+STR+DOX+TMP	AMP+CPR+STR+DOX+TMP
	100.0524246	104.1507103	46.67078443	100.6103516	91.04003906	96.90694626	44.07601573	92.50327654	104.2463958	46.26312539	98.22112415	90.90796788	61.6553428	63.03891291	88.69628906	4.259501966	99.84272608	96.48754915	5.701179554	3.211009174	105.9239843	51.61210624	12.46448425	94.66337245	12.43977764	3.420707733	1.743119266	100.4718218	63.99737877	47.84434836	6.566186107
	81.40933455	81.77863702	50.16916385	102.2777915	93.36469423	97.09765982	36.71068113	106.7198327	107.5565433	12.69937168	98.21169647	91.25181247	48.5258579	54.80908652	83.26318396	3.033076219	105.4647666	94.58752778	5.0594849	7.778794614	107.5565433	51.75205413	4.253262446	80.42532624	25.0362494	2.614720879	4.641129559	107.5565433	12.38070336	15.21266312	-0.797489868
	101.7646201	103.2440056	59.93076035	98.07329052	93.84208538	103.8646638	39.50707306	102.4646347	111.7981625	46.39056289	101.1924606	99.1409155	67.13681241	73.08629311	96.6628888	8.239754995	106.6647222	102.2312965	57.70745224	40.67376404	107.598075	54.59674317	45.45454545	98.52545198	24.02872163	59.57415779	6.970978562	111.5648243	2.304214671	13.56584177	0.437509115
AMP3CPR2STR2DOX2TMP3	AMP	CPR	STR	DOX	TMP	AMP+CPR	AMP+STR	AMP+DOX	AMP+TMP	CPR+STR	CPR+DOX	CPR+TMP	STR+DOX	STR+TMP	DOX+TMP	AMP+CPR+STR	AMP+CPR+DOX	AMP+CPR+TMP	AMP+STR+DOX	AMP+STR+TMP	AMP+DOX+TMP	CPR+STR+DOX	CPR+STR+TMP	CPR+DOX+TMP	STR+DOX+TMP	AMP+CPR+STR+DOX	AMP+CPR+STR+TMP	AMP+CPR+DOX+TMP	AMP+STR+DOX+TMP	CPR+STR+DOX+TMP	AMP+CPR+STR+DOX+TMP
	100.0524246	104.1507103	46.67078443	100.6103516	88.11035156	96.90694626	44.07601573	92.50327654	103.8269987	46.26312539	98.22112415	90.90796788	61.6553428	69.75911056	94.55566406	4.259501966	99.84272608	95.2293578	5.701179554	22.0576671	104.2463958	51.61210624	56.73872761	91.89623224	13.23038913	3.420707733	12.83093054	97.11664482	7.365661861	11.87152563	6.356487549
	81.40933455	81.77863702	50.16916385	102.2777915	91.97821243	97.09765982	36.71068113	106.7198327	106.9290103	12.69937168	98.21169647	91.25181247	48.5258579	54.71242146	86.03614756	3.033076219	105.4647666	94.58752778	5.0594849	51.27467643	104.2097006	51.75205413	3.612856452	86.99855002	24.45625906	2.614720879	2.327101582	106.7198327	9.870571317	10.63315611	10.48503072
	101.7646201	103.2440056	59.93076035	98.07329052	96.6628888	103.8646638	39.50707306	102.4646347	108.9981041	46.39056289	101.1924606	97.70483395	67.13681241	71.65021157	97.46883264	8.239754995	106.6647222	102.9313111	57.70745224	3.704243838	103.8646638	54.59674317	65.76484165	92.98628029	23.0029491	59.57415779	7.437654951	103.6313257	88.40600846	57.96897038	1.837538282
AMP3CPR2STR2DOX3TMP1	AMP	CPR	STR	DOX	TMP	AMP+CPR	AMP+STR	AMP+DOX	AMP+TMP	CPR+STR	CPR+DOX	CPR+TMP	STR+DOX	STR+TMP	DOX+TMP	AMP+CPR+STR	AMP+CPR+DOX	AMP+CPR+TMP	AMP+STR+DOX	AMP+STR+TMP	AMP+DOX+TMP	CPR+STR+DOX	CPR+STR+TMP	CPR+DOX+TMP	STR+DOX+TMP	AMP+CPR+STR+DOX	AMP+CPR+STR+TMP	AMP+CPR+DOX+TMP	AMP+STR+DOX+TMP	CPR+STR+DOX+TMP	AMP+CPR+STR+DOX+TMP
	100.0524246	104.1507103	46.67078443	99.04785156	83.22753906	96.90694626	44.07601573	93.76146789	88.51900393	46.26312539	102.3718345	89.3267449	62.44595429	49.20321186	79.51660156	4.259501966	103.6173001	103.197903	21.42857143	21.42857143	88.72870249	44.69425571	4.360716492	94.07041384	12.0444719	45.96330275	2.765399738	105.5045872	33.38138925	3.174799259	8.453473132
	81.40933455	81.77863702	50.16916385	101.2874474	87.62069819	97.09765982	36.71068113	110.2758531	105.255589	12.69937168	102.0782987	92.41179314	54.22909618	54.03576607	82.47090864	3.033076219	107.9748987	95.21506079	48.215453	7.778794614	108.8116094	58.67568874	1.739971	84.67858869	12.27646206	10.77265002	11.19100536	110.2758531	9.099228657	8.506524891	4.418878285
	101.7646201	103.2440056	59.93076035	100.0881501	85.17818914	103.8646638	39.50707306	110.3981333	101.0646055	46.39056289	103.4491601	87.4471086	58.93063213	57.08424157	84.37224531	8.239754995	112.7315152	109.6981187	34.37363278	0.904185504	104.3313402	54.80189768	10.98858828	91.34504424	28.13181177	57.94079043	5.3376112	91.96441593	49.07393904	42.78753686	70.07437655
AMP3CPR2STR2DOX3TMP2	AMP	CPR	STR	DOX	TMP	AMP+CPR	AMP+STR	AMP+DOX	AMP+TMP	CPR+STR	CPR+DOX	CPR+TMP	STR+DOX	STR+TMP	DOX+TMP	AMP+CPR+STR	AMP+CPR+DOX	AMP+CPR+TMP	AMP+STR+DOX	AMP+STR+TMP	AMP+DOX+TMP	CPR+STR+DOX	CPR+STR+TMP	CPR+DOX+TMP	STR+DOX+TMP	AMP+CPR+STR+DOX	AMP+CPR+STR+TMP	AMP+CPR+DOX+TMP	AMP+STR+DOX+TMP	CPR+STR+DOX+TMP	AMP+CPR+STR+DOX+TMP
	100.0524246	104.1507103	46.67078443	99.04785156	91.04003906	96.90694626	44.07601573	93.76146789	104.2463958	46.26312539	102.3718345	90.90796788	62.44595429	63.03891291	77.56347656	4.259501966	103.6173001	96.48754915	21.42857143	3.211009174	86.63171691	44.69425571	12.46448425	94.46571958	52.40271773	45.96330275	1.743119266	103.8269987	2.791612058	6.93020383	7.404980341
	81.40933455	81.77863702	50.16916385	101.2874474	93.36469423	97.09765982	36.71068113	110.2758531	107.5565433	12.69937168	102.0782987	91.25181247	54.22909618	54.80908652	85.83807873	3.033076219	107.9748987	94.58752778	48.215453	7.778794614	104.8372336	58.67568874	4.253262446	85.06524891	51.71580474	10.77265002	4.641129559	107.765721	4.431951889	5.606573224	6.092299647
	101.7646201	103.2440056	59.93076035	100.0881501	93.84208538	103.8646638	39.50707306	110.3981333	111.7981625	46.39056289	103.4491601	99.1409155	58.93063213	73.08629311	95.65545901	8.239754995	112.7315152	102.2312965	34.37363278	40.67376404	105.7313694	54.80189768	45.45454545	89.08834466	59.52045134	57.94079043	6.970978562	98.26454718	5.104273006	24.52878574	20.27125565
AMP3CPR2STR2DOX3TMP3	AMP	CPR	STR	DOX	TMP	AMP+CPR	AMP+STR	AMP+DOX	AMP+TMP	CPR+STR	CPR+DOX	CPR+TMP	STR+DOX	STR+TMP	DOX+TMP	AMP+CPR+STR	AMP+CPR+DOX	AMP+CPR+TMP	AMP+STR+DOX	AMP+STR+TMP	AMP+DOX+TMP	CPR+STR+DOX	CPR+STR+TMP	CPR+DOX+TMP	STR+DOX+TMP	AMP+CPR+STR+DOX	AMP+CPR+STR+TMP	AMP+CPR+DOX+TMP	AMP+STR+DOX+TMP	CPR+STR+DOX+TMP	AMP+CPR+STR+DOX+TMP
	100.0524246	104.1507103	46.67078443	99.04785156	88.11035156	96.90694626	44.07601573	93.76146789	103.8269987	46.26312539	102.3718345	90.90796788	62.44595429	69.75911056	93.96972656	4.259501966	103.6173001	95.2293578	21.42857143	22.0576671	97.11664482	44.69425571	56.73872761	95.25633107	45.28721433	45.96330275	12.83093054	104.0366972	5.897771953	58.51760346	4.639580603
	81.40933455	81.77863702	50.16916385	101.2874474	91.97821243	97.09765982	36.71068113	110.2758531	106.9290103	12.69937168	102.0782987	91.25181247	54.22909618	54.71242146	101.0893786	3.033076219	107.9748987	94.58752778	48.215453	51.27467643	102.9546346	58.67568874	3.612856452	97.0517158	52.13871435	10.77265002	2.327101582	109.2299647	6.523728592	0.193330111	46.1236763
	101.7646201	103.2440056	59.93076035	100.0881501	96.6628888	103.8646638	39.50707306	110.3981333	108.9981041	46.39056289	103.4491601	97.70483395	58.93063213	71.65021157	104.5208412	8.239754995	112.7315152	102.9313111	34.37363278	3.704243838	109.2314423	54.80189768	65.76484165	99.34607001	64.239005	57.94079043	7.437654951	107.8314132	7.437654951	6.475189127	8.954353216
AMP3CPR2STR3DOX1TMP1	AMP	CPR	STR	DOX	TMP	AMP+CPR	AMP+STR	AMP+DOX	AMP+TMP	CPR+STR	CPR+DOX	CPR+TMP	STR+DOX	STR+TMP	DOX+TMP	AMP+CPR+STR	AMP+CPR+DOX	AMP+CPR+TMP	AMP+STR+DOX	AMP+STR+TMP	AMP+DOX+TMP	CPR+STR+DOX	CPR+STR+TMP	CPR+DOX+TMP	STR+DOX+TMP	AMP+CPR+STR+DOX	AMP+CPR+STR+TMP	AMP+CPR+DOX+TMP	AMP+STR+DOX+TMP	CPR+STR+DOX+TMP	AMP+CPR+STR+DOX+TMP
	100.0524246	104.1507103	93.55176933	103.9306641	83.22753906	96.90694626	62.5163827	98.37483617	88.51900393	61.26003706	104.3483632	89.3267449	83.48623853	89.56749672	83.61816406	48.25688073	101.1009174	103.197903	33.38138925	53.11926606	96.27785059	48.44966028	42.91537986	78.85114268	49.97378768	46.82830931	55.4259502	100.2621232	51.23197903	41.32180358	48.26998689
	81.40933455	81.77863702	85.80206563	102.6739292	87.62069819	97.09765982	61.74663355	105.4647666	105.255589	58.48235863	103.8182697	92.41179314	93.33246176	96.88848215	76.7269126	40.20133351	103.3729899	95.21506079	47.37874232	48.34618904	97.3068375	54.42242629	58.86901885	76.1720638	53.15727546	42.69839195	33.28539678	100.6536802	51.4838541	5.413243113	41.94012289
	101.7646201	103.2440056	94.53113607	98.47626244	85.17818914	103.8646638	74.23071314	104.3313402	101.0646055	66.52134889	102.2182331	87.4471086	92.43109231	96.63117982	85.17818914	56.73034855	107.3647368	109.6981187	37.64036751	56.672014	89.63103398	58.2895243	53.16066162	81.90793691	57.83870497	54.33863205	66.23887998	106.6647222	64.60551261	43.40300038	69.03893831
AMP3CPR2STR3DOX1TMP2	AMP	CPR	STR	DOX	TMP	AMP+CPR	AMP+STR	AMP+DOX	AMP+TMP	CPR+STR	CPR+DOX	CPR+TMP	STR+DOX	STR+TMP	DOX+TMP	AMP+CPR+STR	AMP+CPR+DOX	AMP+CPR+TMP	AMP+STR+DOX	AMP+STR+TMP	AMP+DOX+TMP	CPR+STR+DOX	CPR+STR+TMP	CPR+DOX+TMP	STR+DOX+TMP	AMP+CPR+STR+DOX	AMP+CPR+STR+TMP	AMP+CPR+DOX+TMP	AMP+STR+DOX+TMP	CPR+STR+DOX+TMP	AMP+CPR+STR+DOX+TMP
	100.0524246	104.1507103	93.55176933	103.9306641	91.04003906	96.90694626	62.5163827	98.37483617	104.2463958	61.26003706	104.3483632	90.90796788	83.48623853	88.30930537	84.20410156	48.25688073	101.1009174	96.48754915	33.38138925	52.49017038	98.16513761	48.44966028	52.79802347	82.80420012	42.1887287	46.82830931	29.81651376	102.9882045	37.57536042	48.04200124	42.1887287
	81.40933455	81.77863702	85.80206563	102.6739292	93.36469423	97.09765982	61.74663355	105.4647666	107.5565433	58.48235863	103.8182697	91.25181247	93.33246176	92.91410642	80.49022035	40.20133351	103.3729899	94.58752778	47.37874232	52.94809779	96.26094914	54.42242629	34.35234413	85.25857902	37.33821415	42.69839195	26.46097529	100.2353249	23.53248791	46.91880135	25.20590927
	101.7646201	103.2440056	94.53113607	98.47626244	93.84208538	103.8646638	74.23071314	104.3313402	111.7981625	66.52134889	102.2182331	99.1409155	92.43109231	96.86451801	87.79750661	56.73034855	107.3647368	102.2312965	37.64036751	54.33863205	91.73107773	58.2895243	53.77612514	80.26670086	54.44071751	54.33863205	44.07175149	97.79787079	58.77205775	52.95550712	37.53828205
AMP3CPR2STR3DOX1TMP3	AMP	CPR	STR	DOX	TMP	AMP+CPR	AMP+STR	AMP+DOX	AMP+TMP	CPR+STR	CPR+DOX	CPR+TMP	STR+DOX	STR+TMP	DOX+TMP	AMP+CPR+STR	AMP+CPR+DOX	AMP+CPR+TMP	AMP+STR+DOX	AMP+STR+TMP	AMP+DOX+TMP	CPR+STR+DOX	CPR+STR+TMP	CPR+DOX+TMP	STR+DOX+TMP	AMP+CPR+STR+DOX	AMP+CPR+STR+TMP	AMP+CPR+DOX+TMP	AMP+STR+DOX+TMP	CPR+STR+DOX+TMP	AMP+CPR+STR+DOX+TMP
	100.0524246	104.1507103	93.55176933	103.9306641	88.11035156	96.90694626	62.5163827	98.37483617	103.8269987	61.26003706	104.3483632	90.90796788	83.48623853	94.60026212	94.36035156	48.25688073	101.1009174	95.2293578	33.38138925	54.75753604	101.310616	48.44966028	53.19332922	107.3131563	56.68414155	46.82830931	41.16644823	98.58453473	46.40891219	44.49660284	35.47837484
	81.40933455	81.77863702	85.80206563	102.6739292	91.97821243	97.09765982	61.74663355	105.4647666	106.9290103	58.48235863	103.8182697	91.25181247	93.33246176	94.16917244	99.10869027	40.20133351	103.3729899	94.58752778	47.37874232	57.98143548	106.0922996	54.42242629	58.09569841	104.2049299	58.59589489	42.69839195	40.81579291	89.14890835	35.45561511	59.09859836	14.53784808
	101.7646201	103.2440056	94.53113607	98.47626244	96.6628888	103.8646638	74.23071314	104.3313402	108.9981041	66.52134889	102.2182331	97.70483395	92.43109231	93.1311069	109.1550183	56.73034855	107.3647368	102.9313111	37.64036751	49.67186816	114.1315444	58.2895243	52.95550712	106.5264778	45.93845705	54.33863205	52.70526469	104.7980166	67.63890914	50.17309912	31.10689806
AMP3CPR2STR3DOX2TMP1	AMP	CPR	STR	DOX	TMP	AMP+CPR	AMP+STR	AMP+DOX	AMP+TMP	CPR+STR	CPR+DOX	CPR+TMP	STR+DOX	STR+TMP	DOX+TMP	AMP+CPR+STR	AMP+CPR+DOX	AMP+CPR+TMP	AMP+STR+DOX	AMP+STR+TMP	AMP+DOX+TMP	CPR+STR+DOX	CPR+STR+TMP	CPR+DOX+TMP	STR+DOX+TMP	AMP+CPR+STR+DOX	AMP+CPR+STR+TMP	AMP+CPR+DOX+TMP	AMP+STR+DOX+TMP	CPR+STR+DOX+TMP	AMP+CPR+STR+DOX+TMP
	100.0524246	104.1507103	93.55176933	100.6103516	83.22753906	96.90694626	62.5163827	92.50327654	88.51900393	61.26003706	98.22112415	89.3267449	95.64875491	89.56749672	83.81347656	48.25688073	99.84272608	103.197903	56.22542595	53.11926606	105.2948886	59.08585547	42.91537986	95.84928968	51.86107471	53.28964613	55.4259502	87.26081258	48.71559633	61.49474985	51.65137615
	81.40933455	81.77863702	85.80206563	102.2777915	87.62069819	97.09765982	61.74663355	106.7198327	105.255589	58.48235863	98.21169647	92.41179314	96.67930448	96.88848215	84.84773459	40.20133351	105.4647666	95.21506079	58.39979082	48.34618904	106.3014773	53.26244563	58.86901885	81.19864669	51.69303177	47.50947836	33.28539678	80.78180154	47.71865603	54.42242629	39.84834619
	101.7646201	103.2440056	94.53113607	98.07329052	85.17818914	103.8646638	74.23071314	102.4646347	101.0646055	66.52134889	101.1924606	87.4471086	99.19789996	96.63117982	95.65545901	56.73034855	106.6647222	109.6981187	72.83068397	56.672014	108.0647513	51.33991537	53.16066162	96.2687524	49.20519177	56.96368674	66.23887998	109.4647805	57.13869039	72.03487627	46.97389529
AMP3CPR2STR3DOX2TMP2	AMP	CPR	STR	DOX	TMP	AMP+CPR	AMP+STR	AMP+DOX	AMP+TMP	CPR+STR	CPR+DOX	CPR+TMP	STR+DOX	STR+TMP	DOX+TMP	AMP+CPR+STR	AMP+CPR+DOX	AMP+CPR+TMP	AMP+STR+DOX	AMP+STR+TMP	AMP+DOX+TMP	CPR+STR+DOX	CPR+STR+TMP	CPR+DOX+TMP	STR+DOX+TMP	AMP+CPR+STR+DOX	AMP+CPR+STR+TMP	AMP+CPR+DOX+TMP	AMP+STR+DOX+TMP	CPR+STR+DOX+TMP	AMP+CPR+STR+DOX+TMP
	100.0524246	104.1507103	93.55176933	100.6103516	91.04003906	96.90694626	62.5163827	92.50327654	104.2463958	61.26003706	98.22112415	90.90796788	95.64875491	88.30930537	88.69628906	48.25688073	99.84272608	96.48754915	56.22542595	52.49017038	105.9239843	59.08585547	52.79802347	94.66337245	59.82961992	53.28964613	29.81651376	100.4718218	48.29619921	52.99567634	37.1559633
	81.40933455	81.77863702	85.80206563	102.2777915	93.36469423	97.09765982	61.74663355	106.7198327	107.5565433	58.48235863	98.21169647	91.25181247	96.67930448	92.91410642	83.26318396	40.20133351	105.4647666	94.58752778	58.39979082	52.94809779	107.5565433	53.26244563	34.35234413	80.42532624	61.73355994	47.50947836	26.46097529	107.5565433	48.55536671	37.63895602	36.91985881
	101.7646201	103.2440056	94.53113607	98.07329052	93.84208538	103.8646638	74.23071314	102.4646347	111.7981625	66.52134889	101.1924606	99.1409155	99.19789996	96.86451801	96.6628888	56.73034855	106.6647222	102.2312965	72.83068397	54.33863205	107.598075	51.33991537	53.77612514	98.52545198	62.50546886	56.96368674	44.07175149	111.5648243	60.63876331	61.98230542	44.30508969
AMP3CPR2STR3DOX2TMP3	AMP	CPR	STR	DOX	TMP	AMP+CPR	AMP+STR	AMP+DOX	AMP+TMP	CPR+STR	CPR+DOX	CPR+TMP	STR+DOX	STR+TMP	DOX+TMP	AMP+CPR+STR	AMP+CPR+DOX	AMP+CPR+TMP	AMP+STR+DOX	AMP+STR+TMP	AMP+DOX+TMP	CPR+STR+DOX	CPR+STR+TMP	CPR+DOX+TMP	STR+DOX+TMP	AMP+CPR+STR+DOX	AMP+CPR+STR+TMP	AMP+CPR+DOX+TMP	AMP+STR+DOX+TMP	CPR+STR+DOX+TMP	AMP+CPR+STR+DOX+TMP
	100.0524246	104.1507103	93.55176933	100.6103516	88.11035156	96.90694626	62.5163827	92.50327654	103.8269987	61.26003706	98.22112415	90.90796788	95.64875491	94.60026212	94.55566406	48.25688073	99.84272608	95.2293578	56.22542595	54.75753604	104.2463958	59.08585547	53.19332922	91.89623224	70.90432503	53.28964613	41.16644823	97.11664482	45.95019659	51.01914762	55.84534731
	81.40933455	81.77863702	85.80206563	102.2777915	91.97821243	97.09765982	61.74663355	106.7198327	106.9290103	58.48235863	98.21169647	91.25181247	96.67930448	94.16917244	86.03614756	40.20133351	105.4647666	94.58752778	58.39979082	57.98143548	104.2097006	53.26244563	58.09569841	86.99855002	67.81278599	47.50947836	40.81579291	106.7198327	45.62687933	51.1358144	55.45822983

	101.7646201	103.2440056	94.53113607	98.07329052	96.6628888	103.8646638	74.23071314	102.4646347	108.9981041	66.52134889	101.1924606	97.70483395	99.19789996	93.1311069	97.46883264	56.73034855	106.6647222	102.9313111	72.83068397	49.67186816	103.8646638	51.33991537	52.95550712	92.98628029	70.73064022	56.96368674	52.70526469	103.6313257	55.50532303	56.85344275	61.57211609
AMP3CPR2STR3DOX3TMP1	AMP	CPR	STR	DOX	TMP	AMP+CPR	AMP+STR	AMP+DOX	AMP+TMP	CPR+STR	CPR+DOX	CPR+TMP	STR+DOX	STR+TMP	DOX+TMP	AMP+CPR+STR	AMP+CPR+DOX	AMP+CPR+TMP	AMP+STR+DOX	AMP+STR+TMP	AMP+DOX+TMP	CPR+STR+DOX	CPR+STR+TMP	CPR+DOX+TMP	STR+DOX+TMP	AMP+CPR+STR+DOX	AMP+CPR+STR+TMP	AMP+CPR+DOX+TMP	AMP+STR+DOX+TMP	CPR+STR+DOX+TMP	AMP+CPR+STR+DOX+TMP
	100.0524246	104.1507103	93.55176933	99.04785156	83.22753906	96.90694626	62.5163827	93.76146789	88.51900393	61.26003706	102.3718345	89.3267449	91.03538663	89.56749672	79.51660156	48.25688073	103.6173001	103.197903	50.81258191	53.11926606	88.72870249	38.56701668	42.91537986	94.07041384	72.16251638	48.92529489	55.4259502	105.5045872	51.86107471	52.6003706	63.60419397
	81.40933455	81.77863702	85.80206563	101.2874474	87.62069819	97.09765982	61.74663355	110.2758531	105.255589	58.48235863	102.0782987	92.41179314	94.37835011	96.88848215	82.47090864	40.20133351	107.9748987	95.21506079	43.95345797	48.34618904	108.8116094	59.83566941	58.86901885	84.67858869	74.50647143	48.34618904	33.28539678	110.2758531	51.06549876	42.66553891	45.49614329
	101.7646201	103.2440056	94.53113607	100.0881501	85.17818914	103.8646638	74.23071314	110.3981333	101.0646055	66.52134889	103.4491601	87.4471086	96.86451801	96.63117982	84.37224531	56.73034855	112.7315152	109.6981187	46.69680618	56.672014	104.3313402	53.16066162	53.16066162	91.34504424	78.66413884	54.10529386	66.23887998	91.96441593	64.83885081	48.64726247	52.2385883
AMP3CPR2STR3DOX3TMP2	AMP	CPR	STR	DOX	TMP	AMP+CPR	AMP+STR	AMP+DOX	AMP+TMP	CPR+STR	CPR+DOX	CPR+TMP	STR+DOX	STR+TMP	DOX+TMP	AMP+CPR+STR	AMP+CPR+DOX	AMP+CPR+TMP	AMP+STR+DOX	AMP+STR+TMP	AMP+DOX+TMP	CPR+STR+DOX	CPR+STR+TMP	CPR+DOX+TMP	STR+DOX+TMP	AMP+CPR+STR+DOX	AMP+CPR+STR+TMP	AMP+CPR+DOX+TMP	AMP+STR+DOX+TMP	CPR+STR+DOX+TMP	AMP+CPR+STR+DOX+TMP
	100.0524246	104.1507103	93.55176933	99.04785156	91.04003906	96.90694626	62.5163827	93.76146789	104.2463958	61.26003706	102.3718345	90.90796788	91.03538663	88.30930537	77.56347656	48.25688073	103.6173001	96.48754915	50.81258191	52.49017038	86.63171691	38.56701668	52.79802347	94.46571958	72.37221494	48.92529489	29.81651376	103.8269987	50.3931848	54.37924645	62.9750983
	81.40933455	81.77863702	85.80206563	101.2874474	93.36469423	97.09765982	61.74663355	110.2758531	107.5565433	58.48235863	102.0782987	91.25181247	94.37835011	92.91410642	85.83807873	40.20133351	107.9748987	94.58752778	43.95345797	52.94809779	104.8372336	59.83566941	34.35234413	85.06524891	85.38371029	48.34618904	26.46097529	107.765721	50.43796575	65.05558241	63.19780363
	101.7646201	103.2440056	94.53113607	100.0881501	93.84208538	103.8646638	74.23071314	110.3981333	111.7981625	66.52134889	103.4491601	99.1409155	96.86451801	96.86451801	95.65545901	56.73034855	112.7315152	102.2312965	46.69680618	54.33863205	105.7313694	53.16066162	53.77612514	89.08834466	97.09785621	54.10529386	44.07175149	98.26454718	58.07204317	51.10911655	64.13883623
AMP3CPR2STR3DOX3TMP3	AMP	CPR	STR	DOX	TMP	AMP+CPR	AMP+STR	AMP+DOX	AMP+TMP	CPR+STR	CPR+DOX	CPR+TMP	STR+DOX	STR+TMP	DOX+TMP	AMP+CPR+STR	AMP+CPR+DOX	AMP+CPR+TMP	AMP+STR+DOX	AMP+STR+TMP	AMP+DOX+TMP	CPR+STR+DOX	CPR+STR+TMP	CPR+DOX+TMP	STR+DOX+TMP	AMP+CPR+STR+DOX	AMP+CPR+STR+TMP	AMP+CPR+DOX+TMP	AMP+STR+DOX+TMP	CPR+STR+DOX+TMP	AMP+CPR+STR+DOX+TMP
	100.0524246	104.1507103	93.55176933	99.04785156	88.11035156	96.90694626	62.5163827	93.76146789	103.8269987	61.26003706	102.3718345	90.90796788	91.03538663	94.60026212	93.96972656	48.25688073	103.6173001	95.2293578	50.81258191	54.75753604	97.11664482	38.56701668	53.19332922	95.25633107	66.50065531	48.92529489	41.16644823	104.0366972	53.07994758	50.03088326	54.79685452
	81.40933455	81.77863702	85.80206563	101.2874474	91.97821243	97.09765982	61.74663355	110.2758531	106.9290103	58.48235863	102.0782987	91.25181247	94.37835011	94.16917244	101.0893786	40.20133351	107.9748987	94.58752778	43.95345797	57.98143548	102.9546346	59.83566941	58.09569841	97.0517158	73.25140541	48.34618904	40.81579291	109.2299647	46.88194535	52.68245529	47.50947836
	101.7646201	103.2440056	94.53113607	100.0881501	96.6628888	103.8646638	74.23071314	110.3981333	108.9981041	66.52134889	103.4491601	97.70483395	96.86451801	93.1311069	104.5208412	56.73034855	112.7315152	102.9313111	46.69680618	49.67186816	109.2314423	53.16066162	52.95550712	99.34607001	80.064168	54.10529386	52.70526469	107.8314132	51.77191191	60.95653289	56.90535219
AMP3CPR3STR1DOX1TMP1	AMP	CPR	STR	DOX	TMP	AMP+CPR	AMP+STR	AMP+DOX	AMP+TMP	CPR+STR	CPR+DOX	CPR+TMP	STR+DOX	STR+TMP	DOX+TMP	AMP+CPR+STR	AMP+CPR+DOX	AMP+CPR+TMP	AMP+STR+DOX	AMP+STR+TMP	AMP+DOX+TMP	CPR+STR+DOX	CPR+STR+TMP	CPR+DOX+TMP	STR+DOX+TMP	AMP+CPR+STR+DOX	AMP+CPR+STR+TMP	AMP+CPR+DOX+TMP	AMP+STR+DOX+TMP	CPR+STR+DOX+TMP	AMP+CPR+STR+DOX+TMP
	100.0524246	86.21231979	97.48535156	103.9306641	83.22753906	98.16513761	3.630406291	98.37483617	88.51900393	58.33551769	101.5203145	95.64875491	84.20410156	81.07910156	83.61816406	4.469200524	93.34207077	88.09960682	1.743119266	2.162516383	96.27785059	1.323722149	2.752293578	90.40629096	59.59472656	2.372214941	2.581913499	95.43905636	10.09174312	8.204456094	5.910878113
	81.40933455	102.3271016	89.60138648	102.6739292	87.62069819	84.12864427	5.961563603	105.4647666	105.255589	45.28696562	106.0922996	97.09765982	80.29215152	82.66897747	76.7269126	8.60243169	106.510655	102.3271016	5.26866257	6.523728592	97.3068375	3.804418878	6.314550922	86.22042097	50.97796484	2.196365538	10.35429468	96.26094914	34.8280821	9.452215976	2.745456922
	101.7646201	104.3313402	86.58859086	98.47626244	85.17818914	103.8646638	15.93991541	104.3313402	101.0646055	59.10748141	103.8646638	108.0647513	67.85039668	82.15589976	85.17818914	4.273005688	112.2648389	101.0646055	3.106314715	3.470905644	89.63103398	20.14000292	1.006270964	82.86422634	59.18650044	1.837538282	4.739682077	108.2980895	4.637596617	4.404258422	3.470905644
AMP3CPR3STR1DOX1TMP2	AMP	CPR	STR	DOX	TMP	AMP+CPR	AMP+STR	AMP+DOX	AMP+TMP	CPR+STR	CPR+DOX	CPR+TMP	STR+DOX	STR+TMP	DOX+TMP	AMP+CPR+STR	AMP+CPR+DOX	AMP+CPR+TMP	AMP+STR+DOX	AMP+STR+TMP	AMP+DOX+TMP	CPR+STR+DOX	CPR+STR+TMP	CPR+DOX+TMP	STR+DOX+TMP	AMP+CPR+STR+DOX	AMP+CPR+STR+TMP	AMP+CPR+DOX+TMP	AMP+STR+DOX+TMP	CPR+STR+DOX+TMP	AMP+CPR+STR+DOX+TMP
	100.0524246	86.21231979	97.48535156	103.9306641	91.04003906	98.16513761	3.630406291	98.37483617	104.2463958	58.33551769	101.5203145	101.1009174	84.20410156	81.07910156	84.20410156	4.469200524	93.34207077	90.82568807	1.743119266	5.727391874	98.16513761	1.323722149	60.01310616	89.35779817	46.72851563	2.372214941	0.655307995	98.16513761	12.1887287	6.526867628	3.630406291
	81.40933455	102.3271016	89.60138648	102.6739292	93.36469423	84.12864427	5.961563603	105.4647666	107.5565433	45.28696562	106.0922996	103.1638123	80.29215152	81.08442684	80.49022035	8.60243169	106.510655	102.9546346	5.26866257	7.778794614	96.26094914	3.804418878	8.406327625	94.16917244	56.77147809	2.196365538	1.921819846	109.2299647	16.56425677	8.615505295	3.582167604
	101.7646201	104.3313402	86.58859086	98.47626244	93.84208538	103.8646638	15.93991541	104.3313402	111.7981625	59.10748141	103.8646638	106.6647222	67.85039668	85.17818914	87.79750661	4.273005688	112.2648389	112.2648389	3.106314715	3.704243838	91.73107773	20.14000292	1.706285548	85.43094648	51.69374134	1.837538282	3.470905644	112.0315007	2.129211025	4.170920228	6.270963978
AMP3CPR3STR1DOX1TMP3	AMP	CPR	STR	DOX	TMP	AMP+CPR	AMP+STR	AMP+DOX	AMP+TMP	CPR+STR	CPR+DOX	CPR+TMP	STR+DOX	STR+TMP	DOX+TMP	AMP+CPR+STR	AMP+CPR+DOX	AMP+CPR+TMP	AMP+STR+DOX	AMP+STR+TMP	AMP+DOX+TMP	CPR+STR+DOX	CPR+STR+TMP	CPR+DOX+TMP	STR+DOX+TMP	AMP+CPR+STR+DOX	AMP+CPR+STR+TMP	AMP+CPR+DOX+TMP	AMP+STR+DOX+TMP	CPR+STR+DOX+TMP	AMP+CPR+STR+DOX+TMP
	100.0524246	86.21231979	97.48535156	103.9306641	88.11035156	98.16513761	3.630406291	98.37483617	103.8269987	58.33551769	101.5203145	100.2621232	84.20410156	86.54785156	94.36035156	4.469200524	93.34207077	93.76146789	1.743119266	7.365661861	101.310616	1.323722149	7.798165138	110.9567497	48.68164063	2.372214941	2.54259502	104.4560944	13.44692005	5.688073394	3.591087811
	81.40933455	102.3271016	89.60138648	102.6739292	91.97821243	84.12864427	5.961563603	105.4647666	106.9290103	45.28696562	106.0922996	98.77108119	80.29215152	84.45159693	99.10869027	8.60243169	106.510655	103.1638123	5.26866257	19.91109949	106.0922996	3.804418878	6.732906262	107.138188	1.262688784	2.196365538	2.327101582	102.3271016	15.10001307	7.360439273	4.418878285
	101.7646201	104.3313402	86.58859086	98.47626244	96.6628888	103.8646638	15.93991541	104.3313402	108.9981041	59.10748141	103.8646638	99.66457635	67.85039668	90.01385216	109.1550183	4.273005688	112.2648389	103.1646493	3.106314715	6.329298527	114.1315444	20.14000292	53.74070293	112.498177	62.57398313	1.837538282	4.170920228	104.3313402	4.404258422	4.870934811	4.170920228
AMP3CPR3STR1DOX2TMP1	AMP	CPR	STR	DOX	TMP	AMP+CPR	AMP+STR	AMP+DOX	AMP+TMP	CPR+STR	CPR+DOX	CPR+TMP	STR+DOX	STR+TMP	DOX+TMP	AMP+CPR+STR	AMP+CPR+DOX	AMP+CPR+TMP	AMP+STR+DOX	AMP+STR+TMP	AMP+DOX+TMP	CPR+STR+DOX	CPR+STR+TMP	CPR+DOX+TMP	STR+DOX+TMP	AMP+CPR+STR+DOX	AMP+CPR+STR+TMP	AMP+CPR+DOX+TMP	AMP+STR+DOX+TMP	CPR+STR+DOX+TMP	AMP+CPR+STR+DOX+TMP
	100.0524246	86.21231979	97.48535156	100.6103516	83.22753906	98.16513761	3.630406291	92.50327654	88.51900393	58.33551769	96.69724771	95.64875491	90.45410156	81.07910156	83.81347656	4.469200524	85.37352556	88.09960682	4.259501966	2.162516383	105.2948886	2.346002621	2.752293578	103.197903	53.34472656	3.630406291	2.581913499	98.16513761	12.39842726	7.994757536	4.639580603
	81.40933455	102.3271016	89.60138648	102.2777915	87.62069819	84.12864427	5.961563603	106.7198327	105.255589	45.28696562	105.255589	97.09765982	92.37435009	82.66897747	84.84773459	8.60243169	106.9290103	102.3271016	5.26866257	6.523728592	106.3014773	7.151261603	6.314550922	92.70492875	56.37534043	2.549352857	10.35429468	96.47012681	15.30919074	7.151261603	3.595241208
	101.7646201	104.3313402	86.58859086	98.07329052	85.17818914	103.8646638	15.93991541	102.4646347	101.0646055	59.10748141	104.098002	108.0647513	86.79007682	82.15589976	95.65545901	4.273005688	112.9648534	101.0646055	1.939623742	3.470905644	108.0647513	38.80705848	1.006270964	105.7313694	54.71603073	4.039667493	4.739682077	94.29779787	2.304214671	26.67347236	42.0737932
AMP3CPR3STR1DOX2TMP2	AMP	CPR	STR	DOX	TMP	AMP+CPR	AMP+STR	AMP+DOX	AMP+TMP	CPR+STR	CPR+DOX	CPR+TMP	STR+DOX	STR+TMP	DOX+TMP	AMP+CPR+STR	AMP+CPR+DOX	AMP+CPR+TMP	AMP+STR+DOX	AMP+STR+TMP	AMP+DOX+TMP	CPR+STR+DOX	CPR+STR+TMP	CPR+DOX+TMP	STR+DOX+TMP	AMP+CPR+STR+DOX	AMP+CPR+STR+TMP	AMP+CPR+DOX+TMP	AMP+STR+DOX+TMP	CPR+STR+DOX+TMP	AMP+CPR+STR+DOX+TMP
	100.0524246	86.21231979	97.48535156	100.6103516	91.04003906	98.16513761	3.630406291	92.50327654	104.2463958	58.33551769	96.69724771	101.1009174	90.45410156	81.07910156	88.69628906	4.469200524	85.37352556	90.82568807	4.259501966	5.727391874	105.9239843	2.346002621	60.01310616	104.6657929	50.24414063	3.630406291	0.655307995	107.1821756	8.623853211	6.107470511	2.123197903
	81.40933455	102.3271016	89.60138648	102.2777915	93.36469423	84.12864427	5.961563603	106.7198327	107.5565433	45.28696562	105.255589	103.1638123	92.37435009	81.08442684	83.26318396	8.60243169	106.9290103	102.9546346	5.26866257	7.778794614	107.5565433	7.151261603	8.406327625	105.0464113	62.11933647	2.549352857	1.921819846	110.0666754	10.49810433	6.942083933	3.386063538
	101.7646201	104.3313402	86.58859086	98.07329052	93.84208538	103.8646638	15.93991541	102.4646347	111.7981625	59.10748141	104.098002	106.6647222	86.79007682	85.17818914	96.6628888	4.273005688	112.9648534	112.2648389	1.939623742	3.704243838	107.598075	38.80705848	1.706285548	110.6314715	64.58884272	4.039667493	3.470905644	108.5314277	2.87297652	58.87414321	-2.829225609
AMP3CPR3STR1DOX2TMP3	AMP	CPR	STR	DOX	TMP	AMP+CPR	AMP+STR	AMP+DOX	AMP+TMP	CPR+STR	CPR+DOX	CPR+TMP	STR+DOX	STR+TMP	DOX+TMP	AMP+CPR+STR	AMP+CPR+DOX	AMP+CPR+TMP	AMP+STR+DOX	AMP+STR+TMP	AMP+DOX+TMP	CPR+STR+DOX	CPR+STR+TMP	CPR+DOX+TMP	STR+DOX+TMP	AMP+CPR+STR+DOX	AMP+CPR+STR+TMP	AMP+CPR+DOX+TMP	AMP+STR+DOX+TMP	CPR+STR+DOX+TMP	AMP+CPR+STR+DOX+TMP
	100.0524246	86.21231979	97.48535156	100.6103516	88.11035156	98.16513761	3.630406291	92.50327654	103.8269987	58.33551769	96.69724771	100.2621232	90.45410156	86.54785156	94.55566406	4.469200524	85.37352556	93.76146789	4.259501966	7.365661861	104.2463958	2.346002621	7.798165138	101.7300131	50.83007813	3.630406291	2.54259502	101.5203145	6.526867628	5.268676278	1.323722149
	81.40933455	102.3271016	89.60138648	102.2777915	91.97821243	84.12864427	5.961563603	106.7198327	106.9290103	45.28696562	105.255589	98.77108119	92.37435009	84.45159693	86.03614756	8.60243169	106.9290103	103.1638123	5.26866257	19.91109949	104.2097006	7.151261603	6.732906262	102.1179239	54.39465214	2.549352857	2.327101582	104.8372336	11.12563734	5.26866257	6.523728592
	101.7646201	104.3313402	86.58859086	98.07329052	96.6628888	103.8646638	15.93991541	102.4646347	108.9981041	59.10748141	104.098002	99.66457635	86.79007682	90.01385216	97.46883264	4.273005688	112.9648534	103.1646493	1.939623742	6.329298527	103.8646638	38.80705848	53.74070293	103.1646493	51.8952273	4.039667493	4.170920228	102.2312965	1.837538282	1.370861893	2.77089106
AMP3CPR3STR1DOX3TMP1	AMP	CPR	STR	DOX	TMP	AMP+CPR	AMP+STR	AMP+DOX	AMP+TMP	CPR+STR	CPR+DOX	CPR+TMP	STR+DOX	STR+TMP	DOX+TMP	AMP+CPR+STR	AMP+CPR+DOX	AMP+CPR+TMP	AMP+STR+DOX	AMP+STR+TMP	AMP+DOX+TMP	CPR+STR+DOX	CPR+STR+TMP	CPR+DOX+TMP	STR+DOX+TMP	AMP+CPR+STR+DOX	AMP+CPR+STR+TMP	AMP+CPR+DOX+TMP	AMP+STR+DOX+TMP	CPR+STR+DOX+TMP	AMP+CPR+STR+DOX+TMP
	100.0524246	86.21231979	97.48535156	99.04785156	83.22753906	98.16513761	3.630406291	93.76146789	88.51900393	58.33551769	103.8269987	95.64875491	81.86035156	81.07910156	79.51660156	4.469200524	88.51900393	88.09960682	4.259501966	2.162516383	88.72870249	1.703800786	2.752293578	97.7457405	43.01757813	2.791612058	2.581913499	103.8269987	4.010484928	23.34207077	1.743119266
	81.40933455	102.3271016	89.60138648	101.2874474	87.62069819	84.12864427	5.961563603	110.2758531	105.255589	45.28696562	105.4647666	97.09765982	87.22456053	82.66897747	82.47090864	8.60243169	108.1840763	102.3271016	5.477840241	6.523728592	108.8116094	4.431951889	6.314550922	93.12328409	60.13864818	1.921819846	10.35429468	109.4391424	10.07974899	5.477840241	17.80624918
	101.7646201	104.3313402	86.58859086	100.0881501	85.17818914	103.8646638	15.93991541	110.3981333	101.0646055	59.10748141	113.8982062	108.0647513	86.18561894	82.15589976	84.37224531	4.273005688	104.3313402	101.0646055	59.3408196	3.470905644	104.3313402	3.106314715	1.006270964	94.29779787	47.90328674	5.906373049	4.739682077	102.6979729	6.270963978	5.570949395	10.70438967
AMP3CPR3STR1DOX3TMP2	AMP	CPR	STR	DOX	TMP	AMP+CPR	AMP+STR	AMP+DOX	AMP+TMP	CPR+STR	CPR+DOX	CPR+TMP	STR+DOX	STR+TMP	DOX+TMP	AMP+CPR+STR	AMP+CPR+DOX	AMP+CPR+TMP	AMP+STR+DOX	AMP+STR+TMP	AMP+DOX+TMP	CPR+STR+DOX	CPR+STR+TMP	CPR+DOX+TMP	STR+DOX+TMP	AMP+CPR+STR+DOX	AMP+CPR+STR+TMP	AMP+CPR+DOX+TMP	AMP+STR+DOX+TMP	CPR+STR+DOX+TMP	AMP+CPR+STR+DOX+TMP
	100.0524246	86.21231979	97.48535156	99.04785156	91.04003906	98.16513761	3.630406291	93.76146789	104.2463958	58.33551769	103.8269987	101.1009174	81.86035156	81.07910156	77.56347656	4.469200524	88.51900393	90.82568807	4.259501966	5.727391874	86.63171691	1.703800786	60.01310616	97.7457405	47.29003906	2.791612058	0.655307995	99.42332896	3.001310616	23.76146789	0.249017038
	81.40933455	102.3271016	89.60138648	101.2874474	93.36469423	84.12864427	5.961563603	110.2758531	107.5565433	45.28696562	105.4647666	103.1638123	87.22456053	81.08442684	85.83807873	8.60243169	108.1840763	102.9546346	5.477840241	7.778794614	104.8372336	4.431951889	8.406327625	93.33246176	51.57217133	1.921819846	1.921819846	104.0005229	6.105373251	4.418878285	11.94927441
	101.7646201	104.3313402	86.58859086	100.0881501	93.84208538	103.8646638	15.93991541	110.3981333	111.7981625	59.10748141	113.8982062	106.6647222	86.18561894	85.17818914	95.65545901	4.273005688	104.3313402	112.2648389	59.3408196	3.704243838	105.7313694	3.106314715	1.706285548	99.19789996	70.26822818	5.906373049	3.470905644	104.098002	5.104273006	1.137523698	17.70453551
AMP3CPR3STR1DOX3TMP3	AMP	CPR	STR	DOX	TMP	AMP+CPR	AMP+STR	AMP+DOX	AMP+TMP	CPR+STR	CPR+DOX	CPR+TMP	STR+DOX	STR+TMP	DOX+TMP	AMP+CPR+STR	AMP+CPR+DOX	AMP+CPR+TMP	AMP+STR+DOX	AMP+STR+TMP	AMP+DOX+TMP	CPR+STR+DOX	CPR+STR+TMP	CPR+DOX+TMP	STR+DOX+TMP	AMP+CPR+STR+DOX	AMP+CPR+STR+TMP	AMP+CPR+DOX+TMP	AMP+STR+DOX+TMP	CPR+STR+DOX+TMP	AMP+CPR+STR+DOX+TMP
	100.0524246	86.21231979	97.48535156	99.04785156	88.11035156	98.16513761	3.630406291	93.76146789	103.8269987	58.33551769	103.8269987	100.2621232	81.86035156	86.54785156	93.96972656	4.469200524	88.51900393	93.76146789	4.259501966	7.365661861	97.11664482	1.703800786	7.798165138	93.55176933	52.00195313	2.791612058	2.54259502	108.2306684	54.79685452	5.307994758	3.591087811
	81.40933455	102.3271016	89.60138648	101.2874474	91.97821243	84.12864427	5.961563603	110.2758531	106.9290103	45.28696562	105.4647666	98.77108119	87.22456053	84.45159693	101.0893786	8.60243169	108.1840763	103.1638123	5.477840241	19.91109949	102.9546346	4.431951889	6.732906262	98.77108119	42.42386729	1.921819846	2.327101582	112.1584521	10.07974899	7.556543339	19.28356648
	101.7646201	104.3313402	86.58859086	100.0881501	96.6628888	103.8646638	15.93991541	110.3981333	108.9981041	59.10748141	113.8982062	99.66457635	86.18561894	90.01385216	104.5208412	4.273005688	104.3313402	103.1646493	59.3408196	6.329298527	109.2314423	3.106314715	53.74070293	105.264693	48.26848004	5.906373049	4.170920228	109.6981187	4.404258422	5.3376112	10.35438238
AMP3CPR3STR2DOX1TMP1	AMP	CPR	STR	DOX	TMP	AMP+CPR	AMP+STR	AMP+DOX	AMP+TMP	CPR+STR	CPR+DOX	CPR+TMP	STR+DOX	STR+TMP	DOX+TMP	AMP+CPR+STR	AMP+CPR+DOX	AMP+CPR+TMP	AMP+STR+DOX	AMP+STR+TMP	AMP+DOX+TMP	CPR+STR+DOX	CPR+STR+TMP	CPR+DOX+TMP	STR+DOX+TMP	AMP+CPR+STR+DOX	AMP+CPR+STR+TMP	AMP+CPR+DOX+TMP	AMP+STR+DOX+TMP	CPR+STR+DOX+TMP	AMP+CPR+STR+DOX+TMP
	100.0524246	86.21231979	46.67078443	103.9306641	83.22753906	98.16513761	44.07601573	98.37483617	88.51900393	58.15203145	101.5203145	95.64875491	45.07720815	49.20321186	83.61816406	3.211009174	93.34207077	88.09960682	5.688073394	21.42857143	96.27785059	17.0249017	28.55832241	90.40629096	3.347745522	1.952817824	22.0576671	95.43905636	11.55963303	8.427260813	12.83093054
	81.40933455	102.3271016	50.16916385	102.6739292	87.62069819	84.12864427	36.71068113	105.4647666	105.255589	56.92247353	106.0922996	97.09765982	3.28661189	54.03576607	76.7269126	4.915675252	106.510655	102.3271016	4.431951889	7.778794614	97.3068375	54.28160544	5.0594849	86.22042097	2.319961334	3.595241208	3.45143156	96.26094914	14.8908354	7.569616943	3.595241208
	101.7646201	104.3313402	59.93076035	98.47626244	85.17818914	103.8646638	39.50707306	104.3313402	101.0646055	64.13883623	103.8646638	108.0647513	6.475189127	57.08424157	85.17818914	9.873122357	112.2648389	101.0646055	62.37421613	0.904185504	89.63103398	32.27358903	62.14087793	82.86422634	47.50609052	3.339652909	35.07364737	108.2980895	6.504302173	10.10646055	3.470905644
AMP3CPR3STR2DOX1TMP2	AMP	CPR	STR	DOX	TMP	AMP+CPR	AMP+STR	AMP+DOX	AMP+TMP	CPR+STR	CPR+DOX	CPR+TMP	STR+DOX	STR+TMP	DOX+TMP	AMP+CPR+STR	AMP+CPR+DOX	AMP+CPR+TMP	AMP+STR+DOX	AMP+STR+TMP	AMP+DOX+TMP	CPR+STR+DOX	CPR+STR+TMP	CPR+DOX+TMP	STR+DOX+TMP	AMP+CPR+STR+DOX	AMP+CPR+STR+TMP	AMP+CPR+DOX+TMP	AMP+STR+DOX+TMP	CPR+STR+DOX+TMP	AMP+CPR+STR+DOX+TMP
	100.0524246	86.21231979	46.67078443	103.9306641	91.04003906	98.16513761	44.07601573	98.37483617	104.2463958	58.15203145	101.5203145	101.1009174	45.07720815	63.03891291	84.20410156	3.211009174	93.34207077	90.82568807	5.688073394	3.211009174	98.16513761	17.0249017	58.12581913	89.35779817	5.126621371	1.952817824	7.378768021	98.16513761	13.23722149	3.171690695	8.872870249
	81.40933455	102.3271016	50.16916385	102.6739292	93.36469423	84.12864427	36.71068113	105.4647666	107.5565433	56.92247353	106.0922996	103.1638123	3.28661189	54.80908652	80.49022035	4.915675252	106.510655	102.9546346	4.431951889	7.778794614	96.26094914	54.28160544	5.26866257	94.16917244	1.739971	3.595241208	3.595241208	109.2299647	14.68165773	8.406327625	4.641129559
	101.7646201	104.3313402	59.93076035	98.47626244	93.84208538	103.8646638	39.50707306	104.3313402	111.7981625	64.13883623	103.8646638	106.6647222	6.475189127	73.08629311	87.79750661	9.873122357	112.2648389	112.2648389	62.37421613	40.67376404	91.73107773	32.27358903	39.04039667	85.43094648	1.051416848	3.339652909	28.30683973	112.0315007	3.806329299	4.870934811	2.537552866
AMP3CPR3STR2DOX1TMP3	AMP	CPR	STR	DOX	TMP	AMP+CPR	AMP+STR	AMP+DOX	AMP+TMP	CPR+STR	CPR+DOX	CPR+TMP	STR+DOX	STR+TMP	DOX+TMP	AMP+CPR+STR	AMP+CPR+DOX	AMP+CPR+TMP	AMP+STR+DOX	AMP+STR+TMP	AMP+DOX+TMP	CPR+STR+DOX	CPR+STR+TMP	CPR+DOX+TMP	STR+DOX+TMP	AMP+CPR+STR+DOX	AMP+CPR+STR+TMP	AMP+CPR+DOX+TMP	AMP+STR+DOX+TMP	CPR+STR+DOX+TMP	AMP+CPR+STR+DOX+TMP
	100.0524246	86.21231979	46.67078443	103.9306641	88.11035156	98.16513761	44.07601573	98.37483617	103.8269987	58.15203145	101.5203145	100.2621232	45.07720815	69.75911056	94.36035156	3.211009174	93.34207077	93.76146789	5.688073394	22.0576671	101.310616	17.0249017	67.77195282	110.9567497	5.719579988	1.952817824	10.94364351	104.4560944	13.44692005	4.639580603	24.5740498
	81.40933455	102.3271016	50.16916385	102.6739292	91.97821243	84.12864427	36.71068113	105.4647666	106.9290103	56.92247353	106.0922996	98.77108119	3.28661189	54.71242146	99.10869027	4.915675252	106.510655	103.1638123	4.431951889	51.27467643	106.0922996	54.28160544	23.32331024	107.138188	3.093281779	3.595241208	3.386063538	102.3271016	13.84494705	28.34357432	5.687017911
	101.7646201	104.3313402	59.93076035	98.47626244	96.6628888	103.8646638	39.50707306	104.3313402	108.9981041	64.13883623	103.8646638	99.66457635	6.475189127	71.65021157	109.1550183	9.873122357	112.2648389	103.1646493	62.37421613	3.704243838	114.1315444	32.27358903	43.24048418	112.498177	1.051416848	3.339652909	2.537552866	104.3313402	1.939623742	6.737640368	1.837538282
AMP3CPR3STR2DOX2TMP1	AMP	CPR	STR	DOX	TMP	AMP+CPR	AMP+STR	AMP+DOX	AMP+TMP	CPR+STR	CPR+DOX	CPR+TMP	STR+DOX	STR+TMP	DOX+TMP	AMP+CPR+STR	AMP+CPR+DOX	AMP+CPR+TMP	AMP+STR+DOX	AMP+STR+TMP	AMP+DOX+TMP	CPR+STR+DOX	CPR+STR+TMP	CPR+DOX+TMP	STR+DOX+TMP	AMP+CPR+STR+DOX	AMP+CPR+STR+TMP	AMP+CPR+DOX+TMP	AMP+STR+DOX+TMP	CPR+STR+DOX+TMP	AMP+CPR+STR+DOX+TMP
	100.0524246	86.21231979	46.67078443	100.6103516	83.22753906	98.16513761	44.07601573	92.50327654	88.51900393	58.15203145	96.69724771	95.64875491	61.6553428	49.20321186	83.81347656	3.211009174	85.37352556	88.09960682	5.701179554	21.42857143	105.2948886	56.47444299	28.55832241	103.197903	5.324274243	3.420707733	22.0576671	98.16513761	13.02752294	5.478374836	6.526867628
	81.40933455	102.3271016	50.16916385	102.2777915	87.62069819	84.12864427	36.71068113	106.7198327	105.255589	56.92247353	105.255589	97.09765982	48.5258579	54.03576607	84.84773459	4.915675252	106.9290103	102.3271016	5.0594849	7.778794614	106.3014773	56.92247353	5.0594849	92.70492875	39.37892702	4.85030723	3.45143156	96.47012681	13.21741404	6.105373251	5.477840241
	101.7646201	104.3313402	59.93076035	98.07329052	85.17818914	103.8646638	39.50707306	102.4646347	101.0646055	64.13883623	104.098002	108.0647513	67.13681241	57.08424157	95.65545901	9.873122357	112.9648534	101.0646055	57.70745224	0.904185504	108.0647513	67.87224734	62.14087793	105.7313694	15.00192332	2.172961937	35.07364737	94.29779787	4.404258422	7.306402217	2.304214671
AMP3CPR3STR2DOX2TMP2	AMP	CPR	STR	DOX	TMP	AMP+CPR	AMP+STR	AMP+DOX	AMP+TMP	CPR+STR	CPR+DOX	CPR+TMP	STR+DOX	STR+TMP	DOX+TMP	AMP+CPR+STR	AMP+CPR+DOX	AMP+CPR+TMP	AMP+STR+DOX	AMP+STR+TMP	AMP+DOX+TMP	CPR+STR+DOX	CPR+STR+TMP	CPR+DOX+TMP	STR+DOX+TMP	AMP+CPR+STR+DOX	AMP+CPR+STR+TMP	AMP+CPR+DOX+TMP	AMP+STR+DOX+TMP	CPR+STR+DOX+TMP	AMP+CPR+STR+DOX+TMP
	100.0524246	86.21231979	46.67078443	100.6103516	91.04003906	98.16513761	44.07601573	92.50327654	104.2463958	58.15203145	96.69724771	101.1009174	61.6553428	63.03891291	88.69628906	3.211009174	85.37352556	90.82568807	5.701179554	3.211009174	105.9239843	56.47444299	58.12581913	104.6657929	12.43977764	3.420707733	7.378768021	107.1821756	63.99737877	28.34862385	5.688073394
	81.40933455	102.3271016	50.16916385	102.2777915	93.36469423	84.12864427	36.71068113	106.7198327	107.5565433	56.92247353	105.255589	103.1638123	48.5258579	54.80908652	83.26318396	4.915675252	106.9290103	102.9546346	5.0594849	7.778794614	107.5565433	56.92247353	5.26866257	105.0464113	25.0362494	4.85030723	3.595241208	110.0666754	12.38070336	5.0594849	32.52712773
	101.7646201	104.3313402	59.93076035	98.07329052	93.84208538	103.8646638	39.50707306	102.4646347	111.7981625	64.13883623	104.098002	106.6647222	67.13681241	73.08629311	96.6628888	9.873122357	112.9648534	112.2648389	57.70745224	40.67376404	107.598075	67.87224734	39.04039667	110.6314715	24.02872163	2.172961937	28.30683973	108.5314277	2.304214671	4.870934811	1.837538282
AMP3CPR3STR2DOX2TMP3	AMP	CPR	STR	DOX	TMP	AMP+CPR	AMP+STR	AMP+DOX	AMP+TMP	CPR+STR	CPR+DOX	CPR+TMP	STR+DOX	STR+TMP	DOX+TMP	AMP+CPR+STR	AMP+CPR+DOX	AMP+CPR+TMP	AMP+STR+DOX	AMP+STR+TMP	AMP+DOX+TMP	CPR+STR+DOX	CPR+STR+TMP	CPR+DOX+TMP	STR+DOX+TMP	AMP+CPR+STR+DOX	AMP+CPR+STR+TMP	AMP+CPR+DOX+TMP	AMP+STR+DOX+TMP	CPR+STR+DOX+TMP	AMP+CPR+STR+DOX+TMP
	100.0524246	86.21231979	46.67078443	100.6103516	88.11035156	98.16513761	44.07601573	92.50327654	103.8269987	58.15203145	96.69724771	100.2621232	61.6553428	69.75911056	94.55566406	3.211009174	85.37352556	93.76146789	5.701179554	22.0576671	104.2463958	56.47444299	67.77195282	101.7300131	13.23038913	3.420707733	10.94364351	101.5203145	7.365661861	32.54259502	56.65792923
	81.40933455	102.3271016	50.16916385	102.2777915	91.97821243	84.12864427	36.71068113	106.7198327	106.9290103	56.92247353	105.255589	98.77108119	48.5258579	54.71242146	86.03614756	4.915675252	106.9290103	103.1638123	5.0594849	51.27467643	104.2097006	56.92247353	23.32331024	102.1179239	24.45625906	4.85030723	3.386063538	104.8372336	9.870571317	5.255588966	5.26866257
	101.7646201	104.3313402	59.93076035	98.07329052	96.6628888	103.8646638	39.50707306	102.4646347	108.9981041	64.13883623	104.098002	99.66457635	67.13681241	71.65021157	97.46883264	9.873122357	112.9648534	103.1646493	57.70745224	3.704243838	103.8646638	67.87224734	43.24048418	103.1646493	23.0029491	2.172961937	2.537552866	102.2312965	88.40600846	20.37334111	1.604200088
AMP3CPR3STR2DOX3TMP1	AMP	CPR	STR	DOX	TMP	AMP+CPR	AMP+STR	AMP+DOX	AMP+TMP	CPR+STR	CPR+DOX	CPR+TMP	STR+DOX	STR+TMP	DOX+TMP	AMP+CPR+STR	AMP+CPR+DOX	AMP+CPR+TMP	AMP+STR+DOX	AMP+STR+TMP	AMP+DOX+TMP	CPR+STR+DOX	CPR+STR+TMP	CPR+DOX+TMP	STR+DOX+TMP	AMP+CPR+STR+DOX	AMP+CPR+STR+TMP	AMP+CPR+DOX+TMP	AMP+STR+DOX+TMP	CPR+STR+DOX+TMP	AMP+CPR+STR+DOX+TMP
	100.0524246	86.21231979	46.67078443	99.04785156	83.22753906	98.16513761	44.07601573	93.76146789	88.51900393	58.15203145	103.8269987	95.64875491	62.44595429	49.20321186	79.51660156	3.211009174	88.51900393	88.09960682	21.42857143	21.42857143	88.72870249	66.74967235	28.55832241	97.7457405	12.0444719	38.83355177	22.0576671	103.8269987	33.38138925	31.07470511	22.89646134
	81.40933455	102.3271016	50.16916385	101.2874474	87.62069819	84.12864427	36.71068113	110.2758531	105.255589	56.92247353	105.4647666	97.09765982	54.22909618	54.03576607	82.47090864	4.915675252	108.1840763	102.3271016	48.215453	7.778794614	108.8116094	67.79971238	5.0594849	93.12328409	12.27646206	4.85030723	3.45143156	109.4391424	9.099228657	7.765721009	5.896195581
	101.7646201	104.3313402	59.93076035	100.0881501	85.17818914	103.8646638	39.50707306	110.3981333	101.0646055	64.13883623	113.8982062	108.0647513	58.93063213	57.08424157	84.37224531	9.873122357	104.3313402	101.0646055	34.37363278	0.904185504	104.3313402	70.20562928	62.14087793	94.29779787	28.13181177	43.47382237	35.07364737	102.6979729	49.07393904	69.84103835	4.273005688
AMP3CPR3STR2DOX3TMP2	AMP	CPR	STR	DOX	TMP	AMP+CPR	AMP+STR	AMP+DOX	AMP+TMP	CPR+STR	CPR+DOX	CPR+TMP	STR+DOX	STR+TMP	DOX+TMP	AMP+CPR+STR	AMP+CPR+DOX	AMP+CPR+TMP	AMP+STR+DOX	AMP+STR+TMP	AMP+DOX+TMP	CPR+STR+DOX	CPR+STR+TMP	CPR+DOX+TMP	STR+DOX+TMP	AMP+CPR+STR+DOX	AMP+CPR+STR+TMP	AMP+CPR+DOX+TMP	AMP+STR+DOX+TMP	CPR+STR+DOX+TMP	AMP+CPR+STR+DOX+TMP
	100.0524246	86.21231979	46.67078443	99.04785156	91.04003906	98.16513761	44.07601573	93.76146789	104.2463958	58.15203145	103.8269987	101.1009174	62.44595429	63.03891291	77.56347656	3.211009174	88.51900393	90.82568807	21.42857143	3.211009174	86.63171691	66.74967235	58.12581913	97.7457405	52.40271773	38.83355177	7.378768021	99.42332896	2.791612058	44.94102228	0.655307995
	81.40933455	102.3271016	50.16916385	101.2874474	93.36469423	84.12864427	36.71068113	110.2758531	107.5565433	56.92247353	105.4647666	103.1638123	54.22909618	54.80908652	85.83807873	4.915675252	108.1840763	102.9546346	48.215453	7.778794614	104.8372336	67.79971238	5.26866257	93.33246176	51.71580474	4.85030723	3.595241208	104.0005229	4.431951889	38.80245784	7.569616943
	101.7646201	104.3313402	59.93076035	100.0881501	93.84208538	103.8646638	39.50707306	110.3981333	111.7981625	64.13883623	113.8982062	106.6647222	58.93063213	73.08629311	95.65545901	9.873122357	104.3313402	112.2648389	34.37363278	40.67376404	105.7313694	70.20562928	39.04039667	99.19789996	59.52045134	43.47382237	28.30683973	104.098002	5.104273006	1.837538282	6.270963978
AMP3CPR3STR2DOX3TMP3	AMP	CPR	STR	DOX	TMP	AMP+CPR	AMP+STR	AMP+DOX	AMP+TMP	CPR+STR	CPR+DOX	CPR+TMP	STR+DOX	STR+TMP	DOX+TMP	AMP+CPR+STR	AMP+CPR+DOX	AMP+CPR+TMP	AMP+STR+DOX	AMP+STR+TMP	AMP+DOX+TMP	CPR+STR+DOX	CPR+STR+TMP	CPR+DOX+TMP	STR+DOX+TMP	AMP+CPR+STR+DOX	AMP+CPR+STR+TMP	AMP+CPR+DOX+TMP	AMP+STR+DOX+TMP	CPR+STR+DOX+TMP	AMP+CPR+STR+DOX+TMP
	100.0524246	86.21231979	46.67078443	99.04785156	88.11035156	98.16513761	44.07601573	93.76146789	103.8269987	58.15203145	103.8269987	100.2621232	62.44595429	69.75911056	93.96972656	3.211009174	88.51900393	93.76146789	21.42857143	22.0576671	97.11664482	66.74967235	67.77195282	93.55176933	45.28721433	38.83355177	10.94364351	108.2306684	5.897771953	65.88466579	24.36435125
	81.40933455	102.3271016	50.16916385	101.2874474	91.97821243	84.12864427	36.71068113	110.2758531	106.9290103	56.92247353	105.4647666	98.77108119	54.22909618	54.71242146	101.0893786	4.915675252	108.1840763	103.1638123	48.215453	51.27467643	102.9546346	67.79971238	23.32331024	98.77108119	52.13871435	4.85030723	3.386063538	112.1584521	6.523728592	69.13322003	53.65407243
	101.7646201	104.3313402	59.93076035	100.0881501	96.6628888	103.8646638	39.50707306	110.3981333	108.9981041	64.13883623	113.8982062	99.66457635	58.93063213	71.65021157	104.5208412	9.873122357	104.3313402	103.1646493	34.37363278	3.704243838	109.2314423	70.20562928	43.24048418	105.264693	64.239005	43.47382237	2.537552866	109.6981187	7.437654951	57.00743765	19.67332653
AMP3CPR3STR3DOX1TMP1	AMP	CPR	STR	DOX	TMP	AMP+CPR	AMP+STR	AMP+DOX	AMP+TMP	CPR+STR	CPR+DOX	CPR+TMP	STR+DOX	STR+TMP	DOX+TMP	AMP+CPR+STR	AMP+CPR+DOX	AMP+CPR+TMP	AMP+STR+DOX	AMP+STR+TMP	AMP+DOX+TMP	CPR+STR+DOX	CPR+STR+TMP	CPR+DOX+TMP	STR+DOX+TMP	AMP+CPR+STR+DOX	AMP+CPR+STR+TMP	AMP+CPR+DOX+TMP	AMP+STR+DOX+TMP	CPR+STR+DOX+TMP	AMP+CPR+STR+DOX+TMP
	100.0524246	86.21231979	93.55176933	103.9306641	83.22753906	98.16513761	62.5163827	98.37483617	88.51900393	72.5819135	101.5203145	95.64875491	83.48623853	89.56749672	83.61816406	38.85976409	93.34207077	88.09960682	33.38138925	53.11926606	96.27785059	53.32896461	60.66841415	90.40629096	49.97378768	21.42857143	45.98951507	95.43905636	51.23197903	44.52162516	54.16775885
	81.40933455	102.3271016	85.80206563	102.6739292	87.62069819	84.12864427	61.74663355	105.4647666	105.255589	68.64949667	106.0922996	97.09765982	93.33246176	96.88848215	76.7269126	38.31873448	106.510655	102.3271016	47.37874232	48.34618904	97.3068375	58.38671722	64.45286966	86.22042097	53.15727546	19.76728984	45.62687933	96.26094914	51.4838541	21.4407112	33.78219375
	101.7646201	104.3313402	94.53113607	98.47626244	85.17818914	103.8646638	74.23071314	104.3313402	101.0646055	74.46405133	103.8646638	108.0647513	92.43109231	96.63117982	85.17818914	47.86349716	112.2648389	101.0646055	37.64036751	56.672014	89.63103398	65.77220359	71.37232026	82.86422634	57.83870497	38.57372029	60.17208692	108.2980895	64.60551261	73.47236401	51.07189733
AMP3CPR3STR3DOX1TMP2	AMP	CPR	STR	DOX	TMP	AMP+CPR	AMP+STR	AMP+DOX	AMP+TMP	CPR+STR	CPR+DOX	CPR+TMP	STR+DOX	STR+TMP	DOX+TMP	AMP+CPR+STR	AMP+CPR+DOX	AMP+CPR+TMP	AMP+STR+DOX	AMP+STR+TMP	AMP+DOX+TMP	CPR+STR+DOX	CPR+STR+TMP	CPR+DOX+TMP	STR+DOX+TMP	AMP+CPR+STR+DOX	AMP+CPR+STR+TMP	AMP+CPR+DOX+TMP	AMP+STR+DOX+TMP	CPR+STR+DOX+TMP	AMP+CPR+STR+DOX+TMP
	100.0524246	86.21231979	93.55176933	103.9306641	91.04003906	98.16513761	62.5163827	98.37483617	104.2463958	72.5819135	101.5203145	101.1009174	83.48623853	88.30930537	84.20410156	38.85976409	93.34207077	90.82568807	33.38138925	52.49017038	98.16513761	53.32896461	59.61992136	89.35779817	42.1887287	21.42857143	57.10353866	98.16513761	37.57536042	46.40891219	39.27916121
	81.40933455	102.3271016	85.80206563	102.6739292	93.36469423	84.12864427	61.74663355	105.4647666	107.5565433	68.64949667	106.0922996	103.1638123	93.33246176	92.91410642	80.49022035	38.31873448	106.510655	102.9546346	47.37874232	52.94809779	96.26094914	58.38671722	66.33546869	94.16917244	37.33821415	19.76728984	36.84141718	109.2299647	23.53248791	40.60661524	39.63916852
	101.7646201	104.3313402	94.53113607	98.47626244	93.84208538	103.8646638	74.23071314	104.3313402	111.7981625	74.46405133	103.8646638	106.6647222	92.43109231	96.86451801	87.79750661	47.86349716	112.2648389	112.2648389	37.64036751	54.33863205	91.73107773	65.77220359	73.23902581	85.43094648	54.44071751	38.57372029	60.40542511	112.0315007	58.77205775	55.27198483	44.64051334
AMP3CPR3STR3DOX1TMP3	AMP	CPR	STR	DOX	TMP	AMP+CPR	AMP+STR	AMP+DOX	AMP+TMP	CPR+STR	CPR+DOX	CPR+TMP	STR+DOX	STR+TMP	DOX+TMP	AMP+CPR+STR	AMP+CPR+DOX	AMP+CPR+TMP	AMP+STR+DOX	AMP+STR+TMP	AMP+DOX+TMP	CPR+STR+DOX	CPR+STR+TMP	CPR+DOX+TMP	STR+DOX+TMP	AMP+CPR+STR+DOX	AMP+CPR+STR+TMP	AMP+CPR+DOX+TMP	AMP+STR+DOX+TMP	CPR+STR+DOX+TMP	AMP+CPR+STR+DOX+TMP
	100.0524246	86.21231979	93.55176933	103.9306641	88.11035156	98.16513761	62.5163827	98.37483617	103.8269987	72.5819135	101.5203145	100.2621232	83.48623853	94.60026212	94.36035156	38.85976409	93.34207077	93.76146789	33.38138925	54.75753604	101.310616	53.32896461	80.1310616	110.9567497	56.68414155	21.42857143	48.08650066	104.4560944	46.40891219	68.84665793	40.93053735
	81.40933455	102.3271016	85.80206563	102.6739292	91.97821243	84.12864427	61.74663355	105.4647666	106.9290103	68.64949667	106.0922996	98.77108119	93.33246176	94.16917244	99.10869027	38.31873448	106.510655	103.1638123	47.37874232	57.98143548	106.0922996	58.38671722	56.08576284	107.138188	58.59589489	19.76728984	44.17570924	102.3271016	35.45561511	58.17753955	7.778794614
	101.7646201	104.3313402	94.53113607	98.47626244	96.6628888	103.8646638	74.23071314	104.3313402	108.9981041	74.46405133	103.8646638	99.66457635	92.43109231	93.1311069	109.1550183	47.86349716	112.2648389	103.1646493	37.64036751	49.67186816	114.1315444	65.77220359	57.37202858	112.498177	45.93845705	38.57372029	64.19717077	104.3313402	67.63890914	74.87239317	46.27388071
AMP3CPR3STR3DOX2TMP1	AMP	CPR	STR	DOX	TMP	AMP+CPR	AMP+STR	AMP+DOX	AMP+TMP	CPR+STR	CPR+DOX	CPR+TMP	STR+DOX	STR+TMP	DOX+TMP	AMP+CPR+STR	AMP+CPR+DOX	AMP+CPR+TMP	AMP+STR+DOX	AMP+STR+TMP	AMP+DOX+TMP	CPR+STR+DOX	CPR+STR+TMP	CPR+DOX+TMP	STR+DOX+TMP	AMP+CPR+STR+DOX	AMP+CPR+STR+TMP	AMP+CPR+DOX+TMP	AMP+STR+DOX+TMP	CPR+STR+DOX+TMP	AMP+CPR+STR+DOX+TMP
	100.0524246	86.21231979	93.55176933	100.6103516	83.22753906	98.16513761	62.5163827	92.50327654	88.51900393	72.5819135	96.69724771	95.64875491	95.64875491	89.56749672	83.81347656	38.85976409	85.37352556	88.09960682	56.22542595	53.11926606	105.2948886	64.82306684	60.66841415	103.197903	51.86107471	56.68414155	45.98951507	98.16513761	48.71559633	68.0078637	45.57011796
	81.40933455	102.3271016	85.80206563	102.2777915	87.62069819	84.12864427	61.74663355	106.7198327	105.255589	68.64949667	105.255589	97.09765982	96.67930448	96.88848215	84.84773459	38.31873448	106.9290103	102.3271016	58.39979082	48.34618904	106.3014773	64.8842986	64.45286966	92.70492875	51.69303177	50.64714342	45.62687933	96.47012681	47.71865603	62.98862596	52.94809779
	101.7646201	104.3313402	94.53113607	98.07329052	85.17818914	103.8646638	74.23071314	102.4646347	101.0646055	74.46405133	104.098002	108.0647513	99.19789996	96.63117982	95.65545901	47.86349716	112.9648534	101.0646055	72.83068397	56.672014	108.0647513	71.19731661	71.37232026	105.7313694	49.20519177	51.77191191	60.17208692	94.29779787	57.13869039	72.07233484	51.77191191
AMP3CPR3STR3DOX2TMP2	AMP	CPR	STR	DOX	TMP	AMP+CPR	AMP+STR	AMP+DOX	AMP+TMP	CPR+STR	CPR+DOX	CPR+TMP	STR+DOX	STR+TMP	DOX+TMP	AMP+CPR+STR	AMP+CPR+DOX	AMP+CPR+TMP	AMP+STR+DOX	AMP+STR+TMP	AMP+DOX+TMP	CPR+STR+DOX	CPR+STR+TMP	CPR+DOX+TMP	STR+DOX+TMP	AMP+CPR+STR+DOX	AMP+CPR+STR+TMP	AMP+CPR+DOX+TMP	AMP+STR+DOX+TMP	CPR+STR+DOX+TMP	AMP+CPR+STR+DOX+TMP
	100.0524246	86.21231979	93.55176933	100.6103516	91.04003906	98.16513761	62.5163827	92.50327654	104.2463958	72.5819135	96.69724771	101.1009174	95.64875491	88.30930537	88.69628906	38.85976409	85.37352556	90.82568807	56.22542595	52.49017038	105.9239843	64.82306684	59.61992136	104.6657929	59.82961992	56.68414155	57.10353866	107.1821756	48.29619921	67.37876802	49.34469201
	81.40933455	102.3271016	85.80206563	102.2777915	93.36469423	84.12864427	61.74663355	106.7198327	107.5565433	68.64949667	105.255589	103.1638123	96.67930448	92.91410642	83.26318396	38.31873448	106.9290103	102.9546346	58.39979082	52.94809779	107.5565433	64.8842986	66.33546869	105.0464113	61.73355994	50.64714342	36.84141718	110.0666754	48.55536671	68.00889005	54.62151915
	101.7646201	104.3313402	94.53113607	98.07329052	93.84208538	103.8646638	74.23071314	102.4646347	111.7981625	74.46405133	104.098002	106.6647222	99.19789996	96.86451801	96.6628888	47.86349716	112.9648534	112.2648389	72.83068397	54.33863205	107.598075	71.19731661	73.23902581	110.6314715	62.50546886	51.77191191	60.40542511	108.5314277	60.63876331	74.63905498	51.53857372
AMP3CPR3STR3DOX2TMP3	AMP	CPR	STR	DOX	TMP	AMP+CPR	AMP+STR	AMP+DOX	AMP+TMP	CPR+STR	CPR+DOX	CPR+TMP	STR+DOX	STR+TMP	DOX+TMP	AMP+CPR+STR	AMP+CPR+DOX	AMP+CPR+TMP	AMP+STR+DOX	AMP+STR+TMP	AMP+DOX+TMP	CPR+STR+DOX	CPR+STR+TMP	CPR+DOX+TMP	STR+DOX+TMP	AMP+CPR+STR+DOX	AMP+CPR+STR+TMP	AMP+CPR+DOX+TMP	AMP+STR+DOX+TMP	CPR+STR+DOX+TMP	AMP+CPR+STR+DOX+TMP
	100.0524246	86.21231979	93.55176933	100.6103516	88.11035156	98.16513761	62.5163827	92.50327654	103.8269987	72.5819135	96.69724771	100.2621232	95.64875491	94.60026212	94.55566406	38.85976409	85.37352556	93.76146789	56.22542595	54.75753604	104.2463958	64.82306684	80.1310616	101.7300131	70.90432503	56.68414155	48.08650066	101.5203145	45.95019659	63.18479685	48.29619921
	81.40933455	102.3271016	85.80206563	102.2777915	91.97821243	84.12864427	61.74663355	106.7198327	106.9290103	68.64949667	105.255589	98.77108119	96.67930448	94.16917244	86.03614756	38.31873448	106.9290103	103.1638123	58.39979082	57.98143548	104.2097006	64.8842986	56.08576284	102.1179239	67.81278599	50.64714342	44.17570924	104.8372336	45.62687933	61.3152046	51.4838541
	101.7646201	104.3313402	94.53113607	98.07329052	96.6628888	103.8646638	74.23071314	102.4646347	108.9981041	74.46405133	104.098002	99.66457635	99.19789996	93.1311069	97.46883264	47.86349716	112.9648534	103.1646493	72.83068397	49.67186816	103.8646638	71.19731661	57.37202858	103.1646493	70.73064022	51.77191191	64.19717077	102.2312965	55.50532303	75.33906956	52.93860289
AMP3CPR3STR3DOX3TMP1	AMP	CPR	STR	DOX	TMP	AMP+CPR	AMP+STR	AMP+DOX	AMP+TMP	CPR+STR	CPR+DOX	CPR+TMP	STR+DOX	STR+TMP	DOX+TMP	AMP+CPR+STR	AMP+CPR+DOX	AMP+CPR+TMP	AMP+STR+DOX	AMP+STR+TMP	AMP+DOX+TMP	CPR+STR+DOX	CPR+STR+TMP	CPR+DOX+TMP	STR+DOX+TMP	AMP+CPR+STR+DOX	AMP+CPR+STR+TMP	AMP+CPR+DOX+TMP	AMP+STR+DOX+TMP	CPR+STR+DOX+TMP	AMP+CPR+STR+DOX+TMP
	100.0524246	86.21231979	93.55176933	99.04785156	83.22753906	98.16513761	62.5163827	93.76146789	88.51900393	72.5819135	103.8269987	95.64875491	91.03538663	89.56749672	79.51660156	38.85976409	88.51900393	88.09960682	50.81258191	53.11926606	88.72870249	60.87811271	60.66841415	97.7457405	72.16251638	52.49017038	45.98951507	103.8269987	51.86107471	71.36304063	46.82830931
	81.40933455	102.3271016	85.80206563	101.2874474	87.62069819	84.12864427	61.74663355	110.2758531	105.255589	68.64949667	105.4647666	97.09765982	94.37835011	96.88848215	82.47090864	38.31873448	108.1840763	102.3271016	43.95345797	48.34618904	108.8116094	59.85096091	64.45286966	93.12328409	74.50647143	43.95345797	45.62687933	109.4391424	51.06549876	54.20316381	39.35154922
	101.7646201	104.3313402	94.53113607	100.0881501	85.17818914	103.8646638	74.23071314	110.3981333	101.0646055	74.46405133	113.8982062	108.0647513	96.86451801	96.63117982	84.37224531	47.86349716	104.3313402	101.0646055	46.69680618	56.672014	104.3313402	63.90549803	71.37232026	94.29779787	78.66413884	59.53040688	60.17208692	102.6979729	64.83885081	52.4719265	49.90520636
AMP3CPR3STR3DOX3TMP2	AMP	CPR	STR	DOX	TMP	AMP+CPR	AMP+STR	AMP+DOX	AMP+TMP	CPR+STR	CPR+DOX	CPR+TMP	STR+DOX	STR+TMP	DOX+TMP	AMP+CPR+STR	AMP+CPR+DOX	AMP+CPR+TMP	AMP+STR+DOX	AMP+STR+TMP	AMP+DOX+TMP	CPR+STR+DOX	CPR+STR+TMP	CPR+DOX+TMP	STR+DOX+TMP	AMP+CPR+STR+DOX	AMP+CPR+STR+TMP	AMP+CPR+DOX+TMP	AMP+STR+DOX+TMP	CPR+STR+DOX+TMP	AMP+CPR+STR+DOX+TMP
	100.0524246	86.21231979	93.55176933	99.04785156	91.04003906	98.16513761	62.5163827	93.76146789	104.2463958	72.5819135	103.8269987	101.1009174	91.03538663	88.30930537	77.56347656	38.85976409	88.51900393	90.82568807	50.81258191	52.49017038	86.63171691	60.87811271	59.61992136	97.7457405	72.37221494	52.49017038	57.10353866	99.42332896	50.3931848	67.58846658	44.52162516
	81.40933455	102.3271016	85.80206563	101.2874474	93.36469423	84.12864427	61.74663355	110.2758531	107.5565433	68.64949667	105.4647666	103.1638123	94.37835011	92.91410642	85.83807873	38.31873448	108.1840763	102.9546346	43.95345797	52.94809779	104.8372336	59.85096091	66.33546869	93.33246176	85.38371029	43.95345797	36.84141718	104.0005229	50.43796575	63.82533665	42.98601124
	101.7646201	104.3313402	94.53113607	100.0881501	93.84208538	103.8646638	74.23071314	110.3981333	111.7981625	74.46405133	113.8982062	106.6647222	96.86451801	96.86451801	95.65545901	47.86349716	104.3313402	112.2648389	46.69680618	54.33863205	105.7313694	63.90549803	73.23902581	99.19789996	97.09785621	59.53040688	60.40542511	104.098002	58.07204317	71.83899665	57.13869039
AMP3CPR3STR3DOX3TMP3	AMP	CPR	STR	DOX	TMP	AMP+CPR	AMP+STR	AMP+DOX	AMP+TMP	CPR+STR	CPR+DOX	CPR+TMP	STR+DOX	STR+TMP	DOX+TMP	AMP+CPR+STR	AMP+CPR+DOX	AMP+CPR+TMP	AMP+STR+DOX	AMP+STR+TMP	AMP+DOX+TMP	CPR+STR+DOX	CPR+STR+TMP	CPR+DOX+TMP	STR+DOX+TMP	AMP+CPR+STR+DOX	AMP+CPR+STR+TMP	AMP+CPR+DOX+TMP	AMP+STR+DOX+TMP	CPR+STR+DOX+TMP	AMP+CPR+STR+DOX+TMP
	100.0524246	86.21231979	93.55176933	99.04785156	88.11035156	98.16513761	62.5163827	93.76146789	103.8269987	72.5819135	103.8269987	100.2621232	91.03538663	94.60026212	93.96972656	38.85976409	88.51900393	93.76146789	50.81258191	54.75753604	97.11664482	60.87811271	80.1310616	93.55176933	66.50065531	52.49017038	48.08650066	108.2306684	53.07994758	58.15203145	46.82830931
	81.40933455	102.3271016	85.80206563	101.2874474	91.97821243	84.12864427	61.74663355	110.2758531	106.9290103	68.64949667	105.4647666	98.77108119	94.37835011	94.16917244	101.0893786	38.31873448	108.1840763	103.1638123	43.95345797	57.98143548	102.9546346	59.85096091	56.08576284	98.77108119	73.25140541	43.95345797	44.17570924	112.1584521	46.88194535	66.33546869	45.84913061
	101.7646201	104.3313402	94.53113607	100.0881501	96.6628888	103.8646638	74.23071314	110.3981333	108.9981041	74.46405133	113.8982062	99.66457635	96.86451801	93.1311069	104.5208412	47.86349716	104.3313402	103.1646493	46.69680618	49.67186816	109.2314423	63.90549803	57.37202858	105.264693	80.064168	59.53040688	64.19717077	109.6981187	51.77191191	63.90549803	65.072189
AMP1CPR1STR1ERY1DOX1	AMP	CPR	STR	ERY	DOX	AMP+CPR	AMP+STR	AMP+ERY	AMP+DOX	CPR+STR	CPR+ERY	CPR+DOX	STR+ERY	STR+DOX	ERY+DOX	AMP+CPR+STR	AMP+CPR+ERY	AMP+CPR+DOX	AMP+STR+ERY	AMP+STR+DOX	AMP+ERY+DOX	CPR+STR+ERY	CPR+STR+DOX	CPR+ERY+DOX	STR+ERY+DOX	AMP+CPR+STR+ERY	AMP+CPR+STR+DOX	AMP+CPR+ERY+DOX	AMP+STR+ERY+DOX	CPR+STR+ERY+DOX	AMP+CPR+STR+ERY+DOX
	58.58840475	91.42960029	92.00576161	83.6514224	101.2243428	61.46921138	21.33597407	50.5221462	44.76053295	75.87324451	86.82030969	100.3601008	78.46597047	96.03889089	95.46272956	27.22362261	57.4360821	55.95966871	1.260352899	8.174288801	47.06517825	80.77061577	69.24738927	76.7374865	71.84011523	35.83003241	23.44256392	55.1314368	2.124594887	45.01260353	30.35649982
	79.70049917	99.22351636	85.0249584	82.95433537	100.9983361	53.96561287	3.679053429	68.45997412	46.9033093	90.64522093	84.72915511	100.4067295	79.40469588	92.71584396	95.67387687	22.51802551	56.92364578	62.5439083	0.12941394	-1.904233685	53.66980958	88.2787946	76.15085968	88.57459789	82.36272879	43.52005916	35.03420226	56.33203919	11.4069144	49.56553892	6.248844518
	65.22658143	104.4903851	94.26199928	90.30262412	96.57163479	56.64793525	6.784554313	54.9981956	50.04897665	81.06408207	94.59194721	108.7797082	84.36356138	93.60210342	93.27215549	38.19147291	54.66824767	61.92710213	9.094189823	4.144970872	49.38908079	83.37371759	67.53621694	75.12501933	77.43465484	25.98339949	38.50079909	31.24194463	4.804866732	42.56328298	31.92246224
AMP1CPR1STR1ERY1DOX2	AMP	CPR	STR	ERY	DOX	AMP+CPR	AMP+STR	AMP+ERY	AMP+DOX	CPR+STR	CPR+ERY	CPR+DOX	STR+ERY	STR+DOX	ERY+DOX	AMP+CPR+STR	AMP+CPR+ERY	AMP+CPR+DOX	AMP+STR+ERY	AMP+STR+DOX	AMP+ERY+DOX	CPR+STR+ERY	CPR+STR+DOX	CPR+ERY+DOX	STR+ERY+DOX	AMP+CPR+STR+ERY	AMP+CPR+STR+DOX	AMP+CPR+ERY+DOX	AMP+STR+ERY+DOX	CPR+STR+ERY+DOX	AMP+CPR+STR+ERY+DOX
	58.58840475	91.42960029	92.00576161	83.6514224	95.46272956	61.46921138	21.33597407	50.5221462	42.74396831	75.87324451	86.82030969	80.19445445	78.46597047	98.91969752	99.20777818	27.22362261	57.4360821	47.35325891	1.260352899	19.98559597	45.62477494	80.77061577	52.25063018	81.92293842	81.3467771	35.83003241	34.06553835	41.87972632	-1.422398272	47.64133958	34.67770976
	79.70049917	99.22351636	85.0249584	82.95433537	98.0403032	53.96561287	3.679053429	68.45997412	51.59918654	90.64522093	84.72915511	87.68718802	79.40469588	92.71584396	103.956369	22.51802551	56.92364578	52.19079312	0.12941394	20.87261971	51.63616195	88.2787946	50.71177667	90.64522093	89.16620447	43.52005916	43.90830098	45.38731743	5.786651877	53.66980958	42.42928453
	65.22658143	104.4903851	94.26199928	90.30262412	99.54116616	56.64793525	6.784554313	54.9981956	59.28751869	81.06408207	94.59194721	110.4294479	84.36356138	95.91173893	102.5106975	38.19147291	54.66824767	53.34845595	9.094189823	26.58143012	44.1099139	83.37371759	46.52265814	63.57684178	74.13517554	25.98339949	40.8104346	29.92215291	4.804866732	59.28751869	31.26256638
AMP1CPR1STR1ERY1DOX3	AMP	CPR	STR	ERY	DOX	AMP+CPR	AMP+STR	AMP+ERY	AMP+DOX	CPR+STR	CPR+ERY	CPR+DOX	STR+ERY	STR+DOX	ERY+DOX	AMP+CPR+STR	AMP+CPR+ERY	AMP+CPR+DOX	AMP+STR+ERY	AMP+STR+DOX	AMP+ERY+DOX	CPR+STR+ERY	CPR+STR+DOX	CPR+ERY+DOX	STR+ERY+DOX	AMP+CPR+STR+ERY	AMP+CPR+STR+DOX	AMP+CPR+ERY+DOX	AMP+STR+ERY+DOX	CPR+STR+ERY+DOX	AMP+CPR+STR+ERY+DOX
	58.58840475	91.42960029	92.00576161	83.6514224	101.2243428	61.46921138	21.33597407	50.5221462	45.62477494	75.87324451	86.82030969	74.14476053	78.46597047	81.63485776	98.63161685	27.22362261	57.4360821	70.68779258	1.260352899	9.326611451	43.03204897	80.77061577	44.18437162	61.18113072	75.29708318	35.83003241	16.52862802	39.28700036	-2.232625135	51.06229744	35.25387108
	79.70049917	99.22351636	85.0249584	82.95433537	101.2941394	53.96561287	3.679053429	68.45997412	56.92364578	90.64522093	84.72915511	90.64522093	79.40469588	99.51931965	101.885746	22.51802551	56.92364578	72.00961361	0.12941394	12.59012757	41.54187465	88.2787946	71.71381032	81.17951562	77.33407284	43.52005916	2.107598447	48.64115363	1.349602514	80.92068774	37.40062858
	65.22658143	104.4903851	94.26199928	90.30262412	101.8508017	56.64793525	6.784554313	54.9981956	58.62762283	81.06408207	94.59194721	93.60210342	84.36356138	104.1604372	102.1807496	38.19147291	54.66824767	79.41434242	9.094189823	2.495231221	52.35861216	83.37371759	71.82554003	73.80522761	75.78491519	25.98339949	24.3130381	26.29272568	4.474918802	41.57343919	19.36381915
AMP1CPR1STR1ERY2DOX1	AMP	CPR	STR	ERY	DOX	AMP+CPR	AMP+STR	AMP+ERY	AMP+DOX	CPR+STR	CPR+ERY	CPR+DOX	STR+ERY	STR+DOX	ERY+DOX	AMP+CPR+STR	AMP+CPR+ERY	AMP+CPR+DOX	AMP+STR+ERY	AMP+STR+DOX	AMP+ERY+DOX	CPR+STR+ERY	CPR+STR+DOX	CPR+ERY+DOX	STR+ERY+DOX	AMP+CPR+STR+ERY	AMP+CPR+STR+DOX	AMP+CPR+ERY+DOX	AMP+STR+ERY+DOX	CPR+STR+ERY+DOX	AMP+CPR+STR+ERY+DOX
	58.58840475	91.42960029	92.00576161	96.90313288	101.2243428	61.46921138	21.33597407	45.01260353	44.76053295	75.87324451	88.54879366	100.3601008	91.42960029	96.03889089	99.7839395	27.22362261	46.16492618	55.95966871	4.051134318	8.174288801	57.14800144	69.24738927	69.24738927	60.60496939	69.24738927	29.49225783	23.44256392	61.46921138	-1.368383147	59.99279798	41.87972632
	79.70049917	99.22351636	85.0249584	96.56128674	100.9983361	53.96561287	3.679053429	64.31872805	46.9033093	90.64522093	93.30745055	100.4067295	88.87040118	92.71584396	106.0269921	22.51802551	53.96561287	62.5439083	1.201700869	-1.904233685	61.95230172	79.10889259	76.15085968	87.98299131	90.94102422	31.09632095	35.03420226	68.16417083	2.532815678	69.97596598	49.23276021
	65.22658143	104.4903851	94.26199928	96.90158272	96.57163479	56.64793525	6.784554313	41.90338712	50.04897665	81.06408207	96.90158272	108.7797082	91.62241584	93.60210342	99.54116616	38.19147291	57.96772697	61.92710213	22.29210703	4.144970872	45.42970562	80.07423828	67.53621694	80.73413414	84.69350931	33.9846368	38.50079909	57.32845285	2.165283291	53.01850802	31.92246224
AMP1CPR1STR1ERY2DOX2	AMP	CPR	STR	ERY	DOX	AMP+CPR	AMP+STR	AMP+ERY	AMP+DOX	CPR+STR	CPR+ERY	CPR+DOX	STR+ERY	STR+DOX	ERY+DOX	AMP+CPR+STR	AMP+CPR+ERY	AMP+CPR+DOX	AMP+STR+ERY	AMP+STR+DOX	AMP+ERY+DOX	CPR+STR+ERY	CPR+STR+DOX	CPR+ERY+DOX	STR+ERY+DOX	AMP+CPR+STR+ERY	AMP+CPR+STR+DOX	AMP+CPR+ERY+DOX	AMP+STR+ERY+DOX	CPR+STR+ERY+DOX	AMP+CPR+STR+ERY+DOX
	58.58840475	91.42960029	92.00576161	96.90313288	95.46272956	61.46921138	21.33597407	45.01260353	42.74396831	75.87324451	88.54879366	80.19445445	91.42960029	98.91969752	99.20777818	27.22362261	46.16492618	47.35325891	4.051134318	19.98559597	52.25063018	69.24738927	52.25063018	69.24738927	74.4328412	29.49225783	34.06553835	50.23406554	15.66438603	61.4332013	23.19049334
	79.70049917	99.22351636	85.0249584	96.56128674	98.0403032	53.96561287	3.679053429	64.31872805	51.59918654	90.64522093	93.30745055	87.68718802	88.87040118	92.71584396	106.6185986	22.51802551	53.96561287	52.19079312	1.201700869	20.87261971	58.69846552	79.10889259	50.71177667	84.43335182	89.75781106	31.09632095	43.90830098	77.03826955	18.80199667	71.45498244	35.33000555
	65.22658143	104.4903851	94.26199928	96.90158272	99.54116616	56.64793525	6.784554313	41.90338712	59.28751869	81.06408207	96.90158272	110.4294479	91.62241584	95.91173893	103.1705934	38.19147291	57.96772697	53.34845595	22.29210703	26.58143012	45.75965355	80.07423828	46.52265814	57.63777904	72.48543589	33.9846368	40.8104346	60.95788008	17.34288808	72.25859669	23.67376398
AMP1CPR1STR1ERY2DOX3	AMP	CPR	STR	ERY	DOX	AMP+CPR	AMP+STR	AMP+ERY	AMP+DOX	CPR+STR	CPR+ERY	CPR+DOX	STR+ERY	STR+DOX	ERY+DOX	AMP+CPR+STR	AMP+CPR+ERY	AMP+CPR+DOX	AMP+STR+ERY	AMP+STR+DOX	AMP+ERY+DOX	CPR+STR+ERY	CPR+STR+DOX	CPR+ERY+DOX	STR+ERY+DOX	AMP+CPR+STR+ERY	AMP+CPR+STR+DOX	AMP+CPR+ERY+DOX	AMP+STR+ERY+DOX	CPR+STR+ERY+DOX	AMP+CPR+STR+ERY+DOX
	58.58840475	91.42960029	92.00576161	96.90313288	101.2243428	61.46921138	21.33597407	45.01260353	45.62477494	75.87324451	88.54879366	74.14476053	91.42960029	81.63485776	102.0885848	27.22362261	46.16492618	70.68779258	4.051134318	9.326611451	61.75729204	69.24738927	44.18437162	71.55203457	77.31364782	29.49225783	16.52862802	40.43932301	6.733885488	51.92653943	27.79978394
	79.70049917	99.22351636	85.0249584	96.56128674	101.2941394	53.96561287	3.679053429	64.31872805	56.92364578	90.64522093	93.30745055	90.64522093	88.87040118	99.51931965	110.7598447	22.51802551	53.96561287	72.00961361	1.201700869	12.59012757	51.89498983	79.10889259	71.71381032	88.87040118	94.19486042	31.09632095	2.107598447	62.5439083	1.349602514	48.97393233	39.0830098
	65.22658143	104.4903851	94.26199928	96.90158272	101.8508017	56.64793525	6.784554313	41.90338712	58.62762283	81.06408207	96.90158272	93.60210342	91.62241584	104.1604372	95.91173893	38.19147291	57.96772697	79.41434242	22.29210703	2.495231221	61.92710213	80.07423828	71.82554003	50.72949425	73.47527968	33.9846368	24.3130381	36.87168119	4.144970872	53.01850802	31.59251431
AMP1CPR1STR1ERY3DOX1	AMP	CPR	STR	ERY	DOX	AMP+CPR	AMP+STR	AMP+ERY	AMP+DOX	CPR+STR	CPR+ERY	CPR+DOX	STR+ERY	STR+DOX	ERY+DOX	AMP+CPR+STR	AMP+CPR+ERY	AMP+CPR+DOX	AMP+STR+ERY	AMP+STR+DOX	AMP+ERY+DOX	CPR+STR+ERY	CPR+STR+DOX	CPR+ERY+DOX	STR+ERY+DOX	AMP+CPR+STR+ERY	AMP+CPR+STR+DOX	AMP+CPR+ERY+DOX	AMP+STR+ERY+DOX	CPR+STR+ERY+DOX	AMP+CPR+STR+ERY+DOX
	58.58840475	91.42960029	92.00576161	93.44616493	101.2243428	61.46921138	21.33597407	49.62189413	44.76053295	75.87324451	94.31040691	100.3601008	88.83687432	96.03889089	97.4792942	27.22362261	54.23118473	55.95966871	13.84587685	8.174288801	79.90637378	64.35001801	69.24738927	75.00900252	83.36334174	36.73028448	23.44256392	51.09830753	7.886208138	55.09542672	22.90241268
	79.70049917	99.22351636	85.0249584	94.49066371	100.9983361	53.96561287	3.679053429	55.74043261	46.9033093	90.64522093	86.20817157	100.4067295	85.0249584	92.71584396	107.8018118	22.51802551	49.52856351	62.5439083	15.21538177	-1.904233685	64.61453134	73.48863006	76.15085968	96.26548345	95.08227029	33.16694398	35.03420226	68.16417083	2.495840266	56.62784248	31.78036606
	65.22658143	104.4903851	94.26199928	96.59225653	96.57163479	56.64793525	6.784554313	51.47187709	50.04897665	81.06408207	89.64272826	108.7797082	83.37371759	93.60210342	95.91173893	38.19147291	40.15053874	61.92710213	6.784554313	4.144970872	63.57684178	73.80522761	67.53621694	74.7950714	75.12501933	45.86276228	38.50079909	47.40939321	3.155127082	61.04036707	24.27179461
AMP1CPR1STR1ERY3DOX2	AMP	CPR	STR	ERY	DOX	AMP+CPR	AMP+STR	AMP+ERY	AMP+DOX	CPR+STR	CPR+ERY	CPR+DOX	STR+ERY	STR+DOX	ERY+DOX	AMP+CPR+STR	AMP+CPR+ERY	AMP+CPR+DOX	AMP+STR+ERY	AMP+STR+DOX	AMP+ERY+DOX	CPR+STR+ERY	CPR+STR+DOX	CPR+ERY+DOX	STR+ERY+DOX	AMP+CPR+STR+ERY	AMP+CPR+STR+DOX	AMP+CPR+ERY+DOX	AMP+STR+ERY+DOX	CPR+STR+ERY+DOX	AMP+CPR+STR+ERY+DOX
	58.58840475	91.42960029	92.00576161	93.44616493	95.46272956	61.46921138	21.33597407	49.62189413	42.74396831	75.87324451	94.31040691	80.19445445	88.83687432	98.91969752	97.4792942	27.22362261	54.23118473	47.35325891	13.84587685	19.98559597	82.49909975	64.35001801	52.25063018	94.59848758	100.0720202	36.73028448	34.06553835	60.31688873	9.290601368	64.06193734	22.86640259
	79.70049917	99.22351636	85.0249584	94.49066371	98.0403032	53.96561287	3.679053429	55.74043261	51.59918654	90.64522093	86.20817157	87.68718802	85.0249584	92.71584396	108.0976151	22.51802551	49.52856351	52.19079312	15.21538177	20.87261971	66.98095766	73.48863006	50.71177667	88.87040118	84.43335182	33.16694398	43.90830098	64.31872805	12.25734886	69.34738399	49.23276021
	65.22658143	104.4903851	94.26199928	96.59225653	99.54116616	56.64793525	6.784554313	51.47187709	59.28751869	81.06408207	89.64272826	110.4294479	83.37371759	95.91173893	103.8304893	38.19147291	40.15053874	53.34845595	6.784554313	26.58143012	64.56668557	73.80522761	46.52265814	86.34324896	84.69350931	45.86276228	40.8104346	57.65840078	12.10496469	57.74088777	36.80981595
AMP1CPR1STR1ERY3DOX3	AMP	CPR	STR	ERY	DOX	AMP+CPR	AMP+STR	AMP+ERY	AMP+DOX	CPR+STR	CPR+ERY	CPR+DOX	STR+ERY	STR+DOX	ERY+DOX	AMP+CPR+STR	AMP+CPR+ERY	AMP+CPR+DOX	AMP+STR+ERY	AMP+STR+DOX	AMP+ERY+DOX	CPR+STR+ERY	CPR+STR+DOX	CPR+ERY+DOX	STR+ERY+DOX	AMP+CPR+STR+ERY	AMP+CPR+STR+DOX	AMP+CPR+ERY+DOX	AMP+STR+ERY+DOX	CPR+STR+ERY+DOX	AMP+CPR+STR+ERY+DOX
	58.58840475	91.42960029	92.00576161	93.44616493	101.2243428	61.46921138	21.33597407	49.62189413	45.62477494	75.87324451	94.31040691	74.14476053	88.83687432	81.63485776	102.9528268	27.22362261	54.23118473	70.68779258	13.84587685	9.326611451	82.78718041	64.35001801	44.18437162	97.76737486	91.14151963	36.73028448	16.52862802	73.85667987	17.10478934	58.84047533	20.84983795
	79.70049917	99.22351636	85.0249584	94.49066371	101.2941394	53.96561287	3.679053429	55.74043261	56.92364578	90.64522093	86.20817157	90.64522093	85.0249584	99.51931965	108.985025	22.51802551	49.52856351	72.00961361	15.21538177	12.59012757	60.76908856	73.48863006	71.71381032	90.34941764	96.56128674	33.16694398	2.107598447	65.7977445	22.35163616	56.62784248	38.58384175
	65.22658143	104.4903851	94.26199928	96.59225653	101.8508017	56.64793525	6.784554313	51.47187709	58.62762283	81.06408207	89.64272826	93.60210342	83.37371759	104.1604372	113.3989792	38.19147291	40.15053874	79.41434242	6.784554313	2.495231221	60.27736248	73.80522761	71.82554003	83.37371759	90.96251998	45.86276228	24.3130381	69.18595659	13.71346084	64.00989844	31.26256638
AMP1CPR1STR2ERY1DOX1	AMP	CPR	STR	ERY	DOX	AMP+CPR	AMP+STR	AMP+ERY	AMP+DOX	CPR+STR	CPR+ERY	CPR+DOX	STR+ERY	STR+DOX	ERY+DOX	AMP+CPR+STR	AMP+CPR+ERY	AMP+CPR+DOX	AMP+STR+ERY	AMP+STR+DOX	AMP+ERY+DOX	CPR+STR+ERY	CPR+STR+DOX	CPR+ERY+DOX	STR+ERY+DOX	AMP+CPR+STR+ERY	AMP+CPR+STR+DOX	AMP+CPR+ERY+DOX	AMP+STR+ERY+DOX	CPR+STR+ERY+DOX	AMP+CPR+STR+ERY+DOX
	58.58840475	91.42960029	82.42586353	83.6514224	101.2243428	61.46921138	-0.216060497	50.5221462	44.76053295	75.00900252	86.82030969	100.3601008	73.20799362	85.71795993	95.46272956	16.24054735	57.4360821	55.95966871	11.63125675	-2.574720922	47.06517825	80.77061577	50.81022686	76.7374865	90.32689488	28.33993518	28.88008642	55.1314368	9.902772776	68.67122794	12.40547353
	79.70049917	99.22351636	81.02667338	82.95433537	100.9983361	53.96561287	7.413569976	68.45997412	46.9033093	83.25013866	84.72915511	100.4067295	67.11625566	73.88022144	95.67387687	11.85061934	56.92364578	62.5439083	16.10279164	1.645405805	53.66980958	85.32076169	51.89498983	88.57459789	79.03371917	13.64392679	0.314290996	56.33203919	19.09779996	74.37603993	1.016823812
	65.22658143	104.4903851	87.03696851	90.30262412	96.57163479	56.64793525	9.094189823	54.9981956	50.04897665	76.77475898	94.59194721	108.7797082	65.2283857	75.96491877	93.27215549	10.78517296	54.66824767	61.92710213	37.13976388	4.804866732	49.38908079	85.02345724	74.46512347	75.12501933	82.91208723	15.73439192	23.65314224	31.24194463	6.124658452	80.73413414	9.754085683
AMP1CPR1STR2ERY1DOX2	AMP	CPR	STR	ERY	DOX	AMP+CPR	AMP+STR	AMP+ERY	AMP+DOX	CPR+STR	CPR+ERY	CPR+DOX	STR+ERY	STR+DOX	ERY+DOX	AMP+CPR+STR	AMP+CPR+ERY	AMP+CPR+DOX	AMP+STR+ERY	AMP+STR+DOX	AMP+ERY+DOX	CPR+STR+ERY	CPR+STR+DOX	CPR+ERY+DOX	STR+ERY+DOX	AMP+CPR+STR+ERY	AMP+CPR+STR+DOX	AMP+CPR+ERY+DOX	AMP+STR+ERY+DOX	CPR+STR+ERY+DOX	AMP+CPR+STR+ERY+DOX
	58.58840475	91.42960029	82.42586353	83.6514224	95.46272956	61.46921138	-0.216060497	50.5221462	42.74396831	75.00900252	86.82030969	80.19445445	73.20799362	90.32689488	99.20777818	16.24054735	57.4360821	47.35325891	11.63125675	19.6975153	45.62477494	80.77061577	56.28375945	81.92293842	82.42586353	28.33993518	21.71407994	41.87972632	4.105149442	48.2175009	30.64458048
	79.70049917	99.22351636	81.02667338	82.95433537	98.0403032	53.96561287	7.413569976	68.45997412	51.59918654	83.25013866	84.72915511	87.68718802	67.11625566	77.42325113	103.956369	11.85061934	56.92364578	52.19079312	16.10279164	11.55481605	51.63616195	85.32076169	62.24810501	90.64522093	89.01862104	13.64392679	24.97689037	45.38731743	16.43557035	66.38935108	14.91957848
	65.22658143	104.4903851	87.03696851	90.30262412	99.54116616	56.64793525	9.094189823	54.9981956	59.28751869	76.77475898	94.59194721	110.4294479	65.2283857	74.70179723	102.5106975	10.78517296	54.66824767	53.34845595	37.13976388	4.804866732	44.1099139	85.02345724	57.74088777	63.57684178	89.22769493	15.73439192	34.50018044	29.92215291	25.2616384	63.57684178	13.09480848
AMP1CPR1STR2ERY1DOX3	AMP	CPR	STR	ERY	DOX	AMP+CPR	AMP+STR	AMP+ERY	AMP+DOX	CPR+STR	CPR+ERY	CPR+DOX	STR+ERY	STR+DOX	ERY+DOX	AMP+CPR+STR	AMP+CPR+ERY	AMP+CPR+DOX	AMP+STR+ERY	AMP+STR+DOX	AMP+ERY+DOX	CPR+STR+ERY	CPR+STR+DOX	CPR+ERY+DOX	STR+ERY+DOX	AMP+CPR+STR+ERY	AMP+CPR+STR+DOX	AMP+CPR+ERY+DOX	AMP+STR+ERY+DOX	CPR+STR+ERY+DOX	AMP+CPR+STR+ERY+DOX
	58.58840475	91.42960029	82.42586353	83.6514224	101.2243428	61.46921138	-0.216060497	50.5221462	45.62477494	75.00900252	86.82030969	74.14476053	73.20799362	94.27741056	98.63161685	16.24054735	57.4360821	70.68779258	11.63125675	6.445804825	43.03204897	80.77061577	62.62153403	61.18113072	61.35644659	28.33993518	27.18761253	39.28700036	3.817068779	68.09506662	21.71407994
	79.70049917	99.22351636	81.02667338	82.95433537	101.2941394	53.96561287	7.413569976	68.45997412	56.92364578	83.25013866	84.72915511	90.64522093	67.11625566	77.74534474	101.885746	11.85061934	56.92364578	72.00961361	16.10279164	21.76002958	41.54187465	85.32076169	59.58587539	81.17951562	100.9360845	13.64392679	36.51321871	48.64115363	14.06914402	77.92567942	13.44056203
	65.22658143	104.4903851	87.03696851	90.30262412	101.8508017	56.64793525	9.094189823	54.9981956	58.62762283	76.77475898	94.59194721	93.60210342	65.2283857	77.85960108	102.1807496	10.78517296	54.66824767	79.41434242	37.13976388	4.804866732	52.35861216	85.02345724	73.47527968	73.80522761	82.28052647	15.73439192	28.93230912	26.29272568	13.38351291	65.55652936	12.43491262
AMP1CPR1STR2ERY2DOX1	AMP	CPR	STR	ERY	DOX	AMP+CPR	AMP+STR	AMP+ERY	AMP+DOX	CPR+STR	CPR+ERY	CPR+DOX	STR+ERY	STR+DOX	ERY+DOX	AMP+CPR+STR	AMP+CPR+ERY	AMP+CPR+DOX	AMP+STR+ERY	AMP+STR+DOX	AMP+ERY+DOX	CPR+STR+ERY	CPR+STR+DOX	CPR+ERY+DOX	STR+ERY+DOX	AMP+CPR+STR+ERY	AMP+CPR+STR+DOX	AMP+CPR+ERY+DOX	AMP+STR+ERY+DOX	CPR+STR+ERY+DOX	AMP+CPR+STR+ERY+DOX
	58.58840475	91.42960029	82.42586353	96.90313288	101.2243428	61.46921138	-0.216060497	45.01260353	44.76053295	75.00900252	88.54879366	100.3601008	85.65623312	85.71795993	99.7839395	16.24054735	46.16492618	55.95966871	5.203456968	-2.574720922	57.14800144	81.92293842	50.81022686	60.60496939	68.59905867	35.25387108	28.88008642	61.46921138	12.74756932	58.87648542	28.33993518
	79.70049917	99.22351636	81.02667338	96.56128674	100.9983361	53.96561287	7.413569976	64.31872805	46.9033093	83.25013866	93.30745055	100.4067295	76.83945647	73.88022144	106.0269921	11.85061934	53.96561287	62.5439083	5.158069884	1.645405805	61.95230172	89.75781106	51.89498983	87.98299131	82.89884248	18.37677944	0.314290996	68.16417083	17.9145868	69.34738399	19.06082455
	65.22658143	104.4903851	87.03696851	96.90158272	96.57163479	56.64793525	9.094189823	41.90338712	50.04897665	76.77475898	96.90158272	108.7797082	82.93182351	75.96491877	99.54116616	10.78517296	57.96772697	61.92710213	14.70330463	4.804866732	45.42970562	88.9828324	74.46512347	80.73413414	96.80642416	19.13697995	23.65314224	57.32845285	46.52265814	52.02866423	23.34381605
AMP1CPR1STR2ERY2DOX2	AMP	CPR	STR	ERY	DOX	AMP+CPR	AMP+STR	AMP+ERY	AMP+DOX	CPR+STR	CPR+ERY	CPR+DOX	STR+ERY	STR+DOX	ERY+DOX	AMP+CPR+STR	AMP+CPR+ERY	AMP+CPR+DOX	AMP+STR+ERY	AMP+STR+DOX	AMP+ERY+DOX	CPR+STR+ERY	CPR+STR+DOX	CPR+ERY+DOX	STR+ERY+DOX	AMP+CPR+STR+ERY	AMP+CPR+STR+DOX	AMP+CPR+ERY+DOX	AMP+STR+ERY+DOX	CPR+STR+ERY+DOX	AMP+CPR+STR+ERY+DOX
	58.58840475	91.42960029	82.42586353	96.90313288	95.46272956	61.46921138	-0.216060497	45.01260353	42.74396831	75.00900252	88.54879366	80.19445445	85.65623312	90.32689488	99.20777818	16.24054735	46.16492618	47.35325891	5.203456968	19.6975153	52.25063018	81.92293842	56.28375945	69.24738927	63.66091407	35.25387108	21.71407994	50.23406554	15.37630537	64.63809867	23.73064458
	79.70049917	99.22351636	81.02667338	96.56128674	98.0403032	53.96561287	7.413569976	64.31872805	51.59918654	83.25013866	93.30745055	87.68718802	76.83945647	77.42325113	106.6185986	11.85061934	53.96561287	52.19079312	5.158069884	11.55481605	58.69846552	89.75781106	62.24810501	84.43335182	81.28837443	18.37677944	24.97689037	77.03826955	14.36494731	70.23479386	30.89295618
	65.22658143	104.4903851	87.03696851	96.90158272	99.54116616	56.64793525	9.094189823	41.90338712	59.28751869	76.77475898	96.90158272	110.4294479	82.93182351	74.70179723	103.1705934	10.78517296	57.96772697	53.34845595	14.70330463	4.804866732	45.75965355	88.9828324	57.74088777	57.63777904	94.59596146	19.13697995	34.50018044	60.95788008	13.38351291	55.32814353	20.04433675
AMP1CPR1STR2ERY2DOX3	AMP	CPR	STR	ERY	DOX	AMP+CPR	AMP+STR	AMP+ERY	AMP+DOX	CPR+STR	CPR+ERY	CPR+DOX	STR+ERY	STR+DOX	ERY+DOX	AMP+CPR+STR	AMP+CPR+ERY	AMP+CPR+DOX	AMP+STR+ERY	AMP+STR+DOX	AMP+ERY+DOX	CPR+STR+ERY	CPR+STR+DOX	CPR+ERY+DOX	STR+ERY+DOX	AMP+CPR+STR+ERY	AMP+CPR+STR+DOX	AMP+CPR+ERY+DOX	AMP+STR+ERY+DOX	CPR+STR+ERY+DOX	AMP+CPR+STR+ERY+DOX
	58.58840475	91.42960029	82.42586353	96.90313288	101.2243428	61.46921138	-0.216060497	45.01260353	45.62477494	75.00900252	88.54879366	74.14476053	85.65623312	94.27741056	102.0885848	16.24054735	46.16492618	70.68779258	5.203456968	6.445804825	61.75729204	81.92293842	62.62153403	71.55203457	75.77994393	35.25387108	27.18761253	40.43932301	22.29024127	69.82355059	22.0021606
	79.70049917	99.22351636	81.02667338	96.56128674	101.2941394	53.96561287	7.413569976	64.31872805	56.92364578	83.25013866	93.30745055	90.64522093	76.83945647	77.74534474	110.7598447	11.85061934	53.96561287	72.00961361	5.158069884	21.76002958	51.89498983	89.75781106	59.58587539	88.87040118	80.96628083	18.37677944	36.51321871	62.5439083	20.53984101	74.67184322	27.34331669
	65.22658143	104.4903851	87.03696851	96.90158272	101.8508017	56.64793525	9.094189823	41.90338712	58.62762283	76.77475898	96.90158272	93.60210342	82.93182351	77.85960108	95.91173893	10.78517296	57.96772697	79.41434242	14.70330463	4.804866732	61.92710213	88.9828324	73.47527968	50.72949425	78.49116185	19.13697995	28.93230912	36.87168119	14.74454813	58.2976749	22.95200289
AMP1CPR1STR2ERY3DOX1	AMP	CPR	STR	ERY	DOX	AMP+CPR	AMP+STR	AMP+ERY	AMP+DOX	CPR+STR	CPR+ERY	CPR+DOX	STR+ERY	STR+DOX	ERY+DOX	AMP+CPR+STR	AMP+CPR+ERY	AMP+CPR+DOX	AMP+STR+ERY	AMP+STR+DOX	AMP+ERY+DOX	CPR+STR+ERY	CPR+STR+DOX	CPR+ERY+DOX	STR+ERY+DOX	AMP+CPR+STR+ERY	AMP+CPR+STR+DOX	AMP+CPR+ERY+DOX	AMP+STR+ERY+DOX	CPR+STR+ERY+DOX	AMP+CPR+STR+ERY+DOX
	58.58840475	91.42960029	82.42586353	93.44616493	101.2243428	61.46921138	-0.216060497	49.62189413	44.76053295	75.00900252	94.31040691	100.3601008	74.52483218	85.71795993	97.4792942	16.24054735	54.23118473	55.95966871	1.548433561	-2.574720922	79.90637378	70.97587324	50.81022686	75.00900252	66.89128366	36.37018365	28.88008642	51.09830753	34.96579042	69.82355059	18.54519265
	79.70049917	99.22351636	81.02667338	94.49066371	100.9983361	53.96561287	7.413569976	55.74043261	46.9033093	83.25013866	86.20817157	100.4067295	83.28132864	73.88022144	107.8018118	11.85061934	49.52856351	62.5439083	3.716028841	1.645405805	64.61453134	73.78443335	51.89498983	96.26548345	86.76396578	24.88445184	0.314290996	68.16417083	19.35662784	93.30745055	29.70974302
	65.22658143	104.4903851	87.03696851	96.59225653	96.57163479	56.64793525	9.094189823	51.47187709	50.04897665	76.77475898	89.64272826	108.7797082	84.51072543	75.96491877	95.91173893	10.78517296	40.15053874	61.92710213	11.40382533	4.804866732	63.57684178	76.11486312	74.46512347	74.7950714	76.91225993	12.86796927	23.65314224	47.40939321	9.424137753	70.83569624	27.24132598
AMP1CPR1STR2ERY3DOX2	AMP	CPR	STR	ERY	DOX	AMP+CPR	AMP+STR	AMP+ERY	AMP+DOX	CPR+STR	CPR+ERY	CPR+DOX	STR+ERY	STR+DOX	ERY+DOX	AMP+CPR+STR	AMP+CPR+ERY	AMP+CPR+DOX	AMP+STR+ERY	AMP+STR+DOX	AMP+ERY+DOX	CPR+STR+ERY	CPR+STR+DOX	CPR+ERY+DOX	STR+ERY+DOX	AMP+CPR+STR+ERY	AMP+CPR+STR+DOX	AMP+CPR+ERY+DOX	AMP+STR+ERY+DOX	CPR+STR+ERY+DOX	AMP+CPR+STR+ERY+DOX
	58.58840475	91.42960029	82.42586353	93.44616493	95.46272956	61.46921138	-0.216060497	49.62189413	42.74396831	75.00900252	94.31040691	80.19445445	74.52483218	90.32689488	97.4792942	16.24054735	54.23118473	47.35325891	1.548433561	19.6975153	82.49909975	70.97587324	56.28375945	94.59848758	83.022556	36.37018365	21.71407994	60.31688873	37.84659705	76.44940583	13.89989197
	79.70049917	99.22351636	81.02667338	94.49066371	98.0403032	53.96561287	7.413569976	55.74043261	51.59918654	83.25013866	86.20817157	87.68718802	83.28132864	77.42325113	108.0976151	11.85061934	49.52856351	52.19079312	3.716028841	11.55481605	66.98095766	73.78443335	62.24810501	88.87040118	86.76396578	24.88445184	24.97689037	64.31872805	31.78036606	87.39138473	27.0475134
	65.22658143	104.4903851	87.03696851	96.59225653	99.54116616	56.64793525	9.094189823	51.47187709	59.28751869	76.77475898	89.64272826	110.4294479	84.51072543	74.70179723	103.8304893	10.78517296	40.15053874	53.34845595	11.40382533	4.804866732	64.56668557	76.11486312	57.74088777	86.34324896	73.45841197	12.86796927	34.50018044	57.65840078	20.02371501	80.73413414	37.13976388
AMP1CPR1STR2ERY3DOX3	AMP	CPR	STR	ERY	DOX	AMP+CPR	AMP+STR	AMP+ERY	AMP+DOX	CPR+STR	CPR+ERY	CPR+DOX	STR+ERY	STR+DOX	ERY+DOX	AMP+CPR+STR	AMP+CPR+ERY	AMP+CPR+DOX	AMP+STR+ERY	AMP+STR+DOX	AMP+ERY+DOX	CPR+STR+ERY	CPR+STR+DOX	CPR+ERY+DOX	STR+ERY+DOX	AMP+CPR+STR+ERY	AMP+CPR+STR+DOX	AMP+CPR+ERY+DOX	AMP+STR+ERY+DOX	CPR+STR+ERY+DOX	AMP+CPR+STR+ERY+DOX

	58.58840475	91.42960029	82.42586353	93.44616493	101.2243428	61.46921138	-0.216060497	49.62189413	45.62477494	75.00900252	94.31040691
	79.70049917	99.22351636	81.02667338	94.49066371	101.2941394	53.96561287	7.413569976	55.74043261	56.92364578	83.25013866	86.20817157
	65.22658143	104.4903851	87.03696851	96.59225653	101.8508017	56.64793525	9.094189823	51.47187709	58.62762283	76.77475898	89.64272826
AMP1CPR1STR3ERY1DOX1	AMP	CPR	STR	ERY	DOX	AMP+CPR	AMP+STR	AMP+ERY	AMP+DOX	CPR+STR	CPR+ERY
	58.58840475	91.42960029	94.30051813	83.6514224	101.2243428	61.46921138	14.42203817	50.5221462	44.76053295	76.44940583	86.82030969
	79.70049917	99.22351636	98.24046921	82.95433537	100.9983361	53.96561287	16.10279164	68.45997412	46.9033093	78.8130893	84.72915511
	65.22658143	104.4903851	92.94362467	90.30262412	96.57163479	56.64793525	12.72361705	54.9981956	50.04897665	86.67319689	94.59194721
AMP1CPR1STR3ERY1DOX2	AMP	CPR	STR	ERY	DOX	AMP+CPR	AMP+STR	AMP+ERY	AMP+DOX	CPR+STR	CPR+ERY
	58.58840475	91.42960029	94.30051813	83.6514224	95.46272956	61.46921138	14.42203817	50.5221462	42.74396831	76.44940583	86.82030969
	79.70049917	99.22351636	98.24046921	82.95433537	98.0403032	53.96561287	16.10279164	68.45997412	51.59918654	78.8130893	84.72915511
	65.22658143	104.4903851	92.94362467	90.30262412	99.54116616	56.64793525	12.72361705	54.9981956	59.28751869	86.67319689	94.59194721
AMP1CPR1STR3ERY1DOX3	AMP	CPR	STR	ERY	DOX	AMP+CPR	AMP+STR	AMP+ERY	AMP+DOX	CPR+STR	CPR+ERY
	58.58840475	91.42960029	94.30051813	83.6514224	101.2243428	61.46921138	14.42203817	50.5221462	45.62477494	76.44940583	86.82030969
	79.70049917	99.22351636	98.24046921	82.95433537	101.2941394	53.96561287	16.10279164	68.45997412	56.92364578	78.8130893	84.72915511
	65.22658143	104.4903851	92.94362467	90.30262412	101.8508017	56.64793525	12.72361705	54.9981956	58.62762283	86.67319689	94.59194721
AMP1CPR1STR3ERY2DOX1	AMP	CPR	STR	ERY	DOX	AMP+CPR	AMP+STR	AMP+ERY	AMP+DOX	CPR+STR	CPR+ERY
	58.58840475	91.42960029	94.30051813	96.90313288	101.2243428	61.46921138	14.42203817	45.01260353	44.76053295	76.44940583	88.54879366
	79.70049917	99.22351636	98.24046921	96.56128674	100.9983361	53.96561287	16.10279164	64.31872805	46.9033093	78.8130893	93.30745055
	65.22658143	104.4903851	92.94362467	96.90158272	96.57163479	56.64793525	12.72361705	41.90338712	50.04897665	86.67319689	96.90158272

[illegible]

	65.22658143	97.78660613	87.03696851	90.30262412	99.54116616	70.83569624	9.094189823	54.9981956	59.28751869	68.65304578	77.8093076	75.00945895	65.2283857	74.70179723	102.5106975	16.72423571	[illegible]	[illegible]	[illegible]	[illegible]
AMP1CPR3STR2ERY1DOX3	AMP	CPR	STR	ERY	DOX	AMP+CPR	AMP+STR	AMP+ERY	AMP+DOX	CPR+STR	CPR+ERY	CPR+DOX	STR+ERY	STR+DOX	ERY+DOX	AMP+CPR+STR	AMP+CPR+ERY	AMP+CPR+DOX	AMP+STR+ERY	AMP+STR+DOX
	58.58840475	96.1427749	82.42586353	83.6514224	101.2243428	56.85992078	-0.216060497	50.5221462	45.62477494	61.77317214	74.95682211	65.76472846	73.20799362	94.27741056	98.63161685	8.138278718	45.62477494	53.69103349	11.63125675	6.445804825
	79.70049917	99.70674487	81.02667338	82.95433537	101.2941394	84.72915511	7.413569976	68.45997412	56.92364578	66.03739003	75.07331378	68.38343109	67.11625566	77.74534474	101.885746	43.02089111	73.19282677	53.70678499	16.10279164	21.76002958
	65.22658143	97.78660613	87.03696851	90.30262412	101.8508017	70.83569624	9.094189823	54.9981956	58.62762283	68.65304578	77.8093076	72.28528188	65.2283857	77.85960108	102.1807496	16.72423571	51.36876837	48.83229365	37.13976388	4.804866732
AMP1CPR3STR2ERY2DOX1	AMP	CPR	STR	ERY	DOX	AMP+CPR	AMP+STR	AMP+ERY	AMP+DOX	CPR+STR	CPR+ERY	CPR+DOX	STR+ERY	STR+DOX	ERY+DOX	AMP+CPR+STR	AMP+CPR+ERY	AMP+CPR+DOX	AMP+STR+ERY	AMP+STR+DOX
	58.58840475	96.1427749	82.42586353	96.90313288	101.2243428	56.85992078	-0.216060497	45.01260353	44.76053295	61.77317214	73.11456534	69.14219919	85.65623312	85.71795993	99.7839395	8.138278718	55.70759813	41.84371624	5.203456968	-2.574720922
	79.70049917	99.70674487	81.02667338	96.56128674	100.9983361	84.72915511	7.413569976	64.31872805	46.9033093	66.03739003	74.48680352	69.26319648	76.83945647	73.88022144	106.0269921	43.02089111	70.53059715	64.02292476	5.158069884	1.645405805
	65.22658143	97.78660613	87.03696851	96.90158272	96.57163479	70.83569624	9.094189823	41.90338712	50.04897665	68.65304578	85.0737798	71.07453651	82.93182351	75.96491877	99.54116616	16.72423571	52.02866423	53.12161674	14.70330463	4.804866732
AMP1CPR3STR2ERY2DOX2	AMP	CPR	STR	ERY	DOX	AMP+CPR	AMP+STR	AMP+ERY	AMP+DOX	CPR+STR	CPR+ERY	CPR+DOX	STR+ERY	STR+DOX	ERY+DOX	AMP+CPR+STR	AMP+CPR+ERY	AMP+CPR+DOX	AMP+STR+ERY	AMP+STR+DOX
	58.58840475	96.1427749	82.42586353	96.90313288	95.46272956	56.85992078	-0.216060497	45.01260353	42.74396831	61.77317214	73.11456534	70.98445596	85.65623312	90.32689488	99.20777818	8.138278718	55.70759813	47.06517825	5.203456968	19.6975153
	79.70049917	99.70674487	81.02667338	96.56128674	98.0403032	84.72915511	7.413569976	64.31872805	51.59918654	66.03739003	74.48680352	70.72947214	76.83945647	77.42325113	106.6185986	43.02089111	70.53059715	52.81937512	5.158069884	11.55481605
	65.22658143	97.78660613	87.03696851	96.90158272	99.54116616	70.83569624	9.094189823	41.90338712	59.28751869	68.65304578	85.0737798	75.00945895	82.93182351	74.70179723	103.1705934	16.72423571	52.02866423	64.6697943	14.70330463	4.804866732
AMP1CPR3STR2ERY2DOX3	AMP	CPR	STR	ERY	DOX	AMP+CPR	AMP+STR	AMP+ERY	AMP+DOX	CPR+STR	CPR+ERY	CPR+DOX	STR+ERY	STR+DOX	ERY+DOX	AMP+CPR+STR	AMP+CPR+ERY	AMP+CPR+DOX	AMP+STR+ERY	AMP+STR+DOX
	58.58840475	96.1427749	82.42586353	96.90313288	101.2243428	56.85992078	-0.216060497	45.01260353	45.62477494	61.77317214	73.11456534	65.76472846	85.65623312	94.27741056	102.0885848	8.138278718	55.70759813	53.69103349	5.203456968	6.445804825
	79.70049917	99.70674487	81.02667338	96.56128674	101.2941394	84.72915511	7.413569976	64.31872805	56.92364578	66.03739003	74.48680352	68.38343109	76.83945647	77.74534474	110.7598447	43.02089111	70.53059715	53.70678499	5.158069884	21.76002958
	65.22658143	97.78660613	87.03696851	96.90158272	101.8508017	70.83569624	9.094189823	41.90338712	58.62762283	68.65304578	85.0737798	72.28528188	82.93182351	77.85960108	95.91173893	16.72423571	52.02866423	48.83229365	14.70330463	4.804866732
AMP1CPR3STR2ERY3DOX1	AMP	CPR	STR	ERY	DOX	AMP+CPR	AMP+STR	AMP+ERY	AMP+DOX	CPR+STR	CPR+ERY	CPR+DOX	STR+ERY	STR+DOX	ERY+DOX	AMP+CPR+STR	AMP+CPR+ERY	AMP+CPR+DOX	AMP+STR+ERY	AMP+STR+DOX
	58.58840475	96.1427749	82.42586353	93.44616493	101.2243428	56.85992078	-0.216060497	49.62189413	44.76053295	61.77317214	72.80752255	69.14219919	74.52483218	85.71795993	97.4792942	8.138278718	60.60496939	41.84371624	1.548433561	-2.574720922
	79.70049917	99.70674487	81.02667338	94.49066371	100.9983361	84.72915511	7.413569976	55.74043261	46.9033093	66.03739003	75.65982405	69.26319648	83.28132864	73.88022144	107.8018118	43.02089111	62.5439083	64.02292476	3.716028841	1.645405805
	65.22658143	97.78660613	87.03696851	96.59225653	96.57163479	70.83569624	9.094189823	51.47187709	50.04897665	68.65304578	81.74423004	71.07453651	84.51072543	75.96491877	95.91173893	16.72423571	44.87291849	53.12161674	11.40382533	4.804866732
AMP1CPR3STR2ERY3DOX2	AMP	CPR	STR	ERY	DOX	AMP+CPR	AMP+STR	AMP+ERY	AMP+DOX	CPR+STR	CPR+ERY	CPR+DOX	STR+ERY	STR+DOX	ERY+DOX	AMP+CPR+STR	AMP+CPR+ERY	AMP+CPR+DOX	AMP+STR+ERY	AMP+STR+DOX
	58.58840475	96.1427749	82.42586353	93.44616493	95.46272956	56.85992078	-0.216060497	49.62189413	42.74396831	61.77317214	72.80752255	70.98445596	74.52483218	90.32689488	97.4792942	8.138278718	60.60496939	47.06517825	1.548433561	19.6975153
	79.70049917	99.70674487	81.02667338	94.49066371	98.0403032	84.72915511	7.413569976	55.74043261	51.59918654	66.03739003	75.65982405	70.72947214	83.28132864	77.42325113	108.0976151	43.02089111	62.5439083	52.81937512	3.716028841	11.55481605
	65.22658143	97.78660613	87.03696851	96.59225653	99.54116616	70.83569624	9.094189823	51.47187709	59.28751869	68.65304578	81.74423004	75.00945895	84.51072543	74.70179723	103.8304893	16.72423571	44.87291849	64.6697943	11.40382533	4.804866732
AMP1CPR3STR2ERY3DOX3	AMP	CPR	STR	ERY	DOX	AMP+CPR	AMP+STR	AMP+ERY	AMP+DOX	CPR+STR	CPR+ERY	CPR+DOX	STR+ERY	STR+DOX	ERY+DOX	AMP+CPR+STR	AMP+CPR+ERY	AMP+CPR+DOX	AMP+STR+ERY	AMP+STR+DOX
	58.58840475	96.1427749	82.42586353	93.44616493	101.2243428	56.85992078	-0.216060497	49.62189413	45.62477494	61.77317214	72.80752255	65.76472846	74.52483218	94.27741056	102.9528268	8.138278718	60.60496939	53.69103349	1.548433561	6.445804825
	79.70049917	99.70674487	81.02667338	94.49066371	101.2941394	84.72915511	7.413569976	55.74043261	56.92364578	66.03739003	75.65982405	68.38343109	83.28132864	77.74534474	108.985025	43.02089111	62.5439083	53.70678499	3.716028841	21.76002958
	65.22658143	97.78660613	87.03696851	96.59225653	101.8508017	70.83569624	9.094189823	51.47187709	58.62762283	68.65304578	81.74423004	72.28528188	84.51072543	77.85960108	113.3989792	16.72423571	44.87291849	48.83229365	11.40382533	4.804866732
AMP1CPR3STR3ERY1DOX1	AMP	CPR	STR	ERY	DOX	AMP+CPR	AMP+STR	AMP+ERY	AMP+DOX	CPR+STR	CPR+ERY	CPR+DOX	STR+ERY	STR+DOX	ERY+DOX	AMP+CPR+STR	AMP+CPR+ERY	AMP+CPR+DOX	AMP+STR+ERY	AMP+STR+DOX
	58.58840475	96.1427749	94.30051813	83.6514224	101.2243428	56.85992078	14.42203817	50.5221462	44.76053295	57.45538284	74.95682211	69.14219919	69.73709461	57.16753022	95.46272956	17.06877926	45.62477494	41.84371624	13.93590205	24.88296723
	79.70049917	99.70674487	98.24046921	82.95433537	100.9983361	84.72915511	16.10279164	68.45997412	46.9033093	60.11730205	75.07331378	69.26319648	65.98240469	62.22507331	95.67387687	26.45590682	73.19282677	64.02292476	20.24403771	19.06082455
	65.22658143	97.78660613	92.94362467	90.30262412	96.57163479	70.83569624	12.72361705	54.9981956	50.04897665	61.99394627	77.8093076	71.07453651	63.58304956	67.82065834	93.27215549	23.98309017	51.36876837	53.12161674	8.104346033	27.94246533
AMP1CPR3STR3ERY1DOX2	AMP	CPR	STR	ERY	DOX	AMP+CPR	AMP+STR	AMP+ERY	AMP+DOX	CPR+STR	CPR+ERY	CPR+DOX	STR+ERY	STR+DOX	ERY+DOX	AMP+CPR+STR	AMP+CPR+ERY	AMP+CPR+DOX	AMP+STR+ERY	AMP+STR+DOX
	58.58840475	96.1427749	94.30051813	83.6514224	95.46272956	56.85992078	14.42203817	50.5221462	42.74396831	57.45538284	74.95682211	70.98445596	69.73709461	62.69430052	99.20777818	17.06877926	45.62477494	47.06517825	13.93590205	24.59488657
	79.70049917	99.70674487	98.24046921	82.95433537	98.0403032	84.72915511	16.10279164	68.45997412	51.59918654	60.11730205	75.07331378	70.72947214	65.98240469	61.87683284	103.956369	26.45590682	73.19282677	52.81937512	20.24403771	26.45590682
	65.22658143	97.78660613	92.94362467	90.30262412	99.54116616	70.83569624	12.72361705	54.9981956	59.28751869	61.99394627	77.8093076	75.00945895	63.58304956	70.54483541	102.5106975	23.98309017	51.36876837	64.6697943	8.104346033	33.22163221

[illegible]

AMP2CPR2STR1ERY2DOX2	AMP	CPR	STR	ERY	DOX	AMP+CPR	AMP+STR	AMP+ERY	AMP+DOX	CPR+STR	CPR+ERY	CPR+DOX	STR+ERY	STR+DOX	ERY+DOX	AMP+CPR+STR	AMP+CPR+ERY	AMP+CPR+DOX	AMP+STR+ERY	AMP+STR+DOX	AMP+ERY+DOX	CPR+STR+ERY	CPR+STR+DOX	CPR+ERY+DOX	STR+ERY+DOX	AMP+CPR+STR+ERY	AMP+CPR+STR+DOX	AMP+CPR+ERY+DOX	AMP+STR+ERY+DOX	CPR+STR+ERY+DOX	AMP+CPR+STR+ERY+DOX
	77.81692858	94.93582984	92.00576161	96.90313288	95.46272956	83.74270209	6.707646408	59.31946195	89.93595844	84.07191173	89.66847561	105.1413287	91.42960029	98.91969752	99.20777818	10.7610401	58.39355984	51.4801574	13.72392685	-1.357989764	64.09300172	82.75507317	59.81327641	65.08063064	74.4328412	28.43548263	19.97891001	58.72276948	20.30811965	87.1376765	34.79334379
	70.5988928	92.29994967	85.0249584	96.56128674	98.0403032	67.76044288	7.146451938	54.81630599	72.00805234	82.57674887	77.10115752	86.76396578	88.87040118	92.71584396	106.6185986	2.757926522	43.9859084	49.01862104	12.62204328	1.469552089	56.48716658	74.84650226	71.24308002	59.70810267	89.75781106	8.817312531	4.126824358	81.28837443	30.3975843	63.57322597	3.663814796
	67.43884839	88.28035377	94.26199928	96.90158272	99.54116616	57.0180957	6.809014543	67.123068	59.56407505	80.70162454	83.543648	86.38567147	91.62241584	95.91173893	103.1705934	15.01930454	62.09031812	68.50460719	6.611651803	3.019649928	51.03800466	75.64913839	62.18899949	69.64931108	72.48543589	13.12462224	3.651210697	88.59613416	3.138067572	42.17641762	16.28242608
AMP2CPR2STR1ERY2DOX3	AMP	CPR	STR	ERY	DOX	AMP+CPR	AMP+STR	AMP+ERY	AMP+DOX	CPR+STR	CPR+ERY	CPR+DOX	STR+ERY	STR+DOX	ERY+DOX	AMP+CPR+STR	AMP+CPR+ERY	AMP+CPR+DOX	AMP+STR+ERY	AMP+STR+DOX	AMP+ERY+DOX	CPR+STR+ERY	CPR+STR+DOX	CPR+ERY+DOX	STR+ERY+DOX	AMP+CPR+STR+ERY	AMP+CPR+STR+DOX	AMP+CPR+ERY+DOX	AMP+STR+ERY+DOX	CPR+STR+ERY+DOX	AMP+CPR+STR+ERY+DOX
	77.81692858	94.93582984	92.00576161	96.90313288	101.2243428	83.74270209	6.707646408	59.31946195	84.33939456	84.07191173	89.66847561	91.97294308	91.42960029	81.63485776	102.0885848	10.7610401	58.39355984	53.55829325	13.72392685	17.67444253	58.39355984	82.75507317	62.01486587	75.12152465	77.31364782	28.43548263	14.27946812	59.05197912	25.90468352	56.85038965	21.85128983
	70.5988928	92.29994967	85.0249584	96.56128674	101.2941394	67.76044288	7.146451938	54.81630599	67.17664821	82.57674887	77.10115752	87.08605939	88.87040118	99.51931965	110.7598447	2.757926522	43.9859084	56.16507297	12.62204328	29.57221943	58.74182184	74.84650226	43.28132864	86.76396578	94.19486042	8.817312531	33.29642677	79.35581278	17.51383996	82.57674887	17.83593357
	67.43884839	88.28035377	94.26199928	96.90158272	101.8508017	57.0180957	6.809014543	67.123068	54.51158889	80.70162454	83.543648	93.33283993	91.62241584	104.1604372	95.91173893	15.01930454	62.09031812	52.39980757	6.611651803	22.59803377	51.66956543	75.64913839	53.03136834	65.54416608	73.47527968	13.12462224	39.65017454	84.49098916	-1.085495072	58.59699762	17.861328
AMP2CPR2STR1ERY3DOX1	AMP	CPR	STR	ERY	DOX	AMP+CPR	AMP+STR	AMP+ERY	AMP+DOX	CPR+STR	CPR+ERY	CPR+DOX	STR+ERY	STR+DOX	ERY+DOX	AMP+CPR+STR	AMP+CPR+ERY	AMP+CPR+DOX	AMP+STR+ERY	AMP+STR+DOX	AMP+ERY+DOX	CPR+STR+ERY	CPR+STR+DOX	CPR+ERY+DOX	STR+ERY+DOX	AMP+CPR+STR+ERY	AMP+CPR+STR+DOX	AMP+CPR+ERY+DOX	AMP+STR+ERY+DOX	CPR+STR+ERY+DOX	AMP+CPR+STR+ERY+DOX
	77.81692858	94.93582984	92.00576161	93.44616493	101.2243428	83.74270209	6.707646408	86.64386204	81.04729817	84.07191173	93.28978164	90.32689488	88.83687432	96.03889089	97.4792942	10.7610401	57.01499447	56.52118001	57.67341375	7.530670507	64.97775263	44.23754533	64.751421	72.81705717	83.36334174	12.96262956	1.543170186	77.48771894	30.7399501	66.29459119	23.92942568
	70.5988928	92.29994967	85.0249584	94.49066371	100.9983361	67.76044288	7.146451938	74.90689482	68.34423754	82.57674887	78.06743835	90.30699547	85.0249584	92.71584396	107.8018118	2.757926522	49.78359336	65.44539507	36.57775541	-5.294413689	54.55460493	43.54302969	66.7337695	92.88374434	95.08227029	7.347760443	2.838449925	83.8651233	-2.838449925	64.53950679	11.33366885
	67.43884839	88.28035377	94.26199928	96.59225653	96.57163479	57.0180957	6.809014543	64.39946219	51.66956543	80.70162454	80.72136082	84.80676954	83.37371759	93.60210342	95.91173893	15.01930454	66.82702389	64.39946219	39.66991082	28.91364147	59.87985543	60.17589954	57.64965647	57.0180957	75.12501933	11.54572031	1.756528389	79.438503	-1.085495072	53.54451147	20.38757108
AMP2CPR2STR1ERY3DOX2	AMP	CPR	STR	ERY	DOX	AMP+CPR	AMP+STR	AMP+ERY	AMP+DOX	CPR+STR	CPR+ERY	CPR+DOX	STR+ERY	STR+DOX	ERY+DOX	AMP+CPR+STR	AMP+CPR+ERY	AMP+CPR+DOX	AMP+STR+ERY	AMP+STR+DOX	AMP+ERY+DOX	CPR+STR+ERY	CPR+STR+DOX	CPR+ERY+DOX	STR+ERY+DOX	AMP+CPR+STR+ERY	AMP+CPR+STR+DOX	AMP+CPR+ERY+DOX	AMP+STR+ERY+DOX	CPR+STR+ERY+DOX	AMP+CPR+STR+ERY+DOX
	77.81692858	94.93582984	92.00576161	93.44616493	95.46272956	83.74270209	6.707646408	86.64386204	89.93595844	84.07191173	93.28978164	105.1413287	88.83687432	98.91969752	97.4792942	10.7610401	57.01499447	51.4801574	57.67341375	-1.357989764	70.5743165	44.23754533	59.81327641	72.81705717	100.0720202	12.96262956	19.97891001	83.74270209	23.2710064	75.18325146	36.33651398
	70.5988928	92.29994967	85.0249584	94.49066371	98.0403032	67.76044288	7.146451938	74.90689482	72.00805234	82.57674887	78.06743835	86.76396578	85.0249584	92.71584396	108.0976151	2.757926522	49.78359336	49.01862104	36.57775541	1.469552089	77.74534474	43.54302969	71.24308002	72.2697534	84.43335182	7.347760443	4.126824358	78.71162557	24.7408153	57.39305486	16.22546553
	67.43884839	88.28035377	94.26199928	96.59225653	99.54116616	57.0180957	6.809014543	64.39946219	59.56407505	80.70162454	80.72136082	86.38567147	83.37371759	95.91173893	103.8304893	15.01930454	66.82702389	68.50460719	39.66991082	3.019649928	60.19563582	60.17589954	62.18899949	73.77419235	84.69350931	11.54572031	3.651210697	83.85942839	2.703869543	62.50477988	36.17659031
AMP2CPR2STR1ERY3DOX3	AMP	CPR	STR	ERY	DOX	AMP+CPR	AMP+STR	AMP+ERY	AMP+DOX	CPR+STR	CPR+ERY	CPR+DOX	STR+ERY	STR+DOX	ERY+DOX	AMP+CPR+STR	AMP+CPR+ERY	AMP+CPR+DOX	AMP+STR+ERY	AMP+STR+DOX	AMP+ERY+DOX	CPR+STR+ERY	CPR+STR+DOX	CPR+ERY+DOX	STR+ERY+DOX	AMP+CPR+STR+ERY	AMP+CPR+STR+DOX	AMP+CPR+ERY+DOX	AMP+STR+ERY+DOX	CPR+STR+ERY+DOX	AMP+CPR+STR+ERY+DOX
	77.81692858	94.93582984	92.00576161	93.44616493	101.2243428	83.74270209	6.707646408	86.64386204	84.33939456	84.07191173	93.28978164	91.97294308	88.83687432	81.63485776	102.9528268	10.7610401	57.01499447	53.55829325	57.67341375	17.67444253	65.90365474	44.23754533	62.01486587	89.6067488	91.14151963	12.96262956	14.27946812	73.53720326	22.18049947	59.05197912	34.13492451
	70.5988928	92.29994967	85.0249584	94.49066371	101.2941394	67.76044288	7.146451938	74.90689482	67.17664821	82.57674887	78.06743835	87.08605939	85.0249584	99.51931965	108.985025	2.757926522	49.78359336	56.16507297	36.57775541	29.57221943	74.84650226	43.54302969	43.28132864	79.09411173	96.56128674	7.347760443	33.29642677	73.23603422	22.80825365	62.92903875	24.09662808
	67.43884839	88.28035377	94.26199928	96.59225653	101.8508017	57.0180957	6.809014543	64.39946219	54.51158889	80.70162454	80.72136082	93.33283993	83.37371759	104.1604372	113.3989792	15.01930454	66.82702389	52.39980757	39.66991082	22.59803377	75.03731389	60.17589954	53.03136834	86.08962735	90.96251998	11.54572031	39.65017454	76.59647954	4.93406851	50.38670762	16.08506334
AMP2CPR2STR2ERY1DOX1	AMP	CPR	STR	ERY	DOX	AMP+CPR	AMP+STR	AMP+ERY	AMP+DOX	CPR+STR	CPR+ERY	CPR+DOX	STR+ERY	STR+DOX	ERY+DOX	AMP+CPR+STR	AMP+CPR+ERY	AMP+CPR+DOX	AMP+STR+ERY	AMP+STR+DOX	AMP+ERY+DOX	CPR+STR+ERY	CPR+STR+DOX	CPR+ERY+DOX	STR+ERY+DOX	AMP+CPR+STR+ERY	AMP+CPR+STR+DOX	AMP+CPR+ERY+DOX	AMP+STR+ERY+DOX	CPR+STR+ERY+DOX	AMP+CPR+STR+ERY+DOX
	77.81692858	94.93582984	82.42586353	83.6514224	101.2243428	83.74270209	7.63354852	67.94063939	81.04729817	82.75507317	86.70558885	90.32689488	73.20799362	85.71795993	95.46272956	9.341323526	75.84167074	56.52118001	24.48496695	24.32036213	68.20812222	85.05954065	61.35644659	71.89115506	90.32689488	25.14338623	20.20524163	60.36881767	24.81417659	71.89115506	14.9378874
	70.5988928	92.29994967	81.02667338	82.95433537	100.9983361	67.76044288	6.240563664	51.65576246	68.34423754	75.49068948	75.81278309	90.30699547	67.11625566	73.88022144	95.67387687	8.112732763	44.24760946	65.44539507	22.02315048	6.059386009	52.68243583	78.38953196	62.8686462	104.4791142	79.03371917	15.2591847	15.90337192	57.77554102	24.27780574	56.80926019	10.89079014
	67.43884839	88.28035377	87.03696851	90.30262412	96.57163479	57.0180957	5.861673389	47.86046454	51.66956543	81.96474608	72.80711493	84.80676954	65.2283857	75.96491877	93.27215549	10.40101642	51.01826839	64.39946219	14.38774377	0.177626466	53.34714873	75.01757762	65.2283857	88.28035377	82.91208723	12.80884185	29.54520224	38.50547065	22.91381416	74.70179723	12.92725949
AMP2CPR2STR2ERY1DOX2	AMP	CPR	STR	ERY	DOX	AMP+CPR	AMP+STR	AMP+ERY	AMP+DOX	CPR+STR	CPR+ERY	CPR+DOX	STR+ERY	STR+DOX	ERY+DOX	AMP+CPR+STR	AMP+CPR+ERY	AMP+CPR+DOX	AMP+STR+ERY	AMP+STR+DOX	AMP+ERY+DOX	CPR+STR+ERY	CPR+STR+DOX	CPR+ERY+DOX	STR+ERY+DOX	AMP+CPR+STR+ERY	AMP+CPR+STR+DOX	AMP+CPR+ERY+DOX	AMP+STR+ERY+DOX	CPR+STR+ERY+DOX	AMP+CPR+STR+ERY+DOX
	77.81692858	94.93582984	82.42586353	83.6514224	95.46272956	83.74270209	7.63354852	67.94063939	89.93595844	82.75507317	86.70558885	105.1413287	73.20799362	90.32689488	99.20777818	9.341323526	75.84167074	51.4801574	24.48496695	13.39471721	56.02736555	85.05954065	55.75988272	63.99012371	82.42586353	25.14338623	35.01967542	75.5124611	19.97891001	65.30696227	19.876032
	70.5988928	92.29994967	81.02667338	82.95433537	98.0403032	67.76044288	6.240563664	51.65576246	72.00805234	75.49068948	75.81278309	86.76396578	67.11625566	77.42325113	103.956369	8.112732763	44.24760946	49.01862104	22.02315048	22.60694514	38.51031706	78.38953196	71.24308002	91.91746351	89.01862104	15.2591847	15.84297937	73.55812783	37.48364368	64.21741319	16.48716658
	67.43884839	88.28035377	87.03696851	90.30262412	99.54116616	57.0180957	5.861673389	47.86046454	59.56407505	81.96474608	72.80711493	86.38567147	65.2283857	74.70179723	102.5106975	10.40101642	51.01826839	68.50460719	14.38774377	10.59837916	43.45927543	75.01757762	71.85977377	96.17486339	89.22769493	12.80884185	14.19038103	50.07092723	31.43988454	68.38618954	29.86098262
AMP2CPR2STR2ERY1DOX3	AMP	CPR	STR	ERY	DOX	AMP+CPR	AMP+STR	AMP+ERY	AMP+DOX	CPR+STR	CPR+ERY	CPR+DOX	STR+ERY	STR+DOX	ERY+DOX	AMP+CPR+STR	AMP+CPR+ERY	AMP+CPR+DOX	AMP+STR+ERY	AMP+STR+DOX	AMP+ERY+DOX	CPR+STR+ERY	CPR+STR+DOX	CPR+ERY+DOX	STR+ERY+DOX	AMP+CPR+STR+ERY	AMP+CPR+STR+DOX	AMP+CPR+ERY+DOX	AMP+STR+ERY+DOX	CPR+STR+ERY+DOX	AMP+CPR+STR+ERY+DOX
	77.81692858	94.93582984	82.42586353	83.6514224	101.2243428	83.74270209	7.63354852	67.94063939	84.33939456	82.75507317	86.70558885	91.97294308	73.20799362	94.27741056	98.63161685	9.341323526	75.84167074	53.55829325	24.48496695	24.15575731	58.00262339	85.05954065	43.24991641	60.63630051	61.35644659	25.14338623	15.92551632	60.69802731	26.23389316	67.61142975	17.01602325
	70.5988928	92.29994967	81.02667338	82.95433537	101.2941394	67.76044288	6.240563664	51.65576246	67.17664821	75.49068948	75.81278309	87.08605939	67.11625566	77.74534474	101.885746	8.112732763	44.24760946	56.16507297	22.02315048	8.173125315	42.95923503	78.38953196	58.74182184	79.67790639	100.9360845	15.2591847	30.3975843	66.79416205	28.78711626	84.50931052	20.73477604
	67.43884839	88.28035377	87.03696851	90.30262412	101.8508017	57.0180957	5.861673389	47.86046454	54.51158889	81.96474608	72.80711493	93.33283993	65.2283857	77.85960108	102.1807496	10.40101642	51.01826839	52.39980757	14.38774377	22.59803377	48.1959812	75.01757762	77.5438207	93.33283993	82.28052647	12.80884185	26.38739839	56.07075454	10.59837916	61.1232407	16.28242608
AMP2CPR2STR2ERY2DOX1	AMP	CPR	STR	ERY	DOX	AMP+CPR	AMP+STR	AMP+ERY	AMP+DOX	CPR+STR	CPR+ERY	CPR+DOX	STR+ERY	STR+DOX	ERY+DOX	AMP+CPR+STR	AMP+CPR+ERY	AMP+CPR+DOX	AMP+STR+ERY	AMP+STR+DOX	AMP+ERY+DOX	CPR+STR+ERY	CPR+STR+DOX	CPR+ERY+DOX	STR+ERY+DOX	AMP+CPR+STR+ERY	AMP+CPR+STR+DOX	AMP+CPR+ERY+DOX	AMP+STR+ERY+DOX	CPR+STR+ERY+DOX	AMP+CPR+STR+ERY+DOX
	77.81692858	94.93582984	82.42586353	96.90313288	101.2243428	83.74270209	7.63354852	59.31946195	81.04729817	82.75507317	89.66847561	90.32689488	85.65623312	85.71795993	99.7839395	9.341323526	58.39355984	56.52118001	16.35760397	24.32036213	72.32324271	78.8045575	61.35644659	53.55829325	68.59905867	23.49733803	20.20524163	71.89115506	14.05313649	66.29459119	30.7399501
	70.5988928	92.29994967	81.02667338	96.56128674	100.9983361	67.76044288	6.240563664	54.81630599	68.34423754	75.49068948	77.10115752	90.30699547	76.83945647	73.88022144	106.0269921	8.112732763	43.9859084	65.44539507	15.84297937	6.059386009	56.48716658	68.7267237	62.8686462	72.91394061	82.89884248	10.42778057	15.90337192	81.28837443	25.50578762	95.46049321	36.83945647
	67.43884839	88.28035377	87.03696851	96.90158272	96.57163479	57.0180957	5.861673389	67.123068	51.66956543	81.96474608	83.543648	84.80676954	82.93182351	75.96491877	99.54116616	10.40101642	62.09031812	64.39946219	9.966818389	0.177626466	52.93268697	72.49133454	65.2283857	80.70162454	96.80642416	7.776091971	29.54520224	94.28018108	14.38774377	47.22890377	30.176763
AMP2CPR2STR2ERY2DOX2	AMP	CPR	STR	ERY	DOX	AMP+CPR	AMP+STR	AMP+ERY	AMP+DOX	CPR+STR	CPR+ERY	CPR+DOX	STR+ERY	STR+DOX	ERY+DOX	AMP+CPR+STR	AMP+CPR+ERY	AMP+CPR+DOX	AMP+STR+ERY	AMP+STR+DOX	AMP+ERY+DOX	CPR+STR+ERY	CPR+STR+DOX	CPR+ERY+DOX	STR+ERY+DOX	AMP+CPR+STR+ERY	AMP+CPR+STR+DOX	AMP+CPR+ERY+DOX	AMP+STR+ERY+DOX	CPR+STR+ERY+DOX	AMP+CPR+STR+ERY+DOX
	77.81692858	94.93582984	82.42586353	96.90313288	95.46272956	83.74270209	7.63354852	59.31946195	89.93595844	82.75507317	89.66847561	105.1413287	85.65623312	90.32689488	99.20777818	9.341323526	58.39355984	51.4801574	16.35760397	13.39471721	64.09300172	78.8045575	55.75988272	65.08063064	63.66091407	23.49733803	35.01967542	58.72276948	23.92942568	64.64854299	25.57547388
	70.5988928	92.29994967	81.02667338	96.56128674	98.0403032	67.76044288	6.240563664	54.81630599	72.00805234	75.49068948	77.10115752	86.76396578	76.83945647	77.42325113	106.6185986	8.112732763	43.9859084	49.01862104	15.84297937	22.60694514	56.48716658	68.7267237	71.24308002	59.70810267	81.28837443	10.42778057	15.84297937	81.28837443	27.82083543	97.71514847	9.461499748
	67.43884839	88.28035377	87.03696851	96.90158272	99.54116616	57.0180957	5.861673389	67.123068	59.56407505	81.96474608	83.543648	86.38567147	82.93182351	74.70179723	103.1705934	10.40101642	62.09031812	68.50460719	9.966818389	10.59837916	51.03800466	72.49133454	71.85977377	69.64931108	94.59596146	7.776091971	14.19038103	88.59613416	17.66396526	50.07092723	22.59803377
AMP2CPR2STR2ERY2DOX3	AMP	CPR	STR	ERY	DOX	AMP+CPR	AMP+STR	AMP+ERY	AMP+DOX	CPR+STR	CPR+ERY	CPR+DOX	STR+ERY	STR+DOX	ERY+DOX	AMP+CPR+STR	AMP+CPR+ERY	AMP+CPR+DOX	AMP+STR+ERY	AMP+STR+DOX	AMP+ERY+DOX	CPR+STR+ERY	CPR+STR+DOX	CPR+ERY+DOX	STR+ERY+DOX	AMP+CPR+STR+ERY	AMP+CPR+STR+DOX	AMP+CPR+ERY+DOX	AMP+STR+ERY+DOX	CPR+STR+ERY+DOX	AMP+CPR+STR+ERY+DOX
	77.81692858	94.93582984	82.42586353	96.90313288	101.2243428	83.74270209	7.63354852	59.31946195	84.33939456	82.75507317	89.66847561	91.97294308	85.65623312	94.27741056	102.0885848	9.341323526	58.39355984	53.55829325	16.35760397	24.15575731	58.39355984	78.8045575	43.24991641	75.12152465	75.77994393	23.49733803	15.92551632	59.05197912	29.8551992	53.88750289	27.44785371
	70.5988928	92.29994967	81.02667338	96.56128674	101.2941394	67.76044288	6.240563664	54.81630599	67.17664821	75.49068948	77.10115752	87.08605939	76.83945647	77.74534474	110.7598447	8.112732763	43.9859084	56.16507297	15.84297937	8.173125315	58.74182184	68.7267237	58.74182184	86.76396578	80.96628083	10.42778057	30.3975843	79.35581278	21.70105687	96.42677403	26.532461
	67.43884839	88.28035377	87.03696851	96.90158272	101.8508017	57.0180957	5.861673389	67.123068	54.51158889	81.96474608	83.543648	93.33283993	82.93182351	77.85960108	95.91173893	10.40101642	62.09031812	52.39980757	9.966818389	22.59803377	51.66956543	72.49133454	77.5438207	65.54416608	78.49116185	7.776091971	26.38739839	84.49098916	28.08471796	57.33387608	38.70283339
AMP2CPR2STR2ERY3DOX1	AMP	CPR	STR	ERY	DOX	AMP+CPR	AMP+STR	AMP+ERY	AMP+DOX	CPR+STR	CPR+ERY	CPR+DOX	STR+ERY	STR+DOX	ERY+DOX	AMP+CPR+STR	AMP+CPR+ERY	AMP+CPR+DOX	AMP+STR+ERY	AMP+STR+DOX	AMP+ERY+DOX	CPR+STR+ERY	CPR+STR+DOX	CPR+ERY+DOX	STR+ERY+DOX	AMP+CPR+STR+ERY	AMP+CPR+STR+DOX	AMP+CPR+ERY+DOX	AMP+STR+ERY+DOX	CPR+STR+ERY+DOX	AMP+CPR+STR+ERY+DOX
	77.81692858	94.93582984	82.42586353	93.44616493	101.2243428	83.74270209	7.63354852	86.64386204	81.04729817	82.75507317	93.28978164	90.32689488	74.52483218	85.71795993	97.4792942	9.341323526	57.01499447	56.52118001	23.76482086	24.32036213	64.97775263	87.69321777	61.35644659	72.81705717	66.89128366	15.92551632	20.20524163	77.48771894	27.22152208	67.28222011	35.6780947
	70.5988928	92.29994967	81.02667338	94.49066371	100.9983361	67.76044288	6.240563664	74.90689482	68.34423754	75.49068948	78.06743835	90.30699547	83.28132864	73.88022144	107.8018118	8.112732763	49.78359336	65.44539507	11.77654756	6.059386009	54.55460493	76.45697031	62.8686462	92.88374434	86.76396578	7.790639155	15.90337192	83.8651233	26.21036739	101.9023654	32.33014595
	67.43884839	88.28035377	87.03696851	96.59225653	96.57163479	57.0180957	5.861673389	64.39946219	51.66956543	81.96474608	80.72136082	84.80676954	84.51072543	75.96491877	95.91173893	10.40101642	66.82702389	64.39946219	5.249848894	0.177626466	59.87985543	69.01775031	65.2283857	57.0180957	76.91225993	11.03257719	29.54520224	79.438503	20.82176911	57.33387608	36.17659031
AMP2CPR2STR2ERY3DOX2	AMP	CPR	STR	ERY	DOX	AMP+CPR	AMP+STR	AMP+ERY	AMP+DOX	CPR+STR	CPR+ERY	CPR+DOX	STR+ERY	STR+DOX	ERY+DOX	AMP+CPR+STR	AMP+CPR+ERY	AMP+CPR+DOX	AMP+STR+ERY	AMP+STR+DOX	AMP+ERY+DOX	CPR+STR+ERY	CPR+STR+DOX	CPR+ERY+DOX	STR+ERY+DOX	AMP+CPR+STR+ERY	AMP+CPR+STR+DOX	AMP+CPR+ERY+DOX	AMP+STR+ERY+DOX	CPR+STR+ERY+DOX	AMP+CPR+STR+ERY+DOX
	77.81692858	94.93582984	82.42586353	93.44616493	95.46272956	83.74270209	7.63354852	86.64386204	89.93595844	82.75507317	93.28978164	105.1413287	74.52483218	90.32689488	97.4792942	9.341323526	57.01499447	51.4801574	23.76482086	13.39471721	70.5743165	87.69321777	55.75988272	72.81705717	83.022556	15.92551632	35.01967542	83.74270209	32.3859983	73.53720326	37.65335254
	70.5988928	92.29994967	81.02667338	94.49066371	98.0403032	67.76044288	6.240563664	74.90689482	72.00805234	75.49068948	78.06743835	86.76396578	83.28132864	77.42325113	108.0976151	8.112732763	49.78359336	49.01862104	11.77654756	22.60694514	77.74534474	76.45697031	71.24308002	72.2697534	86.76396578	7.790639155	15.84297937	78.71162557	58.09763463	83.54302969	31.68595873
	67.43884839	88.28035377	87.03696851	96.59225653	99.54116616	57.0180957	5.861673389	64.39946219	59.56407505	81.96474608	80.72136082	86.38567147	84.51072543	74.70179723	103.8304893	10.40101642	66.82702389	68.50460719	5.249848894	10.59837916	60.19563582	69.01775031	71.85977377	73.77419235	73.45841197	11.03257719	14.19038103	83.85942839	16.59820647	51.96560954	33.01878647
AMP2CPR2STR2ERY3DOX3	AMP	CPR	STR	ERY	DOX	AMP+CPR	AMP+STR	AMP+ERY	AMP+DOX	CPR+STR	CPR+ERY	CPR+DOX	STR+ERY	STR+DOX	ERY+DOX	AMP+CPR+STR	AMP+CPR+ERY	AMP+CPR+DOX	AMP+STR+ERY	AMP+STR+DOX	AMP+ERY+DOX	CPR+STR+ERY	CPR+STR+DOX	CPR+ERY+DOX	STR+ERY+DOX	AMP+CPR+STR+ERY	AMP+CPR+STR+DOX	AMP+CPR+ERY+DOX	AMP+STR+ERY+DOX	CPR+STR+ERY+DOX	AMP+CPR+STR+ERY+DOX
	77.81692858	94.93582984	82.42586353	93.44616493	101.2243428	83.74270209	7.63354852	86.64386204	84.33939456	82.75507317	93.28978164	91.97294308	74.52483218	94.27741056	102.9528268	9.341323526	57.01499447	53.55829325	23.76482086	24.15575731	65.90365474	87.69321777	43.24991641	89.6067488	77.75520177	15.92551632	15.92551632	73.53720326	20.30811965	65.30696227	41.93307785
	70.5988928	92.29994967	81.02667338	94.49066371	101.2941394	67.76044288	6.240563664	74.90689482	67.17664821	75.49068948	78.06743835	87.08605939	83.28132864	77.74534474	108.985025	8.112732763	49.78359336	56.16507297	11.77654756	8.173125315	74.84650226	76.45697031	58.74182184	79.09411173	76.51736286	7.790639155	30.3975843	73.23603422	16.54755913	83.54302969	26.21036739
	67.43884839	88.28035377	87.03696851	96.59225653	101.8508017	57.0180957	5.861673389	64.39946219	54.51158889	81.96474608	80.72136082	93.33283993	84.51072543	77.85960108	113.3989792	10.40101642	66.82702389	52.39980757	5.249848894	22.59803377	75.03731389	69.01775031	77.5438207	86.08962735	86.38567147	11.03257719	26.38739839	76.59647954	10.93389582	61.75480147	22.28225339
AMP2CPR2STR3ERY1DOX1	AMP	CPR	STR	ERY	DOX	AMP+CPR	AMP+STR	AMP+ERY	AMP+DOX	CPR+STR	CPR+ERY	CPR+DOX	STR+ERY	STR+DOX	ERY+DOX	AMP+CPR+STR	AMP+CPR+ERY	AMP+CPR+DOX	AMP+STR+ERY	AMP+STR+DOX	AMP+ERY+DOX	CPR+STR+ERY	CPR+STR+DOX	CPR+ERY+DOX	STR+ERY+DOX	AMP+CPR+STR+ERY	AMP+CPR+STR+DOX	AMP+CPR+ERY+DOX	AMP+STR+ERY+DOX	CPR+STR+ERY+DOX	AMP+CPR+STR+ERY+DOX
	77.81692858	94.93582984	94.30051813	83.6514224	101.2243428	83.74270209	28.60008745	67.94063939	81.04729817	77.48771894	86.70558885	90.32689488	69.73709461	57.16753022	95.46272956	29.09390191	75.84167074	56.52118001	27.77706335	33.97031969	68.20812222	81.10902497	94.93582984	71.89115506	61.15908655	36.33651398	23.49733803	60.36881767	33.37362722	46.54201281	23.82654767
	70.5988928	92.29994967	98.24046921	82.95433537	100.9983361	67.76044288	31.10216407	51.65576246	68.34423754	72.91394061	75.81278309	90.30699547	65.98240469	62.22507331	95.67387687	24.01610468	44.24760946	65.44539507	19.44640161	21.37896326	52.68243583	72.91394061	74.52440866	104.4791142	67.50366569	22.02315048	23.31152491	57.77554102	20.23150478	73.55812783	38.77201812
	67.43884839	88.28035377	92.94362467	90.30262412	96.57163479	57.0180957	30.19649928	47.86046454	51.66956543	78.17538147	72.80711493	84.80676954	63.58304956	67.82065834	93.27215549	34.30164428	51.01826839	64.39946219	11.8615007	19.87442796	53.34714873	71.85977377	77.22804031	88.28035377	68.35035944	15.65086531	19.75601031	38.50547065	18.9270868	93.64862031	16.91398685
AMP2CPR2STR3ERY1DOX2	AMP	CPR	STR	ERY	DOX	AMP+CPR	AMP+STR	AMP+ERY	AMP+DOX	CPR+STR	CPR+ERY	CPR+DOX	STR+ERY	STR+DOX	ERY+DOX	AMP+CPR+STR	AMP+CPR+ERY	AMP+CPR+DOX	AMP+STR+ERY	AMP+STR+DOX	AMP+ERY+DOX	CPR+STR+ERY	CPR+STR+DOX	CPR+ERY+DOX	STR+ERY+DOX	AMP+CPR+STR+ERY	AMP+CPR+STR+DOX	AMP+CPR+ERY+DOX	AMP+STR+ERY+DOX	CPR+STR+ERY+DOX	AMP+CPR+STR+ERY+DOX
	77.81692858	94.93582984	94.30051813	83.6514224	95.46272956	83.74270209	28.60008745	67.94063939	89.93595844	77.48771894	86.70558885	105.1413287	69.73709461	62.69430052	99.20777818	29.09390191	75.84167074	51.4801574	27.77706335	36.33651398	56.02736555	81.10902497	76.17088038	63.99012371	62.08021493	36.33651398	28.10627299	75.5124611	25.80180551	49.83410921	24.48496695
	70.5988928	92.29994967	98.24046921	82.95433537	98.0403032	67.76044288	31.10216407	51.65576246	72.00805234	72.91394061	75.81278309	86.76396578	65.98240469	61.87683284	103.956369	24.01610468	44.24760946	49.01862104	19.44640161	24.59989935	38.51031706	72.91394061	89.98490186	91.91746351	66.33064516	22.02315048	22.6673377	73.55812783	24.59989935	76.1348767	44.24760946
	67.43884839	88.28035377	92.94362467	90.30262412	99.54116616	57.0180957	30.19649928	47.86046454	59.56407505	78.17538147	72.80711493	86.38567147	63.58304956	70.54483541	102.5106975	34.30164428	51.01826839	68.50460719	11.8615007	27.45315719	43.45927543	71.85977377	81.96474608	96.17486339	71.37722285	15.65086531	17.66396526	50.07092723	16.91398685	86.38567147	36.80815108
AMP2CPR2STR3ERY1DOX3	AMP	CPR	STR	ERY	DOX	AMP+CPR	AMP+STR	AMP+ERY	AMP+DOX	CPR+STR	CPR+ERY	CPR+DOX	STR+ERY	STR+DOX	ERY+DOX	AMP+CPR+STR	AMP+CPR+ERY	AMP+CPR+DOX	AMP+STR+ERY	AMP+STR+DOX	AMP+ERY+DOX	CPR+STR+ERY	CPR+STR+DOX	CPR+ERY+DOX	STR+ERY+DOX	AMP+CPR+STR+ERY	AMP+CPR+STR+DOX	AMP+CPR+ERY+DOX	AMP+STR+ERY+DOX	CPR+STR+ERY+DOX	AMP+CPR+STR+ERY+DOX
	77.81692858	94.93582984	94.30051813	83.6514224	101.2243428	83.74270209	28.60008745	67.94063939	84.33939456	77.48771894	86.70558885	91.97294308	69.73709461	70.65822299	98.63161685	29.09390191	75.84167074	53.55829325	27.77706335	38.25004501	58.00262339	81.10902497	57.07672128	60.63630051	62.69430052	36.33651398	21.19287055	60.69802731	28.53836064	61.68565623	23.60021604
	70.5988928	92.29994967	98.24046921	82.95433537	101.2941394	67.76044288	31.10216407	51.65576246	67.17664821	72.91394061	75.81278309	87.08605939	65.98240469	65.68914956	101.885746	24.01610468	44.24760946	56.16507297	19.44640161	33.67891293	42.95923503	72.91394061	76.1348767	79.67790639	57.5329912	22.02315048	21.05686965	66.79416205	43.28132864	69.69300453	46.82435833
	67.43884839	88.28035377	92.94362467	90.30262412	101.8508017	57.0180957	30.19649928	47.86046454	54.51158889	78.17538147	72.80711493	93.33283993	63.58304956	77.20393492	102.1807496	34.30164428	51.01826839	52.39980757	11.8615007	27.03869543	48.1959812	71.85977377	78.80694223	93.33283993	68.0476731	15.65086531	15.3548212	56.07075454	12.80884185	64.28104454	31.75566493
AMP2CPR2STR3ERY2DOX1	AMP	CPR	STR	ERY	DOX	AMP+CPR	AMP+STR	AMP+ERY	AMP+DOX	CPR+STR	CPR+ERY	CPR+DOX	STR+ERY	STR+DOX	ERY+DOX	AMP+CPR+STR	AMP+CPR+ERY	AMP+CPR+DOX	AMP+STR+ERY	AMP+STR+DOX	AMP+ERY+DOX	CPR+STR+ERY	CPR+STR+DOX	CPR+ERY+DOX	STR+ERY+DOX	AMP+CPR+STR+ERY	AMP+CPR+STR+DOX	AMP+CPR+ERY+DOX	AMP+STR+ERY+DOX	CPR+STR+ERY+DOX	AMP+CPR+STR+ERY+DOX
	77.81692858	94.93582984	94.30051813	96.90313288	101.2243428	83.74270209	28.60008745	59.31946195	81.04729817	77.48771894	89.66847561	90.32689488	91.23009019	57.16753022	99.7839395	29.09390191	58.39355984	56.52118001	29.8551992	33.97031969	72.32324271	80.45060569	94.93582984	53.55829325	60.54500096	22.50970911	23.49733803	71.89115506	40.38990767	69.58668759	38.31177182
	70.5988928	92.29994967	98.24046921	96.56128674	100.9983361	67.76044288	31.10216407	54.81630599	68.34423754	72.91394061	77.10115752	90.30699547	89.14956012	62.22507331	106.0269921	24.01610468	43.9859084	65.44539507	22.60694514	21.37896326	56.48716658	68.7267237	74.52440866	72.91394061	57.82624633	7.851031706	23.31152491	81.28837443	33.94061399	75.81278309	46.82435833
	67.43884839	88.28035377	92.94362467	96.90158272	96.57163479	57.0180957	30.19649928	67.123068	51.66956543	78.17538147	83.543648	84.80676954	92.64093833	67.82065834	99.54116616	34.30164428	62.09031812	64.39946219	22.59803377	19.87442796	52.93268697	73.4386757	77.22804031	80.70162454	66.23155505	22.30198966	19.75601031	94.28018108	15.65086531	62.38636223	34.59768839
AMP2CPR2STR3ERY2DOX2	AMP	CPR	STR	ERY	DOX	AMP+CPR	AMP+STR	AMP+ERY	AMP+DOX	CPR+STR	CPR+ERY	CPR+DOX	STR+ERY	STR+DOX	ERY+DOX	AMP+CPR+STR	AMP+CPR+ERY	AMP+CPR+DOX	AMP+STR+ERY	AMP+STR+DOX	AMP+ERY+DOX	CPR+STR+ERY	CPR+STR+DOX	CPR+ERY+DOX	STR+ERY+DOX	AMP+CPR+STR+ERY	AMP+CPR+STR+DOX	AMP+CPR+ERY+DOX	AMP+STR+ERY+DOX	CPR+STR+ERY+DOX	AMP+CPR+STR+ERY+DOX
	77.81692858	94.93582984	94.30051813	96.90313288	95.46272956	83.74270209	28.60008745	59.31946195	89.93595844	77.48771894	89.66847561	105.1413287	91.23009019	62.69430052	99.20777818	29.09390191	58.39355984	51.4801574	29.8551992	36.33651398	64.09300172	80.45060569	76.17088038	65.08063064	65.45768566	22.50970911	28.10627299	58.72276948	16.02839433	60.03960803	24.9170546
	70.5988928	92.29994967	98.24046921	96.56128674	98.0403032	67.76044288	31.10216407	54.81630599	72.00805234	72.91394061	77.10115752	86.76396578	89.14956012	61.87683284	106.6185986	24.01610468	43.9859084	49.01862104	22.60694514	24.59989935	56.48716658	68.7267237	89.98490186	59.70810267	57.23973607	7.851031706	22.6673377	81.28837443	29.04881731	96.74886764	44.56970307
	67.43884839	88.28035377	92.94362467	96.90158272	99.54116616	57.0180957	30.19649928	67.123068	59.56407505	78.17538147	83.543648	86.38567147	92.64093833	70.54483541	103.1705934	34.30164428	62.09031812	68.50460719	22.59803377	27.45315719	51.03800466	73.4386757	81.96474608	69.64931108	72.28528188	22.30198966	17.66396526	88.59613416	6.197190048	55.12341339	38.387053
AMP2CPR2STR3ERY2DOX3	AMP	CPR	STR	ERY	DOX	AMP+CPR	AMP+STR	AMP+ERY	AMP+DOX	CPR+STR	CPR+ERY	CPR+DOX	STR+ERY	STR+DOX	ERY+DOX	AMP+CPR+STR	AMP+CPR+ERY	AMP+CPR+DOX	AMP+STR+ERY	AMP+STR+DOX	AMP+ERY+DOX	CPR+STR+ERY	CPR+STR+DOX	CPR+ERY+DOX	STR+ERY+DOX	AMP+CPR+STR+ERY	AMP+CPR+STR+DOX	AMP+CPR+ERY+DOX	AMP+STR+ERY+DOX	CPR+STR+ERY+DOX	AMP+CPR+STR+ERY+DOX
	77.81692858	94.93582984	94.30051813	96.90313288	101.2243428	83.74270209	28.60008745	59.31946195	84.33939456	77.48771894	89.66847561	91.97294308	91.23009019	70.65822299	102.0885848	29.09390191	58.39355984	53.55829325	29.8551992	38.25004501	58.39355984	80.45060569	57.07672128	75.12152465	71.29149875	22.50970911	21.19287055	59.05197912	23.60021604	51.58303542	31.39836938
	70.5988928	92.29994967	98.24046921	96.56128674	101.2941394	67.76044288	31.10216407	54.81630599	67.17664821	72.91394061	77.10115752	87.08605939	89.14956012	65.68914956	110.7598447	24.01610468	43.9859084	56.16507297	22.60694514	33.67891293	58.74182184	68.7267237	76.1348767	86.76396578	58.65102639	7.851031706	21.05686965	79.35581278	24.8616004	95.1383996	44.24760946
	67.43884839	88.28035377	92.94362467	96.90158272	101.8508017	57.0180957	30.19649928	67.123068	54.51158889	78.17538147	83.543648	93.33283993	92.64093833	77.20393492	95.91173893	34.30164428	62.09031812	52.39980757	22.59803377	27.03869543	51.66956543	73.4386757	78.80694223	65.54416608	67.13961407	22.30198966	15.3548212	84.49098916	24.82823274	43.24217642	46.91312339
AMP2CPR2STR3ERY3DOX1	AMP	CPR	STR	ERY	DOX	AMP+CPR	AMP+STR	AMP+ERY	AMP+DOX	CPR+STR	CPR+ERY	CPR+DOX	STR+ERY	STR+DOX	ERY+DOX	AMP+CPR+STR	AMP+CPR+ERY	AMP+CPR+DOX	AMP+STR+ERY	AMP+STR+DOX	AMP+ERY+DOX	CPR+STR+ERY	CPR+STR+DOX	CPR+ERY+DOX	STR+ERY+DOX	AMP+CPR+STR+ERY	AMP+CPR+STR+DOX	AMP+CPR+ERY+DOX	AMP+STR+ERY+DOX	CPR+STR+ERY+DOX	AMP+CPR+STR+ERY+DOX
	77.81692858	94.93582984	94.30051813	93.44616493	101.2243428	83.74270209	28.60008745	86.64386204	81.04729817	77.48771894	93.28978164	90.32689488	81.09767799	57.16753022	97.4792942	29.09390191	57.01499447	56.52118001	51.4184306	33.97031969	64.97775263	80.77981533	94.93582984	72.81705717	81.73095375	16.35760397	23.49733803	77.48771894	32.3859983	63.00249479	36.66572362
	70.5988928	92.29994967	98.24046921	94.49066371	100.9983361	67.76044288	31.10216407	74.90689482	68.34423754	72.91394061	78.06743835	90.30699547	81.23167155	62.22507331	107.8018118	24.01610468	49.78359336	65.44539507	29.81378963	21.37896326	54.55460493	65.50578762	74.52440866	92.88374434	64.27785924	11.97785606	23.31152491	83.8651233	21.37896326	98.68142929	43.92551585
	67.43884839	88.28035377	92.94362467	96.59225653	96.57163479	57.0180957	30.19649928	64.39946219	51.66956543	78.17538147	80.72136082	84.80676954	82.04691638	67.82065834	95.91173893	34.30164428	66.82702389	64.39946219	30.19649928	19.87442796	59.87985543	65.54416608	77.22804031	57.0180957	77.43094968	17.24950351	19.75601031	79.438503	38.50547065	45.65000185	45.01844108
AMP2CPR2STR3ERY3DOX2	AMP	CPR	STR	ERY	DOX	AMP+CPR	AMP+STR	AMP+ERY	AMP+DOX	CPR+STR	CPR+ERY	CPR+DOX	STR+ERY	STR+DOX	ERY+DOX	AMP+CPR+STR	AMP+CPR+ERY	AMP+CPR+DOX	AMP+STR+ERY	AMP+STR+DOX	AMP+ERY+DOX	CPR+STR+ERY	CPR+STR+DOX	CPR+ERY+DOX	STR+ERY+DOX	AMP+CPR+STR+ERY	AMP+CPR+STR+DOX	AMP+CPR+ERY+DOX	AMP+STR+ERY+DOX	CPR+STR+ERY+DOX	AMP+CPR+STR+ERY+DOX
	77.81692858	94.93582984	94.30051813	93.44616493	95.46272956	83.74270209	28.60008745	86.64386204	89.93595844	77.48771894	93.28978164	105.1413287	81.09767799	62.69430052	97.4792942	29.09390191	57.01499447	51.4801574	51.4184306	36.33651398	70.5743165	80.77981533	76.17088038	72.81705717	73.44079831	16.35760397	28.10627299	83.74270209	33.70283686	64.31933335	44.89596461
	70.5988928	92.29994967	98.24046921	94.49066371	98.0403032	67.76044288	31.10216407	74.90689482	72.00805234	72.91394061	78.06743835	86.76396578	81.23167155	61.87683284	108.0976151	24.01610468	49.78359336	49.01862104	29.81378963	24.59989935	77.74534474	65.50578762	89.98490186	72.2697534	64.80938416	11.97785606	22.6673377	78.71162557	44.89179668	68.4046301	40.06039255
	67.43884839	88.28035377	92.94362467	96.59225653	99.54116616	57.0180957	30.19649928	64.39946219	59.56407505	78.17538147	80.72136082	86.38567147	82.04691638	70.54483541	103.8304893	34.30164428	66.82702389	68.50460719	30.19649928	27.45315719	60.19563582	65.54416608	81.96474608	73.77419235	78.71736663	17.24950351	17.66396526	83.85942839	35.24898543	55.45893005	47.54468416
AMP2CPR2STR3ERY3DOX3	AMP	CPR	STR	ERY	DOX	AMP+CPR	AMP+STR	AMP+ERY	AMP+DOX	CPR+STR	CPR+ERY	CPR+DOX	STR+ERY	STR+DOX	ERY+DOX	AMP+CPR+STR	AMP+CPR+ERY	AMP+CPR+DOX	AMP+STR+ERY	AMP+STR+DOX	AMP+ERY+DOX	CPR+STR+ERY	CPR+STR+DOX	CPR+ERY+DOX	STR+ERY+DOX	AMP+CPR+STR+ERY	AMP+CPR+STR+DOX	AMP+CPR+ERY+DOX	AMP+STR+ERY+DOX	CPR+STR+ERY+DOX	AMP+CPR+STR+ERY+DOX
	77.81692858	94.93582984	94.30051813	93.44616493	101.2243428	83.74270209	28.60008745	86.64386204	84.33939456	77.48771894	93.28978164	91.97294308	81.09767799	70.65822299	102.9528268	29.09390191	57.01499447	53.55829325	51.4184306	38.25004501	65.90365474	80.77981533	57.07672128	89.6067488	65.76472846	16.35760397	21.19287055	73.53720326	32.65348113	57.73514056	42.59149713
	70.5988928	92.29994967	98.24046921	94.49066371	101.2941394	67.76044288	31.10216407	74.90689482	67.17664821	72.91394061	78.06743835	87.08605939	81.23167155	65.68914956	108.985025	24.01610468	49.78359336	56.16507297	29.81378963	33.67891293	74.84650226	65.50578762	76.1348767	79.09411173	58.35777126	11.97785606	21.05686965	73.23603422	27.82083543	78.38953196	30.3975843
	67.43884839	88.28035377	92.94362467	96.59225653	101.8508017	57.0180957	30.19649928	64.39946219	54.51158889	78.17538147	80.72136082	93.33283993	82.04691638	77.20393492	113.3989792	34.30164428	66.82702389	52.39980757	30.19649928	27.03869543	75.03731389	65.54416608	78.80694223	86.08962735	77.20393492	17.24950351	15.3548212	76.59647954	46.30129889	61.43902108	35.54502954
AMP2CPR3STR1ERY1DOX1	AMP	CPR	STR	ERY	DOX	AMP+CPR	AMP+STR	AMP+ERY	AMP+DOX	CPR+STR	CPR+ERY	CPR+DOX	STR+ERY	STR+DOX	ERY+DOX	AMP+CPR+STR	AMP+CPR+ERY	AMP+CPR+DOX	AMP+STR+ERY	AMP+STR+DOX	AMP+ERY+DOX	CPR+STR+ERY	CPR+STR+DOX	CPR+ERY+DOX	STR+ERY+DOX	AMP+CPR+STR+ERY	AMP+CPR+STR+DOX	AMP+CPR+ERY+DOX	AMP+STR+ERY+DOX	CPR+STR+ERY+DOX	AMP+CPR+STR+ERY+DOX
	77.81692858	96.1427749	92.00576161	83.6514224	101.2243428	77.1585093	6.707646408	67.94063939	81.04729817	63.00134331	74.95682211	69.14219919	78.46597047	96.03889089	95.46272956	5.164476222	54.44304416	56.02736555	18.00365217	7.530670507	68.20812222	59.62387258	41.66186912	66.37881405	71.84011523	9.835137985	23.92942568	68.37272704	3.847637664	38.59144118	5.493685862
	70.5988928	99.70674487	85.0249584	82.95433537	100.9983361	60.73477604	7.146451938	51.65576246	68.34423754	69.55645161	75.07331378	69.26319648	79.40469588	92.71584396	95.67387687	10.36738802	49.66280825	53.97081027	5.596376447	-5.294413689	52.68243583	56.35997067	49.74340176	71.02272727	82.36272879	-1.348766985	13.1454454	44.56970307	17.0105687	48.44208211	0.583794665
	67.43884839	97.78660613	94.26199928	90.30262412	96.57163479	71.228213	6.809014543	47.86046454	51.66956543	75.31214529	77.8093076	71.07453651	84.36356138	93.60210342	93.27215549	8.387916466	52.59717031	70.30060812	10.7167968	28.91364147	53.34714873	65.62618237	45.11918275	68.35035944	77.43465484	52.9129507	5.545893005	40.5975157	2.703869543	61.38857359	5.23011262
AMP2CPR3STR1ERY1DOX2	AMP	CPR	STR	ERY	DOX	AMP+CPR	AMP+STR	AMP+ERY	AMP+DOX	CPR+STR	CPR+ERY	CPR+DOX	STR+ERY	STR+DOX	ERY+DOX	AMP+CPR+STR	AMP+CPR+ERY	AMP+CPR+DOX	AMP+STR+ERY	AMP+STR+DOX	AMP+ERY+DOX	CPR+STR+ERY	CPR+STR+DOX	CPR+ERY+DOX	STR+ERY+DOX	AMP+CPR+STR+ERY	AMP+CPR+STR+DOX	AMP+CPR+ERY+DOX	AMP+STR+ERY+DOX	CPR+STR+ERY+DOX	AMP+CPR+STR+ERY+DOX
	77.81692858	96.1427749	92.00576161	83.6514224	95.46272956	77.1585093	6.707646408	67.94063939	89.93595844	63.00134331	74.95682211	70.98445596	78.46597047	98.91969752	99.20777818	5.164476222	54.44304416	57.34420411	18.00365217	-1.357989764	56.02736555	59.62387258	41.35482633	67.60698522	81.3467771	9.835137985	5.22620303	42.69437514	25.57547388	41.04778353	24.58784496
	70.5988928	99.70674487	85.0249584	82.95433537	98.0403032	60.73477604	7.146451938	51.65576246	72.00805234	69.55645161	75.07331378	70.72947214	79.40469588	92.71584396	103.956369	10.36738802	49.66280825	59.44640161	5.596376447	1.469552089	38.51031706	56.35997067	50.62316716	63.98460411	89.16620447	-1.348766985	-1.348766985	46.11977856	20.9964771	56.65322581	-0.905888274
	67.43884839	97.78660613	94.26199928	90.30262412	99.54116616	71.228213	6.809014543	47.86046454	59.56407505	75.31214529	77.8093076	75.00945895	84.36356138	95.91173893	102.5106975	8.387916466	52.59717031	71.24794928	10.7167968	3.019649928	43.45927543	65.62618237	45.95157019	64.71812334	74.13517554	52.9129507	11.54572031	43.77505582	19.12444954	80.15512675	15.13772219
AMP2CPR3STR1ERY1DOX3	AMP	CPR	STR	ERY	DOX	AMP+CPR	AMP+STR	AMP+ERY	AMP+DOX	CPR+STR	CPR+ERY	CPR+DOX	STR+ERY	STR+DOX	ERY+DOX	AMP+CPR+STR	AMP+CPR+ERY	AMP+CPR+DOX	AMP+STR+ERY	AMP+STR+DOX	AMP+ERY+DOX	CPR+STR+ERY	CPR+STR+DOX	CPR+ERY+DOX	STR+ERY+DOX	AMP+CPR+STR+ERY	AMP+CPR+STR+DOX	AMP+CPR+ERY+DOX	AMP+STR+ERY+DOX	CPR+STR+ERY+DOX	AMP+CPR+STR+ERY+DOX
	77.81692858	96.1427749	92.00576161	83.6514224	101.2243428	77.1585093	6.707646408	67.94063939	84.33939456	63.00134331	74.95682211	65.76472846	78.46597047	81.63485776	98.63161685	5.164476222	54.44304416	58.49643785	18.00365217	17.67444253	58.00262339	59.62387258	66.37881405	64.53655728	75.29708318	9.835137985	18.33286181	45.16344744	21.29574857	38.28439839	14.77328258
	70.5988928	99.70674487	85.0249584	82.95433537	101.2941394	60.73477604	7.146451938	51.65576246	67.17664821	69.55645161	75.07331378	68.38343109	79.40469588	99.51931965	101.885746	10.36738802	49.66280825	52.44086563	5.596376447	29.57221943	42.95923503	56.35997067	57.82624633	67.21041056	77.33407284	-1.348766985	19.70810267	55.84297937	20.9964771	45.63782991	-0.261701057
	67.43884839	97.78660613	94.26199928	90.30262412	101.8508017	71.228213	6.809014543	47.86046454	54.51158889	75.31214529	77.8093076	72.28528188	84.36356138	104.1604372	102.1807496	8.387916466	52.59717031	58.93251428	10.7167968	22.59803377	48.1959812	65.62618237	73.49602724	68.35035944	75.78491519	52.9129507	9.966818389	53.56424774	8.387916466	67.74498676	2.388089159
AMP2CPR3STR1ERY2DOX1	AMP	CPR	STR	ERY	DOX	AMP+CPR	AMP+STR	AMP+ERY	AMP+DOX	CPR+STR	CPR+ERY	CPR+DOX	STR+ERY	STR+DOX	ERY+DOX	AMP+CPR+STR	AMP+CPR+ERY	AMP+CPR+DOX	AMP+STR+ERY	AMP+STR+DOX	AMP+ERY+DOX	CPR+STR+ERY	CPR+STR+DOX	CPR+ERY+DOX	STR+ERY+DOX	AMP+CPR+STR+ERY	AMP+CPR+STR+DOX	AMP+CPR+ERY+DOX	AMP+STR+ERY+DOX	CPR+STR+ERY+DOX	AMP+CPR+STR+ERY+DOX
	77.81692858	96.1427749	92.00576161	96.90313288	101.2243428	77.1585093	6.707646408	59.31946195	81.04729817	63.00134331	73.11456534	69.14219919	91.42960029	96.03889089	99.7839395	5.164476222	69.52496078	56.02736555	13.72392685	7.530670507	72.32324271	60.85204375	41.66186912	63.92247169	69.24738927	9.176718706	23.92942568	56.418302	15.7609115	39.05200537	25.90468352
	70.5988928	99.70674487	85.0249584	96.56128674	100.9983361	60.73477604	7.146451938	54.81630599	68.34423754	69.55645161	74.48680352	69.26319648	88.87040118	92.71584396	106.0269921	10.36738802	61.70105687	53.97081027	12.62204328	-5.294413689	56.48716658	66.62390029	49.74340176	68.38343109	90.94102422	8.434826371	13.1454454	63.25113236	21.05686965	50.49486804	19.06391545
	67.43884839	97.78660613	94.26199928	96.90158272	96.57163479	71.228213	6.809014543	67.123068	51.66956543	75.31214529	85.0737798	71.07453651	91.62241584	93.60210342	99.54116616	8.387916466	57.03783197	70.30060812	6.611651803	28.91364147	52.93268697	69.56110481	45.11918275	75.91751797	84.69350931	12.29569873	5.545893005	54.29448988	1.12496762	82.27393114	13.87460065
AMP2CPR3STR1ERY2DOX2	AMP	CPR	STR	ERY	DOX	AMP+CPR	AMP+STR	AMP+ERY	AMP+DOX	CPR+STR	CPR+ERY	CPR+DOX	STR+ERY	STR+DOX	ERY+DOX	AMP+CPR+STR	AMP+CPR+ERY	AMP+CPR+DOX	AMP+STR+ERY	AMP+STR+DOX	AMP+ERY+DOX	CPR+STR+ERY	CPR+STR+DOX	CPR+ERY+DOX	STR+ERY+DOX	AMP+CPR+STR+ERY	AMP+CPR+STR+DOX	AMP+CPR+ERY+DOX	AMP+STR+ERY+DOX	CPR+STR+ERY+DOX	AMP+CPR+STR+ERY+DOX
	77.81692858	96.1427749	92.00576161	96.90313288	95.46272956	77.1585093	6.707646408	59.31946195	89.93595844	63.00134331	73.11456534	70.98445596	91.42960029	98.91969752	99.20777818	5.164476222	69.52496078	57.34420411	13.72392685	-1.357989764	64.09300172	60.85204375	41.35482633	70.06332758	74.4328412	9.176718706	5.22620303	53.22908361	20.30811965	46.88159662	34.79334379
	70.5988928	99.70674487	85.0249584	96.56128674	98.0403032	60.73477604	7.146451938	54.81630599	72.00805234	69.55645161	74.48680352	70.72947214	88.87040118	92.71584396	106.6185986	10.36738802	61.70105687	59.44640161	12.62204328	1.469552089	56.48716658	66.62390029	50.62316716	56.35997067	89.75781106	8.434826371	-1.348766985	74.84650226	30.3975843	50.62316716	13.00452944
	67.43884839	97.78660613	94.26199928	96.90158272	99.54116616	71.228213	6.809014543	67.123068	59.56407505	75.31214529	85.0737798	75.00945895	91.62241584	95.91173893	103.1705934	8.387916466	57.03783197	71.24794928	6.611651803	3.019649928	51.03800466	69.56110481	45.95157019	74.10139992	72.48543589	12.29569873	11.54572031	50.40644389	3.138067572	48.9784336	17.66396526
AMP2CPR3STR1ERY2DOX3	AMP	CPR	STR	ERY	DOX	AMP+CPR	AMP+STR	AMP+ERY	AMP+DOX	CPR+STR	CPR+ERY	CPR+DOX	STR+ERY	STR+DOX	ERY+DOX	AMP+CPR+STR	AMP+CPR+ERY	AMP+CPR+DOX	AMP+STR+ERY	AMP+STR+DOX	AMP+ERY+DOX	CPR+STR+ERY	CPR+STR+DOX	CPR+ERY+DOX	STR+ERY+DOX	AMP+CPR+STR+ERY	AMP+CPR+STR+DOX	AMP+CPR+ERY+DOX	AMP+STR+ERY+DOX	CPR+STR+ERY+DOX	AMP+CPR+STR+ERY+DOX
	77.81692858	96.1427749	92.00576161	96.90313288	101.2243428	77.1585093	6.707646408	59.31946195	84.33939456	63.00134331	73.11456534	65.76472846	91.42960029	81.63485776	102.0885848	5.164476222	69.52496078	58.49643785	13.72392685	17.67444253	58.39355984	60.85204375	66.37881405	74.0548839	77.31364782	9.176718706	18.33286181	73.20799362	25.90468352	52.25484552	26.13101515
	70.5988928	99.70674487	85.0249584	96.56128674	101.2941394	60.73477604	7.146451938	54.81630599	67.17664821	69.55645161	74.48680352	68.38343109	88.87040118	99.51931965	110.7598447	10.36738802	61.70105687	52.44086563	12.62204328	29.57221943	58.74182184	66.62390029	57.82624633	54.60043988	94.19486042	8.434826371	19.70810267	66.47206844	17.51383996	56.06671554	22.34524409
	67.43884839	97.78660613	94.26199928	96.90158272	101.8508017	71.228213	6.809014543	67.123068	54.51158889	75.31214529	85.0737798	72.28528188	91.62241584	104.1604372	95.91173893	8.387916466	57.03783197	58.93251428	6.611651803	22.59803377	51.66956543	69.56110481	73.49602724	82.57661748	73.47527968	12.29569873	9.966818389	66.80728762	-1.085495072	59.87514188	21.65069262
AMP2CPR3STR1ERY3DOX1	AMP	CPR	STR	ERY	DOX	AMP+CPR	AMP+STR	AMP+ERY	AMP+DOX	CPR+STR	CPR+ERY	CPR+DOX	STR+ERY	STR+DOX	ERY+DOX	AMP+CPR+STR	AMP+CPR+ERY	AMP+CPR+DOX	AMP+STR+ERY	AMP+STR+DOX	AMP+ERY+DOX	CPR+STR+ERY	CPR+STR+DOX	CPR+ERY+DOX	STR+ERY+DOX	AMP+CPR+STR+ERY	AMP+CPR+STR+DOX	AMP+CPR+ERY+DOX	AMP+STR+ERY+DOX	CPR+STR+ERY+DOX	AMP+CPR+STR+ERY+DOX
	77.81692858	96.1427749	92.00576161	93.44616493	101.2243428	77.1585093	6.707646408	86.64386204	81.04729817	63.00134331	72.80752255	69.14219919	88.83687432	96.03889089	97.4792942	5.164476222	74.79231501	56.02736555	57.67341375	7.530670507	64.97775263	59.62387258	41.66186912	60.23795817	83.36334174	4.835266583	23.92942568	90.65610452	30.7399501	50.41258875	42.03595587
	70.5988928	99.70674487	85.0249584	94.49066371	100.9983361	60.73477604	7.146451938	74.90689482	68.34423754	69.55645161	75.65982405	69.26319648	85.0249584	92.71584396	107.8018118	10.36738802	64.27780574	53.97081027	36.57775541	-5.294413689	54.55460493	62.81158358	49.74340176	71.60923754	95.08227029	-0.060392552	13.1454454	67.43834927	-2.838449925	39.05791789	4.308002013
	67.43884839	97.78660613	94.26199928	96.59225653	96.57163479	71.228213	6.809014543	64.39946219	51.66956543	75.31214529	81.74423004	71.07453651	83.37371759	93.60210342	95.91173893	8.387916466	70.08350911	70.30060812	39.66991082	28.91364147	59.87985543	69.56110481	45.11918275	94.98675747	75.12501933	1.440748005	5.545893005	65.54416608	-1.085495072	54.42678774	4.282771466
AMP2CPR3STR1ERY3DOX2	AMP	CPR	STR	ERY	DOX	AMP+CPR	AMP+STR	AMP+ERY	AMP+DOX	CPR+STR	CPR+ERY	CPR+DOX	STR+ERY	STR+DOX	ERY+DOX	AMP+CPR+STR	AMP+CPR+ERY	AMP+CPR+DOX	AMP+STR+ERY	AMP+STR+DOX	AMP+ERY+DOX	CPR+STR+ERY	CPR+STR+DOX	CPR+ERY+DOX	STR+ERY+DOX	AMP+CPR+STR+ERY	AMP+CPR+STR+DOX	AMP+CPR+ERY+DOX	AMP+STR+ERY+DOX	CPR+STR+ERY+DOX	AMP+CPR+STR+ERY+DOX
	77.81692858	96.1427749	92.00576161	93.44616493	95.46272956	77.1585093	6.707646408	86.64386204	89.93595844	63.00134331	72.80752255	70.98445596	88.83687432	98.91969752	97.4792942	5.164476222	74.79231501	57.34420411	57.67341375	-1.357989764	70.5743165	59.62387258	41.35482633	74.03569372	100.0720202	4.835266583	5.22620303	84.73033101	23.2710064	49.49146037	28.43548263
	70.5988928	99.70674487	85.0249584	94.49066371	98.0403032	60.73477604	7.146451938	74.90689482	72.00805234	69.55645161	75.65982405	70.72947214	85.0249584	92.71584396	108.0976151	10.36738802	64.27780574	59.44640161	36.57775541	1.469552089	77.74534474	62.81158358	50.62316716	71.02272727	84.43335182	-0.060392552	-1.348766985	80.96628083	24.7408153	43.45674487	13.58832411
	67.43884839	97.78660613	94.26199928	96.59225653	99.54116616	71.228213	6.809014543	64.39946219	59.56407505	75.31214529	81.74423004	75.00945895	83.37371759	95.91173893	103.8304893	8.387916466	70.08350911	71.24794928	39.66991082	3.019649928	60.19563582	69.56110481	45.95157019	78.03632236	84.69350931	1.440748005	11.54572031	63.96526416	2.703869543	61.08588725	15.45350257
AMP2CPR3STR1ERY3DOX3	AMP	CPR	STR	ERY	DOX	AMP+CPR	AMP+STR	AMP+ERY	AMP+DOX	CPR+STR	CPR+ERY	CPR+DOX	STR+ERY	STR+DOX	ERY+DOX	AMP+CPR+STR	AMP+CPR+ERY	AMP+CPR+DOX	AMP+STR+ERY	AMP+STR+DOX	AMP+ERY+DOX	CPR+STR+ERY	CPR+STR+DOX	CPR+ERY+DOX	STR+ERY+DOX	AMP+CPR+STR+ERY	AMP+CPR+STR+DOX	AMP+CPR+ERY+DOX	AMP+STR+ERY+DOX	CPR+STR+ERY+DOX	AMP+CPR+STR+ERY+DOX
	77.81692858	96.1427749	92.00576161	93.44616493	101.2243428	77.1585093	6.707646408	86.64386204	84.33939456	63.00134331	72.80752255	65.76472846	88.83687432	81.63485776	102.9528268	5.164476222	74.79231501	58.49643785	57.67341375	17.67444253	65.90365474	59.62387258	66.37881405	82.34503934	91.14151963	4.835266583	18.33286181	83.74270209	22.18049947	50.41258875	28.209151
	70.5988928	99.70674487	85.0249584	94.49066371	101.2941394	60.73477604	7.146451938	74.90689482	67.17664821	69.55645161	75.65982405	68.38343109	85.0249584	99.51931965	108.985025	10.36738802	64.27780574	52.44086563	36.57775541	29.57221943	74.84650226	62.81158358	57.82624633	78.06085044	96.56128674	-0.060392552	19.70810267	96.42677403	22.80825365	58.99926686	21.19778561
	67.43884839	97.78660613	94.26199928	96.59225653	101.8508017	71.228213	6.809014543	64.39946219	54.51158889	75.31214529	81.74423004	72.28528188	83.37371759	104.1604372	113.3989792	8.387916466	70.08350911	58.93251428	39.66991082	22.59803377	75.03731389	69.56110481	73.49602724	84.69542187	90.96251998	1.440748005	9.966818389	84.49098916	4.93406851	74.70677261	0.296044111
AMP2CPR3STR2ERY1DOX1	AMP	CPR	STR	ERY	DOX	AMP+CPR	AMP+STR	AMP+ERY	AMP+DOX	CPR+STR	CPR+ERY	CPR+DOX	STR+ERY	STR+DOX	ERY+DOX	AMP+CPR+STR	AMP+CPR+ERY	AMP+CPR+DOX	AMP+STR+ERY	AMP+STR+DOX	AMP+ERY+DOX	CPR+STR+ERY	CPR+STR+DOX	CPR+ERY+DOX	STR+ERY+DOX	AMP+CPR+STR+ERY	AMP+CPR+STR+DOX	AMP+CPR+ERY+DOX	AMP+STR+ERY+DOX	CPR+STR+ERY+DOX	AMP+CPR+STR+ERY+DOX
	77.81692858	96.1427749	82.42586353	83.6514224	101.2243428	77.1585093	7.63354852	67.94063939	81.04729817	61.77317214	74.95682211	69.14219919	73.20799362	85.71795993	95.46272956	9.999742805	54.44304416	56.02736555	24.48496695	24.32036213	68.20812222	61.15908655	49.49146037	66.37881405	90.32689488	16.25472596	30.18440884	68.37272704	24.81417659	79.2746114	27.22152208
	70.5988928	99.70674487	81.02667338	82.95433537	100.9983361	60.73477604	6.240563664	51.65576246	68.34423754	66.03739003	75.07331378	69.26319648	67.11625566	73.88022144	95.67387687	12.03824862	49.66280825	53.97081027	22.02315048	6.059386009	52.68243583	63.69134897	42.57697947	71.02272727	79.03371917	14.29290388	28.6059386	44.56970307	24.27780574	71.90249267	19.70810267
	67.43884839	97.78660613	87.03696851	90.30262412	96.57163479	71.228213	5.861673389	47.86046454	51.66956543	68.65304578	77.8093076	71.07453651	65.2283857	75.96491877	93.27215549	17.54554762	52.59717031	70.30060812	14.38774377	0.177626466	53.34714873	72.89065456	71.67990919	68.35035944	82.91208723	16.28242608	21.33491224	40.5975157	22.91381416	75.61483163	15.01930454
AMP2CPR3STR2ERY1DOX2	AMP	CPR	STR	ERY	DOX	AMP+CPR	AMP+STR	AMP+ERY	AMP+DOX	CPR+STR	CPR+ERY	CPR+DOX	STR+ERY	STR+DOX	ERY+DOX	AMP+CPR+STR	AMP+CPR+ERY	AMP+CPR+DOX	AMP+STR+ERY	AMP+STR+DOX	AMP+ERY+DOX	CPR+STR+ERY	CPR+STR+DOX	CPR+ERY+DOX	STR+ERY+DOX	AMP+CPR+STR+ERY	AMP+CPR+STR+DOX	AMP+CPR+ERY+DOX	AMP+STR+ERY+DOX	CPR+STR+ERY+DOX	AMP+CPR+STR+ERY+DOX
	77.81692858	96.1427749	82.42586353	83.6514224	95.46272956	77.1585093	7.63354852	67.94063939	89.93595844	61.77317214	74.95682211	70.98445596	73.20799362	90.32689488	99.20777818	9.999742805	54.44304416	57.34420411	24.48496695	13.39471721	56.02736555	61.15908655	68.22107081	67.60698522	82.42586353	16.25472596	23.60021604	42.69437514	19.97891001	69.44924199	21.29574857
	70.5988928	99.70674487	81.02667338	82.95433537	98.0403032	60.73477604	6.240563664	51.65576246	72.00805234	66.03739003	75.07331378	70.72947214	67.11625566	77.42325113	103.956369	12.03824862	49.66280825	59.44640161	22.02315048	22.60694514	38.51031706	63.69134897	65.15762463	63.98460411	89.01862104	14.29290388	18.41972823	46.11977856	37.48364368	74.24853372	16.86965274
	67.43884839	97.78660613	87.03696851	90.30262412	99.54116616	71.228213	5.861673389	47.86046454	59.56407505	68.65304578	77.8093076	75.00945895	65.2283857	74.70179723	102.5106975	17.54554762	52.59717031	71.24794928	14.38774377	10.59837916	43.45927543	72.89065456	82.87930382	64.71812334	89.22769493	16.28242608	26.38739839	43.77505582	31.43988454	79.54975407	17.97974565
AMP2CPR3STR2ERY1DOX3	AMP	CPR	STR	ERY	DOX	AMP+CPR	AMP+STR	AMP+ERY	AMP+DOX	CPR+STR	CPR+ERY	CPR+DOX	STR+ERY	STR+DOX	ERY+DOX	AMP+CPR+STR	AMP+CPR+ERY	AMP+CPR+DOX	AMP+STR+ERY	AMP+STR+DOX	AMP+ERY+DOX	CPR+STR+ERY	CPR+STR+DOX	CPR+ERY+DOX	STR+ERY+DOX	AMP+CPR+STR+ERY	AMP+CPR+STR+DOX	AMP+CPR+ERY+DOX	AMP+STR+ERY+DOX	CPR+STR+ERY+DOX	AMP+CPR+STR+ERY+DOX
	77.81692858	96.1427749	82.42586353	83.6514224	101.2243428	77.1585093	7.63354852	67.94063939	84.33939456	61.77317214	74.95682211	65.76472846	73.20799362	94.27741056	98.63161685	9.999742805	54.44304416	58.49643785	24.48496695	24.15575731	58.00262339	61.15908655	65.45768566	64.53655728	61.35644659	16.25472596	14.38234613	45.16344744	26.23389316	70.67741316	25.24626424
	70.5988928	99.70674487	81.02667338	82.95433537	101.2941394	60.73477604	6.240563664	51.65576246	67.17664821	66.03739003	75.07331378	68.38343109	67.11625566	77.74534474	101.885746	12.03824862	49.66280825	52.44086563	22.02315048	8.173125315	42.95923503	63.69134897	59.58577713	67.21041056	100.9360845	14.29290388	11.33366885	55.84297937	28.78711626	83.04618768	26.8545546
	67.43884839	97.78660613	87.03696851	90.30262412	101.8508017	71.228213	5.861673389	47.86046454	54.51158889	68.65304578	77.8093076	72.28528188	65.2283857	77.85960108	102.1807496	17.54554762	52.59717031	58.93251428	14.38774377	22.59803377	48.1959812	72.89065456	60.78320091	68.35035944	82.28052647	16.28242608	19.12444954	53.56424774	10.59837916	89.53840333	11.97991834
AMP2CPR3STR2ERY2DOX1	AMP	CPR	STR	ERY	DOX	AMP+CPR	AMP+STR	AMP+ERY	AMP+DOX	CPR+STR	CPR+ERY	CPR+DOX	STR+ERY	STR+DOX	ERY+DOX	AMP+CPR+STR	AMP+CPR+ERY	AMP+CPR+DOX	AMP+STR+ERY	AMP+STR+DOX	AMP+ERY+DOX	CPR+STR+ERY	CPR+STR+DOX	CPR+ERY+DOX	STR+ERY+DOX	AMP+CPR+STR+ERY	AMP+CPR+STR+DOX	AMP+CPR+ERY+DOX	AMP+STR+ERY+DOX	CPR+STR+ERY+DOX	AMP+CPR+STR+ERY+DOX
	77.81692858	96.1427749	82.42586353	96.90313288	101.2243428	77.1585093	7.63354852	59.31946195	81.04729817	61.77317214	73.11456534	69.14219919	85.65623312	85.71795993	99.7839395	9.999742805	69.52496078	56.02736555	16.35760397	24.32036213	72.32324271	56.55344464	49.49146037	63.92247169	68.59905867	13.72392685	30.18440884	56.418302	14.05313649	60.54500096	31.39836938
	70.5988928	99.70674487	81.02667338	96.56128674	100.9983361	60.73477604	6.240563664	54.81630599	68.34423754	66.03739003	74.48680352	69.26319648	76.83945647	73.88022144	106.0269921	12.03824862	61.70105687	53.97081027	15.84297937	6.059386009	56.48716658	56.06671554	42.57697947	68.38343109	82.89884248	1.992954202	28.6059386	63.25113236	25.50578762	77.47434018	35.87317564
	67.43884839	97.78660613	87.03696851	96.90158272	96.57163479	71.228213	5.861673389	67.123068	51.66956543	68.65304578	85.0737798	71.07453651	82.93182351	75.96491877	99.54116616	17.54554762	57.03783197	70.30060812	9.966818389	0.177626466	52.93268697	62.29663261	71.67990919	75.91751797	96.80642416	6.611651803	21.33491224	54.29448988	14.38774377	85.90616723	14.70352416
AMP2CPR3STR2ERY2DOX2	AMP	CPR	STR	ERY	DOX	AMP+CPR	AMP+STR	AMP+ERY	AMP+DOX	CPR+STR	CPR+ERY	CPR+DOX	STR+ERY	STR+DOX	ERY+DOX	AMP+CPR+STR	AMP+CPR+ERY	AMP+CPR+DOX	AMP+STR+ERY	AMP+STR+DOX	AMP+ERY+DOX	CPR+STR+ERY	CPR+STR+DOX	CPR+ERY+DOX	STR+ERY+DOX	AMP+CPR+STR+ERY	AMP+CPR+STR+DOX	AMP+CPR+ERY+DOX	AMP+STR+ERY+DOX	CPR+STR+ERY+DOX	AMP+CPR+STR+ERY+DOX
	77.81692858	96.1427749	82.42586353	96.90313288	95.46272956	77.1585093	7.63354852	59.31946195	89.93595844	61.77317214	73.11456534	70.98445596	85.65623312	90.32689488	99.20777818	9.999742805	69.52496078	57.34420411	16.35760397	13.39471721	64.09300172	56.55344464	68.22107081	70.06332758	63.66091407	13.72392685	23.60021604	53.22908361	23.92942568	60.85204375	39.95782001
	70.5988928	99.70674487	81.02667338	96.56128674	98.0403032	60.73477604	6.240563664	54.81630599	72.00805234	66.03739003	74.48680352	70.72947214	76.83945647	77.42325113	106.6185986	12.03824862	61.70105687	59.44640161	15.84297937	22.60694514	56.48716658	56.06671554	65.15762463	56.35997067	81.28837443	1.992954202	18.41972823	74.84650226	27.82083543	68.09017595	35.55108203
	67.43884839	97.78660613	87.03696851	96.90158272	99.54116616	71.228213	5.861673389	67.123068	59.56407505	68.65304578	85.0737798	75.00945895	82.93182351	74.70179723	103.1705934	17.54554762	57.03783197	71.24794928	9.966818389	10.59837916	51.03800466	62.29663261	82.87930382	74.10139992	94.59596146	6.611651803	26.38739839	50.40644389	17.66396526	69.56110481	9.453675264
AMP2CPR3STR2ERY2DOX3	AMP	CPR	STR	ERY	DOX	AMP+CPR	AMP+STR	AMP+ERY	AMP+DOX	CPR+STR	CPR+ERY	CPR+DOX	STR+ERY	STR+DOX	ERY+DOX	AMP+CPR+STR	AMP+CPR+ERY	AMP+CPR+DOX	AMP+STR+ERY	AMP+STR+DOX	AMP+ERY+DOX	CPR+STR+ERY	CPR+STR+DOX	CPR+ERY+DOX	STR+ERY+DOX	AMP+CPR+STR+ERY	AMP+CPR+STR+DOX	AMP+CPR+ERY+DOX	AMP+STR+ERY+DOX	CPR+STR+ERY+DOX	AMP+CPR+STR+ERY+DOX
	77.81692858	96.1427749	82.42586353	96.90313288	101.2243428	77.1585093	7.63354852	59.31946195	84.33939456	61.77317214	73.11456534	65.76472846	85.65623312	94.27741056	102.0885848	9.999742805	69.52496078	58.49643785	16.35760397	24.15575731	58.39355984	56.55344464	65.45768566	74.0548839	75.77994393	13.72392685	14.38234613	73.20799362	29.8551992	58.08865861	33.04441758
	70.5988928	99.70674487	81.02667338	96.56128674	101.2941394	60.73477604	6.240563664	54.81630599	67.17664821	66.03739003	74.48680352	68.38343109	76.83945647	77.74534474	110.7598447	12.03824862	61.70105687	52.44086563	15.84297937	8.173125315	58.74182184	56.06671554	59.58577713	54.60043988	80.96628083	1.992954202	11.33366885	66.47206844	21.70105687	54.01392962	35.55108203
	67.43884839	97.78660613	87.03696851	96.90158272	101.8508017	71.228213	5.861673389	67.123068	54.51158889	68.65304578	85.0737798	72.28528188	82.93182351	77.85960108	95.91173893	17.54554762	57.03783197	58.93251428	9.966818389	22.59803377	51.66956543	62.29663261	60.78320091	82.57661748	78.49116185	6.611651803	19.12444954	66.80728762	28.08471796	59.2697692	29.54520224
AMP2CPR3STR2ERY3DOX1	AMP	CPR	STR	ERY	DOX	AMP+CPR	AMP+STR	AMP+ERY	AMP+DOX	CPR+STR	CPR+ERY	CPR+DOX	STR+ERY	STR+DOX	ERY+DOX	AMP+CPR+STR	AMP+CPR+ERY	AMP+CPR+DOX	AMP+STR+ERY	AMP+STR+DOX	AMP+ERY+DOX	CPR+STR+ERY	CPR+STR+DOX	CPR+ERY+DOX	STR+ERY+DOX	AMP+CPR+STR+ERY	AMP+CPR+STR+DOX	AMP+CPR+ERY+DOX	AMP+STR+ERY+DOX	CPR+STR+ERY+DOX	AMP+CPR+STR+ERY+DOX
	77.81692858	96.1427749	82.42586353	93.44616493	101.2243428	77.1585093	7.63354852	86.64386204	81.04729817	61.77317214	72.80752255	69.14219919	74.52483218	85.71795993	97.4792942	9.999742805	74.79231501	56.02736555	23.76482086	24.32036213	64.97775263	55.32527346	49.49146037	60.23795817	66.89128366	12.90090275	30.18440884	90.65610452	27.22152208	56.86048743	27.77706335
	70.5988928	99.70674487	81.02667338	94.49066371	100.9983361	60.73477604	6.240563664	74.90689482	68.34423754	66.03739003	75.65982405	69.26319648	83.28132864	73.88022144	107.8018118	12.03824862	64.27780574	53.97081027	11.77654756	6.059386009	54.55460493	63.69134897	42.57697947	71.60923754	86.76396578	6.381479618	28.6059386	67.43834927	26.21036739	62.51832845	38.1278309
	67.43884839	97.78660613	87.03696851	96.59225653	96.57163479	71.228213	5.861673389	64.39946219	51.66956543	68.65304578	81.74423004	71.07453651	84.51072543	75.96491877	95.91173893	17.54554762	70.08350911	70.30060812	5.249848894	0.177626466	59.87985543	68.35035944	71.67990919	94.98675747	76.91225993	15.98638197	21.33491224	65.54416608	20.82176911	64.415437	35.22924916
AMP2CPR3STR2ERY3DOX2	AMP	CPR	STR	ERY	DOX	AMP+CPR	AMP+STR	AMP+ERY	AMP+DOX	CPR+STR	CPR+ERY	CPR+DOX	STR+ERY	STR+DOX	ERY+DOX	AMP+CPR+STR	AMP+CPR+ERY	AMP+CPR+DOX	AMP+STR+ERY	AMP+STR+DOX	AMP+ERY+DOX	CPR+STR+ERY	CPR+STR+DOX	CPR+ERY+DOX	STR+ERY+DOX	AMP+CPR+STR+ERY	AMP+CPR+STR+DOX	AMP+CPR+ERY+DOX	AMP+STR+ERY+DOX	CPR+STR+ERY+DOX	AMP+CPR+STR+ERY+DOX
	77.81692858	96.1427749	82.42586353	93.44616493	95.46272956	77.1585093	7.63354852	86.64386204	89.93595844	61.77317214	72.80752255	70.98445596	74.52483218	90.32689488	97.4792942	9.999742805	74.79231501	57.34420411	23.76482086	13.39471721	70.5743165	55.32527346	68.22107081	74.03569372	83.022556	12.90090275	23.60021604	84.73033101	32.3859983	48.57033199	30.41074046
	70.5988928	99.70674487	81.02667338	94.49066371	98.0403032	60.73477604	6.240563664	74.90689482	72.00805234	66.03739003	75.65982405	70.72947214	83.28132864	77.42325113	108.0976151	12.03824862	64.27780574	59.44640161	11.77654756	22.60694514	77.74534474	63.69134897	65.15762463	71.02272727	86.76396578	6.381479618	18.41972823	80.96628083	58.09763463	59.87903226	30.07549069
	67.43884839	97.78660613	87.03696851	96.59225653	99.54116616	71.228213	5.861673389	64.39946219	59.56407505	68.65304578	81.74423004	75.00945895	84.51072543	74.70179723	103.8304893	17.54554762	70.08350911	71.24794928	5.249848894	10.59837916	60.19563582	68.35035944	82.87930382	78.03632236	73.45841197	15.98638197	26.38739839	63.96526416	16.59820647	58.05902384	23.22959454
AMP2CPR3STR2ERY3DOX3	AMP	CPR	STR	ERY	DOX	AMP+CPR	AMP+STR	AMP+ERY	AMP+DOX	CPR+STR	CPR+ERY	CPR+DOX	STR+ERY	STR+DOX	ERY+DOX	AMP+CPR+STR	AMP+CPR+ERY	AMP+CPR+DOX	AMP+STR+ERY	AMP+STR+DOX	AMP+ERY+DOX	CPR+STR+ERY	CPR+STR+DOX	CPR+ERY+DOX	STR+ERY+DOX	AMP+CPR+STR+ERY	AMP+CPR+STR+DOX	AMP+CPR+ERY+DOX	AMP+STR+ERY+DOX	CPR+STR+ERY+DOX	AMP+CPR+STR+ERY+DOX
	77.81692858	96.1427749	82.42586353	93.44616493	101.2243428	77.1585093	7.63354852	86.64386204	84.33939456	61.77317214	72.80752255	65.76472846	74.52483218	94.27741056	102.9528268	9.999742805	74.79231501	58.49643785	23.76482086	24.15575731	65.90365474	55.32527346	65.45768566	82.34503934	77.75520177	12.90090275	14.38234613	83.74270209	20.30811965	49.79850317	23.82654767
	70.5988928	99.70674487	81.02667338	94.49066371	101.2941394	60.73477604	6.240563664	74.90689482	67.17664821	66.03739003	75.65982405	68.38343109	83.28132864	77.74534474	108.985025	12.03824862	64.27780574	52.44086563	11.77654756	8.173125315	74.84650226	63.69134897	59.58577713	78.06085044	76.51736286	6.381479618	11.33366885	96.42677403	16.54755913	74.83504399	20.73477604
	67.43884839	97.78660613	87.03696851	96.59225653	101.8508017	71.228213	5.861673389	64.39946219	54.51158889	68.65304578	81.74423004	72.28528188	84.51072543	77.85960108	113.3989792	17.54554762	70.08350911	58.93251428	5.249848894	22.59803377	75.03731389	68.35035944	60.78320091	84.69542187	86.38567147	15.98638197	19.12444954	84.49098916	10.93389582	84.99810821	20.38757108
AMP2CPR3STR3ERY1DOX1	AMP	CPR	STR	ERY	DOX	AMP+CPR	AMP+STR	AMP+ERY	AMP+DOX	CPR+STR	CPR+ERY	CPR+DOX	STR+ERY	STR+DOX	ERY+DOX	AMP+CPR+STR	AMP+CPR+ERY	AMP+CPR+DOX	AMP+STR+ERY	AMP+STR+DOX	AMP+ERY+DOX	CPR+STR+ERY	CPR+STR+DOX	CPR+ERY+DOX	STR+ERY+DOX	AMP+CPR+STR+ERY	AMP+CPR+STR+DOX	AMP+CPR+ERY+DOX	AMP+STR+ERY+DOX	CPR+STR+ERY+DOX	AMP+CPR+STR+ERY+DOX
	77.81692858	96.1427749	94.30051813	83.6514224	101.2243428	77.1585093	28.60008745	67.94063939	81.04729817	57.45538284	74.95682211	69.14219919	69.73709461	57.16753022	95.46272956	21.85128983	54.44304416	56.02736555	27.77706335	33.97031969	68.20812222	56.55344464	62.08021493	66.37881405	61.15908655	16.35760397	29.09390191	68.37272704	33.37362722	71.29149875	27.55073172
	70.5988928	99.70674487	98.24046921	82.95433537	100.9983361	60.73477604	31.10216407	51.65576246	68.34423754	60.11730205	75.07331378	69.26319648	65.98240469	62.22507331	95.67387687	14.99748364	49.66280825	53.97081027	19.44640161	21.37896326	52.68243583	58.99926686	68.09017595	71.02272727	67.50366569	16.86965274	29.10920986	44.56970307	20.23150478	64.8643695	19.76849522
	67.43884839	97.78660613	92.94362467	90.30262412	96.57163479	71.228213	30.19649928	47.86046454	51.66956543	61.99394627	77.8093076	71.07453651	63.58304956	67.82065834	93.27215549	24.19667197	52.59717031	70.30060812	11.8615007	19.87442796	53.34714873	68.0476731	68.35035944	68.35035944	68.35035944	19.44022993	20.0717907	40.5975157	18.9270868	71.67990919	27.33473954
AMP2CPR3STR3ERY1DOX2	AMP	CPR	STR	ERY	DOX	AMP+CPR	AMP+STR	AMP+ERY	AMP+DOX	CPR+STR	CPR+ERY	CPR+DOX	STR+ERY	STR+DOX	ERY+DOX	AMP+CPR+STR	AMP+CPR+ERY	AMP+CPR+DOX	AMP+STR+ERY	AMP+STR+DOX	AMP+ERY+DOX	CPR+STR+ERY	CPR+STR+DOX	CPR+ERY+DOX	STR+ERY+DOX	AMP+CPR+STR+ERY	AMP+CPR+STR+DOX	AMP+CPR+ERY+DOX	AMP+STR+ERY+DOX	CPR+STR+ERY+DOX	AMP+CPR+STR+ERY+DOX
	77.81692858	96.1427749	94.30051813	83.6514224	95.46272956	77.1585093	28.60008745	67.94063939	89.93595844	57.45538284	74.95682211	70.98445596	69.73709461	62.69430052	99.20777818	21.85128983	54.44304416	57.34420411	27.77706335	36.33651398	56.02736555	56.55344464	63.30838611	67.60698522	62.08021493	16.35760397	18.00365217	42.69437514	25.80180551	68.8351564	19.64970037
	70.5988928	99.70674487	98.24046921	82.95433537	98.0403032	60.73477604	31.10216407	51.65576246	72.00805234	60.11730205	75.07331378	70.72947214	65.98240469	61.87683284	103.956369	14.99748364	49.66280825	59.44640161	19.44640161	24.59989935	38.51031706	58.99926686	76.59457478	63.98460411	66.33064516	16.86965274	23.77453447	46.11977856	24.59989935	61.05205279	22.02315048
	67.43884839	97.78660613	92.94362467	90.30262412	99.54116616	71.228213	30.19649928	47.86046454	59.56407505	61.99394627	77.8093076	75.00945895	63.58304956	70.54483541	102.5106975	24.19667197	52.59717031	71.24794928	11.8615007	27.45315719	43.45927543	68.0476731	76.52289065	64.71812334	71.37722285	19.44022993	15.9666457	43.77505582	16.91398685	74.70677261	18.49288877
AMP2CPR3STR3ERY1DOX3	AMP	CPR	STR	ERY	DOX	AMP+CPR	AMP+STR	AMP+ERY	AMP+DOX	CPR+STR	CPR+ERY	CPR+DOX	STR+ERY	STR+DOX	ERY+DOX	AMP+CPR+STR	AMP+CPR+ERY	AMP+CPR+DOX	AMP+STR+ERY	AMP+STR+DOX	AMP+ERY+DOX	CPR+STR+ERY	CPR+STR+DOX	CPR+ERY+DOX	STR+ERY+DOX	AMP+CPR+STR+ERY	AMP+CPR+STR+DOX	AMP+CPR+ERY+DOX	AMP+STR+ERY+DOX	CPR+STR+ERY+DOX	AMP+CPR+STR+ERY+DOX
	77.81692858	96.1427749	94.30051813	83.6514224	101.2243428	77.1585093	28.60008745	67.94063939	84.33939456	57.45538284	74.95682211	65.76472846	69.73709461	70.65822299	98.63161685	21.85128983	54.44304416	58.49643785	27.77706335	38.25004501	58.00262339	56.55344464	52.56188831	64.53655728	62.69430052	16.35760397	42.03595587	45.16344744	28.53836064	73.44079831	24.9170546
	70.5988928	99.70674487	98.24046921	82.95433537	101.2941394	60.73477604	31.10216407	51.65576246	67.17664821	60.11730205	75.07331378	68.38343109	65.98240469	65.68914956	101.885746	14.99748364	49.66280825	52.44086563	19.44640161	33.67891293	42.95923503	58.99926686	49.02859238	67.21041056	57.5329912	16.86965274	21.19778561	55.84297937	43.28132864	69.26319648	32.00805234
	67.43884839	97.78660613	92.94362467	90.30262412	101.8508017	71.228213	30.19649928	47.86046454	54.51158889	61.99394627	77.8093076	72.28528188	63.58304956	77.20393492	102.1807496	24.19667197	52.59717031	58.93251428	11.8615007	27.03869543	48.1959812	68.0476731	61.38857359	68.35035944	68.0476731	19.44022993	33.33456685	53.56424774	12.80884185	79.54975407	30.29518065
AMP2CPR3STR3ERY2DOX1	AMP	CPR	STR	ERY	DOX	AMP+CPR	AMP+STR	AMP+ERY	AMP+DOX	CPR+STR	CPR+ERY	CPR+DOX	STR+ERY	STR+DOX	ERY+DOX	AMP+CPR+STR	AMP+CPR+ERY	AMP+CPR+DOX	AMP+STR+ERY	AMP+STR+DOX	AMP+ERY+DOX	CPR+STR+ERY	CPR+STR+DOX	CPR+ERY+DOX	STR+ERY+DOX	AMP+CPR+STR+ERY	AMP+CPR+STR+DOX	AMP+CPR+ERY+DOX	AMP+STR+ERY+DOX	CPR+STR+ERY+DOX	AMP+CPR+STR+ERY+DOX
	77.81692858	96.1427749	94.30051813	96.90313288	101.2243428	77.1585093	28.60008745	59.31946195	81.04729817	57.45538284	73.11456534	69.14219919	91.23009019	57.16753022	99.7839395	21.85128983	69.52496078	56.02736555	29.8551992	33.97031969	72.32324271	53.79005949	62.08021493	63.92247169	60.54500096	33.64111005	29.09390191	56.418302	40.38990767	64.22951449	23.60021604
	70.5988928	99.70674487	98.24046921	96.56128674	100.9983361	60.73477604	31.10216407	54.81630599	68.34423754	60.11730205	74.48680352	69.26319648	89.14956012	62.22507331	106.0269921	14.99748364	61.70105687	53.97081027	22.60694514	21.37896326	56.48716658	56.94648094	68.09017595	68.38343109	57.82624633	30.13588324	29.10920986	63.25113236	33.94061399	70.14296188	38.44992451
	67.43884839	97.78660613	92.94362467	96.90158272	96.57163479	71.228213	30.19649928	67.123068	51.66956543	61.99394627	85.0737798	71.07453651	92.64093833	67.82065834	99.54116616	24.19667197	57.03783197	70.30060812	22.59803377	19.87442796	52.93268697	65.02080969	68.35035944	75.91751797	66.23155505	28.30181697	20.0717907	54.29448988	15.65086531	75.31214529	16.59820647
AMP2CPR3STR3ERY2DOX2	AMP	CPR	STR	ERY	DOX	AMP+CPR	AMP+STR	AMP+ERY	AMP+DOX	CPR+STR	CPR+ERY	CPR+DOX	STR+ERY	STR+DOX	ERY+DOX	AMP+CPR+STR	AMP+CPR+ERY	AMP+CPR+DOX	AMP+STR+ERY	AMP+STR+DOX	AMP+ERY+DOX	CPR+STR+ERY	CPR+STR+DOX	CPR+ERY+DOX	STR+ERY+DOX	AMP+CPR+STR+ERY	AMP+CPR+STR+DOX	AMP+CPR+ERY+DOX	AMP+STR+ERY+DOX	CPR+STR+ERY+DOX	AMP+CPR+STR+ERY+DOX
	77.81692858	96.1427749	94.30051813	96.90313288	95.46272956	77.1585093	28.60008745	59.31946195	89.93595844	57.45538284	73.11456534	70.98445596	91.23009019	62.69430052	99.20777818	21.85128983	69.52496078	57.34420411	29.8551992	36.33651398	64.09300172	53.79005949	63.30838611	70.06332758	65.45768566	33.64111005	18.00365217	53.22908361	16.02839433	59.31682978	39.62861038
	70.5988928	99.70674487	98.24046921	96.56128674	98.0403032	60.73477604	31.10216407	54.81630599	72.00805234	60.11730205	74.48680352	70.72947214	89.14956012	61.87683284	106.6185986	14.99748364	61.70105687	59.44640161	22.60694514	24.59989935	56.48716658	56.94648094	76.59457478	56.35997067	57.23973607	30.13588324	23.77453447	74.84650226	29.04881731	68.67668622	51.65576246
	67.43884839	97.78660613	92.94362467	96.90158272	99.54116616	71.228213	30.19649928	67.123068	59.56407505	61.99394627	85.0737798	75.00945895	92.64093833	70.54483541	103.1705934	24.19667197	57.03783197	71.24794928	22.59803377	27.45315719	51.03800466	65.02080969	76.52289065	74.10139992	72.28528188	28.30181697	15.9666457	50.40644389	6.197190048	74.70677261	11.22993993
AMP2CPR3STR3ERY2DOX3	AMP	CPR	STR	ERY	DOX	AMP+CPR	AMP+STR	AMP+ERY	AMP+DOX	CPR+STR	CPR+ERY	CPR+DOX	STR+ERY	STR+DOX	ERY+DOX	AMP+CPR+STR	AMP+CPR+ERY	AMP+CPR+DOX	AMP+STR+ERY	AMP+STR+DOX	AMP+ERY+DOX	CPR+STR+ERY	CPR+STR+DOX	CPR+ERY+DOX	STR+ERY+DOX	AMP+CPR+STR+ERY	AMP+CPR+STR+DOX	AMP+CPR+ERY+DOX	AMP+STR+ERY+DOX	CPR+STR+ERY+DOX	AMP+CPR+STR+ERY+DOX
	77.81692858	96.1427749	94.30051813	96.90313288	101.2243428	77.1585093	28.60008745	59.31946195	84.33939456	57.45538284	73.11456534	65.76472846	91.23009019	70.65822299	102.0885848	21.85128983	69.52496078	58.49643785	29.8551992	38.25004501	58.39355984	53.79005949	52.56188831	74.0548839	71.29149875	33.64111005	42.03595587	73.20799362	23.60021604	49.18441758	44.56675497
	70.5988928	99.70674487	98.24046921	96.56128674	101.2941394	60.73477604	31.10216407	54.81630599	67.17664821	60.11730205	74.48680352	68.38343109	89.14956012	65.68914956	110.7598447	14.99748364	61.70105687	52.44086563	22.60694514	33.67891293	58.74182184	56.94648094	49.02859238	54.60043988	58.65102639	30.13588324	21.19778561	66.47206844	24.8616004	53.72067449	32.33014595
	67.43884839	97.78660613	92.94362467	96.90158272	101.8508017	71.228213	30.19649928	67.123068	54.51158889	61.99394627	85.0737798	72.28528188	92.64093833	77.20393492	95.91173893	24.19667197	57.03783197	58.93251428	22.59803377	27.03869543	51.66956543	65.02080969	61.38857359	82.57661748	67.13961407	28.30181697	33.33456685	66.80728762	24.82823274	57.7563375	21.65069262
AMP2CPR3STR3ERY3DOX1	AMP	CPR	STR	ERY	DOX	AMP+CPR	AMP+STR	AMP+ERY	AMP+DOX	CPR+STR	CPR+ERY	CPR+DOX	STR+ERY	STR+DOX	ERY+DOX	AMP+CPR+STR	AMP+CPR+ERY	AMP+CPR+DOX	AMP+STR+ERY	AMP+STR+DOX	AMP+ERY+DOX	CPR+STR+ERY	CPR+STR+DOX	CPR+ERY+DOX	STR+ERY+DOX	AMP+CPR+STR+ERY	AMP+CPR+STR+DOX	AMP+CPR+ERY+DOX	AMP+STR+ERY+DOX	CPR+STR+ERY+DOX	AMP+CPR+STR+ERY+DOX
	77.81692858	96.1427749	94.30051813	93.44616493	101.2243428	77.1585093	28.60008745	86.64386204	81.04729817	57.45538284	72.80752255	69.14219919	81.09767799	57.16753022	97.4792942	21.85128983	74.79231501	56.02736555	51.4184306	33.97031969	64.97775263	53.79005949	62.08021493	60.23795817	81.73095375	40.55451249	29.09390191	90.65610452	32.3859983	55.93935905	54.7722538

	70.5988928	99.70674487	98.24046921	94.49066371	100.9983361	60.73477604	31.10216407	74.90689482	68.34423754	60.11730205	75.65982405	69.26319648	81.23167155	62.22507331	107.8018118	14.99748364	64.27780574	53.97081027	29.81378963	21.37896326	54.55460493	61.05205279	68.09017595	71.60923754	64.27785924	29.49169602	29.10920986	67.43834927	21.37896326	57.82624633	65.82788123
	67.43884839	97.78660613	92.94362467	96.59225653	96.57163479	71.228213	30.19649928	64.39946219	51.66956543	61.99394627	81.74423004	71.07453651	82.04691638	67.82065834	95.91173893	24.19667197	70.08350911	70.30060812	30.19649928	19.87442796	59.87985543	62.59931896	68.35035944	94.98675747	77.43094968	39.66991082	20.0717907	65.54416608	38.50547065	72.58796822	36.4923707
AMP2CPR3STR3ERY3DOX2	AMP	CPR	STR	ERY	DOX	AMP+CPR	AMP+STR	AMP+ERY	AMP+DOX	CPR+STR	CPR+ERY	CPR+DOX	STR+ERY	STR+DOX	ERY+DOX	AMP+CPR+STR	AMP+CPR+ERY	AMP+CPR+DOX	AMP+STR+ERY	AMP+STR+DOX	AMP+ERY+DOX	CPR+STR+ERY	CPR+STR+DOX	CPR+ERY+DOX	STR+ERY+DOX	AMP+CPR+STR+ERY	AMP+CPR+STR+DOX	AMP+CPR+ERY+DOX	AMP+STR+ERY+DOX	CPR+STR+ERY+DOX	AMP+CPR+STR+ERY+DOX
	77.81692858	96.1427749	94.30051813	93.44616493	95.46272956	77.1585093	28.60008745	86.64386204	89.93595844	57.45538284	72.80752255	70.98445596	81.09767799	62.69430052	97.4792942	21.85128983	74.79231501	57.34420411	51.4184306	36.33651398	70.5743165	53.79005949	63.30838611	74.03569372	73.44079831	40.55451249	18.00365217	84.73033101	33.70283686	48.2632892	42.59149713
	70.5988928	99.70674487	98.24046921	94.49066371	98.0403032	60.73477604	31.10216407	74.90689482	72.00805234	60.11730205	75.65982405	70.72947214	81.23167155	61.87683284	108.0976151	14.99748364	64.27780574	59.44640161	29.81378963	24.59989935	77.74534474	61.05205279	76.59457478	71.02272727	64.80938416	29.49169602	23.77453447	80.96628083	44.89179668	64.57111437	44.24760946
	67.43884839	97.78660613	92.94362467	96.59225653	99.54116616	71.228213	30.19649928	64.39946219	59.56407505	61.99394627	81.74423004	75.00945895	82.04691638	70.54483541	103.8304893	24.19667197	70.08350911	71.24794928	30.19649928	27.45315719	60.19563582	62.59931896	76.52289065	78.03632236	78.71736663	39.66991082	15.9666457	63.96526416	35.24898543	69.56110481	42.492198
AMP2CPR3STR3ERY3DOX3	AMP	CPR	STR	ERY	DOX	AMP+CPR	AMP+STR	AMP+ERY	AMP+DOX	CPR+STR	CPR+ERY	CPR+DOX	STR+ERY	STR+DOX	ERY+DOX	AMP+CPR+STR	AMP+CPR+ERY	AMP+CPR+DOX	AMP+STR+ERY	AMP+STR+DOX	AMP+ERY+DOX	CPR+STR+ERY	CPR+STR+DOX	CPR+ERY+DOX	STR+ERY+DOX	AMP+CPR+STR+ERY	AMP+CPR+STR+DOX	AMP+CPR+ERY+DOX	AMP+STR+ERY+DOX	CPR+STR+ERY+DOX	AMP+CPR+STR+ERY+DOX
	77.81692858	96.1427749	94.30051813	93.44616493	101.2243428	77.1585093	28.60008745	86.64386204	84.33939456	57.45538284	72.80752255	65.76472846	81.09767799	70.65822299	102.9528268	21.85128983	74.79231501	58.49643785	51.4184306	38.25004501	65.90365474	53.79005949	52.56188831	82.34503934	65.76472846	40.55451249	42.03595587	83.74270209	32.65348113	56.24640184	18.55919344
	70.5988928	99.70674487	98.24046921	94.49066371	101.2941394	60.73477604	31.10216407	74.90689482	67.17664821	60.11730205	75.65982405	68.38343109	81.23167155	65.68914956	108.985025	14.99748364	64.27780574	52.44086563	29.81378963	33.67891293	74.84650226	61.05205279	49.02859238	78.06085044	58.35777126	29.49169602	21.19778561	96.42677403	27.82083543	71.02272727	20.09058883
	67.43884839	97.78660613	92.94362467	96.59225653	101.8508017	71.228213	30.19649928	64.39946219	54.51158889	61.99394627	81.74423004	72.28528188	82.04691638	77.20393492	113.3989792	24.19667197	70.08350911	58.93251428	30.19649928	27.03869543	75.03731389	62.59931896	61.38857359	84.69542187	77.20393492	39.66991082	33.33456685	84.49098916	46.30129889	72.58796822	21.01913185
AMP3CPR1STR1ERY1DOX1	AMP	CPR	STR	ERY	DOX	AMP+CPR	AMP+STR	AMP+ERY	AMP+DOX	CPR+STR	CPR+ERY	CPR+DOX	STR+ERY	STR+DOX	ERY+DOX	AMP+CPR+STR	AMP+CPR+ERY	AMP+CPR+DOX	AMP+STR+ERY	AMP+STR+DOX	AMP+ERY+DOX	CPR+STR+ERY	CPR+STR+DOX	CPR+ERY+DOX	STR+ERY+DOX	AMP+CPR+STR+ERY	AMP+CPR+STR+DOX	AMP+CPR+ERY+DOX	AMP+STR+ERY+DOX	CPR+STR+ERY+DOX	AMP+CPR+STR+ERY+DOX
	74.34273652	91.42960029	92.00576161	83.6514224	101.2243428	74.64977931	16.9449242	44.86662829	61.77317214	75.87324451	86.82030969	100.3601008	78.46597047	96.03889089	95.46272956	51.92861255	52.23565534	57.47457302	24.92803685	32.41220495	38.13087699	80.77061577	69.24738927	76.7374865	71.84011523	51.92861255	44.55958549	42.12243331	14.64210324	45.01260353	47.63001343
	69.79472141	99.22351636	85.0249584	82.95433537	100.9983361	68.91495601	22.34237537	43.40175953	63.69134897	90.64522093	84.72915511	100.4067295	79.40469588	92.71584396	95.67387687	59.53079179	56.01173021	59.29252199	19.99633431	22.92888563	52.84090909	88.2787946	76.15085968	88.57459789	82.36272879	45.16129032	49.61510264	47.56231672	17.35703812	49.56553892	44.86803519
	71.37722285	104.4903851	94.26199928	90.30262412	96.57163479	71.45289444	22.64472191	56.84827847	67.13961407	81.06408207	94.59194721	108.7797082	84.36356138	93.60210342	93.27215549	63.88573591	51.09723799	61.08588725	23.85546727	31.19561105	49.28111994	83.37371759	67.53621694	75.12501933	77.43465484	43.60575104	48.9784336	48.9784336	28.77412032	42.56328298	50.56753689
AMP3CPR1STR1ERY1DOX2	AMP	CPR	STR	ERY	DOX	AMP+CPR	AMP+STR	AMP+ERY	AMP+DOX	CPR+STR	CPR+ERY	CPR+DOX	STR+ERY	STR+DOX	ERY+DOX	AMP+CPR+STR	AMP+CPR+ERY	AMP+CPR+DOX	AMP+STR+ERY	AMP+STR+DOX	AMP+ERY+DOX	CPR+STR+ERY	CPR+STR+DOX	CPR+ERY+DOX	STR+ERY+DOX	AMP+CPR+STR+ERY	AMP+CPR+STR+DOX	AMP+CPR+ERY+DOX	AMP+STR+ERY+DOX	CPR+STR+ERY+DOX	AMP+CPR+STR+ERY+DOX
	74.34273652	91.42960029	92.00576161	83.6514224	95.46272956	74.64977931	16.9449242	44.86662829	55.93935905	75.87324451	86.82030969	80.19445445	78.46597047	98.91969752	99.20777818	51.92861255	52.23565534	58.3957014	24.92803685	10.61216657	45.49990405	80.77061577	52.25063018	81.92293842	81.3467771	51.92861255	45.48071387	44.57877567	10.30512378	47.64133958	60.52581078
	69.79472141	99.22351636	85.0249584	82.95433537	98.0403032	68.91495601	22.34237537	43.40175953	78.94061584	90.64522093	84.72915511	87.68718802	79.40469588	92.71584396	103.956369	59.53079179	56.01173021	66.62390029	19.99633431	6.50659824	53.72067449	88.2787946	50.71177667	90.64522093	89.16620447	45.16129032	51.0813783	47.56231672	18.07184751	53.66980958	49.85337243
	71.37722285	104.4903851	94.26199928	90.30262412	99.54116616	71.45289444	22.64472191	56.84827847	75.31214529	81.06408207	94.59194721	110.4294479	84.36356138	95.91173893	102.5106975	63.88573591	51.09723799	59.87514188	23.85546727	12.48581158	58.96708286	83.37371759	46.52265814	63.57684178	74.13517554	43.60575104	51.47559591	49.88649262	19.20544884	59.28751869	52.98902762
AMP3CPR1STR1ERY1DOX3	AMP	CPR	STR	ERY	DOX	AMP+CPR	AMP+STR	AMP+ERY	AMP+DOX	CPR+STR	CPR+ERY	CPR+DOX	STR+ERY	STR+DOX	ERY+DOX	AMP+CPR+STR	AMP+CPR+ERY	AMP+CPR+DOX	AMP+STR+ERY	AMP+STR+DOX	AMP+ERY+DOX	CPR+STR+ERY	CPR+STR+DOX	CPR+ERY+DOX	STR+ERY+DOX	AMP+CPR+STR+ERY	AMP+CPR+STR+DOX	AMP+CPR+ERY+DOX	AMP+STR+ERY+DOX	CPR+STR+ERY+DOX	AMP+CPR+STR+ERY+DOX
	74.34273652	91.42960029	92.00576161	83.6514224	101.2243428	74.64977931	16.9449242	44.86662829	46.11398964	75.87324451	86.82030969	74.14476053	78.46597047	81.63485776	98.63161685	51.92861255	52.23565534	59.00978699	24.92803685	8.501247361	36.26943005	80.77061577	44.18437162	61.18113072	75.29708318	51.92861255	54.99904049	42.71732873	10.03646133	51.06229744	41.50834773
	69.79472141	99.22351636	85.0249584	82.95433537	101.2941394	68.91495601	22.34237537	43.40175953	63.98460411	90.64522093	84.72915511	90.64522093	79.40469588	99.51931965	101.885746	59.53079179	56.01173021	67.79692082	19.99633431	25.40322581	54.30718475	88.2787946	71.71381032	81.17951562	77.33407284	45.16129032	59.23753666	42.28372434	31.56158358	80.92068774	43.16348974
	71.37722285	104.4903851	94.26199928	90.30262412	101.8508017	71.45289444	22.64472191	56.84827847	76.825577	81.06408207	94.59194721	93.60210342	84.36356138	104.1604372	102.1807496	63.88573591	51.09723799	54.72947408	23.85546727	29.9091941	66.83692773	83.37371759	71.82554003	73.80522761	75.78491519	43.60575104	60.55618615	48.37306092	19.14491109	41.57343919	39.6708286
AMP3CPR1STR1ERY2DOX1	AMP	CPR	STR	ERY	DOX	AMP+CPR	AMP+STR	AMP+ERY	AMP+DOX	CPR+STR	CPR+ERY	CPR+DOX	STR+ERY	STR+DOX	ERY+DOX	AMP+CPR+STR	AMP+CPR+ERY	AMP+CPR+DOX	AMP+STR+ERY	AMP+STR+DOX	AMP+ERY+DOX	CPR+STR+ERY	CPR+STR+DOX	CPR+ERY+DOX	STR+ERY+DOX	AMP+CPR+STR+ERY	AMP+CPR+STR+DOX	AMP+CPR+ERY+DOX	AMP+STR+ERY+DOX	CPR+STR+ERY+DOX	AMP+CPR+STR+ERY+DOX
	74.34273652	91.42960029	92.00576161	96.90313288	101.2243428	74.64977931	16.9449242	47.9562464	61.77317214	75.87324451	88.54879366	100.3601008	91.42960029	96.03889089	99.7839395	51.92861255	46.70888505	57.47457302	17.86605258	32.41220495	49.4722702	69.24738927	69.24738927	60.60496939	69.24738927	48.24409902	44.55958549	58.37651123	13.56745346	59.99279798	62.98215314
	69.79472141	99.22351636	85.0249584	96.56128674	100.9983361	68.91495601	22.34237537	47.85557185	63.69134897	90.64522093	93.30745055	100.4067295	88.87040118	92.71584396	106.0269921	59.53079179	51.31964809	59.29252199	34.07258065	22.92888563	46.38929619	79.10889259	76.15085968	87.98299131	90.94102422	51.31964809	49.61510264	51.61290323	21.29765396	69.97596598	51.31964809
	71.37722285	104.4903851	94.26199928	96.90158272	96.57163479	71.45289444	22.64472191	65.62618237	67.13961407	81.06408207	96.90158272	108.7797082	91.62241584	93.60210342	99.54116616	63.88573591	56.84827847	61.08588725	37.47635263	31.19561105	57.45365115	80.07423828	67.53621694	80.73413414	84.69350931	47.54067348	48.9784336	52.00529701	18.48278471	53.01850802	57.52932274
AMP3CPR1STR1ERY2DOX2	AMP	CPR	STR	ERY	DOX	AMP+CPR	AMP+STR	AMP+ERY	AMP+DOX	CPR+STR	CPR+ERY	CPR+DOX	STR+ERY	STR+DOX	ERY+DOX	AMP+CPR+STR	AMP+CPR+ERY	AMP+CPR+DOX	AMP+STR+ERY	AMP+STR+DOX	AMP+ERY+DOX	CPR+STR+ERY	CPR+STR+DOX	CPR+ERY+DOX	STR+ERY+DOX	AMP+CPR+STR+ERY	AMP+CPR+STR+DOX	AMP+CPR+ERY+DOX	AMP+STR+ERY+DOX	CPR+STR+ERY+DOX	AMP+CPR+STR+ERY+DOX
	74.34273652	91.42960029	92.00576161	96.90313288	95.46272956	74.64977931	16.9449242	47.9562464	55.93935905	75.87324451	88.54879366	80.19445445	91.42960029	98.91969752	99.20777818	51.92861255	46.70888505	58.3957014	17.86605258	10.61216657	51.62156976	69.24738927	52.25063018	69.24738927	74.4328412	48.24409902	45.48071387	68.8351564	20.01535214	61.4332013	55.30608329
	69.79472141	99.22351636	85.0249584	96.56128674	98.0403032	68.91495601	22.34237537	47.85557185	78.94061584	90.64522093	93.30745055	87.68718802	88.87040118	92.71584396	106.6185986	59.53079179	51.31964809	66.62390029	34.07258065	6.50659824	48.38709677	79.10889259	50.71177667	84.43335182	89.75781106	51.31964809	51.0813783	60.70381232	14.84604106	71.45498244	48.97360704

[illegible]

AMP3CPR2STR3ERY1DOX1	AMP	CPR	STR	ERY	DOX	AMP+CPR	AMP+STR	AMP+ERY	AMP+DOX	CPR+STR	CPR+ERY	CPR+DOX	STR+ERY	STR+DOX	ERY+DOX	AMP+CPR+STR	AMP+CPR+ERY	AMP+CPR+DOX	AMP+STR+ERY	AMP+STR+DOX	AMP+ERY+DOX	CPR+STR+ERY	CPR+STR+DOX	CPR+ERY+DOX	STR+ERY+DOX	AMP+CPR+STR+ERY	AMP+CPR+STR+DOX	AMP+CPR+ERY+DOX	AMP+STR+ERY+DOX	CPR+STR+ERY+DOX	AMP+CPR+STR+ERY+DOX
	74.34273652	94.93582984	94.30051813	83.6514224	101.2243428	67.89483784	47.63001343	44.86662829	61.77317214	77.48771894	86.70558885	90.32689488	69.73709461	57.16753022	95.46272956	48.85818461	46.57455383	54.6919977	29.53367876	28.30550758	38.13087699	81.10902497	94.93582984	71.89115506	61.15908655	31.99002111	24.00690846	40.87507196	28.30550758	46.54201281	29.53367876
	69.79472141	92.29994967	98.24046921	82.95433537	100.9983361	65.1026393	41.05571848	43.40175953	63.69134897	72.91394061	75.81278309	90.30699547	65.98240469	62.22507331	95.67387687	39.29618768	42.81524927	50.91642229	41.40395894	60.75879765	52.84090909	72.91394061	74.52440866	104.4791142	67.50366569	35.24560117	32.60630499	44.57478006	56.65322581	73.55812783	27.32771261
	71.37722285	88.28035377	92.94362467	90.30262412	96.57163479	59.34544079	36.34127885	56.84827847	67.13961407	78.17538147	72.80711493	84.80676954	63.58304956	67.82065834	93.27215549	39.59515702	49.58380628	50.87022323	39.59515702	54.1241014	49.28111994	71.85977377	77.22804031	88.28035377	68.35035944	48.67574726	38.98978434	38.1573969	43.22739311	93.64862031	34.75217556

AMP3CPR2STR3ERY1DOX2	AMP	CPR	STR	ERY	DOX	AMP+CPR	AMP+STR	AMP+ERY	AMP+DOX	CPR+STR	CPR+ERY	CPR+DOX	STR+ERY	STR+DOX	ERY+DOX	AMP+CPR+STR	AMP+CPR+ERY	AMP+CPR+DOX	AMP+STR+ERY	AMP+STR+DOX	AMP+ERY+DOX	CPR+STR+ERY	CPR+STR+DOX	CPR+ERY+DOX	STR+ERY+DOX	AMP+CPR+STR+ERY	AMP+CPR+STR+DOX	AMP+CPR+ERY+DOX	AMP+STR+ERY+DOX	CPR+STR+ERY+DOX	AMP+CPR+STR+ERY+DOX
	74.34273652	94.93582984	94.30051813	83.6514224	95.46272956	67.89483784	47.63001343	44.86662829	55.93935905	77.48771894	86.70558885	105.1413287	69.73709461	62.69430052	99.20777818	48.85818461	46.57455383	54.40414508	29.53367876	27.0773364	45.49990405	81.10902497	76.17088038	63.99012371	62.08021493	31.99002111	21.24352332	39.20552677	14.18153905	49.83410921	31.06889273
	69.79472141	92.29994967	98.24046921	82.95433537	98.0403032	65.1026393	41.05571848	43.40175953	78.94061584	72.91394061	75.81278309	86.76396578	65.98240469	61.87683284	103.956369	39.29618768	42.81524927	48.38709677	41.40395894	58.11950147	53.72067449	72.91394061	89.98490186	91.91746351	66.33064516	35.24560117	33.77932551	49.85337243	38.47140762	76.1348767	19.99633431
	71.37722285	88.28035377	92.94362467	90.30262412	99.54116616	59.34544079	36.34127885	56.84827847	75.31214529	78.17538147	72.80711493	86.38567147	63.58304956	70.54483541	102.5106975	39.59515702	49.58380628	49.05410518	39.59515702	57.15096481	58.96708286	71.85977377	81.96474608	96.17486339	71.37722285	48.67574726	38.38441165	43.90843738	39.29247068	86.38567147	32.63337117

AMP3CPR2STR3ERY1DOX3	AMP	CPR	STR	ERY	DOX	AMP+CPR	AMP+STR	AMP+ERY	AMP+DOX	CPR+STR	CPR+ERY	CPR+DOX	STR+ERY	STR+DOX	ERY+DOX	AMP+CPR+STR	AMP+CPR+ERY	AMP+CPR+DOX	AMP+STR+ERY	AMP+STR+DOX	AMP+ERY+DOX	CPR+STR+ERY	CPR+STR+DOX	CPR+ERY+DOX	STR+ERY+DOX	AMP+CPR+STR+ERY	AMP+CPR+STR+DOX	AMP+CPR+ERY+DOX	AMP+STR+ERY+DOX	CPR+STR+ERY+DOX	AMP+CPR+STR+ERY+DOX
	74.34273652	94.93582984	94.30051813	83.6514224	101.2243428	67.89483784	47.63001343	44.86662829	46.11398964	77.48771894	86.70558885	91.97294308	69.73709461	70.65822299	98.63161685	48.85818461	46.57455383	52.54269814	29.53367876	18.32661677	36.26943005	81.10902497	57.07672128	60.63630051	62.69430052	31.99002111	24.29476108	32.27787373	16.6378814	61.68565623	31.68297832
	69.79472141	92.29994967	98.24046921	82.95433537	101.2941394	65.1026393	41.05571848	43.40175953	63.98460411	72.91394061	75.81278309	87.08605939	65.98240469	65.68914956	101.885746	39.29618768	42.81524927	58.06451613	41.40395894	41.69721408	54.30718475	72.91394061	76.1348767	79.67790639	57.5329912	35.24560117	24.34017595	43.1085044	35.24560117	69.69300453	29.67375367
	71.37722285	88.28035377	92.94362467	90.30262412	101.8508017	59.34544079	36.34127885	56.84827847	76.825577	78.17538147	72.80711493	93.33283993	63.58304956	77.20393492	102.1807496	39.59515702	49.58380628	53.29171396	39.59515702	41.71396141	66.83692773	71.85977377	78.80694223	93.33283993	68.0476731	48.67574726	23.55278093	37.17366629	45.3461975	64.28104454	33.23874385

AMP3CPR2STR3ERY2DOX1	AMP	CPR	STR	ERY	DOX	AMP+CPR	AMP+STR	AMP+ERY	AMP+DOX	CPR+STR	CPR+ERY	CPR+DOX	STR+ERY	STR+DOX	ERY+DOX	AMP+CPR+STR	AMP+CPR+ERY	AMP+CPR+DOX	AMP+STR+ERY	AMP+STR+DOX	AMP+ERY+DOX	CPR+STR+ERY	CPR+STR+DOX	CPR+ERY+DOX	STR+ERY+DOX	AMP+CPR+STR+ERY	AMP+CPR+STR+DOX	AMP+CPR+ERY+DOX	AMP+STR+ERY+DOX	CPR+STR+ERY+DOX	AMP+CPR+STR+ERY+DOX
	74.34273652	94.93582984	94.30051813	96.90313288	101.2243428	67.89483784	47.63001343	47.9562464	61.77317214	77.48771894	89.66847561	90.32689488	91.23009019	57.16753022	99.7839395	48.85818461	56.84129726	54.6919977	28.91959317	28.30550758	49.4722702	80.45060569	94.93582984	53.55829325	60.54500096	37.19055843	24.00690846	44.55958549	19.20936481	69.58668759	29.53367876
	69.79472141	92.29994967	98.24046921	96.56128674	100.9983361	65.1026393	41.05571848	47.85557185	63.69134897	72.91394061	77.10115752	90.30699547	89.14956012	62.22507331	106.0269921	39.29618768	50.62316716	50.91642229	34.65909091	60.75879765	46.38929619	68.7267237	74.52440866	72.91394061	57.82624633	32.89956012	32.60630499	46.62756598	26.15469208	75.81278309	29.0872434
	71.37722285	88.28035377	92.94362467	96.90158272	96.57163479	59.34544079	36.34127885	65.62618237	67.13961407	78.17538147	83.543648	84.80676954	92.64093833	67.82065834	99.54116616	39.59515702	61.99394627	50.87022323	38.08172531	54.1241014	57.45365115	73.4386757	77.22804031	80.70162454	66.23155505	41.41127507	38.98978434	48.14604616	37.47635263	62.38636223	38.68709799

AMP3CPR2STR3ERY2DOX2	AMP	CPR	STR	ERY	DOX	AMP+CPR	AMP+STR	AMP+ERY	AMP+DOX	CPR+STR	CPR+ERY	CPR+DOX	STR+ERY	STR+DOX	ERY+DOX	AMP+CPR+STR	AMP+CPR+ERY	AMP+CPR+DOX	AMP+STR+ERY	AMP+STR+DOX	AMP+ERY+DOX	CPR+STR+ERY	CPR+STR+DOX	CPR+ERY+DOX	STR+ERY+DOX	AMP+CPR+STR+ERY	AMP+CPR+STR+DOX	AMP+CPR+ERY+DOX	AMP+STR+ERY+DOX	CPR+STR+ERY+DOX	AMP+CPR+STR+ERY+DOX
	74.34273652	94.93582984	94.30051813	96.90313288	95.46272956	67.89483784	47.63001343	47.9562464	55.93935905	77.48771894	89.66847561	105.1413287	91.23009019	62.69430052	99.20777818	48.85818461	56.84129726	54.40414508	28.91959317	27.0773364	51.62156976	80.45060569	76.17088038	65.08063064	65.45768566	37.19055843	21.24352332	47.32297064	11.8787181	60.03960803	37.5167914
	69.79472141	92.29994967	98.24046921	96.56128674	98.0403032	65.1026393	41.05571848	47.85557185	78.94061584	72.91394061	77.10115752	86.76396578	89.14956012	61.87683284	106.6185986	39.29618768	50.62316716	48.38709677	34.65909091	58.11950147	48.38709677	68.7267237	89.98490186	59.70810267	57.23973607	32.89956012	33.77932551	51.90615836	23.51539589	96.74886764	31.14002933
	71.37722285	88.28035377	92.94362467	96.90158272	99.54116616	59.34544079	36.34127885	65.62618237	75.31214529	78.17538147	83.543648	86.38567147	92.64093833	70.54483541	103.1705934	39.59515702	61.99394627	49.05410518	38.08172531	57.15096481	44.51381006	73.4386757	81.96474608	69.64931108	72.28528188	41.41127507	38.38441165	54.42678774	20.52591752	55.12341339	45.64888384

AMP3CPR2STR3ERY2DOX3	AMP	CPR	STR	ERY	DOX	AMP+CPR	AMP+STR	AMP+ERY	AMP+DOX	CPR+STR	CPR+ERY	CPR+DOX	STR+ERY	STR+DOX	ERY+DOX	AMP+CPR+STR	AMP+CPR+ERY	AMP+CPR+DOX	AMP+STR+ERY	AMP+STR+DOX	AMP+ERY+DOX	CPR+STR+ERY	CPR+STR+DOX	CPR+ERY+DOX	STR+ERY+DOX	AMP+CPR+STR+ERY	AMP+CPR+STR+DOX	AMP+CPR+ERY+DOX	AMP+STR+ERY+DOX	CPR+STR+ERY+DOX	AMP+CPR+STR+ERY+DOX
	74.34273652	94.93582984	94.30051813	96.90313288	101.2243428	67.89483784	47.63001343	47.9562464	46.11398964	77.48771894	89.66847561	91.97294308	91.23009019	70.65822299	102.0885848	48.85818461	56.84129726	52.54269814	28.91959317	18.32661677	56.84129726	80.45060569	57.07672128	75.12152465	71.29149875	37.19055843	24.29476108	52.54269814	17.53981961	51.58303542	44.88581846
	69.79472141	92.29994967	98.24046921	96.56128674	101.2941394	65.1026393	41.05571848	47.85557185	63.98460411	72.91394061	77.10115752	87.08605939	89.14956012	65.68914956	110.7598447	39.29618768	50.62316716	58.06451613	34.65909091	41.69721408	61.58357771	68.7267237	76.1348767	86.76396578	58.65102639	32.89956012	24.34017595	58.65102639	26.39296188	95.1383996	39.64442815
	71.37722285	88.28035377	92.94362467	96.90158272	101.8508017	59.34544079	36.34127885	65.62618237	76.825577	78.17538147	83.543648	93.33283993	92.64093833	77.20393492	95.91173893	39.59515702	61.99394627	53.29171396	38.08172531	41.71396141	55.10783201	73.4386757	78.80694223	65.54416608	67.13961407	41.41127507	23.55278093	75.61483163	35.73590617	43.24217642	51.70261067

AMP3CPR2STR3ERY3DOX1	AMP	CPR	STR	ERY	DOX	AMP+CPR	AMP+STR	AMP+ERY	AMP+DOX	CPR+STR	CPR+ERY	CPR+DOX	STR+ERY	STR+DOX	ERY+DOX	AMP+CPR+STR	AMP+CPR+ERY	AMP+CPR+DOX	AMP+STR+ERY	AMP+STR+DOX	AMP+ERY+DOX	CPR+STR+ERY	CPR+STR+DOX	CPR+ERY+DOX	STR+ERY+DOX	AMP+CPR+STR+ERY	AMP+CPR+STR+DOX	AMP+CPR+ERY+DOX	AMP+STR+ERY+DOX	CPR+STR+ERY+DOX	AMP+CPR+STR+ERY+DOX
	74.34273652	94.93582984	94.30051813	93.44616493	101.2243428	67.89483784	47.63001343	64.53655728	61.77317214	77.48771894	93.28978164	90.32689488	81.09767799	57.16753022	97.4792942	48.85818461	64.21032431	54.6919977	27.0773364	28.30550758	70.65822299	80.77981533	94.93582984	72.81705717	81.73095375	32.27787373	24.00690846	73.74784111	24.90884667	63.00249479	46.42103243
	69.79472141	92.29994967	98.24046921	94.49066371	100.9983361	65.1026393	41.05571848	75.12829912	63.69134897	72.91394061	78.06743835	90.30699547	81.23167155	62.22507331	107.8018118	39.29618768	56.8914956	50.91642229	34.95234604	60.75879765	69.50146628	65.50578762	74.52440866	92.88374434	64.27785924	20.52785924	32.60630499	56.8914956	30.20527859	98.68142929	41.99046921
	71.37722285	88.28035377	92.94362467	96.59225653	96.57163479	59.34544079	36.34127885	72.89065456	67.13961407	78.17538147	80.72136082	84.80676954	82.04691638	67.82065834	95.91173893	39.59515702	49.58380628	50.87022323	37.77903897	54.1241014	81.13885736	65.54416608	77.22804031	57.0180957	77.43094968	27.18501703	38.98978434	71.07453651	36.94665153	45.65000185	49.58380628

AMP3CPR2STR3ERY3DOX2	AMP	CPR	STR	ERY	DOX	AMP+CPR	AMP+STR	AMP+ERY	AMP+DOX	CPR+STR	CPR+ERY	CPR+DOX	STR+ERY	STR+DOX	ERY+DOX	AMP+CPR+STR	AMP+CPR+ERY	AMP+CPR+DOX	AMP+STR+ERY	AMP+STR+DOX	AMP+ERY+DOX	CPR+STR+ERY	CPR+STR+DOX	CPR+ERY+DOX	STR+ERY+DOX	AMP+CPR+STR+ERY	AMP+CPR+STR+DOX	AMP+CPR+ERY+DOX	AMP+STR+ERY+DOX	CPR+STR+ERY+DOX	AMP+CPR+STR+ERY+DOX
	74.34273652	94.93582984	94.30051813	93.44616493	95.46272956	67.89483784	47.63001343	64.53655728	55.93935905	77.48771894	93.28978164	105.1413287	81.09767799	62.69430052	97.4792942	48.85818461	64.21032431	54.40414508	27.0773364	27.0773364	76.18499328	80.77981533	76.17088038	72.81705717	73.44079831	32.27787373	21.24352332	75.28305508	42.41028593	64.31933335	37.8238342
	69.79472141	92.29994967	98.24046921	94.49066371	98.0403032	65.1026393	41.05571848	75.12829912	78.94061584	72.91394061	78.06743835	86.76396578	81.23167155	61.87683284	108.0976151	39.29618768	56.8914956	48.38709677	34.95234604	58.11950147	73.02052786	65.50578762	89.98490186	72.2697534	64.80938416	20.52785924	33.77932551	55.18695015	47.80058651	68.4046301	43.16348974
	71.37722285	88.28035377	92.94362467	96.59225653	99.54116616	59.34544079	36.34127885	72.89065456	75.31214529	78.17538147	80.72136082	86.38567147	82.04691638	70.54483541	103.8304893	39.59515702	49.58380628	49.05410518	37.77903897	57.15096481	70.84752176	65.54416608	81.96474608	73.77419235	78.71736663	27.18501703	38.38441165	68.65304578	50.87022323	55.45893005	47.46500189

AMP3CPR2STR3ERY3DOX3	AMP	CPR	STR	ERY	DOX	AMP+CPR	AMP+STR	AMP+ERY	AMP+DOX	CPR+STR	CPR+ERY	CPR+DOX	STR+ERY	STR+DOX	ERY+DOX	AMP+CPR+STR	AMP+CPR+ERY	AMP+CPR+DOX	AMP+STR+ERY	AMP+STR+DOX	AMP+ERY+DOX	CPR+STR+ERY	CPR+STR+DOX	CPR+ERY+DOX	STR+ERY+DOX	AMP+CPR+STR+ERY	AMP+CPR+STR+DOX	AMP+CPR+ERY+DOX	AMP+STR+ERY+DOX	CPR+STR+ERY+DOX	AMP+CPR+STR+ERY+DOX
	74.34273652	94.93582984	94.30051813	93.44616493	101.2243428	67.89483784	47.63001343	64.53655728	46.11398964	77.48771894	93.28978164	91.97294308	81.09767799	70.65822299	102.9528268	48.85818461	64.21032431	52.54269814	27.0773364	18.32661677	84.47514872	80.77981533	57.07672128	89.6067488	65.76472846	32.27787373	24.29476108	75.28305508	53.77086931	57.73514056	42.42947611
	69.79472141	92.29994967	98.24046921	94.49066371	101.2941394	65.1026393	41.05571848	75.12829912	63.98460411	72.91394061	78.06743835	87.08605939	81.23167155	65.68914956	108.985025	39.29618768	56.8914956	58.06451613	34.95234604	41.69721408	70.38123167	65.50578762	76.1348767	79.09411173	58.35777126	20.52785924	24.34017595	77.47434018	40.17595308	78.38953196	38.47140762
	71.37722285	88.28035377	92.94362467	96.59225653	101.8508017	59.34544079	36.34127885	72.89065456	76.825577	78.17538147	80.72136082	93.33283993	82.04691638	77.20393492	113.3989792	39.59515702	49.58380628	53.29171396	37.77903897	41.71396141	75.61483163	65.54416608	78.80694223	86.08962735	77.20393492	27.18501703	23.55278093	85.90616723	42.31933409	61.43902108	46.55694287

AMP3CPR3STR1ERY1DOX1	AMP	CPR	STR	ERY	DOX	AMP+CPR	AMP+STR	AMP+ERY	AMP+DOX	CPR+STR	CPR+ERY	CPR+DOX	STR+ERY	STR+DOX	ERY+DOX	AMP+CPR+STR	AMP+CPR+ERY	AMP+CPR+DOX	AMP+STR+ERY	AMP+STR+DOX	AMP+ERY+DOX	CPR+STR+ERY	CPR+STR+DOX	CPR+ERY+DOX	STR+ERY+DOX	AMP+CPR+STR+ERY	AMP+CPR+STR+DOX	AMP+CPR+ERY+DOX	AMP+STR+ERY+DOX	CPR+STR+ERY+DOX	AMP+CPR+STR+ERY+DOX
	74.34273652	96.1427749	92.00576161	83.6514224	101.2243428	67.89483784	16.9449242	44.86662829	61.77317214	63.00134331	74.95682211	69.14219919	78.46597047	96.03889089	95.46272956	18.78718096	44.86662829	52.23565534	24.92803685	32.41220495	38.13087699	59.62387258	41.66186912	66.37881405	71.84011523	26.46325082	12.18576089	39.64690079	14.64210324	38.59144118	12.14738054
	69.79472141	99.70674487	85.0249584	82.95433537	100.9983361	67.44868035	22.34237537	43.40175953	63.69134897	69.55645161	75.07331378	69.26319648	79.40469588	92.71584396	95.67387687	17.77859238	42.8702346	50.91642229	19.99633431	22.92888563	52.84090909	56.35997067	49.74340176	71.02272727	82.36272879	24.39516129	28.33577713	42.41202346	17.35703812	48.44208211	22.76392962
	71.37722285	97.78660613	94.26199928	90.30262412	96.57163479	64.71812334	22.64472191	56.84827847	67.13961407	75.31214529	77.8093076	71.07453651	84.36356138	93.60210342	93.27215549	25.06621264	55.33484677	58.36171018	23.85546727	31.19561105	49.28111994	65.62618237	45.11918275	68.35035944	77.43465484	17.57472569	23.93113886	43.90843738	28.77412032	61.38857359	31.80098373

AMP3CPR3STR1ERY1DOX2	AMP	CPR	STR	ERY	DOX	AMP+CPR	AMP+STR	AMP+ERY	AMP+DOX	CPR+STR	CPR+ERY	CPR+DOX	STR+ERY	STR+DOX	ERY+DOX	AMP+CPR+STR	AMP+CPR+ERY	AMP+CPR+DOX	AMP+STR+ERY	AMP+STR+DOX	AMP+ERY+DOX	CPR+STR+ERY	CPR+STR+DOX	CPR+ERY+DOX	STR+ERY+DOX	AMP+CPR+STR+ERY	AMP+CPR+STR+DOX	AMP+CPR+ERY+DOX	AMP+STR+ERY+DOX	CPR+STR+ERY+DOX	AMP+CPR+STR+ERY+DOX
	74.34273652	96.1427749	92.00576161	83.6514224	95.46272956	67.89483784	16.9449242	44.86662829	55.93935905	63.00134331	74.95682211	70.98445596	78.46597047	98.91969752	99.20777818	18.78718096	44.86662829	53.46382652	24.92803685	10.61216657	45.49990405	59.62387258	41.35482633	67.60698522	81.3467771	26.46325082	16.75302245	46.72807523	10.30512378	41.04778353	14.79562464
	69.79472141	99.70674487	85.0249584	82.95433537	98.0403032	67.44868035	22.34237537	43.40175953	78.94061584	69.55645161	75.07331378	70.72947214	79.40469588	92.71584396	103.956369	17.77859238	42.8702346	54.25219941	19.99633431	6.50659824	53.72067449	56.35997067	50.62316716	63.98460411	89.16620447	24.39516129	4.582111437	43.58504399	18.07184751	56.65322581	24.81671554
	71.37722285	97.78660613	94.26199928	90.30262412	99.54116616	64.71812334	22.64472191	56.84827847	75.31214529	75.31214529	77.8093076	75.00945895	84.36356138	95.91173893	102.5106975	25.06621264	55.33484677	50.79455165	23.85546727	12.48581158	58.96708286	65.62618237	45.95157019	64.71812334	74.13517554	17.57472569	7.88876277	50.26485055	19.92054484	80.15512675	28.39576239

AMP3CPR3STR1ERY1DOX3	AMP	CPR	STR	ERY	DOX	AMP+CPR	AMP+STR	AMP+ERY	AMP+DOX	CPR+STR	CPR+ERY	CPR+DOX	STR+ERY	STR+DOX	ERY+DOX	AMP+CPR+STR	AMP+CPR+ERY	AMP+CPR+DOX	AMP+STR+ERY	AMP+STR+DOX	AMP+ERY+DOX	CPR+STR+ERY	CPR+STR+DOX	CPR+ERY+DOX	STR+ERY+DOX	AMP+CPR+STR+ERY	AMP+CPR+STR+DOX	AMP+CPR+ERY+DOX	AMP+STR+ERY+DOX	CPR+STR+ERY+DOX	AMP+CPR+STR+ERY+DOX
	74.34273652	96.1427749	92.00576161	83.6514224	101.2243428	67.89483784	16.9449242	44.86662829	46.11398964	63.00134331	74.95682211	65.76472846	78.46597047	81.63485776	98.63161685	18.78718096	44.86662829	54.07791211	24.92803685	8.501247361	36.26943005	59.62387258	66.37881405	64.53655728	75.29708318	26.46325082	28.91959317	46.11398964	10.03646133	38.28439839	21.70408751
	69.79472141	99.70674487	85.0249584	82.95433537	101.2941394	67.44868035	22.34237537	43.40175953	63.98460411	69.55645161	75.07331378	68.38343109	79.40469588	99.51931965	101.885746	17.77859238	42.8702346	54.728739	19.99633431	25.40322581	54.30718475	56.35997067	57.82624633	67.21041056	77.33407284	24.39516129	17.77859238	51.02639296	31.56158358	45.63782991	29.50879765
	71.37722285	97.78660613	94.26199928	90.30262412	101.8508017	64.71812334	22.64472191	56.84827847	76.825577	75.31214529	77.8093076	72.28528188	84.36356138	104.1604372	102.1807496	25.06621264	55.33484677	66.60991298	23.85546727	29.9091941	66.83692773	65.62618237	73.49602724	68.35035944	75.78491519	17.57472569	23.85546727	50.87022323	19.14491109	67.74498676	26.35262959

AMP3CPR3STR1ERY2DOX1	AMP	CPR	STR	ERY	DOX	AMP+CPR	AMP+STR	AMP+ERY	AMP+DOX	CPR+STR	CPR+ERY	CPR+DOX	STR+ERY	STR+DOX	ERY+DOX	AMP+CPR+STR	AMP+CPR+ERY	AMP+CPR+DOX	AMP+STR+ERY	AMP+STR+DOX	AMP+ERY+DOX	CPR+STR+ERY	CPR+STR+DOX	CPR+ERY+DOX	STR+ERY+DOX	AMP+CPR+STR+ERY	AMP+CPR+STR+DOX	AMP+CPR+ERY+DOX	AMP+STR+ERY+DOX	CPR+STR+ERY+DOX	AMP+CPR+STR+ERY+DOX
	74.34273652	96.1427749	92.00576161	96.90313288	101.2243428	67.89483784	16.9449242	47.9562464	61.77317214	63.00134331	73.11456534	69.14219919	91.42960029	96.03889089	99.7839395	18.78718096	52.54269814	52.23565534	17.86605258	32.41220495	49.4722702	60.85204375	41.66186912	63.92247169	69.24738927	13.26041067	12.18576089	42.71732873	13.56745346	39.05200537	14.48858185
	69.79472141	99.70674487	85.0249584	96.56128674	100.9983361	67.44868035	22.34237537	47.85557185	63.69134897	69.55645161	74.48680352	69.26319648	88.87040118	92.71584396	106.0269921	17.77859238	60.46554252	50.91642229	34.07258065	22.92888563	46.38929619	66.62390029	49.74340176	68.38343109	90.94102422	18.82331378	28.33577713	51.90615836	21.29765396	50.49486804	27.45601173
	71.37722285	97.78660613	94.26199928	96.90158272	96.57163479	64.71812334	22.64472191	65.62618237	67.13961407	75.31214529	85.0737798	71.07453651	91.62241584	93.60210342	99.54116616	25.06621264	69.56110481	58.36171018	37.47635263	31.19561105	57.45365115	69.56110481	45.11918275	75.91751797	84.69350931	33.54143019	23.93113886	51.77828226	18.48278471	82.27393114	28.77412032

AMP3CPR3STR1ERY2DOX2	AMP	CPR	STR	ERY	DOX	AMP+CPR	AMP+STR	AMP+ERY	AMP+DOX	CPR+STR	CPR+ERY	CPR+DOX	STR+ERY	STR+DOX	ERY+DOX	AMP+CPR+STR	AMP+CPR+ERY	AMP+CPR+DOX	AMP+STR+ERY	AMP+STR+DOX	AMP+ERY+DOX	CPR+STR+ERY	CPR+STR+DOX	CPR+ERY+DOX	STR+ERY+DOX	AMP+CPR+STR+ERY	AMP+CPR+STR+DOX	AMP+CPR+ERY+DOX	AMP+STR+ERY+DOX	CPR+STR+ERY+DOX	AMP+CPR+STR+ERY+DOX
	74.34273652	96.1427749	92.00576161	96.90313288	95.46272956	67.89483784	16.9449242	47.9562464	55.93935905	63.00134331	73.11456534	70.98445596	91.42960029	98.91969752	99.20777818	18.78718096	52.54269814	53.46382652	17.86605258	10.61216657	51.62156976	60.85204375	41.35482633	70.06332758	74.4328412	13.26041067	16.75302245	54.07791211	20.01535214	46.88159662	24.62099405
	69.79472141	99.70674487	85.0249584	96.56128674	98.0403032	67.44868035	22.34237537	47.85557185	78.94061584	69.55645161	74.48680352	70.72947214	88.87040118	92.71584396	106.6185986	17.77859238	60.46554252	54.25219941	34.07258065	6.50659824	48.38709677	66.62390029	50.62316716	56.35997067	89.75781106	18.82331378	4.582111437	57.18475073	14.84604106	50.62316716	24.68841642
	71.37722285	97.78660613	94.26199928	96.90158272	99.54116616	64.71812334	22.64472191	65.62618237	75.31214529	75.31214529	85.0737798	75.00945895	91.62241584	95.91173893	103.1705934	25.06621264	69.56110481	50.79455165	37.47635263	12.48581158	44.51381006	69.56110481	45.95157019	74.10139992	72.48543589	33.54143019	7.88876277	62.67499054	22.94740825	48.9784336	35.35754824

AMP3CPR3STR1ERY2DOX3	AMP	CPR	STR	ERY	DOX	AMP+CPR	AMP+STR	AMP+ERY	AMP+DOX	CPR+STR	CPR+ERY	CPR+DOX	STR+ERY	STR+DOX	ERY+DOX	AMP+CPR+STR	AMP+CPR+ERY	AMP+CPR+DOX	AMP+STR+ERY	AMP+STR+DOX	AMP+ERY+DOX	CPR+STR+ERY	CPR+STR+DOX	CPR+ERY+DOX	STR+ERY+DOX	AMP+CPR+STR+ERY	AMP+CPR+STR+DOX	AMP+CPR+ERY+DOX	AMP+STR+ERY+DOX	CPR+STR+ERY+DOX	AMP+CPR+STR+ERY+DOX
	74.34273652	96.1427749	92.00576161	96.90313288	101.2243428	67.89483784	16.9449242	47.9562464	46.11398964	63.00134331	73.11456534	65.76472846	91.42960029	81.63485776	102.0885848	18.78718096	52.54269814	54.07791211	17.86605258	8.501247361	56.84129726	60.85204375	66.37881405	74.0548839	77.31364782	13.26041067	28.91959317	64.51736711	16.33083861	52.25484552	25.54212243
	69.79472141	99.70674487	85.0249584	96.56128674	101.2941394	67.44868035	22.34237537	47.85557185	63.98460411	69.55645161	74.48680352	68.38343109	88.87040118	99.51931965	110.7598447	17.77859238	60.46554252	54.728739	34.07258065	25.40322581	61.58357771	66.62390029	57.82624633	54.60043988	94.19486042	18.82331378	17.77859238	65.68914956	14.25953079	56.06671554	17.06378299
	71.37722285	97.78660613	94.26199928	96.90158272	101.8508017	64.71812334	22.64472191	65.62618237	76.825577	75.31214529	85.0737798	72.28528188	91.62241584	104.1604372	95.91173893	25.06621264	69.56110481	66.60991298	37.47635263	29.9091941	55.10783201	69.56110481	73.49602724	82.57661748	73.47527968	33.54143019	23.85546727	69.33409005	14.16950435	59.87514188	32.02799849

AMP3CPR3STR1ERY3DOX1	AMP	CPR	STR	ERY	DOX	AMP+CPR	AMP+STR	AMP+ERY	AMP+DOX	CPR+STR	CPR+ERY	CPR+DOX	STR+ERY	STR+DOX	ERY+DOX	AMP+CPR+STR	AMP+CPR+ERY	AMP+CPR+DOX	AMP+STR+ERY	AMP+STR+DOX	AMP+ERY+DOX	CPR+STR+ERY	CPR+STR+DOX	CPR+ERY+DOX	STR+ERY+DOX	AMP+CPR+STR+ERY	AMP+CPR+STR+DOX	AMP+CPR+ERY+DOX	AMP+STR+ERY+DOX	CPR+STR+ERY+DOX	AMP+CPR+STR+ERY+DOX
	74.34273652	96.1427749	92.00576161	93.44616493	101.2243428	67.89483784	16.9449242	64.53655728	61.77317214	63.00134331	72.80752255	69.14219919	88.83687432	96.03889089	97.4792942	18.78718096	65.1314527	52.23565534	39.33985799	32.41220495	70.65822299	59.62387258	41.66186912	60.23795817	83.36334174	8.194204567	12.18576089	63.90328152	14.64210324	50.41258875	17.40548839
	69.79472141	99.70674487	85.0249584	94.49066371	100.9983361	67.44868035	22.34237537	75.12829912	63.69134897	69.55645161	75.65982405	69.26319648	85.0249584	92.71584396	107.8018118	17.77859238	52.78592375	50.91642229	22.6356305	22.92888563	69.50146628	62.81158358	49.74340176	71.60923754	95.08227029	22.92888563	28.33577713	61.29032258	23.93695015	39.05791789	32.44134897
	71.37722285	97.78660613	94.26199928	96.59225653	96.57163479	64.71812334	22.64472191	72.89065456	67.13961407	75.31214529	81.74423004	71.07453651	83.37371759	93.60210342	95.91173893	25.06621264	69.56110481	58.36171018	37.47635263	31.19561105	81.13885736	69.56110481	45.11918275	94.98675747	75.12501933	14.24517594	23.93113886	62.97767688	23.02307983	54.42678774	31.49829739

AMP3CPR3STR1ERY3DOX2	AMP	CPR	STR	ERY	DOX	AMP+CPR	AMP+STR	AMP+ERY	AMP+DOX	CPR+STR	CPR+ERY	CPR+DOX	STR+ERY	STR+DOX	ERY+DOX	AMP+CPR+STR	AMP+CPR+ERY	AMP+CPR+DOX	AMP+STR+ERY	AMP+STR+DOX	AMP+ERY+DOX	CPR+STR+ERY	CPR+STR+DOX	CPR+ERY+DOX	STR+ERY+DOX	AMP+CPR+STR+ERY	AMP+CPR+STR+DOX	AMP+CPR+ERY+DOX	AMP+STR+ERY+DOX	CPR+STR+ERY+DOX	AMP+CPR+STR+ERY+DOX
	74.34273652	96.1427749	92.00576161	93.44616493	95.46272956	67.89483784	16.9449242	64.53655728	55.93935905	63.00134331	72.80752255	70.98445596	88.83687432	98.91969752	97.4792942	18.78718096	65.1314527	53.46382652	39.33985799	10.61216657	76.18499328	59.62387258	41.35482633	74.03569372	100.0720202	8.194204567	16.75302245	60.21876799	10.34350413	49.49146037	25.84916523
	69.79472141	99.70674487	85.0249584	94.49066371	98.0403032	67.44868035	22.34237537	75.12829912	78.94061584	69.55645161	75.65982405	70.72947214	85.0249584	92.71584396	108.0976151	17.77859238	52.78592375	54.25219941	22.6356305	6.50659824	73.02052786	62.81158358	50.62316716	71.02272727	84.43335182	22.92888563	4.582111437	71.84750733	19.40982405	43.45674487	27.62096774
	71.37722285	97.78660613	94.26199928	96.59225653	99.54116616	64.71812334	22.64472191	72.89065456	75.31214529	75.31214529	81.74423004	75.00945895	83.37371759	95.91173893	103.8304893	25.06621264	69.56110481	50.79455165	37.47635263	12.48581158	70.84752176	69.56110481	45.95157019	78.03632236	84.69350931	14.24517594	7.88876277	61.46424518	21.1312902	61.08588725	48.37306092

AMP3CPR3STR1ERY3DOX3	AMP	CPR	STR	ERY	DOX	AMP+CPR	AMP+STR	AMP+ERY	AMP+DOX	CPR+STR	CPR+ERY	CPR+DOX	STR+ERY	STR+DOX	ERY+DOX	AMP+CPR+STR	AMP+CPR+ERY	AMP+CPR+DOX	AMP+STR+ERY	AMP+STR+DOX	AMP+ERY+DOX	CPR+STR+ERY	CPR+STR+DOX	CPR+ERY+DOX	STR+ERY+DOX	AMP+CPR+STR+ERY	AMP+CPR+STR+DOX	AMP+CPR+ERY+DOX	AMP+STR+ERY+DOX	CPR+STR+ERY+DOX	AMP+CPR+STR+ERY+DOX
	74.34273652	96.1427749	92.00576161	93.44616493	101.2243428	67.89483784	16.9449242	64.53655728	46.11398964	63.00134331	72.80752255	65.76472846	88.83687432	81.63485776	102.9528268	18.78718096	65.1314527	54.07791211	39.33985799	8.501247361	84.47514872	59.62387258	66.37881405	82.34503934	91.14151963	8.194204567	28.91959317	81.71176358	8.04068317	50.41258875	18.94070236
	69.79472141	99.70674487	85.0249584	94.49066371	101.2941394	67.44868035	22.34237537	75.12829912	63.98460411	69.55645161	75.65982405	68.38343109	85.0249584	99.51931965	108.985025	17.77859238	52.78592375	54.728739	22.6356305	25.40322581	70.38123167	62.81158358	57.82624633	78.06085044	96.56128674	22.92888563	17.77859238	79.47214076	19.8313783	58.99926686	17.94354839
	71.37722285	97.78660613	94.26199928	96.59225653	101.8508017	64.71812334	22.64472191	72.89065456	76.825577	75.31214529	81.74423004	72.28528188	83.37371759	104.1604372	113.3989792	25.06621264	69.56110481	66.60991298	37.47635263	29.9091941	75.61483163	69.56110481	73.49602724	84.69542187	90.96251998	14.24517594	23.85546727	78.03632236	29.30382142	74.70677261	18.48278471

AMP3CPR3STR2ERY1DOX1	AMP	CPR	STR	ERY	DOX	AMP+CPR	AMP+STR	AMP+ERY	AMP+DOX	CPR+STR	CPR+ERY	CPR+DOX	STR+ERY	STR+DOX	ERY+DOX	AMP+CPR+STR	AMP+CPR+ERY	AMP+CPR+DOX	AMP+STR+ERY	AMP+STR+DOX	AMP+ERY+DOX	CPR+STR+ERY	CPR+STR+DOX	CPR+ERY+DOX	STR+ERY+DOX	AMP+CPR+STR+ERY	AMP+CPR+STR+DOX	AMP+CPR+ERY+DOX	AMP+STR+ERY+DOX	CPR+STR+ERY+DOX	AMP+CPR+STR+ERY+DOX
	74.34273652	96.1427749	82.42586353	83.6514224	101.2243428	67.89483784	16.31164844	44.86662829	61.77317214	61.77317214	74.95682211	69.14219919	73.20799362	85.71795993	95.46272956	17.25196699	44.86662829	52.23565534	27.3843792	29.38015736	38.13087699	61.15908655	49.49146037	66.37881405	90.32689488	33.21819229	19.70830935	39.64690079	16.33083861	79.2746114	25.54212243
	69.79472141	99.70674487	81.02667338	82.95433537	100.9983361	67.44868035	19.70307918	43.40175953	63.69134897	66.03739003	75.07331378	69.26319648	67.11625566	73.88022144	95.67387687	19.99633431	42.8702346	50.91642229	31.72653959	33.02785924	52.84090909	63.69134897	42.57697947	71.02272727	79.03371917	41.99046921	24.81671554	42.41202346	37.59164223	71.90249267	18.9516129
	71.37722285	97.78660613	87.03696851	90.30262412	96.57163479	64.71812334	21.43397654	56.84827847	67.13961407	68.65304578	77.8093076	71.07453651	65.2283857	75.96491877	93.27215549	25.36889898	55.33484677	58.36171018	29.30382142	40.2005297	49.28111994	72.89065456	71.67990919	68.35035944	82.91208723	42.92470677	22.11502081	43.90843738	38.98978434	75.61483163	34.44948922

AMP3CPR3STR2ERY1DOX2	AMP	CPR	STR	ERY	DOX	AMP+CPR	AMP+STR	AMP+ERY	AMP+DOX	CPR+STR	CPR+ERY	CPR+DOX	STR+ERY	STR+DOX	ERY+DOX	AMP+CPR+STR	AMP+CPR+ERY	AMP+CPR+DOX	AMP+STR+ERY	AMP+STR+DOX	AMP+ERY+DOX	CPR+STR+ERY	CPR+STR+DOX	CPR+ERY+DOX	STR+ERY+DOX	AMP+CPR+STR+ERY	AMP+CPR+STR+DOX	AMP+CPR+ERY+DOX	AMP+STR+ERY+DOX	CPR+STR+ERY+DOX	AMP+CPR+STR+ERY+DOX
	74.34273652	96.1427749	82.42586353	83.6514224	95.46272956	67.89483784	16.31164844	44.86662829	55.93935905	61.77317214	74.95682211	70.98445596	73.20799362	90.32689488	99.20777818	17.25196699	44.86662829	53.46382652	27.3843792	19.55478795	45.49990405	61.15908655	68.22107081	67.60698522	82.42586353	33.21819229	27.3843792	46.72807523	23.54634427	69.44924199	29.53367876
	69.79472141	99.70674487	81.02667338	82.95433537	98.0403032	67.44868035	19.70307918	43.40175953	78.94061584	66.03739003	75.07331378	70.72947214	67.11625566	77.42325113	103.956369	19.99633431	42.8702346	54.25219941	31.72653959	23.64369501	53.72067449	63.69134897	65.15762463	63.98460411	89.01862104	41.99046921	24.23020528	43.58504399	34.95234604	74.24853372	19.24486804
	71.37722285	97.78660613	87.03696851	90.30262412	99.54116616	64.71812334	21.43397654	56.84827847	75.31214529	68.65304578	77.8093076	75.00945895	65.2283857	74.70179723	102.5106975	25.36889898	55.33484677	50.79455165	29.30382142	31.72531215	58.96708286	72.89065456	82.87930382	64.71812334	89.22769493	42.92470677	31.4226258	50.26485055	31.72531215	79.54975407	28.39576239

AMP3CPR3STR2ERY1DOX3	AMP	CPR	STR	ERY	DOX	AMP+CPR	AMP+STR	AMP+ERY	AMP+DOX	CPR+STR	CPR+ERY	CPR+DOX	STR+ERY	STR+DOX	ERY+DOX	AMP+CPR+STR	AMP+CPR+ERY	AMP+CPR+DOX	AMP+STR+ERY	AMP+STR+DOX	AMP+ERY+DOX	CPR+STR+ERY	CPR+STR+DOX	CPR+ERY+DOX	STR+ERY+DOX	AMP+CPR+STR+ERY	AMP+CPR+STR+DOX	AMP+CPR+ERY+DOX	AMP+STR+ERY+DOX	CPR+STR+ERY+DOX	AMP+CPR+STR+ERY+DOX
	74.34273652	96.1427749	82.42586353	83.6514224	101.2243428	67.89483784	16.31164844	44.86662829	46.11398964	61.77317214	74.95682211	65.76472846	73.20799362	94.27741056	98.63161685	17.25196699	44.86662829	54.07791211	27.3843792	14.64210324	36.26943005	61.15908655	65.45768566	64.53655728	61.35644659	33.21819229	24.62099405	46.11398964	16.02379582	70.67741316	28.30550758
	69.79472141	99.70674487	81.02667338	82.95433537	101.2941394	67.44868035	19.70307918	43.40175953	63.98460411	66.03739003	75.07331378	68.38343109	67.11625566	77.74534474	101.885746	19.99633431	42.8702346	54.728739	31.72653959	31.14002933	54.30718475	63.69134897	59.58577713	67.21041056	100.9360845	41.99046921	18.9516129	51.02639296	25.27492669	83.04618768	20.12463343
	71.37722285	97.78660613	87.03696851	90.30262412	101.8508017	64.71812334	21.43397654	56.84827847	76.825577	68.65304578	77.8093076	72.28528188	65.2283857	77.85960108	102.1807496	25.36889898	55.33484677	66.60991298	29.30382142	36.56829361	66.83692773	72.89065456	60.78320091	68.35035944	82.28052647	42.92470677	27.18501703	50.87022323	28.39576239	89.53840333	31.11993946

AMP3CPR3STR2ERY2DOX1	AMP	CPR	STR	ERY	DOX	AMP+CPR	AMP+STR	AMP+ERY	AMP+DOX	CPR+STR	CPR+ERY	CPR+DOX	STR+ERY	STR+DOX	ERY+DOX	AMP+CPR+STR	AMP+CPR+ERY	AMP+CPR+DOX	AMP+STR+ERY	AMP+STR+DOX	AMP+ERY+DOX	CPR+STR+ERY	CPR+STR+DOX	CPR+ERY+DOX	STR+ERY+DOX	AMP+CPR+STR+ERY	AMP+CPR+STR+DOX	AMP+CPR+ERY+DOX	AMP+STR+ERY+DOX	CPR+STR+ERY+DOX	AMP+CPR+STR+ERY+DOX
	74.34273652	96.1427749	82.42586353	96.90313288	101.2243428	67.89483784	16.31164844	47.9562464	61.77317214	61.77317214	73.11456534	69.14219919	85.65623312	85.71795993	99.7839395	17.25196699	52.54269814	52.23565534	29.22663596	29.38015736	49.4722702	56.55344464	49.49146037	63.92247169	68.59905867	8.942621378	19.70830935	42.71732873	24.62099405	60.54500096	12.95336788
	69.79472141	99.70674487	81.02667338	96.56128674	100.9983361	67.44868035	19.70307918	47.85557185	63.69134897	66.03739003	74.48680352	69.26319648	76.83945647	73.88022144	106.0269921	19.99633431	60.46554252	50.91642229	31.43328446	33.02785924	46.38929619	56.06671554	42.57697947	68.38343109	82.89884248	23.80865103	24.81671554	51.90615836	21.16935484	77.47434018	21.29765396
	71.37722285	97.78660613	87.03696851	96.90158272	96.57163479	64.71812334	21.43397654	65.62618237	67.13961407	68.65304578	85.0737798	71.07453651	82.93182351	75.96491877	99.54116616	25.36889898	69.56110481	58.36171018	25.97427166	40.2005297	57.45365115	62.29663261	71.67990919	75.91751797	96.80642416	31.4226258	22.11502081	51.77828226	23.25009459	85.90616723	27.79038971

AMP3CPR3STR2ERY2DOX2	AMP	CPR	STR	ERY	DOX	AMP+CPR	AMP+STR	AMP+ERY	AMP+DOX	CPR+STR	CPR+ERY	CPR+DOX	STR+ERY	STR+DOX	ERY+DOX	AMP+CPR+STR	AMP+CPR+ERY	AMP+CPR+DOX	AMP+STR+ERY	AMP+STR+DOX	AMP+ERY+DOX	CPR+STR+ERY	CPR+STR+DOX	CPR+ERY+DOX	STR+ERY+DOX	AMP+CPR+STR+ERY	AMP+CPR+STR+DOX	AMP+CPR+ERY+DOX	AMP+STR+ERY+DOX	CPR+STR+ERY+DOX	AMP+CPR+STR+ERY+DOX
	74.34273652	96.1427749	82.42586353	96.90313288	95.46272956	67.89483784	16.31164844	47.9562464	55.93935905	61.77317214	73.11456534	70.98445596	85.65623312	90.32689488	99.20777818	17.25196699	52.54269814	53.46382652	29.22663596	19.55478795	51.62156976	56.55344464	68.22107081	70.06332758	63.66091407	8.942621378	27.3843792	54.07791211	29.22663596	60.85204375	17.86605258
	69.79472141	99.70674487	81.02667338	96.56128674	98.0403032	67.44868035	19.70307918	47.85557185	78.94061584	66.03739003	74.48680352	70.72947214	76.83945647	77.42325113	106.6185986	19.99633431	60.46554252	54.25219941	31.43328446	23.64369501	48.38709677	56.06671554	65.15762463	56.35997067	81.28837443	23.80865103	24.23020528	57.18475073	20.87609971	68.09017595	23.22214076
	71.37722285	97.78660613	87.03696851	96.90158272	99.54116616	64.71812334	21.43397654	65.62618237	75.31214529	68.65304578	85.0737798	75.00945895	82.93182351	74.70179723	103.1705934	25.36889898	69.56110481	50.79455165	25.97427166	31.72531215	44.51381006	62.29663261	82.87930382	74.10139992	94.59596146	31.4226258	31.4226258	62.67499054	19.01248581	69.56110481	27.18501703

AMP3CPR3STR2ERY2DOX3	AMP	CPR	STR	ERY	DOX	AMP+CPR	AMP+STR	AMP+ERY	AMP+DOX	CPR+STR	CPR+ERY	CPR+DOX	STR+ERY	STR+DOX	ERY+DOX	AMP+CPR+STR	AMP+CPR+ERY	AMP+CPR+DOX	AMP+STR+ERY	AMP+STR+DOX	AMP+ERY+DOX	CPR+STR+ERY	CPR+STR+DOX	CPR+ERY+DOX	STR+ERY+DOX	AMP+CPR+STR+ERY	AMP+CPR+STR+DOX	AMP+CPR+ERY+DOX	AMP+STR+ERY+DOX	CPR+STR+ERY+DOX	AMP+CPR+STR+ERY+DOX
	74.34273652	96.1427749	82.42586353	96.90313288	101.2243428	67.89483784	16.31164844	47.9562464	46.11398964	61.77317214	73.11456534	65.76472846	85.65623312	94.27741056	102.0885848	17.25196699	52.54269814	54.07791211	29.22663596	14.64210324	56.84129726	56.55344464	65.45768566	74.0548839	75.77994393	8.942621378	24.62099405	64.51736711	12.95336788	58.08865861	22.1646517
	69.79472141	99.70674487	81.02667338	96.56128674	101.2941394	67.44868035	19.70307918	47.85557185	63.98460411	66.03739003	74.48680352	68.38343109	76.83945647	77.74534474	110.7598447	19.99633431	60.46554252	54.728739	31.43328446	31.14002933	61.58357771	56.06671554	59.58577713	54.60043988	80.96628083	23.80865103	18.9516129	65.68914956	18.07184751	54.01392962	20.28958944
	71.37722285	97.78660613	87.03696851	96.90158272	101.8508017	64.71812334	21.43397654	65.62618237	76.825577	68.65304578	85.0737798	72.28528188	82.93182351	77.85960108	95.91173893	25.36889898	69.56110481	66.60991298	25.97427166	36.56829361	55.10783201	62.29663261	60.78320091	82.57661748	78.49116185	31.4226258	27.18501703	69.33409005	17.57472569	59.2697692	28.39576239

AMP3CPR3STR2ERY3DOX1 [illegible]

AMP3CPR3STR2ERY3DOX2 [illegible]

AMP3CPR3STR2ERY3DOX3 [illegible]

AMP3CPR3STR3ERY1DOX1 [illegible]

AMP3CPR3STR3ERY1DOX2 [illegible]

AMP3CPR3STR3ERY1DOX3 [illegible]

AMP3CPR3STR3ERY2DOX1 [illegible]

AMP3CPR3STR3ERY2DOX2 [illegible]

AMP3CPR3STR3ERY2DOX3 [illegible]

AMP3CPR3STR3ERY3DOX1 [illegible]

AMP3CPR3STR3ERY3DOX2 [illegible]

AMP3CPR3STR3ERY3DOX3 [illegible]

AMP1CPR1STR1ERY1TMP1 [illegible]

AMP1CPR1STR1ERY1TMP2 [illegible]

AMP1CPR1STR1ERY1TMP3 [illegible]

AMP1CPR1STR1ERY2TMP1 [illegible]

AMP1CPR1STR1ERY2TMP2 [illegible]

AMP1CPR1STR1ERY2TMP3 [illegible]

AMP1CPR1STR1ERY3TMP1 [illegible]

AMP1CPR1STR1ERY3TMP2 [illegible]

AMP1CPR1STR1ERY3TMP3 [illegible]

AMP1CPR1STR2ERY1TMP1 [illegible]

AMP1CPR1STR2ERY1TMP2 [illegible]

AMP1CPR1STR2ERY1TMP3 [illegible]

	76.08600036	110.7345217	78.75106564	110.4033531	64.70207757	90.86440196	7.078730175	57.08519831	59.07221029	73.10548239	107.4228351	87.55271532	78.1543052	89.06649616	102.7864738	17.26216657	38.33277276	83.57869137	24.54787716	-7.368502755	54.76701767	75.7548317	42.63796538	94.17608859	114.3009378	20.98781403	44.16962045	83.2475227	-8.568989159	67.14444646	11.71509146
	71.24871001	98.65841073	67.2311001	91.0629515	105.9236326	78.18369453	8.111455108	58.36945304	69.59752322	51.24871001	86.76986584	89.74200206	81.6486538	93.71409904	113.5190918	30.95975232	44.16924665	71.90918473	11.41382869	6.336429309	77.85345717	56.71826625	73.374613	108.5655315	106.3893502	18.34881321	14.59236326	56.05779154	25.28379773	73.374613	16.90402477
AMP1CPR1STR2ERY2TMP1	AMP	CPR	STR	ERY	TMP	AMP+CPR	AMP+STR	AMP+ERY	AMP+TMP	CPR+STR	CPR+ERY	CPR+TMP	STR+ERY	STR+TMP	ERY+TMP	AMP+CPR+STR	AMP+CPR+ERY	AMP+CPR+TMP	AMP+STR+ERY	AMP+STR+TMP	AMP+ERY+TMP	CPR+STR+ERY	CPR+STR+TMP	CPR+ERY+TMP	STR+ERY+TMP	AMP+CPR+STR+ERY	AMP+CPR+STR+TMP	AMP+CPR+ERY+TMP	AMP+STR+ERY+TMP	CPR+STR+ERY+TMP	AMP+CPR+STR+ERY+TMP
	77.83543365	102.552155	68.10886253	104.2465318	116.1071693	58.83723393	8.365985386	50.32299058	60.53161072	62.24716721	85.94726252	75.10325109	46.49036263	60.10058474	88.99714074	5.993857884	38.18701684	37.48808641	15.41882876	14.40220269	66.63136715	54.43185428	35.13713862	82.55850895	86.89751426	12.94080271	12.94080271	52.39860214	17.11320555	66.99142222	5.633802817
	76.08600036	110.7345217	78.75106564	119.344907	104.4423171	90.86440196	7.078730175	36.88390986	59.07221029	73.10548239	77.28648677	95.8319319	74.31798806	102.0247229	95.50076324	17.26216657	43.96264004	75.63064345	2.111200228	17.80031565	54.43584901	62.38389692	70.24915267	77.28648677	103.0477408	22.97482601	42.30679672	85.56570335	-4.71915345	69.4626271	43.17611446
	71.24871001	98.65841073	67.2311001	93.374613	98.65841073	78.18369453	8.111455108	56.3880289	72.23942208	51.24871001	90.73271414	102.621259	72.10954727	77.81558816	113.1888545	30.95975232	57.04850361	76.20227038	8.111455108	40.14447884	96.34674923	55.06707946	57.64705882	112.1981424	96.28399532	23.63261094	36.3880289	48.4623323	7.512899897	53.89060888	14.94324045
AMP1CPR1STR2ERY2TMP2	AMP	CPR	STR	ERY	TMP	AMP+CPR	AMP+STR	AMP+ERY	AMP+TMP	CPR+STR	CPR+ERY	CPR+TMP	STR+ERY	STR+TMP	ERY+TMP	AMP+CPR+STR	AMP+CPR+ERY	AMP+CPR+TMP	AMP+STR+ERY	AMP+STR+TMP	AMP+ERY+TMP	CPR+STR+ERY	CPR+STR+TMP	CPR+ERY+TMP	STR+ERY+TMP	AMP+CPR+STR+ERY	AMP+CPR+STR+TMP	AMP+CPR+ERY+TMP	AMP+STR+ERY+TMP	CPR+STR+ERY+TMP	AMP+CPR+STR+ERY+TMP
	77.83543365	102.552155	68.10886253	104.2465318	100.8577782	58.83723393	8.365985386	50.32299058	62.18362808	62.24716721	85.94726252	74.42550037	46.49036263	64.662993	96.79127396	5.993857884	38.18701684	34.77708355	15.41882876	15.41882876	53.37286879	54.43185428	51.06428042	76.45875252	87.51353563	12.94080271	4.278301387	52.7374775	6.269194112	57.50291221	1.927353595
	76.08600036	110.7345217	78.75106564	119.344907	83.90986003	90.86440196	7.078730175	36.88390986	70.99428217	73.10548239	77.28648677	71.32545083	74.31798806	97.25063939	104.7734858	17.26216657	43.96264004	63.70857158	2.111200228	1.57305115	55.09818633	62.38389692	46.61198934	82.58518538	87.02046036	22.97482601	40.19559649	103.4488112	-5.71265944	72.77431373	10.72158547
	71.24871001	98.65841073	67.2311001	93.374613	100.6398349	78.18369453	8.111455108	56.3880289	73.23013416	51.24871001	90.73271414	105.5933953	72.10954727	86.74488879	111.8679051	30.95975232	57.04850361	57.04850361	8.111455108	3.364293086	97.00722394	55.06707946	74.36532508	121.1145511	68.40715433	23.63261094	8.648090815	50.77399381	14.11764706	70.07223942	16.26418989
AMP1CPR1STR2ERY2TMP3	AMP	CPR	STR	ERY	TMP	AMP+CPR	AMP+STR	AMP+ERY	AMP+TMP	CPR+STR	CPR+ERY	CPR+TMP	STR+ERY	STR+TMP	ERY+TMP	AMP+CPR+STR	AMP+CPR+ERY	AMP+CPR+TMP	AMP+STR+ERY	AMP+STR+TMP	AMP+ERY+TMP	CPR+STR+ERY	CPR+STR+TMP	CPR+ERY+TMP	STR+ERY+TMP	AMP+CPR+STR+ERY	AMP+CPR+STR+TMP	AMP+CPR+ERY+TMP	AMP+STR+ERY+TMP	CPR+STR+ERY+TMP	AMP+CPR+STR+ERY+TMP
	77.83543365	102.552155	68.10886253	104.2465318	81.88075823	58.83723393	8.365985386	50.32299058	52.01736736	62.24716721	85.94726252	76.11987716	46.49036263	78.2154631	100.1800275	5.993857884	38.18701684	55.109605	15.41882876	9.319072329	61.88711215	54.43185428	60.55279043	81.20300752	87.20552494	12.94080271	20.56549825	40.53796463	50.04765435	59.197289	16.66843164
	76.08600036	110.7345217	78.75106564	119.344907	64.70207757	90.86440196	7.078730175	36.88390986	59.07221029	73.10548239	77.28648677	87.55271532	74.31798806	89.06649616	113.7150397	17.26216657	43.96264004	83.57869137	2.111200228	-7.368502755	66.35792088	62.38389692	42.63796538	100.1371245	110.5498721	22.97482601	44.16962045	104.1111485	-5.381490776	69.13145844	4.760549533
	71.24871001	98.65841073	67.2311001	93.374613	105.9236326	78.18369453	8.111455108	56.3880289	69.59752322	51.24871001	90.73271414	89.74200206	72.10954727	93.71409904	113.8493292	30.95975232	57.04850361	71.90918473	8.111455108	6.336429309	101.630547	55.06707946	73.374613	124.747162	98.02629788	23.63261094	14.59236326	55.06707946	10.15479876	68.75128999	11.74406605
AMP1CPR1STR2ERY3TMP1	AMP	CPR	STR	ERY	TMP	AMP+CPR	AMP+STR	AMP+ERY	AMP+TMP	CPR+STR	CPR+ERY	CPR+TMP	STR+ERY	STR+TMP	ERY+TMP	AMP+CPR+STR	AMP+CPR+ERY	AMP+CPR+TMP	AMP+STR+ERY	AMP+STR+TMP	AMP+ERY+TMP	CPR+STR+ERY	CPR+STR+TMP	CPR+ERY+TMP	STR+ERY+TMP	AMP+CPR+STR+ERY	AMP+CPR+STR+TMP	AMP+CPR+ERY+TMP	AMP+STR+ERY+TMP	CPR+STR+ERY+TMP	AMP+CPR+STR+ERY+TMP
	77.83543365	102.552155	68.10886253	111.7017897	116.1071693	58.83723393	8.365985386	55.40612094	60.53161072	62.24716721	76.45875252	75.10325109	50.24424285	60.10058474	100.1800275	5.993857884	53.43640792	37.48808641	17.79095626	14.40220269	75.7810018	66.63136715	35.13713862	86.28613788	69.28315326	18.84994176	12.94080271	53.75410357	8.196547707	64.61929472	13.11024039
	76.08600036	110.7345217	78.75106564	116.0332204	104.4423171	90.86440196	7.078730175	60.31409278	59.07221029	73.10548239	83.2475227	95.8319319	75	102.0247229	121.9942563	17.26216657	48.14364441	75.63064345	-7.823859667	17.80031565	72.65012548	60.06571628	70.24915267	104.7734858	105.4347826	24.96183799	42.30679672	107.7540038	4.015420041	73.43665106	23.88553983
	71.24871001	98.65841073	67.2311001	92.05366357	98.65841073	78.18369453	8.111455108	65.44891641	72.23942208	51.24871001	94.69556244	102.621259	75.94261291	77.81558816	119.4633643	30.95975232	55.39731682	76.20227038	3.488132095	40.14447884	112.8586171	66.29514964	57.64705882	118.8028896	80.95173277	11.80598555	36.3880289	76.8627451	11.14551084	78.32817337	1.73374613
AMP1CPR1STR2ERY3TMP2	AMP	CPR	STR	ERY	TMP	AMP+CPR	AMP+STR	AMP+ERY	AMP+TMP	CPR+STR	CPR+ERY	CPR+TMP	STR+ERY	STR+TMP	ERY+TMP	AMP+CPR+STR	AMP+CPR+ERY	AMP+CPR+TMP	AMP+STR+ERY	AMP+STR+TMP	AMP+ERY+TMP	CPR+STR+ERY	CPR+STR+TMP	CPR+ERY+TMP	STR+ERY+TMP	AMP+CPR+STR+ERY	AMP+CPR+STR+TMP	AMP+CPR+ERY+TMP	AMP+STR+ERY+TMP	CPR+STR+ERY+TMP	AMP+CPR+STR+ERY+TMP
	77.83543365	102.552155	68.10886253	111.7017897	100.8577782	58.83723393	8.365985386	55.40612094	62.18362808	62.24716721	76.45875252	74.42550037	50.24424285	64.662993	89.3360161	5.993857884	53.43640792	34.77708355	17.79095626	15.41882876	77.47537859	66.63136715	51.06428042	87.98051467	81.29556994	18.84994176	4.278301387	66.29249179	1.228423171	55.80853542	3.93942603
	76.08600036	110.7345217	78.75106564	116.0332204	83.90986003	90.86440196	7.078730175	60.31409278	70.99428217	73.10548239	83.2475227	71.32545083	75	97.25063939	93.84491992	17.26216657	48.14364441	63.70857158	-7.823859667	1.57305115	72.65012548	60.06571628	46.61198934	93.51375126	79.85933504	24.96183799	40.19559649	95.16959458	3.353082715	54.22886859	3.891231792
	71.24871001	98.65841073	67.2311001	92.05366357	100.6398349	78.18369453	8.111455108	65.44891641	73.23013416	51.24871001	94.69556244	105.5933953	75.94261291	86.74488879	103.6119711	30.95975232	55.39731682	57.04850361	3.488132095	3.364293086	85.11867905	66.29514964	74.36532508	116.1609907	76.63953393	11.80598555	8.648090815	59.69040248	7.843137255	77.13106295	6.687306502
AMP1CPR1STR2ERY3TMP3	AMP	CPR	STR	ERY	TMP	AMP+CPR	AMP+STR	AMP+ERY	AMP+TMP	CPR+STR	CPR+ERY	CPR+TMP	STR+ERY	STR+TMP	ERY+TMP	AMP+CPR+STR	AMP+CPR+ERY	AMP+CPR+TMP	AMP+STR+ERY	AMP+STR+TMP	AMP+ERY+TMP	CPR+STR+ERY	CPR+STR+TMP	CPR+ERY+TMP	STR+ERY+TMP	AMP+CPR+STR+ERY	AMP+CPR+STR+TMP	AMP+CPR+ERY+TMP	AMP+STR+ERY+TMP	CPR+STR+ERY+TMP	AMP+CPR+STR+ERY+TMP
	77.83543365	102.552155	68.10886253	111.7017897	81.88075823	58.83723393	8.365985386	55.40612094	52.01736736	62.24716721	76.45875252	76.11987716	50.24424285	78.2154631	80.5252568	5.993857884	53.43640792	55.109605	17.79095626	9.319072329	66.29249179	66.63136715	60.55279043	82.21963359	73.96106552	18.84994176	20.56549825	54.43185428	5.972678174	75.1244308	-0.783649264
	76.08600036	110.7345217	78.75106564	116.0332204	64.70207757	90.86440196	7.078730175	60.31409278	59.07221029	73.10548239	83.2475227	87.55271532	75	89.06649616	128.6176296	17.26216657	48.14364441	83.57869137	-7.823859667	-7.368502755	78.27999276	60.06571628	42.63796538	80.26700473	81.22335891	24.96183799	44.16962045	91.85790795	3.767043544	50.37903288	3.228894466
	71.24871001	98.65841073	67.2311001	92.05366357	105.9236326	78.18369453	8.111455108	65.44891641	69.59752322	51.24871001	94.69556244	89.74200206	75.94261291	93.71409904	99.97936017	30.95975232	55.39731682	71.90918473	3.488132095	6.336429309	104.2724458	66.29514964	73.374613	116.1609907	82.69403534	11.80598555	14.59236326	62.33230134	6.191950464	62.47678019	3.694530444
AMP1CPR1STR3ERY1TMP1	AMP	CPR	STR	ERY	TMP	AMP+CPR	AMP+STR	AMP+ERY	AMP+TMP	CPR+STR	CPR+ERY	CPR+TMP	STR+ERY	STR+TMP	ERY+TMP	AMP+CPR+STR	AMP+CPR+ERY	AMP+CPR+TMP	AMP+STR+ERY	AMP+STR+TMP	AMP+ERY+TMP	CPR+STR+ERY	CPR+STR+TMP	CPR+ERY+TMP	STR+ERY+TMP	AMP+CPR+STR+ERY	AMP+CPR+STR+TMP	AMP+CPR+ERY+TMP	AMP+STR+ERY+TMP	CPR+STR+ERY+TMP	AMP+CPR+STR+ERY+TMP
	77.83543365	102.552155	74.20542334	101.535529	116.1071693	58.83723393	5.591443397	32.76501112	60.53161072	100.8577782	107.29641	75.10325109	97.01871727	108.9645875	91.03039288	16.81668961	35.81488934	37.48808641	2.202689823	33.71809806	61.88711215	88.99714074	55.44848036	85.60838717	109.2964173	11.07698825	42.57121677	52.7374775	30.03282855	38.5258922	21.89981997
	76.08600036	110.7345217	60.83351516	110.4033531	104.4423171	90.86440196	22.43667693	57.08519831	59.07221029	103.1176425	107.4228351	95.8319319	81.43137683	106.9168667	89.87089597	26.28651264	38.33277276	75.63064345	11.05275413	17.6761274	68.67610152	77.28648677	69.00727019	78.27999276	116.6921231	41.85143981	75.29947479	61.39039093	26.6176813	31.7093995	34.89689788
	71.24871001	98.65841073	64.81466508	91.0629515	98.65841073	78.18369453	23.30237358	58.36945304	72.23942208	89.74200206	86.76986584	102.621259	42.93255403	51.80368013	112.5283798	13.7874097	44.16924665	76.20227038	18.67905057	45.49019608	97.99793602	86.10939112	60.68111455	97.66769866	73.79668026	16.75954592	45.15995872	80.49535604	35.91331269	54.55108359	28.64809082
AMP1CPR1STR3ERY1TMP2	AMP	CPR	STR	ERY	TMP	AMP+CPR	AMP+STR	AMP+ERY	AMP+TMP	CPR+STR	CPR+ERY	CPR+TMP	STR+ERY	STR+TMP	ERY+TMP	AMP+CPR+STR	AMP+CPR+ERY	AMP+CPR+TMP	AMP+STR+ERY	AMP+STR+TMP	AMP+ERY+TMP	CPR+STR+ERY	CPR+STR+TMP	CPR+ERY+TMP	STR+ERY+TMP	AMP+CPR+STR+ERY	AMP+CPR+STR+TMP	AMP+CPR+ERY+TMP	AMP+STR+ERY+TMP	CPR+STR+ERY+TMP	AMP+CPR+STR+ERY+TMP
	77.83543365	102.552155	74.20542334	101.535529	100.8577782	58.83723393	5.591443397	32.76501112	62.18362808	100.8577782	107.29641	74.42550037	97.01871727	90.7139524	92.04701896	16.81668961	35.81488934	34.77708355	2.202689823	27.61834163	47.31547178	88.99714074	69.34237001	78.1531293	108.6327578	11.07698825	13.08906068	44.94334428	11.35232447	59.17610929	36.13258498
	76.08600036	110.7345217	60.83351516	110.4033531	83.90986003	90.86440196	22.43667693	57.08519831	70.99428217	103.1176425	107.4228351	71.32545083	81.43137683	104.1239363	104.1111485	26.28651264	38.33277276	63.70857158	11.05275413	5.547075108	54.76701767	77.28648677	45.4942951	79.93583607	116.6921231	41.85143981	30.92287392	55.76052366	10.05924814	45.94965201	28.39771287
	71.24871001	98.65841073	64.81466508	91.0629515	100.6398349	78.18369453	23.30237358	58.36945304	73.23013416	89.74200206	86.76986584	105.5933953	42.93255403	58.12435748	107.244582	13.7874097	44.16924665	57.04850361	18.67905057	24.16924665	69.26728586	86.10939112	88.42105263	101.9607843	80.00646853	16.75954592	21.7131063	51.43446852	28.31785346	88.09081527	6.790505676
AMP1CPR1STR3ERY1TMP3	AMP	CPR	STR	ERY	TMP	AMP+CPR	AMP+STR	AMP+ERY	AMP+TMP	CPR+STR	CPR+ERY	CPR+TMP	STR+ERY	STR+TMP	ERY+TMP	AMP+CPR+STR	AMP+CPR+ERY	AMP+CPR+TMP	AMP+STR+ERY	AMP+STR+TMP	AMP+ERY+TMP	CPR+STR+ERY	CPR+STR+TMP	CPR+ERY+TMP	STR+ERY+TMP	AMP+CPR+STR+ERY	AMP+CPR+STR+TMP	AMP+CPR+ERY+TMP	AMP+STR+ERY+TMP	CPR+STR+ERY+TMP	AMP+CPR+STR+ERY+TMP
	77.83543365	102.552155	74.20542334	101.535529	81.88075823	58.83723393	5.591443397	32.76501112	52.01736736	100.8577782	107.29641	76.11987716	97.01871727	91.4605693	100.8577782	16.81668961	35.81488934	55.109605	2.202689823	20.84083448	56.80398179	88.99714074	67.30911787	94.41914646	107.9690984	11.07698825	14.29630414	31.72720534	19.82420841	67.98686858	26.15694165
	76.08600036	110.7345217	60.83351516	110.4033531	64.70207757	90.86440196	22.43667693	57.08519831	59.07221029	103.1176425	107.4228351	87.55271532	81.43137683	102.0292385	102.7864738	26.28651264	38.33277276	83.57869137	11.05275413	0.041396083	54.76701767	77.28648677	75.63064345	94.17608859	109.3606808	41.85143981	28.93586194	83.2475227	4.346588704	45.82546377	16.02028408
	71.24871001	98.65841073	64.81466508	91.0629515	105.9236326	78.18369453	23.30237358	58.36945304	69.59752322	89.74200206	86.76986584	89.74200206	42.93255403	70.72874915	113.5190918	13.7874097	44.16924665	71.90918473	18.67905057	20.86687307	77.85345717	86.10939112	79.50464396	108.5655315	75.57090548	16.75954592	27.9876161	56.05779154	26.81114551	77.52321981	14.92260062
AMP1CPR1STR3ERY2TMP1	AMP	CPR	STR	ERY	TMP	AMP+CPR	AMP+STR	AMP+ERY	AMP+TMP	CPR+STR	CPR+ERY	CPR+TMP	STR+ERY	STR+TMP	ERY+TMP	AMP+CPR+STR	AMP+CPR+ERY	AMP+CPR+TMP	AMP+STR+ERY	AMP+STR+TMP	AMP+ERY+TMP	CPR+STR+ERY	CPR+STR+TMP	CPR+ERY+TMP	STR+ERY+TMP	AMP+CPR+STR+ERY	AMP+CPR+STR+TMP	AMP+CPR+ERY+TMP	AMP+STR+ERY+TMP	CPR+STR+ERY+TMP	AMP+CPR+STR+ERY+TMP
	77.83543365	102.552155	74.20542334	104.2465318	116.1071693	58.83723393	5.591443397	50.32299058	60.53161072	100.8577782	85.94726252	75.10325109	97.68237673	108.9645875	88.99714074	16.81668961	38.18701684	37.48808641	27.95721699	33.71809806	66.63136715	71.0367468	55.44848036	82.55850895	103.9871416	17.51562004	42.57121677	52.39860214	26.60171556	45.62109499	21.89981997
	76.08600036	110.7345217	60.83351516	119.344907	104.4423171	90.86440196	22.43667693	36.88390986	59.07221029	103.1176425	77.28648677	95.8319319	88.76281911	106.9168667	95.50076324	26.28651264	43.96264004	75.63064345	35.8076117	17.6761274	54.43584901	81.92284805	69.00727019	77.28648677	111.1062623	14.48862901	75.29947479	85.56570335	9.396910817	43.96264004	24.29950066
	71.24871001	98.65841073	64.81466508	93.374613	98.65841073	78.18369453	23.30237358	56.3880289	72.23942208	89.74200206	90.73271414	102.621259	64.81466508	51.80368013	113.1888545	13.7874097	57.04850361	76.20227038	8.771929825	45.49019608	96.34674923	89.74200206	60.68111455	112.1981424	73.09438278	11.41382869	45.15995872	48.4623323	42.1878225	72.23942208	18.01857585
AMP1CPR1STR3ERY2TMP2	AMP	CPR	STR	ERY	TMP	AMP+CPR	AMP+STR	AMP+ERY	AMP+TMP	CPR+STR	CPR+ERY	CPR+TMP	STR+ERY	STR+TMP	ERY+TMP	AMP+CPR+STR	AMP+CPR+ERY	AMP+CPR+TMP	AMP+STR+ERY	AMP+STR+TMP	AMP+ERY+TMP	CPR+STR+ERY	CPR+STR+TMP	CPR+ERY+TMP	STR+ERY+TMP	AMP+CPR+STR+ERY	AMP+CPR+STR+TMP	AMP+CPR+ERY+TMP	AMP+STR+ERY+TMP	CPR+STR+ERY+TMP	AMP+CPR+STR+ERY+TMP
	77.83543365	102.552155	74.20542334	104.2465318	100.8577782	58.83723393	5.591443397	50.32299058	62.18362808	100.8577782	85.94726252	74.42550037	97.68237673	90.7139524	96.79127396	16.81668961	38.18701684	34.77708355	27.95721699	27.61834163	53.37286879	71.0367468	69.34237001	76.45875252	106.6417794	17.51562004	13.08906068	52.7374775	15.41882876	42.59239648	25.98750397
	76.08600036	110.7345217	60.83351516	119.344907	83.90986003	90.86440196	22.43667693	36.88390986	70.99428217	103.1176425	77.28648677	71.32545083	88.76281911	104.1239363	104.7734858	26.28651264	43.96264004	63.70857158	35.8076117	5.547075108	55.09818633	81.92284805	45.4942951	82.58518538	106.5677504	14.48862901	30.92287392	103.4488112	1.448862901	49.26133865	19.66313938
	71.24871001	98.65841073	64.81466508	93.374613	100.6398349	78.18369453	23.30237358	56.3880289	73.23013416	89.74200206	90.73271414	105.5933953	64.81466508	58.12435748	111.8679051	13.7874097	57.04850361	57.04850361	8.771929825	24.16924665	97.00722394	89.74200206	88.42105263	121.1145511	77.93653911	11.41382869	21.7131063	50.77399381	26.99690402	61.67182663	16.9246646
AMP1CPR1STR3ERY2TMP3	AMP	CPR	STR	ERY	TMP	AMP+CPR	AMP+STR	AMP+ERY	AMP+TMP	CPR+STR	CPR+ERY	CPR+TMP	STR+ERY	STR+TMP	ERY+TMP	AMP+CPR+STR	AMP+CPR+ERY	AMP+CPR+TMP	AMP+STR+ERY	AMP+STR+TMP	AMP+ERY+TMP	CPR+STR+ERY	CPR+STR+TMP	CPR+ERY+TMP	STR+ERY+TMP	AMP+CPR+STR+ERY	AMP+CPR+STR+TMP	AMP+CPR+ERY+TMP	AMP+STR+ERY+TMP	CPR+STR+ERY+TMP	AMP+CPR+STR+ERY+TMP
	77.83543365	102.552155	74.20542334	104.2465318	81.88075823	58.83723393	5.591443397	50.32299058	52.01736736	100.8577782	85.94726252	76.11987716	97.68237673	91.4605693	100.1800275	16.81668961	38.18701684	55.109605	27.95721699	20.84083448	61.88711215	71.0367468	67.30911787	81.20300752	107.6372686	17.51562004	14.29630414	40.53796463	34.4382082	54.11415864	28.67732712
	76.08600036	110.7345217	60.83351516	119.344907	64.70207757	90.86440196	22.43667693	36.88390986	59.07221029	103.1176425	77.28648677	87.55271532	88.76281911	102.0292385	113.7150397	26.28651264	43.96264004	83.57869137	35.8076117	0.041396083	66.35792088	81.92284805	75.63064345	100.1371245	103.0765874	14.48862901	28.93586194	104.1111485	23.63716333	68.0137642	28.60469328
	71.24871001	98.65841073	64.81466508	93.374613	105.9236326	78.18369453	23.30237358	56.3880289	69.59752322	89.74200206	90.73271414	89.74200206	64.81466508	70.72874915	113.8493292	13.7874097	57.04850361	71.90918473	8.771929825	20.86687307	101.630547	89.74200206	79.50464396	124.747162	88.58189043	11.41382869	27.9876161	55.06707946	45.15995872	70.58823529	8.441692466
AMP1CPR1STR3ERY3TMP1	AMP	CPR	STR	ERY	TMP	AMP+CPR	AMP+STR	AMP+ERY	AMP+TMP	CPR+STR	CPR+ERY	CPR+TMP	STR+ERY	STR+TMP	ERY+TMP	AMP+CPR+STR	AMP+CPR+ERY	AMP+CPR+TMP	AMP+STR+ERY	AMP+STR+TMP	AMP+ERY+TMP	CPR+STR+ERY	CPR+STR+TMP	CPR+ERY+TMP	STR+ERY+TMP	AMP+CPR+STR+ERY	AMP+CPR+STR+TMP	AMP+CPR+ERY+TMP	AMP+STR+ERY+TMP	CPR+STR+ERY+TMP	AMP+CPR+STR+ERY+TMP
	77.83543365	102.552155	74.20542334	111.7017897	116.1071693	58.83723393	5.591443397	55.40612094	60.53161072	100.8577782	76.45875252	75.10325109	92.37310105	108.9645875	100.1800275	16.81668961	53.43640792	37.48808641	19.14645769	33.71809806	75.7810018	75.44212644	55.44848036	86.28613788	100.6688443	3.621730382	42.57121677	53.75410357	45.95997035	49.34872392	37.48808641
	76.08600036	110.7345217	60.83351516	116.0332204	104.4423171	90.86440196	22.43667693	60.31409278	59.07221029	103.1176425	83.2475227	95.8319319	95.0469125	106.9168667	121.9942563	26.28651264	48.14364441	75.63064345	9.976455978	17.6761274	72.65012548	80.5981734	69.00727019	104.7734858	105.8695178	22.31248868	75.29947479	107.7540038	56.09169232	68.67610152	24.29950066
	71.24871001	98.65841073	64.81466508	92.05366357	98.65841073	78.18369453	23.30237358	65.44891641	72.23942208	89.74200206	94.69556244	102.621259	67.47600291	51.80368013	119.4633643	13.7874097	55.39731682	76.20227038	20.99071207	45.49019608	112.8586171	88.42105263	60.68111455	118.8028896	84.14632738	12.79669763	45.15995872	76.8627451	68.60681115	75.54179567	19.87616099
AMP1CPR1STR3ERY3TMP2	AMP	CPR	STR	ERY	TMP	AMP+CPR	AMP+STR	AMP+ERY	AMP+TMP	CPR+STR	CPR+ERY	CPR+TMP	STR+ERY	STR+TMP	ERY+TMP	AMP+CPR+STR	AMP+CPR+ERY	AMP+CPR+TMP	AMP+STR+ERY	AMP+STR+TMP	AMP+ERY+TMP	CPR+STR+ERY	CPR+STR+TMP	CPR+ERY+TMP	STR+ERY+TMP	AMP+CPR+STR+ERY	AMP+CPR+STR+TMP	AMP+CPR+ERY+TMP	AMP+STR+ERY+TMP	CPR+STR+ERY+TMP	AMP+CPR+STR+ERY+TMP
	77.83543365	102.552155	74.20542334	111.7017897	100.8577782	58.83723393	5.591443397	55.40612094	62.18362808	100.8577782	76.45875252	74.42550037	92.37310105	90.7139524	89.3360161	16.81668961	53.43640792	34.77708355	19.14645769	27.61834163	77.47537859	75.44212644	69.34237001	87.98051467	74.45429564	3.621730382	13.08906068	66.29249179	31.04945462	66.29249179	32.74383141
	76.08600036	110.7345217	60.83351516	116.0332204	83.90986003	90.86440196	22.43667693	60.31409278	70.99428217	103.1176425	83.2475227	71.32545083	95.0469125	104.1239363	93.84491992	26.28651264	48.14364441	63.70857158	9.976455978	5.547075108	72.65012548	80.5981734	45.4942951	93.51375126	92.95221471	22.31248868	30.92287392	95.16959458	35.14527438	64.70207757	22.76784559
	71.24871001	98.65841073	64.81466508	92.05366357	100.6398349	78.18369453	23.30237358	65.44891641	73.23013416	89.74200206	94.69556244	105.5933953	67.47600291	58.12435748	103.6119711	13.7874097	55.39731682	57.04850361	20.99071207	24.16924665	85.11867905	88.42105263	88.42105263	116.1609907	85.03343999	12.79669763	21.7131063	59.69040248	39.54592363	80.8255934	20.20639835
AMP1CPR1STR3ERY3TMP3	AMP	CPR	STR	ERY	TMP	AMP+CPR	AMP+STR	AMP+ERY	AMP+TMP	CPR+STR	CPR+ERY	CPR+TMP	STR+ERY	STR+TMP	ERY+TMP	AMP+CPR+STR	AMP+CPR+ERY	AMP+CPR+TMP	AMP+STR+ERY	AMP+STR+TMP	AMP+ERY+TMP	CPR+STR+ERY	CPR+STR+TMP	CPR+ERY+TMP	STR+ERY+TMP	AMP+CPR+STR+ERY	AMP+CPR+STR+TMP	AMP+CPR+ERY+TMP	AMP+STR+ERY+TMP	CPR+STR+ERY+TMP	AMP+CPR+STR+ERY+TMP
	77.83543365	102.552155	74.20542334	111.7017897	81.88075823	58.83723393	5.591443397	55.40612094	52.01736736	100.8577782	76.45875252	76.11987716	92.37310105	91.4605693	80.5252568	16.81668961	53.43640792	55.109605	19.14645769	20.84083448	66.29249179	75.44212644	67.30911787	82.21963359	91.70944159	3.621730382	14.29630414	54.43185428	37.48808641	39.1824632	22.57757069
	76.08600036	110.7345217	60.83351516	116.0332204	64.70207757	90.86440196	22.43667693	60.31409278	59.07221029	103.1176425	83.2475227	87.55271532	95.0469125	102.0292385	128.6176296	26.28651264	48.14364441	83.57869137	9.976455978	0.041396083	78.27999276	80.5981734	75.63064345	80.26700473	98.1889592	22.31248868	28.93586194	91.85790795	29.92936793	57.08519831	38.33277276
	71.24871001	98.65841073	64.81466508	92.05366357	105.9236326	78.18369453	23.30237358	65.44891641	69.59752322	89.74200206	94.69556244	89.74200206	67.47600291	70.72874915	99.97936017	13.7874097	55.39731682	71.90918473	20.99071207	20.86687307	104.2724458	88.42105263	79.50464396	116.1609907	77.3451307	12.79669763	27.9876161	62.33230134	28.31785346	58.36945304	24.499484
AMP1CPR2STR1ERY1TMP1	AMP	CPR	STR	ERY	TMP	AMP+CPR	AMP+STR	AMP+ERY	AMP+TMP	CPR+STR	CPR+ERY	CPR+TMP	STR+ERY	STR+TMP	ERY+TMP	AMP+CPR+STR	AMP+CPR+ERY	AMP+CPR+TMP	AMP+STR+ERY	AMP+STR+TMP	AMP+ERY+TMP	CPR+STR+ERY	CPR+STR+TMP	CPR+ERY+TMP	STR+ERY+TMP	AMP+CPR+STR+ERY	AMP+CPR+STR+TMP	AMP+CPR+ERY+TMP	AMP+STR+ERY+TMP	CPR+STR+ERY+TMP	AMP+CPR+STR+ERY+TMP
	77.83543365	79.50525784	99.50227682	101.535529	116.1071693	56.46510643	2.245049243	32.76501112	60.53161072	59.27280603	48.03041606	72.72902279	112.3795404	83.23625966	91.03039288	2.60510431	33.08270677	65.95361644	-2.880440538	5.591443397	61.88711215	53.72861371	23.67832134	77.34918305	92.38589431	3.621730382	20.90437361	53.41522821	7.624695542	54.96065645	1.419040559
	76.08600036	81.56436488	102.1241365	110.4033531	104.4423171	60.52107319	10.39041681	57.08519831	59.07221029	77.40835465	73.29497016	108.5038363	132.9228222	77.28648677	89.87089597	8.072236165	25.29300665	53.77351168	8.072236165	-8.030840082	68.67610152	69.22421142	43.96845695	108.5038363	78.61116142	3.021914051	36.80111769	52.78000569	-11.1355463	65.96334186	8.527593077
	71.24871001	94.54169276	84.12796698	91.0629515	98.65841073	72.23942208	6.191950464	58.36945304	72.23942208	81.21307816	88.61786405	83.95720469	79.50464396	97.3374613	112.5283798	4.148606811	57.70897833	82.47678019	1.176470588	14.44788442	97.99793602	55.51411537	80.51615713	83.39095636	99.31888545	4.809081527	36.3880289	79.1744066	4.024767802	50.28720769	2.889576883
AMP1CPR2STR1ERY1TMP2	AMP	CPR	STR	ERY	TMP	AMP+CPR	AMP+STR	AMP+ERY	AMP+TMP	CPR+STR	CPR+ERY	CPR+TMP	STR+ERY	STR+TMP	ERY+TMP	AMP+CPR+STR	AMP+CPR+ERY	AMP+CPR+TMP	AMP+STR+ERY	AMP+STR+TMP	AMP+ERY+TMP	CPR+STR+ERY	CPR+STR+TMP	CPR+ERY+TMP	STR+ERY+TMP	AMP+CPR+STR+ERY	AMP+CPR+STR+TMP	AMP+CPR+ERY+TMP	AMP+STR+ERY+TMP	CPR+STR+ERY+TMP	AMP+CPR+STR+ERY+TMP
	77.83543365	79.50525784	99.50227682	101.535529	100.8577782	56.46510643	2.245049243	32.76501112	62.18362808	59.27280603	48.03041606	73.34504416	112.3795404	65.27586572	92.04701896	2.60510431	33.08270677	51.72085142	-2.880440538	19.52769247	47.31547178	53.72861371	45.10431456	81.96934331	85.60838717	3.621730382	6.50217092	65.95361644	8.344805676	61.42888081	8.365985386
	76.08600036	81.56436488	102.1241365	110.4033531	83.90986003	60.52107319	10.39041681	57.08519831	70.99428217	77.40835465	73.29497016	102.7067349	132.9228222	69.00727019	104.1111485	8.072236165	25.29300665	31.25404259	8.072236165	-6.706165429	54.76701767	69.22421142	84.71867008	106.7988065	95.16959458	3.021914051	3.891231792	61.7215596	-8.900157823	47.3998295	4.884737782
	71.24871001	94.54169276	84.12796698	91.0629515	100.6398349	72.23942208	6.191950464	58.36945304	73.23013416	81.21307816	88.61786405	90.57795443	79.50464396	87.76057792	107.244582	4.148606811	57.70897833	59.36016512	1.176470588	25.34571723	69.26728586	55.51411537	89.92459097	78.8609697	100.6398349	4.809081527	3.364293086	73.23013416	5.20123839	74.24386791	8.833849329
AMP1CPR2STR1ERY1TMP3	AMP	CPR	STR	ERY	TMP	AMP+CPR	AMP+STR	AMP+ERY	AMP+TMP	CPR+STR	CPR+ERY	CPR+TMP	STR+ERY	STR+TMP	ERY+TMP	AMP+CPR+STR	AMP+CPR+ERY	AMP+CPR+TMP	AMP+STR+ERY	AMP+STR+TMP	AMP+ERY+TMP	CPR+STR+ERY	CPR+STR+TMP	CPR+ERY+TMP	STR+ERY+TMP	AMP+CPR+STR+ERY	AMP+CPR+STR+TMP	AMP+CPR+ERY+TMP	AMP+STR+ERY+TMP	CPR+STR+ERY+TMP	AMP+CPR+STR+ERY+TMP
	77.83543365	79.50525784	99.50227682	101.535529	81.88075823	56.46510643	2.245049243	32.76501112	52.01736736	59.27280603	48.03041606	72.72902279	112.3795404	74.42550037	100.8577782	2.60510431	33.08270677	41.5545907	-2.880440538	13.42793604	56.80398179	53.72861371	56.19269918	89.66961041	92.38589431	3.621730382	4.130043418	55.44848036	17.11320555	10.76112328	10.06036217
	76.08600036	81.56436488	102.1241365	110.4033531	64.70207757	60.52107319	10.39041681	57.08519831	59.07221029	77.40835465	73.29497016	98.61466326	132.9228222	68.34493286	102.7864738	8.072236165	25.29300665	43.83845179	8.072236165	-7.906651833	54.76701767	69.22421142	77.40835465	110.2088662	92.85141393	3.021914051	2.359576725	48.47481307	-6.250808517	21.80306905	9.396910817
	71.24871001	94.54169276	84.12796698	91.0629515	105.9236326	72.23942208	6.191950464	58.36945304	69.59752322	81.21307816	88.61786405	78.73029701	79.50464396	91.0629515	113.5190918	4.148606811	57.70897833	70.25799794	1.176470588	15.43859649	77.85345717	55.51411537	88.53074892	96.28399532	108.8957688	4.809081527	4.685242518	80.8255934	4.210526316	58.65025998	9.969040248
AMP1CPR2STR1ERY2TMP1	AMP	CPR	STR	ERY	TMP	AMP+CPR	AMP+STR	AMP+ERY	AMP+TMP	CPR+STR	CPR+ERY	CPR+TMP	STR+ERY	STR+TMP	ERY+TMP	AMP+CPR+STR	AMP+CPR+ERY	AMP+CPR+TMP	AMP+STR+ERY	AMP+STR+TMP	AMP+ERY+TMP	CPR+STR+ERY	CPR+STR+TMP	CPR+ERY+TMP	STR+ERY+TMP	AMP+CPR+STR+ERY	AMP+CPR+STR+TMP	AMP+CPR+ERY+TMP	AMP+STR+ERY+TMP	CPR+STR+ERY+TMP	AMP+CPR+STR+ERY+TMP
	77.83543365	79.50525784	99.50227682	104.2465318	116.1071693	56.46510643	2.245049243	50.32299058	60.53161072	59.27280603	56.65471521	72.72902279	98.8245261	83.23625966	88.99714074	2.60510431	43.24896749	65.95361644	7.624695542	5.591443397	66.63136715	26.62367351	23.67832134	89.05358905	88.31939002	15.14349253	20.90437361	47.99322249	11.35232447	42.46697307	8.027110029
	76.08600036	81.56436488	102.1241365	119.344907	104.4423171	60.52107319	10.39041681	36.88390986	59.07221029	77.40835465	80.79710145	108.5038363	95.16959458	77.28648677	95.50076324	8.072236165	31.7093995	53.77351168	-3.518667046	-8.030840082	54.43584901	65.13213981	43.96845695	105.0937766	86.55920933	50.91718196	36.80111769	37.54624719	-3.394478797	61.53026428	2.235388476
	71.24871001	94.54169276	84.12796698	93.374613	98.65841073	72.23942208	6.191950464	56.3880289	72.23942208	81.21307816	86.39642828	83.95720469	80.8255934	97.3374613	113.1888545	4.148606811	61.67182663	82.47678019	2.827657379	14.44788442	96.34674923	52.37797076	80.51615713	64.22562818	111.2074303	44.499484	36.3880289	75.87203302	9.494324045	19.49200991	14.11764706
AMP1CPR2STR1ERY2TMP2	AMP	CPR	STR	ERY	TMP	AMP+CPR	AMP+STR	AMP+ERY	AMP+TMP	CPR+STR	CPR+ERY	CPR+TMP	STR+ERY	STR+TMP	ERY+TMP	AMP+CPR+STR	AMP+CPR+ERY	AMP+CPR+TMP	AMP+STR+ERY	AMP+STR+TMP	AMP+ERY+TMP	CPR+STR+ERY	CPR+STR+TMP	CPR+ERY+TMP	STR+ERY+TMP	AMP+CPR+STR+ERY	AMP+CPR+STR+TMP	AMP+CPR+ERY+TMP	AMP+STR+ERY+TMP	CPR+STR+ERY+TMP	AMP+CPR+STR+ERY+TMP
	77.83543365	79.50525784	99.50227682	104.2465318	100.8577782	56.46510643	2.245049243	50.32299058	62.18362808	59.27280603	56.65471521	73.34504416	98.8245261	65.27586572	96.79127396	2.60510431	43.24896749	51.72085142	7.624695542	19.52769247	53.37286879	26.62367351	45.10431456	85.66547152	88.31939002	15.14349253	6.50217092	45.62109499	8.302446256	4.600909594	6.332733242
	76.08600036	81.56436488	102.1241365	119.344907	83.90986003	60.52107319	10.39041681	36.88390986	70.99428217	77.40835465	80.79710145	102.7067349	95.16959458	69.00727019	104.7734858	8.072236165	31.7093995	31.25404259	-3.518667046	-6.706165429	55.09818633	65.13213981	84.71867008	95.88661552	90.20206463	50.91718196	3.891231792	53.56653127	16.14447233	55.73316283	2.442368891
	71.24871001	94.54169276	84.12796698	93.374613	100.6398349	72.23942208	6.191950464	56.3880289	73.23013416	81.21307816	86.39642828	90.57795443	80.8255934	87.76057792	111.8679051	4.148606811	61.67182663	59.36016512	2.827657379	25.34571723	97.00722394	52.37797076	89.92459097	88.61786405	103.6119711	44.499484	3.364293086	79.1744066	6.522187822	44.71183949	21.7131063
AMP1CPR2STR1ERY2TMP3	AMP	CPR	STR	ERY	TMP	AMP+CPR	AMP+STR	AMP+ERY	AMP+TMP	CPR+STR	CPR+ERY	CPR+TMP	STR+ERY	STR+TMP	ERY+TMP	AMP+CPR+STR	AMP+CPR+ERY	AMP+CPR+TMP	AMP+STR+ERY	AMP+STR+TMP	AMP+ERY+TMP	CPR+STR+ERY	CPR+STR+TMP	CPR+ERY+TMP	STR+ERY+TMP	AMP+CPR+STR+ERY	AMP+CPR+STR+TMP	AMP+CPR+ERY+TMP	AMP+STR+ERY+TMP	CPR+STR+ERY+TMP	AMP+CPR+STR+ERY+TMP
	77.83543365	79.50525784	99.50227682	104.2465318	81.88075823	56.46510643	2.245049243	50.32299058	52.01736736	59.27280603	56.65471521	72.72902279	98.8245261	74.42550037	100.1800275	2.60510431	43.24896749	41.5545907	7.624695542	13.42793604	61.88711215	26.62367351	56.19269918	102.9140698	89.3360161	15.14349253	4.130043418	51.72085142	5.591443397	41.54294102	4.299481097
	76.08600036	81.56436488	102.1241365	119.344907	64.70207757	60.52107319	10.39041681	36.88390986	59.07221029	77.40835465	80.79710145	98.61466326	95.16959458	68.34493286	113.7150397	8.072236165	31.7093995	43.83845179	-3.518667046	-7.906651833	66.35792088	65.13213981	77.40835465	96.56862745	109.4098471	50.91718196	2.359576725	45.94965201	-4.71915345	59.82523444	4.42938087
	71.24871001	94.54169276	84.12796698	93.374613	105.9236326	72.23942208	6.191950464	56.3880289	69.59752322	81.21307816	86.39642828	78.73029701	80.8255934	91.0629515	113.8493292	4.148606811	61.67182663	70.25799794	2.827657379	15.43859649	101.630547	52.37797076	88.53074892	73.63406202	117.4819401	44.499484	4.685242518	90.73271414	7.843137255	74.33098304	11.14551084
AMP1CPR2STR1ERY3TMP1	AMP	CPR	STR	ERY	TMP	AMP+CPR	AMP+STR	AMP+ERY	AMP+TMP	CPR+STR	CPR+ERY	CPR+TMP	STR+ERY	STR+TMP	ERY+TMP	AMP+CPR+STR	AMP+CPR+ERY	AMP+CPR+TMP	AMP+STR+ERY	AMP+STR+TMP	AMP+ERY+TMP	CPR+STR+ERY	CPR+STR+TMP	CPR+ERY+TMP	STR+ERY+TMP	AMP+CPR+STR+ERY	AMP+CPR+STR+TMP	AMP+CPR+ERY+TMP	AMP+STR+ERY+TMP	CPR+STR+ERY+TMP	AMP+CPR+STR+ERY+TMP
	77.83543365	79.50525784	99.50227682	111.7017897	116.1071693	56.46510643	2.245049243	55.40612094	60.53161072	59.27280603	61.64063816	72.72902279	75.10325109	83.23625966	100.1800275	2.60510431	62.52250344	65.95361644	14.74107805	5.591443397	75.7810018	36.30675939	23.67832134	72.4210121	92.38589431	4.807794133	20.90437361	70.69787144	-3.558191253	69.26390259	-1.122524621
	76.08600036	81.56436488	102.1241365	116.0332204	104.4423171	60.52107319	10.39041681	60.31409278	59.07221029	77.40835465	93.49957374	108.5038363	85.56570335	77.28648677	121.9942563	8.072236165	46.61198934	53.77351168	13.03976611	-8.030840082	72.65012548	66.83716965	43.96845695	103.7297528	111.0656904	10.51460506	36.80111769	52.78000569	-4.926133865	19.41602728	4.42938087
	71.24871001	94.54169276	84.12796698	92.05366357	98.65841073	72.23942208	6.191950464	65.44891641	72.23942208	81.21307816	112.1825062	83.95720469	78.18369453	97.3374613	119.4633643	4.148606811	61.15583075	82.47678019	21.32094943	14.44788442	112.8586171	66.22927613	80.51615713	85.13325892	115.8307534	13.06501548	36.3880289	84.12796698	12.13622291	13.5681812	3.157894737
AMP1CPR2STR1ERY3TMP2	AMP	CPR	STR	ERY	TMP	AMP+CPR	AMP+STR	AMP+ERY	AMP+TMP	CPR+STR	CPR+ERY	CPR+TMP	STR+ERY	STR+TMP	ERY+TMP	AMP+CPR+STR	AMP+CPR+ERY	AMP+CPR+TMP	AMP+STR+ERY	AMP+STR+TMP	AMP+ERY+TMP	CPR+STR+ERY	CPR+STR+TMP	CPR+ERY+TMP	STR+ERY+TMP	AMP+CPR+STR+ERY	AMP+CPR+STR+TMP	AMP+CPR+ERY+TMP	AMP+STR+ERY+TMP	CPR+STR+ERY+TMP	AMP+CPR+STR+ERY+TMP
	77.83543365	79.50525784	99.50227682	111.7017897	100.8577782	56.46510643	2.245049243	55.40612094	62.18362808	59.27280603	61.64063816	73.34504416	75.10325109	65.27586572	89.3360161	2.60510431	62.52250344	51.72085142	14.74107805	19.52769247	77.47537859	36.30675939	45.10431456	67.80085184	85.94726252	4.807794133	6.50217092	49.36990363	2.541565181	18.46139038	-8.916657842
	76.08600036	81.56436488	102.1241365	116.0332204	83.90986003	60.52107319	10.39041681	60.31409278	70.99428217	77.40835465	93.49957374	102.7067349	85.56570335	69.00727019	93.84491992	8.072236165	46.61198934	31.25404259	13.03976611	-6.706165429	72.65012548	66.83716965	84.71867008	89.7485081	101.1306305	10.51460506	3.891231792	61.39039093	2.028408062	9.185848252	14.69560943
	71.24871001	94.54169276	84.12796698	92.05366357	100.6398349	72.23942208	6.191950464	65.44891641	73.23013416	81.21307816	112.1825062	90.57795443	78.18369453	87.76057792	103.6119711	4.148606811	61.15583075	59.36016512	21.32094943	25.34571723	85.11867905	66.22927613	89.92459097	87.224022	103.9422085	13.06501548	3.364293086	90.07223942	8.173374613	8.341273514	6.996904025
AMP1CPR2STR1ERY3TMP3	AMP	CPR	STR	ERY	TMP	AMP+CPR	AMP+STR	AMP+ERY	AMP+TMP	CPR+STR	CPR+ERY	CPR+TMP	STR+ERY	STR+TMP	ERY+TMP	AMP+CPR+STR	AMP+CPR+ERY	AMP+CPR+TMP	AMP+STR+ERY	AMP+STR+TMP	AMP+ERY+TMP	CPR+STR+ERY	CPR+STR+TMP	CPR+ERY+TMP	STR+ERY+TMP	AMP+CPR+STR+ERY	AMP+CPR+STR+TMP	AMP+CPR+ERY+TMP	AMP+STR+ERY+TMP	CPR+STR+ERY+TMP	AMP+CPR+STR+ERY+TMP
	77.83543365	79.50525784	99.50227682	111.7017897	81.88075823	56.46510643	2.245049243	55.40612094	52.01736736	59.27280603	61.64063816	72.72902279	75.10325109	74.42550037	80.5252568	2.60510431	62.52250344	41.5545907	14.74107805	13.42793604	66.29249179	36.30675939	56.19269918	82.89337536	65.91125702	4.807794133	4.130043418	47.31547178	3.600550672	73.88406285	0.571852166
	76.08600036	81.56436488	102.1241365	116.0332204	64.70207757	60.52107319	10.39041681	60.31409278	59.07221029	77.40835465	93.49957374	98.61466326	85.56570335	68.34493286	128.6176296	8.072236165	46.61198934	43.83845179	13.03976611	-7.906651833	78.27999276	66.83716965	77.40835465	78.1543052	83.2475227	10.51460506	2.359576725	37.33926677	-8.362008745	1.683716965	10.72158547
	71.24871001	94.54169276	84.12796698	92.05366357	105.9236326	72.23942208	6.191950464	65.44891641	69.59752322	81.21307816	112.1825062	78.73029701	78.18369453	91.0629515	99.97936017	4.148606811	61.15583075	70.25799794	21.32094943	15.43859649	104.2724458	66.22927613	88.53074892	84.65412572	93.70485036	13.06501548	4.685242518	79.50464396	9.432404541	10.43203659	13.60165119
AMP1CPR2STR2ERY1TMP1	AMP	CPR	STR	ERY	TMP	AMP+CPR	AMP+STR	AMP+ERY	AMP+TMP	CPR+STR	CPR+ERY	CPR+TMP	STR+ERY	STR+TMP	ERY+TMP	AMP+CPR+STR	AMP+CPR+ERY	AMP+CPR+TMP	AMP+STR+ERY	AMP+STR+TMP	AMP+ERY+TMP	CPR+STR+ERY	CPR+STR+TMP	CPR+ERY+TMP	STR+ERY+TMP	AMP+CPR+STR+ERY	AMP+CPR+STR+TMP	AMP+CPR+ERY+TMP	AMP+STR+ERY+TMP	CPR+STR+ERY+TMP	AMP+CPR+STR+ERY+TMP
	77.83543365	79.50525784	68.10886253	101.535529	116.1071693	56.46510643	8.365985386	32.76501112	60.53161072	42.02420771	48.03041606	72.72902279	45.93209327	60.10058474	91.03039288	3.282855025	33.08270677	65.95361644	12.03007519	14.40220269	61.88711215	39.56012224	33.30365522	77.34918305	83.20138605	-1.122524621	0.741289844	53.41522821	8.641321614	51.88054961	-2.986339087
	76.08600036	81.56436488	78.75106564	110.4033531	104.4423171	60.52107319	7.078730175	57.08519831	59.07221029	62.74509804	73.29497016	108.5038363	78.1543052	102.0247229	89.87089597	2.773537554	25.29300665	53.77351168	24.54787716	17.80031565	68.67610152	54.56095482	71.6112532	108.5038363	110.8908781	7.327106673	24.87904582	52.78000569	-2.939121886	54.90196078	9.189930403
	71.24871001	94.54169276	67.2311001	91.0629515	98.65841073	72.23942208	8.111455108	58.36945304	72.23942208	53.4233523	88.61786405	83.95720469	81.6486538	77.81558816	112.5283798	4.809081527	57.70897833	82.47678019	11.41382869	40.14447884	97.99793602	68.05869382	75.98617047	83.39095636	75.02790406	4.478844169	2.889576883	79.1744066	34.86068111	56.82084229	3.694530444
AMP1CPR2STR2ERY1TMP2	AMP	CPR	STR	ERY	TMP	AMP+CPR	AMP+STR	AMP+ERY	AMP+TMP	CPR+STR	CPR+ERY	CPR+TMP	STR+ERY	STR+TMP	ERY+TMP	AMP+CPR+STR	AMP+CPR+ERY	AMP+CPR+TMP	AMP+STR+ERY	AMP+STR+TMP	AMP+ERY+TMP	CPR+STR+ERY	CPR+STR+TMP	CPR+ERY+TMP	STR+ERY+TMP	AMP+CPR+STR+ERY	AMP+CPR+STR+TMP	AMP+CPR+ERY+TMP	AMP+STR+ERY+TMP	CPR+STR+ERY+TMP	AMP+CPR+STR+ERY+TMP
	77.83543365	79.50525784	68.10886253	101.535529	100.8577782	56.46510643	8.365985386	32.76501112	62.18362808	42.02420771	48.03041606	73.34504416	45.93209327	64.662993	92.04701896	3.282855025	33.08270677	51.72085142	12.03007519	15.41882876	47.31547178	39.56012224	68.10886253	81.96934331	86.89751426	-1.122524621	-0.275336228	65.95361644	12.7078259	57.11673124	26.15694165
	76.08600036	81.56436488	78.75106564	110.4033531	83.90986003	60.52107319	7.078730175	57.08519831	70.99428217	62.74509804	73.29497016	102.7067349	78.1543052	97.25063939	104.1111485	2.773537554	25.29300665	31.25404259	24.54787716	1.57305115	54.76701767	54.56095482	63.76811594	106.7988065	109.8678602	7.327106673	10.51460506	61.7215596	-4.926133865	44.33077579	9.521099066
	71.24871001	94.54169276	67.2311001	91.0629515	100.6398349	72.23942208	8.111455108	58.36945304	73.23013416	53.4233523	88.61786405	90.57795443	81.6486538	86.74488879	107.244582	4.809081527	57.70897833	59.36016512	11.41382869	3.364293086	69.26728586	68.05869382	76.33463099	78.8609697	191.2830425	4.478844169	10.42311662	73.23013416	5.20123839	71.45618381	3.364293086
AMP1CPR2STR2ERY1TMP3	AMP	CPR	STR	ERY	TMP	AMP+CPR	AMP+STR	AMP+ERY	AMP+TMP	CPR+STR	CPR+ERY	CPR+TMP	STR+ERY	STR+TMP	ERY+TMP	AMP+CPR+STR	AMP+CPR+ERY	AMP+CPR+TMP	AMP+STR+ERY	AMP+STR+TMP	AMP+ERY+TMP	CPR+STR+ERY	CPR+STR+TMP	CPR+ERY+TMP	STR+ERY+TMP	AMP+CPR+STR+ERY	AMP+CPR+STR+TMP	AMP+CPR+ERY+TMP	AMP+STR+ERY+TMP	CPR+STR+ERY+TMP	AMP+CPR+STR+ERY+TMP
	77.83543365	79.50525784	68.10886253	101.535529	81.88075823	56.46510643	8.365985386	32.76501112	52.01736736	42.02420771	48.03041606	72.72902279	45.93209327	78.2154631	100.8577782	3.282855025	33.08270677	41.5545907	12.03007519	9.319072329	56.80398179	39.56012224	41.10017566	89.66961041	74.26907621	-1.122524621	-2.308588372	55.44848036	15.75770412	48.1844214	-3.325214445
	76.08600036	81.56436488	78.75106564	110.4033531	64.70207757	60.52107319	7.078730175	57.08519831	59.07221029	62.74509804	73.29497016	98.61466326	78.1543052	89.06649616	102.7864738	2.773537554	25.29300665	43.83845179	24.54787716	-7.368502755	54.76701767	54.56095482	55.24296675	110.2088662	114.3009378	7.327106673	6.871749761	48.47481307	-8.568989159	42.62574595	9.521099066
	71.24871001	94.54169276	67.2311001	91.0629515	105.9236326	72.23942208	8.111455108	58.36945304	69.59752322	53.4233523	88.61786405	78.73029701	81.6486538	93.71409904	113.5190918	4.809081527	57.70897833	70.25799794	11.41382869	6.336429309	77.85345717	68.05869382	63.09313152	96.28399532	106.3893502	4.478844169	34.73684211	80.8255934	25.28379773	57.86622383	4.809081527
AMP1CPR2STR2ERY2TMP1	AMP	CPR	STR	ERY	TMP	AMP+CPR	AMP+STR	AMP+ERY	AMP+TMP	CPR+STR	CPR+ERY	CPR+TMP	STR+ERY	STR+TMP	ERY+TMP	AMP+CPR+STR	AMP+CPR+ERY	AMP+CPR+TMP	AMP+STR+ERY	AMP+STR+TMP	AMP+ERY+TMP	CPR+STR+ERY	CPR+STR+TMP	CPR+ERY+TMP	STR+ERY+TMP	AMP+CPR+STR+ERY	AMP+CPR+STR+TMP	AMP+CPR+ERY+TMP	AMP+STR+ERY+TMP	CPR+STR+ERY+TMP	AMP+CPR+STR+ERY+TMP
	77.83543365	79.50525784	68.10886253	104.2465318	116.1071693	56.46510643	8.365985386	50.32299058	60.53161072	42.02420771	56.65471521	72.72902279	46.49036263	60.10058474	88.99714074	3.282855025	43.24896749	65.95361644	15.41882876	14.40220269	66.63136715	38.84784753	33.30365522	89.05358905	86.89751426	29.54569522	0.741289844	47.99322249	17.11320555	60.81285945	-0.783649264
	76.08600036	81.56436488	78.75106564	119.344907	104.4423171	60.52107319	7.078730175	36.88390986	59.07221029	62.74509804	80.79710145	108.5038363	74.31798806	102.0247229	95.50076324	2.773537554	31.7093995	53.77351168	2.111200228	17.80031565	54.43584901	55.64791134	71.6112532	105.0937766	103.0477408	8.196424413	24.87904582	37.54624719	-4.71915345	53.87894288	2.56655714
	71.24871001	94.54169276	67.2311001	93.374613	98.65841073	72.23942208	8.111455108	56.3880289	72.23942208	53.4233523	86.39642828	83.95720469	72.10954727	77.81558816	113.1888545	4.809081527	61.67182663	82.47678019	8.111455108	40.14447884	96.34674923	73.98252253	75.98617047	64.22562818	96.28399532	2.167182663	2.889576883	75.87203302	7.512899897	68.3200392	2.167182663
AMP1CPR2STR2ERY2TMP2	AMP	CPR	STR	ERY	TMP	AMP+CPR	AMP+STR	AMP+ERY	AMP+TMP	CPR+STR	CPR+ERY	CPR+TMP	STR+ERY	STR+TMP	ERY+TMP	AMP+CPR+STR	AMP+CPR+ERY	AMP+CPR+TMP	AMP+STR+ERY	AMP+STR+TMP	AMP+ERY+TMP	CPR+STR+ERY	CPR+STR+TMP	CPR+ERY+TMP	STR+ERY+TMP	AMP+CPR+STR+ERY	AMP+CPR+STR+TMP	AMP+CPR+ERY+TMP	AMP+STR+ERY+TMP	CPR+STR+ERY+TMP	AMP+CPR+STR+ERY+TMP
	77.83543365	79.50525784	68.10886253	104.2465318	100.8577782	56.46510643	8.365985386	50.32299058	62.18362808	42.02420771	56.65471521	73.34504416	46.49036263	64.662993	96.79127396	3.282855025	43.24896749	51.72085142	15.41882876	15.41882876	53.37286879	38.84784753	68.10886253	85.66547152	87.51353563	29.54569522	-0.275336228	45.62109499	6.269194112	65.64477705	1.24960288
	76.08600036	81.56436488	78.75106564	119.344907	83.90986003	60.52107319	7.078730175	36.88390986	70.99428217	62.74509804	80.79710145	102.7067349	74.31798806	97.25063939	104.7734858	2.773537554	31.7093995	31.25404259	2.111200228	1.57305115	55.09818633	55.64791134	63.76811594	95.88661552	87.02046036	8.196424413	10.51460506	53.56653127	-5.71265944	48.76385337	1.697239399
	71.24871001	94.54169276	67.2311001	93.374613	100.6398349	72.23942208	8.111455108	56.3880289	73.23013416	53.4233523	86.39642828	90.57795443	72.10954727	86.74488879	111.8679051	4.809081527	61.67182663	59.36016512	8.111455108	3.364293086	97.00722394	73.98252253	76.33463099	88.61786405	68.40715433	2.167182663	10.42311662	79.1744066	14.11764706	58.56314486	6.522187822
AMP1CPR2STR2ERY2TMP3	AMP	CPR	STR	ERY	TMP	AMP+CPR	AMP+STR	AMP+ERY	AMP+TMP	CPR+STR	CPR+ERY	CPR+TMP	STR+ERY	STR+TMP	ERY+TMP	AMP+CPR+STR	AMP+CPR+ERY	AMP+CPR+TMP	AMP+STR+ERY	AMP+STR+TMP	AMP+ERY+TMP	CPR+STR+ERY	CPR+STR+TMP	CPR+ERY+TMP	STR+ERY+TMP	AMP+CPR+STR+ERY	AMP+CPR+STR+TMP	AMP+CPR+ERY+TMP	AMP+STR+ERY+TMP	CPR+STR+ERY+TMP	AMP+CPR+STR+ERY+TMP
	77.83543365	79.50525784	68.10886253	104.2465318	81.88075823	56.46510643	8.365985386	50.32299058	52.01736736	42.02420771	56.65471521	72.72902279	46.49036263	78.2154631	100.1800275	3.282855025	43.24896749	41.5545907	15.41882876	9.319072329	61.88711215	38.84784753	41.10017566	102.9140698	87.20552494	29.54569522	-2.308588372	51.72085142	50.04765435	39.56012224	4.977231812
	76.08600036	81.56436488	78.75106564	119.344907	64.70207757	60.52107319	7.078730175	36.88390986	59.07221029	62.74509804	80.79710145	98.61466326	74.31798806	89.06649616	113.7150397	2.773537554	31.7093995	43.83845179	2.111200228	-7.368502755	66.35792088	55.64791134	55.24296675	96.56862745	110.5498721	8.196424413	6.871749761	45.94965201	-5.381490776	60.35805627	2.56655714
	71.24871001	94.54169276	67.2311001	93.374613	105.9236326	72.23942208	8.111455108	56.3880289	69.59752322	53.4233523	86.39642828	78.73029701	72.10954727	93.71409904	113.8493292	4.809081527	61.67182663	70.25799794	8.111455108	6.336429309	101.630547	73.98252253	63.09313152	73.63406202	98.02629788	2.167182663	34.73684211	90.73271414	10.15479876	88.87920943	2.167182663
AMP1CPR2STR2ERY3TMP1	AMP	CPR	STR	ERY	TMP	AMP+CPR	AMP+STR	AMP+ERY	AMP+TMP	CPR+STR	CPR+ERY	CPR+TMP	STR+ERY	STR+TMP	ERY+TMP	AMP+CPR+STR	AMP+CPR+ERY	AMP+CPR+TMP	AMP+STR+ERY	AMP+STR+TMP	AMP+ERY+TMP	CPR+STR+ERY	CPR+STR+TMP	CPR+ERY+TMP	STR+ERY+TMP	AMP+CPR+STR+ERY	AMP+CPR+STR+TMP	AMP+CPR+ERY+TMP	AMP+STR+ERY+TMP	CPR+STR+ERY+TMP	AMP+CPR+STR+ERY+TMP
	77.83543365	79.50525784	68.10886253	111.7017897	116.1071693	56.46510643	8.365985386	55.40612094	60.53161072	42.02420771	61.64063816	72.72902279	50.24424285	60.10058474	100.1800275	3.282855025	62.52250344	65.95361644	17.79095626	14.40220269	75.7810018	83.20138605	33.30365522	72.4210121	69.28315326	-0.275336228	0.741289844	70.69787144	8.196547707	45.41232524	39.71195595
	76.08600036	81.56436488	78.75106564	116.0332204	104.4423171	60.52107319	7.078730175	60.31409278	59.07221029	62.74509804	93.49957374	108.5038363	75	102.0247229	121.9942563	2.773537554	46.61198934	53.77351168	-7.823859667	17.80031565	72.65012548	92.13554987	71.6112532	103.7297528	105.4347826	12.50161703	24.87904582	52.78000569	4.015420041	71.95225916	2.690745388
	71.24871001	94.54169276	67.2311001	92.05366357	98.65841073	72.23942208	8.111455108	65.44891641	72.23942208	53.4233523	112.1825062	83.95720469	75.94261291	77.81558816	119.4633643	4.809081527	61.15583075	82.47678019	3.488132095	40.14447884	112.8586171	87.92094302	75.98617047	85.13325892	80.95173277	4.478844169	2.889576883	84.12796698	11.14551084	86.43998584	3.034055728
AMP1CPR2STR2ERY3TMP2	AMP	CPR	STR	ERY	TMP	AMP+CPR	AMP+STR	AMP+ERY	AMP+TMP	CPR+STR	CPR+ERY	CPR+TMP	STR+ERY	STR+TMP	ERY+TMP	AMP+CPR+STR	AMP+CPR+ERY	AMP+CPR+TMP	AMP+STR+ERY	AMP+STR+TMP	AMP+ERY+TMP	CPR+STR+ERY	CPR+STR+TMP	CPR+ERY+TMP	STR+ERY+TMP	AMP+CPR+STR+ERY	AMP+CPR+STR+TMP	AMP+CPR+ERY+TMP	AMP+STR+ERY+TMP	CPR+STR+ERY+TMP	AMP+CPR+STR+ERY+TMP
	77.83543365	79.50525784	68.10886253	111.7017897	100.8577782	56.46510643	8.365985386	55.40612094	62.18362808	42.02420771	61.64063816	73.34504416	50.24424285	64.662993	89.3360161	3.282855025	62.52250344	51.72085142	17.79095626	15.41882876	77.47537859	83.20138605	68.10886253	67.80085184	81.29556994	-0.275336228	-0.275336228	49.36990363	1.228423171	60.19683808	-0.444773907
	76.08600036	81.56436488	78.75106564	116.0332204	83.90986003	60.52107319	7.078730175	60.31409278	70.99428217	62.74509804	93.49957374	102.7067349	75	97.25063939	93.84491992	2.773537554	46.61198934	31.25404259	-7.823859667	1.57305115	72.65012548	92.13554987	63.76811594	89.7485081	79.85933504	12.50161703	10.51460506	61.39039093	3.353082715	63.91730605	3.684251378
	71.24871001	94.54169276	67.2311001	92.05366357	100.6398349	72.23942208	8.111455108	65.44891641	73.23013416	53.4233523	112.1825062	90.57795443	75.94261291	86.74488879	103.6119711	4.809081527	61.15583075	59.36016512	3.488132095	3.364293086	85.11867905	87.92094302	76.33463099	87.224022	76.63953393	4.478844169	10.42311662	90.07223942	7.843137255	88.53074892	4.148606811
AMP1CPR2STR2ERY3TMP3	AMP	CPR	STR	ERY	TMP	AMP+CPR	AMP+STR	AMP+ERY	AMP+TMP	CPR+STR	CPR+ERY	CPR+TMP	STR+ERY	STR+TMP	ERY+TMP	AMP+CPR+STR	AMP+CPR+ERY	AMP+CPR+TMP	AMP+STR+ERY	AMP+STR+TMP	AMP+ERY+TMP	CPR+STR+ERY	CPR+STR+TMP	CPR+ERY+TMP	STR+ERY+TMP	AMP+CPR+STR+ERY	AMP+CPR+STR+TMP	AMP+CPR+ERY+TMP	AMP+STR+ERY+TMP	CPR+STR+ERY+TMP	AMP+CPR+STR+ERY+TMP
	77.83543365	79.50525784	68.10886253	111.7017897	81.88075823	56.46510643	8.365985386	55.40612094	52.01736736	42.02420771	61.64063816	72.72902279	50.24424285	78.2154631	80.5252568	3.282855025	62.52250344	41.5545907	17.79095626	9.319072329	66.29249179	83.20138605	41.10017566	82.89337536	73.96106552	-0.275336228	-2.308588372	47.31547178	5.972678174	66.35705176	-2.139150694
	76.08600036	81.56436488	78.75106564	116.0332204	64.70207757	60.52107319	7.078730175	60.31409278	59.07221029	62.74509804	93.49957374	98.61466326	75	89.06649616	128.6176296	2.773537554	46.61198934	43.83845179	-7.823859667	-7.368502755	78.27999276	92.13554987	55.24296675	78.1543052	81.22335891	12.50161703	6.871749761	37.33926677	3.767043544	66.49616368	13.95047994
	71.24871001	94.54169276	67.2311001	92.05366357	105.9236326	72.23942208	8.111455108	65.44891641	69.59752322	53.4233523	112.1825062	78.73029701	75.94261291	93.71409904	99.97936017	4.809081527	61.15583075	70.25799794	3.488132095	6.336429309	104.2724458	87.92094302	63.09313152	84.65412572	82.69403534	4.478844169	34.73684211	79.50464396	6.191950464	82.2584597	5.015479876
AMP1CPR2STR3ERY1TMP1	AMP	CPR	STR	ERY	TMP	AMP+CPR	AMP+STR	AMP+ERY	AMP+TMP	CPR+STR	CPR+ERY	CPR+TMP	STR+ERY	STR+TMP	ERY+TMP	AMP+CPR+STR	AMP+CPR+ERY	AMP+CPR+TMP	AMP+STR+ERY	AMP+STR+TMP	AMP+ERY+TMP	CPR+STR+ERY	CPR+STR+TMP	CPR+ERY+TMP	STR+ERY+TMP	AMP+CPR+STR+ERY	AMP+CPR+STR+TMP	AMP+CPR+ERY+TMP	AMP+STR+ERY+TMP	CPR+STR+ERY+TMP	AMP+CPR+STR+ERY+TMP
	77.83543365	79.50525784	74.20542334	101.535529	116.1071693	56.46510643	5.591443397	32.76501112	60.53161072	65.95278774	48.03041606	72.72902279	97.01871727	108.9645875	91.03039288	8.344805676	33.08270677	65.95361644	2.202689823	33.71809806	61.88711215	34.84370864	80.12127921	77.34918305	109.2964173	16.32955629	1.419040559	53.41522821	30.03282855	61.02461679	6.989304247
	76.08600036	81.56436488	60.83351516	110.4033531	104.4423171	60.52107319	22.43667693	57.08519831	59.07221029	79.51832907	73.29497016	108.5038363	81.43137683	106.9168667	89.87089597	14.61281726	25.29300665	53.77351168	11.05275413	17.6761274	68.67610152	62.80903666	78.1543052	108.5038363	116.6921231	34.81410572	16.80680966	52.78000569	26.6176813	79.85933504	33.03407415
	71.24871001	94.54169276	64.81466508	91.0629515	98.65841073	72.23942208	23.30237358	58.36945304	72.23942208	90.0117061	88.61786405	83.95720469	42.93255403	51.80368013	112.5283798	15.43859649	57.70897833	82.47678019	18.67905057	45.49019608	97.99793602	99.76860044	74.67944355	83.39095636	73.79668026	12.79669763	17.42002064	79.1744066	35.91331269	59.34718101	40.35087719
AMP1CPR2STR3ERY1TMP2	AMP	CPR	STR	ERY	TMP	AMP+CPR	AMP+STR	AMP+ERY	AMP+TMP	CPR+STR	CPR+ERY	CPR+TMP	STR+ERY	STR+TMP	ERY+TMP	AMP+CPR+STR	AMP+CPR+ERY	AMP+CPR+TMP	AMP+STR+ERY	AMP+STR+TMP	AMP+ERY+TMP	CPR+STR+ERY	CPR+STR+TMP	CPR+ERY+TMP	STR+ERY+TMP	AMP+CPR+STR+ERY	AMP+CPR+STR+TMP	AMP+CPR+ERY+TMP	AMP+STR+ERY+TMP	CPR+STR+ERY+TMP	AMP+CPR+STR+ERY+TMP
	77.83543365	79.50525784	74.20542334	101.535529	100.8577782	56.46510643	5.591443397	32.76501112	62.18362808	65.95278774	48.03041606	73.34504416	97.01871727	90.7139524	92.04701896	8.344805676	33.08270677	51.72085142	2.202689823	27.61834163	47.31547178	34.84370864	83.81740742	81.96934331	108.6327578	16.32955629	10.37805782	65.95361644	11.35232447	75.50111895	11.24642592
	76.08600036	81.56436488	60.83351516	110.4033531	83.90986003	60.52107319	22.43667693	57.08519831	70.99428217	79.51832907	73.29497016	102.7067349	81.43137683	104.1239363	104.1111485	14.61281726	25.29300665	31.25404259	11.05275413	5.547075108	54.76701767	62.80903666	93.49957374	106.7988065	116.6921231	34.81410572	22.10550827	61.7215596	10.05924814	95.54560955	33.69641148
	71.24871001	94.54169276	64.81466508	91.0629515	100.6398349	72.23942208	23.30237358	58.36945304	73.23013416	90.0117061	88.61786405	90.57795443	42.93255403	58.12435748	107.244582	15.43859649	57.70897833	59.36016512	18.67905057	24.16924665	69.26728586	99.76860044	68.05869382	78.8609697	80.00646853	12.79669763	18.88544892	73.23013416	28.31785346	72.58868048	30.11351909
AMP1CPR2STR3ERY1TMP3	AMP	CPR	STR	ERY	TMP	AMP+CPR	AMP+STR	AMP+ERY	AMP+TMP	CPR+STR	CPR+ERY	CPR+TMP	STR+ERY	STR+TMP	ERY+TMP	AMP+CPR+STR	AMP+CPR+ERY	AMP+CPR+TMP	AMP+STR+ERY	AMP+STR+TMP	AMP+ERY+TMP	CPR+STR+ERY	CPR+STR+TMP	CPR+ERY+TMP	STR+ERY+TMP	AMP+CPR+STR+ERY	AMP+CPR+STR+TMP	AMP+CPR+ERY+TMP	AMP+STR+ERY+TMP	CPR+STR+ERY+TMP	AMP+CPR+STR+ERY+TMP
	77.83543365	79.50525784	74.20542334	101.535529	81.88075823	56.46510643	5.591443397	32.76501112	52.01736736	65.95278774	48.03041606	72.72902279	97.01871727	91.4605693	100.8577782	8.344805676	33.08270677	41.5545907	2.202689823	20.84083448	56.80398179	34.84370864	75.80912963	89.66961041	107.9690984	16.32955629	9.022556391	55.44848036	19.82420841	79.19724715	3.452292704
	76.08600036	81.56436488	60.83351516	110.4033531	64.70207757	60.52107319	22.43667693	57.08519831	59.07221029	79.51832907	73.29497016	98.61466326	81.43137683	102.0292385	102.7864738	14.61281726	25.29300665	43.83845179	11.05275413	0.041396083	54.76701767	62.80903666	75.42625746	110.2088662	109.3606808	34.81410572	19.45615896	48.47481307	4.346588704	77.1312873	15.81330367
	71.24871001	94.54169276	64.81466508	91.0629515	105.9236326	72.23942208	23.30237358	58.36945304	69.59752322	90.0117061	88.61786405	78.73029701	42.93255403	70.72874915	113.5190918	15.43859649	57.70897833	70.25799794	18.67905057	20.86687307	77.85345717	99.76860044	87.224022	96.28399532	75.57090548	12.79669763	21.52734778	80.8255934	26.81114551	85.48171943	18.34881321
AMP1CPR2STR3ERY2TMP1	AMP	CPR	STR	ERY	TMP	AMP+CPR	AMP+STR	AMP+ERY	AMP+TMP	CPR+STR	CPR+ERY	CPR+TMP	STR+ERY	STR+TMP	ERY+TMP	AMP+CPR+STR	AMP+CPR+ERY	AMP+CPR+TMP	AMP+STR+ERY	AMP+STR+TMP	AMP+ERY+TMP	CPR+STR+ERY	CPR+STR+TMP	CPR+ERY+TMP	STR+ERY+TMP	AMP+CPR+STR+ERY	AMP+CPR+STR+TMP	AMP+CPR+ERY+TMP	AMP+STR+ERY+TMP	CPR+STR+ERY+TMP	AMP+CPR+STR+ERY+TMP
	77.83543365	79.50525784	74.20542334	104.2465318	116.1071693	56.46510643	5.591443397	50.32299058	60.53161072	65.95278774	56.65471521	72.72902279	97.68237673	108.9645875	88.99714074	8.344805676	43.24896749	65.95361644	27.95721699	33.71809806	66.63136715	37.86606348	80.12127921	89.05358905	103.9871416	24.12368951	1.419040559	47.99322249	26.60171556	83.81740742	8.683681034
	76.08600036	81.56436488	60.83351516	119.344907	104.4423171	60.52107319	22.43667693	36.88390986	59.07221029	79.51832907	80.79710145	108.5038363	88.76281911	106.9168667	95.50076324	14.61281726	31.7093995	53.77351168	35.8076117	17.6761274	54.43584901	85.99744246	78.1543052	105.0937766	111.1062623	4.015420041	16.80680966	37.54624719	9.396910817	72.01619778	52.90419394
	71.24871001	94.54169276	64.81466508	93.374613	98.65841073	72.23942208	23.30237358	56.3880289	72.23942208	90.0117061	86.39642828	83.95720469	64.81466508	51.80368013	113.1888545	15.43859649	61.67182663	82.47678019	8.771929825	45.49019608	96.34674923	89.66324558	74.67944355	64.22562818	73.09438278	15.10835913	17.42002064	75.87203302	42.1878225	55.16565486	22.1878225
AMP1CPR2STR3ERY2TMP2	AMP	CPR	STR	ERY	TMP	AMP+CPR	AMP+STR	AMP+ERY	AMP+TMP	CPR+STR	CPR+ERY	CPR+TMP	STR+ERY	STR+TMP	ERY+TMP	AMP+CPR+STR	AMP+CPR+ERY	AMP+CPR+TMP	AMP+STR+ERY	AMP+STR+TMP	AMP+ERY+TMP	CPR+STR+ERY	CPR+STR+TMP	CPR+ERY+TMP	STR+ERY+TMP	AMP+CPR+STR+ERY	AMP+CPR+STR+TMP	AMP+CPR+ERY+TMP	AMP+STR+ERY+TMP	CPR+STR+ERY+TMP	AMP+CPR+STR+ERY+TMP
	77.83543365	79.50525784	74.20542334	104.2465318	100.8577782	56.46510643	5.591443397	50.32299058	62.18362808	65.95278774	56.65471521	73.34504416	97.68237673	90.7139524	96.79127396	8.344805676	43.24896749	51.72085142	27.95721699	27.61834163	53.37286879	37.86606348	83.81740742	85.66547152	106.6417794	24.12368951	10.37805782	45.62109499	15.41882876	90.59364247	4.638356455
	76.08600036	81.56436488	60.83351516	119.344907	83.90986003	60.52107319	22.43667693	36.88390986	70.99428217	79.51832907	80.79710145	102.7067349	88.76281911	104.1239363	104.7734858	14.61281726	31.7093995	31.25404259	35.8076117	5.547075108	55.09818633	85.99744246	93.49957374	95.88661552	106.5677504	4.015420041	22.10550827	53.56653127	1.448862901	84.63341858	19.91151587
	71.24871001	94.54169276	64.81466508	93.374613	100.6398349	72.23942208	23.30237358	56.3880289	73.23013416	90.0117061	86.39642828	90.57795443	64.81466508	58.12435748	111.8679051	15.43859649	61.67182663	59.36016512	8.771929825	24.16924665	97.00722394	89.66324558	68.05869382	88.61786405	77.93653911	15.10835913	18.88544892	79.1744066	26.99690402	63.52870716	10.42311662
AMP1CPR2STR3ERY2TMP3	AMP	CPR	STR	ERY	TMP	AMP+CPR	AMP+STR	AMP+ERY	AMP+TMP	CPR+STR	CPR+ERY	CPR+TMP	STR+ERY	STR+TMP	ERY+TMP	AMP+CPR+STR	AMP+CPR+ERY	AMP+CPR+TMP	AMP+STR+ERY	AMP+STR+TMP	AMP+ERY+TMP	CPR+STR+ERY	CPR+STR+TMP	CPR+ERY+TMP	STR+ERY+TMP	AMP+CPR+STR+ERY	AMP+CPR+STR+TMP	AMP+CPR+ERY+TMP	AMP+STR+ERY+TMP	CPR+STR+ERY+TMP	AMP+CPR+STR+ERY+TMP
	77.83543365	79.50525784	74.20542334	104.2465318	81.88075823	56.46510643	5.591443397	50.32299058	52.01736736	65.95278774	56.65471521	72.72902279	97.68237673	91.4605693	100.1800275	8.344805676	43.24896749	41.5545907	27.95721699	20.84083448	61.88711215	37.86606348	75.80912963	102.9140698	107.6372686	24.12368951	9.022556391	51.72085142	34.4382082	76.425151	16.1389389
	76.08600036	81.56436488	60.83351516	119.344907	64.70207757	60.52107319	22.43667693	36.88390986	59.07221029	79.51832907	80.79710145	98.61466326	88.76281911	102.0292385	113.7150397	14.61281726	31.7093995	43.83845179	35.8076117	0.041396083	66.35792088	85.99744246	75.42625746	96.56862745	103.0765874	4.015420041	19.45615896	45.94965201	23.63716333	72.35720375	31.37823083
	71.24871001	94.54169276	64.81466508	93.374613	105.9236326	72.23942208	23.30237358	56.3880289	69.59752322	90.0117061	86.39642828	78.73029701	64.81466508	70.72874915	113.8493292	15.43859649	61.67182663	70.25799794	8.771929825	20.86687307	101.630547	89.66324558	87.224022	73.63406202	88.58189043	15.10835913	21.52734778	90.73271414	45.15995872	63.87716767	40.20639835
AMP1CPR2STR3ERY3TMP1	AMP	CPR	STR	ERY	TMP	AMP+CPR	AMP+STR	AMP+ERY	AMP+TMP	CPR+STR	CPR+ERY	CPR+TMP	STR+ERY	STR+TMP	ERY+TMP	AMP+CPR+STR	AMP+CPR+ERY	AMP+CPR+TMP	AMP+STR+ERY	AMP+STR+TMP	AMP+ERY+TMP	CPR+STR+ERY	CPR+STR+TMP	CPR+ERY+TMP	STR+ERY+TMP	AMP+CPR+STR+ERY	AMP+CPR+STR+TMP	AMP+CPR+ERY+TMP	AMP+STR+ERY+TMP	CPR+STR+ERY+TMP	AMP+CPR+STR+ERY+TMP
	77.83543365	79.50525784	74.20542334	111.7017897	116.1071693	56.46510643	5.591443397	55.40612094	60.53161072	65.95278774	61.64063816	72.72902279	92.37310105	108.9645875	100.1800275	8.344805676	62.52250344	65.95361644	19.14645769	33.71809806	75.7810018	72.11300142	80.12127921	72.4210121	100.6688443	14.74107805	1.419040559	70.69787144	45.95997035	73.96106552	23.61537647
	76.08600036	81.56436488	60.83351516	116.0332204	104.4423171	60.52107319	22.43667693	60.31409278	59.07221029	79.51832907	93.49957374	108.5038363	95.0469125	106.9168667	121.9942563	14.61281726	46.61198934	53.77351168	9.976455978	17.6761274	72.65012548	89.06649616	78.1543052	103.7297528	105.8695178	5.215906445	16.80680966	52.78000569	56.09169232	66.5601023	38.0016041
	71.24871001	94.54169276	64.81466508	92.05366357	98.65841073	72.23942208	23.30237358	65.44891641	72.23942208	90.0117061	112.1825062	83.95720469	67.47600291	51.80368013	119.4633643	15.43859649	61.15583075	82.47678019	20.99071207	45.49019608	112.8586171	78.8609697	74.67944355	85.13325892	84.14632738	15.76883385	17.42002064	84.12796698	68.60681115	69.10407535	34.40660475
AMP1CPR2STR3ERY3TMP2	AMP	CPR	STR	ERY	TMP	AMP+CPR	AMP+STR	AMP+ERY	AMP+TMP	CPR+STR	CPR+ERY	CPR+TMP	STR+ERY	STR+TMP	ERY+TMP	AMP+CPR+STR	AMP+CPR+ERY	AMP+CPR+TMP	AMP+STR+ERY	AMP+STR+TMP	AMP+ERY+TMP	CPR+STR+ERY	CPR+STR+TMP	CPR+ERY+TMP	STR+ERY+TMP	AMP+CPR+STR+ERY	AMP+CPR+STR+TMP	AMP+CPR+ERY+TMP	AMP+STR+ERY+TMP	CPR+STR+ERY+TMP	AMP+CPR+STR+ERY+TMP
	77.83543365	79.50525784	74.20542334	111.7017897	100.8577782	56.46510643	5.591443397	55.40612094	62.18362808	65.95278774	61.64063816	73.34504416	92.37310105	90.7139524	89.3360161	8.344805676	62.52250344	51.72085142	19.14645769	27.61834163	77.47537859	72.11300142	83.81740742	67.80085184	74.45429564	14.74107805	10.37805782	49.36990363	31.04945462	53.94037106	29.37625755
	76.08600036	81.56436488	60.83351516	116.0332204	83.90986003	60.52107319	22.43667693	60.31409278	70.99428217	79.51832907	93.49957374	102.7067349	95.0469125	104.1239363	93.84491992	14.61281726	46.61198934	31.25404259	9.976455978	5.547075108	72.65012548	89.06649616	93.49957374	89.7485081	92.95221471	5.215906445	22.10550827	61.39039093	35.14527438	84.97442455	18.79382163
	71.24871001	94.54169276	64.81466508	92.05366357	100.6398349	72.23942208	23.30237358	65.44891641	73.23013416	90.0117061	112.1825062	90.57795443	67.47600291	58.12435748	103.6119711	15.43859649	61.15583075	59.36016512	20.99071207	24.16924665	85.11867905	78.8609697	68.05869382	87.224022	85.03343999	15.76883385	18.88544892	90.07223942	39.54592363	85.83017995	29.63880289
AMP1CPR2STR3ERY3TMP3	AMP	CPR	STR	ERY	TMP	AMP+CPR	AMP+STR	AMP+ERY	AMP+TMP	CPR+STR	CPR+ERY	CPR+TMP	STR+ERY	STR+TMP	ERY+TMP	AMP+CPR+STR	AMP+CPR+ERY	AMP+CPR+TMP	AMP+STR+ERY	AMP+STR+TMP	AMP+ERY+TMP	CPR+STR+ERY	CPR+STR+TMP	CPR+ERY+TMP	STR+ERY+TMP	AMP+CPR+STR+ERY	AMP+CPR+STR+TMP	AMP+CPR+ERY+TMP	AMP+STR+ERY+TMP	CPR+STR+ERY+TMP	AMP+CPR+STR+ERY+TMP
	77.83543365	79.50525784	74.20542334	111.7017897	81.88075823	56.46510643	5.591443397	55.40612094	52.01736736	65.95278774	61.64063816	72.72902279	92.37310105	91.4605693	80.5252568	8.344805676	62.52250344	41.5545907	19.14645769	20.84083448	66.29249179	72.11300142	75.80912963	82.89337536	91.70944159	14.74107805	9.022556391	47.31547178	37.48808641	70.88095868	15.14349253
	76.08600036	81.56436488	60.83351516	116.0332204	64.70207757	60.52107319	22.43667693	60.31409278	59.07221029	79.51832907	93.49957374	98.61466326	95.0469125	102.0292385	128.6176296	14.61281726	46.61198934	43.83845179	9.976455978	0.041396083	78.27999276	89.06649616	75.42625746	78.1543052	98.1889592	5.215906445	19.45615896	37.33926677	29.92936793	45.75873828	16.59982924
	71.24871001	94.54169276	64.81466508	92.05366357	105.9236326	72.23942208	23.30237358	65.44891641	69.59752322	90.0117061	112.1825062	78.73029701	67.47600291	70.72874915	99.97936017	15.43859649	61.15583075	70.25799794	20.99071207	20.86687307	104.2724458	78.8609697	87.224022	84.65412572	77.3451307	15.76883385	21.52734778	79.50464396	28.31785346	65.61947023	5.469556244
AMP1CPR3STR1ERY1TMP1	AMP	CPR	STR	ERY	TMP	AMP+CPR	AMP+STR	AMP+ERY	AMP+TMP	CPR+STR	CPR+ERY	CPR+TMP	STR+ERY	STR+TMP	ERY+TMP	AMP+CPR+STR	AMP+CPR+ERY	AMP+CPR+TMP	AMP+STR+ERY	AMP+STR+TMP	AMP+ERY+TMP	CPR+STR+ERY	CPR+STR+TMP	CPR+ERY+TMP	STR+ERY+TMP	AMP+CPR+STR+ERY	AMP+CPR+STR+TMP	AMP+CPR+ERY+TMP	AMP+STR+ERY+TMP	CPR+STR+ERY+TMP	AMP+CPR+STR+ERY+TMP
	77.83543365	86.40016591	99.50227682	101.535529	116.1071693	84.59176109	2.245049243	32.76501112	60.53161072	83.80774615	105.6462902	74.12246591	112.3795404	83.23625966	91.03039288	12.07243461	65.61474108	66.29249179	-2.880440538	5.591443397	61.88711215	88.7851921	32.39487738	94.69590916	92.38589431	12.263052	11.35232447	51.04310071	7.624695542	28.08109089	4.574817325
	76.08600036	75.49639974	102.1241365	110.4033531	104.4423171	88.87738998	10.39041681	57.08519831	59.07221029	63.05913157	92.25398211	102.0292385	132.9228222	77.28648677	89.87089597	-2.069804145	76.62414944	47.15013842	8.072236165	-8.030840082	68.67610152	80.86406284	79.27121973	113.5500764	78.61116142	6.995938009	0.041396083	75.96181211	-11.1355463	56.57866027	4.015420041
	71.24871001	49.1423423	84.12796698	91.0629515	98.65841073	89.41176471	6.191950464	58.36945304	72.23942208	40.67780948	52.09938433	54.87161124	79.50464396	97.3374613	112.5283798	23.30237358	72.23942208	54.07636739	1.176470588	14.44788442	97.99793602	57.66231966	40.2712162	78.52794751	99.31888545	18.34881321	3.364293086	86.43962848	4.024767802	52.0439398	24.16924665
AMP1CPR3STR1ERY1TMP2	AMP	CPR	STR	ERY	TMP	AMP+CPR	AMP+STR	AMP+ERY	AMP+TMP	CPR+STR	CPR+ERY	CPR+TMP	STR+ERY	STR+TMP	ERY+TMP	AMP+CPR+STR	AMP+CPR+ERY	AMP+CPR+TMP	AMP+STR+ERY	AMP+STR+TMP	AMP+ERY+TMP	CPR+STR+ERY	CPR+STR+TMP	CPR+ERY+TMP	STR+ERY+TMP	AMP+CPR+STR+ERY	AMP+CPR+STR+TMP	AMP+CPR+ERY+TMP	AMP+STR+ERY+TMP	CPR+STR+ERY+TMP	AMP+CPR+STR+ERY+TMP
	77.83543365	86.40016591	99.50227682	101.535529	100.8577782	84.59176109	2.245049243	32.76501112	62.18362808	83.80774615	105.6462902	94.44703686	112.3795404	65.27586572	92.04701896	12.07243461	65.61474108	78.49200466	-2.880440538	19.52769247	47.31547178	88.7851921	59.25234614	92.04127132	85.60838717	12.263052	14.06332733	52.05972678	8.344805676	47.30647586	-0.508313036
	76.08600036	75.49639974	102.1241365	110.4033531	83.90986003	88.87738998	10.39041681	57.08519831	70.99428217	63.05913157	92.25398211	104.4730526	132.9228222	69.00727019	104.1111485	-2.069804145	76.62414944	61.39039093	8.072236165	-6.706165429	54.76701767	80.86406284	85.55531311	105.8695178	95.16959458	6.995938009	17.92450389	67.02025821	-8.900157823	45.40693869	13.61931127
	71.24871001	49.1423423	84.12796698	91.0629515	100.6398349	89.41176471	6.191950464	58.36945304	73.23013416	40.67780948	52.09938433	68.06741132	79.50464396	87.76057792	107.244582	23.30237358	72.23942208	62.00206398	1.176470588	25.34571723	69.26728586	57.66231966	67.42055837	71.72675083	100.6398349	18.34881321	8.441692466	84.45820433	5.20123839	50.26971458	4.685242518
AMP1CPR3STR1ERY1TMP3	AMP	CPR	STR	ERY	TMP	AMP+CPR	AMP+STR	AMP+ERY	AMP+TMP	CPR+STR	CPR+ERY	CPR+TMP	STR+ERY	STR+TMP	ERY+TMP	AMP+CPR+STR	AMP+CPR+ERY	AMP+CPR+TMP	AMP+STR+ERY	AMP+STR+TMP	AMP+ERY+TMP	CPR+STR+ERY	CPR+STR+TMP	CPR+ERY+TMP	STR+ERY+TMP	AMP+CPR+STR+ERY	AMP+CPR+STR+TMP	AMP+CPR+ERY+TMP	AMP+STR+ERY+TMP	CPR+STR+ERY+TMP	AMP+CPR+STR+ERY+TMP
	77.83543365	86.40016591	99.50227682	101.535529	81.88075823	84.59176109	2.245049243	32.76501112	52.01736736	83.80774615	105.6462902	105.0655882	112.3795404	74.42550037	100.8577782	12.07243461	65.61474108	57.82060786	-2.880440538	13.42793604	56.80398179	88.7851921	74.84834344	101.6643335	92.38589431	12.263052	11.35232447	55.109605	17.11320555	21.11266656	11.69119983
	76.08600036	75.49639974	102.1241365	110.4033531	64.70207757	88.87738998	10.39041681	57.08519831	59.07221029	63.05913157	92.25398211	102.3783548	132.9228222	68.34493286	102.7864738	-2.069804145	76.62414944	76.29298078	8.072236165	-7.906651833	54.76701767	80.86406284	84.50796422	111.4553786	92.85141393	6.995938009	14.61281726	43.17611446	-6.250808517	26.27100153	3.353082715
	71.24871001	49.1423423	84.12796698	91.0629515	105.9236326	89.41176471	6.191950464	58.36945304	69.59752322	40.67780948	52.09938433	63.04043986	79.50464396	91.0629515	113.5190918	23.30237358	72.23942208	69.92776058	1.176470588	15.43859649	77.85345717	57.66231966	44.94703892	71.13534243	108.8957688	18.34881321	4.685242518	78.18369453	4.210526316	37.31417417	4.35500516
AMP1CPR3STR1ERY2TMP1	AMP	CPR	STR	ERY	TMP	AMP+CPR	AMP+STR	AMP+ERY	AMP+TMP	CPR+STR	CPR+ERY	CPR+TMP	STR+ERY	STR+TMP	ERY+TMP	AMP+CPR+STR	AMP+CPR+ERY	AMP+CPR+TMP	AMP+STR+ERY	AMP+STR+TMP	AMP+ERY+TMP	CPR+STR+ERY	CPR+STR+TMP	CPR+ERY+TMP	STR+ERY+TMP	AMP+CPR+STR+ERY	AMP+CPR+STR+TMP	AMP+CPR+ERY+TMP	AMP+STR+ERY+TMP	CPR+STR+ERY+TMP	AMP+CPR+STR+ERY+TMP
	77.83543365	86.40016591	99.50227682	104.2465318	116.1071693	84.59176109	2.245049243	50.32299058	60.53161072	83.80774615	106.9736092	74.12246591	98.8245261	83.23625966	88.99714074	12.07243461	76.79762787	66.29249179	7.624695542	5.591443397	66.63136715	70.53455696	32.39487738	101.000674	88.31939002	20.8831939	11.35232447	51.38197607	11.35232447	80.82127858	-0.847188394
	76.08600036	75.49639974	102.1241365	119.344907	104.4423171	88.87738998	10.39041681	36.88390986	59.07221029	63.05913157	91.55574951	102.0292385	95.16959458	77.28648677	95.50076324	-2.069804145	67.35142687	47.15013842	-3.518667046	-8.030840082	54.43584901	64.45559677	79.27121973	110.757146	86.55920933	18.25567256	0.041396083	42.51377713	-3.394478797	42.26489199	1.697239399
	71.24871001	49.1423423	84.12796698	93.374613	98.65841073	89.41176471	6.191950464	56.3880289	72.23942208	40.67780948	60.08339783	54.87161124	80.8255934	97.3374613	113.1888545	23.30237358	88.09081527	54.07636739	2.827657379	14.44788442	96.34674923	44.22625992	40.2712162	80.19128366	111.2074303	8.111455108	3.364293086	85.11867905	9.494324045	47.01696834	1.382868937
AMP1CPR3STR1ERY2TMP2	AMP	CPR	STR	ERY	TMP	AMP+CPR	AMP+STR	AMP+ERY	AMP+TMP	CPR+STR	CPR+ERY	CPR+TMP	STR+ERY	STR+TMP	ERY+TMP	AMP+CPR+STR	AMP+CPR+ERY	AMP+CPR+TMP	AMP+STR+ERY	AMP+STR+TMP	AMP+ERY+TMP	CPR+STR+ERY	CPR+STR+TMP	CPR+ERY+TMP	STR+ERY+TMP	AMP+CPR+STR+ERY	AMP+CPR+STR+TMP	AMP+CPR+ERY+TMP	AMP+STR+ERY+TMP	CPR+STR+ERY+TMP	AMP+CPR+STR+ERY+TMP
	77.83543365	86.40016591	99.50227682	104.2465318	100.8577782	84.59176109	2.245049243	50.32299058	62.18362808	83.80774615	106.9736092	94.44703686	98.8245261	65.27586572	96.79127396	12.07243461	76.79762787	78.49200466	7.624695542	19.52769247	53.37286879	70.53455696	59.25234614	90.7139524	88.31939002	20.8831939	14.06332733	57.10049772	8.302446256	64.56162182	1.186063751
	76.08600036	75.49639974	102.1241365	119.344907	83.90986003	88.87738998	10.39041681	36.88390986	70.99428217	63.05913157	91.55574951	104.4730526	95.16959458	69.00727019	104.7734858	-2.069804145	67.35142687	61.39039093	-3.518667046	-6.706165429	55.09818633	64.45559677	85.55531311	103.0765874	90.20206463	18.25567256	17.92450389	158.8781662	16.14447233	15.6229544	1.034902072
	71.24871001	49.1423423	84.12796698	93.374613	100.6398349	89.41176471	6.191950464	56.3880289	73.23013416	40.67780948	60.08339783	68.06741132	80.8255934	87.76057792	111.8679051	23.30237358	88.09081527	62.00206398	2.827657379	25.34571723	97.00722394	44.22625992	67.42055837	77.6408349	103.6119711	8.111455108	8.441692466	89.74200206	6.522187822	56.77520705	5.531475748

AMP1CPR3STR1ERY2TMP3	AMP	CPR	STR	ERY	TMP	AMP+CPR	AMP+STR	AMP+ERY	[illegible]
	77.83543365	86.40016591	99.50227682	104.2465318	81.88075823	84.59176109	2.245049243	50.32299058	[illegible]
	76.08600036	75.49639974	102.1241365	119.344907	64.70207757	88.87738998	10.39041681	36.88390986	[illegible]
	71.24871001	49.1423423	84.12796698	93.374613	105.9236326	89.41176471	6.191950464	56.3880289	[illegible]
AMP1CPR3STR1ERY3TMP1	AMP	CPR	STR	ERY	TMP	AMP+CPR	AMP+STR	AMP+ERY	[illegible]
	77.83543365	86.40016591	99.50227682	111.7017897	116.1071693	84.59176109	2.245049243	55.40612094	[illegible]
	76.08600036	75.49639974	102.1241365	116.0332204	104.4423171	88.87738998	10.39041681	60.31409278	[illegible]
	71.24871001	49.1423423	84.12796698	92.05366357	98.65841073	89.41176471	6.191950464	65.44891641	[illegible]
AMP1CPR3STR1ERY3TMP2	AMP	CPR	STR	ERY	TMP	AMP+CPR	AMP+STR	AMP+ERY	[illegible]
	77.83543365	86.40016591	99.50227682	111.7017897	100.8577782	84.59176109	2.245049243	55.40612094	[illegible]
	76.08600036	75.49639974	102.1241365	116.0332204	83.90986003	88.87738998	10.39041681	60.31409278	[illegible]
	71.24871001	49.1423423	84.12796698	92.05366357	100.6398349	89.41176471	6.191950464	65.44891641	[illegible]
AMP1CPR3STR1ERY3TMP3	AMP	CPR	STR	ERY	TMP	AMP+CPR	AMP+STR	AMP+ERY	[illegible]
	77.83543365	86.40016591	99.50227682	111.7017897	81.88075823	84.59176109	2.245049243	55.40612094	[illegible]
	76.08600036	75.49639974	102.1241365	116.0332204	64.70207757	88.87738998	10.39041681	60.31409278	[illegible]
	71.24871001	49.1423423	84.12796698	92.05366357	105.9236326	89.41176471	6.191950464	65.44891641	[illegible]

[illegible]

	70.66920134	102.552155	74.20542334	111.7017897	100.8577782	56.50070987	32.62988185	68.10886253	84.43342878	100.8577782	76.45875252	74.42550037	92.37310105	90.7139524	89.3360161	17.53735833	69.34090526	61.33262748	20.0014438	37.86606348	70.515196	75.44212644	69.34237001	87.98051467	74.45429564	19.44317444	36.99978343	83.20138605	20.0014438	66.29249179	56.40445653
	85.31543052	110.7345217	60.83351516	116.0332204	83.90986003	70.31116795	38.51236147	104.6675192	93.84057971	103.1176425	83.2475227	71.32545083	95.0469125	104.1239363	93.84491992	23.93435635	85.571185	74.31798806	59.65473146	45.33248082	79.1773231	80.5981734	45.4942951	93.51375126	92.95221471	36.12531969	34.76129582	46.44075021	35.86956522	64.70207757	31.71355499
	91.75400866	98.65841073	64.81466508	92.05366357	100.6398349	81.99711431	41.92415539	112.1825062	86.39642828	89.74200206	94.69556244	105.5933953	67.47600291	58.12435748	103.6119711	34.60648463	86.74488879	75.02790406	60.61035037	27.89861977	76.0732856	88.42105263	88.42105263	116.1609907	85.03343999	35.52119348	39.83339232	54.81719435	56.2110364	80.8255934	34.60648463
AMP2CPR1STR3ERY3TMP3	AMP	CPR	STR	ERY	TMP	AMP+CPR	AMP+STR	AMP+ERY	AMP+TMP	CPR+STR	CPR+ERY	CPR+TMP	STR+ERY	STR+TMP	ERY+TMP	AMP+CPR+STR	AMP+CPR+ERY	AMP+CPR+TMP	AMP+STR+ERY	AMP+STR+TMP	AMP+ERY+TMP	CPR+STR+ERY	CPR+STR+TMP	CPR+ERY+TMP	STR+ERY+TMP	AMP+CPR+STR+ERY	AMP+CPR+STR+TMP	AMP+CPR+ERY+TMP	AMP+STR+ERY+TMP	CPR+STR+ERY+TMP	AMP+CPR+STR+ERY+TMP
	70.66920134	102.552155	74.20542334	111.7017897	81.88075823	56.50070987	32.62988185	68.10886253	76.11714031	100.8577782	76.45875252	76.11987716	92.37310105	91.4605693	80.5252568	17.53735833	69.34090526	79.19724715	20.0014438	49.93623216	82.277354	75.44212644	67.30911787	82.21963359	91.70944159	19.44317444	30.83956975	71.49698005	47.78015737	39.1824632	29.08775898
	85.31543052	110.7345217	60.83351516	116.0332204	64.70207757	70.31116795	38.51236147	104.6675192	94.18158568	103.1176425	83.2475227	87.55271532	95.0469125	102.0292385	128.6176296	23.93435635	85.571185	78.75106564	59.65473146	48.48678602	68.26513214	80.5981734	75.63064345	80.26700473	98.1889592	36.12531969	41.58141517	55.64791134	43.03069054	57.08519831	40.57971014
	91.75400866	98.65841073	64.81466508	92.05366357	105.9236326	81.99711431	41.92415539	112.1825062	88.83565187	89.74200206	94.69556244	89.74200206	67.47600291	70.72874915	99.97936017	34.60648463	86.74488879	52.72643127	60.61035037	27.85506221	79.20943021	88.42105263	79.50464396	116.1609907	77.3451307	35.52119348	36.34878719	63.18024664	26.11275964	58.36945304	31.47034002
AMP2CPR2STR1ERY1TMP1	AMP	CPR	STR	ERY	TMP	AMP+CPR	AMP+STR	AMP+ERY	AMP+TMP	CPR+STR	CPR+ERY	CPR+TMP	STR+ERY	STR+TMP	ERY+TMP	AMP+CPR+STR	AMP+CPR+ERY	AMP+CPR+TMP	AMP+STR+ERY	AMP+STR+TMP	AMP+ERY+TMP	CPR+STR+ERY	CPR+STR+TMP	CPR+ERY+TMP	STR+ERY+TMP	AMP+CPR+STR+ERY	AMP+CPR+STR+TMP	AMP+CPR+ERY+TMP	AMP+STR+ERY+TMP	CPR+STR+ERY+TMP	AMP+CPR+STR+ERY+TMP
	70.66920134	79.50525784	99.50227682	101.535529	116.1071693	61.89089684	30.93582309	64.04697163	85.66547152	59.27280603	48.03041606	72.72902279	112.3795404	83.23625966	91.03039288	11.06913396	67.18483047	89.36159973	9.837091224	34.1506846	75.50111895	53.72861371	23.67832134	77.34918305	92.38589431	19.38542243	1.193541401	84.74143947	9.817840556	54.96065645	-4.273648242
	85.31543052	81.56436488	102.1241365	110.4033531	104.4423171	72.95396419	23.52941176	82.50213129	103.0477408	77.40835465	73.29497016	108.5038363	132.9228222	77.28648677	89.87089597	22.14407502	96.14236999	78.49531117	43.28644501	34.07928389	95.20460358	69.22421142	43.96845695	108.5038363	78.61116142	42.60443308	29.64620631	122.3998295	22.48508099	65.96334186	41.92242114
	91.75400866	94.54169276	84.12796698	91.0629515	98.65841073	99.07167942	22.06190619	82.91182316	83.04249585	81.21307816	88.61786405	83.95720469	79.50464396	97.3374613	112.5283798	13.35039338	93.49631122	78.73029701	40.74810116	49.80807448	82.21490213	55.51411537	80.51615713	83.39095636	99.31888545	42.49040372	19.84047042	75.02790406	8.384831078	50.28720769	28.90044374
AMP2CPR2STR1ERY1TMP2	AMP	CPR	STR	ERY	TMP	AMP+CPR	AMP+STR	AMP+ERY	AMP+TMP	CPR+STR	CPR+ERY	CPR+TMP	STR+ERY	STR+TMP	ERY+TMP	AMP+CPR+STR	AMP+CPR+ERY	AMP+CPR+TMP	AMP+STR+ERY	AMP+STR+TMP	AMP+ERY+TMP	CPR+STR+ERY	CPR+STR+TMP	CPR+ERY+TMP	STR+ERY+TMP	AMP+CPR+STR+ERY	AMP+CPR+STR+TMP	AMP+CPR+ERY+TMP	AMP+STR+ERY+TMP	CPR+STR+ERY+TMP	AMP+CPR+STR+ERY+TMP
	70.66920134	79.50525784	99.50227682	101.535529	100.8577782	61.89089684	30.93582309	64.04697163	84.43342878	59.27280603	48.03041606	73.34504416	112.3795404	65.27586572	92.04701896	11.06913396	67.18483047	76.73316168	9.837091224	3.368866858	80.73730058	53.72861371	45.10431456	81.96934331	85.60838717	19.38542243	12.3011767	71.18896937	-1.867314772	61.42888081	-0.962533388
	85.31543052	81.56436488	102.1241365	110.4033531	83.90986003	72.95396419	23.52941176	82.50213129	93.84057971	77.40835465	73.29497016	102.7067349	132.9228222	69.00727019	104.1111485	22.14407502	96.14236999	81.90537084	43.28644501	32.71526002	70.31116795	69.22421142	84.71867008	106.7988065	95.16959458	42.60443308	30.32821824	86.67945439	23.16709292	47.3998295	19.07502131
	91.75400866	94.54169276	84.12796698	91.0629515	100.6398349	99.07167942	22.06190619	82.91182316	86.39642828	81.21307816	88.61786405	90.57795443	79.50464396	87.76057792	107.244582	13.35039338	93.49631122	72.93714099	40.74810116	49.80807448	77.46712765	55.51411537	89.92459097	78.8609697	100.6398349	42.49040372	13.5681812	59.91342934	16.3558653	74.24386791	5.205128903
AMP2CPR2STR1ERY1TMP3	AMP	CPR	STR	ERY	TMP	AMP+CPR	AMP+STR	AMP+ERY	AMP+TMP	CPR+STR	CPR+ERY	CPR+TMP	STR+ERY	STR+TMP	ERY+TMP	AMP+CPR+STR	AMP+CPR+ERY	AMP+CPR+TMP	AMP+STR+ERY	AMP+STR+TMP	AMP+ERY+TMP	CPR+STR+ERY	CPR+STR+TMP	CPR+ERY+TMP	STR+ERY+TMP	AMP+CPR+STR+ERY	AMP+CPR+STR+TMP	AMP+CPR+ERY+TMP	AMP+STR+ERY+TMP	CPR+STR+ERY+TMP	AMP+CPR+STR+ERY+TMP
	70.66920134	79.50525784	99.50227682	101.535529	81.88075823	61.89089684	30.93582309	64.04697163	76.11714031	59.27280603	48.03041606	72.72902279	112.3795404	74.42550037	100.8577782	11.06913396	67.18483047	74.57708689	9.837091224	15.38128354	70.88095868	53.72861371	56.19269918	89.66961041	92.38589431	19.38542243	6.814736386	77.65719373	-7.661765768	10.76112328	0.442765358
	85.31543052	81.56436488	102.1241365	110.4033531	64.70207757	72.95396419	23.52941176	82.50213129	94.18158568	77.40835465	73.29497016	98.61466326	132.9228222	68.34493286	102.7864738	22.14407502	96.14236999	78.49531117	43.28644501	44.65046888	69.62915601	69.22421142	77.40835465	110.2088662	92.85141393	42.60443308	37.1483376	76.10826939	22.14407502	21.80306905	7.310315431
	91.75400866	94.54169276	84.12796698	91.0629515	105.9236326	99.07167942	22.06190619	82.91182316	88.83565187	81.21307816	88.61786405	78.73029701	79.50464396	91.0629515	113.5190918	13.35039338	93.49631122	65.96793074	40.74810116	44.5811668	74.67944355	55.51411537	88.53074892	96.28399532	108.8957688	42.49040372	17.74970735	65.83725805	30.99120682	58.65025998	7.64435249
AMP2CPR2STR1ERY2TMP1	AMP	CPR	STR	ERY	TMP	AMP+CPR	AMP+STR	AMP+ERY	AMP+TMP	CPR+STR	CPR+ERY	CPR+TMP	STR+ERY	STR+TMP	ERY+TMP	AMP+CPR+STR	AMP+CPR+ERY	AMP+CPR+TMP	AMP+STR+ERY	AMP+STR+TMP	AMP+ERY+TMP	CPR+STR+ERY	CPR+STR+TMP	CPR+ERY+TMP	STR+ERY+TMP	AMP+CPR+STR+ERY	AMP+CPR+STR+TMP	AMP+CPR+ERY+TMP	AMP+STR+ERY+TMP	CPR+STR+ERY+TMP	AMP+CPR+STR+ERY+TMP
	70.66920134	79.50525784	99.50227682	104.2465318	116.1071693	61.89089684	30.93582309	52.34256563	85.66547152	59.27280603	56.65471521	72.72902279	98.8245261	83.23625966	88.99714074	11.06913396	64.41273432	89.36159973	16.28606492	34.1506846	71.80499074	26.62367351	23.67832134	89.05358905	88.31939002	8.605048488	1.193541401	65.95278774	2.444834805	42.46697307	0.750776043
	85.31543052	81.56436488	102.1241365	119.344907	104.4423171	72.95396419	23.52941176	95.46035806	103.0477408	77.40835465	80.79710145	108.5038363	95.16959458	77.28648677	95.50076324	22.14407502	106.0315431	78.49531117	45.67348679	34.07928389	75	65.13213981	43.96845695	105.0937766	86.55920933	52.83461211	29.64620631	69.97016198	22.14407502	61.53026428	9.207161125
	91.75400866	94.54169276	84.12796698	93.374613	98.65841073	99.07167942	22.06190619	97.89562519	83.04249585	81.21307816	86.39642828	83.95720469	80.8255934	97.3374613	113.1888545	13.35039338	97.89562519	78.73029701	50.156535	49.80807448	75.94261291	52.37797076	80.51615713	64.22562818	111.2074303	57.51776332	19.84047042	71.06416574	29.24890426	19.49200991	6.03272262
AMP2CPR2STR1ERY2TMP2	AMP	CPR	STR	ERY	TMP	AMP+CPR	AMP+STR	AMP+ERY	AMP+TMP	CPR+STR	CPR+ERY	CPR+TMP	STR+ERY	STR+TMP	ERY+TMP	AMP+CPR+STR	AMP+CPR+ERY	AMP+CPR+TMP	AMP+STR+ERY	AMP+STR+TMP	AMP+ERY+TMP	CPR+STR+ERY	CPR+STR+TMP	CPR+ERY+TMP	STR+ERY+TMP	AMP+CPR+STR+ERY	AMP+CPR+STR+TMP	AMP+CPR+ERY+TMP	AMP+STR+ERY+TMP	CPR+STR+ERY+TMP	AMP+CPR+STR+ERY+TMP
	70.66920134	79.50525784	99.50227682	104.2465318	100.8577782	61.89089684	30.93582309	52.34256563	84.43342878	59.27280603	56.65471521	73.34504416	98.8245261	65.27586572	96.79127396	11.06913396	64.41273432	76.73316168	16.28606492	3.368866858	65.02875568	26.62367351	45.10431456	85.66547152	88.31939002	8.605048488	12.3011767	70.572948	12.28192603	4.600909594	2.290829463
	85.31543052	81.56436488	102.1241365	119.344907	83.90986003	72.95396419	23.52941176	95.46035806	93.84057971	77.40835465	80.79710145	102.7067349	95.16959458	69.00727019	104.7734858	22.14407502	106.0315431	81.90537084	45.67348679	32.71526002	69.88491049	65.13213981	84.71867008	95.88661552	90.20206463	52.83461211	30.32821824	51.89684569	24.19011083	55.73316283	6.798806479
	91.75400866	94.54169276	84.12796698	93.374613	100.6398349	99.07167942	22.06190619	97.89562519	86.39642828	81.21307816	86.39642828	90.57795443	80.8255934	87.76057792	111.8679051	13.35039338	97.89562519	72.93714099	50.156535	49.80807448	67.01331228	52.37797076	89.92459097	88.61786405	103.6119711	57.51776332	13.5681812	77.68491547	28.68265592	44.71183949	9.168867231
AMP2CPR2STR1ERY2TMP3	AMP	CPR	STR	ERY	TMP	AMP+CPR	AMP+STR	AMP+ERY	AMP+TMP	CPR+STR	CPR+ERY	CPR+TMP	STR+ERY	STR+TMP	ERY+TMP	AMP+CPR+STR	AMP+CPR+ERY	AMP+CPR+TMP	AMP+STR+ERY	AMP+STR+TMP	AMP+ERY+TMP	CPR+STR+ERY	CPR+STR+TMP	CPR+ERY+TMP	STR+ERY+TMP	AMP+CPR+STR+ERY	AMP+CPR+STR+TMP	AMP+CPR+ERY+TMP	AMP+STR+ERY+TMP	CPR+STR+ERY+TMP	AMP+CPR+STR+ERY+TMP
	70.66920134	79.50525784	99.50227682	104.2465318	81.88075823	61.89089684	30.93582309	52.34256563	76.11714031	59.27280603	56.65471521	72.72902279	98.8245261	74.42550037	100.1800275	11.06913396	64.41273432	74.57708689	16.28606492	15.38128354	60.71660611	26.62367351	56.19269918	102.9140698	89.3360161	8.605048488	6.814736386	74.57708689	2.117573453	41.54294102	-2.021320115
	85.31543052	81.56436488	102.1241365	119.344907	64.70207757	72.95396419	23.52941176	95.46035806	94.18158568	77.40835465	80.79710145	98.61466326	95.16959458	68.34493286	113.7150397	22.14407502	106.0315431	78.49531117	45.67348679	44.65046888	79.51832907	65.13213981	77.40835465	96.56862745	109.4098471	52.83461211	37.1483376	63.15004263	29.30520034	59.82523444	5.94629156
	91.75400866	94.54169276	84.12796698	93.374613	105.9236326	99.07167942	22.06190619	97.89562519	88.83565187	81.21307816	86.39642828	78.73029701	80.8255934	91.0629515	113.8493292	13.35039338	97.89562519	65.96793074	50.156535	44.5811668	65.48879754	52.37797076	88.53074892	73.63406202	117.4819401	57.51776332	17.74970735	68.62494215	33.08196989	74.33098304	14.74423543
AMP2CPR2STR1ERY3TMP1	AMP	CPR	STR	ERY	TMP	AMP+CPR	AMP+STR	AMP+ERY	AMP+TMP	CPR+STR	CPR+ERY	CPR+TMP	STR+ERY	STR+TMP	ERY+TMP	AMP+CPR+STR	AMP+CPR+ERY	AMP+CPR+TMP	AMP+STR+ERY	AMP+STR+TMP	AMP+ERY+TMP	CPR+STR+ERY	CPR+STR+TMP	CPR+ERY+TMP	STR+ERY+TMP	AMP+CPR+STR+ERY	AMP+CPR+STR+TMP	AMP+CPR+ERY+TMP	AMP+STR+ERY+TMP	CPR+STR+ERY+TMP	AMP+CPR+STR+ERY+TMP
	70.66920134	79.50525784	99.50227682	111.7017897	116.1071693	61.89089684	30.93582309	68.10886253	85.66547152	59.27280603	61.64063816	72.72902279	75.10325109	83.23625966	100.1800275	11.06913396	64.41273432	89.36159973	31.45559112	34.1506846	58.25252063	36.30675939	23.67832134	72.4210121	92.38589431	1.501552085	1.193541401	60.40859542	-0.654522704	69.26390259	-2.425584137
	85.31543052	81.56436488	102.1241365	116.0332204	104.4423171	72.95396419	23.52941176	104.6675192	103.0477408	77.40835465	93.49957374	108.5038363	85.56570335	77.28648677	121.9942563	22.14407502	103.3034953	78.49531117	41.58141517	34.07928389	81.22335891	66.83716965	43.96845695	103.7297528	111.0656904	39.1943734	29.64620631	62.12702472	29.13469736	19.41602728	5.115089514
	91.75400866	94.54169276	84.12796698	92.05366357	98.65841073	99.07167942	22.06190619	112.1825062	83.04249585	81.21307816	112.1825062	83.95720469	78.18369453	97.3374613	119.4633643	13.35039338	97.19870416	78.73029701	51.55037705	49.80807448	79.55789072	66.22927613	80.51615713	85.13325892	115.8307534	32.03658835	19.84047042	70.71570522	25.76429913	13.5681812	24.41401465
AMP2CPR2STR1ERY3TMP2	AMP	CPR	STR	ERY	TMP	AMP+CPR	AMP+STR	AMP+ERY	AMP+TMP	CPR+STR	CPR+ERY	CPR+TMP	STR+ERY	STR+TMP	ERY+TMP	AMP+CPR+STR	AMP+CPR+ERY	AMP+CPR+TMP	AMP+STR+ERY	AMP+STR+TMP	AMP+ERY+TMP	CPR+STR+ERY	CPR+STR+TMP	CPR+ERY+TMP	STR+ERY+TMP	AMP+CPR+STR+ERY	AMP+CPR+STR+TMP	AMP+CPR+ERY+TMP	AMP+STR+ERY+TMP	CPR+STR+ERY+TMP	AMP+CPR+STR+ERY+TMP
	70.66920134	79.50525784	99.50227682	111.7017897	100.8577782	61.89089684	30.93582309	68.10886253	84.43342878	59.27280603	61.64063816	73.34504416	75.10325109	65.27586572	89.3360161	11.06913396	64.41273432	76.73316168	31.45559112	3.368866858	70.515196	36.30675939	45.10431456	67.80085184	85.94726252	1.501552085	12.3011767	47.47214669	0.577520033	18.46139038	1.809562769
	85.31543052	81.56436488	102.1241365	116.0332204	83.90986003	72.95396419	23.52941176	104.6675192	93.84057971	77.40835465	93.49957374	102.7067349	85.56570335	69.00727019	93.84491992	22.14407502	103.3034953	81.90537084	41.58141517	32.71526002	79.1773231	66.83716965	84.71867008	89.7485081	101.1306305	39.1943734	30.32821824	56.67092924	1.342710997	9.185848252	2.387041773
	91.75400866	94.54169276	84.12796698	92.05366357	100.6398349	99.07167942	22.06190619	112.1825062	86.39642828	81.21307816	112.1825062	90.57795443	78.18369453	87.76057792	103.6119711	13.35039338	97.19870416	72.93714099	51.55037705	49.80807448	76.0732856	66.22927613	89.92459097	87.224022	103.9422085	32.03658835	13.5681812	59.69564152	16.70432581	8.341273514	26.50477772
AMP2CPR2STR1ERY3TMP3	AMP	CPR	STR	ERY	TMP	AMP+CPR	AMP+STR	AMP+ERY	AMP+TMP	CPR+STR	CPR+ERY	CPR+TMP	STR+ERY	STR+TMP	ERY+TMP	AMP+CPR+STR	AMP+CPR+ERY	AMP+CPR+TMP	AMP+STR+ERY	AMP+STR+TMP	AMP+ERY+TMP	CPR+STR+ERY	CPR+STR+TMP	CPR+ERY+TMP	STR+ERY+TMP	AMP+CPR+STR+ERY	AMP+CPR+STR+TMP	AMP+CPR+ERY+TMP	AMP+STR+ERY+TMP	CPR+STR+ERY+TMP	AMP+CPR+STR+ERY+TMP
	70.66920134	79.50525784	99.50227682	111.7017897	81.88075823	61.89089684	30.93582309	68.10886253	76.11714031	59.27280603	61.64063816	72.72902279	75.10325109	74.42550037	80.5252568	11.06913396	64.41273432	74.57708689	31.45559112	15.38128354	82.277354	36.30675939	56.19269918	82.89337536	65.91125702	1.501552085	6.814736386	54.55639242	5.197680295	73.88406285	6.506725702
	85.31543052	81.56436488	102.1241365	116.0332204	64.70207757	72.95396419	23.52941176	104.6675192	94.18158568	77.40835465	93.49957374	98.61466326	85.56570335	68.34493286	128.6176296	22.14407502	103.3034953	78.49531117	41.58141517	44.65046888	68.26513214	66.83716965	77.40835465	78.1543052	83.2475227	39.1943734	37.1483376	61.3597613	-1.641091219	1.683716965	7.502131287
	91.75400866	94.54169276	84.12796698	92.05366357	105.9236326	99.07167942	22.06190619	112.1825062	88.83565187	81.21307816	112.1825062	78.73029701	78.18369453	91.0629515	99.97936017	13.35039338	97.19870416	65.96793074	51.55037705	44.5811668	79.20943021	66.22927613	88.53074892	84.65412572	93.70485036	32.03658835	17.74970735	61.43794408	22.27969401	10.43203659	50.20009256
AMP2CPR2STR2ERY1TMP1	AMP	CPR	STR	ERY	TMP	AMP+CPR	AMP+STR	AMP+ERY	AMP+TMP	CPR+STR	CPR+ERY	CPR+TMP	STR+ERY	STR+TMP	ERY+TMP	AMP+CPR+STR	AMP+CPR+ERY	AMP+CPR+TMP	AMP+STR+ERY	AMP+STR+TMP	AMP+ERY+TMP	CPR+STR+ERY	CPR+STR+TMP	CPR+ERY+TMP	STR+ERY+TMP	AMP+CPR+STR+ERY	AMP+CPR+STR+TMP	AMP+CPR+ERY+TMP	AMP+STR+ERY+TMP	CPR+STR+ERY+TMP	AMP+CPR+STR+ERY+TMP
	70.66920134	79.50525784	68.10886253	101.535529	116.1071693	61.89089684	19.53942777	64.04697163	85.66547152	42.02420771	48.03041606	72.72902279	45.93209327	60.10058474	91.03039288	14.91926751	67.18483047	89.36159973	11.37714464	40.79216498	75.50111895	39.56012224	33.30365522	77.34918305	83.20138605	21.54149722	42.3322184	84.74143947	36.69177275	51.88054961	-3.965637558
	85.31543052	81.56436488	78.75106564	110.4033531	104.4423171	72.95396419	21.48337596	82.50213129	103.0477408	62.74509804	73.29497016	108.5038363	78.1543052	102.0247229	89.87089597	23.16709292	96.14236999	78.49531117	43.62745098	44.4799659	95.20460358	54.56095482	71.6112532	108.5038363	110.8908781	43.62745098	33.22676897	122.3998295	41.26172208	54.90196078	10.89087809
	91.75400866	94.54169276	67.2311001	91.0629515	98.65841073	99.07167942	26.50477772	82.91182316	83.04249585	53.4233523	88.61786405	83.95720469	81.6486538	77.81558816	112.5283798	10.56270928	93.49631122	78.73029701	67.27465766	44.97318487	82.21490213	68.05869382	75.98617047	83.39095636	75.02790406	44.92962731	58.21468434	75.02790406	61.35082895	56.82084229	2.417444804
AMP2CPR2STR2ERY1TMP2	AMP	CPR	STR	ERY	TMP	AMP+CPR	AMP+STR	AMP+ERY	AMP+TMP	CPR+STR	CPR+ERY	CPR+TMP	STR+ERY	STR+TMP	ERY+TMP	AMP+CPR+STR	AMP+CPR+ERY	AMP+CPR+TMP	AMP+STR+ERY	AMP+STR+TMP	AMP+ERY+TMP	CPR+STR+ERY	CPR+STR+TMP	CPR+ERY+TMP	STR+ERY+TMP	AMP+CPR+STR+ERY	AMP+CPR+STR+TMP	AMP+CPR+ERY+TMP	AMP+STR+ERY+TMP	CPR+STR+ERY+TMP	AMP+CPR+STR+ERY+TMP
	70.66920134	79.50525784	68.10886253	101.535529	100.8577782	61.89089684	19.53942777	64.04697163	84.43342878	42.02420771	48.03041606	73.34504416	45.93209327	64.662993	92.04701896	14.91926751	67.18483047	76.73316168	11.37714464	19.05816108	80.73730058	39.56012224	68.10886253	81.96934331	86.89751426	21.54149722	-0.327261352	71.18896937	10.43386192	57.11673124	-4.966672281
	85.31543052	81.56436488	78.75106564	110.4033531	83.90986003	72.95396419	21.48337596	82.50213129	93.84057971	62.74509804	73.29497016	102.7067349	78.1543052	97.25063939	104.1111485	23.16709292	96.14236999	81.90537084	43.62745098	40.92071611	70.31116795	54.56095482	63.76811594	106.7988065	109.8678602	43.62745098	30.66922421	86.67945439	38.00085251	44.33077579	17.02898551
	91.75400866	94.54169276	67.2311001	91.0629515	100.6398349	99.07167942	26.50477772	82.91182316	86.39642828	53.4233523	88.61786405	90.57795443	81.6486538	86.74488879	107.244582	10.56270928	93.49631122	72.93714099	67.27465766	37.65551412	77.46712765	68.05869382	76.33463099	78.8609697	191.2830425	44.92962731	15.65894427	59.91342934	34.86783002	71.45618381	14.26510222
AMP2CPR2STR2ERY1TMP3	AMP	CPR	STR	ERY	TMP	AMP+CPR	AMP+STR	AMP+ERY	AMP+TMP	CPR+STR	CPR+ERY	CPR+TMP	STR+ERY	STR+TMP	ERY+TMP	AMP+CPR+STR	AMP+CPR+ERY	AMP+CPR+TMP	AMP+STR+ERY	AMP+STR+TMP	AMP+ERY+TMP	CPR+STR+ERY	CPR+STR+TMP	CPR+ERY+TMP	STR+ERY+TMP	AMP+CPR+STR+ERY	AMP+CPR+STR+TMP	AMP+CPR+ERY+TMP	AMP+STR+ERY+TMP	CPR+STR+ERY+TMP	AMP+CPR+STR+ERY+TMP
	70.66920134	79.50525784	68.10886253	101.535529	81.88075823	61.89089684	19.53942777	64.04697163	76.11714031	42.02420771	48.03041606	72.72902279	45.93209327	78.2154631	100.8577782	14.91926751	67.18483047	74.57708689	11.37714464	19.84743846	70.88095868	39.56012224	41.10017566	89.66961041	74.26907621	21.54149722	0.34651202	77.65719373	32.37962317	48.1844214	23.67832134
	85.31543052	81.56436488	78.75106564	110.4033531	64.70207757	72.95396419	21.48337596	82.50213129	94.18158568	62.74509804	73.29497016	98.61466326	78.1543052	89.06649616	102.7864738	23.16709292	96.14236999	78.49531117	43.62745098	34.07928389	69.62915601	54.56095482	55.24296675	110.2088662	114.3009378	43.62745098	34.42028986	76.10826939	32.39556692	42.62574595	8.674339301
	91.75400866	94.54169276	67.2311001	91.0629515	105.9236326	99.07167942	26.50477772	82.91182316	88.83565187	53.4233523	88.61786405	78.73029701	81.6486538	93.71409904	113.5190918	10.56270928	93.49631122	65.96793074	67.27465766	70.06234176	74.67944355	68.05869382	63.09313152	96.28399532	106.3893502	44.92962731	17.74970735	65.83725805	37.3070536	57.86622383	22.67171208
AMP2CPR2STR2ERY2TMP1	AMP	CPR	STR	ERY	TMP	AMP+CPR	AMP+STR	AMP+ERY	AMP+TMP	CPR+STR	CPR+ERY	CPR+TMP	STR+ERY	STR+TMP	ERY+TMP	AMP+CPR+STR	AMP+CPR+ERY	AMP+CPR+TMP	AMP+STR+ERY	AMP+STR+TMP	AMP+ERY+TMP	CPR+STR+ERY	CPR+STR+TMP	CPR+ERY+TMP	STR+ERY+TMP	AMP+CPR+STR+ERY	AMP+CPR+STR+TMP	AMP+CPR+ERY+TMP	AMP+STR+ERY+TMP	CPR+STR+ERY+TMP	AMP+CPR+STR+ERY+TMP
	70.66920134	79.50525784	68.10886253	104.2465318	116.1071693	61.89089684	19.53942777	52.34256563	85.66547152	42.02420771	56.65471521	72.72902279	46.49036263	60.10058474	88.99714074	14.91926751	64.41273432	89.36159973	24.46759872	40.79216498	71.80499074	38.84784753	33.30365522	89.05358905	86.89751426	4.908920278	42.3322184	65.95278774	4.966672281	60.81285945	13.51396877
	85.31543052	81.56436488	78.75106564	119.344907	104.4423171	72.95396419	21.48337596	95.46035806	103.0477408	62.74509804	80.79710145	108.5038363	74.31798806	102.0247229	95.50076324	23.16709292	106.0315431	78.49531117	47.03751066	44.4799659	75	55.64791134	71.6112532	105.0937766	103.0477408	50.7885763	33.22676897	69.97016198	31.69224211	53.87894288	5.94629156
	91.75400866	94.54169276	67.2311001	93.374613	98.65841073	99.07167942	26.50477772	97.89562519	83.04249585	53.4233523	86.39642828	83.95720469	72.10954727	77.81558816	113.1888545	10.56270928	97.89562519	78.73029701	50.156535	44.97318487	75.94261291	73.98252253	75.98617047	64.22562818	96.28399532	42.49040372	58.21468434	71.06416574	32.38504887	68.3200392	34.60648463
AMP2CPR2STR2ERY2TMP2	AMP	CPR	STR	ERY	TMP	AMP+CPR	AMP+STR	AMP+ERY	AMP+TMP	CPR+STR	CPR+ERY	CPR+TMP	STR+ERY	STR+TMP	ERY+TMP	AMP+CPR+STR	AMP+CPR+ERY	AMP+CPR+TMP	AMP+STR+ERY	AMP+STR+TMP	AMP+ERY+TMP	CPR+STR+ERY	CPR+STR+TMP	CPR+ERY+TMP	STR+ERY+TMP	AMP+CPR+STR+ERY	AMP+CPR+STR+TMP	AMP+CPR+ERY+TMP	AMP+STR+ERY+TMP	CPR+STR+ERY+TMP	AMP+CPR+STR+ERY+TMP
	70.66920134	79.50525784	68.10886253	104.2465318	100.8577782	61.89089684	19.53942777	52.34256563	84.43342878	42.02420771	56.65471521	73.34504416	46.49036263	64.662993	96.79127396	14.91926751	64.41273432	76.73316168	24.46759872	19.05816108	65.02875568	38.84784753	68.10886253	85.66547152	87.51353563	4.908920278	-0.327261352	70.572948	16.59407561	65.64477705	-3.426618861
	85.31543052	81.56436488	78.75106564	119.344907	83.90986003	72.95396419	21.48337596	95.46035806	93.84057971	62.74509804	80.79710145	102.7067349	74.31798806	97.25063939	104.7734858	23.16709292	106.0315431	81.90537084	47.03751066	40.92071611	69.88491049	55.64791134	63.76811594	95.88661552	87.02046036	50.7885763	30.66922421	51.89684569	35.10230179	48.76385337	8.16283035
	91.75400866	94.54169276	67.2311001	93.374613	100.6398349	99.07167942	26.50477772	97.89562519	86.39642828	53.4233523	86.39642828	90.57795443	72.10954727	86.74488879	111.8679051	10.56270928	97.89562519	72.93714099	50.156535	37.65551412	67.01331228	73.98252253	76.33463099	88.61786405	68.40715433	42.49040372	15.65894427	77.68491547	35.17273296	58.56314486	20.2324885
AMP2CPR2STR2ERY2TMP3	AMP	CPR	STR	ERY	TMP	AMP+CPR	AMP+STR	AMP+ERY	AMP+TMP	CPR+STR	CPR+ERY	CPR+TMP	STR+ERY	STR+TMP	ERY+TMP	AMP+CPR+STR	AMP+CPR+ERY	AMP+CPR+TMP	AMP+STR+ERY	AMP+STR+TMP	AMP+ERY+TMP	CPR+STR+ERY	CPR+STR+TMP	CPR+ERY+TMP	STR+ERY+TMP	AMP+CPR+STR+ERY	AMP+CPR+STR+TMP	AMP+CPR+ERY+TMP	AMP+STR+ERY+TMP	CPR+STR+ERY+TMP	AMP+CPR+STR+ERY+TMP
	70.66920134	79.50525784	68.10886253	104.2465318	81.88075823	61.89089684	19.53942777	52.34256563	76.11714031	42.02420771	56.65471521	72.72902279	46.49036263	78.2154631	100.1800275	14.91926751	64.41273432	74.57708689	24.46759872	19.84743846	60.71660611	38.84784753	41.10017566	102.9140698	87.20552494	4.908920278	0.34651202	74.57708689	23.13930264	39.56012224	7.661765768
	85.31543052	81.56436488	78.75106564	119.344907	64.70207757	72.95396419	21.48337596	95.46035806	94.18158568	62.74509804	80.79710145	98.61466326	74.31798806	89.06649616	113.7150397	23.16709292	106.0315431	78.49531117	47.03751066	34.07928389	79.51832907	55.64791134	55.24296675	96.56862745	110.5498721	50.7885763	34.42028986	63.15004263	28.96419437	60.35805627	52.32310315
	91.75400866	94.54169276	67.2311001	93.374613	105.9236326	99.07167942	26.50477772	97.89562519	88.83565187	53.4233523	86.39642828	78.73029701	72.10954727	93.71409904	113.8493292	10.56270928	97.89562519	65.96793074	50.156535	70.06234176	65.48879754	73.98252253	63.09313152	73.63406202	98.02629788	42.49040372	17.74970735	68.62494215	33.08196989	88.87920943	11.17251518
AMP2CPR2STR2ERY3TMP1	AMP	CPR	STR	ERY	TMP	AMP+CPR	AMP+STR	AMP+ERY	AMP+TMP	CPR+STR	CPR+ERY	CPR+TMP	STR+ERY	STR+TMP	ERY+TMP	AMP+CPR+STR	AMP+CPR+ERY	AMP+CPR+TMP	AMP+STR+ERY	AMP+STR+TMP	AMP+ERY+TMP	CPR+STR+ERY	CPR+STR+TMP	CPR+ERY+TMP	STR+ERY+TMP	AMP+CPR+STR+ERY	AMP+CPR+STR+TMP	AMP+CPR+ERY+TMP	AMP+STR+ERY+TMP	CPR+STR+ERY+TMP	AMP+CPR+STR+ERY+TMP
	70.66920134	79.50525784	68.10886253	111.7017897	116.1071693	61.89089684	19.53942777	68.10886253	85.66547152	42.02420771	61.64063816	72.72902279	50.24424285	60.10058474	100.1800275	14.91926751	64.41273432	89.36159973	10.60711793	40.79216498	58.25252063	83.20138605	33.30365522	72.4210121	69.28315326	5.832952331	42.3322184	60.40859542	15.67004355	45.41232524	31.85985514
	85.31543052	81.56436488	78.75106564	116.0332204	104.4423171	72.95396419	21.48337596	104.6675192	103.0477408	62.74509804	93.49957374	108.5038363	75	102.0247229	121.9942563	23.16709292	103.3034953	78.49531117	46.01449275	44.4799659	81.22335891	92.13554987	71.6112532	103.7297528	105.4347826	43.28644501	33.22676897	62.12702472	49.78687127	71.95225916	30.34953112
	91.75400866	94.54169276	67.2311001	92.05366357	98.65841073	99.07167942	26.50477772	112.1825062	83.04249585	53.4233523	112.1825062	83.95720469	75.94261291	77.81558816	119.4633643	10.56270928	97.19870416	78.73029701	44.92962731	44.97318487	79.55789072	87.92094302	75.98617047	85.13325892	80.95173277	29.59736477	58.21468434	70.71570522	46.36702692	86.43998584	19.6226826
AMP2CPR2STR2ERY3TMP2	AMP	CPR	STR	ERY	TMP	AMP+CPR	AMP+STR	AMP+ERY	AMP+TMP	CPR+STR	CPR+ERY	CPR+TMP	STR+ERY	STR+TMP	ERY+TMP	AMP+CPR+STR	AMP+CPR+ERY	AMP+CPR+TMP	AMP+STR+ERY	AMP+STR+TMP	AMP+ERY+TMP	CPR+STR+ERY	CPR+STR+TMP	CPR+ERY+TMP	STR+ERY+TMP	AMP+CPR+STR+ERY	AMP+CPR+STR+TMP	AMP+CPR+ERY+TMP	AMP+STR+ERY+TMP	CPR+STR+ERY+TMP	AMP+CPR+STR+ERY+TMP
	70.66920134	79.50525784	68.10886253	111.7017897	100.8577782	61.89089684	19.53942777	68.10886253	84.43342878	42.02420771	61.64063816	73.34504416	50.24424285	64.662993	89.3360161	14.91926751	64.41273432	76.73316168	10.60711793	19.05816108	70.515196	83.20138605	68.10886253	67.80085184	81.29556994	5.832952331	-0.327261352	47.47214669	36.61477007	60.19683808	3.34961619
	85.31543052	81.56436488	78.75106564	116.0332204	83.90986003	72.95396419	21.48337596	104.6675192	93.84057971	62.74509804	93.49957374	102.7067349	75	97.25063939	93.84491992	23.16709292	103.3034953	81.90537084	46.01449275	40.92071611	79.1773231	92.13554987	63.76811594	89.7485081	79.85933504	43.28644501	30.66922421	56.67092924	9.867860188	63.91730605	9.548167093
	91.75400866	94.54169276	67.2311001	92.05366357	100.6398349	99.07167942	26.50477772	112.1825062	86.39642828	53.4233523	112.1825062	90.57795443	75.94261291	86.74488879	103.6119711	10.56270928	97.19870416	72.93714099	44.92962731	37.65551412	76.0732856	87.92094302	76.33463099	87.224022	76.63953393	29.59736477	15.65894427	59.69564152	3.811286854	88.53074892	16.39942286
AMP2CPR2STR2ERY3TMP3	AMP	CPR	STR	ERY	TMP	AMP+CPR	AMP+STR	AMP+ERY	AMP+TMP	CPR+STR	CPR+ERY	CPR+TMP	STR+ERY	STR+TMP	ERY+TMP	AMP+CPR+STR	AMP+CPR+ERY	AMP+CPR+TMP	AMP+STR+ERY	AMP+STR+TMP	AMP+ERY+TMP	CPR+STR+ERY	CPR+STR+TMP	CPR+ERY+TMP	STR+ERY+TMP	AMP+CPR+STR+ERY	AMP+CPR+STR+TMP	AMP+CPR+ERY+TMP	AMP+STR+ERY+TMP	CPR+STR+ERY+TMP	AMP+CPR+STR+ERY+TMP
	70.66920134	79.50525784	68.10886253	111.7017897	81.88075823	61.89089684	19.53942777	68.10886253	76.11714031	42.02420771	61.64063816	72.72902279	50.24424285	78.2154631	80.5252568	14.91926751	64.41273432	74.57708689	10.60711793	19.84743846	82.277354	83.20138605	41.10017566	82.89337536	73.96106552	5.832952331	0.34651202	54.55639242	30.45455639	66.35705176	31.68659913
	85.31543052	81.56436488	78.75106564	116.0332204	64.70207757	72.95396419	21.48337596	104.6675192	94.18158568	62.74509804	93.49957374	98.61466326	75	89.06649616	128.6176296	23.16709292	103.3034953	78.49531117	46.01449275	34.07928389	68.26513214	92.13554987	55.24296675	78.1543052	81.22335891	43.28644501	34.42028986	61.3597613	1.427962489	66.49616368	10.23017903
	91.75400866	94.54169276	67.2311001	92.05366357	105.9236326	99.07167942	26.50477772	112.1825062	88.83565187	53.4233523	112.1825062	78.73029701	75.94261291	93.71409904	99.97936017	10.56270928	97.19870416	65.96793074	44.92962731	70.06234176	79.20943021	87.92094302	63.09313152	84.65412572	82.69403534	29.59736477	17.74970735	61.43794408	40.05118014	82.2584597	12.21789671
AMP2CPR2STR3ERY1TMP1	AMP	CPR	STR	ERY	TMP	AMP+CPR	AMP+STR	AMP+ERY	AMP+TMP	CPR+STR	CPR+ERY	CPR+TMP	STR+ERY	STR+TMP	ERY+TMP	AMP+CPR+STR	AMP+CPR+ERY	AMP+CPR+TMP	AMP+STR+ERY	AMP+STR+TMP	AMP+ERY+TMP	CPR+STR+ERY	CPR+STR+TMP	CPR+ERY+TMP	STR+ERY+TMP	AMP+CPR+STR+ERY	AMP+CPR+STR+TMP	AMP+CPR+ERY+TMP	AMP+STR+ERY+TMP	CPR+STR+ERY+TMP	AMP+CPR+STR+ERY+TMP
	70.66920134	79.50525784	74.20542334	101.535529	116.1071693	61.89089684	32.62988185	64.04697163	85.66547152	65.95278774	48.03041606	72.72902279	97.01871727	108.9645875	91.03039288	19.69343312	67.18483047	89.36159973	8.913059172	55.17241379	75.50111895	34.84370864	80.12127921	77.34918305	109.2964173	25.23762543	29.9155377	84.74143947	65.33676637	61.02461679	47.47214669
	85.31543052	81.56436488	60.83351516	110.4033531	104.4423171	72.95396419	38.51236147	82.50213129	103.0477408	79.51832907	73.29497016	108.5038363	81.43137683	106.9168667	89.87089597	41.58141517	96.14236999	78.49531117	40.89940324	77.47229327	95.20460358	62.80903666	78.1543052	108.5038363	116.6921231	51.12958227	33.14151748	122.3998295	54.28388747	79.85933504	83.52514919
	91.75400866	94.54169276	64.81466508	91.0629515	98.65841073	99.07167942	41.92415539	82.91182316	83.04249585	90.0117061	88.61786405	83.95720469	42.93255403	51.80368013	112.5283798	34.25802412	93.49631122	78.73029701	44.5811668	49.93874718	82.21490213	99.76860044	74.67944355	83.39095636	73.79668026	50.156535	41.22723437	75.02790406	53.4233523	59.34718101	41.57569488
AMP2CPR2STR3ERY1TMP2	AMP	CPR	STR	ERY	TMP	AMP+CPR	AMP+STR	AMP+ERY	AMP+TMP	CPR+STR	CPR+ERY	CPR+TMP	STR+ERY	STR+TMP	ERY+TMP	AMP+CPR+STR	AMP+CPR+ERY	AMP+CPR+TMP	AMP+STR+ERY	AMP+STR+TMP	AMP+ERY+TMP	CPR+STR+ERY	CPR+STR+TMP	CPR+ERY+TMP	STR+ERY+TMP	AMP+CPR+STR+ERY	AMP+CPR+STR+TMP	AMP+CPR+ERY+TMP	AMP+STR+ERY+TMP	CPR+STR+ERY+TMP	AMP+CPR+STR+ERY+TMP
	70.66920134	79.50525784	74.20542334	101.535529	100.8577782	61.89089684	32.62988185	64.04697163	84.43342878	65.95278774	48.03041606	73.34504416	97.01871727	90.7139524	92.04701896	19.69343312	67.18483047	76.73316168	8.913059172	37.86606348	80.73730058	34.84370864	83.81740742	81.96934331	108.6327578	25.23762543	40.38790096	71.18896937	48.08816806	75.50111895	22.21527059
	85.31543052	81.56436488	60.83351516	110.4033531	83.90986003	72.95396419	38.51236147	82.50213129	93.84057971	79.51832907	73.29497016	102.7067349	81.43137683	104.1239363	104.1111485	41.58141517	96.14236999	81.90537084	40.89940324	45.33248082	70.31116795	62.80903666	93.49957374	106.7988065	116.6921231	51.12958227	34.76129582	86.67945439	52.9198636	95.54560955	26.32139812
	91.75400866	94.54169276	64.81466508	91.0629515	100.6398349	99.07167942	41.92415539	82.91182316	86.39642828	90.0117061	88.61786405	90.57795443	42.93255403	58.12435748	107.244582	34.25802412	93.49631122	72.93714099	44.5811668	27.89861977	77.46712765	99.76860044	68.05869382	78.8609697	80.00646853	50.156535	28.33419541	59.91342934	60.04410203	72.58868048	39.83339232
AMP2CPR2STR3ERY1TMP3	AMP	CPR	STR	ERY	TMP	AMP+CPR	AMP+STR	AMP+ERY	AMP+TMP	CPR+STR	CPR+ERY	CPR+TMP	STR+ERY	STR+TMP	ERY+TMP	AMP+CPR+STR	AMP+CPR+ERY	AMP+CPR+TMP	AMP+STR+ERY	AMP+STR+TMP	AMP+ERY+TMP	CPR+STR+ERY	CPR+STR+TMP	CPR+ERY+TMP	STR+ERY+TMP	AMP+CPR+STR+ERY	AMP+CPR+STR+TMP	AMP+CPR+ERY+TMP	AMP+STR+ERY+TMP	CPR+STR+ERY+TMP	AMP+CPR+STR+ERY+TMP
	70.66920134	79.50525784	74.20542334	101.535529	81.88075823	61.89089684	32.62988185	64.04697163	76.11714031	65.95278774	48.03041606	72.72902279	97.01871727	91.4605693	100.8577782	19.69343312	67.18483047	74.57708689	8.913059172	49.93623216	70.88095868	34.84370864	75.80912963	89.66961041	107.9690984	25.23762543	43.4680078	77.65719373	49.01220011	79.19724715	28.68349496
	85.31543052	81.56436488	60.83351516	110.4033531	64.70207757	72.95396419	38.51236147	82.50213129	94.18158568	79.51832907	73.29497016	98.61466326	81.43137683	102.0292385	102.7864738	41.58141517	96.14236999	78.49531117	40.89940324	48.48678602	69.62915601	62.80903666	75.42625746	110.2088662	109.3606808	51.12958227	38.51236147	76.10826939	39.27962489	77.1312873	15.32395567
	91.75400866	94.54169276	64.81466508	91.0629515	105.9236326	99.07167942	41.92415539	82.91182316	88.83565187	90.0117061	88.61786405	78.73029701	42.93255403	70.72874915	113.5190918	34.25802412	93.49631122	65.96793074	44.5811668	27.85506221	74.67944355	99.76860044	87.224022	96.28399532	75.57090548	50.156535	35.30340566	65.83725805	57.25641793	85.48171943	21.58277298
AMP2CPR2STR3ERY2TMP1	AMP	CPR	STR	ERY	TMP	AMP+CPR	AMP+STR	AMP+ERY	AMP+TMP	CPR+STR	CPR+ERY	CPR+TMP	STR+ERY	STR+TMP	ERY+TMP	AMP+CPR+STR	AMP+CPR+ERY	AMP+CPR+TMP	AMP+STR+ERY	AMP+STR+TMP	AMP+ERY+TMP	CPR+STR+ERY	CPR+STR+TMP	CPR+ERY+TMP	STR+ERY+TMP	AMP+CPR+STR+ERY	AMP+CPR+STR+TMP	AMP+CPR+ERY+TMP	AMP+STR+ERY+TMP	CPR+STR+ERY+TMP	AMP+CPR+STR+ERY+TMP
	70.66920134	79.50525784	74.20542334	104.2465318	116.1071693	61.89089684	32.62988185	52.34256563	85.66547152	65.95278774	56.65471521	72.72902279	97.68237673	108.9645875	88.99714074	19.69343312	64.41273432	89.36159973	26.46966817	55.17241379	71.80499074	37.86606348	80.12127921	89.05358905	103.9871416	25.29537743	29.9155377	65.95278774	57.0204779	83.81740742	29.29951633
	85.31543052	81.56436488	60.83351516	119.344907	104.4423171	72.95396419	38.51236147	95.46035806	103.0477408	79.51832907	80.79710145	108.5038363	88.76281911	106.9168667	95.50076324	41.58141517	106.0315431	78.49531117	65.11082694	77.47229327	75	85.99744246	78.1543052	105.0937766	111.1062623	69.88491049	33.14151748	69.97016198	35.78431373	72.01619778	12.68115942
	91.75400866	94.54169276	64.81466508	93.374613	98.65841073	99.07167942	41.92415539	97.89562519	83.04249585	90.0117061	86.39642828	83.95720469	64.81466508	51.80368013	113.1888545	34.25802412	97.89562519	78.73029701	63.04957395	49.93874718	75.94261291	89.66324558	74.67944355	64.22562818	73.09438278	60.95881088	41.22723437	71.06416574	42.96953693	55.16565486	32.73350938
AMP2CPR2STR3ERY2TMP2	AMP	CPR	STR	ERY	TMP	AMP+CPR	AMP+STR	AMP+ERY	AMP+TMP	CPR+STR	CPR+ERY	CPR+TMP	STR+ERY	STR+TMP	ERY+TMP	AMP+CPR+STR	AMP+CPR+ERY	AMP+CPR+TMP	AMP+STR+ERY	AMP+STR+TMP	AMP+ERY+TMP	CPR+STR+ERY	CPR+STR+TMP	CPR+ERY+TMP	STR+ERY+TMP	AMP+CPR+STR+ERY	AMP+CPR+STR+TMP	AMP+CPR+ERY+TMP	AMP+STR+ERY+TMP	CPR+STR+ERY+TMP	AMP+CPR+STR+ERY+TMP
	70.66920134	79.50525784	74.20542334	104.2465318	100.8577782	61.89089684	32.62988185	52.34256563	84.43342878	65.95278774	56.65471521	73.34504416	97.68237673	90.7139524	96.79127396	19.69343312	64.41273432	76.73316168	26.46966817	37.86606348	65.02875568	37.86606348	83.81740742	85.66547152	106.6417794	25.29537743	40.38790096	70.572948	32.68763385	90.59364247	28.37548427
	85.31543052	81.56436488	60.83351516	119.344907	83.90986003	72.95396419	38.51236147	95.46035806	93.84057971	79.51832907	80.79710145	102.7067349	88.76281911	104.1239363	104.7734858	41.58141517	106.0315431	81.90537084	65.11082694	45.33248082	69.88491049	85.99744246	93.49957374	95.88661552	106.5677504	69.88491049	34.76129582	51.89684569	32.71526002	84.63341858	10.63512361
	91.75400866	94.54169276	64.81466508	93.374613	100.6398349	99.07167942	41.92415539	97.89562519	86.39642828	90.0117061	86.39642828	90.57795443	64.81466508	58.12435748	111.8679051	34.25802412	97.89562519	72.93714099	63.04957395	27.89861977	67.01331228	89.66324558	68.05869382	88.61786405	77.93653911	60.95881088	28.33419541	77.68491547	39.70271963	63.52870716	27.63727439
AMP2CPR2STR3ERY2TMP3	AMP	CPR	STR	ERY	TMP	AMP+CPR	AMP+STR	AMP+ERY	AMP+TMP	CPR+STR	CPR+ERY	CPR+TMP	STR+ERY	STR+TMP	ERY+TMP	AMP+CPR+STR	AMP+CPR+ERY	AMP+CPR+TMP	AMP+STR+ERY	AMP+STR+TMP	AMP+ERY+TMP	CPR+STR+ERY	CPR+STR+TMP	CPR+ERY+TMP	STR+ERY+TMP	AMP+CPR+STR+ERY	AMP+CPR+STR+TMP	AMP+CPR+ERY+TMP	AMP+STR+ERY+TMP	CPR+STR+ERY+TMP	AMP+CPR+STR+ERY+TMP
	70.66920134	79.50525784	74.20542334	104.2465318	81.88075823	61.89089684	32.62988185	52.34256563	76.11714031	65.95278774	56.65471521	72.72902279	97.68237673	91.4605693	100.1800275	19.69343312	64.41273432	74.57708689	26.46966817	49.93623216	60.71660611	37.86606348	75.80912963	102.9140698	107.6372686	25.29537743	43.4680078	74.57708689	38.53983685	76.425151	47.47214669
	85.31543052	81.56436488	60.83351516	119.344907	64.70207757	72.95396419	38.51236147	95.46035806	94.18158568	79.51832907	80.79710145	98.61466326	88.76281911	102.0292385	113.7150397	41.58141517	106.0315431	78.49531117	65.11082694	48.48678602	79.51832907	85.99744246	75.42625746	96.56862745	103.0765874	69.88491049	38.51236147	63.15004263	46.78175618	72.35720375	30.7544757
	91.75400866	94.54169276	64.81466508	93.374613	105.9236326	99.07167942	41.92415539	97.89562519	88.83565187	90.0117061	86.39642828	78.73029701	64.81466508	70.72874915	113.8493292	34.25802412	97.89562519	65.96793074	63.04957395	27.85506221	65.48879754	89.66324558	87.224022	73.63406202	88.58189043	60.95881088	35.30340566	68.62494215	46.80260256	63.87716767	42.62107642
AMP2CPR2STR3ERY3TMP1	AMP	CPR	STR	ERY	TMP	AMP+CPR	AMP+STR	AMP+ERY	AMP+TMP	CPR+STR	CPR+ERY	CPR+TMP	STR+ERY	STR+TMP	ERY+TMP	AMP+CPR+STR	AMP+CPR+ERY	AMP+CPR+TMP	AMP+STR+ERY	AMP+STR+TMP	AMP+ERY+TMP	CPR+STR+ERY	CPR+STR+TMP	CPR+ERY+TMP	STR+ERY+TMP	AMP+CPR+STR+ERY	AMP+CPR+STR+TMP	AMP+CPR+ERY+TMP	AMP+STR+ERY+TMP	CPR+STR+ERY+TMP	AMP+CPR+STR+ERY+TMP
	70.66920134	79.50525784	74.20542334	111.7017897	116.1071693	61.89089684	32.62988185	68.10886253	85.66547152	65.95278774	61.64063816	72.72902279	92.37310105	108.9645875	100.1800275	19.69343312	64.41273432	89.36159973	20.0014438	55.17241379	58.25252063	72.11300142	80.12127921	72.4210121	100.6688443	21.90725991	29.9155377	60.40859542	32.37962317	73.96106552	41.92795438
	85.31543052	81.56436488	60.83351516	116.0332204	104.4423171	72.95396419	38.51236147	104.6675192	103.0477408	79.51832907	93.49957374	108.5038363	95.0469125	106.9168667	121.9942563	41.58141517	103.3034953	78.49531117	59.65473146	77.47229327	81.22335891	89.06649616	78.1543052	103.7297528	105.8695178	47.37851662	33.14151748	62.12702472	57.01193521	66.5601023	25.98039216
	91.75400866	94.54169276	64.81466508	92.05366357	98.65841073	99.07167942	41.92415539	112.1825062	83.04249585	90.0117061	112.1825062	83.95720469	67.47600291	51.80368013	119.4633643	34.25802412	97.19870416	78.73029701	60.61035037	49.93874718	79.55789072	78.8609697	74.67944355	85.13325892	84.14632738	45.62654834	41.22723437	70.71570522	51.33258922	69.10407535	39.83339232
AMP2CPR2STR3ERY3TMP2	AMP	CPR	STR	ERY	TMP	AMP+CPR	AMP+STR	AMP+ERY	AMP+TMP	CPR+STR	CPR+ERY	CPR+TMP	STR+ERY	STR+TMP	ERY+TMP	AMP+CPR+STR	AMP+CPR+ERY	AMP+CPR+TMP	AMP+STR+ERY	AMP+STR+TMP	AMP+ERY+TMP	CPR+STR+ERY	CPR+STR+TMP	CPR+ERY+TMP	STR+ERY+TMP	AMP+CPR+STR+ERY	AMP+CPR+STR+TMP	AMP+CPR+ERY+TMP	AMP+STR+ERY+TMP	CPR+STR+ERY+TMP	AMP+CPR+STR+ERY+TMP
	70.66920134	79.50525784	74.20542334	111.7017897	100.8577782	61.89089684	32.62988185	68.10886253	84.43342878	65.95278774	61.64063816	73.34504416	92.37310105	90.7139524	89.3360161	19.69343312	64.41273432	76.73316168	20.0014438	37.86606348	70.515196	72.11300142	83.81740742	67.80085184	74.45429564	21.90725991	40.38790096	47.47214669	20.0014438	53.94037106	28.68349496
	85.31543052	81.56436488	60.83351516	116.0332204	83.90986003	72.95396419	38.51236147	104.6675192	93.84057971	79.51832907	93.49957374	102.7067349	95.0469125	104.1239363	93.84491992	41.58141517	103.3034953	81.90537084	59.65473146	45.33248082	79.1773231	89.06649616	93.49957374	89.7485081	92.95221471	47.37851662	34.76129582	56.67092924	35.86956522	84.97442455	33.75959079
	91.75400866	94.54169276	64.81466508	92.05366357	100.6398349	99.07167942	41.92415539	112.1825062	86.39642828	90.0117061	112.1825062	90.57795443	67.47600291	58.12435748	103.6119711	34.25802412	97.19870416	72.93714099	60.61035037	27.89861977	76.0732856	78.8609697	68.05869382	87.224022	85.03343999	45.62654834	28.33419541	59.69564152	56.2110364	85.83017995	43.31799744
AMP2CPR2STR3ERY3TMP3	AMP	CPR	STR	ERY	TMP	AMP+CPR	AMP+STR	AMP+ERY	AMP+TMP	CPR+STR	CPR+ERY	CPR+TMP	STR+ERY	STR+TMP	ERY+TMP	AMP+CPR+STR	AMP+CPR+ERY	AMP+CPR+TMP	AMP+STR+ERY	AMP+STR+TMP	AMP+ERY+TMP	CPR+STR+ERY	CPR+STR+TMP	CPR+ERY+TMP	STR+ERY+TMP	AMP+CPR+STR+ERY	AMP+CPR+STR+TMP	AMP+CPR+ERY+TMP	AMP+STR+ERY+TMP	CPR+STR+ERY+TMP	AMP+CPR+STR+ERY+TMP
	70.66920134	79.50525784	74.20542334	111.7017897	81.88075823	61.89089684	32.62988185	68.10886253	76.11714031	65.95278774	61.64063816	72.72902279	92.37310105	91.4605693	80.5252568	19.69343312	64.41273432	74.57708689	20.0014438	49.93623216	82.277354	72.11300142	75.80912963	82.89337536	91.70944159	21.90725991	43.4680078	54.55639242	47.78015737	70.88095868	24.91036408
	85.31543052	81.56436488	60.83351516	116.0332204	64.70207757	72.95396419	38.51236147	104.6675192	94.18158568	79.51832907	93.49957374	98.61466326	95.0469125	102.0292385	128.6176296	41.58141517	103.3034953	78.49531117	59.65473146	48.48678602	68.26513214	89.06649616	75.42625746	78.1543052	98.1889592	47.37851662	38.51236147	61.3597613	43.03069054	45.75873828	33.75959079
	91.75400866	94.54169276	64.81466508	92.05366357	105.9236326	99.07167942	41.92415539	112.1825062	88.83565187	90.0117061	112.1825062	78.73029701	67.47600291	70.72874915	99.97936017	34.25802412	97.19870416	65.96793074	60.61035037	27.85506221	79.20943021	78.8609697	87.224022	84.65412572	77.3451307	45.62654834	35.30340566	61.43794408	26.11275964	65.61947023	13.26327825
AMP2CPR3STR1ERY1TMP1	AMP	CPR	STR	ERY	TMP	AMP+CPR	AMP+STR	AMP+ERY	AMP+TMP	CPR+STR	CPR+ERY	CPR+TMP	STR+ERY	STR+TMP	ERY+TMP	AMP+CPR+STR	AMP+CPR+ERY	AMP+CPR+TMP	AMP+STR+ERY	AMP+STR+TMP	AMP+ERY+TMP	CPR+STR+ERY	CPR+STR+TMP	CPR+ERY+TMP	STR+ERY+TMP	AMP+CPR+STR+ERY	AMP+CPR+STR+TMP	AMP+CPR+ERY+TMP	AMP+STR+ERY+TMP	CPR+STR+ERY+TMP	AMP+CPR+STR+ERY+TMP
	70.66920134	86.40016591	99.50227682	101.535529	116.1071693	63.18069158	30.93582309	64.04697163	85.66547152	83.80774615	105.6462902	74.12246591	112.3795404	83.23625966	91.03039288	11.06913396	58.868542	63.48870226	9.837091224	34.1506846	75.50111895	88.7851921	32.39487738	94.69590916	92.38589431	20.30945448	9.52908054	81.96934331	9.817840556	28.08109089	17.21009698
	85.31543052	75.49639974	102.1241365	110.4033531	104.4423171	93.49957374	23.52941176	82.50213129	103.0477408	63.05913157	92.25398211	102.0292385	132.9228222	77.28648677	89.87089597	23.16709292	79.77408355	72.01619778	43.28644501	34.07928389	95.20460358	80.86406284	79.27121973	113.5500764	78.61116142	54.19863598	24.19011083	83.26939471	22.48508099	56.57866027	14.13043478
	91.75400866	49.1423423	84.12796698	91.0629515	98.65841073	108.4801132	22.06190619	82.91182316	83.04249585	40.67780948	52.09938433	54.87161124	79.50464396	97.3374613	112.5283798	10.56270928	93.71409904	96.50178314	40.74810116	49.80807448	82.21490213	57.66231966	40.2712162	78.52794751	99.31888545	39.35425911	30.29428579	79.90635124	8.384831078	52.0439398	40.74810116
AMP2CPR3STR1ERY1TMP2	AMP	CPR	STR	ERY	TMP	AMP+CPR	AMP+STR	AMP+ERY	AMP+TMP	CPR+STR	CPR+ERY	CPR+TMP	STR+ERY	STR+TMP	ERY+TMP	AMP+CPR+STR	AMP+CPR+ERY	AMP+CPR+TMP	AMP+STR+ERY	AMP+STR+TMP	AMP+ERY+TMP	CPR+STR+ERY	CPR+STR+TMP	CPR+ERY+TMP	STR+ERY+TMP	AMP+CPR+STR+ERY	AMP+CPR+STR+TMP	AMP+CPR+ERY+TMP	AMP+STR+ERY+TMP	CPR+STR+ERY+TMP	AMP+CPR+STR+ERY+TMP
	70.66920134	86.40016591	99.50227682	101.535529	100.8577782	63.18069158	30.93582309	64.04697163	84.43342878	83.80774615	105.6462902	94.44703686	112.3795404	65.27586572	92.04701896	11.06913396	58.868542	76.05938831	9.837091224	3.368866858	80.73730058	88.7851921	59.25234614	92.04127132	85.60838717	20.30945448	-3.34961619	84.74143947	-1.867314772	47.30647586	11.35789398
	85.31543052	75.49639974	102.1241365	110.4033531	83.90986003	93.49957374	23.52941176	82.50213129	93.84057971	63.05913157	92.25398211	104.4730526	132.9228222	69.00727019	104.1111485	23.16709292	79.77408355	74.06223359	43.28644501	32.71526002	70.31116795	80.86406284	85.55531311	105.8695178	95.16959458	54.19863598	21.46206309	83.95140665	23.16709292	45.40693869	15.32395567
	91.75400866	49.1423423	84.12796698	91.0629515	100.6398349	108.4801132	22.06190619	82.91182316	86.39642828	40.67780948	52.09938433	68.06741132	79.50464396	87.76057792	107.244582	10.56270928	93.71409904	101.7286908	40.74810116	49.80807448	77.46712765	57.66231966	67.42055837	71.72675083	100.6398349	39.35425911	35.86965399	80.60327226	16.3558653	50.26971458	21.9312335
AMP2CPR3STR1ERY1TMP3	AMP	CPR	STR	ERY	TMP	AMP+CPR	AMP+STR	AMP+ERY	AMP+TMP	CPR+STR	CPR+ERY	CPR+TMP	STR+ERY	STR+TMP	ERY+TMP	AMP+CPR+STR	AMP+CPR+ERY	AMP+CPR+TMP	AMP+STR+ERY	AMP+STR+TMP	AMP+ERY+TMP	CPR+STR+ERY	CPR+STR+TMP	CPR+ERY+TMP	STR+ERY+TMP	AMP+CPR+STR+ERY	AMP+CPR+STR+TMP	AMP+CPR+ERY+TMP	AMP+STR+ERY+TMP	CPR+STR+ERY+TMP	AMP+CPR+STR+ERY+TMP
	70.66920134	86.40016591	99.50227682	101.535529	81.88075823	63.18069158	30.93582309	64.04697163	76.11714031	83.80774615	105.6462902	105.0655882	112.3795404	74.42550037	100.8577782	11.06913396	58.868542	64.41273432	9.837091224	15.38128354	70.88095868	88.7851921	74.84834344	101.6643335	92.38589431	20.30945448	2.136824121	89.66961041	-7.661765768	21.11266656	27.99047092
	85.31543052	75.49639974	102.1241365	110.4033531	64.70207757	93.49957374	23.52941176	82.50213129	94.18158568	63.05913157	92.25398211	102.3783548	132.9228222	68.34493286	102.7864738	23.16709292	79.77408355	83.95140665	43.28644501	44.65046888	69.62915601	80.86406284	84.50796422	111.4553786	92.85141393	54.19863598	39.02387042	78.1543052	22.14407502	26.27100153	12.9369139
	91.75400866	49.1423423	84.12796698	91.0629515	105.9236326	108.4801132	22.06190619	82.91182316	88.83565187	40.67780948	52.09938433	63.04043986	79.50464396	91.0629515	113.5190918	10.56270928	93.71409904	82.56336264	40.74810116	44.5811668	74.67944355	57.66231966	44.94703892	71.13534243	108.8957688	39.35425911	23.32507555	66.53417908	30.99120682	37.31417417	28.90044374
AMP2CPR3STR1ERY2TMP1	AMP	CPR	STR	ERY	TMP	AMP+CPR	AMP+STR	AMP+ERY	AMP+TMP	CPR+STR	CPR+ERY	CPR+TMP	STR+ERY	STR+TMP	ERY+TMP	AMP+CPR+STR	AMP+CPR+ERY	AMP+CPR+TMP	AMP+STR+ERY	AMP+STR+TMP	AMP+ERY+TMP	CPR+STR+ERY	CPR+STR+TMP	CPR+ERY+TMP	STR+ERY+TMP	AMP+CPR+STR+ERY	AMP+CPR+STR+TMP	AMP+CPR+ERY+TMP	AMP+STR+ERY+TMP	CPR+STR+ERY+TMP	AMP+CPR+STR+ERY+TMP
	70.66920134	86.40016591	99.50227682	104.2465318	116.1071693	63.18069158	30.93582309	52.34256563	85.66547152	83.80774615	106.9736092	74.12246591	98.8245261	83.23625966	88.99714074	11.06913396	63.79671295	63.48870226	16.28606492	34.1506846	71.80499074	70.53455696	32.39487738	101.000674	88.31939002	22.13826792	9.52908054	69.64891595	2.444834805	80.82127858	1.501552085
	85.31543052	75.49639974	102.1241365	119.344907	104.4423171	93.49957374	23.52941176	95.46035806	103.0477408	63.05913157	91.55574951	102.0292385	95.16959458	77.28648677	95.50076324	23.16709292	89.66325661	72.01619778	45.67348679	34.07928389	75	64.45559677	79.27121973	110.757146	86.55920933	63.4057971	24.19011083	70.99317988	22.14407502	42.26489199	26.40664962
	91.75400866	49.1423423	84.12796698	93.374613	98.65841073	108.4801132	22.06190619	97.89562519	83.04249585	40.67780948	60.08339783	54.87161124	80.8255934	97.3374613	113.1888545	10.56270928	99.28946724	96.50178314	50.156535	49.80807448	75.94261291	44.22625992	40.2712162	80.19128366	111.2074303	45.97500885	30.29428579	70.36724471	29.24890426	47.01696834	28.90044374
AMP2CPR3STR1ERY2TMP2	AMP	CPR	STR	ERY	TMP	AMP+CPR	AMP+STR	AMP+ERY	AMP+TMP	CPR+STR	CPR+ERY	CPR+TMP	STR+ERY	STR+TMP	ERY+TMP	AMP+CPR+STR	AMP+CPR+ERY	AMP+CPR+TMP	AMP+STR+ERY	AMP+STR+TMP	AMP+ERY+TMP	CPR+STR+ERY	CPR+STR+TMP	CPR+ERY+TMP	STR+ERY+TMP	AMP+CPR+STR+ERY	AMP+CPR+STR+TMP	AMP+CPR+ERY+TMP	AMP+STR+ERY+TMP	CPR+STR+ERY+TMP	AMP+CPR+STR+ERY+TMP
	70.66920134	86.40016591	99.50227682	104.2465318	100.8577782	63.18069158	30.93582309	52.34256563	84.43342878	83.80774615	106.9736092	94.44703686	98.8245261	65.27586572	96.79127396	11.06913396	63.79671295	76.05938831	16.28606492	3.368866858	65.02875568	70.53455696	59.25234614	90.7139524	88.31939002	22.13826792	-3.34961619	65.02875568	12.28192603	64.56162182	-5.274682966
	85.31543052	75.49639974	102.1241365	119.344907	83.90986003	93.49957374	23.52941176	95.46035806	93.84057971	63.05913157	91.55574951	104.4730526	95.16959458	69.00727019	104.7734858	23.16709292	89.66325661	74.06223359	45.67348679	32.71526002	69.88491049	64.45559677	85.55531311	103.0765874	90.20206463	63.4057971	21.46206309	66.21909633	24.19011083	15.6229544	8.844842285
	91.75400866	49.1423423	84.12796698	93.374613	100.6398349	108.4801132	22.06190619	97.89562519	86.39642828	40.67780948	60.08339783	68.06741132	80.8255934	87.76057792	111.8679051	10.56270928	99.28946724	101.7286908	50.156535	49.80807448	67.01331228	44.22625992	67.42055837	77.6408349	103.6119711	45.97500885	35.86965399	87.79027033	28.68265592	56.77520705	30.6427463
AMP2CPR3STR1ERY2TMP3	AMP	CPR	STR	ERY	TMP	AMP+CPR	AMP+STR	AMP+ERY	AMP+TMP	CPR+STR	CPR+ERY	CPR+TMP	STR+ERY	STR+TMP	ERY+TMP	AMP+CPR+STR	AMP+CPR+ERY	AMP+CPR+TMP	AMP+STR+ERY	AMP+STR+TMP	AMP+ERY+TMP	CPR+STR+ERY	CPR+STR+TMP	CPR+ERY+TMP	STR+ERY+TMP	AMP+CPR+STR+ERY	AMP+CPR+STR+TMP	AMP+CPR+ERY+TMP	AMP+STR+ERY+TMP	CPR+STR+ERY+TMP	AMP+CPR+STR+ERY+TMP
	70.66920134	86.40016591	99.50227682	104.2465318	81.88075823	63.18069158	30.93582309	52.34256563	76.11714031	83.80774615	106.9736092	105.0655882	98.8245261	74.42550037	100.1800275	11.06913396	63.79671295	64.41273432	16.28606492	15.38128354	60.71660611	70.53455696	74.84834344	100.6688443	89.3360161	22.13826792	2.136824121	73.65305484	2.117573453	61.90698398	45.23906923
	85.31543052	75.49639974	102.1241365	119.344907	64.70207757	93.49957374	23.52941176	95.46035806	94.18158568	63.05913157	91.55574951	102.3783548	95.16959458	68.34493286	113.7150397	23.16709292	89.66325661	83.95140665	45.67348679	44.65046888	79.51832907	64.45559677	84.50796422	97.8398429	109.4098471	63.4057971	39.02387042	78.49531117	29.30520034	29.34758892	19.75703325
	91.75400866	49.1423423	84.12796698	93.374613	105.9236326	108.4801132	22.06190619	97.89562519	88.83565187	40.67780948	60.08339783	63.04043986	80.8255934	91.0629515	113.8493292	10.56270928	99.28946724	82.56336264	50.156535	44.5811668	65.48879754	44.22625992	44.94703892	93.01745348	117.4819401	45.97500885	23.32507555	69.32186317	33.08196989	64.16781214	20.88585196
AMP2CPR3STR1ERY3TMP1	AMP	CPR	STR	ERY	TMP	AMP+CPR	AMP+STR	AMP+ERY	AMP+TMP	CPR+STR	CPR+ERY	CPR+TMP	STR+ERY	STR+TMP	ERY+TMP	AMP+CPR+STR	AMP+CPR+ERY	AMP+CPR+TMP	AMP+STR+ERY	AMP+STR+TMP	AMP+ERY+TMP	CPR+STR+ERY	CPR+STR+TMP	CPR+ERY+TMP	STR+ERY+TMP	AMP+CPR+STR+ERY	AMP+CPR+STR+TMP	AMP+CPR+ERY+TMP	AMP+STR+ERY+TMP	CPR+STR+ERY+TMP	AMP+CPR+STR+ERY+TMP
	70.66920134	86.40016591	99.50227682	111.7017897	116.1071693	63.18069158	30.93582309	68.10886253	85.66547152	83.80774615	98.01420646	74.12246591	75.10325109	83.23625966	100.1800275	11.06913396	66.56880911	63.48870226	31.45559112	34.1506846	58.25252063	76.17566236	32.39487738	93.70041997	92.38589431	13.53321943	9.52908054	69.95692663	-0.654522704	43.65634884	-0.481266694
	85.31543052	75.49639974	102.1241365	116.0332204	104.4423171	93.49957374	23.52941176	104.6675192	103.0477408	63.05913157	102.7274711	102.0292385	85.56570335	77.28648677	121.9942563	23.16709292	96.14236999	72.01619778	41.58141517	34.07928389	81.22335891	86.44992363	79.27121973	91.20663321	111.0656904	40.89940324	24.19011083	70.99317988	29.13469736	53.78572987	18.05200341
	91.75400866	49.1423423	84.12796698	92.05366357	98.65841073	108.4801132	22.06190619	112.1825062	83.04249585	40.67780948	79.59987525	54.87161124	78.18369453	97.3374613	119.4633643	10.56270928	99.63792775	96.50178314	51.55037705	49.80807448	79.55789072	39.92006746	40.2712162	92.72174928	115.8307534	39.0057986	30.29428579	73.85184984	25.76429913	63.28069953	12.87126017
AMP2CPR3STR1ERY3TMP2	AMP	CPR	STR	ERY	TMP	AMP+CPR	AMP+STR	AMP+ERY	AMP+TMP	CPR+STR	CPR+ERY	CPR+TMP	STR+ERY	STR+TMP	ERY+TMP	AMP+CPR+STR	AMP+CPR+ERY	AMP+CPR+TMP	AMP+STR+ERY	AMP+STR+TMP	AMP+ERY+TMP	CPR+STR+ERY	CPR+STR+TMP	CPR+ERY+TMP	STR+ERY+TMP	AMP+CPR+STR+ERY	AMP+CPR+STR+TMP	AMP+CPR+ERY+TMP	AMP+STR+ERY+TMP	CPR+STR+ERY+TMP	AMP+CPR+STR+ERY+TMP
	70.66920134	86.40016591	99.50227682	111.7017897	100.8577782	63.18069158	30.93582309	68.10886253	84.43342878	83.80774615	98.01420646	94.44703686	75.10325109	65.27586572	89.3360161	11.06913396	66.56880911	76.05938831	31.45559112	3.368866858	70.515196	76.17566236	59.25234614	83.08186862	85.94726252	13.53321943	-3.34961619	47.78015737	0.577520033	7.507647638	6.429723031
	85.31543052	75.49639974	102.1241365	116.0332204	83.90986003	93.49957374	23.52941176	104.6675192	93.84057971	63.05913157	102.7274711	104.4730526	85.56570335	69.00727019	93.84491992	23.16709292	96.14236999	74.06223359	41.58141517	32.71526002	79.1773231	86.44992363	85.55531311	99.2363081	101.1306305	40.89940324	21.46206309	72.69820972	1.342710997	113.1355008	6.798806479
	91.75400866	49.1423423	84.12796698	92.05366357	100.6398349	108.4801132	22.06190619	112.1825062	86.39642828	40.67780948	79.59987525	68.06741132	78.18369453	87.76057792	103.6119711	10.56270928	99.63792775	101.7286908	51.55037705	49.80807448	76.0732856	39.92006746	67.42055837	86.80766521	103.9422085	39.0057986	35.86965399	70.71570522	16.70432581	72.44752983	6.947431465
AMP2CPR3STR1ERY3TMP3	AMP	CPR	STR	ERY	TMP	AMP+CPR	AMP+STR	AMP+ERY	AMP+TMP	CPR+STR	CPR+ERY	CPR+TMP	STR+ERY	STR+TMP	ERY+TMP	AMP+CPR+STR	AMP+CPR+ERY	AMP+CPR+TMP	AMP+STR+ERY	AMP+STR+TMP	AMP+ERY+TMP	CPR+STR+ERY	CPR+STR+TMP	CPR+ERY+TMP	STR+ERY+TMP	AMP+CPR+STR+ERY	AMP+CPR+STR+TMP	AMP+CPR+ERY+TMP	AMP+STR+ERY+TMP	CPR+STR+ERY+TMP	AMP+CPR+STR+ERY+TMP
	70.66920134	86.40016591	99.50227682	111.7017897	81.88075823	63.18069158	30.93582309	68.10886253	76.11714031	83.80774615	98.01420646	105.0655882	75.10325109	74.42550037	80.5252568	11.06913396	66.56880911	64.41273432	31.45559112	15.38128354	82.277354	76.17566236	74.84834344	77.44076321	65.91125702	13.53321943	2.136824121	77.04117237	5.197680295	18.12619899	3.34961619
	85.31543052	75.49639974	102.1241365	116.0332204	64.70207757	93.49957374	23.52941176	104.6675192	94.18158568	63.05913157	102.7274711	102.3783548	85.56570335	68.34493286	128.6176296	23.16709292	96.14236999	83.95140665	41.58141517	44.65046888	68.26513214	86.44992363	84.50796422	92.60309841	83.2475227	40.89940324	39.02387042	63.49104859	-1.641091219	24.45996072	2.536231884
	91.75400866	49.1423423	84.12796698	92.05366357	105.9236326	108.4801132	22.06190619	112.1825062	88.83565187	40.67780948	79.59987525	63.04043986	78.18369453	91.0629515	99.97936017	10.56270928	99.63792775	82.56336264	51.55037705	44.5811668	79.20943021	39.92006746	44.94703892	77.93653911	93.70485036	39.0057986	23.32507555	77.68491547	22.27969401	54.40957342	5.553589416
AMP2CPR3STR2ERY1TMP1	AMP	CPR	STR	ERY	TMP	AMP+CPR	AMP+STR	AMP+ERY	AMP+TMP	CPR+STR	CPR+ERY	CPR+TMP	STR+ERY	STR+TMP	ERY+TMP	AMP+CPR+STR	AMP+CPR+ERY	AMP+CPR+TMP	AMP+STR+ERY	AMP+STR+TMP	AMP+ERY+TMP	CPR+STR+ERY	CPR+STR+TMP	CPR+ERY+TMP	STR+ERY+TMP	AMP+CPR+STR+ERY	AMP+CPR+STR+TMP	AMP+CPR+ERY+TMP	AMP+STR+ERY+TMP	CPR+STR+ERY+TMP	AMP+CPR+STR+ERY+TMP
	70.66920134	86.40016591	68.10886253	101.535529	116.1071693	63.18069158	19.53942777	64.04697163	85.66547152	76.83932182	105.6462902	74.12246591	45.93209327	60.10058474	91.03039288	17.84536901	58.868542	63.48870226	11.37714464	40.79216498	75.50111895	75.5120029	60.84927671	94.69590916	83.20138605	26.16165748	37.53880213	81.96934331	36.69177275	65.88894074	19.98219313
	85.31543052	75.49639974	78.75106564	110.4033531	104.4423171	93.49957374	21.48337596	82.50213129	103.0477408	58.73881737	92.25398211	102.0292385	78.1543052	102.0247229	89.87089597	17.71099744	79.77408355	72.01619778	43.62745098	44.4799659	95.20460358	67.81584115	63.40824787	113.5500764	110.8908781	58.29070759	26.5771526	83.26939471	41.26172208	53.50207288	35.61381074
	91.75400866	49.1423423	67.2311001	91.0629515	98.65841073	108.4801132	26.50477772	82.91182316	83.04249585	49.25323138	52.09938433	54.87161124	81.6486538	77.81558816	112.5283798	14.39577492	93.71409904	96.50178314	67.27465766	44.97318487	82.21490213	43.93055572	45.83415153	78.52794751	75.02790406	41.44502219	35.52119348	79.90635124	61.35082895	58.84513647	37.04570822
AMP2CPR3STR2ERY1TMP2	AMP	CPR	STR	ERY	TMP	AMP+CPR	AMP+STR	AMP+ERY	AMP+TMP	CPR+STR	CPR+ERY	CPR+TMP	STR+ERY	STR+TMP	ERY+TMP	AMP+CPR+STR	AMP+CPR+ERY	AMP+CPR+TMP	AMP+STR+ERY	AMP+STR+TMP	AMP+ERY+TMP	CPR+STR+ERY	CPR+STR+TMP	CPR+ERY+TMP	STR+ERY+TMP	AMP+CPR+STR+ERY	AMP+CPR+STR+TMP	AMP+CPR+ERY+TMP	AMP+STR+ERY+TMP	CPR+STR+ERY+TMP	AMP+CPR+STR+ERY+TMP
	70.66920134	86.40016591	68.10886253	101.535529	100.8577782	63.18069158	19.53942777	64.04697163	84.43342878	76.83932182	105.6462902	94.44703686	45.93209327	64.662993	92.04701896	17.84536901	58.868542	76.05938831	11.37714464	19.05816108	80.73730058	75.5120029	85.072847	92.04127132	86.89751426	26.16165748	24.15958804	84.74143947	10.43386192	51.95209208	-4.63941093
	85.31543052	75.49639974	78.75106564	110.4033531	83.90986003	93.49957374	21.48337596	82.50213129	93.84057971	58.73881737	92.25398211	104.4730526	78.1543052	97.25063939	104.1111485	17.71099744	79.77408355	74.06223359	43.62745098	40.92071611	70.31116795	67.81584115	76.54374864	105.8695178	109.8678602	58.29070759	26.93947144	83.95140665	38.00085251	61.31355008	23.84910486
	91.75400866	49.1423423	67.2311001	91.0629515	100.6398349	108.4801132	26.50477772	82.91182316	86.39642828	49.25323138	52.09938433	68.06741132	81.6486538	86.74488879	107.244582	14.39577492	93.71409904	101.7286908	67.27465766	37.65551412	77.46712765	43.93055572	38.14584224	71.72675083	191.2830425	41.44502219	27.15814118	80.60327226	34.86783002	69.786192	23.80420875
AMP2CPR3STR2ERY1TMP3	AMP	CPR	STR	ERY	TMP	AMP+CPR	AMP+STR	AMP+ERY	AMP+TMP	CPR+STR	CPR+ERY	CPR+TMP	STR+ERY	STR+TMP	ERY+TMP	AMP+CPR+STR	AMP+CPR+ERY	AMP+CPR+TMP	AMP+STR+ERY	AMP+STR+TMP	AMP+ERY+TMP	CPR+STR+ERY	CPR+STR+TMP	CPR+ERY+TMP	STR+ERY+TMP	AMP+CPR+STR+ERY	AMP+CPR+STR+TMP	AMP+CPR+ERY+TMP	AMP+STR+ERY+TMP	CPR+STR+ERY+TMP	AMP+CPR+STR+ERY+TMP
	70.66920134	86.40016591	68.10886253	101.535529	81.88075823	63.18069158	19.53942777	64.04697163	76.11714031	76.83932182	105.6462902	105.0655882	45.93209327	78.2154631	100.8577782	17.84536901	58.868542	64.41273432	11.37714464	19.84743846	70.88095868	75.5120029	68.14953077	101.6643335	74.26907621	26.16165748	38.02006882	89.66961041	32.37962317	56.5977083	20.0014438
	85.31543052	75.49639974	78.75106564	110.4033531	64.70207757	93.49957374	21.48337596	82.50213129	94.18158568	58.73881737	92.25398211	102.3783548	78.1543052	89.06649616	102.7864738	17.71099744	79.77408355	83.95140665	43.62745098	34.07928389	69.62915601	67.81584115	68.51407375	111.4553786	114.3009378	58.29070759	29.30520034	78.1543052	32.39556692	185.9917085	24.2114237
	91.75400866	49.1423423	67.2311001	91.0629515	105.9236326	108.4801132	26.50477772	82.91182316	88.83565187	49.25323138	52.09938433	63.04043986	81.6486538	93.71409904	113.5190918	14.39577492	93.71409904	82.56336264	67.27465766	70.06234176	74.67944355	43.93055572	61.08139951	71.13534243	106.3893502	41.44502219	33.47398797	66.53417908	37.3070536	51.91456921	28.90044374
AMP2CPR3STR2ERY2TMP1	AMP	CPR	STR	ERY	TMP	AMP+CPR	AMP+STR	AMP+ERY	AMP+TMP	CPR+STR	CPR+ERY	CPR+TMP	STR+ERY	STR+TMP	ERY+TMP	AMP+CPR+STR	AMP+CPR+ERY	AMP+CPR+TMP	AMP+STR+ERY	AMP+STR+TMP	AMP+ERY+TMP	CPR+STR+ERY	CPR+STR+TMP	CPR+ERY+TMP	STR+ERY+TMP	AMP+CPR+STR+ERY	AMP+CPR+STR+TMP	AMP+CPR+ERY+TMP	AMP+STR+ERY+TMP	CPR+STR+ERY+TMP	AMP+CPR+STR+ERY+TMP
	70.66920134	86.40016591	68.10886253	104.2465318	116.1071693	63.18069158	19.53942777	52.34256563	85.66547152	76.83932182	106.9736092	74.12246591	46.49036263	60.10058474	88.99714074	17.84536901	63.79671295	63.48870226	24.46759872	40.79216498	71.80499074	103.6553119	60.84927671	101.000674	86.89751426	21.21423587	37.53880213	69.64891595	4.966672281	53.94307046	-7.969776452
	85.31543052	75.49639974	78.75106564	119.344907	104.4423171	93.49957374	21.48337596	95.46035806	103.0477408	58.73881737	91.55574951	102.0292385	74.31798806	102.0247229	95.50076324	17.71099744	89.66325661	72.01619778	47.03751066	44.4799659	75	78.63844643	63.40824787	110.757146	103.0477408	61.01875533	26.5771526	70.99317988	31.69224211	58.86973598	10.89087809
	91.75400866	49.1423423	67.2311001	93.374613	98.65841073	108.4801132	26.50477772	97.89562519	83.04249585	49.25323138	60.08339783	54.87161124	72.10954727	77.81558816	113.1888545	14.39577492	99.28946724	96.50178314	50.156535	44.97318487	75.94261291	42.7477389	45.83415153	80.19128366	96.28399532	48.41423243	35.52119348	70.36724471	32.38504887	64.62984995	33.77889092
AMP2CPR3STR2ERY2TMP2	AMP	CPR	STR	ERY	TMP	AMP+CPR	AMP+STR	AMP+ERY	AMP+TMP	CPR+STR	CPR+ERY	CPR+TMP	STR+ERY	STR+TMP	ERY+TMP	AMP+CPR+STR	AMP+CPR+ERY	AMP+CPR+TMP	AMP+STR+ERY	AMP+STR+TMP	AMP+ERY+TMP	CPR+STR+ERY	CPR+STR+TMP	CPR+ERY+TMP	STR+ERY+TMP	AMP+CPR+STR+ERY	AMP+CPR+STR+TMP	AMP+CPR+ERY+TMP	AMP+STR+ERY+TMP	CPR+STR+ERY+TMP	AMP+CPR+STR+ERY+TMP
	70.66920134	86.40016591	68.10886253	104.2465318	100.8577782	63.18069158	19.53942777	52.34256563	84.43342878	76.83932182	106.9736092	94.44703686	46.49036263	64.662993	96.79127396	17.84536901	63.79671295	76.05938831	24.46759872	19.05816108	65.02875568	103.6553119	85.072847	90.7139524	87.51353563	21.21423587	24.15958804	65.02875568	16.59407561	58.92051641	21.52224655
	85.31543052	75.49639974	78.75106564	119.344907	83.90986003	93.49957374	21.48337596	95.46035806	93.84057971	58.73881737	91.55574951	104.4730526	74.31798806	97.25063939	104.7734858	17.71099744	89.66325661	74.06223359	47.03751066	40.92071611	69.88491049	78.63844643	76.54374864	103.0765874	87.02046036	61.01875533	26.93947144	66.21909633	35.10230179	57.34235217	16.0059676
	91.75400866	49.1423423	67.2311001	93.374613	100.6398349	108.4801132	26.50477772	97.89562519	86.39642828	49.25323138	60.08339783	68.06741132	72.10954727	86.74488879	111.8679051	14.39577492	99.28946724	101.7286908	50.156535	37.65551412	67.01331228	42.7477389	38.14584224	77.6408349	68.40715433	48.41423243	27.15814118	87.79027033	35.17273296	62.98499532	25.06737811
AMP2CPR3STR2ERY2TMP3	AMP	CPR	STR	ERY	TMP	AMP+CPR	AMP+STR	AMP+ERY	AMP+TMP	CPR+STR	CPR+ERY	CPR+TMP	STR+ERY	STR+TMP	ERY+TMP	AMP+CPR+STR	AMP+CPR+ERY	AMP+CPR+TMP	AMP+STR+ERY	AMP+STR+TMP	AMP+ERY+TMP	CPR+STR+ERY	CPR+STR+TMP	CPR+ERY+TMP	STR+ERY+TMP	AMP+CPR+STR+ERY	AMP+CPR+STR+TMP	AMP+CPR+ERY+TMP	AMP+STR+ERY+TMP	CPR+STR+ERY+TMP	AMP+CPR+STR+ERY+TMP
	70.66920134	86.40016591	68.10886253	104.2465318	81.88075823	63.18069158	19.53942777	52.34256563	76.11714031	76.83932182	106.9736092	105.0655882	46.49036263	78.2154631	100.1800275	17.84536901	63.79671295	64.41273432	24.46759872	19.84743846	60.71660611	103.6553119	68.14953077	100.6688443	87.20552494	21.21423587	38.02006882	73.65305484	23.13930264	66.22077047	30.6278124
	85.31543052	75.49639974	78.75106564	119.344907	64.70207757	93.49957374	21.48337596	95.46035806	94.18158568	58.73881737	91.55574951	102.3783548	74.31798806	89.06649616	113.7150397	17.71099744	89.66325661	83.95140665	47.03751066	34.07928389	79.51832907	78.63844643	68.51407375	97.8398429	110.5498721	61.01875533	29.30520034	78.49531117	28.96419437	51.53829369	32.73657289
	91.75400866	49.1423423	67.2311001	93.374613	105.9236326	108.4801132	26.50477772	97.89562519	88.83565187	49.25323138	60.08339783	63.04043986	72.10954727	93.71409904	113.8493292	14.39577492	99.28946724	82.56336264	50.156535	70.06234176	65.48879754	42.7477389	61.08139951	93.01745348	98.02629788	48.41423243	33.47398797	69.32186317	33.08196989	73.63034664	17.40124684
AMP2CPR3STR2ERY3TMP1	AMP	CPR	STR	ERY	TMP	AMP+CPR	AMP+STR	AMP+ERY	AMP+TMP	CPR+STR	CPR+ERY	CPR+TMP	STR+ERY	STR+TMP	ERY+TMP	AMP+CPR+STR	AMP+CPR+ERY	AMP+CPR+TMP	AMP+STR+ERY	AMP+STR+TMP	AMP+ERY+TMP	CPR+STR+ERY	CPR+STR+TMP	CPR+ERY+TMP	STR+ERY+TMP	AMP+CPR+STR+ERY	AMP+CPR+STR+TMP	AMP+CPR+ERY+TMP	AMP+STR+ERY+TMP	CPR+STR+ERY+TMP	AMP+CPR+STR+ERY+TMP
	70.66920134	86.40016591	68.10886253	111.7017897	116.1071693	63.18069158	19.53942777	68.10886253	85.66547152	76.83932182	98.01420646	74.12246591	50.24424285	60.10058474	100.1800275	17.84536901	66.56880911	63.48870226	10.60711793	40.79216498	58.25252063	103.3234821	60.84927671	93.70041997	69.28315326	36.32601006	37.53880213	69.95692663	15.67004355	82.48042723	35.38272734
	85.31543052	75.49639974	78.75106564	116.0332204	104.4423171	93.49957374	21.48337596	104.6675192	103.0477408	58.73881737	102.7274711	102.0292385	75	102.0247229	121.9942563	17.71099744	96.14236999	72.01619778	46.01449275	44.4799659	81.22335891	92.25398211	63.40824787	91.20663321	105.4347826	42.26342711	26.5771526	70.99317988	49.78687127	56.42592189	19.07502131
	91.75400866	49.1423423	67.2311001	92.05366357	98.65841073	108.4801132	26.50477772	112.1825062	83.04249585	49.25323138	79.59987525	54.87161124	75.94261291	77.81558816	119.4633643	14.39577492	99.63792775	96.50178314	44.92962731	44.97318487	79.55789072	48.95752717	45.83415153	92.72174928	80.95173277	38.30887758	35.52119348	73.85184984	46.36702692	66.23774156	17.05278632
AMP2CPR3STR2ERY3TMP2	AMP	CPR	STR	ERY	TMP	AMP+CPR	AMP+STR	AMP+ERY	AMP+TMP	CPR+STR	CPR+ERY	CPR+TMP	STR+ERY	STR+TMP	ERY+TMP	AMP+CPR+STR	AMP+CPR+ERY	AMP+CPR+TMP	AMP+STR+ERY	AMP+STR+TMP	AMP+ERY+TMP	CPR+STR+ERY	CPR+STR+TMP	CPR+ERY+TMP	STR+ERY+TMP	AMP+CPR+STR+ERY	AMP+CPR+STR+TMP	AMP+CPR+ERY+TMP	AMP+STR+ERY+TMP	CPR+STR+ERY+TMP	AMP+CPR+STR+ERY+TMP
	70.66920134	86.40016591	68.10886253	111.7017897	100.8577782	63.18069158	19.53942777	68.10886253	84.43342878	76.83932182	98.01420646	94.44703686	50.24424285	64.662993	89.3360161	17.84536901	66.56880911	76.05938831	10.60711793	19.05816108	70.515196	103.3234821	85.072847	83.08186862	81.29556994	36.32601006	24.15958804	47.78015737	36.61477007	54.93855965	3.965637558
	85.31543052	75.49639974	78.75106564	116.0332204	83.90986003	93.49957374	21.48337596	104.6675192	93.84057971	58.73881737	102.7274711	104.4730526	75	97.25063939	93.84491992	17.71099744	96.14236999	74.06223359	46.01449275	40.92071611	79.1773231	92.25398211	76.54374864	99.2363081	79.85933504	42.26342711	26.93947144	72.69820972	9.867860188	62.01178268	6.798806479
	91.75400866	49.1423423	67.2311001	92.05366357	100.6398349	108.4801132	26.50477772	112.1825062	86.39642828	49.25323138	79.59987525	68.06741132	75.94261291	86.74488879	103.6119711	14.39577492	99.63792775	101.7286908	44.92962731	37.65551412	76.0732856	48.95752717	38.14584224	86.80766521	76.63953393	38.30887758	27.15814118	70.71570522	3.811286854	55.59239024	18.22884055
AMP2CPR3STR2ERY3TMP3	AMP	CPR	STR	ERY	TMP	AMP+CPR	AMP+STR	AMP+ERY	AMP+TMP	CPR+STR	CPR+ERY	CPR+TMP	STR+ERY	STR+TMP	ERY+TMP	AMP+CPR+STR	AMP+CPR+ERY	AMP+CPR+TMP	AMP+STR+ERY	AMP+STR+TMP	AMP+ERY+TMP	CPR+STR+ERY	CPR+STR+TMP	CPR+ERY+TMP	STR+ERY+TMP	AMP+CPR+STR+ERY	AMP+CPR+STR+TMP	AMP+CPR+ERY+TMP	AMP+STR+ERY+TMP	CPR+STR+ERY+TMP	AMP+CPR+STR+ERY+TMP
	70.66920134	86.40016591	68.10886253	111.7017897	81.88075823	63.18069158	19.53942777	68.10886253	76.11714031	76.83932182	98.01420646	105.0655882	50.24424285	78.2154631	80.5252568	17.84536901	66.56880911	64.41273432	10.60711793	19.84743846	82.277354	103.3234821	68.14953077	77.44076321	73.96106552	36.32601006	38.02006882	77.04117237	30.45455639	74.18468398	3.657626874
	85.31543052	75.49639974	78.75106564	116.0332204	64.70207757	93.49957374	21.48337596	104.6675192	94.18158568	58.73881737	102.7274711	102.3783548	75	89.06649616	128.6176296	17.71099744	96.14236999	83.95140665	46.01449275	34.07928389	68.26513214	92.25398211	68.51407375	92.60309841	81.22335891	42.26342711	29.30520034	63.49104859	1.427962489	65.85206197	9.015345269
	91.75400866	49.1423423	67.2311001	92.05366357	105.9236326	108.4801132	26.50477772	112.1825062	88.83565187	49.25323138	79.59987525	63.04043986	75.94261291	93.71409904	99.97936017	14.39577492	99.63792775	82.56336264	44.92962731	70.06234176	79.20943021	48.95752717	61.08139951	77.93653911	82.69403534	38.30887758	33.47398797	77.68491547	40.05118014	55.59239024	14.96202325
AMP2CPR3STR3ERY1TMP1	AMP	CPR	STR	ERY	TMP	AMP+CPR	AMP+STR	AMP+ERY	AMP+TMP	CPR+STR	CPR+ERY	CPR+TMP	STR+ERY	STR+TMP	ERY+TMP	AMP+CPR+STR	AMP+CPR+ERY	AMP+CPR+TMP	AMP+STR+ERY	AMP+STR+TMP	AMP+ERY+TMP	CPR+STR+ERY	CPR+STR+TMP	CPR+ERY+TMP	STR+ERY+TMP	AMP+CPR+STR+ERY	AMP+CPR+STR+TMP	AMP+CPR+ERY+TMP	AMP+STR+ERY+TMP	CPR+STR+ERY+TMP	AMP+CPR+STR+ERY+TMP
	70.66920134	86.40016591	74.20542334	101.535529	116.1071693	63.18069158	32.62988185	64.04697163	85.66547152	85.072847	105.6462902	74.12246591	97.01871727	108.9645875	91.03039288	21.23348654	58.868542	63.48870226	8.913059172	55.17241379	75.50111895	103.3234821	104.6508011	94.69590916	109.2964173	24.31359338	40.07989027	81.96934331	65.33676637	78.4362524	32.07161248
	85.31543052	75.49639974	60.83351516	110.4033531	104.4423171	93.49957374	38.51236147	82.50213129	103.0477408	88.76281911	92.25398211	102.0292385	81.43137683	106.9168667	89.87089597	29.7314578	79.77408355	72.01619778	40.89940324	77.47229327	95.20460358	86.66812132	100.6327733	113.5500764	116.6921231	55.56265985	35.78431373	83.26939471	54.28388747	79.33667903	61.44501279

	91.75400866	49.1423423	64.81466508	91.0629515	98.65841073	108.4801132	41.92415539	82.91182316	83.04249585	60.1942869	52.09938433	54.87161124	42.93255403	51.80368013	112.5283798	41.92415539	93.71409904	96.50178314	44.5811668	49.93874718	82.21490213	51.91456921	56.35013226	78.52794751	73.79668026	53.07489179	47.3688509	79.90635124	53.4233523	73.79668026	46.32346936
AMP2CPR3STR3ERY1TMP2	AMP	CPR	STR	ERY	TMP	AMP+CPR	AMP+STR	AMP+ERY	AMP+TMP	CPR+STR	CPR+ERY	CPR+TMP	STR+ERY	STR+TMP	ERY+TMP	AMP+CPR+STR	AMP+CPR+ERY	AMP+CPR+TMP	AMP+STR+ERY	AMP+STR+TMP	AMP+ERY+TMP	CPR+STR+ERY	CPR+STR+TMP	CPR+ERY+TMP	STR+ERY+TMP	AMP+CPR+STR+ERY	AMP+CPR+STR+TMP	AMP+CPR+ERY+TMP	AMP+STR+ERY+TMP	CPR+STR+ERY+TMP	AMP+CPR+STR+ERY+TMP
	70.66920134	86.40016591	74.20542334	101.535529	100.8577782	63.18069158	32.62988185	64.04697163	84.43342878	85.072847	105.6462902	94.44703686	97.01871727	90.7139524	92.04701896	21.23348654	58.868542	76.05938831	8.913059172	37.86606348	80.73730058	103.3234821	104.9826308	92.04127132	108.6327578	24.31359338	24.37134538	84.74143947	48.08816806	88.05931456	39.15585822
	85.31543052	75.49639974	60.83351516	110.4033531	83.90986003	93.49957374	38.51236147	82.50213129	93.84057971	88.76281911	92.25398211	104.4730526	81.43137683	104.1239363	104.1111485	29.7314578	79.77408355	74.06223359	40.89940324	45.33248082	70.31116795	86.66812132	98.8871918	105.8695178	116.6921231	55.56265985	37.23358909	83.95140665	52.9198636	97.8398429	43.03069054
	91.75400866	49.1423423	64.81466508	91.0629515	100.6398349	108.4801132	41.92415539	82.91182316	86.39642828	60.1942869	52.09938433	68.06741132	42.93255403	58.12435748	107.244582	41.92415539	93.71409904	101.7286908	44.5811668	27.89861977	77.46712765	51.91456921	53.09738602	71.72675083	80.00646853	53.07489179	43.18732475	80.60327226	60.04410203	79.11935592	25.54651131
AMP2CPR3STR3ERY1TMP3	AMP	CPR	STR	ERY	TMP	AMP+CPR	AMP+STR	AMP+ERY	AMP+TMP	CPR+STR	CPR+ERY	CPR+TMP	STR+ERY	STR+TMP	ERY+TMP	AMP+CPR+STR	AMP+CPR+ERY	AMP+CPR+TMP	AMP+STR+ERY	AMP+STR+TMP	AMP+ERY+TMP	CPR+STR+ERY	CPR+STR+TMP	CPR+ERY+TMP	STR+ERY+TMP	AMP+CPR+STR+ERY	AMP+CPR+STR+TMP	AMP+CPR+ERY+TMP	AMP+STR+ERY+TMP	CPR+STR+ERY+TMP	AMP+CPR+STR+ERY+TMP
	70.66920134	86.40016591	74.20542334	101.535529	81.88075823	63.18069158	32.62988185	64.04697163	76.11714031	85.072847	105.6462902	105.0655882	97.01871727	91.4605693	100.8577782	21.23348654	58.868542	64.41273432	8.913059172	49.93623216	70.88095868	103.3234821	91.37761186	101.6643335	107.9690984	24.31359338	29.54977501	89.66961041	49.01220011	82.41820916	57.57874726
	85.31543052	75.49639974	60.83351516	110.4033531	64.70207757	93.49957374	38.51236147	82.50213129	94.18158568	88.76281911	92.25398211	102.3783548	81.43137683	102.0292385	102.7864738	29.7314578	79.77408355	83.95140665	40.89940324	48.48678602	69.62915601	86.66812132	105.1712852	111.4553786	109.3606808	55.56265985	45.07672634	78.1543052	39.27962489	96.4433777	51.21483376
	91.75400866	49.1423423	64.81466508	91.0629515	105.9236326	108.4801132	41.92415539	82.91182316	88.83565187	60.1942869	52.09938433	63.04043986	42.93255403	70.72874915	113.5190918	41.92415539	93.71409904	82.56336264	44.5811668	27.85506221	74.67944355	51.91456921	66.10837097	71.13534243	75.57090548	53.07489179	41.7934827	66.53417908	57.25641793	74.38808867	39.83339232
AMP2CPR3STR3ERY2TMP1	AMP	CPR	STR	ERY	TMP	AMP+CPR	AMP+STR	AMP+ERY	AMP+TMP	CPR+STR	CPR+ERY	CPR+TMP	STR+ERY	STR+TMP	ERY+TMP	AMP+CPR+STR	AMP+CPR+ERY	AMP+CPR+TMP	AMP+STR+ERY	AMP+STR+TMP	AMP+ERY+TMP	CPR+STR+ERY	CPR+STR+TMP	CPR+ERY+TMP	STR+ERY+TMP	AMP+CPR+STR+ERY	AMP+CPR+STR+TMP	AMP+CPR+ERY+TMP	AMP+STR+ERY+TMP	CPR+STR+ERY+TMP	AMP+CPR+STR+ERY+TMP
	70.66920134	86.40016591	74.20542334	104.2465318	116.1071693	63.18069158	32.62988185	52.34256563	85.66547152	85.072847	106.9736092	74.12246591	97.68237673	108.9645875	88.99714074	21.23348654	63.79671295	63.48870226	26.46966817	55.17241379	71.80499074	103.9871416	104.6508011	101.000674	103.9871416	24.60235339	40.07989027	69.64891595	57.0204779	91.37761186	53.94037106
	85.31543052	75.49639974	60.83351516	119.344907	104.4423171	93.49957374	38.51236147	95.46035806	103.0477408	88.76281911	91.55574951	102.0292385	88.76281911	106.9168667	95.50076324	29.7314578	89.66325661	72.01619778	65.11082694	77.47229327	75	88.76281911	100.6327733	110.757146	111.1062623	61.3597613	35.78431373	70.99317988	35.78431373	106.9168667	46.09974425
	91.75400866	49.1423423	64.81466508	93.374613	98.65841073	108.4801132	41.92415539	97.89562519	83.04249585	60.1942869	60.08339783	54.87161124	64.81466508	51.80368013	113.1888545	41.92415539	99.28946724	96.50178314	63.04957395	49.93874718	75.94261291	43.33914731	56.35013226	80.19128366	73.09438278	65.48879754	47.3688509	70.36724471	42.96953693	85.32914419	33.08196989
AMP2CPR3STR3ERY2TMP2	AMP	CPR	STR	ERY	TMP	AMP+CPR	AMP+STR	AMP+ERY	AMP+TMP	CPR+STR	CPR+ERY	CPR+TMP	STR+ERY	STR+TMP	ERY+TMP	AMP+CPR+STR	AMP+CPR+ERY	AMP+CPR+TMP	AMP+STR+ERY	AMP+STR+TMP	AMP+ERY+TMP	CPR+STR+ERY	CPR+STR+TMP	CPR+ERY+TMP	STR+ERY+TMP	AMP+CPR+STR+ERY	AMP+CPR+STR+TMP	AMP+CPR+ERY+TMP	AMP+STR+ERY+TMP	CPR+STR+ERY+TMP	AMP+CPR+STR+ERY+TMP
	70.66920134	86.40016591	74.20542334	104.2465318	100.8577782	63.18069158	32.62988185	52.34256563	84.43342878	85.072847	106.9736092	94.44703686	97.68237673	90.7139524	96.79127396	21.23348654	63.79671295	76.05938831	26.46966817	37.86606348	65.02875568	103.9871416	104.9826308	90.7139524	106.6417794	24.60235339	24.37134538	65.02875568	32.68763385	89.38663348	41.00392232
	85.31543052	75.49639974	60.83351516	119.344907	83.90986003	93.49957374	38.51236147	95.46035806	93.84057971	88.76281911	91.55574951	104.4730526	88.76281911	104.1239363	104.7734858	29.7314578	89.66325661	74.06223359	65.11082694	45.33248082	69.88491049	88.76281911	98.8871918	103.0765874	106.5677504	61.3597613	37.23358909	66.21909633	32.71526002	102.7274711	40.64364876
	91.75400866	49.1423423	64.81466508	93.374613	100.6398349	108.4801132	41.92415539	97.89562519	86.39642828	60.1942869	60.08339783	68.06741132	64.81466508	58.12435748	111.8679051	41.92415539	99.28946724	101.7286908	63.04957395	27.89861977	67.01331228	43.33914731	53.09738602	77.6408349	77.93653911	65.48879754	43.18732475	87.79027033	39.70271963	78.23224331	32.03658835
AMP2CPR3STR3ERY2TMP3	AMP	CPR	STR	ERY	TMP	AMP+CPR	AMP+STR	AMP+ERY	AMP+TMP	CPR+STR	CPR+ERY	CPR+TMP	STR+ERY	STR+TMP	ERY+TMP	AMP+CPR+STR	AMP+CPR+ERY	AMP+CPR+TMP	AMP+STR+ERY	AMP+STR+TMP	AMP+ERY+TMP	CPR+STR+ERY	CPR+STR+TMP	CPR+ERY+TMP	STR+ERY+TMP	AMP+CPR+STR+ERY	AMP+CPR+STR+TMP	AMP+CPR+ERY+TMP	AMP+STR+ERY+TMP	CPR+STR+ERY+TMP	AMP+CPR+STR+ERY+TMP
	70.66920134	86.40016591	74.20542334	104.2465318	81.88075823	63.18069158	32.62988185	52.34256563	76.11714031	85.072847	106.9736092	105.0655882	97.68237673	91.4605693	100.1800275	21.23348654	63.79671295	64.41273432	26.46966817	49.93623216	60.71660611	103.9871416	91.37761186	100.6688443	107.6372686	24.60235339	29.54977501	73.65305484	38.53983685	89.05480375	54.24838174
	85.31543052	75.49639974	60.83351516	119.344907	64.70207757	93.49957374	38.51236147	95.46035806	94.18158568	88.76281911	91.55574951	102.3783548	88.76281911	102.0292385	113.7150397	29.7314578	89.66325661	83.95140665	65.11082694	48.48678602	79.51832907	88.76281911	105.1712852	97.8398429	103.0765874	61.3597613	45.07672634	78.49531117	46.78175618	94.3486799	64.17306053
	91.75400866	49.1423423	64.81466508	93.374613	105.9236326	108.4801132	41.92415539	97.89562519	88.83565187	60.1942869	60.08339783	63.04043986	64.81466508	70.72874915	113.8493292	41.92415539	99.28946724	82.56336264	63.04957395	27.85506221	65.48879754	43.33914731	66.10837097	93.01745348	88.58189043	65.48879754	41.7934827	69.32186317	46.80260256	80.89358114	45.40876052
AMP2CPR3STR3ERY3TMP1	AMP	CPR	STR	ERY	TMP	AMP+CPR	AMP+STR	AMP+ERY	AMP+TMP	CPR+STR	CPR+ERY	CPR+TMP	STR+ERY	STR+TMP	ERY+TMP	AMP+CPR+STR	AMP+CPR+ERY	AMP+CPR+TMP	AMP+STR+ERY	AMP+STR+TMP	AMP+ERY+TMP	CPR+STR+ERY	CPR+STR+TMP	CPR+ERY+TMP	STR+ERY+TMP	AMP+CPR+STR+ERY	AMP+CPR+STR+TMP	AMP+CPR+ERY+TMP	AMP+STR+ERY+TMP	CPR+STR+ERY+TMP	AMP+CPR+STR+ERY+TMP
	70.66920134	86.40016591	74.20542334	111.7017897	116.1071693	63.18069158	32.62988185	68.10886253	85.66547152	85.072847	98.01420646	74.12246591	92.37310105	108.9645875	100.1800275	21.23348654	66.56880911	63.48870226	20.0014438	55.17241379	58.25252063	100.3370146	104.6508011	93.70041997	100.6688443	42.79423443	40.07989027	69.95692663	32.37962317	67.48587131	29.9155377
	85.31543052	75.49639974	60.83351516	116.0332204	104.4423171	93.49957374	38.51236147	104.6675192	103.0477408	88.76281911	102.7274711	102.0292385	95.0469125	106.9168667	121.9942563	29.7314578	96.14236999	72.01619778	59.65473146	77.47229327	81.22335891	86.31900502	100.6327733	91.20663321	105.8695178	50.44757033	35.78431373	70.99317988	57.01193521	76.54374864	25.98039216
	91.75400866	49.1423423	64.81466508	92.05366357	98.65841073	108.4801132	41.92415539	112.1825062	83.04249585	60.1942869	79.59987525	54.87161124	67.47600291	51.80368013	119.4633643	41.92415539	99.63792775	96.50178314	60.61035037	49.93874718	79.55789072	46.18530027	56.35013226	92.72174928	84.14632738	63.74649498	47.3688509	73.85184984	51.33258922	93.31315768	27.28881387
AMP2CPR3STR3ERY3TMP2	AMP	CPR	STR	ERY	TMP	AMP+CPR	AMP+STR	AMP+ERY	AMP+TMP	CPR+STR	CPR+ERY	CPR+TMP	STR+ERY	STR+TMP	ERY+TMP	AMP+CPR+STR	AMP+CPR+ERY	AMP+CPR+TMP	AMP+STR+ERY	AMP+STR+TMP	AMP+ERY+TMP	CPR+STR+ERY	CPR+STR+TMP	CPR+ERY+TMP	STR+ERY+TMP	AMP+CPR+STR+ERY	AMP+CPR+STR+TMP	AMP+CPR+ERY+TMP	AMP+STR+ERY+TMP	CPR+STR+ERY+TMP	AMP+CPR+STR+ERY+TMP
	70.66920134	86.40016591	74.20542334	111.7017897	100.8577782	63.18069158	32.62988185	68.10886253	84.43342878	85.072847	98.01420646	94.44703686	92.37310105	90.7139524	89.3360161	21.23348654	66.56880911	76.05938831	20.0014438	37.86606348	70.515196	100.3370146	104.9826308	83.08186862	74.45429564	42.79423443	24.37134538	47.78015737	20.0014438	68.14953077	20.46345983
	85.31543052	75.49639974	60.83351516	116.0332204	83.90986003	93.49957374	38.51236147	104.6675192	93.84057971	88.76281911	102.7274711	104.4730526	95.0469125	104.1239363	93.84491992	29.7314578	96.14236999	74.06223359	59.65473146	45.33248082	79.1773231	86.31900502	98.8871918	99.2363081	92.95221471	50.44757033	37.23358909	72.69820972	35.86956522	98.5380755	26.59846547
	91.75400866	49.1423423	64.81466508	92.05366357	100.6398349	108.4801132	41.92415539	112.1825062	86.39642828	60.1942869	79.59987525	68.06741132	67.47600291	58.12435748	103.6119711	41.92415539	99.63792775	101.7286908	60.61035037	27.89861977	76.0732856	46.18530027	53.09738602	80.80766521	85.03343999	63.74649498	43.18732475	70.71570522	56.2110364	92.72174928	45.40876052
AMP2CPR3STR3ERY3TMP3	AMP	CPR	STR	ERY	TMP	AMP+CPR	AMP+STR	AMP+ERY	AMP+TMP	CPR+STR	CPR+ERY	CPR+TMP	STR+ERY	STR+TMP	ERY+TMP	AMP+CPR+STR	AMP+CPR+ERY	AMP+CPR+TMP	AMP+STR+ERY	AMP+STR+TMP	AMP+ERY+TMP	CPR+STR+ERY	CPR+STR+TMP	CPR+ERY+TMP	STR+ERY+TMP	AMP+CPR+STR+ERY	AMP+CPR+STR+TMP	AMP+CPR+ERY+TMP	AMP+STR+ERY+TMP	CPR+STR+ERY+TMP	AMP+CPR+STR+ERY+TMP
	70.66920134	86.40016591	74.20542334	111.7017897	81.88075823	63.18069158	32.62988185	68.10886253	76.11714031	85.072847	98.01420646	105.0655882	92.37310105	91.4605693	80.5252568	21.23348654	66.56880911	64.41273432	20.0014438	49.93623216	82.277354	100.3370146	91.37761186	77.44076321	91.70944159	42.79423443	29.54977501	77.04117237	47.78015737	53.88085239	16.97908896
	85.31543052	75.49639974	60.83351516	116.0332204	64.70207757	93.49957374	38.51236147	104.6675192	94.18158568	88.76281911	102.7274711	102.3783548	95.0469125	102.0292385	128.6176296	29.7314578	96.14236999	83.95140665	59.65473146	48.48678602	68.26513214	86.31900502	105.1712852	92.60309841	98.1889592	50.44757033	45.07672634	63.49104859	43.03069054	76.54374864	13.10741688
	91.75400866	49.1423423	64.81466508	92.05366357	105.9236326	108.4801132	41.92415539	112.1825062	88.83565187	60.1942869	79.59987525	63.04043986	67.47600291	70.72874915	99.97936017	41.92415539	99.63792775	82.56336264	60.61035037	27.85506221	79.20943021	46.18530027	66.10837097	77.93653911	77.3451307	63.74649498	41.7934827	77.68491547	26.11275964	82.07639795	17.40124684
AMP3CPR1STR1ERY1TMP1	AMP	CPR	STR	ERY	TMP	AMP+CPR	AMP+STR	AMP+ERY	AMP+TMP	CPR+STR	CPR+ERY	CPR+TMP	STR+ERY	STR+TMP	ERY+TMP	AMP+CPR+STR	AMP+CPR+ERY	AMP+CPR+TMP	AMP+STR+ERY	AMP+STR+TMP	AMP+ERY+TMP	CPR+STR+ERY	CPR+STR+TMP	CPR+ERY+TMP	STR+ERY+TMP	AMP+CPR+STR+ERY	AMP+CPR+STR+TMP	AMP+CPR+ERY+TMP	AMP+STR+ERY+TMP	CPR+STR+ERY+TMP	AMP+CPR+STR+ERY+TMP
	90.7139524	102.552155	99.50227682	101.535529	116.1071693	96.68688754	14.0612848	82.41820916	94.69590916	96.11352324	107.29641	75.10325109	112.3795404	83.23625966	91.03039288	51.22621455	81.42271997	51.22621455	40.69062063	46.97464613	73.45880645	43.43958488	38.5258922	85.60838717	92.38589431	46.66355576	44.98366776	66.82221185	22.43998548	1.524939108	50.89438482
	82.47872573	110.7345217	102.1241365	110.4033531	104.4423171	87.71547022	20.27056513	86.31900502	106.5677504	70.78730175	107.4228351	95.8319319	132.9228222	77.28648677	89.87089597	59.61160812	97.1416103	84.92253982	36.6790312	44.25049094	102.0292385	58.98941813	36.34576078	78.27999276	78.61116142	90.15928431	70.95788785	68.51407375	31.33318787	-9.231326486	48.96356099
	54.70527763	98.65841073	84.12796698	91.0629515	98.65841073	73.50097605	35.53994894	53.09738602	59.78769362	50.25799794	86.76986584	102.621259	79.50464396	97.3374613	112.5283798	48.36611877	77.93653911	84.73773578	50.91656752	47.01696834	69.36111721	52.69349845	60.49535604	97.66769866	99.31888545	71.13534243	75.86660968	57.53294907	39.08839939	5.345717234	46.59189355
AMP3CPR1STR1ERY1TMP2	AMP	CPR	STR	ERY	TMP	AMP+CPR	AMP+STR	AMP+ERY	AMP+TMP	CPR+STR	CPR+ERY	CPR+TMP	STR+ERY	STR+TMP	ERY+TMP	AMP+CPR+STR	AMP+CPR+ERY	AMP+CPR+TMP	AMP+STR+ERY	AMP+STR+TMP	AMP+ERY+TMP	CPR+STR+ERY	CPR+STR+TMP	CPR+ERY+TMP	STR+ERY+TMP	AMP+CPR+STR+ERY	AMP+CPR+STR+TMP	AMP+CPR+ERY+TMP	AMP+STR+ERY+TMP	CPR+STR+ERY+TMP	AMP+CPR+STR+ERY+TMP
	90.7139524	102.552155	99.50227682	101.535529	100.8577782	96.68688754	14.0612848	82.41820916	94.11520713	96.11352324	107.29641	74.42550037	112.3795404	65.27586572	92.04701896	51.22621455	81.42271997	71.21895577	40.69062063	51.30917198	67.90065847	43.43958488	43.77846024	78.1531293	85.60838717	46.66355576	33.9710686	69.14501996	11.4896044	15.41882876	58.52646861
	82.47872573	110.7345217	102.1241365	110.4033531	83.90986003	87.71547022	20.27056513	86.31900502	100.1090988	70.78730175	107.4228351	71.32545083	132.9228222	69.00727019	104.1111485	59.61160812	97.1416103	68.51407375	36.6790312	44.25049094	85.09709797	58.98941813	25.54138315	79.93583607	95.16959458	90.15928431	53.85118918	60.83351516	28.19114117	-9.686683398	68.86319005
	54.70527763	98.65841073	84.12796698	91.0629515	100.6398349	73.50097605	35.53994894	53.09738602	79.00846684	50.25799794	86.76986584	105.5933953	79.50464396	87.76057792	107.244582	48.36611877	77.93653911	67.88259619	50.91656752	25.19030183	67.77170711	52.69349845	0.722394221	101.9607843	100.6398349	71.13534243	51.3231608	42.4520347	32.58290691	4.024767802	44.81766833
AMP3CPR1STR1ERY1TMP3	AMP	CPR	STR	ERY	TMP	AMP+CPR	AMP+STR	AMP+ERY	AMP+TMP	CPR+STR	CPR+ERY	CPR+TMP	STR+ERY	STR+TMP	ERY+TMP	AMP+CPR+STR	AMP+CPR+ERY	AMP+CPR+TMP	AMP+STR+ERY	AMP+STR+TMP	AMP+ERY+TMP	CPR+STR+ERY	CPR+STR+TMP	CPR+ERY+TMP	STR+ERY+TMP	AMP+CPR+STR+ERY	AMP+CPR+STR+TMP	AMP+CPR+ERY+TMP	AMP+STR+ERY+TMP	CPR+STR+ERY+TMP	AMP+CPR+STR+ERY+TMP
	90.7139524	102.552155	99.50227682	101.535529	81.88075823	96.68688754	14.0612848	82.41820916	101.0836315	96.11352324	107.29641	76.11987716	112.3795404	74.42550037	100.8577782	51.22621455	81.42271997	68.56431793	40.69062063	44.34074765	81.7545497	43.43958488	9.319072329	94.41914646	92.38589431	46.66355576	32.97557941	79.09991186	7.507647638	43.43958488	69.47684969
	82.47872573	110.7345217	102.1241365	110.4033531	64.70207757	87.71547022	20.27056513	86.31900502	97.66528475	70.78730175	107.4228351	87.55271532	132.9228222	68.34493286	102.7864738	59.61160812	97.1416103	71.65612045	36.6790312	41.80667685	80.03491163	58.98941813	-6.250808517	94.17608859	92.85141393	90.15928431	60.83351516	70.25965525	30.63495527	-6.374996766	33.25332751
	54.70527763	98.65841073	84.12796698	91.0629515	105.9236326	73.50097605	35.53994894	53.09738602	82.26121308	50.25799794	86.76986584	89.74200206	79.50464396	91.0629515	113.5190918	48.36611877	77.93653911	61.67280792	50.91656752	38.73725064	67.47600291	52.69349845	2.373581011	108.5655315	108.8957688	71.13534243	48.8466381	53.39309022	38.79269518	9.494324045	27.85163966
AMP3CPR1STR1ERY2TMP1	AMP	CPR	STR	ERY	TMP	AMP+CPR	AMP+STR	AMP+ERY	AMP+TMP	CPR+STR	CPR+ERY	CPR+TMP	STR+ERY	STR+TMP	ERY+TMP	AMP+CPR+STR	AMP+CPR+ERY	AMP+CPR+TMP	AMP+STR+ERY	AMP+STR+TMP	AMP+ERY+TMP	CPR+STR+ERY	CPR+STR+TMP	CPR+ERY+TMP	STR+ERY+TMP	AMP+CPR+STR+ERY	AMP+CPR+STR+TMP	AMP+CPR+ERY+TMP	AMP+STR+ERY+TMP	CPR+STR+ERY+TMP	AMP+CPR+STR+ERY+TMP
	90.7139524	102.552155	99.50227682	104.2465318	116.1071693	96.68688754	14.0612848	97.1016747	94.69590916	96.11352324	85.94726252	75.10325109	98.8245261	83.23625966	88.99714074	51.22621455	83.16482605	51.22621455	46.33172603	46.97464613	79.43174159	63.58148893	38.5258922	82.55850895	88.31939002	52.30466117	44.98366776	70.14050915	15.80339089	34.28995023	61.18110644
	82.47872573	110.7345217	102.1241365	119.344907	104.4423171	87.71547022	20.27056513	78.98756273	106.5677504	70.78730175	77.28648677	95.8319319	95.16959458	77.28648677	95.50076324	59.61160812	86.66812132	84.92253982	46.34518874	44.25049094	80.73314423	78.94233008	36.34576078	77.28648677	86.55920933	60.48439887	70.95788785	57.86602662	25.57276893	-5.050322113	38.8391883
	54.70527763	98.65841073	84.12796698	93.374613	98.65841073	73.50097605	35.53994894	62.44903145	59.78769362	50.25799794	90.73271414	102.621259	80.8255934	97.3374613	113.1888545	48.36611877	80.30217273	84.73773578	42.04544142	47.01696834	84.62684671	78.51393189	60.49535604	112.1981424	111.2074303	66.40407517	75.86660968	56.64583646	31.10438589	17.42002064	42.04544142
AMP3CPR1STR1ERY2TMP2	AMP	CPR	STR	ERY	TMP	AMP+CPR	AMP+STR	AMP+ERY	AMP+TMP	CPR+STR	CPR+ERY	CPR+TMP	STR+ERY	STR+TMP	ERY+TMP	AMP+CPR+STR	AMP+CPR+ERY	AMP+CPR+TMP	AMP+STR+ERY	AMP+STR+TMP	AMP+ERY+TMP	CPR+STR+ERY	CPR+STR+TMP	CPR+ERY+TMP	STR+ERY+TMP	AMP+CPR+STR+ERY	AMP+CPR+STR+TMP	AMP+CPR+ERY+TMP	AMP+STR+ERY+TMP	CPR+STR+ERY+TMP	AMP+CPR+STR+ERY+TMP
	90.7139524	102.552155	99.50227682	104.2465318	100.8577782	96.68688754	14.0612848	97.1016747	94.11520713	96.11352324	85.94726252	74.42550037	98.8245261	65.27586572	96.79127396	51.22621455	83.16482605	71.21895577	46.33172603	51.30917198	84.49214497	63.58148893	43.77846024	76.45875252	88.31939002	52.30466117	33.9710686	73.45880645	33.39036657	43.43958488	77.10893348
	82.47872573	110.7345217	102.1241365	119.344907	83.90986003	87.71547022	20.27056513	78.98756273	100.1090988	70.78730175	77.28648677	71.32545083	95.16959458	69.00727019	104.7734858	59.61160812	86.66812132	68.51407375	46.34518874	44.25049094	89.63560986	78.94233008	25.54138315	82.58518538	90.20206463	60.48439887	53.85118918	66.9430504	22.25616408	-2.607953222	49.66179358
	54.70527763	98.65841073	84.12796698	93.374613	100.6398349	73.50097605	35.53994894	62.44903145	79.00846684	50.25799794	90.73271414	105.5933953	80.8255934	87.76057792	111.8679051	48.36611877	80.30217273	67.88259619	42.04544142	25.19030183	88.17529715	78.51393189	0.722394221	121.1145511	103.6119711	66.40407517	51.3231608	72.90956765	39.92006746	26.66666667	39.19928846
AMP3CPR1STR1ERY2TMP3	AMP	CPR	STR	ERY	TMP	AMP+CPR	AMP+STR	AMP+ERY	AMP+TMP	CPR+STR	CPR+ERY	CPR+TMP	STR+ERY	STR+TMP	ERY+TMP	AMP+CPR+STR	AMP+CPR+ERY	AMP+CPR+TMP	AMP+STR+ERY	AMP+STR+TMP	AMP+ERY+TMP	CPR+STR+ERY	CPR+STR+TMP	CPR+ERY+TMP	STR+ERY+TMP	AMP+CPR+STR+ERY	AMP+CPR+STR+TMP	AMP+CPR+ERY+TMP	AMP+STR+ERY+TMP	CPR+STR+ERY+TMP	AMP+CPR+STR+ERY+TMP
	90.7139524	102.552155	99.50227682	104.2465318	81.88075823	96.68688754	14.0612848	97.1016747	101.0836315	96.11352324	85.94726252	76.11987716	98.8245261	74.42550037	100.1800275	51.22621455	83.16482605	68.56431793	46.33172603	44.34074765	78.51920983	63.58148893	9.319072329	81.20300752	89.3360161	52.30466117	32.97557941	66.49038212	21.11266656	18.70168379	101.6643335
	82.47872573	110.7345217	102.1241365	119.344907	64.70207757	87.71547022	20.27056513	78.98756273	97.66528475	70.78730175	77.28648677	87.55271532	95.16959458	68.34493286	113.7150397	59.61160812	86.66812132	71.65612045	46.34518874	41.80667685	90.50840061	78.94233008	-6.250808517	100.1371245	109.4098471	60.48439887	60.83351516	62.75365481	22.95439668	6.00243202	89.46105171
	54.70527763	98.65841073	84.12796698	93.374613	105.9236326	73.50097605	35.53994894	62.44903145	82.26121308	50.25799794	90.73271414	89.74200206	80.8255934	91.0629515	113.8493292	48.36611877	80.30217273	61.67280792	42.04544142	38.73725064	78.52794751	78.51393189	2.373581011	124.747162	117.4819401	66.40407517	48.8466381	72.61386344	47.07241288	13.45717234	69.65682141
AMP3CPR1STR1ERY3TMP1	AMP	CPR	STR	ERY	TMP	AMP+CPR	AMP+STR	AMP+ERY	AMP+TMP	CPR+STR	CPR+ERY	CPR+TMP	STR+ERY	STR+TMP	ERY+TMP	AMP+CPR+STR	AMP+CPR+ERY	AMP+CPR+TMP	AMP+STR+ERY	AMP+STR+TMP	AMP+ERY+TMP	CPR+STR+ERY	CPR+STR+TMP	CPR+ERY+TMP	STR+ERY+TMP	AMP+CPR+STR+ERY	AMP+CPR+STR+TMP	AMP+CPR+ERY+TMP	AMP+STR+ERY+TMP	CPR+STR+ERY+TMP	AMP+CPR+STR+ERY+TMP
	90.7139524	102.552155	99.50227682	111.7017897	116.1071693	96.68688754	14.0612848	111.3703531	94.69590916	96.11352324	76.45875252	75.10325109	75.10325109	83.23625966	100.1800275	51.22621455	86.81495308	51.22621455	60.60040442	46.97464613	72.13148753	65.61474108	38.5258922	86.28613788	92.38589431	65.24602064	44.98366776	67.15404158	35.38134495	69.19411204	87.06382537
	82.47872573	110.7345217	102.1241365	116.0332204	104.4423171	87.71547022	20.27056513	86.84267947	106.5677504	70.78730175	83.2475227	95.8319319	85.56570335	77.28648677	121.9942563	59.61160812	84.57342352	84.92253982	66.5939341	44.25049094	98.1889592	74.96830612	36.34576078	104.7734858	111.0656904	55.94588697	70.95788785	65.19746891	22.36526293	2.359576725	89.81016801
	54.70527763	98.65841073	84.12796698	92.05366357	98.65841073	73.50097605	35.53994894	87.87959294	59.78769362	50.25799794	94.69556244	102.621259	78.18369453	97.3374613	119.4633643	48.36611877	72.31815924	84.73773578	37.90558257	47.01696834	85.03343999	69.59752322	60.49535604	118.8028896	115.8307534	60.48999111	75.86660968	76.16231389	12.29020595	9.824561404	79.11935592
AMP3CPR1STR1ERY3TMP2	AMP	CPR	STR	ERY	TMP	AMP+CPR	AMP+STR	AMP+ERY	AMP+TMP	CPR+STR	CPR+ERY	CPR+TMP	STR+ERY	STR+TMP	ERY+TMP	AMP+CPR+STR	AMP+CPR+ERY	AMP+CPR+TMP	AMP+STR+ERY	AMP+STR+TMP	AMP+ERY+TMP	CPR+STR+ERY	CPR+STR+TMP	CPR+ERY+TMP	STR+ERY+TMP	AMP+CPR+STR+ERY	AMP+CPR+STR+TMP	AMP+CPR+ERY+TMP	AMP+STR+ERY+TMP	CPR+STR+ERY+TMP	AMP+CPR+STR+ERY+TMP
	90.7139524	102.552155	99.50227682	111.7017897	100.8577782	96.68688754	14.0612848	111.3703531	94.11520713	96.11352324	76.45875252	74.42550037	75.10325109	65.27586572	89.3360161	51.22621455	86.81495308	71.21895577	60.60040442	51.30917198	70.55529631	65.61474108	43.77846024	87.98051467	85.94726252	65.24602064	33.9710686	52.55353347	19.12168818	57.33347453	77.44076321
	82.47872573	110.7345217	102.1241365	116.0332204	83.90986003	87.71547022	20.27056513	86.84267947	100.1090988	70.78730175	83.2475227	71.32545083	85.56570335	69.00727019	93.84491992	59.61160812	84.57342352	68.51407375	66.5939341	44.25049094	96.4433777	74.96830612	25.54138315	93.51375126	101.1306305	55.94588697	53.85118918	72.52891119	25.50730962	-0.413960829	95.7451451
	54.70527763	98.65841073	84.12796698	92.05366357	100.6398349	73.50097605	35.53994894	87.87959294	79.00846684	50.25799794	94.69556244	105.5933953	78.18369453	87.76057792	103.6119711	48.36611877	72.31815924	67.88259619	37.90558257	25.19030183	84.44203158	69.59752322	0.722394221	116.1609907	103.9422085	60.48999111	51.3231608	68.7697088	10.10938745	16.69762642	82.96351056
AMP3CPR1STR1ERY3TMP3	AMP	CPR	STR	ERY	TMP	AMP+CPR	AMP+STR	AMP+ERY	AMP+TMP	CPR+STR	CPR+ERY	CPR+TMP	STR+ERY	STR+TMP	ERY+TMP	AMP+CPR+STR	AMP+CPR+ERY	AMP+CPR+TMP	AMP+STR+ERY	AMP+STR+TMP	AMP+ERY+TMP	CPR+STR+ERY	CPR+STR+TMP	CPR+ERY+TMP	STR+ERY+TMP	AMP+CPR+STR+ERY	AMP+CPR+STR+TMP	AMP+CPR+ERY+TMP	AMP+STR+ERY+TMP	CPR+STR+ERY+TMP	AMP+CPR+STR+ERY+TMP
	90.7139524	102.552155	99.50227682	111.7017897	81.88075823	96.68688754	14.0612848	111.3703531	101.0836315	96.11352324	76.45875252	76.11987716	75.10325109	74.42550037	80.5252568	51.22621455	86.81495308	68.56431793	60.60040442	44.34074765	62.84025509	65.61474108	9.319072329	82.21963359	65.91125702	65.24602064	32.97557941	66.15855239	20.4490071	4.235941968	76.44527402
	82.47872573	110.7345217	102.1241365	116.0332204	64.70207757	87.71547022	20.27056513	86.84267947	97.66528475	70.78730175	83.2475227	87.55271532	85.56570335	68.34493286	128.6176296	59.61160812	84.57342352	71.65612045	66.5939341	41.80667685	107.9642156	74.96830612	-6.250808517	80.26700473	83.2475227	55.94588697	60.83351516	84.39886537	46.45428758	10.30762464	67.46672485
	54.70527763	98.65841073	84.12796698	92.05366357	105.9236326	73.50097605	35.53994894	87.87959294	82.26121308	50.25799794	94.69556244	89.74200206	78.18369453	91.0629515	99.97936017	48.36611877	72.31815924	61.67280792	37.90558257	38.73725064	90.35611565	69.59752322	2.373581011	116.1609907	93.70485036	60.48999111	48.8466381	54.57590704	13.06642949	6.852425181	85.9205526
AMP3CPR1STR2ERY1TMP1	AMP	CPR	STR	ERY	TMP	AMP+CPR	AMP+STR	AMP+ERY	AMP+TMP	CPR+STR	CPR+ERY	CPR+TMP	STR+ERY	STR+TMP	ERY+TMP	AMP+CPR+STR	AMP+CPR+ERY	AMP+CPR+TMP	AMP+STR+ERY	AMP+STR+TMP	AMP+ERY+TMP	CPR+STR+ERY	CPR+STR+TMP	CPR+ERY+TMP	STR+ERY+TMP	AMP+CPR+STR+ERY	AMP+CPR+STR+TMP	AMP+CPR+ERY+TMP	AMP+STR+ERY+TMP	CPR+STR+ERY+TMP	AMP+CPR+STR+ERY+TMP
	90.7139524	102.552155	68.10886253	101.535529	116.1071693	96.68688754	11.07481723	82.41820916	94.69590916	62.24716721	107.29641	75.10325109	45.93209327	60.10058474	91.03039288	8.088349666	81.42271997	51.22621455	46.31098667	41.35428009	73.45880645	56.12623107	35.13713862	85.60838717	83.20138605	30.07206927	29.38767045	66.82221185	44.65183803	56.48628614	21.0919272
	82.47872573	110.7345217	78.75106564	110.4033531	104.4423171	87.71547022	58.17150338	86.31900502	106.5677504	73.10548239	107.4228351	95.8319319	78.1543052	102.0247229	89.87089597	8.749727253	97.1416103	84.92253982	54.68034039	53.98210779	102.0292385	75.7548317	70.24915267	78.27999276	110.8908781	30.04582151	14.53196596	68.51407375	48.396247	57.20938656	39.31922322
	54.70527763	98.65841073	67.2311001	91.0629515	98.65841073	73.50097605	25.78171023	53.09738602	59.78769362	51.24871001	86.76986584	102.621259	81.6486538	77.81558816	112.5283798	25.72626569	77.93653911	84.73773578	24.24774468	50.56541878	69.36111721	56.71826625	57.64705882	97.66769866	75.02790406	12.29020595	44.94703892	57.53294907	29.33016067	71.39318885	27.20478671
AMP3CPR1STR2ERY1TMP2	AMP	CPR	STR	ERY	TMP	AMP+CPR	AMP+STR	AMP+ERY	AMP+TMP	CPR+STR	CPR+ERY	CPR+TMP	STR+ERY	STR+TMP	ERY+TMP	AMP+CPR+STR	AMP+CPR+ERY	AMP+CPR+TMP	AMP+STR+ERY	AMP+STR+TMP	AMP+ERY+TMP	CPR+STR+ERY	CPR+STR+TMP	CPR+ERY+TMP	STR+ERY+TMP	AMP+CPR+STR+ERY	AMP+CPR+STR+TMP	AMP+CPR+ERY+TMP	AMP+STR+ERY+TMP	CPR+STR+ERY+TMP	AMP+CPR+STR+ERY+TMP
	90.7139524	102.552155	68.10886253	101.535529	100.8577782	96.68688754	11.07481723	82.41820916	94.11520713	62.24716721	107.29641	74.42550037	45.93209327	64.662993	92.04701896	8.088349666	81.42271997	71.21895577	46.31098667	40.66988127	67.90065847	56.12623107	51.06428042	78.1531293	86.89751426	30.07206927	38.01524343	69.14501996	24.76279359	44.62564863	28.39218126
	82.47872573	110.7345217	78.75106564	110.4033531	83.90986003	87.71547022	58.17150338	86.31900502	100.1090988	73.10548239	107.4228351	71.32545083	78.1543052	97.25063939	104.1111485	8.749727253	97.1416103	68.51407375	54.68034039	45.25420031	85.09709797	75.7548317	46.61198934	79.93583607	109.8678602	30.04582151	30.59131573	60.83351516	32.38053677	36.13878037	4.40759328
	54.70527763	98.65841073	67.2311001	91.0629515	100.6398349	73.50097605	25.78171023	53.09738602	79.00846684	51.24871001	86.76986584	105.5933953	81.6486538	86.74488879	107.244582	25.72626569	77.93653911	67.88259619	24.24774468	50.32515911	67.77170711	56.71826625	74.36532508	101.9607843	191.2830425	12.29020595	34.00598339	42.4520347	41.10288427	70.07223942	9.517979047
AMP3CPR1STR2ERY1TMP3	AMP	CPR	STR	ERY	TMP	AMP+CPR	AMP+STR	AMP+ERY	AMP+TMP	CPR+STR	CPR+ERY	CPR+TMP	STR+ERY	STR+TMP	ERY+TMP	AMP+CPR+STR	AMP+CPR+ERY	AMP+CPR+TMP	AMP+STR+ERY	AMP+STR+TMP	AMP+ERY+TMP	CPR+STR+ERY	CPR+STR+TMP	CPR+ERY+TMP	STR+ERY+TMP	AMP+CPR+STR+ERY	AMP+CPR+STR+TMP	AMP+CPR+ERY+TMP	AMP+STR+ERY+TMP	CPR+STR+ERY+TMP	AMP+CPR+STR+ERY+TMP
	90.7139524	102.552155	68.10886253	101.535529	81.88075823	96.68688754	11.07481723	82.41820916	101.0836315	62.24716721	107.29641	76.11987716	45.93209327	78.2154631	100.8577782	8.088349666	81.42271997	68.56431793	46.31098667	35.71317468	81.7545497	56.12623107	60.55279043	94.41914646	74.26907621	30.07206927	15.13973143	79.09991186	29.74023954	39.88139362	46.6428164
	82.47872573	110.7345217	78.75106564	110.4033531	64.70207757	87.71547022	58.17150338	86.31900502	97.66528475	73.10548239	107.4228351	87.55271532	78.1543052	89.06649616	102.7864738	8.749727253	97.1416103	71.65612045	54.68034039	37.61728126	80.03491163	75.7548317	42.63796538	94.17608859	114.3009378	30.04582151	33.03512983	70.25965525	35.47894392	67.14444646	33.88610081
	54.70527763	98.65841073	67.2311001	91.0629515	105.9236326	73.50097605	25.78171023	53.09738602	82.26121308	51.24871001	86.76986584	89.74200206	81.6486538	93.71409904	113.5190918	25.72626569	77.93653911	61.67280792	24.24774468	34.06142793	67.47600291	56.71826625	73.374613	108.5655315	106.3893502	12.29020595	19.27621776	53.39309022	31.9914985	73.374613	24.24774468
AMP3CPR1STR2ERY2TMP1	AMP	CPR	STR	ERY	TMP	AMP+CPR	AMP+STR	AMP+ERY	AMP+TMP	CPR+STR	CPR+ERY	CPR+TMP	STR+ERY	STR+TMP	ERY+TMP	AMP+CPR+STR	AMP+CPR+ERY	AMP+CPR+TMP	AMP+STR+ERY	AMP+STR+TMP	AMP+ERY+TMP	CPR+STR+ERY	CPR+STR+TMP	CPR+ERY+TMP	STR+ERY+TMP	AMP+CPR+STR+ERY	AMP+CPR+STR+TMP	AMP+CPR+ERY+TMP	AMP+STR+ERY+TMP	CPR+STR+ERY+TMP	AMP+CPR+STR+ERY+TMP
	90.7139524	102.552155	68.10886253	104.2465318	116.1071693	96.68688754	11.07481723	97.1016747	94.69590916	62.24716721	85.94726252	75.10325109	46.49036263	60.10058474	88.99714074	8.088349666	83.16482605	51.22621455	52.61575154	41.35428009	79.43174159	54.43185428	35.13713862	82.55850895	86.89751426	29.74023954	29.38767045	70.14050915	35.04951522	66.99142222	18.70690102
	82.47872573	110.7345217	78.75106564	119.344907	104.4423171	87.71547022	58.17150338	78.98756273	106.5677504	73.10548239	77.28648677	95.8319319	74.31798806	102.0247229	95.50076324	8.749727253	86.66812132	84.92253982	38.31551386	53.98210779	80.73314423	62.38389692	70.24915267	77.28648677	103.0477408	26.44555968	14.53196596	57.86602662	29.58760637	69.4626271	5.673139865
	54.70527763	98.65841073	67.2311001	93.374613	98.65841073	73.50097605	25.78171023	62.44903145	59.78769362	51.24871001	90.73271414	102.621259	72.10954727	77.81558816	113.1888545	25.72626569	80.30217273	84.73773578	20.10788583	50.56541878	84.62684671	55.06707946	57.64705882	112.1981424	96.28399532	35.48450441	44.94703892	56.64583646	47.60837675	53.89060888	22.23325979
AMP3CPR1STR2ERY2TMP2	AMP	CPR	STR	ERY	TMP	AMP+CPR	AMP+STR	AMP+ERY	AMP+TMP	CPR+STR	CPR+ERY	CPR+TMP	STR+ERY	STR+TMP	ERY+TMP	AMP+CPR+STR	AMP+CPR+ERY	AMP+CPR+TMP	AMP+STR+ERY	AMP+STR+TMP	AMP+ERY+TMP	CPR+STR+ERY	CPR+STR+TMP	CPR+ERY+TMP	STR+ERY+TMP	AMP+CPR+STR+ERY	AMP+CPR+STR+TMP	AMP+CPR+ERY+TMP	AMP+STR+ERY+TMP	CPR+STR+ERY+TMP	AMP+CPR+STR+ERY+TMP
	90.7139524	102.552155	68.10886253	104.2465318	100.8577782	96.68688754	11.07481723	97.1016747	94.11520713	62.24716721	85.94726252	74.42550037	46.49036263	64.662993	96.79127396	8.088349666	83.16482605	71.21895577	52.61575154	40.66988127	84.49214497	54.43185428	51.06428042	76.45875252	87.51353563	29.74023954	38.01524343	73.45880645	40.33805154	57.50291221	41.66537046
	82.47872573	110.7345217	78.75106564	119.344907	83.90986003	87.71547022	58.17150338	78.98756273	100.1090988	73.10548239	77.28648677	71.32545083	74.31798806	97.25063939	104.7734858	8.749727253	86.66812132	68.51407375	38.31551386	45.25420031	89.63560986	62.38389692	46.61198934	82.58518538	87.02046036	26.44555968	30.59131573	66.9430504	29.93672267	72.77431373	46.45428758
	54.70527763	98.65841073	67.2311001	93.374613	100.6398349	73.50097605	25.78171023	62.44903145	79.00846684	51.24871001	90.73271414	105.5933953	72.10954727	86.74488879	111.8679051	25.72626569	80.30217273	67.88259619	20.10788583	50.32515911	88.17529715	55.06707946	74.36532508	121.1145511	68.40715433	35.48450441	34.00598339	72.90956765	44.94703892	70.07223942	32.39809178
AMP3CPR1STR2ERY2TMP3	AMP	CPR	STR	ERY	TMP	AMP+CPR	AMP+STR	AMP+ERY	AMP+TMP	CPR+STR	CPR+ERY	CPR+TMP	STR+ERY	STR+TMP	ERY+TMP	AMP+CPR+STR	AMP+CPR+ERY	AMP+CPR+TMP	AMP+STR+ERY	AMP+STR+TMP	AMP+ERY+TMP	CPR+STR+ERY	CPR+STR+TMP	CPR+ERY+TMP	STR+ERY+TMP	AMP+CPR+STR+ERY	AMP+CPR+STR+TMP	AMP+CPR+ERY+TMP	AMP+STR+ERY+TMP	CPR+STR+ERY+TMP	AMP+CPR+STR+ERY+TMP
	90.7139524	102.552155	68.10886253	104.2465318	81.88075823	96.68688754	11.07481723	97.1016747	101.0836315	62.24716721	85.94726252	76.11987716	46.49036263	78.2154631	100.1800275	8.088349666	83.16482605	68.56431793	52.61575154	35.71317468	78.51920983	54.43185428	60.55279043	81.20300752	87.20552494	29.74023954	15.13973143	66.49038212	52.61575154	59.197289	6.491419091
	82.47872573	110.7345217	78.75106564	119.344907	64.70207757	87.71547022	58.17150338	78.98756273	97.66528475	73.10548239	77.28648677	87.55271532	74.31798806	89.06649616	113.7150397	8.749727253	86.66812132	71.65612045	38.31551386	37.61728126	90.50840061	62.38389692	42.63796538	100.1371245	110.5498721	26.44555968	33.03512983	62.75365481	27.60200742	69.13145844	59.37159066
	54.70527763	98.65841073	67.2311001	93.374613	105.9236326	73.50097605	25.78171023	62.44903145	82.26121308	51.24871001	90.73271414	89.74200206	72.10954727	93.71409904	113.8493292	25.72626569	80.30217273	61.67280792	20.10788583	34.06142793	78.52794751	55.06707946	73.374613	124.747162	98.02629788	35.48450441	19.27621776	72.61386344	50.56541878	68.75128999	36.42706155
AMP3CPR1STR2ERY3TMP1	AMP	CPR	STR	ERY	TMP	AMP+CPR	AMP+STR	AMP+ERY	AMP+TMP	CPR+STR	CPR+ERY	CPR+TMP	STR+ERY	STR+TMP	ERY+TMP	AMP+CPR+STR	AMP+CPR+ERY	AMP+CPR+TMP	AMP+STR+ERY	AMP+STR+TMP	AMP+ERY+TMP	CPR+STR+ERY	CPR+STR+TMP	CPR+ERY+TMP	STR+ERY+TMP	AMP+CPR+STR+ERY	AMP+CPR+STR+TMP	AMP+CPR+ERY+TMP	AMP+STR+ERY+TMP	CPR+STR+ERY+TMP	AMP+CPR+STR+ERY+TMP
	90.7139524	102.552155	68.10886253	111.7017897	116.1071693	96.68688754	11.07481723	111.3703531	94.69590916	62.24716721	76.45875252	75.10325109	50.24424285	60.10058474	100.1800275	8.088349666	86.81495308	51.22621455	49.5670659	41.35428009	72.13148753	66.63136715	35.13713862	86.28613788	69.28315326	29.07658008	29.38767045	67.15404158	41.60315238	64.61929472	13.70145694
	82.47872573	110.7345217	78.75106564	116.0332204	104.4423171	87.71547022	58.17150338	86.84267947	106.5677504	73.10548239	83.2475227	95.8319319	75	102.0247229	121.9942563	8.749727253	84.57342352	84.92253982	45.99607244	53.98210779	98.1889592	60.06571628	70.24915267	104.7734858	105.4347826	20.16146629	14.53196596	65.19746891	44.01047349	73.43665106	18.17586734
	54.70527763	98.65841073	67.2311001	92.05366357	98.65841073	73.50097605	25.78171023	87.87959294	59.78769362	51.24871001	94.69556244	102.621259	75.94261291	77.81558816	119.4633643	25.72626569	72.31815924	84.73773578	17.446548	50.56541878	85.03343999	66.29514964	57.64705882	118.8028896	80.95173277	39.19928846	44.94703892	76.16231389	64.03844155	78.32817337	48.49584936
AMP3CPR1STR2ERY3TMP2	AMP	CPR	STR	ERY	TMP	AMP+CPR	AMP+STR	AMP+ERY	AMP+TMP	CPR+STR	CPR+ERY	CPR+TMP	STR+ERY	STR+TMP	ERY+TMP	AMP+CPR+STR	AMP+CPR+ERY	AMP+CPR+TMP	AMP+STR+ERY	AMP+STR+TMP	AMP+ERY+TMP	CPR+STR+ERY	CPR+STR+TMP	CPR+ERY+TMP	STR+ERY+TMP	AMP+CPR+STR+ERY	AMP+CPR+STR+TMP	AMP+CPR+ERY+TMP	AMP+STR+ERY+TMP	CPR+STR+ERY+TMP	AMP+CPR+STR+ERY+TMP
	90.7139524	102.552155	68.10886253	111.7017897	100.8577782	96.68688754	11.07481723	111.3703531	94.11520713	62.24716721	76.45875252	74.42550037	50.24424285	64.662993	89.3360161	8.088349666	86.81495308	71.21895577	49.5670659	40.66988127	70.55529631	66.63136715	51.06428042	87.98051467	81.29556994	29.07658008	38.01524343	52.55353347	21.77632602	55.80853542	62.25955307
	82.47872573	110.7345217	78.75106564	116.0332204	83.90986003	87.71547022	58.17150338	86.84267947	100.1090988	73.10548239	83.2475227	71.32545083	75	97.25063939	93.84491992	8.749727253	84.57342352	68.51407375	45.99607244	45.25420031	96.4433777	60.06571628	46.61198934	93.51375126	79.85933504	20.16146629	30.59131573	72.52891119	30.04582151	54.22886859	8.11695396
	54.70527763	98.65841073	67.2311001	92.05366357	100.6398349	73.50097605	25.78171023	87.87959294	79.00846684	51.24871001	94.69556244	105.5933953	75.94261291	86.74488879	103.6119711	25.72626569	72.31815924	67.88259619	17.446548	50.32515911	84.44203158	66.29514964	74.36532508	116.1609907	76.63953393	39.19928846	34.00598339	68.7697088	41.39858847	77.13106295	19.38710683
AMP3CPR1STR2ERY3TMP3	AMP	CPR	STR	ERY	TMP	AMP+CPR	AMP+STR	AMP+ERY	AMP+TMP	CPR+STR	CPR+ERY	CPR+TMP	STR+ERY	STR+TMP	ERY+TMP	AMP+CPR+STR	AMP+CPR+ERY	AMP+CPR+TMP	AMP+STR+ERY	AMP+STR+TMP	AMP+ERY+TMP	CPR+STR+ERY	CPR+STR+TMP	CPR+ERY+TMP	STR+ERY+TMP	AMP+CPR+STR+ERY	AMP+CPR+STR+TMP	AMP+CPR+ERY+TMP	AMP+STR+ERY+TMP	CPR+STR+ERY+TMP	AMP+CPR+STR+ERY+TMP
	90.7139524	102.552155	68.10886253	111.7017897	81.88075823	96.68688754	11.07481723	111.3703531	101.0836315	62.24716721	76.45875252	76.11987716	50.24424285	78.2154631	80.5252568	8.088349666	86.81495308	68.56431793	49.5670659	35.71317468	62.84025509	66.63136715	60.55279043	82.21963359	73.96106552	29.07658008	15.13973143	66.15855239	27.39669207	75.1244308	35.96204697
	82.47872573	110.7345217	78.75106564	116.0332204	64.70207757	87.71547022	58.17150338	86.84267947	97.66528475	73.10548239	83.2475227	87.55271532	75	89.06649616	128.6176296	8.749727253	84.57342352	71.65612045	45.99607244	37.61728126	107.9642156	60.06571628	42.63796538	80.26700473	81.22335891	20.16146629	33.03512983	84.39886537	55.18219507	50.37903288	10.49530875
	54.70527763	98.65841073	67.2311001	92.05366357	105.9236326	73.50097605	25.78171023	87.87959294	82.26121308	51.24871001	94.69556244	89.74200206	75.94261291	93.71409904	99.97936017	25.72626569	72.31815924	61.67280792	17.446548	34.06142793	90.35611565	66.29514964	73.374613	116.1609907	82.69403534	39.19928846	19.27621776	54.57590704	36.96302542	62.47678019	15.97192196
AMP3CPR1STR3ERY1TMP1	AMP	CPR	STR	ERY	TMP	AMP+CPR	AMP+STR	AMP+ERY	AMP+TMP	CPR+STR	CPR+ERY	CPR+TMP	STR+ERY	STR+TMP	ERY+TMP	AMP+CPR+STR	AMP+CPR+ERY	AMP+CPR+TMP	AMP+STR+ERY	AMP+STR+TMP	AMP+ERY+TMP	CPR+STR+ERY	CPR+STR+TMP	CPR+ERY+TMP	STR+ERY+TMP	AMP+CPR+STR+ERY	AMP+CPR+STR+TMP	AMP+CPR+ERY+TMP	AMP+STR+ERY+TMP	CPR+STR+ERY+TMP	AMP+CPR+STR+ERY+TMP
	90.7139524	102.552155	74.20542334	101.535529	116.1071693	96.68688754	51.55804428	82.41820916	94.69590916	100.8577782	107.29641	75.10325109	97.01871727	108.9645875	91.03039288	44.58961995	81.42271997	51.22621455	51.22621455	74.45429564	73.45880645	88.99714074	55.44848036	85.60838717	109.2964173	40.69062063	45.97915695	66.82221185	64.49940374	38.5258922	47.97013532
	82.47872573	110.7345217	60.83351516	110.4033531	104.4423171	87.71547022	44.77416539	86.31900502	106.5677504	103.1176425	107.4228351	95.8319319	81.43137683	106.9168667	89.87089597	39.8865372	97.1416103	84.92253982	52.80384028	66.07025966	102.0292385	77.28648677	69.00727019	78.27999276	116.6921231	59.43704997	51.53829369	68.51407375	74.79816714	31.7093995	38.14095571
	54.70527763	98.65841073	64.81466508	91.0629515	98.65841073	73.50097605	21.45703626	53.09738602	59.78769362	89.74200206	86.76986584	102.621259	42.93255403	51.80368013	112.5283798	38.60788005	77.93653911	84.73773578	31.51097917	43.81966664	69.36111721	86.10939112	60.68111455	97.66769866	73.79668026	66.69977938	54.87161124	57.53294907	45.11337253	54.55108359	38.60788005
AMP3CPR1STR3ERY1TMP2	AMP	CPR	STR	ERY	TMP	AMP+CPR	AMP+STR	AMP+ERY	AMP+TMP	CPR+STR	CPR+ERY	CPR+TMP	STR+ERY	STR+TMP	ERY+TMP	AMP+CPR+STR	AMP+CPR+ERY	AMP+CPR+TMP	AMP+STR+ERY	AMP+STR+TMP	AMP+ERY+TMP	CPR+STR+ERY	CPR+STR+TMP	CPR+ERY+TMP	STR+ERY+TMP	AMP+CPR+STR+ERY	AMP+CPR+STR+TMP	AMP+CPR+ERY+TMP	AMP+STR+ERY+TMP	CPR+STR+ERY+TMP	AMP+CPR+STR+ERY+TMP
	90.7139524	102.552155	74.20542334	101.535529	100.8577782	96.68688754	51.55804428	82.41820916	94.11520713	100.8577782	107.29641	74.42550037	97.01871727	90.7139524	92.04701896	44.58961995	81.42271997	71.21895577	51.22621455	58.85829834	67.90065847	88.99714074	69.34237001	78.1531293	108.6327578	40.69062063	35.69243532	69.14501996	33.63923887	59.17610929	51.22621455
	82.47872573	110.7345217	60.83351516	110.4033531	83.90986003	87.71547022	44.77416539	86.31900502	100.1090988	103.1176425	107.4228351	71.32545083	81.43137683	104.1239363	104.1111485	39.8865372	97.1416103	68.51407375	52.80384028	66.07025966	85.09709797	77.28648677	45.4942951	79.93583607	116.6921231	59.43704997	41.2830024	60.83351516	71.30700415	45.94965201	49.66179358
	54.70527763	98.65841073	64.81466508	91.0629515	100.6398349	73.50097605	21.45703626	53.09738602	79.00846684	89.74200206	86.76986584	105.5933953	42.93255403	58.12435748	107.244582	38.60788005	77.93653911	67.88259619	31.51097917	41.74973722	67.77170711	86.10939112	88.42105263	101.9607843	80.00646853	66.69977938	46.00048514	42.4520347	50.43604819	88.09081527	26.77971192
AMP3CPR1STR3ERY1TMP3	AMP	CPR	STR	ERY	TMP	AMP+CPR	AMP+STR	AMP+ERY	AMP+TMP	CPR+STR	CPR+ERY	CPR+TMP	STR+ERY	STR+TMP	ERY+TMP	AMP+CPR+STR	AMP+CPR+ERY	AMP+CPR+TMP	AMP+STR+ERY	AMP+STR+TMP	AMP+ERY+TMP	CPR+STR+ERY	CPR+STR+TMP	CPR+ERY+TMP	STR+ERY+TMP	AMP+CPR+STR+ERY	AMP+CPR+STR+TMP	AMP+CPR+ERY+TMP	AMP+STR+ERY+TMP	CPR+STR+ERY+TMP	AMP+CPR+STR+ERY+TMP
	90.7139524	102.552155	74.20542334	101.535529	81.88075823	96.68688754	51.55804428	82.41820916	101.0836315	100.8577782	107.29641	76.11987716	97.01871727	91.4605693	100.8577782	44.58961995	81.42271997	68.56431793	51.22621455	53.21719293	81.7545497	88.99714074	67.30911787	94.41914646	107.9690984	40.69062063	30.6527713	79.09991186	49.5670659	67.98686858	32.37413802
	82.47872573	110.7345217	60.83351516	110.4033531	64.70207757	87.71547022	44.77416539	86.31900502	97.66528475	103.1176425	107.4228351	87.55271532	81.43137683	102.0292385	102.7864738	39.8865372	97.1416103	71.65612045	52.80384028	58.04058477	80.03491163	77.28648677	75.63064345	94.17608859	109.3606808	59.43704997	36.04625791	70.25965525	61.88086406	45.82546377	29.41304822
	54.70527763	98.65841073	64.81466508	91.0629515	105.9236326	73.50097605	21.45703626	53.09738602	82.26121308	89.74200206	86.76986584	89.74200206	42.93255403	70.72874915	113.5190918	38.60788005	77.93653911	61.67280792	31.51097917	47.36811708	67.47600291	86.10939112	79.50464396	108.5655315	75.57090548	66.69977938	47.18330195	53.39309022	54.87161124	77.52321981	22.04844467
AMP3CPR1STR3ERY2TMP1	AMP	CPR	STR	ERY	TMP	AMP+CPR	AMP+STR	AMP+ERY	AMP+TMP	CPR+STR	CPR+ERY	CPR+TMP	STR+ERY	STR+TMP	ERY+TMP	AMP+CPR+STR	AMP+CPR+ERY	AMP+CPR+TMP	AMP+STR+ERY	AMP+STR+TMP	AMP+ERY+TMP	CPR+STR+ERY	CPR+STR+TMP	CPR+ERY+TMP	STR+ERY+TMP	AMP+CPR+STR+ERY	AMP+CPR+STR+TMP	AMP+CPR+ERY+TMP	AMP+STR+ERY+TMP	CPR+STR+ERY+TMP	AMP+CPR+STR+ERY+TMP
	90.7139524	102.552155	74.20542334	104.2465318	116.1071693	96.68688754	51.55804428	97.1016747	94.69590916	100.8577782	85.94726252	75.10325109	97.68237673	108.9645875	88.99714074	44.58961995	83.16482605	51.22621455	68.56431793	74.45429564	79.43174159	71.0367468	55.44848036	82.55850895	103.9871416	37.37232333	45.97915695	70.14050915	46.58059833	45.62109499	55.54000104
	82.47872573	110.7345217	60.83351516	119.344907	104.4423171	87.71547022	44.77416539	78.98756273	106.5677504	103.1176425	77.28648677	95.8319319	88.76281911	106.9168667	95.50076324	39.8865372	86.66812132	84.92253982	59.08793367	83.17695832	80.73314423	81.92284805	69.00727019	77.28648677	111.1062623	45.47239799	51.53829369	57.86602662	64.67379446	43.96264004	35.69714161
	54.70527763	98.65841073	64.81466508	93.374613	98.65841073	73.50097605	21.45703626	62.44903145	59.78769362	89.74200206	90.73271414	102.621259	64.81466508	51.80368013	113.1888545	38.60788005	80.30217273	84.73773578	37.90558257	43.81966664	84.62684671	89.74200206	60.68111455	112.1981424	73.09438278	61.37710372	54.87161124	56.64583646	61.96851212	72.23942208	30.32816236
AMP3CPR1STR3ERY2TMP2	AMP	CPR	STR	ERY	TMP	AMP+CPR	AMP+STR	AMP+ERY	AMP+TMP	CPR+STR	CPR+ERY	CPR+TMP	STR+ERY	STR+TMP	ERY+TMP	AMP+CPR+STR	AMP+CPR+ERY	AMP+CPR+TMP	AMP+STR+ERY	AMP+STR+TMP	AMP+ERY+TMP	CPR+STR+ERY	CPR+STR+TMP	CPR+ERY+TMP	STR+ERY+TMP	AMP+CPR+STR+ERY	AMP+CPR+STR+TMP	AMP+CPR+ERY+TMP	AMP+STR+ERY+TMP	CPR+STR+ERY+TMP	AMP+CPR+STR+ERY+TMP
	90.7139524	102.552155	74.20542334	104.2465318	100.8577782	96.68688754	51.55804428	97.1016747	94.11520713	100.8577782	85.94726252	74.42550037	97.68237673	90.7139524	96.79127396	44.58961995	83.16482605	71.21895577	68.56431793	58.85829834	84.49214497	71.0367468	69.34237001	76.45875252	106.6417794	37.37232333	35.69243532	73.45880645	31.98009022	42.59239648	39.28034427
	82.47872573	110.7345217	60.83351516	119.344907	83.90986003	87.71547022	44.77416539	78.98756273	100.1090988	103.1176425	77.28648677	71.32545083	88.76281911	104.1239363	104.7734858	39.8865372	86.66812132	68.51407375	59.08793367	66.07025966	89.63560986	81.92284805	45.4942951	82.58518538	106.5677504	45.47239799	41.2830024	66.9430504	56.99323587	49.26133865	25.22365263
	54.70527763	98.65841073	64.81466508	93.374613	100.6398349	73.50097605	21.45703626	62.44903145	79.00846684	89.74200206	90.73271414	105.5933953	64.81466508	58.12435748	111.8679051	38.60788005	80.30217273	67.88259619	37.90558257	41.74973722	88.17529715	89.74200206	88.42105263	121.1145511	77.93653911	61.37710372	46.00048514	72.90956765	32.58290691	61.67182663	50.14034399
AMP3CPR1STR3ERY2TMP3	AMP	CPR	STR	ERY	TMP	AMP+CPR	AMP+STR	AMP+ERY	AMP+TMP	CPR+STR	CPR+ERY	CPR+TMP	STR+ERY	STR+TMP	ERY+TMP	AMP+CPR+STR	AMP+CPR+ERY	AMP+CPR+TMP	AMP+STR+ERY	AMP+STR+TMP	AMP+ERY+TMP	CPR+STR+ERY	CPR+STR+TMP	CPR+ERY+TMP	STR+ERY+TMP	AMP+CPR+STR+ERY	AMP+CPR+STR+TMP	AMP+CPR+ERY+TMP	AMP+STR+ERY+TMP	CPR+STR+ERY+TMP	AMP+CPR+STR+ERY+TMP
	90.7139524	102.552155	74.20542334	104.2465318	81.88075823	96.68688754	51.55804428	97.1016747	101.0836315	100.8577782	85.94726252	76.11987716	97.68237673	91.4605693	100.1800275	44.58961995	83.16482605	68.56431793	68.56431793	53.21719293	78.51920983	71.0367468	67.30911787	81.20300752	107.6372686	37.37232333	30.6527713	66.49038212	44.25779022	54.11415864	39.67439208
	82.47872573	110.7345217	60.83351516	119.344907	64.70207757	87.71547022	44.77416539	78.98756273	97.66528475	103.1176425	77.28648677	87.55271532	88.76281911	102.0292385	113.7150397	39.8865372	86.66812132	71.65612045	59.08793367	58.04058477	90.50840061	81.92284805	75.63064345	100.1371245	103.0765874	45.47239799	36.04625791	62.75365481	65.37202706	68.0137642	26.96923413
	54.70527763	98.65841073	64.81466508	93.374613	105.9236326	73.50097605	21.45703626	62.44903145	82.26121308	89.74200206	90.73271414	89.74200206	64.81466508	70.72874915	113.8493292	38.60788005	80.30217273	61.67280792	37.90558257	47.36811708	78.52794751	89.74200206	79.50464396	124.747162	88.58189043	61.37710372	47.18330195	72.61386344	64.03844155	70.58823529	53.98449863
AMP3CPR1STR3ERY3TMP1	AMP	CPR	STR	ERY	TMP	AMP+CPR	AMP+STR	AMP+ERY	AMP+TMP	CPR+STR	CPR+ERY	CPR+TMP	STR+ERY	STR+TMP	ERY+TMP	AMP+CPR+STR	AMP+CPR+ERY	AMP+CPR+TMP	AMP+STR+ERY	AMP+STR+TMP	AMP+ERY+TMP	CPR+STR+ERY	CPR+STR+TMP	CPR+ERY+TMP	STR+ERY+TMP	AMP+CPR+STR+ERY	AMP+CPR+STR+TMP	AMP+CPR+ERY+TMP	AMP+STR+ERY+TMP	CPR+STR+ERY+TMP	AMP+CPR+STR+ERY+TMP
	90.7139524	102.552155	74.20542334	111.7017897	116.1071693	96.68688754	51.55804428	111.3703531	94.69590916	100.8577782	76.45875252	75.10325109	92.37310105	108.9645875	100.1800275	44.58961995	86.81495308	51.22621455	69.22797739	74.45429564	72.13148753	75.44212644	55.44848036	86.28613788	100.6688443	39.36330171	45.97915695	67.15404158	29.32545238	49.34872392	46.97464613
	82.47872573	110.7345217	60.83351516	116.0332204	104.4423171	87.71547022	44.77416539	86.84267947	106.5677504	103.1176425	83.2475227	95.8319319	95.0469125	106.9168667	121.9942563	39.8865372	84.57342352	84.92253982	59.96022442	83.17695832	98.1889592	80.5981734	69.00727019	104.7734858	105.8695178	40.2356535	51.53829369	65.19746891	69.21230635	68.67610152	28.49661794
	54.70527763	98.65841073	64.81466508	92.05366357	98.65841073	73.50097605	21.45703626	87.87959294	59.78769362	89.74200206	94.69556244	102.621259	67.47600291	51.80368013	119.4633643	38.60788005	72.31815924	84.73773578	50.32515911	43.81966664	85.03343999	88.42105263	60.68111455	118.8028896	84.14632738	52.80168182	54.87161124	76.16231389	104.8456216	75.54179567	53.68879443
AMP3CPR1STR3ERY3TMP2	AMP	CPR	STR	ERY	TMP	AMP+CPR	AMP+STR	AMP+ERY	AMP+TMP	CPR+STR	CPR+ERY	CPR+TMP	STR+ERY	STR+TMP	ERY+TMP	AMP+CPR+STR	AMP+CPR+ERY	AMP+CPR+TMP	AMP+STR+ERY	AMP+STR+TMP	AMP+ERY+TMP	CPR+STR+ERY	CPR+STR+TMP	CPR+ERY+TMP	STR+ERY+TMP	AMP+CPR+STR+ERY	AMP+CPR+STR+TMP	AMP+CPR+ERY+TMP	AMP+STR+ERY+TMP	CPR+STR+ERY+TMP	AMP+CPR+STR+ERY+TMP
	90.7139524	102.552155	74.20542334	111.7017897	100.8577782	96.68688754	51.55804428	111.3703531	94.11520713	100.8577782	76.45875252	74.42550037	92.37310105	90.7139524	89.3360161	44.58961995	86.81495308	71.21895577	69.22797739	58.85829834	70.55529631	75.44212644	69.34237001	87.98051467	74.45429564	39.36330171	35.69243532	52.55353347	36.62570643	66.29249179	44.25779022
	82.47872573	110.7345217	60.83351516	116.0332204	83.90986003	87.71547022	44.77416539	86.84267947	100.1090988	103.1176425	83.2475227	71.32545083	95.0469125	104.1239363	93.84491992	39.8865372	84.57342352	68.51407375	59.96022442	66.07025966	96.4433777	80.5981734	45.4942951	93.51375126	92.95221471	40.2356535	41.2830024	72.52891119	65.02291076	64.70207757	37.22452542
	54.70527763	98.65841073	64.81466508	92.05366357	100.6398349	73.50097605	21.45703626	87.87959294	79.00846684	89.74200206	94.69556244	105.5933953	67.47600291	58.12435748	103.6119711	38.60788005	72.31815924	67.88259619	50.32515911	41.74973722	84.44203158	88.42105263	88.42105263	116.1609907	85.03343999	52.80168182	46.00048514	68.7697088	57.53294907	80.8255934	31.34464556
AMP3CPR1STR3ERY3TMP3	AMP	CPR	STR	ERY	TMP	AMP+CPR	AMP+STR	AMP+ERY	AMP+TMP	CPR+STR	CPR+ERY	CPR+TMP	STR+ERY	STR+TMP	ERY+TMP	AMP+CPR+STR	AMP+CPR+ERY	AMP+CPR+TMP	AMP+STR+ERY	AMP+STR+TMP	AMP+ERY+TMP	CPR+STR+ERY	CPR+STR+TMP	CPR+ERY+TMP	STR+ERY+TMP	AMP+CPR+STR+ERY	AMP+CPR+STR+TMP	AMP+CPR+ERY+TMP	AMP+STR+ERY+TMP	CPR+STR+ERY+TMP	AMP+CPR+STR+ERY+TMP
	90.7139524	102.552155	74.20542334	111.7017897	81.88075823	96.68688754	51.55804428	111.3703531	101.0836315	100.8577782	76.45875252	76.11987716	92.37310105	91.4605693	80.5252568	44.58961995	86.81495308	68.56431793	69.22797739	53.21719293	62.84025509	75.44212644	67.30911787	82.21963359	91.70944159	39.36330171	30.6527713	66.15855239	32.31191995	39.1824632	33.03779748
	82.47872573	110.7345217	60.83351516	116.0332204	64.70207757	87.71547022	44.77416539	86.84267947	97.66528475	103.1176425	83.2475227	87.55271532	95.0469125	102.0292385	128.6176296	39.8865372	84.57342352	71.65612045	59.96022442	58.04058477	107.9642156	80.5981734	75.63064345	80.26700473	98.1889592	40.2356535	36.04625791	84.39886537	74.09993454	57.08519831	36.52629282
	54.70527763	98.65841073	64.81466508	92.05366357	105.9236326	73.50097605	21.45703626	87.87959294	82.26121308	89.74200206	94.69556244	89.74200206	67.47600291	70.72874915	99.97936017	38.60788005	72.31815924	61.67280792	50.32515911	47.36811708	90.35611565	88.42105263	79.50464396	116.1609907	77.3451307	52.80168182	47.18330195	54.57590704	71.43104663	58.36945304	29.27471614
AMP3CPR2STR1ERY1TMP1	AMP	CPR	STR	ERY	TMP	AMP+CPR	AMP+STR	AMP+ERY	AMP+TMP	CPR+STR	CPR+ERY	CPR+TMP	STR+ERY	STR+TMP	ERY+TMP	AMP+CPR+STR	AMP+CPR+ERY	AMP+CPR+TMP	AMP+STR+ERY	AMP+STR+TMP	AMP+ERY+TMP	CPR+STR+ERY	CPR+STR+TMP	CPR+ERY+TMP	STR+ERY+TMP	AMP+CPR+STR+ERY	AMP+CPR+STR+TMP	AMP+CPR+ERY+TMP	AMP+STR+ERY+TMP	CPR+STR+ERY+TMP	AMP+CPR+STR+ERY+TMP
	90.7139524	79.50525784	99.50227682	101.535529	116.1071693	98.34603619	14.0612848	82.41820916	94.69590916	59.27280603	48.03041606	72.72902279	112.3795404	83.23625966	91.03039288	11.07481723	91.79239903	77.44076321	40.69062063	46.97464613	73.45880645	53.72861371	23.67832134	77.34918305	92.38589431	37.37232333	23.7673044	91.70944159	22.43998548	54.96065645	9.830455747
	82.47872573	81.56436488	102.1241365	110.4033531	104.4423171	102.3783548	20.27056513	86.31900502	106.5677504	77.40835465	73.29497016	108.5038363	132.9228222	77.28648677	89.87089597	18.87409993	93.47588916	81.43137683	36.6790312	44.25049094	102.0292385	69.22421142	43.96845695	108.5038363	78.61116142	35.87169976	27.95112372	95.3960288	31.33318787	65.96334186	25.39821078
	54.70527763	94.54169276	84.12796698	91.0629515	98.65841073	82.66780636	35.53994894	53.09738602	59.78769362	81.21307816	88.61786405	83.95720469	79.50464396	97.3374613	112.5283798	16.91058413	64.22325667	69.25022813	50.91656752	47.01696834	69.36111721	55.51411537	80.51615713	83.39095636	99.31888545	58.90058101	15.72776732	64.33414575	39.08839939	50.28720769	16.61487993
AMP3CPR2STR1ERY1TMP2	AMP	CPR	STR	ERY	TMP	AMP+CPR	AMP+STR	AMP+ERY	AMP+TMP	CPR+STR	CPR+ERY	CPR+TMP	STR+ERY	STR+TMP	ERY+TMP	AMP+CPR+STR	AMP+CPR+ERY	AMP+CPR+TMP	AMP+STR+ERY	AMP+STR+TMP	AMP+ERY+TMP	CPR+STR+ERY	CPR+STR+TMP	CPR+ERY+TMP	STR+ERY+TMP	AMP+CPR+STR+ERY	AMP+CPR+STR+TMP	AMP+CPR+ERY+TMP	AMP+STR+ERY+TMP	CPR+STR+ERY+TMP	AMP+CPR+STR+ERY+TMP
	90.7139524	79.50525784	99.50227682	101.535529	100.8577782	98.34603619	14.0612848	82.41820916	94.11520713	59.27280603	48.03041606	73.34504416	112.3795404	65.27586572	92.04701896	11.07481723	91.79239903	77.52372064	40.69062063	51.30917198	67.90065847	53.72861371	45.10431456	81.96934331	85.60838717	37.37232333	22.10815575	90.7139524	11.4896044	61.42888081	11.82143413
	82.47872573	81.56436488	102.1241365	110.4033531	83.90986003	102.3783548	20.27056513	86.31900502	100.1090988	77.40835465	73.29497016	102.7067349	132.9228222	69.00727019	104.1111485	18.87409993	93.47588916	79.68579533	36.6790312	44.25049094	85.09709797	69.22421142	84.71867008	106.7988065	95.16959458	35.87169976	15.9720707	90.50840061	28.19114117	47.3998295	26.79467598
	54.70527763	94.54169276	84.12796698	91.0629515	100.6398349	82.66780636	35.53994894	53.09738602	79.00846684	81.21307816	88.61786405	90.57795443	79.50464396	87.76057792	107.244582	16.91058413	64.22325667	65.11036928	50.91656752	25.19030183	67.77170711	55.51411537	89.92459097	78.8609697	100.6398349	58.90058101	16.61487993	75.57090548	32.58290691	74.24386791	31.04894136
AMP3CPR2STR1ERY1TMP3	AMP	CPR	STR	ERY	TMP	AMP+CPR	AMP+STR	AMP+ERY	AMP+TMP	CPR+STR	CPR+ERY	CPR+TMP	STR+ERY	STR+TMP	ERY+TMP	AMP+CPR+STR	AMP+CPR+ERY	AMP+CPR+TMP	AMP+STR+ERY	AMP+STR+TMP	AMP+ERY+TMP	CPR+STR+ERY	CPR+STR+TMP	CPR+ERY+TMP	STR+ERY+TMP	AMP+CPR+STR+ERY	AMP+CPR+STR+TMP	AMP+CPR+ERY+TMP	AMP+STR+ERY+TMP	CPR+STR+ERY+TMP	AMP+CPR+STR+ERY+TMP
	90.7139524	79.50525784	99.50227682	101.535529	81.88075823	98.34603619	14.0612848	82.41820916	101.0836315	59.27280603	48.03041606	72.72902279	112.3795404	74.42550037	100.8577782	11.07481723	91.79239903	79.09991186	40.69062063	44.34074765	81.7545497	53.72861371	56.19269918	89.66961041	92.38589431	37.37232333	28.08109089	86.40016591	7.507647638	10.76112328	2.86203142
	82.47872573	81.56436488	102.1241365	110.4033531	64.70207757	102.3783548	20.27056513	86.31900502	97.66528475	77.40835465	73.29497016	98.61466326	132.9228222	68.34493286	102.7864738	18.87409993	93.47588916	79.68579533	36.6790312	41.80667685	80.03491163	69.22421142	77.40835465	110.2088662	92.85141393	35.87169976	14.9247218	78.98756273	30.63495527	21.80306905	20.51058259
	54.70527763	94.54169276	84.12796698	91.0629515	105.9236326	82.66780636	35.53994894	53.09738602	82.26121308	81.21307816	88.61786405	78.73029701	79.50464396	91.0629515	113.5190918	16.91058413	64.22325667	65.51696256	50.91656752	38.73725064	67.47600291	55.51411537	88.53074892	96.28399532	108.8957688	58.90058101	39.08839939	59.01147009	38.79269518	58.65025998	21.45703626
AMP3CPR2STR1ERY2TMP1	AMP	CPR	STR	ERY	TMP	AMP+CPR	AMP+STR	AMP+ERY	AMP+TMP	CPR+STR	CPR+ERY	CPR+TMP	STR+ERY	STR+TMP	ERY+TMP	AMP+CPR+STR	AMP+CPR+ERY	AMP+CPR+TMP	AMP+STR+ERY	AMP+STR+TMP	AMP+ERY+TMP	CPR+STR+ERY	CPR+STR+TMP	CPR+ERY+TMP	STR+ERY+TMP	AMP+CPR+STR+ERY	AMP+CPR+STR+TMP	AMP+CPR+ERY+TMP	AMP+STR+ERY+TMP	CPR+STR+ERY+TMP	AMP+CPR+STR+ERY+TMP
	90.7139524	79.50525784	99.50227682	104.2465318	116.1071693	98.34603619	14.0612848	97.1016747	94.69590916	59.27280603	56.65471521	72.72902279	98.8245261	83.23625966	88.99714074	11.07481723	104.7337585	77.44076321	46.33172603	46.97464613	79.43174159	26.62367351	23.67832134	89.05358905	88.31939002	36.37683414	23.7673044	55.20817131	15.80339089	42.46697307	16.05226318
	82.47872573	81.56436488	102.1241365	119.344907	104.4423171	102.3783548	20.27056513	78.98756273	106.5677504	77.40835465	80.79710145	108.5038363	95.16959458	77.28648677	95.50076324	18.87409993	95.57058695	81.43137683	46.34518874	44.25049094	80.73314423	65.13213981	43.96845695	105.0937766	86.55920933	36.22081606	27.95112372	78.28933013	25.57276893	61.53026428	22.36526293
	54.70527763	94.54169276	84.12796698	93.374613	98.65841073	82.66780636	35.53994894	62.44903145	59.78769362	81.21307816	86.39642828	83.95720469	80.8255934	97.3374613	113.1888545	16.91058413	83.44402989	69.25022813	42.04544142	47.01696834	84.62684671	52.37797076	80.51615713	64.22562818	111.2074303	26.37311864	15.72776732	66.40407517	31.10438589	19.49200991	16.725769
AMP3CPR2STR1ERY2TMP2	AMP	CPR	STR	ERY	TMP	AMP+CPR	AMP+STR	AMP+ERY	AMP+TMP	CPR+STR	CPR+ERY	CPR+TMP	STR+ERY	STR+TMP	ERY+TMP	AMP+CPR+STR	AMP+CPR+ERY	AMP+CPR+TMP	AMP+STR+ERY	AMP+STR+TMP	AMP+ERY+TMP	CPR+STR+ERY	CPR+STR+TMP	CPR+ERY+TMP	STR+ERY+TMP	AMP+CPR+STR+ERY	AMP+CPR+STR+TMP	AMP+CPR+ERY+TMP	AMP+STR+ERY+TMP	CPR+STR+ERY+TMP	AMP+CPR+STR+ERY+TMP
	90.7139524	79.50525784	99.50227682	104.2465318	100.8577782	98.34603619	14.0612848	97.1016747	94.11520713	59.27280603	56.65471521	73.34504416	98.8245261	65.27586572	96.79127396	11.07481723	104.7337585	77.52372064	46.33172603	51.30917198	84.49214497	26.62367351	45.10431456	85.66547152	88.31939002	36.37683414	22.10815575	64.16757401	33.39036657	4.600909594	30.05132991
	82.47872573	81.56436488	102.1241365	119.344907	83.90986003	102.3783548	20.27056513	78.98756273	100.1090988	77.40835465	80.79710145	102.7067349	95.16959458	69.00727019	104.7734858	18.87409993	95.57058695	79.68579533	46.34518874	44.25049094	89.63560986	65.13213981	84.71867008	95.88661552	90.20206463	36.22081606	15.9720707	75.49639974	22.25616408	55.73316283	22.36526293
	54.70527763	94.54169276	84.12796698	93.374613	100.6398349	82.66780636	35.53994894	62.44903145	79.00846684	81.21307816	86.39642828	90.57795443	80.8255934	87.76057792	111.8679051	16.91058413	83.44402989	65.11036928	42.04544142	25.19030183	88.17529715	52.37797076	89.92459097	88.61786405	103.6119711	26.37311864	16.61487993	77.0494265	39.92006746	44.71183949	9.037459716
AMP3CPR2STR1ERY2TMP3	AMP	CPR	STR	ERY	TMP	AMP+CPR	AMP+STR	AMP+ERY	AMP+TMP	CPR+STR	CPR+ERY	CPR+TMP	STR+ERY	STR+TMP	ERY+TMP	AMP+CPR+STR	AMP+CPR+ERY	AMP+CPR+TMP	AMP+STR+ERY	AMP+STR+TMP	AMP+ERY+TMP	CPR+STR+ERY	CPR+STR+TMP	CPR+ERY+TMP	STR+ERY+TMP	AMP+CPR+STR+ERY	AMP+CPR+STR+TMP	AMP+CPR+ERY+TMP	AMP+STR+ERY+TMP	CPR+STR+ERY+TMP	AMP+CPR+STR+ERY+TMP
	90.7139524	79.50525784	99.50227682	104.2465318	81.88075823	98.34603619	14.0612848	97.1016747	101.0836315	59.27280603	56.65471521	72.72902279	98.8245261	74.42550037	100.1800275	11.07481723	104.7337585	79.09991186	46.33172603	44.34074765	78.51920983	26.62367351	56.19269918	102.9140698	89.3360161	36.37683414	28.08109089	59.5219578	21.11266656	41.54294102	0.871053041
	82.47872573	81.56436488	102.1241365	119.344907	64.70207757	102.3783548	20.27056513	78.98756273	97.66528475	77.40835465	80.79710145	98.61466326	95.16959458	68.34493286	113.7150397	18.87409993	95.57058695	79.68579533	46.34518874	41.80667685	90.50840061	65.13213981	77.40835465	96.56862745	109.4098471	36.22081606	14.9247218	81.78049313	22.95439668	59.82523444	21.55793149
	54.70527763	94.54169276	84.12796698	93.374613	105.9236326	82.66780636	35.53994894	62.44903145	82.26121308	81.21307816	86.39642828	78.73029701	80.8255934	91.0629515	113.8493292	16.91058413	83.44402989	65.51696256	42.04544142	38.73725064	78.52794751	52.37797076	88.53074892	73.63406202	117.4819401	26.37311864	39.08839939	80.59787694	47.07241288	74.33098304	3.419079852
AMP3CPR2STR1ERY3TMP1	AMP	CPR	STR	ERY	TMP	AMP+CPR	AMP+STR	AMP+ERY	AMP+TMP	CPR+STR	CPR+ERY	CPR+TMP	STR+ERY	STR+TMP	ERY+TMP	AMP+CPR+STR	AMP+CPR+ERY	AMP+CPR+TMP	AMP+STR+ERY	AMP+STR+TMP	AMP+ERY+TMP	CPR+STR+ERY	CPR+STR+TMP	CPR+ERY+TMP	STR+ERY+TMP	AMP+CPR+STR+ERY	AMP+CPR+STR+TMP	AMP+CPR+ERY+TMP	AMP+STR+ERY+TMP	CPR+STR+ERY+TMP	AMP+CPR+STR+ERY+TMP
	90.7139524	79.50525784	99.50227682	111.7017897	116.1071693	98.34603619	14.0612848	111.3703531	94.69590916	59.27280603	61.64063816	72.72902279	75.10325109	83.23625966	100.1800275	11.07481723	107.3883963	77.44076321	60.60040442	46.97464613	72.13148753	36.30675939	23.67832134	72.4210121	92.38589431	29.07658008	23.7673044	77.19189091	35.38134495	69.26390259	8.15056774
	82.47872573	81.56436488	102.1241365	116.0332204	104.4423171	102.3783548	20.27056513	86.84267947	106.5677504	77.40835465	93.49957374	108.5038363	85.56570335	77.28648677	121.9942563	18.87409993	88.58826096	81.43137683	66.5939341	44.25049094	98.1889592	66.83716965	43.96845695	103.7297528	111.0656904	24.00174558	27.95112372	79.16212088	22.36526293	19.41602728	5.498581715
	54.70527763	94.54169276	84.12796698	92.05366357	98.65841073	82.66780636	35.53994894	87.87959294	59.78769362	81.21307816	112.1825062	83.95720469	78.18369453	97.3374613	119.4633643	16.91058413	89.65381817	69.25022813	37.90558257	47.01696834	85.03343999	66.22927613	80.51615713	85.13325892	115.8307534	17.50199254	15.72776732	72.02245504	12.29020595	13.5681812	31.6957943
AMP3CPR2STR1ERY3TMP2	AMP	CPR	STR	ERY	TMP	AMP+CPR	AMP+STR	AMP+ERY	AMP+TMP	CPR+STR	CPR+ERY	CPR+TMP	STR+ERY	STR+TMP	ERY+TMP	AMP+CPR+STR	AMP+CPR+ERY	AMP+CPR+TMP	AMP+STR+ERY	AMP+STR+TMP	AMP+ERY+TMP	CPR+STR+ERY	CPR+STR+TMP	CPR+ERY+TMP	STR+ERY+TMP	AMP+CPR+STR+ERY	AMP+CPR+STR+TMP	AMP+CPR+ERY+TMP	AMP+STR+ERY+TMP	CPR+STR+ERY+TMP	AMP+CPR+STR+ERY+TMP
	90.7139524	79.50525784	99.50227682	111.7017897	100.8577782	98.34603619	14.0612848	111.3703531	94.11520713	59.27280603	61.64063816	73.34504416	75.10325109	65.27586572	89.3360161	11.07481723	107.3883963	77.52372064	60.60040442	51.30917198	70.55529631	36.30675939	45.10431456	67.80085184	85.94726252	29.07658008	22.10815575	76.86006118	19.12168818	18.46139038	10.14154612
	82.47872573	81.56436488	102.1241365	116.0332204	83.90986003	102.3783548	20.27056513	86.84267947	100.1090988	77.40835465	93.49957374	102.7067349	85.56570335	69.00727019	93.84491992	18.87409993	88.58826096	79.68579533	66.5939341	44.25049094	96.4433777	66.83716965	84.71867008	89.7485081	101.1306305	24.00174558	15.9720707	80.90770238	25.50730962	9.185848252	7.593279511
	54.70527763	94.54169276	84.12796698	92.05366357	100.6398349	82.66780636	35.53994894	87.87959294	79.00846684	81.21307816	112.1825062	90.57795443	78.18369453	87.76057792	103.6119711	16.91058413	89.65381817	65.11036928	37.90558257	25.19030183	84.44203158	66.22927613	89.92459097	87.224022	103.9422085	17.50199254	16.61487993	81.18928534	10.10938745	8.341273514	9.628868123
AMP3CPR2STR1ERY3TMP3	AMP	CPR	STR	ERY	TMP	AMP+CPR	AMP+STR	AMP+ERY	AMP+TMP	CPR+STR	CPR+ERY	CPR+TMP	STR+ERY	STR+TMP	ERY+TMP	AMP+CPR+STR	AMP+CPR+ERY	AMP+CPR+TMP	AMP+STR+ERY	AMP+STR+TMP	AMP+ERY+TMP	CPR+STR+ERY	CPR+STR+TMP	CPR+ERY+TMP	STR+ERY+TMP	AMP+CPR+STR+ERY	AMP+CPR+STR+TMP	AMP+CPR+ERY+TMP	AMP+STR+ERY+TMP	CPR+STR+ERY+TMP	AMP+CPR+STR+ERY+TMP
	90.7139524	79.50525784	99.50227682	111.7017897	81.88075823	98.34603619	14.0612848	111.3703531	101.0836315	59.27280603	61.64063816	72.72902279	75.10325109	74.42550037	80.5252568	11.07481723	107.3883963	79.09991186	60.60040442	44.34074765	62.84025509	36.30675939	56.19269918	82.89337536	65.91125702	29.07658008	28.08109089	71.5507855	20.4490071	73.88406285	7.48690828
	82.47872573	81.56436488	102.1241365	116.0332204	64.70207757	102.3783548	20.27056513	86.84267947	97.66528475	77.40835465	93.49957374	98.61466326	85.56570335	68.34493286	128.6176296	18.87409993	88.58826096	79.68579533	66.5939341	41.80667685	107.9642156	66.83716965	77.40835465	78.1543052	83.2475227	24.00174558	14.9247218	88.23914467	46.45428758	1.683716965	10.14619245
	54.70527763	94.54169276	84.12796698	92.05366357	105.9236326	82.66780636	35.53994894	87.87959294	82.26121308	81.21307816	112.1825062	78.73029701	78.18369453	91.0629515	99.97936017	16.91058413	89.65381817	65.51696256	37.90558257	38.73725064	90.35611565	66.22927613	88.53074892	84.65412572	93.70485036	17.50199254	39.08839939	81.48498955	13.06642949	10.43203659	11.99450175
AMP3CPR2STR2ERY1TMP1	AMP	CPR	STR	ERY	TMP	AMP+CPR	AMP+STR	AMP+ERY	AMP+TMP	CPR+STR	CPR+ERY	CPR+TMP	STR+ERY	STR+TMP	ERY+TMP	AMP+CPR+STR	AMP+CPR+ERY	AMP+CPR+TMP	AMP+STR+ERY	AMP+STR+TMP	AMP+ERY+TMP	CPR+STR+ERY	CPR+STR+TMP	CPR+ERY+TMP	STR+ERY+TMP	AMP+CPR+STR+ERY	AMP+CPR+STR+TMP	AMP+CPR+ERY+TMP	AMP+STR+ERY+TMP	CPR+STR+ERY+TMP	AMP+CPR+STR+ERY+TMP
	90.7139524	79.50525784	68.10886253	101.535529	116.1071693	98.34603619	11.07481723	82.41820916	94.69590916	42.02420771	48.03041606	72.72902279	45.93209327	60.10058474	91.03039288	10.7429875	91.79239903	77.44076321	46.31098667	41.35428009	73.45880645	39.56012224	33.30365522	77.34918305	83.20138605	37.0404936	66.22077047	91.70944159	44.65183803	51.88054961	13.81241251
	82.47872573	81.56436488	78.75106564	110.4033531	104.4423171	102.3783548	58.17150338	86.31900502	106.5677504	62.74509804	73.29497016	108.5038363	78.1543052	102.0247229	89.87089597	10.84442505	93.47588916	81.43137683	54.68034039	53.98210779	102.0292385	54.56095482	71.6112532	108.5038363	110.8908781	51.18917739	53.98210779	95.3960288	48.396247	54.90196078	43.15950251
	54.70527763	94.54169276	67.2311001	91.0629515	98.65841073	82.66780636	25.78171023	53.09738602	59.78769362	53.4233523	88.61786405	83.95720469	81.6486538	77.81558816	112.5283798	39.08839939	64.22325667	69.25022813	24.24774468	50.56541878	69.36111721	68.05869382	75.98617047	83.39095636	75.02790406	45.40907673	52.6353482	64.33414575	29.33016067	56.82084229	27.50049092
AMP3CPR2STR2ERY1TMP2	AMP	CPR	STR	ERY	TMP	AMP+CPR	AMP+STR	AMP+ERY	AMP+TMP	CPR+STR	CPR+ERY	CPR+TMP	STR+ERY	STR+TMP	ERY+TMP	AMP+CPR+STR	AMP+CPR+ERY	AMP+CPR+TMP	AMP+STR+ERY	AMP+STR+TMP	AMP+ERY+TMP	CPR+STR+ERY	CPR+STR+TMP	CPR+ERY+TMP	STR+ERY+TMP	AMP+CPR+STR+ERY	AMP+CPR+STR+TMP	AMP+CPR+ERY+TMP	AMP+STR+ERY+TMP	CPR+STR+ERY+TMP	AMP+CPR+STR+ERY+TMP
	90.7139524	79.50525784	68.10886253	101.535529	100.8577782	98.34603619	11.07481723	82.41820916	94.11520713	42.02420771	48.03041606	73.34504416	45.93209327	64.662993	92.04701896	10.7429875	91.79239903	77.52372064	46.31098667	40.66988127	67.90065847	39.56012224	68.10886253	81.96934331	86.89751426	37.0404936	23.7673044	90.7139524	24.76279359	57.11673124	25.09462332
	82.47872573	81.56436488	78.75106564	110.4033531	83.90986003	102.3783548	58.17150338	86.31900502	100.1090988	62.74509804	73.29497016	102.7067349	78.1543052	97.25063939	104.1111485	10.84442505	93.47588916	79.68579533	54.68034039	45.25420031	85.09709797	54.56095482	63.76811594	106.7988065	109.8678602	51.18917739	24.45996072	90.50840061	32.38053677	44.33077579	25.74732708
	54.70527763	94.54169276	67.2311001	91.0629515	100.6398349	82.66780636	25.78171023	53.09738602	79.00846684	53.4233523	88.61786405	90.57795443	81.6486538	86.74488879	107.244582	39.08839939	64.22325667	65.11036928	24.24774468	50.32515911	67.77170711	68.05869382	76.33463099	78.8609697	191.2830425	45.40907673	42.28570108	75.57090548	41.10288427	71.45618381	30.45753295
AMP3CPR2STR2ERY1TMP3	AMP	CPR	STR	ERY	TMP	AMP+CPR	AMP+STR	AMP+ERY	AMP+TMP	CPR+STR	CPR+ERY	CPR+TMP	STR+ERY	STR+TMP	ERY+TMP	AMP+CPR+STR	AMP+CPR+ERY	AMP+CPR+TMP	AMP+STR+ERY	AMP+STR+TMP	AMP+ERY+TMP	CPR+STR+ERY	CPR+STR+TMP	CPR+ERY+TMP	STR+ERY+TMP	AMP+CPR+STR+ERY	AMP+CPR+STR+TMP	AMP+CPR+ERY+TMP	AMP+STR+ERY+TMP	CPR+STR+ERY+TMP	AMP+CPR+STR+ERY+TMP
	90.7139524	79.50525784	68.10886253	101.535529	81.88075823	98.34603619	11.07481723	82.41820916	101.0836315	42.02420771	48.03041606	72.72902279	45.93209327	78.2154631	100.8577782	10.7429875	91.79239903	79.09991186	46.31098667	35.71317468	81.7545497	39.56012224	41.10017566	89.66961041	74.26907621	37.0404936	17.1307098	86.40016591	29.74023954	48.1844214	6.82324882
	82.47872573	81.56436488	78.75106564	110.4033531	64.70207757	102.3783548	58.17150338	86.31900502	97.66528475	62.74509804	73.29497016	98.61466326	78.1543052	89.06649616	102.7864738	10.84442505	93.47588916	79.68579533	54.68034039	37.61728126	80.03491163	54.56095482	55.24296675	110.2088662	114.3009378	51.18917739	22.36526293	78.98756273	35.47894392	42.62574595	29.93672267
	54.70527763	94.54169276	67.2311001	91.0629515	105.9236326	82.66780636	25.78171023	53.09738602	82.26121308	53.4233523	88.61786405	78.73029701	81.6486538	93.71409904	113.5190918	39.08839939	64.22325667	65.51696256	24.24774468	34.06142793	67.47600291	68.05869382	63.09313152	96.28399532	106.3893502	45.40907673	21.05044298	59.01147009	31.9914985	57.86622383	35.35513382

AMP3CPR2STR2ERY2TMP1	AMP	CPR	STR	ERY	TMP	AMP+CPR	AMP+STR	AMP+ERY	AMP+TMP	CPR+STR	CPR+ERY	CPR+TMP	STR+ERY	STR+TMP	ERY+TMP	AMP+CPR+STR	AMP+CPR+ERY	AMP+CPR+TMP	AMP+STR+ERY	AMP+STR+TMP	AMP+ERY+TMP	CPR+STR+ERY	CPR+STR+TMP	CPR+ERY+TMP	STR+ERY+TMP	AMP+CPR+STR+ERY	AMP+CPR+STR+TMP	AMP+CPR+ERY+TMP	AMP+STR+ERY+TMP	CPR+STR+ERY+TMP	AMP+CPR+STR+ERY+TMP
	90.7139524	79.50525784	68.10886253	104.2465318	116.1071693	98.34603619	11.07481723	97.1016747	94.69590916	42.02420771	56.65471521	72.72902279	46.49036263	60.10058474	88.99714074	10.7429875	104.7337585	77.44076321	52.61575154	41.35428009	79.43174159	38.84784753	33.30365522	89.05358905	86.89751426	38.36781252	66.22077047	55.20817131	35.04951522	60.81285945	28.08109089
	82.47872573	81.56436488	78.75106564	119.344907	104.4423171	102.3783548	58.17150338	78.98756273	106.5677504	62.74509804	80.79710145	108.5038363	74.31798806	102.0247229	95.50076324	10.84442505	95.57058695	81.43137683	38.31551386	53.98210779	80.73314423	55.64791134	71.6112532	105.0937766	103.0477408	42.46126991	53.98210779	78.28933013	29.58760637	53.87894288	20.51058259
	54.70527763	94.54169276	67.2311001	93.374613	98.65841073	82.66780636	25.78171023	62.44903145	59.78769362	53.4233523	86.39642828	83.95720469	72.10954727	77.81558816	113.1888545	39.08839939	83.44402989	69.25022813	20.10788583	50.56541878	84.62684671	73.98252253	75.98617047	64.22562818	96.28399532	31.04894136	52.6353482	66.40407517	47.60837675	68.3200392	36.37161702

AMP3CPR2STR2ERY2TMP2	AMP	CPR	STR	ERY	TMP	AMP+CPR	AMP+STR	AMP+ERY	AMP+TMP	CPR+STR	CPR+ERY	CPR+TMP	STR+ERY	STR+TMP	ERY+TMP	AMP+CPR+STR	AMP+CPR+ERY	AMP+CPR+TMP	AMP+STR+ERY	AMP+STR+TMP	AMP+ERY+TMP	CPR+STR+ERY	CPR+STR+TMP	CPR+ERY+TMP	STR+ERY+TMP	AMP+CPR+STR+ERY	AMP+CPR+STR+TMP	AMP+CPR+ERY+TMP	AMP+STR+ERY+TMP	CPR+STR+ERY+TMP	AMP+CPR+STR+ERY+TMP
	90.7139524	79.50525784	68.10886253	104.2465318	100.8577782	98.34603619	11.07481723	97.1016747	94.11520713	42.02420771	56.65471521	73.34504416	46.49036263	64.662993	96.79127396	10.7429875	104.7337585	77.52372064	52.61575154	40.66988127	84.49214497	38.84784753	68.10886253	85.66547152	87.51353563	38.36781252	23.7673044	64.16757401	40.33805154	65.64477705	33.36962721
	82.47872573	81.56436488	78.75106564	119.344907	83.90986003	102.3783548	58.17150338	78.98756273	100.1090988	62.74509804	80.79710145	102.7067349	74.31798806	97.25063939	104.7734858	10.84442505	95.57058695	79.68579533	38.31551386	45.25420031	89.63560986	55.64791134	63.76811594	95.88661552	87.02046036	42.46126991	24.45996072	75.49639974	29.93672267	48.76385337	30.04582151
	54.70527763	94.54169276	67.2311001	93.374613	100.6398349	82.66780636	25.78171023	62.44903145	79.00846684	53.4233523	86.39642828	90.57795443	72.10954727	86.74488879	111.8679051	39.08839939	83.44402989	65.11036928	20.10788583	50.32515911	88.17529715	73.98252253	76.33463099	88.61786405	68.40715433	31.04894136	42.28570108	77.0494265	44.94703892	58.56314486	17.1508438

AMP3CPR2STR2ERY2TMP3	AMP	CPR	STR	ERY	TMP	AMP+CPR	AMP+STR	AMP+ERY	AMP+TMP	CPR+STR	CPR+ERY	CPR+TMP	STR+ERY	STR+TMP	ERY+TMP	AMP+CPR+STR	AMP+CPR+ERY	AMP+CPR+TMP	AMP+STR+ERY	AMP+STR+TMP	AMP+ERY+TMP	CPR+STR+ERY	CPR+STR+TMP	CPR+ERY+TMP	STR+ERY+TMP	AMP+CPR+STR+ERY	AMP+CPR+STR+TMP	AMP+CPR+ERY+TMP	AMP+STR+ERY+TMP	CPR+STR+ERY+TMP	AMP+CPR+STR+ERY+TMP
	90.7139524	79.50525784	68.10886253	104.2465318	81.88075823	98.34603619	11.07481723	97.1016747	101.0836315	42.02420771	56.65471521	72.72902279	46.49036263	78.2154631	100.1800275	10.7429875	104.7337585	79.09991186	52.61575154	35.71317468	78.51920983	38.84784753	41.10017566	102.9140698	87.20552494	38.36781252	17.1307098	59.5219578	52.61575154	39.56012224	3.173121792
	82.47872573	81.56436488	78.75106564	119.344907	64.70207757	102.3783548	58.17150338	78.98756273	97.66528475	62.74509804	80.79710145	98.61466326	74.31798806	89.06649616	113.7150397	10.84442505	95.57058695	79.68579533	38.31551386	37.61728126	90.50840061	55.64791134	55.24296675	96.56862745	110.5498721	42.46126991	22.36526293	81.78049313	27.60200742	60.35805627	38.0754964
	54.70527763	94.54169276	67.2311001	93.374613	105.9236326	82.66780636	25.78171023	62.44903145	82.26121308	53.4233523	86.39642828	78.73029701	72.10954727	93.71409904	113.8493292	39.08839939	83.44402989	65.51696256	20.10788583	34.06142793	78.52794751	73.98252253	63.09313152	73.63406202	98.02629788	31.04894136	21.05044298	80.59787694	50.56541878	88.87920943	23.23126148

AMP3CPR2STR2ERY3TMP1	AMP	CPR	STR	ERY	TMP	AMP+CPR	AMP+STR	AMP+ERY	AMP+TMP	CPR+STR	CPR+ERY	CPR+TMP	STR+ERY	STR+TMP	ERY+TMP	AMP+CPR+STR	AMP+CPR+ERY	AMP+CPR+TMP	AMP+STR+ERY	AMP+STR+TMP	AMP+ERY+TMP	CPR+STR+ERY	CPR+STR+TMP	CPR+ERY+TMP	STR+ERY+TMP	AMP+CPR+STR+ERY	AMP+CPR+STR+TMP	AMP+CPR+ERY+TMP	AMP+STR+ERY+TMP	CPR+STR+ERY+TMP	AMP+CPR+STR+ERY+TMP
	90.7139524	79.50525784	68.10886253	111.7017897	116.1071693	98.34603619	11.07481723	111.3703531	94.69590916	42.02420771	61.64063816	72.72902279	50.24424285	60.10058474	100.1800275	10.7429875	107.3883963	77.44076321	49.5670659	41.35428009	72.13148753	83.20138605	33.30365522	72.4210121	69.28315326	32.39487738	66.22077047	77.19189091	41.60315238	45.41232524	32.70596775
	82.47872573	81.56436488	78.75106564	116.0332204	104.4423171	102.3783548	58.17150338	86.84267947	106.5677504	62.74509804	93.49957374	108.5038363	75	102.0247229	121.9942563	10.84442505	88.58826096	81.43137683	45.99607244	53.98210779	98.1889592	92.13554987	71.6112532	103.7297528	105.4347826	34.12611826	53.98210779	79.16212088	44.01047349	71.95225916	51.34191578
	54.70527763	94.54169276	67.2311001	92.05366357	98.65841073	82.66780636	25.78171023	87.87959294	59.78769362	53.4233523	112.1825062	83.95720469	75.94261291	77.81558816	119.4633643	39.08839939	89.65381817	69.25022813	17.446548	50.56541878	85.03343999	87.92094302	75.98617047	85.13325892	80.95173277	25.78171023	52.6353482	72.02245504	64.03844155	86.43998584	33.28520439

AMP3CPR2STR2ERY3TMP2	AMP	CPR	STR	ERY	TMP	AMP+CPR	AMP+STR	AMP+ERY	AMP+TMP	CPR+STR	CPR+ERY	CPR+TMP	STR+ERY	STR+TMP	ERY+TMP	AMP+CPR+STR	AMP+CPR+ERY	AMP+CPR+TMP	AMP+STR+ERY	AMP+STR+TMP	AMP+ERY+TMP	CPR+STR+ERY	CPR+STR+TMP	CPR+ERY+TMP	STR+ERY+TMP	AMP+CPR+STR+ERY	AMP+CPR+STR+TMP	AMP+CPR+ERY+TMP	AMP+STR+ERY+TMP	CPR+STR+ERY+TMP	AMP+CPR+STR+ERY+TMP
	90.7139524	79.50525784	68.10886253	111.7017897	100.8577782	98.34603619	11.07481723	111.3703531	94.11520713	42.02420771	61.64063816	73.34504416	50.24424285	64.662993	89.3360161	10.7429875	107.3883963	77.52372064	49.5670659	40.66988127	70.55529631	83.20138605	68.10886253	67.80085184	81.29556994	32.39487738	23.7673044	76.86006118	21.77632602	60.19683808	-2.115414528
	82.47872573	81.56436488	78.75106564	116.0332204	83.90986003	102.3783548	58.17150338	86.84267947	100.1090988	62.74509804	93.49957374	102.7067349	75	97.25063939	93.84491992	10.84442505	88.58826096	79.68579533	45.99607244	45.25420031	96.4433777	92.13554987	63.76811594	89.7485081	79.85933504	34.12611826	24.45996072	80.90770238	30.04582151	63.91730605	0.960069823
	54.70527763	94.54169276	67.2311001	92.05366357	100.6398349	82.66780636	25.78171023	87.87959294	79.00846684	53.4233523	112.1825062	90.57795443	75.94261291	86.74488879	103.6119711	39.08839939	89.65381817	65.11036928	17.446548	50.32515911	84.44203158	87.92094302	76.33463099	87.224022	76.63953393	25.78171023	42.28570108	81.18928534	41.39858847	88.53074892	19.81218163

AMP3CPR2STR2ERY3TMP3	AMP	CPR	STR	ERY	TMP	AMP+CPR	AMP+STR	AMP+ERY	AMP+TMP	CPR+STR	CPR+ERY	CPR+TMP	STR+ERY	STR+TMP	ERY+TMP	AMP+CPR+STR	AMP+CPR+ERY	AMP+CPR+TMP	AMP+STR+ERY	AMP+STR+TMP	AMP+ERY+TMP	CPR+STR+ERY	CPR+STR+TMP	CPR+ERY+TMP	STR+ERY+TMP	AMP+CPR+STR+ERY	AMP+CPR+STR+TMP	AMP+CPR+ERY+TMP	AMP+STR+ERY+TMP	CPR+STR+ERY+TMP	AMP+CPR+STR+ERY+TMP
	90.7139524	79.50525784	68.10886253	111.7017897	81.88075823	98.34603619	11.07481723	111.3703531	101.0836315	42.02420771	61.64063816	72.72902279	50.24424285	78.2154631	80.5252568	10.7429875	107.3883963	79.09991186	49.5670659	35.71317468	62.84025509	83.20138605	41.10017566	82.89337536	73.96106552	32.39487738	17.1307098	71.5507855	27.39669207	66.35705176	9.477886659
	82.47872573	81.56436488	78.75106564	116.0332204	64.70207757	102.3783548	58.17150338	86.84267947	97.66528475	62.74509804	93.49957374	98.61466326	75	89.06649616	128.6176296	10.84442505	88.58826096	79.68579533	45.99607244	37.61728126	107.9642156	92.13554987	55.24296675	78.1543052	81.22335891	34.12611826	22.36526293	88.23914467	55.18219507	66.49616368	28.64935632
	54.70527763	94.54169276	67.2311001	92.05366357	105.9236326	82.66780636	25.78171023	87.87959294	82.26121308	53.4233523	112.1825062	78.73029701	75.94261291	93.71409904	99.97936017	39.08839939	89.65381817	65.51696256	17.446548	34.06142793	90.35611565	87.92094302	63.09313152	84.65412572	82.69403534	25.78171023	21.05044298	81.48498955	36.96302542	82.2584597	29.86612454

AMP3CPR2STR3ERY1TMP1	AMP	CPR	STR	ERY	TMP	AMP+CPR	AMP+STR	AMP+ERY	AMP+TMP	CPR+STR	CPR+ERY	CPR+TMP	STR+ERY	STR+TMP	ERY+TMP	AMP+CPR+STR	AMP+CPR+ERY	AMP+CPR+TMP	AMP+STR+ERY	AMP+STR+TMP	AMP+ERY+TMP	CPR+STR+ERY	CPR+STR+TMP	CPR+ERY+TMP	STR+ERY+TMP	AMP+CPR+STR+ERY	AMP+CPR+STR+TMP	AMP+CPR+ERY+TMP	AMP+STR+ERY+TMP	CPR+STR+ERY+TMP	AMP+CPR+STR+ERY+TMP
	90.7139524	79.50525784	74.20542334	101.535529	116.1071693	98.34603619	51.55804428	82.41820916	94.69590916	65.95278774	48.03041606	72.72902279	97.01871727	108.9645875	91.03039288	71.7996578	91.79239903	77.44076321	51.22621455	74.45429564	73.45880645	34.84370864	80.12127921	77.34918305	109.2964173	55.20817131	49.89889563	91.70944159	64.49940374	61.02461679	48.57157671
	82.47872573	81.56436488	60.83351516	110.4033531	104.4423171	102.3783548	44.77416539	86.31900502	106.5677504	79.51832907	73.29497016	108.5038363	81.43137683	106.9168667	89.87089597	50.01090988	93.47588916	81.43137683	52.80384028	83.17695832	102.0292385	62.80903666	78.1543052	108.5038363	116.6921231	57.34235217	51.05825878	95.3960288	74.79816714	79.85933504	61.53174776
	54.70527763	94.54169276	64.81466508	91.0629515	98.65841073	82.66780636	21.45703626	53.09738602	59.78769362	90.0117061	88.61786405	83.95720469	42.93255403	51.80368013	112.5283798	45.70478094	64.22325667	69.25022813	31.51097917	43.81966664	69.36111721	99.76860044	74.67944355	83.39095636	73.79668026	51.618865	44.81766833	64.33414575	45.11337253	59.34718101	46.29618934

AMP3CPR2STR3ERY1TMP2	AMP	CPR	STR	ERY	TMP	AMP+CPR	AMP+STR	AMP+ERY	AMP+TMP	CPR+STR	CPR+ERY	CPR+TMP	STR+ERY	STR+TMP	ERY+TMP	AMP+CPR+STR	AMP+CPR+ERY	AMP+CPR+TMP	AMP+STR+ERY	AMP+STR+TMP	AMP+ERY+TMP	CPR+STR+ERY	CPR+STR+TMP	CPR+ERY+TMP	STR+ERY+TMP	AMP+CPR+STR+ERY	AMP+CPR+STR+TMP	AMP+CPR+ERY+TMP	AMP+STR+ERY+TMP	CPR+STR+ERY+TMP	AMP+CPR+STR+ERY+TMP
	90.7139524	79.50525784	74.20542334	101.535529	100.8577782	98.34603619	51.55804428	82.41820916	94.11520713	65.95278774	48.03041606	73.34504416	97.01871727	90.7139524	92.04701896	71.7996578	91.79239903	77.52372064	51.22621455	58.85829834	67.90065847	34.84370864	83.81740742	81.96934331	108.6327578	55.20817131	41.60315238	90.7139524	33.63923887	75.50111895	61.18110644
	82.47872573	81.56436488	60.83351516	110.4033531	83.90986003	102.3783548	44.77416539	86.31900502	100.1090988	79.51832907	73.29497016	102.7067349	81.43137683	104.1239363	104.1111485	50.01090988	93.47588916	79.68579533	52.80384028	66.07025966	85.09709797	62.80903666	93.49957374	106.7988065	116.6921231	57.34235217	52.80384028	90.50840061	71.30700415	95.54560955	63.62644556
	54.70527763	94.54169276	64.81466508	91.0629515	100.6398349	82.66780636	21.45703626	53.09738602	79.00846684	90.0117061	88.61786405	90.57795443	42.93255403	58.12435748	107.244582	45.70478094	64.22325667	65.11036928	31.51097917	41.74973722	67.77170711	99.76860044	68.05869382	78.8609697	80.00646853	51.618865	33.76572372	75.57090548	50.43604819	72.58868048	69.65682141

AMP3CPR2STR3ERY1TMP3	AMP	CPR	STR	ERY	TMP	AMP+CPR	AMP+STR	AMP+ERY	AMP+TMP	CPR+STR	CPR+ERY	CPR+TMP	STR+ERY	STR+TMP	ERY+TMP	AMP+CPR+STR	AMP+CPR+ERY	AMP+CPR+TMP	AMP+STR+ERY	AMP+STR+TMP	AMP+ERY+TMP	CPR+STR+ERY	CPR+STR+TMP	CPR+ERY+TMP	STR+ERY+TMP	AMP+CPR+STR+ERY	AMP+CPR+STR+TMP	AMP+CPR+ERY+TMP	AMP+STR+ERY+TMP	CPR+STR+ERY+TMP	AMP+CPR+STR+ERY+TMP
	90.7139524	79.50525784	74.20542334	101.535529	81.88075823	98.34603619	51.55804428	82.41820916	101.0836315	65.95278774	48.03041606	72.72902279	97.01871727	91.4605693	100.8577782	71.7996578	91.79239903	79.09991186	51.22621455	53.21719293	81.7545497	34.84370864	75.80912963	89.66961041	107.9690984	55.20817131	43.59413076	86.40016591	49.5670659	79.19724715	49.89889563
	82.47872573	81.56436488	60.83351516	110.4033531	64.70207757	102.3783548	44.77416539	86.31900502	97.66528475	79.51832907	73.29497016	98.61466326	81.43137683	102.0292385	102.7864738	50.01090988	93.47588916	79.68579533	52.80384028	58.04058477	80.03491163	62.80903666	75.42625746	110.2088662	109.3606808	57.34235217	64.67379446	78.98756273	61.88086406	77.1312873	45.47239799
	54.70527763	94.54169276	64.81466508	91.0629515	105.9236326	82.66780636	21.45703626	53.09738602	82.26121308	90.0117061	88.61786405	78.73029701	42.93255403	70.72874915	113.5190918	45.70478094	64.22325667	65.51696256	31.51097917	47.36811708	67.47600291	99.76860044	87.224022	96.28399532	75.57090548	51.618865	42.7477389	59.01147009	54.87161124	85.48171943	53.68879443

AMP3CPR2STR3ERY2TMP1	AMP	CPR	STR	ERY	TMP	AMP+CPR	AMP+STR	AMP+ERY	AMP+TMP	CPR+STR	CPR+ERY	CPR+TMP	STR+ERY	STR+TMP	ERY+TMP	AMP+CPR+STR	AMP+CPR+ERY	AMP+CPR+TMP	AMP+STR+ERY	AMP+STR+TMP	AMP+ERY+TMP	CPR+STR+ERY	CPR+STR+TMP	CPR+ERY+TMP	STR+ERY+TMP	AMP+CPR+STR+ERY	AMP+CPR+STR+TMP	AMP+CPR+ERY+TMP	AMP+STR+ERY+TMP	CPR+STR+ERY+TMP	AMP+CPR+STR+ERY+TMP
	90.7139524	79.50525784	74.20542334	104.2465318	116.1071693	98.34603619	51.55804428	97.1016747	94.69590916	65.95278774	56.65471521	72.72902279	97.68237673	108.9645875	88.99714074	71.7996578	104.7337585	77.44076321	68.56431793	74.45429564	79.43174159	37.86606348	80.12127921	89.05358905	103.9871416	55.29112874	49.89889563	55.20817131	46.58059833	83.81740742	44.58961995
	82.47872573	81.56436488	60.83351516	119.344907	104.4423171	102.3783548	44.77416539	78.98756273	106.5677504	79.51832907	80.79710145	108.5038363	88.76281911	106.9168667	95.50076324	50.01090988	95.57058695	81.43137683	59.08793367	83.17695832	80.73314423	85.99744246	78.1543052	105.0937766	111.1062623	68.6886319	51.05825878	78.28933013	64.67379446	72.01619778	50.01090988
	54.70527763	94.54169276	64.81466508	93.374613	98.65841073	82.66780636	21.45703626	62.44903145	59.78769362	90.0117061	86.39642828	83.95720469	64.81466508	51.80368013	113.1888545	45.70478094	83.44402989	69.25022813	37.90558257	43.81966664	84.62684671	89.66324558	74.67944355	64.22562818	73.09438278	64.22325667	44.81766833	66.40407517	61.96851212	55.16565486	57.23724487

AMP3CPR2STR3ERY2TMP2	AMP	CPR	STR	ERY	TMP	AMP+CPR	AMP+STR	AMP+ERY	AMP+TMP	CPR+STR	CPR+ERY	CPR+TMP	STR+ERY	STR+TMP	ERY+TMP	AMP+CPR+STR	AMP+CPR+ERY	AMP+CPR+TMP	AMP+STR+ERY	AMP+STR+TMP	AMP+ERY+TMP	CPR+STR+ERY	CPR+STR+TMP	CPR+ERY+TMP	STR+ERY+TMP	AMP+CPR+STR+ERY	AMP+CPR+STR+TMP	AMP+CPR+ERY+TMP	AMP+STR+ERY+TMP	CPR+STR+ERY+TMP	AMP+CPR+STR+ERY+TMP
	90.7139524	79.50525784	74.20542334	104.2465318	100.8577782	98.34603619	51.55804428	97.1016747	94.11520713	65.95278774	56.65471521	73.34504416	97.68237673	90.7139524	96.79127396	71.7996578	104.7337585	77.52372064	68.56431793	58.85829834	84.49214497	37.86606348	83.81740742	85.66547152	106.6417794	55.29112874	41.60315238	64.16757401	31.98009022	90.59364247	36.2938767
	82.47872573	81.56436488	60.83351516	119.344907	83.90986003	102.3783548	44.77416539	78.98756273	100.1090988	79.51832907	80.79710145	102.7067349	88.76281911	104.1239363	104.7734858	50.01090988	95.57058695	79.68579533	59.08793367	66.07025966	89.63560986	85.99744246	93.49957374	95.88661552	106.5677504	68.6886319	52.80384028	75.49639974	56.99323587	84.63341858	37.79183941
	54.70527763	94.54169276	64.81466508	93.374613	100.6398349	82.66780636	21.45703626	62.44903145	79.00846684	90.0117061	86.39642828	90.57795443	64.81466508	58.12435748	111.8679051	45.70478094	83.44402989	65.11036928	37.90558257	41.74973722	88.17529715	89.66324558	68.05869382	88.61786405	77.93653911	64.22325667	33.76572372	77.0494265	32.58290691	53.52870716	48.36611877

AMP3CPR2STR3ERY2TMP3	AMP	CPR	STR	ERY	TMP	AMP+CPR	AMP+STR	AMP+ERY	AMP+TMP	CPR+STR	CPR+ERY	CPR+TMP	STR+ERY	STR+TMP	ERY+TMP	AMP+CPR+STR	AMP+CPR+ERY	AMP+CPR+TMP	AMP+STR+ERY	AMP+STR+TMP	AMP+ERY+TMP	CPR+STR+ERY	CPR+STR+TMP	CPR+ERY+TMP	STR+ERY+TMP	AMP+CPR+STR+ERY	AMP+CPR+STR+TMP	AMP+CPR+ERY+TMP	AMP+STR+ERY+TMP	CPR+STR+ERY+TMP	AMP+CPR+STR+ERY+TMP
	90.7139524	79.50525784	74.20542334	104.2465318	81.88075823	98.34603619	51.55804428	97.1016747	101.0836315	65.95278774	56.65471521	72.72902279	97.68237673	91.4605693	100.1800275	71.7996578	104.7337585	79.09991186	68.56431793	53.21719293	78.51920983	37.86606348	75.80912963	102.9140698	107.6372686	55.29112874	43.59413076	59.5219578	44.25779022	76.425151	53.88085239
	82.47872573	81.56436488	60.83351516	119.344907	64.70207757	102.3783548	44.77416539	78.98756273	97.66528475	79.51832907	80.79710145	98.61466326	88.76281911	102.0292385	113.7150397	50.01090988	95.57058695	79.68579533	59.08793367	58.04058477	90.50840061	85.99744246	75.42625746	96.56862745	103.0765874	68.6886319	64.67379446	81.78049313	65.37202706	72.35720375	53.15295658
	54.70527763	94.54169276	64.81466508	93.374613	105.9236326	82.66780636	21.45703626	62.44903145	82.26121308	90.0117061	86.39642828	78.73029701	64.81466508	70.72874915	113.8493292	45.70478094	83.44402989	65.51696256	37.90558257	47.36811708	78.52794751	89.66324558	87.224022	73.63406202	88.58189043	64.22325667	42.7477389	80.59787694	64.03844155	63.87716767	64.62984995

AMP3CPR2STR3ERY3TMP1	AMP	CPR	STR	ERY	TMP	AMP+CPR	AMP+STR	AMP+ERY	AMP+TMP	CPR+STR	CPR+ERY	CPR+TMP	STR+ERY	STR+TMP	ERY+TMP	AMP+CPR+STR	AMP+CPR+ERY	AMP+CPR+TMP	AMP+STR+ERY	AMP+STR+TMP	AMP+ERY+TMP	CPR+STR+ERY	CPR+STR+TMP	CPR+ERY+TMP	STR+ERY+TMP	AMP+CPR+STR+ERY	AMP+CPR+STR+TMP	AMP+CPR+ERY+TMP	AMP+STR+ERY+TMP	CPR+STR+ERY+TMP	AMP+CPR+STR+ERY+TMP
	90.7139524	79.50525784	74.20542334	111.7017897	116.1071693	98.34603619	51.55804428	111.3703531	94.69590916	65.95278774	61.64063816	72.72902279	92.37310105	108.9645875	100.1800275	71.7996578	107.3883963	77.44076321	69.22797739	74.45429564	72.13148753	72.11300142	80.12127921	72.4210121	100.6688443	55.29112874	49.89889563	77.19189091	29.32545238	73.96106552	50.23072536
	82.47872573	81.56436488	60.83351516	116.0332204	104.4423171	102.3783548	44.77416539	86.84267947	106.5677504	79.51832907	93.49957374	108.5038363	95.0469125	106.9168667	121.9942563	50.01090988	88.58826096	81.43137683	59.96072442	83.17695832	98.1889592	89.06649616	78.1543052	103.7297528	105.8695178	54.37486363	51.05825878	79.16212088	69.21230635	66.5601023	41.2830024
	54.70527763	94.54169276	64.81466508	92.05366357	98.65841073	82.66780636	21.45703626	87.87959294	59.78769362	90.0117061	112.1825062	83.95720469	67.47600291	51.80368013	119.4633643	45.70478094	89.65381817	69.25022813	50.32515911	43.81966664	85.03343999	78.8609697	74.67944355	85.13325892	84.14632738	49.1423423	44.81766833	72.02245504	104.8456216	69.10407535	53.39309022

AMP3CPR2STR3ERY3TMP2	AMP	CPR	STR	ERY	TMP	AMP+CPR	AMP+STR	AMP+ERY	AMP+TMP	CPR+STR	CPR+ERY	CPR+TMP	STR+ERY	STR+TMP	ERY+TMP	AMP+CPR+STR	AMP+CPR+ERY	AMP+CPR+TMP	AMP+STR+ERY	AMP+STR+TMP	AMP+ERY+TMP	CPR+STR+ERY	CPR+STR+TMP	CPR+ERY+TMP	STR+ERY+TMP	AMP+CPR+STR+ERY	AMP+CPR+STR+TMP	AMP+CPR+ERY+TMP	AMP+STR+ERY+TMP	CPR+STR+ERY+TMP	AMP+CPR+STR+ERY+TMP
	90.7139524	79.50525784	74.20542334	111.7017897	100.8577782	98.34603619	51.55804428	111.3703531	94.11520713	65.95278774	61.64063816	73.34504416	92.37310105	90.7139524	89.3360161	71.7996578	107.3883963	77.52372064	69.22797739	58.85829834	70.55529631	72.11300142	83.81740742	67.80085184	74.45429564	55.29112874	41.60315238	76.86006118	36.62570643	53.94037106	45.58510914
	82.47872573	81.56436488	60.83351516	116.0332204	83.90986003	102.3783548	44.77416539	86.84267947	100.1090988	79.51832907	93.49957374	102.7067349	95.0469125	104.1239363	93.84491992	50.01090988	88.58826096	79.68579533	59.96072442	66.07025966	96.4433777	89.06649616	93.49957374	89.7485081	92.95221471	54.37486363	52.80384028	80.90770238	65.02291076	84.97442455	40.2356535
	54.70527763	94.54169276	64.81466508	92.05366357	100.6398349	82.66780636	21.45703626	87.87959294	79.00846684	90.0117061	112.1825062	90.57795443	67.47600291	58.12435748	103.6119711	45.70478094	89.65381817	65.11036928	50.32515911	41.74973722	84.44203158	78.8609697	68.05869382	87.224022	85.03343999	49.1423423	33.76572372	81.18928534	57.53294907	85.83017995	62.85562473

AMP3CPR2STR3ERY3TMP3	AMP	CPR	STR	ERY	TMP	AMP+CPR	AMP+STR	AMP+ERY	AMP+TMP	CPR+STR	CPR+ERY	CPR+TMP	STR+ERY	STR+TMP	ERY+TMP	AMP+CPR+STR	AMP+CPR+ERY	AMP+CPR+TMP	AMP+STR+ERY	AMP+STR+TMP	AMP+ERY+TMP	CPR+STR+ERY	CPR+STR+TMP	CPR+ERY+TMP	STR+ERY+TMP	AMP+CPR+STR+ERY	AMP+CPR+STR+TMP	AMP+CPR+ERY+TMP	AMP+STR+ERY+TMP	CPR+STR+ERY+TMP	AMP+CPR+STR+ERY+TMP
	90.7139524	79.50525784	74.20542334	111.7017897	81.88075823	98.34603619	51.55804428	111.3703531	101.0836315	65.95278774	61.64063816	72.72902279	92.37310105	91.4605693	80.5252568	71.7996578	107.3883963	79.09991186	69.22797739	53.21719293	62.84025509	72.11300142	75.80912963	82.89337536	91.70944159	55.29112874	43.59413076	71.5507855	32.31191995	70.88095868	20.36604967
	82.47872573	81.56436488	60.83351516	116.0332204	64.70207757	102.3783548	44.77416539	86.84267947	97.66528475	79.51832907	93.49957374	98.61466326	95.0469125	102.0292385	128.6176296	50.01090988	88.58826096	79.68579533	59.96072442	58.04058477	107.9642156	89.06649616	75.42625746	78.1543052	98.1889592	54.37486363	64.67379446	88.23914467	74.09993454	45.75873828	22.91075715
	54.70527763	94.54169276	64.81466508	92.05366357	105.9236326	82.66780636	21.45703626	87.87959294	82.26121308	90.0117061	112.1825062	78.73029701	67.47600291	70.72874915	99.97936017	45.70478094	89.65381817	65.51696256	50.32515911	47.36811708	90.35611565	78.8609697	87.224022	84.65412572	77.3451307	49.1423423	42.7477389	81.48498955	71.43104663	65.61947023	51.91456921

AMP3CPR3STR1ERY1TMP1	AMP	CPR	STR	ERY	TMP	AMP+CPR	AMP+STR	AMP+ERY	AMP+TMP	CPR+STR	CPR+ERY	CPR+TMP	STR+ERY	STR+TMP	ERY+TMP	AMP+CPR+STR	AMP+CPR+ERY	AMP+CPR+TMP	AMP+STR+ERY	AMP+STR+TMP	AMP+ERY+TMP	CPR+STR+ERY	CPR+STR+TMP	CPR+ERY+TMP	STR+ERY+TMP	AMP+CPR+STR+ERY	AMP+CPR+STR+TMP	AMP+CPR+ERY+TMP	AMP+STR+ERY+TMP	CPR+STR+ERY+TMP	AMP+CPR+STR+ERY+TMP
	90.7139524	86.40016591	99.50227682	101.535529	116.1071693	113.278374	14.0612848	82.41820916	94.69590916	83.80774615	105.6462902	74.12246591	112.3795404	83.23625966	91.03039288	24.0161767	102.6598227	80.17835848	40.69062063	46.97464613	73.45880645	88.7851921	32.39487738	94.69590916	92.38589431	44.34074765	26.09011251	97.01871727	22.43998548	28.08109089	7.507647638
	82.47872573	75.49639974	102.1241365	110.4033531	104.4423171	105.1712852	20.27056513	86.31900502	106.5677504	63.05913157	92.25398211	102.0292385	132.9228222	77.28648677	89.87089597	28.30024002	94.52323805	86.84267947	36.6790312	44.25049094	102.0292385	80.86406284	79.27121973	113.5500764	78.61116142	35.87169976	34.82435086	94.6977962	31.33318787	56.57866027	20.85969889
	54.70527763	49.1423423	84.12796698	91.0629515	98.65841073	66.99548358	35.53994894	53.09738602	59.78769362	40.67780948	52.09938433	54.87161124	79.50464396	97.3374613	112.5283798	25.19030183	57.82865328	67.77170711	50.91656752	47.01696834	69.36111721	57.66231966	40.2712162	78.52794751	99.31888545	24.59889342	24.59889342	62.26421633	39.08839939	52.0439398	39.08839939

AMP3CPR3STR1ERY1TMP2	AMP	CPR	STR	ERY	TMP	AMP+CPR	AMP+STR	AMP+ERY	AMP+TMP	CPR+STR	CPR+ERY	CPR+TMP	STR+ERY	STR+TMP	ERY+TMP	AMP+CPR+STR	AMP+CPR+ERY	AMP+CPR+TMP	AMP+STR+ERY	AMP+STR+TMP	AMP+ERY+TMP	CPR+STR+ERY	CPR+STR+TMP	CPR+ERY+TMP	STR+ERY+TMP	AMP+CPR+STR+ERY	AMP+CPR+STR+TMP	AMP+CPR+ERY+TMP	AMP+STR+ERY+TMP	CPR+STR+ERY+TMP	AMP+CPR+STR+ERY+TMP
	90.7139524	86.40016591	99.50227682	101.535529	100.8577782	113.278374	14.0612848	82.41820916	94.11520713	83.80774615	105.6462902	94.44703686	112.3795404	65.27586572	92.04701896	24.0161767	102.6598227	87.14678281	40.69062063	51.30917198	67.90065847	88.7851921	59.25234614	92.04127132	85.60838717	44.34074765	21.42375693	100.0051848	11.4896044	47.30647586	10.47337585
	82.47872573	75.49639974	102.1241365	110.4033531	83.90986003	105.1712852	20.27056513	86.31900502	100.1090988	63.05913157	92.25398211	104.4730526	132.9228222	69.00727019	104.1111485	28.30024002	94.52323805	101.1564477	36.6790312	44.25049094	85.09709797	80.86406284	85.55531311	105.8695178	95.16959458	35.87169976	25.74732708	98.5380755	28.19114117	45.40693869	25.74732708
	54.70527763	49.1423423	84.12796698	91.0629515	100.6398349	66.99548358	35.53994894	53.09738602	79.00846684	40.67780948	52.09938433	68.06741132	79.50464396	87.76057792	107.244582	25.19030183	57.82865328	81.37410047	50.91656752	25.19030183	67.77170711	57.66231966	67.42055837	71.72675083	100.6398349	24.59889342	32.87861111	71.43104663	32.58290691	50.26971458	34.65283633

AMP3CPR3STR1ERY1TMP3	AMP	CPR	STR	ERY	TMP	AMP+CPR	AMP+STR	AMP+ERY	AMP+TMP	CPR+STR	CPR+ERY	CPR+TMP	STR+ERY	STR+TMP	ERY+TMP	AMP+CPR+STR	AMP+CPR+ERY	AMP+CPR+TMP	AMP+STR+ERY	AMP+STR+TMP	AMP+ERY+TMP	CPR+STR+ERY	CPR+STR+TMP	CPR+ERY+TMP	STR+ERY+TMP	AMP+CPR+STR+ERY	AMP+CPR+STR+TMP	AMP+CPR+ERY+TMP	AMP+STR+ERY+TMP	CPR+STR+ERY+TMP	AMP+CPR+STR+ERY+TMP
	90.7139524	86.40016591	99.50227682	101.535529	81.88075823	113.278374	14.0612848	82.41820916	101.0836315	83.80774615	105.6462902	105.0655882	112.3795404	74.42550037	100.8577782	24.0161767	102.6598227	72.79514699	40.69062063	44.34074765	81.7545497	88.7851921	74.84834344	101.6643335	92.38589431	44.34074765	18.45802872	89.38663348	7.507647638	21.11266656	14.14424224
	82.47872573	75.49639974	102.1241365	110.4033531	64.70207757	105.1712852	20.27056513	86.31900502	97.66528475	63.05913157	92.25398211	102.0292385	132.9228222	68.34493286	102.7864738	28.30024002	94.52323805	102.2037966	36.6790312	41.80667685	80.03491163	80.86406284	84.50796422	111.4553786	92.85141393	35.87169976	24.52542003	85.27165612	30.63495527	26.27100153	25.74732708
	54.70527763	49.1423423	84.12796698	91.0629515	105.9236326	66.99548358	35.53994894	53.09738602	82.26121308	40.67780948	52.09938433	63.04043986	79.50464396	91.0629515	113.5190918	25.19030183	57.82865328	76.93853742	50.91656752	38.73725064	67.47600291	57.66231966	44.94703892	71.13534243	108.8957688	24.59889342	16.02347152	81.96550888	38.79269518	37.31417417	23.41607661

AMP3CPR3STR1ERY2TMP1	AMP	CPR	STR	ERY	TMP	AMP+CPR	AMP+STR	AMP+ERY	AMP+TMP	CPR+STR	CPR+ERY	CPR+TMP	STR+ERY	STR+TMP	ERY+TMP	AMP+CPR+STR	AMP+CPR+ERY	AMP+CPR+TMP	AMP+STR+ERY	AMP+STR+TMP	AMP+ERY+TMP	CPR+STR+ERY	CPR+STR+TMP	CPR+ERY+TMP	STR+ERY+TMP	AMP+CPR+STR+ERY	AMP+CPR+STR+TMP	AMP+CPR+ERY+TMP	AMP+STR+ERY+TMP	CPR+STR+ERY+TMP	AMP+CPR+STR+ERY+TMP
	90.7139524	86.40016591	99.50227682	104.2465318	116.1071693	113.278374	14.0612848	97.1016747	94.69590916	83.80774615	106.9736092	74.12246591	98.845261	83.23625966	88.99714074	24.0161767	100.0881423	80.17835848	46.33172603	46.97464613	79.43174159	70.53455696	32.39487738	101.000674	88.31939002	49.98185306	26.09011251	54.87634158	15.80339089	80.82127858	-3.463472806
	82.47872573	75.49639974	102.1241365	119.344907	104.4423171	105.1712852	20.27056513	78.98756273	106.5677504	63.05913157	91.55574951	102.0292385	95.16959458	77.28648677	95.50076324	28.30024002	107.7896574	86.84267947	46.34518874	44.25049094	80.73314423	64.45559677	79.27121973	110.757146	86.55920933	53.67663103	34.82435086	84.22430722	25.57276893	42.26489199	24.35086188
	54.70527763	49.1423423	84.12796698	93.374613	98.65841073	66.99548358	35.53994894	62.44903145	59.78769362	40.67780948	60.08339783	54.87161124	80.8255934	97.3374613	113.1888545	25.19030183	56.83065159	67.77170711	42.04544142	47.01696834	84.62684671	44.22625992	40.2712162	80.19128366	111.2074303	30.80868169	24.59889342	66.8845945	31.10438589	47.01696834	20.16333037

AMP3CPR3STR1ERY2TMP2	AMP	CPR	STR	ERY	TMP	AMP+CPR	AMP+STR	AMP+ERY	AMP+TMP	CPR+STR	CPR+ERY	CPR+TMP	STR+ERY	STR+TMP	ERY+TMP	AMP+CPR+STR	AMP+CPR+ERY	AMP+CPR+TMP	AMP+STR+ERY	AMP+STR+TMP	AMP+ERY+TMP	CPR+STR+ERY	CPR+STR+TMP	CPR+ERY+TMP	STR+ERY+TMP	AMP+CPR+STR+ERY	AMP+CPR+STR+TMP	AMP+CPR+ERY+TMP	AMP+STR+ERY+TMP	CPR+STR+ERY+TMP	AMP+CPR+STR+ERY+TMP
	90.7139524	86.40016591	99.50227682	104.2465318	100.8577782	113.278374	14.0612848	97.1016747	94.11520713	83.80774615	106.9736092	94.44703686	98.845261	65.27586572	96.79127396	24.0161767	100.0881423	87.14678281	46.33172603	51.30917198	84.49214497	70.53455696	59.25234614	90.7139524	88.31939002	49.98185306	21.42375693	69.47684969	33.39036657	64.56162182	20.4490071
	82.47872573	75.49639974	102.1241365	119.344907	83.90986003	105.1712852	20.27056513	78.98756273	100.1090988	63.05913157	91.55574951	104.4730526	95.16959458	69.00727019	104.7734858	28.30024002	107.7896574	101.1564477	46.34518874	44.25049094	89.63560986	64.45559677	85.55531311	103.0765874	90.20206463	53.67663103	25.74732708	83.87519092	22.25616408	15.6229544	25.04909448
	54.70527763	49.1423423	84.12796698	93.374613	100.6398349	66.99548358	35.53994894	62.44903145	79.00846684	40.67780948	60.08339783	68.06741132	80.8255934	87.76057792	111.8679051	25.19030183	56.83065159	81.37410047	42.04544142	25.19030183	88.17529715	44.22625992	67.42055837	77.6408349	103.6119711	30.80868169	32.87861111	73.09438278	39.92006746	56.77520705	10.99650006

AMP3CPR3STR1ERY2TMP3	AMP	CPR	STR	ERY	TMP	AMP+CPR	AMP+STR	AMP+ERY	AMP+TMP	CPR+STR	CPR+ERY	CPR+TMP	STR+ERY	STR+TMP	ERY+TMP	AMP+CPR+STR	AMP+CPR+ERY	AMP+CPR+TMP	AMP+STR+ERY	AMP+STR+TMP	AMP+ERY+TMP	CPR+STR+ERY	CPR+STR+TMP	CPR+ERY+TMP	STR+ERY+TMP	AMP+CPR+STR+ERY	AMP+CPR+STR+TMP	AMP+CPR+ERY+TMP	AMP+STR+ERY+TMP	CPR+STR+ERY+TMP	AMP+CPR+STR+ERY+TMP
	90.7139524	86.40016591	99.50227682	104.2465318	81.88075823	113.278374	14.0612848	97.1016747	101.0836315	83.80774615	106.9736092	105.0655882	98.845261	74.42550037	100.1800275	24.0161767	100.0881423	72.79514699	46.33172603	44.34074765	78.51920983	70.53455696	74.84834344	100.6688443	89.3360161	49.98185306	18.45802872	71.88261523	21.11266656	61.90698398	13.48058278
	82.47872573	75.49639974	102.1241365	119.344907	64.70207757	105.1712852	20.27056513	78.98756273	97.66528475	63.05913157	91.55574951	102.3783548	95.16959458	68.34493286	113.7150397	28.30024002	107.7896574	102.2037966	46.34518874	41.80667685	90.50840061	64.45559677	84.50796422	97.8398429	109.4098471	53.67663103	24.52542003	94.6977962	22.95439668	29.34758892	12.4809077
	54.70527763	49.1423423	84.12796698	93.374613	105.9236326	66.99548358	35.53994894	62.44903145	82.26121308	40.67780948	60.08339783	63.04043986	80.8255934	91.0629515	113.8493292	25.19030183	56.83065159	76.93853742	42.04544142	38.73725064	78.52794751	44.22625992	44.94703892	93.01745348	117.4819401	30.80868169	16.02347152	90.94752405	47.07241288	64.16781214	18.09340094

AMP3CPR3STR1ERY3TMP1	AMP	CPR	STR	ERY	TMP	AMP+CPR	AMP+STR	AMP+ERY	AMP+TMP	CPR+STR	CPR+ERY	CPR+TMP	STR+ERY	STR+TMP	ERY+TMP	AMP+CPR+STR	AMP+CPR+ERY	AMP+CPR+TMP	AMP+STR+ERY	AMP+STR+TMP	AMP+ERY+TMP	CPR+STR+ERY	CPR+STR+TMP	CPR+ERY+TMP	STR+ERY+TMP	AMP+CPR+STR+ERY	AMP+CPR+STR+TMP	AMP+CPR+ERY+TMP	AMP+STR+ERY+TMP	CPR+STR+ERY+TMP	AMP+CPR+STR+ERY+TMP
	90.7139524	86.40016591	99.50227682	111.7017897	116.1071693	113.278374	14.0612848	111.3703531	94.69590916	83.80774615	98.01420646	74.12246591	75.10325109	83.23625966	100.1800275	24.0161767	86.15129362	80.17835848	60.60040442	46.97464613	72.13148753	76.17566236	32.39487738	93.70041997	92.38589431	42.681599	26.09011251	58.27759631	35.38134495	43.65634884	11.4896044
	82.47872573	75.49639974	102.1241365	116.0332204	104.4423171	105.1712852	20.27056513	86.84267947	106.5677504	63.05913157	102.7274711	102.0292385	85.56570335	77.28648677	121.9942563	28.30024002	99.41086624	86.84267947	66.5939341	44.25049094	98.1889592	86.44992363	79.27121973	91.20663321	111.0656904	43.20314205	34.82435086	93.30133101	22.36526293	53.78572987	26.09644338
	54.70527763	49.1423423	84.12796698	92.05366357	98.65841073	66.99548358	35.53994894	87.87959294	59.78769362	40.67780948	79.59987525	54.87161124	78.18369453	97.3374613	119.4633643	25.19030183	63.92755247	67.77170711	37.90558257	47.01696834	85.03343999	39.92006746	40.2712162	92.72174928	115.8307534	26.37311864	24.59889342	90.65181985	12.29020595	63.28069953	14.84065471

AMP3CPR3STR1ERY3TMP2	AMP	CPR	STR	ERY	TMP	AMP+CPR	AMP+STR	AMP+ERY	AMP+TMP	CPR+STR	CPR+ERY	CPR+TMP	STR+ERY	STR+TMP	ERY+TMP	AMP+CPR+STR	AMP+CPR+ERY	AMP+CPR+TMP	AMP+STR+ERY	AMP+STR+TMP	AMP+ERY+TMP	CPR+STR+ERY	CPR+STR+TMP	CPR+ERY+TMP	STR+ERY+TMP	AMP+CPR+STR+ERY	AMP+CPR+STR+TMP	AMP+CPR+ERY+TMP	AMP+STR+ERY+TMP	CPR+STR+ERY+TMP	AMP+CPR+STR+ERY+TMP
	90.7139524	86.40016591	99.50227682	111.7017897	100.8577782	113.278374	14.0612848	111.3703531	94.11520713	83.80774615	98.01420646	94.44703686	75.10325109	65.27586572	89.3360161	24.0161767	86.15129362	87.14678281	60.60040442	51.30917198	70.55529631	76.17566236	59.25234614	83.08186862	85.94726252	42.681599	21.42375693	75.44978483	19.12168818	7.507647638	17.79436926
	82.47872573	75.49639974	102.1241365	116.0332204	83.90986003	105.1712852	20.27056513	86.84267947	100.1090988	63.05913157	102.7274711	104.4730526	85.56570335	69.00727019	93.84491992	28.30024002	99.41086624	101.1564477	66.5939341	44.25049094	96.4433777	86.44992363	85.55531311	99.2363081	101.1306305	43.20314205	25.74732708	93.9995636	25.50730962	113.1355008	21.66703033
	54.70527763	49.1423423	84.12796698	92.05366357	100.6398349	66.99548358	35.53994894	87.87959294	79.00846684	40.67780948	79.59987525	68.06741132	78.18369453	87.76057792	103.6119711	25.19030183	63.92755247	81.37410047	37.90558257	25.19030183	84.44203158	39.92006746	67.42055837	86.80766521	103.9422085	26.37311864	32.87861111	93.60886189	10.10938745	72.44752983	8.150347106

AMP3CPR3STR1ERY3TMP3	AMP	CPR	STR	ERY	TMP	AMP+CPR	AMP+STR	AMP+ERY	AMP+TMP	CPR+STR	CPR+ERY	CPR+TMP	STR+ERY	STR+TMP	ERY+TMP	AMP+CPR+STR	AMP+CPR+ERY	AMP+CPR+TMP	AMP+STR+ERY	AMP+STR+TMP	AMP+ERY+TMP	CPR+STR+ERY	CPR+STR+TMP	CPR+ERY+TMP	STR+ERY+TMP	AMP+CPR+STR+ERY	AMP+CPR+STR+TMP	AMP+CPR+ERY+TMP	AMP+STR+ERY+TMP	CPR+STR+ERY+TMP	AMP+CPR+STR+ERY+TMP
	90.7139524	86.40016591	99.50227682	111.7017897	81.88075823	113.278374	14.0612848	111.3703531	101.0836315	83.80774615	98.01420646	105.0655882	75.10325109	74.42550037	80.5252568	24.0161767	86.15129362	72.79514699	60.60040442	44.34074765	62.84025509	76.17566236	74.84834344	77.44076321	65.91125702	42.681599	18.45802872	76.86006118	20.4490071	18.12619899	12.48509359
	82.47872573	75.49639974	102.1241365	116.0332204	64.70207757	105.1712852	20.27056513	86.84267947	97.66528475	63.05913157	102.7274711	102.3783548	85.56570335	68.34493286	128.6176296	28.30024002	99.41086624	102.2037966	66.5939341	41.80667685	107.9642156	86.44992363	84.50796422	92.60309841	83.2475227	43.20314205	24.52542003	92.77765656	46.45428758	24.45996072	17.47763474
	54.70527763	49.1423423	84.12796698	92.05366357	105.9236326	66.99548358	35.53994894	87.87959294	82.26121308	40.67780948	79.59987525	63.04043986	78.18369453	91.0629515	99.97936017	25.19030183	63.92755247	76.93853742	37.90558257	38.73725064	90.35611565	39.92006746	44.94703892	77.93653911	93.70485036	26.37311864	16.02347152	90.94752405	13.06642949	54.40957342	8.92657064

AMP3CPR3STR2ERY1TMP1	AMP	CPR	STR	ERY	TMP	AMP+CPR	AMP+STR	AMP+ERY	AMP+TMP	CPR+STR	CPR+ERY	CPR+TMP	STR+ERY	STR+TMP	ERY+TMP	AMP+CPR+STR	AMP+CPR+ERY	AMP+CPR+TMP	AMP+STR+ERY	AMP+STR+TMP	AMP+ERY+TMP	CPR+STR+ERY	CPR+STR+TMP	CPR+ERY+TMP	STR+ERY+TMP	AMP+CPR+STR+ERY	AMP+CPR+STR+TMP	AMP+CPR+ERY+TMP	AMP+STR+ERY+TMP	CPR+STR+ERY+TMP	AMP+CPR+STR+ERY+TMP
	90.7139524	86.40016591	68.10886253	101.535529	116.1071693	113.278374	11.07481723	82.41820916	94.69590916	76.83932182	105.6462902	74.12246591	45.93209327	60.10058474	91.03039288	18.37507129	102.6598227	80.17835848	46.31098667	41.35428009	73.45880645	75.5120029	60.84927671	94.69590916	83.20138605	44.00891792	40.3587909	97.01871727	44.65183803	65.88894074	32.04230829
	82.47872573	75.49639974	78.75106564	110.4033531	104.4423171	105.1712852	58.17150338	86.31900502	106.5677504	58.78881737	92.25398211	102.0292385	78.1543052	102.0247229	89.87089597	32.48963561	94.52323805	86.84267947	54.68034039	53.98210779	102.0292385	67.81584115	63.40824787	113.5500764	110.8908781	59.21885228	39.36286275	94.6977962	48.396247	53.50207288	52.93475889
	54.70527763	49.1423423	67.2311001	91.0629515	98.65841073	66.99548358	25.78171023	53.09738602	59.78769362	49.25323138	52.09938433	54.87161124	81.6486538	77.81558816	112.5283798	45.00248345	57.82865328	67.77170711	24.24774468	50.56541878	69.36111721	43.93055572	45.83415153	78.52794751	75.02790406	42.7477389	32.82316658	62.26421633	29.33016067	58.84513647	54.57590704

AMP3CPR3STR2ERY1TMP2	AMP	CPR	STR	ERY	TMP	AMP+CPR	AMP+STR	AMP+ERY	AMP+TMP	CPR+STR	CPR+ERY	CPR+TMP	STR+ERY	STR+TMP	ERY+TMP	AMP+CPR+STR	AMP+CPR+ERY	AMP+CPR+TMP	AMP+STR+ERY	AMP+STR+TMP	AMP+ERY+TMP	CPR+STR+ERY	CPR+STR+TMP	CPR+ERY+TMP	STR+ERY+TMP	AMP+CPR+STR+ERY	AMP+CPR+STR+TMP	AMP+CPR+ERY+TMP	AMP+STR+ERY+TMP	CPR+STR+ERY+TMP	AMP+CPR+STR+ERY+TMP
	90.7139524	86.40016591	68.10886253	101.535529	100.8577782	113.278374	11.07481723	82.41820916	94.11520713	76.83932182	105.6462902	94.44703686	45.93209327	64.662993	92.04701896	18.37507129	102.6598227	87.14678281	46.31098667	40.66988127	67.90065847	75.5120029	85.072847	92.04127132	86.89751426	44.00891792	30.38315964	100.0051848	24.76279359	51.95209208	23.08290558
	82.47872573	75.49639974	78.75106564	110.4033531	83.90986003	105.1712852	58.17150338	86.31900502	100.1090988	58.78881737	92.25398211	104.4730526	78.1543052	97.25063939	104.1111485	32.48963561	94.52323805	101.1564477	54.68034039	45.25420031	85.09709797	67.81584115	76.54374864	105.8695178	109.8678602	59.21885228	35.87169976	98.5380755	32.38053677	61.31355008	30.28583897
	54.70527763	49.1423423	67.2311001	91.0629515	100.6398349	66.99548358	25.78171023	53.09738602	79.00846684	49.25323138	52.09938433	68.06741132	81.6486538	86.74488879	107.244582	45.00248345	57.82865328	81.37410047	24.24774468	50.32515911	67.77170711	43.93055572	38.14584224	71.72675083	191.2830425	42.7477389	33.8766128	71.43104663	41.10288427	69.786192	37.85013803

AMP3CPR3STR2ERY1TMP3	AMP	CPR	STR	ERY	TMP	AMP+CPR	AMP+STR	AMP+ERY	AMP+TMP	CPR+STR	CPR+ERY	CPR+TMP	STR+ERY	STR+TMP	ERY+TMP	AMP+CPR+STR	AMP+CPR+ERY	AMP+CPR+TMP	AMP+STR+ERY	AMP+STR+TMP	AMP+ERY+TMP	CPR+STR+ERY	CPR+STR+TMP	CPR+ERY+TMP	STR+ERY+TMP	AMP+CPR+STR+ERY	AMP+CPR+STR+TMP	AMP+CPR+ERY+TMP	AMP+STR+ERY+TMP	CPR+STR+ERY+TMP	AMP+CPR+STR+ERY+TMP
	90.7139524	86.40016591	68.10886253	101.535529	81.88075823	113.278374	11.07481723	82.41820916	101.0836315	76.83932182	105.6462902	105.0655882	45.93209327	78.2154631	100.8577782	18.37507129	102.6598227	72.79514699	46.31098667	35.71317468	81.7545497	75.5120029	68.14953077	101.6643335	74.26907621	44.00891792	31.37864883	89.38663348	29.74023954	56.5977083	37.01975424
	82.47872573	75.49639974	78.75106564	110.4033531	64.70207757	105.1712852	58.17150338	86.31900502	97.66528475	58.78881737	92.25398211	102.3783548	78.1543052	89.06649616	102.7864738	32.48963561	94.52323805	102.2037966	54.68034039	37.61728126	80.03491163	67.81584115	68.51407375	111.4553786	114.3009378	59.21885228	33.18786821	85.27165612	35.47894392	185.9917085	30.46039712
	54.70527763	49.1423423	67.2311001	91.0629515	105.9236326	66.99548358	25.78171023	53.09738602	82.26121308	49.25323138	52.09938433	63.04043986	81.6486538	93.71409904	113.5190918	45.00248345	57.82865328	76.93853742	24.24774468	34.06142793	67.47600291	43.93055572	61.08139951	71.13534243	106.3893502	42.7477389	44.0599263	81.96550888	31.9914985	51.91456921	52.21027341

AMP3CPR3STR2ERY2TMP1	AMP	CPR	STR	ERY	TMP	AMP+CPR	AMP+STR	AMP+ERY	AMP+TMP	CPR+STR	CPR+ERY	CPR+TMP	STR+ERY	STR+TMP	ERY+TMP	AMP+CPR+STR	AMP+CPR+ERY	AMP+CPR+TMP	AMP+STR+ERY	AMP+STR+TMP	AMP+ERY+TMP	CPR+STR+ERY	CPR+STR+TMP	CPR+ERY+TMP	STR+ERY+TMP	AMP+CPR+STR+ERY	AMP+CPR+STR+TMP	AMP+CPR+ERY+TMP	AMP+STR+ERY+TMP	CPR+STR+ERY+TMP	AMP+CPR+STR+ERY+TMP
	90.7139524	86.40016591	68.10886253	104.2465318	116.1071693	113.278374	11.07481723	97.1016747	94.69590916	76.83932182	106.9736092	74.12246591	46.49036263	60.10058474	88.99714074	18.37507129	100.0881423	80.17835848	52.61575154	41.35428009	79.43174159	103.6553119	60.84927671	101.000674	86.89751426	49.65002333	40.3587909	54.87634158	35.04951522	53.94307046	6.512158449
	82.47872573	75.49639974	78.75106564	119.344907	104.4423171	105.1712852	58.17150338	78.98756273	106.5677504	58.78881737	91.55574951	102.0292385	74.31798806	102.0247229	95.50076324	32.48963561	107.7896574	86.84267947	38.31551386	53.98210779	80.73314423	78.63844643	63.40824787	110.757146	103.0477408	59.96072442	39.36286275	84.22430722	29.58760637	58.86973598	29.34758892
	54.70527763	49.1423423	67.2311001	93.374613	98.65841073	66.99548358	25.78171023	62.44903145	59.78769362	49.25323138	60.08339783	54.87161124	72.10954727	77.81558816	113.1888545	45.00248345	56.83065159	67.77170711	20.10788583	50.56541878	84.62684671	42.7477389	45.83415153	80.19128366	96.28399532	54.46501796	32.82316658	66.8845945	47.60837675	64.62984995	31.9914985

AMP3CPR3STR2ERY2TMP2	AMP	CPR	STR	ERY	TMP	AMP+CPR	AMP+STR	AMP+ERY	AMP+TMP	CPR+STR	CPR+ERY	CPR+TMP	STR+ERY	STR+TMP	ERY+TMP	AMP+CPR+STR	AMP+CPR+ERY	AMP+CPR+TMP	AMP+STR+ERY	AMP+STR+TMP	AMP+ERY+TMP	CPR+STR+ERY	CPR+STR+TMP	CPR+ERY+TMP	STR+ERY+TMP	AMP+CPR+STR+ERY	AMP+CPR+STR+TMP	AMP+CPR+ERY+TMP	AMP+STR+ERY+TMP	CPR+STR+ERY+TMP	AMP+CPR+STR+ERY+TMP
	90.7139524	86.40016591	68.10886253	104.2465318	100.8577782	113.278374	11.07481723	97.1016747	94.11520713	76.83932182	106.9736092	94.44703686	46.49036263	64.662993	96.79127396	18.37507129	100.0881423	87.14678281	52.61575154	40.66988127	84.49214497	103.6553119	85.072847	90.7139524	87.51353563	49.65002333	30.38315964	69.47684969	40.33805154	58.92051641	27.0856017
	82.47872573	75.49639974	78.75106564	119.344907	83.90986003	105.1712852	58.17150338	78.98756273	100.1090988	58.78881737	91.55574951	104.4730526	74.31798806	97.25063939	104.7734858	32.48963561	107.7896574	101.1564477	38.31551386	45.25420031	89.63560986	78.63844643	76.54374864	103.0765874	87.02046036	59.96072442	35.87169976	83.87519092	29.93672267	57.34235217	64.25921885
	54.70527763	49.1423423	67.2311001	93.374613	100.6398349	66.99548358	25.78171023	62.44903145	79.00846684	49.25323138	60.08339783	68.06741132	72.10954727	86.74488879	111.8679051	45.00248345	56.83065159	81.37410047	20.10788583	50.32515911	88.17529715	42.7477389	38.14584224	77.6408349	68.40715433	54.46501796	33.8766128	73.09438278	44.94703892	62.98499532	57.53294907

AMP3CPR3STR2ERY2TMP3	AMP	CPR	STR	ERY	TMP	AMP+CPR	AMP+STR	AMP+ERY	AMP+TMP	CPR+STR	CPR+ERY	CPR+TMP	STR+ERY	STR+TMP	ERY+TMP	AMP+CPR+STR	AMP+CPR+ERY	AMP+CPR+TMP	AMP+STR+ERY	AMP+STR+TMP	AMP+ERY+TMP	CPR+STR+ERY	CPR+STR+TMP	CPR+ERY+TMP	STR+ERY+TMP	AMP+CPR+STR+ERY	AMP+CPR+STR+TMP	AMP+CPR+ERY+TMP	AMP+STR+ERY+TMP	CPR+STR+ERY+TMP	AMP+CPR+STR+ERY+TMP
	90.7139524	86.40016591	68.10886253	104.2465318	81.88075823	113.278374	11.07481723	97.1016747	101.0836315	76.83932182	106.9736092	105.0655882	46.49036263	78.2154631	100.1800275	18.37507129	100.0881423	72.79514699	52.61575154	35.71317468	78.51920983	103.6553119	68.14953077	100.6688443	87.20552494	49.65002333	31.37864883	71.88261523	52.61575154	66.22077047	40.33805154
	82.47872573	75.49639974	78.75106564	119.344907	64.70207757	105.1712852	58.17150338	78.98756273	97.66528475	58.78881737	91.55574951	102.3783548	74.31798806	89.06649616	113.7150397	32.48963561	107.7896574	102.2037966	38.31551386	37.61728126	90.50840061	78.63844643	68.51407375	97.8398429	110.5498721	59.96072442	33.18786821	94.6977962	27.60200742	51.53829369	59.02247436
	54.70527763	49.1423423	67.2311001	93.374613	105.9236326	66.99548358	25.78171023	62.44903145	82.26121308	49.25323138	60.08339783	63.04043986	72.10954727	93.71409904	113.8493292	45.00248345	56.83065159	76.93853742	20.10788583	34.06142793	78.52794751	42.7477389	61.08139951	93.01745348	98.02629788	54.46501796	44.0599263	90.94752405	50.56541878	73.63034664	56.35013226

AMP3CPR3STR2ERY3TMP1	AMP	CPR	STR	ERY	TMP	AMP+CPR	AMP+STR	AMP+ERY	AMP+TMP	CPR+STR	CPR+ERY	CPR+TMP	STR+ERY	STR+TMP	ERY+TMP	AMP+CPR+STR	AMP+CPR+ERY	AMP+CPR+TMP	AMP+STR+ERY	AMP+STR+TMP	AMP+ERY+TMP	CPR+STR+ERY	CPR+STR+TMP	CPR+ERY+TMP	STR+ERY+TMP	AMP+CPR+STR+ERY	AMP+CPR+STR+TMP	AMP+CPR+ERY+TMP	AMP+STR+ERY+TMP	CPR+STR+ERY+TMP	AMP+CPR+STR+ERY+TMP
	90.7139524	86.40016591	68.10886253	111.7017897	116.1071693	113.278374	11.07481723	111.3703531	94.69590916	76.83932182	98.01420646	74.12246591	50.24424285	60.10058474	100.1800275	18.37507129	86.15129362	80.17835848	49.5670659	41.35428009	72.13148753	103.3234821	60.84927671	93.70041997	69.28315326	42.34976927	40.3587909	58.27759631	41.60315238	82.48042723	34.69694613
	82.47872573	75.49639974	78.75106564	116.0332204	104.4423171	105.1712852	58.17150338	86.84267947	106.5677504	58.78881737	102.7274711	102.0292385	75	102.0247229	121.9942563	32.48963561	99.41086624	86.84267947	45.99607244	53.98210779	98.1889592	92.25398211	63.40824787	91.20663321	105.4347826	48.61444469	39.36286275	93.30133101	44.01047349	56.42592189	25.04909448
	54.70527763	49.1423423	67.2311001	92.05366357	98.65841073	66.99548358	25.78171023	87.87959294	59.78769362	49.25323138	79.59987525	54.87161124	75.94261291	77.81558816	119.4633643	45.00248345	63.92755247	67.77170711	17.446548	50.56541878	85.03343999	48.95752717	45.83415153	92.72174928	80.95173277	31.9914985	32.82316658	90.65181985	64.03844155	66.23774156	39.19928846

AMP3CPR3STR2ERY3TMP2	AMP	CPR	STR	ERY	TMP	AMP+CPR	AMP+STR	AMP+ERY	AMP+TMP	CPR+STR	CPR+ERY	CPR+TMP	STR+ERY	STR+TMP	ERY+TMP	AMP+CPR+STR	AMP+CPR+ERY	AMP+CPR+TMP	AMP+STR+ERY	AMP+STR+TMP	AMP+ERY+TMP	CPR+STR+ERY	CPR+STR+TMP	CPR+ERY+TMP	STR+ERY+TMP	AMP+CPR+STR+ERY	AMP+CPR+STR+TMP	AMP+CPR+ERY+TMP	AMP+STR+ERY+TMP	CPR+STR+ERY+TMP	AMP+CPR+STR+ERY+TMP
	90.7139524	86.40016591	68.10886253	111.7017897	100.8577782	113.278374	11.07481723	111.3703531	94.11520713	76.83932182	98.01420646	94.44703686	50.24424285	64.662993	89.3360161	18.37507129	86.15129362	87.14678281	49.5670659	40.66988127	70.55529631	103.3234821	85.072847	83.08186862	81.29556994	42.34976927	30.38315964	75.44978483	21.77632602	54.93855965	10.49411521
	82.47872573	75.49639974	78.75106564	116.0332204	83.90986003	105.1712852	58.17150338	86.84267947	100.1090988	58.78881737	102.7274711	104.4730526	75	97.25063939	93.84491992	32.48963561	99.41086624	101.1564477	45.99607244	45.25420031	96.4433777	92.25398211	76.54374864	99.2363081	79.85933504	48.61444469	35.87169976	93.9995636	30.04582151	62.01178268	83.11149902
	54.70527763	49.1423423	67.2311001	92.05366357	100.6398349	66.99548358	25.78171023	87.87959294	79.00846684	49.25323138	79.59987525	68.06741132	75.94261291	86.74488879	103.6119711	45.00248345	63.92755247	81.37410047	17.446548	50.32515911	84.44203158	48.95752717	38.14584224	86.80766521	76.63953393	31.9914985	33.8766128	93.60886189	41.39858847	55.59239024	20.56992365

AMP3CPR3STR2ERY3TMP3	AMP	CPR	STR	ERY	TMP	AMP+CPR	AMP+STR	AMP+ERY	AMP+TMP	CPR+STR	CPR+ERY	CPR+TMP	STR+ERY	STR+TMP	ERY+TMP	AMP+CPR+STR	AMP+CPR+ERY	AMP+CPR+TMP	AMP+STR+ERY	AMP+STR+TMP	AMP+ERY+TMP	CPR+STR+ERY	CPR+STR+TMP	CPR+ERY+TMP	STR+ERY+TMP	AMP+CPR+STR+ERY	AMP+CPR+STR+TMP	AMP+CPR+ERY+TMP	AMP+STR+ERY+TMP	CPR+STR+ERY+TMP	AMP+CPR+STR+ERY+TMP
	90.7139524	86.40016591	68.10886253	111.7017897	81.88075823	113.278374	11.07481723	111.3703531	101.0836315	76.83932182	98.01420646	105.0655882	50.24424285	78.2154631	80.5252568	18.37507129	86.15129362	72.79514699	49.5670659	35.71317468	62.84025509	103.3234821	68.14953077	77.44076321	73.96106552	42.34976927	31.37864883	76.86006118	27.39669207	74.18468398	12.1325245
	82.47872573	75.49639974	78.75106564	116.0332204	64.70207757	105.1712852	58.17150338	86.84267947	97.66528475	58.78881737	102.7274711	102.3783548	75	89.06649616	128.6176296	32.48963561	99.41086624	102.2037966	45.99607244	37.61728126	107.9642156	92.25398211	68.51407375	92.60309841	81.22335891	48.61444469	33.18786821	92.77765656	55.18219507	65.85206197	16.08116954
	54.70527763	49.1423423	67.2311001	92.05366357	105.9236326	66.99548358	25.78171023	87.87959294	82.26121308	49.25323138	79.59987525	63.04043986	75.94261291	93.71409904	99.97936017	45.00248345	63.92755247	76.93853742	17.446548	34.06142793	90.35611565	48.95752717	61.08139951	77.93653911	82.69403534	31.9914985	44.0599263	90.94752405	36.96302542	55.59239024	42.87710949

AMP3CPR3STR3ERY1TMP1	AMP	CPR	STR	ERY	TMP	AMP+CPR	AMP+STR	AMP+ERY	AMP+TMP	CPR+STR	CPR+ERY	CPR+TMP	STR+ERY	STR+TMP	ERY+TMP	AMP+CPR+STR	AMP+CPR+ERY	AMP+CPR+TMP	AMP+STR+ERY	AMP+STR+TMP	AMP+ERY+TMP	CPR+STR+ERY	CPR+STR+TMP	CPR+ERY+TMP	STR+ERY+TMP	AMP+CPR+STR+ERY	AMP+CPR+STR+TMP	AMP+CPR+ERY+TMP	AMP+STR+ERY+TMP	CPR+STR+ERY+TMP	AMP+CPR+STR+ERY+TMP
	90.7139524	86.40016591	74.20542334	101.535529	116.1071693	113.278374	51.55804428	82.41820916	94.69590916	85.072847	105.6462902	74.12246591	97.01871727	108.9645875	91.03039288	67.15404158	102.6598227	80.17835848	51.22621455	74.45429564	73.45880645	103.3234821	104.6508011	94.69590916	109.2964173	77.44076321	38.36781252	97.01871727	64.49940374	78.4362524	75.1179551
	82.47872573	75.49639974	60.83351516	110.4033531	104.4423171	105.1712852	44.77416539	86.31900502	106.5677504	88.76281911	92.25398211	102.0292385	81.43137683	106.9168667	89.87089597	45.47239799	94.52323805	86.84267947	52.80384028	83.17695832	102.0292385	86.66812132	100.6327733	113.5500764	116.6921231	45.47239799	58.21514292	94.6977962	74.79816714	79.33667903	72.70346934
	54.70527763	49.1423423	64.81466508	91.0629515	98.65841073	66.99548358	21.45703626	53.09738602	59.78769362	60.1942869	52.09938433	54.87161124	42.93255403	51.80368013	112.5283798	39.67980779	57.82865328	67.77170711	31.51097917	43.81966664	69.36111721	51.91456921	56.35013226	78.52794751	73.79668026	33.5809086	42.1563305	62.26421633	45.11337253	73.79668026	54.28020283

AMP3CPR3STR3ERY1TMP2	AMP	CPR	STR	ERY	TMP	AMP+CPR	AMP+STR	AMP+ERY	AMP+TMP	CPR+STR	CPR+ERY	CPR+TMP	STR+ERY	STR+TMP	ERY+TMP	AMP+CPR+STR	AMP+CPR+ERY	AMP+CPR+TMP	AMP+STR+ERY	AMP+STR+TMP	AMP+ERY+TMP	CPR+STR+ERY	CPR+STR+TMP	CPR+ERY+TMP	STR+ERY+TMP	AMP+CPR+STR+ERY	AMP+CPR+STR+TMP	AMP+CPR+ERY+TMP	AMP+STR+ERY+TMP	CPR+STR+ERY+TMP	AMP+CPR+STR+ERY+TMP
	90.7139524	86.40016591	74.20542334	101.535529	100.8577782	113.278374	51.55804428	82.41820916	94.11520713	85.072847	105.6462902	94.44703686	97.01871727	90.7139524	92.04701896	67.15404158	102.6598227	87.14678281	51.22621455	58.85829834	67.90065847	103.3234821	104.9826308	92.04127132	108.6327578	77.44076321	31.98009022	100.0051848	33.63923887	88.05931456	61.51293617
	82.47872573	75.49639974	60.83351516	110.4033531	83.90986003	105.1712852	44.77416539	86.31900502	100.1090988	88.76281911	92.25398211	104.4730526	81.43137683	104.1239363	104.1111485	45.47239799	94.52323805	101.1564477	52.80384028	66.07025966	85.09709797	86.66812132	98.8871918	105.8695178	116.6921231	45.47239799	52.80384028	98.5380755	71.30700415	97.8398429	76.54374864
	54.70527763	49.1423423	64.81466508	91.0629515	100.6398349	66.99548358	21.45703626	53.09738602	79.00846684	60.1942869	52.09938433	68.06741132	42.93255403	58.12435748	107.244582	39.67980779	57.82865328	81.37410047	31.51097917	41.74973722	67.77170711	51.91456921	53.09738602	71.72675083	80.00646853	33.5809086	48.66182297	71.43104663	50.43604819	79.11935592	76.16231389

AMP3CPR3STR3ERY1TMP3	AMP	CPR	STR	ERY	TMP	AMP+CPR	AMP+STR	AMP+ERY	AMP+TMP	CPR+STR	CPR+ERY	CPR+TMP	STR+ERY	STR+TMP	ERY+TMP	AMP+CPR+STR	AMP+CPR+ERY	AMP+CPR+TMP	AMP+STR+ERY	AMP+STR+TMP	AMP+ERY+TMP	CPR+STR+ERY	CPR+STR+TMP	CPR+ERY+TMP	STR+ERY+TMP	AMP+CPR+STR+ERY	AMP+CPR+STR+TMP	AMP+CPR+ERY+TMP	AMP+STR+ERY+TMP	CPR+STR+ERY+TMP	AMP+CPR+STR+ERY+TMP
	90.7139524	86.40016591	74.20542334	101.535529	81.88075823	113.278374	51.55804428	82.41820916	101.0836315	85.072847	105.6462902	105.0655882	97.01871727	91.4605693	100.8577782	67.15404158	102.6598227	72.79514699	51.22621455	53.21719293	81.7545497	103.3234821	91.37761186	101.6643335	107.9690984	77.44076321	47.90791725	89.38663348	49.5670659	82.41820916	55.87183077
	82.47872573	75.49639974	60.83351516	110.4033531	64.70207757	105.1712852	44.77416539	86.31900502	97.66528475	88.76281911	92.25398211	102.3783548	81.43137683	102.0292385	102.7864738	45.47239799	94.52323805	102.2037966	52.80384028	58.04058477	80.03491163	86.66812132	105.1712852	111.4553786	109.3606808	45.47239799	65.37202706	85.27165612	61.88086406	96.4433777	65.02291076
	54.70527763	49.1423423	64.81466508	91.0629515	105.9236326	66.99548358	21.45703626	53.09738602	82.26121308	60.1942869	52.09938433	63.04043986	42.93255403	70.72874915	113.5190918	39.67980779	57.82865328	76.93853742	31.51097917	47.36811708	67.47600291	51.91456921	66.10837097	71.13534243	75.57090548	33.5809086	48.95752717	81.96550888	54.87161124	74.38808867	62.85562473

AMP3CPR3STR3ERY2TMP1	AMP	CPR	STR	ERY	TMP	AMP+CPR	AMP+STR	AMP+ERY	AMP+TMP	CPR+STR	CPR+ERY	CPR+TMP	STR+ERY	STR+TMP	ERY+TMP	AMP+CPR+STR	AMP+CPR+ERY	AMP+CPR+TMP	AMP+STR+ERY	AMP+STR+TMP	AMP+ERY+TMP	CPR+STR+ERY	CPR+STR+TMP	CPR+ERY+TMP	STR+ERY+TMP	AMP+CPR+STR+ERY	AMP+CPR+STR+TMP	AMP+CPR+ERY+TMP	AMP+STR+ERY+TMP	CPR+STR+ERY+TMP	AMP+CPR+STR+ERY+TMP
	90.7139524	86.40016591	74.20542334	104.2465318	116.1071693	113.278374	51.55804428	97.1016747	94.69590916	85.072847	106.9736092	74.12246591	97.68237673	108.9645875	88.99714074	67.15404158	100.0881423	80.17835848	68.56431793	74.45429564	79.43174159	103.9871416	104.6508011	101.000674	103.9871416	67.81770104	38.36781252	54.87634158	46.58059833	91.37761186	52.8853632
	82.47872573	75.49639974	60.83351516	119.344907	104.4423171	105.1712852	44.77416539	78.98756273	106.5677504	88.76281911	91.55574951	102.0292385	88.76281911	106.9168667	95.50076324	45.47239799	107.7896574	86.84267947	59.08793367	83.17695832	80.73314423	88.76281911	100.6327733	110.757146	111.1062623	79.16212088	58.21514292	84.22430722	64.67379446	106.9168667	62.22998036
	54.70527763	49.1423423	64.81466508	93.374613	98.65841073	66.99548358	21.45703626	62.44903145	59.78769362	60.1942869	60.08339783	54.87161124	64.81466508	51.80368013	113.1888545	39.67980779	56.83065159	67.77170711	37.90558257	43.81966664	84.62684671	43.33914731	56.35013226	80.19128366	73.09438278	37.90558257	42.1563305	66.8845945	61.96851212	85.32914419	61.96851212

AMP3CPR3STR3ERY2TMP2	AMP	CPR	STR	ERY	TMP	AMP+CPR	AMP+STR	AMP+ERY	AMP+TMP	CPR+STR	CPR+ERY	CPR+TMP	STR+ERY	STR+TMP	ERY+TMP	AMP+CPR+STR	AMP+CPR+ERY	AMP+CPR+TMP	AMP+STR+ERY	AMP+STR+TMP	AMP+ERY+TMP	CPR+STR+ERY	CPR+STR+TMP	CPR+ERY+TMP	STR+ERY+TMP	AMP+CPR+STR+ERY	AMP+CPR+STR+TMP	AMP+CPR+ERY+TMP	AMP+STR+ERY+TMP	CPR+STR+ERY+TMP	AMP+CPR+STR+ERY+TMP
	90.7139524	86.40016591	74.20542334	104.2465318	100.8577782	113.278374	51.55804428	97.1016747	94.11520713	85.072847	106.9736092	94.44703686	97.68237673	90.7139524	96.79127396	67.15404158	100.0881423	87.14678281	68.56431793	58.85829834	84.49214497	103.9871416	104.9826308	90.7139524	106.6417794	67.81770104	31.98009022	69.47684969	31.98009022	89.38663348	32.31191995
	82.47872573	75.49639974	60.83351516	119.344907	83.90986003	105.1712852	44.77416539	78.98756273	100.1090988	88.76281911	91.55574951	104.4730526	88.76281911	104.1239363	104.7734858	45.47239799	107.7896574	101.1564477	59.08793367	66.07025966	89.63560986	88.76281911	98.8871918	103.0765874	106.5677504	79.16212088	52.80384028	83.87519092	56.99323587	102.7274711	69.56142265
	54.70527763	49.1423423	64.81466508	93.374613	100.6398349	66.99548358	21.45703626	62.44903145	79.00846684	60.1942869	60.08339783	68.06741132	64.81466508	58.12435748	111.8679051	39.67980779	56.83065159	81.37410047	37.90558257	41.74973722	88.17529715	43.33914731	53.09738602	77.6408349	77.93653911	37.90558257	48.66182297	73.09438278	32.58290691	78.23224331	63.44703314

AMP3CPR3STR3ERY2TMP3	AMP	CPR	STR	ERY	TMP	AMP+CPR	AMP+STR	AMP+ERY	AMP+TMP	CPR+STR	CPR+ERY	CPR+TMP	STR+ERY	STR+TMP	ERY+TMP	AMP+CPR+STR	AMP+CPR+ERY	AMP+CPR+TMP	AMP+STR+ERY	AMP+STR+TMP	AMP+ERY+TMP	CPR+STR+ERY	CPR+STR+TMP	CPR+ERY+TMP	STR+ERY+TMP	AMP+CPR+STR+ERY	AMP+CPR+STR+TMP	AMP+CPR+ERY+TMP	AMP+STR+ERY+TMP	CPR+STR+ERY+TMP	AMP+CPR+STR+ERY+TMP
	90.7139524	86.40016591	74.20542334	104.2465318	81.88075823	113.278374	51.55804428	97.1016747	101.0836315	85.072847	106.9736092	105.0655882	97.68237673	91.4605693	100.1800275	67.15404158	100.0881423	72.79514699	68.56431793	53.21719293	78.51920983	103.9871416	91.37761186	100.6688443	107.6372686	67.81770104	47.90791725	71.88261523	44.25779022	89.05480375	55.20817131
	82.47872573	75.49639974	60.83351516	119.344907	64.70207757	105.1712852	44.77416539	78.98756273	97.66528475	88.76281911	91.55574951	102.3783548	88.76281911	102.0292385	113.7150397	45.47239799	107.7896574	102.2037966	59.08793367	58.04058477	90.50840061	88.76281911	105.1712852	97.8398429	103.0765874	79.16212088	65.37202706	94.6977962	65.37202706	94.3486799	66.07025966
	54.70527763	49.1423423	64.81466508	93.374613	105.9236326	66.99548358	21.45703626	62.44903145	82.26121308	60.1942869	60.08339783	63.04043986	64.81466508	70.72874915	113.8493292	39.67980779	56.83065159	76.93853742	37.90558257	47.36811708	78.52794751	43.33914731	66.10837097	93.01745348	88.58189043	37.90558257	48.95752717	90.94752405	64.03844155	80.89358114	84.73773578

AMP3CPR3STR3ERY3TMP1	AMP	CPR	STR	ERY	TMP	AMP+CPR	AMP+STR	AMP+ERY	AMP+TMP	CPR+STR	CPR+ERY	CPR+TMP	STR+ERY	STR+TMP	ERY+TMP	AMP+CPR+STR	AMP+CPR+ERY	AMP+CPR+TMP	AMP+STR+ERY	AMP+STR+TMP	AMP+ERY+TMP	CPR+STR+ERY	CPR+STR+TMP	CPR+ERY+TMP	STR+ERY+TMP	AMP+CPR+STR+ERY	AMP+CPR+STR+TMP	AMP+CPR+ERY+TMP	AMP+STR+ERY+TMP	CPR+STR+ERY+TMP	AMP+CPR+STR+ERY+TMP
	90.7139524	86.40016591	74.20542334	111.7017897	116.1071693	113.278374	51.55804428	111.3703531	94.69590916	85.072847	98.01420646	74.12246591	92.37310105	108.9645875	100.1800275	67.15404158	86.15129362	80.17835848	69.22797739	74.45429564	72.13148753	100.3370146	104.6508011	93.70041997	100.6688443	56.61844766	38.36781252	58.27759631	29.32545238	67.48587131	51.55804428
	82.47872573	75.49639974	60.83351516	116.0332204	104.4423171	105.1712852	44.77416539	86.84267947	106.5677504	88.76281911	102.7274711	102.0292385	95.0469125	106.9168667	121.9942563	45.47239799	99.41086624	86.84267947	59.96072442	83.17695832	98.1889592	86.31900502	100.6327733	91.20663321	105.8695178	60.65895702	58.21514292	93.30133101	69.21230635	76.54374864	63.62644556
	54.70527763	49.1423423	64.81466508	92.05366357	98.65841073	66.99548358	21.45703626	87.87959294	59.78769362	60.1942869	79.59987525	54.87161124	67.47600291	51.80368013	119.4633643	39.67980779	63.92755247	67.77170711	50.32515911	43.81966664	85.03343999	46.18530027	56.35013226	92.72174928	84.14632738	46.77670867	42.1563305	90.65181985	104.8456216	93.31315768	71.72675083

AMP3CPR3STR3ERY3TMP2	AMP	CPR	STR	ERY	TMP	AMP+CPR	AMP+STR	AMP+ERY	AMP+TMP	CPR+STR	CPR+ERY	CPR+TMP	STR+ERY	STR+TMP	ERY+TMP	AMP+CPR+STR	AMP+CPR+ERY	AMP+CPR+TMP	AMP+STR+ERY	AMP+STR+TMP	AMP+ERY+TMP	CPR+STR+ERY	CPR+STR+TMP	CPR+ERY+TMP	STR+ERY+TMP	AMP+CPR+STR+ERY	AMP+CPR+STR+TMP	AMP+CPR+ERY+TMP	AMP+STR+ERY+TMP	CPR+STR+ERY+TMP	AMP+CPR+STR+ERY+TMP
	90.7139524	86.40016591	74.20542334	111.7017897	100.8577782	113.278374	51.55804428	111.3703531	94.11520713	85.072847	98.01420646	94.44703686	92.37310105	90.7139524	89.3360161	67.15404158	86.15129362	87.14678281	69.22797739	58.85829834	70.55529631	100.3370146	104.9826308	83.08186862	74.45429564	56.61844766	31.98009022	75.44978483	36.62570643	68.14953077	49.23523617
	82.47872573	75.49639974	60.83351516	116.0332204	83.90986003	105.1712852	44.77416539	86.84267947	100.1090988	88.76281911	102.7274711	104.4730526	95.0469125	104.1239363	93.84491992	45.47239799	99.41086624	101.1564477	59.96072442	66.07025966	96.4433777	86.31900502	98.8871918	99.2363081	92.95221471	60.65895702	52.80384028	93.9995636	65.02291076	98.5380755	43.0285839
	54.70527763	49.1423423	64.81466508	92.05366357	100.6398349	66.99548358	21.45703626	87.87959294	79.00846684	60.1942869	79.59987525	68.06741132	67.47600291	58.12435748	103.6119711	39.67980779	63.92755247	81.37410047	50.32515911	41.74973722	84.44203158	46.18530027	53.09738602	86.80766521	85.03343999	46.77670867	48.66182297	93.60886189	57.53294907	92.72174928	82.96351056

AMP3CPR3STR3ERY3TMP3	AMP	CPR	STR	ERY	TMP	AMP+CPR	AMP+STR	AMP+ERY	AMP+TMP	CPR+STR	CPR+ERY	CPR+TMP	STR+ERY	STR+TMP	ERY+TMP	AMP+CPR+STR	AMP+CPR+ERY	AMP+CPR+TMP	AMP+STR+ERY	AMP+STR+TMP	AMP+ERY+TMP	CPR+STR+ERY	CPR+STR+TMP	CPR+ERY+TMP	STR+ERY+TMP	AMP+CPR+STR+ERY	AMP+CPR+STR+TMP	AMP+CPR+ERY+TMP	AMP+STR+ERY+TMP	CPR+STR+ERY+TMP	AMP+CPR+STR+ERY+TMP
	90.7139524	86.40016591	74.20542334	111.7017897	81.88075823	113.278374	51.55804428	111.3703531	101.0836315	85.072847	98.01420646	105.0655882	92.37310105	91.4605693	80.5252568	67.15404158	86.15129362	72.79514699	69.22797739	53.21719293	62.84025509	100.3370146	91.37761186	77.44076321	91.70944159	56.61844766	47.90791725	76.86006118	32.31191995	53.88085239	29.71950018
	82.47872573	75.49639974	60.83351516	116.0332204	64.70207757	105.1712852	44.77416539	86.84267947	97.66528475	88.76281911	102.7274711	102.3783548	95.0469125	102.0292385	128.6176296	45.47239799	99.41086624	102.2037966	59.96072442	58.04058477	107.9642156	86.31900502	105.1712852	92.60309841	98.1889592	60.65895702	65.37202706	92.77765656	74.09993454	76.54374864	38.4246127
	54.70527763	49.1423423	64.81466508	92.05366357	105.9236326	66.99548358	21.45703626	87.87959294	82.26121308	60.1942869	79.59987525	63.04043986	67.47600291	70.72874915	99.97936017	39.67980779	63.92755247	76.93853742	50.32515911	47.36811708	90.35611565	46.18530027	66.10837097	77.93653911	77.3451307	46.77670867	48.95752717	90.94752405	71.43104663	82.07639795	74.97949707

AMP1CPR1STR1FUS1DOX1	AMP	CPR	STR	FUS	DOX	AMP+CPR	AMP+STR	AMP+FUS	AMP+DOX	CPR+STR	CPR+FUS	CPR+DOX	STR+FUS	STR+DOX	FUS+DOX	AMP+CPR+STR	AMP+CPR+FUS	AMP+CPR+DOX	AMP+STR+FUS	AMP+STR+DOX	AMP+FUS+DOX	CPR+STR+FUS	CPR+STR+DOX	CPR+FUS+DOX	STR+FUS+DOX	AMP+CPR+STR+FUS	AMP+CPR+STR+DOX	AMP+CPR+FUS+DOX	AMP+STR+FUS+DOX	CPR+STR+FUS+DOX	AMP+CPR+STR+FUS+DOX
	89.79762502	93.00886436	63.30490049	91.26944305	85.78357585	73.20622178	10.72085633	41.34470647	89.79762502	67.31894966	82.97374143	86.85398896	70.93159391	84.98076601	86.18498077	10.58705469	33.73473825	99.16373976	5.368790768	6.43920388	27.69693929	18.59842783	72.67101522	85.38217093	83.77655126	0.669008195	41.62903496	15.92239505	8.847633384	35.72503763	6.840608797
	77.50142126	90.9181353	76.59181353	55.15918135	103.8800455	79.09323479	14.51108584	16.33030131	69.94030699	69.31495168	55.61398522	90.23592951	46.74530984	95.0113701	62.43604321	25.35531552	20.36668562	101.8334281	4.960204662	16.10289937	10.64525298	20.19613417	66.13132462	63.57305287	63.8004548	4.675952246	39.0704496	18.83172257	7.234223991	9.280841387	6.779420125
	85.36160325	91.17049085	73.27911705	59.57014232	97.90880046	72.34969503	11.007842	15.17571885	80.24978217	67.70258495	68.90792913	93.49404589	61.47255301	91.17049085	65.42259657	30.99041534	35.39064769	91.63520186	2.686610514	16.58437409	31.44060412	19.18385129	70.2584955	65.42259657	63.56375254	3.61603253	27.27272727	26.09642753	4.313099042	10.29625327	4.255010166

AMP1CPR1STR1FUS1DOX2	AMP	CPR	STR	FUS	DOX	AMP+CPR	AMP+STR	AMP+FUS	AMP+DOX	CPR+STR	CPR+FUS	CPR+DOX	STR+FUS	STR+DOX	FUS+DOX	AMP+CPR+STR	AMP+CPR+FUS	AMP+CPR+DOX	AMP+STR+FUS	AMP+STR+DOX	AMP+FUS+DOX	CPR+STR+FUS	CPR+STR+DOX	CPR+FUS+DOX	STR+FUS+DOX	AMP+CPR+STR+FUS	AMP+CPR+STR+DOX	AMP+CPR+FUS+DOX	AMP+STR+FUS+DOX	CPR+STR+FUS+DOX	AMP+CPR+STR+FUS+DOX
	89.79762502	93.00886436	63.30490049	91.26944305	92.74126108	73.20622178	10.72085633	41.34470647	88.45960863	67.31894966	82.97374143	88.99481519	70.93159391	69.1921726	94.74828567	10.58705469	33.73473825	107.7270447	5.368790768	12.46027764	32.66432514	18.59842783	66.51613982	91.26944305	90.73423649	0.669008195	41.36143168	28.38267269	5.368790768	10.18564977	2.960361264
	77.50142126	90.9181353	76.59181353	55.15918135	104.7896532	79.09323479	14.51108584	16.33030131	75.45480387	69.31495168	55.61398522	87.05230244	46.74530984	85.23308698	63.8004548	25.35531552	20.36668562	95.6935759	4.960204662	21.1057419	18.3769187	20.19613417	68.86014781	69.48550313	61.29903354	4.675952246	51.35019898	31.9641842	2.913587265	9.053439454	11.78226265
	85.36160325	91.17049085	73.27911705	59.57014232	96.51466744	72.34969503	11.007842	15.17571885	69.83735115	67.70258495	68.90792913	92.56462387	61.47255301	83.73511473	63.79610805	30.99041534	35.39064769	93.9587569	2.686610514	31.91983735	37.94655823	19.18385129	63.98489689	69.37264014	66.35201859	3.61603253	45.86116759	35.62300319	8.96020912	5.416787685	16.56985187

AMP1CPR1STR1FUS1DOX3	AMP	CPR	STR	FUS	DOX	AMP+CPR	AMP+STR	AMP+FUS	AMP+DOX	CPR+STR	CPR+FUS	CPR+DOX	STR+FUS	STR+DOX	FUS+DOX	AMP+CPR+STR	AMP+CPR+FUS	AMP+CPR+DOX	AMP+STR+FUS	AMP+STR+DOX	AMP+FUS+DOX	CPR+STR+FUS	CPR+STR+DOX	CPR+FUS+DOX	STR+FUS+DOX	AMP+CPR+STR+FUS	AMP+CPR+STR+DOX	AMP+CPR+FUS+DOX	AMP+STR+FUS+DOX	CPR+STR+FUS+DOX	AMP+CPR+STR+FUS+DOX
	89.79762502	93.00886436	63.30490049	91.26944305	93.27646764	73.20622178	10.72085633	41.34470647	82.83993979	67.31894966	82.97374143	95.14969058	70.93159391	74.54423817	89.93142666	10.58705469	33.73473825	116.5579528	5.368790768	11.12226125	38.55159726	18.59842783	67.85415621	87.25539388	81.63572504	0.669008195	50.19233986	31.71098846	7.242013715	30.25589563	0.819535039
	77.50142126	90.9181353	76.59181353	55.15918135	95.0113701	79.09323479	14.51108584	16.33030131	81.82205799	69.31495168	55.61398522	90.23592951	46.74530984	85.00568505	67.66628766	25.35531552	20.36668562	104.3348493	4.960204662	22.01534963	16.50085276	20.19613417	70.90676521	70.62251279	68.12109153	4.675952246	64.08470722	28.5531552	3.993746447	12.91927231	6.324616259
	85.36160325	91.17049085	73.27911705	59.57014232	97.67644496	72.34969503	11.007842	15.17571885	74.67325007	67.70258495	68.90792913	95.12053442	61.47255301	85.82631426	77.50508278	30.99041534	35.39064769	96.74702295	2.686610514	15.65495208	22.61109498	19.18385129	68.86436247	70.76677316	76.34330526	3.61603253	52.13476619	47.24077839	6.869009585	11.45803079	4.719721173

AMP1CPR1STR1FUS2DOX1	AMP	CPR	STR	FUS	DOX	AMP+CPR	AMP+STR	AMP+FUS	AMP+DOX	CPR+STR	CPR+FUS	CPR+DOX	STR+FUS	STR+DOX	FUS+DOX	AMP+CPR+STR	AMP+CPR+FUS	AMP+CPR+DOX	AMP+STR+FUS	AMP+STR+DOX	AMP+FUS+DOX	CPR+STR+FUS	CPR+STR+DOX	CPR+FUS+DOX	STR+FUS+DOX	AMP+CPR+STR+FUS	AMP+CPR+STR+DOX	AMP+CPR+FUS+DOX	AMP+STR+FUS+DOX	CPR+STR+FUS+DOX	AMP+CPR+STR+FUS+DOX
	89.79762502	93.00886436	63.30490049	82.03712995	85.78357585	73.20622178	10.72085633	62.48536545	89.79762502	67.31894966	74.81184145	86.85398896	81.36812176	84.98076601	74.67803981	10.58705469	41.76283659	99.16373976	3.227964543	6.43920388	60.07693594	57.01622345	72.67101522	72.53721358	77.08646931	21.00685733	41.62903496	56.59809333	2.274627864	32.78140157	14.31677538
	77.50142126	90.9181353	76.59181353	95.6935759	103.8800455	79.09323479	14.51108584	59.53666856	69.94030699	69.31495168	85.91529278	90.23592951	95.6935759	95.0113701	66.30187607	25.35531552	30.82717453	101.8334281	7.234223991	16.10289937	50.44059125	32.64638999	66.13132462	62.26549176	63.8004548	1.947129051	39.0704496	49.01932916	6.779420125	25.88118249	12.00966458
	85.36160325	91.17049085	73.27911705	95.81760093	97.90880046	72.34969503	11.007842	60.94975312	80.24978217	67.70258495	85.12924775	93.49404589	100	91.17049085	76.80801626	30.99041534	42.36131281	91.63520186	2.918966018	16.58437409	40.96717979	48.46064479	70.2584955	68.44321812	75.41388324	16.56985187	27.27272727	47.47313389	5.707232065	18.1963404	4.022654662

AMP1CPR1STR1FUS2DOX2	AMP	CPR	STR	FUS	DOX	AMP+CPR	AMP+STR	AMP+FUS	AMP+DOX	CPR+STR	CPR+FUS	CPR+DOX	STR+FUS	STR+DOX	FUS+DOX	AMP+CPR+STR	AMP+CPR+FUS	AMP+CPR+DOX	AMP+STR+FUS	AMP+STR+DOX	AMP+FUS+DOX	CPR+STR+FUS	CPR+STR+DOX	CPR+FUS+DOX	STR+FUS+DOX	AMP+CPR+STR+FUS	AMP+CPR+STR+DOX	AMP+CPR+FUS+DOX	AMP+STR+FUS+DOX	CPR+STR+FUS+DOX	AMP+CPR+STR+FUS+DOX
	89.79762502	93.00886436	63.30490049	82.03712995	92.74126108	73.20622178	10.72085633	62.48536545	88.45960863	67.31894966	74.81184145	88.99481519	81.36812176	69.1921726	67.72035457	10.58705469	41.76283659	107.7270447	3.227964543	12.46027764	56.59809333	57.01622345	66.51613982	76.68506439	64.77671851	21.00685733	41.36143168	64.24151196	6.823883593	42.41511959	40.27429336
	77.50142126	90.9181353	76.59181353	95.6935759	104.7896532	79.09323479	14.51108584	59.53666856	75.45480387	69.31495168	85.91529278	87.05230244	95.6935759	85.23308698	58.11540648	25.35531552	30.82717453	95.6935759	7.234223991	21.1057419	47.71176805	32.64638999	68.86014781	67.26833428	53.56736782	1.947129051	51.35019898	43.33428084	6.779420125	34.06765208	7.91642979
	85.36160325	91.17049085	73.27911705	95.81760093	96.51466744	72.34969503	11.007842	60.94975312	69.83735115	67.70258495	85.12924775	92.56462387	100	83.73511473	72.8579727	30.99041534	42.36131281	93.9587569	2.918966018	31.91983735	20.34562881	48.46064479	63.98489689	72.39326169	70.76677316	16.56985187	45.86116759	53.99361022	2.686610514	38.70171362	4.719721173

AMP1CPR1STR1FUS2DOX3	AMP	CPR	STR	FUS	DOX	AMP+CPR	AMP+STR	AMP+FUS	AMP+DOX	CPR+STR	CPR+FUS	CPR+DOX	STR+FUS	STR+DOX	FUS+DOX	AMP+CPR+STR	AMP+CPR+FUS	AMP+CPR+DOX	AMP+STR+FUS	AMP+STR+DOX	AMP+FUS+DOX	CPR+STR+FUS	CPR+STR+DOX	CPR+FUS+DOX	STR+FUS+DOX	AMP+CPR+STR+FUS	AMP+CPR+STR+DOX	AMP+CPR+FUS+DOX	AMP+STR+FUS+DOX	CPR+STR+FUS+DOX	AMP+CPR+STR+FUS+DOX
	89.79762502	93.00886436	63.30490049	82.03712995	93.27646764	73.20622178	10.72085633	62.48536545	82.83993979	67.31894966	74.81184145	95.14969058	81.36812176	74.54423817	112.0086971	10.58705469	41.76283659	116.5579528	3.227964543	11.12226125	90.86803813	57.01622345	67.85415621	111.7410938	94.34688075	21.00685733	50.19233986	79.62870045	5.502592407	58.08663656	39.75581201
	77.50142126	90.9181353	76.59181353	95.6935759	95.0113701	79.09323479	14.51108584	59.53666856	81.82205799	69.31495168	85.91529278	90.23592951	95.6935759	85.00568505	97.96759522	25.35531552	30.82717453	104.3348493	7.234223991	22.01534963	84.09607732	32.64638999	70.90676521	94.78396816	35.14781126	1.947129051	64.08470722	61.52643547	8.143831723	49.9289369	19.7413303

	85.36160325	91.17049085	73.27911705	95.81760093	97.67644496	72.34969503	11.007842	60.94975312	74.67325007	67.70258495	85.12924775	95.12053442	100	85.82631426	94.42346791	30.99041534	42.36131281	96.74702295	2.918966018	15.65495208	71.18791751	48.46064479	68.86436247	80.71449317	59.14899797	16.56985187	52.13476619	67.00551844	1.524832994	57.05779843	25.41388324
AMP1CPR1STR1FUS3DOX1	AMP	CPR	STR	FUS	DOX	AMP+CPR	AMP+STR	AMP+FUS	AMP+DOX	CPR+STR	CPR+FUS	CPR+DOX	STR+FUS	STR+DOX	FUS+DOX	AMP+CPR+STR	AMP+CPR+FUS	AMP+CPR+DOX	AMP+STR+FUS	AMP+STR+DOX	AMP+FUS+DOX	CPR+STR+FUS	CPR+STR+DOX	CPR+FUS+DOX	STR+FUS+DOX	AMP+CPR+STR+FUS	AMP+CPR+STR+DOX	AMP+CPR+FUS+DOX	AMP+STR+FUS+DOX	CPR+STR+FUS+DOX	AMP+CPR+STR+FUS+DOX
	89.79762502	93.00886436	63.30490049	78.82589062	85.78357585	73.20622178	10.72085633	78.69208898	89.79762502	67.31894966	78.82589062	86.85398896	71.86820539	84.98076601	109.8678709	10.58705469	53.26977755	99.16373976	7.091486871	6.43920388	93.81167419	55.1430005	72.67101522	112.5439037	110.4030774	14.33350059	41.62903496	98.09332664	10.85465797	58.08663656	52.33316608
	77.50142126	90.9181353	76.59181353	99.55940875	103.8800455	79.09323479	14.51108584	63.62990335	69.94030699	69.31495168	86.59749858	90.23592951	90.69073337	95.0113701	97.05798749	25.35531552	60.44627629	101.8334281	18.14951677	16.10289937	72.72598067	63.17509949	66.13132462	92.50994883	94.78396816	20.87833997	39.0704946	68.80329733	10.64525298	48.10972143	24.74417283
	85.36160325	91.17049085	73.27911705	93.49404589	97.90880046	72.34969503	11.007842	55.89602091	80.24978217	67.70258495	84.19982573	93.49404589	89.54400232	91.17049085	88.84693581	30.99041534	59.38135347	91.63520186	19.60499564	16.58437409	50.74063317	64.91431891	70.2584955	84.19982573	86.29102527	24.25210572	27.27272727	66.07609643	2.45425501	56.36073192	37.49636945
AMP1CPR1STR1FUS3DOX2	AMP	CPR	STR	FUS	DOX	AMP+CPR	AMP+STR	AMP+FUS	AMP+DOX	CPR+STR	CPR+FUS	CPR+DOX	STR+FUS	STR+DOX	FUS+DOX	AMP+CPR+STR	AMP+CPR+FUS	AMP+CPR+DOX	AMP+STR+FUS	AMP+STR+DOX	AMP+FUS+DOX	CPR+STR+FUS	CPR+STR+DOX	CPR+FUS+DOX	STR+FUS+DOX	AMP+CPR+STR+FUS	AMP+CPR+STR+DOX	AMP+CPR+FUS+DOX	AMP+STR+FUS+DOX	CPR+STR+FUS+DOX	AMP+CPR+STR+FUS+DOX
	89.79762502	93.00886436	63.30490049	78.82589062	92.74126108	73.20622178	10.72085633	78.69208898	88.45960863	67.31894966	78.82589062	88.99481519	71.86820539	69.1921726	101.5721693	10.58705469	53.26977755	107.7270447	7.091486871	12.46027764	74.54423817	55.1430005	66.51613982	102.6425824	96.75531025	14.33350059	41.36143168	85.24836929	10.31945141	45.8939622	41.89663823
	77.50142126	90.9181353	76.59181353	99.55940875	104.7896532	79.09323479	14.51108584	63.62990335	75.45480387	69.31495168	86.59749858	87.05230244	90.69073337	85.23308698	91.14553724	25.35531552	60.44627629	95.6935759	18.14951677	21.1057419	71.36156907	63.17509949	68.86014781	87.27970438	85.46048891	20.87833997	51.35019898	55.6708357	9.50824332	50.15633883	39.01364412
	85.36160325	91.17049085	73.27911705	93.49404589	96.51466744	72.34969503	11.007842	55.89602091	69.83735115	67.70258495	84.19982573	92.56462387	89.54400232	83.73511473	93.49404589	30.99041534	59.38135347	93.9587569	19.60499564	31.91983735	49.81121115	64.91431891	63.98489689	86.52338077	88.61458031	24.25210572	45.86116759	55.66366541	0.130699971	51.01655533	34.47574789
AMP1CPR1STR1FUS3DOX3	AMP	CPR	STR	FUS	DOX	AMP+CPR	AMP+STR	AMP+FUS	AMP+DOX	CPR+STR	CPR+FUS	CPR+DOX	STR+FUS	STR+DOX	FUS+DOX	AMP+CPR+STR	AMP+CPR+FUS	AMP+CPR+DOX	AMP+STR+FUS	AMP+STR+DOX	AMP+FUS+DOX	CPR+STR+FUS	CPR+STR+DOX	CPR+FUS+DOX	STR+FUS+DOX	AMP+CPR+STR+FUS	AMP+CPR+STR+DOX	AMP+CPR+FUS+DOX	AMP+STR+FUS+DOX	CPR+STR+FUS+DOX	AMP+CPR+STR+FUS+DOX
	89.79762502	93.00886436	63.30490049	78.82589062	93.27646764	73.20622178	10.72085633	78.69208898	82.83993979	67.31894966	78.82589062	95.14969058	71.86820539	74.54423817	98.36092992	10.58705469	53.26977755	116.5579528	7.091486871	11.12226125	73.47382505	55.1430005	67.85415621	98.6285332	90.60043486	14.33350059	50.19233986	77.48787423	15.92239505	57.81903328	25.57283827
	77.50142126	90.9181353	76.59181353	99.55940875	95.0113701	79.09323479	14.51108584	63.62990335	81.82205799	69.31495168	86.59749858	90.23592951	90.69073337	85.00568505	95.6935759	25.35531552	60.44627629	104.3348493	18.14951677	22.01534963	65.22171688	63.17509949	70.90676521	94.3291643	94.3291643	20.87833997	64.08470722	62.03808982	7.461625924	58.79761228	23.37976123
	85.36160325	91.17049085	73.27911705	93.49404589	97.67644496	72.34969503	11.007842	55.89602091	74.67325007	67.70258495	84.19982573	95.12053442	89.54400232	85.82631426	107.4353761	30.99041534	59.38135347	96.74702295	19.60499564	15.65495208	52.17833285	64.91431891	68.86436247	101.6264885	103.4853326	24.25210572	52.13476619	46.5582341	2.918966018	52.41068835	34.46122568
AMP1CPR1STR2FUS1DOX1	AMP	CPR	STR	FUS	DOX	AMP+CPR	AMP+STR	AMP+FUS	AMP+DOX	CPR+STR	CPR+FUS	CPR+DOX	STR+FUS	STR+DOX	FUS+DOX	AMP+CPR+STR	AMP+CPR+FUS	AMP+CPR+DOX	AMP+STR+FUS	AMP+STR+DOX	AMP+FUS+DOX	CPR+STR+FUS	CPR+STR+DOX	CPR+FUS+DOX	STR+FUS+DOX	AMP+CPR+STR+FUS	AMP+CPR+STR+DOX	AMP+CPR+FUS+DOX	AMP+STR+FUS+DOX	CPR+STR+FUS+DOX	AMP+CPR+STR+FUS+DOX
	89.79762502	93.00886436	84.06610539	91.26944305	85.78357585	73.20622178	18.06322127	41.34470647	89.79762502	59.29085131	82.97374143	86.85398896	54.02245089	77.08138447	86.18498077	5.78692089	33.73473825	99.16373976	3.227964543	11.92507108	27.69693929	6.171600602	59.82605787	85.38217093	64.74898659	0.669008195	53.13597592	15.92239505	4.565980933	10.03512293	1.756146513
	77.50142126	90.9181353	84.06610539	55.15918135	103.8800455	79.09323479	13.82888005	16.33030131	69.94030699	62.94769756	55.61398522	90.23592951	54.02245089	77.08138447	62.43604321	29.44855031	20.36668562	101.8334281	4.505400796	25.42637862	10.64525298	8.371233655	61.81068789	63.57305287	64.74898659	3.766344514	60.90108016	18.83172257	4.732802729	9.280841387	8.598635588
	85.36160325	91.17049085	71.4091527	59.57014232	97.90880046	72.34969503	12.40197502	15.17571885	80.24978217	61.42898635	68.90792913	93.49404589	44.19272013	82.28364295	65.42259657	29.82863782	35.39064769	91.63520186	3.151321522	21.46383967	31.44060412	13.6073192	65.61138542	65.42259657	69.56653074	3.848388034	52.5994772	26.09642753	5.242521057	8.66976474	1.466744119
AMP1CPR1STR2FUS1DOX2	AMP	CPR	STR	FUS	DOX	AMP+CPR	AMP+STR	AMP+FUS	AMP+DOX	CPR+STR	CPR+FUS	CPR+DOX	STR+FUS	STR+DOX	FUS+DOX	AMP+CPR+STR	AMP+CPR+FUS	AMP+CPR+DOX	AMP+STR+FUS	AMP+STR+DOX	AMP+FUS+DOX	CPR+STR+FUS	CPR+STR+DOX	CPR+FUS+DOX	STR+FUS+DOX	AMP+CPR+STR+FUS	AMP+CPR+STR+DOX	AMP+CPR+FUS+DOX	AMP+STR+FUS+DOX	CPR+STR+FUS+DOX	AMP+CPR+STR+FUS+DOX
	89.79762502	93.00886436	84.06610539	91.26944305	92.74126108	73.20622178	18.06322127	41.34470647	88.45960863	59.29085131	82.97374143	88.99481519	54.02245089	86.8101029	94.74828567	5.78692089	33.73473825	107.7270447	3.227964543	11.90834588	32.66432514	6.171600602	52.0655628	91.26944305	69.73807297	0.669008195	35.74176284	28.38267269	6.974410437	5.903997324	2.826559625
	77.50142126	90.9181353	84.06610539	55.15918135	104.7896532	79.09323479	13.82888005	16.33030131	75.45480387	62.94769756	55.61398522	87.05230244	54.02245089	86.8101029	63.8004548	29.44855031	20.36668562	95.6935759	4.505400796	16.55770324	18.3769187	8.371233655	54.9886299	69.48550313	69.73807297	3.766344514	52.25980671	31.9641842	3.823194997	5.64241046	4.732802729
	85.36160325	91.17049085	71.4091527	59.57014232	96.51466744	72.34969503	12.40197502	15.17571885	69.83735115	61.42898635	68.90792913	92.56462387	44.19272013	89.29164779	63.79610805	29.82863782	35.39064769	93.9587569	3.151321522	23.32268371	37.94655823	13.6073192	57.71129829	69.37264014	53.8589337	3.848388034	33.77868138	35.62300319	6.404298577	3.32558815	2.396166134
AMP1CPR1STR2FUS1DOX3	AMP	CPR	STR	FUS	DOX	AMP+CPR	AMP+STR	AMP+FUS	AMP+DOX	CPR+STR	CPR+FUS	CPR+DOX	STR+FUS	STR+DOX	FUS+DOX	AMP+CPR+STR	AMP+CPR+FUS	AMP+CPR+DOX	AMP+STR+FUS	AMP+STR+DOX	AMP+FUS+DOX	CPR+STR+FUS	CPR+STR+DOX	CPR+FUS+DOX	STR+FUS+DOX	AMP+CPR+STR+FUS	AMP+CPR+STR+DOX	AMP+CPR+FUS+DOX	AMP+STR+FUS+DOX	CPR+STR+FUS+DOX	AMP+CPR+STR+FUS+DOX
	89.79762502	93.00886436	84.06610539	91.26944305	93.27646764	73.20622178	18.06322127	41.34470647	82.83993979	59.29085131	82.97374143	95.14969058	54.02245089	87.30901154	89.93142666	5.78692089	33.73473825	116.5579528	3.227964543	6.573005519	38.55159726	6.171600602	57.41762837	87.25539388	65.99625819	0.669008195	59.02324803	31.71098846	7.509616993	6.974410437	1.889948152
	77.50142126	90.9181353	84.06610539	55.15918135	95.0113701	79.09323479	13.82888005	16.33030131	81.82205799	62.94769756	55.61398522	90.23592951	54.02245089	87.30901154	67.66628766	29.44855031	20.36668562	104.3348493	4.505400796	22.69755543	16.50085276	8.371233655	67.49573621	70.62251279	65.99625819	3.766344514	45.43774872	28.5531552	8.996588971	4.05059693	2.174530984
	85.36160325	91.17049085	71.4091527	59.57014232	97.67644496	72.34969503	12.40197502	15.17571885	74.67325007	61.42898635	68.90792913	95.12053442	44.19272013	92.67482253	77.50508278	29.82863782	35.39064769	96.74702295	3.151321522	13.56375254	22.61109498	13.6073192	62.35840836	70.76677316	64.25011328	3.848388034	51.90241069	47.24077839	2.45425501	8.495498112	4.719721173
AMP1CPR1STR2FUS2DOX1	AMP	CPR	STR	FUS	DOX	AMP+CPR	AMP+STR	AMP+FUS	AMP+DOX	CPR+STR	CPR+FUS	CPR+DOX	STR+FUS	STR+DOX	FUS+DOX	AMP+CPR+STR	AMP+CPR+FUS	AMP+CPR+DOX	AMP+STR+FUS	AMP+STR+DOX	AMP+FUS+DOX	CPR+STR+FUS	CPR+STR+DOX	CPR+FUS+DOX	STR+FUS+DOX	AMP+CPR+STR+FUS	AMP+CPR+STR+DOX	AMP+CPR+FUS+DOX	AMP+STR+FUS+DOX	CPR+STR+FUS+DOX	AMP+CPR+STR+FUS+DOX
	89.79762502	93.00886436	84.06610539	82.03712995	85.78357585	73.20622178	18.06322127	62.48536545	89.79762502	59.29085131	74.81184145	86.85398896	83.81665108	77.08138447	74.67803981	5.78692089	41.76283659	99.16373976	2.542231142	11.92507108	60.07693594	27.0446563	59.82605787	72.53721358	65.99625819	2.274627864	53.13597592	56.59809333	0.401404917	2.15755143	3.646094665
	77.50142126	90.9181353	84.06610539	95.6935759	103.8800455	79.09323479	13.82888005	59.53666856	69.94030699	62.94769756	85.91529278	90.23592951	83.81665108	77.08138447	66.30187607	29.44855031	30.82717453	101.8334281	3.823194997	25.42637862	50.44059125	26.79079022	61.81068789	62.26549176	65.99625819	2.4587834	60.90108016	49.01932916	6.324616259	6.324616259	1.947129051
	85.36160325	91.17049085	71.4091527	95.81760093	97.90880046	72.34969503	12.40197502	60.94975312	80.24978217	61.42898635	85.12924775	93.49404589	85.42516236	82.28364295	76.80801626	29.82863782	42.36131281	91.63520186	3.61603253	21.46383967	40.96717979	33.35753703	65.61138542	68.44321812	64.97507929	3.848388034	52.5994772	47.47313389	5.939587569	15.46616323	4.022654662
AMP1CPR1STR2FUS2DOX2	AMP	CPR	STR	FUS	DOX	AMP+CPR	AMP+STR	AMP+FUS	AMP+DOX	CPR+STR	CPR+FUS	CPR+DOX	STR+FUS	STR+DOX	FUS+DOX	AMP+CPR+STR	AMP+CPR+FUS	AMP+CPR+DOX	AMP+STR+FUS	AMP+STR+DOX	AMP+FUS+DOX	CPR+STR+FUS	CPR+STR+DOX	CPR+FUS+DOX	STR+FUS+DOX	AMP+CPR+STR+FUS	AMP+CPR+STR+DOX	AMP+CPR+FUS+DOX	AMP+STR+FUS+DOX	CPR+STR+FUS+DOX	AMP+CPR+STR+FUS+DOX
	89.79762502	93.00886436	84.06610539	82.03712995	92.74126108	73.20622178	18.06322127	62.48536545	88.45960863	59.29085131	74.81184145	88.99481519	83.81665108	86.8101029	67.72035457	5.78692089	41.76283659	107.7270447	2.542231142	11.90834588	56.59809333	27.0446563	52.0655628	76.68506439	45.54100405	2.274627864	35.74176284	64.24151196	-1.204214752	3.361766182	8.730556949
	77.50142126	90.9181353	84.06610539	95.6935759	104.7896532	79.09323479	13.82888005	59.53666856	75.45480387	62.94769756	85.91529278	87.05230244	83.81665108	86.8101029	58.11540648	29.44855031	30.82717453	95.6935759	3.823194997	16.55770324	47.71176805	26.79079022	54.9886299	67.26833428	45.54100405	2.4587834	52.25980671	43.33428084	5.64241046	5.415008528	2.856736782
	85.36160325	91.17049085	71.4091527	95.81760093	96.51466744	72.34969503	12.40197502	60.94975312	69.83735115	61.42898635	85.12924775	92.56462387	85.42516236	89.29164779	72.8579727	29.82863782	42.36131281	93.9587569	3.61603253	23.32268371	20.34562881	33.35753703	57.71129829	72.39326169	35.46292101	3.848388034	33.77868138	53.99361022	3.848388034	7.566076096	1.757188498
AMP1CPR1STR2FUS2DOX3	AMP	CPR	STR	FUS	DOX	AMP+CPR	AMP+STR	AMP+FUS	AMP+DOX	CPR+STR	CPR+FUS	CPR+DOX	STR+FUS	STR+DOX	FUS+DOX	AMP+CPR+STR	AMP+CPR+FUS	AMP+CPR+DOX	AMP+STR+FUS	AMP+STR+DOX	AMP+FUS+DOX	CPR+STR+FUS	CPR+STR+DOX	CPR+FUS+DOX	STR+FUS+DOX	AMP+CPR+STR+FUS	AMP+CPR+STR+DOX	AMP+CPR+FUS+DOX	AMP+STR+FUS+DOX	CPR+STR+FUS+DOX	AMP+CPR+STR+FUS+DOX
	89.79762502	93.00886436	84.06610539	82.03712995	93.27646764	73.20622178	18.06322127	62.48536545	82.83993979	59.29085131	74.81184145	95.14969058	83.81665108	87.30901154	112.0086971	5.78692089	41.76283659	116.5579528	2.542231142	6.573005519	90.86803813	27.0446563	57.41762837	111.7410938	94.29373246	2.274627864	59.02324803	79.62870045	3.094162903	55.67820706	6.589730724
	77.50142126	90.9181353	84.06610539	95.6935759	95.0113701	79.09323479	13.82888005	59.53666856	81.82205799	62.94769756	85.91529278	90.23592951	83.81665108	87.30901154	97.96759522	29.44855031	30.82717453	104.3348493	3.823194997	22.69755543	84.09607732	26.79079022	67.49573621	94.78396816	94.29373246	2.4587834	45.43774872	61.52643547	7.006822058	48.79192723	9.906196703
	85.36160325	91.17049085	71.4091527	95.81760093	97.67644496	72.34969503	12.40197502	60.94975312	74.67325007	61.42898635	85.12924775	95.12053442	85.42516236	92.67482253	94.42346791	29.82863782	42.36131281	96.74702295	3.61603253	13.56375254	71.18791751	33.35753703	62.35840836	80.71449317	82.28364295	3.848388034	51.90241069	67.00551844	5.242521057	69.37264014	19.35811792
AMP1CPR1STR2FUS3DOX1	AMP	CPR	STR	FUS	DOX	AMP+CPR	AMP+STR	AMP+FUS	AMP+DOX	CPR+STR	CPR+FUS	CPR+DOX	STR+FUS	STR+DOX	FUS+DOX	AMP+CPR+STR	AMP+CPR+FUS	AMP+CPR+DOX	AMP+STR+FUS	AMP+STR+DOX	AMP+FUS+DOX	CPR+STR+FUS	CPR+STR+DOX	CPR+FUS+DOX	STR+FUS+DOX	AMP+CPR+STR+FUS	AMP+CPR+STR+DOX	AMP+CPR+FUS+DOX	AMP+STR+FUS+DOX	CPR+STR+FUS+DOX	AMP+CPR+STR+FUS+DOX
	89.79762502	93.00886436	84.06610539	78.82589062	85.78357585	73.20622178	18.06322127	78.69208898	89.79762502	59.29085131	78.82589062	86.85398896	86.31119426	77.08138447	109.8678709	5.78692089	53.26977755	99.16373976	2.007024586	11.92507108	93.81167419	35.87556448	59.82605787	112.5439037	95.04209542	14.58437866	53.13597592	98.09332664	8.446228466	49.64040809	12.71115571
	77.50142126	90.9181353	84.06610539	99.55940875	103.8800455	79.09323479	13.82888005	63.62990335	69.94030699	62.94769756	86.59749858	90.23592951	86.31119426	77.08138447	97.05798749	29.44855031	60.44627629	101.8334281	6.267765776	25.42637862	72.72598067	31.11142695	61.81068789	92.50994883	95.04209542	8.371233655	60.90108016	68.80329733	8.598635588	32.93064241	19.22967595
	85.36160325	91.17049085	71.4091527	93.49404589	97.90880046	72.34969503	12.40197502	55.89602091	80.24978217	61.42898635	84.19982573	93.49404589	86.63343906	82.28364295	88.84693581	29.82863782	59.38135347	91.63520186	2.410688353	21.46383967	50.74063317	37.3075806	65.61138542	84.19982573	90.25826914	14.72553006	52.5994772	66.07609643	2.918966018	49.3900668	11.92274179
AMP1CPR1STR2FUS3DOX2	AMP	CPR	STR	FUS	DOX	AMP+CPR	AMP+STR	AMP+FUS	AMP+DOX	CPR+STR	CPR+FUS	CPR+DOX	STR+FUS	STR+DOX	FUS+DOX	AMP+CPR+STR	AMP+CPR+FUS	AMP+CPR+DOX	AMP+STR+FUS	AMP+STR+DOX	AMP+FUS+DOX	CPR+STR+FUS	CPR+STR+DOX	CPR+FUS+DOX	STR+FUS+DOX	AMP+CPR+STR+FUS	AMP+CPR+STR+DOX	AMP+CPR+FUS+DOX	AMP+STR+FUS+DOX	CPR+STR+FUS+DOX	AMP+CPR+STR+FUS+DOX
	89.79762502	93.00886436	84.06610539	78.82589062	92.74126108	73.20622178	18.06322127	78.69208898	88.45960863	59.29085131	78.82589062	88.99481519	86.31119426	86.8101029	101.5721693	5.78692089	53.26977755	107.7270447	2.007024586	11.90834588	74.54423817	35.87556448	52.0655628	102.6425824	91.05082632	14.58437866	35.74176284	85.24836929	8.713831744	49.37280482	3.077437699
	77.50142126	90.9181353	84.06610539	99.55940875	104.7896532	79.09323479	13.82888005	63.62990335	75.45480387	62.94769756	86.59749858	87.05230244	86.31119426	86.8101029	91.14553724	29.44855031	60.44627629	95.6935759	6.267765776	16.55770324	71.36156907	31.11142695	54.9886299	87.27970438	91.05082632	8.371233655	52.25980671	55.6708357	7.006822058	49.2467311	4.448550313
	85.36160325	91.17049085	71.4091527	93.49404589	96.51466744	72.34969503	12.40197502	55.89602091	69.83735115	61.42898635	84.19982573	92.56462387	86.63343906	89.29164779	93.49404589	29.82863782	59.38135347	93.9587569	2.410688353	23.32268371	49.81121115	37.3075806	57.71129829	86.52338077	90.0166138	14.72553006	33.77868138	55.66366541	1.524832994	61.47255301	7.740342724
AMP1CPR1STR2FUS3DOX3	AMP	CPR	STR	FUS	DOX	AMP+CPR	AMP+STR	AMP+FUS	AMP+DOX	CPR+STR	CPR+FUS	CPR+DOX	STR+FUS	STR+DOX	FUS+DOX	AMP+CPR+STR	AMP+CPR+FUS	AMP+CPR+DOX	AMP+STR+FUS	AMP+STR+DOX	AMP+FUS+DOX	CPR+STR+FUS	CPR+STR+DOX	CPR+FUS+DOX	STR+FUS+DOX	AMP+CPR+STR+FUS	AMP+CPR+STR+DOX	AMP+CPR+FUS+DOX	AMP+STR+FUS+DOX	CPR+STR+FUS+DOX	AMP+CPR+STR+FUS+DOX
	89.79762502	93.00886436	84.06610539	78.82589062	93.27646764	73.20622178	18.06322127	78.69208898	82.83993979	59.29085131	78.82589062	95.14969058	86.31119426	87.30901154	98.36092992	5.78692089	53.26977755	116.5579528	2.007024586	6.573005519	73.47382505	35.87556448	57.41762837	98.6285332	93.29591519	14.58437866	59.02324803	77.48787423	5.502592407	63.17109885	41.61230975
	77.50142126	90.9181353	84.06610539	99.55940875	95.0113701	79.09323479	13.82888005	63.62990335	81.82205799	62.94769756	86.59749858	90.23592951	86.31119426	87.30901154	95.6935759	29.44855031	60.44627629	104.3348493	6.267765776	22.69755543	65.22171688	31.11142695	67.49573621	94.3291643	93.29591519	8.371233655	45.43774872	62.03808982	2.231381467	59.47981808	3.766344514
	85.36160325	91.17049085	71.4091527	93.49404589	97.67644496	72.34969503	12.40197502	55.89602091	74.67325007	61.42898635	84.19982573	95.12053442	86.63343906	92.67482253	107.4353761	29.82863782	59.38135347	96.74702295	2.410688353	13.56375254	52.17833285	37.3075806	62.35840836	101.6264885	89.04999245	14.72553006	51.90241069	46.5582341	3.151321522	36.37815858	13.54923032
AMP1CPR1STR3FUS1DOX1	AMP	CPR	STR	FUS	DOX	AMP+CPR	AMP+STR	AMP+FUS	AMP+DOX	CPR+STR	CPR+FUS	CPR+DOX	STR+FUS	STR+DOX	FUS+DOX	AMP+CPR+STR	AMP+CPR+FUS	AMP+CPR+DOX	AMP+STR+FUS	AMP+STR+DOX	AMP+FUS+DOX	CPR+STR+FUS	CPR+STR+DOX	CPR+FUS+DOX	STR+FUS+DOX	AMP+CPR+STR+FUS	AMP+CPR+STR+DOX	AMP+CPR+FUS+DOX	AMP+STR+FUS+DOX	CPR+STR+FUS+DOX	AMP+CPR+STR+FUS+DOX
	89.79762502	93.00886436	82.14559387	91.26944305	85.78357585	73.20622178	12.17594916	41.34470647	89.79762502	66.24853654	82.97374143	86.85398896	68	85.82375479	86.18498077	16.87573173	33.73473825	99.16373976	5.636394046	13.53069075	27.69693929	43.63605954	65.71332999	85.38217093	69.96168582	1.087138317	36.81217595	15.92239505	3.763171099	28.7673524	11.12226125
	77.50142126	90.9181353	79.41176471	55.15918135	103.8800455	79.09323479	21.1057419	16.33030131	69.94030699	64.53951109	55.61398522	90.23592951	68.59632742	94.10208528	62.43604321	26.26492325	20.36668562	101.8334281	2.856736782	33.84025014	10.64525298	27.41614554	77.50142126	63.57305287	63.86554622	4.448550313	54.9886299	18.83172257	6.097214326	27.70039795	7.91642979
	85.36160325	91.17049085	69.70740103	59.57014232	97.90880046	72.34969503	19.83735115	15.17571885	80.24978217	63.05547488	68.90792913	93.49404589	68.17399468	72.46127367	65.42259657	24.71681673	35.39064769	91.63520186	5.242521057	40.28463549	31.44060412	32.42811502	78.15858263	65.42259657	68.17399468	4.313099042	40.7493465	26.09642753	3.151321522	28.88469358	1.931455126
AMP1CPR1STR3FUS1DOX2	AMP	CPR	STR	FUS	DOX	AMP+CPR	AMP+STR	AMP+FUS	AMP+DOX	CPR+STR	CPR+FUS	CPR+DOX	STR+FUS	STR+DOX	FUS+DOX	AMP+CPR+STR	AMP+CPR+FUS	AMP+CPR+DOX	AMP+STR+FUS	AMP+STR+DOX	AMP+FUS+DOX	CPR+STR+FUS	CPR+STR+DOX	CPR+FUS+DOX	STR+FUS+DOX	AMP+CPR+STR+FUS	AMP+CPR+STR+DOX	AMP+CPR+FUS+DOX	AMP+STR+FUS+DOX	CPR+STR+FUS+DOX	AMP+CPR+STR+FUS+DOX
	89.79762502	93.00886436	82.14559387	91.26944305	92.74126108	73.20622178	12.17594916	41.34470647	88.45960863	66.24853654	82.97374143	88.99481519	68	96.85823755	94.74828567	16.87573173	33.73473825	107.7270447	5.636394046	31.99531694	32.66432514	43.63605954	66.24853654	91.26944305	57.21072797	1.087138317	45.10787757	28.38267269	5.636394046	72.40341194	5.903997324
	77.50142126	90.9181353	79.41176471	55.15918135	104.7896532	79.09323479	21.1057419	16.33030131	75.45480387	64.53951109	55.61398522	87.05230244	68.59632742	102.0697168	63.8004548	26.26492325	20.36668562	95.6935759	2.856736782	44.98294486	18.3769187	27.41614554	72.27117681	69.48550313	53.90600685	4.448550313	50.89539511	31.9641842	4.277998863	79.09323479	2.686185333
	85.36160325	91.17049085	69.70740103	59.57014232	96.51466744	72.34969503	19.83735115	15.17571885	69.83735115	63.05547488	68.90792913	92.56462387	68.17399468	74.71444218	63.79610805	24.71681673	35.39064769	93.9587569	5.242521057	41.21405751	37.94655823	32.42811502	72.58205054	69.37264014	54.40463151	4.313099042	46.32587859	35.62300319	3.383677026	79.55271565	1.002033111
AMP1CPR1STR3FUS1DOX3	AMP	CPR	STR	FUS	DOX	AMP+CPR	AMP+STR	AMP+FUS	AMP+DOX	CPR+STR	CPR+FUS	CPR+DOX	STR+FUS	STR+DOX	FUS+DOX	AMP+CPR+STR	AMP+CPR+FUS	AMP+CPR+DOX	AMP+STR+FUS	AMP+STR+DOX	AMP+FUS+DOX	CPR+STR+FUS	CPR+STR+DOX	CPR+FUS+DOX	STR+FUS+DOX	AMP+CPR+STR+FUS	AMP+CPR+STR+DOX	AMP+CPR+FUS+DOX	AMP+STR+FUS+DOX	CPR+STR+FUS+DOX	AMP+CPR+STR+FUS+DOX
	89.79762502	93.00886436	82.14559387	91.26944305	93.27646764	73.20622178	12.17594916	41.34470647	82.83993979	66.24853654	82.97374143	95.14969058	68	104.2145594	89.93142666	16.87573173	33.73473825	116.5579528	5.636394046	22.612477	38.55159726	43.63605954	75.61465128	87.25539388	78.29885057	1.087138317	35.47415956	31.71098846	8.847633384	62.63589229	2.291353069
	77.50142126	90.9181353	79.41176471	55.15918135	95.0113701	79.09323479	21.1057419	16.33030131	81.82205799	64.53951109	55.61398522	90.23592951	68.59632742	100.3267974	67.66628766	26.26492325	20.36668562	104.3348493	2.856736782	27.24559409	16.50085276	27.41614554	75.90960773	70.62251279	68.59632742	4.448550313	55.89823764	28.5531552	9.223990904	44.92609437	11.04320637
	85.36160325	91.17049085	69.70740103	59.57014232	97.67644496	72.34969503	19.83735115	15.17571885	74.67325007	63.05547488	68.90792913	95.12053442	68.17399468	81.97465185	77.50508278	24.71681673	35.39064769	96.74702295	5.242521057	31.68748185	22.61109498	32.42811502	73.27911705	70.76677316	66.42153028	4.313099042	46.5582341	47.24077839	3.383677026	50.78419983	2.396166134
[truncated: 13,412,647 more chars]
