# Supplementary material for: Prevalence and patterns of higher-order drug interactions in Escherichia coli
Source: NPJ Syst Biol Appl. 2018 Sep 3;4:31. doi: 10.1038/s41540-018-0069-9 (PMC6119685; doi:10.1038/s41540-018-0069-9)
Supplement: Supplementary file 1 — Supplementary Information [file 41540_2018_69_MOESM1_ESM.pdf]

Supplementary Information

Supplementary Methods

S1 Text. Emergent *N*-way measure (*N*>3)

Here, we provide details of the construction of emergent interaction measures when there are more than three drugs in the environment. As defined in the Materials and Methods, we use the notation $[\mathrm{N}_N]_{D_1 \perp D_2 \perp \cdots \perp D_n}$ to represent the lower-order contributions due to the interactions only within the drug subsets D_i with at least two drugs. In other words, $D_1 \perp D_2 \perp \cdots \perp D_n$ denotes a factorization of *N* drugs, where ⊥ signifies no-interaction among drug subsets, whereas drugs within a drug subset can interact. Therefore, the net interaction effect due to the lower-order factorization of $D_1 \perp D_2 \perp \cdots \perp D_n$ is calculated as

$$[\mathrm{N}_N]_{D_1 \perp D_2 \perp \cdots \perp D_n} = w_{D_1} w_{D_2} \dots w_{D_n} - w_{X_1} w_{X_2} \dots w_{X_N} \qquad \text{(Eq. S1)}$$

by the independence of each drug subset. The naive case that each subset $D_i$contains a single drug, then $[\mathrm{N}_N]_{D_1 \perp D_2 \perp \cdots \perp D_n}$ is identical to the net *N*-way interaction, i.e. N_N. Moreover, the k-drug effect, $[\mathrm{N}_N]_{X_1 X_2 \dots X_k \perp X_{k+1} \perp \cdots \perp X_N}$, where the $X_1 X_2 \dots X_k$ drugs are interacting and the remaining $N - k$ drugs interact additively with all other drugs, is given by $w_{X_{k+1}} \dots w_{X_N} [\mathrm{N}_k]_{X_1 X_2 \dots X_k}$, or equivalently

$$\left(\prod_{i=k+1}^{N} w_{X_i} \right) [\mathrm{N}_k]_{X_1 X_2 \dots X_k} \qquad \text{(Eq. S2)}$$

As introduced in the main text, the critical component of defining higher-order emergent interactions is to make sure that each of the distinct lower-order interaction effects are subtracted exactly once from the net *N*-way interaction. For 4-drug combinations, there are three different drug factorizations for lower-order contributions: (i) the contribution coming solely from 3-drug

combination effects, such as $[\mathrm{N}_4]_{X_1X_2X_3\perp X_4}$, (ii) the contribution coming solely from 2-drug combination effects, such as $[\mathrm{N}_4]_{X_1X_2\perp X_3\perp X_4}$, (iii) the contribution due to interactions of distinct 2-drug combinations, such as $[\mathrm{N}_4]_{X_1X_2\perp X_3X_4}$. Therefore, for constructing the 4-way emergent measure, we first subtract all lower-order interaction contributions from $\mathrm{N}_4 \coloneqq [\mathrm{N}_4]_{X_1X_2X_3X_4}$ to obtain

$$
\begin{aligned}
[\mathrm{N}_4]_{X_1X_2X_3X_4} - \big([\mathrm{N}_4]_{X_1\perp X_2X_3X_4} + [\mathrm{N}_4]_{X_2\perp X_1X_3X_4} + [\mathrm{N}_4]_{X_3\perp X_1X_2X_4} + [\mathrm{N}_4]_{X_4\perp X_1X_2X_3}\big) \\
- \big([\mathrm{N}_4]_{X_1X_2\perp X_3X_4} + [\mathrm{N}_4]_{X_1X_3\perp X_2X_4} + [\mathrm{N}_4]_{X_1X_4\perp X_2X_3}\big) \\
- \big([\mathrm{N}_4]_{X_1X_2\perp X_3\perp X_4} + [\mathrm{N}_4]_{X_1X_3\perp X_2\perp X_4} + [\mathrm{N}_4]_{X_1X_4\perp X_2\perp X_3} + [\mathrm{N}_4]_{X_2X_3\perp X_1\perp X_4} \\
+ [\mathrm{N}_4]_{X_2X_4\perp X_1\perp X_3} + [\mathrm{N}_4]_{X_3X_4\perp X_1\perp X_2}\big)
\end{aligned}
$$

When X_1, X_2, and the combined drug pair X_3X_4 are all non-interacting with respect to each other, the terms $[\mathrm{N}_4]_{X_1\perp X_2X_3X_4}$, $[\mathrm{N}_4]_{X_2\perp X_1X_3X_4}$, and $[\mathrm{N}_4]_{X_1X_2\perp X_3X_4}$ in the above expression are simply equal to the pairwise lower-order effect due to the X_3X_4 pair, i.e. $[\mathrm{N}_4]_{X_3X_4\perp X_1\perp X_2}$. Thus, this equation subtracts the term corresponding to the pairwise interaction effect between X_3 and X_4 four times from the overall interaction N_4. This observation can be checked by S1 Fig. To correct for this extra-counting issue, we must add back the term $[\mathrm{N}_4]_{X_3X_4\perp X_1\perp X_2}$ three times to the equation above to form the correct measure. Applying analogous logic to all drug pairs and singles, we eventually quantify emergent 4-way interactions by characterizing the net 4-way interaction relative to all lower-order interaction effects. The final equation is given by

$$
\begin{aligned}
\mathrm{E}_4 := [\mathrm{N}_4]_{X_1X_2X_3X_4} &- \big([\mathrm{N}_4]_{X_1\perp X_2X_3X_4} + [\mathrm{N}_4]_{X_2\perp X_1X_3X_4} + [\mathrm{N}_4]_{X_3\perp X_1X_2X_4} + [\mathrm{N}_4]_{X_4\perp X_1X_2X_3}\big) \\
&- \big([\mathrm{N}_4]_{X_1X_2\perp X_3X_4} + [\mathrm{N}_4]_{X_1X_3\perp X_2X_4} + [\mathrm{N}_4]_{X_1X_4\perp X_2X_3}\big) \\
&+ 2\big([\mathrm{N}_4]_{X_1X_2\perp X_3\perp X_4} + [\mathrm{N}_4]_{X_1X_3\perp X_2\perp X_4} + [\mathrm{N}_4]_{X_1X_4\perp X_2\perp X_3} + [\mathrm{N}_4]_{X_2X_3\perp X_1\perp X_4} \\
&+ [\mathrm{N}_4]_{X_2X_4\perp X_1\perp X_3} + [\mathrm{N}_4]_{X_3X_4\perp X_1\perp X_2}\big)
\end{aligned}
$$

By utilizing equations (S1) and (S2), we have the following formula for emergent 4-way interactions

$$
\begin{aligned}
\mathrm{E}_4 = [\mathrm{N}_4]_{X_1X_2X_3X_4} &- w_{X_1}[\mathrm{N}_3]_{X_2X_3X_4} - w_{X_2}[\mathrm{N}_3]_{X_1X_3X_4} - w_{X_3}[\mathrm{N}_3]_{X_1X_2X_4} \\
&- w_{X_4}[\mathrm{N}_3]_{X_1X_2X_3} - [\mathrm{N}_4]_{X_1X_2\perp X_3X_4} - [\mathrm{N}_4]_{X_1X_3\perp X_2X_4} - [\mathrm{N}_4]_{X_1X_4\perp X_2X_3} \\
&+ 2\big(w_{X_1}w_{X_2}[\mathrm{N}_2]_{X_3X_4} + w_{X_1}w_{X_3}[\mathrm{N}_2]_{X_2X_4} + w_{X_1}w_{X_4}[\mathrm{N}_2]_{X_2X_3} + w_{X_2}w_{X_3}[\mathrm{N}_2]_{X_1X_4} \\
&+ w_{X_2}w_{X_4}[\mathrm{N}_2]_{X_1X_3} + w_{X_3}w_{X_4}[\mathrm{N}_2]_{X_1X_2}\big)
\end{aligned}
$$

Overall, we subtract each lower-order interaction effect only once, as required by the definition of emergent interaction. Finally, substituting net interaction terms in the above form gives

$$
\begin{aligned}
\mathrm{E}_4 = {} & w_{X_1X_2X_3X_4} - w_{X_1}w_{X_2X_3X_4} - w_{X_2}w_{X_1X_3X_4} - w_{X_3}w_{X_1X_2X_4} - w_{X_4}w_{X_1X_2X_3} - w_{X_1X_2}w_{X_3X_4} - \\
& w_{X_1X_3}w_{X_2X_4} - w_{X_1X_4}w_{X_2X_3} + 2w_{X_1}w_{X_2}w_{X_3X_4} + 2w_{X_1}w_{X_3}w_{X_2X_4} + 2w_{X_1}w_{X_4}w_{X_2X_3} + \\
& 2w_{X_2}w_{X_3}w_{X_1X_4} + 2w_{X_2}w_{X_4}w_{X_1X_3} + 2w_{X_3}w_{X_4}w_{X_1X_2} - 6w_{X_1}w_{X_2}w_{X_3}w_{X_4}
\end{aligned}
$$

Moreover, note that the net and emergent interaction measures are symmetric with respect to all *k*-subsets of *N*-drug combinations as they must be. Indeed, in terms of the combinatorics, construction of the emergent interaction measure follows similar logic as in the inclusion-exclusion principle, which counts the number of elements in a union of a finite number of sets [1]. This correspondence is exact because we are considering and counting all lower-order subsets of elements (i.e., drugs), which is precisely what the inclusion-exclusion principle does. The same

types of terms appear in higher-order covariances [2], ANOVAs [3], and other measures [4], but the mathematical definitions and operations differ. Here we have used Bliss independence as our starting point to define no interaction versus interactions, as is commonly done in the drug literature [5]. However, in other contexts, other starting definitions of no interaction could be used, and the same combinatorics and logic of subtracting terms could be used to construct the corresponding emergent metrics based on these other definitions.

S2 Text. Rescaling of net and emergent interaction metrics

The normalization (rescaling) approach facilitates the separation of interaction classes [6-8] and was first established for pairwise interactions by Segre et al. [6], who rescaled with respect to the special reference or baseline cases of synergy (complete lethality) and antagonism (complete buffering). The interaction strength (i.e., magnitude of the interaction) is quantified relative to these baselines. Explicitly, for two-drug combinations, the interaction metric (N_2 or equivalently E_2) is divided by the same functional form as the metric except the pairwise fitness term $w_{X_1X_2}$ is replaced by 0 for negative values of the interaction metric and by the strongest individual drug effect on fitness (i.e. by $\min(w_{X_1}, w_{X_2})$) when the interaction metric is positive [6,9].

For higher-order drug combinations, we follow an extension of the 2-way rescaling method introduced by Sanjuan et al. [7] and Tekin et al. [8] that correctly defines baselines for quantifying interaction strength for net interactions and separately for emergent interactions. Thus, based on previous studies, we rescale higher-order interaction metrics by substituting the complete lethality fitness ($w_{X_1 \ldots X_N} = 0$) when the unscaled metric is negative for the both net and emergent interactions. When the unscaled metric is positive, we replace $w_{X_1 \ldots X_N}$ with $\min(w_{X_1}, \ldots, w_{X_N})$ for rescaling net N-way interactions (N_N), but for the emergent interactions

(E_N) we instead use the minimum of weighted fitnesses given in emergent interaction formulas, such as $\min(w_{X_1}w_{X_2X_3}, w_{X_2}w_{X_1X_3}, w_{X_3}w_{X_1X_2})$ in the case of the $X_1X_2X_3$ combination.

Supplementary Figures

S1 Fig. Illustrations of the construction of (A) emergent 3-way and (B) emergent 4-way interaction measures. The emergent *N*-way measure is expressed as the net *N*-drug interaction relative to the effects originating solely from each lower-order combination effect, as described in the section "Mathematical Framework".

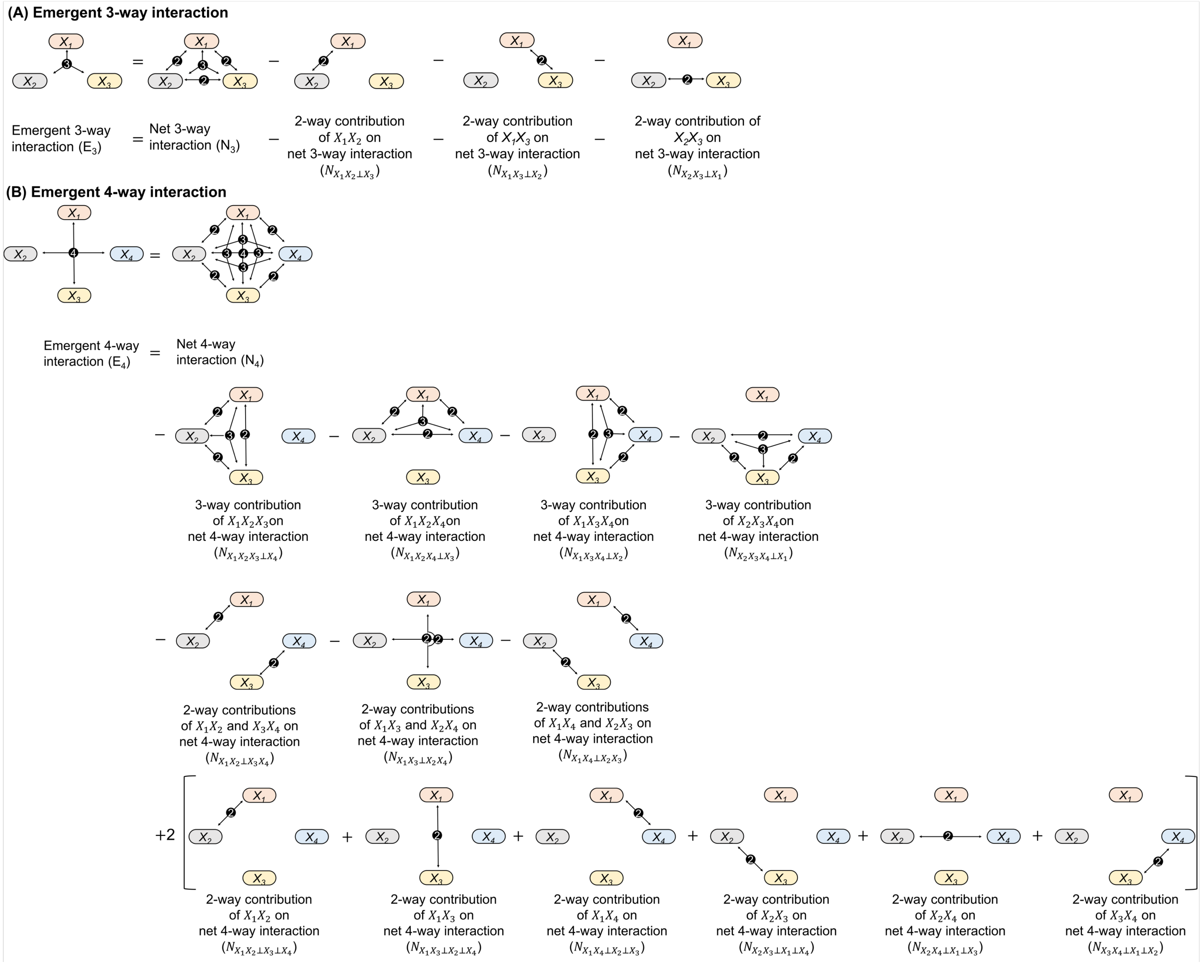

S2 Fig. Frequencies of interaction types versus number of drugs. The frequencies of (A) synergy and (B) antagonism are plotted as a function of the number of drugs (*N*) in the environment for net and emergent interactions.

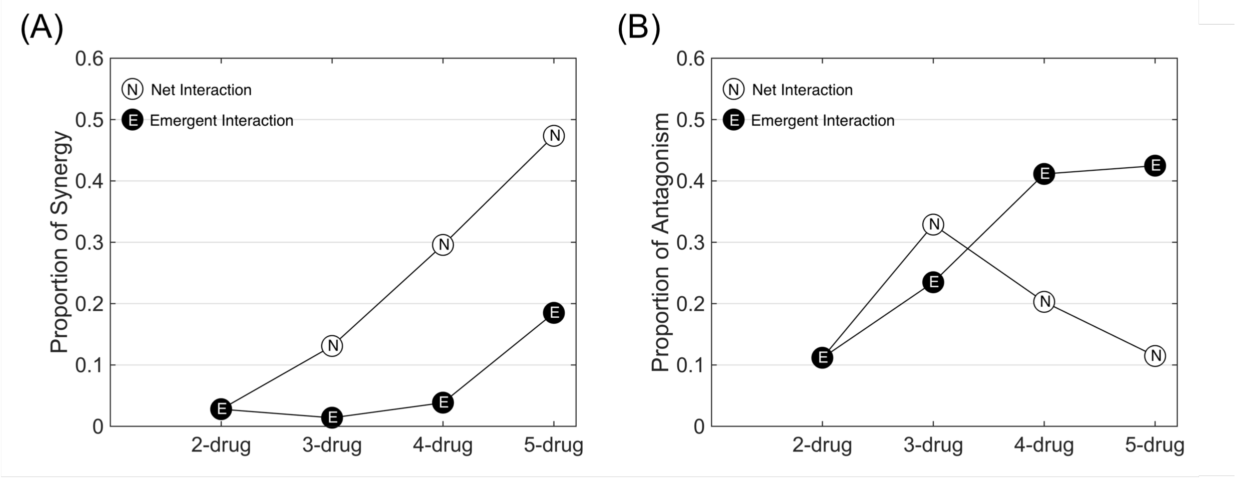

Supplementary Tables

S1 Table. 3-way breakdown profile and breakdown scores.

All possible breakdown profiles of 3-drug combinations (as denoted by $X_1X_2X_3$ in the Mathematical Framework above) into three pairwise parts (i.e., X_1X_2, X_1X_3, and X_2X_3) based on their interaction types (synergy, no-interaction, or antagonism) are listed with their corresponding breakdown scores and color codes. When calculating pairwise interaction scores, the interaction types synergy, no-interaction, and antagonism are simply represented by -1, 0, and 1, respectively. Therefore, the minimum (or maximum) breakdown score is -3 (or 3) and attained by the case that all pairwise-combinations are synergistic (or antagonistic). Breakdown score is equal to 0 when the number of synergies is equal to the number of antagonisms within the 3-way breakdown profile. An analogous table can be constructed for 4-way and 5-way breakdown scores, where the range of breakdown scores will depend on the number of lower-order combinations. For 4-drug combinations, there are 10 lower-order combinations (total of 4 different 3-drug combinations, 6 different 2-drug combinations), hence the minimum breakdown value is -10 (all synergies) and the maximum breakdown score is 10 (all antagonisms). Following a similar logic, the breakdown score for 5-way combinations ranges between -25 and 25 (Fig 3b).

3-way breakdown profile	Breakdown score
synergy+synergy+synergy	-3
no interaction+synergy+synergy	-2
no interaction+no interaction+synergy	-1
antagonism+synergy+synergy	-1
no interaction+no interaction+no interaction	0
no interaction+antagonism+synergy	0
no interaction+no interaction+antagonism	1
antagonism+antagonism+synergy	1
no interaction+antagonism+antagonism	2
antagonism+antagonism+antagonism	3

Supplementary References

1 Allenby, R. B. & Slomson, A. *How to count: An introduction to combinatorics*. (CRC Press, 2011).

2 Staude, B., Grün, S. & Rotter, S. Higher-order correlations and cumulants. *Analysis of parallel spike trains*, 253-280 (2010).

3 Cohen, B. H. *Explaining psychological statistics*. (John Wiley & Sons, 2008).

4 Isserlis, L. On a formula for the product-moment coefficient of any order of a normal frequency distribution in any number of variables. *Biometrika* **12**, 134-139 (1918).

5 Bliss, C. I. The toxicity of poisons applied jointly *Annals of Applied Biology* **26**, 585-615 (1939).

6 Segre, D., DeLuna, A., Church, G. & Kishony, R. Modular epistasis in yeast metabolism. *Nature Genetics* **37**, 77-83, doi:10.1038/ng1489 (2005).

7 Sanjuán, R. & Elena, S. F. Epistasis correlates to genomic complexity. *Proceedings of the National Academy of Sciences* **103**, 14402-14405 (2006).

8 Tekin, E. *et al.* Enhanced identification of synergistic and antagonistic emergent interactions among three or more drugs. *Journal of The Royal Society Interface* **13**, 20160332 (2016).

9 Yeh, P., Tschumi, A. & Kishony, R. Functional classification of drugs by properties of their pairwise interactions. *Nature Genetics* **38**, 489-494, doi:10.1038/ng1755 (2006).
